# Supplementary material for: An in vivo “turning model” reveals new RanBP9 interactions in lung macrophages
Source: Cell Death Discov. 2025 Apr 13;11:171. doi: 10.1038/s41420-025-02456-2 (PMC11994786; doi:10.1038/s41420-025-02456-2)
Supplement: Supplementary file 2 — Original Data [file 41420_2025_2456_MOESM2_ESM.pdf]

WholeLung HA_59.644 Sec 0021.JPG

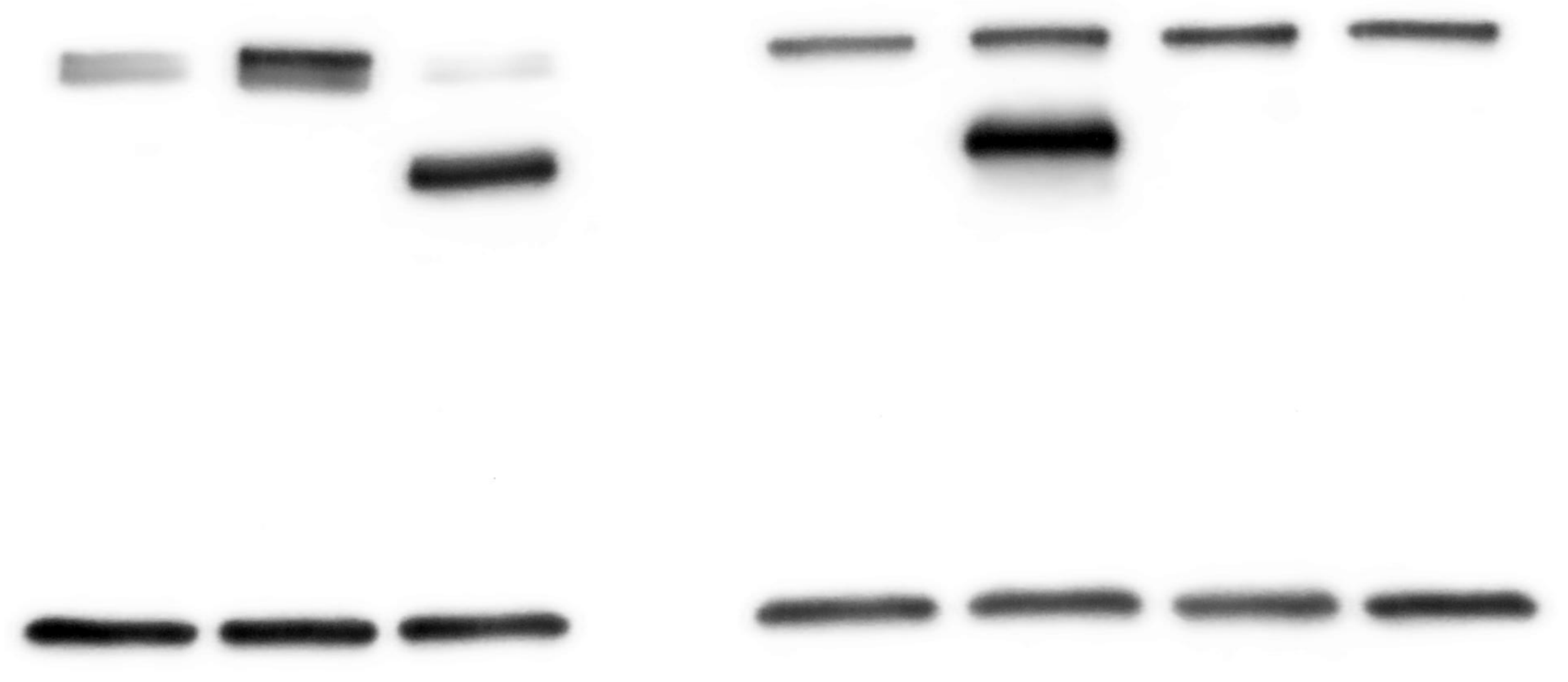

WholeLung RanBP9_59.644 Sec 002_NS.JPG

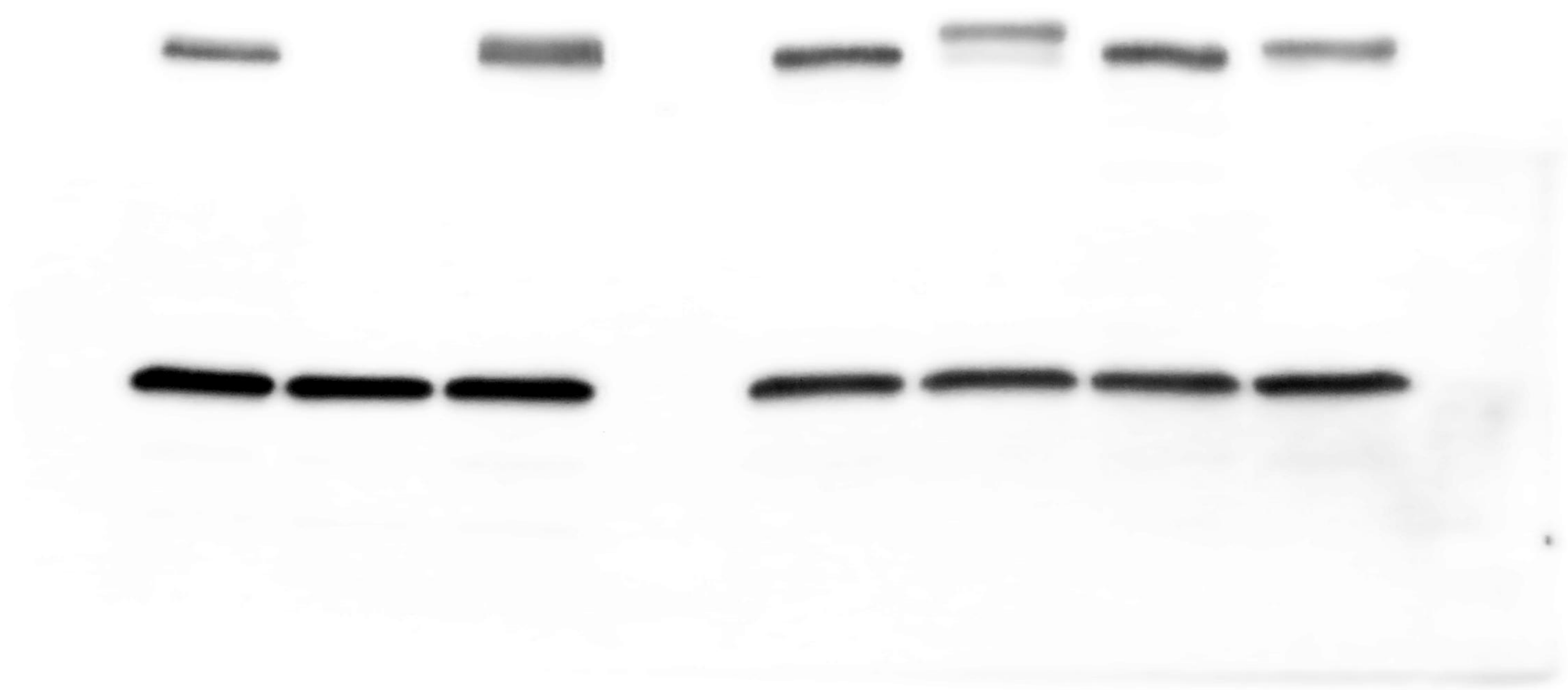

WholeLung V5_119.439 Sec 0013.JPG

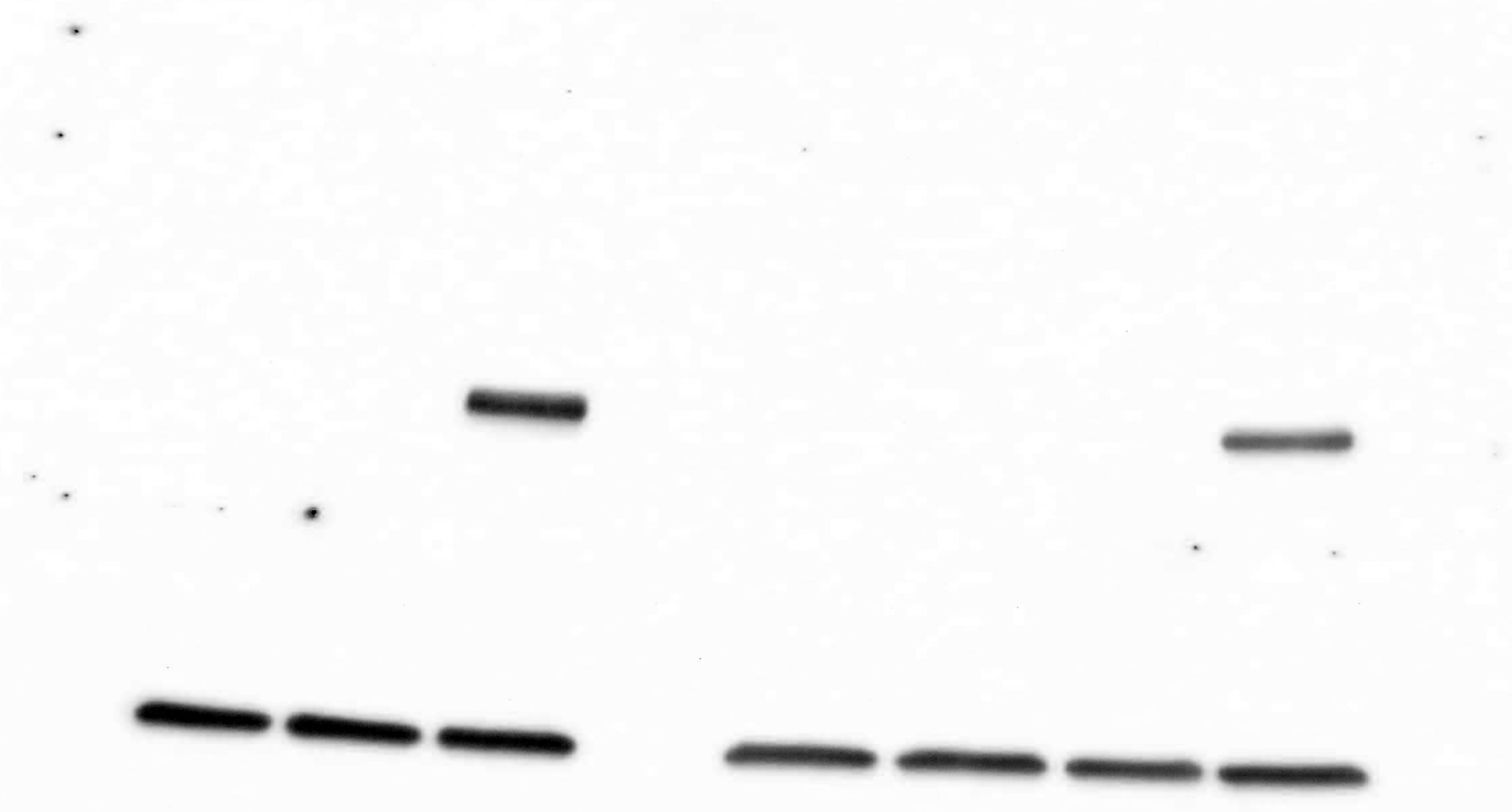

Liver RanBP9_HA_239.785 Sec 0009.JPG

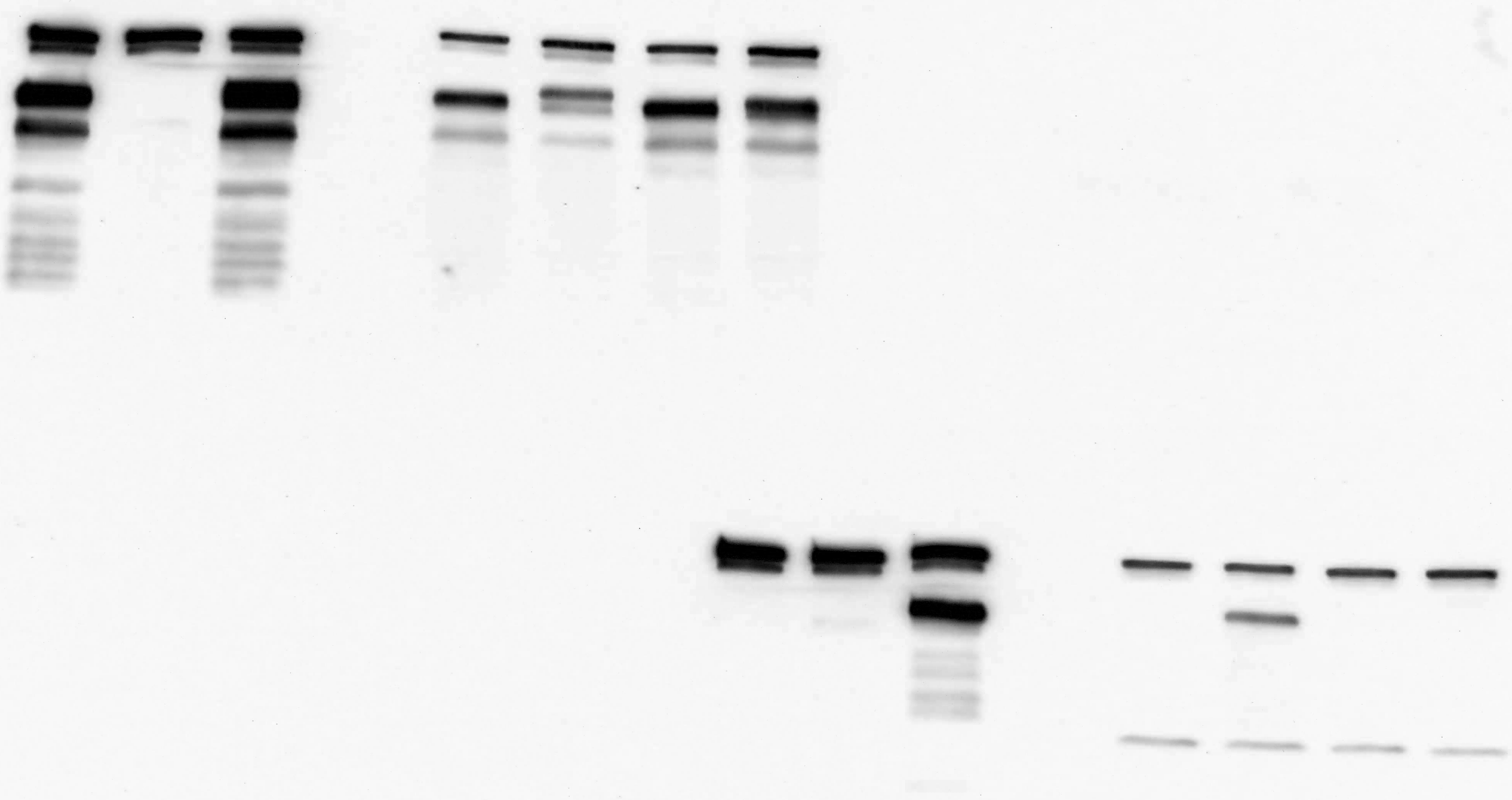

V5tag2_239.181 Sec 0024.JPG

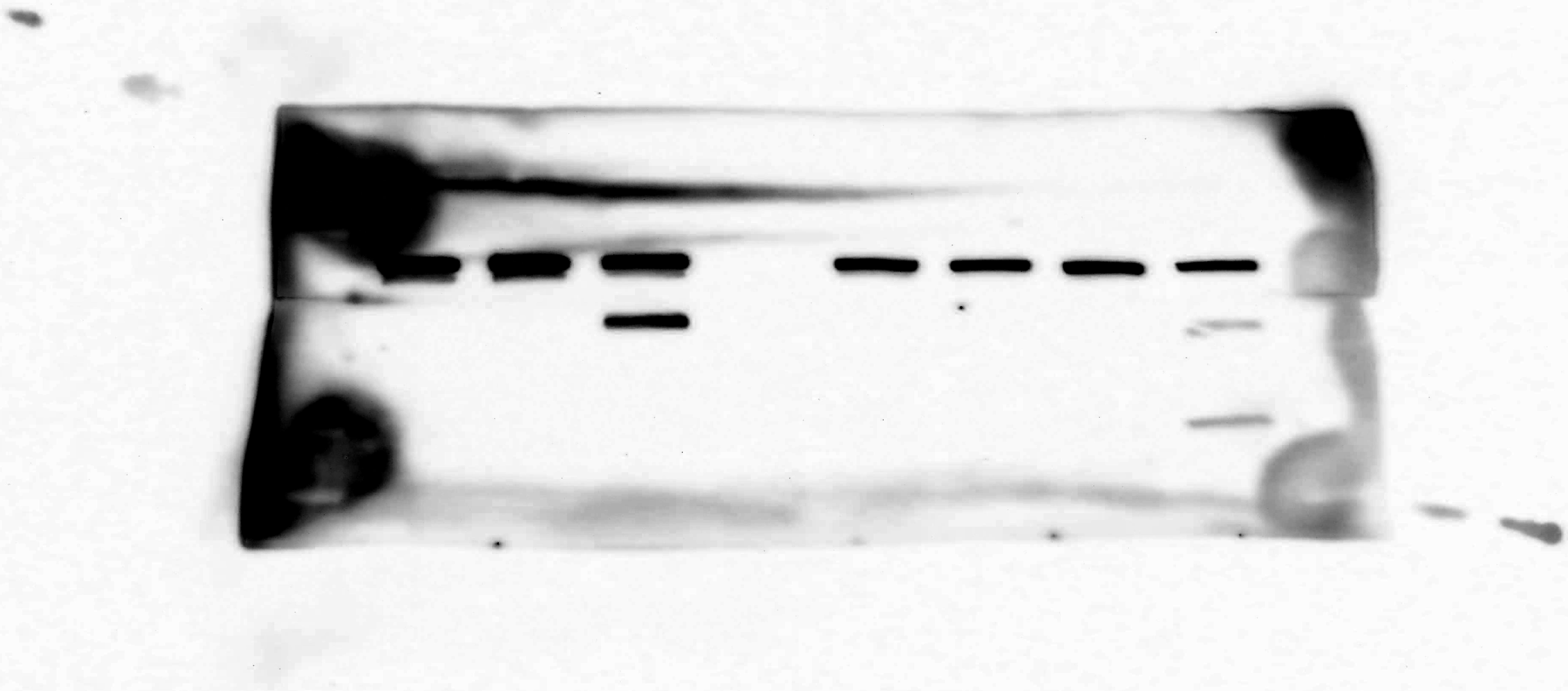

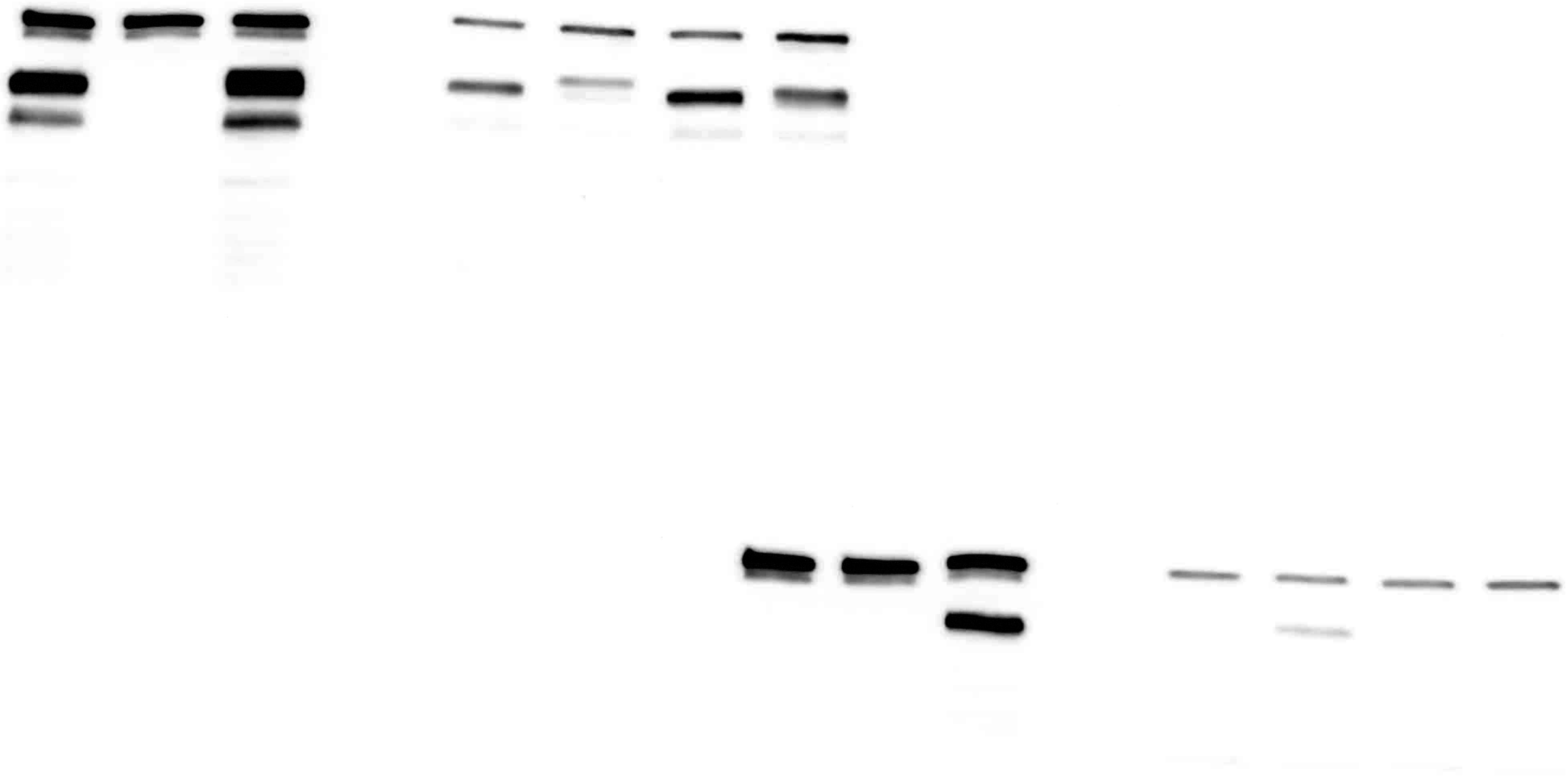

V5tag2_239.181 Sec 0024.JPG

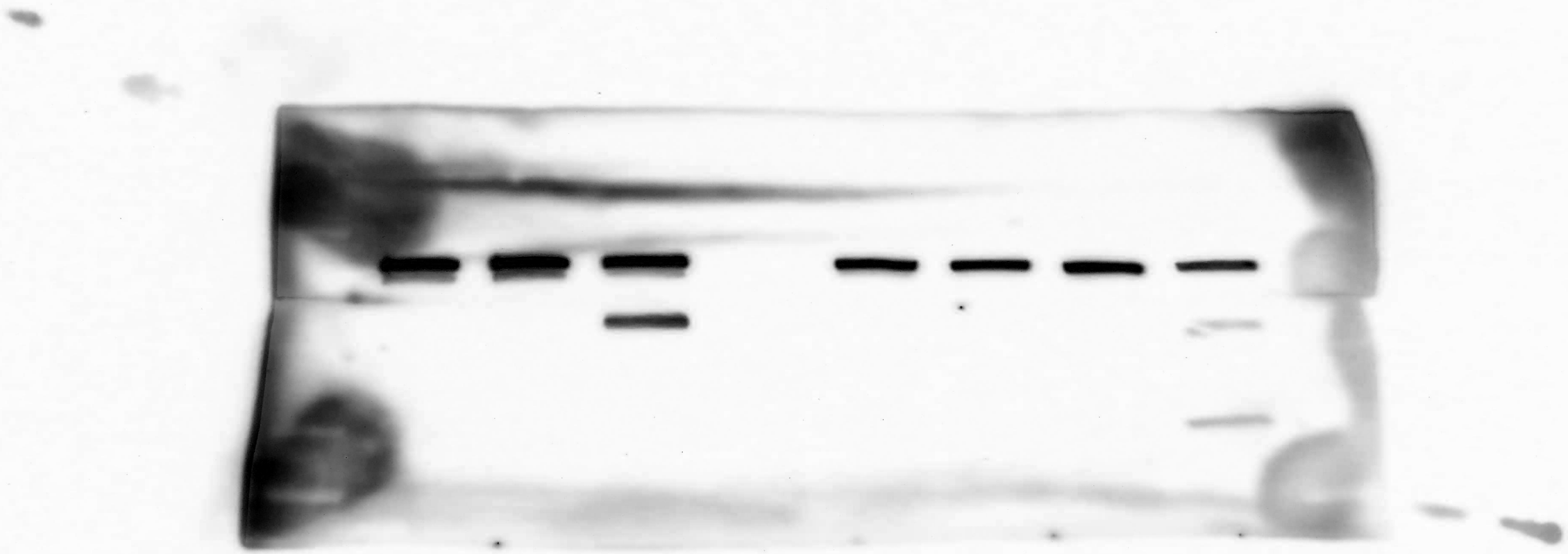

Kidney HA tag_14.949 Sec 001_NS.JPG

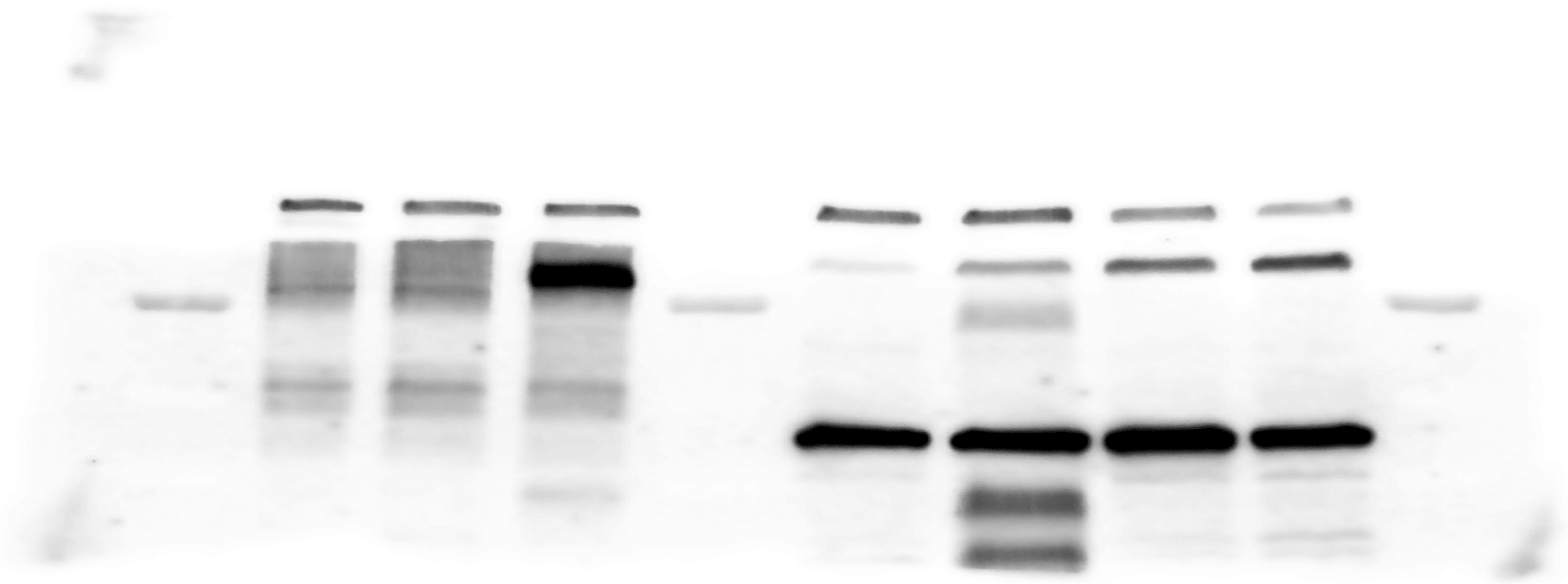

RanBP9 kidney_29.898 Sec 000_NS.JPG

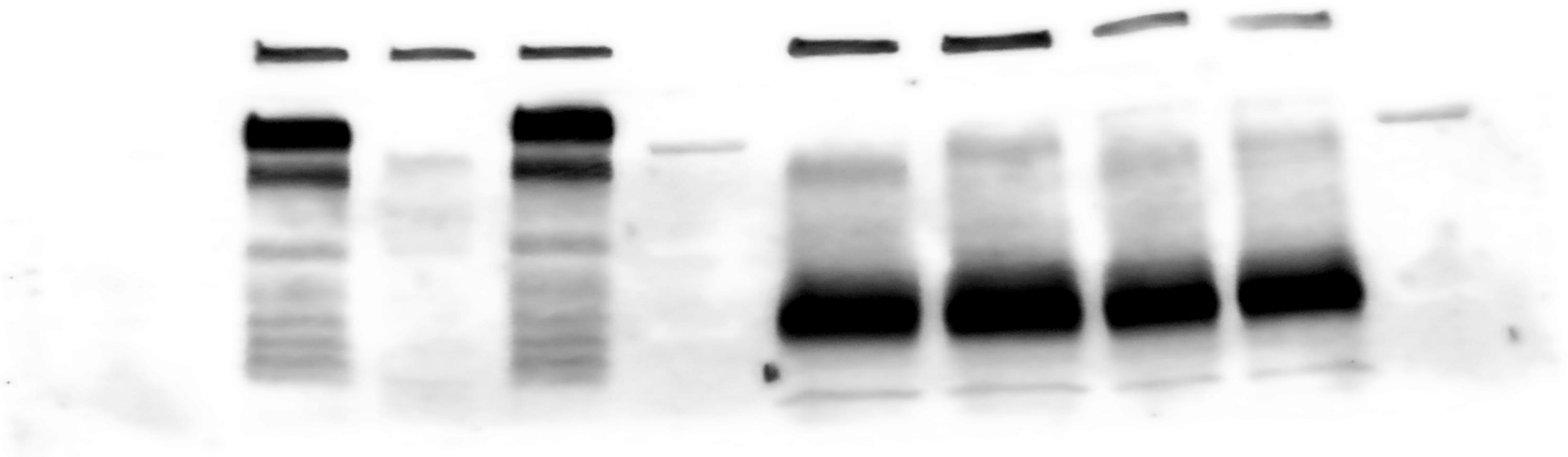

Kidney HA tag_29.898 Sec 001_NS.JPG

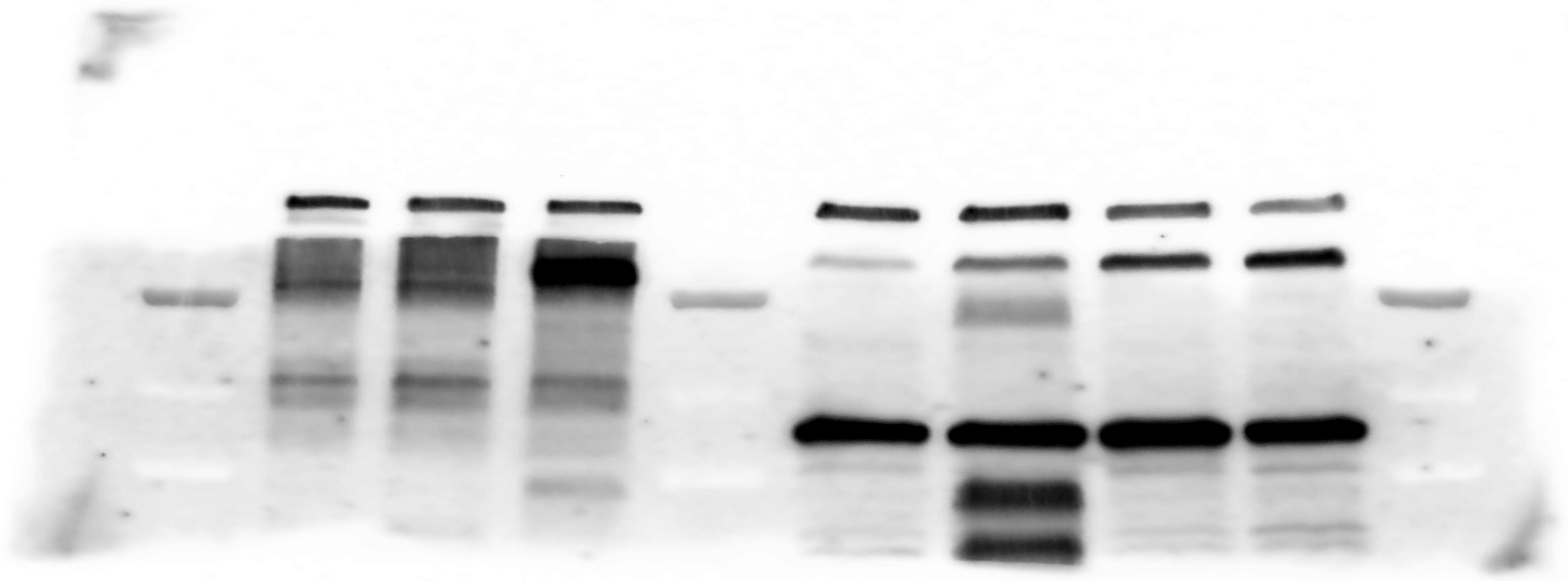

v5 tag again_119.892 Sec 000_NS.JPG

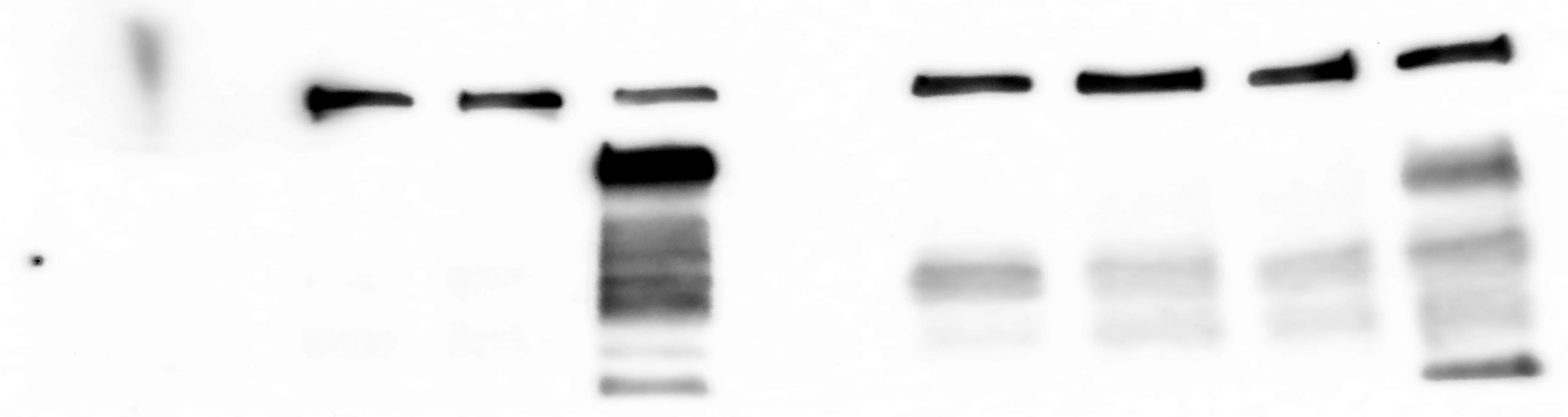

RanBP9 kidney_59.795 Sec 000_NS.JPG

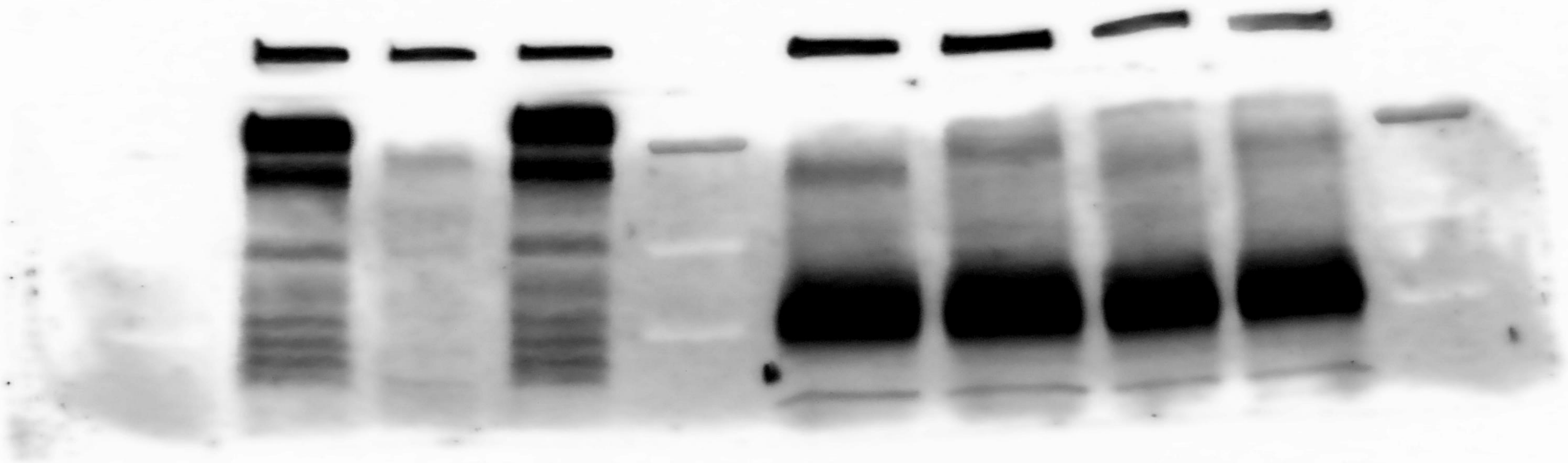

Spleen HA_V5_14.798 Sec 000_NS.JPG

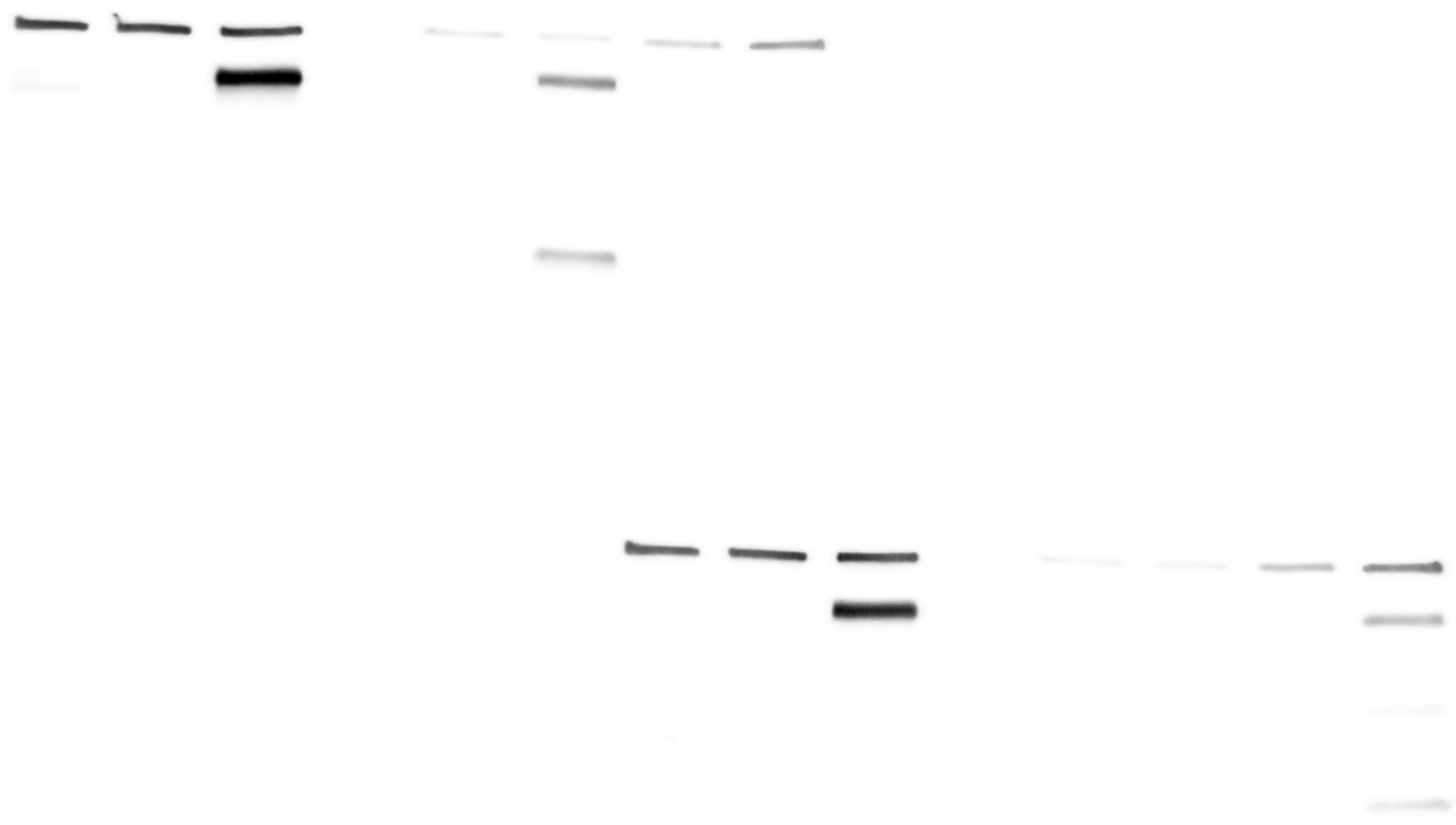

Spleen RanBP9_RanBP10_29.747 Sec 001_NS.JPG

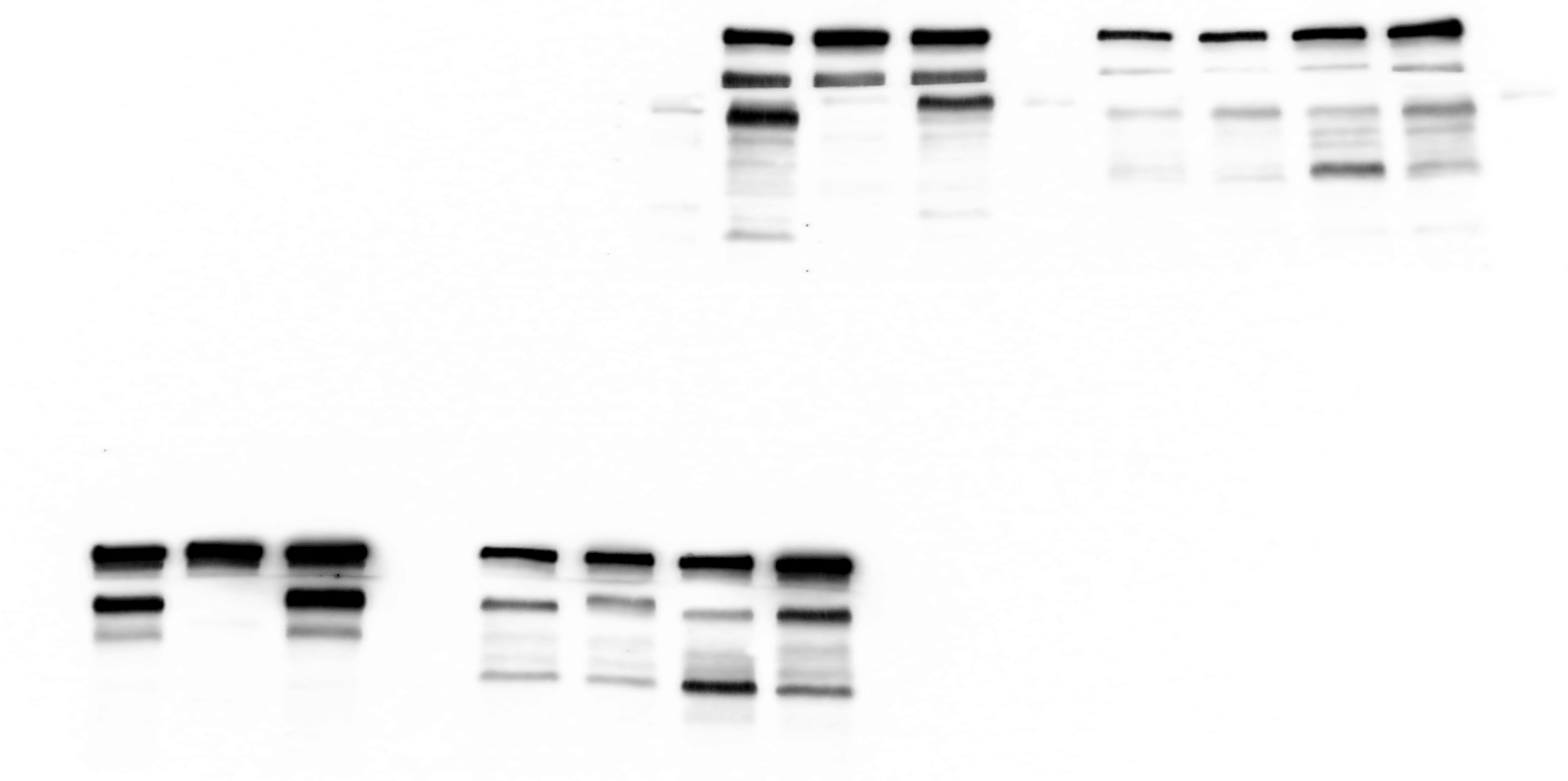

Spleen HA_V5_59.644 Sec 000_NS.JPG

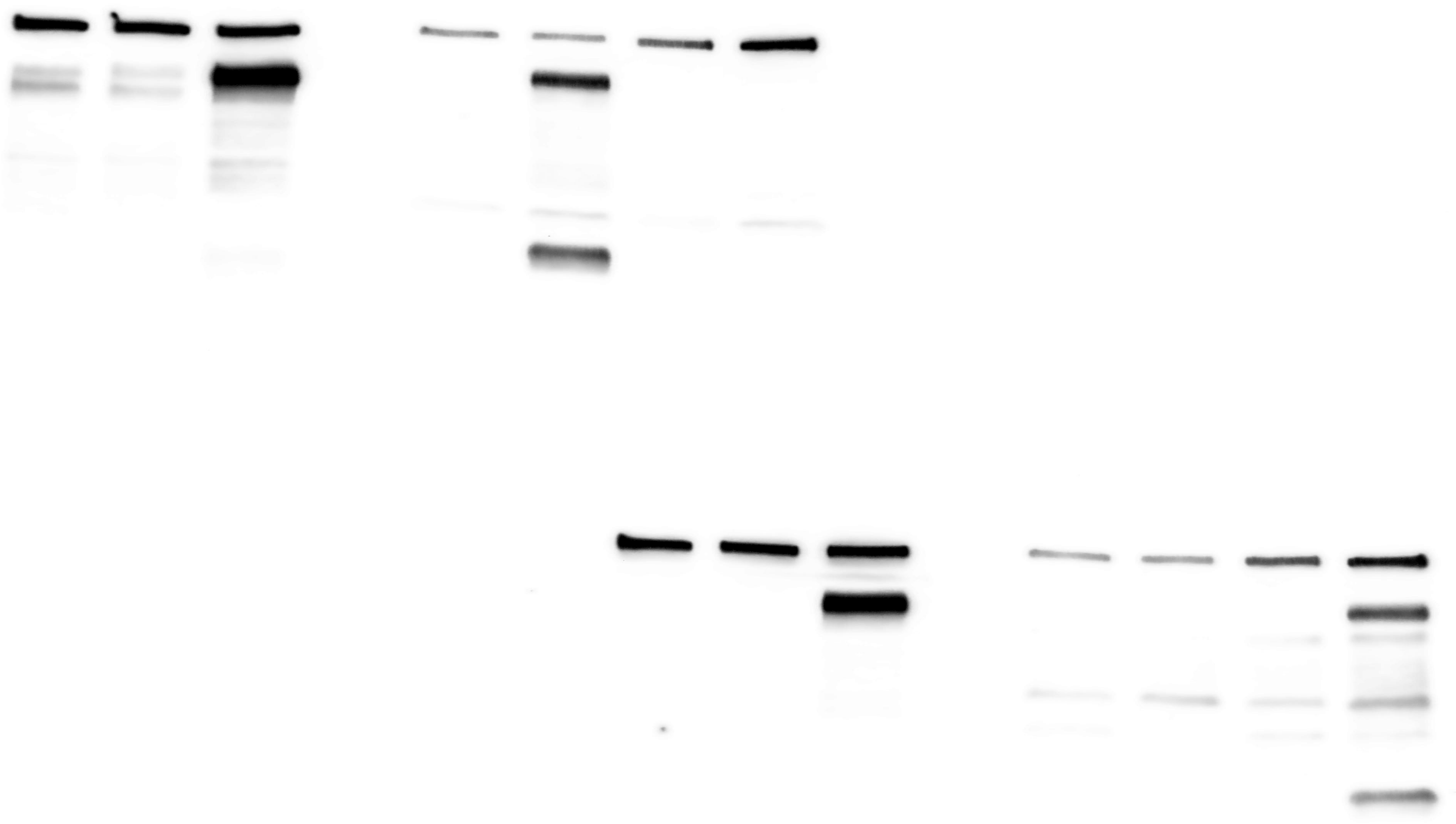

Spleen HA_V5_119.439 Sec 000_NS.JPG

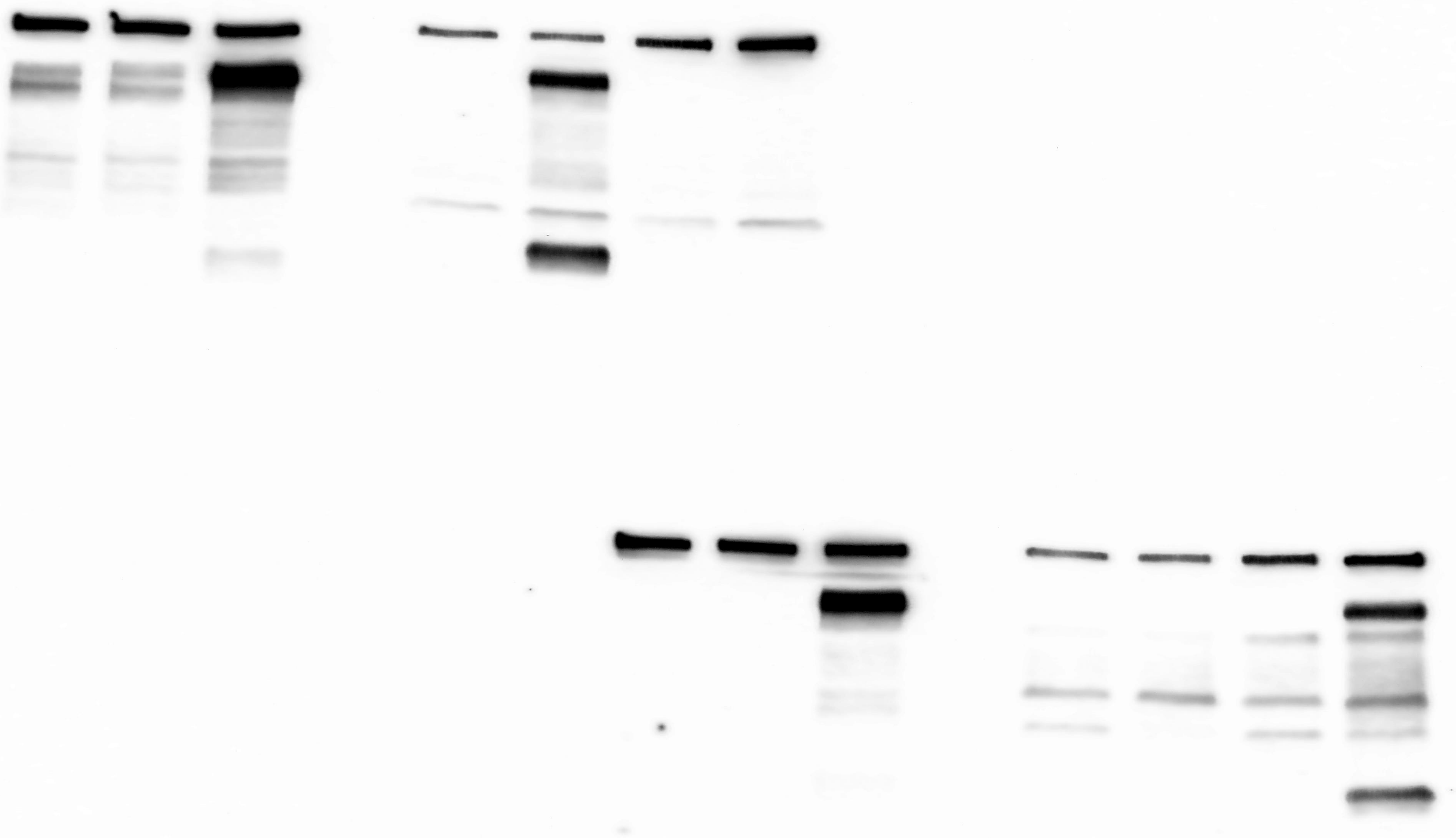

Spleen RanBP9_RanBP10_3.926 Sec 000_NS.JPG

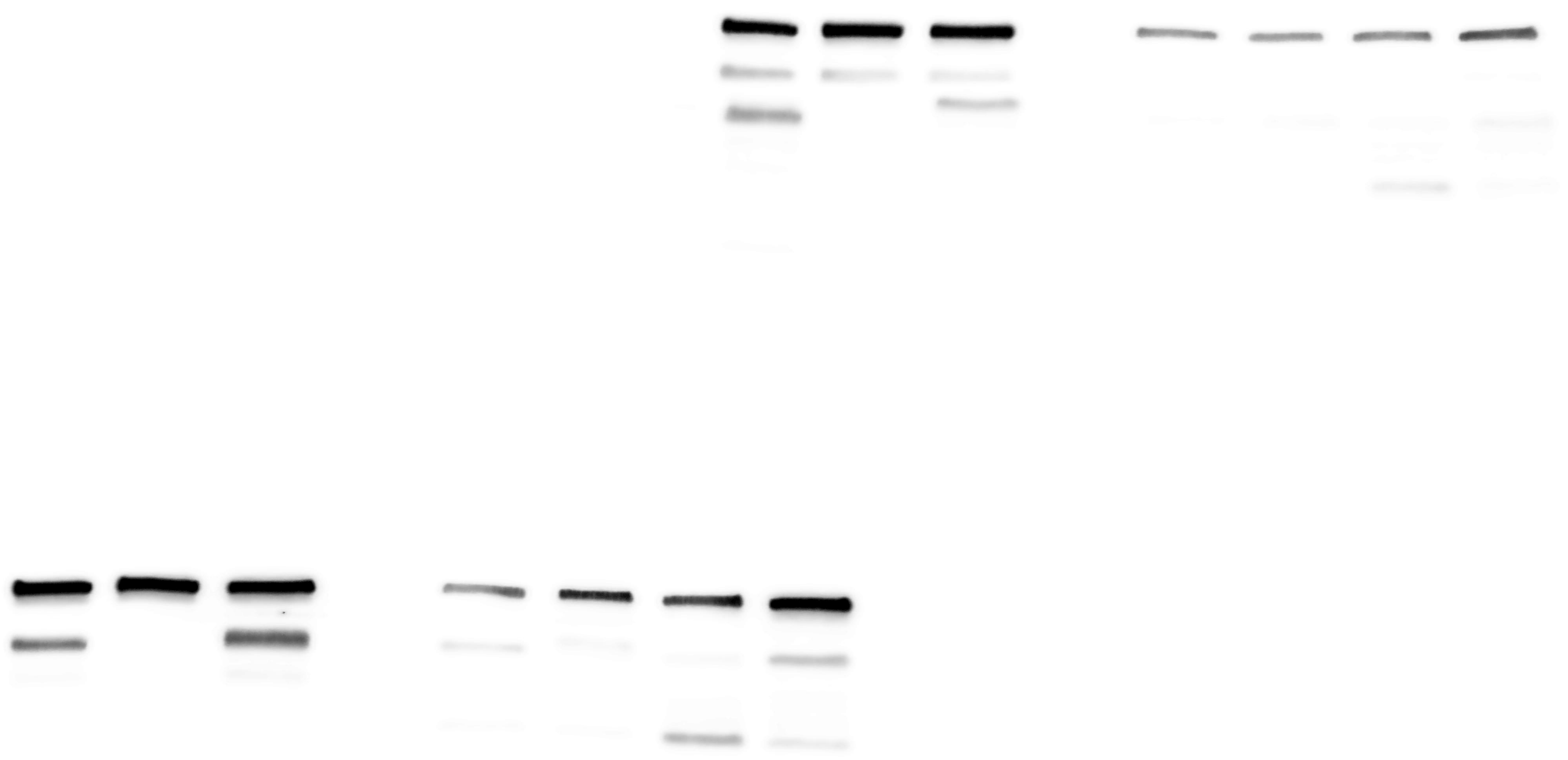

Thymus HA_V5_7.852 Sec 0004.JPG

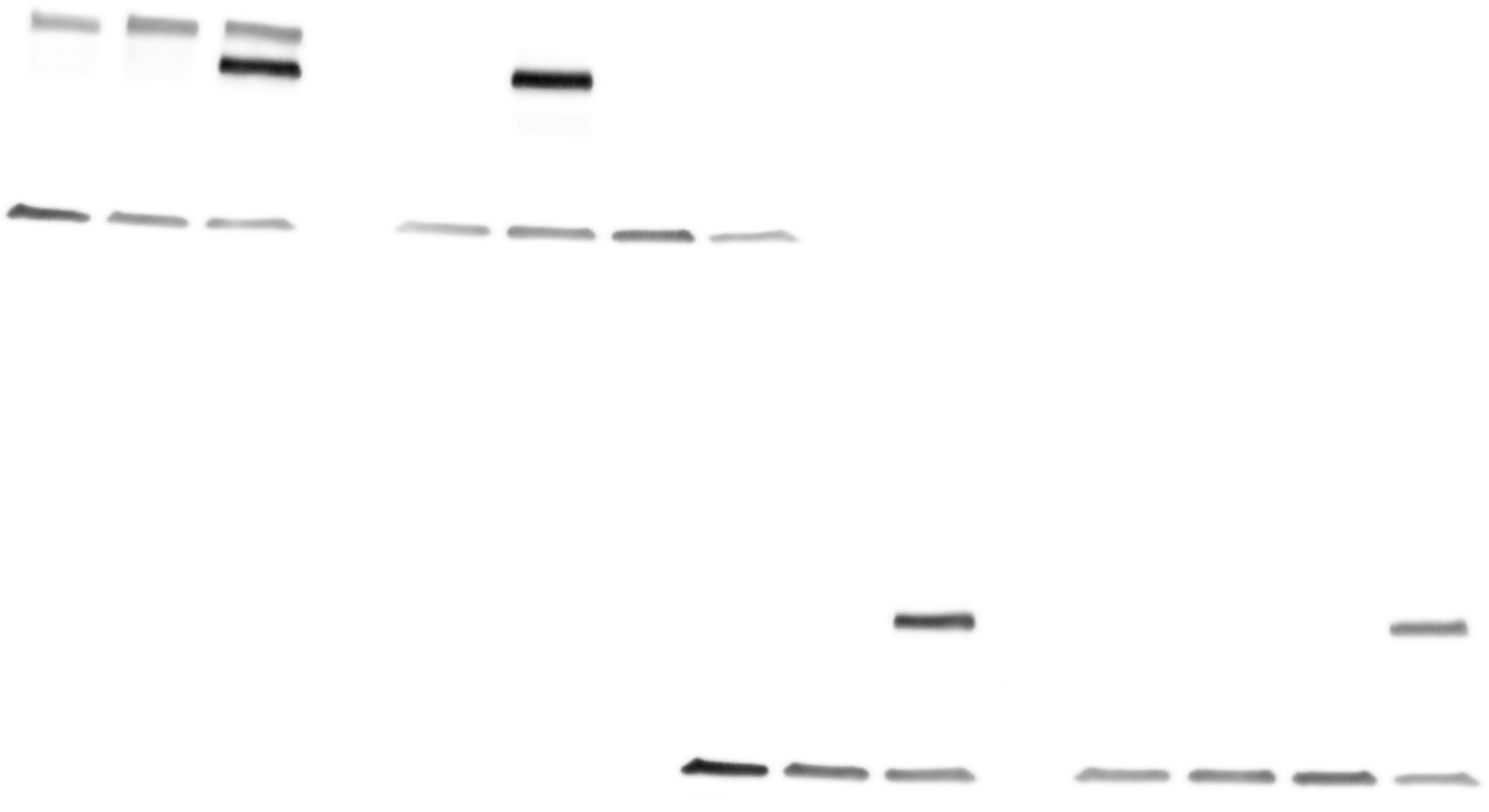

Thymus RanBP9_RanBP10_238.879 Sec 0009.JPG

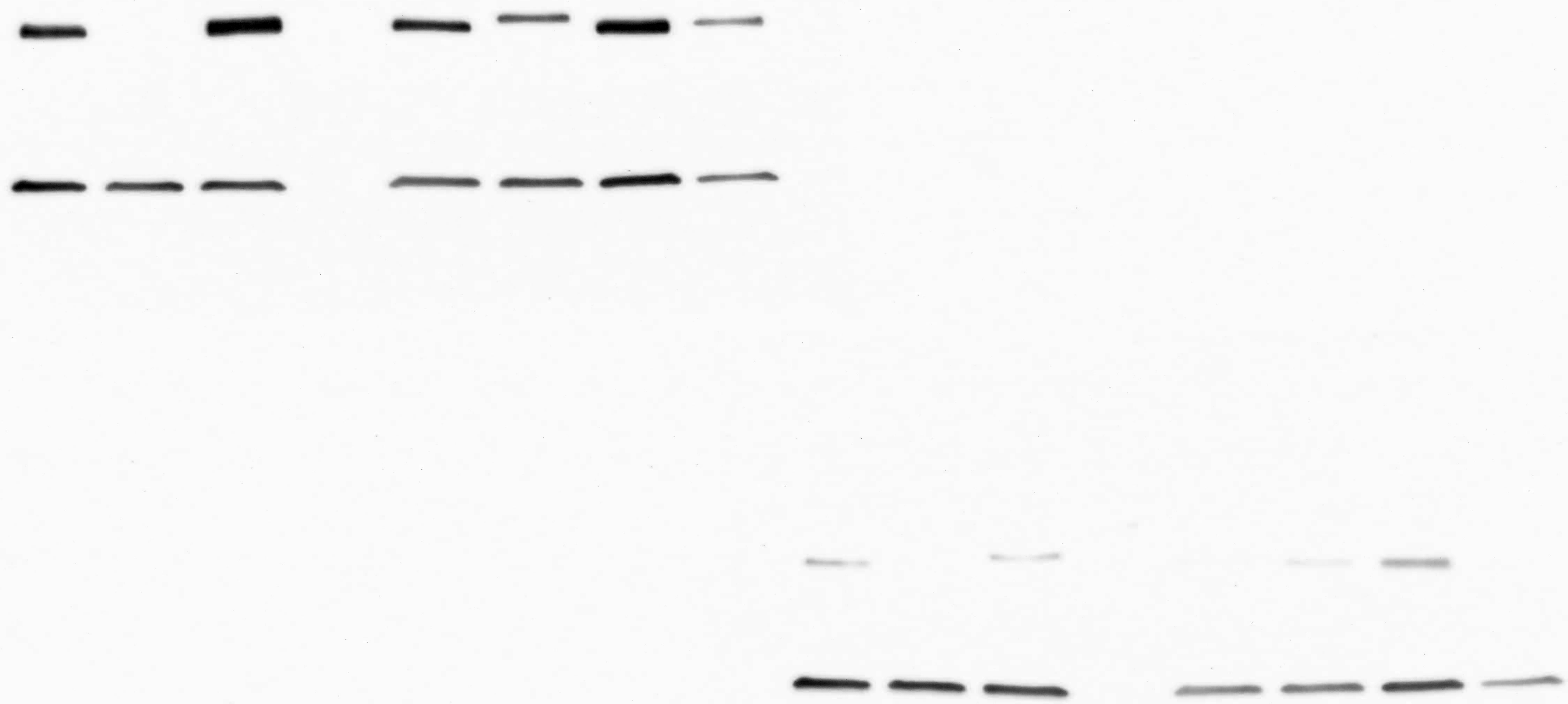

Thymus HA_V5_29.143 Sec 000_NS.JPG

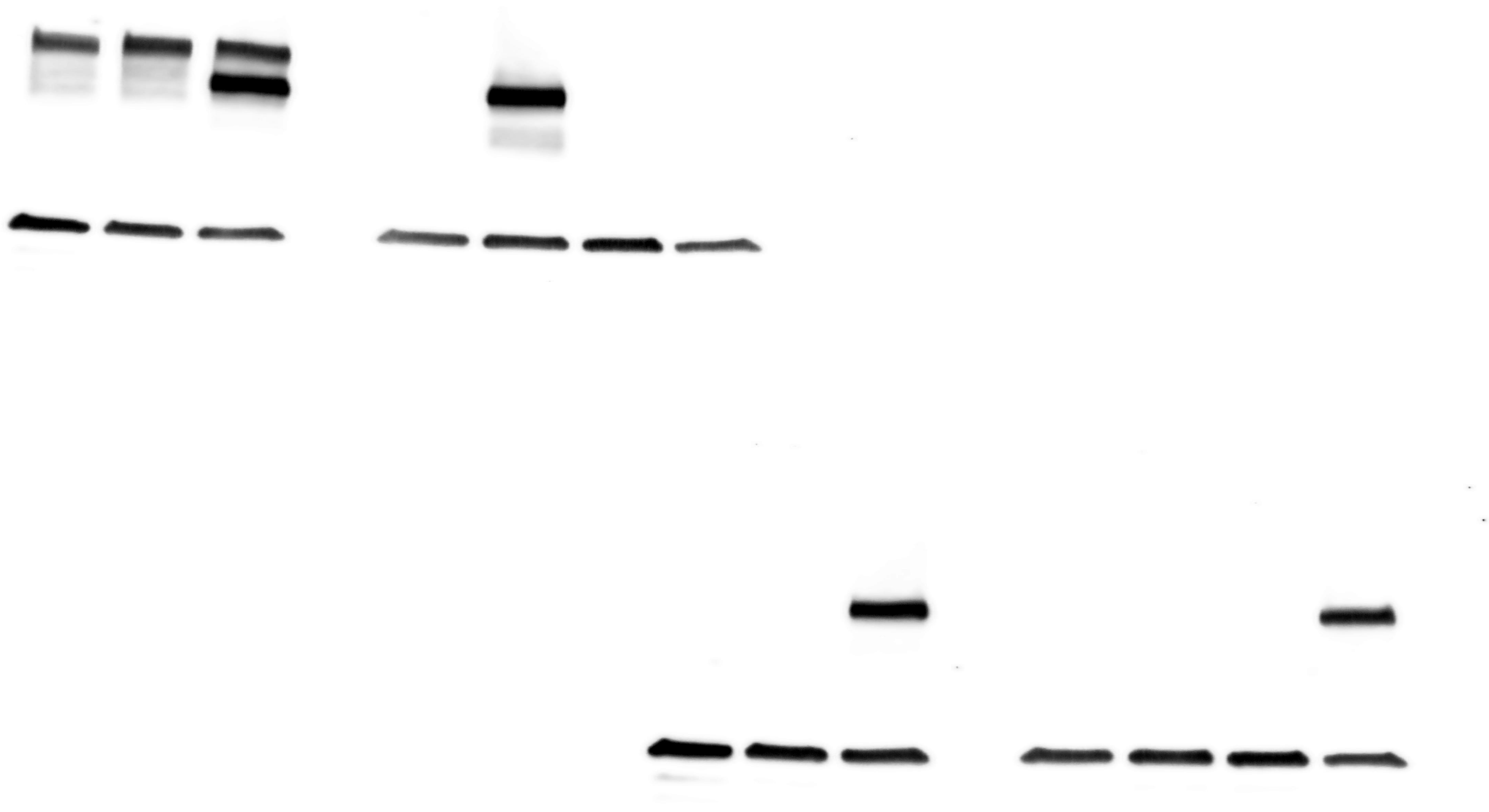

IP_BMDM BP9_MAEA_GID8_7.852 Sec 000_NS.JPG

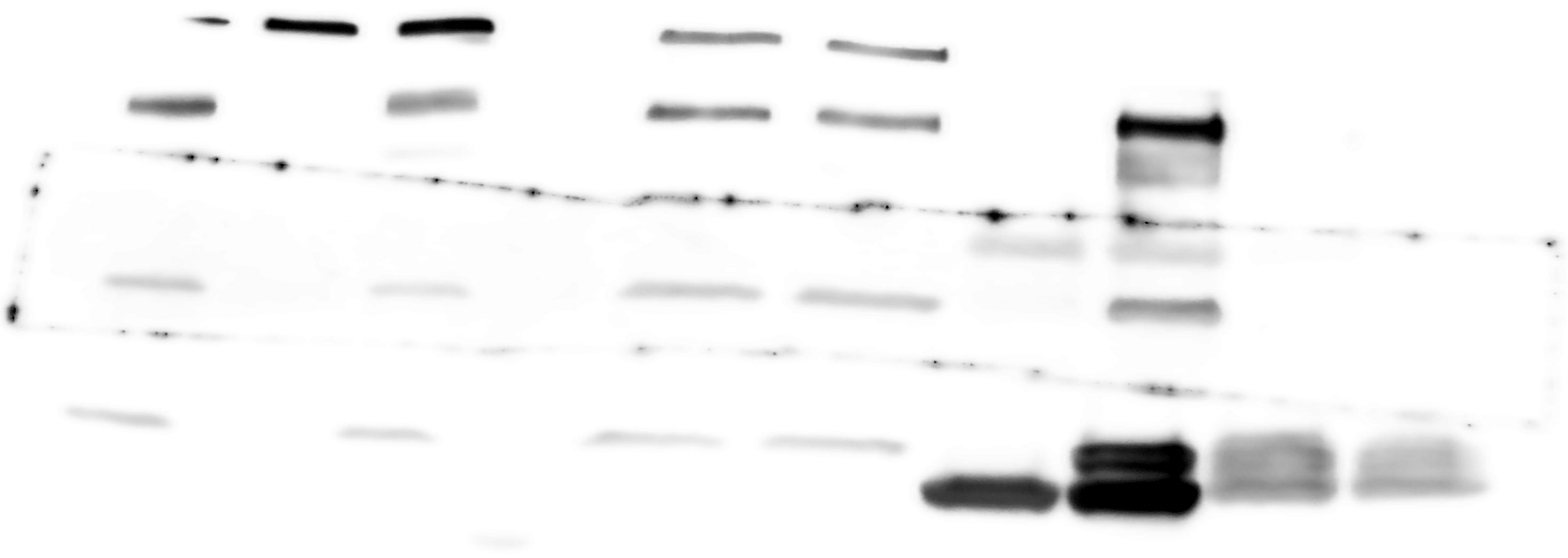

IP_BMDM BP9_MAEA_GID8_29.898 Sec 000_NS.JPG

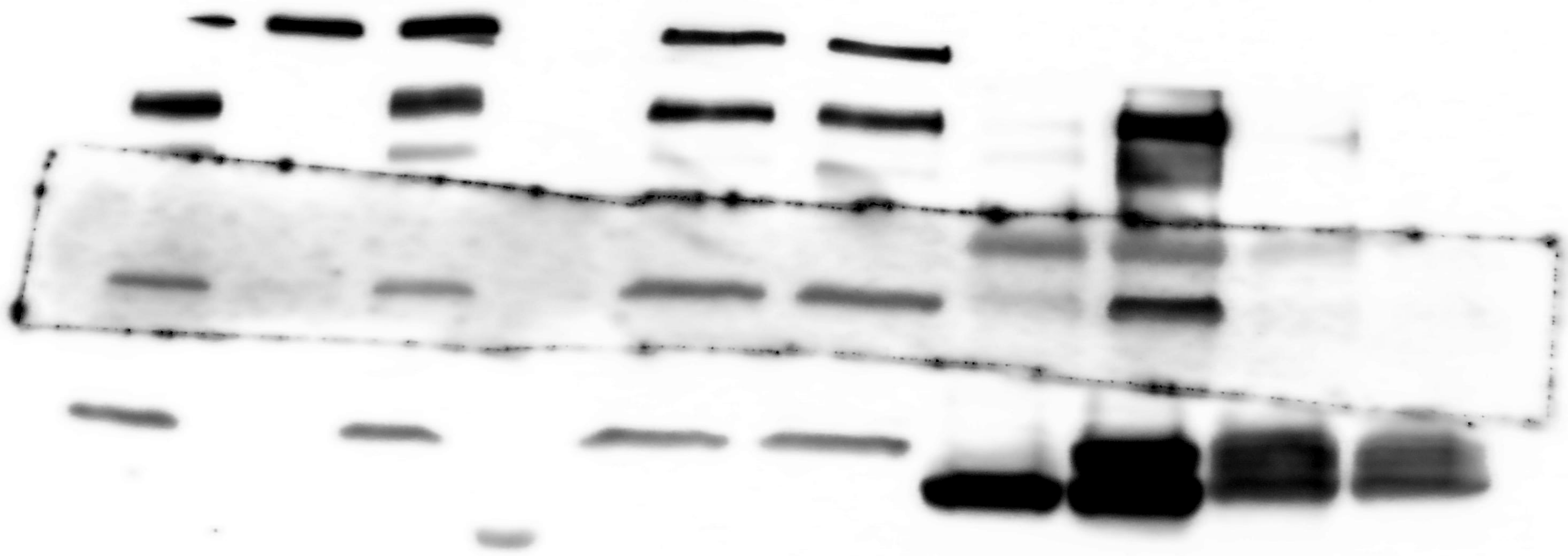

IP_liver GID8_59.644 Sec 001_NS.JPG

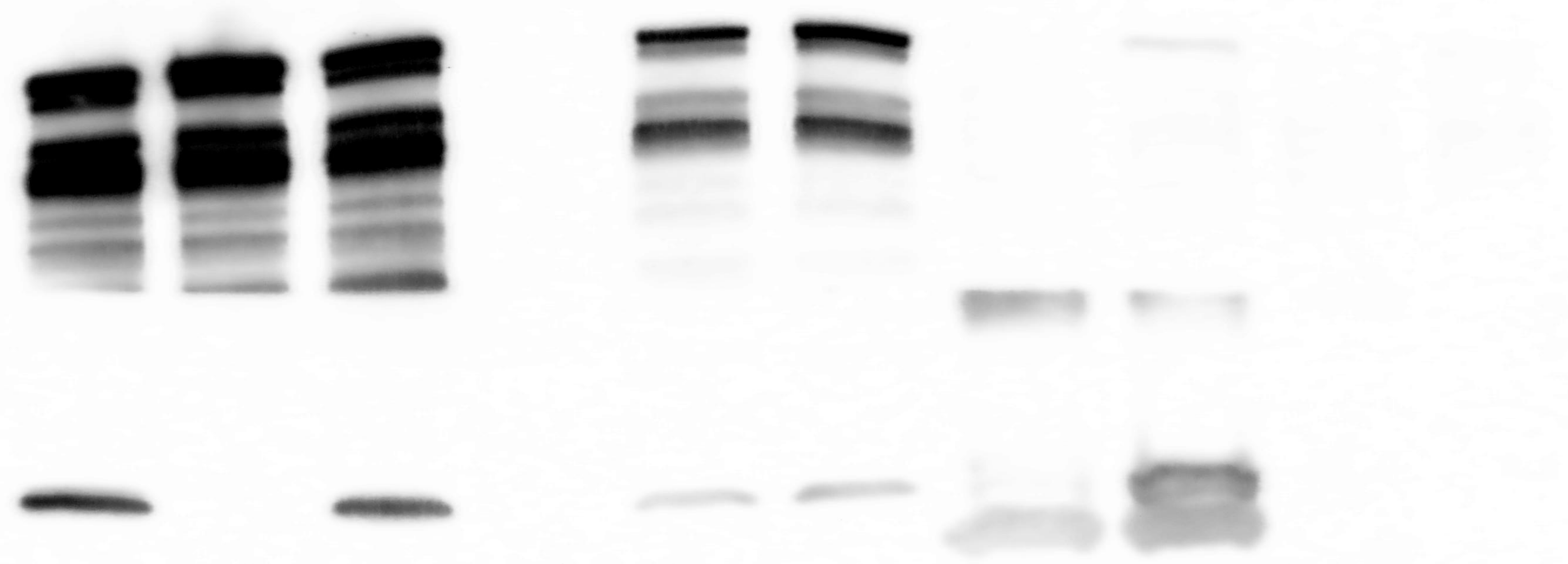

IP Liver_BMDM_reprobe_239.785 Sec 000_NS.JPG

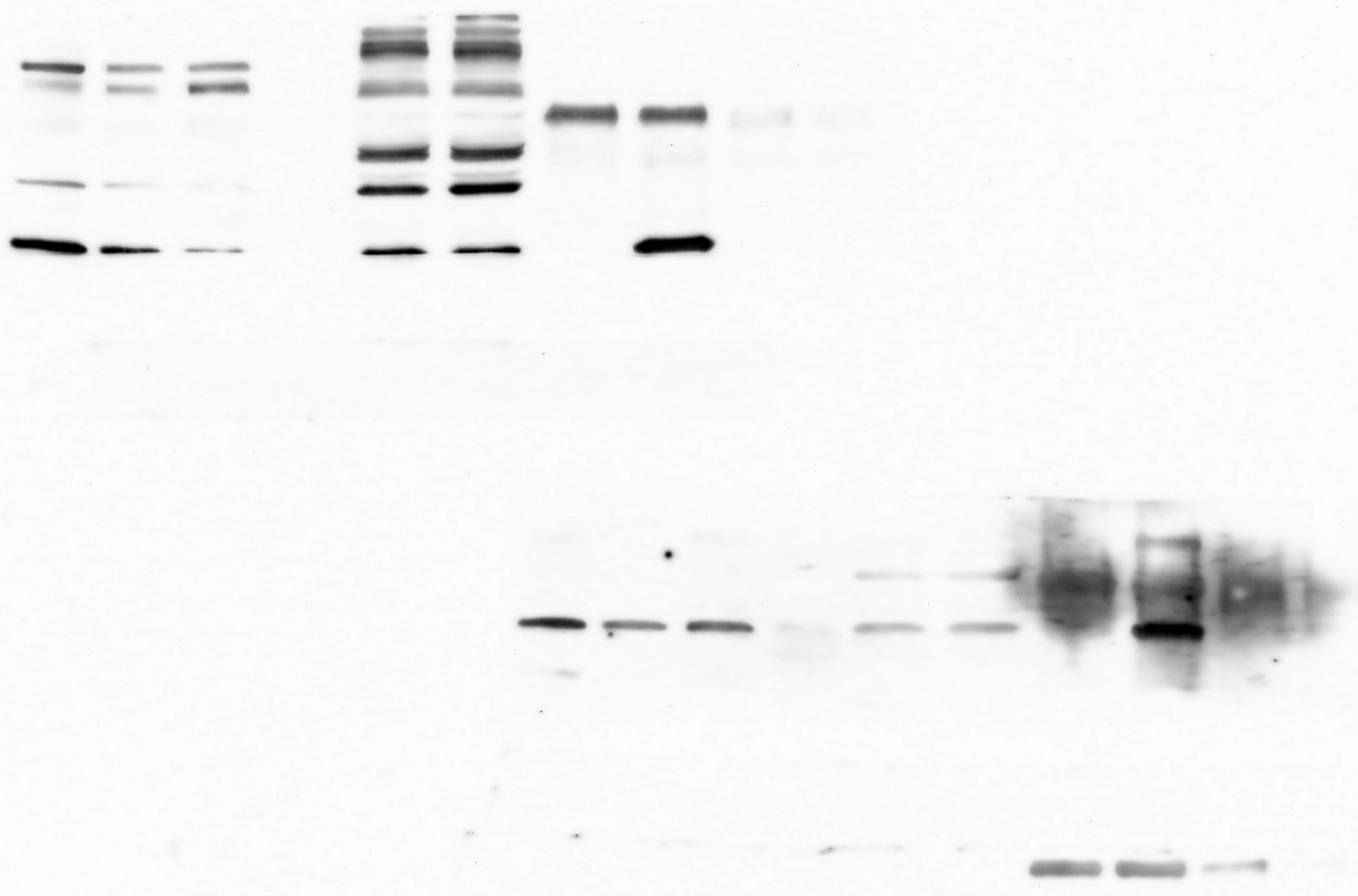

IP_liver GID8_59.644 Sec 001_NS.JPG

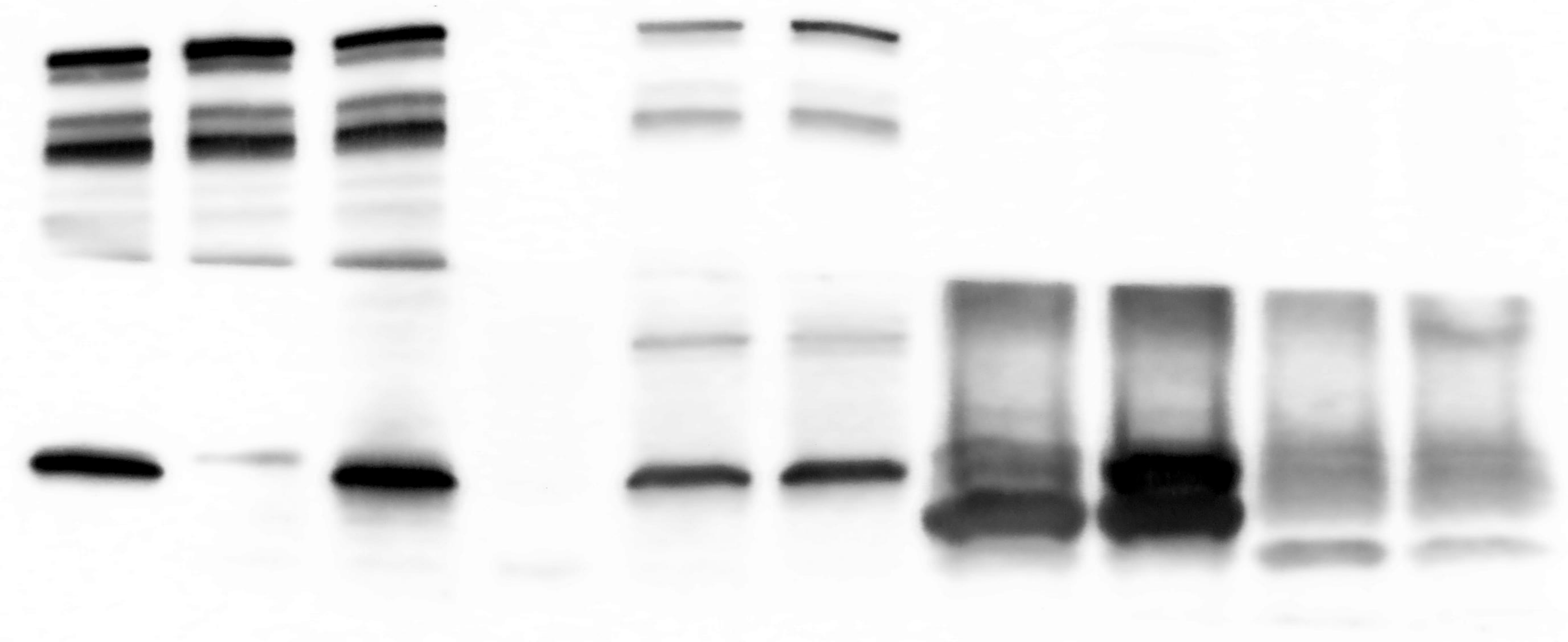

IP_liver RanBP9_119.439 Sec 000_NS.JPG

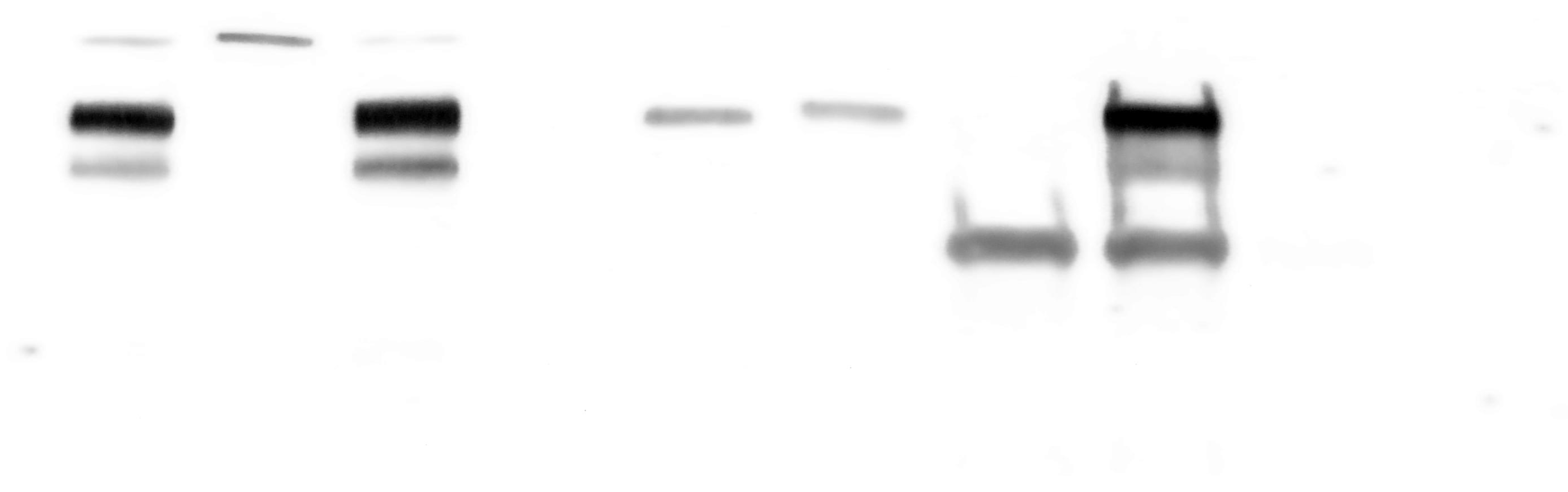

IP_liver RanBP9_59.644 Sec 000_NS.JPG

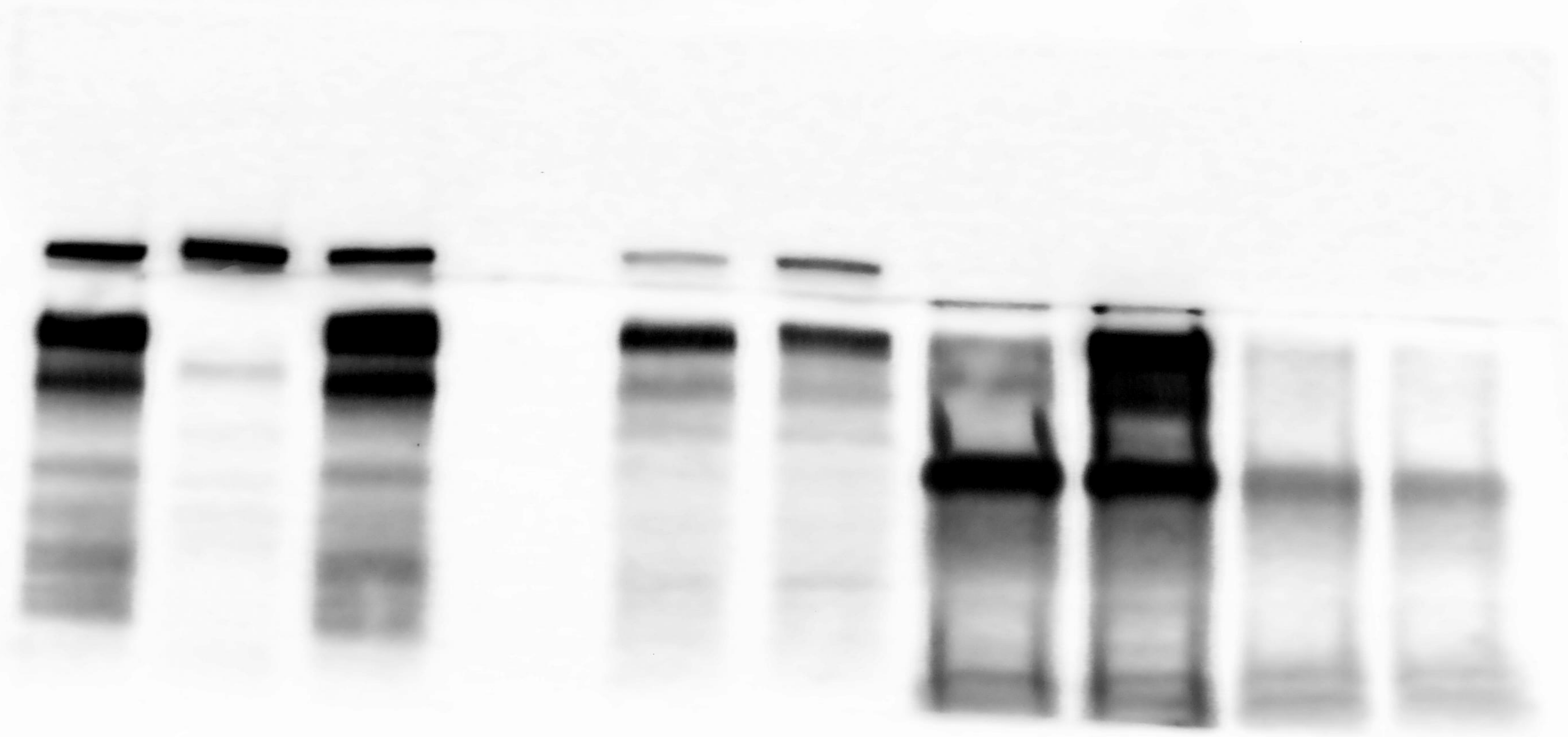

IP V5 whole V5_MAEA_GID8_119.439 Sec 000_NS.JPG

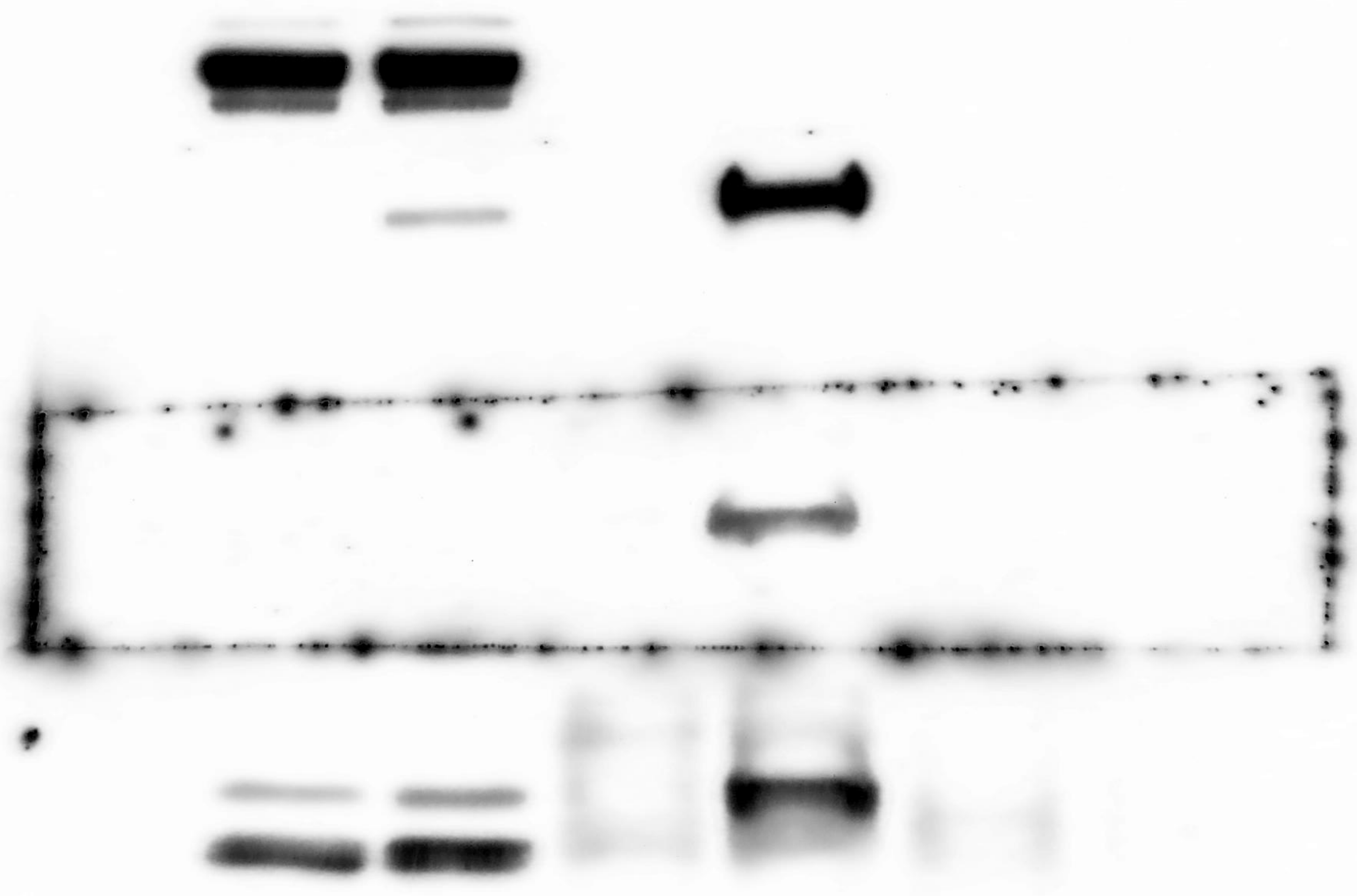

TurnX_V5 RanBP9_HA_14.798 Sec 000_NS.JPG

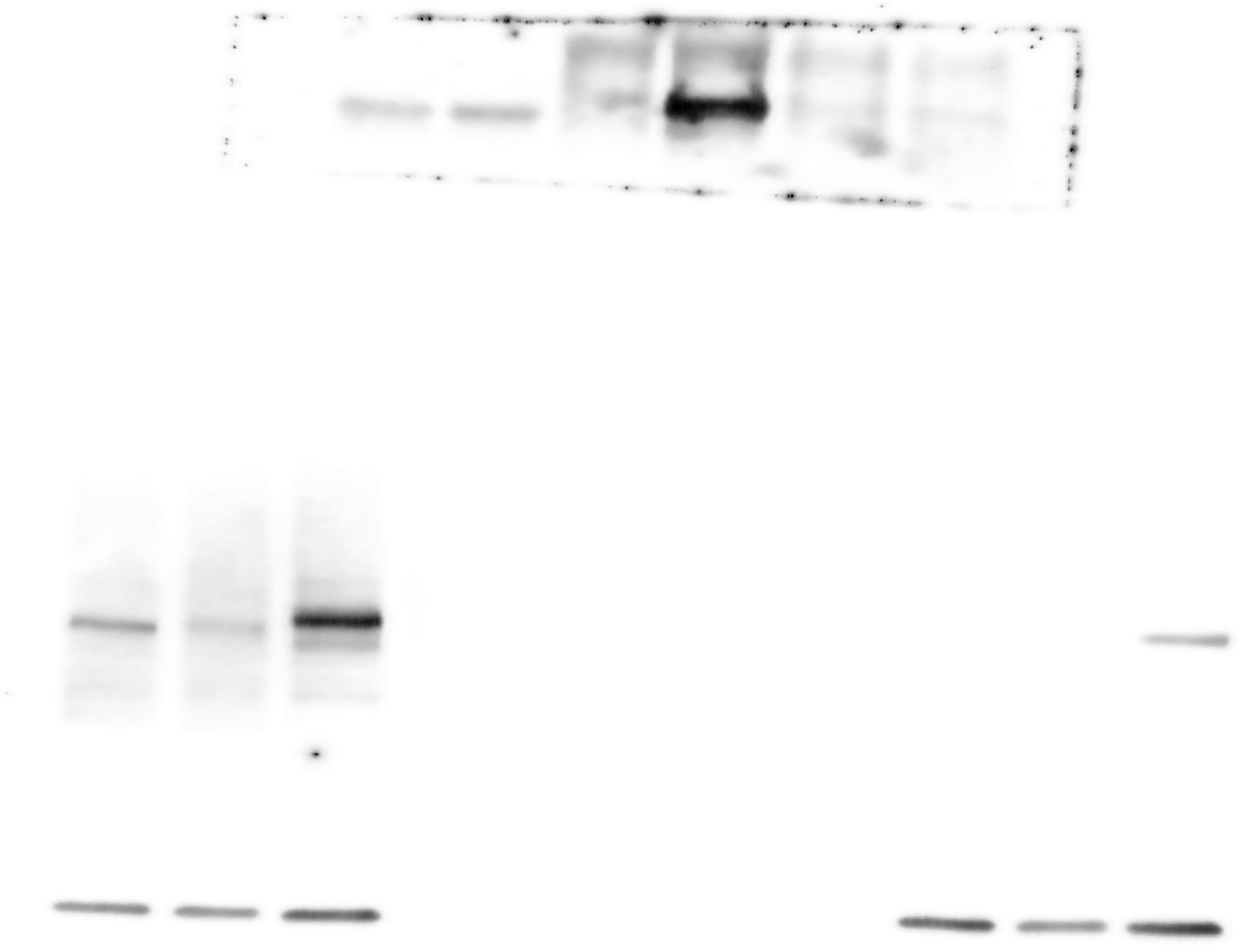

IP V5 whole V5_MAEA_GID8_239.03 Sec 0009.JPG

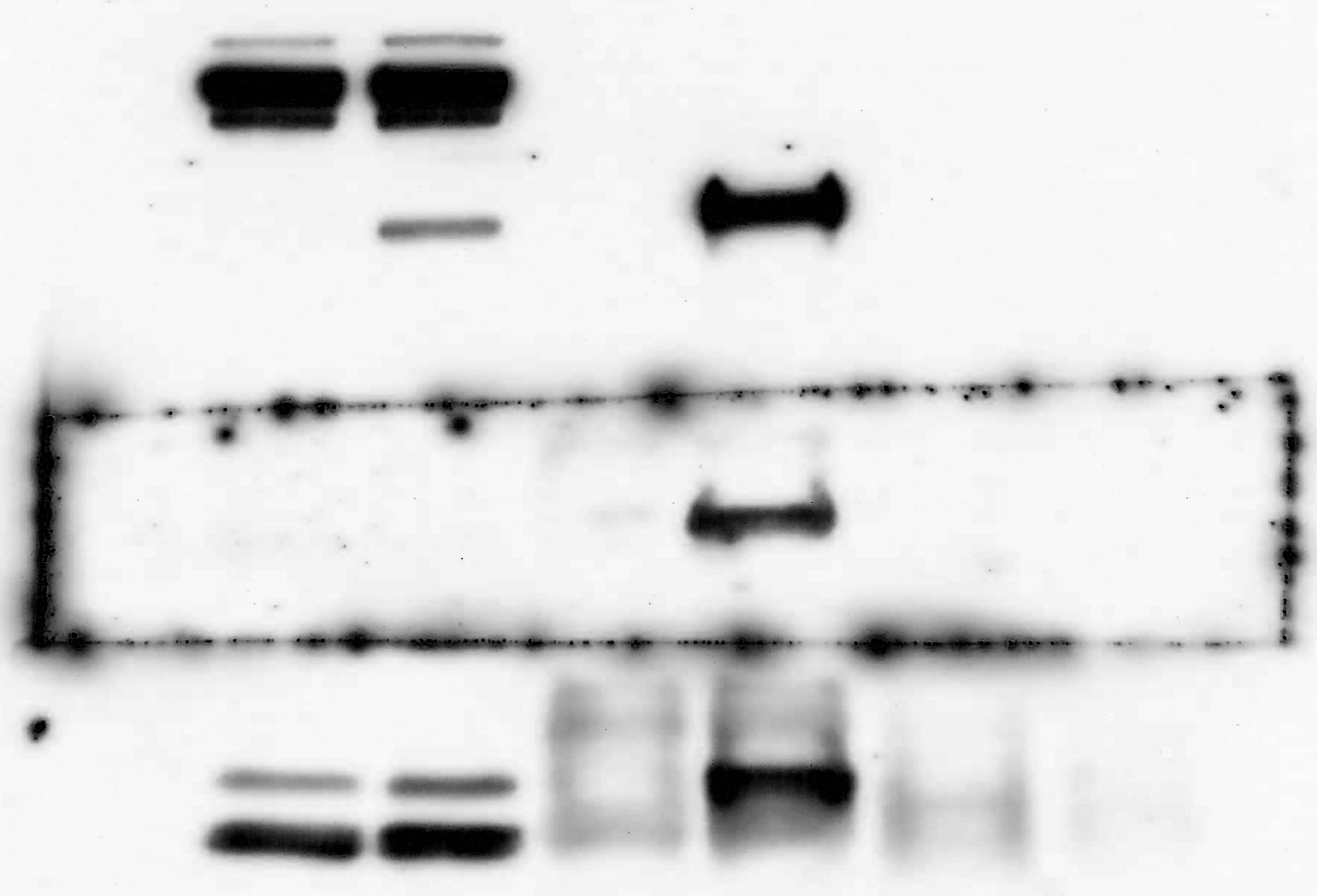

IP V5 whole V5_MAEA_GID8_7.852 Sec 0004.JPG

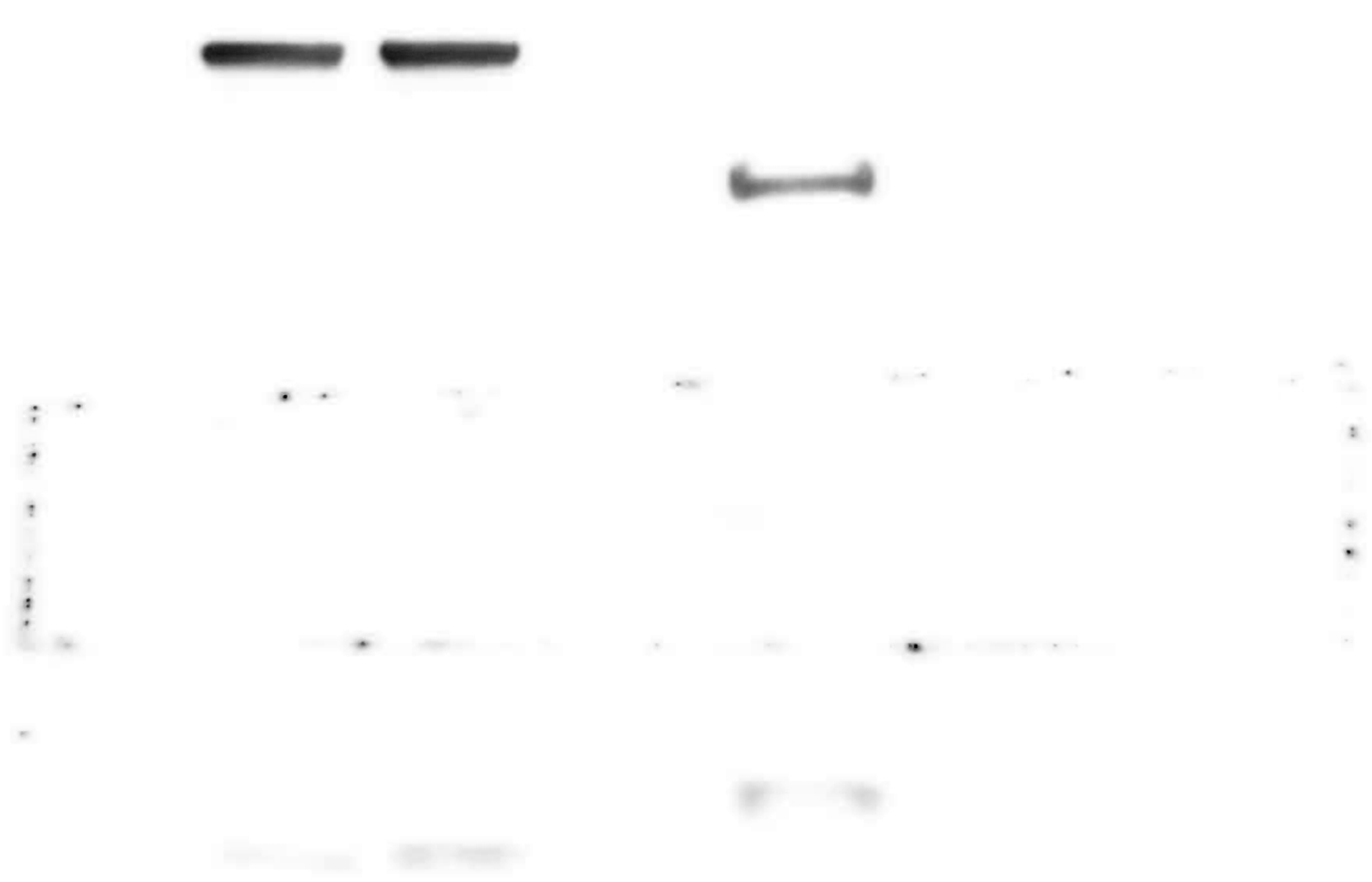

LysMXlung BP_1.812 Sec 000_NS.JPG

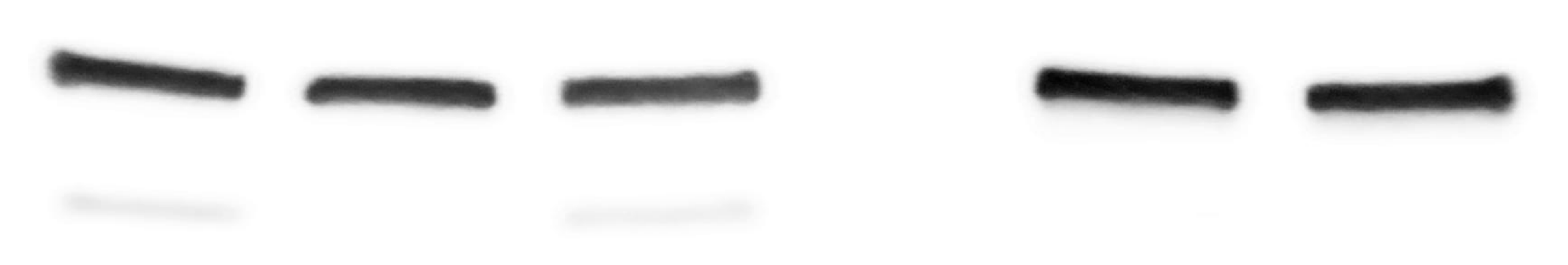

LysMX_Spleen_Lung HA_59.795 Sec 000_NS.JPG

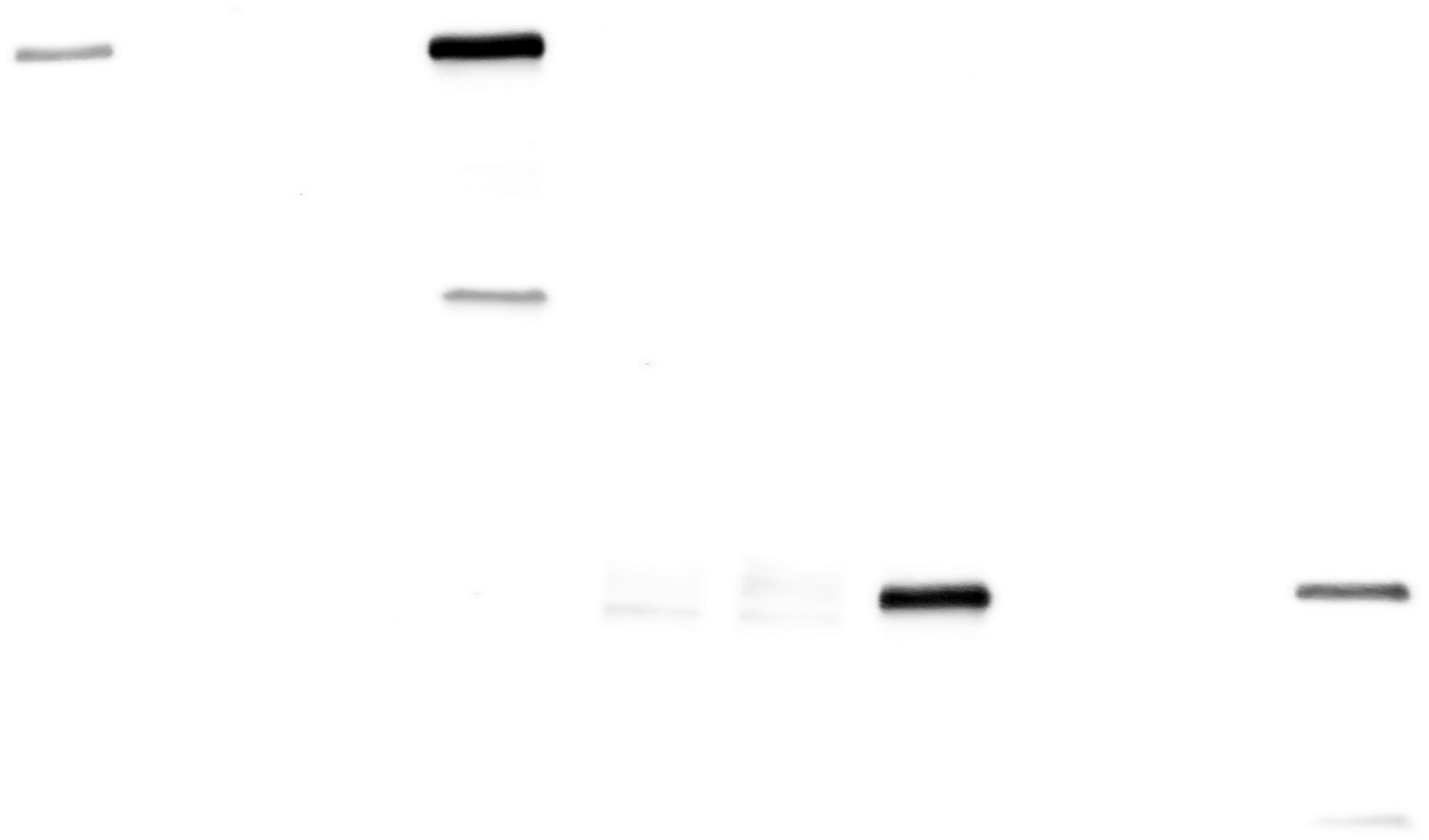

LysMX_lung BP9_119.741 Sec 000_NS.JPG

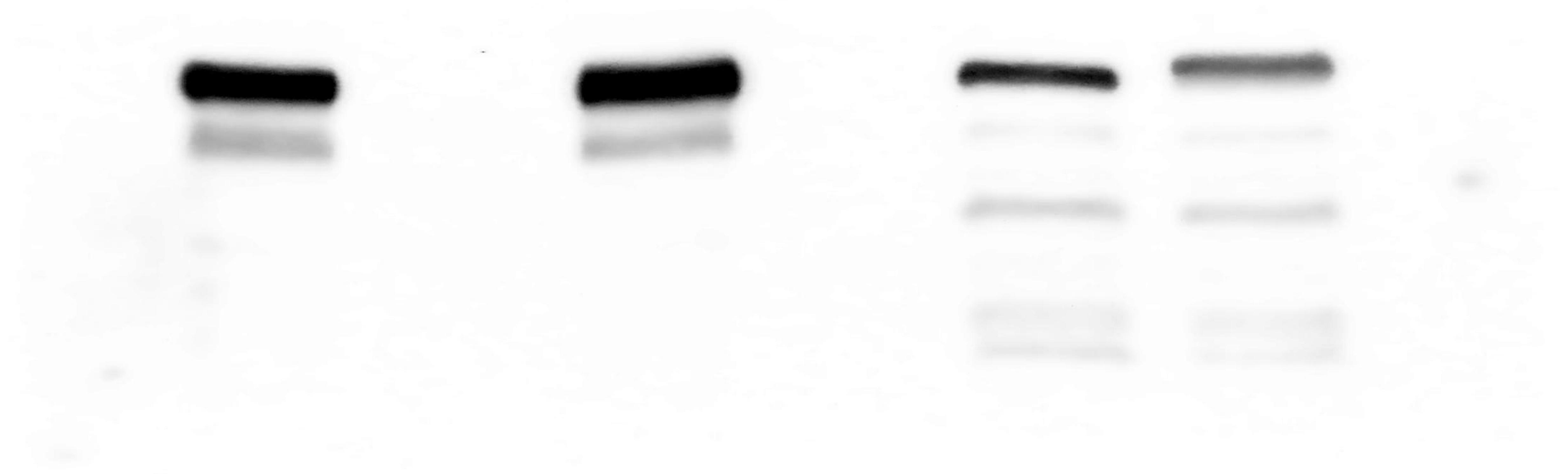

LysMX_lung V5_59.946 Sec 000_NS.JPG

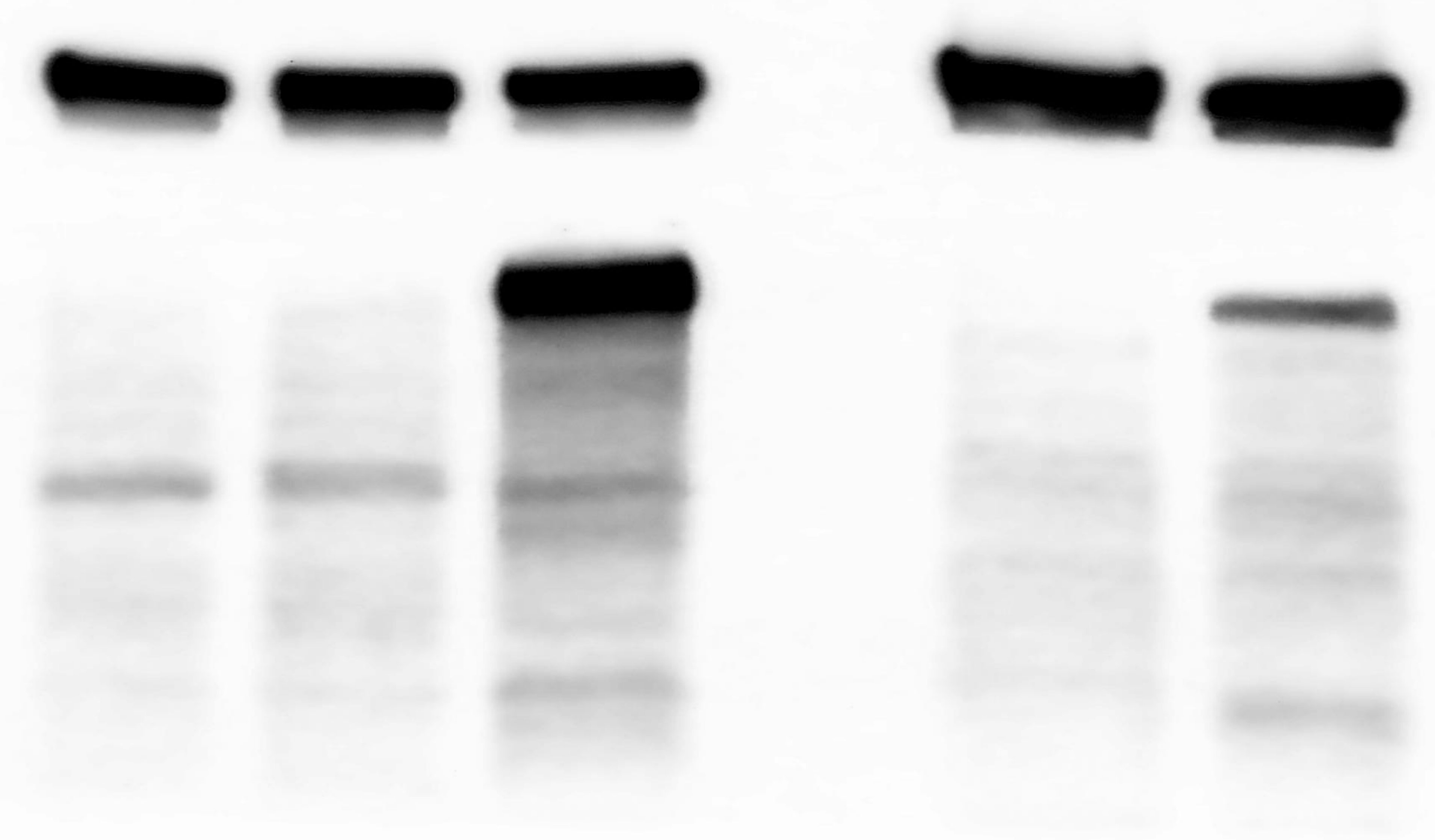

LysMX_Spleen_Lung HA_1.812 Sec 000_NS.JPG

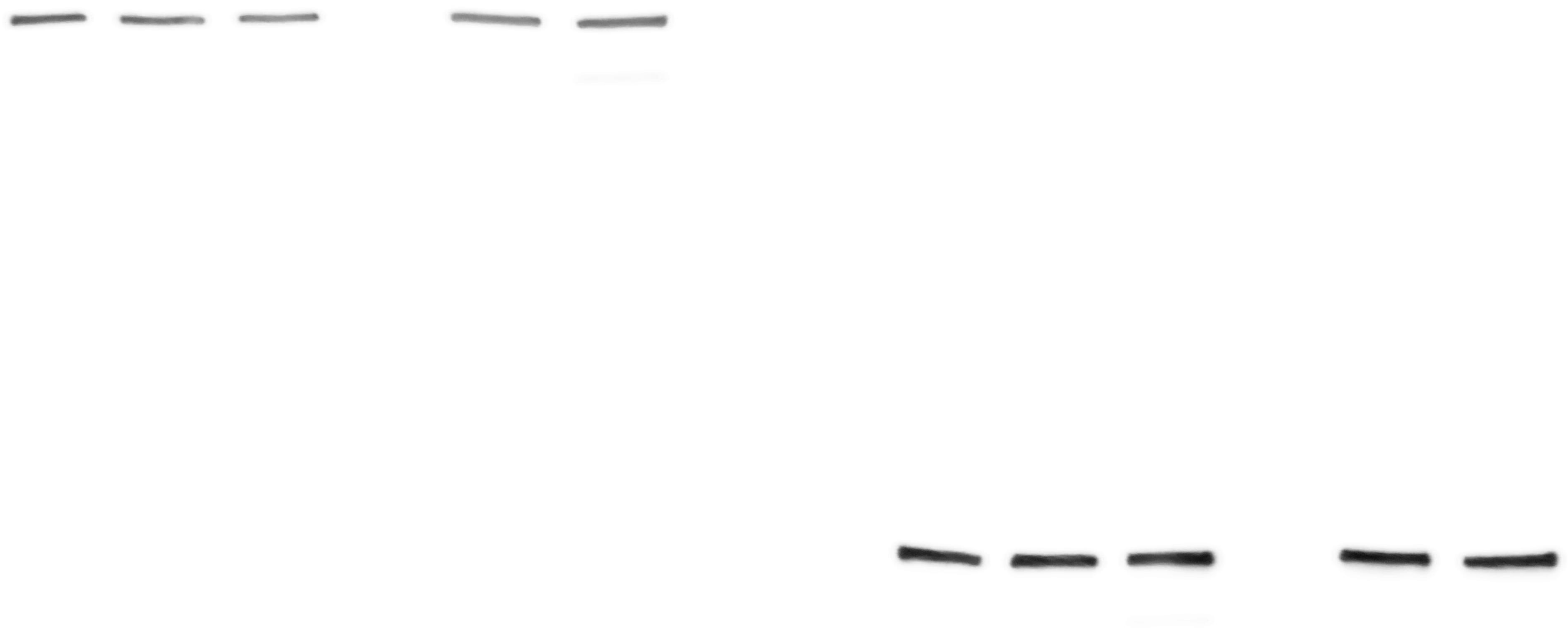

LysMX_lung V5_1.963 Sec 0002.JPG

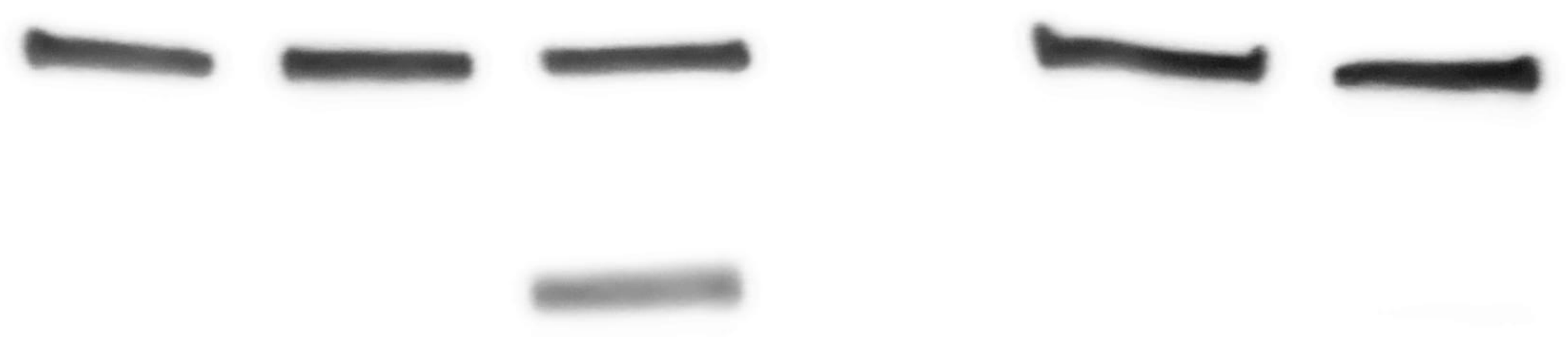

Mitochondria enrichment **EXP1**

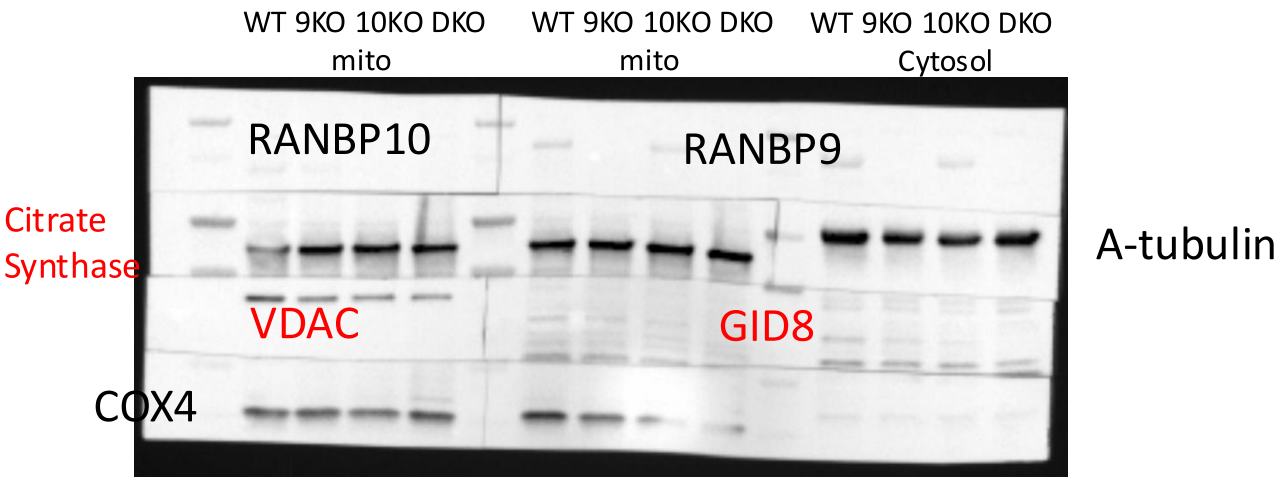

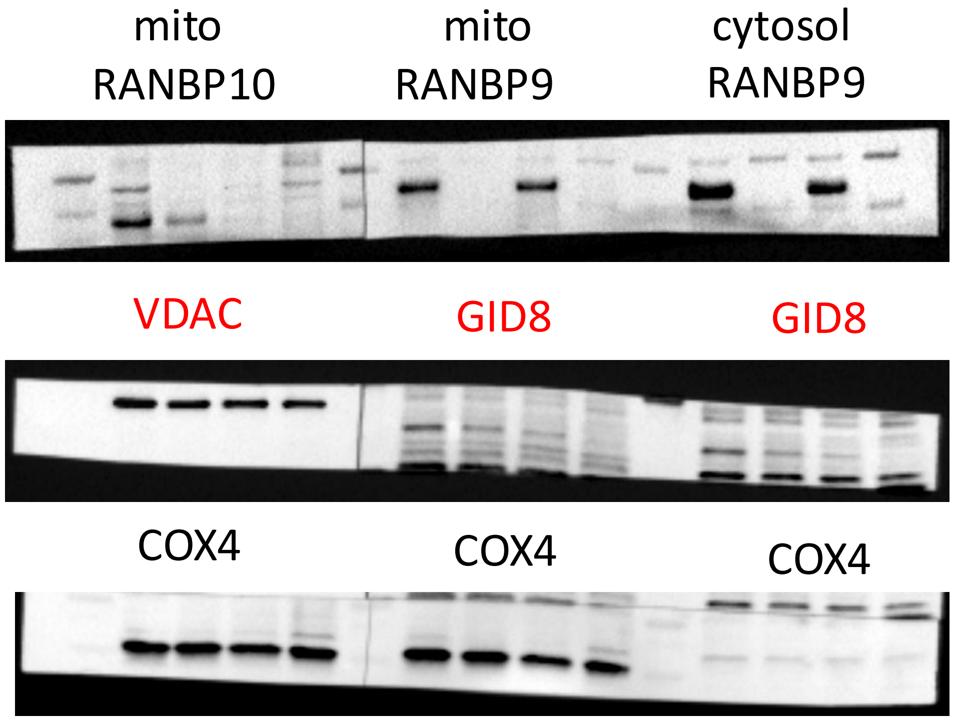

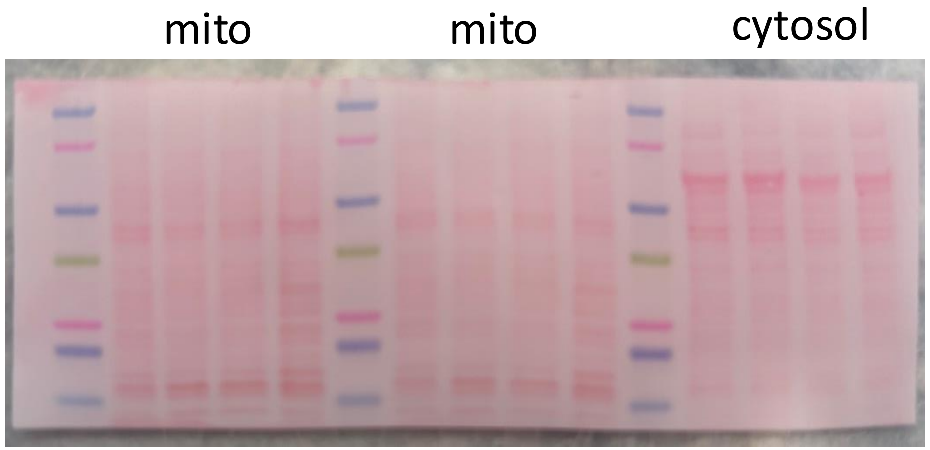

NOTE: In red, blot with antibodies not used for final figure 5.

Mitochondria enrichment **EXP2**

NOTE: In red, blot with antibodies not used for final figure 5.

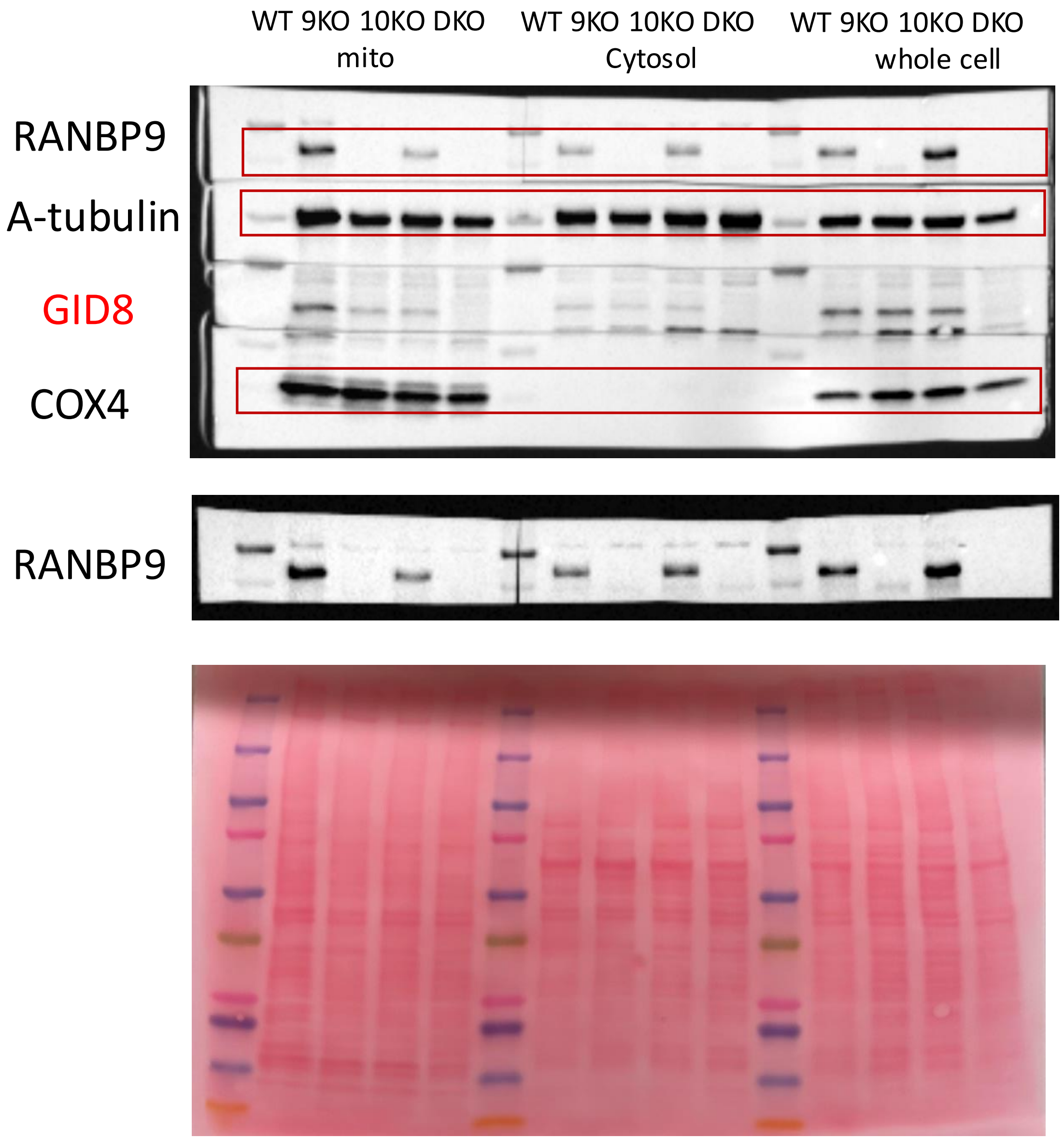

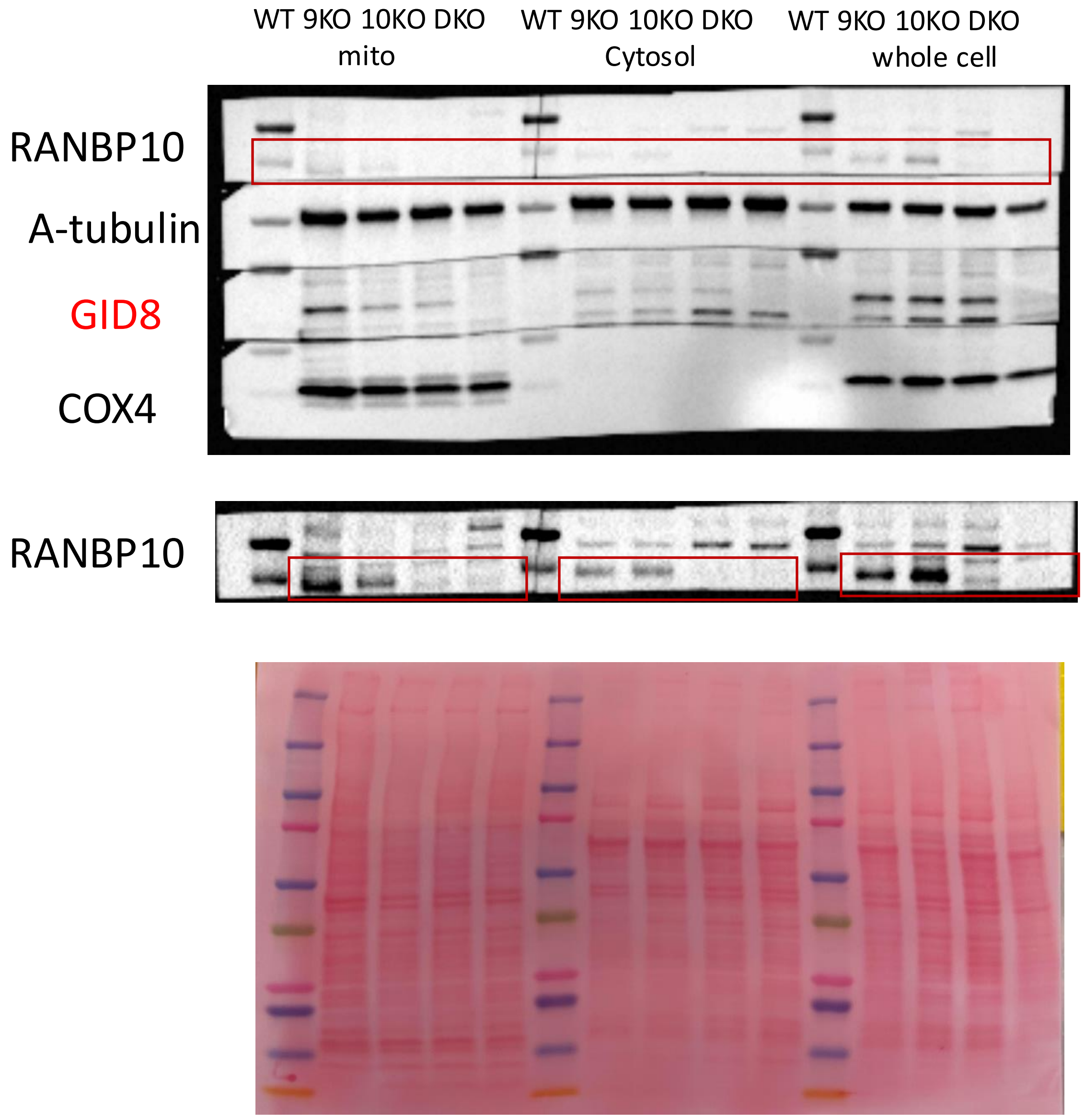

Mitochondria enrichment **EXP3**

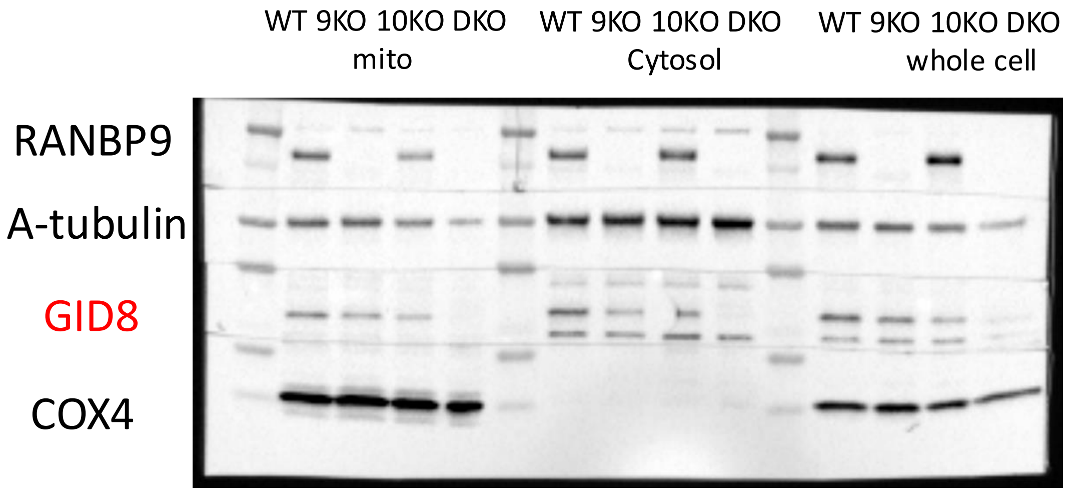

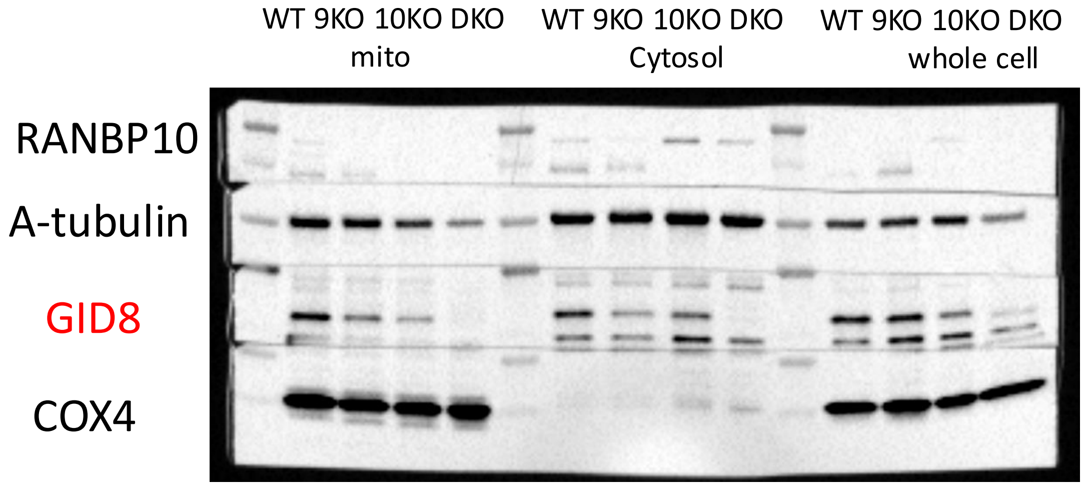

NOTE: In red, blot with antibodies not used for final figure 5.

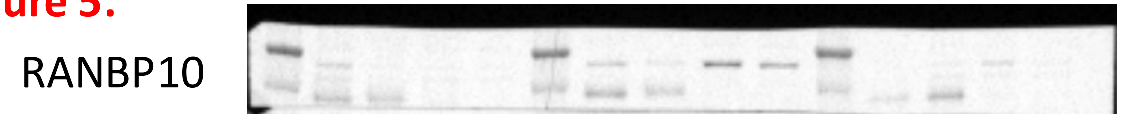

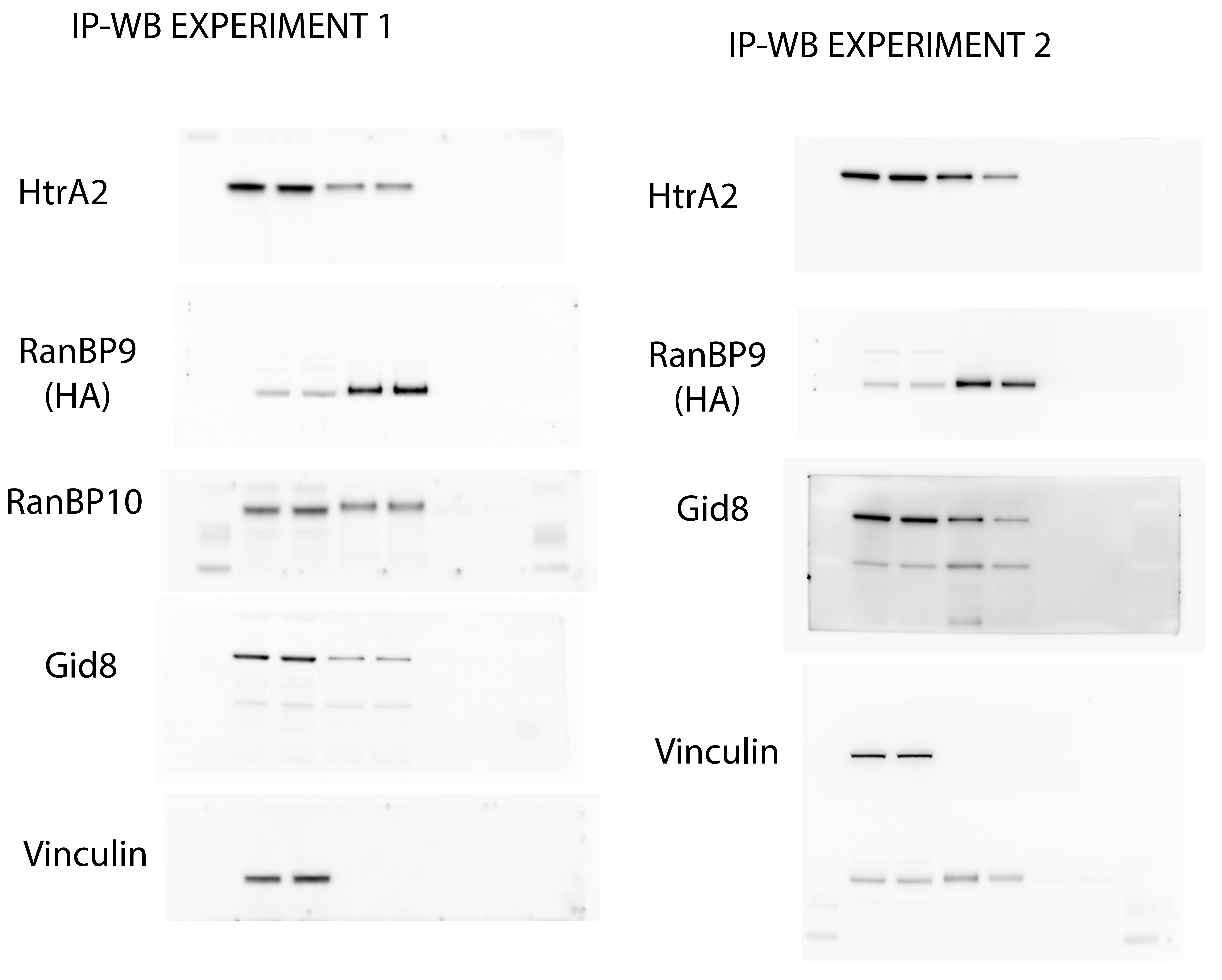
IP-WB EXPERIMENT 1
IP-WB EXPERIMENT 2
HtrA2
RanBP9
(HA)
RanBP10
Gid8
Vinculin
HtrA2
RanBP9
(HA)
Gid8
Vinculin
