# Supplementary material for: An in vivo “turning model” reveals new RanBP9 interactions in lung macrophages
Source: Cell Death Discov. 2025 Apr 13;11:171. doi: 10.1038/s41420-025-02456-2 (PMC11994786; doi:10.1038/s41420-025-02456-2)
Supplement: Supplementary file 1 — Supplementary Material [file 41420_2025_2456_MOESM1_ESM.pdf]

An in vivo “turning model” reveals new RanBP9 interactions in lung macrophages.

Supplementary Figure Legends

Supplementary Figure 1**. **Generation of the RanBP9-TurnX allele.

A) The 3x copies of HA are inserted right before the RanBP9 STOP codon of the last coding exon. **B**) Guide RNA used to target RanBP9 insertion site (same as Soliman *et al.*, 2020). **C**) Sequence of the ssODN used to engineer the RanBP9-TurnX allele. **D**) Sanger sequencing of RanBP9-Turnx homozygous animals confirms the correct insertion of the loxP-3xHA-loxP-V5 cassette as designed. **E**) Forward and reverse primers used to genotype RanBP9-TurnX animals. They amplify a product of 256 bp from the RanBP9 WT allele and a 455 bp product form the RanBP9-TurnX allele, respectively. **F**) RanBP9-TurnX heterozygous parents produce the expected numbers of WT, heterozygous, and homozygous male and female animals born from RanBP9 heterozygous parents. RanBP9-TurnX homozygous animals are fertile and do not show any obvious phenotype.

Supplementary Figure 2**. **Cre-lox recombination in RanBP9-TurnX mice turns RanBP9-3xHA into RanBP9-V5. A) Sanger sequencing of RanBP9-V5 turned homozygous animals confirms the excision of the loxP-3xHA-loxP cassette as designed. B) RanBP9-V5 heterozygous turned parents produce the expected numbers of WT, heterozygous, and homozygous animals with no gender bias. RanBP9-TurnX homozygous animals are fertile and do not show any obvious phenotype. C) RanBP9-V5 homozygous animals do not show any significant difference in body weight compared to WT animals measured at 2 (2M) and 3 (3M) months of age.

Supplementary Figure 3. Schematic representation of immunoprecipitation experiments and proteomic analysis.

Lungs from 8–10-week-old LysMBP9 WT and LysMBP9X mice were harvested. Single cell suspensions were obtained as described in Materials and Methods, and lysed for protein extraction. Each mouse lung lysate was immunoprecipitated with magnetic beads conjugated to IgG (isotype control), or anti-HA, or anti-V5, each in quadruplicate. The 96 samples were stored at -80°C until they were analyzed by mass spectrometry all at the same time. To identify positive RanBP9 interactors, anti-HA-immunoprecipitated fractions from LysMBP9X mice (red) were compared to IgG- and anti-HA-immunoprecipitated fractions (black) from LysMBP9 WT mice plus IgG immunoprecipitated from LysMBP9X mice (green). Similarly, anti-V5-immunoprecipitated fractions from LysMBP9X mice (blue) were compared to IgG- and anti-HA-immunoprecipitated from LysMBP9 WT mice (black) plus IgG-immunoprecipitated fractions from LysMBP9X mice (green).

A

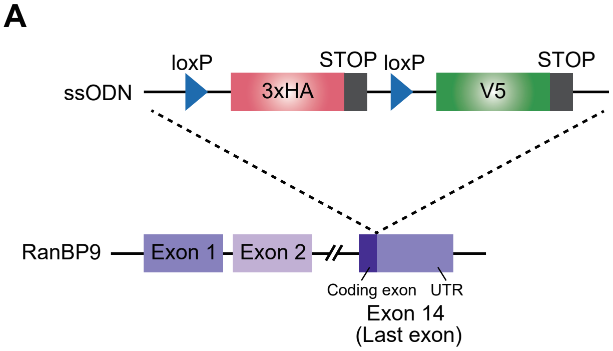

B

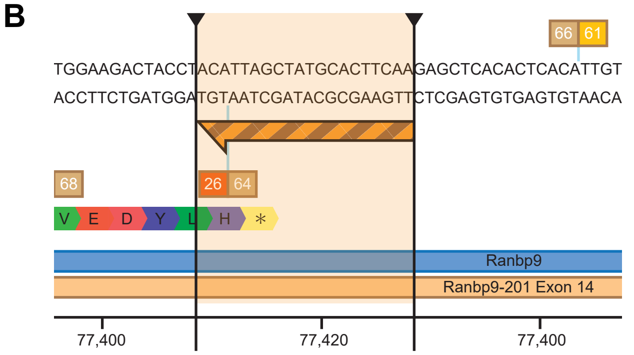

Position	Sequence	PAM	On-Target Score	Off-Target Score
77412	TTGAAGTGCATAGCTAATGT	AGG	26.2	64.2

C

GGTCAGGAGTTGGGTCCTGTGCATTTGCCACAGTGGAAGACTACCTACATATAACTTCGTATAGCCTACATTATACGAAGTTATCT
TACCCATACGATGTTCCAGATTACGCTTACCCATACGATGTTCCAGATTACGCTTACCCATACGATGTTCCAGATTACGCTTAGCAT
AACTTCGTATAGCCTACATTATACGAAGTTATCTGGTAAGCCTATCCCTAACCCTCTCCTCGGTCTCGATTCTACGTAGCTATGCAC
TTCAAGAGCTCACACTCACATTGTGGCAAACA

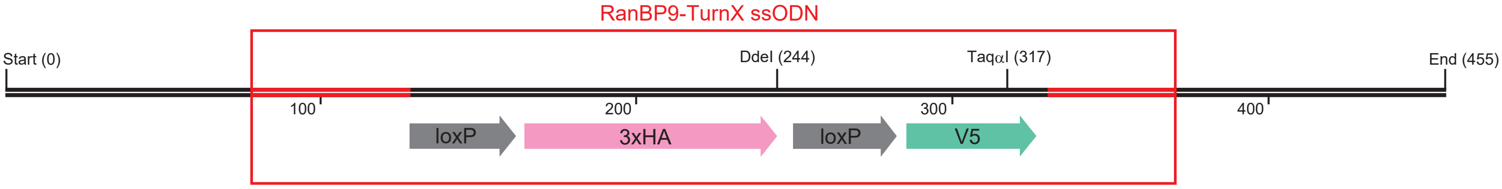

D

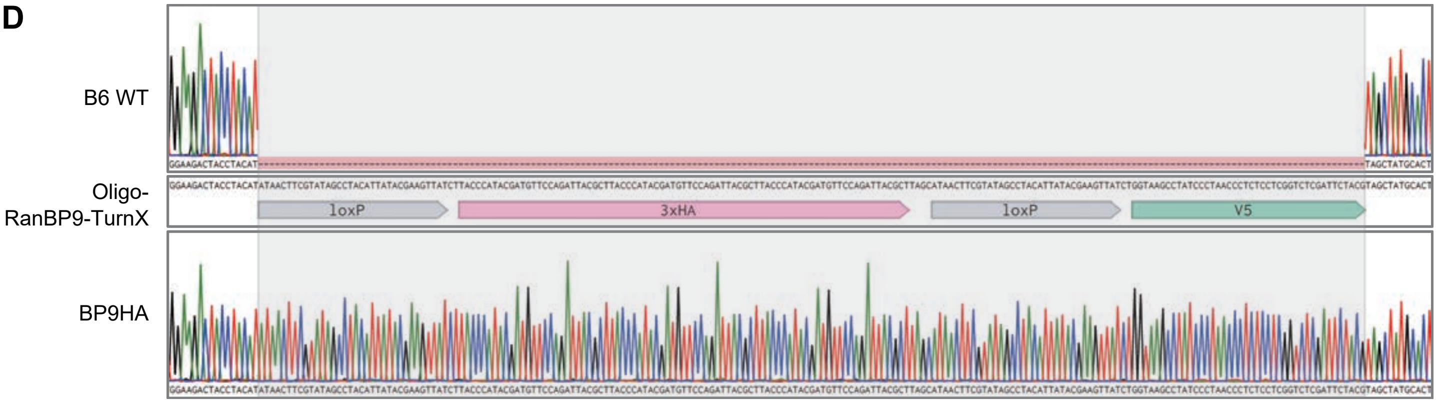

E

Forward Primer: **AAACCCACAATCTGCCAAAG**
Reverse Primer: **AAAGCGACAAAAACCTGTCC**

WT: **256 bp**
RanBP9-TurnX: **455 bp**
RanBP9-TurnX (Cre+): **334 bp**

F

Female breeder genotype		Male breeder genotype	PROGENY GENOTYPE				
HET	x	HET		WT	HET	HOMO	Tot
			Females	3	2	2	7
			Males	1	5	4	10
			Tot	4	7	6	17
HOMO	x	HOMO		WT	HET	HOMO	Tot
			Females	0	0	3	3
			Males	0	0	3	3
			Tot	0	0	6	6

A

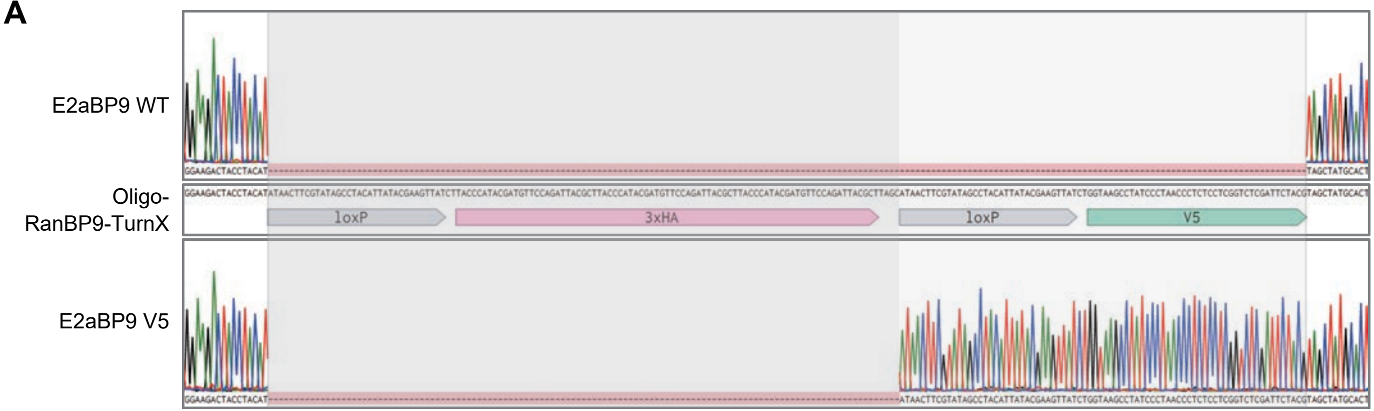

B

Female breeder genotype		Male breeder genotype	PROGENY GENOTYPE				
HET	x	HET		WT	HET	HOMO	Tot
			Females	4	9	6	19
			Males	4	4	4	12
			Tot	8	13	10	31
HOMO	x	HOMO		WT	HET	HOMO	Tot
			Females	0	0	20	20
			Males	0	0	28	28
			Tot	0	0	48	48

C

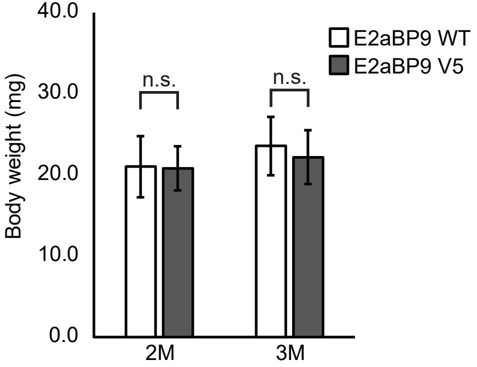

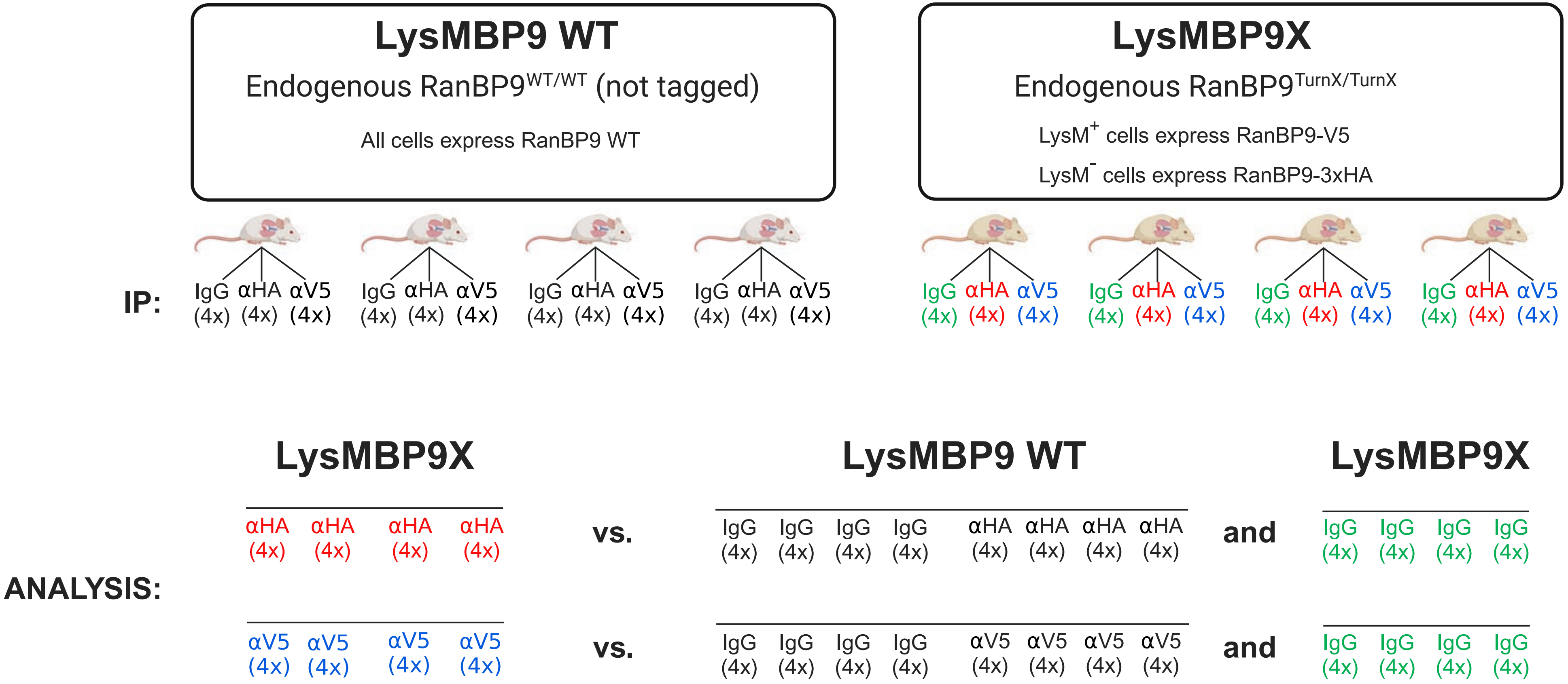
LysMBP9 WT
Endogenous RanBP9WT/WT (not tagged)
All cells express RanBP9 WT
LysMBP9X
Endogenous RanBP9TurnX/TurnX
LysM+ cells express RanBP9-V5
LysM- cells express RanBP9-3xHA
IP:
IgG (4x) αHA (4x) αV5 (4x)
ANALYSIS:
LysMBP9X
αHA (4x) αHA (4x) αHA (4x) αHA (4x)
vs.
LysMBP9 WT
IgG (4x) IgG (4x) IgG (4x) IgG (4x) αHA (4x) αHA (4x) αHA (4x) αHA (4x)
and
LysMBP9X
IgG (4x) IgG (4x) IgG (4x) IgG (4x)
αV5 (4x) αV5 (4x) αV5 (4x) αV5 (4x)
vs.
IgG (4x) IgG (4x) IgG (4x) IgG (4x) αV5 (4x) αV5 (4x) αV5 (4x) αV5 (4x)
and
IgG (4x) IgG (4x) IgG (4x) IgG (4x)

Legend

Supp Table 1: **3xHA IP raw data**

Supp Table 2: **3xHA IP *p* value**

Supp Table 3: **V5 IP raw data**

Supp Table 4: **V5 IP *p* value**

Supp Table 5: **List of common hits between RanBP9-3xHA- and RanBP9-V5 immunoprecipitatate**

Supp Table 6: **List of previously reported CTLH interactors and references (PMID).**

Supp Table 7: **List of RanBP9-3xHA interactors previously reported.**

Supp Table 8: **List of RanBP9-V5 interactors previously reported.**

d proteins

Suppl Table 1. 3xHA IP raw data.

Accession	Descriptio	Sum PEP S	Coverage	# Peptides	# PSMs	# Unique P	# AAs	MW [kDa]
Q8VDD5	Myosin-9 (	1741.064	73	198	14815	150	1960	226.2
Q02566	Myosin-6 (	1531.969	69	167	7492	57	1938	223.4
Q9QXS1	Plectin OS	1288.274	52	253	4548	249	4691	533.9
Q6URW6	Myosin-14	1120.505	63	145	7223	132	2000	228.4
B1ARU4	Microtubu	956.398	31	189	1832	40	7353	831.1
Q91Z83	Myosin-7 (	955.438	47	111	4546	7	1935	222.7
A0A338P6	Myosin-11	881.599	55	131	5286	2	1979	227.7
E9QPE7	Myosin-11	878.457	55	131	5319	2	1972	227
Q3UH59	Myosin-10	853.282	52	124	4885	92	2013	233.3
A3KGU9	Spectrin a	852.836	58	136	2329	1	2498	287.4
P16546	Spectrin a	848.773	58	136	2319	1	2472	284.4
F7ACR9	Microtubu	806.248	35	155	1623	4	5333	607.9
Q62261	Spectrin b	682.526	54	110	1909	107	2363	274.1
E9Q616	AHNAK nu	662.858	46	116	1317	116	5656	603.9
Q9JKF1	Ras GTPas	617.768	53	76	2701	73	1657	188.6
Q8BTM8	Filamin-A	569.518	45	87	1479	79	2647	281
E9Q6R7	Utrophin (	468.903	31	91	1232	89	3430	392.5
Q9WTI7	Unconvent	466.042	64	68	3212	67	1063	121.9
P26039	Talin-1 OS	448.779	41	83	1129	70	2541	269.7
S4R1P5	Dystonin (	426.983	16	101	607	97	7717	870
Q9JMH9	Unconvent	414.161	43	74	916	74	2050	232.6
P46735	Unconvent	406.581	53	61	2237	1	1107	128.5
E9QNH6	Unconvent	405.284	55	61	2206	1	1078	125
Q9JHU4	Cytoplasm	365.145	21	84	710	84	4644	531.7
E9Q175	Unconvent	355.94	50	57	1184	57	1253	144.7
A0A1L1SQ	Dedicator	299.704	31	52	818	49	2111	236.4
B9EHJ3	Tight junct	293.801	30	46	1243	1	1685	188.7
P39447	Tight junct	288.91	29	46	1231	1	1745	194.6
Q5SYD0	Unconvent	284.573	53	53	1375	51	1006	116
Q00PI9	Heterogen	274.704	47	38	1082	38	745	84.9
F6XCT0	Microtubu	272.433	34	52	504	1	1837	211.6
Q5SX40	Myosin-1 (	263.814	16	38	1313	3	1942	223.2
F8WID5	Tropomyo:	255.106	71	44	1709	3	326	37.4
Q3UQ28	Peroxidasi	249.929	36	43	736	36	1475	165
Q68FD5	Clathrin he	249.838	29	42	487	42	1675	191.4
Q9JHL1	Na(+)/H(+	248.73	68	27	1524	27	337	37.4
Q61554	Fibrillin-1	248.398	23	47	300	47	2873	312.1
Q3U0S6	Ras-intera	244.227	44	33	1125	33	961	103.5
A0A0R4J0	DNA-direc	242.793	29	47	409	47	1932	213.3
Q7TPV4	Myb-bindi	242.201	36	40	555	40	1344	151.9
A0A1D5RL	Unconvent	239.717	32	46	369	45	1963	222.5
Q6ZPE2	Myotubula	237.984	29	47	673	46	1867	208.6

Q6ZPQ6	Membrane	231.824	43	38	660	38	1335	147.9
P57780	Alpha-acti	227.341	47	34	504	19	912	104.9
E9Q450	Tropomyo:	227.032	69	40	1560	2	284	32.8
Q6P9Q4	FH1/FH2 c	225.194	38	38	593	38	1197	129.5
Q9JJ28	Protein flig	224.838	38	39	512	39	1271	144.7
H3BJU7	Guanine n	220.569	48	41	660	41	956	108.5
Q7TPR4	Alpha-acti	220.06	46	36	503	3	892	103
Q6A0A9	Constitutiv	215.986	42	33	535	32	1112	121.6
P20152	Vimentin (	213.417	71	34	728	31	466	53.7
F6SMY7	RCR-type I	211.161	14	52	420	52	4636	509
Q8CFI7	DNA-direc	210.729	38	40	457	40	1174	133.8
E9PUF7	Rho guani	209.129	39	32	586	32	976	108.6
Q8CGB6	Tensin-2 C	202.709	34	36	518	36	1400	151.9
G5E8G6	Unconvent	202.173	26	38	395	34	1818	210.4
Q61656	Probable A	200.823	54	31	1420	22	614	69.2
E9Q5J9	Tropomyo:	198.624	63	37	1227	1	285	33.3
Q62167	ATP-deper	197.188	50	30	801	10	662	73.1
P13541	Myosin-3 (	196.915	13	31	1245	1	1940	223.7
Q9JIK5	Nucleolar	196.711	49	36	609	36	851	93.5
Q9CZJ2	Heat shocl	193.864	49	30	819	27	685	76.1
E9Q7Q3	Tropomyo:	193.324	66	30	1423	6	248	28.7
O54988	STE20-like	193.24	25	39	842	38	1233	141.4
P35441	Thrombos	192.767	43	34	267	33	1170	129.6
P26041	Moesin OS	191.548	50	38	1164	26	577	67.7
E9Q8I7	Nuclear tr	190.88	44	30	622	30	918	101.4
Q8CIZ8	von Willeb	190.823	19	35	221	35	2813	309.1
A2AIM4	Tropomyo:	186.807	66	35	1172	20	284	33
O08692	Neutrophi	186.264	77	23	1562	23	167	19.3
Q8VEK3	Heterogen	186.063	42	29	843	29	800	87.9
P13020	Gelsolin O	182.737	38	22	840	7	780	85.9
Q80UE5	Band 4.1-l	180.033	32	29	607	26	918	101.8
Q8CG29	Myosin-1F	179.548	35	39	878	31	1098	125.1
Q64012	RNA-bindi	179.282	70	25	938	25	312	33.2
A2AJ88	Patatin-lik	176.981	30	34	388	32	1352	150.4
D3Z2H9	Tropomyo:	175.871	63	31	1359	0	248	29
A0A0R4J2	Enhancer c	175.655	53	27	485	26	833	93
Q61140	Breast car	172.1	40	25	322	24	874	94.2
Q61789	Laminin su	170.712	17	40	300	40	3330	366
E9Q0S6	Tensin 1 C	170.616	28	35	265	35	1888	201.3
Q64727	Vinculin O	170.149	35	33	568	33	1066	116.6
E9Q448	Tropomyo:	169.024	65	32	1229	2	284	32.8
Q6ZPF4	Formin-lik	168.519	31	34	652	27	1028	117.1
P62960	Y-box-binc	167.395	58	18	694	13	322	35.7
E9Q456	Tropomyo:	166.789	69	28	1251	5	248	28.7
Q5SUA5	Unconvent	163.892	37	31	419	29	1024	117.2

F8VPU2	FERM, AR	163.565	35	27	851	27	1048	118.8
A0A140LI5	Patatin-lik	161.688	31	32	382	1	1345	148.6
Q9WVB4	Slit homol	161.603	29	31	395	31	1523	167.6
A0A087W	Collagen, t	161.457	16	35	284	35	2677	288.5
Q3TRM4	Patatin-lik	160.068	31	32	370	1	1355	149.4
P68033	Actin, alph	159.118	57	21	2588	7	377	42
A2AHK0	Diacylglyce	155.837	32	28	808	5	1123	125
Q8BMK4	Cytoskelet	155.123	52	31	508	31	575	63.7
Q61001	Laminin su	154.239	13	30	216	30	3718	403.8
Q9QZS8	SH2 doma	154.07	42	30	296	30	854	94.3
E9Q8K5	Titin OS=M	153.516	2	42	197	42	33467	3713.7
Q3UZ39	Leucine-ric	153.374	48	22	227	20	729	79.2
Q7TMM9	Tubulin be	153.241	58	19	994	1	445	49.9
Q9CWF2	Tubulin be	151.446	58	19	942	1	445	49.9
Q61164	Transcripti	151.446	32	21	415	21	736	83.7
Q80X90	Filamin-B	150.723	17	33	262	26	2602	277.7
A2AJI0	MAP7 dom	150.454	39	29	619	2	846	93.2
A2AS03	Helicase w	149.596	16	35	258	35	2970	333.8
E9Q906	Catenin de	147.913	32	23	477	23	839	93.4
Q8K284	General tr	147.42	18	26	359	26	2101	237.3
Q9R1B9	Slit homol	147.279	25	26	228	26	1521	168.7
A0A0J9YU	Actin-depo	146.558	41	16	648	1	568	62.3
A0A0A0M	Protein PR	145.993	12	26	286	26	2844	310.5
P26043	Radixin OS	145.813	46	31	627	19	583	68.5
A0A668KL	Diacylglyce	145.418	35	25	785	2	934	104.1
A0A087W	E3 ubiquit	145.172	19	30	212	30	1992	220.5
Q8BVY0	Ribosoma	145.059	46	23	314	23	452	50.4
Q6PDN3	Myosin lig	144.749	19	27	287	26	1941	212.8
Q8BGS1	Band 4.1-l	144.535	39	23	623	21	731	81.6
A8Y5P4	MAP7 dom	144.013	39	28	589	1	814	90
Q9DBG3	AP-2 comp	143.919	28	25	413	11	937	104.5
Q6IRU2	Tropomyo	143.34	67	26	781	23	248	28.5
Q501J6	Probable A	143.217	38	27	890	18	650	72.4
E9QMR2	Dedicator	142.91	20	31	271	30	2058	234.8
A0A286YD	Myoferlin	142.729	20	30	281	30	2061	234.4
Q9EQK5	Major vau	141.234	40	29	384	29	861	95.9
A0A5F8M	Periaxin (F	140.728	24	27	299	27	1525	162.3
P12970	60S riboso	140.718	58	20	891	20	266	30
A2AEX6	Four and a	140.085	63	19	819	19	309	35.1
P27659	60S riboso	140.033	47	21	745	21	403	46.1
Q3TJ91	LLGL scrib	139.095	32	22	455	22	1027	114.3
P99024	Tubulin be	139.058	58	19	1149	4	444	49.6
Q9Z2D6	Methyl-Cp	138.573	44	21	340	21	484	52.3
G5E924	Heterogen	138.56	53	22	647	22	615	66.9
A2A841	Band 4.1 G	137.977	31	24	391	2	869	97.2

P68372	Tubulin be	137.828	58	19	1091	1	445	49.8
E9QLK7	Transform	137.547	11	35	162	35	3847	435.5
Q8BYC6	Serine/thr	136.548	20	20	411	18	898	105.3
A2A842	Band 4.1 (	135.624	35	23	371	0	769	85.6
Q99PV0	Pre-mRNA	135.301	17	36	263	36	2335	273.4
P62908	40S riboso	135.296	74	23	1337	23	243	26.7
Q3TEA8	Heterochr	133.86	43	26	983	23	554	60.8
Q3U9G9	Delta(14)-	133.837	34	22	851	22	626	71.4
P62702	40S riboso	133.446	66	18	2447	18	263	29.6
S4R189	Rho guani	132.68	23	23	205	23	1526	166.1
E9PXF8	Myotubula	132.226	21	31	208	31	1872	210.3
D3Z6Z7	RAS prote	130.413	30	26	264	26	1019	112.1
H3BIV5	A-kinase a	129.742	41	22	440	22	752	80.2
Q99104	Unconvent	129.735	17	28	247	22	1853	215.4
Q9CWW7	CXXC-type	129.156	35	21	386	21	660	76.1
P63017	Heat shocl	127.954	36	22	543	14	646	70.8
A2RT67	DENN dom	126.835	30	30	354	30	1274	143.8
E0CYH4	WD repeat	126.242	39	17	140	1	625	68.8
Q61087	Laminin su	126.231	29	26	285	26	1168	128.8
P47754	F-actin-ca	126.217	72	14	614	11	286	32.9
A0A3B2W	Tight junct	126.14	25	25	497	23	1165	131
A0A571BG	LIM and ca	126.008	21	25	217	25	1511	168.4
Q8BH64	EH domair	125.543	43	20	479	18	543	61.1
P19973	Lymphocyt	125.095	53	13	274	2	330	36.7
P26231	Catenin al	125.004	34	24	268	19	906	100
Q8K4L2	Archvillin (	124.884	16	28	136	28	2031	226.7
P23298	Protein kir	124.535	34	21	349	18	683	77.9
Q9ERG0	LIM domai	124.037	41	24	165	24	753	84
A2AN08	E3 ubiquit	123.356	7	30	169	30	5180	571.9
P97351	40S riboso	123.23	59	21	1032	21	264	29.9
A0A494BB	WD repeat	123.122	36	18	146	2	729	78.5
F8VPR2	Formin-lik	122.302	24	30	451	20	1083	122.8
Q6QI06	Rapamycir	122.269	21	30	178	30	1708	191.4
Q5RKN9	F-actin-ca	122.26	74	15	557	12	286	32.9
A0A2R8VK	Myosin-9 (	121.46	71	21	1005	1	211	25.4
A2AMW0	F-actin-ca	120.878	64	18	590	5	260	29.3
Q1HFZ0	RNA cytos	120.871	37	23	351	23	757	85.4
B1AY10	Transcript	120.728	35	28	182	28	1114	123.7
Q91VR5	ATP-deper	120.576	41	24	526	24	740	82.4
E9Q634	Unconvent	120.464	28	27	381	19	1107	126.7
D3Z3A8	Unconvent	120.102	12	26	210	25	2631	301.6
O08553	Dihydropyr	119.906	53	20	427	16	572	62.2
E9Q586	Dynactin s	119.627	26	21	145	21	1239	136.8
Q7TSC1	Protein PR	118.498	18	25	217	25	2158	229.1
Q62095	ATP-deper	118.359	37	24	569	4	658	73.4

Q9Z204	Heterogen	117.811	42	21	802	21	313	34.4
Q5BLK4	Terminal u	117.792	20	24	179	22	1491	169
Q6ZQ29	Serine/thr	117.711	15	25	386	20	1240	139.2
O54774	AP-3 comp	117.561	21	20	241	20	1199	135
Q9D8E6	60S riboso	117.009	44	22	534	22	419	47.1
Q921F4	Heterogen	116.637	40	18	266	18	591	64.1
Q61823	Programm	116.379	43	16	316	16	469	51.7
Q8BHZ4	Zinc finger	116.295	25	22	312	22	1262	137.4
E9Q2H1	E3 ubiquit	115.969	13	24	140	24	2792	308.1
Q91WN1	DnaJ homo	115.831	53	18	466	18	259	30
E9Q1F5	Myosin VC	115.615	18	31	274	25	1742	202.6
E9QPM7	Guanine n	115.566	28	24	258	24	1149	129.2
P63276	40S riboso	115.48	60	12	579	12	135	15.5
Q62318	Transcript	114.902	29	18	275	18	834	88.8
Q4FE56	Ubiquitiny	114.453	13	27	194	27	2554	290
O55106	Striatin OS	114.409	39	20	133	19	780	85.9
P68369	Tubulin alp	113.501	49	18	528	6	451	50.1
Q9QZK2	Breast car	113.317	35	24	171	24	820	92.2
P20029	Endoplasm	112.731	40	21	437	19	655	72.4
Q80UM7	Mannosyl-	112.487	29	19	288	19	834	91.8
Q6NZQ2	Probable A	112.373	37	19	274	19	687	76.9
O89050	Muskelin (	112.362	31	17	116	17	735	84.8
Q61584	Fragile X r	112.127	36	21	228	18	677	76.2
Q3U1Y4	DENN dom	111.983	20	23	196	23	1499	164.6
Q05512	Serine/thr	111.862	36	23	203	19	776	86.3
Q6A068	Cell divisio	110.626	30	22	347	22	802	92.1
P11499	Heat shock	110.353	31	22	345	15	724	83.2
Q9D6F9	Tubulin be	110.282	51	16	1014	1	444	49.6
A0A1W2P	Myosin lig	110.103	86	11	879	1	139	15.4
Q8BG05	Heterogen	110.013	40	15	235	15	379	39.6
P16054	Protein kir	110.012	28	19	243	16	737	83.5
Q60605	Myosin lig	109.82	85	12	1011	2	151	16.9
Q8CIE6	Coatomer	109.674	23	25	260	25	1224	138.3
A2A6J7	Lymphocyt	108.959	53	12	220	1	324	35.9
Q6ZWQ9	Myosin, lig	108.671	63	10	994	6	172	19.9
Q62151	Advanced	108.321	48	12	361	12	402	42.6
Q925I1	ATPase fa	108.244	43	24	361	24	591	66.7
Q3TDT0	Tripartite	108.139	33	19	292	18	717	78.2
A0A0A0M	Tubulin alp	108.131	46	18	449	6	477	52.9
Q91ZW3	SWI/SNF-	107.437	19	22	250	22	1051	121.6
Q8C3J5	Dedicator	107.399	16	26	144	25	1828	211.6
A0A1Y7VL	Phosphatic	107.337	29	20	161	19	891	98.5
Q60790	Ras GTPas	107.099	33	22	285	22	834	95.9
Q7TT50	Serine/thr	106.92	17	26	228	22	1713	194.6
A6X8Z5	Rho GTPas	106.395	22	21	165	20	1425	155.2

Q8BZ03	Serine/thr	105.09	26	16	221	13	875	96.5
Q9WVK4	EH domair	104.743	48	18	196	11	534	60.6
Q9QZS3	Protein nu	104.289	25	17	481	13	653	70.8
Q9WUM4	Coronin-1C	104.126	35	22	338	22	474	53.1
Q5F2E8	Serine/thr	103.925	20	22	196	19	1001	116
Q99JY9	Actin-relat	103.876	51	17	372	17	418	47.3
P35700	Peroxiredc	103.581	72	15	404	13	199	22.2
Q9CZ19	Myosin lig	103.352	88	15	687	13	193	21.2
Q9JHJ0	Tropomod	102.902	46	17	695	16	352	39.5
P49222	Protein 4.2	102.636	34	20	268	19	691	76.7
P47757	F-actin-ca	102.379	58	15	531	2	277	31.3
P15864	Histone H1	102.36	46	17	1061	4	212	21.3
P62242	40S riboso	102.01	59	13	1089	13	208	24.2
P07356	Annexin A.	101.957	50	16	368	16	339	38.7
Q9CQ19	Myosin reg	101.871	63	11	595	7	172	19.8
E9QNT8	Ankyrin-1 (	100.598	13	18	95	18	1862	204.1
Q6ZPL9	ATP-deper	100.463	35	20	238	20	600	68.4
A0A0R4J0	Dolichyl-di	99.866	20	17	484	16	823	93.3
Q8C405	Nuclear au	99.079	25	15	210	1	591	68.1
Q9CPR4	60S riboso	98.835	53	12	1693	12	184	21.4
P32067	Lupus La p	98.739	43	21	309	21	415	47.7
Q7TT37	Elongator	98.291	22	21	221	21	1333	149.5
P26040	Ezrin OS=M	98.198	37	28	519	16	586	69.4
Q6A065	Centrosom	96.891	19	23	185	22	1588	174.9
A0A0R4J1	Cytoplasm	96.596	17	19	342	19	1251	144.8
Q9JI91	Alpha-acti	96.115	26	18	223	8	894	103.8
P62082	40S riboso	95.985	58	12	604	12	194	22.1
B1ASP2	Tyrosine-p	95.548	20	17	209	16	1153	133.3
O55201	Transcripti	95.498	23	21	180	21	1082	120.6
P21981	Protein-glu	95.466	37	19	220	19	686	77
G3X9G2	Misshaper	95.445	18	21	204	14	1344	151.1
E9QA05	Nuclear au	95.204	28	14	196	1	494	56.1
Q99LF4	RNA-splici	95.109	44	18	337	18	505	55.2
Q99JX7	Nuclear RI	94.893	39	19	254	19	618	70.3
P97479	Unconvent	94.312	12	21	167	21	2215	254.8
Q8C8U0	Liprin-beta	94.305	23	19	181	16	969	108.5
Q02257	Junction p	94.251	28	19	371	16	745	81.7
Q9ER67	MAGE fam	94.137	32	16	244	16	616	65.4
Q8BJS4	SUN doma	94.131	32	16	79	15	731	81.6
P25444	40S riboso	93.798	52	15	1378	15	293	31.2
Q91VN6	Probable A	93.722	38	19	133	19	622	69.8
P11247	Myeloperc	93.683	31	22	512	18	718	81.1
E9Q0Y4	Signal-indi	93.523	27	20	114	20	1038	112.2
E9PXY8	Ubiquitin c	93.493	20	20	188	20	1143	132.8
E9PYD1	Family wit	93.26	42	15	324	14	344	37.2

Q80YR5	Scaffold a	92.759	24	18	157	9	991	111.8
A2A5Z6	E3 ubiquit	92.752	37	25	242	16	748	86.1
A0A2I3BPY	Ankycorbir	92.472	27	19	116	19	950	105.7
A2A9M4	Dedicator	92.35	13	23	111	21	2130	241.3
P0DP27	Calmoduli	92.339	60	13	1130	13	149	16.8
Q9D0G0	28S riboso	92.256	36	14	287	14	442	49.9
A2A484	Zinc finger	91.98	24	21	166	21	1235	136.9
G3X920	Armadillo	91.933	31	16	105	16	673	75.4
Q8BK67	Protein RC	91.867	47	18	275	18	520	55.9
Q5FWH6	Rho guani	91.593	30	16	213	16	849	92.9
P19096	Fatty acid	91.583	12	24	157	24	2504	272.3
Q8VCC9	Spondin-1	91.453	24	16	335	16	807	90.8
P49718	DNA replic	91.426	34	21	233	21	734	82.4
Q91V88	Nephroned	91.347	34	16	188	16	561	61.5
P43274	Histone H1	91.344	43	16	1160	4	219	22
P09542	Myosin lig	91.098	74	16	456	12	204	22.4
F6WL90	Nuclear au	90.931	41	15	213	5	302	35.8
P09405	Nucleolin (	90.687	30	18	317	18	707	76.7
O88569	Heterogen	90.646	55	16	222	14	353	37.4
Q9Z321	DNA topoi	90.578	27	17	150	17	862	96.9
E9PWB1	Pleckstrin	90.384	20	21	88	21	1324	145
E9Q557	Desmopla	90.357	10	24	149	24	2883	332.7
Q8R5G7	Arf-GAP w	90.108	16	18	184	18	1538	169.6
Q9EPU0	Regulator	89.974	25	19	161	19	1124	123.9
Q8BMC4	Nucleolar	89.944	28	15	123	15	636	70
Q5SVG5	AP comple	89.895	21	19	283	5	916	101.1
P43277	Histone H1	89.863	44	16	1041	1	221	22.1
Q9CW46	Ribonucle	89.571	36	16	84	16	748	79.3
A0A0G2JE	Leucine-ri	89.567	36	18	238	2	424	48.4
P47962	60S riboso	89.504	44	16	575	16	297	34.4
D3YUW7	Cingulin O	89.438	19	19	184	18	1183	135.5
O55143	Sarcoplasr	89.394	21	20	200	11	1044	114.8
Q80UW5	Serine/thr	88.888	18	19	110	18	1551	172
Q3TKT4	Transcript	88.728	17	21	129	12	1613	181.3
P62918	60S riboso	88.355	48	13	646	13	257	28
O35071	Kinesin-lik	88.327	22	21	148	16	1100	122.4
Q9CQE8	RNA trans	88.01	47	12	185	12	244	28.1
Q8VCQ8	Caldesmor	87.836	36	20	199	20	530	60.4
Q8BX02	KN motif a	87.762	23	16	134	16	843	90.2
Q8R151	NFX1-type	87.446	16	20	121	20	1909	218.7
A0A0R4J0	Ran-bindir	87.262	34	14	106	13	648	70
Q9WV32	Actin-relat	87.081	40	13	303	12	372	41
P62751	60S riboso	87.08	52	12	881	12	156	17.7
A0A1D5RL	Band 4.1 (	86.972	35	15	236	2	476	54
Q69Z99	Zinc finger	86.764	33	15	160	15	562	63.9

A0A140LJJ	A-kinase a	86.662	9	20	96	11	2794	305.8
O54724	Caveolae-a	86.408	29	12	420	12	392	43.9
E9PW37	RAS prote	86.394	21	21	218	18	1286	144.5
D3YXK2	Scaffold a	86.301	20	15	86	6	937	105
A0A0A6YV	Mitogen-a	86.065	20	20	239	12	1288	146.6
Q61768	Kinesin-1 l	85.936	26	22	216	22	963	109.5
F8VQC1	Signal rec	85.929	30	19	216	19	671	74.6
O55098	Serine/thr	85.786	18	18	241	17	966	111.8
A0A286YD	Microtubu	85.767	12	14	174	1	1358	147.1
Q9CVB6	Actin-relat	85.72	51	15	443	15	300	34.3
Q4VC33	E3 ubiquit	85.557	41	17	126	17	396	45.3
E9Q3L2	Phosphatic	85.463	12	20	109	20	2105	236.9
Q9D6Z1	Nucleolar	85.441	36	16	230	16	580	64.4
Q8JZQ9	Eukaryotic	84.811	26	15	146	15	803	91.3
Q3U7K7	E3 ubiquit	84.577	38	16	304	16	462	53.3
P17427	AP-2 comp	84.262	22	20	246	13	938	104
A8DUK4	Beta-globi	83.841	73	10	240	9	147	15.7
Q8C147	Dedicator	83.759	11	20	141	18	2100	238.8
G3UWZ0	Bromodon	83.685	15	19	130	19	1552	178.1
Q8K224	RNA cytidi	83.53	23	22	143	22	1024	115.3
P31001	Desmin OS	83.285	35	19	264	16	469	53.5
E9QN52	Leucine-ric	83.211	37	17	205	1	415	47.1
P17426	AP-2 comp	82.975	20	20	313	13	977	107.6
P69566	Ran-bindir	82.94	30	15	126	14	653	71
Q8BH44	Coronin-2E	82.562	35	16	151	16	480	54.9
Q8K1A6	Coiled-coil	82.557	22	18	159	18	943	103.6
Q7TPQ3	E3 ubiquit	82.406	13	17	110	17	1674	191.4
Q61029	Lamina-as	82.405	44	14	91	14	452	50.3
Q80Y17	Lethal(2) g	82.311	23	16	163	16	1036	112.5
B1B0C7	Basement	82.061	5	16	110	16	4375	468.7
A0A0A0M	Glyceralde	81.868	47	13	339	13	359	38.6
Q9Z0N1	Eukaryotic	81.817	40	14	246	3	472	51
Q9Z2C4	Myotubula	81.815	31	20	239	19	669	75.3
P35550	rRNA 2'-O	81.729	44	12	334	12	327	34.3
P61164	Alpha-cen	81.58	36	11	208	5	376	42.6
Q8BKJ9	NAD-depe	81.509	39	13	213	13	402	45.1
P84091	AP-2 comp	81.467	43	18	444	18	435	49.6
Q91VC3	Eukaryotic	81.446	37	16	303	14	411	46.8
P28740	Kinesin-lik	81.121	28	18	208	18	705	79.7
Q6P4T2	U5 small r	80.966	10	20	79	20	2136	244.4
E9QK41	Actin-bind	80.953	22	16	94	16	861	96.8
Q8BKF1	DNA-direc	80.508	21	17	159	17	1207	136.6
Q63918	Caveolae-a	80.496	26	13	479	13	418	46.7
Q99ME2	WD repeat	80.184	17	12	97	12	1125	121.8
A0A140T8	60S riboso	79.81	51	9	816	9	192	21.8

P97434	Myosin ph	79.108	23	19	146	19	1024	116.3
A0A1L1SQ	Talin-2 OS	79.061	7	18	188	5	2544	271.8
P68254	14-3-3 pro	78.814	46	12	256	9	245	27.8
Q9EQP2	EH domair	78.776	34	16	317	13	541	61.4
Q8BMA6	Signal reco	78.739	32	16	153	16	625	70.5
P61161	Actin-relat	78.554	39	13	307	13	394	44.7
Q6DIC0	Probable g	78.244	13	18	132	9	1577	180.1
Q4ACU6	SH3 and m	77.77	12	14	120	14	1730	185.3
Q3UPF5	Zinc finger	77.72	17	13	220	13	946	106.6
Q9Z329	Inositol 1,4	77.425	10	21	68	15	2701	307.3
P25206	DNA replic	77.231	27	18	77	18	812	91.5
Q5SXC4	Vascular e	77.163	28	12	246	11	518	56.5
Q9DBE9	pre-rRNA 2	77.028	21	15	333	15	838	95.5
P10107	Annexin A	76.759	50	13	130	13	346	38.7
Q03141	MAP/micr	76.71	27	18	150	11	753	84.3
Q8VH51	RNA-bindi	76.396	34	14	320	14	530	59.4
Q6ZWN5	40S riboso	76.216	59	18	1467	18	194	22.6
A0A0R4J0	CMP-N-ace	76.205	41	14	348	14	432	48
Q6ZPJ3	(E3-indepe	76.201	15	12	109	12	1288	140.7
P14206	40S riboso	76.054	54	11	153	11	295	32.8
P11032	Granzyme	75.99	50	13	299	13	260	28.6
Q03265	ATP syntha	75.869	30	17	202	17	553	59.7
O08810	116 kDa U	75.566	25	19	130	18	971	109.3
P62137	Serine/thr	75.493	55	15	161	5	330	37.5
P29341	Polyadeny	75.165	23	15	164	11	636	70.6
E9PYK3	Protein mo	75.126	12	18	70	18	1969	216
Q9Z1P7	KN motif a	75.021	24	16	183	16	791	84.1
Q3UMT1	Protein ph	74.987	26	16	87	16	782	84.6
Q80SU7	Interferon-	74.797	9	19	126	19	2427	280.6
Q9DBV3	Probable A	74.671	18	14	96	14	1145	128.4
Q8R0W0	Epiplakin C	74.616	16	18	128	15	6548	724.2
O08529	Calpain-2 c	74.451	30	16	151	16	700	79.8
P51432	1-phospha	74.321	17	19	162	19	1234	139.4
Q8C845	EF-hand d	73.983	45	12	307	12	240	26.8
P48678	Prelamin-A	73.959	29	18	199	17	665	74.2
Q61233	Plastin-2 C	73.918	31	16	239	12	627	70.1
Q5NCC3	E3 ubiquit	73.859	27	12	144	12	630	71.8
Q8BVK9	Sp110 nuc	73.838	39	18	263	18	445	50.1
F8VQJ3	Laminin su	73.736	16	17	93	17	1607	177.1
Q04857	Collagen a	73.615	21	16	129	16	1025	108.4
P49813	Tropomod	73.587	51	12	190	11	359	40.4
Q8BQ30	Phostensir	73.45	30	13	86	13	594	65.6
P58871	182 kDa ta	73.099	13	16	106	14	1720	181.7
Q61235	Beta-2-syr	72.969	30	12	136	11	520	56.3
Q9R0Q6	Actin-relat	72.776	52	14	174	13	370	41.6

D3Z0K6	Round spe	72.753	25	14	107	14	821	91.7
K3W4R7	Troponin T	72.377	28	9	271	9	301	35.8
P54818	Galactoce	72.093	20	9	139	9	684	77.2
Q9ERU3	Zinc finger	71.988	41	12	314	10	237	27.3
P60122	RuvB-like	71.947	36	13	156	13	456	50.2
Q9Z0N2	Eukaryotic	71.935	38	13	227	2	472	51.1
P14131	40S riboso	71.733	75	15	742	15	146	16.4
G5E874	Laminin su	71.647	15	14	145	14	1193	130.3
P10126	Elongation	71.579	27	12	287	12	462	50.1
P70257	Nuclear fa	71.572	34	10	231	3	488	53.4
Q9JKB3	Y-box-binc	71.46	47	11	248	6	361	38.8
Q8K4F6	28S rRNA	71.346	39	15	133	15	465	51
Q8BFR5	Elongation	71.172	37	15	126	15	452	49.5
Q8CBE3	WD repeat	70.571	41	13	156	13	496	55
P17563	Methanetl	70.496	38	14	129	14	472	52.5
Q9EQ61	Pescadillo	70.468	27	15	261	15	584	67.8
Q99L88	Beta-1-syr	70.446	30	13	119	12	537	58
Q9D0D4	Probable c	70.37	49	14	329	14	313	35.3
Q99L45	Eukaryotic	70.172	44	14	237	14	331	38.1
A2ACV6	Protein SC	70.161	14	21	95	19	1654	183
Q8BJH1	Zinc finger	70.099	54	15	219	15	324	35.1
B1AS46	Zinc finger	69.844	10	11	107	11	1762	191.2
Q99MJ9	ATP-deper	69.818	23	13	159	13	734	82.1
P28660	Nck-associ	69.483	16	16	137	15	1128	128.7
Q640N3	Rho GTPas	69.421	20	18	64	17	1101	120
O55222	Integrin-lir	69.408	29	12	221	12	452	51.3
Q8CGB3	Uveal auto	68.999	14	16	90	16	1411	160.7
Q921R2	40S riboso	68.859	54	11	770	11	140	16.1
Q6WKZ7	Nostrin OS	68.784	38	17	202	17	506	57.6
Q9CZM2	60S riboso	68.703	50	11	650	11	204	24.1
Q4U2R1	E3 ubiquit	68.359	5	19	82	19	4836	527.1
Q8K363	ATP-deper	68.331	26	13	108	13	660	74.1
Q3TYK3	Nuclear fa	68.291	44	9	211	2	391	43.6
O08784	Treacle pr	68.238	11	14	118	14	1320	134.9
E9PVA6	ARF GTPas	68.155	26	18	140	4	759	84.4
B1AR17	DNA helica	68.151	10	17	134	14	2055	232.6
P47911	60S riboso	68.095	38	15	442	15	296	33.5
P35831	Tyrosine-p	68.067	30	17	45	17	775	86.5
A2AVJ7	Ribosome-	68.042	26	15	135	15	1464	158.3
E9PUA3	IQ motif a	68.038	17	16	101	13	1099	122.6
A0A0N4SU	Pumilio hc	67.965	26	12	112	12	648	72.9
Q8CCF0	U4/U6 sm	67.938	34	15	237	15	499	55.4
A0A0R4J0	Rho guani	67.932	26	14	85	11	646	73
Q8R1B4	Eukaryotic	67.725	15	14	136	14	911	105.5
Q0VEE6	Zinc finger	67.503	27	16	176	16	662	74.8

Q61191	Host cell fa	67.233	11	16	98	16	2045	210.3
Q76MZ3	Serine/thr	67.204	27	13	186	13	589	65.3
Q5SWD9	Pre-rRNA-	67.178	17	13	231	13	803	92
P47738	Aldehyde d	67.021	31	13	181	12	519	56.5
O70503	Very-long-	67.01	41	12	119	12	312	34.7
Q91YQ5	Dolichyl-di	66.917	29	13	81	13	608	68.5
P42932	T-complex	66.915	36	17	131	17	548	59.5
P62754	40S riboso	66.892	40	13	529	13	249	28.7
O55029	Coatomer	66.881	20	14	108	14	905	102.4
P11531	Dystrophir	66.874	4	12	118	10	3678	425.6
Q8BMS1	Trifunctior	66.548	26	14	170	14	763	82.6
Q9WV92	Band 4.1-l	66.526	17	12	267	8	929	103.3
Q8K3A9	7SK snRNA	66.522	38	15	123	15	666	72
F8VQB6	Unconvent	66.481	11	19	106	19	2062	237.2
O09118	Netrin-1 C	66.43	27	13	150	13	604	67.8
Q80YQ8	E3 ubiquit	66.3	39	14	64	12	391	44
E9Q1G8	Septin OS=	66.29	34	12	230	12	437	50.6
Q80X80	Phospholip	66.041	21	9	80	9	706	76.3
Q9ERU9	E3 SUMO-	66.022	6	14	161	14	3053	340.9
Q8BLR5	PH and SE	66.002	17	17	108	17	1005	112.7
F8VPP8	Zinc finger	65.979	16	14	111	12	982	110.2
P49290	Eosinophil	65.933	23	17	159	13	716	81.3
Q9Z0H1	WD repeat	65.701	26	14	180	14	622	69
P07901	Heat shock	65.408	18	13	178	7	733	84.7
Q9ERL9	Guanylate	65.26	29	16	108	16	691	77.5
Q8CIB5	Fermitin fa	65.252	23	14	163	14	680	77.8
D4AFX7	DnaJ heat	65.106	11	20	98	20	2248	254.9
A0A2U3TZ	TRIO and F	65.082	26	15	105	15	627	71.6
Q99LL5	Periodic tr	65.049	34	12	148	12	501	55.6
Q08048	Hepatocyte	64.995	23	13	77	13	728	82.9
M0QWP1	Agrin OS=	64.837	9	11	83	11	2034	216.5
Q9JMC8	Band 4.1-l	64.837	27	13	183	11	527	59.5
P62270	40S riboso	64.693	56	16	1223	16	152	17.7
P62264	40S riboso	64.45	72	9	283	9	151	16.3
Q5XG71	Small subu	64.358	8	19	110	19	2788	317.5
Q69ZA1	Cyclin-dep	64.018	14	16	85	5	1511	164.5
P63087	Serine/thr	63.976	45	12	135	1	323	37
O88738	Baculovira	63.962	5	15	61	15	4882	531.8
P61979	Heterogen	63.931	32	11	279	11	463	50.9
E9QAS5	DNA helica	63.928	11	14	98	11	1922	218.5
Q64511	DNA topoi	63.921	12	19	124	19	1612	181.8
P11983	T-complex	63.862	25	14	175	14	556	60.4
A2AH75	Kinesin-lik	63.847	18	16	70	12	1150	130
Q91V55	40S riboso	63.845	43	10	393	10	204	22.9
Q9ESN6	Tripartite	63.815	24	11	63	10	744	81.4

Q8K4L0	ATP-deper	63.79	18	13	162	13	874	97.7
A0A0U1RI	Myc-assoc	63.786	28	13	146	12	493	51.2
Q4QRL3	Coiled-coil	63.64	15	19	63	19	1481	166.5
Q8BTI8	Serine/arg	63.587	8	16	41	16	2703	294.7
O88342	WD repeat	63.567	30	12	64	12	606	66.4
A0A0J9YTI	Cytospin-B	63.55	19	16	67	16	1058	117
Q5SQ27	SEC14-like	63.536	45	12	156	12	401	46.1
P63037	DnaJ hom	63.451	29	10	113	10	397	44.8
Q9CXW4	60S riboso	63.327	43	8	1218	8	178	20.2
Q9WTJ4	Flt3-intera	63.313	24	7	92	7	500	52.7
Q7TNV0	Protein DE	63.298	33	15	191	15	380	43.1
Q8C0C7	Phenylalar	63.19	29	13	75	13	508	57.6
Q9Z1T1	AP-3 comp	63.136	16	16	119	16	1105	122.7
Q61586	Glycerol-3	63.119	17	11	146	11	827	93.6
O08582	GTP-bindi	63.103	36	13	127	13	668	72.3
P54823	Probable A	62.853	33	11	143	11	483	54.2
P84099	60S riboso	62.848	37	11	434	11	196	23.5
Q8BFZ3	Beta-actin	62.718	34	12	561	3	376	42
P62281	40S riboso	62.679	53	11	872	11	158	18.4
Z4YJT3	La-related	62.568	18	15	116	15	1072	121.1
Q9D902	General tr	62.457	43	10	79	10	292	33
O54865	Guanylate	62.186	31	14	79	14	620	70.6
F6TFN2	LIM domai	62.15	12	13	106	13	1189	133.9
Q921K2	Poly [ADP-	61.814	20	16	61	16	1014	112.7
Q6A085	Zinc finger	61.694	22	14	97	10	867	96
Q6ZQ38	Cullin-asso	61.627	16	17	105	17	1230	136.2
P20918	Plasminog	61.543	25	13	96	13	812	90.7
Q8K4Q6	Endonucle	61.352	40	11	146	11	389	43.6
Q8BJL0	SWI/SNF-	61.296	19	13	118	13	910	100.8
Q8BIF9	Zinc finger	61.224	30	9	141	9	381	40.5
A0A1C7CY	Plastin-3 (	61.164	30	15	112	10	629	70.6
Q69ZH9	Rho GTPas	61.116	15	15	51	15	1483	161.7
P70168	Importin s	61.055	17	12	136	12	876	97.1
Q91YU8	Suppresso	60.97	32	13	207	13	470	52.7
Q6DID5	PWWP do	60.937	30	14	106	14	682	76
O54824	Pro-interle	60.911	12	13	104	13	1322	141.3
Q91W89	Alpha-mar	60.901	15	13	58	13	1039	115.6
A0A171EB	E3 ubiquit	60.698	4	15	84	15	5151	584.3
E9PYZ4	Tripartite	60.313	36	16	157	16	485	55.9
Q8BZI0	Actin filam	60.167	17	11	117	11	768	86.6
P51859	Hepatoma	60	64	11	130	11	237	26.3
P11679	Keratin, ty	59.757	26	15	210	10	490	54.5
A2AT37	Regulator	59.661	15	14	83	14	1269	147.5
E9QMY5	Protein Sh	59.639	11	15	48	15	1980	214.2
Q08EC4	Cas scaffo	59.484	22	13	60	13	804	88.6

Q80XP8	Protein FA	59.36	38	12	144	10	339	38.5
Q9R112	Sulfide:qu	59.309	30	12	131	12	450	50.3
A0A0A0M	Microtubu	59.209	31	9	103	1	369	42.7
P14148	60S riboso	59.092	39	12	366	12	270	31.4
P70218	Mitogen-a	59.02	21	13	106	13	827	91.5
Q9CZX8	40S riboso	58.893	65	13	592	13	145	16.1
G3UW40	Mutated ir	58.792	22	12	78	12	828	92.8
Q9JKK7	Tropomod	58.739	34	11	272	10	351	39.5
Q8R310	Transmem	58.696	35	14	71	14	477	53.7
P62900	60S riboso	58.612	47	11	339	11	125	14.5
A2BE28	Ribosoma	58.524	21	14	196	14	776	89.4
Q9R117	Non-recep	58.324	16	14	82	14	1184	133.2
A0A0R4J0	Interferon	58.285	36	13	190	13	368	41
P43275	Histone H1	58.124	30	9	399	5	213	21.8
A0A1L1SS	Serine/thr	57.977	25	9	161	5	407	45.8
Q922F4	Tubulin be	57.94	25	10	272	2	447	50.1
Q63844	Mitogen-a	57.811	40	13	95	7	380	43
Q80T69	Lysine-spe	57.721	20	12	143	12	795	89.2
Q9D2E2	Target of I	57.712	31	11	170	11	511	56.8
Q6PCM2	Integrator	57.593	21	15	75	13	883	99.6
Q9DCA5	Ribosome	57.253	50	11	174	11	353	41.2
Q9JLQ2	ARF GTPa	57.12	25	15	136	1	708	78.7
P62141	Serine/thr	57.073	42	11	118	1	327	37.2
Q497V5	S1 RNA-bi	56.976	16	13	100	13	982	110.2
I7HLV2	60S riboso	56.882	46	10	255	10	201	23.1
O88967	ATP-deper	56.57	15	10	84	10	715	80
Q8BP48	Methionin	56.558	34	9	101	9	386	43.2
Q5S006	Leucine-ri	56.023	7	14	64	14	2527	284.5
P97315	Cysteine a	55.923	56	9	281	9	193	20.6
Q3TCR7	Dynamin (	55.913	20	14	85	11	869	97.9
F8WIZ7	Oxysterol-	55.897	15	11	94	11	967	109.7
A2API8	Paralemm	55.869	12	11	123	11	921	101.5
S4R1W5	RNA-bindi	55.756	16	16	49	16	1118	128.3
Q8BX70	Vacuolar p	55.684	5	12	49	12	3748	419.8
Q8BXK8	Arf-GAP w	55.623	17	12	81	3	857	94.4
Q8BY46	Zinc finger	55.608	21	15	84	15	900	99.4
Q8VHM5	Heterogen	55.589	21	10	88	7	632	70.8
A0A0N4SV	Multimeri	55.473	11	12	96	12	1209	135.8
P49312	Heterogen	55.374	26	8	74	6	320	34.2
P47963	60S riboso	55.316	40	12	615	12	211	24.3
A2AQ25	Sickle tail	55.219	8	11	60	11	1946	212.9
Q64152	Transcript	55.124	45	6	96	5	204	22
O88939	Zinc finger	55.008	28	9	205	9	569	60.2
A0A286YC	Trafficking	54.785	12	13	57	13	1437	160.8
Q3UIJ9	Myocardia	54.754	24	10	111	10	466	53.9

P97868	E3 ubiquit	54.502	7	13	62	13	1790	199.5
P80314	T-complex	54.45	21	9	99	9	535	57.4
P61255	60S riboso	53.938	48	12	719	12	145	17.2
Q3UHD6	Sorting ne	53.912	25	10	144	10	539	61
Q62074	Protein kir	53.911	24	10	78	9	595	68.2
Q3UPL0	Protein tra	53.843	14	14	90	14	1230	133.5
A0A0R4J1	Drebrin OS	53.8	23	12	95	12	661	72.5
O35744	Chitinase-l	53.769	28	11	89	11	398	44.4
P62830	60S riboso	53.683	64	9	273	9	140	14.9
P43276	Histone H1	53.568	35	10	708	8	223	22.6
E9Q8N5	CLIP-assoc	53.55	13	14	49	12	1286	140.6
Q68FE6	Rho family	53.453	11	11	106	11	1223	132.3
G3X960	Rho GTPas	53.432	22	12	59	12	737	80.4
Q9D4G5	Processing	53.411	20	13	65	13	1015	114
Q64318	Zinc finger	53.367	11	9	83	7	1117	122.4
P35980	60S riboso	53.305	42	9	317	9	188	21.6
P97760	DNA-direc	53.295	41	7	107	7	275	31.4
Q9DCE5	p21-activa	53.174	31	9	165	9	382	42.1
A0A2I3BQ	Leucine-ric	52.915	21	10	77	9	682	76
Q99MR6	Serrate RN	52.883	16	14	47	14	875	100.4
Q8CD15	Ribosomal	52.865	30	10	104	10	465	53.5
Q9JKC8	AP-3 comp	52.823	39	10	110	10	418	46.9
Q9DC47	Zinc finger	52.811	23	11	123	8	529	61.6
D3YYN8	Non-speci	52.742	9	13	65	10	1691	192.6
Q505N6	Ribosomal	52.658	20	13	90	7	735	82.8
Q6AXB7	Synaptic fu	52.651	25	12	104	10	614	68.8
P62267	40S riboso	52.63	50	8	308	8	143	15.8
A0A0N4SV	Zinc finger	52.61	10	13	44	13	1926	214.3
P23116	Eukaryotic	52.585	12	13	95	13	1344	161.8
Q60737	Casein kin	52.577	35	8	57	8	391	45.1
Q64324	Syntaxin-b	52.557	24	11	83	11	593	66.3
A2A8U2	Transmem	52.49	13	7	113	7	664	72.5
Q9Z277	Tyrosine-p	52.41	11	13	95	13	1479	170.5
Q8R5C5	Beta-centr	52.347	34	9	129	3	376	42.3
E9Q559	Calcium-tr	52.286	15	13	102	8	1026	112.4
Q922Q8	Leucine-ric	52.16	44	10	88	10	307	34.9
P13864	DNA (cyto:	52.105	12	14	52	14	1620	183.1
P63101	14-3-3 pro	52.036	37	10	191	7	245	27.8
A0A1D5RN	Non-speci	52.014	12	11	108	9	1305	142.5
A2AUY4	Bromodon	51.889	7	10	86	10	2123	233.9
Q02248	Catenin be	51.885	16	10	174	7	781	85.4
Q3TMP1	General tr	51.865	16	11	99	11	882	100.6
P55284	Cadherin-5	51.849	18	11	58	11	784	87.8
Q9CPY6	Glucose-ir	51.808	38	8	22	8	217	24.8
Q9R045	Angiopoie	51.785	27	12	91	12	493	57.1

Q99KV1	DnaJ homo	51.673	31	10	150	10	358	40.5
Q9JI11	Serine/thr	51.537	18	10	90	8	487	55.5
D3Z7R7	Rho GTPas	51.503	14	13	69	13	1127	124
A0A1Y7VL	Cyclin-dep	51.488	19	12	73	1	837	93
Q8C7V3	U3 small r	51.24	24	11	198	11	528	59.3
A0A0R4J2	Retinoblas	51.22	26	11	84	11	537	59
Q8BH59	Calcium-bi	51.206	18	11	130	10	677	74.5
Q7TND5	Ribosome	51.189	32	9	121	9	349	40
Q8BI72	CDKN2A-ir	51.102	30	11	21	11	563	59.7
O08967	Cytohesin-	50.98	24	9	105	6	399	46.3
Q8K1N2	Pleckstrin	50.927	11	12	68	12	1249	141.4
Q3TZX8	Polynuclec	50.908	15	9	107	9	714	80.8
E9Q2E5	Rap guani	50.895	15	10	85	10	909	102.3
P52480	Pyruvate k	50.883	28	10	121	9	531	57.8
D3Z5M7	Peroxidasi	50.875	36	8	122	1	307	34.8
Q6P5E4	UDP-gluco	50.856	11	12	74	11	1551	176.3
G3UZI2	Heterogen	50.563	24	10	97	7	527	58.7
Q6PDM1	Male-spec	50.453	25	10	50	10	616	67.3
E9QNN1	DEAH box	50.444	12	12	85	12	1384	149.6
P60766	Cell divisic	50.434	48	9	328	8	191	21.2
Q9D7M1	Glucose-ir	50.392	34	7	57	7	228	26.8
A0A0R4J0	Paladin OS	50.293	19	14	117	14	859	96.7
Q9JIF7	Coatomer	50.274	14	9	66	9	953	107
P49135	General tr	50.177	16	9	90	9	783	89.1
F8VPV0	Pericentrir	50.126	6	14	57	14	2916	331.1
Q91YK2	Ribosoma	50.107	16	10	92	10	724	80.5
E9Q133	T-complex	50.084	24	11	88	11	507	56.5
Q3UFY8	tRNA met	49.988	29	10	81	10	414	48.4
P09021	Homeobox	49.969	39	8	150	7	270	29.2
H7BWY4	Disks large	49.911	17	9	45	8	872	96.8
Q91WQ3	Tyrosine--	49.841	27	13	157	13	528	59.1
Q5SU71	E3 ubiquit	49.809	18	10	62	10	626	70.7
Q9EQH3	Vacuolar p	49.799	16	11	83	11	796	91.7
D3YYT1	Glyoxylate	49.771	21	10	119	10	552	60.4
A2AIV2	Protein vir	49.719	7	11	70	11	1811	201.3
Q8QZY1	Eukaryotic	49.638	26	14	137	14	564	66.6
P97452	Ribosome	49.502	22	11	66	11	732	82.5
Q8BWT5	Disco-inte	49.44	11	14	73	10	1523	165.1
A0A1W2P	Bromodon	49.348	8	12	87	12	1890	209.6
E9QAQ5	Glycogen s	49.313	31	11	81	10	433	48
Q8C9B9	Death-indu	49.286	7	8	19	8	2256	247
Q8K1Y2	Serine/thr	49.182	17	11	102	7	889	100
Q8VC03	Echinoderr	49.135	18	10	25	10	897	95.6
Q8CAQ8	MICOS cor	49.127	17	10	61	4	757	83.8
P08752	Guanine n	49.097	37	10	86	7	355	40.5

O89090	Transcript	49.055	7	6	194	6	784	80.7
P27546	Microtubu	48.94	13	13	80	13	1125	117.4
B1AXN9	Non-speci	48.929	16	10	102	4	712	80.6
Q09XV5	Chromodo	48.912	6	12	54	10	2582	290.7
Q9CQH7	Transcript	48.684	47	8	89	7	158	17.3
Q8R5K4	Nucleolar	48.545	12	10	84	10	1152	129.1
P14115	60S riboso	48.536	36	8	599	8	148	16.6
Q8CD10	Calcium u	48.528	22	9	124	9	432	49.4
E9Q652	Regulator	48.407	11	13	61	13	1451	156.4
Q3UID0	SWI/SNF	48.399	15	12	96	8	1130	124.6
Q5NBY9	POZ (BTB)	48.398	15	10	57	10	687	74.1
Q8K0D0	Cyclin-dep	48.386	28	13	91	12	523	59.5
Q6NVF2	Phospholi	48.343	15	11	55	11	1036	119.5
Q9JL61	DNA-bindi	48.225	24	10	68	10	658	69.7
Q68FF6	ARF GTPa	48.196	20	12	89	9	770	85.2
P70670	Nascent p	48.178	2	4	354	4	2187	220.4
P35979	60S riboso	48.099	55	6	151	6	165	17.8
O35286	Pre-mRNA	48.086	15	12	85	12	795	90.9
Q3U1J4	DNA dama	48.057	12	13	125	13	1140	126.8
Q99J95	Cyclin-dep	48.051	28	11	156	9	372	42.7
Q91VB8	Alpha glob	47.949	74	7	147	6	142	15.1
O35954	Membrane	47.918	15	14	106	14	1243	134.9
Q8C2Q7	Heterogen	47.917	29	9	116	3	472	51.2
Q9QZE5	Coatomer	47.883	16	11	75	11	874	97.5
Q80WQ6	Inactive rh	47.784	16	12	64	12	827	93.4
Q99LH2	Phosphati	47.75	21	7	66	7	473	55.6
Q5RJ54	Zinc finge	47.643	29	12	66	11	466	53.5
Q8VDP4	Cell cycle	47.604	15	8	49	8	922	102.9
Q8BHL3	TBC1 dom	47.588	17	10	166	10	798	87.2
E9QPR4	Non-speci	47.534	8	13	53	9	2441	265.9
Q9CY58	Plasminog	47.451	22	8	81	8	407	44.7
Q61171	Peroxired	47.405	60	8	169	8	198	21.8
Q3UYU2	tRNA (ura	47.388	23	10	104	10	613	67.4
Q9JMK2	Casein kin	47.298	22	7	148	4	416	47.3
Q8C033	Rho guani	47.11	10	10	120	10	1345	147.9
Q91W50	Cold shock	47.057	23	15	79	15	798	88.7
P56480	ATP synth	47.052	17	8	105	8	529	56.3
F8WIY7	StAR-relat	47.044	15	11	51	10	1132	126.8
O35613	Death dom	46.984	16	9	133	9	739	81.4
Q6P3Z4	Zinc finge	46.862	24	11	75	9	590	66.7
E9Q1K3	Alpha-add	46.749	21	9	57	9	660	72.9
Q9D903	Probable r	46.682	28	10	214	10	306	34.7
E9QKY4	FYVE, Rho	46.629	8	10	49	10	1514	166.8
A0A668KL	Chloride ir	46.587	15	7	98	7	485	54.1
Q99KP6	Pre-mRNA	46.514	21	8	57	8	504	55.2

Q62511	E3 ubiquit	46.51	24	12	120	12	572	63.4
F8WIE5	HECT-type	46.494	6	13	80	13	2610	289
Q6PGC1	ATP-deper	46.474	12	13	49	12	1365	153.9
A0A5F8MI	Microtubu	46.435	28	9	117	1	326	38.1
Q5SVQ0	Histone ac	46.346	18	8	93	7	613	70.6
Q9JIY0	Pleckstrin	46.06	24	9	65	9	408	46
Q9WTU0	Lysine-spe	46.057	13	9	44	8	1096	120.7
P97857	A disinteg	45.974	17	11	68	11	968	105.7
P15508	Spectrin b	45.942	6	9	85	6	2128	245.1
Q62009	Periostin C	45.816	17	11	58	11	838	93.1
B2RY56	RNA-bindi	45.798	15	10	61	10	838	99.5
E9PWE8	Dihydropyı	45.69	21	11	108	7	683	73.8
P63085	Mitogen-a	45.682	36	10	82	5	358	41.2
B2RXP1	DISP comp	45.63	15	9	89	8	742	82.2
A2AUK7	Band 4.1-l	45.584	18	10	100	7	730	81.8
P33267	Cytochrom	45.47	24	11	92	10	491	55.9
P12382	ATP-deper	45.344	14	9	67	7	780	85.3
P05977	Myosin lig	45.211	51	10	268	6	188	20.6
O70551	SRSF prote	45.18	17	9	54	7	648	73
Q62219	Transform	45.158	23	8	85	8	461	50.1
Q8BX80	Cytosolic e	45.014	23	12	48	12	734	82.9
D3Z030	F-actin-un	44.988	11	10	39	10	1332	147.1
Q6ZWX6	Eukaryotic	44.905	36	9	114	9	315	36.1
G5E866	Splicing fa	44.899	12	12	59	12	1304	145.7
Q7TPM9	Myotubula	44.822	19	10	56	10	771	88.1
E9QKB1	Rho GTPas	44.691	16	14	77	13	1092	123.3
Q8CHP0	Zinc finger	44.627	13	9	89	9	950	103.2
P42227	Signal trar	44.564	15	9	76	8	770	88
Q62101	Serine/thr	44.52	11	8	83	2	918	102
P04919	Band 3 ani	44.487	13	9	79	9	929	103.1
A2A7S8	Uncharact	44.448	13	6	22	6	1013	104.8
Q3UQI9	Probable u	44.427	20	11	44	11	744	82.9
P42208	Septin-2 C	44.379	35	9	97	9	361	41.5
Q3U5D9	Aryl hydro	44.356	13	11	120	11	805	90.3
B7ZCR6	Zinc finger	44.271	15	8	56	8	869	95.3
A0A0R4J0	E3 ubiquit	44.268	20	11	65	11	734	79.5
G3UZM9	Protein kir	44.209	15	10	54	9	967	109.8
A2AQ07	Tubulin be	44.172	24	9	234	6	451	50.4
Q5SSI6	U3 small r	44.106	20	8	65	8	552	61.2
Q8BKH7	Target of r	44.091	27	8	75	8	522	59
Q60972	Histone-bi	44.053	29	8	107	4	425	47.6
Q9CPW4	Actin-relat	44.049	45	7	139	7	151	16.3
P70333	Heterogen	44.043	30	9	75	4	449	49.2
O70494	Transcript	43.98	12	9	134	9	783	82.3
Q8BKC5	Importin-5	43.884	13	11	38	11	1097	123.5

P25911	Tyrosine-p	43.875	21	10	111	8	512	58.8
A0A338P6	DNA-direc	43.837	52	3	78	3	108	12.2
Q69Z38	Inactive ty	43.806	7	8	41	8	1735	191
P60762	Mortality f	43.804	28	8	87	1	362	41.5
A0A1B0GS	L-lactate c	43.768	32	10	150	9	315	34.6
Q8CI11	Guanine n	43.765	21	8	96	8	538	60.7
Q8K2H4	Arf-GAP w	43.765	17	8	66	8	740	81.7
Q9DB20	ATP syntha	43.742	38	5	59	5	213	23.3
A7YQ68	Prickle-like	43.712	15	10	99	10	901	102.2
Q5SFM8	RNA-bindi	43.71	12	10	79	10	1060	118.5
Q8BL48	RING finge	43.497	17	9	45	8	810	88
P28293	Cathepsin	43.49	33	8	193	8	261	29.1
P07724	Albumin C	43.431	19	10	111	9	608	68.6
Q8VEB1	G protein-	43.415	22	10	55	9	590	67.7
P40936	Indolethyla	43.404	34	7	119	7	264	29.4
Q8K045	Serine/thr	43.344	16	11	88	10	878	97.8
P24549	Retinal de	43.329	25	10	101	7	501	54.4
A0A0R4J0	Olfactome	43.324	27	9	98	9	406	45.8
J3JS23	Zinc finger	43.294	18	12	76	10	893	102.1
O89032	SH3 and P	43.292	8	7	101	7	1124	124.1
Q5FWH2	Putative E	43.228	14	8	73	7	727	79.6
Q8BJ90	Zinc finger	43.208	36	11	147	11	317	35.7
P28867	Protein kir	43.192	22	10	95	9	674	77.5
A0A087W	Mortality f	43.147	35	8	92	1	296	34.3
Q8K4I3	Rho guani	43.127	17	11	64	8	771	87
Q9CZX0	Elongator	43.04	24	12	108	12	547	62.3
Q5DU09	Zinc finger	43.002	17	10	117	7	608	69.4
O35129	Prohibitin-	42.958	37	10	72	10	299	33.3
Q6PE01	U5 small r	42.839	38	8	37	8	358	39.3
Q99KJ8	Dynactin s	42.824	28	9	135	9	402	44.1
Q9QYB5	Gamma-a	42.81	16	10	40	10	706	78.7
Q6ZWQ0	Nesprin-2	42.747	2	12	43	11	6874	782.2
P61358	60S riboso	42.683	51	8	390	8	136	15.8
A2A791	Zinc finger	42.637	9	12	52	12	1549	172.3
P62827	GTP-bindi	42.618	36	7	197	7	216	24.4
Q5F2F2	Protein AB	42.618	22	8	63	8	459	51.1
Q60710	Deoxynucl	42.593	16	10	110	10	658	75.8
Q9CQV8	14-3-3 pro	42.441	41	8	188	5	246	28.1
Q99JF8	PC4 and SI	42.396	18	10	52	10	528	59.7
B1AYL1	Sodium ch	42.349	7	10	57	10	1681	192.1
Q69ZP3	Probable h	42.3	31	8	102	8	385	43
Q02788	Collagen a	42.264	12	10	113	10	1034	110.3
P97376	Protein FR	42.243	43	10	109	10	258	29.1
D2EAC2	Zinc finger	42.228	13	10	52	10	980	109.1
A0A3B2W	Ribosomal	42.191	35	8	154	8	190	21.6

Q8BVE8	Histone-ly:	42.174	7	7	48	7	1365	152.2
A0A087W!	Fibronecti	42.169	6	11	50	11	2266	249.4
F6RL59	Microtubu	42.13	30	7	86	1	349	37.7
A0A0R4J0.	Cytospin-A	42.11	9	7	32	7	1135	126.5
P70279	Surfeit loc	42.102	23	11	257	11	355	41.2
E9Q6L0	CLIP-assoc	42.061	9	10	55	10	1452	160.1
Q9QXY6	EH domair	42.053	22	8	63	1	535	60.8
A0A5F8MI	Histone ac	41.976	6	9	89	9	2053	230.4
Q99ME9	GTP-bindi	41.837	19	12	163	12	634	74.1
A2AM70	Heterochr	41.822	44	5	87	2	150	16.5
Q9CZU3	Exosome F	41.646	13	10	40	10	1040	117.6
Q8C119	Protein NE	41.644	21	9	143	9	568	65
Q8VHK1	Caskin-2 C	41.609	13	10	42	10	1201	126.7
Q8CGC7	Bifunction	41.561	9	9	39	9	1512	170
Q6DFW4	Nucleolar	41.544	19	7	72	7	536	60.3
Q3USZ8	Divergent	41.532	25	8	78	8	430	49.4
Q9CXS4	Centromer	41.506	35	7	115	7	252	27.5
A2BEF2	Disks large	41.463	26	10	51	9	512	57.9
E9Q1W0	Calcium/c	41.452	21	9	113	7	512	57.7
Q8VIJ6	Splicing fa	41.382	15	10	91	9	699	75.4
P58252	Elongation	41.368	11	10	104	9	858	95.3
P60335	Poly(rC)-bi	41.277	29	8	148	6	356	37.5
Q9JIA7	Sphingosir	41.27	16	8	48	8	617	65.6
Q91YI4	Beta-arres	41.228	26	8	105	7	410	46.3
Q8BV49	Pyrin and I	41.221	23	8	73	7	420	46.9
P97384	Annexin A	41.214	22	11	132	10	503	54
Q8CI03	FLYWCH-ty	41.212	21	9	119	9	673	77
P70121	Zinc finger	41.129	15	7	32	7	873	97.5
Q8K2C9	Very-long-	41.019	23	7	100	7	362	43.1
A2AB60	Formin-lik	40.934	11	11	108	7	1090	121.5
Q6ZQ08	CCR4-NOT	40.917	6	12	34	12	2375	266.6
Q9Z315	U4/U6.U5	40.902	15	9	39	9	806	90.8
Q5SWU9	Acetyl-CoA	40.75	6	9	45	9	2345	265.1
E9Q1M1	PDZ doma	40.722	6	11	34	11	2796	296.5
A0A5F8MI	Non-speci	40.721	12	11	56	10	1093	123.7
P62874	Guanine n	40.711	28	9	98	5	340	37.4
Q8C1B7	Septin-11	40.703	25	9	61	5	431	49.7
Q9EP69	Phosphatic	40.623	24	11	52	11	587	66.9
A2AAE1	Transmem	40.424	3	9	27	9	5005	555
A0A0A6YX	cAMP-dep	40.36	23	6	45	4	380	43.1
P67778	Prohibitin	40.301	38	9	67	9	272	29.8
P17012	Zinc finger	40.261	15	9	62	9	799	90
Q6A0D4	Raftlin OS	40.249	23	8	73	8	554	61.5
A0A0R4J0!	Cdc42 effe	40.238	25	6	51	6	409	43
Q8VHX6	Filamin-C	40.225	3	9	159	1	2726	290.9

P48962	ADP/ATP t	40.181	29	10	260	5	298	32.9
P51881	ADP/ATP t	40.071	24	8	226	3	298	32.9
Q9D4J7	PHD finger	40.035	26	6	62	6	364	41.1
Q9QUI0	Transform	39.764	27	6	116	6	193	21.8
Q3UHX9	Putative m	39.656	23	9	90	9	385	42.9
Q99MQ4	Asporin OS	39.574	23	6	150	6	373	42.5
Q52KG9	Chaperoni	39.545	23	9	66	9	531	58
F8VQC7	Kinectin O	39.493	7	7	41	7	1327	152.4
A0A140LH	A-kinase a	39.424	16	10	53	1	607	69.1
Q9R190	Metastasis	39.378	18	11	146	10	668	75
E9PUK6	Serine/arg	39.366	11	7	32	7	923	104.2
Q91Z67	SLIT-ROBO	39.352	12	8	45	7	1071	120.7
Q91W18	Tudor dom	39.284	15	9	76	9	743	82.2
D3Z197	Histone-ly	39.236	8	8	35	8	1397	156
Q9R0Q4	Mortality f	39.223	34	8	121	8	288	32.2
Q91WB4	Pleckstrin	39.204	44	8	38	7	249	27.7
Q5DU25	IQ motif a	39.184	9	10	43	7	1478	161.7
Q14BI5	Myomesin	39.148	6	8	65	8	1463	164.6
O08583	THO comp	39.078	29	6	96	5	255	26.9
Q9CWX4	Mitochond	38.986	21	9	125	9	377	42.4
Q8CI51	PDZ and LI	38.828	19	8	115	8	591	63.3
P63325	40S riboso	38.779	33	6	130	6	165	18.9
Q9D898	Actin-relat	38.765	46	5	62	5	153	17
Q8C0G2	TRAF3-inte	38.747	21	6	29	6	513	58.5
Q9JJ89	Coiled-coil	38.644	25	10	63	10	426	46.5
P26323	Friend leu	38.627	22	7	40	7	452	51
Q6P5B5	Fragile X r	38.543	14	9	53	5	674	74.2
Q8CGF1	Rho GTPas	38.478	11	11	41	11	1266	142.3
Q8QZW8	Rho GTPas	38.431	24	9	47	9	473	53.1
Q3URQ0	Testis-exp	38.429	12	9	54	9	928	105.1
Q6P3B9	Putative ri	38.395	29	8	102	8	350	39.3
Q80TJ7	Histone lys	38.375	11	9	51	8	1023	113.5
Q9D8B3	Charged m	38.337	30	8	170	7	224	24.9
Q9DBR7	Protein ph	38.323	11	9	44	8	1029	114.9
Q8BH02	Torsin-4A	38.29	24	7	79	7	426	47.6
P60843	Eukaryotic	38.28	23	9	122	4	406	46.1
E9Q2I4	Mitotic de	38.265	12	7	33	7	1089	119.6
Q8BZ20	Protein mo	38.223	15	9	27	9	711	79.9
Q6GT24	Peroxiredo	38.222	39	8	132	8	224	24.8
Q91UZ1	1-phospha	38.2	9	11	78	5	1175	134.4
Q6ZWV7	60S riboso	38.152	30	6	1098	6	123	14.5
Q8BP92	Reticuloca	38.13	30	7	53	7	320	37.2
I7HIP8	Nuclear fa	38.091	18	8	142	6	493	55.1
Q80Y44	Probable A	38.084	14	11	59	11	875	100.7
A0A0R4J1	SRSF prote	38.04	21	10	67	8	682	76.8

Q8BP67	60S riboso	37.993	46	10	534	10	157	17.8
Q8BL66	Early endo	37.992	9	11	40	11	1411	160.8
J3QK13	Serine/thr	37.961	11	7	32	7	789	85.1
Q09200	Beta-1,4 N	37.953	19	11	39	11	533	59.2
Q9CR62	Mitochond	37.95	30	9	137	9	314	34.1
E9PYL2	Proline-ric	37.863	6	6	31	6	2035	211.7
G3UY70	Lethal(3)m	37.696	17	8	44	7	739	82.5
Q922I7	Polypyrimi	37.637	17	7	57	5	555	59.3
Q9DB77	Cytochrom	37.631	18	6	26	6	453	48.2
Q8BK63	Casein kin	37.622	29	8	218	1	337	38.9
P49817	Caveolin-1	37.57	44	8	92	5	178	20.5
F8WHM5	Golgi appa	37.56	9	9	28	9	1163	132.3
Q3TFD2	Lysophosp	37.461	15	6	75	6	534	59.7
P48787	Troponin I,	37.396	31	7	145	7	211	24.2
P11214	Tissue-typ	37.387	18	8	81	8	559	63.1
P70224	GTPase IM	37.245	40	9	53	9	277	30.8
Q8R1F1	Protein Ni	37.23	12	9	139	9	749	84.8
Q91V41	Ras-relate	37.205	45	8	69	7	215	23.9
A0A0A0M	Y-box-binc	37.108	21	5	170	1	282	31.4
A0A2I3BS	Vinexin OS	37.107	14	6	31	6	715	80.3
P80316	T-complex	36.957	20	9	70	9	541	59.6
P62983	Ubiquitin-	36.948	35	5	263	5	156	17.9
P63038	60 kDa hea	36.927	17	9	64	9	573	60.9
E9Q3K3	Arf-GAP w	36.914	10	10	59	2	1056	115.7
P17156	Heat shocl	36.868	14	9	200	2	633	69.6
Q9JJG9	Nitric oxid	36.847	18	9	30	9	693	77.3
E9Q7K9	Methyltra	36.842	23	9	86	9	486	55
E9Q3C1	C2 domair	36.832	16	7	62	7	696	76.5
Q9Z2A7	Diacylglyce	36.65	15	6	46	6	498	56.8
Q8VDN2	Sodium/pc	36.621	10	9	104	9	1023	112.9
Q9DC28	Casein kin	36.587	20	5	115	2	415	47.3
Q6P5D3	Putative A	36.565	8	9	39	7	1388	155.7
E9Q4K7	Kinesin far	36.55	6	10	49	8	1843	204.4
Q3UXF4	Nuclear re	36.508	14	6	79	6	531	57.1
Q9CQI1	Pulmonary	36.502	23	6	190	6	248	26.2
P68181	cAMP-dep	36.433	28	8	52	4	351	40.7
P29788	Vitronectii	36.424	19	5	115	5	478	54.8
Q5RJG1	Nucleolar	36.416	15	10	77	10	687	80
O35350	Calpain-1	36.402	17	9	56	9	713	82.1
Q75N62	GTPase IM	36.388	17	8	42	8	688	76.8
Q08857	Platelet gl	36.37	23	6	35	6	472	52.7
A0A0R4J1	Enhancer	36.358	6	6	77	6	1390	150.6
E9PWU7	Kinesin-lik	36.261	12	7	70	6	778	87.4
Q8BVD5	MAGUK p5	36.214	18	8	74	8	576	65.5
K4DI71	GON-4-like	36.125	5	7	42	7	2243	246.7

P62245	40S riboso	36.07	72	9	374	9	130	14.8
Q9D0E1	Heterogen	36.02	16	10	68	10	729	77.6
A2AS45	Plakophilir	35.991	11	11	50	11	1163	128.4
A0A140LH	Mitotic ch	35.95	28	6	54	6	324	36.7
Q8BFU2	Histone H2	35.917	35	5	339	2	130	14.1
Q7TNC4	Putative R	35.877	25	9	80	9	392	46.6
Q8BTU6	RNA helica	35.869	25	9	104	4	362	41.3
Q04750	DNA topoi	35.795	15	10	40	10	767	90.8
Q8C079	Striatin-in	35.787	13	7	33	7	837	95.5
Q921N6	Probable A	35.786	13	11	85	11	760	85.9
Q9WU78	Programm	35.782	11	8	52	8	869	96
P62849	40S riboso	35.733	36	6	121	6	133	15.4
Q9DC07	LIM zinc-b	35.659	24	8	64	7	270	31.1
Q921S7	39S riboso	35.627	20	8	41	8	423	48.3
Q8R429	Sarcoplasr	35.626	11	9	57	1	994	109.4
A0A0R3P9	Replicatio	35.598	18	9	71	9	598	67.7
P62843	40S riboso	35.576	41	4	213	4	145	17
Q924W7	DENN dom	35.564	10	10	85	10	1134	126.8
Q80VD1	Protein FA	35.507	21	8	107	7	429	45.3
Q8CH25	SAFB-like	35.501	9	9	41	9	1031	116.9
Q80UG5	Septin-9 C	35.469	20	11	59	11	583	65.5
A0A5F8MI	PR domair	35.454	9	10	40	10	1172	133.7
Q7JJ13	Bromodon	35.448	11	7	49	5	798	88
E9Q3W1	Casein kin	35.416	27	8	191	1	365	41.9
P80315	T-complex	35.301	20	9	89	9	539	58
E9PWW6	Zinc finger	35.26	9	9	44	7	970	110.7
P19123	Troponin C	35.258	47	7	107	7	161	18.4
Q9D8M4	60S riboso	35.21	35	7	121	7	246	28.5
P62911	60S riboso	35.131	33	6	325	6	135	15.9
Q62181	Semaphor	35.117	14	7	52	7	751	85.2
Q6P5B0	RRP12-like	35.113	10	10	45	10	1295	143
Q8C2Q8	ATP syntha	35.018	20	7	256	7	274	30.2
A2AT93	Phospholip	35.018	36	8	88	2	255	29.4
Q80VQ1	Leucine-ric	34.963	16	7	58	4	524	59.4
Q3TZZ7	Extended s	34.949	12	8	60	8	845	94.1
B1AUB9	Nuclear fa	34.9	17	6	145	3	487	53.6
G3X957	Liprin-beta	34.855	10	7	49	6	882	98.6
Q3UFM5	Nucleolar	34.821	12	9	51	9	854	95.9
Q80WJ7	Protein LY	34.81	15	8	43	8	579	63.8
Q923T9	Calcium/ca	34.801	17	7	66	5	529	59.6
Q3UM18	Large subu	34.794	17	10	55	10	644	73.1
E9Q9E1	Eukaryotic	34.747	7	9	31	8	1593	175.2
Q60668	Heterogen	34.708	20	7	93	1	355	38.3
Q3V1N5	Hdc homol	34.618	20	8	18	8	544	59.1
Q9DBR0	A-kinase a	34.592	16	7	66	7	687	76.2

E0CY98	Traf2 and	34.587	9	10	128	2	1305	148.7
G5E8B9	Zinc finger	34.581	9	9	80	9	1050	118.5
Q9DBS1	Transmem	34.545	17	5	39	5	400	44.8
B7ZC46	Septin OS=	34.542	20	8	65	6	440	50.9
E9QAI5	Aspartate	34.537	6	11	44	11	2162	236.1
A0A1Y7VN	Kinase D-i	34.525	7	10	24	10	1672	185.4
Q61316	Heat shocl	34.51	11	8	59	8	841	94.1
A0A6Q6PU	Pre-mRNA	34.509	9	8	65	8	966	109.8
Q64475	Histone H2	34.485	42	6	562	6	126	13.9
P97496	SWI/SNF (	34.475	11	9	63	5	1104	122.8
Q60973	Histone-bi	34.455	19	6	89	2	425	47.8
P04187	Granzyme	34.391	34	7	109	7	247	27.5
Q5UE59	Kinesin lig	34.354	19	10	69	8	542	61.6
B2RRE7	OTU doma	34.33	8	6	9	6	1107	123
D3YZ08	Telomeric	34.284	17	6	41	6	540	60.1
P70460	Vasodilato	34.257	22	6	67	6	375	39.6
D3YWS8	Leukocyte	34.252	10	6	32	6	801	88.4
E9QLG3	MAX gene	34.248	5	9	32	9	3042	332.9
E9QKR0	Guanine n	34.237	25	8	114	4	382	41.4
P29351	Tyrosine-p	34.214	16	7	34	7	595	67.5
Q8CBA2	Schlafen fa	34.203	11	8	45	8	884	100.8
P70336	Rho-assoc	34.187	7	9	66	8	1388	160.5
Q9QUG9	RAS guany	34.182	16	8	37	8	608	69.4
Q8CI32	BAG famil	34.178	14	6	62	6	447	50.9
Q9CWX9	Probable A	34.16	21	7	114	7	455	50.6
Q6RHR9	Membrane	34.137	9	8	29	8	1471	161.9
P62806	Histone H4	34.124	52	7	290	7	103	11.4
Q5PSV9	Mediator (	34.112	7	5	23	5	1707	184.6
E9Q0J2	Ral GTPas	34.107	6	8	52	8	1507	167.8
P99027	60S acidic	34.087	49	5	88	5	115	11.6
P61750	ADP-ribosy	34.07	39	6	72	2	180	20.4
B2RQC7	DIP2 disco	34.035	8	10	54	7	1340	146.6
Q8BFW7	Lipoma-pr	34.032	18	6	36	6	613	65.8
P28798	Progranuli	34.014	11	4	46	4	589	63.4
Q01853	Transition	33.99	11	7	59	7	806	89.3
Q8CI08	SLAIN mot	33.985	18	8	21	8	581	62.3
Q9QXK7	Cleavage a	33.973	13	8	31	8	684	77.5
P27870	Proto-onco	33.938	12	9	58	9	845	98.1
Q8R4W6	Procollage	33.862	22	5	72	5	414	45.4
Q8CI94	Glycogen p	33.847	12	8	65	8	843	96.7
Q8BFZ9	Erlin-2 OS=	33.826	23	7	57	5	340	37.8
O70311	Glycylpept	33.813	16	6	35	6	529	60.4
Q4VA93	Protein kir	33.812	14	8	60	6	672	76.8
P14869	60S acidic	33.792	24	6	117	6	317	34.2
Q6PGA2	GTP-bindi	33.725	27	6	68	5	307	33.2

Q9WTX6	Cullin-1 OS	33.704	13	9	81	9	776	89.6
Q8R409	Protein HE	33.601	22	6	138	6	356	40.2
H3BLL3	Leucine-ric	33.579	15	6	38	6	649	69.9
Q8CCP0	Nuclear ex	33.574	11	11	27	11	1064	121.1
P08113	Endoplasm	33.474	9	7	85	6	802	92.4
Q3UHU8	General tr	33.442	11	9	50	9	962	108.3
Q8R2V5	Arf-GAP w	33.425	30	9	68	9	381	44
Q80ZM5	H1 histone	33.404	32	8	156	8	188	20.1
D3Z0M9	RNA helica	33.376	14	11	43	11	819	95.4
Q9EPK8	Transient	33.368	13	8	56	8	871	98
Q07797	Galectin-3	33.342	12	4	46	4	577	64.5
Q9DBD5	Proline-, g	33.317	9	7	65	7	1123	118
Q9QZD9	Eukaryotic	33.28	32	8	48	8	325	36.4
P17897	Lysozyme	33.214	35	3	111	2	148	16.8
O08919	Numb-like	33.104	11	7	204	3	604	64.1
P60867	40S riboso	33.098	29	4	232	4	119	13.4
P14824	Annexin A	33.097	17	10	53	10	673	75.8
Q6P5F9	Exportin-1	33.07	9	8	35	8	1071	123
E9PYX7	Afadin OS	33.043	7	9	40	9	1663	188.8
Q8BKW4	rRNA N6-a	33.008	19	8	44	8	512	58.5
P70372	ELAV-like	32.992	29	8	57	8	326	36.1
Q9JK83	Partitionin	32.96	21	5	85	5	371	41
Q8C052	Microtubu	32.886	10	9	49	9	973	102.9
Q99PG2	Opioid gro	32.856	25	9	82	9	633	70.6
Q9WV55	Vesicle-as	32.837	40	7	84	7	249	27.8
E9PUQ9	Piezo-type	32.829	4	8	72	8	2546	291.8
Q8BIE6	FERM dom	32.797	10	8	11	8	1020	113.8
Q6PGG6	Guanine n	32.792	17	8	72	8	577	65.2
Q99J47	Dehydroge	32.763	19	5	40	5	323	35
Q6NZL1	DEAH (Asp	32.744	8	7	68	7	1150	128.2
O55022	Membrane	32.722	49	6	71	5	195	21.7
Q8R370	Usher sync	32.719	19	9	69	9	680	74.9
Q80XK2	Bromodon	32.681	10	9	74	9	1246	140.7
Q91Z92	Beta-1,3-g	32.649	29	7	46	7	325	37
Q61696	Heat shock	32.644	14	7	135	4	641	70
P18760	Cofilin-1 C	32.625	40	8	76	7	166	18.5
P31324	cAMP-dep	32.607	18	5	16	3	416	46.1
E9PV41	Protein dia	32.604	8	9	37	9	1264	140.3
Q64523	Histone H2	32.541	36	4	352	1	129	14
E9PYH6	Histone-ly	32.534	6	7	78	6	1716	185.9
Q8CGC6	RNA-bindi	32.493	12	9	78	9	750	84.2
E9QP59	Inner nucle	32.478	12	8	21	8	918	100.1
Q9D2D7	Zinc finger	32.477	8	8	30	8	1237	130.3
G3X928	SEC23-inte	32.464	10	8	55	8	998	110.7
P84096	Rho-relate	32.451	37	7	124	6	191	21.3

Q3UCQ1	Forkhead l	32.444	13	8	170	6	651	68.4
Q4QQP3	Zinc finger	32.43	17	10	68	2	973	111.4
A0A494BB	PHD finger	32.415	17	7	53	7	558	61
Q922G2	Protein FA	32.395	23	8	61	6	307	35.1
A0A0G2JD	Ras-relate	32.358	70	9	87	1	118	13.4
H9H9S3	Zinc finger	32.322	6	7	42	5	1214	136.4
Q99LH1	Nucleolar	32.319	15	8	21	8	728	83.3
G3X926	Ribosome	32.294	32	7	104	7	273	31.5
Q9D658	Protein tyr	32.26	46	6	61	6	173	19.6
Q6ZPJ0	Testis-exp	32.255	6	8	68	8	1128	125.1
Q8BTU1	Cilia- and	32.243	38	7	101	7	193	22.7
Q9CWT6	Probable A	32.218	17	8	55	8	540	59.5
Q5SSK3	Transcript	32.201	24	7	99	7	364	41.8
Q3V117	ATP-citrat	32.155	11	8	75	8	1101	120.7
P59999	Actin-relat	32.091	47	6	225	6	168	19.7
D3YTT6	Oxysterol-	32.072	12	7	35	7	886	100.4
P68040	Receptor c	32.044	34	7	85	7	317	35.1
G3X9F6	SH3 doma	32.03	6	7	50	7	1346	149.3
F8WHU5	Sister chrc	32.026	7	8	53	8	1449	164.7
Q6PAQ4	RNA exonu	32.025	23	8	80	8	432	47.6
Q8CBY8	Dynactin s	32.019	21	6	51	6	467	53
P84084	ADP-ribosy	32.015	36	5	57	1	180	20.5
P70227	Inositol 1,4	32.006	4	9	36	5	2670	304.1
Q6NZL8	Signal pep	31.992	11	7	70	5	1018	111.5
Q922H9	Zinc finger	31.98	12	4	57	4	316	35.6
Q3U3M5	Cyclin-K OS	31.88	18	6	57	6	582	64.3
P54822	Adenylosu	31.853	15	5	24	5	484	54.8
P48036	Annexin A.	31.841	29	9	88	9	319	35.7
Q61292	Laminin su	31.835	6	7	32	7	1799	196.5
Q569Z6	Thyroid ho	31.799	8	7	48	7	951	108.1
B2KF50	UHRF1 (IC	31.747	8	9	65	9	1429	156.9
Q3TJZ6	Protein FA	31.7	19	6	48	5	515	55
Q9R0P5	Destrin OS	31.671	32	6	52	6	165	18.5
P83887	Tubulin ga	31.664	22	5	32	5	451	51.1
E9PWF0	Thrombos	31.644	11	8	40	7	919	100.1
D0QMC3	Myeloid ce	31.581	21	8	84	1	538	60.5
P35585	AP-1 comp	31.548	23	8	111	8	423	48.5
A2A716	Oxysterol-	31.447	11	6	50	6	837	95
Q9Z2X1	Heterogen	31.435	19	5	78	3	415	45.7
Q9EQ32	Phosphoin	31.395	11	7	31	7	811	90.9
P70268	Serine/thr	31.374	11	9	65	8	946	104.3
Q9Z1M8	Protein Re	31.37	16	9	61	9	557	65.6
Q02526	Zinc finger	31.319	27	5	77	4	198	22.7
Q8K3Z9	Nuclear er	31.286	7	8	49	8	1200	120.9
Q02053	Ubiquitin-	31.279	9	8	41	8	1058	117.7

P47857	ATP-deper	31.278	12	8	44	6	780	85.2
Q6P542	ATP-bindir	31.265	8	5	42	5	837	94.9
Q5NCI0	Up-regula	31.249	10	8	64	8	926	104.6
Q8R054	Sushi repe	31.167	16	6	45	6	468	53.1
O70400	PDZ and LI	31.153	37	7	24	7	327	35.8
Q9CWU3	Serine/thr	31.146	23	6	85	4	367	42.6
P39876	Metallopro	31.128	30	6	234	6	211	24.2
Q61288	Serine/thr	31.106	18	7	100	7	502	56.5
Q91YU6	Leucine zi	31.097	14	8	26	7	671	72.5
Q9QZB7	Actin-relat	31.082	21	6	48	6	417	46.2
P10833	Ras-relate	31.058	37	7	110	6	218	23.7
Q8BGD9	Eukaryotic	31.058	13	5	41	5	611	68.8
Q91YQ7	E3 ubiquit	31.002	20	6	20	4	393	44.4
A2A517	Cytohesin-	30.982	17	5	76	2	460	52.5
Q99P88	Nuclear pc	30.915	8	7	21	7	1391	155
Q8K019	Bcl-2-asso	30.88	6	5	59	5	919	105.9
P62852	40S riboso	30.864	41	8	540	8	125	13.7
Q8K2B3	Succinate	30.795	11	8	78	8	664	72.5
P38647	Stress-70	30.748	14	8	55	7	679	73.4
Q3U4F0	Sideroflex	30.693	31	7	58	7	281	30.9
A0A5F8MI	Septin-4 C	30.675	8	6	36	6	971	110
Q78PY7	Staphyloco	30.512	13	8	50	8	910	102
Q9CS00	Cactin OS=	30.463	13	7	54	7	772	90.6
A0A1L1SR	PR domair	30.439	8	9	79	9	1115	126.4
Q3UG20	Inactive hi	30.406	5	7	20	6	1868	204.4
Q99M87	DnaJ homo	30.39	21	7	42	7	480	52.4
Q9CX11	rRNA-proc	30.38	30	6	62	6	249	28.4
Q8C605	ATP-deper	30.366	11	8	85	7	784	85.5
Q60749	KH domair	30.331	11	7	67	7	443	48.3
O54950	5'-AMP-ac	30.322	24	6	42	6	330	37.5
Q9WTM5	RuvB-like	30.283	19	7	47	7	463	51.1
Q9CYH6	Ribosome	30.28	21	9	94	9	365	41.5
Q9JHK5	Pleckstrin	30.261	11	2	20	2	350	39.9
A0A1Y7VJ	Ena/VASP-	30.251	23	7	49	7	426	46
A0A0A6YV	Glutathior	30.234	52	7	67	7	145	16.5
O35382	Exocyst co	30.219	10	8	49	8	975	110.5
E9QAR6	Zinc finger	30.208	17	8	36	6	568	61.7
Q8BHB0	Nucleotide	30.202	10	9	56	9	953	107.7
Q9D8N0	Elongatior	30.194	15	6	58	6	437	50
O70194	Eukaryotic	30.179	14	6	38	6	548	63.9
P14602	Heat shocl	30.165	31	7	109	7	209	23
Q61081	Hsp90 co-c	30.126	24	8	66	8	379	44.6
Q9EPC1	Alpha-parv	30.108	23	8	99	7	372	42.3
Q8BSH9	Nucleosom	30.09	22	5	38	4	383	44.6
Q9CQ73	Plakophilir	30.077	12	8	66	8	795	88

P61205	ADP-ribosy	30.051	34	5	45	1	181	20.6
Q9Z2N8	Actin-like	29.982	17	6	56	6	429	47.4
Q921H8	3-ketoacyl	29.952	24	6	36	6	424	43.9
P61965	WD repeat	29.948	30	7	63	7	334	36.6
Q91YR1	Twinfilin-1	29.943	21	6	95	5	350	40.1
Q8BI66	Zinc finger	29.918	13	6	63	6	675	73.9
Q9Z1J3	Cysteine d	29.85	14	6	49	6	459	50.5
P55096	ATP-bindir	29.83	12	6	25	6	659	75.4
Q3TYX8	RUN and F	29.822	14	6	24	6	563	64
E9Q6A7	Bromodon	29.747	4	9	37	9	2921	321.4
Q9DBR1	5'-3' exori	29.743	8	5	20	5	951	108.6
A0A140LH	Oxygen-re	29.676	8	8	27	8	1371	153.2
Q8R4H2	Rho guani	29.664	5	8	31	8	1543	172.2
P16330	2',3'-cyclic	29.605	17	7	66	7	420	47.1
O88351	Inhibitor o	29.603	11	8	27	8	757	86.6
Q9CW03	Structural	29.6	10	8	37	8	1217	141.5
Q60634	Flotillin-2	29.559	20	7	34	7	428	47
Q8BMJ3	Eukaryotic	29.549	41	6	67	2	144	16.5
Q9QXW0	F-box/LRR	29.535	13	5	57	5	535	59.2
Q8R2T8	General tr	29.529	18	8	73	8	520	60.5
P46935	E3 ubiquit	29.501	9	6	71	5	887	102.6
Q9D1C9	Ribosomal	29.45	23	5	107	5	280	32.4
A0A0R4IZY	tRNA-dihy	29.442	17	7	40	7	637	71.1
Q07076	Annexin A	29.437	19	8	92	8	463	49.9
P19324	Serpin H1	29.43	19	6	28	6	417	46.5
Q8BPU7	Engulfmer	29.414	10	6	49	4	727	83.9
A0A286YD	G protein-	29.402	15	6	29	5	557	63.5
Q8BMS9	Ras associ	29.35	25	7	47	7	326	37.9
Q8K0U4	Heat shocl	29.339	11	7	56	4	675	74.8
Q8K310	Matrin-3 C	29.298	13	9	59	9	846	94.6
P41105	60S riboso	29.223	30	6	351	6	137	15.7
P54729	NEDD8 ult	29.215	14	7	38	7	614	70.3
Q3UR97	Sorting ne	29.202	20	6	47	6	363	40.3
Q9D735	Telomeras	29.172	43	7	63	7	173	18.4
E9Q4K9	E3 ubiquit	29.149	11	10	94	1	728	83.1
G3UXT7	RNA-bindi	29.14	42	5	76	3	130	14
Q8BU14	Translocat	29.074	12	8	148	8	398	45.6
Q14AX6	Cyclin-dep	29.067	8	8	53	5	1484	163.6
Q99JI6	Ras-relate	29.064	45	9	74	1	184	20.8
Q8K301	Probable A	29.06	14	5	31	5	598	67.4
A0A2I3BP(	Ribosomal	29.034	28	7	273	7	106	12.4
G3X9Q6	La-related	29.014	15	8	45	8	719	79.7
Q8VCM7	Fibrinoger	29.013	17	5	44	5	436	49.4
Q8JZM0	Dimethyla	28.98	25	6	51	6	345	38.9
Q08093	Calponin-2	28.969	24	6	61	5	305	33.1

Q9DCX2	ATP syntha	28.896	46	7	47	7	161	18.7
Q62523	Zyxin OS=I	28.873	16	6	100	6	564	60.5
Q8K3H0	DCC-intera	28.839	12	7	52	7	707	79.3
Q3UHU5	Microtubu	28.816	6	10	20	8	1945	213.7
Q61503	5'-nucleot	28.811	14	7	67	7	576	63.8
A0A0H2UI	Histone-ly	28.726	7	8	30	8	1289	141.2
Q3LAC4	Phosphati	28.695	6	8	34	8	1598	181.6
Q80VN4	AA987161	28.619	12	8	63	1	1045	120.1
E9Q2E4	HECT dom	28.579	3	8	39	8	4418	483.8
Q9JJD0	THAP dom	28.563	20	6	109	6	305	33.3
Q9CQF3	Cleavage a	28.535	26	4	67	4	227	26.2
Q8VCX5	Calcium u	28.434	21	9	76	9	477	54.3
P70426	GTP-bindi	28.413	26	5	102	5	219	25.2
Q9CPT5	Nucleolar	28.396	28	6	127	6	178	21.1
Q9Z0P5	Twinfilin-2	28.335	27	7	54	6	349	39.4
P84104	Serine/arg	28.327	45	7	136	7	164	19.3
Q8BGH2	Sorting an	28.3	17	6	44	6	469	51.8
P14106	Compleme	28.294	22	5	208	5	253	26.7
A0A0U1RI	MICOS cor	28.291	18	7	43	1	488	53.7
Q6NS46	Protein RR	28.285	5	7	48	7	1862	207.6
Q8VCC1	15-hydroxy	28.249	24	5	60	5	269	29.2
P41241	Tyrosine-p	28.137	22	8	48	8	450	50.7
Q9WUN2	Serine/thr	28.126	13	7	25	7	729	83.4
Q921R4	DnaJ homo	28.107	11	8	88	8	703	78.9
G5E8G0	Heterogen	28.085	22	7	93	1	287	30.6
Q9ES46	Beta-parvi	28.061	19	6	45	5	365	41.6
E9Q784	Zinc finge	28.034	5	9	30	9	1729	203.6
Q9EP53	Hamartin	27.963	8	7	39	7	1161	128.7
Q9JJZ6	Krueppel-l	27.955	21	7	71	7	289	31.1
Q99JY0	Trifunctio	27.95	14	6	76	6	475	51.4
Q6PGH1	Protein BU	27.928	49	7	53	7	144	17
Q3U2S8	Voltage-ga	27.92	29	4	14	4	269	31.2
Q9DBG6	Dolichyl-di	27.919	10	3	39	3	631	69
Q640M1	U3 small r	27.903	7	5	32	4	767	87.2
A0A0R4J1	Transcript	27.878	32	6	105	6	199	23.4
Q8VHJ5	Serine/thr	27.779	10	8	58	1	795	88.3
A0A087W	Myeloid ce	27.774	23	8	59	1	485	54.8
Q99MK9	Ras associ	27.733	24	9	77	9	340	38.8
Q8BK35	Ribosome	27.718	18	6	74	6	484	55.8
Q9D024	PAT compl	27.699	17	5	36	5	483	55.8
Q8BK64	Activator c	27.668	20	6	44	6	338	38.1
Q3T9T1	Transmem	27.6	23	6	44	5	471	51.9
Q61792	LIM and SI	27.598	21	7	94	6	263	30
Q64444	Carbonic a	27.597	17	4	62	4	305	34.3
Q8C3F2	Constitutiv	27.59	7	5	60	4	1091	119.6

Q8VDP3	[F-actin]-r	27.57	9	7	48	7	1048	116.7
Q9CQT2	RNA-bindi	27.555	42	6	14	6	265	30.1
A2AUX5	Disabled h	27.554	8	8	59	5	1124	123.9
P27773	Protein dis	27.531	17	7	27	7	505	56.6
A0A5F8MI	Very-long-	27.477	24	6	86	6	206	24
B7ZNL2	Nap1l4 pro	27.443	17	4	55	3	386	43.9
Q6W4W7	DIA3 OS=M	27.436	7	7	48	7	1102	125.3
P45481	Histone lys	27.429	4	8	29	3	2441	265.3
Q8C2Q3	RNA-bindi	27.42	12	6	23	6	669	69.4
O89079	Coatomer	27.411	20	5	30	5	308	34.5
F8VQ28	Paxillin OS	27.345	14	7	68	7	591	64.5
E9PU93	Set1/Ash2	27.267	17	6	47	6	534	60.1
Q6PIX5	Inactive rh	27.256	9	7	50	7	856	97.2
Q6PCQ0	IQ domain	27.254	12	7	30	7	778	86.3
A0A0R4J0	Histone de	27.182	16	6	68	4	488	55.3
E9Q4M2	Hormone-s	27.057	7	7	91	7	1072	117.2
Q8CBM2	Aspartyl/a	27.054	12	6	34	6	658	75.1
O54833	Casein kin	27.046	25	6	26	6	350	41.2
Q08943	FACT com	27.04	10	6	49	6	708	80.8
Q9D937	Uncharact	27.034	40	5	131	5	123	14.1
Q8CH09	SURP and	27.006	7	8	46	8	1067	118
Q6PAC3	DDB1- and	26.972	13	5	58	5	445	51.4
P62821	Ras-relate	26.966	33	7	93	3	205	22.7
O89053	Coronin-1A	26.959	18	9	114	9	461	51
Q61990	Poly(rC)-bi	26.958	20	6	84	3	362	38.2
H7BXC3	Triosephos	26.949	49	5	42	5	167	18
A0A0U1RI	Zinc finger	26.943	14	7	73	5	565	62.4
Q80TM9	Nischarin	26.913	5	6	21	6	1593	174.9
Q9D666	SUN doma	26.822	10	6	33	5	913	101.9
Q9Z2B9	Ribosoma	26.814	11	8	38	8	773	85.6
E9Q1S3	Protein tra	26.732	9	5	82	3	736	82.9
B1AZ46	Brain-spec	26.707	13	5	29	5	521	57.6
P63168	Dynein ligh	26.582	63	4	118	2	89	10.4
P62320	Small nucl	26.547	48	4	79	4	126	13.9
A2BFF8	Cytoplasm	26.497	12	8	52	8	611	68.2
P16460	Argininosu	26.476	15	5	41	5	412	46.6
P47915	60S riboso	26.474	36	6	329	6	160	17.6
Q3TUQ7	Acetyl-CoA	26.448	15	7	35	7	550	62.7
Q91Z49	UAP56-int	26.401	23	7	77	7	317	35.9
Q505F5	Leucine-ric	26.386	13	6	51	6	581	63.6
A0A0A0M	Microtubu	26.361	10	6	59	6	733	76.2
Q61166	Microtubu	26.318	24	7	65	6	268	30
P61982	14-3-3 pro	26.272	28	6	128	3	247	28.3
Q91YZ8	Polyadeny	26.245	10	5	52	1	615	67.8
A2AI52	Ubiquitin	26.199	8	8	22	8	1091	123.8

Q8CCK0	Core histo	26.188	18	6	40	5	372	40.1
Q8CI43	Myosin lig	26.099	20	4	88	2	207	22.7
Q69ZX8	Actin-bind	26.084	10	5	45	5	682	77.6
Q3TWJ1	Retinoic a	26.069	16	5	14	5	524	56.3
A0A286YE	Histone-ly	26.059	4	8	29	8	2588	283.9
A0A668KL	Non-speci	26.056	5	7	40	3	1714	188.5
Q05144	Ras-relate	26.04	33	7	206	2	192	21.4
Q9CX86	Heterogen	26.027	23	5	56	5	305	30.5
Q99LH4	Zinc finger	26.022	17	7	59	7	468	52.1
Q7TPD1	F-box only	25.997	9	7	28	7	930	103.7
Q9QVP4	Myosin reg	25.995	24	6	88	6	175	19.4
Q8K2R5	Zinc finger	25.988	14	8	45	8	619	68.3
P68404	Protein kir	25.971	12	7	39	5	671	76.7
Q9JJI8	60S riboso	25.962	36	4	207	4	70	8.2
Q9D3W6	POC1 cent	25.958	19	6	23	6	434	48.9
Q9DCC5	Cbx3 prote	25.939	30	4	31	4	183	20.8
Q9JM93	ADP-ribosy	25.924	29	6	47	6	229	25.5
O88844	Isocitrate	25.884	17	6	30	6	414	46.6
Q80TE0	RNA polym	25.856	6	7	15	7	1409	155.2
Q9Z2E1	Methyl-Cp	25.827	17	4	48	4	414	43.5
Q8CBW3	Abl interac	25.775	12	6	35	6	481	52.3
B1B1D8	39S riboso	25.744	18	6	112	6	304	33.1
Q8R0X7	Sphingosir	25.685	11	6	29	6	568	63.6
O88532	Zinc finger	25.664	6	5	18	5	1074	116.8
Q9CY64	Biliverdin	25.614	27	5	29	5	295	33.5
O35730	E3 ubiquit	25.575	20	6	11	5	406	42.6
P57784	U2 small r	25.571	26	6	84	6	255	28.3
Q02956	Protein kir	25.567	15	6	51	5	592	67.6
Q8BHF7	CDP-diacyl	25.562	15	6	31	6	553	62.5
Q60597	2-oxogluta	25.558	9	7	26	7	1023	116.4
O35130	Ribosoma	25.481	26	5	40	5	244	27
E0CYH0	Female-le	25.476	15	4	39	4	396	44.2
E0CXC5	PTB doma	25.464	18	6	25	6	299	34.2
P17182	Alpha-eno	25.451	17	7	35	7	434	47.1
Q8BL97	Serine/arg	25.421	24	7	106	7	267	30.8
E9PZ11	Zinc finger	25.381	10	5	80	2	729	80.7
Q8CGA0	Protein ph	25.328	18	5	50	5	452	49.6
E9QLQ3	Phosphodi	25.326	7	4	11	4	1099	122
Q99020	Heterogen	25.288	21	7	74	6	285	30.8
Q91VL8	Telomeric	25.202	25	7	25	7	393	43.3
Q99K94	Signal trar	25.181	10	7	39	7	712	83.1
Q5SWU0	DNA-bindi	25.166	17	6	51	4	428	47.9
E9PYL4	Chloride cl	25.131	7	4	83	4	783	86.4
A2AA59	E3 ubiquit	25.101	14	6	59	6	576	65.4
A0A498W	Asparagin	25.093	16	8	34	8	558	64.2

Q99PU7	Ubiquitin c	25.084	11	6	49	6	728	80.4
Q810D6	Glutamate	25.081	20	6	54	6	446	49.2
Q8CJG0	Protein arg	25.08	10	8	46	5	860	97.2
Q9QXV1	Chromobo	25.057	23	7	38	7	362	39.8
Q99JX4	Eukaryotic	24.999	14	5	24	5	374	42.5
E9QP62	LIM and se	24.955	17	6	112	5	387	44.3
G3X972	Sec24-rela	24.949	6	4	43	4	1096	118.5
Q9CQL1	Protein ma	24.94	41	6	45	6	146	17.1
O88456	Calpain sm	24.915	18	3	33	3	269	28.4
Q8BMQ2	General tr	24.896	11	8	44	8	817	91.6
Q6NSQ5	Sp140 nuc	24.876	16	7	31	7	534	62.6
Q8VDY9	Caspase a	24.86	16	4	36	4	356	37.8
A0A3Q4EI	Ras-relate	24.833	32	6	61	6	212	23.8
Q8C6B0	Methyltrai	24.81	22	4	100	4	244	28.1
Q6PEE2	CBP80/20-	24.785	12	6	25	6	600	67.8
O54786	DNA fragn	24.768	24	6	38	6	331	36.6
Q8R1Q8	Cytoplasm	24.742	15	7	50	6	523	56.6
A2BDX2	Dolichol-p	24.726	35	5	51	5	208	23.4
E9QN31	Probable 2	24.709	9	6	46	6	794	86.9
A0A1D5RL	60S riboso	24.704	53	8	132	8	147	17.4
Q8CB77	Elongin-A	24.698	8	5	24	5	773	87.1
Q9EPL8	Importin-7	24.692	7	5	61	5	1038	119.4
A0A1Y7VJ	Dystrobrev	24.665	16	7	39	6	567	64.7
E9Q7G0	Nuclear m	24.661	5	8	17	8	2094	235.5
Q68FM7	Rho guani	24.659	5	6	31	6	1552	171.7
Q3UQ44	Ras GTPas	24.631	5	7	88	4	1575	180.4
Q6KCD5	Nipped-B-	24.584	3	6	15	6	2798	315.3
Q99KC8	von Willeb	24.558	8	6	42	6	793	87.1
P54071	Isocitrate	24.526	17	7	36	7	452	50.9
P68510	14-3-3 pro	24.519	24	6	141	3	246	28.2
O09161	Calseques	24.498	14	5	26	5	415	48.1
Q9WVC3	Caveolin-2	24.492	32	3	35	3	162	18.2
Q9JKX4	Protein AA	24.352	12	4	12	4	526	59.4
A0A0B4J1	Obscurin-l	24.352	9	8	21	8	1025	113.8
A2AU83	Predicted	24.326	14	6	40	1	715	83.4
Q8K031	StAR-relat	24.302	7	6	42	6	1019	113
A2CG76	Histone-ly	24.294	9	6	27	6	1229	134.6
Q6PAR5	GTPase-ac	24.272	7	6	20	6	1458	162.3
P60898	DNA-direc	24.218	32	2	9	2	125	14.5
O35295	Transcripti	24.218	20	5	86	5	324	33.9
A0A1B0GS	60S riboso	24.199	13	6	148	6	446	50.8
E0CXB9	Alpha N-ca	24.15	8	6	52	1	966	106.7
Q8VHE0	Translocat	24.144	15	8	30	8	760	87.8
P52633	Signal trar	24.136	8	6	32	6	837	93.4
Q61771	Kinesin-lik	24.132	11	7	23	6	747	85.2

Q8BIJ7	RUN and F	24.073	10	7	34	7	712	80.3
Q00899	Transcript	24.047	22	7	27	7	414	44.7
Q922B2	Aspartate-	24.036	14	5	77	5	501	57.1
Q8BSD5	SH2 doma	24.035	16	6	28	6	492	53.3
Q91VA7	Isocitrate	24.025	11	4	48	4	384	42.2
Q8R349	Cell divisic	24.013	13	6	27	6	620	71.4
Q8R1G6	PDZ and LI	23.995	12	4	56	4	349	37.7
P22518	Dual speci	23.986	16	6	21	5	483	57.1
Q8CE64	Zinc finger	23.981	10	5	42	5	614	67.3
Q9WUU9	Germinal-	23.965	5	5	15	5	1971	217.2
Q9CR02	Translatio	23.96	26	5	52	5	221	25.8
P97425	Eosinophil	23.951	31	6	119	6	156	17.6
Q99PT1	Rho GDP-c	23.946	30	4	51	4	204	23.4
P61514	60S riboso	23.93	38	3	300	3	92	10.3
Q91YR7	Pre-mRNA	23.928	8	6	13	6	941	106.7
Q99MU3	Double-str	23.922	7	8	50	8	1178	130.4
O35343	Importin s	23.898	14	6	46	2	521	57.9
P62889	60S riboso	23.887	48	4	163	4	115	12.8
Q3US17	Zinc finger	23.881	17	6	35	5	591	64.6
Q9CRA9	FGFR1 onc	23.869	20	4	16	4	253	29.4
P97742	Carnitine C	23.833	9	7	46	7	773	88.2
Q8BG81	Polymeras	23.806	15	4	35	4	420	46.1
Q8K212	Phosphofu	23.793	11	8	47	8	961	104.8
Q9QVP9	Protein-tyr	23.784	8	7	39	6	1009	115.7
Q9QYL7	Activator c	23.773	26	7	70	7	269	30.6
Q8BPM0	Disheveled	23.771	7	7	26	6	1077	123.3
Q99LG0	Ubiquitin c	23.754	9	6	16	6	825	93.4
P11881	Inositol 1,4	23.68	3	7	34	1	2749	313
Q9JJA9	General re	23.679	17	6	37	6	392	42.3
A0A0G2JF	Integrator	23.663	6	5	21	5	1035	117.2
Q3TLP8	Ras-relate	23.61	26	6	204	1	211	23.4
F8WJA0	RNA helica	23.594	8	5	54	5	903	101.3
Q8C3X8	Lipase ma	23.575	10	4	30	4	702	79.9
A0A2R8VH	PHD finger	23.566	9	7	57	7	987	111
Q3UFB2	Box C/D sr	23.526	12	4	76	4	460	52.2
Q8BX10	Serine/thr	23.522	23	6	45	6	288	32
Q923D5	WW doma	23.516	8	7	57	7	641	69.8
L7N267	Zinc finger	23.515	22	6	40	2	546	64
Q8BUK6	Protein Ho	23.511	12	5	22	5	718	83.2
A0A0R4J2	Dedicator	23.482	4	6	29	6	1816	207.4
Q7TPN9	Proline-ric	23.459	11	6	18	6	612	67.8
Q60980	Krueppel-l	23.449	17	7	64	4	344	38.5
Q80Z96	Vang-like	23.421	14	6	37	6	526	60
Q8BHL5	Engulfmer	23.406	10	5	22	3	732	83.8
O70310	Glycylpept	23.405	10	3	27	3	496	56.9

Q921G9	Tight junct	23.399	6	6	119	5	904	99.1
F8VQ70	S phase cy	23.395	7	7	25	7	1398	157.7
Q8C166	Copine-1 C	23.388	10	5	35	4	536	58.8
Q8BT60	Copine-3 C	23.288	12	6	53	5	533	59.5
P31428	Dipeptidas	23.261	15	5	27	5	410	45.7
A0A1W2P	40S riboso	23.247	35	5	115	5	148	16
A0A494BB	Eukaryotic	23.208	18	5	61	1	254	28.6
Q91VT4	3-oxoacyl-	23.165	26	5	46	5	236	25.4
Q9JLB2	Protein PA	23.143	9	5	50	5	675	77.2
P05132	cAMP-dep	23.123	19	6	55	2	351	40.5
P70275	Semaphor	23.084	7	4	35	4	775	89.5
A0A0G2JD	Ubiquitin-	23.06	5	4	52	4	1067	112.4
Q60644	Oxysterols	23.028	15	5	21	5	446	49.7
Q3UW53	Protein Ni	23.018	10	6	30	6	926	102.6
Q921T2	Torsin-1A-	22.985	17	7	34	7	595	66.7
Q8R2K4	TAF6-like	22.951	11	6	19	6	616	67.2
A0A0R4J0	Zinc finger	22.94	16	6	15	6	580	65.1
Q99JY3	GTPase IM	22.92	17	5	39	5	328	38
F6WIU1	Protein KR	22.92	9	5	32	5	705	82.2
G3UY93	Valyl-tRNA	22.9	7	5	52	5	1278	141.3
P60670	Nuclear pr	22.846	11	5	41	5	608	68
B2RR83	3'-5' RNA	22.844	5	6	43	6	1445	161
P67984	60S riboso	22.821	24	4	108	4	128	14.8
G3X9G7	Zinc finger	22.814	19	6	57	6	402	46.9
Q9D4H1	Exocyst co	22.749	9	6	28	6	924	103.9
Q62425	Cytochrom	22.734	48	6	71	6	82	9.3
Q9DC51	Guanine n	22.733	18	6	57	3	354	40.5
O35326	Serine/arg	22.676	20	5	81	4	269	30.9
P10922	Histone H1	22.641	25	6	383	6	194	20.8
B2RWS6	Histone ac	22.632	3	7	21	2	2412	263.1
Q9CYA6	Zinc finger	22.587	9	4	18	4	709	78
Q80T06	Elongation	22.575	20	4	51	1	281	31.3
Q5DTX6	Junctional	22.573	7	6	24	6	1320	144.7
Q3U2K0	Protein FA	22.569	13	5	20	5	892	95
P62715	Serine/thr	22.549	17	4	58	1	309	35.6
Q9JJF3	Ribosoma	22.549	14	6	19	6	603	67.5
Q9ESW4	Acylglycer	22.547	12	3	9	3	421	46.9
Q99LR1	Lysophosp	22.537	15	5	50	5	398	45.2
A2BE23	Novel KRA	22.533	27	6	54	2	498	57.6
Q6KAR6	Exocyst co	22.528	8	6	32	6	755	86.4
Q9Z1D9	Zinc finger	22.519	16	6	35	5	521	59
Q99LX5	Multiple m	22.517	18	4	53	4	260	29.3
Q8VDM4	26S protea	22.498	7	6	34	6	908	100.1
Q60598	Src substra	22.485	17	7	39	7	546	61.2
A6H8H2	DENN dom	22.462	4	7	31	7	1906	211.3

Q8K426	Resistin-li	22.454	38	4	101	4	117	12.5
Q8BQM4	HEAT repe	22.443	12	5	36	5	679	74.3
Q9EP97	Sentrin-sp	22.323	10	4	89	4	568	64.4
B2RXQ4	Nek1 prote	22.319	9	8	17	8	1178	133.8
Q921M7	CYFIP-rela	22.297	20	4	29	4	324	36.8
P63330	Serine/thr	22.26	17	4	60	1	309	35.6
Q9Z1N5	Spliceosor	22.233	16	6	60	6	428	49
Q91WJ7	SPATS2-lik	22.232	10	5	78	5	558	61.6
Q9QY76	Vesicle-as	22.224	23	6	44	6	243	26.9
Q4VA98	Zfp219 prc	22.211	14	6	39	5	681	73.2
Q9D8U0	Interferon-	22.204	17	6	37	6	441	47.7
P97499	Telomeras	22.185	4	6	19	6	2629	291.3
P01901	H-2 class I	22.165	23	5	42	4	369	41.3
Q08288	Cell growt	22.149	25	7	21	7	388	43.7
Q91YL3	Uridine-cy	22.131	14	5	52	5	548	60.8
Q8BUR4	Dedicator	22.13	3	6	24	6	1865	214.9
Q80U72	Protein scı	22.119	4	5	43	2	1612	174
Q4VBE8	WD repeat	22.108	15	5	31	5	431	47.2
Q9CPR7	Suppresso	22.079	31	4	15	4	207	23.5
Q9D7S7	60S riboso	22.067	45	4	106	4	122	14.5
Q60932	Voltage-de	22.026	20	5	34	4	296	32.3
P62317	Small nucl	21.962	32	4	57	4	118	13.5
Q61187	Tumor sus	21.932	15	5	36	5	391	44.1
A2CG35	Ras-relate	21.923	18	6	41	5	291	32.2
B2RXC1	Trafficking	21.915	5	5	25	5	1133	128.3
Q9DCH4	Eukaryotic	21.906	17	5	64	5	361	38
P58404	Striatin-4 (	21.895	10	6	19	4	760	81.6
A0A1B0G)	BCL2-asso	21.879	7	5	17	5	1107	115.3
P97450	ATP syntha	21.874	34	4	28	4	108	12.5
Q9CPS7	RNA-bindi	21.868	23	4	16	4	248	27.4
Q91XD2	LIM and se	21.844	17	6	35	5	341	39
Q8K4Q7	Ceramide	21.827	11	5	39	5	531	59.8
H3BJY2	Arf-GAP w	21.759	8	6	14	6	1144	126.9
Q9DCN2	NADH-cytc	21.734	24	6	19	6	301	34.1
Q9ERR8	Zinc finger	21.725	11	6	34	4	581	65.6
Q9Z2X8	Kelch-like	21.72	10	5	29	5	624	69.5
Q60843	Krueppel-l	21.703	21	4	34	4	354	37.6
O35368	Interferon-	21.687	16	6	36	5	408	46.3
Q9DCG9	Multifunct	21.676	53	4	20	4	125	14.1
Q99J25	rRNA met	21.663	22	5	52	5	320	34.8
D3YZ09	H/ACA ribc	21.661	25	5	71	5	198	20.4
Q6NWW9	Fibronecti	21.66	6	4	50	4	1207	132.7
Q5XJY5	Coatomer	21.659	13	6	30	6	511	57.2
Q91V81	RNA-bindi	21.62	11	3	44	3	478	50.2
A2AR02	Peptidyl-pi	21.618	6	5	71	5	752	88.3

Q8BYH8	Chromodo	21.585	2	5	19	4	2885	323.7
P26350	Prothymos	21.584	23	4	26	4	111	12.2
Q9JHN8	Serine/thr	21.567	24	5	31	5	254	28.1
Q9WUM3	Coronin-1E	21.565	14	5	61	5	484	53.9
P61222	ATP-bindir	21.564	11	5	28	5	599	67.3
Q5F293	Zinc finger	21.492	7	6	44	5	982	102.6
Q6P1Y9	Exocyst co	21.491	7	6	18	6	901	102.6
J3QNU6	Beta-arres	21.485	12	5	81	4	419	47.1
Q3ULL6	UPF3 regu	21.482	11	5	28	5	472	57
A0A0R4J2	Extracellul	21.476	8	5	29	5	862	99.9
Q8R323	Replicatio	21.451	13	3	22	3	356	40.5
Q8VD75	Huntingtin	21.423	7	5	33	5	1029	115.1
E9QMU3	Ensconsin	21.411	11	5	16	4	730	81.9
Q8JZR0	Long-chair	21.41	10	6	21	4	683	76.2
D3YVM9	Signal pep	21.402	6	4	26	2	968	106.6
H7BX08	Calmoduli	21.39	7	7	15	7	1478	166
O35344	Importin s	21.383	13	7	52	3	521	57.7
P60229	Eukaryotic	21.381	15	6	67	6	445	52.2
P36371	Antigen pe	21.324	9	6	29	6	702	77.4
Q8C9V1	Carabin OS	21.315	14	6	53	6	444	49.9
O35182	Mothers a	21.303	13	4	33	4	495	53.7
Q9JI33	Netrin-4 C	21.301	13	6	19	6	628	69.8
P61406	Telomeras	21.295	8	7	13	7	1418	160.4
D3Z5N6	Zinc finger	21.222	17	4	21	4	405	45.8
Q9DC48	Pre-mRNA	21.221	13	6	24	6	579	65.4
P59997	Lysine-spe	21.201	7	5	10	5	1161	132.6
A0A338P6	Zinc finger	21.09	10	5	14	5	752	83.7
A0A1B0GF	Receptor-t	21.044	9	4	14	4	719	82.6
Q9WVG9	Male-spec	21.021	11	5	50	5	525	60.3
G3UX26	Outer mito	21.015	19	5	57	5	283	30.4
Q3TIX9	U4/U6.U5	21.013	11	5	22	5	564	65.1
A0A0R4J0	Prolow-de	20.912	1	4	20	4	4545	504.4
Q64462	Cytochrom	20.826	10	6	63	6	511	58.9
Q9D554	Splicing fa	20.825	12	5	19	5	501	58.8
Q80U35	Rho guani	20.825	3	5	53	5	2057	221.5
Q922V4	Pleiotropic	20.71	10	3	10	3	513	56.9
H7BX01	Dynamin-l	20.667	6	5	40	5	978	113.2
Q9D945	Protein LLI	20.665	33	4	51	4	130	15.4
Q4FZF3	Probable A	20.644	10	4	42	4	480	54.1
Q8BWY3	Eukaryotic	20.641	14	5	35	5	437	49
Q9CQ65	S-methyl-5	20.596	24	5	43	5	283	31
E9Q8T2	PR domair	20.581	5	7	37	7	1174	132.8
Q99KN1	Arrestin do	20.566	12	5	84	5	434	46.3
Q8R3N6	THO comp	20.56	10	7	16	7	657	75.4
Q3TJD7	PDZ and LI	20.544	10	3	8	3	457	50.1

O35218	Cleavage a	20.531	8	5	15	5	782	88.3
Q6P9R1	ATP-deper	20.528	9	5	24	5	639	70.3
Q8K2L8	Trafficking	20.515	7	5	18	5	797	87.6
Q9DC33	High mobi	20.485	14	4	70	4	346	39.9
Q62137	Tyrosine-p	20.472	7	7	34	7	1100	122.6
D3YUY3	Phosphatic	20.447	11	4	13	4	497	55.3
F6ZFU0	Elongation	20.305	10	4	34	1	552	61.4
P10649	Glutathion	20.212	21	4	38	4	218	26
Q9R1C0	Transcripti	20.153	18	5	17	5	341	39.1
O35841	Apoptosis	20.135	13	5	13	5	504	56.8
P01899	H-2 class I	20.088	20	6	20	5	362	40.8
Q91WG4	Elongator	20.086	7	5	26	5	831	93
A0A668KL	A-kinase a	20.07	6	6	22	6	1604	172.5
Q8K3B1	F-box/SPR	20.06	17	5	30	5	286	30.6
A2A4N9	RNA helica	20.053	7	6	19	6	1174	134.3
Q9ESP1	Stromal ce	20.044	33	5	20	5	221	23.6
Q31125	Zinc transp	20.011	7	3	32	3	476	50.6
Q8VCE9	Pleckstrin	20.007	11	6	20	6	796	85.8
Q8R0L9	Transcripti	19.99	13	4	15	4	432	48.9
B1ASA5	Zinc finger	19.967	18	6	36	4	418	45.6
Q9EQI8	39S riboso	19.953	22	6	50	6	283	32.1
O88632	Semaphor	19.927	9	5	29	4	785	88.5
Q9JMD1	Scm-like w	19.927	6	4	30	4	863	97.3
Q61878	Bone marr	19.873	21	3	36	3	223	24.2
Q91WD5	NADH deh	19.869	10	5	21	5	463	52.6
Q9D819	Inorganic p	19.86	17	3	16	3	289	32.6
A0A0G2JE	Nexilin OS	19.857	8	6	40	6	671	80
Q3UDC3	Target of I	19.849	13	5	32	5	516	57.1
P55097	Cathepsin	19.829	13	3	21	3	329	36.9
O70585	Dystrobrev	19.818	10	4	24	3	659	74.4
E9QQ91	Transcripti	19.814	7	3	75	2	459	51.2
Q61937	Nucleopho	19.809	25	4	19	4	292	32.5
P09055	Integrin be	19.788	7	4	30	4	798	88.2
Q3TDD9	Protein ph	19.767	6	4	23	4	780	88.3
E9QAG4	RIKEN cDN	19.747	14	6	54	4	666	78.4
A0A0A6YV	Cyclic nucl	19.693	4	6	26	6	1644	184
O54788	DNA fragn	19.676	13	3	13	3	344	39.4
A0A1W2P	Cysteine a	19.676	20	3	51	3	197	21.6
D3Z3L3	Tripartite	19.67	10	4	23	4	497	57.8
Q8BTV2	Cleavage a	19.669	15	6	46	6	471	52
Q61581	Insulin-like	19.651	25	5	68	5	281	29
Q9ESX5	H/ACA ribo	19.635	11	3	16	3	509	57.4
Q922M3	BTB/POZ d	19.634	18	5	32	5	315	35.7
P62855	40S riboso	19.631	37	4	151	4	115	13
A0A0A0M	Eukaryotic	19.566	41	5	28	5	149	16.3

Q8VDJ3	Vigilin OS=	19.555	5	5	31	5	1268	141.7
Q8K2D3	Enhancer c	19.554	8	3	24	3	508	55.9
Q6WKZ8	E3 ubiquit	19.553	4	5	32	5	1755	199
Q8JZU2	Tricarboxy	19.512	14	5	82	5	311	33.9
Q99LE6	ATP-bindir	19.499	8	5	19	5	628	71.7
A2ARW8	Predicted g	19.496	23	5	49	2	610	70.9
A0A2I3BQ	Non-specit	19.494	11	5	30	3	495	56.6
P50544	Very long-	19.459	7	4	45	4	656	70.8
Q5U5Q9	BRCA1-A c	19.456	7	5	15	5	727	81.4
F6YZ95	Liprin-beta	19.444	20	3	50	1	193	20.5
E9QAF9	Protein TA	19.425	4	6	17	6	1849	199.9
E9PUD2	Dynamin-1	19.422	10	5	23	5	712	79.5
Q921Y2	U3 small r	19.411	25	4	42	4	184	21.8
Q921M3	Splicing fa	19.394	5	5	11	5	1217	135.5
G5E898	Periplakin	19.393	4	5	17	5	1754	203.7
Q91YR5	eEF1A lysi	19.386	10	6	26	6	698	78.7
Q91VJ5	Polyglutan	19.384	19	2	13	2	263	30.6
Q6P8X1	Sorting ne	19.381	17	7	41	7	406	46.6
Q80TE4	Signal-ind	19.374	4	5	25	4	1722	189.3
Q9JLV5	Cullin-3 OS	19.372	8	6	24	5	768	88.9
Q99JP6	Homer pro	19.364	15	6	30	6	356	39.7
E9Q264	Myosin, he	19.337	2	5	121	1	1925	221.7
Q6PD29	Zinc finger	19.333	12	5	22	4	541	58
A0A0R3P9	NADH deh	19.318	14	4	28	4	373	42.1
P63328	Serine/thr	19.311	11	6	30	6	521	58.6
Q6Q899	Antiviral ir	19.283	7	5	44	5	926	105.9
Q3UMF0	Cordon-ble	19.265	5	4	24	4	1273	137.3
E9PUA5	Kinesin-lik	19.258	11	5	19	2	672	73.6
A0A5F8MI	Tenascin X	19.252	2	6	19	6	4356	473.8
Q3TFP0	Serine/arg	19.248	23	5	18	5	182	22.1
E9PWH2	Caspase re	19.244	6	6	23	6	1175	131.4
Q9CR57	60S riboso	19.225	15	3	87	3	217	23.5
Q7TPX5	Expressed	19.216	19	5	28	3	406	47.9
Q8K4Z5	Splicing fa	19.21	7	6	29	6	791	88.5
A0A140LJ7	Synemin O	19.199	8	6	22	6	674	76.7
A0A5F8MI	Polycomb	19.177	11	5	57	5	500	56.1
Z4YL66	Chromatin	19.141	6	5	15	5	1141	131.6
P28301	Protein-lys	19.14	12	3	17	3	411	46.7
Q9CZ13	Cytochrom	19.124	10	4	44	3	480	52.8
Q8BHL4	Retinoic a	19.122	13	3	37	3	356	40.1
P62915	Transcripti	19.093	20	6	20	6	316	34.8
Q8BG17	Nucleolar	19.09	24	3	41	3	217	25.3
A0A494BB	Ras-relate	19.07	31	5	68	1	189	21.1
Q9CR86	Calcium-re	19.065	45	4	43	4	148	16.1
O70325	Phospholip	19.056	25	5	90	5	197	22.2

P10820	Perforin-1	19.054	12	5	35	5	554	62
Q5SRY7	F-box/WD	19.04	12	5	19	5	542	62
P62259	14-3-3 pro	19.028	19	5	130	2	255	29.2
Q9D2H6	Transcript	19.024	11	4	36	4	612	64.9
A2AQE2	SECIS bind	19.011	9	5	7	5	1089	119.7
Q9R0A0	Peroxisom	18.973	19	5	13	5	376	41.2
Q9JJA4	Ribosome	18.97	9	3	36	3	423	47.3
Q6A028	Switch-ass	18.95	8	4	32	4	585	69
Q9DBS5	Kinesin lig	18.915	15	7	19	6	619	68.6
Q5U4C3	Splicing fa	18.906	6	6	29	6	1256	133.8
O88783	Coagulatic	18.877	3	5	6	5	2183	247.1
G3UXL2	Ribose-ph	18.871	18	6	42	4	318	34.8
P59222	Scavenger	18.868	10	5	16	5	833	87.8
Q9Z2D1	Myotubula	18.855	13	7	43	6	643	73.2
Z4YKC4	Eukaryotic	18.837	3	4	15	3	1567	173.4
Q8C6P8	Zinc finger	18.823	11	4	13	4	421	47.9
Q7TN16	Hedgehog-	18.817	9	4	18	4	700	78.5
Q8BVQ9	26S protea	18.789	11	5	43	5	475	52.8
P35969	Vascular e	18.773	5	6	37	5	1333	149.8
B1AZI6	THO comp	18.762	3	5	20	5	1594	182.7
P59016	Vacuolar p	18.76	11	5	13	5	617	70.5
Q5SRX1	TOM1-like	18.732	15	4	19	4	507	55.6
Q91VE6	MKI67 FHA	18.719	19	4	35	4	317	36.2
Q8VD65	Phosphoin	18.716	6	5	29	5	1358	152.5
E9QPI5	Sister chro	18.715	5	4	21	4	1332	150.1
G5E8J3	WD repeat	18.705	5	4	14	4	1223	135.9
Q7TT28	RNA exonu	18.685	6	5	12	5	1213	130.7
Q9JMD0	BUB3-inte	18.659	7	3	99	3	495	52.8
Q8K1K2	26S protea	18.645	16	4	30	4	348	38.7
Q6RI63	Constitutiv	18.643	8	4	11	4	786	89.3
A0A2R8VK	Cleavage a	18.628	4	6	31	6	1450	161.7
Q810V0	U3 small r	18.603	8	4	22	4	681	78.7
Q9EQ06	Estradiol 1	18.59	16	4	28	4	298	32.9
P08207	Protein S1	18.582	28	4	121	4	97	11.2
Q3B7Z2	Oxysterol-	18.579	9	5	29	5	805	88.7
B1AVH5	Coronin OS	18.577	8	3	12	3	543	61.7
P61021	Ras-relate	18.576	25	4	51	2	215	23.7
Q8K2I3	Dimethyla	18.56	11	5	25	5	535	60.9
Q9R1T4	Septin-6 C	18.557	15	5	28	1	434	49.6
A3KG36	Glucose-6-	18.557	13	4	12	4	396	45.2
D3Z132	THO comp	18.553	18	5	8	5	337	37.1
Q9DBC7	cAMP-dep	18.518	15	5	15	5	381	43.2
P52431	DNA polyn	18.496	5	7	26	6	1105	123.7
P62071	Ras-relate	18.478	30	6	45	5	204	23.4
Q6ZWZ4	60S riboso	18.468	30	4	78	4	105	12.2

Q07802	Transcript	18.419	8	4	48	4	591	64.4
Q99LG7	Zinc finger	18.419	12	5	30	1	467	54.8
Q8BGA5	KRR1 sma	18.409	16	6	53	6	380	43.5
Q922B6	E3 ubiquit	18.396	10	5	14	5	594	66.4
F6RJV6	LanC-like p	18.392	12	4	22	4	441	49.7
Q99L43	Phosphatic	18.391	12	3	35	3	444	51.3
Q61080	Forkhead l	18.373	14	3	28	3	378	39.9
Q5SV54	Ortholog c	18.34	7	4	6	4	1104	121.7
D3YXJ0	Diacylglyce	18.314	7	5	35	4	1211	134
Q91W94	Zfp71-rs1	18.304	14	5	40	1	390	44.9
Q91VY9	Zinc finger	18.295	11	5	24	5	476	53.4
Q810B6	Rabankyrin	18.286	7	6	17	6	1169	128.6
P14733	Lamin-B1	18.276	10	7	44	5	588	66.7
Q9D0L8	mRNA cap	18.263	11	4	42	4	465	53.3
O88286	Protein W	18.258	3	3	16	3	1684	184.2
Q7M6Y3	Phosphatic	18.235	8	5	62	5	660	71.5
P61027	Ras-relate	18.214	29	4	54	2	200	22.5
Q9D0M5	Dynein ligl	18.204	38	3	116	1	89	10.3
P97350	Plakophilir	18.19	7	5	22	5	728	80.8
Q9D880	Mitochonc	18.185	12	3	17	3	353	39.8
P47226	Testin OS=	18.162	13	5	31	5	423	48
Q9CX99	GRB2-rela	18.158	18	2	9	2	217	25.3
A0A0N4SV	Cellular nu	18.147	32	5	37	5	171	18.8
O35608	Angiopoiet	18.126	10	5	17	5	496	56.5
Q8CJG1	Protein arg	18.117	6	5	33	2	857	97.2
Q9QYJ0	DnaJ homo	18.117	15	6	77	6	412	45.7
P62700	Protein yip	18.115	47	5	29	5	121	13.8
A0A0B4J1	Low affinit	18.109	22	5	22	5	249	28.4
B2RVL6	Zinc finger	18.098	18	4	42	4	241	26.9
Q8CG46	Structural	18.092	7	6	24	6	1101	128.7
Q8R0F5	RNA-bindi	18.09	16	5	24	5	326	37.5
Q3U6N9	UPF0488 p	18.078	29	4	51	4	222	24.5
P58391	Small conc	18.071	10	5	22	5	732	81.3
O08900	Zinc finger	18.069	9	4	47	2	507	57.9
Q9Z1Z0	General ve	18.061	7	6	28	6	959	106.9
Q9Z1D1	Eukaryotic	18.054	13	4	25	4	320	35.6
Q6NXK5	RNA/RNP	18.042	17	4	31	4	321	37.8
A2A5R2	Brefeldin A	18.04	4	6	28	4	1792	202.1
Q99MD8	Myoneurin	18.019	9	5	13	4	610	68.5
P50172	Corticoste	17.989	16	4	75	4	292	32.3
Q925E7	Serine/thr	17.97	9	3	33	1	453	51.9
D3Z158	Glutaminy	17.932	7	3	25	3	751	85
Q8BH61	Coagulatic	17.91	8	6	42	5	732	83.2
A2AGQ2	MAP kinas	17.899	5	6	24	6	1554	172.5
Q80WR5	UPF0688 p	17.885	25	3	10	3	230	24.8

Q8K214	Polycomb	17.884	10	4	15	4	706	78.6
A0A0N4SU	Pleckstrin	17.878	6	3	11	3	956	108.5
Q9CQN1	Heat shock	17.863	8	5	16	4	706	80.2
E9QAH1	Golgi auto	17.821	2	5	13	5	3197	365.1
Q8BH43	Wiskott-A	17.804	10	4	13	4	497	54
Q3TSG4	RNA deme	17.792	14	4	18	4	395	44.4
E9QLL2	Dynamin G	17.755	5	4	29	1	863	97.2
P70290	55 kDa ery	17.743	12	4	28	4	466	52.2
D3YYK8	Microtubu	17.731	17	3	36	3	260	29.4
Q6P5H2	Nestin OS=	17.69	4	6	28	6	1864	207
A0A2R8VH	Predicted	17.679	8	6	67	1	535	57.9
Q5DTZ0	Protein NY	17.677	5	6	11	6	1840	202.9
Q8BZZ3	NEDD4-like	17.648	9	6	21	2	918	104.6
A2ALK8	Tyrosine-p	17.627	5	4	40	4	913	103.8
P32921	Tryptopha	17.621	13	5	37	5	481	54.3
Q8CDG5	CREB3 reg	17.609	8	5	22	5	640	72.6
A0A1L1ST	CLIP-assoc	17.568	10	3	15	1	600	66.3
P70441	Na(+)/H(+	17.559	17	4	24	4	355	38.6
Q8BTI9	Phosphatid	17.556	6	4	43	4	1064	121.6
Q62187	Transcript	17.545	7	5	41	5	859	97.7
Q3THW5	Histone H2	17.491	31	4	256	2	128	13.5
B1AQF4	Dual speci	17.484	29	4	26	4	210	23.2
Q922Q4	Pyrroline-5	17.475	19	4	23	4	320	33.6
Q9JIK9	28S riboso	17.462	26	5	26	5	218	25.8
T1NXB5	V-set and	17.451	16	4	29	4	319	35.9
H9KV15	Protein SC	17.412	2	5	68	5	2343	253.9
A0A1L1ST	Interleukin	17.409	7	6	41	6	908	97.4
Q8VC34	Putative R	17.361	8	4	26	4	614	68.5
Q8VEH3	ADP-ribosy	17.342	26	5	41	2	186	21.4
Q7TMY8	E3 ubiquit	17.337	1	5	33	5	4377	482.3
E9PVX6	Proliferati	17.314	2	5	23	5	3177	350.7
G8JL40	Lysine-spe	17.3	6	3	19	3	683	75.5
Q05CL8	La-related	17.285	9	4	17	4	570	64.8
G5E905	Zinc finger	17.265	10	5	27	5	615	71.5
Q61635	GTP-bindin	17.259	14	5	21	4	420	46.8
G5E881	Phosphatid	17.214	13	3	23	3	337	37.9
P42669	Transcript	17.195	19	5	69	5	321	34.9
Q6NZI9	Zinc finger	17.172	10	4	27	2	655	73.8
O88665	Bromodom	17.164	10	5	15	5	651	74
A0A1B0GF	MHC class	17.136	7	4	14	4	910	98
Q8CDJ8	Stonin-1 O	17.13	9	5	20	5	730	81.7
Q8K1X4	Nck-assoc	17.112	6	6	25	5	1134	128.8
P49586	Choline-ph	17.1	14	4	19	4	367	41.6
Q9WVA4	Transgelin	17.096	25	4	38	4	199	22.4
O35704	Serine pal	17.049	12	3	64	3	473	52.5

P48999	Polyunsatu	17.03	9	5	21	5	674	77.9
P51660	Peroxisom	17.016	7	4	22	4	735	79.4
D3YWF6	Ubiquitiny	17.015	17	4	43	4	241	28
B1AW40	Rho GTPas	17.015	9	3	24	3	477	55
Q6P9J5	KN motif a	17.01	8	4	10	4	1016	110.3
Q9Z1Q2	Phosphatid	17.004	9	5	41	5	558	63
Q6NVF4	DNA helica	17.004	6	5	27	5	1074	121.4
E9Q3R9	Zinc finger	16.996	6	4	16	4	1066	118.1
Q9Z0Y1	Dynactin s	16.986	26	4	29	4	186	21
Q3TML0	Protein dis	16.967	11	4	36	4	445	48.7
P62315	Small nucl	16.964	20	3	51	3	119	13.3
Q01730	Ras suppre	16.951	18	5	46	5	277	31.5
Q62311	Transcripti	16.945	6	3	6	3	678	72.6
E9PUR6	Glucocorti	16.943	7	4	19	4	792	87.1
B1AXS8	Zinc finger	16.942	4	4	38	4	1356	151.3
P08032	Spectrin al	16.908	2	5	57	4	2415	279.7
A0A0N4SV	T-complex	16.906	14	6	36	6	502	55
Q8R205	Zinc finger	16.896	12	3	7	3	435	46.1
Q91VV4	DENN dom	16.87	10	3	37	3	469	53.2
Q9D0F4	NF-kappa-	16.855	13	4	18	4	415	47.2
Q07230	Zinc finger	16.823	10	5	28	3	614	68.7
P14483	H-2 class I	16.818	16	3	32	3	265	30.1
Q62172	RalA-bindi	16.812	7	4	20	4	648	75
F2Z4B7	Ras associ	16.801	6	3	25	3	645	72.7
Q8R4U7	Leucine zip	16.778	6	6	16	6	1068	119.2
A0A1D5RN	Dynein ligh	16.775	14	5	25	4	470	51.5
Q9D0I9	Arginine--t	16.753	7	3	11	3	660	75.6
Q8VE37	Regulator	16.746	13	4	16	4	421	44.9
P97484	Leukocyte	16.686	5	4	35	4	841	93
Q9D1H9	Microfibril	16.663	17	2	16	2	257	28.9
P49717	DNA replic	16.631	6	5	30	5	862	96.7
Q6PFA2	Clathrin lig	16.593	21	4	24	4	218	23.6
Q9D7N3	28S riboso	16.582	11	4	13	4	390	44.9
P42128	Forkhead b	16.579	7	6	40	4	719	74.9
Q9DBH0	NEDD4-like	16.577	9	7	20	3	870	98.7
Q60739	BAG famil	16.574	15	4	14	4	355	39.7
Q4VBE4	Pikachurin	16.572	7	4	17	4	1017	110.7
A2BFG5	Predicted g	16.572	20	4	44	1	554	64.4
P26443	Glutamate	16.559	10	4	23	4	558	61.3
O09106	Histone de	16.528	9	4	62	2	482	55
P03975	IgE-binding	16.526	11	5	34	5	557	62.7
Q3TGM7	HBS1-like	16.525	8	3	19	3	679	74.8
Q6P069	Sorcin OS=	16.479	21	3	26	3	198	21.6
Q6NZD2	Sorting ne	16.478	7	4	20	4	521	58.8
Q3UKJ7	WD40 repe	16.469	14	5	15	5	513	57.5

Q02111	Protein kir	16.46	6	4	43	3	707	81.5
G3X956	FACT com	16.399	5	5	20	5	1047	119.8
A0A3B2W	Mitogen-a	16.399	17	4	19	3	307	35.3
Q921K8	TRPM8 cha	16.374	5	4	38	4	919	101.5
Q8VE19	GATOR co	16.357	6	5	11	5	875	98.3
E9PZM7	SR-relatec	16.355	3	4	34	4	1456	162
P62869	Elongin-B	16.347	42	4	50	4	118	13.2
Q3UHX2	28 kDa hea	16.345	18	4	10	4	181	20.6
Q80TH2	Erbin OS=I	16.341	4	5	25	5	1402	157.2
Q3UJK4	GTP-bindir	16.335	9	5	24	5	602	65.7
P27661	Histone H2	16.331	34	4	212	1	143	15.1
K4DI62	Transcript	16.331	3	3	15	3	780	81.6
O55142	60S riboso	16.325	33	5	123	5	110	12.5
O35945	Aldehyde (	16.325	12	4	55	1	501	54.6
P36916	Guanine n	16.318	7	4	31	4	607	68.7
P39654	Polyunsatu	16.31	9	4	26	4	663	75.4
P58468	Protein FA	16.297	19	4	29	4	219	24.8
Q3V384	AFG1-like	16.281	11	3	21	3	480	54.3
Q9D3A8	Carboxyl-te	16.267	14	5	20	5	503	55.8
A0A0N4SV	Electron tr	16.26	21	3	16	3	221	24.1
A2AQA7	RNA helica	16.252	5	5	10	5	1400	161.8
Q8K2M0	39S riboso	16.244	14	5	47	5	380	45
Q5DTM8	E3 ubiquit	16.212	5	4	15	4	973	113.5
Q9CZT6	Protein CM	16.208	13	3	35	3	276	31.6
P97468	Chemokine	16.192	15	3	18	3	371	41.8
E9Q5F9	Histone-lys	16.18	3	6	22	6	2537	285.5
P56546	C-termina	16.141	10	5	34	4	445	48.9
Q60793	Krueppel-l	16.094	6	2	56	2	483	51.8
Q9R0M4	Podocalyxi	16.088	9	3	37	3	503	53.4
Q9DB42	Zinc finger	16.086	34	4	55	4	134	15.1
Q3UN02	Lysocardio	16.057	11	4	42	4	376	44.4
Q9DB94	WD repeat	16.053	18	4	11	4	358	38.6
Q80VQ0	Aldehyde (	16.048	13	5	14	5	468	52.3
A0A0N4SU	Selenocyst	16.043	9	5	33	5	534	58
Q6PDY2	2-aminoet	16.033	15	3	34	3	256	28.4
Q80ZJ1	Ras-relate	16.009	20	4	27	2	183	20.6
Q8VHH5	Arf-GAP w	15.995	6	5	11	1	910	97.9
Q8R3F5	Malonyl-C	15.992	13	3	8	3	381	41.9
Q99JR8	SWI/SNF-	15.978	9	3	27	3	531	59
Q99LC3	NADH deh	15.973	13	4	25	4	355	40.6
Q8K0E8	Fibrinoger	15.967	8	3	24	3	481	54.7
Q9DB34	Charged m	15.939	18	6	109	6	222	25.1
Q2NL51	Glycogen s	15.897	14	4	24	3	490	51.6
Q9DC23	DnaJ homo	15.89	7	4	31	4	793	90.5
Q99LB0	Deoxynucl	15.883	12	3	20	3	328	36.8

Q8R2U2	Nucleolus	15.881	7	4	62	4	564	63.4
F7BJK1	Protocadh	15.863	5	3	23	3	1077	116.4
E0CX30	Zinc finger	15.856	6	4	13	4	1152	128.9
Q9CQQ7	ATP syntha	15.817	16	4	45	4	256	28.9
P18406	CCN family	15.794	12	4	65	4	379	41.7
E9PZJ8	Activating	15.789	3	5	16	5	2198	250.4
Q921I6	SH3 doma	15.76	4	3	15	3	962	107.5
Q8K2F8	Protein LS	15.751	9	4	11	4	462	50.5
A0A140LH	39S riboso	15.749	35	4	29	4	113	12.9
G5E8U9	BCL2/aden	15.717	11	4	25	4	348	39.8
P59110	Sentrin-sp	15.706	10	4	21	4	640	72.5
Q8CFG0	Extracellul	15.701	7	5	13	5	875	100.4
D3YZJ1	Sequestos	15.7	17	3	16	3	382	41.7
P10711	Transcript	15.695	19	5	14	5	301	33.9
Q3UU56	Predicted	15.691	6	4	6	4	907	100.2
P50171	(3R)-3-hyd	15.691	19	3	5	3	259	26.6
Q9Z0E6	Guanylate	15.685	8	3	14	3	589	66.7
O89051	Integral m	15.681	18	4	34	4	266	30.2
F8WI26	Terminal r	15.666	8	5	37	5	637	69.3
Q8BGN9	Uncharact	15.66	35	3	23	3	141	15.4
P35564	Calnexin C	15.656	10	5	21	5	591	67.2
Q5NBU8	XIAP-asso	15.654	16	3	17	3	273	31.1
P01869	Ig gamma	15.649	12	3	68	3	393	43.4
Q9D1N9	39S riboso	15.623	21	3	21	3	209	23.4
F8WI37	[Histone H	15.616	1	4	17	4	4904	539.9
P27784	C-C motif	15.611	28	2	28	2	116	13
Q9CQA1	Trafficking	15.61	26	5	46	5	188	20.8
Q9R0L7	A-kinase a	15.598	7	4	38	4	642	71.4
Q5SW28	Phosphoin	15.593	6	4	24	4	871	96.9
Q80X98	RNA helica	15.574	5	4	13	4	1228	140.5
Q9QWT9	Kinesin-lik	15.566	10	4	18	1	674	74.1
A0A0G2JD	Arf-GAP w	15.558	12	5	12	2	578	62.3
Q6R891	Neurabin-	15.53	6	4	5	4	817	89.5
Q8BJY1	26S protea	15.527	9	4	20	4	504	55.9
Q8BX32	Structure-	15.519	16	3	42	3	270	30.6
Q8BTV1	Tumor sup	15.505	14	5	39	5	347	39.5
Q9QXD8	LIM doma	15.491	6	3	9	3	668	71.4
Q6PHQ8	N-alpha-a	15.488	8	5	20	5	725	83.3
Q3UI84	Replicatio	15.476	14	4	33	4	370	40.9
A6H5X4	PHD finger	15.473	12	4	47	4	337	37.7
A0A1X7SB	RING-type	15.451	10	3	10	3	488	54.1
S4R211	Serine-rich	15.443	23	4	8	4	261	30.3
Q80U70	Polycomb	15.439	8	5	23	5	741	83
Q62448	Eukaryotic	15.403	6	5	16	5	906	102
Q9WV34	MAGUK p5	15.37	10	4	13	4	552	61.5

B1AUE5	Peroxisom	15.361	11	3	14	3	324	37.1
Q9CQM8	60S riboso	15.361	27	5	44	5	160	18.6
A2AWA9	Rab GTPas	15.348	5	4	19	4	1064	120.7
Q9R226	KH domair	15.341	10	4	64	4	346	38.8
A0A286YC	Methyl-Cp	15.325	9	3	32	3	467	50.3
Q8CAS9	Protein mo	15.309	5	4	10	4	866	96.6
Q9D0T1	NHP2-like	15.302	29	4	31	4	128	14.2
F6XC25	Coiled-coil	15.275	6	4	7	4	770	85
Q6NV83	U2 snRNP-	15.254	5	4	36	4	1029	118.2
Q6PAL8	DENN dom	15.186	5	5	18	3	1287	146.6
A0A1L1ST	Nesprin-1	15.176	1	6	13	5	8799	1009.3
Q8CJ53	Cdc42-inte	15.161	10	5	20	5	603	68.4
Q9JK53	Prolargin (	15.151	11	5	25	5	378	43.3
Q6PAM1	Alpha-taxi	15.144	11	6	31	6	554	62.3
A0A494BA	Cathepsin	15.137	13	3	28	3	342	38.8
Q8BFY9	Transporti	15.115	5	4	16	4	898	102.3
Q9WTX5	S-phase ki	15.114	26	3	78	3	163	18.7
Q9WUD0	Unspecific	15.105	8	3	23	3	491	55.8
O54941	SWI/SNF-	15.102	10	3	23	3	411	46.6
Q91WK2	Eukaryotic	15.081	15	4	31	4	352	39.8
E9PWR4	Disco-inte	15.07	3	4	31	1	1557	171
Q3U288	Zinc finger	15.068	5	3	21	2	666	75
P26645	Myristoyla	15.06	13	3	46	3	309	29.6
P43135	COUP tran	15.041	14	5	54	5	414	45.5
P97371	Proteasom	15.004	15	2	9	2	249	28.7
Q14B46	Rhotekin-2	14.978	7	4	21	4	604	66.9
A0A0A6YV	Calcium lo	14.966	16	3	44	3	200	22.4
O55013	Trafficking	14.949	21	4	44	4	180	20.3
Q8CHE4	PH domair	14.947	4	4	9	4	1687	182.3
P55258	Ras-relate	14.941	20	4	51	2	207	23.7
Q05117	Tartrate-re	14.937	15	3	7	3	327	36.8
H3BL92	Ecotropic v	14.921	6	4	14	4	797	91.7
P43680	Transcripti	14.917	14	4	39	3	377	40.9
Q99JB2	Stomatin-	14.915	10	4	19	4	353	38.4
Q6NVF9	Cleavage a	14.899	8	3	20	3	551	59.1
Q8VDI1	Epithelial-	14.896	12	4	33	4	314	36.1
B9EJ86	Oxysterol-	14.89	5	3	25	3	889	101.2
Q6PHN9	Ras-relate	14.851	22	4	35	2	201	23
Q9CX97	WD repeat	14.843	12	3	10	3	388	42.6
Q99JP0	Mitogen-a	14.809	6	4	19	4	894	101.1
E9PUQ8	Diacylglyce	14.796	4	4	15	3	1220	135.1
Q8CHI8	E1A-bindir	14.792	2	5	30	5	3072	337
A0A0B4J1	SNW dom	14.782	7	3	14	3	536	61.4
P50285	Dimethyla	14.782	7	4	57	4	532	59.9
P56873	Protein ZN	14.78	23	2	41	2	199	21.3

Q61466	SWI/SNF-	14.758	13	4	15	4	515	58.2
P26450	Phosphatic	14.717	8	4	21	4	724	83.5
Q61818	Retinoic ac	14.67	3	4	14	4	1889	201.4
Q9QZQ8	Core histo	14.667	16	4	26	3	372	39.7
Q9DCT5	Stromal ce	14.651	26	3	17	3	211	23.1
Q6NZB0	DnaJ home	14.628	23	5	30	5	253	29.8
A0A0A0M	Glycosyltra	14.614	25	4	33	4	191	22.4
A0A5K1VV	Protein un	14.603	5	5	16	5	1088	119.1
Q9D1M0	Protein SE	14.601	18	4	13	4	322	35.5
O08917	Flotillin-1	14.576	11	4	10	4	428	47.5
Q8BJA3	Homeobox	14.575	14	4	10	4	419	47.1
G3X924	Plasmalen	14.571	6	3	7	3	438	49.9
Q9D0F6	Replicatio	14.566	17	4	14	4	339	38.1
Q8BG07	5'-3' exonu	14.557	8	2	4	2	503	56.1
A0A571BE	InaD-like p	14.554	4	6	21	5	1858	200.8
P46460	Vesicle-fu	14.545	6	5	35	5	744	82.6
Q91VE0	Long-chair	14.542	9	3	10	3	643	72.3
P53995	Anaphase-	14.537	2	4	15	4	1944	215.9
A0A087W	Snf2-relate	14.482	2	4	11	4	3114	333.4
Q60591	Nuclear fa	14.466	4	2	7	2	927	100
A0A087W	Selenoprot	14.465	35	3	39	3	123	13.5
Q80XC6	Nuclear ex	14.464	4	4	23	4	1172	133.4
Q3UHE1	Membrane	14.416	5	3	17	3	974	106.4
Q8BK72	28S riboso	14.398	9	3	9	3	415	47.7
Q922U1	U4/U6 sm	14.389	6	4	7	4	683	77.4
P27005	Protein S1	14.377	49	2	26	2	89	10.3
Q6PR54	Telomere-	14.343	2	4	12	4	2419	266.1
Q9ESL4	Mitogen-a	14.338	5	4	34	4	802	91.7
Q9DAP7	Histone ch	14.302	18	3	75	2	202	22.5
Q8BSQ9	Protein po	14.296	3	5	16	5	1634	187.1
P97363	Serine pal	14.286	7	3	24	3	560	62.9
Q9DAW6	U4/U6 sm	14.285	10	4	11	4	521	58.3
Q9QXK3	Coatomer	14.28	7	4	12	4	871	97.6
P98203	Armadillo	14.278	6	4	34	4	962	105
G3X973	Stabilin-1	14.259	3	5	15	5	2571	276.3
A0A338P6	Arf-GAP w	14.243	8	5	7	5	805	91.1
Q9QYS9	Protein qu	14.21	12	4	39	4	341	37.6
P40124	Adenylyl cy	14.198	8	4	19	4	474	51.5
P08074	Carbonyl re	14.187	15	4	48	4	244	25.9
Q80ZW2	Protein TH	14.179	31	5	33	5	207	23.8
Q99M04	Lipoyl synt	14.164	14	4	28	4	373	41.9
E9QMN5	Transcripti	14.135	10	5	11	5	630	67.4
Q99JH1	Ribonuclea	14.134	25	3	18	3	163	17.7
A0A1Y7VL	Tetratricop	14.115	6	5	19	5	860	96
F8VQK5	SAM and S	14.096	7	4	13	4	1230	135.5

Q64707	U2 small r	14.077	10	3	32	3	428	51.3
P28028	Serine/thr	14.074	5	4	31	3	751	82.5
A0A0X1KG	E3 ubiquit	14.061	4	3	10	3	961	109.3
E9Q5K9	YTH doma	14.055	6	4	24	4	736	85.6
O35972	39S riboso	14.053	25	2	20	2	146	17.1
Q8VDF3	Death-ass	14.037	11	3	24	3	370	42.8
A2ADH1	Dolichyl-di	14.032	8	4	48	4	368	41.6
Q04841	DNA-3-me	14.012	13	4	5	4	333	36.4
Q61655	ATP-deper	13.986	9	3	15	3	478	53.9
A0A1W2P	Protein str	13.96	3	4	9	3	1230	136.7
Q3U0V1	Far upstre	13.959	8	4	21	3	748	76.7
O35250	Exocyst co	13.95	6	4	11	4	697	79.9
F6RJE8	Phosphatic	13.946	21	5	23	5	214	24.7
Q69ZR9	Protein TA	13.939	3	4	10	4	1610	181.3
P70335	Rho-assoc	13.936	3	5	18	4	1354	158.1
Q99LJ0	CTTNBP2 I	13.931	7	4	18	4	638	69.8
Q9Z2C5	Myotubula	13.93	9	4	13	4	603	69.5
Q7TSI3	Serine/thr	13.876	8	5	9	5	856	94.5
Q8JZN5	Complex I	13.876	8	3	10	3	625	68.7
A0A286YC	Sorbin and	13.866	4	4	31	4	1290	143
Q9D289	Trafficking	13.848	32	5	20	5	158	17.9
P61620	Protein tra	13.846	8	4	56	4	476	52.2
P83940	Elongin-C	13.834	40	4	17	4	112	12.5
D3Z3Q3	Smootheli	13.828	6	4	11	4	946	103
Q99N91	39S riboso	13.828	25	3	46	3	92	10.5
P14685	26S protea	13.819	8	4	20	4	530	60.7
Q02105	Compleme	13.81	11	2	69	2	246	26
G3UYV7	40S riboso	13.805	57	3	61	3	56	6.3
Q8BHS6	Armadillo	13.801	12	4	16	4	379	42.6
A0A0R4J1	Bromodon	13.779	8	3	25	3	597	66.9
Q60960	Importin s	13.776	11	4	16	2	538	60.1
Q9D1R9	60S riboso	13.773	23	4	154	4	117	13.3
D3YW97	Ubiquitin-	13.747	28	2	35	2	82	8.8
Q9JIX8	Apoptotic	13.747	3	3	28	3	1338	150.6
H7BX64	Sarcolemr	13.736	7	5	16	5	828	95
Q8CIH5	1-phospha	13.732	4	4	19	4	1265	147.5
O08538	Angiopoie	13.728	9	4	14	4	498	57.5
Q99KY4	Cyclin-G-a	13.701	3	4	8	4	1305	143.6
Q9ESU6	Bromodon	13.643	2	3	9	1	1400	155.8
P04627	Serine/thr	13.64	7	4	24	3	604	67.5
E9PYI8	Ubiquitin	13.634	8	3	12	3	458	52.3
Q8BH73	Glutaminy	13.631	10	4	21	4	383	42.7
P32233	Developm	13.614	13	3	12	3	367	40.5
E9Q0U7	Heat shocl	13.606	7	5	7	5	817	91.6
Q99KI0	Aconitate	13.598	7	3	17	3	780	85.4

Q00417	Transcripti	13.57	9	4	63	3	419	45.4
Q9D1P2	Histone ac	13.559	11	3	26	2	458	52.5
E9Q166	ATPase fa	13.556	3	4	15	3	1460	164.6
Q8BU31	Ras-relate	13.545	20	4	21	2	183	20.7
A0A7N9VF	AHNAK nu	13.544	9	3	13	3	3501	368.2
P21279	Guanine n	13.538	12	5	32	4	359	42.1
E9QP49	EH domair	13.536	3	4	9	4	1718	184.7
P50516	V-type pro	13.516	7	4	11	4	617	68.3
Q9DBZ5	Eukaryotic	13.515	18	3	16	3	218	25.1
P08103	Tyrosine-p	13.497	7	4	49	1	524	59.1
D3YX27	Serine pro	13.497	9	3	5	3	426	45.8
Q0VD84	Latent-tra	13.495	3	4	14	4	1786	192.7
P26369	Splicing fa	13.49	10	3	32	3	475	53.5
Q6PI63	DNA-direc	13.479	25	3	36	3	117	13.3
P37804	Transgelin	13.465	20	3	16	3	201	22.6
Q9D0M3	Cytochrom	13.453	11	2	27	2	325	35.3
Q9ERS5	Pleckstrin	13.449	13	3	19	3	425	47.4
P25799	Nuclear fa	13.372	5	4	9	4	971	105.5
H7BX38	C-X-C moti	13.371	24	3	126	3	119	13.6
P17433	Transcripti	13.361	8	3	28	3	272	31.3
A2A8W8	MAP kinas	13.343	10	3	13	3	415	46.5
P41245	Matrix me	13.34	7	3	8	3	730	80.5
Q9QYY0	GRB2-asso	13.338	6	2	13	2	695	76.8
Q80TL7	Protein M(	13.338	2	3	24	3	1715	189
Q9EPE9	Endoplasm	13.326	5	5	21	5	1200	132.3
J3QNU1	Arginyl-tR	13.311	9	3	19	3	509	58.2
Q9DC71	28S riboso	13.281	13	3	43	3	258	29.4
A0A0J9YU	C-termina	13.244	8	2	9	1	337	37
Q3V0K9	Plastin-1 (	13.242	3	3	33	1	630	70.4
P63094	Guanine n	13.21	8	3	36	2	394	45.6
P05480	Neuronal p	13.197	9	4	39	2	535	59.9
Q9ES74	Serine/thr	13.196	18	4	15	3	302	34.5
Q923B1	Lariat deb	13.178	5	3	16	3	550	62.3
A2ABV5	Mediator c	13.177	3	3	11	3	1459	160.9
A0A0R4J0	cAMP-resp	13.161	23	2	40	2	123	14
Q3UMR5	Calcium ur	13.159	10	3	24	3	350	39.7
Q91W39	Nuclear re	13.153	11	4	6	4	579	65.3
A0A140LH	Patatin-lik	13.15	21	3	10	1	224	24.4
O35929	GTP-bindir	13.126	12	4	20	3	297	32.9
Q8CIP4	MAP/micr	13.089	5	3	38	2	752	82.6
Q3UL36	Arginine a	13.085	14	5	94	5	271	32.9
Q9D777	Tumor nec	13.068	19	3	15	3	241	26.9
D3Z6I4	Quinone o	13.058	12	3	15	3	333	37
G5E902	Phosphate	13.05	11	4	45	4	358	39.7
E9PV45	Ubiquitiny	13.039	2	4	9	4	2618	293.9

J3QMG3	Voltage-de	13.032	12	3	16	2	284	30.8
Q5ND52	rRNA met	13.02	9	3	14	3	418	46.8
A0A0R4J1	Actin filam	13.017	5	3	19	3	769	85.7
Q3LR78	CtBP-inter	13.017	4	5	23	4	1197	134.1
P06798	Homeobox	13.012	14	3	27	3	285	30.4
Q0P557	Mitochond	12.993	8	4	14	4	537	60.4
Q8C0V9	FERM dom	12.988	6	3	26	3	622	71.6
E9PVA2	Ribosoma	12.975	7	3	10	3	503	56.5
Q9CQR2	40S riboso	12.973	40	3	40	3	83	9.1
Q8K1B8	Fermitin fa	12.962	6	3	17	3	665	75.6
Q9CQE6	Histone ch	12.958	17	3	62	2	204	22.9
Q924L1	LETM1 dor	12.95	9	3	35	3	360	41.7
Q6GV12	3-ketodihy	12.943	11	3	5	3	332	35.9
Q80YW0	Cytohesin-	12.917	13	4	16	3	393	45.3
Q9ER00	Syntaxin-1	12.913	13	3	14	3	274	31.2
A0A140LIU	Cytochrom	12.912	79	3	46	3	43	4.9
Q9R0M3	Sushi-repe	12.912	10	4	18	4	464	51.5
P46425	Glutathior	12.906	17	2	10	2	210	23.5
Q8BJZ4	28S riboso	12.904	14	3	16	3	320	36
Q8K4B0	Metastasis	12.891	6	3	37	2	715	80.7
Q6IRU5	Clathrin lig	12.886	18	4	33	4	229	25.2
Q9JMJ2	F-box/WD	12.867	11	3	11	3	410	46.1
A0A0A0M(	Vesicle-fus	12.863	7	5	12	5	892	97.1
O35738	Krueppel-l	12.859	8	4	37	1	402	44.1
Q9CRB9	MICOS cor	12.859	18	4	10	4	227	26.3
Q8BH95	Enoyl-CoA	12.852	11	3	33	3	290	31.5
E9QMU1	Kinesin-lik	12.841	3	4	16	4	1347	151.5
A0A0R4J0	ADP-ribosy	12.82	7	4	20	4	524	57.5
Q9DBS4	Acyl-coenz	12.815	6	3	25	3	632	70.7
Q8CJ78	Zinc finger	12.814	6	5	23	4	1038	109
P18872	Guanine n	12.813	9	3	42	1	354	40.1
P51150	Ras-relate	12.793	17	3	14	3	207	23.5
Q9Z1Y4	Thyroid re	12.769	11	3	19	3	480	50.9
P31725	Protein S1	12.759	21	2	44	2	113	13
Q8BUP8	Protein FA	12.755	8	4	32	4	424	46.2
Q4VBD2	Transmem	12.754	7	3	7	3	564	63.9
Q06138	Calcium-bi	12.752	13	4	42	4	341	39.8
O35492	Dual speci	12.728	6	4	19	4	638	73.8
P11087	Collagen a	12.727	2	2	18	2	1453	137.9
Q8BGT7	Survival of	12.706	18	3	29	3	238	26.7
Q9D174	SS18-like p	12.684	27	2	22	2	77	8.9
Q9ET54	Palladin O	12.68	3	4	21	4	1408	152
Q9CU62	Structural	12.676	3	5	33	5	1233	143.1
Q8BVV6	Eukaryotic	12.672	7	2	14	2	375	42.6
C0HKD9	Microfibril	12.665	10	2	5	2	439	51.9

Q9CX83	Armadillo	12.657	8	4	26	4	456	50.6
Q8BGZ4	Cell divisio	12.656	8	4	19	4	597	68.5
Q8R4E9	DNA replic	12.647	9	4	10	4	557	61.5
Q99K48	Non-POU (	12.641	9	4	46	3	473	54.5
E9PX30	Mitogen-a	12.619	6	3	6	3	847	94.9
F8WJK8	Hsc70-inte	12.611	8	3	29	3	362	40.5
Q64521	Glycerol-3	12.603	6	4	4	4	727	80.9
Q6GQR8	Zinc finger	12.591	5	3	26	1	522	59.8
Q62048	Astrocytic	12.589	21	3	17	3	130	15
P59326	YTH doma	12.582	4	2	21	1	559	60.8
Q922D8	C-1-tetrah	12.573	4	4	18	4	935	101.1
P59764	Dedicator	12.572	2	4	17	4	1978	226.4
Q922H2	[Pyruvate (	12.57	11	4	35	4	415	47.9
P84444	C-C motif (	12.566	26	3	21	3	133	14.5
P15533	Tripartite	12.547	7	3	8	3	496	57.3
Q02013	Aquaporin	12.543	11	2	25	2	269	28.8
O70161	Phosphatic	12.523	6	3	5	3	661	72.4
P35279	Ras-relate	12.518	15	3	54	2	208	23.6
Q8BMP6	Golgi resic	12.516	8	3	15	3	525	60.1
P56857	Claudin-18	12.516	17	3	34	3	264	28.1
A0A384DV	High mobi	12.507	32	4	38	1	95	10.5
B1AVF5	RING-type	12.504	7	4	15	4	715	81.1
Q3UP87	Neutrophi	12.503	18	3	41	3	265	28.6
E9Q4R1	Protein FA	12.501	9	2	16	2	370	40.2
O35639	Annexin A	12.482	14	4	22	3	323	36.4
A2AF67	Dedicator	12.48	3	5	32	4	1902	218.1
G3X963	ATPase fa	12.478	4	3	6	2	1364	155.2
P45591	Cofilin-2 C	12.475	23	4	17	3	166	18.7
Q9JJL8	Serine--tR	12.464	8	2	14	2	518	58.3
Q62419	Endophilin	12.454	10	4	21	3	368	41.5
Q9CR80	Protein FA	12.442	28	3	19	3	112	13.2
Q9CPR5	39S riboso	12.432	13	3	16	3	295	33.5
Q925F2	Endothelia	12.413	8	2	18	2	394	41.8
Q9WU22	Tyrosine-p	12.408	6	4	15	4	926	105.8
Q91VS8	FERM, AR	12.408	5	4	19	4	1065	121.2
Q3TB82	Pleckstrin	12.397	18	4	16	3	279	31.1
B1AVZ0	Uracil pho	12.375	12	2	24	2	310	34.3
Q6PGE4	Zinc finger	12.374	5	4	25	4	1016	110.7
Q6PFQ7	Ras GTPas	12.369	7	4	12	4	802	90
Q3TIR1	Trafficking	12.358	12	5	27	5	417	46.4
Q8BPB5	EGF-conta	12.328	6	3	6	3	493	54.9
Q9QZ06	Toll-intera	12.31	13	3	18	3	274	30.3
Q8C4B4	Protein un	12.308	15	2	18	2	251	28.3
O08530	Sphingosir	12.27	6	2	23	2	382	42.6
Q9DAW9	Calponin-3	12.261	12	3	17	2	330	36.4

Q9WVS8	Mitogen-a	12.258	7	4	11	4	806	87.7
Q8VC31	Coiled-coil	12.217	9	4	20	4	543	61.4
Q99LM9	Transcripti	12.2	18	3	7	3	335	37.4
E9Q748	Antileukop	12.197	30	4	27	4	107	11.9
P99028	Cytochrom	12.194	29	2	12	2	89	10.4
Q9JMG7	Hepatoma	12.168	21	3	16	2	202	22.4
Q91VH2	Sorting ne	12.166	9	3	10	3	595	66.5
D3Z3S1	Prolactin r	12.161	16	4	14	4	350	37.9
Q9DCT8	Cysteine-ri	12.161	26	2	31	2	208	22.7
P97821	Dipeptidyl	12.135	5	2	57	2	462	52.3
Q6PDZ2	TATA box-	12.132	5	4	5	4	836	92
Q8VCW8	Medium-c	12.071	6	3	16	3	615	67.9
Q61084	Mitogen-a	12.063	7	4	15	3	626	70.7
Q5SSZ5	Tensin-3 C	12.061	4	4	31	4	1440	155.5
Q99K30	Epidermal	12.056	5	4	25	4	729	82.2
P57722	Poly(rC)-bi	12.041	10	3	60	1	371	39.3
F8WJG3	Transform	12.035	20	3	60	3	188	21.9
Q3U7R1	Extended s	12.022	5	4	13	4	1092	121.5
Q8R0P4	Mth938 dc	11.996	37	4	19	4	122	13.2
D3Z4J9	Echinoderi	11.993	6	3	16	3	800	88.3
E9Q9T1	Leucine-ric	11.981	7	4	28	3	628	71.1
Q9D4H9	PHD finger	11.96	4	3	49	3	881	99
Q8BP00	IQ calmod	11.954	6	3	12	3	598	68.7
Q91YT7	YTH doma	11.95	4	2	17	1	579	62.2
Q8VDT9	39S riboso	11.942	20	2	22	2	159	18.2
Q9CXG3	Peptidyl-p	11.936	6	2	2	2	492	57.2
Q9JJW6	Aly/REF ex	11.923	18	3	49	2	218	23.7
Q9DA08	SAGA-asso	11.909	10	2	13	2	293	33.3
P68433	Histone H3	11.904	24	4	142	1	136	15.4
A0A0U1RN	Zinc finger	11.889	3	4	11	4	1562	172.3
Q5XFZ0	UPF0711 p	11.883	15	3	23	3	217	24.1
Q6GYP7	Ral GTPas	11.881	2	4	10	4	2035	229.2
Q9JM76	Actin-relat	11.878	16	3	40	3	178	20.5
Q8BND4	Integrator	11.865	4	4	21	2	861	96.9
D3Z041	Long-chair	11.85	4	3	18	1	699	78
A0A6I8MX	Girdin OS=	11.842	2	4	8	4	2016	231.5
Q8BIW1	Exopolyph	11.836	7	2	9	2	454	50.2
Q8BQ46	TAF15 RN/	11.833	11	3	38	1	557	58.6
Q62120	Tyrosine-p	11.819	4	4	21	3	1132	130.5
Q9WVE8	Protein kir	11.819	11	4	14	4	486	55.8
P70698	CTP syntha	11.799	9	4	25	4	591	66.6
E9Q8M1	Zinc finger	11.795	6	3	28	3	571	59.7
O70589	Peripheral	11.793	4	3	13	3	926	105
G5E8T6	Membrane	11.79	3	4	5	4	1476	161.5
A0A1Y7VJ	Fibulin-5 C	11.789	7	3	25	3	461	51.7

Q6VGS5	Protein Da	11.777	3	3	7	3	2009	226.4
A0A1Y7VK	Nuclear fa	11.776	7	3	84	2	461	51.3
Q9Z1M2	Immunity-	11.769	14	4	7	4	395	45.2
A0A0G2JF	RNA-bindi	11.744	22	3	17	3	171	19.1
D3Z0J2	Caveolin (I	11.735	66	4	58	1	47	5.4
P00329	Alcohol de	11.715	13	4	23	4	375	39.7
Q3TPX4	Exocyst co	11.69	5	2	13	2	708	81.7
Q6ZWU9	40S riboso	11.686	23	2	84	1	84	9.5
P15306	Thrombom	11.684	8	3	7	3	577	61.8
Q3TUQ5	Pinin OS=M	11.682	6	4	20	4	726	82.5
Q7TPL6	Zinc finger	11.68	6	3	30	1	888	103.4
A0A0R4J0	ATP-bindir	11.679	5	4	16	4	741	83.4
Q8VDU5	SNF-relate	11.669	9	3	6	3	748	81.9
Q80X44	Zinc finger	11.663	6	3	15	3	710	78.7
Q8BKT7	THO comp	11.653	5	4	9	4	683	78.6
F8WH81	Vascular e	11.641	8	3	31	3	392	43.1
Q91XA5	snRNA-act	11.623	18	3	3	3	359	38.3
Q8BHS3	Pre-mRNA	11.621	7	2	15	2	420	46.9
Q08874	Microphth	11.615	5	2	11	1	526	58.6
Q9Z2Q5	39S riboso	11.615	13	2	64	2	206	24.3
Q3UK02	39S riboso	11.608	11	3	54	3	268	30.8
E9Q7W0	Recombin	11.595	7	3	23	3	485	54.3
F6YPZ1	Receptor a	11.59	22	2	12	2	141	16
Q8BSF4	Phosphatic	11.587	13	5	24	5	406	45.9
Q05DG3	Phosphoin	11.586	8	2	12	2	503	57.3
Q9WUA2	Phenylalar	11.576	6	4	17	4	589	65.7
A0A1W2P	Band 4.1-l	11.559	26	4	17	3	201	22.5
Q4FJZ2	Importin s	11.545	9	3	13	1	533	59.6
Q91WF3	Adenylate	11.514	3	2	12	2	1077	120.3
Q61136	Serine/thr	11.507	4	3	18	3	1007	116.9
Q80VM4	Zinc finger	11.506	8	3	15	3	562	60.8
D3Z2E7	Interferon-	11.499	4	2	6	1	971	103.8
Q9CY73	39S riboso	11.495	12	3	9	3	333	37.5
P17095	High mobi	11.485	38	4	38	1	107	11.6
B1AV77	Aldehyde c	11.481	6	3	23	3	487	54.3
Q9DCU6	39S riboso	11.48	10	3	57	3	294	33.1
E9PXC0	Signal reco	11.468	6	2	9	2	504	55.8
Q9CQW2	ADP-ribosy	11.463	20	4	24	1	186	21.5
Q9R0L6	Pericentric	11.456	2	3	17	3	2025	228.7
Q61146	Occludin C	11.444	9	3	14	3	521	59
Q4VAA7	Sorting ne	11.444	5	2	24	2	574	65.3
Q8R2S9	Actin-relat	11.443	6	2	18	2	624	70.5
Q9CR47	Ribosome	11.442	17	4	24	4	260	30
Q8R3C6	Probable F	11.429	3	3	12	3	952	106
O88543	COP9 signa	11.425	7	2	27	2	423	47.8

F8VQ05	FRY-like tr	11.425	2	4	20	4	3007	337.6
P62331	ADP-ribosy	11.419	18	2	11	2	175	20.1
Q9D0L7	Armadillo	11.41	15	4	9	4	306	33.3
Q64282	Interferon-	11.38	9	3	12	3	463	53.7
Q8BU30	Isoleucine-	11.378	3	3	9	3	1262	144.2
O88662	Epithelial	11.378	5	2	14	2	172	19.7
Q9QUK4	Baculovira	11.377	2	3	11	3	1447	164
Q9WV95	Pleckstrin	11.376	22	3	32	3	125	13.7
F6XLV1	Ciliary roo	11.374	4	4	16	4	1638	186.5
D3YUE7	GRAM dor	11.358	8	3	4	3	627	72.1
A0A1L1SV	Platelet-ac	11.357	14	2	17	2	197	22
Q9CQ69	Cytochrom	11.346	38	3	81	3	82	9.8
Q7TMF2	3'-5' exori	11.343	9	3	19	3	345	39.5
Q8C0D5	Elongation	11.339	4	4	36	4	1127	125.7
B2RX31	Zinc finger	11.338	6	3	22	2	543	60.7
Q3TBA3	Antigen pe	11.336	5	4	26	4	696	75.9
A0A0U1RF	Ataxin-2-li	11.335	4	4	15	4	1096	115.2
Q8C176	Transcripti	11.33	3	3	5	3	1104	126.9
F6W7C7	39S riboso	11.326	28	3	42	3	156	17.5
Q80VP0	Tectonin b	11.322	5	3	10	3	1166	130.2
Q8K2W3	Thioredoxi	11.306	5	4	6	4	948	105.9
P63158	High mobi	11.293	13	3	18	3	215	24.9
Q60520	Paired am	11.277	3	3	5	3	1274	145
Q62418	Drebrin-lik	11.271	9	3	19	3	436	48.7
P61079	Ubiquitin-	11.252	20	2	18	2	147	16.7
Q8C0E2	Vacuolar p	11.242	7	2	19	1	336	39.1
Q8C4J7	Transducir	11.241	5	2	17	2	801	88.2
Q3TWW8	Serine/arg	11.237	9	4	62	3	339	39
Q9D952	Envoplakir	11.233	3	4	7	4	2035	231.9
Q80T21	ADAMTS-l	11.231	4	3	13	3	1036	113.2
Q8VI93	2'-5'-oligo	11.22	3	3	19	3	1138	126.3
O35864	COP9 sign	11.219	10	3	23	3	334	37.5
Q3UHC2	Leucine-ric	11.218	2	3	8	3	2014	225.3
Q62036	Centrosom	11.213	3	3	6	3	1060	120.2
Q9D883	Splicing fa	11.212	12	3	49	3	239	27.8
P84244	Histone H3	11.192	24	4	144	1	136	15.3
Q4LFA9	Semaphor	11.18	5	4	19	4	780	86.6
A0A0R4J1	[Histone H	11.177	7	4	8	4	750	85.5
P14211	Calreticuli	11.175	8	3	7	3	416	48
Q62513	Zinc finger	11.162	9	3	9	3	407	46.4
O55187	E3 SUMO-	11.153	10	4	12	4	551	60.5
A0A494BA	Atlastin-3	11.153	14	2	21	2	232	25.7
O70572	Sphingomy	11.15	10	3	27	3	419	47.4
Q3UHJ0	AP2-assoc	11.139	5	3	8	3	959	103.3
Q9CQI7	U2 small r	11.139	13	3	67	3	225	25.3

O09110	Dual speci	11.11	9	4	43	4	347	39.3
A0A571BE	182 kDa ta	11.094	11	3	27	1	276	30.1
A0A6I8MX	L-lactate c	11.092	10	3	13	2	341	37.3
Q58A65	C-Jun-ami	11.077	3	3	5	3	1321	146.1
Q00547	Hyalurona	11.051	9	2	7	2	794	91.7
Q6NXL1	Sec24-rela	11.029	3	3	9	3	1032	112.6
Q9D1I6	39S riboso	11.022	24	3	44	3	145	15.9
O08807	Peroxiredo	11.015	14	3	96	1	274	31
Q8VDP2	STING ER	10.982	13	3	13	3	222	25.6
Q9R099	Transducir	10.961	10	3	10	3	442	49.6
E9Q638	Disintegrir	10.956	5	3	5	3	841	91.8
Q9CZ83	39S riboso	10.953	25	3	7	3	127	15.1
Q9CQ06	39S riboso	10.935	14	3	12	3	216	24.9
D3YXZ3	Kinesin lig	10.929	6	3	11	1	617	68.1
O35239	Tyrosine-p	10.917	5	3	12	3	593	67.9
P18293	Atrial natr	10.914	4	4	7	4	1057	119
P56183	Ribosoma	10.91	5	2	10	2	494	54.7
E0CXE7	CST compl	10.903	4	3	13	3	965	106.4
O09100	Endothelia	10.902	9	3	10	3	480	50.4
Q9ET78	Junctophil	10.899	4	3	25	3	696	74.6
Q9Z2F2	2'-5'-oligo	10.892	9	3	18	3	508	58.7
P56387	Dynein ligl	10.853	29	2	7	2	116	12.9
Q8BIS1	Zinc finger	10.849	11	4	14	2	454	52.9
Q8K1N4	Spermatog	10.843	6	3	14	3	545	58.9
P53395	Lipoamide	10.841	7	4	7	4	482	53.2
A0A1B0GI	Ferritin OS	10.818	18	2	18	2	160	18
E9PX07	Cytosolic c	10.811	9	3	17	3	332	37.1
Q9WUK4	Replicatio	10.804	8	3	6	3	349	38.7
P15105	Glutamine	10.795	12	4	11	4	373	42.1
A2ALV6	Proteasom	10.779	4	3	13	3	942	104.5
B9EJ54	Nucleopor	10.773	2	3	31	3	2008	227.3
Q8R1F0	Leydig cell	10.76	26	4	31	4	94	10.2
Q3UU43	Hexosyltra	10.748	8	4	8	4	768	85.6
A0A494B9	WD repeat	10.738	10	3	9	3	488	53.8
A0A3B2W	Bromodon	10.735	6	3	11	3	870	99.3
Q8C7R4	Ubiquitin-	10.729	2	2	12	2	1053	117.9
Q8VE22	28S riboso	10.726	16	2	16	2	177	20.3
P70452	Syntaxin-4	10.722	13	3	9	3	298	34.1
E9QAJ9	Rho GTPas	10.716	4	3	18	3	818	88.9
A0A075B5	Immunogl	10.711	28	2	6	2	95	10.3
Q8VE47	Ubiquitin-	10.708	8	3	19	3	403	44.8
Q8VHQ3	Protein ph	10.707	7	3	10	3	568	63.5
G3UW85	Enhancer c	10.707	12	2	32	2	123	14.3
Q9R1E0	Forkhead l	10.696	6	3	13	3	652	69.5
J3QNT7	Epsin-2 OS	10.69	4	3	7	3	640	68.3

D3Z4D8	BRISC com	10.687	10	3	9	3	289	33.5
P49138	MAP kinas	10.681	9	3	19	2	386	44
E9Q2I3	Protein ph	10.667	5	2	15	2	462	51.2
E9Q7D5	Rho guani	10.657	3	3	13	3	1581	176.6
Q6PEB6	MOB-like	10.648	10	2	14	2	225	26
A2A5K6	Zinc finger	10.64	4	3	14	3	1337	145.5
Q3UEB3	Poly(U)-bi	10.639	7	2	12	2	564	60.2
A0A0H2UK	Ubiquitin	10.632	3	2	17	2	897	101.4
A0A452J8/	Enoyl-CoA	10.623	9	2	6	2	286	32
Q8C6B9	Active reg	10.622	18	2	24	2	143	16
Q9CQD1	Ras-relate	10.622	16	3	45	1	215	23.6
P40201	Chromodo	10.617	3	4	9	3	1711	196.3
P07310	Creatine k	10.608	9	3	4	3	381	43
A3KGF9	Coiled-coil	10.606	5	3	19	3	545	58.7
Q9R1C6	Diacylglyce	10.592	5	3	15	3	564	63.6
A0A494B9	E3 ubiquit	10.587	3	3	18	2	912	105
Q8BJZ7	Thymocyte	10.583	17	3	23	3	167	19.3
D3YUP0	Vacuolar p	10.582	3	4	22	4	1429	161.2
Q9CQJ4	E3 ubiquit	10.58	12	3	37	2	336	37.6
Q60865	Caprin-1 C	10.569	4	3	36	3	707	78.1
B1ARB3	Platelet er	10.566	3	1	14	1	732	81.9
P10639	Thioredoxi	10.562	21	2	72	2	105	11.7
P01027	Compleme	10.537	2	4	19	4	1663	186.4
Q9ESX4	Nucleolar	10.532	5	1	27	1	241	27.5
Q9CQA5	Mediator	10.532	10	2	3	2	270	29.8
A0A087W	Leucine-ric	10.531	8	2	9	1	323	37
P61211	ADP-ribosy	10.522	20	3	40	3	181	20.4
A2AJ72	Far upstre	10.518	6	3	15	2	569	61.4
Q8BLF1	Neutral ch	10.516	6	2	3	2	408	45.7
Q9NWG9	Melanoma	10.505	9	1	20	1	218	24.3
P62334	26S protea	10.496	10	3	10	3	389	44.1
F8WHT3	Protein PR	10.493	3	3	9	3	2230	243
Q5SVR0	TBC1 dom	10.49	3	3	9	3	1263	141.7
Q91X78	Erlin-1 OS	10.489	12	3	36	1	348	39.2
E9Q9M8	PWWP do	10.478	4	2	10	2	600	65.4
Q5SUH7	Clathrin in	10.478	8	3	22	3	623	67.7
E0CYE2	Inosine-5'-	10.474	6	3	11	2	489	52.4
Q80YE7	Death-ass	10.473	3	3	13	3	1442	161.3
Q9D938	Transmem	10.453	11	2	57	2	188	19.6
Q9CR39	WD repeat	10.434	7	2	4	2	344	38
A3KFM7	Chromodo	10.431	1	3	18	2	2711	305.2
Q8VCG1	Deoxyuridi	10.427	23	4	11	4	200	21.2
B7ZMP1	Xaa-Pro ar	10.413	6	2	8	2	506	56.6
Q6P4S8	Integrator	10.409	2	3	13	3	2195	245
Q8C522	Endonucle	10.399	7	3	30	3	501	55.2

A0A0G2JE	SPT20 SAG	10.395	6	2	12	2	529	59.4
Q9WVM1	Rac GTPas	10.379	8	3	5	3	628	70.1
Q61263	Sterol O-a	10.351	7	3	19	3	540	63.8
D3Z3K7	E3 SUMO-	10.349	9	2	8	2	412	43.8
Q9CY21	Probable 1	10.346	10	2	17	2	281	31.6
Q9JJU8	SH3 doma	10.346	22	2	12	2	114	12.8
Q497V9	Zinc finger	10.326	8	3	14	1	528	60.8
A0A1D5RL	RNA-bindi	10.317	9	3	19	3	422	46.7
Q71FD7	Filamin-bi	10.312	9	2	37	2	375	41
E9PVA8	eIF-2-alph	10.301	2	4	6	4	2671	292.8
E9Q4Q2	Splicing fa	10.286	7	3	10	3	548	59.7
Q9D104	Signal reco	10.26	11	2	21	2	144	16.1
Q9Z1Q5	Chloride ir	10.259	14	3	12	3	241	27
Q3TE80	RIKEN cDN	10.258	10	3	5	3	284	32.4
G3X9K3	Brefeldin /	10.244	1	3	14	1	1846	208.4
O88673	Diacylglyc	10.225	5	2	6	2	730	82.7
A0A0R4J1	Transmem	10.214	9	2	17	2	246	27.9
G3X9H7	Protein M	10.208	4	3	5	3	759	82.4
Q9DCD2	Pre-mRNA	10.208	4	3	10	3	855	99.9
Q9ERG2	Striatin-3	10.201	4	3	15	1	796	87.1
Q56A04	Probable c	10.19	12	3	17	3	373	41.1
Q9WUM5	Succinate-	10.179	7	2	47	2	346	36.1
P97390	Vacuolar p	10.166	5	3	7	3	570	65
Q8BSK8	Ribosomal	10.156	5	2	6	2	525	59.1
F8VPU8	Expressed	10.155	8	2	21	1	497	57.7
Q01149	Collagen a	10.154	2	3	16	3	1372	129.5
Q3UVG3	Protein FA	10.153	3	2	7	2	837	93.4
Q542V3	Serine/arg	10.149	7	3	45	2	491	56.2
Q61598	Rab GDP c	10.147	7	3	12	3	445	50.5
Q80YR4	E3 ubiquit	10.146	2	2	6	2	908	99.1
O54785	LIM doma	10.135	5	3	18	3	638	72.2
Q3UHQ6	Protein do	10.134	1	2	13	2	2295	257.3
A0A0R4J0	Lamin-B2	10.131	6	4	16	2	615	69
Q8R035	Peptidyl-tF	10.123	16	3	11	3	206	23.5
Q9DCT2	NADH deh	10.115	10	2	9	2	263	30.1
Q9JIB4	General tr	10.114	8	3	13	3	396	44.7
Q6PE54	Probable A	10.099	4	3	12	3	779	88.5
Q6NXW6	Cell cycle c	10.082	5	2	3	2	688	77.3
A0A0G2JG	RNA helica	10.074	2	3	36	3	1186	132.9
Q9CQA3	Succinate	10.073	11	3	21	3	282	31.8
Q9DB96	Neuroguid	10.067	11	3	15	3	315	35.6
A2AFQ2	3-hydroxya	10.058	15	3	7	3	271	28.4
Z4YL78	Cytoskelet	10.053	1	2	9	2	1972	218.6
A2A715	39S riboso	10.051	13	2	39	2	184	20.8
Q922J3	CAP-Gly do	10.05	3	4	18	3	1391	155.7

Q8C142	Low densi	10.046	10	3	16	3	308	34
Q80SY4	E3 ubiquit	10.045	4	3	12	3	1006	110
Q9Z0H8	CAP-Gly do	10.028	4	4	9	3	1047	115.8
A2ARS0	Ankyrin re	10.013	7	3	32	3	390	41
A0A589P9	Neuron na	10.011	2	3	8	3	2098	225.8
Q9DCC1	mRNA-cap	10	7	3	4	3	581	66.8
A0A0R4J0	Long-chair	9.991	8	3	8	3	430	47.9
Q9DBB4	N-alpha-a	9.983	6	3	10	3	864	101.2
Q8VHK9	ATP-deper	9.981	4	3	18	2	1001	113.8
A2ALW5	DnaJ homo	9.961	6	2	19	2	357	41.9
Q8CG79	Apoptosis-	9.957	5	3	8	2	1128	125.2
Q64355	Embryona	9.954	4	1	7	1	560	58.9
Q8R1I1	Cytochrom	9.946	38	2	46	2	64	7.4
Q03160	Growth fa	9.939	9	3	3	2	535	59.9
I1E4X1	Syntaxin 5	9.934	6	3	19	3	442	49.1
Q8BSL0	Zinc finger	9.93	5	3	42	2	588	66.6
P50543	Protein S1	9.924	28	3	46	3	98	11.1
O35680	28S riboso	9.92	25	2	2	2	139	15.4
Q9R111	Guanine d	9.903	8	4	10	4	454	51
Q922Q2	Serine/thr	9.872	5	3	7	3	567	64.9
Q80X19	Collagen a	9.865	3	3	5	3	1797	192.9
Q9DCL9	Multifunct	9.858	6	2	8	2	425	47
O35226	26S protea	9.84	7	2	7	2	376	40.7
A0A286YE	Protein sp	9.84	10	2	9	2	344	39.6
Q9JLF6	Protein-glu	9.822	4	3	5	3	815	89.8
E9QKZ2	Importin-9	9.815	4	4	21	4	1040	115.9
S4R299	Zinc finger	9.801	2	3	20	1	1847	202.7
Q78YZ6	Short coile	9.801	14	1	12	1	125	14.1
Q8VCD5	Mediator c	9.801	8	3	6	3	649	72.4
A0A2R8VH	Aquaporin	9.786	16	2	35	2	148	16.3
B2KF11	BTB/POZ c	9.761	13	3	23	3	286	32.6
P59598	Polycomb	9.739	2	2	10	2	1514	162.6
Q68FM4	Protein-tyr	9.736	3	3	9	3	1486	167
Q9ES97	Reticulon-	9.735	3	3	10	3	964	103.8
E9PVC7	Cyclin-dep	9.731	8	3	33	2	451	50.4
Q4FK66	Pre-mRNA	9.722	10	2	5	2	312	37.4
Q14AI6	Mitochond	9.714	7	2	23	2	344	38.1
A0A494BA	Endoplasm	9.712	10	1	5	1	247	27.7
A0A140LJ7	Protein arg	9.711	12	2	10	2	254	28.9
Q8CCJ3	E3 UFM1-	9.706	5	3	10	3	793	89.5
A2A4B1	Phosphatic	9.699	5	3	4	3	858	95.4
Q8BG60	Thioredoxi	9.693	7	2	20	2	397	44.3
Q9CQ10	Charged m	9.69	13	3	24	3	224	25.2
Q6P5D8	Structural	9.681	2	2	12	2	2007	225.5
E9QMH1	DNA dC->c	9.67	6	2	30	2	429	51

Q8C402	Lymphoid	9.64	6	2	41	1	331	37
A0A087W	Lymphocyt	9.617	25	2	13	2	147	16
Q8VCL2	Protein SC	9.613	12	2	7	2	255	28.9
Q68FL4	Putative a	9.601	5	3	11	1	613	66.9
Q8CF60	Zinc finger	9.595	5	3	9	1	680	77.5
Q8BG51	Mitochond	9.594	4	2	10	2	631	72.2
Q9WUQ5	C-X-C moti	9.564	26	3	65	3	99	11.7
Q99KH8	Serine/thr	9.56	8	3	12	1	431	47.9
Q0VAW7	Zinc finger	9.555	7	3	21	1	879	98.4
Q8BUN5	Mothers a	9.552	7	3	8	2	425	48.1
Q60841	Reelin OS=	9.55	1	3	6	2	3461	387.2
D3Z6R9	DNA-(apur	9.544	18	3	4	3	214	23.7
Q0PHV7	Dapper ho	9.541	7	3	20	3	610	63.2
F6WV21	Putative P	9.53	2	2	13	1	1370	147
A0A0R4J0	Tyrosine-p	9.529	4	3	11	2	849	97.6
G5E8P1	Bromodon	9.503	3	3	11	3	1058	119.6
Q9D0B0	Serine/arg	9.49	17	3	8	3	222	25.6
Q8K4Q0	Regulatory	9.479	2	2	21	2	1335	149.4
Q9JMB0	G kinase-a	9.468	15	3	7	3	366	41.7
Q64435	UDP-glucu	9.462	7	3	16	1	531	60.4
Q3ULG5	DNA helica	9.458	4	3	7	3	794	89.7
P28741	Kinesin-lik	9.452	5	3	10	3	701	80.1
F8VQK3	Guanylate	9.451	5	3	17	3	730	81.8
Q1W617	Protein Sh	9.439	2	2	2	2	1475	163.1
B1AU25	Apoptosis-	9.437	7	3	9	3	608	66.1
Q8K1C0	Protein an	9.43	6	2	3	2	544	62.4
P24369	Peptidyl-p	9.426	10	2	35	2	216	23.7
P47856	Glutamine	9.423	6	3	7	3	697	78.5
Q921F2	TAR DNA-	9.42	8	3	27	3	414	44.5
Q6P9N1	Hyccin OS=	9.42	4	1	2	1	521	57.3
Q9QWV9	Cyclin-T1 (	9.41	4	3	13	3	724	80.5
Q3U5V8	Syntaxin-1	9.409	12	3	17	3	287	33.4
Q8BZQ7	Anaphase-	9.408	4	3	11	3	837	95.2
Q6PD03	Serine/thr	9.405	8	3	8	3	486	56.3
O08599	Syntaxin-b	9.399	5	2	6	2	594	67.5
P16254	Signal rec	9.39	19	3	10	3	110	12.5
Q8C7U1	NEDD4-bir	9.387	7	3	17	3	537	60
A2AWT7	Nucleolar	9.384	5	3	10	3	752	88.3
G3X925	Pyruvate k	9.38	6	2	15	1	574	62.2
Q8K2J0	1-phospha	9.378	4	3	23	3	785	88.6
S4R1H5	Zinc finger	9.366	4	2	3	2	779	81.7
F7AI47	Serine/arg	9.365	21	2	18	2	106	11.6
E9PY25	G patch dc	9.36	8	2	11	2	375	42.2
D3Z0S1	Annexin (F	9.355	14	3	5	3	254	28.5
Q9CR08	Ribonuclea	9.353	10	2	16	2	221	25.6

Q80SW1	S-adenosy	9.334	5	3	8	1	530	58.9
A2ARA0	Tissue fac	9.333	12	2	12	2	300	34.4
A0A1Y7VN	Serine/thr	9.316	6	2	20	2	413	46.8
F6TLV3	Fibronecti	9.315	3	3	22	3	1154	127.2
Q5RL20	39S riboso	9.31	19	3	6	3	159	17.9
Q91WT8	RNA-bindi	9.308	7	3	9	3	590	64
F6VQH5	Heterogen	9.306	8	3	55	1	323	35.6
P83093	Stromal in	9.3	4	2	10	2	746	83.9
Q91VJ2	Caveolae-a	9.294	11	3	9	3	260	27.8
A0A1L1SR	POC1 cent	9.289	13	2	11	2	299	33.2
Q9Z1A1	TFG protei	9.289	11	3	17	3	397	43
Q9R118	Serine pro	9.287	7	3	16	3	480	51.2
Q8CB76	Zinc finger	9.281	6	3	22	2	518	59.8
Q8BMF4	Dihydrolip	9.279	6	3	18	3	642	67.9
O88845	A-kinase a	9.272	3	1	14	1	662	73.6
E9Q7E2	AT-rich int	9.27	2	3	10	3	1828	195.9
Q8BGS5	Zinc finger	9.27	6	3	24	1	496	55.4
Q99MR8	Methylcro	9.264	4	2	5	2	717	79.3
Q9D8U8	Sorting ne	9.246	8	3	8	3	404	46.8
Q09014	Neutrophi	9.244	7	3	32	3	390	44.6
Q8VDM6	Heterogen	9.236	4	2	12	2	859	95.9
A0A0A0M	Mitogen-a	9.22	4	4	7	4	1503	162.8
Q91ZJ0	Crossover	9.205	5	3	16	3	551	61.5
Q3TXS7	26S protea	9.197	3	3	7	3	953	105.7
Q8K0V4	CCR4-NOT	9.195	4	2	7	2	751	81.9
Q9JIM3	DNA excisi	9.188	2	2	9	2	1537	173.8
Q4V9W2	Protein SR	9.159	16	2	28	2	153	18.1
J3QQ02	Eukaryotic	9.155	15	2	17	1	144	16.6
Q3UZG4	Aminoacyl	9.155	12	2	16	2	319	35.1
P10284	Homeobox	9.144	11	2	15	2	250	27.5
P09103	Protein dis	9.134	5	2	6	2	509	57
Q9D0R4	Probable A	9.134	5	2	9	2	546	61.2
Q8CEC4	Bteb1 prot	9.131	17	3	6	3	244	27.2
A0A0G2JG	Guanine n	9.124	9	3	22	1	316	37
Z4YK03	Leukocyte	9.117	13	3	20	3	188	20.9
I7HPV9	Phosphatic	9.117	2	2	21	2	1480	166.1
D3Z383	Mesoderm	9.113	8	2	2	2	342	39.5
E0CXR5	Cerebral c	9.11	13	2	7	2	243	26.8
Q8R1G1	Threonine	9.103	6	3	28	3	420	44.3
F6V8M6	Ataxin-2 (I	9.099	4	2	7	2	662	70.4
A0A1D5RL	WD repeat	9.097	1	3	16	3	3512	392.6
Q99KK1	Receptor e	9.085	9	2	3	2	254	29.2
Q8BTW3	Exosome c	9.071	17	3	9	3	273	28.4
P97872	Flavin-con	9.068	7	2	12	2	533	60
P29268	CCN family	9.068	8	3	11	3	348	37.8

Q8BVW3	Tripartite	9.062	8	2	3	2	440	49.6
Q7M6Y0	Regulator	9.06	6	3	9	2	485	56.9
Q9WTP7	GTP:AMP	9.058	15	3	7	3	227	25.4
O35493	Dual speci	9.047	7	2	17	1	481	57.3
O35245	Polycystin-	9.038	2	2	4	2	966	108.9
Q8BZF8	Phosphogl	9.03	6	4	18	4	567	62.2
Q9CQH8	Ribonuclea	9.028	27	3	19	3	122	13.6
Q99K41	EMILIN-1 (	9.027	4	3	11	3	1017	107.5
A0A0G2JE	Myocyte-s	9.024	5	2	23	1	434	47
A2RSQ0	DENN dom	9.024	3	3	15	1	1274	144.5
Q9QYB1	Chloride ir	9.014	17	3	10	3	253	28.7
Q80V42	Carboxype	9.004	4	1	12	1	443	50.5
P46978	Dolichyl-di	9.002	3	3	28	2	705	80.5
O08759	Ubiquitin-	8.997	4	2	6	2	870	99.8
Q6P2B1	Transporti	8.994	4	3	6	3	923	104.1
O88796	Ribonuclea	8.993	10	2	7	2	268	29.5
Q9CRB6	Tubulin po	8.989	13	2	51	2	176	19
Q93092	Transaldol	8.979	8	3	8	3	337	37.4
D3YZW1	SLIT-ROBO	8.979	3	2	20	1	1085	124
P27601	Guanine n	8.965	7	3	28	1	377	44
B1Q2W7	Transcripti	8.964	1	2	10	2	1893	214.5
Q6I6G8	E3 ubiquit	8.958	2	2	9	2	1578	176.1
Q3TNL8	Inositol 1,4	8.948	7	3	12	3	555	63.2
A2A5R8	Double-str	8.944	6	3	17	3	485	53.7
Q9JMG1	Endothelia	8.932	16	2	10	2	148	16.4
P09470	Angiotens	8.919	2	3	7	3	1312	150.8
Q8BJZ0	Zinc finger	8.917	4	2	11	2	548	64.2
Q80UW8	DNA-direc	8.902	14	3	16	3	210	24.6
A0A5F8MI	Rho guani	8.9	3	3	8	3	1405	155.8
Q9R0Q1	Synaptotag	8.881	3	2	4	2	673	76
Q05D44	Eukaryotic	8.878	2	2	16	2	1216	137.5
Q91VD9	NADH-ubi	8.865	3	1	10	1	727	79.7
F6WR04	Cathepsin	8.857	5	1	1	1	341	38.6
Q64339	Ubiquitin-	8.834	16	2	8	2	161	17.9
D3Z598	Latent-tra	8.831	3	3	6	3	1558	166.7
Q3U2X6	Programm	8.824	7	3	3	3	484	54.5
P55041	GTP-bindi	8.822	9	3	23	2	295	33.7
Q2M3X8	Phosphata	8.815	5	2	16	2	580	66.2
P63254	Cysteine-ri	8.806	49	2	7	2	77	8.5
Q9WVJ2	26S protea	8.793	9	3	12	3	376	42.8
A0A338P7	Ubiquitin-	8.791	21	2	9	2	146	16.9
P10493	Nidogen-1	8.779	3	3	17	3	1245	136.5
B1AU76	Nuclear au	8.774	4	1	6	1	448	48.7
Q3UGS4	Mapk-regu	8.772	19	1	5	1	97	11.1
A0A2R8W	DNA helica	8.765	3	2	8	2	1173	129.6

Q91Z96	BMP-2-inc	8.753	3	2	2	2	1138	126.1
Q01768	Nucleoside	8.751	17	2	21	2	152	17.4
D3YXK6	ETS domai	8.748	15	1	19	1	149	17.5
Q8VIG1	RE1-silenc	8.74	4	3	8	2	1082	117.7
A2BDX3	Adenylyltra	8.736	7	3	13	3	460	49.3
Q5U430	E3 ubiquit	8.733	2	3	7	3	1889	212.6
B1AYU7	Pre-mRNA	8.732	4	4	14	4	903	104.3
Q80XC3	USP6 N-te	8.731	8	3	3	3	819	93.5
Q6NS86	Zinc finger	8.72	4	3	6	2	746	84.8
Q3UMW7	MAP kinas	8.701	7	2	8	1	384	43.3
Q99L48	60S riboso	8.69	6	3	16	3	503	57.6
Q9CZA6	Nuclear di	8.68	10	3	6	3	344	38.5
Q8R5F7	Interferon-	8.672	3	3	13	3	1025	115.9
F6VRG5	Proline-ric	8.659	8	2	30	2	238	26.3
Q9CQN7	39S riboso	8.651	17	2	9	2	135	15.3
Q9JIX0	Transcripti	8.637	24	2	10	2	101	11.5
P09411	Phosphogl	8.626	8	2	5	2	417	44.5
Q76LS9	Ubiquitin c	8.592	8	2	9	2	468	51.2
A0A087W	MISP fami	8.585	17	2	10	2	206	23.2
E9Q4T4	Coiled-coil	8.584	10	2	15	2	218	24.2
O08915	AH recepto	8.578	6	1	4	1	330	37.6
Q8K0L9	Zinc finger	8.558	6	3	12	3	741	81
Q9JHG7	Phosphatic	8.557	4	3	8	3	1102	126.3
Q9D7S9	Charged m	8.557	7	1	23	1	219	24.6
A0A0G2JE	Syntaxin-b	8.546	16	3	10	3	236	27.2
E9QLT6	Aryl hydro	8.542	5	2	15	2	771	84.5
Q99PI5	Phosphatic	8.533	2	2	26	1	893	99.6
E9Q2S7	Zinc finger	8.524	4	2	5	1	604	68.4
E9Q9H3	DnaJ homo	8.515	7	3	8	3	619	71.2
Q9Z103	Activity-de	8.508	3	3	8	3	1108	124.2
B1AQH6	Mediator c	8.496	7	3	5	3	629	70.1
A0A571BG	Active brea	8.495	3	3	18	2	1185	132.9
Q9CQI6	Coactosin-	8.491	30	3	9	3	142	15.9
A2BE20	Novel KRA	8.486	11	2	14	1	318	36.7
P11928	2'-5'-oligo	8.486	7	1	4	1	367	42.4
E9QP44	Arf-GAP w	8.485	1	2	7	2	1703	193.3
Q5F2E7	Nuclear fra	8.484	3	2	10	2	692	75.6
A6H5Z3	Exocyst co	8.482	3	2	9	2	810	94.1
Q8CEI3	Coiled-coil	8.468	14	2	4	2	192	21.1
G3X9W4	Nuclear re	8.459	6	2	8	2	629	68.8
Q60821	Zinc finger	8.451	4	3	11	3	794	86.7
B2RRL2	Jerky prote	8.448	6	2	17	2	523	59.7
Q80TF6	StAR-relat	8.446	1	3	8	1	4561	499.9
P26151	High affini	8.428	5	2	30	2	404	44.9
Q8VDD9	PH-interac	8.425	2	3	6	3	1821	206.6

Q8C0T5	Signal-ind	8.425	2	2	4	1	1782	196.9
E9Q8B6	Compleme	8.412	5	3	15	3	820	92.8
Q5U4C1	G-protein	8.411	2	2	6	2	1347	151.7
Q9DCE9	Interferon	8.406	9	3	23	3	423	48.5
F8WIV2	Serine (or	8.396	8	3	28	3	399	44.7
Q9QUK9	TESP4 OS=	8.393	8	1	221	1	246	26.3
Q9CQ62	2,4-dienoy	8.38	9	2	9	2	335	36.2
P06909	Compleme	8.348	2	2	18	2	1234	139
Q8JZL0	Zinc finger	8.348	3	2	23	2	594	65.6
Q8JZX9	Cdc42 effe	8.346	16	2	6	2	214	23
Q9D0D5	General tr	8.346	5	2	26	2	440	49.6
G3UWX1	Replicatio	8.344	3	3	5	3	1145	127.5
Q8BGS0	Protein M/	8.34	9	2	7	2	296	35.2
G3X8R7	Zinc finger	8.337	7	3	6	2	492	55.4
B2LVG5	Cleavage a	8.334	17	3	3	3	243	27.4
Q8R4I1	Ataxin-7 C	8.33	7	3	15	3	867	92.6
P01872	Immunogl	8.329	6	2	20	2	454	49.9
Q3UYV9	Nuclear ca	8.319	4	2	18	2	790	91.9
Q3UZA1	CapZ-inter	8.312	8	3	12	3	412	44.1
F7D3E1	Coiled-coil	8.31	6	2	17	2	469	54.6
Q5FWK3	Rho GTPas	8.308	7	3	10	3	439	50.4
Q5SUT0	RNA-bindi	8.307	5	2	21	2	618	64.9
Q9WUU7	Cathepsin	8.297	9	2	17	2	306	34
P97352	Protein S1	8.295	23	2	5	2	98	11.2
E9Q3M0	Mothers a	8.275	8	2	6	1	434	48.7
G3UZ01	Polypyrimi	8.265	8	3	30	1	457	49.6
Q8BXV2	BRI3-bindi	8.262	10	2	14	2	253	28.2
Q791T5	Mitochonc	8.239	7	3	10	3	389	41.5
Q8BVG4	Dipeptidyl	8.239	4	3	8	3	862	97.9
Q62077	1-phospha	8.236	3	3	9	3	1302	149.6
E9Q9B3	SPRY dom	8.236	7	3	18	3	442	49.7
Q9JJZ5	Epidermal	8.232	8	3	17	3	550	61.5
Q9JLQ0	CD2-assoc	8.231	7	3	5	3	637	70.4
S4R1X1	WD repeat	8.221	4	2	7	2	604	67.7
Q8C788	Sorting ne	8.22	5	3	12	3	615	67.7
Q9JK48	Endophilin	8.212	9	3	10	3	365	40.8
E9PUK3	Formin-bi	8.21	5	3	6	3	551	63.9
A0A0R4IZ\	Cadherin-1	8.206	3	2	10	2	884	98.2
E9QAT4	Protein tra	8.202	2	3	7	3	2357	254
Q91WQ5	TAF5-like	8.196	4	2	15	2	589	65.9
P62748	Hippocalci	8.193	11	2	5	2	193	22.3
Q9DB15	39S riboso	8.185	10	3	17	3	201	21.7
Q9QWH1	Polyhome	8.185	3	1	5	1	850	89.7
Q8BPP0	Zinc finger	8.181	8	3	8	2	452	52.3
Q7TQF2	F-box only	8.17	3	3	12	3	950	104.4

P22315	Ferrochela	8.168	5	1	13	1	420	47.1
Q62465	Synaptic v	8.164	7	2	17	2	406	43.1
Q9D824	Pre-mRNA	8.16	9	3	5	3	581	64.9
Q9CX34	Protein SG	8.156	8	2	9	2	336	38.1
Q6PD21	SH2 doma	8.132	7	2	2	2	503	54.7
O54734	Dolichyl-di	8.122	5	2	6	2	441	49
Q9CU65	Zinc finger	8.122	3	3	6	3	1376	154.5
H9KV21	RBR-type	8.116	6	2	9	2	476	56.2
A0A087W	Arf-GAP d	8.115	8	2	3	2	521	53.9
Q99P91	Transmem	8.106	3	1	11	1	574	63.6
B9EHX4	Serine-pro	8.101	1	3	7	3	3063	348.8
H3BKX4	Transcript	8.091	5	1	5	1	387	43.9
Q6PGF5	BMS1 hom	8.083	2	2	16	2	1284	145.3
P08905	Lysozyme	8.079	27	2	5	2	148	16.7
Q3UIR3	E3 ubiquit	8.067	6	3	18	3	748	83
O35682	Myeloid-a	8.063	8	2	23	2	320	35.3
A0A0R4J1	Peptide-m	8.059	11	2	14	2	186	20.2
A0A286YD	Signal seq	8.044	20	2	28	2	115	13.1
Q9CRB2	H/ACA ribo	8.038	22	2	11	2	153	17.2
Q62426	Cystatin-B	8.031	33	2	13	2	98	11
P46737	Lys-63-spe	8.024	10	2	7	2	291	33.3
Q9Z0G0	PDZ doma	8.022	9	2	5	2	333	36.1
D3Z7R9	Cell cycle	8.017	2	1	4	1	778	89.1
Q925B0	PRKC apop	8.016	15	2	13	2	333	35.9
Q64133	Amine oxi	8.012	7	3	8	3	526	59.6
P97333	Neuropilin	8.009	5	3	14	3	923	102.9
Q9Z116	KRAB-cont	8.005	6	2	30	1	606	71.1
Q7TMI3	E3 ubiquit	8.003	5	2	6	2	803	90
Q9Z2I9	Succinate-	7.995	5	2	21	2	463	50.1
D3YZV8	Coiled-coil	7.989	12	3	9	3	685	73.9
Q9JLL0	Cysteine-ri	7.987	3	2	6	2	1037	114
Q9QXM1	Junction-m	7.979	2	1	10	1	983	110.5
Q61142	Spindlin-1	7.978	13	3	6	3	262	29.6
Q6PGF3	Mediator	7.971	6	3	6	3	828	91.7
E9PX66	STAGA cor	7.963	10	3	10	3	344	38.5
Q91XD6	Vacuolar p	7.963	9	3	4	3	386	43.7
Q8BTW9	Serine/thr	7.958	5	2	12	2	593	64.6
B2RXQ2	Ppfia1 pro	7.95	2	2	9	2	1266	142.6
Q9CQJ6	Density-re	7.939	9	1	8	1	198	22.2
Q8BMN7	Guanylate	7.923	2	1	6	1	724	80.4
P27612	Phospholip	7.91	4	2	5	2	794	87.2
Q8BU00	Zinc finger	7.903	5	3	17	2	419	46.4
Q91ZX6	Sentrin-sp	7.903	6	2	4	2	588	67.5
O08856	RNA polyn	7.9	7	3	4	3	602	67.1
P59708	Splicing fa	7.888	18	2	21	2	125	14.6

Q923L7	Ear6 prote	7.883	8	1	30	1	155	17.3
Q8CGK3	Lon protea	7.88	3	2	13	2	949	105.8
A0A0R4J0	Protein arg	7.874	5	2	9	2	637	72.7
Q91YS8	Calcium/ca	7.869	5	1	8	1	374	41.6
B2RX14	Terminal u	7.868	2	3	12	1	1644	184.5
Q8C570	mRNA exp	7.868	9	3	12	3	368	40.9
P0C7Q1	Coiled-coil	7.865	12	2	20	2	202	23.4
A0A2I3BPI	Tuberin OS	7.862	2	3	13	3	1785	198.7
Q3UV55	Nuclear re	7.856	5	2	16	2	615	66.8
Q9D071	MMS19 nu	7.856	3	3	7	3	1031	113
Q8VI84	Nucleolar	7.835	5	2	3	2	807	93.2
P62881	Guanine n	7.825	5	1	8	1	395	43.5
A0A0N4SV	Rab11 fam	7.817	3	3	4	3	1318	139.5
E0CXR8	Partitionin	7.807	2	2	22	2	990	108.8
Q3U0M1	Trafficking	7.803	2	2	7	2	1148	128.2
Q9D832	DnaJ hom	7.803	8	2	14	2	337	37.8
Q3TIV5	Zinc finger	7.771	5	1	3	1	426	48.3
G3UVV3	Zinc finger	7.771	2	3	10	2	2246	245.8
Q9QXB9	Developm	7.766	7	2	20	2	364	40.7
Q91XU3	Phosphatic	7.763	3	1	9	1	421	47.3
P10076	Zinc finger	7.76	4	2	31	1	861	97.9
P63154	Crooked ne	7.75	5	3	5	3	690	83.4
E9Q967	Segment p	7.742	3	2	13	2	718	78.3
Q9JKS5	Intracellula	7.742	6	2	12	2	411	45.9
Q8BXZ1	Protein dis	7.735	6	3	27	3	456	51.8
A0A0A0M	Rho GTPas	7.735	2	2	8	2	1949	216.3
P49962	Signal reco	7.727	13	1	10	1	86	10.2
A0A0R4J2	BTB/POZ d	7.721	6	2	5	2	327	35.9
Q62376	U1 small n	7.709	7	3	16	3	448	52
D3Z5I9	RNA-bindi	7.709	3	2	3	2	1191	132.7
Q6ZQM8	UDP-glucu	7.709	6	3	10	1	531	59.7
G3UYY9	Histone-ly	7.707	3	2	8	2	606	69
P97377	Cyclin-dep	7.706	7	2	30	1	346	39
Q9CS42	Ribose-ph	7.705	9	3	22	1	318	34.8
Q5NCF2	Trafficking	7.702	18	2	6	2	145	16.9
P70122	Ribosome	7.699	7	1	15	1	250	28.8
Q8CEC0	Nuclear po	7.699	6	3	6	3	753	84.9
Q7TQG1	Pleckstrin	7.699	3	3	4	3	1173	131.3
F6V084	Thioredoxi	7.697	19	2	6	2	124	14.4
P60003	Transcripti	7.697	22	1	11	1	83	9.5
Q6PCN7	Helicase-li	7.696	4	2	21	2	1003	113.2
Q3UBZ5	MIF4G dor	7.691	14	2	8	2	222	25.5
A0A0A6YV	Musclebli	7.671	8	3	26	3	347	37.8
B1AX39	Zinc finger	7.667	5	2	10	2	541	63
Z4YLG9	Disrupted	7.664	3	2	3	2	789	85.4

Q640N1	Adipocyte	7.663	3	2	4	2	1128	128.3
D3Z359	Serine/thr	7.648	14	3	11	1	234	26.3
Q8BIZ6	Smad nucl	7.631	5	2	4	2	383	44.4
P97770	THUMP dc	7.627	5	2	5	2	505	56.4
Q99L04	Dehydroge	7.62	7	2	11	2	313	34
E9Q411	Neuroblas	7.615	1	3	4	3	2356	265.6
P62814	V-type pro	7.613	4	1	7	1	511	56.5
Q8CJ40	Rootletin (	7.596	1	3	6	2	2009	226.8
Q80ZE3	Sialic acid-	7.583	5	2	9	2	688	76.8
Q62241	U1 small r	7.573	11	1	7	1	159	17.4
Q60698	Ski oncoge	7.568	4	2	3	2	725	80.1
P24547	Inosine-5'-	7.567	5	2	12	1	514	55.8
E9Q722	Upstream	7.565	6	1	14	1	296	32
P46467	Vacuolar p	7.562	4	1	15	1	444	49.4
K3W4S6	Glycogenin	7.562	6	2	11	2	377	41.9
Q5SW75	Protein ph	7.544	2	2	4	2	1423	158.1
Q9Z2U1	Proteasom	7.534	8	1	1	1	241	26.4
Q8K327	Chromoso	7.53	2	2	20	2	802	87.5
Q8R5H1	Ubiquitin c	7.529	2	2	16	1	981	112.3
P16951	Cyclic AMP	7.528	6	2	8	2	487	52.3
F8WJ98	Transmem	7.526	3	2	12	1	690	76.1
P50247	Adenosylh	7.522	5	2	5	2	432	47.7
Q8BG79	CWF19-lik	7.512	2	2	16	2	887	103.1
P13597	Intercellula	7.51	3	2	25	2	537	58.8
Q3UMU9	Hepatoma	7.504	5	2	7	1	669	74.2
Q52KG4	Zinc finger	7.501	5	2	12	2	520	55
E9Q5E0	Myocyte-s	7.493	4	2	18	1	506	54.1
Q9Z0R6	Intersectin	7.49	2	2	13	2	1659	188.8
Q3TFZ4	Zinc finger	7.49	3	2	10	2	685	78.7
Q3UMB9	WASH con	7.485	2	2	10	2	1173	136.3
Q91VL9	Zinc finger	7.476	3	2	13	2	713	81.9
A0A1D5RL	Protein IW	7.47	3	2	17	2	765	85.3
P57746	V-type pro	7.469	9	2	18	2	247	28.4
P24788	Cyclin-dep	7.466	2	2	35	2	784	91.5
Q9DBE8	Alpha-1,3/	7.454	4	1	19	1	415	47.4
Q3TFK5	G patch dc	7.453	8	2	6	2	415	46.5
P11152	Lipoprotei	7.438	4	1	6	1	474	53.1
D6RGR4	WD repeat	7.431	13	3	7	3	262	29
O08804	NK13 OS=	7.42	6	2	20	2	377	42.5
Q8K245	UV radiati	7.418	3	2	13	2	698	77.5
Q80ZX0	Sec24-rela	7.41	2	2	2	2	1251	135.5
E9QAD3	Metal-resp	7.398	4	2	13	2	536	60.5
P28574	Protein ma	7.395	13	2	30	2	160	18.2
P97813	Phospholip	7.387	2	2	11	2	933	106.1
A0A0R4J0	TRMT1-lik	7.384	5	2	9	2	728	80.9

O88545	COP9 signa	7.37	7	2	9	2	324	35.9
Q99P72	Reticulon-	7.368	3	3	6	3	1162	126.5
Q8VHR5	Transcripti	7.359	4	1	3	1	594	65.4
O55071	Cytochrom	7.358	3	1	5	1	492	56
P28653	Biglycan O	7.356	5	2	24	2	369	41.6
Q8K2A4	Zinc finger	7.344	6	2	22	1	540	63
Q9QXX4	Calcium-bi	7.334	4	2	19	1	676	74.4
Q61249	Immunogl	7.327	9	2	12	2	340	38.9
G3X941	RIKEN cDN	7.326	6	3	7	3	543	62.1
O54825	Bystin OS=	7.315	5	2	3	2	436	49.8
Q9D0J8	Parathymo	7.314	23	2	5	2	101	11.4
Q99L20	Glutathior	7.314	9	2	6	2	241	27.4
E9PW20	Chromatin	7.294	8	1	5	1	203	22.1
Q8K3W3	Protein CA	7.292	3	2	7	2	698	75.7
Q9D8L3	Signal seq	7.283	17	2	4	2	173	19
G3UX45	URB2 ribo	7.283	2	2	11	2	1296	145.6
P97348	Rho-relate	7.27	19	2	3	2	210	23.5
Q91VN1	Zinc finger	7.254	5	2	10	1	368	41.9
G5E8R4	Serine/thr	7.234	2	2	6	2	873	97.8
Q6ZWY3	40S riboso	7.234	23	2	40	1	84	9.5
A2BHP5	Rhombotir	7.233	12	2	5	2	220	24.6
Q9ESJ0	Exportin-4	7.233	2	2	8	2	1151	129.9
P62192	26S protea	7.231	5	2	10	2	440	49.2
Q8BG48	Serine/thr	7.227	4	1	6	1	372	42
G3X8X7	Vacuolar p	7.225	4	3	10	3	839	94.8
P62876	DNA-direc	7.225	30	2	30	2	67	7.6
A0A087W	Basic leuc	7.219	6	3	17	3	451	51.2
Q9JK84	Partitionin	7.215	5	2	22	2	382	42.3
Q9CYX7	RRP15-like	7.195	8	3	14	3	281	31
A0A3B2W	CCAAT/en	7.194	7	3	6	3	461	52.6
Q5XJV7	Histone-ly	7.192	2	2	2	1	1441	157.3
F6VQ81	Tumor pro	7.181	19	2	6	2	161	17.7
Q9CQW1	Synaptobr	7.176	12	3	26	3	198	22.3
Q8BGQ1	Spermatog	7.175	5	2	17	2	491	56.6
F6SIJ4	Transcripti	7.173	10	2	16	2	342	39.2
D3YTR6	Protein sp	7.16	6	2	6	2	476	51.4
Q9Z1Z2	Serine-thr	7.151	5	2	10	2	350	38.4
Q9JL16	Interferon	7.151	6	2	30	2	300	32.5
A2AIV8	Caspase re	7.141	4	2	2	2	536	62.4
Q9D8X2	Coiled-coil	7.138	8	2	8	2	217	25.3
Q6A009	E3 ubiquit	7.137	1	3	5	3	1767	198.8
Q66GT5	Phosphatic	7.133	10	2	24	2	193	21.9
Q6PHZ5	Putative R	7.123	2	1	19	1	887	97
P42337	Phosphatic	7.101	4	3	5	3	1068	124.3
A2AST1	Coiled-coil	7.1	2	2	19	2	1451	164.8

V9GX65	39S riboso	7.094	22	2	8	2	96	11.6
Q9DC04	Regulator	7.09	2	2	7	2	966	106.2
P70392	Ras-specif	7.088	2	2	2	2	1189	135.6
Q9D1F0	CAAX box	7.087	13	2	49	2	113	13.6
Q61169	Transcripti	7.083	4	2	11	2	589	59.3
Q99K23	Ufm1-spe	7.083	6	2	3	2	461	52.5
Q6P8P8	Pulmonary	7.079	9	1	29	1	193	21
P40142	Transketol	7.076	3	1	17	1	623	67.6
O08528	Hexokinas	7.076	3	2	4	2	917	102.5
Q3UC82	DNA ligase	7.07	3	3	4	3	1011	112.6
Q9DAT2	MRG/MOI	7.062	7	1	12	1	204	22.4
Q8K337	Type II ino	7.06	2	2	15	2	993	112.7
Q99N93	39S riboso	7.057	8	2	4	2	251	28.8
A0A140LIZ	26S protea	7.054	5	2	9	2	387	43.5
Q60949	TBC1 dom	7.054	2	2	2	2	1255	141.9
Q5SUF2	Luc7-like p	7.034	6	3	12	3	432	51.4
E9PX53	Serine/thr	7.031	3	2	6	2	934	104.4
Q9CXW3	Calcyclin-b	7.024	14	2	7	2	229	26.5
Q06806	Tyrosine-p	7.023	2	2	8	2	1134	124.5
Q9DBA6	Peroxisom	7.021	5	2	9	2	568	59
E9Q9E4	Cyclin-dep	7.02	8	2	29	1	253	28
G3UZF0	Ubiquitin c	7.017	5	2	3	2	476	53.6
P61202	COP9 signa	7.013	5	2	11	2	443	51.6
O35465	Peptidyl-p	7.007	5	2	17	2	402	43.5
B2RUP2	Protein un	7.003	2	2	8	2	1085	123
P62862	40S riboso	7.002	31	2	121	2	59	6.6
Q9ES52	Phosphatid	7.002	2	2	5	2	1191	133.5
D6RCH5	Transmem	7.001	18	1	1	1	131	14.1
P09023	Homeobox	7	9	2	55	1	224	25.3
Q9CZP3	Zinc finger	6.999	4	2	10	2	541	63.3
A6PWX9	Ubiquinol-	6.994	7	2	5	2	269	31.3
P97855	Ras GTPas	6.993	7	2	11	2	465	51.8
D3YVC9	Graves dis	6.991	10	2	7	2	322	34.9
P41183	B-cell lym	6.988	4	3	9	2	707	78.9
F6UP63	Lysophosp	6.988	13	1	6	1	123	13.4
Q9R233	Tapasin OS	6.987	4	2	9	2	465	49.7
E9Q811	Serine/thr	6.968	6	1	2	1	310	34.6
H3BJD8	WD repeat	6.957	26	1	13	1	61	6.8
F7A043	DNA repai	6.957	4	1	17	1	519	58.8
Q3U829	AP-5 comp	6.952	3	2	5	2	807	89.4
E9QB02	Methionin	6.951	3	2	13	2	910	102.3
Q99LF7	DDB1 and	6.943	4	2	2	2	519	58.1
A0A3Q4EC	pre-mRNA	6.934	8	2	9	2	271	31.4
Q9R1P3	Proteasom	6.926	14	2	3	2	201	22.9
Q925I7	Platelet-de	6.923	6	2	22	2	370	42.8

Q8C2K1	Differentia	6.919	4	2	9	2	630	73.4
Q8CG50	Ras-relate	6.918	12	2	9	1	210	23.2
Q3THK7	GMP synth	6.915	4	2	5	2	693	76.7
Q61398	Procollage	6.894	7	2	5	2	468	50.1
E9Q4X2	UDP-gluco	6.894	2	2	14	1	1504	172.7
Q61114	BPI fold-co	6.881	7	2	4	2	474	52.4
L0N7N1	Kinesin-lik	6.879	2	2	2	1	1674	186.3
P01837	Immunogl	6.873	25	2	12	2	107	11.9
Q5SV80	Unconvent	6.866	3	2	16	2	963	108
A0A5F8M(	Chromodo	6.862	9	2	9	2	385	42.2
Q9JI95	CPN10-like	6.862	22	2	3	2	102	11
E9Q1L8	Zinc finger	6.849	12	2	29	1	384	44.4
Q9JKN1	Zinc transp	6.839	8	2	14	2	378	41.8
E9QM38	Solute car	6.825	3	2	5	2	1206	130.6
Q60631	Growth fa	6.815	9	2	23	2	217	25.2
Q9D8T7	SRA stem-	6.815	17	2	8	2	112	12.6
Q8K0H5	Transcripti	6.807	6	1	6	1	218	21.8
G5E8K5	Ankyrin-3 (	6.802	1	2	8	2	1961	213.9
A0A1L1SV	Choline tra	6.792	5	2	9	2	554	62.6
Q64471	Glutathion	6.783	5	1	11	1	240	27.4
Q9CXT8	Mitochonc	6.777	4	2	6	1	489	54.6
Q921K9	B-cell CLL/	6.777	12	1	3	1	202	22.2
Q61473	Transcripti	6.768	6	2	15	1	419	44.6
F8WGX5	DmX-like p	6.76	1	2	14	2	3025	337.2
F8WI14	Extracellul	6.75	5	2	3	2	558	62.7
Q91VL6	Zinc finger	6.747	9	2	9	1	274	32.3
P97794	ATP-sensit	6.74	5	2	3	2	424	47.9
Q9R257	Heme-binc	6.738	8	1	2	1	190	21.1
Q3UDK1	TRAF-type	6.734	7	2	5	2	580	64.2
Q8VHY0	Chondroiti	6.732	1	2	13	2	2327	252.2
Q3TTA7	E3 ubiquit	6.73	3	2	5	2	982	109
Q8VEK0	Cell cycle c	6.73	7	2	6	2	364	41
Q64343	ATP-bindir	6.727	3	2	3	2	666	74
Q9DCJ7	Aurora kin	6.726	11	2	51	2	200	23.3
P53994	Ras-relate	6.709	8	2	13	2	212	23.5
P27048	Small nucl	6.708	6	2	78	2	231	23.6
G3UZX4	Casein kin	6.69	5	1	21	1	257	29.2
Q8VE73	Cullin-7 OS	6.684	1	1	5	1	1689	192.2
Q9JKY0	CCR4-NOT	6.67	6	1	2	1	299	33.6
A2CF83	RIKEN cDN	6.667	33	2	3	1	109	11
G3X8S5	DnaJ homo	6.666	8	2	4	2	261	29.5
D6RI62	Putative P	6.666	8	2	14	1	403	44.7
A0A0A6YV	Ubiquitin c	6.662	3	2	23	1	915	103.7
D3Z5Q9	Tropomod	6.662	7	1	15	1	184	21
Q91YM2	Rho GTPas	6.656	1	2	10	2	1499	170.3

Q9R1P0	Proteasom	6.655	7	2	21	2	261	29.5
A2A7P4	Zinc finger	6.652	9	2	15	1	283	32.3
Q6PAJ1	Breakpoint	6.645	2	2	8	1	1270	143
Q3TMW1	Coiled-coil	6.64	3	1	2	1	549	62.6
Q8VI94	2'-5'-oligo	6.631	7	2	5	2	511	59.1
Q8C0M0	GATOR co	6.623	3	2	8	2	992	111.7
Q8BG95	Protein ph	6.614	2	2	2	1	976	109
Q9EST5	Acidic leuc	6.607	7	1	11	1	272	31.1
A2ASR8	Polycomb	6.604	10	2	3	2	197	23.1
Q8C8V1	Zinc finger	6.594	2	2	18	2	858	90.7
P46412	Glutathior	6.59	6	1	18	1	226	25.4
Q8BMD8	Calcium-bi	6.578	4	2	5	2	475	52.9
Q8R092	Protein C1	6.574	7	1	2	1	253	28.7
Q8K297	Procollage	6.563	4	2	5	2	617	71
O35638	Cohesin su	6.56	3	2	8	2	1231	141.2
A0A0J9YT)	Nuclear re	6.557	18	1	9	1	88	10.6
O88685	26S protea	6.551	5	2	19	2	442	49.5
A0A2I3BR	Predicted g	6.545	5	2	5	2	388	42.2
E9Q6L8	BTB/POZ d	6.544	6	2	20	2	427	48.3
Q8BVE3	V-type pro	6.527	6	2	16	2	483	55.8
Q8CE04	Calpain inl	6.516	6	2	3	2	704	76.4
A0A140T8	Immunogl	6.508	11	1	102	1	120	13.2
Q9CZ91	Serum res	6.496	7	2	10	2	441	48.7
Q922M7	Ashwin OS	6.494	14	2	6	2	232	26
Q9QUM9	Proteasom	6.49	9	2	7	2	246	27.4
Q9QXX8	Nuclear fra	6.487	5	2	17	2	484	54.7
Q8CIM8	Integrator	6.482	2	2	5	2	964	108.1
Q924T7	E3 ubiquit	6.461	3	2	15	2	1066	119.2
Q9EQ20	Methylma	6.458	8	2	4	2	535	57.9
Q9DCL4	12S rRNA	6.453	3	1	5	1	406	45.3
Q9CZD5	Translatio	6.445	9	2	8	2	276	31.7
F8VPZ9	BRD4-inte	6.443	1	2	9	2	1578	161.3
Q9D8Y8	Inhibitor o	6.437	10	2	3	2	240	27.8
Q05816	Fatty acid-	6.435	13	2	10	2	135	15.1
Q8VE80	THO comp	6.435	10	2	4	2	351	38.7
Q9D3D9	ATP syntha	6.428	8	1	8	1	168	17.6
E9Q9R9	Disks large	6.418	1	2	5	2	1921	214.3
A2AWE9	Serine/thr	6.408	10	2	9	2	281	31.8
Q8VCT4	Carboxyles	6.403	3	2	3	2	565	61.7
Q8R3G9	Tetraspan	6.398	15	2	8	2	235	25.6
P63271	Transcripti	6.391	14	2	41	2	117	13.2
Q9QXN3	Activating	6.376	4	2	9	2	581	66.2
P31938	Dual speci	6.376	6	2	12	2	393	43.4
Q9DC70	NADH deh	6.363	8	2	10	2	224	24.7
P97379	Ras GTPas	6.352	4	1	5	1	482	54.1

P48758	Carbonyl r	6.345	10	2	11	2	277	30.6
P50518	V-type pro	6.344	10	2	5	2	226	26.1
F6U6X3	Dual-speci	6.343	4	2	4	2	725	81.5
Q99LT0	Protein dp	6.33	11	2	19	2	99	11.2
Q9CQS5	Serine/thr	6.33	7	2	4	2	547	62.5
Q9ERI2	Ras-relate	6.328	9	2	7	2	221	25
A2AFP4	RAB9, me	6.326	27	1	14	1	59	6.5
A2AQR4	Zinc finger	6.322	4	2	4	2	676	74
D6RFU9	Synaptoph	6.321	14	2	10	2	79	8.9
A2BH40	AT-rich int	6.314	1	2	8	2	2283	241.9
E9PUN6	A disinteg	6.304	2	2	3	2	1931	215.6
B2RRI2	Protein str	6.292	2	2	4	1	1391	153.8
P12815	Programm	6.281	10	2	3	2	191	21.9
Q9D338	39S riboso	6.277	7	2	15	2	292	33.6
P08551	Neurofilar	6.275	3	2	58	1	543	61.5
Q91Z40	Gbp6 prot	6.27	2	1	14	1	638	72.7
F8VPZ5	DNA excisi	6.262	1	2	4	2	1481	165.9
Q9WUB4	Dynactin s	6.245	9	1	3	1	190	20.7
Q99N57	RAF proto-	6.241	3	2	24	1	648	72.9
Q3UI43	BRISC and	6.238	6	2	9	2	333	36.8
D3Z0C1	SIN3-HDA(	6.237	13	2	3	2	156	17.4
E9Q6Y8	Ubiquitin-	6.23	1	1	8	1	1344	146.2
A2AP82	Transcript	6.227	6	2	8	2	293	32
P21550	Beta-enola	6.225	4	2	5	2	434	47
Q543M9	Non-speci	6.218	7	1	3	1	244	27.4
Q9D4L6	E3 ubiquit	6.218	5	1	6	1	353	39.4
Q91WE6	Threonylca	6.217	4	2	4	2	578	65.2
Q3UNA4	NTF2-rela	6.208	21	2	13	2	142	16.2
Q8VE70	Programm	6.206	9	2	10	2	212	24.7
Q8BHG1	Nardilysin	6.206	2	2	10	2	1161	132.8
D3Z486	Palmdelph	6.205	15	1	9	1	122	14.6
Q8BVP5	Casein kin	6.202	4	2	14	2	415	47.6
Q64267	DNA repai	6.2	5	1	6	1	272	31.4
Q99388	Componer	6.193	9	2	9	1	208	24
A0A0G2JE	Anaphase-	6.185	4	2	11	2	719	80.8
Q80VE5	TBC1 dom	6.184	6	2	4	2	505	59.1
A0A0G2JF	Phosphodi	6.183	2	2	7	2	833	94.4
Q6P5G3	MBT doma	6.183	3	2	5	1	631	70.6
P08249	Malate de	6.181	6	2	3	2	338	35.6
E9Q668	Semaphor	6.173	2	2	8	1	688	78.5
B2RT41	Zinc finger	6.167	1	1	5	1	1992	224.9
P10852	4F2 cell-su	6.165	3	2	9	2	526	58.3
Q9DBG9	Tax1-bindi	6.138	14	1	9	1	124	13.7
Q9DBP5	UMP-CMP	6.137	7	1	11	1	196	22.2
O09046	L-amino-a	6.115	3	1	13	1	630	70.1

Q8BWM0	Prostaglar	6.109	5	1	13	1	384	43.3
Q8VC65	Nurim OS=	6.108	8	1	8	1	262	29.4
Q9DBG5	Perilipin-3	6.101	4	1	5	1	437	47.2
Q80YW9	Peptidylpr	6.1	3	2	6	2	756	82.2
Q8BSM6	Lipopolysa	6.098	3	2	4	2	758	85.4
P81269	Cyclic AMF	6.097	6	2	45	2	269	29.2
Q8BIG4	F-box only	6.096	6	2	16	2	368	41
D3Z2U5	Inactive se	6.094	14	1	8	1	175	19.4
Q3UH06	Ras-respo	6.093	1	2	12	2	1700	184
Q8C4U3	Secreted f	6.09	5	1	10	1	314	35.4
Q99PL6	UBX doma	6.089	4	1	3	1	442	49.8
A0A0G2JF	Protein FA	6.088	17	2	8	2	109	13
Q80WW9	DDRGK do	6.088	5	1	3	1	315	36
Q6P1H6	Ankyrin re	6.082	3	2	10	2	964	106.1
Q8BHN3	Neutral al	6.077	3	2	2	2	944	106.8
A2BHN6	F-box only	6.073	3	1	12	1	361	40.9
Q8BSI6	R3H and c	6.065	4	2	13	1	488	54.1
P00493	Hypoxanth	6.064	13	2	9	2	218	24.6
Q7TMC8	L-fucose k	6.053	1	1	4	1	1090	119.2
Q9EPT5	Solute car	6.052	3	2	9	2	643	70.1
A2A6Q2	Cdc42 effe	6.047	9	2	4	2	193	21
H3BLI5	Coiled-coil	6.047	7	2	15	2	241	27.1
G3V001	Compleme	6.045	9	2	10	2	324	34
Q3TL44	NLR family	6.045	2	2	15	2	975	107.8
Q4VBD9	GDNF-indu	6.043	3	2	2	2	706	79.5
A0A087W	Cytoplasm	6.042	23	2	11	1	132	15.2
P15066	Transcript	6.04	8	2	20	2	341	34.9
P29037	TATA-box-	6.032	13	2	2	2	316	34.7
P83870	PHD finger	6.018	21	2	18	2	110	12.4
Q9QXT5	Epidermal	6.013	5	1	14	1	275	29.7
D6REV6	F-box only	6.006	11	2	2	2	166	18.3
Q3TIU4	2',5'-phos	6.002	5	2	4	2	608	67.5
G5E8J4	Tyrosine-p	5.995	2	2	3	1	1176	133.4
Q80X85	28S riboso	5.99	5	1	10	1	242	28
A2AL50	Alkylglycer	5.98	2	1	6	1	671	74.3
A0A0R4J0	Leucine-ric	5.978	2	1	9	1	602	68
A2A816	Parkinson	5.969	13	1	16	1	125	12.7
F6Y6L6	Predicted	5.968	6	2	10	2	278	29.9
P52293	Importin s	5.968	5	2	11	2	529	57.9
Q9DBM1	G patch dc	5.965	2	2	4	2	930	102.9
E9PYD6	Beclin-1 O	5.964	12	2	2	2	277	32.1
B9EKF4	Zinc finger	5.961	3	2	3	2	885	99.4
A2AEW0	Transcript	5.956	3	2	2	1	537	57.7
P97501	Dimethyla	5.954	5	2	11	2	534	60.5
Q99K28	ADP-ribosy	5.942	4	2	5	2	520	56.6

Q9Z2R6	Protein un	5.937	11	2	6	2	240	27
B9EIX2	AW55546	5.934	2	2	2	1	1539	166.8
Q07231	Zinc finger	5.933	3	2	14	1	555	63
P05622	Platelet-de	5.933	1	2	24	1	1098	122.7
Q8K124	Pleckstrin	5.933	4	2	2	2	495	53.8
Q9D892	Inosine tri	5.928	8	1	3	1	198	21.9
Z4YJE9	Queuine tI	5.927	6	1	14	1	345	38.5
A2AKX3	Probable h	5.921	1	2	6	2	2646	297.4
Q80UP5	Ankyrin re	5.919	3	1	7	1	588	67.1
F2Z4B3	Cyclin-dep	5.919	21	2	9	2	87	9.6
P70303	CTP syntha	5.914	3	2	11	2	586	65.5
Q6P9R2	Serine/thr	5.913	3	2	17	2	527	58.2
D3YVU8	ETS transl	5.909	16	1	7	1	146	17.3
P08228	Superoxide	5.901	8	1	4	1	154	15.9
S4R1J5	2-5A-depe	5.897	3	2	5	2	651	73.5
Q7TPE5	Probable F	5.889	7	2	6	2	306	35
Q8K183	Pyridoxal k	5.88	5	1	4	1	312	35
Q8R050	Eukaryotic	5.875	3	2	11	2	636	68.6
E9Q8D0	DnaJ homo	5.873	3	2	16	2	531	61.7
D3Z687	RIKEN cDN	5.872	14	2	2	1	191	20
Q8CE90	Dual speci	5.858	3	2	10	2	535	59.3
F6W623	[Histone H	5.847	1	1	2	1	2014	219.9
O70126	Aurora kin	5.841	7	2	2	2	345	39.4
Q59J91	Polypeptid	5.841	3	1	1	1	607	69.5
Q8R2U0	Nucleopor	5.832	6	2	5	2	360	39.7
P98086	Compleme	5.829	6	1	9	1	245	26
Q80UJ7	Rab3 GTPa	5.829	2	1	7	1	981	110.1
Q3UJV1	Centrosom	5.818	4	2	3	2	511	57.3
E9Q1M6	Ankyrin re	5.806	1	2	2	2	2485	263.2
Q9Z2Z6	Mitochond	5.802	6	2	10	2	301	33
Q9CZ96	Zinc finger	5.796	7	1	2	1	217	24.6
P27790	Major cent	5.796	6	2	2	2	599	65.3
Q921I9	Exosome c	5.795	6	1	2	1	245	26.2
Q9CTY5	Calcium u	5.792	5	2	3	2	523	59.8
D3Z0I8	Transmem	5.785	6	1	12	1	213	22.7
Q3U367	4-trimethy	5.785	4	2	8	2	518	55.9
Q2VPQ9	Chromatin	5.776	12	2	10	2	191	21.6
Q61687	Transcripti	5.775	1	2	2	2	2476	278.4
Q6A0A2	La-related	5.775	5	2	5	2	741	81.6
Q91W96	Anaphase-	5.77	3	2	5	2	807	91.6
Q8R317	Ubiquilin-1	5.767	3	1	7	1	582	61.9
D3YYC3	Histone-ly	5.765	1	1	12	1	1308	144.5
Q8BG98	RIKEN cDN	5.755	4	1	7	1	337	38
Q9QYC7	Vitamin K-	5.748	4	2	6	2	757	87.1
Q91YS7	Dual-speci	5.745	5	2	17	2	400	44.3

Q8BGB5	LIM domai	5.743	16	2	7	2	128	14.2
B2RY04	Dedicator	5.742	1	2	2	1	1868	214.3
Q9D7F7	Charged m	5.739	8	2	7	1	232	26.3
Q64514	Tripeptidy	5.737	2	1	1	1	1262	139.8
Q8BGZ3	DDB1- anc	5.725	4	2	2	2	453	50.4
Q62086	Serum par	5.722	5	1	9	1	354	39.6
O35955	Proteasom	5.715	8	2	11	2	273	29
Q8BHG9	CGG triple	5.696	11	1	14	1	167	18.8
Q3TLI0	Trafficking	5.695	2	2	3	2	1258	141.4
A0A498W	Thioredoxi	5.694	7	1	5	1	292	32.7
P35918	Vascular e	5.692	2	2	20	1	1345	150.4
Q9CZW5	Mitochonc	5.687	4	2	8	2	611	67.5
P42232	Signal trar	5.686	3	2	5	2	786	89.9
E9PV24	Fibrinoger	5.682	2	2	6	2	789	87.4
Q8BHJ5	F-box-like/	5.68	8	2	2	2	514	55.6
Q8BJ34	Meiosis re	5.676	1	2	19	2	1730	191.9
Q8R0Y8	Mitochonc	5.676	5	2	9	2	318	35.2
O89023	Tripeptidy	5.662	2	1	10	1	562	61.3
A0A1B0GF	RIKEN cDN	5.659	2	2	7	2	1767	192.6
Q9JJ00	Phospholip	5.651	5	2	6	2	328	35.9
Q3UQ17	Zinc finger	5.651	3	1	5	1	673	74.2
Q80U38	Protein KH	5.637	4	2	7	2	671	74.5
Q99L28	Probable r	5.633	7	1	17	1	163	19.6
Q5SPX8	TBC1 dom	5.632	5	2	20	2	534	59.2
Q8BGC4	Prostaglar	5.632	3	1	12	1	377	40.5
Q8BNW9	Kelch repe	5.624	3	2	14	2	633	67.9
Q920A7	AFG3-like	5.622	3	2	3	2	789	87
G3X9J0	Signal-ind	5.613	1	2	7	1	1776	194.9
Q8BMI0	F-box only	5.613	1	2	6	2	1194	133.8
D3Z2B4	Xenotropic	5.609	2	1	5	1	630	74
A0A0G2JD	Adhesion (	5.608	1	1	14	1	1403	157
Q9JHE7	Protein TS	5.607	7	1	1	1	317	33.5
A0A1L1SR	Alpha-crys	5.605	13	2	12	2	130	15.4
A0A1Y7VJ	Apoptosis-	5.597	2	2	5	1	964	104.4
A0A0U1RF	Nucleolysi	5.596	7	1	6	1	244	26.9
Q9R0B7	Zinc finger	5.596	4	1	8	1	294	32.7
Q6P9J9	Anoctamir	5.594	2	1	1	1	911	106.2
P10605	Cathepsin	5.593	4	1	2	1	339	37.3
Q3UHD2	Glucose-fr	5.586	6	2	12	2	390	43.3
Q8BG32	26S protea	5.583	6	2	15	2	422	47.4
P98083	SHC-transf	5.582	3	2	6	2	579	62.6
O35685	Nuclear m	5.58	8	2	2	2	332	38.3
Q9CUX1	52 kDa rep	5.575	2	2	13	2	758	87
Q91WM3	U3 small r	5.572	4	2	8	2	475	52.1
Q3UHH0	Calcium-tr	5.565	1	1	6	1	1194	132

A0A087W	CMP-N-ac	5.563	6	1	4	1	266	30.2
Q8QZY9	Splicing fa	5.547	5	1	3	1	424	44.3
Q91VS7	Microsoma	5.544	12	1	3	1	155	17.5
Q8VDQ8	NAD-depe	5.541	5	2	2	2	389	43.2
D3Z286	Transmem	5.536	3	1	12	1	447	49.4
Q9DBF7	Pre-mRNA	5.532	5	2	4	2	416	48.8
Q61846	Maternal e	5.527	3	2	3	2	643	72.7
P40646	Transcripti	5.512	4	1	19	1	380	41.5
A0A0R4J0	Glutaminy	5.511	6	2	7	2	362	41.1
D6RDS0	RUS famil	5.501	3	1	12	1	347	36.8
O88792	Junctional	5.501	10	2	3	2	300	32.4
Q5FW53	Myosin-bi	5.501	3	1	8	1	355	38.7
O70583	E3 ubiquit	5.499	4	2	32	2	680	76.1
A0A3Q4L2	Poly(A) RN	5.498	7	1	3	1	338	37.4
Q6NZQ6	Zinc finger	5.492	7	1	6	1	180	20.9
D3Z1U9	NADH deh	5.49	5	2	2	2	385	41.8
Q5IRJ6	Zinc transp	5.472	5	2	7	2	567	62.8
Q9CPN7	RIKEN cDN	5.471	8	1	20	1	247	26.5
Q9DBC3	Cap-specif	5.468	2	1	4	1	837	95.6
Q7TN98	Cytoplasm	5.463	3	1	3	1	729	80.1
Q8CH18	Cell divisic	5.462	3	2	2	2	1146	132
P15327	Bisphosph	5.452	7	1	12	1	259	30
P31240	Platelet-de	5.452	8	2	10	2	241	27.4
Q9DB91	Mediator c	5.446	7	1	2	1	199	21
Q99K74	Mediator c	5.441	2	1	1	1	987	109.9
Q9WTZ2	Membrane	5.434	2	2	2	2	1052	117.4
P23188	Furin OS=I	5.427	2	1	11	1	793	86.7
Q8VCG9	Regulatory	5.415	8	2	3	2	231	24.8
E9Q309	Centrosom	5.412	1	2	4	2	3095	346.2
A0A1D5RN	Cytosolic i	5.407	24	1	18	1	91	9.4
Q8R480	Nuclear pc	5.394	4	2	9	2	656	74.7
Q9Z0X4	cGMP-inhi	5.391	2	1	2	1	1141	124.4
Q3U0V2	Tumor nec	5.375	10	2	6	2	310	34.6
F6UFG6	Acidic leuc	5.374	12	1	1	1	138	15.4
P49615	Cyclin-dep	5.371	5	2	29	1	292	33.3
F7CBX2	Uridine/cy	5.369	11	2	4	2	208	23.4
E9QN70	Laminin su	5.369	1	2	8	2	1834	202.3
Q80ZJ8	EF-hand ca	5.367	4	1	2	1	394	44.4
O54791	Transcripti	5.36	10	2	15	2	156	16.9
Q61074	Protein ph	5.348	12	2	3	2	542	58.7
Q8VCM8	Nicalin OS	5.348	3	2	4	2	563	62.9
P56960	Exosome c	5.34	3	2	7	2	887	100.9
B2RXC2	Kinase OS=	5.333	2	2	5	2	942	102.6
G3X952	Zinc finger	5.331	3	2	15	1	960	110.4
Q9Z2Q6	Septin-5 C	5.331	4	1	11	1	369	42.7

Q5FWI3	Cell surfac	5.329	2	2	8	2	1383	153.7
Q8C4V4	F-box/LRR	5.327	9	2	2	2	428	48.7
D3YYC2	Dr1-associ	5.326	7	1	12	1	162	17.6
Q8CG73	Protein fa	5.324	2	2	4	2	1264	144.9
F8WGD9	1-acyl-sn-g	5.323	4	1	2	1	291	33.8
Q99M15	Proline-se	5.323	5	1	10	1	334	38.9
Q8BMJ2	Leucine--t	5.313	1	1	3	1	1178	134.1
D3Z7C6	Prostaglar	5.313	13	2	3	2	130	15
Q91WM0	Zfp617 pro	5.307	4	2	5	2	559	64.2
A0A140LI7	CREB/ATF	5.303	16	2	7	2	125	13.6
H3BKP8	RIKEN cDN	5.303	1	2	5	2	2027	218.8
Q99LD4	COP9 signa	5.291	3	2	18	2	471	53.4
Q61151	Serine/thr	5.284	3	1	3	1	467	54.7
Q80VW7	Microtubu	5.284	2	2	3	2	1404	153
P54728	UV excisio	5.283	4	2	7	2	416	43.5
F8VPM7	ELKS/Rab6	5.283	2	2	7	2	1120	128.3
Q80ZS3	28S riboso	5.27	9	2	4	2	200	23.4
Q6PIJ4	Nuclear fa	5.268	1	1	6	1	1296	138.7
Q69ZT9	TBC1 dom	5.259	4	2	4	2	766	84.8
Q9WTR5	Cadherin-1	5.258	2	1	1	1	714	78.1
Q5DU37	Zinc finger	5.257	1	2	7	2	2529	282.8
A0A0G2JD	Ubiquitin c	5.256	3	1	5	1	410	46
Q6PAR0	Kelch dom	5.246	3	1	4	1	439	49
Q8N7N5	DDB1- and	5.234	3	2	11	2	591	66
Q3UJB0	Splicing fa	5.232	2	2	13	2	878	98.1
Q99JH8	ER lumen	5.23	9	2	14	2	212	24.5
D3YW09	Splicing fa	5.23	7	1	2	1	194	22.1
Q9D517	1-acyl-sn-g	5.229	5	2	10	2	376	43.3
D3YZG8	Probable b	5.224	5	1	12	1	338	36.4
Q99LB2	Dehydroge	5.218	8	2	5	2	279	29.9
A0A1B0GS	Proteasom	5.212	7	2	12	2	238	26.5
P12787	Cytochrom	5.207	16	2	6	2	146	16.1
P58854	Gamma-tu	5.206	3	2	5	2	905	103.4
Q6PFY1	F-BAR and	5.205	2	1	1	1	688	76.2
B0QZN5	Synaptobr	5.198	15	2	7	2	163	17.9
E9QL25	[Histone H	5.195	1	1	3	1	1303	149
Q3UHC1	Rap guani	5.186	2	2	3	2	1224	136.4
Q3TIR3	Synembryn	5.183	4	2	2	2	530	59.8
Q8BYZ1	ABI gene f	5.163	4	1	3	1	367	39.1
F8WHU7	Cohesin su	5.154	2	2	8	2	1221	140
Q9ES70	Serine/thr	5.138	5	2	11	1	313	35.7
A2AKB9	DDB1- and	5.138	5	2	7	2	566	61.5
A0A494BB	Peroxisom	5.13	9	2	3	2	257	27.4
A2AKA9	Bromodon	5.129	3	1	3	1	682	75.3
A0A140LH	Kinase sup	5.128	4	2	9	2	432	47.7

Q3U3K9	Lipid dropl	5.127	3	1	2	1	439	49.3
P55200	Histone-ly:	5.127	1	2	5	2	3966	429.4
O35205	Granzyme	5.117	11	2	6	2	263	29.2
Q80WC3	Trinucleoti	5.113	1	1	1	1	2878	307.4
Q9CQ26	STAM-binc	5.109	5	1	5	1	424	48.5
Q6PD28	Serine/thr	5.108	5	2	4	2	497	57.3
Q8CBY1	Protein Sm	5.107	2	1	7	1	711	78.3
A0A1L1SR	Kinesin-lik	5.107	4	2	2	2	563	64.1
Q8CDC7	Zinc finger	5.099	3	1	1	1	459	49.5
Q8K4X7	1-acyl-sn-g	5.094	5	2	3	2	378	43.8
Q8C0Q9	Rap guani	5.094	2	1	1	1	814	93.7
O88852	Testis-spe	5.093	4	1	13	1	379	43
Q505D7	Optic atro	5.092	11	2	6	2	179	20.1
Q8BGC0	HIV Tat-sp	5.091	2	2	6	2	757	86.2
Q9QZ85	Interferon-	5.088	4	1	6	1	413	47.5
P98078	Disabled h	5.083	4	2	7	2	766	82.3
Q8K355	Leukotrien	5.082	15	1	2	1	113	12.8
Q80W41	Condensin	5.079	1	1	7	1	1223	139
Q6PER3	Microtubu	5.073	6	2	9	1	281	31.9
A2AVR9	Dynein ligl	5.069	12	1	5	1	104	11.9
A0A5F8MI	Exocyst co	5.069	3	2	3	2	811	94
Q62312	TGF-beta r	5.068	4	2	4	2	592	67.1
Q9JI78	Peptide-N(	5.067	2	1	5	1	651	74.2
E9Q1X8	Voltage-de	5.065	2	2	6	2	1091	123.1
Q8K2Y7	39S riboso	5.064	4	1	12	1	252	29.7
Q8R516	E3 ubiquit	5.064	3	2	3	2	973	105.9
Q8C6G1	Cilia- and f	5.06	8	2	3	2	249	28.2
A0A0G2JF	Far upstre	5.059	3	2	9	1	654	68.8
Q9Z1R9	Protease, s	5.057	8	1	13	1	246	26.1
Q9D1Q6	Endoplasm	5.056	3	1	4	1	406	46.8
Q91VY5	Lysine-spe	5.043	2	1	11	1	1086	121.5
Q52KG5	Kinesin-lik	5.041	1	1	4	1	1881	196.2
Q8CD91	SPARC-rel:	5.039	3	1	3	1	447	49.9
H3BJJ9	Zinc finger	5.019	10	2	10	2	241	25.4
P06339	H-2 class I	4.998	5	2	5	2	357	40.8
Q8K4F5	Protein AB	4.996	4	1	5	1	307	33.5
Q922M5	Cell divisic	4.995	4	2	3	2	438	50.2
A3KG38	NF-kappa-	4.993	6	1	6	1	265	30.7
T1ECW4	RNA-bindi	4.992	9	1	7	1	191	20.7
Q9D5V5	Cullin-5 OS	4.992	3	2	4	2	780	90.9
P54116	Stomatin (	4.992	5	2	18	2	284	31.4
F8WJ43	Merlin (Fra	4.986	6	2	7	2	521	61
Q8BML1	[F-actin]-r	4.982	2	1	4	1	960	110
P11031	Activated	4.969	9	1	26	1	127	14.4
Q8R5A6	TBC1 dom	4.969	6	2	6	2	516	59.3

P56528	ADP-ribosy	4.969	4	1	2	1	304	34.4
P40336	Vacuolar p	4.969	6	2	7	1	327	38.1
A0A0A0M(	Mitogen-a	4.963	1	2	7	1	1291	142.9
D6RHT6	RING-type	4.963	2	2	3	2	1135	130.6
Q8R5A3	Amyloid be	4.961	3	1	2	1	670	74.3
A2ALV3	Endophilin	4.956	7	2	4	1	431	48.3
P41233	Phospholip	4.954	1	2	5	2	2261	253.7
Q8BJ25	THAP dom	4.954	13	1	1	1	218	24.6
A0A1L1SR	AMP deam	4.953	2	2	9	2	775	89.5
J3JS28	Protein W	4.951	5	1	3	1	365	40.4
A0A2R8VH	Tudor-inte	4.95	9	2	11	2	199	22.1
Q91WA4	CXXC-type	4.945	3	1	5	1	317	32.8
Q8BFY6	Peflin OS=	4.936	4	1	8	1	275	29.2
Q9JLI8	Squamous	4.922	2	2	8	2	962	109.6
Q3TYA6	M-phase p	4.918	2	2	6	2	858	97.4
Q8R2M2	Deoxynucl	4.918	2	1	2	1	758	84.2
Q8BZR9	Nuclear ca	4.904	3	2	6	2	615	70
P51863	V-type pro	4.904	5	2	3	2	351	40.3
Q64737	Trifunction	4.904	3	2	2	2	1010	107.4
Q91VC7	Protein ph	4.902	16	1	1	1	147	16.6
P51437	Cathelicidi	4.902	10	1	6	1	172	19.4
Q8R2V3	Zinc finger	4.887	2	1	17	1	986	114.7
S4R2J8	Bridging ir	4.881	3	1	3	1	473	50.9
Q8VCG3	WD repeat	4.88	7	2	2	2	384	42.6
P53986	Monocarbo	4.878	5	2	4	2	493	53.2
E9PXC2	Upstream	4.876	7	1	10	1	215	24
P70188	Kinesin-as	4.876	3	2	3	2	793	91.2
Q61193	Ral guanin	4.875	2	2	5	2	778	83.8
G3UYP4	Potassium	4.866	2	2	8	2	823	90
Q924X2	Carnitine (	4.86	4	2	3	2	772	88.2
A0A0G2JD	Glutamate	4.855	16	1	10	1	98	10.9
O88952	Protein lin	4.847	6	1	17	1	197	21.8
Q8BZQ2	Cysteine-ri	4.846	7	2	3	2	495	55.4
Q3U381	Zinc finger	4.844	3	1	4	1	531	59
Q8BIR2	Protein as	4.836	3	1	5	1	672	76.3
O35099	Mitogen-a	4.831	1	2	7	1	1380	154.4
B8JJ56	Tripartite	4.831	3	1	4	1	394	45.5
F8WJE3	KAT8 regu	4.83	3	1	1	1	452	50.5
Q6PGG2	GEM-inter	4.825	3	2	2	2	971	107.5
Q9Z2U0	Proteasom	4.822	7	2	2	2	248	27.8
Q99JZ4	GTP-bindir	4.817	6	1	7	1	198	22.4
B2Z894	6-phospho	4.815	4	2	5	2	374	43.5
Q99LC5	Electron tr	4.811	7	2	6	2	333	35
A2AJG0	Ribonuclea	4.799	7	2	6	2	280	31.2
D3YTV2	Fizzy-relat	4.795	9	2	3	2	404	44.8

Q8K215	LYR motif-	4.79	11	1	4	1	91	10.8
Q5SVP0	RIKEN cDN	4.768	2	2	15	1	844	95.5
A0A0A6YV	N-alpha-a	4.761	2	2	5	2	815	95.2
Q3TE00	Mediator c	4.76	2	1	2	1	766	83.7
A0A0J9YU	Cytoplasm	4.759	16	2	12	1	142	16.2
B1AYJ9	Obg-like A	4.752	4	1	7	1	281	31.7
Q9QZS2	E3 ubiquit	4.747	9	1	6	1	194	21.9
Q9CQ39	Mediator c	4.742	13	1	2	1	144	15.6
Q8BHE8	m-AAA pro	4.737	5	1	1	1	291	33
Q80UU9	Membrane	4.735	8	2	16	1	217	23.3
Q64310	Surfeit loc	4.733	5	1	13	1	269	30.4
A0A0N4SV	Zinc finger	4.733	3	2	3	1	651	69.7
A0A0A0M	Actin-relat	4.732	2	1	13	1	608	68.1
A0A338P6	Translatio	4.73	8	2	3	2	303	34.4
A2A6A1	G patch do	4.73	2	2	4	2	1505	164.9
A2RSY1	KAT8 regu	4.725	2	1	6	1	903	96.1
Q9CQD4	Charged m	4.717	9	2	9	2	199	22.1
Q9D823	60S riboso	4.714	15	2	35	2	97	11.1
Q5SSH7	Zinc finger	4.71	1	2	6	2	2924	328.1
Q5SYL3	Protein KIA	4.709	1	1	10	1	2234	254.3
Q8C1Q6	Small inte	4.701	14	1	3	1	80	9.7
Q6P3Z8	Histone ac	4.698	3	2	2	2	829	93.3
Q99P21	Adenine D	4.694	4	1	1	1	515	57.7
Q99J09	Methyloso	4.687	5	1	6	1	342	36.9
Q9CQP0	39S riboso	4.682	18	1	4	1	65	7.4
Q3UQL6	Zinc finger	4.682	7	1	7	1	416	46.8
Q8K349	GTPase IM	4.681	10	2	9	2	305	34.1
Q9JHS9	Spliceosor	4.679	5	1	8	1	229	26.6
Q91X84	CREB-regu	4.678	3	1	3	1	619	67
Q62504	Msx2-inter	4.677	1	2	2	2	3644	398.5
J3QMC5	Midasin O	4.673	0	2	4	2	5582	629.2
Q91VW5	Golgin sub	4.67	1	1	2	1	2238	257.4
Q65Z40	Wings apa	4.668	2	2	3	2	1200	134
E9Q317	18 kDa Sin	4.661	9	2	21	2	172	19.6
P58742	Aladin OS=	4.646	3	2	4	2	546	59.4
Q8VDF2	E3 ubiquit	4.643	3	2	3	2	782	88.2
A0A494B9	Predicted g	4.638	20	1	10	1	80	9.4
Q7TQJ8	Wilms tun	4.637	3	1	4	1	398	42.2
Q99LI5	Zinc finger	4.635	1	1	5	1	893	96.6
O70481	E3 ubiquit	4.63	1	2	3	2	1757	200.1
Q80X73	Protein pe	4.629	3	1	6	1	385	43.3
Q9EQI5	Chemokine	4.627	10	1	8	1	113	12.2
Q3TYS2	Cytochrom	4.625	5	1	4	1	187	20.9
Q9WTW4	Transcript	4.617	5	2	2	1	317	35.1
E9Q3G5	ADP-ribosy	4.615	3	1	2	1	366	40.8

Q8BJ71	Nuclear pc	4.614	2	2	2	2	819	93.2
A2AMN2	BEN doma	4.608	5	1	15	1	401	44.9
A0A140LJ(	Microsom:	4.584	13	2	13	2	123	14.2
A0A0R4J0:	Pyridoxal-(	4.583	4	2	3	2	789	87.4
E9Q442	Ubiquitin (	4.581	3	1	5	1	547	62.6
Q5PRF0	HEAT repe	4.58	1	1	3	1	2038	219.8
A0A5F8MI	F-actin mc	4.579	1	2	5	1	1968	220.6
Q8BKX6	Serine/thr	4.577	1	1	3	1	3658	409.5
Q9CXU1	Mediator (	4.571	24	2	8	2	131	15.8
Q9R1P1	Proteasom	4.566	9	1	3	1	205	22.9
Q8BVY4	SPT3, SAG	4.549	9	2	2	2	374	41.2
Q60610	Rho guani	4.548	1	2	3	2	1591	177.4
A1L3T7	RIPOR fam	4.546	2	1	3	1	938	104.1
B2RXS4	Plexin-B2 (	4.545	2	2	2	2	1842	206.1
A2AI08	Taperin OS	4.533	4	2	2	2	749	80
Q8VCI5	Peroxisom	4.532	6	1	6	1	299	32.7
Q9CR56	NF-kappa-	4.528	9	2	3	2	191	21.5
F6QSC4	Macrophag	4.525	5	1	2	1	374	42.6
B2RXM6	Ly6g6f pro	4.522	8	1	1	1	300	33.4
A0A1L1SQ	Rab effect	4.515	2	1	4	1	539	60.2
E9QAX7	Exportin-2	4.512	4	1	5	1	415	48
E9PUV9	Caspase re	4.51	1	1	6	1	1070	119.1
S4R225	WD repeat	4.51	4	1	5	1	393	43
A0A494BA	Vacuolar p	4.51	16	1	7	1	104	11.9
Q9D198	Pre-mRNA	4.503	5	1	4	1	242	28.7
Q3UPD8	SH3 doma	4.493	2	1	1	1	1228	138.3
A2AG30	DnaJ homo	4.49	3	1	16	1	382	43
Q3V3Y6	Kinesin-lik	4.485	4	2	2	1	802	91
Q9D8M7	PHD finger	4.476	4	2	2	2	497	55.8
Q99KQ4	Nicotinam	4.468	4	1	14	1	491	55.4
Q8K4L4	Protein PC	4.467	5	2	2	2	587	67.7
P35235	Tyrosine-p	4.461	2	1	2	1	593	68
Q9D8W5	26S protea	4.458	4	1	4	1	456	52.9
A0A0U1RF	Protein-se	4.447	19	1	2	1	100	11.3
E9PV86	Multiple C	4.443	1	1	3	1	951	106.7
Q61194	Phosphatic	4.439	1	1	4	1	1686	190.6
P58022	Lysyl oxida	4.439	2	1	3	1	776	86.9
P0DOV1	Interferon	4.437	4	2	11	1	425	46.9
Q9WVG6	Histone-ar	4.437	3	1	3	1	608	65.8
Q673H1	Tumor sup	4.431	12	2	3	2	205	22.7
Q9QY23	Plakophilir	4.423	2	2	5	2	797	87.3
Q3UGP9	Leucine-ric	4.419	4	1	2	1	366	40.1
Q9CQF8	Ribosomal	4.415	13	1	8	1	102	11.9
A0A0U1RF	Tyrosine-p	4.413	2	1	1	1	820	93.5
E9PY39	Predicted g	4.412	4	2	25	2	371	42.1

A0A0G2JE	Bone morp	4.407	3	1	5	1	443	50.1
A0A0U1RF	Selenoprot	4.407	11	1	13	1	187	21.2
O70333	Cysteine-ri	4.403	17	1	4	1	101	11.3
Q922Q1	Mitochond	4.401	4	2	8	2	338	38.2
Q06185	ATP syntha	4.4	17	1	3	1	71	8.2
Q3U182	CREB-regu	4.396	3	1	4	1	692	73.2
Q80SZ7	Guanine n	4.393	24	2	11	2	68	7.3
Q921J2	GTP-bindir	4.392	8	2	9	2	184	20.4
Q6Y685	Transform	4.39	2	2	2	2	774	83.9
E9Q197	Glyoxalase	4.384	10	2	3	2	279	31.2
Q9Z120	tRNA (gua	4.382	9	1	4	1	268	30.6
Q8BM55	Transmem	4.379	4	2	2	2	687	76.4
B2RSU6	Cingulin-li	4.379	1	2	4	1	1297	148.1
E9Q9S3	U3 small r	4.364	2	2	3	1	780	88.6
P60853	Leucine zi	4.35	3	2	4	1	599	67.2
Q61733	28S riboso	4.341	4	2	5	2	384	43.9
Q7TQ48	Sarcalume	4.341	4	1	1	1	472	54.3
G3X9Z4	PCF11 clea	4.336	1	1	2	1	1553	172.6
E9PZM4	Chromodo	4.326	1	2	3	1	1827	210.7
Q69ZX6	ATPase M	4.308	2	2	4	2	1030	117.3
P39428	TNF recep	4.302	3	1	5	1	409	45.4
Q8BH58	TIP41-like	4.3	7	1	3	1	271	31.2
Q3US59	Cyclic AMF	4.291	4	2	18	2	414	44.8
A0A087W	Eukaryotic	4.285	8	1	9	1	184	20.1
Q06180	Tyrosine-p	4.285	7	2	5	2	406	47.3
Q9R0E1	Multifunct	4.284	3	1	3	1	741	84.9
A0A0N4SV	WASH con	4.272	2	1	6	1	900	98.5
Q62470	Integrin al	4.272	1	1	5	1	1053	116.7
Q9CZ57	5-methylc	4.27	5	2	2	2	381	42.8
Q9EP89	Serine bet	4.256	2	1	7	1	551	60.7
E9Q3P4	Centromer	4.255	1	2	2	2	2997	342.3
Q60760	Growth fa	4.251	3	2	3	1	621	70.5
F6RGI3	Ligand-dep	4.242	1	1	11	1	1242	136.1
Q5SS87	PHD finger	4.238	5	1	1	1	278	29.3
B2RQG2	PHD finger	4.238	1	2	3	2	2025	225.4
Q9Z0H3	SWI/SNF-	4.238	7	1	5	1	385	44.1
Q9EQE2	Protein-se	4.234	6	1	14	1	323	35.9
F6TKU5	NMDA rec	4.229	4	2	2	2	509	57.4
Q8CFT2	Histone-ly	4.228	1	2	17	1	1985	215.2
Q80X91	Protein FA	4.226	8	1	10	1	271	28.7
Q9CXI0	2-methoxy	4.225	5	1	7	1	327	37.3
P83741	Serine/thr	4.223	1	2	5	2	2377	250.8
E9QKD9	General tr	4.223	3	2	2	2	553	62.4
Q8VD62	UPF0696 p	4.212	4	1	2	1	298	31.8
Q3UYC0	Protein ph	4.212	3	1	8	1	513	56.3

A0A1L1SS	Rho GTPas	4.208	7	1	4	1	180	20.8
Q9CSH3	Exosome c	4.207	1	1	2	1	958	108.8
A0A2I3BR	Triple func	4.206	1	1	3	1	1849	211.5
A0A286YD	mRNA-dec	4.203	3	1	1	1	419	45.7
Q8K4Q8	Collectin-1	4.203	2	1	2	1	742	81.3
Q9ERH4	Nucleolar	4.203	8	1	1	1	427	48.5
A0A0R4J1	Menin OS=	4.196	4	1	2	1	617	68
A0A0A6YX	Protoporpl	4.193	3	1	4	1	389	41.2
M1VPI1	Peroxisom	4.189	4	1	1	1	475	54.5
Q8BYW1	Rho GTPas	4.182	2	2	4	1	648	73.3
G5E8B1	Tyrosine-p	4.176	1	2	4	2	2451	270.1
D3YTL5	Macrophag	4.168	4	1	3	1	254	28
B1ART2	Vacuolar p	4.164	0	1	1	1	4390	490.7
Q8BJ05	Zinc finger	4.163	2	1	2	1	735	82.4
Q62087	Serum par	4.163	3	1	3	1	354	39.3
Q6PFX9	Poly [ADP-	4.159	1	2	3	2	1320	140.9
Q8K274	Ketosamir	4.152	6	1	13	1	309	34.4
Q8BU88	39S riboso	4.149	8	2	7	2	206	23.8
Q8BXC0	Prostacycl	4.149	3	1	4	1	509	57.7
Q6DFX2	Anthrax to	4.142	3	1	1	1	487	53.2
P39429	TNF recep	4.135	2	1	7	1	501	56
Q8CAK3	Shiftless a	4.115	4	1	3	1	290	33
Q6DVA0	LEM doma	4.114	3	1	7	1	511	57.5
S4R2T5	Ubiquitin-l	4.113	3	2	3	2	671	74.5
P17932	Putative 60	4.081	7	1	8	1	135	16
Q9D787	RING-type	4.077	3	2	4	2	521	59
Q3U276	Succinate	4.072	13	1	2	1	118	13.1
Q9D7H4	Arginine va	4.072	6	1	6	1	142	15.8
Q91WC0	Actin-histi	4.07	4	1	1	1	594	67.1
A0A1W2P	Ras-relate	4.069	12	1	6	1	94	9.8
O89103	Compleme	4.066	3	1	3	1	644	69.3
Q8CE33	Kelch-like	4.061	2	1	4	1	709	80.4
Q8K354	Carbonyl re	4.058	9	2	5	2	277	30.9
A0A0U1RF	Pleckstrin	4.057	1	1	1	1	1212	137.2
Q9JII5	DAZ-assoc	4.053	4	1	1	1	406	43.2
A0A0A6YX	Nucleoside	4.049	14	1	5	1	96	10.6
B8JKU8	Protein AN	4.049	7	1	3	1	215	23.5
P52927	High mobi	4.048	21	1	3	1	108	11.8
Q8R3P6	Integrator	4.048	5	1	2	1	515	57.2
E9Q4P1	WD repeat	4.041	3	1	2	1	410	46.2
Q9JII6	Aldo-keto	4.041	2	1	6	1	325	36.6
Q9JJQ0	GPI mann	4.041	2	1	1	1	542	63.1
Q9QY66	Zinc finger	4.04	3	1	5	1	399	42.9
Q70FJ1	A-kinase a	4.032	1	2	2	2	3797	435.9
B9EI38	RalBP1-as	4.016	1	1	6	1	648	70.6

P16110	Galectin-3	4.009	5	1	1	1	264	27.5
A0A140LH	Protein YIF	4.005	4	1	7	1	315	34.3
Q80U19	Disheveled	3.995	1	2	11	1	1115	128.3
P08122	Collagen a	3.994	1	1	5	1	1707	167.2
Q8R3I3	Conserved	3.992	3	1	2	1	657	73
P97465	Docking pr	3.991	3	1	9	1	482	52.4
Q8R5L3	Vam6/Vps	3.99	2	1	1	1	886	101.6
Q9CWV6	PRKR-inter	3.986	5	1	34	1	186	21.5
Q8BT07	Centrosom	3.985	6	2	3	2	462	53.9
E9PUG7	Eukaryotic	3.967	2	1	4	1	552	60.8
O70435	Proteasom	3.959	5	1	1	1	255	28.4
A0A1L1ST	Isocitrate	3.955	3	1	1	1	384	41.5
D3Z4C9	Mitochond	3.942	9	1	5	1	127	15.4
D3YYY8	Rho guani	3.937	1	1	2	1	869	97.3
A0A0G2JF	Receptor-t	3.936	21	1	4	1	81	9.9
Q8BHZ0	CYFIP-rela	3.927	3	1	1	1	323	37.3
A0A0G2JG	Protein S1	3.926	21	2	6	2	78	9.1
B2RXC6	DNA-direc	3.925	1	1	3	1	1390	155.6
Q8BVL3	Sorting ne	3.919	3	1	2	1	470	52.8
E9Q4F4	Small nucl	3.916	12	1	2	1	91	10.9
P97370	Sodium/po	3.909	4	1	2	1	278	31.8
E9Q9D8	Ankyrin re	3.904	2	1	1	1	996	109.3
Q9CWK8	Sorting ne	3.892	2	1	4	1	519	58.4
Q8BRF7	Sec1 fami	3.889	3	1	2	1	639	72.3
Q9CZG9	PDZ doma	3.888	11	1	1	1	140	16.2
A0A494BA	Prefoldin s	3.866	11	1	3	1	104	12.3
Q8VHZ7	U3 small r	3.862	3	1	16	1	291	33.6
Q5DTW7	Retroelem	3.857	1	1	2	1	1521	168.9
Q9CQR6	Serine/thr	3.854	4	1	2	1	305	35.1
Q792Z1	Trypsin 10	3.849	4	1	6	1	246	26.2
Q924C1	Exportin-5	3.848	1	1	1	1	1204	136.9
Q9CQE0	E3 ubiquit	3.846	4	1	1	1	245	28.3
Q9DCF9	Translocor	3.845	8	1	7	1	185	21.1
Q8R3F9	Speckle ta	3.841	3	1	5	1	869	94.5
P62488	DNA-direc	3.838	6	1	5	1	172	19.3
Q5NCI3	DNA-direc	3.821	3	1	3	1	511	56.5
Q9WUP4	Polyprenol	3.818	4	1	7	1	330	37.9
E9QLA5	Inverted fo	3.805	1	1	1	1	1271	138.3
A2BE93	Protein SE	3.801	5	1	6	1	215	24.9
Q9D1A2	Cytosolic r	3.799	4	1	8	1	475	52.7
Q6NZF1	Zinc finger	3.798	3	1	8	1	792	86.4
Q8BID8	F-box/LRR	3.793	4	1	12	1	400	43.8
E9Q4X5	Epidermal	3.791	1	1	8	1	777	87.1
Q06318	Uteroglobi	3.788	10	1	9	1	96	10.5
Q04692	SWI/SNF-	3.784	2	1	7	1	1021	116.4

Q8C0J6	Ankyrin re	3.781	2	1	1	1	512	54.9
P30416	Peptidyl-p	3.781	3	1	4	1	458	51.5
Q3TXV4	Rab22B O:	3.781	5	1	3	1	195	21.4
O89100	GRB2-rela	3.78	6	1	4	1	322	36.8
D3YY75	Vang-like	3.775	3	1	1	1	561	63.9
P23249	Putative h	3.771	1	1	6	1	1004	113.5
Q61550	Double-str	3.767	2	1	7	1	635	72
Q8BID6	Zinc finger	3.764	3	1	2	1	600	65.5
A0A5H1ZR	Negative e	3.764	2	1	2	1	631	70.3
Q9Z0F8	Disintegrir	3.763	2	1	2	1	827	93
Q9D0M0	Exosome c	3.758	4	1	3	1	291	31.8
E9Q7F8	Histidine t	3.756	17	1	6	1	86	9.6
Q3U492	Kielin/cho	3.751	1	1	4	1	1550	166.5
Q5SWN2	Replicatio	3.748	3	1	2	1	644	71.4
A0A1L1SR	Vacuolar p	3.745	6	1	1	1	407	45.2
A0A0A6YV	REST core	3.744	2	1	8	1	447	49.9
Q8BJT9	ER degrad	3.74	3	1	3	1	577	64.6
A0A1W2P	HCLS1-bin	3.724	11	1	3	1	157	17.6
Q5U3K5	Rab-like p	3.723	2	1	17	1	725	79.8
A0A140LIV	Protein Sm	3.722	7	1	4	1	230	25.7
E9PW43	Predicted	3.719	9	1	21	1	113	11.8
Q8CHP6	Polyhome	3.719	1	1	3	1	981	105.3
Q3UFY7	7-methylg	3.719	4	1	2	1	300	34.4
Q9DB05	Alpha-solu	3.718	6	1	3	1	295	33.2
Q3TLK9	CCR4-NOT	3.717	4	1	1	1	248	28.6
Q9D0S9	Histidine t	3.715	12	1	3	1	163	17.3
A0A1L1SR	Predicted	3.714	7	1	7	1	188	21.8
O88653	Ragulator	3.71	9	1	3	1	124	13.5
Q9Z126	Platelet fa	3.704	11	1	4	1	105	11.2
Q9D6Y9	1,4-alpha-	3.702	2	1	1	1	702	80.3
Q8CIW5	Twinkle pr	3.7	2	1	2	1	685	76.9
F6WMC0	NEDD8-co	3.698	6	1	5	1	172	20
Q9CXY6	Interleukir	3.697	3	1	4	1	390	43
Q8BIV3	Ran-bindir	3.689	1	1	5	1	1105	124.5
Q9D287	Pre-mRNA	3.686	6	1	3	1	225	26.1
H3BK68	tRNA (gua	3.68	9	1	2	1	221	24.1
Q9R1W5	Calcitonin	3.67	2	1	5	1	463	53.2
Q9ER73	Elongator	3.658	2	1	1	1	422	46.3
Q8BG40	Katanin p8	3.656	1	1	4	1	658	72.6
Q1H9T6	Telomere	3.654	1	1	7	1	681	76.8
B1ATL6	Dual-speci	3.648	8	1	3	1	206	22.5
A2CEK1	EF-hand ca	3.645	2	1	5	1	466	53.2
P50580	Proliferati	3.642	4	1	3	1	394	43.7
A0A5F8MI	Neurobea	3.639	3	1	6	1	575	64.8
Q3TVI8	Pre-B-cell	3.634	2	1	2	1	727	81.1

E9PYP3	Potassium	3.628	15	1	3	1	129	14.5
Q3UB74	Transform	3.627	4	1	2	1	406	44.9
Q923D2	Flavin redu	3.615	8	1	3	1	206	22.2
Q8CFI5	Probable p	3.605	2	1	4	1	475	53.5
B1AUH1	Receptor-t	3.601	1	1	1	1	1446	162.4
Q91XC9	Peroxisom	3.58	7	1	3	1	336	38.7
Q60870	Receptor e	3.579	5	1	8	1	185	21
Q9CQ49	Nuclear ca	3.579	7	1	2	1	156	18
Q8R1H0	Homeodor	3.578	12	1	5	1	73	8.3
O88273	Gremlin-2	3.57	5	1	15	1	168	19.3
Q059U7	Protein int	3.568	2	1	7	1	942	104.8
P35486	Pyruvate d	3.567	2	1	7	1	390	43.2
P53347	Oncostatir	3.563	6	1	2	1	263	30.1
P60761	Neurograr	3.561	19	1	2	1	78	7.5
G3UZN5	DNA (cyto:	3.561	8	1	6	1	184	19.9
P30681	High mobi	3.554	7	1	5	1	210	24.1
Q8BUE4	Ferroptosi	3.553	2	1	9	1	373	40.6
H3BKL7	Deoxyribor	3.552	4	1	12	1	203	23
Q9Z1F9	SUMO-act	3.55	2	1	8	1	638	70.5
Q8CI71	Syndetin C	3.549	1	1	4	1	964	111.1
A0A0R4J0	Phospholip	3.546	2	1	1	1	550	63
E9PZQ1	DExD/H bc	3.546	0	1	13	1	1711	197.5
Q78IK2	ATP syntha	3.54	17	1	2	1	58	6.4
Q9CZJ1	Probable U	3.533	4	1	2	1	253	30.5
E0CZ18	Phosphatic	3.532	5	1	2	1	238	24.8
Q7TQD2	Tubulin po	3.525	4	1	11	1	218	23.6
Q9R0A5	Serine/thr	3.519	3	1	5	1	511	57.4
Q9JHP7	Protein O-	3.518	1	1	3	1	502	57.9
Q4G0F8	Ubinucleir	3.517	2	1	7	1	1135	122.3
Q9CZX7	Type 2 pho	3.508	4	1	1	1	257	28
D3Z3D6	DNA-direc	3.507	7	1	1	1	260	29.4
P81122	Insulin rec	3.506	1	1	3	1	1321	136.7
Q61749	Translatio	3.503	2	1	1	1	524	57.6
Q9DB16	Calcium-bi	3.501	3	1	1	1	337	39.1
Q9DCM2	Glutathior	3.496	4	1	4	1	226	25.7
Q9ER58	Testican-2	3.486	2	1	6	1	423	46.8
P20065	Thymosin	3.485	24	1	3	1	50	5.7
Q9QUJ7	Long-chair	3.485	2	1	1	1	711	79
A0A0A6YV	Rab3 GTPa	3.483	1	1	2	1	1367	152.4
E9Q109	60S riboso	3.479	7	1	3	1	133	15.2
Q9DBG1	Sterol 26-l	3.466	4	1	10	1	533	60.7
Q6PAL7	AT-hook D	3.466	2	1	2	1	1594	168
A0A1W2P	Coenzyme	3.461	3	1	3	1	249	27.9
Q8VDC1	FYVE and	3.46	1	1	3	1	1437	162.2
Q8R379	GTPase, IN	3.453	5	1	6	1	293	33.8

A6PWR8	Ubiquitin c	3.452	1	1	2	1	1127	123.3
F2Z455	Four and a	3.435	3	1	1	1	280	31.1
Q08376	Zinc finger	3.425	2	1	6	1	449	50.9
Q3TAP4	AP-5 comp	3.425	1	1	3	1	876	94.6
D3YWF5	NKAP dom	3.42	12	1	1	1	125	15
E9PUC5	PH and SE	3.42	6	1	3	1	376	42.3
P58196	Phospholip	3.417	2	1	9	1	326	36.6
Q05186	Reticuloca	3.414	4	1	1	1	325	38.1
P51827	AF4/FMR2	3.412	1	1	2	1	1254	136
Q9R1T2	SUMO-act	3.412	2	1	4	1	350	38.6
Q0VFX2	Cilia- and	3.407	3	1	3	1	523	61
Q8BIX3	ARL14 effe	3.391	6	1	2	1	276	31
Q9D7M8	DNA-direc	3.389	13	1	3	1	142	16.3
Q8R1B8	Nuclear re	3.388	3	1	9	1	470	53.1
F6QMA6	Predicted	3.384	5	1	1	1	170	18.3
P50637	Translocat	3.376	8	1	1	1	169	18.8
Q8BI08	Protein M/	3.373	6	1	4	1	175	19.1
F6Q475	FtsJ RNA r	3.37	16	1	3	1	111	12.4
E0CY64	General tr	3.37	7	1	3	1	184	20.4
E0CX53	TBC1 dom	3.368	1	1	1	1	1306	147.2
Q80U93	Nuclear pc	3.365	1	1	2	1	2085	212.8
Q3UTQ8	Cyclin-dep	3.364	2	1	2	1	938	105.4
Q61093	Cytochrom	3.36	2	1	3	1	570	65.3
F6ZLH4	Lysine-rich	3.359	5	1	1	1	347	38.9
Q9DCI9	39S riboso	3.358	5	1	5	1	187	21.7
A0A668KL	Steroid ho	3.356	2	1	2	1	499	53.2
A2A7K5	c-Myc-bin	3.354	6	1	5	1	146	16.6
Q3ULJ0	Glycerol-3	3.339	2	1	12	1	351	38.2
Q8K2J7	RELT-like	3.337	3	1	9	1	272	29.3
Q80ZI6	E3 ubiquit	3.335	1	1	3	1	727	83.9
A0A338P6	Presenilins	3.333	3	1	7	1	343	38.4
D3Z065	Beta-1,4-g	3.331	4	1	5	1	220	25.8
Q91VT1	E3 SUMO-	3.326	4	1	4	1	247	28.2
D3YXN0	AP-1 comp	3.323	5	1	4	1	189	22
Q9D051	Pyruvate d	3.317	3	1	1	1	359	38.9
J3QNK5	Histone de	3.311	1	1	1	1	1057	111.2
A0A286YD	NAD(P)H p	3.311	11	1	1	1	137	15.8
A0A6Q6Q/	INO80 con	3.306	3	1	9	1	358	38.8
E0CYW1	Ubiquitin-	3.298	16	1	5	1	56	6.5
Q61666	Protein HI	3.295	1	1	8	1	1015	111.7
A0A0B4J1	Immunogl	3.293	17	1	3	1	117	13
A0A338P7	Predicted	3.284	7	1	15	1	117	13.1
Q05DP1	Serine/thr	3.283	6	1	4	1	227	25.3
Q9WTK5	Nuclear fa	3.275	2	1	1	1	899	96.8
Q6PE65	G patch do	3.273	2	1	1	1	482	54

Q9JKY5	Huntingtin	3.273	1	1	1	1	1068	119.4
Q91UZ5	Inositol m	3.27	4	1	2	1	290	31.7
D3Z2D6	Spastic pa	3.269	17	1	4	1	116	13.2
Q8BZW8	NHL repea	3.269	2	1	8	1	725	78.4
Q60766	Immunity-	3.267	2	1	1	1	409	46.5
Q9CX48	Zinc finger	3.266	7	1	1	1	178	19.1
Q8BTZ7	Mannose-	3.266	3	1	2	1	360	39.9
Q8VBZ3	Cleft lip ar	3.253	3	1	2	1	664	75.2
Q9QZB9	Dynactin s	3.251	6	1	2	1	182	20.1
Q3TZA2	Cyclin-dep	3.248	2	1	2	1	342	39.4
Q8K2V6	Importin-1	3.246	2	1	1	1	975	112.3
P14069	Protein S1	3.239	9	1	19	1	89	10
K3W4L3	Prosaposir	3.238	2	1	1	1	556	61.3
Q64430	Copper-tra	3.222	1	1	3	1	1491	161.9
Q99N50	Synaptotag	3.22	1	1	2	1	950	106.7
Q3UK78	Polycomb	3.218	6	1	5	1	256	29.6
A0A1W2P	ABRA C-te	3.206	16	1	5	1	57	6.4
A0A2I3BPI	TATA-box-	3.205	1	1	1	1	1030	104.1
P52785	Retinal gu	3.205	1	1	23	1	1108	120.6
P32020	Sterol carr	3.202	1	1	16	1	547	59.1
Q61107	Guanylate	3.198	2	1	6	1	620	70.8
Q9CQS3	Fin bud ini	3.198	4	1	6	1	217	24.7
P17665	Cytochrom	3.195	14	1	5	1	63	7.3
A0A2I3BP	TBC1 dom	3.191	8	1	1	1	97	10.5
O70230	Zinc finger	3.184	1	1	5	1	638	69
E9PY43	Glutamate	3.176	3	1	7	1	332	37.3
Q91X46	Rho guani	3.175	2	1	2	1	524	59.5
P97490	Adenylate	3.175	1	1	2	1	1249	140
Q9CQL5	39S riboso	3.175	5	1	13	1	180	20.7
A0A0N4SV	Endoplasm	3.16	7	1	3	1	198	22.1
Q8BVC4	Coiled-coil	3.159	3	1	6	1	333	38
A0A1L1SU	Rho guani	3.157	13	1	4	1	106	11.5
Q80UR4	Mast cell	3.151	3	1	3	1	318	35
Q9CQX4	PCNA-asso	3.151	15	1	4	1	110	12
Q03963	Interferon-	3.15	3	1	2	1	515	58.2
O54784	Death-asso	3.149	2	1	6	1	448	51.4
Q8K010	5-oxoproli	3.148	2	1	2	1	1288	137.5
Q14C51	Pentatrico	3.145	1	1	8	1	685	77.7
Q7TSF4	Leucine-ric	3.138	5	1	3	1	339	37.6
O88307	Sortilin-rel	3.136	1	1	1	1	2215	246.9
Q9JL35	High mobi	3.135	2	1	2	1	406	45.3
F6R6F1	RalBP1-as	3.134	1	1	1	1	755	81.9
Q3UC65	Arginine/s	3.133	5	1	2	1	298	34.5
Q8VD76	General tr	3.13	8	1	5	1	309	34.2
P49935	Pro-cathep	3.129	5	1	3	1	333	37.1

E9QLS6	Phosphatic	3.127	1	1	15	1	848	96.8
F6XMS4	Pseudouric	3.124	6	1	3	1	176	19.4
E9Q6J5	Biorientati	3.123	0	1	5	1	3032	327.3
A0A0A6YY	Rab GTPas	3.12	6	1	2	1	234	26.9
Q07113	Cation-ind	3.117	1	1	1	1	2483	273.6
Q9DAU9	Zinc finger	3.115	2	1	3	1	571	65.1
P29416	Beta-hexo	3.114	4	1	4	1	528	60.6
Q99LJ6	Glutathior	3.111	9	1	1	1	186	21
O08797	SPI6 OS=M	3.111	4	1	4	1	374	42.2
Q8BX57	PX domair	3.104	2	1	3	1	582	65.2
Q6NV56	Short trans	3.102	4	1	1	1	406	45.9
H7BX59	tRNA pseu	3.102	2	1	3	1	441	49.6
Q69Z89	Ras-associ	3.098	1	1	2	1	1099	120.3
Q60649	Caseinolyt	3.097	3	1	5	1	677	76
E9Q3F3	EGF-conta	3.097	4	1	1	1	280	31.6
E9PYP2	Neurobeac	3.096	0	1	3	1	2688	306
Q9JMC3	DnaJ homo	3.093	3	1	1	1	397	44.9
O88983	Syntaxin-8	3.09	3	1	9	1	236	26.9
P20491	High affini	3.089	10	1	6	1	86	9.6
Q8BHN5	RNA-bindi	3.081	1	1	7	1	476	53.3
Q8BKN5	Gamma-tu	3.078	1	1	2	1	1024	117.9
Q3V3R4	Integrin al	3.077	1	1	5	1	1179	130.7
Q9CY16	28S riboso	3.075	5	1	5	1	186	20.5
Q9DCR2	AP-3 comp	3.075	6	1	9	1	193	21.7
Q9JI39	ATP-bindir	3.069	1	1	3	1	715	77.1
Q8BLQ0	Protein FA	3.067	5	1	2	1	456	52
A0A140T8	Immunogl	3.066	13	1	2	1	120	13.1
G3X983	T cell activ	3.066	3	1	2	1	509	58.5
P02104	Hemoglob	3.062	5	1	4	1	147	16.1
Q9CZ62	Centrosom	3.058	1	1	3	1	856	94.6
P70245	3-beta-hyc	3.058	4	1	9	1	230	26.2
A0A0N4SV	Dysferlin C	3.057	0	1	1	1	2103	239.1
F8VPX2	F-box and	3.057	3	1	1	1	458	50.5
A0A2I3BQ	MAPK-inte	3.052	7	1	3	1	153	16.3
Q80WQ2	Protein VA	3.051	1	1	6	1	782	88
Q9CQJ2	PIH1 doma	3.049	3	1	4	1	290	32.2
P56382	ATP syntha	3.048	15	1	14	1	52	5.8
Q8R3N1	Nucleolar	3.046	1	1	2	1	860	98.7
Q8K4W8	Chloride cl	3.039	1	1	11	1	791	87.9
P08920	T-cell surfa	3.036	3	1	2	1	344	38.4
Q9D6T0	Nitric oxid	3.034	7	1	2	1	301	33.2
Q8BH40	Syntaxin-7	3.032	3	1	3	1	261	29.7
G5E8Q0	Lysosomal	3.032	0	1	1	1	3787	425.1
Q922J9	Fatty acyl-	3.032	3	1	5	1	515	59.4
Q9D2U5	N-alpha-ac	3.031	7	1	5	1	125	13.4

Q3V1M1	Immunogl	3.028	1	1	1	1	2594	285.4
A0A286YC	Centrin-3 (	3.024	10	1	5	1	87	10
Q9Z2G0	Protein fer	3.021	4	1	6	1	627	70.2
Q62189	U1 small r	3.015	3	1	2	1	287	31.8
D3Z7P0	BRISC and	3.005	3	1	7	1	385	43.7
G3X9X1	Kelch repe	3.003	3	1	3	1	623	71.2
A0A494B9	39S riboso	3.002	8	1	2	1	114	12.3
Q60898	Src-like-ac	2.997	5	1	2	1	281	31.7
D3YWT0	Signal pep	2.996	5	1	1	1	170	19.6
Q9CZX5	PIN2/TERF	2.995	6	1	5	1	332	37.2
Q9R1K9	Centrin-2 (	2.988	5	1	8	1	172	19.8
P61963	DDB1- and	2.986	6	1	1	1	342	38.9
Q0VBL3	RNA-bindi	2.985	1	1	1	1	962	105.7
Q8K1G9	RNA helica	2.985	1	1	1	1	679	75.8
Q8R2Q8	Bone marr	2.985	5	1	1	1	172	19.1
O08586	Phosphatid	2.984	2	1	1	1	403	47.1
Q9CQI9	Mediator c	2.981	4	1	11	1	178	20.3
A0A1B0GT	Glutaredox	2.979	7	1	2	1	163	18.1
Q8BWZ3	N-alpha-ac	2.977	1	1	2	1	972	111.6
F7BJB9	MORC fam	2.97	1	1	2	1	942	106.5
A0A1W2P	Peroxisom	2.966	8	1	1	1	102	11.2
D3YVI6	COP9 signa	2.957	5	1	1	1	176	19.5
P14152	Malate de	2.956	4	1	3	1	334	36.5
Q8VBT9	Tether cor	2.951	3	1	1	1	550	59.8
P70424	Receptor t	2.941	1	1	4	1	1256	138.5
Q9D2L9	Serine pro	2.941	2	1	3	1	613	69.9
Q8VDM1	Zinc finger	2.94	4	1	5	1	511	56.4
Q9DAT5	Mitochond	2.936	3	1	1	1	417	47.2
A0A0R4IZ	Myeloblas	2.935	4	1	1	1	254	27.6
Q8CB27	Ubiquitin t	2.932	4	1	1	1	343	37.5
E9Q9D5	Rab-like p	2.931	6	1	4	1	223	25.6
P46664	Adenylosu	2.931	4	1	1	1	456	50
B1AVD2	Xaa-Pro ar	2.931	1	1	1	1	741	83.7
Q8C6N3	Synaptotag	2.929	2	1	1	1	418	47.2
B1AY86	Plexin dom	2.927	3	1	2	1	479	53.7
Q9WV54	Acid ceran	2.925	2	1	2	1	394	44.6
Q9CX00	IST1 homo	2.923	3	1	2	1	362	39.4
P97287	Induced m	2.923	2	1	1	1	331	35.2
F8WHP8	ATP syntha	2.923	16	1	7	1	76	9
Q9JIZ9	Phospholip	2.92	4	1	3	1	296	31.8
A0A2R8W	E3 ubiquit	2.919	14	1	15	1	59	6.9
Q8BL06	Inactive ub	2.916	0	1	10	1	1588	176.6
A0A0A0M	Protein LS	2.914	7	1	1	1	137	15.4
E9Q9M1	Cytosolic p	2.914	1	1	5	1	586	67.7
Q8CHT3	Integrator	2.91	1	1	1	1	1018	108.3

Q9DBP0	Sodium-de	2.909	1	1	4	1	697	76.2
A2ACH6	Delta-sarc	2.908	5	1	2	1	192	21.7
Q91W69	Epsin-3 OS	2.908	2	1	1	1	636	68.2
Q8BKG3	Inactive ty	2.906	2	1	2	1	1062	117.5
P59729	Ras and Ra	2.906	1	1	6	1	980	107.2
Q3UPV7	Zinc finger	2.903	3	1	7	1	304	35.3
Q3UVL4	Vacuolar p	2.903	1	1	1	1	782	86.1
P48722	Heat shock	2.901	2	1	1	1	838	94.3
B1AXH1	NHS-like p	2.901	2	1	8	1	1219	132.3
Q810A7	ATP-deper	2.894	2	1	7	1	929	101.9
G3UYV5	Integrin al	2.89	7	1	4	1	133	14.7
Q9JME7	Trafficking	2.888	6	1	2	1	139	16
Q3UKC1	Tax1-bindi	2.886	1	1	4	1	814	93.6
F6RCM5	Adaptin ea	2.886	13	1	6	1	77	8.8
A0A1L1SS	tRNA (gua	2.886	23	1	4	1	82	9.5
Q9Z1B3	1-phospha	2.884	1	1	4	1	1216	138.3
Q8CJ69	BMP-bindi	2.884	2	1	1	1	685	76.1
F8WGD2	Ras and Ra	2.883	1	1	4	1	858	96.5
E9PUS2	BTB (POZ)	2.881	1	1	2	1	523	56.4
Q8K3A2	tRNA 2'-pl	2.88	7	1	6	1	249	27.3
P47758	Signal reco	2.874	4	1	2	1	269	29.6
Q8R2N2	U3 small n	2.872	1	1	7	1	686	76.9
Q99L90	Microsphe	2.871	2	1	15	1	462	51.7
Q8CHC4	Synaptojar	2.868	1	1	5	1	1574	172.5
Q80UU1	Ankyrin re	2.866	3	1	1	1	748	82.9
F6TN13	RNA-bindi	2.864	3	1	2	1	463	52.6
Q80X82	Symplekin	2.859	1	1	2	1	1288	142.5
O08547	Vesicle-tra	2.856	4	1	1	1	215	24.7
Q07417	Short-chai	2.854	2	1	4	1	412	44.9
Q8C0I4	Enhancer o	2.853	1	1	1	1	808	90.9
Q80WG5	Volume-re	2.846	2	1	3	1	810	94.1
Q3UK98	Chromobo	2.845	2	1	2	1	396	42.5
A0A2I3BR!	Krueppel-l	2.845	2	1	2	1	464	50.2
P51830	Adenylate	2.844	1	1	1	1	1353	150.9
Q3UWE6	WD repeat	2.843	2	1	1	1	569	62.8
E9Q9M5	Ubiquitin o	2.843	1	1	6	1	1299	143.4
Q8CAA7	Glucose 1,	2.84	1	1	1	1	621	70.2
B1AVN9	Phosphata	2.833	1	1	1	1	556	61.3
A2AJW4	Protein ph	2.831	5	1	4	1	279	30.5
Q9R244	Short tran	2.829	1	1	2	1	1172	130.4
Q91YN0	Protein C1	2.829	3	1	1	1	552	63.6
E9QAQ3	Rho GTPas	2.828	1	1	9	1	759	85.9
Q7TMG8	Glioblasto	2.827	4	1	4	1	281	32.9
Q60847	Collagen a	2.823	1	1	1	1	3120	340
Q9CRD2	ER membr	2.821	4	1	1	1	297	34.9

Q924T2	28S riboso	2.82	4	1	3	1	291	32.3
Q9CQV5	28S riboso	2.819	5	1	13	1	167	18.9
Q9R210	Transcripti	2.814	2	1	7	1	475	52.6
Q9R1Q7	Proteolipic	2.811	8	1	2	1	152	16.6
E9Q0B5	Fc fragme	2.809	0	1	21	1	2583	275
Q9DBH5	Vesicular i	2.806	3	1	3	1	358	40.4
Q9QY40	Plexin-B3 (	2.805	0	1	1	1	1902	208.2
Q9CZH7	Matrix-rer	2.803	4	1	6	1	178	19.4
Q80TN4	DnaJ homo	2.798	2	1	4	1	772	89.1
P97762	Retinitis p	2.796	4	1	2	1	213	25.2
E9Q8K8	Zinc finger	2.792	2	1	1	1	1255	135.9
Q9EST1	Gasdermi	2.782	2	1	3	1	446	49.6
Q5HZH2	18S rRNA	2.782	2	1	6	1	323	35.3
A0A0U1RI	S-adenosy	2.779	3	1	2	1	362	39.7
P70404	Isocitrate (	2.779	3	1	2	1	393	42.8
E0CYT5	Cullin-2 (F	2.777	2	1	1	1	582	67.7
G5E8I8	Calcium hc	2.773	1	1	8	1	949	107.6
E9PYI6	SUMO-int	2.772	4	1	4	1	453	52.2
P58466	Carboxy-te	2.771	6	1	1	1	261	29.2
Q8BGS2	BolA-like p	2.769	22	1	3	1	86	10.2
G3UZM1	Probable J	2.769	0	1	4	1	2531	281.4
Q8R124	Kelch-like	2.768	2	1	2	1	613	69.9
A2AC16	L-xylulose	2.768	5	1	1	1	236	25
Q9D6K5	Synaptojar	2.763	8	1	5	1	145	15.8
Q8VCH6	Delta(24)-	2.754	2	1	1	1	516	60.1
Q8C0C0	Zinc finger	2.75	1	1	3	1	836	92.2
Q3TIV9	AP comple	2.749	6	1	3	1	157	18.6
A0A1Y7VL	Phosphofu	2.748	1	1	1	1	894	98.4
Q8K449	ATP-bindir	2.748	1	1	3	1	1623	183
Q80UX8	Protein AB	2.747	2	1	2	1	337	38.5
Q9JI13	Something	2.747	2	1	12	1	469	53.4
Q9D2G2	Dihydrolip	2.746	2	1	9	1	454	49
O08997	Copper tra	2.743	21	1	1	1	68	7.3
K4DI77	WD repeat	2.741	0	1	3	1	1934	211.8
Q61335	B-cell rece	2.74	4	1	2	1	245	27.9
P21956	Lactadheri	2.74	4	1	1	1	463	51.2
Q8CFW1	Anoctamir	2.738	2	1	11	1	1002	114.1
Z4YKM2	CDGSH iro	2.738	7	1	7	1	115	13.1
Q9D1B9	39S riboso	2.736	4	1	3	1	257	30.2
Q60772	Cyclin-dep	2.731	5	1	2	1	168	18.1
Q91X96	Guanine n	2.73	9	1	1	1	123	13.9
Q60902	Epidermal	2.727	1	1	3	1	907	99.2
Q91VX2	Ubiquitin-a	2.727	1	1	1	1	1132	117.9
G8JL51	DNA-direc	2.726	3	1	1	1	407	45.9
D3Z342	Paraplegir	2.722	3	1	1	1	230	26.2

E9QK48	Echinoderm	2.717	1	1	1	1	841	90.7
J3QNR9	RIKEN cDN	2.717	7	1	1	1	108	12.6
P62774	Myotrophi	2.716	14	1	2	1	118	12.9
E9PWE9	Tyrosine-p	2.713	1	1	2	1	583	66
D3YVS7	Cysteine-ri	2.708	12	1	1	1	84	9.2
Q9CPW5	Translocor	2.704	4	1	3	1	183	20
E0CYJ7	MPN doma	2.703	4	1	2	1	437	47.7
A0A338P6	ABI family	2.7	1	1	1	1	1637	179.4
Q99LD8	N(G),N(G)	2.699	3	1	6	1	285	29.6
Q3UXU8	CST compl	2.699	3	1	2	1	351	40.4
Q6NVE8	WD repeat	2.699	1	1	2	1	915	101.5
P28665	Murinoglo	2.698	1	1	1	1	1476	165.2
Q8R3L2	Transcript	2.697	1	1	4	1	676	76.6
Q6PG04	Coiled-coil	2.692	1	1	1	1	518	61
D3YVJ7	Aldo-keto	2.692	5	1	11	1	176	19.6
P35330	Intercellula	2.69	3	1	14	1	277	31.4
P31695	Neurogeni	2.688	1	1	1	1	1964	206.6
Q9D1Q4	Dolichol-p	2.685	11	1	1	1	92	10.1
Q6PAV2	Probable E	2.684	2	1	6	1	1057	118.3
Q80TG1	KAT8 regu	2.677	1	1	1	1	1036	113.1
Q8BP78	Protein FR	2.671	3	1	1	1	315	37.2
Q811S7	Upstream-	2.666	3	1	1	1	540	60.2
Q8K1C9	Leucine-ric	2.661	1	1	4	1	807	88.1
Q8VEE1	LIM and cy	2.66	3	1	6	1	365	41
Q8BSA9	tRNA wyb	2.658	7	1	1	1	257	28.6
Q8K3W2	Leucine-ric	2.653	4	1	1	1	274	31.2
Q6NT99	Dual speci	2.652	9	1	4	1	150	16.6
Q7TT36	Adhesion G	2.648	1	1	2	1	1310	144.6
Q9CZU4	GTPase Er	2.648	3	1	1	1	437	48.2
Q62481	Vacuolar p	2.647	2	1	4	1	368	40.8
Q922Z0	D-aspartat	2.644	2	1	3	1	341	37.5
Q5SXR2	Vacuole m	2.643	10	1	6	1	70	8.3
S0DHL8	Capping pr	2.643	1	1	2	1	1397	151.7
P70196	TNF recep	2.641	2	1	1	1	530	60
P99026	Proteasom	2.639	3	1	1	1	264	29.1
E9QA79	Major urin	2.639	7	1	1	1	235	26.8
B2RQE8	Rho GTPas	2.638	2	1	2	1	841	94.6
A0A1B0GS	Growth ho	2.636	2	1	4	1	289	32.9
Q80X32	UPF0461 p	2.633	6	1	1	1	188	20.1
P97457	Myosin reg	2.632	6	1	6	1	169	18.9
S4R2P2	HECT-type	2.632	3	1	5	1	243	27.8
Q6P3A9	ADP-ribosy	2.627	5	1	6	1	176	19.2
A0A286YC	Nidogen-2	2.624	1	1	1	1	1171	128.4
Q99PM0	Microfibril	2.623	5	1	1	1	185	20.8
Q6PB66	Leucine-ric	2.623	1	1	1	1	1392	156.5

D3Z6G2	Ras associ	2.621	2	1	1	1	321	35.6
O88895	Histone de	2.62	4	1	3	1	424	48.3
Q7SIG6	Arf-GAP w	2.62	1	1	1	1	958	106.7
Q99JR5	Tubulointe	2.619	2	1	8	1	466	52.6
A2A6J2	Troponin T	2.617	13	1	2	1	107	13
Q8VFZ5	Olfactory r	2.617	2	1	10	1	314	35.6
P57787	Monocarbo	2.613	3	1	2	1	470	50.3
A0A087W	5'-3' exori	2.612	1	1	1	1	969	112.4
Q9CWL8	Beta-cater	2.612	1	1	1	1	563	64.9
Q9D0B1	Zinc finger	2.607	2	1	12	1	321	34.8
A0A140LJI	Zinc finger	2.606	4	1	2	1	230	25.3
Q3U0J8	TBC1 dom	2.605	1	1	6	1	965	109.9
O55091	Protein IM	2.605	5	1	2	1	318	36.3
Q9CQM5	Thioredoxi	2.598	7	1	2	1	123	14
Q9D7H3	RNA 3'-ter	2.597	3	1	4	1	366	39.2
A0A0G2JF	LIM doma	2.593	5	1	3	1	371	42.5
Q3V1T4	Prolyl 3-hy	2.593	1	1	1	1	739	83.6
P60840	Alpha-end	2.591	14	1	2	1	121	13.3
E9Q933	Transmem	2.591	5	1	2	1	176	19.8
Q6ZQI3	Malectin C	2.59	2	1	6	1	291	32.3
P46638	Ras-relate	2.59	5	1	5	1	218	24.5
F2Z461	E3 ISG15--	2.585	1	1	5	1	1003	112.9
E9PWU4	Adiponect	2.58	5	1	4	1	168	17.9
R4H4V1	N-termina	2.579	1	1	3	1	749	82.4
A2RSJ4	UHRF1-bir	2.577	1	1	1	1	1457	161.8
F7BCK0	Doublecor	2.575	4	1	1	1	340	37
Q6IRU7	Centrosom	2.575	1	1	1	1	790	86.8
Q8CGV5	Matrix me	2.573	3	1	2	1	510	57.3
P56375	Acylphosp	2.57	12	1	3	1	106	11.9
Q3TWM5	Nephrocys	2.566	1	1	1	1	690	77.3
Q923D4	Splicing fa	2.563	13	1	2	1	86	10.1
Q8CD26	Solute car	2.563	2	1	2	1	409	44.3
E9Q804	Ankyrin re	2.562	0	1	2	1	2602	274
Q8BW75	Amine oxi	2.558	2	1	7	1	520	58.5
O89112	Glutathior	2.551	2	1	3	1	399	45.3
Q9EQH2	Endoplasm	2.551	1	1	2	1	930	106.5
P70191	TNF recep	2.55	3	1	1	1	558	64.1
Q99LC8	Translatio	2.549	3	1	4	1	305	33.8
Q91VZ6	Stromal m	2.548	2	1	2	1	440	47.6
Q3TKY6	Spliceosor	2.547	3	1	1	1	469	53.5
P53349	Mitogen-a	2.547	1	1	1	1	1493	161.2
P55849	Desmocoll	2.542	1	1	7	1	886	98.9
Q9D8P4	39S riboso	2.54	5	1	4	1	176	20.2
A2AGH8	Mediator c	2.54	1	1	2	1	2157	240.7
D3Z4B0	Serine and	2.54	4	1	2	1	192	19.3

Q9JM51	Prostaglar	2.539	7	1	2	1	153	17.3
E9QP99	Golgin sub	2.539	1	1	2	1	1487	167.2
E9Q8V6	DENN/MA	2.537	1	1	2	1	1869	209.2
P11416	Retinoic a	2.533	2	1	1	1	462	50.7
Q3UFK8	FERM dom	2.533	2	1	2	1	466	51.8
Q9EQJ0	Two pore	2.53	1	1	3	1	817	94.4
Z4YLR9	Calcium-bi	2.53	3	1	2	1	263	30
A2AWI7	Endophilin	2.528	4	1	2	1	404	44.9
Q8VID5	ATP-deper	2.527	1	1	2	1	982	108.2
D3Z079	Syntaxin-b	2.527	1	1	22	1	1116	123.5
Q3UWM4	Lysine-spe	2.526	2	1	1	1	940	106
Q9CT10	Ran-bindir	2.525	2	1	2	1	491	52.5
Q6NZK2	NCK-assoc	2.522	1	1	2	1	1841	200.9
P62892	60S riboso	2.521	20	1	20	1	51	6.4
Q61133	Glutathior	2.521	6	1	3	1	244	27.6
Q60953	Protein PM	2.517	2	1	3	1	885	98.2
Q6NXJ0	Protein W	2.516	1	1	1	1	1187	132.5
Q8K1H1	Tudor dom	2.515	1	1	17	1	1086	122.1
Q8CGC4	Protein LS	2.514	8	1	2	1	385	42.3
Q921W0	Charged m	2.514	4	1	1	1	196	21.6
Q6GQT9	Nodal moc	2.51	1	1	2	1	1214	133.3
G3X9T7	Galectin-9	2.506	2	1	2	1	353	40
Q99M01	Phenylalar	2.501	3	1	23	1	451	52.3
Q3UM45	Protein ph	2.501	2	1	1	1	361	41.3
Q9JK91	DNA mism	2.5	2	1	1	1	760	84.6
O35973	Period circ	2.498	1	1	1	1	1291	136.3
Q32MT0	Mefv prote	2.497	2	1	1	1	808	90.4
Q8CCM6	Mitochond	2.496	8	1	3	1	244	27.9
Q8VE88	Protein FA	2.495	2	1	1	1	497	54
Q09324	Beta-1,3-g	2.495	2	1	52	1	428	49.8
A2A6T1	Cerebellar	2.494	2	1	1	1	465	53.2
A0A0A6YV	Protocadh	2.494	1	1	5	1	1203	132.2
A0A1L1SQ	General tr	2.491	18	1	2	1	66	7.6
Q80V23	Zinc finger	2.49	6	1	5	1	272	30.8
Q8CI33	CWF19-lik	2.489	3	1	3	1	537	60.2
A0A0J9YV	Neuronal v	2.488	24	1	16	1	33	3.6
Q80UK0	SEC14 don	2.487	1	1	1	1	696	79.3
Q8BTN6	Leukocyte	2.483	1	1	5	1	485	52.6
P55772	Ectonuclec	2.482	4	1	1	1	510	57.2
Q9D6Y7	Mitochonc	2.481	5	1	4	1	233	26
H7BWX9	Small ubic	2.481	19	1	3	1	53	6
A2ADY9	Protein DD	2.481	2	1	1	1	399	44.6
E9QNY8	Sacsin OS=	2.48	0	1	3	1	4582	520.4
Q07139	Protein EC	2.474	2	1	2	1	913	103.1
P24604	Tyrosine-p	2.474	2	1	1	1	630	73.4

A0A2I3BPI	Ral GTPas	2.47	1	1	1	1	1957	218.9
P62313	U6 snRNA	2.47	9	1	6	1	80	9.1
Q924N4	Solute car	2.469	1	1	1	1	1150	127.4
O35453	Serine pro	2.468	2	1	11	1	436	46.8
Q9DCC8	Mitochonc	2.467	5	1	33	1	145	16.3
D3YTQ8	Complex I	2.466	22	1	3	1	77	8.7
A2A5L7	Tumor nec	2.455	14	1	1	1	121	13.3
Q91WU5	Arsenite m	2.454	2	1	5	1	376	41.8
Q8CHT0	Delta-1-py	2.453	3	1	2	1	562	61.8
Q8C729	Protein FA	2.453	2	1	1	1	530	58.6
Q9D1M1	DNA-direc	2.452	6	1	5	1	126	14.4
E9PVW8	Protein-tyr	2.452	0	1	2	1	1506	169.3
Q8BND3	WD repeat	2.451	1	1	2	1	1181	133.9
E9Q6K3	Progestero	2.449	1	1	3	1	756	89.5
E9Q2B2	Nuclear re	2.446	0	1	8	1	2386	263.9
E9QPI2	Putative m	2.446	1	1	1	1	705	79.7
Q8BHG2	CXXC moti	2.443	11	1	3	1	160	18
E0CX32	Coiled-coil	2.438	2	1	5	1	545	59.6
P97310	DNA replic	2.437	2	1	3	1	904	102
Q9D1E6	Tubulin-fo	2.436	3	1	1	1	244	27.4
Q9DCM7	Nucleus ac	2.436	2	1	3	1	586	63.2
Q63829	COMM do	2.435	7	1	2	1	195	22
E9Q1U1	Coiled-coil	2.433	1	1	3	1	1324	152.2
Q8BKX7	Zinc finger	2.433	2	1	1	1	478	52.2
Q8CFJ9	GATOR co	2.431	1	1	2	1	790	88.1
Q9D168	Integrator	2.428	5	1	2	1	461	48.5
Q2M4I6	SCAN dom	2.424	6	1	1	1	142	15.6
A0A2I3BR.	Transcripti	2.423	1	1	1	1	675	72.8
H3BJM1	Forkhead I	2.422	1	1	2	1	675	75.1
P22907	Porphobili	2.421	2	1	2	1	361	39.3
Q5SF07	Insulin-like	2.421	2	1	2	1	592	65.5
Q571G4	Protein lin	2.42	3	1	4	1	749	79.5
A0A494BA	Histone ac	2.42	2	1	4	1	494	56.2
Q543K9	Purine nuc	2.418	3	1	1	1	289	32.2
P61089	Ubiquitin-c	2.418	5	1	1	1	152	17.1
Q3U9N9	Monocarb	2.418	2	1	1	1	512	55.3
Q8K039	Uncharact	2.417	6	1	2	1	155	17.5
Q9CQ80	Vacuolar p	2.417	5	1	2	1	176	20.7
A7E1Z5	Anion exch	2.414	1	1	1	1	1070	120.4
Q8BKE6	Cytochrom	2.41	2	1	2	1	462	52.1
G3X9H5	Huntingtin	2.409	0	1	2	1	3120	344.6
A0A0A6YX	Choline/et	2.409	4	1	2	1	247	27
Q64277	ADP-ribosy	2.409	3	1	1	1	311	34.6
Q69ZB3	Testis-spe	2.408	4	1	3	1	406	44.3
Q9CPX7	28S riboso	2.408	6	1	2	1	135	15.2

Q3ULJ3	UPF3 regu	2.405	3	1	1	1	422	48.7
Q9WU28	Prefoldin s	2.404	5	1	12	1	154	17.3
A0A140LIC	Protein fuz	2.404	2	1	2	1	387	42.5
B2RXP3	Histone-ly:	2.401	2	1	2	1	697	78.8
A2AI89	Phosphory	2.401	1	1	1	1	1211	135.5
Q9JK92	Heat shocl	2.401	5	1	5	1	196	21.5
Q791V5	Mitochonc	2.4	3	1	6	1	303	33.5
Q9DCA2	28S riboso	2.398	5	1	2	1	191	20.2
A0A2R8VK	Vacuolar p	2.396	10	1	2	1	183	21.1
A2AJ28	Chloride ir	2.395	3	1	3	1	235	26.6
Q5NCB7	E3 ubiquit	2.395	2	1	2	1	510	57
A2A6M1	Vacuolar-s	2.395	4	1	1	1	233	26
D3Z091	Phosphatic	2.394	6	1	2	1	310	33.6
Q99PP9	Tripartite	2.391	1	1	1	1	556	62.9
Q3TPE9	Ankyrin re	2.388	2	1	1	1	440	48.7
Q9Z266	SNARE-as:	2.386	10	1	1	1	136	14.9
Q9CZ44	NSFL1 cofa	2.383	2	1	1	1	370	40.7
Q00422	GA-binding	2.383	2	1	1	1	454	51.3
A0A0G2JG	Threonine-	2.381	6	1	1	1	264	29.7
A0A0G2JE	Zinc finger	2.379	1	1	2	1	657	76.1
D3Z558	Protein DG	2.377	6	1	2	1	198	22.8
Q6PDY0	Coiled-coil	2.375	3	1	6	1	202	22.1
Q99N92	39S riboso	2.371	10	1	1	1	148	15.9
E9PYZ7	RNA-bindi	2.37	1	1	2	1	1009	113.7
Q8BLB7	Lethal(3)m	2.369	2	1	5	1	883	99.1
Q8BN57	Protein C3	2.368	4	1	2	1	294	33.6
Q9R078	5'-AMP-ac	2.368	6	1	1	1	270	30.3
Q9CX56	26S protea	2.368	2	1	2	1	353	39.9
P21995	Embigin O	2.366	2	1	3	1	330	37
D3YYJ4	Hermansky	2.362	4	1	2	1	202	22.2
P16056	Hepatocyt	2.361	1	1	1	1	1379	153.5
Q8R411	Myc targe	2.358	4	1	4	1	188	21.1
A6H6A9	Rab GTPas	2.353	3	1	1	1	815	92.3
Q8R015	Biogenesis	2.351	10	1	3	1	185	21.3
Q9CR68	Cytochrom	2.349	3	1	4	1	274	29.3
A0A0G2JE	Vacuolar p	2.349	6	1	10	1	111	12.7
B1AWT4	Ras-relate	2.347	5	1	2	1	279	32
H3BJD6	Protein ph	2.346	2	1	4	1	1292	144.3
A2AWR3	G protein-	2.344	1	1	2	1	868	96.3
F6YTL8	YLP motif-	2.344	1	1	2	1	1577	178.5
Q8K072	Receptor e	2.342	3	1	6	1	257	29.7
A2ALN0	ATP-bindir	2.34	2	1	2	1	504	55.8
G5E8R3	Pyruvate c	2.337	1	1	2	1	1178	129.6
A2ADA5	tRNA pseu	2.337	3	1	7	1	291	32
Q8CDV6	Coiled-coil	2.337	2	1	24	1	558	65.2

P13405	Retinoblas	2.333	1	1	1	1	921	105.3
Q9Z1B5	Mitotic sp	2.331	3	1	2	1	205	23.6
D3Z1J8	Midnolin (	2.33	10	1	1	1	82	9
P97471	Mothers a	2.33	2	1	1	1	551	60.3
Q9JHR7	Insulin-deg	2.33	1	1	4	1	1019	117.7
Q80YR9	RNA-bindi	2.329	1	1	2	1	836	96.5
A0A5F8MI	cGMP-dep	2.328	2	1	9	1	382	43.2
P98192	Dihydroxya	2.328	1	1	4	1	678	76.8
Q91WT7	3-alpha-hy	2.327	3	1	2	1	323	37.2
Q9D172	Glutamine	2.322	3	1	1	1	266	28.1
Q5F4T0	Transient	2.322	1	1	1	1	1721	196.1
A0A494BA	Leucine-ric	2.321	2	1	1	1	372	41.9
P70451	Tyrosine-p	2.319	1	1	14	1	823	94.5
A2AFJ4	Gamma-ta	2.317	4	1	3	1	202	23.4
P62307	Small nucl	2.317	9	1	1	1	86	9.7
Q9WV91	Prostaglar	2.314	1	1	1	1	879	98.7
Q8CJ27	Abnormal	2.309	1	1	1	1	3122	364
Q4ZJN1	Compleme	2.305	3	1	1	1	333	34.5
F7CTH4	Zinc finger	2.301	5	1	1	1	551	62.8
P49452	Centromer	2.301	1	1	1	1	906	102.1
Q6ZWQ7	Signal pep	2.297	4	1	1	1	180	20.3
D3YUP5	Exocyst co	2.294	1	1	7	1	789	87.1
Q5SUR0	Phosphoril	2.294	1	1	2	1	1337	144.5
Q8BGD6	Sodium-co	2.293	2	1	5	1	560	63.4
Q9JLN9	Serine/thr	2.292	1	1	1	1	2549	288.6
Q8R4K2	Interleukir	2.289	3	1	2	1	459	50.8
A0A0N4SV	Guanine n	2.287	8	1	14	1	83	9.2
P50462	Cysteine a	2.284	9	1	1	1	194	20.9
Q8C8N2	Protein SC	2.28	1	1	1	1	606	70.2
Q4VAC9	Pleckstrin	2.28	1	1	7	1	1341	148.4
H3BKF3	PAN2-PAN	2.28	2	1	2	1	887	95.4
Q60992	Guanine n	2.279	1	1	1	1	868	99.9
P04925	Major pric	2.278	4	1	5	1	254	28
Q7TPC1	Corneodes	2.277	2	1	1	1	561	54.3
Q9QZR9	Collagen a	2.275	1	1	3	1	1682	164
V9GX00	Interleukir	2.275	2	1	2	1	856	95.3
A0A087W	Aspartyl a	2.271	8	1	6	1	85	9.5
Q62240	Lysine-spe	2.271	1	1	2	1	1548	176.9
P97503	Homeobox	2.269	3	1	1	1	333	35.1
Q06186	Proheparir	2.266	4	1	1	1	208	22.8
Q9JLJ1	Selenoprot	2.265	9	1	2	1	94	10.6
Q68FG3	Protein SP	2.261	2	1	2	1	682	74.8
A0A0R4J0	Cysteine p	2.259	4	1	2	1	393	44.4
Q8BVD7	Compleme	2.254	2	1	12	1	289	30.5
Q9D706	RNA polym	2.249	1	1	1	1	660	74.1

P59017	Bcl-2-like	2.247	4	1	1	1	434	46.7
O70468	Myosin-bi	2.245	1	1	4	1	1270	140.5
E9Q8D5	CCR4-NOT	2.244	2	1	1	1	490	54.2
Q9D1W4	RIKEN cDN	2.244	8	1	1	1	143	15.7
P97358	TATA box-	2.243	2	1	4	1	586	67.9
Q7TQK4	Exosome c	2.243	3	1	2	1	274	29.5
B2RXC8	3222402P1	2.24	1	1	1	1	1150	130.2
Q9JLM8	Serine/thr	2.239	1	1	1	1	756	84.1
Q8C3X4	Translatio	2.234	2	1	4	1	651	72.4
O88327	Alpha-catu	2.232	1	1	1	1	731	81.4
Q9CXF7	Chromodo	2.231	2	1	1	1	900	101.4
Q3TCH7	Cullin-4A (	2.231	1	1	3	1	759	87.7
A6PW28	Rho family	2.228	1	1	3	1	1053	116.3
Q61830	Macropha	2.226	1	1	1	1	1456	164.9
V9GXE0	Meiosis-sp	2.224	3	1	10	1	219	26.8
Q0VBM2	Protein FA	2.223	1	1	1	1	1012	114.6
P52624	Uridine ph	2.222	7	1	1	1	311	34.1
B7ZP08	AT-rich int	2.222	2	1	1	1	379	40.5
D3YXP6	Phosphom	2.222	5	1	1	1	158	17.9
P70324	T-box tran	2.222	1	1	2	1	741	79.1
Q6P5D4	Centrosom	2.217	1	1	2	1	1140	133.3
A0A1L1SQ	FAT atypic	2.217	0	1	2	1	4645	511.9
Q9WVL3	Solute car	2.215	1	1	5	1	1083	119.4
E9QP00	Transform	2.211	2	1	8	1	282	32.6
Q5H8C4	Vacuolar p	2.21	0	1	2	1	3166	359.2
A6PWZ2	Eukaryotic	2.206	31	1	1	1	58	6.5
A0A087W	Nuclear ub	2.203	6	1	1	1	233	26.2
E9Q564	Olfactory r	2.203	3	1	1	1	316	35.5
E0CYF3	Nuclear tra	2.197	2	1	6	1	341	36.3
A0A0R4J0	Leukocyte	2.197	5	1	2	1	151	16.4
Q6ZPG2	WD repeat	2.196	1	1	1	1	1874	202.8
Q99N87	28S riboso	2.191	2	1	2	1	432	48.2
Q9Z1M7	LARGE xyl	2.19	3	1	5	1	756	87.9
Q80YW5	B box and	2.189	2	1	1	1	473	52.7
O54692	Centromer	2.187	2	1	1	1	779	88
A2AS70	Protein AF	2.187	2	1	1	1	990	104.5
Q8BVT3	Glutamate	2.186	3	1	2	1	517	58.9
Q6NZJ3	Zinc finger	2.185	2	1	1	1	561	64
P97875	Jun dimeri	2.184	4	1	1	1	163	18.7
A2TJV2	Paralemm	2.181	2	1	1	1	734	78.7
Q91YD9	Neural Wi	2.178	2	1	7	1	501	54.2
F7BWC1	Tyrosine--	2.178	16	1	1	1	125	14.1
Q8BH47	Vesicle-tra	2.177	4	1	1	1	307	35
Q99MP8	BRCA1-ass	2.176	2	1	4	1	591	66.9
A0A140LIV	FERM and	2.175	1	1	2	1	1774	195.2

J3QPC9	CTD nuclea	2.171	21	1	1	1	89	10
Q04690	Neurofibro	2.17	0	1	1	1	2841	319.4
Q8VEB3	Macropha	2.168	4	1	8	1	207	23.1
Q9EQG9	Ceramide	2.167	1	1	1	1	624	71.1
Q3U6K5	Spermatog	2.166	2	1	1	1	488	56.1
F7DF70	Phosphory	2.165	4	1	3	1	314	35.6
Q3V3Q4	Pyrin dom	2.164	1	1	3	1	588	63.8
Q8R344	Coiled-coil	2.164	4	1	2	1	166	18.9
Q9QZS7	Nephrin O	2.163	1	1	1	1	1256	136.3
Q9JHI8	NADPH ox	2.163	1	1	1	1	578	66.5
Q9CS74	Protein ec	2.162	2	1	2	1	641	71.7
Q9Z2A0	3-phospho	2.162	3	1	2	1	559	63.7
Q7TR54	Olfactory	2.161	2	1	1	1	326	36.8
Q8CE08	Prostatic a	2.16	2	1	1	1	381	43.7
O09044	Synaptoso	2.16	3	1	4	1	210	23.2
Q8BPB0	MOB kinas	2.159	3	1	2	1	216	25.1
P97440	Histone R	2.159	3	1	1	1	275	31.6
E9Q8A3	Phosphati	2.157	1	1	6	1	828	92.8
Q8CEZ4	Protein ma	2.155	2	1	1	1	452	50.5
G3UYV4	Transcripti	2.155	4	1	1	1	369	39.5
Q6NSQ7	Protein LT	2.154	1	1	4	1	470	54
Q4LDD4	Arf-GAP w	2.154	1	1	3	1	1452	162.2
Q8BWQ4	Cap-specif	2.152	1	1	1	1	767	87.1
P49182	Heparin co	2.148	1	1	1	1	478	54.5
Q80U63	Mitofusin-	2.147	1	1	3	1	757	86.1
B0QZT2	Dehydroge	2.147	5	1	2	1	326	35.7
Q5U458	DnaJ hom	2.146	1	1	2	1	559	63.2
Q9D8T2	Gasdermi	2.146	2	1	3	1	487	53.2
Q80Y14	Glutaredo	2.146	8	1	4	1	152	16.3
Q9ERS2	NADH deh	2.143	6	1	1	1	144	16.8
Q80XJ3	Tetratrico	2.142	0	1	1	1	2450	267.3
Q9JJY4	Probable A	2.139	2	1	1	1	825	91.7
Q9QXA5	U6 snRNA	2.138	5	1	2	1	137	15.1
Q3UNV2	Cytochrom	2.136	3	1	1	1	504	57.5
Q8CDM8	FHF comp	2.135	1	1	1	1	764	86
Q9DCJ5	NADH deh	2.133	8	1	1	1	172	20
Q9Z2G9	Oxidoredu	2.13	3	1	1	1	242	26.9
A0A0N4SV	C->U-editi	2.128	7	1	3	1	113	13.5
Q8BGB2	Tetratrico	2.127	1	1	1	1	858	96.1
Q9CQL7	MORF4 fa	2.126	6	1	1	1	125	14.2
Q3U213	Protein SE	2.126	2	1	1	1	654	73.9
Z4YJP0	Hypermeth	2.125	2	1	3	1	893	94.3
Q3U2A8	Valine--tR	2.123	2	1	2	1	1060	118.4
Q80U95	Ubiquitin-	2.123	1	1	2	1	1083	123.9
Q91YP1	RNA 5'-mo	2.119	5	1	1	1	285	32

A0A0G2JD	Alpha-S2-c	2.119	6	1	1	1	132	15.6
Q8BH60	Golgi-asso	2.118	2	1	1	1	463	50.6
Q91WF7	Polyphosp	2.117	1	1	1	1	907	103.4
E9PUP1	Synapse de	2.116	1	1	1	1	1314	144.5
A0A338P7	Diphospho	2.116	9	1	8	1	77	8.7
G3XA69	Myotubula	2.115	4	1	3	1	159	17.6
P59240	Nephrocys	2.113	1	1	1	1	1425	157.2
Q99NF3	Centrosom	2.113	5	1	1	1	373	41.4
A2AJQ0	[Pyruvate (	2.111	2	1	1	1	597	67.6
A0A0N4SV	Anthrax to	2.111	1	1	4	1	500	56
Q63850	Nuclear po	2.11	3	1	1	1	526	53.2
Q9WUK2	Eukaryotic	2.11	3	1	12	1	248	27.3
O88848	ADP-ribosy	2.108	7	1	2	1	186	20.9
A0A1Y7VJ	WD repeat	2.107	1	1	1	1	710	77.2
F7CV40	Methyl-Cp	2.103	11	1	2	1	111	12.3
Q0VF55	Calcium-tr	2.102	1	1	3	1	1220	134.3
A2ATY4	Zinc finger	2.102	2	1	1	1	518	57.6
E9Q4Y4	Centrosom	2.102	0	1	1	1	2514	276.2
A2AKD7	Alpha-1-sy	2.1	1	1	2	1	499	53.2
O08663	Methionin	2.1	1	1	2	1	478	52.9
A0A1B0GF	Stromal in	2.099	2	1	3	1	540	62.2
A0A0U1RF	AP-2 comp	2.098	12	1	25	1	60	7.1
O35125	Leucine-ric	2.098	2	1	7	1	340	39.3
Q9CQQ4	Gem-asso	2.095	3	1	6	1	269	30.4
A0A140LIX	Ras-relate	2.094	7	1	1	1	188	19.7
Q64105	Sepiapteri	2.093	3	1	5	1	261	27.9
Q9EQC0	Carbohydr	2.092	4	1	1	1	411	46.9
Q9CTH6	rRNA-proc	2.092	5	1	6	1	198	23.3
D3YYQ8	Dynein, ax	2.091	0	1	3	1	4591	529.2
Q9JMA9	Sodium- a	2.091	3	1	7	1	638	71.4
P16374	Zinc finger	2.091	1	1	2	1	707	82.2
Q8BN21	Serine/thr	2.09	3	1	1	1	503	58.1
Q8R3C1	Uncharact	2.087	2	1	3	1	574	64
Q9Z2I8	Succinate-	2.086	2	1	2	1	433	46.8
Q8K114	Integrator	2.083	3	1	3	1	658	74
Q8R2M0	Transcript	2.079	3	1	5	1	207	24.2
Q03526	Tyrosine-p	2.076	1	1	1	1	625	72.2
Q6NY15	Testis-spe	2.074	1	1	3	1	697	81.2
B1AWG4	Leucine-ric	2.071	0	1	6	1	3298	366.5
Q80VP1	Epsin-1 OS	2.07	1	1	3	1	575	60.2
D3Z4U5	Janus kina	2.07	2	1	1	1	831	97.2
A0A1L1SV	NTPase KA	2.063	1	1	1	1	675	75.5
A2AUC9	Kelch-like	2.061	1	1	1	1	606	68.1
P28843	Dipeptidyl	2.061	1	1	1	1	760	87.4
V9GXS9	Reproduct	2.06	9	1	1	1	159	17.6

O35566	CD151 ant	2.06	3	1	4	1	253	28.2
Q9Z1R4	Uncharact	2.057	6	1	1	1	293	32
Q8CGY8	UDP-N-ace	2.057	1	1	1	1	1046	116.9
F6VAN0	Cyclic AMF	2.057	2	1	2	1	656	72.6
Q80XH2	Interphoto	2.056	1	1	2	1	1243	138
P30415	NK-tumor	2.053	1	1	2	1	1453	163.4
A2A432	Cullin-4B (	2.052	1	1	1	1	970	110.6
G3UY49	Calumenir	2.052	11	1	1	1	141	16.5
A2AVZ9	Solute car	2.051	4	1	2	1	502	56
Q8BGQ7	Alanine--t	2.05	1	1	2	1	968	106.8
A0A286YD	RAS p21 p	2.05	2	1	1	1	497	56.9
Q9WTX2	Interferon-	2.049	3	1	2	1	313	34.3
Q3TWF6	WD repeat	2.046	2	1	2	1	657	73.1
A0A0B4J1	Immunogl	2.045	11	1	1	1	120	13.1
B1AU71	DBF4-type	2.042	14	1	13	1	50	6
Q8CGF7	Transcript	2.04	1	1	1	1	1100	123.7
O08749	Dihydrolipo	2.038	3	1	2	1	509	54.2
Q8CC88	von Willeb	2.036	1	1	1	1	1905	213.3
Q8C2E7	WASH con	2.03	1	1	1	1	1159	134
P46414	Cyclin-dep	2.03	7	1	1	1	197	22.2
A2AIL4	NADH deh	2.03	4	1	2	1	333	38.3
A0A0U1RF	BRCA2-int	2.029	1	1	1	1	735	77.5
Q9DAX9	Amyloid p	2.026	4	1	1	1	585	66.8
Q99NA9	Polycomb	2.021	2	1	1	1	353	39.8
Q9CQY7	Monocyte	2.021	3	1	5	1	238	27.7
A0A1Y7VN	Dehydroge	2.02	2	1	1	1	362	40.7
E9Q3I3	Spermatog	2.018	1	1	1	1	1244	138
E9PWH3	Amyloid-b	2.017	3	1	1	1	738	80.6
D3YUQ2	CPED1 OS=	2.017	1	1	9	1	473	53.8
Q6RFH4	Germinal	2.016	9	1	2	1	181	21
A0A0A6YV	SURF1-like	2.014	3	1	1	1	240	27.1
A0A140LJF	Ryanodine	2.014	0	1	1	1	4858	550.5
Q80X68	Citrate syr	2.014	2	1	4	1	466	52.3
Q0VGT3	Pogo trans	2.013	1	1	2	1	1400	154
Q80W49	Beta/gam	2.012	2	1	1	1	1005	114.2
Q9WVF7	DNA polyn	2.01	0	1	1	1	2283	261.9
Q61712	DnaJ homo	2.006	3	1	1	1	552	63.8
Q6NZH9	RAS, guan	2.005	1	1	1	1	691	78.1
Q9JI44	DNA meth	2.004	3	1	2	1	468	53.1
A2AQD6	Protein ITF	2.003	1	1	2	1	1219	133.4
Q8BVQ5	Protein ph	2.001	6	1	1	1	386	42.2
Q6P3Y5	Zinc finger	2	1	1	6	1	742	83.1
Q64374	Regucalcir	1.999	3	1	4	1	299	33.4
Q8K0Q5	Rho GTPas	1.998	1	1	2	1	663	74.9
Q7TQK0	Cyclin-T2 (	1.997	1	1	3	1	723	80.2

Q8VC16	Leucine-ric	1.996	3	1	3	1	493	54.9
Q80YA3	Phospholip	1.995	1	1	2	1	547	61.8
D3YVU0	Ubiquitin c	1.994	5	1	1	1	339	39.5
A2BDW0	Tyrosine-p	1.994	2	1	1	1	483	56
Q8BXJ2	Transcripti	1.993	2	1	2	1	1205	132.3
Q9D8C4	Interferon-	1.992	2	1	4	1	286	31.9
Q9WUR2	Enoyl-CoA	1.99	2	1	1	1	391	43.2
Q8BG30	Negative e	1.99	4	1	2	1	530	57.5
Q9CWZ7	Gamma-so	1.987	3	1	5	1	312	34.7
Q9D3B1	Very-long-	1.985	4	1	1	1	254	28.4
A0A0G2JE	Piwi-like p	1.983	3	1	1	1	493	56.8
Q8BJ64	Choline de	1.983	1	1	2	1	596	66.4
A0A1Y7VN	Zinc finger	1.982	2	1	14	1	253	28.1
Q9WTL7	Acyl-prote	1.98	3	1	5	1	231	24.8
P40645	Transcripti	1.979	2	1	1	1	827	91.7
P54923	[Protein A	1.978	2	1	3	1	362	40
P21552	Protein W	1.976	2	1	3	1	360	40.5
Q80SY5	Pre-mRNA	1.974	1	1	1	1	542	63.7
Q8BFV2	PCI domai	1.973	2	1	3	1	399	46.1
Q8BJF9	Charged m	1.973	5	1	3	1	213	23.9
D3Z4N1	Rhotekin (	1.97	3	1	2	1	217	24
D3YWK1	WD repeat	1.969	4	1	2	1	425	46.4
Q9QXH4	Integrin al	1.969	1	1	1	1	1169	129.1
H3BKL6	Melanoma	1.968	3	1	1	1	701	79.3
A2A7B5	PR domain	1.967	1	1	1	1	1709	187.1
E9QJS7	Adhesion G	1.967	1	1	2	1	818	90.4
B1AVH7	TBC1 dom	1.965	1	1	1	1	922	104.2
A0A0R4J1	Histone de	1.965	1	1	1	1	588	65.6
Q5KU39	Vacuolar p	1.965	1	1	2	1	853	98.5
E9Q2M9	WD repeat	1.965	0	1	2	1	3183	354.1
A0A0G2JG	Phosphogl	1.964	21	1	1	1	33	3.7
E0CXC2	E3 ubiquit	1.964	1	1	1	1	719	81.3
Q8CB87	Ras-relate	1.963	1	1	2	1	973	106.3
Q0VF22	Coiled-coil	1.961	1	1	1	1	680	77.9
Q6Q477	Plasma me	1.961	1	1	1	1	1205	133
Q8R307	Vacuolar p	1.96	1	1	4	1	973	110.1
E9Q7U2	Calcium-bi	1.959	2	1	2	1	633	70.9
E9QN37	Macrophag	1.959	1	1	5	1	720	79.1
A0A498W	UBX doma	1.958	3	1	1	1	262	29.9
Q8BWR4	Ubiquitin c	1.958	1	1	1	1	1235	139.9
H3BJQ0	Ceramide	1.956	3	1	1	1	251	29.1
B0R0U1	Methyltrar	1.954	5	1	1	1	341	38.9
Q9ESJ4	NCK-intera	1.953	2	1	1	1	714	78.5
Q8CHP5	Partner of	1.953	4	1	1	1	203	22.7
Q07079	Insulin-like	1.953	4	1	1	1	271	30.4

A0A1B0GS	Ribonuclea	1.952	2	1	4	1	492	53.9
Q6P9L6	Kinesin-lik	1.95	1	1	1	1	1387	160
Q9QZF2	Glypican-1	1.946	2	1	1	1	557	61.3
Q91YN9	BAG famil	1.944	4	1	1	1	210	23.5
Q3UKT9	N-acetyltra	1.944	3	1	1	1	344	39
Q9CXU0	Mediator c	1.943	7	1	1	1	135	15.7

calc. pI	Score Sequ	# Peptides	# Razor Pe	Abundance	Abundance	Abundance	Abundance	Abundance
5.66	49518.04	198	48	1.1	0.716447	0.960634	0	103.6
5.73	29258.23	167	108	0.937	0.327172	0.806293	0	112.7
5.96	13001.9	253	4	2.444	0.006642	0.082054	0	57.8
5.55	23303.42	145	5	1.103	0.724523	0.96164	0	100.5
5.41	5288.64	189	146	1.699	0.189539	0.663939	0	78.7
5.76	16954.87	111	0	0.555	0.00114	0.020072	0	126.6
5.48	17095.66	131	0	0.905	0.163328	0.629076	0	110
5.48	17167.67	131	107	1.156	0.86348	0.981617	0	95.5
5.57	15511.38	124	0	1.13	0.7943	0.97006	0	95.7
5.35	7235.84	136	135	2.069	0.038303	0.276649	0	82.8
5.33	7176.69	136	0	2.065	0.04412	0.299906	0	65.2
5.4	4723.19	155	3	1.687	0.199155	0.677163	0	77.7
5.58	6094.88	110	3	2.003	0.051484	0.32867	0	72
6.3	4339.05	116	0	1.502	0.402699	0.862461	0	73.2
6.48	8642.17	76	3	0.977	0.412383	0.864761	0	111
6.04	4799.98	87	8	1.6	0.280066	0.76495	0	89
5.33	3550.02	91	2	1.862	0.09587	0.479124	0	69.6
9.35	8697.13	68	1	1.279	0.827515	0.974887	0	95.5
6.18	3431.82	83	12	1.421	0.532454	0.90897	0	88.8
5.45	1602.83	101	0	1.582	0.299083	0.788679	0	37.7
6.28	2713.98	74	0	1.109	0.739528	0.96414	0	101.1
9.26	6922.3	61	59	1.139	0.818783	0.973737	0	94.9
9.14	6796.91	61	0	1.171	0.832326	0.976224	0	94.6
6.42	1856.2	84	0	1.332	0.707937	0.959206	0	92.9
8.51	3682.03	57	0	1.105	0.728805	0.962129	0	99.7
6.87	2290.64	52	3	3.814	9.45E-06	0.000254	0	42.2
6.67	3935.84	46	0	5.716	8.09E-11	2.9E-09	0	35.6
6.64	3879.01	46	44	1.647	0.233021	0.711914	0	82.5
9.41	3764.77	53	2	1.073	0.646797	0.940359	0	99.8
4.89	3225.85	38	0	1.632	0.246866	0.726377	0	77
5.73	1357.91	52	0	1.57	0.360899	0.838257	0	86.1
5.76	4283.32	38	1	0.84	0.096581	0.480956	0	113.8
4.72	5321.62	44	41	0.959	0.373282	0.847074	0	104.9
7.14	2172.78	43	7	1.716	0.176881	0.64543	0	62.6
5.69	1469.55	42	0	1.811	0.119123	0.536734	0	66.4
7.59	5564.92	27	0	0.767	0.079542	0.430417	0	108.8
4.92	752.91	47	0	1.509	0.392483	0.861697	0	77.8
7.83	3566.67	33	0	1.734	0.164305	0.630832	0	74.2
7.43	1137.74	47	0	3.631	2.24E-05	0.000566	0	10.2
8.95	1729.12	40	0	1.761	0.14671	0.598768	0	74.4
8.4	1063.88	46	1	1.318	0.739295	0.96414	0	91
7.12	1920.83	47	1	1.197	0.969165	0.997215	0	90.1

7.93	2104.7	38	0	1.229	0.949395	0.991422	0	96
5.41	1669.07	34	0	0.996	0.456634	0.890836	0	98.6
4.78	4851.38	40	2	0.851	0.176362	0.64543	0	114.6
6.28	1792.38	38	0	2.113	0.031365	0.243738	0	57
6.06	1560.32	39	0	1.018	0.507778	0.90897	0	101.6
6.87	1922.77	41	0	1.862	0.095829	0.479105	0	68.4
5.38	1660.62	36	33	1.074	0.648549	0.941124	0	105.4
8.92	1806.54	33	1	1.38	0.609721	0.927586	0	91.2
5.12	2222.98	34	3	1.873	0.091271	0.46632	0	66.6
7.09	1039.56	52	0	0.852	0.177444	0.645798	0	112.4
6.87	1412.82	40	0	3.886	6.77E-06	0.000186	0	18.4
6.09	1872.57	32	0	1.911	0.077398	0.421975	0	61.7
8.48	1576.39	36	0	1.5	0.405682	0.862461	0	87.9
7.09	1165.3	38	4	1.17	0.899586	0.987004	0	98.1
8.92	4103.12	31	9	1.978	0.057632	0.351479	0	70.6
4.77	3619.52	37	16	1.113	0.751275	0.96537	0	100.5
7.18	2579.36	30	19	1.452	0.479771	0.898839	0	83.4
5.81	3743.58	31	0	3.233	2.31E-05	0.000581	0	92.3
9.11	1665.76	36	0	1.774	0.139198	0.582954	0	64.3
8.47	2440.73	30	3	0.97	0.396569	0.861859	0	100
4.72	4370.56	30	0	1.013	0.495495	0.906872	0	107
5.14	2509.21	39	1	0.727	0.050221	0.324724	0	129.2
4.96	742.31	34	1	2.038	0.044031	0.299465	0	35.9
6.6	3269.71	38	12	0.88	0.221239	0.701127	0	106.9
8.4	1806.86	30	0	0.998	0.459489	0.891873	0	108.5
5.5	659.45	35	0	1.41	0.553076	0.910945	0	84.2
4.69	3614.07	35	0	1.158	0.867953	0.981617	0	99.4
5.31	4825.81	23	0	1.951	0.065022	0.379785	0	66.9
6.24	2440.93	29	0	1.452	0.479913	0.898839	0	81.4
6.18	2955.18	22	15	1.097	0.709279	0.959398	0	100
5.33	1873.8	29	3	1.068	0.634315	0.936153	0	108.3
9.17	2360.9	39	7	1.057	0.60646	0.926994	0	100.4
8.84	3092.5	25	0	1.617	0.26167	0.746056	0	82.2
6.92	1179.22	34	2	1.186	0.940691	0.991397	0	98
4.81	4002.68	31	3	0.994	0.451215	0.888233	0	99.7
6.58	1385.49	27	1	1.114	0.753986	0.96597	0	5.5
5.68	1092.37	25	0	1.298	0.782637	0.97006	0	5.4
6.65	698.17	40	0	1.216	0.981447	0.997735	0	98.6
7.75	669.41	35	0	1.479	0.436997	0.88436	0	78.6
6	1555.5	33	0	1.206	0.993324	0.99978	0	100.5
4.75	3680.88	32	0	1.253	0.899506	0.987004	0	90.1
6.67	1762.36	34	7	1.166	0.8896	0.984715	0	98.2
9.88	2387.53	18	5	1.245	0.910197	0.98846	0	91.9
4.77	3832.39	28	0	0.971	0.397654	0.862453	0	99.4
8.59	1199.66	31	0	1.04	0.562156	0.912326	0	87.1

7.88	2462.36	27	0	1.212	0.992231	0.999087	0	96.2
7.74	1101.16	32	0	1.183	0.867092	0.981617	0	90.5
7.62	1154.34	31	0	0.958	0.36978	0.846091	0	106
8.53	787.15	35	0	1.173	0.909232	0.988314	0	88.6
7.83	1070.03	32	29	1.429	0.519349	0.90897	0	88.5
5.39	7390.8	21	0	0.987	0.435114	0.88312	0	95.5
8.91	2366.33	28	23	1.003	0.472993	0.898019	0	102.3
5.64	1510.27	31	0	2.094	0.034228	0.258024	0	60.5
6.73	605.29	30	0	1.35	0.669282	0.95015	0	102.3
7.53	859.62	30	0	1.216	0.980699	0.997735	0	5.9
6.37	475.97	42	0	1.098	0.702546	0.958512	0	103.9
4.82	807.04	22	2	1.257	0.880466	0.983083	0	82.3
4.89	3029.3	19	4	1.563	0.321304	0.803178	0	77.9
4.89	2863.2	19	0	1.079	0.613396	0.929068	0	93.3
6.9	1189.25	21	0	1.02	0.513328	0.90897	0	95.9
5.71	676.09	33	1	1.344	0.681635	0.951658	0	99.1
10.15	1675.8	29	27	1.291	0.799803	0.97006	0	99.3
8	603.24	35	0	1.065	0.625848	0.932683	0	104.5
6.3	1473.15	23	0	1.469	0.452139	0.888233	0	75.1
7.25	1007.51	26	0	0.846	0.169003	0.636618	0	108.5
7.11	629.33	26	0	0.884	0.227773	0.705743	0	110.7
6.92	2349.35	16	0	1.035	0.558194	0.910977	0	102
9.1	807.45	26	0	1.39	0.590427	0.924028	0	77.9
6.2	1593.38	31	0	0.865	0.19653	0.673222	0	101.8
7.96	2303.63	25	0	1.356	0.647469	0.940571	0	89.2
8.44	607.41	30	0	1.626	0.253301	0.734875	0	78.9
9.98	1033.47	23	0	1.003	0.472418	0.898019	0	111
6.25	763.98	27	1	1.598	0.28232	0.76495	0	68.8
6.39	1871.38	23	2	1.105	0.729481	0.962129	0	91.4
10.04	1582.96	28	0	0.878	0.169618	0.637245	0	101
5.38	1171.13	25	14	1.183	0.934157	0.991048	0	89.9
4.68	2291.78	26	0	1.039	0.559901	0.911314	0	97.9
8.59	2529.43	27	0	1.349	0.672684	0.95081	0	90.1
7.56	691.39	31	1	1.168	0.89554	0.986348	0	60.6
6.14	846.35	30	0	0.848	0.172642	0.640909	0	110.2
5.59	1067.34	29	0	1.486	0.426501	0.874364	0	30.3
8.41	769.52	27	0	1.812	0.118713	0.535654	0	56
10.56	3198.66	20	0	0.941	0.334729	0.813024	0	99.7
8.44	2325.13	19	0	1.565	0.319022	0.802274	0	76
10.21	2432.6	21	0	1.039	0.560559	0.911389	0	101.8
7.46	1573.85	22	0	1.085	0.676078	0.951158	0	97.8
4.89	3467.19	19	0	1.405	0.562552	0.912724	0	86.3
9.96	1029.2	21	0	1.599	0.280819	0.76495	0	87
8.18	2022.96	22	0	1.652	0.228586	0.70719	0	80.2
5.49	1133.61	24	22	1.088	0.684146	0.952102	0	95.1

4.89	3267.43	19	18	1.511	0.390146	0.859539	0	77.2
8.22	306.28	35	0	1.919	0.088058	0.455262	0	55.1
7.36	1380.99	20	0	0.642	0.014793	0.148158	0	129.8
5.39	1072.96	23	1	0.739	0.067854	0.388103	0	115
8.84	609.33	36	0	1.759	0.14824	0.60077	0	71.4
9.66	3626.66	23	0	0.93	0.313374	0.799567	0	100.5
9.7	2638.93	26	3	1.071	0.640456	0.937741	0	99.5
9.36	2601.76	22	0	1.284	0.816448	0.972384	0	102.8
10.15	7977.19	18	0	1.103	0.723579	0.96164	0	93.8
5.55	606.64	23	0	1.84	0.105261	0.503662	0	64.4
7.28	555.85	31	0	1.287	0.80956	0.970418	0	85.4
8.92	804.63	26	0	1.325	0.72387	0.96164	0	92.6
4.77	1296.73	22	0	1.173	0.907171	0.988314	0	93
8.63	698.15	28	0	1.019	0.510917	0.90897	0	99.3
8.27	1221.38	21	0	0.869	0.203657	0.682501	0	108.9
5.52	1726.1	22	8	1.497	0.409244	0.862461	0	82.3
7.53	832.32	30	0	1.226	0.957522	0.992528	0	97.5
6.15	498.81	17	0	16.494	1E-17	3.97E-16	0	11.4
7.47	747.26	26	0	1.278	0.828808	0.975565	0	86.7
5.85	2334.22	14	0	1.127	0.786595	0.97006	0	102
6.79	1264.06	25	1	1.615	0.264042	0.748141	0	76.4
5.41	608.73	25	0	1.523	0.373155	0.847074	0	69.5
6.51	1355.17	20	2	1.199	0.974081	0.997505	0	87.8
4.82	1099.24	13	11	1.498	0.408612	0.862461	0	29.6
6.23	832.98	24	5	1.7	0.188938	0.663939	0	71.4
7.94	392.31	28	0	1.662	0.219928	0.700211	0	56.6
7.68	1131.55	21	3	1.017	0.506366	0.90897	0	119.9
6.6	512.91	24	0	1.759	0.148338	0.600844	0	51.1
6.06	465.91	30	0	1.324	0.726102	0.96164	0	79.1
9.73	2839.78	21	0	1.473	0.445864	0.888233	0	93.5
6.34	506.67	18	16	77.551	1E-17	3.97E-16	0	1.6
7.4	1058.48	30	3	1.046	0.576797	0.919586	0	99.5
7.23	438.19	30	0	2.081	0.036198	0.266599	0	58.9
5.55	2014.13	15	3	1.319	0.736013	0.962867	0	86.2
5.39	3141.04	21	0	0.07	1E-17	3.97E-16	0	192.3
6.92	1756.25	18	13	1.056	0.602349	0.925915	0	104.1
6.58	1024.12	23	0	1.103	0.724301	0.96164	0	91.4
8.31	496.74	28	0	1.521	0.376354	0.850309	0	66.9
7.21	1524.19	24	0	1.127	0.787008	0.97006	0	98.3
9.07	1072.46	27	0	1.348	0.674373	0.951158	0	93.4
8.9	569.22	26	0	1.318	0.738145	0.96414	0	84.8
6.38	1267.73	20	4	1.703	0.186201	0.66049	0	70.3
5.45	418.19	21	0	1.933	0.070445	0.397148	0	93.9
9.39	645.39	25	0	1.481	0.43432	0.882876	0	92.4
7.53	1611.65	24	0	1.486	0.453535	0.888233	0	59.5

5.05	2491.19	21	0	1.425	0.526236	0.90897	0	77.5
6.65	492.26	24	2	1.164	0.885683	0.983234	0	96
7.06	1147.73	25	5	0.826	0.142276	0.592887	0	117.9
7.37	790.81	20	0	1.216	0.980932	0.997735	0	85.8
11	1771.04	22	0	0.835	0.153805	0.61332	0	121
5.85	888.59	18	0	1.347	0.676697	0.951158	0	78.2
5.16	1013.07	16	0	5.557	4.11E-09	1.44E-07	0	21.6
7.96	872.2	22	0	1.425	0.525188	0.90897	0	76.4
5.83	380.92	24	0	1.014	0.484148	0.90176	0	100.2
5.94	1291.68	18	0	0.858	0.186976	0.66212	0	117.8
7.59	700.28	31	3	1.141	0.82533	0.974684	0	89.6
5.91	636.05	24	0	1.303	0.772006	0.97006	0	85.2
9.85	1933.09	12	0	1.077	0.656341	0.943998	0	98.4
5.77	892.4	18	0	1.146	0.836124	0.976616	0	96.3
5.83	497.61	27	0	1.368	0.632294	0.934814	0	83.1
5.27	402.11	20	1	1.928	0.072023	0.403429	0	44.5
5.06	1605.46	18	0	1.362	0.646297	0.940359	0	90.4
7.81	494.89	24	0	1.197	0.969214	0.997215	0	5.6
5.16	1399.99	21	0	1.403	0.565274	0.913241	0	82.7
9	820.48	19	0	1.339	0.692608	0.952781	0	96.2
9.74	838.44	19	0	1.23	0.94741	0.991422	0	88
6.34	424.89	17	0	68.643	1E-17	3.97E-16	0	1.4
6.98	594.38	21	3	1.487	0.425088	0.873992	0	93.9
7.71	555.18	23	0	1.334	0.703019	0.958655	0	83.9
9.67	544.18	23	4	2.266	0.015442	0.151397	0	51
8.02	932.32	22	0	1.204	0.987263	0.997793	0	103
5.03	1011.92	22	7	1.066	0.627005	0.932683	0	101.9
4.88	3001.5	16	0	0.962	0.354625	0.831572	0	102.7
4.54	2600.27	11	0	2.649	0.002912	0.044821	0	47.1
9.01	820.06	15	0	1.681	0.203423	0.682501	0	77.8
7.03	693.47	19	0	0.636	0.013422	0.138274	0	160.5
4.65	2897.51	12	8	1.07	0.639451	0.937605	0	111.7
7.65	705.15	25	0	1.141	0.824055	0.974465	0	91.5
4.73	778.62	12	0	0.909	0.253014	0.734875	0	98.7
4.81	3241.97	10	0	1.098	0.710468	0.959398	0	105.8
6.07	1332.13	12	0	1.62	0.25867	0.740595	0	75.2
9.29	1052.92	24	0	2.274	0.014831	0.148417	0	59.1
8.16	884.64	19	1	1.969	0.060082	0.358302	0	58
5.19	1369.48	18	12	1.365	0.63847	0.937605	0	82.7
8.15	712.78	22	0	0.841	0.162601	0.628295	0	106.7
6.99	360.41	26	1	1.907	0.078681	0.427128	0	59.6
6.79	445.4	20	1	1.72	0.174211	0.64202	0	14
7.34	766.78	22	0	1.34	0.691298	0.952547	0	84.7
6.46	585.28	26	4	1.661	0.220522	0.700517	0	64.7
5.77	488.99	21	1	1.664	0.218141	0.697874	0	65.1

6.89	663.49	16	3	1.66	0.221416	0.701127	0	71
6.83	630	18	6	1.444	0.492912	0.906679	0	81.4
8.65	1459.93	17	4	1.073	0.646469	0.940359	0	101
7.08	950.56	22	0	2.018	0.048117	0.315327	0	41
7.55	569.54	22	2	0.457	0.000177	0.003804	0	144.7
5.88	1195.27	17	0	1.004	0.473815	0.898019	0	101.5
8.12	1150.15	15	2	1.334	0.70389	0.958927	0	95
5.03	2058.41	15	1	0.826	0.14285	0.593057	0	112.8
5.14	2011.22	17	1	0.996	0.454987	0.889002	0	101
7.31	747.43	20	1	0.967	0.388932	0.859423	0	101.4
5.74	1563.57	15	0	1.313	0.792618	0.97006	0	97.4
11	3105.89	17	13	0.857	0.184381	0.656441	0	110.5
10.32	2922.32	13	0	1.001	0.466815	0.89585	0	105.4
7.69	1278.76	16	0	1.187	0.94403	0.991397	0	97.2
4.92	2165.01	11	4	1.413	0.547423	0.909321	0	94.2
6.55	270.08	18	0	0.805	0.083133	0.440653	0	123.1
9.38	617	20	0	1.046	0.576957	0.919639	0	102.5
9	1229.97	17	1	0.7	0.035431	0.263531	0	121
9.31	717.21	15	14	0.976	0.410685	0.863065	0	103.8
10.18	5599.62	12	0	1.273	0.840531	0.977862	0	85.5
9.77	749.45	21	0	1.153	0.854868	0.981617	0	109.3
6	522.61	21	0	1.306	0.766169	0.967627	0	87.5
6.1	1219.97	28	1	0.821	0.13558	0.575267	0	111.3
7.17	510.59	23	1	1.5	0.405537	0.862461	0	28.9
6.95	845.93	19	0	1.16	0.87366	0.983083	0	97.6
5.45	680.2	18	0	1.065	0.575354	0.919147	0	103.9
10.1	2016.09	12	0	1.201	0.980029	0.997735	0	101.5
7.53	582	17	1	1.199	0.975334	0.997505	0	95.6
5.05	544.4	21	0	1.047	0.580691	0.920669	0	105.4
5.1	604.11	19	0	1.309	0.758418	0.966419	0	87.4
7.96	476.27	21	7	0.93	0.312334	0.798743	0	96.8
8.06	685.4	14	0	1.05	0.478192	0.898527	0	108.5
7.23	1032.41	18	0	1.204	0.989145	0.998104	0	97.6
8.73	704.65	19	0	1.773	0.139899	0.585058	0	81.5
8.54	523.73	21	0	1.447	0.487476	0.903626	0	63
5.49	483.57	19	3	2.176	0.023417	0.205542	0	44.8
6.14	981.42	19	3	1.928	0.071972	0.403429	0	80.9
9.14	784.18	16	0	0.869	0.203622	0.682501	0	104
7.02	234.2	16	1	2.125	0.035644	0.264382	0	64.9
10.24	4387.91	15	0	0.994	0.451948	0.888233	0	98.7
6.7	439.34	19	0	1.621	0.258041	0.740472	0	75.8
9.55	1344.58	22	4	1.094	0.700597	0.95805	0	99.7
6.44	286.42	20	0	1.782	0.143478	0.594744	0	69.6
5.66	510.68	20	0	1.291	0.798657	0.97006	0	86.7
8.62	961.92	15	1	1.11	0.741776	0.964372	0	99.4

6.38	487.14	18	9	1.483	0.431186	0.879563	0	71.2
7.96	502.07	25	9	1.041	0.564456	0.913241	0	98.8
6.04	329.27	19	0	1.688	0.220843	0.700979	0	54.7
6.76	338.35	23	0	1.469	0.451559	0.888233	0	72
4.22	3566.68	13	0	1.134	0.805106	0.97006	0	98.3
9.38	939.3	14	0	0.972	0.401634	0.862461	0	101
7.52	452.66	21	0	1.652	0.228633	0.70719	0	70.7
6.73	319.89	16	0	73.642	1E-17	3.97E-16	0	1.2
8.72	796.78	18	0	1.132	0.800249	0.97006	0	94.5
8.34	561.93	16	0	1.332	0.708198	0.959275	0	93.4
6.58	368.15	24	0	1.238	0.925747	0.99022	0	87.8
6.02	944.35	16	0	1.141	0.82388	0.974465	0	92.5
8.29	641.16	21	0	2.211	0.019898	0.181907	0	57.5
8.35	574.24	16	0	1.297	0.786143	0.97006	0	80.1
11.11	3259.14	16	3	0.701	0.035866	0.265133	0	99.9
5.1	1365.13	16	4	0.795	0.106097	0.507084	0	112.6
10.05	710.6	15	0	1.31	0.810219	0.97073	0	86.2
4.75	878.95	18	0	0.917	0.2866	0.770335	0	110.8
8.95	549.93	16	2	2.025	0.046699	0.308092	0	69.7
7.93	400.91	17	0	1.569	0.315045	0.800259	0	77.4
8.88	235.71	21	0	1.681	0.204008	0.682501	0	69.1
6.8	339.38	24	0	1.031	0.54016	0.90897	0	89.8
6.84	495	18	0	1.495	0.41247	0.864761	0	81.2
6.61	445.78	19	0	1.336	0.699301	0.957318	0	82.4
7.27	395.72	15	0	1.393	0.584453	0.922551	0	86.2
5.22	777.73	19	0	0.944	0.268206	0.751491	0	109.6
11.03	3036.41	16	0	0.01	1E-17	3.97E-16	0	200
8.72	248.83	16	0	1.6	0.280055	0.76495	0	89.7
5.54	756.37	18	16	1.098	0.711726	0.959883	0	96.8
9.77	1693.6	16	0	1.212	0.991441	0.999059	0	95.3
5.86	512.73	19	1	1.137	0.814579	0.971737	0	90
5.34	560.22	20	9	1.135	0.807553	0.97006	0	99.2
6.05	305.61	19	0	1.752	0.152775	0.610761	0	72.6
8	296.06	21	9	1.003	0.472747	0.898019	0	99.5
11.03	2236.73	13	0	1.343	0.685346	0.952226	0	97.7
7.03	375.4	21	5	1.547	0.341841	0.820014	0	81.1
6.89	622.79	12	0	1.061	0.616474	0.930636	0	107.9
7.37	514.7	20	0	1.824	0.112849	0.525241	0	64.4
5.55	391.55	16	0	1.652	0.228261	0.706907	0	65.5
7.47	273.05	20	0	1.434	0.543187	0.90897	0	76.8
6.68	348.74	14	0	80.081	1E-17	3.97E-16	0	1.9
8.35	869.56	13	0	1.212	0.99242	0.999087	0	94.6
10.45	2749.34	12	0	1.1	0.716832	0.960634	0	97.6
9.09	669.91	15	0	1.069	0.557582	0.910977	0	92
9.51	456.03	15	0	1.239	0.924051	0.989893	0	91.6

5.35	247.55	20	9	1.748	0.172121	0.640909	0	61.6
5.52	1499.21	12	0	0.67	0.02282	0.201179	0	151.6
7.46	539.92	21	3	1.066	0.628295	0.932683	0	89.7
5.35	298.15	15	0	1.944	0.06721	0.386072	0	28
7.49	525.08	20	1	1.156	0.863811	0.981617	0	93.5
6.44	538.17	22	0	1.25	0.897873	0.987004	0	94.8
9.23	597.54	19	0	1.197	0.969753	0.997215	0	92.5
7.15	646.31	18	0	0.324	3.95E-07	1.25E-05	0	149.1
4.51	536.12	14	0	3.704	4.44E-06	0.000126	0	53.9
7.36	1310.8	15	0	1.155	0.859968	0.981617	0	96.2
8.69	367.5	17	0	56.488	1E-17	3.97E-16	0	1.2
7.06	309.64	20	0	1.735	0.163328	0.629076	0	60.9
9.14	654.6	16	0	1.431	0.515689	0.90897	0	82.5
5.02	485.53	15	0	2.245	0.017042	0.163447	0	51.2
6.7	897.54	16	0	1.213	0.98981	0.998535	0	89.1
6.93	682.05	20	0	0.96	0.374316	0.84814	0	91.1
7.69	799.59	10	1	1.311	0.753622	0.96597	0	92.5
6.96	365.5	20	0	1.592	0.303697	0.79167	0	72.8
6.54	351.66	19	0	1.035	0.551273	0.910041	0	113.3
8.32	355.91	22	0	1.26	0.871496	0.98284	0	76.5
5.27	709.88	19	0	1.082	0.670318	0.95015	0	94.9
5.68	667.52	17	0	1.205	0.947999	0.991422	0	85.8
7.03	790.95	20	7	1.153	0.856286	0.981617	0	89.1
6.84	424.66	15	1	66.295	1E-17	3.97E-16	0	2.6
8.27	442.22	16	0	1.623	0.256013	0.739039	0	74.1
7.84	467.4	18	0	0.874	0.210677	0.691171	0	104.5
7.46	243.55	17	0	1.078	0.608198	0.927253	0	94.4
9.45	296.33	14	0	1.661	0.220367	0.700356	0	77.2
6.48	537.56	16	0	0.898	0.252395	0.734489	0	107.8
6.48	269.15	16	0	1.235	0.933985	0.991048	0	78.4
8.97	1078.72	13	0	1.63	0.248982	0.729277	0	85.4
8.4	818.15	14	11	1.047	0.580221	0.920669	0	102.5
6.8	575.31	20	0	1.275	0.836079	0.976616	0	89.1
10.24	1021.7	12	0	1.119	0.765572	0.967498	0	93.2
6.64	651.71	11	6	5.166	2.16E-08	7.35E-07	0	30.6
9.58	684.65	13	0	1.011	0.491957	0.906372	0	97.9
9.54	1230.96	18	0	0.933	0.317385	0.801098	0	134.8
6.73	928.13	16	1	5.264	1.42E-08	4.87E-07	0	28.2
6.73	528.52	18	0	1.075	0.65183	0.942225	0	99.1
6.06	208.93	20	0	2.26	0.015829	0.154238	0	46.7
8.63	288.17	16	0	1.875	0.103027	0.499812	0	55.7
9.09	471.64	17	0	1.045	0.573769	0.919147	0	101.9
5.21	1478.09	13	0	0.971	0.398445	0.862453	0	120.2
6.98	302.5	12	0	1.678	0.220416	0.700356	0	76.8
9.88	3040.54	9	0	1.087	0.683314	0.951842	0	94.3

6.21	380.15	19	0	1.484	0.4288	0.876895	0	68
5.57	516.14	18	0	1.201	0.909304	0.988314	0	97.1
4.78	692.08	12	3	1.377	0.614719	0.929812	0	88
6.76	850.09	16	1	1.231	0.943901	0.991397	0	86.6
8.57	438.21	16	0	0.8	0.111914	0.522779	0	111.4
6.74	989.37	13	0	1.132	0.801606	0.97006	0	95
7.2	317.86	18	0	0.845	0.125268	0.55005	0	108.7
8.88	325.88	14	0	1.552	0.335558	0.81415	0	59.3
8.27	764.68	13	0	1.11	0.742261	0.964372	0	92.7
6.44	171.81	21	6	2.192	0.025701	0.215987	0	36.8
5.55	207.12	18	0	2.287	0.015771	0.153786	0	41.9
9.57	785.94	12	0	1.628	0.250833	0.734036	0	81.3
8.38	866.5	15	0	1.328	0.716734	0.960634	0	89.7
7.37	428.99	13	0	1.467	0.45602	0.889824	0	80.7
9.51	405.4	18	3	1.756	0.168372	0.636322	0	73.1
10.1	815.53	14	0	2.027	0.046247	0.306097	0	68.3
10.65	3746.89	18	0	0.96	0.373665	0.847732	0	109.2
8.1	1013.52	14	0	1.279	0.826441	0.974887	0	98.3
5.06	363.32	12	0	0.601	0.006333	0.079355	0	131.4
4.87	585.79	11	0	1.416	0.542629	0.90897	0	84.3
9.36	904.52	13	0	0.659	0.019441	0.17877	0	112.6
9.19	554.77	17	0	1.074	0.648115	0.940966	0	91
5	297.58	19	1	1.58	0.301765	0.791593	0	83.2
6.33	452.27	15	10	1.494	0.41415	0.865638	0	83.7
9.5	386.17	15	4	1.649	0.231081	0.710918	0	67.6
6.07	178.01	18	0	1.07	0.558415	0.910977	0	79.9
5.2	485.72	16	0	1.597	0.282982	0.765299	0	56.9
6	264.33	16	0	1.646	0.261882	0.746056	0	57.2
6.57	295.52	19	0	1.305	0.768598	0.968389	0	86.9
8.16	291.29	14	0	1.491	0.454591	0.888779	0	98.1
6.07	326.62	18	0	1.575	0.315122	0.800259	0	10
4.96	429.61	16	0	1.256	0.881844	0.983083	0	96.1
5.94	431.81	19	0	1.135	0.80777	0.97006	0	97.9
5.14	1081.48	12	0	1.06	0.613738	0.929068	0	95.1
6.98	469.16	18	1	1.193	0.959472	0.993976	0	89.5
5.33	645.37	16	4	1.014	0.498835	0.906872	0	101.8
5.05	466.38	12	0	1.011	0.49003	0.905226	0	100.5
9.86	735.26	18	0	1.442	0.496723	0.906872	0	73.7
5.19	224.45	17	0	1.313	0.861674	0.981617	0	84.2
5.36	346.6	16	0	1.323	0.72655	0.96164	0	82.9
5.1	695.17	12	0	0.966	0.387097	0.858404	0	96.1
5.39	255.66	13	0	1.341	0.688688	0.952468	0	86.8
4.88	275.6	16	2	1.483	0.460619	0.892334	0	56
8.69	423.65	12	0	1.383	0.604094	0.925915	0	83.4
8.18	384.52	14	1	1.086	0.679577	0.951158	0	105.3

8.95	261.56	14	0	1.133	0.80189	0.97006	0	93.2
5.06	896.84	9	0	1.134	0.805263	0.97006	0	87.3
6.74	441.73	9	0	1.547	0.340896	0.81884	0	76.8
10.29	807.91	12	0	1.187	0.945477	0.991408	0	89.3
6.42	543.39	13	0	1.143	0.828681	0.975565	0	91.2
8.5	757.94	13	0	1.189	0.908483	0.988314	0	87.6
10.21	1945.09	15	0	1.398	0.575343	0.919147	0	91.7
6.47	378.67	14	0	1.195	0.963999	0.995536	0	98.1
9.01	837.04	12	0	1.8	0.124667	0.548943	0	67.6
8.68	744.26	10	7	1.571	0.312657	0.798743	0	73.9
9.69	790.29	11	0	1.196	0.840475	0.977862	0	92
9.19	380.06	15	0	1.432	0.513649	0.90897	0	79.9
7.56	351.28	15	0	1.304	0.770707	0.970049	0	93.6
7.23	422.82	13	0	1.216	0.980415	0.997735	0	80.4
6.29	395.73	14	0	1.607	0.271817	0.756222	0	70.5
6.84	642.36	15	0	0.891	0.23933	0.718542	0	107.3
8.31	360.83	13	1	1.514	0.388133	0.859423	0	79.1
9.99	1037.11	14	0	1.02	0.513296	0.90897	0	97.2
5.8	675.92	14	0	1.264	0.862089	0.981617	0	91.9
6.52	217.69	21	2	1.347	0.675674	0.951158	0	104.1
9.88	546.43	15	0	1.858	0.097655	0.483817	0	59.4
7.14	298.31	11	0	0.76	0.038611	0.278395	0	136.4
9.25	462.38	13	0	1.362	0.64604	0.940359	0	93.9
6.62	375.26	16	1	1.231	0.943622	0.991397	0	88.3
4.84	157.95	18	0	2.462	0.00527	0.069476	0	35.5
8.07	618.03	12	0	1.263	0.865299	0.981617	0	93.4
7.2	235.71	16	0	1.775	0.154078	0.613633	0	53
10.71	2107.66	11	0	0.908	0.270329	0.753413	0	106.4
9.11	579.91	17	0	0.816	0.130467	0.561328	0	105.6
11.62	1747.7	11	0	0.911	0.274947	0.759196	0	100.7
6.27	209.56	19	0	1.051	0.463748	0.89433	0	96
9.52	307.89	13	0	1.442	0.496884	0.906872	0	76.8
7.99	681.71	9	0	2.027	0.036234	0.266599	0	33
9.35	336.53	14	0	2.078	0.036743	0.269872	0	74.9
7.59	336.29	18	14	1.523	0.372823	0.847074	0	66.1
6.27	308.93	17	3	1.228	0.952458	0.991422	0	78.8
10.7	1102.61	15	0	0.849	0.173564	0.64202	0	106.5
5.99	112.38	17	0	1.535	0.410446	0.863065	0	47.7
9.19	350.31	15	0	1.152	0.795167	0.97006	0	91.9
8.47	258.95	16	3	1.284	0.815804	0.972276	0	84.5
9.66	305.94	12	0	0.855	0.182433	0.652641	0	106.9
5.67	598.97	15	0	1.291	0.798497	0.97006	0	93.3
7.28	286.5	14	3	1.486	0.425587	0.873992	0	100.1
5.78	365.65	14	0	1.811	0.119067	0.536734	0	65.5
9.42	490.9	16	0	1.033	0.546024	0.909126	0	92.9

7.18	265.36	16	0	1.232	0.940558	0.991397	0	87.7
5.11	564.13	13	0	1.498	0.40773	0.862461	0	82.7
6.99	671.43	13	0	0.839	0.159691	0.622208	0	108.3
7.62	470.36	13	1	1.119	0.765633	0.967498	0	107.3
9.52	358.06	12	0	1.816	0.116448	0.532131	0	65.1
6.46	251.71	13	0	0.824	0.132862	0.566984	0	110.6
5.62	334.65	17	0	1.285	0.813525	0.971399	0	81
10.84	1486.47	13	0	1.14	0.822531	0.974456	0	95
5.3	257.89	14	0	1.318	0.738353	0.96414	0	78.3
5.94	389.12	12	0	2.027	0.046188	0.305872	0	73.2
9.14	534.34	14	0	1.231	0.943929	0.991397	0	99
5.31	805.38	12	0	0.957	0.36834	0.84602	0	99
9.25	318.13	15	0	1.101	0.719139	0.96164	0	97.6
6.25	257.24	19	0	1.055	0.600057	0.925915	0	94.6
8.78	441.54	13	0	0.704	0.037291	0.272942	0	129.8
6.06	203.21	14	2	62.08	1E-17	3.97E-16	0	1.8
8.57	684.14	12	0	1.406	0.560354	0.911314	0	84.3
7.21	237.05	9	0	1.784	0.159324	0.622125	0	58.5
6.18	458.47	14	0	1.13	0.794611	0.97006	0	101.4
5.24	268.42	17	0	1.653	0.251344	0.734036	0	58.2
7.42	289.78	14	2	1.128	0.791035	0.97006	0	98.2
10.35	330.3	17	0	0.829	0.136581	0.578149	0	120.4
9.77	458.45	14	0	1.47	0.450129	0.888233	0	94.3
5.01	503.5	13	0	0.981	0.414812	0.866217	0	105.5
7.17	273.27	16	0	1.26	0.873201	0.983083	0	94.3
6.7	398.5	14	0	1.306	0.765604	0.967498	0	84
6.77	276.04	20	0	0.931	0.312987	0.798743	0	100.5
6.86	282.79	15	0	2.193	0.026177	0.217952	0	47
4.82	431.34	12	0	1.076	0.652973	0.942654	0	86.4
8.12	219.36	13	0	0.7	0.021275	0.190489	0	120.9
6.15	205.13	11	0	1.391	0.592129	0.924683	0	38.7
9.29	486.08	13	0	1	0.466116	0.89585	0	92.1
10.99	3131.18	16	0	1.182	0.93195	0.990812	0	104.9
10.05	898.16	9	0	2.143	0.02725	0.221896	0	87.8
8.09	236.21	19	0	1.064	0.573257	0.918743	0	96.2
9.69	248.48	16	11	1.374	0.620759	0.932207	0	81.6
6.54	387.17	12	1	1.353	0.754064	0.96597	0	84.7
6.07	150.39	15	0	1.654	0.285141	0.767147	0	67.3
5.54	802.05	11	0	1.54	0.350789	0.828843	0	79.6
5.86	281.17	14	0	0.968	0.307716	0.795133	0	104.6
8.29	288.3	19	0	1.051	0.58878	0.922984	0	89.4
6.16	446.44	14	0	1.318	0.739627	0.96414	0	84.8
8.38	184.22	16	0	1.509	0.476151	0.898527	0	52.9
9.72	1220.91	10	0	1.609	0.269954	0.753413	0	80.5
6.96	203.53	11	0	1.503	0.462798	0.893486	0	51.1

10.01	377.64	13	0	1.128	0.791199	0.97006	0	93.9
9.1	395.82	13	1	1.769	0.142185	0.592887	0	63.5
5.33	152.6	19	0	1.279	0.882671	0.983083	0	108.8
12.03	112.65	16	0	1.489	0.446871	0.888233	0	27.9
6.6	184.68	12	0	1.863	0.097419	0.48322	0	60.3
6.57	179.18	16	0	1.884	0.104112	0.500065	0	57.5
5.92	477.44	12	0	1.193	0.959254	0.993831	0	91.4
7.08	364.41	10	0	1.168	0.895515	0.986348	0	88.7
9.6	4102.65	8	0	1.218	0.976728	0.997505	0	95.3
7.94	297.67	7	0	1.495	0.41325	0.864761	0	79.8
6.86	496.93	15	0	0.544	0.002069	0.033176	0	128.8
8.28	197.65	13	0	1.564	0.321004	0.803178	0	59.4
5.66	289.37	16	0	1.202	0.981667	0.997735	0	106.5
7.84	407.58	11	0	1.837	0.106609	0.507493	0	65.6
8.29	362.44	13	0	1.445	0.490679	0.905656	0	85.7
8.66	420.36	11	0	1.022	0.516674	0.90897	0	107.2
11.47	1422.31	11	0	0.946	0.34526	0.823197	0	112.9
5.49	1587.72	12	0	0.865	0.197199	0.673765	0	108.5
10.3	2850.9	11	0	1.143	0.830151	0.975858	0	92.7
8.79	264.62	15	0	1.248	0.900806	0.987276	0	108.7
9.64	199.93	10	0	2.577	0.003657	0.053589	0	42.1
5.31	203.81	14	0	1.286	0.81039	0.97073	0	91.2
5.99	290.89	13	0	1.355	0.660645	0.945736	0	78.8
8.95	172.71	16	0	1.534	0.414982	0.866217	0	78.2
7.78	253.12	14	4	1.831	0.109215	0.515033	0	101.1
5.78	259.86	17	0	1.167	0.891078	0.985364	0	94.6
6.6	277.64	13	0	0.962	0.354974	0.832019	0	103
9.83	411.59	11	0	1.352	0.665948	0.94808	0	85.1
8.9	377.28	13	0	0.926	0.304002	0.79167	0	106.3
8.24	428.89	9	0	1.572	0.310842	0.798212	0	70.2
5.62	260.34	15	1	1.022	0.39816	0.862453	0	97.3
8.98	119.54	15	0	1.521	0.40717	0.862461	0	54.3
4.78	370.35	12	0	1.134	0.806037	0.97006	0	97.5
10.32	611.14	13	0	0.844	0.166878	0.636322	0	104.2
8.46	269.01	14	0	1.031	0.521095	0.90897	0	95.4
7.78	283.89	13	0	1.502	0.402303	0.862461	0	47.5
6.51	148.82	13	0	3.206	0.00017	0.003677	0	67.6
6.79	216.09	15	0	1.156	0.862788	0.981617	0	103.2
8.09	416.84	16	0	1.233	0.938271	0.99128	0	88.6
6.77	357.2	11	0	1.52	0.376518	0.850346	0	83
4.83	385.9	11	0	0.7	0.03534	0.263531	0	106.4
5.82	571.13	15	0	1.334	0.704351	0.958927	0	91.5
5.64	204.95	14	0	1.073	0.56942	0.915274	0	103.9
7.97	121.52	15	0	1.658	0.265126	0.748188	0	40
7.06	180.79	13	0	1.196	0.967557	0.996855	0	55.2

9.29	366.77	12	2	1.737	0.162172	0.627599	0	71.5
9.09	380.09	12	0	1.23	0.945821	0.991419	0	89.2
4.73	393.9	9	0	1.75	0.111979	0.522788	0	70.3
10.89	1082.65	12	0	0.716	0.043677	0.298833	0	117
7.85	246.4	13	0	2.786	0.001288	0.022452	0	40.9
10.4	1590.42	13	0	2.314	0.012297	0.129436	0	65.1
5.48	213.89	12	0	1.36	0.700412	0.957995	0	72.2
5.35	751.82	11	0	1.041	0.564204	0.913186	0	98.6
8.6	198.85	14	0	1.893	0.083855	0.443178	0	59.1
10.54	992.61	11	0	1.034	0.548501	0.909321	0	108.2
4.44	468.59	14	0	0.83	0.14776	0.599917	0	108.8
7.12	207.41	14	0	1.151	0.778561	0.97006	0	91.9
9.98	529.84	13	0	1.055	0.600219	0.925915	0	96.4
10.93	1119.32	9	0	1.084	0.675188	0.951158	0	101.6
9.69	608.89	9	0	0.606	0.008068	0.094833	0	127.8
4.89	707.15	10	0	1.496	0.475475	0.898384	0	61.9
6.61	226.56	13	6	1.739	0.160662	0.623978	0	70.1
8.65	399.55	12	0	0.97	0.396206	0.861859	0	103.3
6.64	436.42	11	0	1.049	0.585503	0.922744	0	92.9
8.78	202.7	15	2	1.332	0.743124	0.964372	0	12.5
10.02	573.4	11	0	1.197	0.970555	0.997215	0	100.8
7.72	332.41	15	0	1.508	0.389754	0.859423	0	82.5
6.19	332.24	11	0	2.002	0.036231	0.266599	0	64.5
8.78	290.62	13	0	1.107	0.734317	0.962304	0	97.9
10.01	675.09	10	0	1.119	0.766875	0.968042	0	103.9
8.97	235.43	10	0	1.509	0.452284	0.888233	0	66.4
7.17	357.91	9	0	1.016	0.503337	0.90794	0	97.6
6.83	174.75	14	0	1.298	0.887778	0.983874	0	81.1
8.57	927.91	9	0	1.954	0.064209	0.376664	0	79.2
7.44	227.02	14	3	1.346	0.678629	0.951158	0	88.2
7.27	265.25	11	0	1.363	0.64281	0.939584	0	94.6
5.2	341.04	11	0	1.505	0.398203	0.862453	0	84.8
6.16	114.85	16	0	2.029	0.046179	0.305872	0	45.6
6.81	122.37	12	0	1.452	0.551116	0.910041	0	71.4
7.94	229.64	12	9	1.438	0.540765	0.90897	0	74.8
8.1	208.2	15	0	1.107	0.675222	0.951158	0	89.7
8.13	275.73	10	3	1.543	0.346779	0.824064	0	82.7
7.69	247.5	12	0	1.195	0.965248	0.995861	0	69.2
9.23	217.92	8	0	1.941	0.057672	0.351479	0	69.8
11.55	1669.49	12	0	1.328	0.715817	0.960634	0	100.3
7.77	159.29	11	0	2.129	0.03576	0.264505	0	52.8
9.52	397.91	6	0	0.638	0.0102	0.112824	0	140.7
5.12	599.3	9	0	1.075	0.613363	0.929068	0	99.3
7.01	136.11	13	0	1.74	0.176169	0.64543	0	62.8
6.51	327.68	10	0	1.583	0.298328	0.787763	0	97

9.63	170.05	13	0	2.311	0.014359	0.146251	0	62.8
6.4	320.32	9	0	1.231	0.942935	0.991397	0	87.7
10.55	1795.39	12	0	0.925	0.30263	0.79167	0	113.3
6.4	407.27	10	0	0.984	0.427567	0.875084	0	97.7
5.85	206.78	10	1	0.853	0.179298	0.6475	0	99.7
6.76	211.76	14	0	1.628	0.281873	0.76495	0	68.9
4.44	266.65	12	0	1.384	0.671892	0.950601	0	93.5
5.69	270.78	11	0	3.764	1.19E-05	0.000319	0	31.4
10.51	723.92	9	0	1.458	0.47008	0.896915	0	82.3
10.92	1903.23	10	0	0.754	0.069083	0.392282	0	108.7
8.63	116.03	14	2	1.458	0.531383	0.90897	0	56.1
5.88	298.14	11	0	1.754	0.151061	0.606568	0	64.9
8.57	173.06	12	0	1.34	0.69898	0.957318	0	76
9.33	167.93	13	0	0.877	0.188734	0.663939	0	98.3
4.88	185.34	9	2	1.113	0.693132	0.953331	0	88.8
11.78	917.21	9	0	0.799	0.110597	0.518357	0	116.9
4.92	440.83	7	0	7.247	5.08E-12	1.88E-10	0	9
8.47	442.84	9	0	1.186	0.942915	0.991397	0	94.6
6.09	222.94	10	1	2.318	0.012081	0.128021	0	36.6
5.97	112.78	14	0	2.07	0.045536	0.303974	0	45.4
6.38	343.56	10	0	0.812	0.124862	0.549593	0	113.4
6.93	325.51	10	0	1.259	0.875151	0.983083	0	90.4
7.52	321.25	11	3	1.263	0.866135	0.981617	0	78.1
6.39	144.06	13	0	1.145	0.779719	0.97006	0	90.3
7.97	242.68	13	5	1.255	0.884993	0.983234	0	88.7
7.42	293.52	12	1	1.582	0.299632	0.788806	0	78.5
10.49	1086.89	8	0	1.298	0.783991	0.97006	0	87.5
6.76	121.82	13	0	1.919	0.103794	0.500065	0	58.5
6.77	236.44	13	0	2.343	0.010741	0.117481	0	50.8
7.74	165.07	8	0	0.688	0.013827	0.14151	0	123.5
6.74	211.13	11	0	1.595	0.32944	0.806293	0	60.9
9.2	293.42	7	0	1.372	0.666139	0.94811	0	102.6
8.6	248.97	13	0	0.68	0.022974	0.202111	0	122.6
6.4	388.71	9	0	4.849	9.68E-08	3.19E-06	0	25.2
6.09	252.02	13	1	1.106	0.628175	0.932683	0	96
9.52	288.28	10	0	1.113	0.750819	0.96537	0	92.4
7.74	108.09	14	0	1.333	0.706778	0.958927	0	157.3
4.79	458.9	10	0	1.476	0.441335	0.88796	0	82.8
8.65	284.02	11	0	1.232	0.997749	0.99992	0	81.9
6.55	256.76	10	0	0.777	0.0554	0.343726	0	108.7
5.86	416.96	10	0	1.954	0.064147	0.376477	0	53.2
5.15	278.45	11	0	0.811	0.124351	0.547744	0	72
5.3	159.9	11	0	2.692	0.002022	0.032677	0	28.2
6.52	80.07	8	0	5.198	1.4E-08	4.79E-07	0	10.2
7.75	253.69	12	0	1.201	0.981384	0.997735	0	74.3

6.32	411.82	10	0	0.913	0.278631	0.764536	0	104
5.19	259.12	10	2	1.034	0.546856	0.909321	0	97.4
5.8	172.02	13	0	1.684	0.216627	0.697874	0	83.2
6.4	220.6	12	0	2.408	0.014371	0.146251	0	68.7
8.79	501.96	11	0	1.254	0.887156	0.983874	0	93.1
5.1	239	11	0	1.002	0.469949	0.896915	0	107.1
8.25	363.68	11	1	1.014	0.498527	0.906872	0	104.7
9.99	350.16	9	0	1.015	0.501165	0.906872	0	105
9.16	65.29	11	0	1.357	0.681904	0.951842	0	99.5
5.54	321.22	9	3	1.545	0.343455	0.821383	0	76.5
7.68	179.47	12	0	1.714	0.230418	0.709745	0	65.4
9.45	288.74	9	0	0.743	0.06097	0.363082	0	119.7
7.47	240.91	10	0	0.978	0.371301	0.846091	0	95.3
7.47	341.38	10	1	0.914	0.280996	0.76495	0	120.4
7.77	343.76	8	0	2.835	0.000493	0.009839	0	46.1
5.62	198.02	12	1	1.163	0.824184	0.974465	0	105.3
7.56	295.25	10	0	1.238	0.982414	0.997735	0	88.8
9	153.44	10	0	1.197	0.839751	0.977862	0	74
6.83	259.01	12	0	1.273	0.893844	0.985818	0	83.9
6.55	884.48	9	1	0.727	0.050125	0.324435	0	117.1
4.97	246.03	7	0	83.942	1E-17	3.97E-16	0	0.8
6.57	247.41	14	0	1.078	0.620943	0.932207	0	100.4
6	199.44	9	0	1.355	0.753027	0.96597	0	79.2
7.18	239.86	9	0	1.212	0.897844	0.987004	0	87.6
5.38	125.1	14	0	1.541	0.362735	0.840197	0	78.6
9.72	237.47	10	0	0.805	0.080744	0.435326	0	113.1
6.7	226.23	11	0	1.055	0.600044	0.925915	0	99.9
9.38	236.41	10	0	1.509	0.414354	0.865776	0	45.3
9.36	446.37	8	1	1.189	0.948611	0.991422	0	86.3
5.9	144.44	9	0	2.308	0.015483	0.151446	0	64
7.01	302.42	13	0	0.907	0.2693	0.752881	0	95
8.12	179.28	10	0	1.536	0.391252	0.861096	0	73.4
5.44	269.47	11	0	1.199	0.930794	0.99077	0	90.2
9.13	297.1	10	0	1.467	0.455899	0.889816	0	85.4
4.96	174.05	11	0	1.858	0.125209	0.55005	0	39.9
6.44	320.04	14	0	1.987	0.055364	0.343675	0	60.9
6.28	180.52	11	0	1.03	0.538054	0.90897	0	60.7
7.61	192.99	14	4	0.885	0.211814	0.692087	0	97.2
6.47	198.48	12	0	0.77	0.068606	0.39045	0	112.9
8.78	258.37	11	1	1.132	0.800691	0.97006	0	100.1
7.91	55.75	8	0	1.367	0.629504	0.933166	0	70.9
6.81	258.34	11	3	1.302	0.774236	0.97006	0	84.6
7.05	76.3	10	0	1.487	0.488689	0.904371	0	77.3
6.61	163.83	10	6	1.713	0.213644	0.694929	0	63.3
5.45	229.71	10	3	1.585	0.295741	0.785454	0	77.7

7.34	494.56	6	0	0.998	0.459315	0.891873	0	99.5
4.98	214.86	13	0	1.584	0.319225	0.802299	0	70.5
7.15	255.35	10	0	1.465	0.528308	0.90897	0	89
6.42	128.37	12	2	0.758	0.042805	0.296459	0	117.8
6.35	298.93	8	1	0.342	1.15E-06	3.47E-05	0	142.8
6.79	212.77	10	0	1.104	0.711291	0.959439	0	98.1
11.12	1772.68	8	0	0.963	0.381223	0.852622	0	97.3
9.45	403.58	9	0	1.435	0.507977	0.90897	0	85.3
7.8	111.44	13	0	1.439	0.572529	0.918743	0	78
5.47	253.86	12	4	1.133	0.804375	0.97006	0	90.7
8.35	151	10	0	1.185	0.824639	0.974465	0	87.4
9.03	225.96	13	0	1.998	0.052741	0.333729	0	58.6
8.7	144.67	11	0	1.306	0.866366	0.981617	0	84.4
9.57	183.88	10	0	1.185	0.868995	0.981633	0	86.3
6.93	236.13	12	0	1.567	0.370367	0.846091	0	66.6
9.35	1245.65	4	0	0.541	0.001932	0.03139	0	140.3
9.42	523.25	6	0	1.318	0.739432	0.96414	0	95.5
7.46	200.07	12	0	1.324	0.735108	0.962383	0	91.1
5.26	308.85	13	0	1.209	0.99992	0.99992	0	95.8
8.79	367.63	11	0	1.486	0.426257	0.874364	0	82.7
8.22	466.85	7	1	1.224	0.961613	0.995026	0	101.9
6.06	212.18	14	0	1.125	0.674436	0.951158	0	84.1
6.8	363.47	9	6	1.271	0.8463	0.978694	0	90.3
5.35	167.46	11	0	1.275	0.836956	0.976744	0	89.6
8.76	170.43	12	0	1.652	0.267738	0.75135	0	69.8
8.28	206.15	7	0	1.533	0.394774	0.861859	0	65.1
8.6	170.86	12	1	1.481	0.453334	0.888233	0	78.5
5.25	124.44	8	0	1.738	0.185573	0.658634	0	34.8
8.9	463.87	10	0	0.974	0.40489	0.862461	0	83
8.65	119.87	13	4	1.022	0.482841	0.90163	0	97
8.54	245.62	8	0	2.402	0.008128	0.094833	0	54.9
5.41	487.11	8	0	1.25	0.897741	0.987004	0	91.6
7.18	285.17	10	0	1.211	0.994065	0.99978	0	106.9
9.66	432.96	7	3	1.582	0.299427	0.788806	0	66.5
5.73	311.48	10	0	0.727	0.025066	0.211359	0	110.8
6.37	168.42	15	0	1.204	0.932276	0.990812	0	97.2
5.34	280.47	8	0	1.1	0.717197	0.960634	0	111
7.52	140.1	11	0	2.029	0.045307	0.303872	0	46.1
4.7	354.75	9	0	0.852	0.177456	0.645798	0	104.6
8.75	157.17	11	0	1.453	0.507201	0.90897	0	85
6.32	188.98	9	0	1.435	0.587701	0.922744	0	75.1
10.08	622.97	10	0	0.952	0.357431	0.834204	0	106.8
4.91	150.53	10	0	1.547	0.358903	0.835303	0	72.1
4.61	346.07	7	0	1.218	0.977093	0.997505	0	86.4
6.61	165.26	8	0	2.343	0.010715	0.117302	0	51.9

7.49	251.51	12	0	1.23	0.945732	0.991408	0	84.9
5.36	186.55	13	0	0.897	0.190059	0.663939	0	107.7
7.94	114.61	13	1	1.42	0.60807	0.927253	0	64
8.28	309.24	9	0	1.112	0.661828	0.946683	0	94
8.72	297.61	8	1	1.326	0.769617	0.968967	0	92
8.97	202.15	9	0	1.122	0.774991	0.97006	0	82.3
9.17	107.56	9	0	2.378	0.008128	0.094833	0	39.8
7.83	173.91	11	0	0.829	0.086618	0.450781	0	97.4
5.33	254.39	9	0	1.108	0.651218	0.942212	0	80
7.53	141.57	11	0	1.349	0.718928	0.96164	0	92.2
6.32	161.69	10	0	1.768	0.168867	0.636618	0	63.8
6.46	250.46	11	0	1.314	0.746916	0.964844	0	94.9
6.98	211.42	10	0	1.377	0.70469	0.958927	0	95.4
6.77	247.56	9	0	2.931	0.00064	0.012301	0	42.9
6.67	291.22	10	0	0.877	0.14874	0.600981	0	101
7.9	201.75	11	1	1.073	0.645658	0.940359	0	95.3
7.17	194.59	9	2	1.164	0.828763	0.975565	0	96.7
5.03	718.1	10	0	0.802	0.113354	0.525991	0	108.1
6.19	153.48	9	0	0.87	0.154788	0.615875	0	101.1
6.73	249.6	8	0	1.358	0.652704	0.942618	0	89.1
6.65	120.24	12	0	1.159	0.766554	0.967919	0	84.9
7.87	121.8	10	0	1.528	0.400511	0.862461	0	68.7
5.08	296.91	9	0	1.09	0.691599	0.952547	0	105.1
7.09	159.85	12	0	1.387	0.678782	0.951158	0	74.8
8.68	149.3	10	0	1.55	0.35442	0.831572	0	69.3
7.58	168.51	14	1	1.638	0.281708	0.76495	0	69.1
10.58	267.14	9	0	0.935	0.318695	0.802274	0	103.9
6.3	237.14	9	1	1.222	0.977978	0.99755	0	96.4
6.58	220.96	8	0	2.655	0.001727	0.028761	0	47.5
5.45	158.33	9	0	0.933	0.306469	0.79451	0	151.4
9.69	96.89	6	0	1.754	0.149656	0.603466	0	58.2
6.18	110.56	11	0	1.31	0.87445	0.983083	0	55.1
6.55	286.59	9	0	1.266	0.85746	0.981617	0	84.5
6.47	270.94	11	0	0.75	0.065755	0.381997	0	118.1
9.77	174.88	8	0	1.328	0.781013	0.97006	0	79.2
7.88	147.36	11	0	0.955	0.270281	0.753413	0	101.2
6.25	144.15	10	0	1.231	0.932127	0.990812	0	95.1
5.07	594.2	9	0	1.571	0.339095	0.81779	0	76.7
8.78	214.25	8	0	0.81	0.104028	0.500065	0	100.4
7.53	245.11	8	0	1.954	0.074536	0.412636	0	43.7
4.89	341.43	8	4	1.382	0.613987	0.929248	0	87.3
5.67	520.01	7	0	0.882	0.223787	0.701603	0	93.7
6.3	229.05	9	0	1.426	0.48971	0.905208	0	71.5
5.26	359.41	9	0	1.16	0.874765	0.983083	0	93.3
4.93	98.95	11	0	1.506	0.460653	0.892334	0	75.2

7.15	270.68	10	1	1.292	0.847527	0.979304	0	88
4.41	261.06	3	0	9.262	4.22E-15	1.64E-13	0	11.3
6.87	112.42	8	0	2.077	0.049332	0.321449	0	88.5
9.32	247.82	8	7	1.801	0.14247	0.593057	0	61.6
8.56	376.09	10	1	1.297	0.78591	0.97006	0	78.6
9.11	335.77	8	0	0.935	0.234123	0.711914	0	114.4
7.52	222.16	8	0	1.352	0.699223	0.957318	0	108.3
9.99	196.04	5	0	2.844	0.000973	0.017576	0	44.4
7.8	230.6	10	0	1.185	0.929207	0.99077	0	91
9.19	175.89	10	0	1.27	0.95256	0.991422	0	91.2
6.87	138.15	9	1	1.739	0.18412	0.655884	0	54
10.56	585.26	8	0	1.053	0.596093	0.925915	0	97.1
6.07	264.48	10	0	1.09	0.652237	0.942495	0	93
8.19	134.05	10	1	0.981	0.347655	0.825624	0	122.8
6.39	365.96	7	0	1.293	0.794826	0.97006	0	85.6
8.79	234.87	11	1	1.154	0.857997	0.981617	0	88.6
7.8	238.83	10	2	1.157	0.75717	0.966419	0	91
6.37	307.76	9	0	1.37	0.636743	0.937605	0	87.6
8.85	192.37	12	1	1.336	0.790465	0.97006	0	80
8.75	297.4	7	0	2.124	0.029783	0.235661	0	78.7
7.99	217.77	8	0	1.327	0.834706	0.976576	0	70.3
8.82	359.4	11	0	0.997	0.457631	0.891247	0	89.3
7.39	271.6	10	1	1.267	0.856477	0.981617	0	94.1
9.09	255.2	8	0	2.051	0.043582	0.298346	0	53.8
5.87	162.98	11	0	1.53	0.44707	0.888233	0	73.7
8.9	213.38	12	0	1.208	0.997145	0.99992	0	93.5
8.75	256.22	10	3	1.269	0.858331	0.981617	0	85.1
9.83	194.69	10	0	2.171	0.027603	0.224041	0	61.4
8.1	114.77	8	0	2.051	0.04557	0.303974	0	48.3
5.26	365.09	9	0	3.36	8.11E-05	0.001926	0	18.2
5.95	114.63	10	0	1.458	0.487738	0.903626	0	85.4
5.33	102.39	12	1	1.528	0.415958	0.866217	0	85.2
10.56	988.06	8	0	0.928	0.308929	0.796152	0	102.1
6.8	133.74	12	0	0.937	0.251037	0.734036	0	76.9
7.49	535.41	7	0	1.243	0.913723	0.98911	0	87
7.99	172.33	8	0	1.09	0.601687	0.925915	0	98.4
7.93	283.55	10	0	1.194	0.961781	0.995026	0	94.9
4.83	440.01	8	0	1.185	0.881489	0.983083	0	92.9
9.13	134.29	10	0	1.306	0.83031	0.975858	0	56
7.42	135.77	10	0	1.368	0.720766	0.96164	0	79.9
8.94	350.71	8	0	1.032	0.501275	0.906872	0	96.8
6.42	320.97	10	0	1.476	0.440817	0.887484	0	81.4
8.92	279.08	10	0	1.173	0.908344	0.988314	0	89
6.73	124.49	10	0	1.355	0.75146	0.96537	0	67.7
9.98	510.37	8	0	1.171	0.902436	0.987276	0	91.8

8.51	128.39	7	0	1.035	0.437319	0.884686	0	104
5.82	92.94	11	0	1.348	0.76053	0.966419	0	78.2
9.96	295.89	7	0	1.069	0.664756	0.947803	0	82.6
5.94	89.91	7	0	1.763	0.14925	0.602216	0	59.9
10.52	580.2	11	0	1.073	0.645759	0.940359	0	87.1
8.68	107.85	10	0	1.377	0.657252	0.943998	0	69.6
6.46	217.64	8	0	1.187	0.931429	0.990812	0	86.1
5.66	247.27	9	0	1.078	0.554856	0.910977	0	96.6
9.52	387.59	12	0	0.783	0.094732	0.476653	0	104.7
5.49	331.9	5	0	0.918	0.220277	0.700269	0	111.2
6.4	122.07	10	0	2.422	0.008336	0.097079	0	39.2
8.97	383.88	9	0	0.78	0.092075	0.467959	0	111.9
7.39	117	10	0	1.079	0.541095	0.90897	0	100.1
7.66	121.47	9	0	1.427	0.565838	0.913241	0	80
8.34	218.14	7	0	1.211	0.882578	0.983083	0	73
8.63	219.86	8	0	1.142	0.716801	0.960634	0	96.5
9.79	367.92	7	0	0.991	0.443238	0.888233	0	101.1
9.19	150.79	10	1	1.361	0.646874	0.940359	0	100.2
7.15	316.17	9	2	1.443	0.494876	0.906872	0	80.7
9.44	220.34	10	1	1.812	0.118706	0.535654	0	62.1
6.83	287.89	10	0	1.123	0.776094	0.97006	0	93.6
7.09	436.88	8	2	1.224	0.961989	0.995026	0	99.5
6.57	139	8	0	1.498	0.45241	0.888233	0	76.7
7.66	261.03	8	1	1.06	0.490756	0.905656	0	93.1
9.45	192.2	8	1	1.663	0.245756	0.725301	0	75.9
7.66	326.6	11	1	1.465	0.459209	0.891873	0	92
9.91	241.9	9	0	1.008	0.438271	0.885616	0	112.4
5.99	119.77	7	0	0.619	0.005462	0.071401	0	123.5
9.13	250.07	7	0	1.131	0.798319	0.97006	0	85.7
5.71	252.78	11	0	1.2	0.841792	0.977999	0	91
7.11	85.7	12	0	1.461	0.498324	0.906872	0	69.7
5.82	112.5	9	0	1.258	0.954269	0.991422	0	47
6.39	106.05	9	0	1.223	0.932335	0.990812	0	94.9
7.65	100.36	11	0	1.227	0.96194	0.995026	0	87
6.68	134.43	11	1	1.635	0.244106	0.725276	0	93.7
6	263.03	9	4	1.349	0.672981	0.95105	0	85.3
6.68	188.18	9	4	1.508	0.393479	0.861859	0	79.9
7.3	130.91	11	0	1.243	0.970505	0.997215	0	89.8
6.61	82.2	9	0	1.533	0.403689	0.862461	0	48.5
4.93	133.41	6	2	1.744	0.172898	0.641122	0	54.3
5.76	159.84	9	0	2.517	0.005788	0.07428	0	39.6
6.19	167.77	9	0	1.12	0.646668	0.940359	0	111.3
7.68	235.47	8	0	1.326	0.811262	0.971	0	85
7.21	203.42	6	0	1.676	0.211437	0.692087	0	44.8
5.95	375.43	9	0	1.585	0.31912	0.802274	0	82.7

9.72	603.23	10	5	1.294	0.791517	0.97006	0	90.6
9.73	543.33	8	0	1.274	0.838213	0.977517	0	86.2
8.73	196.73	6	0	1.346	0.775286	0.97006	0	90.9
6.1	362.23	6	0	1.217	0.978641	0.997657	0	96.3
8.03	221.54	9	0	1.035	0.550111	0.909434	0	78
8.57	502.15	6	0	1.103	0.724274	0.96164	0	92.3
6.9	170.4	9	0	1.132	0.800448	0.97006	0	92.3
5.9	113.84	7	0	1.292	0.879736	0.983083	0	84.5
9.07	112.24	10	0	0.877	0.247258	0.726511	0	112.9
9.67	318.91	11	1	1.187	0.944765	0.991397	0	93.6
11.85	101.8	7	0	2.189	0.0187	0.173611	0	45.3
6.64	128.79	8	1	1.333	0.771401	0.97006	0	71.1
9.11	191.67	9	0	2.252	0.020952	0.188345	0	52.4
8.22	71.31	8	0	1.29	0.908398	0.988314	0	85.2
9.72	309.67	8	0	1.541	0.379576	0.850854	0	76
8.21	104.78	8	1	1.03	0.536847	0.90897	0	105.9
8.56	98.89	10	0	1.755	0.153193	0.611546	0	49.6
5.77	173.36	8	0	1.223	0.928747	0.99077	0	64.6
11.15	286.22	6	1	1.345	0.680597	0.951158	0	77.8
10.18	282.03	9	0	0.952	0.357204	0.83383	0	113.2
8.25	296.58	8	0	1.113	0.698551	0.957318	0	101.3
10.15	433.77	6	0	3.195	0.000179	0.003829	0	36.6
6.8	190.34	5	0	1.101	0.719021	0.96164	0	80.3
8.27	122.75	6	0	1.459	0.534602	0.90897	0	72
9.64	163.84	10	0	1.015	0.492338	0.906569	0	90.4
7.06	139.51	7	0	0.989	0.346277	0.824064	0	69.7
6.23	112.34	9	0	1.487	0.440471	0.887298	0	86.9
7.17	107.1	11	0	1.313	0.840043	0.977862	0	80.8
6.55	126.61	9	0	1.639	0.244614	0.725301	0	74.7
9.16	124.15	9	0	1.513	0.445859	0.888233	0	94.3
7.33	284.75	8	0	1.261	0.87142	0.98284	0	89.2
8.44	112.59	9	0	2.12	0.029736	0.235647	0	49.2
4.82	552.11	8	1	0.475	0.000323	0.006595	0	144.7
5.49	102.24	9	1	2.25	0.015057	0.149576	0	52.1
8.62	264.2	7	0	1.157	0.808507	0.970193	0	87.7
5.48	353.6	9	3	1.199	0.974599	0.997505	0	95.4
9.19	112.64	7	0	1.645	0.236133	0.713558	0	76
8.54	74.69	9	0	2.995	0.000553	0.01082	0	33.5
6.37	372.19	8	0	1.16	0.873532	0.983083	0	93.7
6.9	158.19	11	6	1.074	0.594729	0.925915	0	109.2
11.05	2561.28	6	0	1.042	0.566591	0.913335	0	99.1
4.42	157.83	7	0	1.405	0.637759	0.937605	0	77.9
9.03	376.85	8	1	1.16	0.874605	0.983083	0	93.3
7.75	150.55	11	0	0.945	0.342856	0.821196	0	104.5
4.91	144.61	10	2	0.99	0.338685	0.817591	0	123.8

11.25	1336.14	10	0	1.128	0.791171	0.97006	0	96.2
5.77	87.82	11	0	1.427	0.567998	0.913456	0	72.7
6.2	84.47	7	0	1.447	0.540331	0.90897	0	82.9
8.59	85.9	11	0	1.361	0.646728	0.940359	0	75.8
9.94	345.36	9	0	1.782	0.134667	0.572164	0	72.7
7.65	136.1	6	0	1.055	0.499115	0.906872	0	75.4
6.95	137.42	8	1	1.364	0.72556	0.96164	0	84.9
9.16	181.89	7	2	1.383	0.603617	0.925915	0	61.7
9.25	77.49	6	0	2.228	0.01748	0.166846	0	33.7
9.57	493.33	8	0	1.082	0.613312	0.929068	0	93.4
6.02	267.69	8	3	2.756	0.001489	0.025294	0	46.8
6.93	72.38	9	0	0.96	0.280623	0.76495	0	89.1
6.34	228.88	6	0	0.995	0.424426	0.873769	0	97
9.55	470.72	7	0	1.164	0.884905	0.983234	0	101.4
8.15	213.28	8	0	1.145	0.776424	0.97006	0	89.2
9	109.16	9	0	1.258	0.978875	0.997657	0	90.5
5.94	374.83	9	0	1.108	0.7373	0.963849	0	88.3
6.21	203.74	8	1	1.199	0.974411	0.997505	0	86.6
11.12	580.43	5	0	1.725	0.308542	0.795949	0	73.4
9	77.45	6	0	1.247	0.969728	0.997215	0	59.3
6.02	161.81	9	0	1.2	0.962498	0.995026	0	110.7
9.64	776.37	5	0	0.973	0.40291	0.862461	0	108.1
6.18	160	9	0	1.207	0.935664	0.99128	0	92.8
8.46	149.82	10	0	0.917	0.343591	0.821552	0	160
5.67	527.67	9	0	1.303	0.759095	0.966419	0	66.8
8.12	75.25	9	0	2.79	0.000859	0.015884	0	43.7
9.29	235.99	9	0	0.92	0.26613	0.749344	0	98
7.72	154.71	7	0	1.185	0.830155	0.975858	0	85.3
9.39	176.47	6	0	1.367	0.743521	0.964372	0	44.9
5.45	255.89	9	0	1.428	0.54475	0.90897	0	85.7
9.74	333.98	5	0	1.352	0.663852	0.947209	0	75.5
7.87	92.79	9	1	1.149	0.737971	0.96414	0	112.7
5.8	85.85	10	0	1.321	0.830308	0.975858	0	79.9
6.1	235.37	6	0	0.2	1.95E-12	7.22E-11	0	157.4
5.43	662.49	6	0	1.093	0.698712	0.957318	0	104
8.56	131.92	8	4	1.577	0.305159	0.79242	0	100.3
5.88	435.65	5	0	0.736	0.055832	0.345222	0	117
8.19	202.14	10	0	0.786	0.09311	0.470557	0	121.4
5.87	162.26	9	0	1.123	0.668393	0.949385	0	104.5
7.27	112.04	8	0	1.242	0.956091	0.992024	0	99.6
8.35	122.86	6	0	1.386	0.680576	0.951158	0	69.8
5.68	251.51	6	0	0.625	0.008779	0.100288	0	119.5
6.4	169.44	7	0	1.257	0.964623	0.995625	0	82.7
7.3	184.8	8	0	1.655	0.25806	0.740472	0	65.3
4.86	122.42	7	0	1.154	0.73255	0.962196	0	101.1

10.13	876.33	9	0	1.398	0.575703	0.919147	0	89
8.63	173.56	10	0	1.719	0.193705	0.669022	0	70.3
8.91	106.52	11	0	1.417	0.584496	0.922551	0	68.7
6.84	139.71	6	0	1.674	0.226853	0.704106	0	37
11.05	1104.7	5	3	0.913	0.280352	0.76495	0	103.3
10.1	218.14	9	0	1.085	0.561497	0.911853	0	94.4
5.64	306.81	9	0	1.043	0.449433	0.888233	0	114.9
9.33	100.92	10	0	1.081	0.553981	0.910977	0	100
6.25	104.21	7	0	1.366	0.679865	0.951158	0	86
9.25	198.45	11	0	1.086	0.644727	0.9403	0	95.2
6.52	148.83	8	0	1.382	0.682015	0.951842	0	89.4
10.78	382.41	6	0	1.4	0.572039	0.918743	0	89.6
8.31	174.63	8	1	1.688	0.221217	0.701127	0	76.4
8.84	105.49	8	0	1.321	0.837588	0.977329	0	77.8
5.22	122.16	9	0	0.564	0.013457	0.13852	0	138.3
10.05	154.75	9	0	1.187	0.816244	0.972341	0	80
10.39	859.21	4	0	1.777	0.137676	0.578504	0	41.8
9.11	187.95	10	0	1.524	0.389332	0.859423	0	76.2
8.5	301.6	8	1	1.256	0.882423	0.983083	0	93.7
7.72	99.69	9	0	1.481	0.495689	0.906872	0	90.7
8.9	140.39	11	0	1.26	0.871588	0.98284	0	91.3
6.13	94.23	10	0	0.472	2.73E-05	0.000684	0	142.7
9.13	165.73	7	1	0.234	4.52E-11	1.64E-09	0	183.2
9.6	425.99	8	7	1.267	0.856152	0.981617	0	97.4
8.02	192.45	9	0	1.153	0.800776	0.97006	0	92.7
7.53	125.68	9	0	1.134	0.710441	0.959398	0	92.1
4.18	395.07	7	0	0.83	0.147361	0.599009	0	100.5
10.64	329.34	7	0	1.209	0.99992	0.99992	0	91.4
11.33	924.36	6	0	0.91	0.273472	0.756978	0	113.6
8.65	146.3	7	0	1.26	0.995652	0.99978	0	93.2
8.91	110.69	10	0	1.116	0.704014	0.958927	0	83.8
8.84	700.86	7	0	2.358	0.010018	0.111113	0	73.3
9.14	214.76	8	0	0.605	0.005957	0.075981	0	125.8
5.16	182.47	7	3	1.175	0.809539	0.970418	0	95.3
7.75	160.2	8	0	1.292	0.899082	0.987004	0	90.3
9.01	434.52	6	0	1.139	0.748996	0.96537	0	99.3
6.27	144.51	7	0	1.329	0.784444	0.97006	0	80.2
8.87	146.08	9	0	0.978	0.375534	0.849828	0	106.7
9.33	101.91	8	0	1.643	0.284111	0.766046	0	78
7.58	199.92	7	0	1.587	0.343204	0.82125	0	74
6.51	132.75	10	0	1.565	0.369877	0.846091	0	39.3
5.38	76.51	9	1	1.679	0.246279	0.725581	0	70
7.81	200.62	7	6	1.986	0.055667	0.344372	0	66.3
8.6	48.63	8	0	1.494	0.444298	0.888233	0	44.4
5.14	176.89	7	0	1.084	0.5766	0.919586	0	91.8

6.92	230.8	10	0	0.741	0.024378	0.208842	0	117.7
8.25	183.19	9	0	0.968	0.305646	0.793357	0	102.1
7.36	129.87	5	0	1.651	0.25795	0.740472	0	71.2
6.09	178.25	8	0	1.472	0.486892	0.903617	0	87.9
6.49	74.31	11	0	1.257	0.939311	0.99128	0	102.4
6.7	57	10	0	0.66	0.007406	0.08896	0	114.6
5.24	152.27	8	0	1.223	0.909965	0.988314	0	94
8.12	167.11	8	0	1.72	0.174018	0.64202	0	76.2
10.32	1429.1	6	0	0.719	0.045078	0.303872	0	122.9
5.73	157.62	9	0	1.069	0.564811	0.913241	0	68.5
5.05	249.72	6	0	1.651	0.272508	0.75652	0	74.7
9.76	313.69	7	0	0.931	0.314639	0.800259	0	98.2
5.76	173.94	10	2	1.179	0.879724	0.983083	0	91.5
6.76	25.18	6	0	2.102	0.02505	0.211359	0	100.6
9.5	116.28	6	0	0.807	0.083066	0.440653	0	102.6
8.53	154.46	6	0	1.621	0.304305	0.79167	0	59.1
9.16	108.05	6	0	1.299	0.855549	0.981617	0	80.7
7.58	93.07	9	0	1.185	0.851678	0.980769	0	86.6
6.6	294.08	8	0	1.318	0.833494	0.976224	0	117
7.81	102.18	7	0	1.682	0.227197	0.704826	0	68.5
6.93	107.15	8	0	1.029	0.486292	0.903584	0	98.4
5.99	143.51	9	1	1.373	0.711182	0.959398	0	91.1
7.66	88.73	8	0	1.498	0.498407	0.906872	0	71.5
6.05	221.99	6	0	1.231	0.960578	0.994786	0	89.5
9.1	305.22	7	0	1.418	0.5377	0.90897	0	75.2
7.36	77.73	8	0	1.392	0.612448	0.929068	0	28
11.36	763.84	7	0	1.01	0.488232	0.904275	0	100.4
5.19	95.08	5	0	1.9	0.058725	0.355319	0	91.7
6.79	108.67	8	0	1.553	0.415943	0.866217	0	71.2
4.54	308.04	5	0	1.031	0.540568	0.90897	0	98.3
7.14	227.3	6	3	1.273	0.842134	0.978048	0	88.9
8.03	121.32	10	1	0.998	0.370413	0.846091	0	101.3
7.37	107.6	6	0	1.247	0.972201	0.997505	0	72.1
6.8	129.39	4	0	1.347	0.789483	0.97006	0	87.5
5.26	158.89	7	0	1.207	0.877266	0.983083	0	74.8
9.47	56.82	8	0	2.063	0.026977	0.221673	0	72.6
5.6	75.17	8	0	1.818	0.107729	0.509851	0	44.4
6.51	143.82	9	0	1.119	0.65635	0.943998	0	106.4
9.09	230	5	0	0.96	0.282329	0.76495	0	117.8
6.73	150.49	8	0	1.198	0.850796	0.980769	0	82.1
5.5	183.83	7	2	1.899	0.081761	0.438691	0	21.1
7.8	107.45	6	0	0.986	0.357942	0.834469	0	99.8
7.05	165.91	8	2	1.132	0.687058	0.952468	0	95.7
6.25	421.35	6	0	0.98	0.419286	0.867443	0	98
9.26	177.12	6	1	1.373	0.671586	0.950381	0	84.3

8	174.5	9	0	0.976	0.36601	0.842834		0	104.6
5.38	486.36	6	0	1.039	0.522635	0.90897		0	98.3
6.89	109.54	6	0	1.729	0.18735	0.662699		0	56.3
6.8	62.68	11	0	1.443	0.596715	0.925915		0	62.2
4.82	256.98	7	0	0.914	0.192902	0.667684		0	107.3
7.8	111.89	9	0	1.282	0.913679	0.98911		0	66.7
9.14	171.83	9	0	1.069	0.552414	0.910945		0	101.2
11.22	439.46	8	0	1.202	0.982695	0.997735		0	92.8
9.58	106.75	11	0	1.391	0.662438	0.946953		0	79.1
7.33	148.18	8	0	0.905	0.194194	0.669726		0	102.6
5.14	161.21	4	0	1.156	0.761293	0.96666		0	77.3
4.36	177.48	7	0	1.098	0.593667	0.925417		0	100.6
5.64	120.37	8	0	2.423	0.009006	0.10251		0	46.7
9.41	442.48	3	1	1.286	0.810659	0.970851		0	98.1
8.82	630.53	7	0	0.983	0.346802	0.824064		0	91
9.94	727.86	4	0	1.135	0.809219	0.970418		0	82.5
5.5	118.26	10	0	1.269	0.920855	0.989178		0	92.7
6.07	46.35	8	0	1.303	0.819038	0.973778		0	101.7
6.42	74.87	9	0	1.352	0.730999	0.962129		0	73.6
8.15	111.68	8	0	1.054	0.542671	0.90897		0	97.6
9.04	157.09	8	0	1.963	0.069856	0.394889		0	62.9
5.59	300.68	5	0	1.109	0.739662	0.96414		0	94
7.02	110.07	9	0	1.559	0.378374	0.850346		0	93.7
4.77	227.43	9	0	0.87	0.148753	0.600981		0	130.1
8.4	261.82	7	0	2.617	0.003361	0.049539		0	53.6
7.55	158.27	8	0	1.033	0.496678	0.906872		0	121.1
8.92	28.91	8	0	1.112	0.657596	0.944169		0	52
8.6	199.88	8	0	1.182	0.871116	0.98284		0	88
9.63	118.73	5	0	1.355	0.745695	0.964844		0	45.6
8.79	204.29	7	0	1.317	0.793875	0.97006		0	81.7
4.7	187.77	6	1	1.009	0.427911	0.875646		0	103.2
5.54	176.08	9	0	1.582	0.329508	0.806293		0	67.4
7.97	150.14	9	0	1.095	0.628572	0.932683		0	104.8
8.82	104.66	7	0	0.928	0.271998	0.756425		0	97.7
5.72	394.1	7	0	0.954	0.29447	0.78291		0	90.9
8.09	211.92	8	1	1.368	0.663029	0.946953		0	70
4.98	55.22	5	0	1.943	0.090351	0.463687		0	61.5
5.58	79.98	9	0	1.195	0.86871	0.981617		0	86.9
10.9	1131.36	4	0	1.091	0.603891	0.925915		0	93.7
5.06	198.87	7	1	0.881	0.193725	0.669022		0	106.5
9.54	181.95	9	0	1.004	0.475313	0.898384		0	100.8
7.55	49.1	8	0	1.287	0.8932	0.985818		0	86.4
8.12	80.06	8	0	1.424	0.555675	0.910977		0	80
5.72	134.75	8	0	1.4	0.611452	0.929068		0	89.7
8.12	351.05	7	0	1.276	0.879985	0.983083		0	85.7

9.51	473.94	8	2	0.94	0.331582	0.808657	0	98.8
9.25	144.97	10	6	1.225	0.982834	0.997735	0	77.9
8.84	144.63	7	0	1.293	0.861829	0.981617	0	91.3
9.19	143.44	8	0	1.786	0.160981	0.624336	0	45.9
9.22	225.63	9	0	1.118	0.672719	0.95081	0	97.5
6.38	79.99	7	0	1.427	0.542704	0.90897	0	54.6
9.2	57.58	8	0	1.442	0.474654	0.898019	0	66.5
9.98	307.09	7	0	1	0.464717	0.895777	0	106
9.32	179.67	6	0	1.312	0.80369	0.97006	0	83.1
5.8	159.06	8	0	0.708	0.031724	0.245013	0	116.7
9.76	274.58	7	0	0.942	0.325626	0.806293	0	101.9
10.04	139.07	8	0	1.379	0.656953	0.943998	0	92.5
9.51	290.02	7	0	1.015	0.453463	0.888233	0	96.4
7.44	180.02	8	0	1.124	0.725248	0.96164	0	96.8
8.43	570.91	6	0	1.132	0.801162	0.97006	0	95.1
6.93	108.93	7	0	0.991	0.352766	0.83025	0	78.2
7.69	217.72	7	0	1.439	0.539434	0.90897	0	92.8
6.24	141.55	7	0	1.024	0.443335	0.888233	0	98.5
8.54	119.17	8	0	1.192	0.873021	0.983083	0	93.9
9.91	184.02	8	0	1.734	0.176666	0.64543	0	68.7
7.72	137.05	6	0	2.414	0.009948	0.110632	0	38.4
6.79	209.38	5	0	1.01	0.41017	0.863065	0	98.8
6.54	74.23	9	0	1.501	0.405872	0.862461	0	88.3
6.8	178.24	7	2	1.14	0.776555	0.97006	0	89.9
6.16	157.64	4	0	0.539	0.001653	0.027713	0	116.9
8.41	178.44	6	0	2.007	0.050074	0.324416	0	62.1
7.27	88.94	5	0	1.178	0.839231	0.977862	0	89
4.96	192.42	9	0	1.057	0.552136	0.910945	0	108.2
6.67	62.19	7	0	1.31	0.79765	0.97006	0	97.5
10.17	144.73	7	0	1.944	0.082807	0.440653	0	81.1
6.21	157.59	9	0	0.935	0.25909	0.740955	0	108.1
8.95	190.39	6	0	1.193	0.952809	0.991422	0	74.3
7.97	130.13	6	0	1.115	0.699231	0.957318	0	89.3
6.02	104.34	5	0	1.663	0.261768	0.746056	0	25
4.65	97.6	8	0	2.595	0.003197	0.047463	0	48.7
9.28	223.75	8	7	0.893	0.174546	0.642502	0	108.1
7.3	261.34	8	0	0.914	0.282158	0.76495	0	113.6
7.9	122.4	6	0	1.179	0.805008	0.97006	0	91.3
5.49	261.55	5	0	1.858	0.08621	0.449586	0	72.4
5.17	98.42	7	0	0.923	0.231999	0.711304	0	102.6
6.27	132.43	9	0	1.082	0.558617	0.910977	0	114.8
6.64	138.25	9	0	1.509	0.394154	0.861859	0	77.2
9.8	205.17	5	0	1.329	0.713527	0.960068	0	88.8
10.2	107.5	8	0	1.481	0.523362	0.90897	0	70.5
5.66	91.44	8	0	1.177	0.810675	0.970851	0	98.4

8	113.87	8	0	0.929	0.229512	0.708001	0	104.6
6.51	141.27	5	0	1.238	0.980598	0.997735	0	80.5
6.32	149.3	8	0	1.068	0.581731	0.920669	0	101.5
7.43	123.06	6	0	1.068	0.505722	0.90897	0	91.4
6.84	80.82	7	0	1.528	0.391124	0.861096	0	71.6
5.36	265.67	6	2	1.277	0.839448	0.977862	0	95.2
8.76	481.42	6	0	1.05	0.588674	0.922984	0	101
7.72	276.49	7	0	1.188	0.945684	0.991408	0	92.9
7.11	69.58	8	1	1.231	0.986181	0.997735	0	76.7
7.61	151.87	6	0	2.46	0.009582	0.107322	0	33.8
6.79	280.56	7	1	1.279	0.827758	0.974887	0	93.5
5.67	121.59	5	0	1.471	0.490873	0.905701	0	58.4
6.57	52.44	6	0	2.464	0.006037	0.076534	0	69.6
5.2	240.14	5	0	1.782	0.116479	0.532131	0	75.4
6.15	57.18	7	0	1.271	0.911247	0.988683	0	138.1
9.99	195.14	5	0	1.335	0.797815	0.97006	0	88.3
10.11	1501.85	8	0	0.884	0.227964	0.706161	0	105.1
7.37	199.19	8	0	1.208	0.947364	0.991422	0	95.4
6.07	127.67	8	0	1.402	0.648382	0.941029	0	84.2
8.98	171.88	7	0	1.436	0.527452	0.90897	0	83.5
8.35	127.23	6	0	1.197	0.912548	0.989072	0	88.2
7.43	115.35	8	0	1.297	0.850946	0.980769	0	87.6
9.79	134.48	7	0	1.057	0.505436	0.90897	0	96.7
6.84	178.92	9	0	1.005	0.430658	0.879393	0	97.9
7.42	57.48	7	1	1.175	0.772283	0.97006	0	86.5
9.22	96.92	7	0	1.047	0.477737	0.898527	0	108.8
10.32	185.38	6	0	1.025	0.524097	0.90897	0	92.2
6.89	236.21	8	0	1.098	0.646601	0.940359	0	91.1
8.72	181.16	7	0	1.7	0.188313	0.663939	0	73.7
7.14	96.82	6	0	1.417	0.581448	0.920669	0	94.9
5.64	111.68	7	0	1.146	0.752609	0.96597	0	94.4
10.77	206.02	9	0	0.961	0.377311	0.850346	0	104.8
8.34	84.35	2	0	1.401	0.620977	0.932207	0	101.5
9.22	125.03	7	0	1.343	0.741331	0.964372	0	69.7
6.55	193.62	7	0	1.783	0.133877	0.569968	0	56.6
6.49	90.48	8	0	1.661	0.24407	0.725276	0	68.2
8.9	83.84	8	2	2.744	0.001508	0.025449	0	51.3
7.71	110.9	9	0	0.976	0.410651	0.863065	0	107
6.74	144.81	6	0	1.128	0.676985	0.951158	0	86.9
6.05	107.94	6	0	2.02	0.052459	0.332531	0	68.3
6.55	315.7	7	0	1.254	0.887367	0.983874	0	89.4
5.34	136.9	8	0	1.282	0.889975	0.984958	0	90.5
5.95	238.41	8	1	1.24	0.920444	0.989178	0	92
4.56	155.02	5	1	0.735	0.045236	0.303872	0	100.8
9.33	134.11	8	0	1.7	0.241752	0.722317	0	74

7.43	169.26	5	0	0.986	0.359349	0.835583	0	104
5.6	150.48	6	0	1.216	0.960492	0.994786	0	91.4
8.44	102.68	6	0	1.24	0.993483	0.99978	0	99.7
8.27	147.31	7	0	0.718	0.044792	0.303815	0	108.7
6.67	281.39	6	0	1.921	0.074154	0.411064	0	72.6
6.39	138.44	6	0	0.916	0.219425	0.700036	0	92.3
8.16	138.98	6	0	1.372	0.733434	0.962304	0	91.5
9.26	71.59	6	0	1.039	0.473851	0.898019	0	88.1
7.36	60.49	6	0	0.983	0.300361	0.790393	0	88.7
7.59	77.02	9	0	1.314	0.805919	0.97006	0	75.4
7.59	60.56	5	0	1.671	0.165695	0.634023	0	87.3
8.09	65.02	8	0	1.278	0.915391	0.98911	0	94.9
5.74	50.43	8	0	1.831	0.105654	0.505157	0	64.5
8.97	177.32	7	0	1.115	0.703846	0.958927	0	96.5
6.4	58.74	8	0	1.247	0.976255	0.997505	0	80.7
7.18	62.57	8	0	0.969	0.309788	0.796853	0	100.7
5.2	98.24	7	0	1.562	0.372751	0.847074	0	65.3
5.24	193.25	6	4	0.94	0.331737	0.808821	0	91.8
9.33	180.29	5	0	1.013	0.495782	0.906872	0	97.1
6.73	134.35	8	0	0.66	0.011357	0.123467	0	111.4
5.26	207.9	6	1	1.258	0.97616	0.997505	0	86.1
9.48	333.85	5	0	1.127	0.725116	0.96164	0	91.7
7.9	130.47	7	0	0.95	0.260873	0.745037	0	112.2
6.18	219.71	8	0	1.398	0.575604	0.919147	0	91.4
8.82	78.74	6	0	1.231	0.963999	0.995536	0	72.1
6.28	144.97	6	2	1.744	0.196602	0.673222	0	73.6
8.34	80.75	6	0	0.765	0.052242	0.331488	0	135.3
7.94	124.57	7	0	0.62	0.009094	0.103321	0	123
6.77	139.98	7	0	1.212	0.924026	0.989893	0	87.7
6.25	113.39	9	0	1.395	0.627511	0.932683	0	70.4
12.02	932.2	6	0	0.73	0.051963	0.330812	0	106.2
5.88	109.23	7	0	0.764	0.047841	0.313684	0	106.1
5.36	141.67	6	0	0.611	0.006825	0.083585	0	126.2
9.67	172.03	7	0	1.224	0.973402	0.997505	0	87.1
7.27	155.68	10	0	2.026	0.053085	0.334142	0	67.2
8.97	187.4	5	2	1.281	0.877037	0.983083	0	90.7
7.31	360.4	8	0	0.973	0.402648	0.862461	0	100
9.47	127.69	8	0	1.139	0.765992	0.967501	0	82.4
5.78	181.96	9	8	1.138	0.815973	0.972341	0	102.1
9.57	69.7	5	0	1.449	0.584994	0.922744	0	91.2
10.51	705.48	7	0	0.971	0.398783	0.862461	0	96
6.51	107.55	8	0	1.546	0.379607	0.850854	0	45.1
5.86	134.46	5	0	1.129	0.660614	0.945736	0	89.1
9.47	153.51	6	0	0.899	0.17207	0.640909	0	104
7.62	173.9	6	1	1.458	0.470595	0.897624	0	79.3

5.69	136.43	7	0	1.51	0.45491	0.888988	0	71.6
6.4	271.57	6	0	1.924	0.086694	0.450801	0	69.6
5.41	128.18	7	0	1.238	0.956241	0.992098	0	93.6
6.14	42.53	10	0	1.765	0.125744	0.550237	0	62.6
6.64	162.78	7	0	1.343	0.792604	0.97006	0	63.5
5.8	61.33	8	0	1.238	0.999902	0.99992	0	89
7.34	66.21	8	0	1.141	0.741056	0.964372	0	96
9.31	135.41	8	0	1.168	0.772364	0.97006	0	75.8
6.06	90.34	8	0	1.698	0.171556	0.640297	0	67.7
8.82	276.26	6	0	1.314	0.747216	0.964844	0	84.2
8.82	279.63	4	0	1.139	0.711185	0.959398	0	92.2
8.59	136.34	9	0	1.255	0.941757	0.991397	0	92.7
9.2	281.81	5	0	1.646	0.233633	0.711914	0	84.4
9.91	304.83	6	0	1.12	0.76856	0.968389	0	98.7
6.8	117.12	7	1	1.445	0.565303	0.913241	0	86.5
11.65	296.11	7	0	1.101	0.719489	0.96164	0	89.5
6.8	91.3	6	0	1.983	0.055508	0.343726	0	58.8
8.15	554.12	5	0	1.086	0.678653	0.951158	0	97.2
8.66	108.41	7	0	3.038	0.000898	0.016454	0	42.8
8.75	128.27	7	0	0.958	0.265067	0.748188	0	108.5
6.54	172.25	5	0	1.462	0.501898	0.907555	0	84.9
7.06	112.76	8	0	1.146	0.774458	0.97006	0	94.9
6.87	59.21	7	0	1.487	0.441428	0.888005	0	84.1
8.35	186.21	8	0	1.09	0.689999	0.952547	0	97.9
8.41	196.15	7	0	2.121	0.014919	0.14906	0	69.8
6.33	126.46	6	0	1.101	0.639515	0.937605	0	93.3
9.38	68.07	9	0	1.236	0.942858	0.991397	0	14
6.37	108.93	7	0	0.9	0.169001	0.636618	0	118.1
9.64	183.97	7	0	1.202	0.984118	0.997735	0	87.3
9.38	214.76	6	0	1.219	0.974432	0.997505	0	88.4
8.82	161.1	7	0	1.439	0.545955	0.909126	0	79.5
7.06	50.18	4	0	1.716	0.12431	0.547744	0	47.9
5.81	176.25	3	0	1.766	0.14686	0.598768	0	65.7
9.2	109.99	5	1	0.788	0.071663	0.402216	0	112.7
9.72	250.8	6	0	1.312	0.751143	0.96537	0	93.7
9.39	136.91	8	0	0.73	0.068809	0.391157	0	119.4
9.29	140.15	8	0	1.61	0.360655	0.838156	0	83.3
9.04	160.99	9	0	0.678	0.02043	0.18504	0	124.6
10.35	209.03	6	0	1.286	0.86697	0.981617	0	97
4.84	84.7	5	0	1.125	0.648981	0.94118	0	91.7
5.53	117.4	6	0	1.255	0.986594	0.997735	0	89.1
6.24	106.07	6	1	2.004	0.059935	0.358302	0	26.2
7.05	229.25	7	0	1.682	0.202716	0.682501	0	87.8
8.21	174.51	4	0	1.79	0.146817	0.598768	0	58.5
8.46	188.56	5	0	1.74	0.154317	0.61439	0	78.8

6.05	102.41	7	0	1.119	0.668816	0.949664	0	103.9
9.38	37.36	6	0	2.652	0.001426	0.024527	0	16.8
7.28	125.09	8	0	1.736	0.190712	0.663939	0	62.1
6.21	84.23	7	0	1.021	0.431681	0.879939	0	85.4
9.28	199.7	6	0	1.115	0.755127	0.96597	0	96.1
4.68	221.82	4	0	0.956	0.280281	0.76495	0	97.8
6.77	93.41	7	0	2.372	0.006075	0.076783	0	57.9
8.54	65.27	8	5	1.215	0.94276	0.991397	0	81.5
9.67	66.76	6	0	1.578	0.343193	0.82125	0	66.5
5.06	85.57	5	0	1.335	0.806697	0.97006	0	84.8
6.02	144.95	7	0	1.251	0.95073	0.991422	0	83.1
6.95	131.73	6	0	0.744	0.040159	0.284706	0	111.9
8.59	107.52	7	0	1.737	0.202305	0.682501	0	68.1
7.18	76.69	7	0	1.725	0.223623	0.701603	0	54.1
5.91	209.07	6	2	1.097	0.69208	0.952547	0	96.3
6.83	201.14	7	0	1.122	0.773686	0.97006	0	100.2
4.91	98.15	6	0	2.005	0.041606	0.29131	0	68.7
8.56	68.98	6	0	0.731	0.025066	0.211359	0	114.2
6.76	88.74	6	0	0.835	0.083126	0.440653	0	105
11.47	385.23	5	0	1.075	0.651709	0.942225	0	98
8.31	85.45	8	0	1.06	0.499879	0.906872	0	86.5
9.32	150.87	5	0	1.079	0.60782	0.927253	0	96.9
6.21	209.33	7	3	1.189	0.950765	0.991422	0	99.5
6.48	256.53	9	0	1.113	0.751592	0.96537	0	88.3
6.79	241.96	6	1	1.381	0.688804	0.952468	0	95.5
5.58	124.25	5	0	1.053	0.476301	0.898527	0	97.6
8.22	155.41	7	0	1.228	0.991385	0.999059	0	97.8
5.16	49.11	6	0	1.191	0.884279	0.983234	0	69.5
6.81	89.31	6	0	1.758	0.156055	0.616338	0	67.8
8.27	105.2	8	0	1.109	0.740796	0.964372	0	102.6
7.46	245.34	5	2	1.139	0.695962	0.955969	0	93.4
8.79	92.02	5	0	1.861	0.078483	0.426426	0	58.1
7.4	291.35	4	2	1.511	0.390099	0.859539	0	74
10.32	261.45	4	0	1.499	0.406298	0.862461	0	78.3
5.29	108.15	8	0	1.365	0.639985	0.937605	0	110.5
8.22	121.26	5	0	1.14	0.739541	0.96414	0	93.2
11.84	885.07	6	0	1.307	0.763318	0.967182	0	97
7.59	76.97	7	0	1.099	0.590917	0.924336	0	89.3
11.84	181.38	7	0	1.375	0.62009	0.932207	0	78.8
8.1	125.84	6	0	1.093	0.604129	0.925915	0	91.7
6.79	129.94	6	0	1.776	0.160508	0.623978	0	72.3
5.22	153.75	7	1	1.403	0.612164	0.929068	0	81.3
4.89	277.21	6	0	1.233	0.998607	0.99992	0	93.9
9.5	137.14	5	0	2.664	0.006192	0.077949	0	54.6
8.54	49.03	8	0	1.205	0.887439	0.983874	0	90.6

9.69	103.48	6	1	1.229	0.924457	0.990131	0	93.4
5.53	248.82	4	0	0.773	0.045072	0.303872	0	111.3
8.54	113.53	5	0	1.433	0.586322	0.922744	0	72
8.35	48.59	5	0	1.686	0.210827	0.691171	0	60.4
7.87	65.21	8	0	1.16	0.765914	0.967498	0	102.3
7.44	68.81	7	0	1.429	0.502667	0.907771	0	81.6
7.61	519.57	7	4	1.141	0.824839	0.974465	0	93.2
9.31	134.71	5	0	2.091	0.047093	0.309907	0	55.8
9.36	109.15	7	0	1.205	0.931938	0.990812	0	94.6
7.05	55.54	7	0	1.339	0.808872	0.970261	0	93.9
4.81	227.63	6	0	1.155	0.860052	0.981617	0	90.5
8.87	100.43	8	0	1.118	0.635293	0.936718	0	94.1
7.01	85.79	7	0	0.17	1E-17	3.97E-16	0	190.1
10.1	713.97	4	0	1.717	0.175965	0.645275	0	72
8.05	57.05	6	0	1.558	0.352725	0.83025	0	79.8
5.33	102.78	4	0	2.161	0.024002	0.208842	0	59.6
11.19	145.24	6	0	2.018	0.056563	0.347689	0	69.7
7.17	80.11	6	0	1.099	0.581386	0.920669	0	89.7
6.65	36.97	7	0	1.358	0.680996	0.951611	0	15.4
10.04	173.13	4	0	1.035	0.468851	0.896668	0	98.6
7.64	83.97	6	0	1.013	0.377684	0.850346	0	101.7
11.06	321.67	6	0	1.019	0.511752	0.90897	0	86.6
9.1	85.32	6	0	1.245	0.971949	0.997505	0	90.2
9.04	37.72	5	0	1.311	0.740457	0.964272	0	55.2
7.02	68.82	5	0	0.921	0.220907	0.700979	0	99.5
5.74	29.24	6	0	1.425	0.539326	0.90897	0	56.2
8.62	239.35	6	0	1.193	0.96082	0.994799	0	87.7
5.76	111.81	6	0	1.339	0.735043	0.962383	0	105.9
8.9	46.95	6	0	1.368	0.729824	0.962129	0	81.5
6.83	72.49	7	0	1.664	0.244877	0.725301	0	62.9
8.91	116.28	5	0	1.181	0.886327	0.983774	0	89.5
5.25	91.86	4	0	1.668	0.21538	0.696844	0	81.5
8.98	69.52	6	0	1.332	0.803012	0.97006	0	80.3
6.8	89.08	7	0	1.158	0.765559	0.967498	0	96.3
11.9	248.58	7	0	1.397	0.575882	0.919147	0	93.9
5.83	142.06	5	0	1.602	0.270017	0.753413	0	73.9
5.3	126.42	5	0	1.206	0.88523	0.983234	0	88.5
5.74	41.5	4	0	1.675	0.155815	0.616259	0	74.8
7.91	184.97	7	0	2.261	0.017508	0.166846	0	45.4
4.81	62.41	7	0	1.285	0.862686	0.981617	0	103.3
6.02	115.72	7	0	1.174	0.798195	0.97006	0	84.6
6.6	133.25	6	1	1.491	0.490319	0.905476	0	88.1
6.9	212.26	4	0	1.134	0.723253	0.96164	0	86.9
6.13	144.97	6	0	0.954	0.288403	0.772192	0	101.1
5.86	77.88	8	0	1.335	0.79793	0.97006	0	91.9

6.77	114.13	6	0	1.163	0.763202	0.967182	0	85.2
4.81	131.95	6	0	1.018	0.457452	0.891247	0	92.9
9.19	87.15	8	3	1.202	0.851434	0.980769	0	90.1
9.94	95.56	7	0	1.585	0.296171	0.785454	0	79.5
5.74	75.54	5	0	1.824	0.103565	0.500065	0	71.8
7.88	300.55	6	0	1.48	0.435268	0.88312	0	82
6.84	141.3	4	0	1.33	0.818152	0.973232	0	73.7
6.39	145.09	6	0	2.089	0.040664	0.28711	0	55.2
5.63	105.53	3	0	1.315	0.842631	0.978048	0	84
6.73	101.89	8	0	1.137	0.685184	0.952226	0	91.5
8.79	81.02	7	0	1.108	0.628658	0.932683	0	102.4
4.72	110.03	4	0	0.877	0.159567	0.622135	0	104.6
5.25	149.24	6	0	1.371	0.678128	0.951158	0	79.1
8.31	285.38	4	0	1.275	0.836659	0.976711	0	86.7
6.65	41.2	6	0	1.035	0.470053	0.896915	0	121.9
4.91	111.4	6	0	2.287	0.012402	0.130027	0	40.1
6.42	112.04	7	1	1.32	0.786952	0.97006	0	76.4
9.31	157.19	5	0	1.278	0.828947	0.975565	0	93.4
9.19	136.55	6	0	1.367	0.744542	0.964801	0	92.1
10.89	264.84	8	0	1.125	0.782686	0.97006	0	100.4
9.61	58.19	5	0	1.937	0.039931	0.283687	0	52
4.82	147.95	5	0	1.207	0.918104	0.98911	0	83.1
8.43	69.18	7	1	1.568	0.331544	0.808657	0	85.4
5.87	41.11	8	0	1.236	0.968606	0.997215	0	68.7
5.58	46.26	6	0	1.737	0.142801	0.593057	0	54
5.64	197.6	7	0	1.289	0.846251	0.978694	0	103
7.91	35.69	6	0	1.442	0.459005	0.891873	0	43.9
6.58	117.69	6	0	1.33	0.783884	0.97006	0	85.8
8.69	114.12	7	0	1.134	0.805952	0.97006	0	65.3
4.89	307.45	6	0	1.283	0.880152	0.983083	0	93
4.27	68.29	5	0	0.857	0.141843	0.591866	0	123.6
5.63	119.66	3	0	2.835	0.000523	0.010281	0	30
4.93	38.41	4	0	1.129	0.719251	0.96164	0	82.2
5.66	43.79	8	0	1.154	0.785051	0.97006	0	92
9.41	86.64	6	3	0.968	0.304673	0.792264	0	93.6
5.68	121.49	6	0	1.045	0.45872	0.891873	0	103.4
5.96	82.52	6	0	1.463	0.472697	0.898019	0	79.6
5.19	41.11	6	0	1.377	0.66429	0.947596	0	66.3
5.14	43.05	2	0	1.332	0.756034	0.96597	0	52.7
5.43	244.61	5	0	1.209	0.995031	0.99978	0	79.8
10.23	352.41	6	0	0.873	0.209939	0.690199	0	103.5
5.69	135.21	6	0	1.646	0.296487	0.785454	0	77.8
5.38	61.75	8	0	0.786	0.05635	0.346891	0	110.4
6.23	52.47	6	0	1.227	0.915888	0.98911	0	87.5
7.69	62.58	7	1	0.963	0.311206	0.798212	0	85.4

5.68	77.92	7	0	1.044	0.481408	0.900513	0	101.7
6.29	65.75	7	0	1.513	0.44521	0.888233	0	49.5
6.49	205.68	5	0	1.161	0.747334	0.964844	0	94.6
9.17	59.21	6	0	2.596	0.002421	0.03768	0	29.8
8.6	130.7	4	0	1.29	0.918631	0.98911	0	93.6
5.76	84.04	6	0	1.15	0.702951	0.958655	0	90.2
8.7	174.48	4	0	1.318	0.844964	0.97823	0	72.8
8.94	61.29	6	1	0.951	0.270602	0.753841	0	100
8.29	126.41	5	0	1.18	0.828492	0.975485	0	88.8
6.61	36.36	5	0	1.135	0.718653	0.96164	0	80.8
9.19	134.37	5	0	1.224	0.918337	0.98911	0	88.6
9.35	296.99	6	0	1.248	0.901924	0.987276	0	97.8
5.2	175.38	4	0	1.126	0.67775	0.951158	0	95.1
10.43	878.71	3	0	1.438	0.50246	0.907771	0	90.3
8.09	38.38	6	0	1.612	0.271175	0.754632	0	62
8.7	108.92	8	0	1.355	0.659114	0.945081	0	95.8
4.94	105.78	6	0	1.449	0.553925	0.910977	0	64.4
9.63	492.09	4	0	0.864	0.195583	0.672335	0	116
9.41	80.98	6	0	1.41	0.659207	0.945081	0	80.2
5.83	49.21	4	0	1.747	0.143878	0.59581	0	54.4
8.62	109.89	7	0	1.29	0.90112	0.987276	0	94.9
10.05	125.17	4	0	2.747	0.000931	0.016856	0	31.4
7.74	72.73	8	0	0.909	0.199961	0.678574	0	105.9
6.35	98.05	7	0	1.151	0.705498	0.958927	0	83.5
10.01	147.61	7	0	1.047	0.579346	0.920301	0	99.3
7.46	57.89	7	1	1.41	0.580635	0.920669	0	78.6
6.18	42.33	6	0	1.464	0.48093	0.899886	0	82
6.04	66.28	7	0	1.871	0.178226	0.646437	0	70.4
9.1	100.19	6	0	1.431	0.561363	0.911753	0	71.2
5.83	54.51	5	0	1.488	0.483557	0.90176	0	52.7
8.69	551.36	6	0	1.103	0.633419	0.935444	0	88.9
9.36	140.85	5	0	0.966	0.310365	0.797711	0	108.9
9.99	106.28	4	0	2.193	0.015767	0.153786	0	58.7
7.37	137.68	7	0	1.453	0.520753	0.90897	0	89.8
5.57	195.79	4	0	0.808	0.079852	0.431628	0	105.4
9.04	115.47	6	0	1.416	0.583328	0.922392	0	93.7
8.4	152.16	7	0	1.144	0.831441	0.976036	0	87.7
8.82	82.08	6	0	0.944	0.374182	0.84814	0	101.9
5.19	70.19	5	0	1.349	0.665538	0.948004	0	64.7
8.03	74.89	6	0	1.418	0.537229	0.90897	0	69.8
9.94	42.87	6	0	1.504	0.443112	0.888233	0	67.2
9.39	143.97	7	2	0.978	0.307371	0.795133	0	95.9
8.81	87.43	6	0	1.812	0.124152	0.547442	0	82.7
5.95	66.35	5	0	1.579	0.254935	0.736744	0	71.5
8	104.71	3	0	1.218	0.893824	0.985818	0	89.4

6.76	340.82	6	0	1.267	0.979531	0.997735	0	79
7.31	65.87	7	0	1.569	0.36414	0.841747	0	89.4
5.66	90.77	5	0	1.293	0.887725	0.983874	0	84.8
5.78	125.68	6	1	1.258	0.996305	0.99992	0	89.2
6.37	63.54	5	0	1.009	0.406974	0.862461	0	85.7
8.16	355.02	5	0	1.037	0.554367	0.910977	0	93.4
7.44	176.81	5	0	0.684	0.037596	0.27332	0	118.7
9.69	134.81	5	0	1.518	0.448981	0.888233	0	71.2
6.09	126.97	5	0	1.076	0.569865	0.915756	0	99.4
8.79	87.85	6	0	1.48	0.441775	0.888136	0	68.7
7.9	105.45	4	0	1.458	0.534567	0.90897	0	81.3
6.6	138.58	4	0	1.226	0.968668	0.997215	0	88.7
7.93	46.56	5	0	1.709	0.187725	0.66328	0	65
4.78	66.3	6	0	1.316	0.78998	0.97006	0	64.9
7.05	73.3	7	0	1.574	0.309018	0.796152	0	69.7
8.92	45.77	6	0	1.258	0.903309	0.987276	0	79.5
5.21	35.69	6	0	1.889	0.063224	0.372105	0	45.9
6.23	112.63	5	0	0.984	0.323928	0.805731	0	103
5.15	84.05	5	0	1.298	0.889055	0.984355	0	86.4
7.75	128.83	5	0	1.211	0.941301	0.991397	0	95.1
6.46	99.99	5	0	1.301	0.825852	0.974684	0	63.5
8.51	91.46	6	0	1.083	0.672719	0.95081	0	130.1
9.19	325.94	4	0	0.87	0.205271	0.683931	0	109.1
9.16	87.99	6	0	0.892	0.184345	0.656441	0	104.2
7.18	67.19	6	0	1.294	0.907397	0.988314	0	92.2
9.52	154.99	6	0	1.195	0.966168	0.996402	0	100.7
5.69	132.99	6	0	1.133	0.719007	0.96164	0	93.9
11.56	218.36	5	1	0.841	0.162139	0.627599	0	118.5
10.9	1033.34	6	0	0.979	0.415813	0.866217	0	111.2
8.54	54.48	7	0	1.298	0.79326	0.97006	0	82.5
4.73	56.74	4	0	3.046	0.00011	0.002557	0	20.9
5.06	147.11	4	0	1.48	0.453486	0.888233	0	85.6
6.52	52.14	6	0	1.437	0.548876	0.909321	0	70.2
7.96	44.17	5	0	1.794	0.167286	0.636322	0	91.7
5.43	156.85	4	3	1.43	0.538346	0.90897	0	84
8.66	49.7	6	0	1.418	0.610729	0.928335	0	78.8
8.4	38.55	3	0	1.641	0.250792	0.734036	0	46.4
8.72	123.76	5	0	1.11	0.65162	0.942225	0	95.9
9.14	111.14	6	0	0.97	0.409117	0.862461	0	108
6.2	74.8	6	0	1.09	0.575761	0.919147	0	94.2
7.37	89.95	6	0	1.076	0.569818	0.915756	0	92.4
9.86	156.69	4	0	1.589	0.296957	0.785775	0	85.9
5.17	88.15	6	0	1.363	0.702609	0.958512	0	76.5
5.4	88.97	7	0	1.618	0.291318	0.777817	0	85.9
6.4	70.4	7	0	0.915	0.197238	0.673765	0	104.9

7.39	247.83	4	0	1.494	0.414121	0.865638	0	74.2
5.01	90.54	5	0	1.213	0.893753	0.985818	0	93.8
8.75	239.03	4	0	0.961	0.375751	0.850167	0	95.9
5.38	38.04	8	0	1.525	0.396596	0.861859	0	56.5
6.06	81.45	4	0	1.354	0.689782	0.952547	0	81.3
5.54	165.53	4	0	1.281	0.8942	0.985818	0	96.1
5.67	134.84	6	0	1.126	0.7328	0.962196	0	95.2
9.69	159.23	5	0	1.498	0.446565	0.888233	0	76.6
7.78	125.7	6	0	1.746	0.178439	0.646437	0	60.1
9.33	93.25	6	1	1.376	0.645742	0.940359	0	78.5
6.95	104.29	6	0	1.1	0.717491	0.960802	0	79.1
7.24	36.47	6	0	1.411	0.533685	0.90897	0	65.9
6.39	106.8	5	0	1.31	0.79749	0.97006	0	62.4
9.5	53.82	7	0	0.84	0.099377	0.490417	0	102.7
7.15	128.79	5	0	1.145	0.761504	0.966779	0	91
7.62	48.69	6	0	1.547	0.348851	0.827128	0	78.5
5.12	109.81	5	0	1.332	0.688713	0.952468	0	89.5
6.89	79.11	5	0	1.2	0.881016	0.983083	0	100.6
5.38	38.03	4	0	1.379	0.657514	0.944169	0	93.7
9.45	364.03	4	0	0.727	0.050377	0.325565	0	109.4
8.43	90.39	5	1	1.251	0.95096	0.991422	0	87.8
9.91	197.46	4	0	1.665	0.216803	0.697874	0	76.3
6.71	97.24	5	0	1.332	0.792745	0.97006	0	84.8
8.84	100.04	6	0	1.405	0.638641	0.937605	0	93.6
7.58	64.43	5	0	1.856	0.10049	0.494074	0	67.2
5.58	154.14	5	0	1.738	0.178669	0.646718	0	73.9
5.38	35.48	6	1	2.003	0.045527	0.303974	0	25.6
5.72	48.38	5	0	1.155	0.773173	0.97006	0	89.1
9.36	114.23	4	0	1.202	0.920182	0.989178	0	54.5
9.79	52.73	4	0	1.365	0.72214	0.96164	0	78.3
7.97	85.86	6	1	1.542	0.348214	0.826171	0	96.4
7.62	102.56	5	0	0.931	0.250509	0.733579	0	98.4
7.43	22.94	6	0	1.412	0.519832	0.90897	0	71
8.38	60.32	6	0	1.024	0.406955	0.862461	0	98.4
8.37	82.28	6	0	1.058	0.466164	0.89585	0	88.2
6.44	94.42	5	0	1.293	0.909638	0.988314	0	84.3
8.4	68.09	4	0	1.38	0.624302	0.932683	0	64.6
9.35	87.11	6	0	1.003	0.396076	0.861859	0	91.9
5.27	53.04	4	0	1.635	0.277805	0.763386	0	64.6
8.81	142.1	5	0	0.99	0.398327	0.862453	0	97.9
10.14	147.59	5	0	1.097	0.604345	0.925915	0	97.2
6.29	121.14	4	0	1.092	0.615468	0.929812	0	93.8
6.21	79.09	6	0	1.264	0.973844	0.997505	0	76.4
9.63	151.22	3	0	1.131	0.796689	0.97006	0	98.8
10.27	213.25	5	0	1.01	0.404605	0.862461	0	106

7.5	56.05	5	0	0.988	0.361705	0.839819	0	110.4
3.79	65.59	4	0	1.002	0.39141	0.861129	0	113.7
9.6	95.52	5	0	1.547	0.389809	0.859423	0	66.8
5.78	122.95	5	0	1.846	0.114854	0.529439	0	64.2
8.34	80.32	5	0	1.377	0.678197	0.951158	0	71.7
8.66	129.75	6	1	1.071	0.549422	0.909371	0	90.3
6.47	41.48	6	0	1.244	0.995535	0.99978	0	85.5
6.55	152.22	5	0	1.086	0.588806	0.922984	0	97.7
9.44	71.29	5	0	1.194	0.864139	0.981617	0	96.9
9.07	54.19	5	0	1.216	0.954176	0.991422	0	89.3
8.38	81.06	3	0	1.263	0.993651	0.99978	0	83.7
5.43	82.89	5	0	1.279	0.94391	0.991397	0	86.9
9.36	49.92	5	0	1.292	0.795622	0.97006	0	55
7.09	53.96	6	2	1.305	0.857961	0.981617	0	87.2
6.81	79.65	4	0	1.119	0.711148	0.959398	0	90.4
6.84	24	7	0	1.648	0.181595	0.651757	0	63.9
4.94	96.8	7	4	1.558	0.380854	0.852217	0	66.8
6.04	171.56	6	0	1.811	0.127955	0.55391	0	54.8
7.24	76.94	6	0	1.626	0.282666	0.76495	0	107.9
8.9	126.75	6	0	1.082	0.616531	0.930636	0	97.1
8.22	98.24	4	0	1.743	0.175265	0.643347	0	50.9
8.21	45.45	6	0	1.122	0.69786	0.956851	0	90.8
6.77	33.2	7	0	1.396	0.580753	0.920669	0	64.3
7.14	55.58	4	0	1.638	0.248378	0.728205	0	85.1
7.06	45.52	6	0	1.101	0.600603	0.925915	0	73.2
7.44	28.55	5	0	1.467	0.424167	0.873523	0	98.2
6.13	34.11	5	0	2.632	0.000693	0.013195	0	52.6
7.64	40.38	4	0	1.761	0.144	0.59581	0	63.9
9.55	107.66	5	0	1.148	0.711773	0.959883	0	105.7
7.58	147.43	5	0	1.419	0.53607	0.90897	0	80.7
8.9	63.73	5	0	1.719	0.174971	0.643316	0	57.5
5.36	45.12	4	0	1.434	0.490725	0.905656	0	78.4
8.44	138.12	6	0	1.049	0.488557	0.904371	0	96.3
5.34	41.24	5	0	1.412	0.592696	0.924683	0	63.7
6.3	125.89	5	0	1.548	0.344095	0.822131	0	61.5
9.17	33.49	3	0	2.194	0.012452	0.13031	0	57.9
7.33	93.05	5	0	1.395	0.665285	0.947803	0	75.3
10.36	178.82	4	0	1.801	0.142823	0.593057	0	91.8
8.78	138.22	4	0	1.598	0.311227	0.798212	0	63.6
5.71	68.69	5	0	1.391	0.646921	0.940359	0	83.1
7.14	92.83	5	0	1.287	0.865353	0.981617	0	93.8
8.24	74.6	7	0	1.231	0.928445	0.99077	0	77.5
7.21	207.57	5	0	1.275	0.851406	0.980769	0	91.4
4.97	38.22	7	0	1.768	0.155681	0.616223	0	28.3
8.47	27.59	3	0	1.788	0.105171	0.503662	0	60.3

5.11	44.72	5	0	1.727	0.178698	0.646718	0	49.3
9.47	71.41	5	0	1.173	0.806957	0.97006	0	94.8
4.81	50.25	5	0	1.348	0.789441	0.97006	0	76.9
6.4	238.69	4	0	1.178	0.863458	0.981617	0	85.6
6.87	47.66	7	0	1.135	0.713612	0.960068	0	97.4
8.05	33.15	4	0	1.12	0.685824	0.952226	0	86
7.39	88.69	4	3	1.353	0.748833	0.96537	0	97.9
7.94	123.48	4	0	1.174	0.78329	0.97006	0	94.8
5.33	36.95	5	0	1.276	0.954067	0.991422	0	77.9
5.92	35.98	5	0	1.365	0.67015	0.95015	0	87.3
6.73	43.7	6	1	1.581	0.328644	0.806293	0	79.4
5.82	63.56	5	0	1.269	0.971257	0.997505	0	85.8
4.48	46.43	6	0	1.129	0.659974	0.945604	0	61.6
7.85	71.25	5	0	0.915	0.197382	0.673765	0	110.8
7.28	36.52	6	0	1.86	0.099859	0.49241	0	48.8
7.42	63.23	5	0	1.16	0.795172	0.97006	0	90
6.87	87.45	3	0	1.229	0.97564	0.997505	0	90
7.3	53.65	6	0	1.227	0.988118	0.998053	0	68.7
6.27	32.72	4	0	1.431	0.508353	0.90897	0	61.2
9.64	73.15	6	0	1.473	0.46398	0.89463	0	78.9
7.4	103.01	6	0	1.019	0.462287	0.893486	0	108.8
8.25	73.71	5	1	1.202	0.907129	0.988314	0	95.9
6.54	86.87	4	0	1	0.387516	0.859031	0	101.3
6.2	108.88	3	0	1.104	0.726365	0.96164	0	89.4
6.99	58.1	5	0	2.31	0.011804	0.126756	0	43.3
5.6	53.98	3	0	1.614	0.253665	0.735398	0	56.2
5.22	86.2	6	0	0.975	0.309031	0.796152	0	109.2
4.97	59.33	5	0	1.137	0.730358	0.962129	0	76.3
8.37	66.86	3	0	1.045	0.435604	0.883346	0	137.6
8.62	67.05	4	0	0.574	0.000824	0.015305	0	160.2
8.5	206.03	3	0	1.063	0.541572	0.90897	0	103.3
4.77	43.5	4	0	0.612	0.002104	0.033398	0	137.1
5.94	84.2	4	0	1.319	0.84437	0.97823	0	73.2
6.9	68.49	4	0	1.305	0.757932	0.966419	0	74.3
9.1	132.38	6	0	1.199	0.843605	0.97823	0	75.2
6.64	49.27	6	0	1.605	0.316343	0.800491	0	79
8.7	47.35	3	0	1.268	0.908899	0.988314	0	95.7
8.78	134.59	3	0	1.602	0.319746	0.802626	0	81.6
6.95	61.04	4	0	1.664	0.241901	0.722317	0	66.6
8	110.03	6	0	1.113	0.657981	0.944397	0	117.6
8.31	154.56	5	0	0.779	0.046744	0.308092	0	121.6
9.28	49.45	3	0	0.83	0.101995	0.497489	0	108.7
6.34	37.69	5	0	1.007	0.40386	0.862461	0	98
11	413.68	4	0	1.118	0.763692	0.96741	0	95.9
5	70.44	5	0	1.225	0.925394	0.99022	0	94.3

6.87	97.26	5	0	1.317	0.831212	0.975858	0	86.6
7.09	91.02	3	0	1.089	0.553707	0.910977	0	87.9
6.33	73.22	5	0	1.276	0.892391	0.985818	0	90.1
9.89	202.82	5	0	1.175	0.912793	0.98911	0	93.5
7.05	55.6	5	0	1.464	0.556911	0.910977	0	84.4
9.29	91.1	5	0	0.988	0.451844	0.888233	0	118.7
4.96	77.07	5	0	1.067	0.558735	0.910977	0	95.5
8.75	102.85	4	0	1.331	0.812514	0.971206	0	84.1
6.51	41.14	5	0	0.742	0.030743	0.240536	0	166.2
10.42	139.11	3	0	1.849	0.107575	0.509851	0	72.7
8.32	39.61	6	0	1.434	0.58082	0.920669	0	79.8
7.08	53.32	5	0	1.261	0.96684	0.996606	0	84.2
9.5	106.86	4	0	1.231	0.951804	0.991422	0	87.2
5.26	39.48	5	0	1.963	0.052402	0.33234	0	116.2
5.48	40.47	5	0	1.203	0.915857	0.98911	0	87.3
6.83	63.53	6	0	1.655	0.252233	0.734489	0	49
6.23	58.65	2	0	1.003	0.395652	0.861859	0	119.4
6.16	72.17	7	0	1.214	0.89688	0.986959	0	91.1
6.79	43.83	5	1	1.42	0.535465	0.90897	0	105
8.46	51.93	6	0	1.16	0.783698	0.97006	0	78.9
5.36	53.76	6	0	1.491	0.478633	0.898527	0	56.2
6.13	317.48	5	0	0.634	0.01182	0.126756	0	122.4
7.52	51.48	5	0	1.561	0.27574	0.760747	0	111.3
9.09	57.49	4	0	1.592	0.312824	0.798743	0	70.6
5.86	69.21	6	0	1.216	0.938107	0.99128	0	90.8
6.65	105.86	5	0	1.203	0.885402	0.983234	0	85.8
8.16	72.52	4	0	1.428	0.487586	0.903626	0	63.3
8.81	52.95	5	3	1.981	0.053865	0.337868	0	44.8
5.39	26.67	6	0	1.249	0.926551	0.990519	0	83.8
10.33	49.25	5	0	1.823	0.125538	0.550237	0	66.2
8.09	48.01	6	0	1.476	0.478158	0.898527	0	54.2
11.02	213.31	3	0	0.967	0.389276	0.859423	0	101.7
9.38	43.14	5	0	1.228	0.986452	0.997735	0	103.4
5.22	58.17	6	0	1.588	0.313888	0.799862	0	70.9
6.49	46.66	6	0	1.223	0.964063	0.995536	0	93.2
8.29	146.49	5	0	1.238	0.994598	0.99978	0	84.4
9.28	42.35	5	0	1.203	0.856116	0.981617	0	96.5
8.43	54.51	3	0	1.294	0.815049	0.972021	0	126.5
6.21	127.84	4	1	1.244	0.99824	0.99992	0	90.3
7.62	102.2	3	0	1.335	0.740983	0.964372	0	94.1
8.35	35.06	6	0	1.029	0.423906	0.873413	0	88.4
10.13	137.65	3	0	0.929	0.21711	0.697874	0	99.6
7.59	166.15	5	0	100	1E-17	3.97E-16	0	
8.21	126.29	4	0	1.711	0.232106	0.711304	0	65.7
8.46	207.61	5	0	1.198	0.973227	0.997505	0	81.2

8.07	51.89	5	0	1.534	0.384711	0.854992	0	46.2
7.12	48.18	5	0	1.68	0.19371	0.669022	0	77.4
4.74	277.57	5	0	1.288	0.881148	0.983083	0	82.4
9.88	103.28	4	0	1.122	0.691235	0.952547	0	78.6
6.23	23.31	5	0	1.744	0.128075	0.55391	0	53.5
5.11	30.29	5	0	2.792	0.00023	0.004828	0	43.4
5.6	119.45	3	0	0.744	0.040604	0.28711	0	110
6.05	83.47	4	0	1.28	0.920583	0.989178	0	90
6.09	45.29	7	0	1.334	0.739477	0.96414	0	73
9.45	53.67	6	0	0.94	0.229726	0.708133	0	98.7
6.05	18.4	5	0	1.409	0.627833	0.932683	0	47.2
6.98	61.98	6	2	1.125	0.688654	0.952468	0	92.3
8.22	23.85	5	0	1.606	0.236434	0.713801	0	79.1
7.25	90.73	7	0	1.475	0.486035	0.903584	0	70
5.53	39.08	4	0	1.205	0.973073	0.997505	0	92.6
9.83	27.18	4	0	1.073	0.548601	0.909321	0	97.9
7.91	40.68	4	0	1.114	0.686165	0.952468	0	93.3
6.28	106.49	5	0	1.051	0.474975	0.898087	0	96.3
8.31	74.07	6	0	0.947	0.253345	0.734875	0	131.5
8.44	39.81	5	0	3.093	6.28E-05	0.0015	0	20.5
6.86	29.43	5	0	1.388	0.627764	0.932683	0	108
4.82	55.36	4	0	1.347	0.717954	0.961119	0	87.3
10.24	94.23	4	0	1.169	0.808723	0.970261	0	86.4
7.12	56.09	5	0	1.345	0.680216	0.951158	0	90.5
7.85	35.19	4	0	1.005	0.476834	0.898527	0	97.5
7.11	34.21	4	0	1.419	0.578134	0.919803	0	79.3
8.97	31.91	5	0	0.786	0.054196	0.33944	0	47.7
9.1	315.69	3	0	1.172	0.906607	0.988314	0	103.6
5.96	92.08	4	0	1.299	0.847693	0.979317	0	81.2
6.52	33	4	0	1.524	0.330017	0.806871	0	89.2
6.58	66.86	6	0	1.989	0.045733	0.303974	0	59.8
4.87	77.25	4	0	1.205	0.893271	0.985818	0	68.6
8.66	89.81	4	0	1.3	0.84435	0.97823	0	85.5
6.77	365.59	4	0	1.011	0.492085	0.906476	0	98
7.2	87.54	5	0	1.124	0.699107	0.957318	0	97.4
8.15	36.26	3	0	2.745	0.000927	0.016836	0	38
8.13	123.17	4	2	0.981	0.377598	0.850346	0	96.9
8.47	66.43	5	0	1.152	0.852617	0.981581	0	89
6.43	83.54	5	0	1.53	0.466148	0.89585	0	75.6
6.27	30.5	4	0	1.25	0.975473	0.997505	0	77.5
7.65	19.63	5	0	2.786	0.000441	0.008833	0	32.7
5.35	37.14	5	0	1.413	0.596695	0.925915	0	86.5
7.61	31.93	7	1	1.506	0.415774	0.866217	0	100.7
6.01	94.93	6	0	1.166	0.806952	0.97006	0	92
11.59	206.23	4	0	1.211	0.994077	0.99978	0	99.6

8.76	124.63	4	0	1.063	0.52836	0.90897	0	95
8.95	47.57	5	1	0.665	0.009214	0.104312	0	118.4
9.79	126.3	6	0	1.014	0.450568	0.888233	0	107.6
6.99	38.27	5	0	1.148	0.75847	0.966419	0	104.6
7.14	54.05	4	0	1.197	0.83196	0.976224	0	96.4
7.05	110.41	3	0	1.285	0.878343	0.983083	0	72.4
9.1	85.82	3	0	1.131	0.679395	0.951158	0	105.8
8.69	17.57	4	0	1.492	0.399979	0.862461	0	142.9
6.52	85.63	5	1	1.239	0.996688	0.99992	0	95.1
8.92	102.62	5	1	1.621	0.280133	0.76495	0	26.5
6.1	56.18	5	0	1.327	0.823882	0.974465	0	81
5.91	38.03	6	0	1.69	0.160775	0.623978	0	79.4
5.16	72.89	7	1	1.351	0.722887	0.96164	0	91.2
6.48	106.66	4	0	1.438	0.57685	0.919586	0	85.4
7.01	48.73	3	0	1.639	0.218225	0.697874	0	68.5
7.91	131.57	5	0	1.308	0.813809	0.971463	0	84.4
8.38	139.56	4	0	1.046	0.566643	0.913335	0	87.5
7.37	280.54	3	0	1.606	0.287848	0.771989	0	61.9
8.91	48.79	5	0	0.934	0.247995	0.728205	0	132
8.13	60.37	3	0	1.1	0.619305	0.932207	0	83.7
8.31	57.11	5	0	1.474	0.426303	0.874364	0	62.4
7.03	32.55	2	0	1.183	0.894078	0.985818	0	62.2
7.71	60.79	5	0	1.152	0.776997	0.97006	0	93.6
5.57	49.81	5	0	1.125	0.667411	0.948458	0	93
9.16	69.9	5	0	1.238	0.943051	0.991397	0	89.9
6.48	177.95	6	0	0.982	0.422923	0.872243	0	100.9
7.31	69.51	5	0	92.915	1E-17	3.97E-16	0	1.3
8.09	54.78	5	0	1.257	0.937105	0.99128	0	98
8.7	114.38	4	0	1.463	0.541558	0.90897	0	67.5
8.56	45.05	6	0	1.442	0.537992	0.90897	0	68.7
9.72	61.9	5	0	1.344	0.780561	0.97006	0	78.9
10.56	147.38	4	0	1.338	0.797865	0.97006	0	84
8.95	49.14	5	0	0.743	0.038265	0.276649	0	115.2
6.33	123.52	4	0	0.669	0.007088	0.086385	0	122.5
4.93	63.3	6	0	1.068	0.50855	0.90897	0	98.1
5.9	66.88	4	0	1.974	0.048749	0.318803	0	28.4
9.57	88.32	4	0	1.095	0.589565	0.923701	0	94.6
6.55	45.22	6	2	1.091	0.619875	0.932207	0	91.8
8.34	28.12	5	0	1.407	0.624646	0.932683	0	81.4
8.46	212.47	4	0	1.062	0.59545	0.925915	0	95.4
6.49	125.63	3	0	1.083	0.601984	0.925915	0	92.6
7.3	70.25	3	0	1.336	0.696844	0.956785	0	84.1
5.92	77.06	6	0	1.076	0.566206	0.913256	0	82.7
6.15	62.47	6	0	0.869	0.147005	0.598768	0	98.2
6.04	31.67	3	0	1.338	0.721742	0.96164	0	98

9.31	43.1	4	0	1.116	0.701378	0.958482	0	83.1
8.84	37	3	0	1.339	0.729033	0.962129	0	65.3
6.68	50.96	5	0	0.929	0.229399	0.708001	0	114.4
5.06	32.16	5	0	1.386	0.666415	0.948182	0	18.5
5.53	37.12	4	0	1.221	0.94341	0.991397	0	86.4
9.09	41.42	4	0	1.962	0.055541	0.343758	0	49.8
8.56	95.52	4	0	1.309	0.800611	0.97006	0	86.6
7.2	73.97	4	0	0.988	0.307909	0.795133	0	83.5
6.44	67.72	3	0	1.474	0.485977	0.903584	0	74.7
4.34	62.65	6	0	1.518	0.42143	0.87002	0	75.6
6.2	160.41	6	0	0.658	0.018734	0.173801	0	100.7
6.99	26.02	6	0	1.149	0.770812	0.97006	0	79.7
6.38	53.4	6	0	1.515	0.427064	0.874762	0	67.8
7.2	106.41	4	0	1.11	0.66719	0.948458	0	98
6.89	89.53	5	0	1.057	0.494983	0.906872	0	141.5
4.89	65.17	5	0	0.723	0.032816	0.250224	0	136.7
6.55	41.14	3	0	1.451	0.468707	0.896668	0	98
5.9	60.4	4	0	1.414	0.586509	0.922744	0	103.5
7.09	100.59	4	0	1.084	0.578531	0.920051	0	93
9.38	108.45	5	0	0.961	0.256132	0.739213	0	93.8
10.58	705.19	4	0	1.518	0.380223	0.851107	0	87.6
7.31	73.2	4	0	0.853	0.133648	0.569567	0	120.8
7.77	54.28	4	0	1.083	0.580362	0.920669	0	93.4
10.43	48.3	5	0	1.034	0.446669	0.888233	0	94
9.89	60.99	4	0	1.397	0.557413	0.910977	0	68
5.4	168.13	5	0	1.164	0.839826	0.977862	0	77
8.92	81.6	6	0	1.165	0.778954	0.97006	0	97.1
7.96	65.9	4	0	1.206	0.959919	0.994357	0	11.9
7.77	75.58	5	3	1.505	0.458009	0.891247	0	80.5
5.22	76.46	5	0	1.106	0.656707	0.943998	0	96.8
9.72	56.49	5	0	1.494	0.448939	0.888233	0	78.8
6.8	58.41	3	0	1.056	0.496456	0.906872	0	115.7
9.54	47.13	4	0	1.113	0.691812	0.952547	0	62.4
8.28	41.79	5	0	1.088	0.556149	0.910977	0	99.1
6.74	56.74	5	1	1.053	0.463032	0.893624	0	87.8
9.11	73.46	3	0	1.174	0.798877	0.97006	0	78.3
6.44	165.58	5	0	1.03	0.53627	0.90897	0	90.8
8.13	32.16	4	0	1.264	0.949333	0.991422	0	117.7
6.38	40.8	5	0	1.008	0.374148	0.84814	0	95.7
8.13	38.07	4	0	0.423	2.7E-06	7.86E-05	0	164.4
6.35	44.36	5	0	1.459	0.524554	0.90897	0	98.4
6.64	47.9	6	0	0.702	0.016488	0.159664	0	111.2
7.03	31.67	4	0	0.819	0.074046	0.410648	0	108.5
8.24	91.99	4	0	1.247	0.99416	0.99978	0	82.3
6.4	157.39	3	0	1.044	0.434309	0.882876	0	92.9

6.18	41.93	5	0	1	0.363137	0.840355	0	103.3
8.57	57.97	4	0	1.036	0.469524	0.896668	0	95.3
5.29	78.17	4	0	1.299	0.84166	0.977999	0	96.7
9.58	89.48	3	0	2.113	0.029753	0.235647	0	51.7
4.88	19.38	4	0	1.29	0.805872	0.97006	0	46.8
8.25	71.11	5	0	1.546	0.39213	0.861372	0	75.3
5.82	45.17	5	0	0.758	0.035753	0.264505	0	124
8.46	24.3	4	0	1.18	0.88711	0.983874	0	75.7
6.06	86.66	4	0	1.647	0.268838	0.752656	0	59.5
5.19	85.24	4	0	1.107	0.652643	0.942618	0	93.7
11.56	150.8	3	0	1.367	0.722191	0.96164	0	76.1
8.88	87.18	5	0	1.184	0.916818	0.98911	0	92.2
8.6	21.66	3	0	1.309	0.747451	0.964844	0	57.6
6.35	41.5	4	0	1.09	0.546707	0.909321	0	101.9
6.39	73.28	4	0	1.059	0.609344	0.927253	0	101.7
5.03	172.08	5	0	1.086	0.680332	0.951158	0	111.3
7.83	79.6	6	0	1.564	0.39357	0.861859	0	68.7
7.62	25.09	3	0	1.017	0.426337	0.874364	0	95.3
7.83	121.52	3	0	1.213	0.908459	0.988314	0	74.8
10.13	51.97	4	0	1.3	0.894055	0.985818	0	74
7.28	72.28	5	0	1.834	0.117986	0.535254	0	40.4
7.24	112.05	3	0	1.245	0.96425	0.995625	0	88.4
5.92	48.61	4	0	1.52	0.341483	0.819624	0	117.4
6.35	90.81	3	0	0.921	0.205381	0.683931	0	128.2
7.99	30.28	6	0	1.343	0.798128	0.97006	0	83.8
6.54	65.07	5	0	1.163	0.796094	0.97006	0	108.9
7.55	27.62	3	0	0.98	0.3362	0.814964	0	104.5
8.1	42.18	4	0	1.021	0.437017	0.88436	0	97.2
6.87	82.9	4	0	1.993	0.045306	0.303872	0	59.3
5.44	68.64	2	0	1.464	0.55901	0.910987	0	81.2
7.2	60.09	5	0	1.363	0.681177	0.951658	0	94.7
4.5	66.8	4	0	2.459	0.009533	0.106862	0	55.2
8.81	32.16	4	0	1.089	0.6251	0.932683	0	122.8
9.17	61.92	6	0	1.17	0.822514	0.974456	0	92.9
7.11	46.09	7	4	1.397	0.667183	0.948458	0	104.9
8.46	38.1	4	0	1.876	0.081585	0.438217	0	60.4
7.12	36.22	4	0	1.19	0.878714	0.983083	0	84.3
9.32	88.78	4	0	0.889	0.312774	0.798743	0	105.6
8	66.85	4	0	1.077	0.600582	0.925915	0	104.8
5.48	170.04	4	0	1.21	0.893142	0.985818	0	93.4
9.31	83.15	5	0	1.171	0.812763	0.971224	0	109.6
6.35	60.6	3	0	1.326	0.729411	0.962129	0	71.1
5.59	79.81	3	0	1.399	0.626357	0.932683	0	76.5
5.26	48.98	4	0	1.274	0.960728	0.994786	0	77.3
7.18	40.82	5	0	1.965	0.051408	0.328513	0	42.1

7.71	121.48	4	0	1.147	0.784325	0.97006	0	90.9
5.66	39.85	5	0	1.14	0.684491	0.952226	0	98.1
8.97	45.19	4	0	1.334	0.698953	0.957318	0	96.5
6.52	98.04	4	0	0.947	0.267422	0.751128	0	102
6.71	25.69	5	0	1.583	0.288381	0.772192	0	71.4
6.77	61.67	4	0	1.29	0.916158	0.98911	0	73
5.01	107.85	4	0	1.552	0.405105	0.862461	0	79
7.39	20.7	4	0	1.444	0.527607	0.90897	0	86.9
5.69	55	5	0	1.413	0.613681	0.929068	0	70.8
8.05	56.53	5	0	1.136	0.667439	0.948458	0	86.3
10.74	575.32	4	1	0.87	0.126782	0.551889	0	113.8
6.89	33.63	3	0	1.134	0.742307	0.964372	0	91.9
10.89	246.87	5	0	0.948	0.348847	0.827128	0	107.4
7.71	117.43	4	0	1.466	0.580981	0.920669	0	73.2
5.68	63.98	4	0	1.292	0.910331	0.98846	0	73.3
6.11	73.15	4	0	1.117	0.685301	0.952226	0	83.6
11	70	4	0	1.052	0.545242	0.90897	0	92.8
8.03	52.31	3	0	1.625	0.228938	0.707612	0	91.1
6.09	49.51	5	0	0.935	0.252607	0.734602	0	101.6
6.1	40.7	3	0	1.058	0.506435	0.90897	0	75.3
7.17	23.35	5	0	1.648	0.218896	0.698703	0	56.6
8.1	92.29	5	0	1.073	0.539695	0.90897	0	91.3
5.96	35.55	4	0	0.603	0.002859	0.044055	0	106.4
8.46	102.17	3	0	0.729	0.033762	0.255274	0	98.3
8.31	59.64	3	0	1.371	0.6128	0.929068	0	78.2
6.3	39.85	6	0	1.001	0.388174	0.859423	0	78.4
6.95	78.28	5	1	1.598	0.325345	0.806293	0	52.4
8.35	127.06	2	0	1.197	0.862334	0.981617	0	98.3
4.97	119.36	3	0	0.991	0.435258	0.88312	0	97.6
9.55	117.69	4	0	1.307	0.762057	0.966993	0	78.6
8.53	118.02	4	0	0.869	0.203804	0.682501	0	102.6
7.09	27.08	4	0	1.083	0.601238	0.925915	0	90.3
7.55	27.3	5	0	1.227	0.970027	0.997215	0	85.9
7.33	58.59	5	0	1.487	0.476634	0.898527	0	79.9
5.97	97.75	3	0	1.027	0.412505	0.864761	0	102.7
4.82	61.42	4	2	1.524	0.412008	0.864761	0	107.2
7.75	27.33	5	1	1.208	0.930731	0.99077	0	82.7
8.1	24.09	3	0	0.657	0.018349	0.17238	0	115.9
9.64	67.77	3	0	1.352	0.726825	0.96164	0	85.2
7.78	50.09	4	0	1.082	0.582198	0.920997	0	94.1
7.08	50.94	3	0	1.242	0.979799	0.997735	0	87.5
5.97	250.57	6	0	0.513	0.000951	0.017196	0	120.8
8.81	42.99	4	0	1.026	0.504154	0.908772	0	137.9
6.96	54.84	4	0	1.264	0.984535	0.997735	0	83.1
9.07	55.39	3	0	1.355	0.71311	0.960068	0	86.1

9.94	148.96	4	0	0.964	0.343355	0.821383	0	98.6
5.08	56.66	3	0	1.288	0.816848	0.972536	0	69
7.62	27.9	4	0	1.274	0.853999	0.981617	0	65.1
9.06	126.92	4	0	1.514	0.421687	0.870265	0	69.8
8.12	133.7	4	0	1.145	0.725014	0.96164	0	89.3
7.02	35.14	5	0	1.315	0.81042	0.97073	0	69.7
7.34	43.73	3	0	1.309	0.868895	0.981633	0	88.9
9.52	27.38	4	0	1.619	0.217501	0.697874	0	67.5
5.86	56.98	4	0	1.263	0.951818	0.991422	0	99.3
5.22	63.75	4	0	1.129	0.70715	0.958927	0	83.3
8.4	43.08	4	0	1.521	0.404032	0.862461	0	87.5
9.07	22.57	5	0	1.048	0.485683	0.903584	0	94.8
7.72	54.88	3	0	0.974	0.30768	0.795133	0	101.5
8.38	35.41	5	0	1.777	0.116713	0.532131	0	70.2
6.76	16.79	4	0	1.136	0.704958	0.958927	0	85.4
6.54	16.15	3	0	1.648	0.193708	0.669022	0	42.1
5.71	38.78	3	0	1.218	0.991648	0.999087	0	103.3
5.3	87.59	4	0	1.32	0.84401	0.97823	0	85.2
8.66	79.81	5	0	1.182	0.812869	0.971224	0	92.6
9.82	61.34	3	0	1.224	0.973349	0.997505	0	81.8
4.64	42.09	5	0	1.069	0.563422	0.912986	0	102.4
8.54	32.88	3	0	1.531	0.405327	0.862461	0	77.8
6.44	194.38	3	0	0.86	0.169869	0.637245	0	115.1
9.99	59.53	3	0	0.843	0.110478	0.51799	0	107.2
6.55	32.35	4	0	1.1	0.626847	0.932683	0	95.2
9.33	66.25	2	0	1.556	0.30728	0.795133	0	68.7
9.66	94.92	5	0	1.588	0.327482	0.806293	0	83.2
5.05	82.32	4	0	0.998	0.356179	0.832518	0	104.2
6.95	70.67	4	0	1.133	0.715551	0.960634	0	100.5
6.49	29.36	4	0	1.076	0.581918	0.920669	0	98.3
8.72	51.13	4	0	1.332	0.734207	0.962304	0	91
10.56	24.88	5	0	1.352	0.690173	0.952547	0	123.9
4.92	14.63	4	0	1.575	0.291644	0.778132	0	124.1
5.21	43.81	4	0	1.124	0.699688	0.957457	0	82.5
8.03	98.81	3	0	0.666	0.007213	0.087392	0	125.2
10.11	97.01	5	0	0.677	0.015017	0.149576	0	124
6.32	27.48	3	0	1.084	0.60712	0.927253	0	54.3
7.3	30.02	5	0	0.88	0.165318	0.633157	0	111
8.53	80.83	4	0	1.425	0.564136	0.913186	0	58.5
6.07	110.94	4	0	1.049	0.483284	0.901744	0	100.5
8.7	34.99	3	0	2.321	0.004302	0.060243	0	46.5
8.98	23.21	4	0	1.707	0.18172	0.651757	0	71.4
8.81	41.25	5	0	1.372	0.67626	0.951158	0	98.4
7.14	33.51	5	0	1.648	0.259081	0.740955	0	61.8
6.44	27.31	4	0	1.602	0.277546	0.763386	0	51.2

9.94	32.1	3	0	1.257	0.886772	0.983874	0	85.3
10.49	116.45	5	0	0.984	0.383576	0.854992	0	99.9
5.25	41.85	4	0	1.232	0.944409	0.991397	0	91.4
8.1	145.3	4	0	0.956	0.29813	0.787651	0	106.3
7.85	85.27	3	0	0.932	0.251656	0.734036	0	100.4
7.09	26.2	4	0	1.579	0.309114	0.796152	0	79.7
8.46	82.59	4	0	1.183	0.873498	0.983083	0	85.6
5.87	12.43	4	0	1.603	0.234262	0.711914	0	69.1
8.47	58.51	4	0	1.389	0.647635	0.940704	0	89.5
6.7	29.65	5	2	1.064	0.532443	0.90897	0	110.7
5.58	26.5	6	0	1.623	0.257524	0.740472	0	76.5
5.9	36.52	5	0	1.74	0.175243	0.643347	0	44.1
9.54	59.6	5	0	1.295	0.7913	0.97006	0	100.6
6.74	63.56	6	0	0.995	0.329274	0.806293	0	101.5
8.92	74.69	3	0	0.906	0.1628	0.62851	0	106.4
4.98	22.44	4	0	1.217	0.975603	0.997505	0	91.1
4.54	196.19	3	0	1.01	0.488143	0.904243	0	95.9
7.83	75.7	3	0	1.049	0.50094	0.906872	0	94.5
4.88	54.6	3	0	1.083	0.601385	0.925915	0	111.1
6.67	84.05	4	0	2.541	0.004822	0.065479	0	54.1
7.62	75.11	4	0	0.809	0.13164	0.564264	0	110.2
6.8	50.44	3	0	1.193	0.930163	0.99077	0	98
4.34	104.75	3	0	1.443	0.535894	0.90897	0	69.3
8.28	99.25	5	0	1.406	0.646007	0.940359	0	92.5
5.97	35.25	2	0	1.247	0.924078	0.989893	0	92.5
8.07	54.32	4	0	0.852	0.106346	0.507311	0	98.8
10.01	120.4	3	0	1.072	0.525289	0.90897	0	98.4
4.96	89.54	4	0	1.826	0.148675	0.600981	0	66.4
6.19	18.54	4	0	1.895	0.088849	0.458828	0	70.7
9.07	128.2	4	0	1.254	0.946088	0.991422	0	83
8.9	23.51	3	0	1.887	0.063797	0.374776	0	72.2
5.27	19.09	4	0	0.406	9.08E-07	2.78E-05	0	123.1
7.69	113.51	4	1	0.872	0.155368	0.616223	0	105.4
8.87	43.64	4	0	1.442	0.541436	0.90897	0	64.5
7.15	57.59	3	0	1.106	0.656854	0.943998	0	81.3
10.01	70.06	4	0	1.263	0.991999	0.999087	0	61.7
6.96	38.79	3	0	1.18	0.876181	0.983083	0	64.4
8.29	82.69	4	0	1.374	0.617061	0.930979	0	86.2
4.89	28.99	3	0	1.302	0.820447	0.973904	0	91.4
7.64	36.95	4	0	1.338	0.793461	0.97006	0	84.6
7.81	30.27	4	0	1.387	0.575504	0.919147	0	78.1
9.14	45.98	5	0	1.454	0.539347	0.90897	0	68.9
9.48	33.43	3	0	1.758	0.196364	0.673222	0	61
8.47	130.93	4	0	1.012	0.396569	0.861859	0	95.5
5.12	128.75	2	0	2.781	0.001602	0.026932	0	26.2

9.25	17.94	4	0	0.813	0.109887	0.516376	0	109.7
6.28	41.2	4	0	1.766	0.107039	0.508287	0	99.1
8.75	34.8	4	0	0.806	0.101051	0.496149	0	93.1
9.8	65.48	4	0	1.349	0.7065	0.958927	0	75.9
7.33	49.46	3	0	0.918	0.199151	0.677163	0	107.9
9.06	49.74	5	0	1.315	0.787035	0.97006	0	66.9
9.36	75.94	4	0	0.66	0.006933	0.084734	0	135.8
6.9	36.41	5	0	1.233	0.969359	0.997215	0	81.5
5.38	31.88	4	0	1.732	0.162351	0.628098	0	66.7
7.15	20.81	4	0	1.688	0.177855	0.645941	0	63
6.19	24.73	4	0	1.469	0.419159	0.867334	0	50.2
8.4	15.73	3	0	0.859	0.212906	0.694012	0	114.7
7.81	38.02	4	0	1.26	0.885876	0.98336	0	66.8
7.31	17.27	2	0	1.015	0.483757	0.90176	0	89.2
4.98	31.04	6	0	1.42	0.575447	0.919147	0	107.6
6.95	28.33	5	0	1.318	0.781444	0.97006	0	77.6
8.59	25.39	3	0	1.233	0.96321	0.995391	0	93
6.35	30.54	4	0	1.51	0.355675	0.832518	0	69.1
5.57	19.57	4	0	0.905	0.18945	0.663939	0	113.3
6.76	33.11	2	0	3.617	2.98E-05	0.000738	0	22.2
9.45	90.33	3	0	0.889	0.222957	0.701525	0	89.1
7.93	43.96	4	0	1.22	0.967189	0.996855	0	88.1
7.12	65.28	3	0	1.087	0.536941	0.90897	0	106.9
5.5	27	3	0	1.02	0.442394	0.888233	0	87
9.5	19.88	4	0	1.197	0.896615	0.986881	0	68.6
5.68	110.02	2	0	1.715	0.198884	0.677056	0	66
5.57	24.81	4	0	1.312	0.777109	0.97006	0	83.9
7.74	59.71	4	0	1.272	0.896369	0.98688	0	91.9
4.44	194.32	3	0	1.084	0.674878	0.951158	0	80.7
6.9	26.21	5	0	1.174	0.841252	0.977978	0	81.5
8.18	50.06	3	0	1.223	0.931887	0.990812	0	83.4
7.28	24.1	4	0	1.74	0.190301	0.663939	0	61.9
5.8	31.34	4	0	1.158	0.811551	0.971105	0	85.3
6.64	88.03	4	0	1.097	0.628664	0.932683	0	92.3
6.61	13.52	5	0	1.352	0.690633	0.952547	0	105.7
6.43	16.87	5	0	1.242	0.957302	0.992381	0	91.5
8.56	87.65	4	0	1.079	0.531702	0.90897	0	96
7.52	42.47	4	0	1.233	0.997726	0.99992	0	95.7
9.01	119.98	4	0	1.196	0.908753	0.988314	0	84.4
9.11	44.25	5	0	1.132	0.71626	0.960634	0	85.9
8.88	38.96	4	0	1.242	0.955988	0.991999	0	100.3
9.89	21.42	5	0	1.461	0.519373	0.90897	0	34.4
10.46	47.84	3	0	1.472	0.458036	0.891247	0	77.9
6.87	26.79	5	0	1.434	0.585604	0.922744	0	83.4
6.2	24.18	4	0	1.149	0.800324	0.97006	0	62.9

8.97	87.42	3	0	1.078	0.576139	0.91931	0	97.2
7.68	60.33	4	1	1.084	0.559957	0.911314	0	92.2
6.79	31.32	3	0	0.379	2E-05	0.000512	0	156.9
6.16	44.21	4	0	1.111	0.692007	0.952547	0	95.1
9.76	67.32	2	0	0.7	0.022104	0.196236	0	135.2
7.28	78.33	3	0	1.474	0.479997	0.898839	0	76.4
10.05	114.31	4	0	0.657	0.018864	0.174389	0	127.7
9.45	13.45	4	0	1.426	0.539233	0.90897	0	83.1
6.67	47.08	3	0	0.766	0.041799	0.291972	0	114.6
6.83	17	4	1	0.56	0.005551	0.072148	0	163.3
7.33	39.22	4	1	1.225	0.968916	0.997215	0	83.3
6.98	21.95	4	0	1.095	0.556103	0.910977	0	96.1
5.29	27.27	5	0	1.054	0.505658	0.90897	0	82.2
5.67	19.39	4	0	1.406	0.540659	0.90897	0	53.8
5.86	39	5	0	1.812	0.122696	0.542733	0	55.8
7.71	40.5	4	0	1.168	0.803452	0.97006	0	99.3
7.75	31.61	4	0	1.337	0.697825	0.956851	0	70.9
4.64	15.78	5	0	1.15	0.797463	0.97006	0	106.4
7.46	26.94	3	0	1.635	0.226152	0.702971	0	57.6
8.31	64.5	4	0	1.535	0.409753	0.862461	0	80.9
8.68	36.2	5	0	1.752	0.168738	0.63638	0	67.5
8.06	130.43	4	0	0.901	0.226488	0.703494	0	107.4
4.78	43.99	4	0	1.6	0.301936	0.791593	0	82.4
8.63	29.87	4	0	1.172	0.868139	0.981617	0	81.2
11.81	122.14	3	0	1.534	0.392125	0.861372	0	80.1
8.44	41.05	4	0	1.142	0.75742	0.966419	0	110.2
8.54	229.96	2	0	1.094	0.655412	0.943998	0	97.1
9.96	191.33	3	0	0.901	0.257516	0.740472	0	91.6
8.68	37.96	4	0	1.186	0.802713	0.97006	0	85.8
6.27	57.25	3	0	1.265	0.903395	0.987276	0	89.6
5.01	45.23	4	2	1.378	0.66644	0.948182	0	77.3
11.47	357.83	4	0	1.297	0.78513	0.97006	0	95.2
8.51	101.84	2	0	0.961	0.376007	0.850287	0	104.6
5.91	55.07	3	0	1.609	0.316124	0.800259	0	69
5.35	35.02	5	0	2.228	0.01788	0.169121	0	59.5
6.77	47.76	4	0	1.075	0.574781	0.919147	0	95.8
6.76	30.33	4	0	1.719	0.18115	0.651757	0	50.9
5.73	17.1	4	0	1.327	0.717133	0.960634	0	60.4
9.19	30.06	3	0	0.415	3.31E-06	9.48E-05	0	144.2
9.11	60.4	4	0	1.065	0.544189	0.90897	0	98.6
5.66	32.28	3	0	0.78	0.115698	0.531189	0	90.7
9.29	55.86	4	0	1.352	0.771308	0.97006	0	61.6
8.9	33.03	3	0	1.983	0.038937	0.27934	0	43.6
5.59	15.4	5	0	1.483	0.396034	0.861859	0	53.4
7.93	50.13	3	0	0.856	0.117949	0.535254	0	110.5

7.97	168.88	4	1	1.329	0.722801	0.96164	0	95.9
8.38	81.55	3	0	1.33	0.738441	0.96414	0	98.4
6.96	31.01	4	0	0.838	0.101972	0.497489	0	117.4
4.94	45.24	4	0	1.106	0.687824	0.952468	0	92.1
5.88	34.16	3	0	1.484	0.516266	0.90897	0	88.1
5.68	34.29	5	1	1.602	0.307317	0.795133	0	79.5
4.74	23.15	4	0	0.782	0.065102	0.379785	0	126.7
5.58	21.91	4	0	1.154	0.783436	0.97006	0	111.7
4.93	44.93	3	0	1.363	0.751332	0.96537	0	32.1
7.24	115.96	4	1	1.154	0.774751	0.97006	0	91
9.76	16.1	3	0	2.181	0.010208	0.112824	0	36.2
5.43	33.27	4	0	1.719	0.137547	0.578504	0	58.6
9.09	82.54	3	0	1.366	0.722852	0.96164	0	94.8
5.86	102.37	3	0	0.887	0.202558	0.682501	0	101.8
8.81	47.74	3	0	1.591	0.296472	0.785454	0	83.3
9.16	90.01	2	0	1.026	0.422835	0.872243	0	112
8.47	56.97	3	0	1.197	0.930879	0.99077	0	55.7
5.39	18.03	4	0	1.143	0.773015	0.97006	0	127.5
10.35	347.62	3	0	0.997	0.457457	0.891247	0	92.4
6.84	65.64	3	0	1.111	0.688745	0.952468	0	104
6.96	36.57	3	0	1.261	0.923627	0.989893	0	90.3
6.09	27.05	3	0	1.51	0.380048	0.851107	0	79.9
5.67	42.16	2	0	1.766	0.11774	0.534716	0	83.4
6.13	42.73	3	0	1.277	0.831064	0.975858	0	82.1
8.03	23.19	5	0	0.896	0.174886	0.643192	0	107
7.25	49.63	3	0	1.117	0.684727	0.952226	0	94.3
10.13	122.89	3	0	1.261	0.914296	0.98911	0	82.1
6.74	29.68	2	0	1.701	0.167758	0.636322	0	75.6
5.38	78.38	3	0	1.101	0.653461	0.943181	0	115.4
5.96	92.81	3	0	1.104	0.638494	0.937605	0	100
7.42	75.13	4	0	1.837	0.078337	0.426	0	57.4
8.25	35.29	4	1	1.492	0.367541	0.845865	0	82.4
5.22	38.13	3	0	1.219	0.944772	0.991397	0	91.5
8.53	32.87	3	0	1.683	0.182739	0.653552	0	43.1
9.86	118.37	2	0	0.631	0.007405	0.08896	0	120.7
8.56	59.39	3	0	1.637	0.222021	0.701127	0	63.3
9.82	17.46	4	0	1.849	0.06339	0.372904	0	56
7.01	27.29	3	0	0.742	0.121137	0.540013	0	119.1
9.1	45.78	4	0	0.802	0.175508	0.643974	0	139.5
9.67	94.93	3	0	1.322	0.797413	0.97006	0	74.6
10.36	219.79	5	0	2.206	0.020425	0.18504	0	72.8
10.04	41.88	3	0	0.876	0.16084	0.623978	0	105.9
6.09	42.77	3	0	2.004	0.027907	0.225492	0	61.5
9.29	88.07	4	0	1.217	0.980012	0.997735	0	85.9
6.21	20.09	4	0	1.377	0.586939	0.922744	0	84

8.79	45.24	3	0	1.621	0.289832	0.775164	0	66
9.39	36.47	3	0	1.457	0.512925	0.90897	0	82.9
5.58	51.48	3	0	0.958	0.288313	0.772192	0	107
8.19	51.82	5	0	1.04	0.465538	0.89585	0	95.2
9.91	73.9	3	0	1.41	0.580806	0.920669	0	68.7
8.59	29.89	4	0	1.312	0.855101	0.981617	0	94
7.68	64.73	3	0	1.198	0.94267	0.991397	0	91.3
9.95	25.94	3	0	1.045	0.461458	0.892914	0	105.6
8.51	97.92	3	0	1.565	0.369698	0.846091	0	79.6
7.05	46.21	3	0	1.329	0.764588	0.967498	0	78.7
4.41	142.63	3	1	0.783	0.094617	0.476451	0	107.9
10.54	84.47	3	0	1.261	0.923333	0.989893	0	97.1
7.9	15.3	3	0	1.106	0.624445	0.932683	0	115.4
6.65	38.78	4	0	1.351	0.710394	0.959398	0	84.1
5.44	38.56	3	0	1.214	0.882436	0.983083	0	107.3
7.99	108.88	3	0	1.048	0.468505	0.896668	0	94.8
8.56	42.31	4	0	1.016	0.389802	0.859423	0	88.3
7.87	26.89	2	0	0.984	0.35172	0.829488	0	99.4
8.59	44.93	3	0	0.946	0.266686	0.750236	0	98.1
9.29	75.67	3	0	1.491	0.434249	0.882876	0	88.3
4.63	82.55	4	0	1.49	0.483276	0.901744	0	67.2
8.22	23.34	3	0	0.988	0.367735	0.84602	0	99.4
8.24	21.09	5	0	1.63	0.218249	0.697874	0	63.1
9.7	75.08	4	0	1.052	0.580128	0.920669	0	104.5
8.37	20.84	4	0	1.861	0.097338	0.48322	0	72.6
8.48	82.2	3	0	0.886	0.182433	0.652641	0	111.9
6.52	35.09	4	0	1.002	0.388808	0.859423	0	94.9
8.47	29.63	4	0	0.925	0.205685	0.683931	0	70.7
8.62	69.62	3	0	0.914	0.206761	0.68606	0	111.3
8.15	27.6	5	0	1.26	0.998481	0.99992	0	87.3
5.53	105.95	3	0	0.43	2.81E-05	0.000699	0	128.7
6.7	33.34	3	0	1.031	0.450305	0.888233	0	91.3
7.31	47.05	3	0	1.375	0.586302	0.922744	0	86
7.17	170.63	2	0	1.746	0.156181	0.616351	0	75.6
5.54	66.35	4	0	0.579	0.002917	0.044841	0	131.7
8.22	20	3	0	1.206	0.960281	0.99465	0	114.2
6.89	76.27	4	0	1.085	0.548063	0.909321	0	97.7
9.94	43.94	4	0	1.4	0.608478	0.927253	0	102.9
5.85	57.51	2	0	0.681	0.042187	0.294073	0	105.3
7.24	77.11	3	0	1.712	0.201959	0.682501	0	48.9
7.28	58.45	2	0	0.865	0.118706	0.535654	0	76.1
6.25	56.48	4	0	1.459	0.486496	0.903584	0	72.7
7.64	51.7	5	0	0.866	0.145077	0.596922	0	110.9
5.33	41.96	2	0	1.22	0.998044	0.99992	0	82
4.98	10.29	2	0	1.853	0.118851	0.536086	0	44.3

9.33	59.69	4	0	0.817	0.087537	0.454248	0	108.3
7.18	48.23	4	0	1.263	0.903797	0.987276	0	83
9.57	18.35	4	0	1.26	0.882737	0.983083	0	49.5
8.95	101.68	4	0	2.351	0.005706	0.073523	0	68.3
7.91	18.04	3	0	1.086	0.62995	0.933268	0	100.8
5.3	59.34	3	0	1.277	0.943956	0.991397	0	91.2
6.61	8.79	4	0	1.195	0.883009	0.9831	0	84.2
8.87	72.78	3	0	1.126	0.773692	0.97006	0	88.1
5.02	25.41	3	0	1.344	0.677146	0.951158	0	88.1
8.95	48.78	2	0	1.796	0.083307	0.440653	0	72.1
7.14	38.14	4	0	0.945	0.246176	0.725581	0	125.2
7.65	38.22	4	0	1.539	0.343126	0.82125	0	75.4
8.82	75.59	4	0	1.285	0.883142	0.9831	0	84.6
9.88	53.89	3	0	0.758	0.049774	0.32349	0	112.2
6.98	19.74	3	0	1.851	0.067222	0.386072	0	58.3
7.43	95.2	2	0	1.889	0.10113	0.496344	0	89.1
5.59	12.51	3	0	1.479	0.40249	0.862461	0	76.2
5.54	138.39	3	0	1.11	0.743021	0.964372	0	57.6
5.11	39.28	3	0	1.108	0.675381	0.951158	0	86.2
7.87	84.27	3	0	1.75	0.175094	0.643347	0	70.7
10.43	99.76	4	3	1.26	0.872343	0.983058	0	59.4
7.47	37.52	4	0	1.202	0.898862	0.987004	0	81
10.01	105.44	3	0	0.829	0.104903	0.502905	0	101.8
6.46	45.99	2	0	0.817	0.095554	0.478873	0	103.2
5.76	52.51	4	0	1.141	0.72405	0.96164	0	73.2
7.77	42.68	5	0	1.13	0.751375	0.96537	0	91.9
7.2	18.57	3	0	1.572	0.397779	0.862453	0	85
7.88	40.95	4	0	0.952	0.261951	0.746056	0	138.1
7.9	37.03	2	0	1.078	0.657104	0.943998	0	52.4
5.72	43.21	4	1	1.131	0.798788	0.97006	0	119.4
9.94	47.85	3	0	1.506	0.408547	0.862461	0	87.2
10.07	52.15	3	0	1.185	0.866327	0.981617	0	98.6
9.26	51	2	0	1.261	0.925216	0.99022	0	78.6
7.25	29.92	4	0	1.457	0.443215	0.888233	0	51
8.44	45.83	4	0	1.592	0.362385	0.840171	0	70.5
8.1	34.99	4	0	0.801	0.127698	0.55391	0	106
6.23	73.29	2	0	1.026	0.43582	0.883358	0	100.3
5.34	52.13	4	0	1.107	0.619441	0.932207	0	92.4
8	26.58	4	0	0.769	0.041663	0.29155	0	113.5
5.49	40.45	5	0	1.806	0.109661	0.515698	0	55.2
5.14	15.35	3	0	1.052	0.492819	0.906679	0	93.5
5.17	53.69	3	0	1.708	0.196415	0.673222	0	76.7
5.72	61.12	2	0	2.033	0.027415	0.222926	0	54.5
9.42	56.87	2	0	1.406	0.641569	0.938716	0	82.4
5.72	45.44	3	0	1.061	0.515016	0.90897	0	112.7

5.76	27.83	4	0	0.899	0.162579	0.628295	0	107.6
5	48.95	4	0	2.206	0.02041	0.18504	0	75.5
7.62	20.14	3	0	1.776	0.206675	0.685956	0	33.3
8.85	57.55	4	0	1.084	0.561299	0.911753	0	92.4
4.87	38.94	2	0	1.704	0.191727	0.666276	0	94.9
8.4	45.99	3	1	1.029	0.454171	0.888233	0	105.4
5.52	25.32	3	0	1.019	0.376748	0.850346	0	110.5
8.56	35.44	4	0	1.121	0.6265	0.932683	0	96.1
8.63	129.54	2	0	2.387	0.008717	0.099938	0	61.8
6.89	175.54	2	0	1.425	0.576197	0.91931	0	83.4
9.04	9.72	4	0	0.648	0.005165	0.068223	0	119.8
8.18	39.73	3	0	1.008	0.407638	0.862461	0	103.1
8.87	24.09	4	0	1.729	0.20902	0.689196	0	70.5
6.65	46.05	4	0	1.221	0.907898	0.988314	0	78.4
7.18	56.08	4	0	1.161	0.792207	0.97006	0	76.5
7.52	162.84	3	0	1.212	0.973855	0.997505	0	86.7
10.15	126.44	3	0	1.046	0.475846	0.898384	0	99.9
5.95	27.17	4	0	1.199	0.937275	0.99128	0	88.6
7.99	42.48	4	0	1.464	0.49508	0.906872	0	8
7.94	33.95	3	0	1.32	0.715482	0.960634	0	56.2
5.83	70.72	4	0	1.249	0.919577	0.98911	0	76
5.34	62.52	3	0	1.231	0.954846	0.991422	0	102.5
9.31	25.91	3	0	1.204	0.961864	0.995026	0	75.4
8.79	37.87	2	1	1.076	0.538004	0.90897	0	91.9
9.33	75.5	2	0	0.959	0.29082	0.777146	0	102.6
6.11	8.34	2	0	2.274	0.037992	0.275191	0	63
10.04	127.51	3	0	1.578	0.314939	0.800259	0	74.5
8.1	49.02	2	0	1.686	0.144923	0.596922	0	69.2
11.12	271.71	4	3	0.994	0.451859	0.888233	0	104.3
7.2	14.72	4	0	1.196	0.938046	0.99128	0	83.3
10.52	41.49	3	0	1.199	0.840007	0.977862	0	73.4
6.1	19.63	4	0	1.214	0.988696	0.998053	0	88.3
8.59	98.01	3	0	1.509	0.392392	0.861647	0	79.9
8.63	40.53	4	0	1.095	0.609355	0.927253	0	146.3
7.47	48.66	3	0	1.016	0.512769	0.90897	0	95.4
5.94	18.95	4	0	1.744	0.122316	0.542733	0	46.2
5.11	40.65	2	0	1.526	0.37024	0.846091	0	88.3
8.4	101.07	3	0	0.01	1E-17	3.97E-16	0	200
7.44	37.98	4	0	0.903	0.222068	0.701127	0	111.4
5.2	32.72	4	0	1.18	0.820613	0.973951	0	82.9
6.58	48.71	4	0	1.196	0.832678	0.976224	0	105.6
7.01	72.81	3	0	1.253	0.889066	0.984355	0	101.2
6.43	33.46	3	0	1.481	0.416851	0.867107	0	84.4
8.05	11.78	4	0	1.217	0.984443	0.997735	0	77.9
4.78	54.09	3	0	1.184	0.833012	0.976224	0	87.8

6.15	10.05	3	0	1.877	0.154751	0.615875	0	63
8.31	189.11	3	0	0.842	0.122322	0.542733	0	108.1
6.96	16.48	4	0	1.248	0.946739	0.991422	0	125
4.97	46.27	3	0	2.511	0.00412	0.058012	0	54.4
4.41	131.86	4	0	3.564	0.000186	0.003944	0	40.7
8.1	45.72	4	0	1.101	0.636271	0.937605	0	97.9
6.71	44.07	2	0	1.754	0.106403	0.507351	0	54.1
9.45	226.58	2	1	1.052	0.592967	0.924683	0	91.7
4.6	18.77	3	0	1.372	0.618696	0.932116	0	71.2
7.01	38.27	4	0	1.955	0.06984	0.394889	0	70.5
8.06	82.65	3	0	0.849	0.122914	0.542991	0	113.9
9.09	32.16	4	0	1.03	0.462478	0.893486	0	111.3
7.49	15.93	3	0	0.899	0.272716	0.75652	0	104.2
8.03	28.67	3	0	1.249	0.947518	0.991422	0	91.7
7.12	20.9	4	0	1.78	0.115151	0.529645	0	47.8
9.03	62.61	3	0	0.887	0.147523	0.599278	0	99
6.81	8.91	3	0	1.241	0.963548	0.995498	0	80.4
8.54	45.05	2	0	1.504	0.403914	0.862461	0	64.6
6.33	44.33	2	0	1.213	0.971792	0.997505	0	100.3
9.47	210.01	2	0	0.863	0.132844	0.566984	0	104.5
10.08	74.95	3	0	1.419	0.634878	0.936435	0	86.5
8.02	66.17	3	0	0.858	0.107157	0.508287	0	120.7
4.73	31.25	2	0	1.208	0.892282	0.985818	0	87.8
9.57	35.96	5	0	1.272	0.909751	0.988314	0	89.4
5.94	36.64	2	0	1.13	0.767574	0.968389	0	94.2
7.12	37.48	4	0	1.146	0.704073	0.958927	0	92.1
5.17	24.01	4	0	1.248	0.962699	0.995026	0	86.4
4.98	29.66	3	0	1.096	0.685689	0.952226	0	92.1
7.88	20.42	2	0	1.128	0.790001	0.97006	0	98.5
10.23	33.29	3	0	1.053	0.521811	0.90897	0	109.2
8.78	28.71	3	0	1.063	0.523133	0.90897	0	97.6
9.35	19.89	2	0	2.128	0.050912	0.327386	0	77.1
8.51	19.79	3	0	0.675	0.013406	0.138226	0	122.7
10.32	101.2	4	0	0.812	0.084388	0.443601	0	109.8
8.48	55.02	3	0	1.282	0.898786	0.987004	0	86.3
9.82	111.34	3	0	0.99	0.321361	0.803178	0	103.6
8.92	28.7	2	0	1.929	0.062248	0.367735	0	61.1
8.43	52.63	4	0	1.208	0.913709	0.98911	0	84.4
5.01	21.81	3	0	1.21	0.974152	0.997505	0	84
6.48	33.74	3	0	1.191	0.860699	0.981617	0	97.5
6.79	58.49	2	0	0.887	0.143892	0.59581	0	103.3
7.77	14.14	2	0	0.917	0.234757	0.711914	0	101.9
10.24	55.18	4	0	1.078	0.543807	0.90897	0	100.5
6.57	30.79	3	0	1.003	0.344975	0.822985	0	100.7
6.65	73.95	2	0	1.237	0.989265	0.998146	0	90.1

5.63	33.56	4	0	1.265	0.881124	0.983083	0	77.4
8.95	41.67	2	0	1.324	0.728631	0.962129	0	106.7
7.99	23.29	4	0	0.968	0.318467	0.802274	0	103.6
7.52	32.87	3	0	1.047	0.501125	0.906872	0	88.4
6.55	23.45	3	0	1.395	0.580478	0.920669	0	117.6
7.72	44.26	2	0	1.261	0.941882	0.991397	0	91.5
6.51	32.74	3	0	1.087	0.610661	0.928335	0	120.3
9.67	81.44	3	0	1.363	0.759337	0.966419	0	93
6.13	24.5	4	0	0.955	0.381995	0.853306	0	87.1
8.59	13.22	3	0	0.806	0.110275	0.517618	0	114.2
6.14	50.51	2	0	1.012	0.370304	0.846091	0	111.8
10.26	157.86	3	0	1.43	0.523828	0.90897	0	89.2
8.31	41.21	3	0	0.851	0.130833	0.562137	0	105.4
6.16	38.5	4	0	1.074	0.551552	0.910336	0	90.9
7.75	42.47	3	0	1.234	0.952134	0.991422	0	89.4
8.7	48.06	4	0	1.223	0.938745	0.99128	0	104.2
8.66	24.72	4	0	1.367	0.629463	0.933166	0	80
8.1	13.34	3	0	1.639	0.288286	0.772192	0	84.9
9.44	87.86	3	0	1.308	0.833828	0.976224	0	105.9
6.48	17.74	3	0	1.351	0.709919	0.959398	0	116.1
7.88	14.71	4	0	1.046	0.476306	0.898527	0	118.9
5.74	47.1	3	0	0.899	0.169957	0.637245	0	100.1
7.25	10.01	3	0	0.672	0.008951	0.102053	0	105.3
4.92	35.48	3	0	1.669	0.199161	0.677163	0	63.1
7.8	50.45	2	0	1.366	0.684145	0.952102	0	95.7
7.37	39	2	0	1.265	0.880872	0.983083	0	88.9
6.81	25.92	2	0	1.174	0.89418	0.985818	0	83.1
11.46	137.82	4	0	0.867	0.148272	0.60077	0	98
6.6	14.27	4	0	1.19	0.912251	0.989005	0	72.3
8.15	22.05	3	0	1.135	0.797943	0.97006	0	91.9
8.72	46.72	3	0	1.592	0.273426	0.756978	0	83
6.54	43.13	3	0	1.273	0.841814	0.977999	0	115.7
6.68	16.11	3	0	1.116	0.720176	0.96164	0	165.6
8.72	10.74	3	0	1.353	0.755861	0.96597	0	78.5
8.81	98.71	3	0	1.285	0.868071	0.981617	0	96.8
11.27	278.01	4	0	0.924	0.210025	0.690199	0	101.3
8.56	36.99	4	0	1.353	0.71702	0.960634	0	77.3
7.72	13.79	4	0	1.381	0.663296	0.946953	0	78.4
4.49	15.83	3	0	1.113	0.604599	0.925915	0	99.3
8.84	18.91	3	0	1.179	0.898595	0.987004	0	84.1
9.33	26.92	4	0	1.368	0.621933	0.932683	0	76.1
5.24	63.84	2	0	1.316	0.777912	0.97006	0	88.6
6.44	44.54	3	0	1.063	0.521952	0.90897	0	123.4
6.7	19.46	3	0	1.197	0.938108	0.99128	0	83.2
9.72	155.45	3	0	1.399	0.595049	0.925915	0	91.6

7.43	82.89	4	0	1.053	0.501598	0.907273	0	97.7
5.06	59.21	3	0	2.007	0.128068	0.55391	0	67
6.25	30.91	3	0	0.777	0.062145	0.36753	0	119.9
5.15	15.23	3	0	1.338	0.81545	0.972276	0	84.5
5.52	20.23	2	0	1.021	0.439923	0.887298	0	159.7
7.18	23.76	3	0	1.392	0.604572	0.925915	0	79.2
10.99	121.77	3	0	1.969	0.063612	0.374035	0	67.2
7.15	213.94	3	0	1.471	0.597919	0.925915	0	84.8
8.73	25.23	3	0	1.168	0.823893	0.974465	0	95.2
9.04	24.99	3	0	1.014	0.395603	0.861859	0	129
7.37	12.48	3	0	1.957	0.087423	0.45384	0	85.6
10.51	9.04	3	0	1.468	0.491334	0.905851	0	79.3
9.5	22.64	3	0	0.866	0.114492	0.529344	0	99
7.06	26.11	3	0	1.241	0.934124	0.991048	0	89.2
8.1	25.95	3	0	1.225	0.982102	0.997735	0	95.6
6.89	17.55	4	0	0.719	0.033566	0.254553	0	118.3
5.17	23.74	2	0	0.679	0.032748	0.249856	0	113.5
8.28	34.61	3	0	1.004	0.410725	0.863065	0	105.6
9.31	20.3	3	0	1.274	0.888179	0.984008	0	87.3
8.16	54.74	3	0	1.309	0.816927	0.972536	0	81.3
6.4	37.07	3	0	0.868	0.208292	0.688238	0	101.5
5.12	26.11	2	0	1.072	0.539544	0.90897	0	65.4
9.38	33.9	4	0	2.123	0.041297	0.290284	0	41.6
9	33.35	3	0	1.348	0.707913	0.959206	0	85.5
8.6	10.51	4	0	0.888	0.201536	0.681843	0	101.8
6.28	66.85	2	0	1.186	0.861592	0.981617	0	92
9.57	36.43	3	0	0.517	0.000589	0.011435	0	164.5
6.44	14.75	3	0	1.267	0.916312	0.98911	0	91.6
7.08	26.09	4	0	1.452	0.537158	0.90897	0	78.7
8.25	29.8	3	0	1.129	0.757699	0.966419	0	100.8
6.34	79.54	3	0	1.189	0.860972	0.981617	0	78.4
11.63	46.61	4	0	1.971	0.064504	0.377826	0	39.6
7.72	6.25	4	0	1.229	0.969321	0.997215	0	57
6.13	18.32	3	0	1.846	0.091097	0.46581	0	107.8
7.11	16.18	3	0	1.263	0.90442	0.987276	0	99.5
6.11	24.39	2	0	1.115	0.752755	0.96597	0	95.1
8.59	28.51	2	0	1.121	0.714772	0.960203	0	86.2
6.14	22.92	3	0	1.097	0.64466	0.9403	0	76.2
8.12	37.84	3	0	1.469	0.473268	0.898019	0	55.9
6.25	19.39	2	0	1.851	0.124195	0.547442	0	57.3
4.96	68.75	3	0	1.023	0.391905	0.861372	0	106.5
6.58	18.98	3	0	1.052	0.479419	0.898527	0	84.3
5.54	87.16	2	0	2.866	0.001054	0.018876	0	41.3
6.95	34.42	3	0	1.175	0.877305	0.983083	0	105.5
7.46	18.72	3	0	0.824	0.098847	0.488567	0	96.3

7.75	19.74	3	0	1.158	0.787051	0.97006	0	117.3
8.57	27.52	3	1	1.272	0.90276	0.987276	0	85.3
6.54	33.43	2	0	1.555	0.290814	0.777146	0	85.9
5.83	26.02	3	0	1.396	0.625618	0.932683	0	109.6
5.78	38.44	2	0	4.43	2.63E-07	8.43E-06	0	20.3
6.38	17.64	3	0	0.763	0.079794	0.431496	0	97.9
5.29	33.27	2	0	1.673	0.236001	0.71344	0	72.9
5.68	57.49	2	0	1.223	0.994878	0.99978	0	94.8
8.98	18.87	2	0	1.05	0.495545	0.906872	0	98.4
10.83	68.65	2	0	1.319	0.789233	0.97006	0	89.3
8.15	109.58	3	0	1	0.385969	0.856625	0	100.1
7.37	16.46	4	1	0.79	0.066497	0.383654	0	89.8
7.06	10.58	3	0	0.805	0.070234	0.396493	0	103.3
9.55	46.69	3	0	1.39	0.695392	0.955463	0	98.2
7.44	31.4	3	0	1.047	0.482674	0.90163	0	127.8
5.58	33.08	3	0	1.364	0.733055	0.962196	0	85.2
9.41	43.76	3	0	1.583	0.291241	0.777817	0	75.5
5.6	26.8	4	0	1.138	0.760852	0.966437	0	123.3
6.84	97.38	3	0	1.748	0.166063	0.635043	0	69.1
5.25	77.44	3	0	1.095	0.61198	0.929068	0	97.1
6.87	52.36	1	0	1.493	0.400739	0.862461	0	78.8
4.92	246.88	2	0	1.106	0.731502	0.962196	0	95.9
6.73	35.82	4	0	1.228	0.957235	0.992381	0	102.9
9.63	66.65	1	0	0.837	0.11648	0.532131	0	104.4
5.05	11.74	2	0	1.022	0.445126	0.888233	0	68
6.18	28.74	2	0	1.429	0.474435	0.898019	0	90
5.72	96.55	3	0	1.165	0.809964	0.970646	0	101.1
8.28	26.79	3	0	1.233	0.954553	0.991422	0	82.2
7.05	9.71	2	0	1.115	0.735414	0.96266	0	107.5
9.48	77.69	1	0	1.447	0.548933	0.909321	0	95
7.49	19.95	3	0	1.222	0.992022	0.999087	0	109.7
8.47	10.81	3	0	1.237	0.986625	0.997735	0	49.1
5.29	17.81	3	0	1.054	0.552438	0.910945	0	76.5
7.21	94.62	3	0	5.041	2.13E-05	0.000542	0	33.1
8.32	22.14	2	0	1.491	0.419594	0.867775	0	77.1
6.42	41.74	3	0	1.45	0.470902	0.897802	0	61.9
6.55	28.91	3	1	1.359	0.709844	0.959398	0	85.3
6.89	19.21	3	0	1.434	0.531297	0.90897	0	67.1
7.3	171.25	2	0	0.987	0.389408	0.859423	0	103.6
7.56	11.22	2	0	1.408	0.527106	0.90897	0	84.1
6.38	41.66	3	0	1.613	0.225981	0.702787	0	47.5
9.58	26.26	4	0	1.362	0.701287	0.958482	0	88.7
7.68	26.98	2	0	0.646	0.005811	0.074493	0	109.3
6.28	17.91	3	0	1.489	0.474583	0.898019	0	65.9
6.16	39.78	3	0	1.28	0.881171	0.983083	0	58.4

7.08	27.89	2	0	1.737	0.264394	0.748141	0	77.4
8.51	14.12	3	0	1.229	0.985524	0.997735	0	78
9.01	46.06	3	0	1.353	0.660022	0.945604	0	85.6
9.25	16.91	2	0	0.98	0.435422	0.88312	0	42.1
8.88	44.7	2	0	0.91	0.172612	0.640909	0	118.2
4.92	33.57	2	0	1.171	0.824661	0.974465	0	78.8
9.04	38.39	3	0	0.953	0.281971	0.76495	0	98.4
9.98	26.63	3	0	1.611	0.243823	0.725132	0	85.3
6.51	84.7	2	0	1.222	0.98355	0.997735	0	91.5
7.36	13.64	4	0	1.107	0.63816	0.937605	0	99.6
9.5	28.85	3	0	1.495	0.431362	0.879563	0	60.6
9.88	63.19	2	0	1.09	0.592645	0.924683	0	89
5.17	30.42	3	0	0.971	0.3267	0.806293	0	107.3
6.32	12.64	3	0	0.945	0.232938	0.711914	0	91.9
5.86	25.32	3	0	1.57	0.427007	0.874762	0	82.2
6.49	17.14	2	0	1.323	0.708981	0.959398	0	87.1
9.66	48.16	2	0	1.111	0.656384	0.943998	0	94.7
6.92	9.54	3	0	1.488	0.531687	0.90897	0	73.2
6.23	24.86	3	0	1.643	0.218527	0.698217	0	80
5.29	33.52	3	0	1.813	0.093397	0.47163	0	71.1
7.46	55.65	3	0	1.092	0.604724	0.925915	0	102.8
9.39	123.97	2	0	1.502	0.441076	0.887814	0	81.8
8.25	14.2	3	0	1.179	0.8415	0.977999	0	113.5
6.81	18.73	2	0	1.16	0.830082	0.975858	0	101.6
8.76	28.82	2	0	0.807	0.088894	0.458828	0	113.9
9.19	35.79	3	0	0.705	0.015385	0.151197	0	108
6.35	17.09	2	0	1.981	0.077329	0.421973	0	64.6
11.37	101.94	3	0	0.494	0.003911	0.056523	0	134.8
6.25	26.85	3	0	1.069	0.549	0.909321	0	81.7
8.34	15.29	2	0	1.573	0.347505	0.825423	0	52.4
7.5	23.75	3	0	1.452	0.490123	0.905247	0	87.3
6.13	44.02	2	0	1.399	0.618884	0.932176	0	87.2
5.55	31.49	4	0	0.988	0.440598	0.887298	0	104.3
10.18	22.11	3	0	1.743	0.13407	0.570205	0	66.4
7.17	22.75	2	0	1.057	0.535528	0.90897	0	99.1
6.83	22.08	3	0	1.203	0.898067	0.987004	0	93.3
8.63	15.53	3	0	1.142	0.787985	0.97006	0	92
6.14	10.3	2	0	0.956	0.384535	0.854992	0	103.2
8.59	66.95	3	0	0.923	0.194675	0.670706	0	111.8
8.68	52.3	3	0	1.08	0.572245	0.918743	0	87.4
9.58	42.31	3	0	1.323	0.752697	0.96597	0	71.7
8.76	13.36	3	0	2.028	0.067492	0.386559	0	69.2
8.12	27.59	2	0	1.163	0.854764	0.981617	0	85
9.99	92.32	2	0	1.009	0.373161	0.847074	0	98.7
5.24	37.93	4	1	1.626	0.20866	0.688551	0	84.8

6.58	42.27	3	0	0.777	0.062597	0.369278	0	127.5
6.92	25.04	3	0	1.002	0.371848	0.846203	0	98.4
6.48	20.75	4	0	1.368	0.616963	0.930979	0	49.5
10.83	75.22	3	0	1.62	0.296826	0.785775	0	66.5
8.75	17.71	3	0	1.627	0.260966	0.745135	0	61.1
8.22	6.15	3	0	1.238	0.933881	0.991048	0	87.2
8.15	23.71	3	0	1.148	0.713801	0.960068	0	95.3
8.35	16.65	3	0	0.916	0.286817	0.770436	0	114.3
8.29	34.78	3	0	1.136	0.774199	0.97006	0	91.4
9.11	55.34	2	0	1.494	0.441121	0.887814	0	59.7
6.34	18.99	3	1	1.298	0.814343	0.971737	0	83
5.12	19.84	1	0	0.872	0.231424	0.711304	0	128.7
9.19	126.4	2	0	1.268	0.938088	0.99128	0	90.1
8.19	9.63	3	1	1.978	0.076353	0.417553	0	59.8
8.98	49.23	3	0	1.085	0.526738	0.90897	0	105.4
8.07	91.87	3	0	1.14	0.821492	0.974168	0	144.6
5.45	111.74	3	0	1.396	0.579231	0.920235	0	91.8
10.71	6.59	2	0	1.653	0.217331	0.697874	0	93.3
5.53	17.77	4	0	1.266	0.870963	0.982839	0	75.8
6.57	14.25	3	0	1.331	0.71098	0.959398	0	72.5
5.1	12.28	3	0	1.586	0.413091	0.864761	0	133.7
7.23	22.73	2	0	1.298	0.774507	0.97006	0	102.5
4.79	19.85	2	0	1.072	0.514056	0.90897	0	92
9.63	27.41	2	0	0.964	0.346578	0.824064	0	107.5
6.51	11.03	3	0	0.784	0.114581	0.529344	0	114.9
4.82	27.96	4	0	1.141	0.755397	0.96597	0	96.8
8.02	58.69	3	0	1.583	0.356132	0.832518	0	77.4
4.68	49.18	1	0	1.151	0.816126	0.972341	0	89.7
7.3	18.77	3	0	1.239	0.96733	0.996855	0	80.8
7.5	69.14	2	0	1.656	0.251466	0.734036	0	80.5
6.19	48.87	3	0	1.386	0.640063	0.937605	0	14.4
5.99	38.74	2	0	0.94	0.254474	0.735945	0	99.1
6.65	21.02	3	0	1.267	0.884481	0.983234	0	79.3
4.92	26.34	3	0	1.198	0.875825	0.983083	0	103.3
8.88	81.37	3	0	3.333	0.000234	0.004863	0	37.1
10.01	9.69	2	0	1.497	0.475371	0.898384	0	73.3
9.52	53.43	2	0	1.237	0.950094	0.991422	0	113.1
7.61	26.43	1	0	1.569	0.353494	0.83025	0	77.9
6.18	31.87	2	0	1.153	0.808366	0.970116	0	89.1
6.67	28.41	3	0	1.144	0.73458	0.962304	0	92.9
5.78	10.5	3	0	1.145	0.790503	0.97006	0	99.9
6.87	53.71	2	0	0.821	0.092335	0.468508	0	104.3
5.06	41.67	3	0	0.884	0.147279	0.598888	0	96.4
7.24	28.73	2	0	1.234	0.981437	0.997735	0	93.3
8.97	80.69	2	0	0.865	0.109351	0.515033	0	96.8

9.51	126.92	2	0	0.542	0.000337	0.00682	0	104.1
7.23	41.4	2	0	1.465	0.514857	0.90897	0	73.8
8.29	22.75	2	0	0.86	0.167376	0.636322	0	131.2
7.36	24.37	3	2	1.072	0.500076	0.906872	0	94.5
6.87	24.96	3	0	0.726	0.099254	0.490005	0	115.9
6.49	33.97	2	0	1.154	0.760438	0.966419	0	84.8
9.88	131.38	3	0	1.576	0.324044	0.805731	0	78.9
5.43	22.57	3	2	1.778	0.113248	0.525991	0	53.8
8.12	56.3	3	0	0.947	0.413032	0.864761	0	104.1
7.15	18.49	3	1	1.37	0.725889	0.96164	0	78.2
5.73	10.02	3	0	1.889	0.147081	0.598835	0	57.7
6.79	8.65	3	0	1.151	0.734818	0.962304	0	83.3
10.42	42.93	3	0	1.032	0.439487	0.886779	0	89.1
8.88	38.34	2	1	0.864	0.120669	0.539718	0	102.4
8.19	9.25	3	1	2.082	0.045672	0.303974	0	64.5
8.7	19.1	3	0	1.178	0.782649	0.97006	0	117.7
8.65	17.85	3	0	1.089	0.621559	0.932683	0	95.3
6.87	42.82	2	0	1.224	0.979549	0.997735	0	95.4
8.82	17.71	3	0	0.99	0.433942	0.882822	0	94.7
8.62	32.04	3	0	1.194	0.931625	0.990812	0	100.7
5.9	9.16	3	0	1.355	0.701195	0.958482	0	75.6
6.54	24.42	3	0	1.134	0.725609	0.96164	0	80.5
6.87	33.77	3	0	1.244	0.927303	0.99077	0	86.7
6.73	5.05	2	0	1.208	0.971828	0.997505	0	85
9.11	21.84	3	0	1.552	0.315442	0.800259	0	65.7
8.21	7.74	2	0	1.671	0.303564	0.79167	0	76.3
9.55	64.96	2	0	1.131	0.653876	0.943563	0	82.9
6.84	16.29	3	0	1.208	0.941354	0.991397	0	87.4
6.7	63.12	3	0	1.265	0.976753	0.997505	0	83
7.88	9.52	1	0	1.297	0.788627	0.97006	0	76.3
8.68	18.89	3	0	1.243	0.972111	0.997505	0	90.7
7.12	37.07	3	0	1.437	0.516627	0.90897	0	76.1
5.31	18.61	3	0	1.12	0.711023	0.959398	0	114.9
7.03	19.79	3	0	1.783	0.096002	0.479403	0	66.8
6.96	16.44	2	0	1.238	0.963551	0.995498	0	85.1
10.17	22.41	3	0	0.735	0.027699	0.22439	0	133.4
8.16	29.17	3	0	1.744	0.125754	0.550237	0	76.2
9.89	24.4	3	0	1.037	0.472688	0.898019	0	67.4
7.09	38.64	2	0	1.365	0.734492	0.962304	0	84.6
6.83	49.05	3	0	1.073	0.53816	0.90897	0	97.9
7.83	10.65	2	0	1.08	0.630284	0.933268	0	57.3
9.76	48.62	2	0	0.854	0.118624	0.535654	0	110.7
8.13	30.57	2	0	1.073	0.625474	0.932683	0	105.5
5.35	9.79	3	0	1.076	0.581859	0.920669	0	100
9.74	40.36	2	0	0.834	0.097024	0.482778	0	101.3

6.89	18.54	3	0	0.705	0.037562	0.27332	0	119.6
8.4	28.9	2	0	1.258	0.932857	0.991048	0	83.7
8.69	59.01	2	0	1.283	0.825574	0.974684	0	75.7
7.15	16.55	3	0	1.029	0.503316	0.90794	0	94
10.58	13.05	3	0	0.807	0.077958	0.42412	0	93.7
8.19	17.3	3	0	1.33	0.730298	0.962129	0	78.1
7.8	136.98	3	0	1.66	0.204634	0.683145	0	75.2
6.79	21.94	2	0	1.623	0.296852	0.785775	0	63.6
5.57	16.53	3	0	1.321	0.715889	0.960634	0	79.7
8.94	25.1	2	0	1.118	0.759941	0.966419	0	104.2
5.1	44.38	3	0	1.317	0.740393	0.964272	0	85.6
7.65	29.07	3	0	0.874	0.122688	0.542733	0	106.7
6.74	56.91	3	0	1.544	0.406632	0.862461	0	84.1
8.57	27.77	3	0	1.091	0.628601	0.932683	0	105.8
6.79	44.86	1	0	1.692	0.214917	0.695703	0	73.3
7.47	25.79	3	0	1.351	0.643496	0.939787	0	68
7.33	66.84	3	0	1.968	0.15726	0.61786	0	63.2
7.83	11.85	2	0	1.981	0.039562	0.28165	0	69.4
6.62	11.15	3	0	1.599	0.253255	0.734875	0	73.9
9.01	60.24	3	0	1.071	0.495503	0.906872	0	128.7
6.58	36.7	2	0	0.688	0.012116	0.128238	0	121
6.71	7.35	4	0	0.999	0.454068	0.888233	0	107.9
9.41	34.4	3	0	0.902	0.176873	0.64543	0	118.4
5.39	15.88	3	0	1.331	0.705448	0.958927	0	78.8
6.2	14.37	2	0	1.562	0.359844	0.836424	0	43.9
8.28	16.35	2	0	1.406	0.59972	0.925915	0	88.5
9.91	66	2	0	1.099	0.623772	0.932683	0	95
5.35	51.78	2	0	0.487	0.000821	0.015305	0	133.5
8.57	34.26	2	0	1.553	0.335581	0.81415	0	46.6
9.77	42.41	2	0	1.178	0.884818	0.983234	0	95.2
4.88	14.13	2	0	1.19	0.92882	0.99077	0	66.7
9.17	24.66	2	0	1.257	0.888019	0.983918	0	72.9
8.51	8.08	3	0	1.189	0.929639	0.99077	0	68.6
9.7	51.96	3	0	0.871	0.142942	0.593057	0	106.6
5.57	45.39	3	0	1.461	0.484466	0.901944	0	86.6
6.42	66.78	2	0	1.389	0.612028	0.929068	0	89.1
9.5	6.03	2	0	1.394	0.590225	0.924028	0	79.5
6.18	16.96	2	0	1.527	0.419164	0.867334	0	79.1
7.55	41.52	3	0	0.995	0.358189	0.834582	0	95.9
9.44	19.41	2	0	1.308	0.82137	0.974168	0	101.2
6.74	27.51	3	0	1.492	0.467032	0.89585	0	88.7
9.58	9.1	2	0	1.085	0.593049	0.924683	0	103.1
6.11	14.38	3	0	1.493	0.416458	0.86672	0	72.5
8.73	35.84	2	0	1.419	0.542494	0.90897	0	90.8
7.81	17.02	3	0	1.134	0.705685	0.958927	0	87.6

7.93	6.78	2	0	0.993	0.459216	0.891873	0	99
8.84	19.18	3	0	1.262	0.89673	0.986881	0	98.5
8.84	12.67	3	0	1.069	0.540887	0.90897	0	96.2
8.57	50.02	2	0	1.06	0.607632	0.927253	0	91.6
5.66	9.57	2	0	1.162	0.802223	0.97006	0	88.2
7.18	35.86	4	0	1.145	0.720735	0.96164	0	87.8
8.28	24.34	3	0	1.523	0.334117	0.812319	0	76.6
5.3	20.02	3	0	1.77	0.1035	0.500065	0	48.8
8.48	56.12	2	0	0.682	0.024386	0.208842	0	112.5
6.68	25.82	3	0	0.898	0.276859	0.762666	0	105.4
5.59	26.31	3	0	1.421	0.557413	0.910977	0	86.7
7.78	36.27	1	0	1.01	0.499362	0.906872	0	99.5
8.1	35.01	3	0	1.238	0.973671	0.997505	0	88.9
5.08	17.7	2	0	1.267	0.866248	0.981617	0	63.9
5.57	12.16	3	0	1.02	0.521793	0.90897	0	72.9
9.06	21.85	2	0	0.609	0.002288	0.035863	0	142.2
9.11	145.31	2	0	1.409	0.634234	0.936144	0	80.5
7.03	13.24	3	0	1.416	0.646581	0.940359	0	71.7
6.87	44.1	2	0	1.836	0.178774	0.646718	0	78.1
8.21	58.48	3	0	1.662	0.177756	0.645897	0	71.8
5.12	29	2	0	1.163	0.848087	0.979322	0	95
5.41	24.14	2	0	1.051	0.572893	0.918743	0	94.7
5.72	15.96	3	0	1.089	0.623739	0.932683	0	95.9
9.55	32.02	3	0	1.112	0.676222	0.951158	0	96.6
9.99	30.37	2	0	1.729	0.125603	0.550237	0	70.1
6.55	9.33	3	0	1.121	0.679595	0.951158	0	89.4
9.03	24.82	2	0	0.616	0.004087	0.058012	0	112.2
5.95	39.25	3	0	2.988	0.000426	0.008564	0	57.5
6.6	20.83	3	0	1.812	0.090053	0.462725	0	60
8.84	11.29	2	0	1.627	0.230084	0.708889	0	67.2
5.59	52.04	2	0	1.306	0.806585	0.97006	0	86.6
5.72	37.72	1	0	0.936	0.347184	0.824816	0	103.3
7.15	4.75	1	0	1.312	0.829881	0.975858	0	93.5
7.9	13.84	2	0	1.178	0.883071	0.9831	0	90.5
5.11	9.83	3	0	1.242	0.953505	0.991422	0	41.4
10.07	7.99	3	0	1.089	0.619573	0.932207	0	98
8.44	54.73	3	0	0.985	0.329859	0.806641	0	96.5
6.96	41.05	2	0	1.293	0.787801	0.97006	0	88.2
8.57	14.98	2	0	3.173	2.14E-05	0.000542	0	34.9
5.71	19.95	3	0	1.194	0.929419	0.99077	0	77.7
8.38	23.4	2	0	0.999	0.384099	0.854992	0	100.9
5.44	36.97	3	0	1.113	0.670932	0.95015	0	89
4.45	20.86	1	0	0.01	1E-17	3.97E-16	0	200
9.14	17.81	1	0	1.958	0.095207	0.477821	0	64.2
8.44	24.09	2	0	1.276	0.880896	0.983083	0	97.8

6.8	7.67	2	0	6.001	1.93E-08	6.58E-07	0	28.6
7.5	38.82	2	0	0.835	0.110785	0.518851	0	97.7
8.76	69	1	0	1.116	0.720612	0.96164	0	91.5
6.65	19.15	3	0	0.807	0.148725	0.600981	0	128.2
7.49	26.56	3	0	1.11	0.647167	0.940458	0	110.3
5.9	12.91	3	0	1.225	0.973059	0.997505	0	102.9
8.09	30.05	4	0	2	0.045258	0.303872	0	60.9
9.13	5.99	3	0	1.156	0.82931	0.975809	0	102.3
7.97	12.18	3	1	1.356	0.714434	0.960203	0	82.9
8.09	14.28	2	0	1.059	0.615829	0.930023	0	85.2
7.17	28.04	3	0	1.055	0.503111	0.90794	0	104.3
5.34	14.94	3	0	1.386	0.601733	0.925915	0	80.4
6.25	15.79	3	0	1.533	0.35114	0.829363	0	71.6
9.85	52.68	2	0	2.084	0.030276	0.238214	0	52.9
9.82	19.95	2	0	0.808	0.071212	0.400039	0	110
9.33	28.31	2	0	1.621	0.282606	0.76495	0	77.7
7.9	18.27	2	0	0.718	0.040765	0.287531	0	133.7
4.73	15.54	2	0	1.33	0.76568	0.967498	0	78.1
10.73	30.5	2	0	0.817	0.131418	0.564264	0	110.9
11.52	37.23	2	0	1.12	0.687785	0.952468	0	99.5
6.4	16.96	1	0	1.674	0.224334	0.701603	0	76.3
6.55	23.4	3	0	1.453	0.474019	0.898019	0	88.7
7.21	17.95	3	0	0.955	0.297917	0.787642	0	93.2
4.79	58.29	1	0	0.15	1E-17	3.97E-16	0	175.2
8.16	24.14	3	0	1.063	0.477242	0.898527	0	99.9
6.79	41.5	2	0	1.107	0.709776	0.959398	0	87
5.47	66	2	0	1.302	0.824184	0.974465	0	83.7
6.76	9.17	2	0	1.042	0.592127	0.924683	0	101.4
8.24	16.28	3	0	1.175	0.84052	0.977862	0	97.8
6.86	16.7	3	0	1.729	0.139593	0.584296	0	39.1
7.72	10.71	3	0	1.623	0.296071	0.785454	0	39.6
5.96	37.27	3	1	1.285	0.80756	0.97006	0	86.5
5.4	22.63	3	0	1.089	0.590728	0.924336	0	108.8
8.88	32.26	2	0	3.449	3.82E-06	0.000108	0	43.5
8.09	13.53	1	0	0.01	1E-17	3.97E-16	0	200
7.17	15.32	2	0	0.891	0.240581	0.72094	0	104
8.7	28.1	2	0	0.76	0.036347	0.267276	0	119
6.39	27.57	2	0	1.346	0.654898	0.943956	0	17.6
8.57	10.16	2	0	2.29	0.023446	0.205542	0	56.9
6.24	15.81	2	0	1.377	0.585624	0.922744	0	74.8
6.54	23.08	3	0	0.914	0.212379	0.692653	0	76.7
7.91	48.08	2	0	1.107	0.705453	0.958927	0	89
6.29	13.66	3	0	1.006	0.531688	0.90897	0	99.7
6.09	74.07	2	0	2.845	0.00124	0.021657	0	46.1
8.82	4.3	3	0	0.936	0.276246	0.761477	0	109

8.13	10.98	2	0	2.003	0.041336	0.290284	0	61.7
7.17	34.7	3	0	1.381	0.578211	0.919803	0	77.8
5.08	10.95	2	0	1.511	0.492541	0.906569	0	77.7
7.77	52.7	3	0	0.788	0.055008	0.342708	0	107.1
6.38	54.13	3	0	1.515	0.445835	0.888233	0	56.6
5.3	842.03	1	0	0.73	0.051913	0.330736	0	111.9
8.95	25.23	2	0	1.152	0.831083	0.975858	0	91.1
6.99	40.27	2	0	1.313	0.747069	0.964844	0	86.8
8.87	53.6	2	0	0.956	0.291964	0.778384	0	98.2
5.54	17.8	2	0	1.381	0.596585	0.925915	0	77.2
4.86	58.18	2	0	1.994	0.074529	0.412636	0	53.9
9.31	12.25	3	0	1.174	0.833998	0.976224	0	98.6
5.31	17.38	2	0	1.081	0.609693	0.927586	0	103.5
8.06	9.22	3	1	1.084	0.554279	0.910977	0	94
8.22	9.04	3	0	1.233	0.997032	0.99992	0	70.5
9.86	6.41	3	0	1.102	0.712145	0.959974	0	75.2
7.01	64.38	2	0	1.366	0.613794	0.929068	0	79.7
6.64	40.08	2	0	1.199	0.941099	0.991397	0	92.4
5.16	17.45	3	0	0.905	0.191997	0.66659	0	124.1
9.66	8.48	2	0	1.542	0.452545	0.888233	0	70.7
6.44	18.1	3	0	1.182	0.816969	0.972536	0	72.8
9.38	30.68	2	0	1.3	0.777228	0.97006	0	79.5
6.6	37.43	2	0	0.962	0.29882	0.788463	0	97.1
6.13	10.45	2	0	0.929	0.336642	0.814964	0	97.7
6.81	15.01	2	0	0.01	1E-17	3.97E-16	0	200
8.82	66.26	3	0	0.01	1E-17	3.97E-16	0	200
9.52	30.52	2	0	1.199	0.910335	0.98846	0	85
9.32	17.07	3	0	1.39	0.637787	0.937605	0	89.5
6.65	13.08	3	0	0.747	0.031419	0.244008	0	123.3
5.8	15.38	3	0	1.526	0.316577	0.800707	0	81.7
6.16	43.35	3	0	0.843	0.09052	0.46399	0	114.7
8.32	35.87	3	0	1.391	0.609326	0.927253	0	95.7
6.38	14.27	3	0	0.983	0.438127	0.885468	0	107.2
6.67	14.86	2	0	1.579	0.273126	0.75652	0	79.1
6.46	23.58	3	0	0.924	0.234564	0.711914	0	107.6
6.04	18.23	3	0	1.263	0.981698	0.997735	0	89.7
6.27	14.49	3	0	1.217	0.99084	0.999059	0	109
4.84	25.65	2	0	1.891	0.051119	0.328135	0	69.7
5.81	4.36	3	0	1.712	0.13617	0.577188	0	82.9
5.92	30.05	2	0	2.654	0.000755	0.014267	0	59.3
5.5	12.46	2	0	1.266	0.886704	0.983874	0	54.1
9.29	39.76	3	0	1.027	0.417973	0.867334	0	94.9
8.75	21.48	1	0	2.057	0.041816	0.291972	0	64.5
7.25	3.96	3	0	0.863	0.21838	0.69794	0	59.3
7.85	25.35	3	0	0.977	0.335271	0.814026	0	107.4

8.91	47.01	1	0	0.888	0.223962	0.701603	0	117.2
6.37	35.66	2	0	1.055	0.527755	0.90897	0	92.7
5.77	10.55	3	0	1.856	0.12035	0.539718	0	120.9
5.45	19.72	2	0	1.061	0.602907	0.925915	0	87.2
8.78	5.12	2	0	2.318	0.037908	0.274738	0	45.6
5.83	17.13	2	0	1.332	0.810965	0.971	0	84
6.37	12.12	3	0	0.879	0.196552	0.673222	0	109.7
5.36	24.95	2	0	1.038	0.512551	0.90897	0	102
8.92	8.39	2	0	1.476	0.53535	0.90897	0	81.2
7.61	45.04	1	0	1.089	0.560086	0.911314	0	100.1
6.86	12.15	3	0	1.177	0.890431	0.985116	0	94.6
7.74	19.03	1	0	2.866	0.00147	0.025043	0	55.3
6.27	42.03	2	0	1.074	0.558303	0.910977	0	86.8
8.81	11.96	2	0	1.377	0.658752	0.945081	0	79.8
8.09	29.74	3	0	1.548	0.326683	0.806293	0	65.7
8.31	49.96	2	0	1.676	0.24717	0.726506	0	80.8
6.71	30.67	2	0	1.41	0.567314	0.913335	0	74.5
3.81	53.81	2	0	1.81	0.119848	0.538493	0	63.3
8.41	13.04	2	0	0.817	0.159519	0.622135	0	114.5
7.39	38.57	2	0	0.997	0.380207	0.851107	0	105.5
5.83	19.45	2	0	1.479	0.394042	0.861859	0	79.2
5.91	14.82	2	0	1.16	0.848266	0.979336	0	102.8
6.06	13.94	1	0	1.128	0.765261	0.967498	0	85.4
5.92	28.52	2	0	1.605	0.324049	0.805731	0	80.1
7.81	18.86	3	0	1.004	0.377979	0.850346	0	96.5
5.9	15.57	3	0	1.093	0.675076	0.951158	0	86.8
8.32	79.12	2	0	0.682	0.069715	0.394625	0	106.8
8.03	17.77	2	0	0.829	0.167648	0.636322	0	129.8
7.01	49.92	2	0	1.339	0.759128	0.966419	0	84.9
9.09	18.84	3	0	0.813	0.07576	0.415394	0	100.1
5.34	7.95	2	0	1.101	0.691868	0.952547	0	98.2
6.13	34.2	1	0	1.071	0.625391	0.932683	0	96.9
6.96	14.36	3	0	0.688	0.010967	0.119739	0	137.6
7.75	11.8	3	0	1.517	0.458254	0.891247	0	83.1
5.35	19.9	3	0	1.517	0.432095	0.880257	0	64.8
7.15	7.01	3	0	1.085	0.647105	0.940458	0	86.2
9.85	29.65	2	0	1.139	0.789103	0.97006	0	71.4
6.25	27.38	2	0	1.588	0.310878	0.798212	0	76.1
5.3	29.26	1	0	1.524	0.405696	0.862461	0	88.3
6.11	23.18	1	0	2.529	0.007167	0.087008	0	50
6.14	10.55	2	0	1.319	0.794223	0.97006	0	84.6
7.21	36.47	3	0	0.927	0.240172	0.720574	0	96
9.61	12.03	2	0	1.821	0.163277	0.629076	0	43.6
9.19	8.46	3	0	1.768	0.223739	0.701603	0	88.7
9.38	50.34	2	0	1.22	0.981261	0.997735	0	82.9

8.43	83.83	1	0	0.853	0.1026	0.499086	0	113.3
6.57	30.37	2	0	1.13	0.739483	0.96414	0	63.5
6.34	16.59	2	0	2.086	0.059041	0.356311	0	62.3
5.35	24.63	1	0	1.043	0.542039	0.90897	0	100.5
8.19	22.08	3	0	0.601	0.001775	0.029286	0	149.4
7.83	13.43	3	0	0.884	0.158268	0.620057	0	106.6
9.44	51.38	2	0	0.916	0.203603	0.682501	0	96.9
7.05	20.58	3	0	1.467	0.418255	0.867334	0	81.7
8.32	30.59	2	0	0.848	0.146029	0.598768	0	136.5
6.21	10.81	3	0	1.419	0.514242	0.90897	0	87.8
9.09	10.67	2	0	1.889	0.150173	0.604664	0	71.4
6.46	9.03	1	0	1.706	0.305319	0.792672	0	68.2
5.12	4.91	3	0	1.447	0.542518	0.90897	0	24.8
6.51	38.31	2	0	1.661	0.234911	0.711914	0	75.4
6.47	2.59	2	0	1.323	0.811262	0.971	0	62.6
8.59	32.15	2	0	1.002	0.389969	0.859539	0	106.9
5.3	13.05	1	0	2.636	0.000664	0.012735	0	64.4
6.47	22.94	3	0	1.135	0.777569	0.97006	0	91.2
8.88	51.63	2	0	1.231	0.988847	0.998053	0	106.4
6.89	22	1	0	0.98	0.441632	0.888132	0	103.5
8.29	79.14	2	0	1.458	0.624975	0.932683	0	81.5
6.93	11.28	3	0	1.616	0.223635	0.701603	0	66.4
6.79	14.7	2	0	1.419	0.5801	0.920669	0	73.4
6.84	33.56	2	0	1.179	0.920966	0.989178	0	85.9
5.16	35.25	3	0	1.07	0.561223	0.911753	0	98.1
7.71	11.52	2	0	0.01	1E-17	3.97E-16	0	200
7.99	24.82	1	0	0.9	0.170328	0.637412	0	105.8
5.81	13.25	2	0	1.219	0.946976	0.991422	0	90.9
9.94	21.93	3	0	1.193	0.92335	0.989893	0	91.3
6.87	2.06	2	0	1.275	0.904364	0.987276	0	95.8
8.35	18.81	3	2	1.216	0.981591	0.997735	0	93.9
6.74	20.06	2	0	0.885	0.168671	0.636322	0	91.5
8.54	75.51	2	0	1.926	0.135116	0.573491	0	62.9
6.61	36.7	3	0	1.099	0.673431	0.951142	0	94.2
9.16	18.21	2	0	1.525	0.318914	0.802274	0	66.6
8.76	48.53	1	0	5.889	5E-09	1.74E-07	0	29
6.27	16.62	3	0	1.618	0.296983	0.785775	0	52
8.97	8.16	3	0	1.968	0.084224	0.443601	0	66.6
5.06	15.11	2	0	1.688	0.158127	0.619949	0	47.4
8	40.05	1	0	2.652	0.000723	0.013713	0	51.7
8.09	24.42	2	0	0.876	0.216037	0.697303	0	106.4
5.12	23.74	2	0	0.851	0.15845	0.620057	0	123.4
8.75	54.19	3	0	1.498	0.466947	0.89585	0	80.5
8.62	20.11	2	0	0.894	0.156282	0.616556	0	111.1
6.3	5.04	2	0	1.978	0.150449	0.604728	0	72.7

5.08	6.98	2	0	1.8	0.177777	0.645897	0	61.9
5.49	18.74	3	0	2.2	0.033864	0.255582	0	62.5
9.89	8.81	2	0	1.906	0.094319	0.475332	0	57
7.43	15.87	2	0	0.661	0.010637	0.116743	0	118.8
8.35	29.28	2	0	0.939	0.252888	0.734875	0	107
5.95	4.51	3	0	1.271	0.91176	0.988983	0	86.6
5.81	22.45	1	0	1.233	0.998655	0.99992	0	90.8
5.55	10.73	3	0	0.976	0.410466	0.863065	0	103.3
6.71	18.52	2	0	1.815	0.144358	0.596041	0	65.6
9.67	21.32	1	0	1.784	0.184514	0.656728	0	71.3
7.85	8.1	2	0	2.226	0.062832	0.370139	0	54
7.28	35.55	2	0	1.251	0.930245	0.99077	0	88.4
5.5	58.73	1	0	1.154	0.774325	0.97006	0	92.9
7.11	58.08	1	0	1.192	0.911631	0.988929	0	93.7
6.02	20.32	2	0	1.628	0.239381	0.718542	0	70.8
5.67	9.26	2	0	0.942	0.364616	0.841928	0	110.8
4.79	3.5	1	0	0.957	0.378474	0.850346	0	94.5
7.85	51.49	2	0	1.109	0.620504	0.932207	0	100.5
5.17	34.78	2	1	1.107	0.656793	0.943998	0	97.7
7.23	24.24	2	0	0.891	0.183896	0.655884	0	108.2
6.55	30.71	2	0	0.724	0.022613	0.19963	0	113.3
6.54	13.32	2	0	0.01	1E-17	3.97E-16	0	200
8.5	19.24	2	0	1.289	0.80924	0.970418	0	89.1
6.13	52.95	2	0	0.996	0.379999	0.851107	0	107.3
8.66	20.04	2	0	1.227	0.995317	0.99978	0	90.3
7.01	13.94	2	0	1.315	0.73723	0.963849	0	81.5
8.38	45.7	2	1	0.82	0.082215	0.440242	0	106.6
8.13	27.78	2	0	1.292	0.833717	0.976224	0	83.5
9.11	23.45	2	0	1.841	0.108885	0.514159	0	84.5
7.37	22.39	2	0	0.912	0.268781	0.752656	0	105.9
6.38	17.14	2	0	1.101	0.699181	0.957318	0	96.1
4.69	42.15	2	0	1.271	0.962262	0.995026	0	72.2
9.45	14.99	2	0	0.9	0.231604	0.711304	0	107.4
5.39	82.39	2	0	1.5	0.447367	0.888233	0	46.8
7.97	51.57	1	0	1.148	0.76958	0.968967	0	89.8
9.39	16.99	2	0	1.016	0.505599	0.90897	0	91.8
7.87	19.92	1	0	1.156	0.83208	0.976224	0	96.3
7.06	15.68	3	0	1.416	0.536946	0.90897	0	59.9
5.26	42.26	2	0	1.002	0.388492	0.859423	0	146.3
7.97	28.72	2	0	1.214	0.983643	0.997735	0	92.7
6.96	3	2	0	0.01	1E-17	3.97E-16	0	200
8.21	31.19	2	0	1.282	0.836826	0.976711	0	87.2
6.3	79.38	2	0	0.538	0.000826	0.01531	0	112.7
7.39	27.57	2	0	1.256	0.905583	0.98773	0	133.5
7.61	22.42	2	0	0.874	0.145141	0.596922	0	122.4

5.73	24.32	2	0	1.266	0.863362	0.981617	0	86
4.54	15.81	3	0	1.345	0.682212	0.951842	0	92.4
9.7	7.72	1	0	1.808	0.156994	0.61786	0	71.2
7.68	18.3	1	0	1.283	0.83416	0.976224	0	84.9
7.27	50.47	2	0	1.015	0.365698	0.842753	0	104.9
8.53	52.72	2	0	0.953	0.281971	0.76495	0	98.4
8.6	41.21	2	0	1.287	0.843649	0.97823	0	88.9
6.18	15.29	2	0	1.108	0.72359	0.96164	0	84.5
8.82	9.92	3	0	1.034	0.472691	0.898019	0	106.2
7.78	4.54	2	0	2.023	0.073707	0.409236	0	64.3
4.22	14.31	2	0	1.034	0.442532	0.888233	0	91.9
7.74	18.34	2	0	1.117	0.6782	0.951158	0	96.4
11.97	21.24	1	0	4.574	2.6E-08	8.74E-07	0	41.4
6.44	17.97	2	0	0.969	0.301935	0.791593	0	107.7
5.78	12.11	2	0	0.927	0.242736	0.723954	0	106.6
7.27	13.78	2	0	1.23	0.975948	0.997505	0	100.5
8.18	6.54	2	0	0.518	0.006521	0.080726	0	131.8
6.04	24.18	2	0	1.119	0.742977	0.964372	0	85.8
4.58	11.87	2	0	1.2	0.952697	0.991422	0	92.2
9.45	93.41	2	0	1.187	0.840514	0.977862	0	94.1
8.35	11.64	2	0	1.099	0.650263	0.941696	0	76.4
5.12	7.9	2	0	3.701	1.34E-06	4.04E-05	0	31.3
6.21	21.49	2	0	0.981	0.297959	0.787642	0	101.4
5.31	25.19	1	0	1.17	0.859174	0.981617	0	96.2
6.92	17.11	3	0	0.955	0.295636	0.785346	0	87.6
7.77	79.35	2	0	5.4	5.78E-09	2.01E-07	0	16.8
6.95	23.24	3	0	1.218	0.951633	0.991422	0	96.3
7.87	43.19	2	0	0.997	0.362483	0.840171	0	90.4
8.76	18.69	3	0	1.592	0.24888	0.729277	0	110
6.62	13.36	3	0	1.475	0.443009	0.888233	0	85.7
8.38	5.82	2	0	0.917	0.252226	0.734489	0	105.6
8.19	17.08	2	0	1.861	0.073648	0.409161	0	79.4
6.35	45.98	3	0	1.18	0.844465	0.97823	0	97.9
7.17	22.99	2	0	1.295	0.796317	0.97006	0	81.5
9.28	17.6	2	0	0.911	0.266037	0.749344	0	103.7
7.44	16.33	2	0	0.935	0.334279	0.812319	0	64.7
5.12	22.93	2	0	0.878	0.176341	0.64543	0	105.8
8.15	75.55	2	0	1.484	0.512158	0.90897	0	84.5
5.73	3.79	2	0	0.981	0.432333	0.880544	0	83.1
9.64	18.55	2	0	2.059	0.036213	0.266599	0	52.4
6.55	11.16	3	0	1.253	0.927429	0.99077	0	111.6
9.72	63.57	2	0	0.76	0.045091	0.303872	0	115
9.91	57.9	1	0	1.866	0.137522	0.578504	0	69.5
7.15	12.46	3	0	1.364	0.692656	0.952781	0	51.3
5.63	38.63	2	0	1.231	0.97493	0.997505	0	91.9

11.91	20.56	2	0	0.831	0.101767	0.496954	0	107.7
5.86	18.51	2	0	0.877	0.139134	0.582887	0	88.1
7.85	4.72	2	0	1.356	0.650236	0.941696	0	69.8
8.4	89.88	2	0	1.03	0.43765	0.885072	0	101
8.31	21.04	2	0	1.702	0.225205	0.701603	0	59.1
6.76	8.78	2	0	1.09	0.699676	0.957457	0	89.5
6.52	58.52	1	0	1.081	0.556467	0.910977	0	92.4
7.5	47.15	1	0	1.19	0.928872	0.99077	0	92.6
6.11	6.31	2	0	1.254	0.944883	0.991397	0	88.9
8.98	8.93	3	0	1.211	0.98391	0.997735	0	71.3
5.83	40.29	1	0	1.703	0.182305	0.652641	0	85.1
5.64	26.16	2	0	1.85	0.131038	0.562824	0	89.7
10.27	11.62	2	0	1.223	0.990967	0.999059	0	66.9
5.26	22.91	2	0	1.347	0.727455	0.962129	0	93.8
7.24	6.14	2	0	1.378	0.617128	0.930979	0	34.4
9.77	22.81	3	0	1.695	0.205974	0.684532	0	86.8
4.86	15.01	2	0	2.058	0.042818	0.296459	0	54
7.87	13.8	2	0	1.119	0.716649	0.960634	0	118.6
6.92	16.87	2	0	1.366	0.609956	0.927831	0	81.6
7.23	18.07	2	0	4.882	1.26E-07	4.15E-06	0	26.1
7.09	72.53	2	0	1.445	0.588779	0.922984	0	85
8.24	7.7	2	0	1.276	0.871765	0.982864	0	75.2
5.53	18.48	2	0	1.407	0.52221	0.90897	0	131.5
5.16	39.29	2	0	1.26	0.926026	0.990378	0	89.9
6.62	16.82	2	0	1.826	0.121791	0.54235	0	52.5
12.15	349.87	2	0	1.294	0.792154	0.97006	0	83.9
7.8	11.83	2	0	1.243	0.936354	0.99128	0	75.3
9.66	4.92	1	0	1.104	0.722281	0.96164	0	100
8.21	119.42	2	0	0.769	0.081786	0.438691	0	114.4
6.81	23.27	2	0	1.249	0.911535	0.98891	0	78.5
8.51	12.48	2	0	1.499	0.407813	0.862461	0	79.7
5.59	25.35	2	0	1.729	0.177249	0.645657	0	72.4
9.95	12.23	2	0	1.511	0.375532	0.849828	0	70.9
8	13.39	3	0	1.082	0.671163	0.95015	0	122.4
6.99	22.49	1	0	1.169	0.864861	0.981617	0	89.2
8.5	20.25	2	0	1.082	0.566078	0.913241	0	102.1
5.44	8.3	1	0	0.936	0.336805	0.814964	0	106
9.57	31.53	1	0	1.263	0.89427	0.985818	0	91.6
8.47	44.96	1	0	1.392	0.630884	0.933676	0	81.9
6.95	9.92	2	0	0.986	0.473858	0.898019	0	68.8
6.92	22.7	2	0	1.066	0.551668	0.910336	0	95.2
8.37	5.09	2	0	1.199	0.95109	0.991422	0	77.7
8.97	19.93	2	0	2.817	0.000506	0.010028	0	43
7.02	8.53	2	0	0.698	0.021756	0.194248	0	98
7.61	52.94	2	0	1.074	0.544306	0.90897	0	102.2

6.27	19.17	2	0	1.026	0.465654	0.89585	0	93.9
5.86	20.14	2	0	1.758	0.285533	0.767743	0	69
6.73	13.57	2	0	1.148	0.819767	0.973778	0	108.2
8.41	13.13	2	0	0.953	0.384743	0.854992	0	78.3
6.2	21.81	2	0	0.855	0.254984	0.736744	0	136.4
6.38	8.89	2	0	0.788	0.167186	0.636322	0	136.4
7.84	5.41	2	0	0.991	0.495801	0.906872	0	97.2
5.9	25.85	2	0	1.133	0.753485	0.96597	0	111.7
7.9	39.5	2	0	1.162	0.878238	0.983083	0	95.6
10.59	15.19	2	0	1.11	0.689132	0.952468	0	97.8
8.92	6.05	2	0	0.912	0.203998	0.682501	0	100.7
9.13	84.31	2	0	0.817	0.104385	0.500992	0	102.7
6.84	30	2	0	1.301	0.794221	0.97006	0	74.4
6.52	7.76	2	0	0.948	0.340486	0.81848	0	101.4
6.32	49.32	2	0	1.576	0.316963	0.800933	0	77.9
9.82	12.41	2	0	1.178	0.900621	0.987276	0	93.4
6.55	19.36	1	0	1.766	0.111594	0.52167	0	77.7
6.7	16.34	2	0	1.716	0.214276	0.694929	0	55.6
8.21	25.91	2	0	1.184	0.897652	0.987004	0	95.6
7.27	35.4	1	0	1.127	0.638675	0.937605	0	94
6.99	9.81	2	0	0.991	0.459495	0.891873	0	104.4
4.75	12.37	1	0	1.252	0.918201	0.98911	0	88.8
6.28	41.6	2	0	0.244	1.13E-12	4.27E-11	0	160.8
6.42	13.59	2	0	1.447	0.530881	0.90897	0	88.8
6.89	6.07	2	0	0.999	0.482612	0.90163	0	157
7.69	12.72	2	0	0.886	0.303171	0.79167	0	98
9.2	9.05	2	0	0.98	0.342634	0.820822	0	90.4
5.26	6.12	1	0	0.969	0.450644	0.888233	0	101.6
5.48	10.04	2	0	2.01	0.067784	0.388057	0	70.7
5.44	27.72	2	0	0.902	0.258591	0.740536	0	199.6
7.96	13.61	2	0	1.262	0.914459	0.98911	0	81.3
8.37	12.63	2	0	1.214	0.988819	0.998053	0	96.7
7.06	7.86	2	0	1.073	0.617622	0.931228	0	103.7
10.73	132.69	2	0	0.953	0.358963	0.835303	0	99.4
6.54	27.73	2	0	1.092	0.560841	0.911611	0	95.7
10.9	148.86	2	0	1.554	0.332526	0.809391	0	79.8
6.25	65.01	1	0	0.679	0.014564	0.146355	0	119.6
6.29	17.06	1	0	2.382	0.014538	0.146355	0	59.6
8.03	8.43	1	0	0.01	1E-17	3.97E-16	0	200
9.5	7.79	2	0	2.137	0.088999	0.458995	0	67.8
4.3	10.97	2	0	0.782	0.115979	0.531511	0	105.9
9.6	35.68	2	0	0.994	0.504079	0.908772	0	89.7
5.48	33.61	2	0	0.767	0.101563	0.496535	0	113.5
4.41	59.66	1	0	0.71	0.018457	0.172886	0	130
6.61	9.26	2	0	1.34	0.727414	0.962129	0	71.7

7.72	37.51	2	0	1.11	0.665203	0.947803	0	101.6
8.43	44.82	2	0	1.859	0.216522	0.697874	0	61.5
6.92	15.26	2	0	0.98	0.474838	0.898087	0	95.1
5.69	5.95	1	0	0.932	0.341724	0.819889	0	101
7.14	11.8	2	0	1.113	0.754693	0.96597	0	84.8
7.85	20.93	2	0	1.437	0.500638	0.906872	0	80.1
5.74	4.83	2	0	1.648	0.320566	0.802626	0	76
4.01	36.55	1	0	1.314	0.785896	0.97006	0	86.7
7.65	8.17	2	0	1.801	0.08962	0.461254	0	80.6
7.78	29.14	2	0	0.908	0.197131	0.673765	0	87.7
8.22	54.76	1	0	1.029	0.507491	0.90897	0	91.8
7.43	10.78	2	0	1.29	0.819403	0.973778	0	78.3
9.36	7.15	1	0	1.197	0.940472	0.991397	0	88.3
7.28	6.3	2	0	0.966	0.407012	0.862461	0	116.8
5.43	7.49	2	0	1.315	0.834961	0.976576	0	110.4
9.17	28.29	1	0	1.176	0.882233	0.983083	0	91.9
5.19	39.89	2	0	1.223	0.959109	0.993763	0	93.7
9.99	12.56	2	0	1.842	0.107695	0.509851	0	68.9
6.8	42.39	2	0	1.744	0.157325	0.61786	0	112.8
6.61	17.6	2	0	1.209	0.977531	0.997505	0	100.2
5.2	6.03	2	0	1.271	0.930567	0.99077	0	84.9
8.02	297.75	1	0	0.881	0.222218	0.701127	0	102.7
9.54	11.63	2	0	3.009	6.47E-05	0.001543	0	53.6
9.55	17.08	2	0	0.905	0.281879	0.76495	0	104.3
6.76	14.05	2	0	1.023	0.522855	0.90897	0	122.2
9.47	18.27	2	0	1.657	0.23353	0.711914	0	53.1
6.42	8.39	2	0	1.535	0.461321	0.892914	0	75
6.52	15.08	2	0	1.628	0.234299	0.711914	0	28.6
8.07	9.73	2	0	0.883	0.255044	0.736747	0	103
8.54	11.74	1	0	1.977	0.137279	0.578504	0	76.8
9.42	17.86	2	0	1.429	0.49564	0.906872	0	74.5
6.76	13.4	2	0	0.729	0.047369	0.311237	0	116.1
7.64	9.18	2	0	0.885	0.161421	0.625848	0	115.7
6.54	21.45	2	0	0.875	0.213793	0.694929	0	120.5
6.09	7.46	2	0	1.537	0.336732	0.814964	0	74.8
5.08	22.7	1	0	1.127	0.722377	0.96164	0	94
7.4	9.41	2	0	1.525	0.418715	0.867334	0	81.7
6.52	12.72	2	0	1.046	0.53028	0.90897	0	107.9
6.61	5.26	2	0	1.333	0.740455	0.964272	0	84.6
5.16	20.77	2	0	0.927	0.268861	0.752656	0	106.3
8.06	68.19	2	0	1.111	0.681599	0.951658	0	101.1
7.55	18.71	2	0	1.29	0.825679	0.974684	0	89.6
6.7	26.43	2	0	1.194	0.9258	0.99022	0	85
9.92	21.46	2	0	1.266	0.979244	0.997735	0	92.9
5.62	16.73	1	0	1.129	0.766429	0.967858	0	93.9

8.31	26.26	2	0	1.267	0.868667	0.981617	0	94.1
8.43	12.27	2	0	1.322	0.7556	0.96597	0	94.5
8.79	10.93	2	0	1.734	0.207894	0.687824	0	45.2
4.88	50.19	2	0	0.753	0.046604	0.307854	0	116.9
6.43	10.37	2	0	1.425	0.542292	0.90897	0	87.1
5.36	14.45	2	0	1.275	0.874975	0.983083	0	87.8
7.42	34.53	1	0	1.054	0.600137	0.925915	0	92.8
8.27	8.11	2	0	0.87	0.204606	0.683145	0	93.2
9.29	25.72	2	0	1.222	0.984538	0.997735	0	85.5
6.68	13.89	2	0	1.478	0.480085	0.898839	0	69
7.64	2.64	2	0	1.134	0.805726	0.97006	0	92.1
8.07	8.52	2	0	0.704	0.086536	0.450538	0	103.2
5.4	8.54	2	0	1.306	0.776336	0.97006	0	91.7
9.44	34.1	2	0	1.173	0.849852	0.980281	0	97.5
4.64	143.75	2	0	0.918	0.214455	0.694929	0	112
6.33	42.64	1	0	1.272	0.854659	0.981617	0	97.1
7.97	9.91	2	0	1.354	0.633537	0.935444	0	75.3
6.67	9.27	1	0	4.337	4.32E-05	0.001051	0	37.5
9.16	51.54	2	0	1.273	0.861331	0.981617	0	81.2
4.55	21.92	2	0	1.585	0.243728	0.725132	0	64.7
9.79	6.44	2	0	1.037	0.478408	0.898527	0	95.4
9.17	21.1	1	0	0.94	0.396568	0.861859	0	103.9
5.43	14.39	2	0	1.286	0.857664	0.981617	0	74.8
7.18	8.32	2	0	0.789	0.12225	0.542733	0	75.9
9.23	9.67	1	0	1.31	0.805887	0.97006	0	83.5
5.66	21.28	1	0	1.564	0.349038	0.827192	0	80.2
7.65	10.74	2	0	1.192	0.893713	0.985818	0	94.4
5.81	28.91	2	0	2.157	0.031065	0.242303	0	66.7
8.19	24.57	2	0	2.576	0.001196	0.020992	0	58.5
4.87	16.88	2	0	0.873	0.234287	0.711914	0	97.3
6.57	21.21	1	0	1.891	0.125922	0.550237	0	68.6
9.06	35.22	2	0	1.066	0.55293	0.910945	0	98.4
7.43	15.83	1	0	3.612	0.000149	0.003267	0	41.6
7.88	17.89	2	0	2.036	0.025641	0.215624	0	74.5
6.77	24.99	2	0	1.26	0.880448	0.983083	0	91
7.43	10.47	2	0	1.016	0.419976	0.867873	0	83.1
6.05	14.71	2	0	1.076	0.633833	0.935661	0	123
7.77	9.58	2	0	1.528	0.442659	0.888233	0	87.1
8.68	7.7	2	0	0.722	0.020449	0.18504	0	119.5
6.68	17.27	2	0	1.351	0.643176	0.939584	0	92.2
8.15	16.52	1	0	1.042	0.572285	0.918743	0	100.3
5.91	13.24	2	0	1.15	0.825	0.974563	0	84.1
8.48	28.33	1	0	1.239	0.957149	0.992381	0	90.4
5.83	18.16	1	0	1.229	0.964472	0.995625	0	86
6.83	17.45	1	0	0.01	1E-17	3.97E-16	0	200

9	37.06	1	0	1.054	0.610029	0.927831	0	98.8
8.21	20.96	1	0	1.561	0.295494	0.785136	0	75.7
5.62	5.19	1	0	1.801	0.152749	0.610761	0	70.9
6.55	14.08	2	0	1.222	0.977238	0.997505	0	89.3
6.3	6.35	2	0	1.244	0.928763	0.99077	0	83.7
8.37	112.19	2	0	0.562	0.003178	0.047463	0	141.3
9.54	31.2	2	0	1.127	0.697813	0.956851	0	104.4
9.19	10.69	1	0	1.478	0.549213	0.909321	0	95.5
6.73	24.93	2	0	0.516	0.000159	0.00346	0	135.2
8.94	30.28	1	0	1.005	0.425533	0.873992	0	121.4
8.54	9.02	1	0	1.569	0.471506	0.898019	0	77.1
10.42	17.93	2	0	2.346	0.005053	0.066814	0	51.6
5.35	6.28	1	0	0.01	1E-17	3.97E-16	0	200
7.37	19.45	2	0	1.18	0.872675	0.983083	0	102
6.06	4.48	2	0	1.076	0.650638	0.942006	0	111.4
6.14	14.78	1	0	2.063	0.067424	0.386559	0	65.9
4.94	24.21	2	0	0.01	1E-17	3.97E-16	0	200
6.68	7.29	2	0	1.044	0.523547	0.90897	0	42.1
6.6	9.25	1	0	1.088	0.669459	0.95015	0	100
8.75	21.61	2	0	1.118	0.700861	0.958307	0	89.2
9.48	4.62	2	0	1.077	0.644994	0.9403	0	86.7
10.21	33.16	2	0	1.579	0.251877	0.73434	0	86
9	6.07	2	0	0.931	0.239293	0.718542	0	93.1
7.37	22.79	2	0	1.058	0.566478	0.913335	0	92.3
7.97	4.34	2	0	1.5	0.358174	0.834582	0	85.2
8.81	21.85	2	0	1.3	0.879698	0.983083	0	87
7.44	52.39	2	0	0.707	0.018281	0.171879	0	115.8
9.79	7.2	2	0	1.116	0.712926	0.960068	0	96.4
8.41	29.51	2	0	1.643	0.232996	0.711914	0	66.8
7.23	25.43	1	0	1.374	0.723305	0.96164	0	82
8.94	4.28	2	0	1.095	0.677206	0.951158	0	112.5
6.92	9.23	2	0	1.462	0.541867	0.90897	0	82.3
7.85	7.39	2	0	0.813	0.075364	0.413941	0	107.5
9.94	29.86	1	0	1.278	0.840767	0.977978	0	89
7.66	14.64	1	0	4.01	1.84E-05	0.000474	0	28.5
6.84	19.92	1	0	2.223	0.032022	0.246259	0	65.1
6.05	50.77	1	0	1.388	0.673559	0.951142	0	107.4
6.67	20.62	2	0	1.123	0.702413	0.958512	0	97.1
5.68	12.38	2	0	1.485	0.529189	0.90897	0	86
6.98	9.21	2	0	1.048	0.533724	0.90897	0	94.2
5.1	5.22	2	0	1.218	0.989015	0.998053	0	80.9
6.8	8.68	2	0	0.892	0.16436	0.630832	0	95.1
6.37	4.61	2	1	0.951	0.381909	0.853306	0	97.6
8.37	10.27	2	0	1.886	0.130623	0.561618	0	67.5
8.18	10.53	2	0	1.086	0.698982	0.957318	0	92.9

6.15	11.33	2	0	2.53	0.03054	0.239622	0	40
6.74	2.46	2	0	1.044	0.598918	0.925915	0	97.9
7.17	35.85	2	0	2.524	0.00851	0.098553	0	57.4
5.12	43.55	2	0	1.826	0.14154	0.591386	0	72.4
5.5	2.51	2	0	1.091	0.660538	0.945736	0	153.9
5.87	8.97	1	0	0.73	0.108082	0.511221	0	116.6
6.52	46.38	1	0	1.28	0.899353	0.987004	0	84.5
6.83	12.24	2	0	0.879	0.241096	0.721283	0	105.8
5.08	24.63	1	0	1.15	0.790385	0.97006	0	92.2
9.19	21.72	2	0	1.526	0.327159	0.806293	0	65.5
6.49	20.92	2	0	1.46	0.500713	0.906872	0	43.9
6.43	10.83	2	0	1.405	0.527789	0.90897	0	79.9
9.54	19.38	1	0	0.899	0.281434	0.76495	0	105.3
6.51	11.32	1	0	1.737	0.195246	0.672306	0	73.1
6.39	10.6	2	0	1.136	0.757807	0.966419	0	99.6
4.58	10.17	2	0	1.154	0.856789	0.981617	0	133.5
6.29	5.67	1	0	1.365	0.67176	0.950521	0	83.4
5.21	26.37	2	0	0.93	0.247068	0.726377	0	97.3
5.9	14.55	2	0	1.081	0.64695	0.940359	0	93.8
5.66	5.62	2	1	1.503	0.366016	0.842834	0	81
9.11	19.18	2	0	1.395	0.561303	0.911753	0	76.8
7.06	5.59	1	0	0.01	1E-17	3.97E-16	0	200
9.44	5.6	2	0	1.267	0.899066	0.987004	0	105.6
6.4	3	1	0	0.804	0.137823	0.578543	0	113.8
8.05	9.98	2	0	1.531	0.41375	0.865638	0	64.7
9.11	28.88	1	0	0.147	1E-17	3.97E-16	0	174.4
5.73	22.98	1	0	1.842	0.129965	0.559936	0	73.3
10.49	6.84	2	0	0.89	0.18956	0.663939	0	103
5.92	5.74	2	0	2.035	0.058156	0.353174	0	28.1
9.11	21.14	2	0	1.094	0.599934	0.925915	0	95.8
8.54	6.21	1	0	0.889	0.311579	0.798223	0	105.9
4.53	2.05	2	0	1.291	0.866482	0.981617	0	87.3
6.15	7.7	1	0	1.739	0.197233	0.673765	0	74.5
7.15	7.12	2	0	1.208	0.968807	0.997215	0	87.9
9.31	33.85	1	0	0.94	0.240971	0.721124	0	104.4
7.46	7.06	2	0	1.073	0.577911	0.919803	0	92.2
9.32	17.85	2	0	0.893	0.148208	0.60077	0	108.5
6.68	6.43	2	0	1.452	0.51005	0.90897	0	81.9
7.49	5.25	2	0	1.272	0.936509	0.99128	0	54.4
5.4	10.98	2	0	1.286	0.839875	0.977862	0	87
4.94	20.39	1	0	1.054	0.603767	0.925915	0	94.4
5.99	17.48	1	0	1.247	0.923333	0.989893	0	101.3
8.47	16.13	1	0	2.443	0.011321	0.123289	0	64.9
8.02	15.08	2	0	0.908	0.212444	0.692684	0	101.1
7.05	19.47	2	0	1.121	0.746092	0.964844	0	92.7

9.04	6.81	2	0	1.856	0.059281	0.357282	0	76.9
7.55	2.85	2	0	0.916	0.354894	0.831984	0	103.6
6.98	16.03	2	0	0.583	0.006341	0.079355	0	122.1
6.58	4.48	1	0	100	1E-17	3.97E-16	0	
9.03	2.23	2	0	1.05	0.603781	0.925915	0	85.8
5.83	24.06	1	0	2.373	0.012954	0.134438	0	57.8
6.87	23.28	2	0	1.326	0.712785	0.960068	0	84.9
8.95	47.49	1	0	1.513	0.376145	0.850309	0	85
5.92	6.09	2	0	0.848	0.192021	0.66659	0	90.4
5.07	12.9	1	0	0.782	0.128299	0.55391	0	117
6.15	38	2	0	0.895	0.317373	0.801098	0	105.5
7.53	19.12	2	0	0.896	0.198261	0.67586	0	110.2
6.05	9.2	2	0	1.26	0.887801	0.983874	0	105.3
6.11	14.65	2	0	1.089	0.614397	0.929757	0	93.9
5.63	6.21	2	0	2.014	0.120706	0.539718	0	67.9
8.22	34.75	2	0	1.126	0.719283	0.96164	0	86.9
10.05	19.78	2	0	3.207	1.87E-05	0.00048	0	45.3
6.57	35.6	1	0	1.443	0.456757	0.890938	0	84.5
9.04	20.22	2	0	1.209	0.950364	0.991422	0	79.4
5.06	10.11	2	0	1.329	0.78641	0.97006	0	79
6.44	5.56	1	0	0.953	0.425443	0.873992	0	109.2
7.87	12.69	2	0	1.607	0.231939	0.711304	0	77.2
9.98	47.62	1	0	0.875	0.14435	0.596041	0	106.8
8.02	44.53	2	0	1.567	0.315564	0.800259	0	78
7.42	20.77	1	0	1.31	0.846246	0.978694	0	87.4
5.39	17.59	2	0	0.848	0.132542	0.566984	0	106.6
8.85	7.2	2	0	0.941	0.328463	0.806293	0	121
8.32	13.59	2	0	1.567	0.420143	0.868075	0	76.6
6.09	5.76	2	0	1.322	0.755094	0.96597	0	136.8
7.68	8.05	1	0	0.528	0.00744	0.089117	0	112.3
6.54	34.05	1	0	1.266	0.919515	0.98911	0	93.1
5.14	3.21	1	0	0.781	0.151284	0.60692	0	112.3
6.61	26.88	2	0	1.046	0.468131	0.896668	0	79.9
7.23	10.1	2	0	1.452	0.495536	0.906872	0	82.5
8.75	13.77	1	0	1.416	0.680243	0.951158	0	90.1
8.88	24.32	1	0	1.706	0.141672	0.591545	0	92.5
6.76	4.67	1	0	0.675	0.025964	0.216605	0	119.4
5.91	3.24	1	0	1.361	0.755479	0.96597	0	100.8
5.92	21.13	2	0	1.697	0.237545	0.716106	0	65.4
6.48	18.51	2	0	1.162	0.849452	0.979998	0	114.5
6.54	13.83	2	0	1.283	0.864975	0.981617	0	72.2
5.26	5.74	2	0	1.356	0.666072	0.94811	0	87.7
5.92	31.53	2	0	0.914	0.281574	0.76495	0	115.2
7.88	9.33	2	0	1.158	0.838174	0.977517	0	91.5
5.96	16.57	1	0	1.693	0.231753	0.711304	0	73.9

9.8	5.27	1	0	2.233	0.074638	0.412657	0	60.6
8.56	10.64	1	0	0.879	0.242415	0.723465	0	101.7
9.66	9.84	1	0	1.1	0.714826	0.960203	0	95.2
5.35	4.67	2	0	0.947	0.267055	0.750771	0	128.1
6.89	13.23	1	0	0.01	1E-17	3.97E-16	0	200
10.23	5.87	2	0	1.47	0.432544	0.880832	0	75
8.72	3.88	2	0	0.985	0.395433	0.861859	0	96.6
6.48	49.53	1	0	1.048	0.506449	0.90897	0	93.5
6.8	15.89	2	0	0.922	0.220151	0.700223	0	105.9
7.75	12.36	1	0	0.989	0.491386	0.905851	0	100.9
6.77	7.7	2	0	1.341	0.680527	0.951158	0	72.9
8.32	21.05	1	0	1.029	0.399994	0.862461	0	94.7
6.93	104.24	2	0	1.104	0.728002	0.962129	0	105.2
8.21	11.95	1	0	1.283	0.819883	0.973778	0	87.6
9.19	16.88	1	0	1.078	0.656327	0.943998	0	91.5
6.96	3.72	2	0	0.865	0.191801	0.666276	0	111.2
8.69	9.56	2	0	0.926	0.223178	0.701603	0	104.3
6.93	80.78	1	0	0.789	0.075059	0.413941	0	113.3
7.27	13.23	1	0	1.283	0.861729	0.981617	0	87.6
7.18	9.13	1	0	0.936	0.344682	0.822615	0	103.3
5.76	5.72	2	0	1.103	0.709767	0.959398	0	100.6
7.06	38.06	1	0	1.485	0.489469	0.9051	0	101.8
9.39	23.15	2	0	1.026	0.44675	0.888233	0	101.1
6.29	6.1	1	0	1.045	0.577575	0.919803	0	93
6.83	3.9	1	0	0.522	0.003233	0.047943	0	132.5
8.76	4.6	2	0	3.736	0.000207	0.004393	0	43.3
6.34	22.75	1	0	1.387	0.656867	0.943998	0	84.1
5.41	5.75	2	0	1.737	0.204443	0.683145	0	55.5
6.35	6.76	2	0	1.215	0.96282	0.995069	0	98.6
4.72	20.23	1	0	1.208	0.988181	0.998053	0	96.8
5.57	1.94	2	0	0.854	0.216131	0.697303	0	127.2
6.3	8	1	0	1.391	0.62659	0.932683	0	81.8
5.2	10.01	2	0	0.796	0.107399	0.509248	0	112.5
5.36	3.59	1	0	1.418	0.583959	0.922392	0	87
7.66	71.04	2	0	1.82	0.076338	0.417553	0	74.6
7.5	9.09	2	0	1.258	0.891281	0.985364	0	65.1
5.01	18.08	2	0	1.168	0.747871	0.964844	0	89.2
5.2	6.62	1	0	1.311	0.845327	0.978239	0	87
9.8	30.69	2	0	0.817	0.0746	0.412657	0	108.3
4.39	0	2	0	1.194	0.934227	0.991048	0	57.1
6.49	8	2	0	2.043	0.021875	0.194897	0	48.4
8.29	15.19	2	0	1.944	0.093027	0.47033	0	49.3
8	11.74	2	0	1.264	0.887119	0.983874	0	74.1
7.85	23.83	2	0	1.526	0.345418	0.823418	0	86.8
6.67	15.71	1	0	1.506	0.522532	0.90897	0	82

7.78	5.44	2	0	1.302	0.862976	0.981617	0	82.3
6.79	3.02	2	0	0.502	0.005966	0.075981	0	119.3
5.44	31.58	1	0	1.057	0.533099	0.90897	0	98.9
5.43	12.1	2	0	1.633	0.290168	0.775732	0	93
9.2	5.64	1	0	1.565	0.304997	0.792264	0	56.2
8.37	13.31	1	0	1.701	0.336792	0.814964	0	76
7.05	4.36	1	0	1.075	0.664313	0.947596	0	96.4
4.79	6.76	2	0	1.398	0.622222	0.932683	0	88.3
9.04	10.97	2	0	0.829	0.0905	0.46399	0	111.2
7.25	15.62	2	0	0.818	0.147017	0.598768	0	149.4
9.04	15.02	2	0	0.666	0.035681	0.264382	0	152.9
6.84	20.34	2	0	1.434	0.49811	0.906872	0	82.8
6.95	9.7	1	0	1.327	0.772676	0.97006	0	85.2
6.19	4.36	2	0	1.4	0.708933	0.959398	0	87.5
4.83	15.58	2	0	1.326	0.757141	0.966419	0	87.5
5.91	8.76	2	0	1.233	0.985635	0.997735	0	77
9.96	9.86	2	0	1.817	0.135868	0.576101	0	82.6
9.22	13.99	1	0	1.212	0.97645	0.997505	0	84.3
8.53	4.3	2	0	1.099	0.715726	0.960634	0	84.7
5.12	3.57	1	0	1.326	0.764128	0.967498	0	94.5
6.21	7.9	2	0	0.598	0.013292	0.137158	0	136.3
8.37	13.48	1	0	1.194	0.927835	0.99077	0	94.3
9.32	8.71	1	0	2.55	0.027658	0.224346	0	71.1
5.87	18.89	2	0	1.285	0.844917	0.97823	0	89.2
5.62	19.16	2	0	1.229	0.999919	0.99992	0	84.8
8.62	21.35	2	0	1.373	0.670974	0.95015	0	85.9
9.42	6.52	1	0	1.423	0.557215	0.910977	0	82.3
8.51	20.05	2	0	1.232	0.954794	0.991422	0	91.8
9.39	26.34	1	0	1.086	0.679109	0.951158	0	100.7
9.38	10.63	2	0	0.77	0.075595	0.414669	0	119.2
6.54	19.82	2	0	0.944	0.261536	0.746056	0	135.8
6.54	5.82	2	0	0.985	0.41382	0.865638	0	99.7
8.32	6.83	2	0	1.625	0.23644	0.713801	0	90.6
5.55	3.55	1	0	0.01	1E-17	3.97E-16	0	200
9.38	15.29	2	0	0.903	0.159001	0.621055	0	108.2
8.37	8.22	1	0	1.43	0.486449	0.903584	0	85.9
6.24	6.36	2	0	1.937	0.122601	0.542733	0	59.8
5.68	4.68	2	0	1.041	0.595314	0.925915	0	115
5.54	10.47	1	0	0.942	0.272144	0.756464	0	104.6
5.45	9.2	2	0	0.575	0.006763	0.082899	0	130.5
7.9	26.65	2	0	1.025	0.556752	0.910977	0	96.5
8.16	2.64	2	0	1.3	0.827659	0.974887	0	79.9
8.46	6.55	2	0	1.131	0.785661	0.97006	0	95.4
9.29	11.22	1	0	0.01	1E-17	3.97E-16	0	200
9.67	14.89	2	0	1.437	0.472103	0.898019	0	70.6

9.25	6.68	1	0	0.848	0.144113	0.59581	0	111.5
9.16	10.08	2	0	1.128	0.686828	0.952468	0	84.8
9.74	5.95	2	0	1.189	0.902284	0.987276	0	101.8
8.4	3.7	1	0	0.546	0.001899	0.031043	0	129.4
6.64	11.93	1	0	1.259	0.93826	0.99128	0	88.5
6.84	0	2	0	1.182	0.890541	0.985152	0	62.9
7.97	14.35	1	0	1.385	0.615802	0.930023	0	80.9
7.36	5.54	2	0	1.198	0.945249	0.991397	0	89.5
7.03	3.57	1	0	0.707	0.061514	0.365458	0	113.8
8.38	6.66	2	0	1.055	0.545986	0.909126	0	108.2
6.16	4.09	1	0	1.339	0.807094	0.97006	0	81
8.16	23.14	1	0	0.907	0.287068	0.770685	0	102.4
8.34	6.27	2	0	1.249	0.925591	0.99022	0	103.3
4.4	12.03	2	0	1.096	0.70175	0.958482	0	89.8
6.33	13.07	1	0	0.992	0.445278	0.888233	0	102.9
6.1	13.56	2	0	1.21	0.983826	0.997735	0	79.1
9.01	6.62	1	0	0.998	0.513766	0.90897	0	104.6
7.11	10.21	1	0	2.646	0.005012	0.066487	0	54.6
5.54	18.16	2	0	1.396	0.551044	0.910041	0	83.5
6.06	14.48	1	0	1.719	0.173297	0.64202	0	90.8
6.05	4.6	2	0	1.368	0.678749	0.951158	0	81.8
6.35	6.76	2	0	0.995	0.474648	0.898019	0	65.4
6.89	2.58	1	0	1.387	0.725971	0.96164	0	84.4
5.17	8.95	2	0	1.244	0.954748	0.991422	0	67
10.21	33.54	1	0	0.01	1E-17	3.97E-16	0	200
8.09	4.33	2	0	0.578	0.001847	0.03039	0	147.3
6.93	8.09	2	0	1.117	0.681266	0.951658	0	80.6
7.93	16.99	2	0	1.261	0.905412	0.987715	0	87.3
4.94	42.45	1	0	0.793	0.104307	0.500809	0	110.8
5.27	11.83	1	0	1.256	0.937166	0.99128	0	88.4
7.42	19.74	1	0	1.477	0.45959	0.891873	0	76.9
8.68	13.2	1	0	0.723	0.063777	0.374776	0	108.1
8.51	8.86	1	0	1.305	0.849047	0.979622	0	75.2
10.9	22.01	2	0	1.774	0.120926	0.539718	0	56.3
6.35	9.98	2	0	1.517	0.448587	0.888233	0	77.5
9.61	9.93	1	0	1.287	0.88358	0.983234	0	82.1
7.65	6.96	2	0	1.729	0.27047	0.753639	0	117.7
5.92	15.76	1	0	1.594	0.338947	0.817591	0	83.6
7.78	20.59	1	0	1.722	0.257364	0.740472	0	67
7.81	9.06	2	0	1.064	0.607795	0.927253	0	99.6
6.93	34.99	2	0	1.629	0.317777	0.801543	0	70
6.1	9.75	2	0	2.173	0.031837	0.245432	0	153
8.1	9.94	1	0	1.316	0.817204	0.972656	0	84.8
9.6	74.5	1	0	1.234	0.939976	0.991397	0	90
6.46	7.13	2	0	1.428	0.590838	0.924336	0	60

8.29	6.55	1	0	1.067	0.619159	0.932207	0	100.8
6.57	15.01	2	1	1.046	0.503909	0.908772	0	103.3
6.62	9.82	2	0	1.26	0.955037	0.991422	0	91.5
7.24	3.87	2	0	3.105	0.004114	0.058012	0	48.7
5.35	6.65	1	0	1.246	0.948445	0.991422	0	91.9
5.69	8.27	2	0	1.462	0.592946	0.924683	0	81.2
6.81	6.14	2	0	1.399	0.538943	0.90897	0	112.7
10.14	3.92	1	0	0.351	2.51E-07	8.09E-06	0	135.8
7.14	18.67	2	0	1.944	0.039426	0.281208	0	57.7
8.65	6.09	1	0	0.757	0.132698	0.566984	0	122
9.2	21.05	2	0	1.637	0.193926	0.669224	0	62.5
9.1	10.27	1	0	0.506	0.003916	0.056532	0	134.2
6.3	25.13	1	0	1.197	0.898634	0.987004	0	90.6
5.24	16.35	2	0	1.949	0.081326	0.438089	0	128.1
5.85	3.64	2	0	1.637	0.304114	0.79167	0	77.1
6.44	4.9	1	0	2.054	0.082381	0.440567	0	65.5
5.8	15.7	2	0	1.44	0.540116	0.90897	0	81.6
5	6	2	0	1.076	0.630089	0.933268	0	81
6.68	4.37	2	0	1.226	0.986421	0.997735	0	87.6
7.34	4.34	1	0	2.22	0.017098	0.163796	0	59.7
8.68	23.45	1	0	0.952	0.370786	0.846091	0	77.2
9.32	26.22	1	0	1.161	0.861194	0.981617	0	88.3
4.88	8.92	1	0	1.41	0.594387	0.925738	0	83.9
7.78	4.76	2	0	0.858	0.211986	0.692087	0	107.6
7.47	8.02	2	0	0.778	0.098996	0.489111	0	117.4
5.86	26.42	1	0	0.923	0.26754	0.751128	0	95.7
5.11	6.93	2	0	0.893	0.199557	0.677705	0	135.5
6.73	10.24	2	0	1.263	0.886902	0.983874	0	79.3
9.42	9.47	2	0	1.404	0.61778	0.931228	0	67.3
8.38	7.28	2	0	1.549	0.355347	0.832273	0	51.3
6.29	24.42	1	0	2.077	0.056693	0.348019	0	59.7
8.43	45.67	1	0	1.103	0.644938	0.9403	0	93.4
8.22	4.54	2	0	1.176	0.899899	0.987261	0	84.2
6.95	2.45	1	0	0.282	5.8E-07	1.81E-05	0	158.7
7.55	13.59	1	0	1.256	0.912962	0.98911	0	93.2
5.78	7.76	2	1	1.363	0.637228	0.937605	0	82.5
7.03	9.91	1	0	1.097	0.689028	0.952468	0	94.1
7.8	2.72	1	0	1.677	0.254455	0.735945	0	74.7
5.77	4.86	2	0	2.208	0.059872	0.358302	0	64.2
8.46	4.29	2	0	0.87	0.19587	0.672983	0	106.2
6.68	19.16	1	0	1.149	0.798537	0.97006	0	90.4
7.36	9.83	2	0	1.472	0.504881	0.908772	0	82.3
8.38	12.61	2	0	1.693	0.145764	0.59831	0	77
9.73	9.18	2	0	1.349	0.731753	0.962196	0	82.5
9.03	5.32	2	0	1.535	0.469204	0.896668	0	94.1

10.13	10.33	1	0	1.273	0.880733	0.983083	0	83
6.32	17.53	2	0	0.99	0.439005	0.886247	0	99
6.98	2.1	2	0	0.859	0.182211	0.652641	0	105.5
9.36	5.84	1	0	0.663	0.056378	0.346891	0	120.3
9.11	25.77	2	1	2.157	0.013849	0.14151	0	58.1
7.8	18.3	1	0	1.78	0.117012	0.532563	0	68.7
7.64	9.96	1	0	2.239	0.051978	0.330812	0	68.9
4.53	7.05	1	0	1.097	0.691416	0.952547	0	95.4
9.14	3.12	1	0	1.46	0.565024	0.913241	0	82.3
5.15	29.79	2	0	1.261	0.878282	0.983083	0	94.3
7.78	34.07	1	0	1.247	0.947586	0.991422	0	85.9
6.79	5.77	2	0	1.982	0.1511	0.606568	0	67
5.16	18.17	1	0	1.084	0.674973	0.951158	0	79.3
4.45	4.94	2	0	1.213	0.951021	0.991422	0	61.3
7.64	4.86	2	0	1.09	0.67588	0.951158	0	75.1
9.41	13.62	1	0	1.421	0.631355	0.933951	0	74.6
8.1	18.26	2	0	1.448	0.45712	0.891247	0	78.9
11.74	78.75	2	0	2.31	0.012535	0.131067	0	69.9
6.11	4.47	2	0	1.142	0.780949	0.97006	0	89.5
7.34	25.04	1	0	1.462	0.574282	0.919147	0	80.1
10.93	9.29	1	0	2.086	0.017894	0.169121	0	60.8
9.07	1.88	2	0	2.257	0.007584	0.090321	0	177.2
9.41	4.02	1	0	1.372	0.705864	0.958927	0	86
5.27	18.87	1	0	1.423	0.596305	0.925915	0	88.7
10.89	12.53	1	0	0.746	0.072275	0.404387	0	114.6
7.87	8.61	1	0	1.32	0.781935	0.97006	0	79.5
5.27	19.96	2	0	1.502	0.453301	0.888233	0	85.3
5.71	25.09	1	0	1.373	0.593027	0.924683	0	84.9
6.83	7.22	1	0	1.919	0.147775	0.599917	0	76.7
8.54	4.08	2	0	1.045	0.606265	0.926809	0	104.6
5.73	5.86	2	0	1.18	0.82347	0.974465	0	109.4
5.36	4.32	1	0	1.237	0.999869	0.99992	0	85.2
5.41	1.89	2	0	1.698	0.277765	0.763386	0	61.8
9.8	31.08	2	0	1.472	0.485988	0.903584	0	82.4
6.87	6.58	2	0	0.833	0.092961	0.47033	0	118.4
8.31	6.68	2	0	0.71	0.01711	0.163796	0	102.2
5.08	19.27	1	0	0.993	0.488859	0.904371	0	108.3
7.78	4.79	1	0	1.605	0.429382	0.877943	0	70.8
8.56	9.79	1	0	1.034	0.52072	0.90897	0	93.9
6.05	6.46	2	0	1.733	0.199613	0.677705	0	71.1
5.99	16.11	1	0	0.793	0.068219	0.389606	0	109
8.72	21.22	1	0	1.43	0.489801	0.905208	0	81.8
8.59	7.56	1	0	1.51	0.43325	0.881555	0	86.4
6.79	4.6	2	0	1.687	0.176737	0.64543	0	63.1
6.92	6.56	1	0	1.344	0.69113	0.952547	0	100.7

5.72	4.06	2	0	0.865	0.150449	0.604728	0	110.4
9.73	27.31	1	0	1.103	0.734652	0.962304	0	88.2
8.6	24.55	2	0	1.142	0.780705	0.97006	0	93.1
5.67	6.44	2	0	1.161	0.855747	0.981617	0	94.4
4.94	13.89	1	0	1.676	0.303718	0.79167	0	73.8
6.76	5.42	1	0	1.466	0.561901	0.912156	0	82
5.47	6.6	2	0	0.856	0.11868	0.535654	0	106.3
6.4	3.67	1	0	0.01	1E-17	3.97E-16	0	200
8.54	20.75	2	0	1.955	0.11667	0.532131	0	75.4
6.55	9.48	1	0	1.286	0.848501	0.979336	0	94.6
8.54	6.07	2	0	1.305	0.784468	0.97006	0	75.4
6.67	3.81	2	0	2.11	0.046352	0.306471	0	139.3
6.79	7.77	1	0	0.01	1E-17	3.97E-16	0	200
5.87	2.33	2	0	2.118	0.065786	0.382001	0	51.2
7.09	2.27	2	0	0.833	0.229385	0.708001	0	146.5
4.34	10.64	1	0	1.208	0.939971	0.991397	0	83.5
8.05	6.4	2	0	1.56	0.312386	0.798743	0	73.6
5.67	8.08	1	0	0.01	1E-17	3.97E-16	0	200
8.92	4.46	1	0	1.402	0.624107	0.932683	0	83.3
8.47	10.09	1	0	1.427	0.496197	0.906872	0	82
5.11	11.21	1	0	0.822	0.155519	0.616223	0	111
6.27	18.64	1	0	0.955	0.370886	0.846091	0	110.2
7.46	12.14	1	0	2.033	0.085551	0.448192	0	74.5
4.61	10.6	1	0	1.297	0.867066	0.981617	0	87
8.66	11.55	1	0	1.073	0.574459	0.919147	0	95.7
6.01	2.37	1	0	1.013	0.545689	0.90897	0	98.9
9.32	11.05	1	0	1.039	0.532904	0.90897	0	98.9
6.44	5.54	2	0	1.164	0.811008	0.971	0	101.1
6.61	5.15	2	0	8.136	1.33E-15	5.24E-14	0	21.9
7.15	30.85	1	0	1.077	0.647989	0.940966	0	103.8
6.23	4.95	2	0	0.926	0.322703	0.805255	0	98.9
7.3	5.06	1	0	0.863	0.139662	0.584315	0	109.6
7.06	5.57	1	0	1.444	0.604447	0.925915	0	84.5
4.74	6.24	1	0	0.857	0.183046	0.653906	0	108.3
8.37	7.52	1	0	0.989	0.453773	0.888233	0	109.2
8.02	5.24	1	0	1.168	0.871286	0.98284	0	92.3
6.57	2.23	1	0	1.885	0.196341	0.673222	0	65.6
8	7.21	2	0	0.63	0.017887	0.169121	0	126.2
6.77	8.69	1	0	0.988	0.425645	0.873992	0	98
11.09	3.6	2	0	0.679	0.014164	0.144377	0	106.3
9.32	6.35	2	0	2.007	0.043954	0.299299	0	80.8
6.62	4.7	1	0	1.314	0.822717	0.974456	0	83.9
10.15	18.4	1	0	1.109	0.68551	0.952226	0	92.2
6.86	3.2	1	0	1.556	0.425422	0.873992	0	78.2
6.76	47.56	2	0	0.887	0.157136	0.61786	0	105

9.42	15.06	1	0	0.696	0.019688	0.180515		0	116.1
9.39	25.31	1	0	1.783	0.157496	0.61786		0	73.2
9.57	8.66	1	0	1.367	0.688028	0.952468		0	89.1
8.68	9.44	2	0	1.058	0.502375	0.907771		0	103.2
9.35	8.88	1	0	1.285	0.853476	0.981617		0	85.9
7.01	15.43	1	0	0.748	0.030799	0.240675		0	114.4
9.85	22.3	2	0	1.386	0.597829	0.925915		0	69
5.92	5.17	2	0	1.267	0.868859	0.981633		0	86.4
5.03	4.38	2	0	0.903	0.210596	0.691133		0	106.9
5.33	2.81	2	0	0.924	0.328209	0.806293		0	107.9
7.34	15.02	1	0	1.145	0.814867	0.971896		0	95.2
9.29	2.16	2	0	1.773	0.214622	0.694929		0	81
5.77	6.27	2	0	0.876	0.188378	0.663939		0	110
9.06	7.07	2	0	1.183	0.806907	0.97006		0	95.7
7.56	7.85	2	0	0.713	0.019723	0.180708		0	116.8
8.51	6.98	2	0	0.965	0.327957	0.806293		0	101.2
6.64	3.35	1	0	0.805	0.130435	0.561328		0	108.2
8.43	6.26	1	0	1.122	0.724718	0.96164		0	91
8.06	6.12	2	0	1.412	0.592969	0.924683		0	87.3
8.34	3.71	2	0	1.476	0.545617	0.90897		0	47.9
5.78	14.09	1	0	1.211	0.953576	0.991422		0	93.2
5.63	4.45	1	0	3.835	0.000491	0.009804		0	41.4
8.51	29.64	2	0	0.74	0.031588	0.244263		0	113.5
4.56	28.73	1	0	0.944	0.301767	0.791593		0	102.8
8.37	7.96	2	0	1.306	0.763391	0.967182		0	87.8
6.23	9.23	1	0	0.01	1E-17	3.97E-16		0	200
4.55	6.53	1	0	1.041	0.596293	0.925915		0	101
6.57	8.27	1	0	3.711	2.14E-06	6.31E-05		0	44.3
8.25	4.5	2	0	1.22	0.999883	0.99992		0	85.8
8.9	17.85	1	0	0.97	0.357756	0.834469		0	121.9
5.16	3.21	2	0	0.894	0.244271	0.725276		0	101.7
9.07	6.89	2	0	1.249	0.900442	0.987276		0	86.6
7.52	26.9	1	0	1.3	0.797335	0.97006		0	90.1
8.24	2.53	1	0	1.366	0.655094	0.943998		0	83.7
6.7	4.15	2	0	1.55	0.446303	0.888233		0	63.6
6.23	5.9	1	0	3.091	5.09E-05	0.001226		0	45.2
5.67	26.78	1	0	0.935	0.354652	0.831572		0	102.1
8.51	0	2	0	1.067	0.533379	0.90897		0	85.8
4.96	31.74	2	0	0.845	0.102868	0.499812		0	105.9
9.57	12.14	1	0	1.387	0.70048	0.957995		0	81
7.49	0	1	0	1.199	0.922323	0.989893		0	91.9
6.43	10.38	2	0	1.588	0.235479	0.71295		0	88.3
8.76	1.86	2	0	1.298	0.885378	0.983234		0	85.2
6.1	5.72	1	0	1.879	0.067377	0.386559		0	63.1
6.73	9.57	1	0	1.245	0.967259	0.996855		0	90.3

5.26	10.96	1	0	1.537	0.469089	0.896668	0	73
7.53	5.4	1	0	0.936	0.346663	0.824064	0	104.9
6.19	9.82	1	0	1.099	0.692474	0.952739	0	96.7
6.42	2.74	1	0	1.634	0.389453	0.859423	0	62.9
5.49	3.35	1	0	1.06	0.634566	0.936195	0	94.4
9.89	4.25	1	0	1.062	0.600101	0.925915	0	103.9
6.39	7.29	1	0	0.941	0.353403	0.83025	0	103
8.84	11.14	1	0	0.522	0.001929	0.03139	0	120.1
6.68	3.83	1	0	3.528	3.22E-06	9.29E-05	0	17
6.38	8.93	2	0	1.154	0.824745	0.974465	0	96
6.35	2.1	2	0	1.375	0.662628	0.946953	0	67.3
6.54	6.62	1	0	1.038	0.534707	0.90897	0	100.1
6.55	3.26	1	0	1.534	0.394366	0.861859	0	78.9
7.37	5.64	1	0	1.03	0.520339	0.90897	0	91.4
5.74	7.28	1	0	1.583	0.329404	0.806293	0	78.6
7.05	6.18	2	0	1.129	0.736001	0.962867	0	91.9
7.87	26.12	1	0	1.27	0.881049	0.983083	0	92.2
9.86	8.29	2	0	0.849	0.097603	0.483748	0	103.7
8.65	11.29	1	0	1.161	0.826753	0.974887	0	92.1
7.56	2.51	1	0	1.417	0.645752	0.940359	0	82.7
6.96	15.1	1	0	1.239	0.956975	0.992381	0	90.6
7.28	2.49	1	0	1.403	0.667544	0.9485	0	108.4
9.01	17.54	1	0	1.17	0.876218	0.983083	0	91.3
6.24	4.68	2	0	1.35	0.70807	0.959206	0	78.8
11.44	21.9	1	0	1.051	0.458261	0.891247	0	98.4
8.38	4.94	2	0	1.448	0.507661	0.90897	0	81.4
11.24	6.24	1	0	1.556	0.370095	0.846091	0	78.2
10.71	15.69	1	0	0.995	0.436539	0.884247	0	96
5.6	3.41	1	0	100	1E-17	3.97E-16	0	
9.42	14.19	1	0	1.346	0.673818	0.951158	0	87.6
5.07	9.97	1	0	1.523	0.478586	0.898527	0	82.8
6.01	2.62	1	0	1.225	0.97171	0.997505	0	89.9
6.57	6.62	2	0	1.749	0.232077	0.711304	0	75.1
9.28	2.66	1	0	2.25	0.0389	0.27934	0	60.6
8.56	3.06	1	0	1.488	0.469416	0.896668	0	80
8.63	6.35	1	0	1.018	0.54932	0.909321	0	93.6
7.27	5.67	1	0	1.166	0.850578	0.980769	0	92.3
10.62	8.99	1	0	1.03	0.538928	0.90897	0	101.2
5.17	2.96	1	0	1.859	0.189752	0.663939	0	69.4
7.34	3.19	1	0	1.427	0.63038	0.933286	0	83.8
7.39	10.69	1	0	1.017	0.517819	0.90897	0	97.7
9.23	2.48	1	0	1.263	0.889446	0.984632	0	86.3
6.3	4.93	1	0	3.177	0.002386	0.037274	0	49
5.03	4.78	2	0	0.969	0.457242	0.891247	0	92.2
7.25	8.19	1	0	44.716	1E-17	3.97E-16	0	4.1

8.38	3.74	1	0	1.109	0.74645	0.964844	0	88.8
7.03	20.22	1	0	0.999	0.452955	0.888233	0	101.2
6.92	13.2	2	0	1.672	0.255715	0.738347	0	78.2
8.48	13.04	1	0	1.018	0.425996	0.874364	0	109.7
6.14	6.25	1	0	1.794	0.25851	0.740475	0	70.5
6.57	27.44	1	0	1.344	0.667247	0.948458	0	83
6.99	2.54	1	0	1.326	0.7958	0.97006	0	87
9.42	74.48	1	0	1.332	0.760786	0.966437	0	80.4
7.94	4.14	2	0	1.435	0.567661	0.913335	0	76.9
7.75	8.43	1	0	1.381	0.663154	0.946953	0	88.7
5.44	3.1	1	0	0.897	0.270107	0.753413	0	105.1
6.93	2.87	1	0	0.902	0.264504	0.748141	0	101.5
9.7	12.19	1	0	1.481	0.48388	0.90176	0	83.8
8.72	5.62	1	0	1.096	0.714832	0.960203	0	87.8
7.93	11.39	1	0	2.268	0.02373	0.207601	0	62.6
6.01	2.19	1	0	0.691	0.041344	0.290284	0	118.3
5.21	8.12	2	0	0.783	0.097173	0.482952	0	115.1
8.5	2.49	1	0	0.544	0.012281	0.129379	0	125.3
7.49	6.19	1	0	1.48	0.499919	0.906872	0	77.7
8.48	5.26	1	0	2.607	0.00209	0.033368	0	52.6
8.51	6.06	1	0	0.937	0.331577	0.808657	0	108.4
6.07	0	1	0	1.272	0.933832	0.991048	0	85.1
5.12	10.27	1	0	1.458	0.449111	0.888233	0	81.5
6.38	6.85	1	0	0.889	0.270204	0.753413	0	105.9
7.14	3.28	1	0	1.331	0.797387	0.97006	0	102.7
6.8	7	1	0	1.163	0.842944	0.978048	0	95.9
9.32	37.83	1	0	1.233	0.999899	0.99992	0	85.6
7.44	5.39	1	0	2.071	0.103314	0.500065	0	81
5.69	5.66	1	0	1.65	0.207573	0.687824	0	78.1
5.83	15.04	1	0	1.437	0.539514	0.90897	0	83.4
5.82	2.99	1	0	0.953	0.323824	0.805731	0	109.4
7.31	2.68	1	0	1.153	0.823614	0.974465	0	92.9
9.61	18.99	1	0	1.1	0.690452	0.952547	0	92.2
5.81	11.42	1	0	1.127	0.792103	0.97006	0	91
5.54	11.82	1	0	1.068	0.562976	0.912724	0	98.1
9.04	11.73	1	0	1.239	0.947852	0.991422	0	83.7
9.79	0	1	0	1.115	0.76275	0.967182	0	99.1
5.22	2.24	1	0	1.028	0.567851	0.913335	0	98.6
5.55	15.59	1	0	1.057	0.532826	0.90897	0	97.2
5.66	10.37	1	0	1.16	0.846908	0.978953	0	92.9
8.13	7.06	1	0	1.331	0.797108	0.97006	0	88.4
8.65	15.26	1	0	0.963	0.437309	0.884686	0	108.5
6.76	20.68	1	0	1.482	0.39736	0.862453	0	93.2
6.49	23.6	1	0	1.114	0.644995	0.9403	0	92
5.68	16.6	1	0	1.014	0.440432	0.887298	0	100.3

6	2.29	1	0	5.988	1.48E-07	4.84E-06	0	26.3
5.72	11.26	1	0	0.974	0.457944	0.891247	0	101.3
7.4	7.99	1	0	1.385	0.697316	0.956785	0	83.9
6.58	11.49	1	0	1.555	0.442857	0.888233	0	83.5
9.28	3.2	1	0	0.998	0.466985	0.89585	0	100.1
9.01	0	1	0	0.867	0.274158	0.758044	0	99.4
4.64	4.52	1	0	0.843	0.18411	0.655884	0	106.6
5.64	0	1	0	0.543	0.011447	0.124235	0	129.6
6.1	4.86	1	0	0.773	0.152091	0.608994	0	115.3
5.94	2.5	1	0	1.035	0.553846	0.910977	0	99.4
5.19	8.01	1	0	1.392	0.655347	0.943998	0	78.7
5.15	7.48	1	0	1.237	0.9857	0.997735	0	88.3
6.06	4.9	1	0	1.355	0.789418	0.97006	0	85.2
8.18	3.34	1	0	2.272	0.03246	0.248109	0	61.1
7.53	3.23	1	0	0.807	0.180784	0.650812	0	111.7
6.16	20.43	1	0	0.954	0.284109	0.766046	0	91.3
5.55	7.95	1	0	1.098	0.709102	0.959398	0	95.3
4.92	8.69	1	0	1.018	0.551202	0.910041	0	97.9
5.53	38.76	1	0	0.909	0.292502	0.778968	0	102.2
5.73	9.71	1	0	2.105	0.081593	0.438217	0	64.4
11.58	44.25	1	0	1.058	0.48308	0.90163	0	97.3
6.6	7.1	1	0	1.584	0.279917	0.76495	0	80.5
6.24	2.06	1	0	1.447	0.597118	0.925915	0	81
5.45	7.78	1	0	0.876	0.272055	0.756425	0	108.6
4.87	2.72	1	0	0.768	0.042747	0.296459	0	113.1
9.82	9.05	1	0	0.756	0.03055	0.239622	0	97.5
10.29	18.79	1	0	0.854	0.192437	0.667196	0	102.4
7.34	8.42	1	0	1.105	0.680465	0.951158	0	94.1
9.29	8.56	1	0	0.909	0.244001	0.725276	0	103.8
6.43	3.39	1	0	1.091	0.714078	0.960068	0	104.2
9.13	5.14	1	0	1.225	0.9706	0.997215	0	89.9
7.69	11.26	1	0	1.056	0.527764	0.90897	0	97.1
5.26	9.29	1	0	1.118	0.726116	0.96164	0	96.2
5.03	7.22	1	0	1.321	0.8444	0.97823	0	89.1
5.66	8.48	1	0	1.448	0.543869	0.90897	0	89.2
5.66	5.17	1	0	1.228	0.975335	0.997505	0	100.1
6.71	13.19	1	0	1.368	0.686369	0.952468	0	88.1
8.78	2.36	1	0	1.637	0.355816	0.832518	0	77.1
7.27	5.84	1	0	1.623	0.368913	0.846091	0	84.1
7.81	11.36	1	0	1.324	0.773091	0.97006	0	85.2
8.65	8.94	1	0	1.256	0.950153	0.991422	0	93.2
5.5	9.6	1	0	0.786	0.122211	0.542733	0	113.5
6.86	2.78	1	0	1.297	0.855798	0.981617	0	86.2
6	7.46	1	0	1.125	0.759756	0.966419	0	93.4
5.36	5.63	1	0	0.01	1E-17	3.97E-16	0	200

5.14	5.84	1	0	5.175	2.59E-06	7.57E-05	0	32.4
7.59	2.36	1	0	0.814	0.198884	0.677056	0	94
7.01	9.9	1	0	0.749	0.083114	0.440653	0	114.2
7.84	9.86	1	0	1.41	0.578983	0.920235	0	95.9
6.86	3.36	1	0	1.572	0.46776	0.896564	0	81.2
9.72	4.7	1	0	1.278	0.903631	0.987276	0	82.7
8.38	24.04	1	0	1.351	0.745941	0.964844	0	81.9
7.81	4.16	1	0	1.643	0.278487	0.764307	0	73.4
4.88	10.96	1	0	1.128	0.682992	0.951842	0	92.4
9.17	34.23	1	0	1.042	0.518097	0.90897	0	98.4
6.27	8.75	1	0	0.891	0.311119	0.798212	0	106
8.19	14.26	1	0	0.736	0.029943	0.23662	0	114.8
11.78	5.24	1	0	3.547	0.001368	0.023555	0	44
7.05	5.21	1	0	0.045	1E-17	3.97E-16	0	191.4
4.64	4.97	1	0	1.233	0.995809	0.99978	0	98.4
7.31	14.74	1	0	0.625	0.010976	0.119739	0	117.1
8.98	20.26	1	0	1.129	0.767227	0.96838	0	94.4
6.93	28.27	1	0	1.253	0.926393	0.990434	0	90
5.24	16.18	1	0	1.056	0.591723	0.924683	0	91
6.07	9.33	1	0	0.01	1E-17	3.97E-16	0	200
8.87	2.28	1	0	1.331	0.789612	0.97006	0	85.8
7.55	5.7	1	0	1.193	0.924046	0.989893	0	93.3
9.83	4.02	1	0	1.03	0.459501	0.891873	0	99.7
10.26	5.32	1	0	0.983	0.395707	0.861859	0	100.9
6.76	4.67	1	0	1.369	0.620702	0.932207	0	83.8
9.42	19.48	1	0	1.181	0.887527	0.983874	0	88.8
6.87	9.82	1	0	0.837	0.199586	0.677705	0	105.2
7.9	1.7	1	0	1.356	0.778148	0.97006	0	71.8
9.31	2.19	1	0	0.748	0.123249	0.54384	0	106.6
8.68	2.75	1	0	1.328	0.777489	0.97006	0	87.9
5.83	3.09	1	0	1.367	0.720802	0.96164	0	83.2
8.69	8.27	1	0	0.734	0.027696	0.22439	0	119.1
9.25	2.53	1	0	1.268	0.925745	0.99022	0	94
8.31	2.77	1	0	1.031	0.462027	0.893486	0	96.4
8.88	8.98	1	0	1.636	0.226065	0.702873	0	82
4.87	14.35	1	0	1.146	0.804524	0.97006	0	96.5
5.06	8.47	1	0	1.389	0.612514	0.929068	0	81.4
8.28	4.07	1	0	1.481	0.457749	0.891247	0	85.8
6.18	5.12	1	0	1.598	0.323454	0.805731	0	77
7.91	7.47	1	0	1.07	0.603237	0.925915	0	92.9
9.14	14.9	1	0	0.617	0.029259	0.233124	0	123.7
8.97	3.1	1	0	0.776	0.10845	0.512299	0	110.1
9.99	6.71	1	0	1.138	0.783903	0.97006	0	95.5
4.98	5.18	1	0	1.386	0.732338	0.962196	0	84.7
8.19	4.54	1	0	1.315	0.838035	0.977517	0	86.4

9.39	2.04	1	0	0.924	0.371387	0.846091	0	99.9
6.34	2.4	1	0	1.621	0.303467	0.79167	0	50.3
6.04	14.1	1	0	1.647	0.195569	0.672335	0	79
6.07	2.75	1	0	1.243	0.987097	0.997793	0	88.6
5.96	3.28	1	0	2.127	0.085781	0.448636	0	63.6
9.6	8.04	1	0	1.967	0.114791	0.529344	0	63
6.21	9.47	1	0	0.848	0.208453	0.688551	0	105.6
4.84	3.05	1	0	1.368	0.663268	0.946953	0	88.9
8.15	5.48	1	0	0.01	1E-17	3.97E-16	0	200
5.36	8.21	1	0	1.443	0.602793	0.925915	0	94.1
9.07	9.89	1	0	2.463	0.005582	0.072148	0	62.3
8.24	6.96	1	0	2.341	0.026012	0.216865	0	59.8
4.79	10.22	1	0	0.446	0.000379	0.007662	0	146.3
7.65	15.83	1	0	1.289	0.870921	0.982839	0	93
5.77	2.87	1	0	0.01	1E-17	3.97E-16	0	200
9.52	2.54	1	0	100	1E-17	3.97E-16	0	
6.49	7.74	1	0	1.541	0.326639	0.806293	0	72.1
4.87	8.55	1	0	1.401	0.663593	0.947055	0	87.1
10.52	4.95	1	0	0.924	0.328287	0.806293	0	113
7.39	0	1	0	1.11	0.737768	0.96406	0	94.8
7.08	4.29	1	0	1.462	0.60679	0.927253	0	86.1
9.58	3.26	1	0	1.174	0.851609	0.980769	0	88.8
7.72	6.91	1	0	0.903	0.292301	0.778625	0	100.5
10.23	2.42	1	0	4.086	0.000262	0.005389	0	39.3
9.7	11.59	1	0	0.979	0.41912	0.867334	0	98.1
8.28	4.56	1	0	0.01	1E-17	3.97E-16	0	200
9.06	11.7	1	0	2.08	0.05045	0.325702	0	61
6.77	19.58	1	0	1.176	0.887901	0.983874	0	94.1
7.85	22.2	1	0	3.919	2.96E-07	9.46E-06	0	46.9
6.14	8.46	1	0	1.75	0.175271	0.643347	0	73.3
9.95	15.95	1	0	1.084	0.60553	0.926361	0	98.3
7.75	11.32	1	0	1.123	0.754964	0.96597	0	90.6
8	11.05	1	0	1.653	0.3421	0.820067	0	78.8
5.52	8.14	1	0	1.36	0.751071	0.96537	0	79.2
6.87	2.73	1	0	0.01	1E-17	3.97E-16	0	200
9.95	2.22	1	0	0.445	0.001734	0.028761	0	137.3
8.95	2.87	1	0	1.433	0.561632	0.911955	0	79.5
9.54	20.98	1	0	1.329	0.819874	0.973778	0	84
9.66	12.35	1	0	2.562	0.001368	0.023555	0	63.1
8.06	17.94	1	0	0.859	0.127581	0.55391	0	108.7
9.06	8.21	1	0	0.548	0.001031	0.018568	0	124.8
9.79	13.18	1	0	1.532	0.407796	0.862461	0	76.5
7.3	2.3	1	0	1.113	0.747653	0.964844	0	91
6.37	2.38	1	0	1.063	0.648822	0.941124	0	92.6
8.25	2.28	1	0	1.878	0.160042	0.62319	0	56.3

6.52	2.25	1	0	0.984	0.444531	0.888233	0	98.2
6.23	5.33	1	0	1.139	0.811568	0.971105	0	92.4
5.99	8.91	1	0	1.216	0.983854	0.997735	0	89.9
5.54	17.39	1	0	1.297	0.824201	0.974465	0	86.4
8.28	2.4	1	0	1.14	0.804797	0.97006	0	88.7
9.42	2.97	1	0	1.565	0.385516	0.855981	0	93.6
6.74	5.07	1	0	1.589	0.265287	0.748477	0	78.8
6.3	5.11	1	0	1.192	0.922525	0.989893	0	103
8.02	4.17	1	0	1.4	0.546212	0.909197	0	83.3
9.16	1.95	1	0	1.782	0.195628	0.672335	0	67.5
5.26	0	1	0	0.01	1E-17	3.97E-16	0	200
5.48	47.89	1	0	1.253	0.889813	0.984865	0	88.5
5.24	2.28	1	0	0.976	0.378336	0.850346	0	92.5
6.62	7.59	1	0	2.103	0.069442	0.393608	0	64.5
6.57	4.39	1	0	1.382	0.615277	0.929812	0	84
6.74	2.46	1	0	1.237	0.99571	0.99978	0	89.1
8.92	12.02	1	0	1.122	0.746509	0.964844	0	91.4
9.85	2.73	1	0	4.459	2.28E-07	7.39E-06	0	58.2
7.77	56.21	1	0	0.541	0.001478	0.025138	0	121.9
7.44	12.74	1	0	1.321	0.728719	0.962129	0	87.3
6.64	14.56	1	0	1.607	0.423048	0.872359	0	86
5.66	9.89	1	0	1.706	0.251419	0.734036	0	74.4
11	5.51	1	0	1.494	0.525819	0.90897	0	73.6
4.5	0	1	0	0.927	0.370978	0.846091	0	107.1
6.19	9.71	1	0	1.586	0.269791	0.753413	0	73.4
5.45	18.37	1	0	0.744	0.0396	0.28165	0	114.7
6.14	5.64	1	0	1.434	0.57319	0.918743	0	86.6
6.93	3.21	1	0	2.529	0.029321	0.233324	0	56.7
9.28	29.4	1	0	0.858	0.125389	0.550237	0	101.2
8.06	8.96	1	0	1.01	0.500525	0.906872	0	98.4
7.33	15.16	1	0	1.122	0.716484	0.960634	0	102.6
6.89	7.6	1	0	2.093	0.07279	0.406003	0	63.8
7.15	7.56	1	0	1.019	0.500016	0.906872	0	98.2
9.89	5.07	1	0	1.384	0.734994	0.962383	0	103.9
8.57	2.75	1	0	1.146	0.817026	0.972536	0	90
8.7	12.99	1	0	1.277	0.839659	0.977862	0	94.5
6.28	6.49	1	0	0.894	0.280063	0.76495	0	105.6
5.88	15.98	1	0	1.035	0.488555	0.904371	0	102.5
8.76	8.56	1	0	0.377	3.64E-05	0.000896	0	145.3
5.54	0	1	0	1.072	0.662615	0.946953	0	98.1
4.37	5.1	1	0	1.29	0.856402	0.981617	0	93.3
5.91	2.33	1	0	1.277	0.844953	0.97823	0	87.8
11.77	6.35	1	0	0.824	0.084759	0.445275	0	86
6.76	7.57	1	0	0.845	0.238691	0.718542	0	108.4
8.4	7.96	1	0	0.976	0.427325	0.874762	0	115.6

7.2	17.68	1	0	1.342	0.734761	0.962304		0	91.9
4.59	2.33	1	0	0.832	0.172236	0.640909		0	95.1
5.33	10.34	1	0	0.808	0.138186	0.57968		0	106.8
6.58	5.13	1	0	0.01	1E-17	3.97E-16		0	200
5.71	2.11	1	0	1.84	0.232071	0.711304		0	65.7
6.25	5.96	1	0	0.01	1E-17	3.97E-16		0	200
6.54	4.78	1	0	1.619	0.41035	0.863065		0	73.8
8.27	2.92	1	0	1.18	0.900291	0.987276		0	90.5
5.4	10.5	1	0	0.705	0.01452	0.146355		0	114.4
9.38	8.47	1	0	1.269	0.890814	0.98528		0	87.5
7.64	2.59	1	0	1.289	0.860509	0.981617		0	89.7
8.84	8.52	1	0	0.922	0.319381	0.802373		0	113.5
6.57	0	1	0	1.505	0.552697	0.910945		0	81.4
8.51	4.23	1	0	0.817	0.203554	0.682501		0	110
4.92	2.41	1	0	1.348	0.714122	0.960068		0	86.1
6.39	5.05	1	0	1.609	0.304254	0.79167		0	69.7
7.58	2.26	1	0	1.011	0.463753	0.89433		0	101.8
5.01	5.02	1	0	1.616	0.370134	0.846091		0	75.4
7.97	12.85	1	0	1.53	0.339966	0.818435		0	101
7.69	1.84	1	0	1.278	0.891481	0.98549		0	96.5
5.99	2.23	1	0	1.018	0.548757	0.909321		0	99.1
6.2	8.24	1	0	1.155	0.846629	0.978896		0	94
8.92	10.89	1	0	0.817	0.101201	0.4965		0	110.1
5.39	22.42	1	0	1.301	0.81449	0.971737		0	85.9
9.6	3.91	1	0	1.13	0.779435	0.97006		0	93.7
8.19	5.68	1	0	1.225	0.984722	0.997735		0	92.8
8.28	4.86	1	0	0.684	0.072745	0.405935		0	106.8
9.25	2.72	1	0	1.291	0.898789	0.987004		0	85
8.18	9.86	1	0	0.712	0.034751	0.261254		0	118.4
5	6.6	1	0	0.9	0.281981	0.76495		0	105.2
7.56	20.25	1	0	1.437	0.466135	0.89585		0	93.4
5.6	2.2	1	0	2.073	0.055072	0.342708		0	65.1
6.9	0	1	0	1.273	0.917842	0.98911		0	92.8
9.39	7.99	1	0	0.942	0.252493	0.734602		0	99.1
6.13	13.3	1	0	1.317	0.745223	0.964844		0	88
5.21	7.55	1	0	2.006	0.124902	0.549593		0	63.2
10.01	24.77	1	0	1.904	0.05264	0.333512		0	76.6
7.59	5.91	1	0	0.981	0.408399	0.862461		0	101
7.03	1.68	1	0	1.899	0.104025	0.500065		0	75.4
9.63	4.7	1	0	1.261	0.939532	0.991397		0	93.1
8.62	5.55	1	0	1.072	0.654446	0.943629		0	94.9
5.58	7.37	1	0	2.14	0.040188	0.284706		0	67.3
6.6	2.65	1	0	1.551	0.447842	0.888233		0	78.4
9.19	4.42	1	0	2.879	0.002498	0.038725		0	53.3
5.5	11.54	1	0	0.725	0.023986	0.208842		0	117.9

9.29	2.14	1	0	1.427	0.667808	0.948661	0	84.3
4.88	9.12	1	0	0.995	0.378631	0.850346	0	103
6.61	23.13	1	0	0.804	0.10833	0.512115	0	110.9
9.8	5.57	1	0	1.181	0.862429	0.981617	0	91.7
6.19	13.96	1	0	1.49	0.447255	0.888233	0	80.5
5.62	4.46	1	0	1.596	0.437406	0.884721	0	53.2
9.99	3.82	1	0	1.575	0.406423	0.862461	0	84
7.99	5.31	1	0	1.148	0.830475	0.975858	0	95.5
9.48	2.57	1	0	1.196	0.938883	0.99128	0	95.1
9.63	10.56	1	0	0.812	0.158411	0.620057	0	104.2
5	8.09	1	0	1.12	0.704147	0.958927	0	102.6
5.52	3.47	1	0	2.169	0.040362	0.285617	0	76
10.08	2.46	1	0	1.482	0.482333	0.90163	0	95.3
8.46	2.31	1	0	1.383	0.651901	0.942225	0	79.3
7.34	2.32	1	0	1.266	0.908129	0.988314	0	88.7
6.37	1.85	1	0	0.714	0.061219	0.364045	0	118.3
8.28	11.26	1	0	2.921	0.002264	0.035531	0	43.8
4.78	5.84	1	0	1.162	0.838294	0.977517	0	91.9
6.52	4.4	1	0	0.604	0.028533	0.228515	0	134.9
5.68	4.77	1	0	1.211	0.995141	0.99978	0	91.8
11.56	1.87	1	0	0.01	1E-17	3.97E-16	0	200
5.38	2.43	1	0	1.453	0.508052	0.90897	0	81.5
6.58	6.26	1	0	0.01	1E-17	3.97E-16	0	200
6.96	2.43	1	0	1.163	0.867308	0.981617	0	92.5
5.97	7.68	1	0	1.636	0.223483	0.701603	0	75.9
8.44	7.7	1	0	1.202	0.944533	0.991397	0	92.9
5.33	13.75	1	0	0.876	0.235132	0.712243	0	132.7
8.4	2.22	1	0	0.89	0.176756	0.64543	0	99.5
7.2	2.32	1	0	0.856	0.210201	0.690199	0	102.7
5.76	3.64	1	0	0.01	1E-17	3.97E-16	0	200
5.66	4.28	1	0	0.156	1E-17	3.97E-16	0	177.5
6.38	2.11	1	0	1.021	0.553446	0.910977	0	96.5
6.67	1.97	1	0	1.48	0.536498	0.90897	0	62.8
7.94	1.62	1	0	1.047	0.613061	0.929068	0	102.8
6.65	4.39	1	0	1.706	0.31388	0.799862	0	74.9
8.46	5.67	1	0	1.024	0.527459	0.90897	0	101.2
5.44	3.5	1	0	0.932	0.384007	0.854992	0	107.5
6.2	2.19	1	0	1.437	0.651072	0.942212	0	76.5
9.88	17.32	1	0	0.838	0.121028	0.539718	0	105.7
6.38	6.93	1	0	1.835	0.092407	0.468508	0	74.1
6.48	34.78	1	0	1.3	0.82216	0.974318	0	87.9
7.62	17.62	1	0	1.013	0.490777	0.905656	0	96.7
8.16	2.31	1	0	2.312	0.030154	0.237556	0	61
6.74	5.13	1	0	0.852	0.196795	0.673222	0	111.2
7.06	1.98	1	0	100	1E-17	3.97E-16	0	

7.68	1.85	1	0	1.072	0.623048	0.932683	0	96.3
9.48	4.23	1	0	1.409	0.620571	0.932207	0	91.2
5.88	2.26	1	0	0.01	1E-17	3.97E-16	0	200
6.84	2.57	1	0	1.419	0.651631	0.942225	0	84.6
7.23	11.43	1	0	0.781	0.084984	0.44596	0	112.3
8.92	13.84	1	0	1.165	0.869256	0.981722	0	91.7
6.24	1.89	1	0	1.219	0.956415	0.992197	0	96.8
5.74	0	1	0	1.03	0.584697	0.922551	0	98.5
8.48	12.33	1	0	1.552	0.446122	0.888233	0	79
6.98	15.1	1	0	1.164	0.849529	0.979998	0	95.6
9.57	1.83	1	0	0.898	0.281247	0.76495	0	99.2
6.77	4.42	1	0	1.869	0.057939	0.352705	0	76.4
5.33	5.93	1	0	1.449	0.596738	0.925915	0	81.7
8.16	13.47	1	0	1.117	0.704918	0.958927	0	95.5
9.35	10.79	1	0	1.692	0.286901	0.770436	0	74.5
6.13	7.22	1	0	1.195	0.920767	0.989178	0	92
7.8	2.02	1	0	1.05	0.627397	0.932683	0	97.5
7.03	7.99	1	0	1.074	0.653022	0.942654	0	95.7
6.16	0	1	0	0.715	0.066168	0.382986	0	113.4
10.1	12.64	1	0	1.205	0.923431	0.989893	0	89.6
9.28	4.46	1	0	1.219	0.998816	0.99992	0	90.1
8.87	3.55	1	0	0.925	0.353027	0.83025	0	109.2
9.32	35.6	1	0	1.123	0.71729	0.960634	0	95.6
6.89	11.22	1	0	1.153	0.835392	0.976576	0	94.5
8.37	3.29	1	0	0.97	0.451444	0.888233	0	101.5
8.15	2.47	1	0	1.718	0.21602	0.697303	0	66.9
6.05	1.74	1	0	1.214	0.966605	0.996606	0	89.1
8.51	2.43	1	0	1.304	0.860894	0.981617	0	82.1
8.47	7.9	1	0	1.41	0.65813	0.944397	0	83
8.73	1.67	1	0	0.837	0.236195	0.713574	0	96.8
7.94	8.32	1	0	2.171	0.067929	0.388333	0	64
9.95	4.22	1	0	2.261	0.008707	0.099921	0	55.5
8.72	4.18	1	0	1.7	0.200532	0.679907	0	65
7.21	1.65	1	0	0.88	0.25407	0.735945	0	104.7
8	0	1	0	1.366	0.764963	0.967498	0	80.1
6.23	12.7	1	0	1.052	0.598916	0.925915	0	99.9
6.49	1.61	1	0	1.076	0.667263	0.948458	0	106.7
5.69	2.39	1	0	0.567	0.016335	0.158309	0	115.7
8.25	6.13	1	0	1.154	0.806069	0.97006	0	83.8
8.25	5.01	1	0	1.724	0.214537	0.694929	0	75.4
6.18	2.24	1	0	2.271	0.058943	0.355892	0	68.5
6.74	0	1	0	0.797	0.059769	0.358302	0	111.3
9.33	11.06	1	0	1.147	0.788739	0.97006	0	95
5.64	3.36	1	0	1.143	0.783552	0.97006	0	93.3
6.81	2.75	1	0	1.104	0.724755	0.96164	0	98.6

9.14	7.96	1	0	0.745	0.031828	0.245432	0	112.6
9.76	29.49	1	0	1.356	0.639865	0.937605	0	109.6
6.29	13.05	1	0	0.61	0.00953	0.106862	0	93.6
7.14	5.67	1	0	1.113	0.688618	0.952468	0	94.7
5.03	46.19	1	0	0.598	0.006732	0.082762	0	123.8
6.95	5.98	1	0	1.089	0.655891	0.943998	0	103.8
5.95	0	1	0	0.01	1E-17	3.97E-16	0	200
4.26	12.59	1	0	1.283	0.847447	0.979302	0	93.2
7.55	6.32	1	0	1.333	0.796947	0.97006	0	84.5
9.83	4.89	1	0	1.747	0.119392	0.537564	0	68
6.35	2.63	1	0	0.01	1E-17	3.97E-16	0	200
5.57	6.69	1	0	0.64	0.021547	0.19252	0	122
5.1	13.44	1	0	1.546	0.311193	0.798212	0	77.2
6.04	4.5	1	0	1.041	0.581843	0.920669	0	95.7
9.01	4.88	1	0	2.116	0.047303	0.310965	0	63.5
6.43	2.72	1	0	0.499	0.005752	0.073967	0	124.6
9.33	14.52	1	0	1.248	0.969116	0.997215	0	84.8
7.84	9.8	1	0	0.761	0.100135	0.493385	0	113.6
6.13	0	1	0	1.759	0.287117	0.770685	0	72.5
6.16	7.01	1	0	1.303	0.876572	0.983083	0	86.8
7.88	6.29	1	0	0.922	0.350143	0.828718	0	106.7
5.9	4.82	1	0	0.583	0.007941	0.093952	0	126.3
6.55	2.22	1	0	1.77	0.238539	0.718542	0	74.6
6.3	6.52	1	0	0.01	1E-17	3.97E-16	0	200
8.16	2.36	1	0	1.134	0.764742	0.967498	0	94.6
7.46	6.55	1	0	1.452	0.523255	0.90897	0	86.7
5.47	7.46	1	0	1.239	0.952774	0.991422	0	100.6
6.64	2.09	1	0	2.511	0.008594	0.099168	0	53
6.93	4.52	1	0	1.056	0.589025	0.923211	0	94
8.48	3.71	1	0	2.426	0.024246	0.208842	0	66
5.54	22.86	1	0	1.123	0.676346	0.951158	0	90.1
8.95	13.69	1	0	0.969	0.440516	0.887298	0	104.9
6.51	3.12	1	0	0.938	0.271185	0.754632	0	100.6
5.77	1.72	1	0	1.636	0.352274	0.83025	0	75.9
8.7	3.97	1	0	2.683	0.017972	0.1696	0	54.3
6.52	3.59	1	0	0.01	1E-17	3.97E-16	0	200
7.36	28.43	1	0	1.274	0.892594	0.985818	0	88.6
9.83	13.15	1	0	1.47	0.460342	0.892334	0	81
9.29	6.15	1	0	1.558	0.316683	0.800707	0	71.4
6	3.67	1	0	1.76	0.281932	0.76495	0	82.6
5.52	1.91	1	0	0.01	1E-17	3.97E-16	0	200
5.02	4.03	1	0	1.089	0.668689	0.949591	0	115.1
7.72	2.3	1	0	1.358	0.777414	0.97006	0	81
9.16	2.45	1	0	4.391	3.39E-06	9.65E-05	0	37.1
8.69	0	1	0	1.518	0.514501	0.90897	0	80.2

6.98	2.01	1	0	1.001	0.510811	0.90897	0	100
9.58	2.3	1	0	2.298	0.021943	0.195086	0	63
5.52	5.4	1	0	1.168	0.880485	0.983083	0	93.8
7.15	4.88	1	0	1.044	0.498179	0.906872	0	98.6
4.44	2.25	1	0	2.487	0.016597	0.16035	0	50.4
8.35	7.7	1	0	0.659	0.043276	0.297868	0	117
5.17	4.7	1	0	2.789	0.012877	0.133753	0	56.4
9.83	1.96	1	0	0.01	1E-17	3.97E-16	0	200
6.01	11.6	1	0	0.83	0.09523	0.477821	0	107.1
6.8	4.93	1	0	0.995	0.436524	0.884247	0	112.6
5.24	5.27	1	0	1.185	0.917874	0.98911	0	89.6
6.42	2.7	1	0	100	1E-17	3.97E-16	0	
6.51	4.39	1	0	1.243	0.98455	0.997735	0	97.2
5.43	1.71	1	0	1.122	0.678461	0.951158	0	101.6
5.21	20.2	1	0	1.575	0.30764	0.795133	0	76.7
7.91	17.66	1	0	1.391	0.581539	0.920669	0	84.6
5.86	2.1	1	0	1.739	0.219559	0.700211	0	70.3
7.08	2.63	1	0	0.615	0.004516	0.063023	0	123.8
6.29	17.17	1	0	1.372	0.690271	0.952547	0	81.5
8.47	2.11	1	0	11.741	3E-14	1.16E-12	0	15.7
7.28	1.64	1	0	0.917	0.35879	0.835303	0	106.1
6.27	3.58	1	0	1.526	0.500799	0.906872	0	82.6
8.31	1.78	1	0	0.988	0.436657	0.884344	0	100.6
7.93	13.32	1	0	1.41	0.586685	0.922744	0	87.1
7.78	2.95	1	0	0.796	0.167313	0.636322	0	118.6
8.63	2.6	1	0	0.938	0.260741	0.744831	0	101.2
8.21	5.31	1	0	1.014	0.540764	0.90897	0	92.8
8.4	0	1	0	1.128	0.785024	0.97006	0	93.7
8.78	1.76	1	0	1.325	0.782308	0.97006	0	85.2
6.4	8.19	1	0	0.9	0.244332	0.725276	0	114.1
8.53	3.54	1	0	2.993	0.001151	0.020243	0	62.2
8.88	11.58	1	0	1.573	0.419728	0.867775	0	68.7
6.96	0	1	0	1.355	0.760098	0.966419	0	88.5
6.55	0	1	0	1.179	0.889027	0.984355	0	91.3
5.64	2.03	1	0	1.206	0.964544	0.995625	0	89.6
5.16	2.07	1	0	0.545	0.011942	0.126756	0	129.4
8	5.52	1	0	1.358	0.755538	0.96597	0	83
7.65	1.6	1	0	1.482	0.531509	0.90897	0	80.3
9.92	3.12	1	0	1.536	0.414003	0.865638	0	78.9
4.92	14.14	1	0	0.456	0.000234	0.004863	0	124.8
9.61	0	1	0	1.368	0.746256	0.964844	0	87.3
5.34	8.01	1	0	0.941	0.359592	0.835994	0	103.4
5.76	2.33	1	0	1.493	0.567351	0.913335	0	79
4.89	2.44	1	0	0.01	1E-17	3.97E-16	0	200
6.83	2.14	1	0	2.612	0.02046	0.18504	0	55.4

9.48	0	1	0	1.267	0.94291	0.991397	0	94.5
5.26	0	1	0	1.554	0.482998	0.90163	0	85.1
6.65	2.13	1	0	0.986	0.450968	0.888233	0	89.7
6.77	15.07	1	0	0.967	0.398103	0.862453	0	99.4
4.81	0	1	0	0.847	0.22549	0.701603	0	105.2
8.57	12.56	1	0	0.962	0.298515	0.787841	0	107.4
7.96	0	1	0	0.827	0.222708	0.701327	0	104.8
6.19	0	1	0	1.765	0.200757	0.679938	0	72.3
5.07	2.16	1	0	1.726	0.144914	0.596922	0	85.2
8.62	24.05	1	0	0.982	0.296372	0.785454	0	101.7
9.99	3.5	1	0	2.67	0.004043	0.058012	0	54.5
6.02	11.92	1	0	1.027	0.5575	0.910977	0	98.6
5.05	6.16	1	0	0.709	0.05676	0.348053	0	131.5
4.77	3.6	1	0	1.088	0.691886	0.952547	0	95.5
7.9	8.05	1	0	1.371	0.601479	0.925915	0	85.1
6.93	6.77	1	0	1.791	0.163621	0.629529	0	79.2
5.14	1.69	1	0	1.115	0.749228	0.96537	0	98.5
7.24	0	1	0	1.085	0.685582	0.952226	0	90.3
6.25	4.04	1	0	1.703	0.147974	0.600335	0	84.2
6.05	11.65	1	0	0.922	0.318179	0.802235	0	108.3
5.94	12.34	1	0	0.01	1E-17	3.97E-16	0	200
7.52	1.69	1	0	0.754	0.118258	0.535654	0	109.2
6.16	5.77	1	0	1.356	0.754096	0.96597	0	88.4
6.25	7.5	1	0	1.28	0.867516	0.981617	0	89.4
6.61	0	1	0	1.721	0.313513	0.79976	0	66.3
7.58	2.3	1	0	2.492	0.021126	0.189429	0	57.3
8.54	1.96	1	0	0.757	0.122389	0.542733	0	127
9.47	0	1	0	1.402	0.671271	0.95015	0	83
9.26	7.06	1	0	1.008	0.419897	0.867852	0	102.4
5.17	1.68	1	0	1.016	0.54258	0.90897	0	89.2
6.35	3.97	1	0	1.243	0.992342	0.999087	0	89.2
9.88	3.91	1	0	0.01	1E-17	3.97E-16	0	200
6.52	5.18	1	0	1.439	0.607334	0.927253	0	82
8.29	9.14	1	0	1.123	0.759851	0.966419	0	95
7.77	6.79	1	0	1.19	0.909846	0.988314	0	94.6
6.2	5.28	1	0	1.173	0.883863	0.983234	0	98.9
7.62	2.37	1	0	1.17	0.864463	0.981617	0	94.8
8.32	5.67	1	0	1.234	0.975255	0.997505	0	86.7
8.51	3.34	1	0	0.884	0.246855	0.726377	0	104.8
5.53	2.35	1	0	1.451	0.531449	0.90897	0	81.6
7.9	3.3	1	0	0.992	0.500567	0.906872	0	87.8
5.47	14.15	1	0	0.775	0.103551	0.500065	0	107.9
9.82	7.1	1	0	1.182	0.898857	0.987004	0	95.5
7.17	2.04	1	0	1.264	0.954692	0.991422	0	86.7
4.84	1.74	1	0	1.08	0.64014	0.937605	0	85.6

9.39	4.94	1	0	1.112	0.744738	0.964844	0	94.7
5.38	2.16	1	0	1.556	0.451003	0.888233	0	83.3
7.56	2.5	1	0	1.105	0.725994	0.96164	0	88.4
7.9	2.27	1	0	0.794	0.129726	0.559289	0	110.6
6.44	5.01	1	0	1.144	0.799203	0.97006	0	89.2
8.53	0	1	0	1.369	0.755668	0.96597	0	82.5
6.65	5.32	1	0	2.041	0.043492	0.297889	0	65
5.58	5.96	1	0	100	1E-17	3.97E-16	0	
8.68	3.88	1	0	1.722	0.197329	0.673765	0	90.6
7.11	42.62	1	0	0.797	0.066438	0.383654	0	109.9
7.85	0	1	0	1.521	0.522278	0.90897	0	79.3
5.12	5.72	1	0	0.01	1E-17	3.97E-16	0	200
7.21	4.46	1	0	1.194	0.925554	0.99022	0	94
12.56	57.07	1	0	1.518	0.379438	0.850854	0	75.1
7.55	8.79	1	0	1.184	0.918778	0.98911	0	89.2
5.63	7.64	1	0	2.069	0.101487	0.496535	0	65.2
5.71	1.78	1	0	2.245	0.066818	0.385044	0	59.6
6.7	27.58	1	0	1.088	0.665287	0.947803	0	99.1
9.63	5.89	1	0	0.986	0.450396	0.888233	0	86.5
8.06	1.89	1	0	1.394	0.552918	0.910945	0	87.2
6.09	1.88	1	0	1.422	0.626647	0.932683	0	92
9.31	4.34	1	0	1.324	0.725723	0.96164	0	88.4
7.17	63.05	1	0	0.346	9.11E-07	2.78E-05	0	144.5
4.92	2.04	1	0	0.909	0.35273	0.83025	0	104.8
5.62	2.13	1	0	1.318	0.845563	0.978239	0	83
6.13	2.57	1	0	1.027	0.565613	0.913241	0	98.7
7.24	0	1	0	0.909	0.350596	0.828795	0	93.7
9.95	0	1	0	1.457	0.620234	0.932207	0	93.8
4.93	2.58	1	0	1.6	0.422895	0.872243	0	83.4
8.34	107.18	1	0	1.197	0.970089	0.997215	0	89.1
5.76	2.15	1	0	2.575	0.005924	0.075863	0	55.9
5.47	13.5	1	0	1.268	0.948717	0.991422	0	79.9
4.81	5.49	1	0	0.778	0.075192	0.413941	0	100.6
9.33	11.74	1	0	1.224	0.988499	0.998053	0	97.6
7.14	4.72	1	0	1.908	0.103409	0.500065	0	68.8
4.61	31.85	1	0	0.628	0.003328	0.049115	0	132.8
5.1	2.23	1	0	1.31	0.806059	0.97006	0	87.8
5.83	1.88	1	0	1.03	0.575033	0.919147	0	97.8
5.86	2.47	1	0	100	1E-17	3.97E-16	0	
8.41	9.19	1	0	0.939	0.350591	0.828795	0	105.1
8.91	6.23	1	0	1.379	0.711107	0.959398	0	84.1
5.05	1.8	1	0	1.236	0.957106	0.992381	0	89.3
7.05	2.11	1	0	1.202	0.923736	0.989893	0	90.4
7.44	4.3	1	0	0.651	0.050024	0.324416	0	121.1
8.66	2.46	1	0	1.194	0.933269	0.991048	0	83.2

6.39	2.26	1	0	1.205	0.982493	0.997735	0	97.6
9.58	7.02	1	0	1.342	0.771891	0.97006	0	83.6
7.03	1.68	1	0	1.317	0.794734	0.97006	0	88.3
7.43	21.85	1	0	0.107	1E-17	3.97E-16	0	189.2
8.6	56.33	1	0	0.756	0.037669	0.27332	0	112.5
5.14	6.87	1	0	1.144	0.811931	0.971105	0	94.7
6.93	0	1	0	1.087	0.691945	0.952547	0	95.8
5.86	9.3	1	0	1.577	0.351582	0.829488	0	77.6
8.24	4.51	1	0	1.261	0.889117	0.984355	0	88.7
7.72	0	1	0	2.447	0.032022	0.246259	0	62.6
9.95	8.95	1	0	1.385	0.605525	0.926361	0	87
6.71	2.04	1	0	1.393	0.624121	0.932683	0	80.5
6.34	0	1	0	1.272	0.934289	0.991048	0	86.8
6.16	7.2	1	0	0.668	0.02151	0.192322	0	119.9
7.31	0	1	0	0.01	1E-17	3.97E-16	0	200
8.02	1.68	1	0	1.026	0.565612	0.913241	0	91.8
5.12	6.52	1	0	1.123	0.778608	0.97006	0	98.8
8.68	9.74	1	0	0.437	3.35E-05	0.000826	0	143.6
5.72	0	1	0	1.434	0.631413	0.933951	0	81
5.24	2.33	1	0	1.047	0.588514	0.922984	0	88.6
5.9	8.94	1	0	1.147	0.812218	0.971105	0	91.1
5.59	2.68	1	0	1.358	0.714091	0.960068	0	84.8
6.76	1.69	1	0	0.847	0.247287	0.726511	0	112.8
6.21	2.16	1	0	100	1E-17	3.97E-16	0	
6.46	5.13	1	0	0.01	1E-17	3.97E-16	0	200
9.69	4.56	1	0	1.104	0.707613	0.959205	0	100.5
9.69	1.65	1	0	0.97	0.417245	0.867334	0	95.7
6.13	2.34	1	0	0.085	1E-17	3.97E-16	0	184.3
6.61	4.2	1	0	1.349	0.731092	0.962129	0	86
6.87	3.64	1	0	0.987	0.491613	0.905872	0	94.9
8.03	4.99	1	0	0.925	0.329078	0.806293	0	109.4
9.01	2.14	1	0	1.216	0.954973	0.991422	0	78.8
8.53	8.41	1	0	1.374	0.682938	0.951842	0	81.4
6.16	2.28	1	0	1.64	0.299479	0.788806	0	75.8
6.57	2.07	1	0	2.699	0.000484	0.00969	0	54
8.06	1.96	1	0	1.223	0.986539	0.997735	0	90
8.48	1.78	1	0	1.848	0.219401	0.700036	0	73.7
6.34	4.16	1	0	1.075	0.557089	0.910977	0	101.9
6.84	0	1	0	2.088	0.085921	0.448636	0	68.6
6.95	3.81	1	0	1.242	0.963814	0.995536	0	91.6
6.28	3.86	1	0	1.342	0.772734	0.97006	0	90.3
8.41	3.97	1	0	1.103	0.603588	0.925915	0	96.3
5.8	2.2	1	0	0.841	0.184007	0.655884	0	105.2
9.77	2.31	1	0	1.583	0.449201	0.888233	0	77.3
9.67	1.8	1	0	0.91	0.21232	0.692641	0	104.7

9.35	2.43	1	0	0.863	0.205896	0.684452	0	107.4
6.33	18.55	1	0	0.894	0.179202	0.64734	0	103.4
5.96	3.56	1	0	1.035	0.587479	0.922744	0	102.4
7.96	4.82	1	0	4.431	3.53E-08	1.17E-06	0	20.2
5.86	2.98	1	0	1.269	0.939036	0.99128	0	90.6
5.02	10.24	1	0	0.605	0.007714	0.091615	0	114
8.25	11.42	1	0	0.864	0.155708	0.616223	0	107.8
10.77	1.78	1	0	1.087	0.650702	0.942006	0	93.3
5.31	2.23	1	0	0.492	0.003384	0.049772	0	112.1
6.28	5.65	1	0	2.194	0.043959	0.299299	0	44.8
8.05	4.2	1	0	0.699	0.024332	0.208842	0	137
6.23	1.78	1	0	1.819	0.21998	0.700211	0	54.9
6.58	2.29	1	0	0.716	0.093535	0.471951	0	111.6
5.86	2.12	1	0	1.296	0.845595	0.978239	0	94.5
6.67	2.22	1	0	0.751	0.127092	0.552662	0	114.2
9.31	0	1	0	1.146	0.81432	0.971737	0	92.9
5.15	1.87	1	0	2.415	0.004413	0.061656	0	52
5	2.36	1	0	0.921	0.318512	0.802274	0	104.1
8	2.13	1	0	1.63	0.398271	0.862453	0	76
7.88	0	1	0	0.01	1E-17	3.97E-16	0	200
8.03	4.74	1	0	1.34	0.744432	0.964801	0	85.5
5.08	13.31	1	0	1.267	0.873162	0.983083	0	89.8
10.17	2.59	1	0	1.195	0.911166	0.988683	0	91.1
9.16	3.61	1	0	1.385	0.578301	0.919803	0	84.3
7.15	9.57	1	0	0.962	0.386246	0.85682	0	97.7
9.89	4.57	1	0	1.572	0.371288	0.846091	0	80.4
6.23	2.53	1	0	0.706	0.08395	0.443492	0	121.9
9.58	1.76	1	0	1.185	0.915649	0.98911	0	91.2
6.02	0	1	0	1.697	0.337547	0.815932	0	76
8.27	3.95	1	0	1.033	0.550892	0.910041	0	96.9
7.05	2.54	1	0	1.516	0.382096	0.853306	0	77.9
11	7.14	1	0	2.374	0.00767	0.091172	0	61.7
5.31	3.11	1	0	0.724	0.100855	0.49538	0	116
7.34	9.84	1	0	1.052	0.468135	0.896668	0	97.5
8.7	7.41	1	0	0.964	0.306688	0.794751	0	102.5
9.25	21.92	1	0	1.572	0.313998	0.799862	0	73.6
7.49	0	1	0	0.637	0.043931	0.299299	0	113.8
5.55	6.86	1	0	1.187	0.912111	0.989005	0	90.4
6.87	1.64	1	0	1.242	0.999019	0.99992	0	85.4
6.64	4.3	1	0	0.933	0.38609	0.856625	0	103.5
9.79	12.6	1	0	4.857	1.7E-06	5.04E-05	0	41.5
8.9	4.15	1	0	0.306	5.65E-07	1.77E-05	0	156.9
6.71	3.87	1	0	0.238	1.59E-11	5.83E-10	0	161.6
10.04	11.46	1	0	1.025	0.532505	0.90897	0	100.8
9.04	51.08	1	0	1.264	0.918051	0.98911	0	98.3

7.93	2.35	1	0	5.089	8.43E-07	2.6E-05	0	32.8
5.2	1.89	1	0	1.313	0.836016	0.976616	0	89.6
11.43	1.87	1	0	1.167	0.876431	0.983083	0	94.8
6.99	1.77	1	0	0.625	0.016693	0.160905	0	139.3
6.54	5.59	1	0	1.344	0.805251	0.97006	0	88.9
8.07	1.65	1	0	2.1	0.08693	0.451468	0	64.5
5.03	11.62	1	0	1.8	0.091534	0.466715	0	71
8.13	7.37	1	0	1.49	0.550056	0.909434	0	80.3
7.2	4.39	1	0	0.068	1E-17	3.97E-16	0	187.2
8.78	1.81	1	0	0.01	1E-17	3.97E-16	0	200
7.64	1.81	1	0	1.426	0.532796	0.90897	0	89.2
8.9	2.5	1	0	0.994	0.466102	0.89585	0	106.3
7.05	35.42	1	0	1.29	0.801541	0.97006	0	84.9
7.46	6.65	1	0	0.943	0.364631	0.841928	0	92
4.67	2.37	1	0	1.414	0.58789	0.922926	0	102.5
6.61	2.01	1	0	1.183	0.885046	0.983234	0	91.6
10.62	0	1	0	100	1E-17	3.97E-16	0	
7.91	2.47	1	0	0.971	0.312773	0.798743	0	100.1
9.31	3.79	1	0	0.66	0.007117	0.086567	0	120.5
9.67	1.78	1	0	1.061	0.619722	0.932207	0	107
8.62	1.75	1	0	2.369	0.045695	0.303974	0	46.1
9.22	12.58	1	0	1.438	0.605103	0.926158	0	83.4
5.67	0	1	0	0.954	0.312931	0.798743	0	98.2
7.5	12.23	1	0	0.997	0.458909	0.891873	0	103.1
7.17	2.82	1	0	0.844	0.248158	0.728205	0	105.2
5.4	0	1	0	1.618	0.400874	0.862461	0	76.4
9.25	29.72	1	0	1.29	0.875355	0.983083	0	92.4
8.54	0	1	0	0.992	0.497115	0.906872	0	87.8
8.6	2.21	1	0	1.552	0.388895	0.859423	0	74.3
5.68	13.86	1	0	1.009	0.480555	0.899427	0	97.2
8.34	2.09	1	0	1.923	0.177136	0.64543	0	69.2
6.84	1.85	1	0	1.897	0.050536	0.326089	0	73.3
9.33	9.12	1	0	0.69	0.011857	0.126756	0	90.7
9.28	1.74	1	0	0.602	0.009869	0.11005	0	126.9
8.54	0	1	0	0.837	0.09757	0.483748	0	92.5
5.55	0	1	0	1.473	0.596088	0.925915	0	84.1
9.72	7.13	1	0	1.419	0.517888	0.90897	0	78.9
6.16	2.08	1	0	1.467	0.600358	0.925915	0	78.5
7.69	1.94	1	0	0.794	0.052221	0.331488	0	115.7
9.45	0	1	0	0.668	0.029676	0.235647	0	113.4
10.86	4.67	1	0	0.22	2.26E-09	7.95E-08	0	164
9.76	5.4	1	0	0.789	0.165608	0.63388	0	115.2
5.07	2.02	1	0	1.567	0.469409	0.896668	0	86
5.26	23.67	1	0	1.04	0.491568	0.905872	0	103.7
7.99	2.23	1	0	1.491	0.521026	0.90897	0	111.6

4.59	3	1	0	1.231	0.996237	0.99992	0	85.2
6.46	5.62	1	0	1.127	0.779566	0.97006	0	99.2
7.94	2.23	1	0	1.326	0.763435	0.967182	0	86
10.23	2.35	1	0	1.381	0.713691	0.960068	0	82.4
8.03	9.15	1	0	0.965	0.388923	0.859423	0	104.4
8.06	3.94	1	0	1.558	0.369786	0.846091	0	76.3
5.2	0	1	0	1.082	0.639682	0.937605	0	96.4
8.87	1.75	1	0	1.554	0.384316	0.854992	0	73.4
8.76	0	1	0	0.857	0.26389	0.748141	0	116.2
6.55	1.74	1	0	1.111	0.743035	0.964372	0	92.7
6.64	2.1	1	0	0.01	1E-17	3.97E-16	0	200
8.35	0	1	0	1.339	0.808796	0.970261	0	91.1
5.52	6.69	1	0	0.559	0.000636	0.01228	0	138.1
6.83	1.66	1	0	1.318	0.846912	0.978953	0	82.5
8.16	13.63	1	0	0.01	1E-17	3.97E-16	0	199.9
9.11	2.34	1	0	1.351	0.768008	0.968389	0	85.1
6.58	3.15	1	0	0.852	0.254214	0.735945	0	108
7.37	2.19	1	0	0.01	1E-17	3.97E-16	0	200
9.86	1.95	1	0	0.01	1E-17	3.97E-16	0	200
8	4.15	1	0	0.739	0.053178	0.334226	0	113.5
6.48	2.24	1	0	1.62	0.416096	0.866217	0	69.6
5	5.36	1	0	1.925	0.083213	0.440653	0	62.1
6.51	1.68	1	0	0.98	0.474148	0.898019	0	113.2
11.27	9.51	1	0	1.012	0.41296	0.864761	0	107.6
6.19	2.1	1	0	1.086	0.683205	0.951842	0	93.5
6.76	2.57	1	0	0.01	1E-17	3.97E-16	0	200
5.12	0	1	0	1.568	0.47739	0.898527	0	76.2
8.56	2.28	1	0	0.966	0.449217	0.888233	0	108.9
8.94	14.02	1	0	1.063	0.548715	0.909321	0	98.3
9.1	4.01	1	0	0.834	0.113026	0.525241	0	109
6.65	0	1	0	0.989	0.49659	0.906872	0	101.4
10.14	3.67	1	0	0.788	0.070782	0.398693	0	100.2
7.47	15.89	1	0	0.649	0.016107	0.1567	0	115.5
6.38	2.35	1	0	0.76	0.095932	0.479242	0	113.7
5.92	2.08	1	0	1.668	0.364686	0.841928	0	72.6
8.78	3.06	1	0	0.761	0.086146	0.449437	0	113.6
5.68	5.47	1	0	1.252	0.946543	0.991422	0	88.8
9.35	2.04	1	0	0.607	0.02996	0.23662	0	125.1
9.26	1.77	1	0	1.357	0.743064	0.964372	0	83.5
4.5	2.19	1	0	1.746	0.164044	0.630581	0	84.9
7.93	14.09	1	0	1.407	0.558146	0.910977	0	81.6
7.96	0	1	0	2.371	0.012405	0.130027	0	82.2
8.37	3.09	1	0	0.469	5.02E-05	0.001213	0	136.1
5.64	0	1	0	1.047	0.60745	0.927253	0	82.5
8.02	5.36	1	0	0.525	0.000525	0.010302	0	144.6

6.28	2.32	1	0	0.821	0.076418	0.417728	0	92.9
7.39	1.64	1	0	1.218	0.986561	0.997735	0	89.8
9.45	15.62	1	0	1.181	0.881119	0.983083	0	90.9
5.44	2.07	1	0	1.065	0.63583	0.937143	0	104.3
8.47	2.48	1	0	1.283	0.865933	0.981617	0	73.7
8.15	0	1	0	1.462	0.574939	0.919147	0	81.3
7.85	6.64	1	0	1.294	0.836771	0.976711	0	87.2
7.21	3.57	1	0	2.244	0.026384	0.219236	0	81.9
5.59	1.76	1	0	1.435	0.652106	0.942414	0	82.7
8.66	1.69	1	0	0.704	0.028336	0.227574	0	124.4
4.94	5.24	1	0	1.347	0.682677	0.951842	0	89.3
7.37	5.19	1	0	1.025	0.51671	0.90897	0	101.4
8.78	0	1	0	1.141	0.793154	0.97006	0	94.1
6.24	0	1	0	1.171	0.879836	0.983083	0	91.4
4.98	6.43	1	0	1.251	0.913691	0.98911	0	93.9
6.73	3.9	1	0	0.893	0.262104	0.746056	0	105.6
6.8	1.93	1	0	1.39	0.689156	0.952468	0	73.4
6.38	1.69	1	0	1.602	0.315483	0.800259	0	80.9
6.52	1.91	1	0	0.957	0.391625	0.861129	0	107.9
8.05	2.47	1	0	1.149	0.827727	0.974887	0	93.1
4.92	8.04	1	0	1.951	0.052952	0.334142	0	75.3
6.18	3.79	1	0	1.368	0.760213	0.966419	0	81
7.08	2.06	1	0	0.86	0.239127	0.718542	0	98.8
7.34	1.77	1	0	1.096	0.611427	0.929068	0	91.4
6.77	1.68	1	0	1.636	0.282732	0.76495	0	73.4
7.17	0	1	0	0.87	0.283836	0.766046	0	113.9
8.32	3.71	1	0	0.898	0.238938	0.718542	0	103.9
5.1	6.28	1	0	0.5	0.001703	0.028401	0	132.5
6.55	7.58	1	0	1.732	0.209147	0.689434	0	70.2
9.48	2.33	1	0	1.306	0.789864	0.97006	0	95
7.15	2.35	1	0	0.01	1E-17	3.97E-16	0	200
6.74	0	1	0	0.962	0.43955	0.886779	0	102.8
10.05	4.25	1	0	1.245	0.985773	0.997735	0	92.1
8.05	2.81	1	0	0.709	0.017912	0.16916	0	109
5.25	1.72	1	0	1.306	0.86854	0.981617	0	82.4
8.46	2.05	1	0	2.246	0.047528	0.311955	0	60.3
8.59	2.37	1	0	0.901	0.279165	0.76495	0	102.9
7.43	5.33	1	0	0.74	0.049081	0.319978	0	118.8
6.2	1.85	1	0	2.043	0.126568	0.551751	0	69.1
4.73	2.01	1	0	0.889	0.302426	0.79167	0	109
8.18	1.84	1	0	1.349	0.700295	0.957985	0	74.8
8.41	2.18	1	0	1.38	0.742887	0.964372	0	89.5
7.14	2.22	1	0	0.01	1E-17	3.97E-16	0	200
6.39	0	1	0	1.311	0.859966	0.981617	0	84.3
6.65	0	1	0	0.715	0.091985	0.46769	0	115.9

8.07	0	1	0	0.853	0.177647	0.645798	0	108.4
6.25	2.65	1	0	1.129	0.789075	0.97006	0	92.9
6.98	1.89	1	0	0.817	0.159847	0.622621	0	110.1
8.66	3.6	1	0	1.466	0.605805	0.926444	0	78.9
4.88	14.31	1	0	0.906	0.187227	0.662699	0	96.3
6.67	0	1	0	1.321	0.739973	0.96414	0	87.1
7.77	0	1	0	1.021	0.563188	0.912951	0	102.1
8.12	0	1	0	0.01	1E-17	3.97E-16	0	200
7.06	0	1	0	1.184	0.907562	0.988314	0	91.7
6.6	4.22	1	0	1.509	0.340818	0.818809	0	82.6
5.31	2.04	1	0	1.459	0.595482	0.925915	0	77.3
7.23	24.49	1	0	1.559	0.356755	0.833315	0	82.8
8.25	4.13	1	0	0.99	0.493649	0.906872	0	102.6
7.52	2.15	1	0	1.116	0.751121	0.96537	0	91.2
11.81	5.36	1	0	0.761	0.039219	0.280531	0	113.6
5.69	3.51	1	0	1.253	0.9304	0.99077	0	88.7
6.28	2.58	1	0	0.944	0.348106	0.82609	0	115.1
5.53	1.82	1	0	0.767	0.106626	0.507493	0	113.7
6.8	4.53	1	0	1.69	0.177061	0.64543	0	72.2
5.82	1.63	1	0	1.035	0.518437	0.90897	0	98.9
6.39	5.19	1	0	1.444	0.60207	0.925915	0	83.8
7.42	43.96	1	0	1.418	0.585518	0.922744	0	82.7
4.67	13.5	1	0	0.776	0.111527	0.521551	0	109.9
5.53	10.13	1	0	0.914	0.311035	0.798212	0	102.1
12.43	2.07	1	0	0.01	1E-17	3.97E-16	0	200
5.74	9.4	1	0	1.236	0.952628	0.991422	0	92.1
8.79	0	1	0	1.231	0.988079	0.998053	0	84.9
9.67	13.52	1	0	1.125	0.757923	0.966419	0	88
5.74	3.73	1	0	0.581	0.001206	0.021107	0	124.4
7.24	2.06	1	0	1.201	0.929081	0.99077	0	90
8.91	1.71	1	0	1.526	0.430618	0.879393	0	84.2
9.04	2.26	1	0	1.078	0.674121	0.951158	0	99
9	3.67	1	0	0.845	0.183616	0.655756	0	105.7
7.02	4.8	1	0	1.044	0.57564	0.919147	0	104.7
6.4	2.44	1	0	1.254	0.971039	0.997499	0	98.5
10.05	3.33	1	0	1.309	0.827829	0.974887	0	85.9
7.14	1.69	1	0	1.108	0.731471	0.962196	0	96.7
5.8	1.65	1	0	1.168	0.852221	0.981305	0	92
4.88	11.81	1	0	0.711	0.040907	0.28818	0	117.7
4.81	3.54	1	0	1.299	0.86711	0.981617	0	80.9
5.8	0	1	0	2.529	0.028315	0.227574	0	56.7
8.68	1.95	1	0	0.01	1E-17	3.97E-16	0	200
5.17	1.72	1	0	1.199	0.953094	0.991422	0	91
6.42	2.35	1	0	1.012	0.409634	0.862461	0	93.9
5.17	2.5	1	0	1.059	0.541457	0.90897	0	93.1

7.47	7.32	1	0	1.148	0.82449	0.974465	0	99
6.89	0	1	0	1.612	0.402683	0.862461	0	76.6
6.7	2.19	1	0	1.46	0.518876	0.90897	0	85.6
7.77	5.17	1	0	0.789	0.067126	0.386072	0	122.7
4.78	4.57	1	0	0.785	0.051815	0.330448	0	105.5
10.05	3.46	1	0	0.778	0.109012	0.514374	0	116.4
8.37	1.97	1	0	0.945	0.393415	0.861859	0	102.8
5.08	2.8	1	0	100	1E-17	3.97E-16	0	
8.56	4.54	1	0	0.736	0.081943	0.439346	0	137
5.67	4.18	1	0	0.982	0.477562	0.898527	0	102.1
7.75	2.13	1	0	0.01	1E-17	3.97E-16	0	200
8.43	4.18	1	0	1.004	0.400418	0.862461	0	98.5
5.97	5.02	1	0	1.398	0.629046	0.932971	0	92.3
7.24	2.5	1	0	0.574	0.00479	0.065115	0	127.1
11.27	24.36	1	0	0.08	1E-17	3.97E-16	0	182.6
8.65	2.23	1	0	1.113	0.72867	0.962129	0	99.3
7.9	4.11	1	0	1.054	0.584109	0.922513	0	96.2
6.6	2.45	1	0	1.014	0.541518	0.90897	0	99.3
7.12	0	1	0	1.287	0.852593	0.981581	0	88.2
7.02	1.75	1	0	0.957	0.388071	0.859423	0	103.1
9.44	2.56	1	0	2.171	0.077154	0.421202	0	62.1
10.04	2.31	1	0	1.098	0.701656	0.958482	0	96.4
6.9	2.96	1	0	0.676	0.009866	0.11005	0	118.5
5.16	0	1	0	0.01	1E-17	3.97E-16	0	200
8.87	7.64	1	0	1.43	0.492816	0.906679	0	79.1
8.32	1.79	1	0	1.407	0.526856	0.90897	0	85.3
7.44	0	1	0	1.093	0.70747	0.959114	0	95.5
6.05	2.08	1	0	1.508	0.548278	0.909321	0	76
8.41	17.13	1	0	0.872	0.159574	0.622135	0	121.8
6.98	2.49	1	0	0.879	0.160645	0.623978	0	107.5
8.69	1.92	1	0	1.894	0.125715	0.550237	0	90.8
5.8	0	1	0	0.353	6.97E-06	0.00019	0	165.7
8.79	7.38	1	0	1.46	0.523977	0.90897	0	77.2
7.52	5.8	1	0	0.01	1E-17	3.97E-16	0	200
5.67	2.84	1	0	2.346	0.050866	0.327386	0	59.8
6.44	0	1	0	0.921	0.372298	0.84692	0	104.1
8.97	2.12	1	0	1.14	0.789485	0.97006	0	95.5
6.71	0	1	0	0.01	1E-17	3.97E-16	0	200
9.5	4.73	1	0	1.706	0.32402	0.805731	0	76.1
5.21	4.86	1	0	0.526	0.000151	0.003298	0	157.1
5.97	3.71	1	0	0.533	0.000713	0.013537	0	156.3
9.06	9.35	1	0	1.102	0.694968	0.955203	0	97.7
5.34	8.17	1	0	0.609	0.002199	0.034741	0	93.6
6.73	3.91	1	0	0.982	0.416048	0.866217	0	99.3
8.91	6.78	1	0	0.967	0.309842	0.796853	0	108

6.67	0	1	0	1.308	0.866429	0.981617	0	86.1
6.61	3.91	1	0	0.975	0.395882	0.861859	0	104.2
7.36	2.9	1	0	0.01	1E-17	3.97E-16	0	200
8.03	0	1	0	1.566	0.47365	0.898019	0	81.9
6.77	2.05	1	0	1.429	0.638937	0.937605	0	82.4
5.87	0	1	0	1.316	0.779136	0.97006	0	86.1
8.92	0	1	0	1.506	0.550183	0.909434	0	81
9.11	2.18	1	0	1.529	0.519035	0.90897	0	76.9
5.41	5.73	1	0	1.11	0.724493	0.96164	0	96.8
9.58	1.78	1	0	0.707	0.031162	0.242703	0	111.2
9.44	2.64	1	0	2.075	0.048504	0.317367	0	83.3
8.51	3.41	1	0	2.608	0.00303	0.045999	0	50.4
8.12	13.72	1	0	1.134	0.786225	0.97006	0	91.1
7.23	5.32	1	0	1.375	0.615457	0.929812	0	85.2
7.78	1.84	1	0	3.373	9.01E-05	0.00212	0	45.2
5.76	1.69	1	0	1.173	0.875651	0.983083	0	91
8.88	5.66	1	0	0.01	1E-17	3.97E-16	0	200
10.54	2.26	1	0	100	1E-17	3.97E-16	0	
8.53	4.02	1	0	1.01	0.489815	0.905208	0	103.1
8.78	2.33	1	0	0.887	0.307437	0.795133	0	100.3
6.35	1.92	1	0	1.542	0.384482	0.854992	0	76.3
5.85	4.63	1	0	1.011	0.506193	0.90897	0	100.2
6.64	1.6	1	0	1.707	0.32251	0.80499	0	74.8
5.21	2.7	1	0	0.01	1E-17	3.97E-16	0	200
8	2.34	1	0	0.895	0.246859	0.726377	0	100.4
7.3	3.4	1	0	1.367	0.697168	0.956785	0	84.5
6.43	1.98	1	0	0.802	0.137575	0.578504	0	107.2
8.72	2.12	1	0	2.232	0.008109	0.094833	0	54.2
5.81	0	1	0	1.471	0.602992	0.925915	0	82.9
6.34	1.8	1	0	1.194	0.912118	0.989005	0	90.8
8.16	0	1	0	0.01	1E-17	3.97E-16	0	200
6.81	1.68	1	0	8.961	2.86E-13	1.09E-11	0	22.1
4.93	1.65	1	0	2.014	0.137158	0.578504	0	62.1
8.16	0	1	0	1.452	0.472749	0.898019	0	93.5
6.13	1.7	1	0	2.003	0.142575	0.593057	0	59.1
6.09	6.81	1	0	1.011	0.407556	0.862461	0	101.7
4.86	4.56	1	0	0.971	0.416147	0.866217	0	136.6
6.04	8.95	1	0	0.931	0.239254	0.718542	0	97.6
5.38	1.63	1	0	1.46	0.534992	0.90897	0	86.6
5.63	2.93	1	0	1.059	0.637174	0.937605	0	99.4
8.79	0	1	0	2.017	0.076298	0.417553	0	65.1
7.27	2.44	1	0	0.813	0.150193	0.604664	0	110.3
6.05	2.59	1	0	0.94	0.395848	0.861859	0	111.3
9.5	2.2	1	0	1.659	0.314746	0.800259	0	75.2
8.12	0	1	0	1.067	0.631965	0.934548	0	96.8

5.01	5.59	1	0	1.185	0.918367	0.98911	0	93.8
6.05	1.95	1	0	0.803	0.062425	0.368436	0	107.9
7.05	2.67	1	0	0.927	0.199184	0.677163	0	104.9
6.42	1.74	1	0	1.111	0.733805	0.962304	0	98.4
7.36	1.91	1	0	0.726	0.049702	0.323358	0	114.4
6.19	2.15	1	0	0.735	0.048818	0.319091	0	114.7

Abundanc	Found in S	Found in S	Found in S	Found in S	Found in S	Found in S	Found in S	Found in S
96.4	High	High	High	High	High	High	High	High
87.3	High	High	High	High	High	High	High	High
142.2	High	High	High	High	High	High	High	High
99.5	High	High	High	High	High	High	High	High
121.3	High	High	High	High	Peak Foun	High	High	High
73.4	High	High	High	High	Not Found	High	Peak Foun	High
90	High	High	High	High	Not Found	Not Found	High	High
104.5	High	High	High	High	Peak Foun	High	High	High
104.3	High	High	High	High	High	High	High	High
117.2	High	High	High	High	High	High	High	High
134.8	High	High	High	High	High	High	High	High
122.3	High	High	Peak Foun	High	High	High	High	Peak Foun
128	High	High	High	High	High	High	High	High
126.8	High	High	High	High	High	High	High	High
89	High	High	High	High	High	High	High	High
111	High	High	High	High	High	High	High	High
130.4	High	High	High	High	High	High	High	High
104.5	High	High	High	High	High	High	High	High
111.2	High	High	High	High	High	High	High	High
162.3	High	High	High	High	High	High	High	High
98.9	High	High	High	High	Peak Foun	High	High	High
105.1	High	High	High	High	High	High	High	High
105.4	Peak Foun	High	High	High	Not Found	Peak Foun	High	High
107.1	High	High	High	High	High	High	High	High
100.3	High	High	High	High	High	High	High	High
157.8	High	High	High	High	High	High	High	High
164.4	High	High	High	High	Peak Foun	Peak Foun	High	High
117.5	High	High	High	High	Not Found	Not Found	Peak Foun	Peak Foun
100.2	High	High	High	High	High	High	High	High
123	High	High	High	High	High	High	High	High
113.9	High	High	High	Peak Foun	Peak Foun	High	Peak Foun	Peak Foun
86.2	High	High	High	High	High	High	High	High
95.1	High	High	High	High	Peak Foun	High	High	High
137.4	High	High	High	High	High	High	High	High
133.6	High	High	High	High	High	High	High	High
91.2	High	High	High	High	High	High	High	High
122.2	High	High	High	High	High	High	High	High
125.8	High	High	High	High	High	High	High	High
189.8	High	High	High	High	High	Peak Foun	Peak Foun	High
125.6	High	High	High	High	High	High	High	High
109	High	High	High	High	High	High	High	High
109.9	High	High	High	High	High	High	High	High

104	High	High	High	High	High	High	High	High
101.4	High	High	High	High	Peak Foun	High	High	High
85.4	High	High	High	High	Peak Foun	High	High	High
143	High	High	High	High	High	High	High	High
98.4	High	High	High	High	High	High	High	High
131.6	High	High	High	High	High	High	High	High
94.6	High	High	High	Peak Foun	Not Found	High	High	High
108.8	High	High	High	High	High	High	High	High
133.4	High	High	High	High	High	High	High	High
87.6	High	High	High	High	High	High	High	High
181.6	High	High	High	High	High	High	High	High
138.3	High	High	High	High	High	High	High	High
112.1	High	High	High	High	High	High	High	High
101.9	High	High	High	High	High	High	High	High
129.4	High	High	High	High	High	High	High	High
99.5	High	Peak Foun	High	Peak Foun	High	Peak Foun	High	Peak Foun
116.6	High	High	High	High	High	High	High	High
107.7	Peak Foun	High	Peak Foun	Peak Foun	Peak Foun	High	Peak Foun	Peak Foun
135.7	High	High	High	High	High	High	High	High
100	High	High	High	High	High	High	High	High
93	High	High	High	High	High	High	High	High
70.8	High	High	High	High	High	High	High	High
164.1	High	High	High	High	Peak Foun	High	High	High
93.1	High	High	High	High	High	High	High	High
91.5	High	High	High	High	High	High	High	High
115.8	High	High	High	High	Peak Foun	High	High	High
100.6	High	High	High	High	High	High	High	High
133.1	High	High	High	High	High	High	High	High
118.6	High	High	High	High	High	High	High	High
100	High	High	High	High	High	High	High	High
91.7	High	High	High	High	High	High	High	High
99.6	High	High	High	High	High	High	High	High
117.8	High	High	High	High	High	High	High	High
102	High	High	High	High	High	High	High	High
100.3	Not Found	Not Found	Not Found	Not Found	Not Found	Not Found	Not Found	Not Found
194.5	High	High	High	High	Peak Foun	Peak Foun	Peak Foun	Peak Foun
194.6	High	High	High	High	Peak Foun	Peak Foun	Peak Foun	Peak Foun
101.4	High	High	High	High	High	High	High	High
121.4	High	High	High	High	High	High	High	High
99.5	High	High	High	High	High	High	High	High
109.9	Not Found	Not Found	High	Peak Foun	Not Found	High	High	Peak Foun
101.8	High	High	High	High	High	High	High	High
108.1	High	High	High	High	High	High	High	High
100.6	High	High	High	High	High	High	High	High
112.9	High	High	High	High	High	High	High	High

103.8	High	High	High	High	High	High	High	High
109.5	High	High	High	High	Peak Foun	Peak Foun	Peak Foun	High
94	High	High	High	High	High	High	High	High
111.4	High	High	High	High	High	High	High	High
111.5	High	Peak Foun	Peak Foun	Peak Foun	Peak Foun	Peak Foun	Peak Foun	Peak Foun
104.5	High	High	High	High	High	High	High	High
97.7	High	High	High	High	High	Peak Foun	High	Peak Foun
139.5	High	High	High	High	High	High	High	High
97.7	High	High	High	High	High	High	High	High
194.1	High	High	High	High	Peak Foun	Peak Foun	Peak Foun	Peak Foun
96.1	High	High	High	High	High	High	High	High
117.7	High	High	High	High	High	High	High	High
122.1	High	High	High	High	High	High	High	High
106.7	Peak Foun	Peak Foun	Peak Foun	Peak Foun	Not Found	High	Peak Foun	Peak Foun
104.1	High	High	High	High	High	High	High	High
100.9	High	High	High	High	Peak Foun	High	High	High
100.7	High	High	High	High	High	High	High	High
95.5	High	High	High	High	High	High	High	High
124.9	High	High	High	High	High	High	High	High
91.5	High	High	High	High	High	High	High	High
89.3	High	High	High	High	High	High	High	High
98	Not Found	Not Found	Not Found	Peak Foun	Not Found	Not Found	High	Peak Foun
122.1	High	High	High	High	High	High	High	High
98.2	High	High	High	High	High	High	High	High
110.8	High	High	High	High	High	High	High	High
121.1	High	High	High	High	High	High	High	High
89	High	High	High	High	High	High	High	High
131.2	High	High	High	High	High	High	High	High
108.6	High	High	High	High	High	High	High	High
99	High	High	High	High	High	High	Peak Foun	High
110.1	High	High	High	High	High	High	High	High
102.1	High	High	High	High	High	High	High	High
109.9	High	High	High	High	High	High	High	High
139.4	High	High	High	High	High	High	High	High
89.8	High	High	High	High	High	High	High	High
169.7	High	High	High	High	Peak Foun	Peak Foun	Peak Foun	High
144	High	High	High	High	High	High	High	High
100.3	High	High	High	High	High	High	High	High
124	High	High	High	High	High	High	High	High
98.2	High	High	High	High	High	High	High	High
102.2	High	High	High	High	High	High	High	High
113.7	High	High	High	High	High	High	High	High
113	High	High	High	High	High	High	High	High
119.8	High	High	High	High	High	High	High	High
104.9	High	High	High	Peak Foun	High	High	High	High

122.8	High	High	High	High	High	High	High	High
144.9	High	High	High	High	Peak Foun	High	High	High
70.2	High	High	High	High	High	High	High	High
85	Not Found	Not Found	Not Found	Not Found	Not Found	Not Found	Not Found	Not Found
128.6	High	High	High	High	High	High	High	High
99.5	High	High	High	High	High	High	High	High
100.5	High	High	High	High	High	High	High	High
97.2	High	High	High	High	High	High	High	High
106.2	High	High	High	High	High	High	High	High
135.6	High	High	High	High	High	High	High	High
114.6	High	High	High	High	High	High	High	High
107.4	High	High	High	High	High	High	High	High
107	High	High	High	High	High	High	High	High
100.7	High	High	High	High	Peak Foun	High	High	High
91.1	High	High	High	High	High	High	High	High
117.7	High	High	High	High	High	High	High	High
102.5	High	High	High	High	High	High	High	High
188.6	Not Found	Not Found	Not Found	Not Found	Not Found	Not Found	Not Found	Not Found
113.3	High	High	High	High	High	High	High	High
98	High	High	High	High	High	High	High	High
123.6	High	High	High	High	High	High	High	High
130.5	High	High	High	High	High	High	High	High
112.2	High	High	High	High	High	High	High	High
170.4	High	High	High	High	Peak Foun	Not Found	High	High
128.6	High	High	High	High	High	High	High	High
143.4	High	High	High	High	Peak Foun	Peak Foun	High	High
80.1	High	High	High	High	High	High	High	High
148.9	High	High	High	High	Peak Foun	Peak Foun	High	High
120.9	High	High	High	High	High	High	High	High
106.5	High	High	High	High	High	High	High	High
198.4	Not Found	Not Found	Not Found	Not Found	Not Found	Not Found	Not Found	Not Found
100.5	High	High	High	High	High	High	High	High
141.1	High	High	High	High	High	High	High	High
113.8	High	High	High	High	High	High	High	High
7.7	Peak Foun	Not Found	Peak Foun	Not Found	Peak Foun	High	High	Peak Foun
95.9	High	High	High	High	High	High	High	High
108.6	High	High	High	High	High	High	High	High
133.1	High	High	High	High	High	High	High	High
101.7	High	High	High	High	High	High	High	High
106.6	High	High	High	High	High	High	High	High
115.2	High	High	High	High	High	High	High	High
129.7	High	High	High	High	High	High	High	High
106.1	High	High	High	High	Peak Foun	High	High	High
107.6	High	High	High	High	High	High	High	High
140.5	High	High	High	High	High	High	High	High

122.5	High	High	High	High	High	High	High	High
104	High	High	High	High	High	High	High	High
82.1	High	High	High	High	High	High	High	High
114.2	High	High	High	High	High	High	High	High
79	High	High	High	High	High	High	High	High
121.8	High	High	High	High	High	High	High	High
178.4	High	High	High	High	High	High	High	High
123.6	High	High	High	High	High	High	High	High
99.8	High	High	High	High	High	High	High	High
82.2	High	High	High	High	High	High	High	High
110.4	High	High	High	High	Peak Foun	High	High	High
114.8	High	High	High	High	High	High	High	High
101.6	High	High	High	High	High	High	High	High
103.7	High	High	High	High	High	High	High	High
116.9	High	High	High	High	High	High	High	High
155.5	High	High	High	High	Peak Foun	Peak Foun	Peak Foun	Peak Foun
109.6	High	High	High	High	High	High	High	High
194.4	High	High	High	High	Peak Foun	Peak Foun	Peak Foun	Peak Foun
117.3	High	High	High	High	High	High	High	High
103.8	High	High	High	High	High	High	High	High
112	High	High	High	High	High	High	High	High
198.6	High	Peak Foun	High	Peak Foun	Peak Foun	Peak Foun	High	Peak Foun
106.1	High	High	High	High	High	High	High	High
116.1	High	High	High	High	High	High	High	High
149	High	High	High	High	Peak Foun	Peak Foun	Peak Foun	High
97	High	High	High	High	High	High	High	High
98.1	High	High	High	High	High	High	High	High
97.3	Peak Foun	Peak Foun	Peak Foun	Not Found	Peak Foun	Peak Foun	Peak Foun	Peak Foun
152.9	Not Found	Not Found	High	High	Not Found	High	High	High
122.2	High	High	High	High	High	High	High	High
39.5	High	High	High	High	High	High	High	High
88.3	High	High	High	High	High	High	High	High
108.5	High	High	High	High	High	High	High	High
101.3	Not Found	High	High	Peak Foun	Not Found	Not Found	Not Found	Not Found
94.2	High	High	High	High	High	High	High	High
124.8	High	High	High	High	High	High	High	High
140.9	High	High	High	High	High	High	High	High
142	High	High	High	High	High	High	High	High
117.3	High	High	High	High	High	High	High	High
93.3	High	High	High	High	High	High	High	High
140.4	High	High	High	High	High	High	High	High
186	High	High	High	High	Peak Foun	Peak Foun	Peak Foun	Peak Foun
115.3	High	High	High	High	High	High	High	High
135.3	High	High	High	High	High	High	High	High
134.9	High	High	High	High	High	High	High	High

129	High	High	High	High	High	High	High	High
118.6	High	High	High	High	High	High	High	High
99	High	High	High	High	High	High	High	High
159	High	High	High	High	Peak Foun	High	High	High
55.3	High	High	High	High	High	High	High	High
98.5	High	High	High	High	High	High	High	High
105	High	High	High	High	High	High	High	High
87.2	High	High	High	High	High	High	High	High
99	High	High	High	High	High	High	High	High
98.6	High	High	High	High	High	High	High	High
102.6	High	High	High	High	Peak Foun	High	High	High
89.5	High	High	High	High	High	High	High	High
94.6	High	High	High	High	High	High	High	High
102.8	High	High	High	High	High	High	High	High
105.8	High	High	High	High	High	High	High	High
76.9	High	High	High	High	High	High	High	High
97.5	High	High	High	High	High	High	High	High
79	High	High	High	High	High	High	High	High
96.2	High	High	High	High	High	High	High	High
114.5	High	High	High	High	High	High	High	High
90.7	High	High	High	High	High	High	High	High
112.5	High	High	High	High	High	High	High	High
88.7	High	High	High	High	High	High	High	High
171.1	High	High	High	High	Peak Foun	Peak Foun	Peak Foun	High
102.4	High	High	High	High	High	High	High	High
96.1	Peak Foun	High	High	High	Peak Foun	High	High	High
98.5	High	High	High	High	High	High	High	High
104.4	High	High	High	High	High	High	High	High
94.6	High	High	High	High	High	High	High	High
112.6	High	High	High	High	High	High	High	High
103.2	High	High	High	High	High	High	High	High
91.5	High	High	High	High	Peak Foun	High	High	High
102.4	High	High	High	High	High	High	High	High
118.5	High	High	High	High	High	High	High	High
137	High	High	High	High	Peak Foun	High	High	High
155.2	High	High	High	High	High	Peak Foun	High	High
119.1	High	High	High	High	High	High	High	High
96	High	High	High	High	High	High	High	High
135.1	High	High	High	High	Peak Foun	High	High	High
101.3	High	High	High	High	High	High	High	High
124.2	High	High	High	High	High	High	High	High
100.3	High	High	High	High	High	High	High	High
130.4	High	High	High	High	Peak Foun	High	High	High
113.3	High	High	High	High	High	High	High	High
100.6	High	High	High	High	High	High	High	High

128.8	High	High	High	High	High	High	High	High
101.2	High	High	High	High	High	High	High	High
145.3	High	High	High	High	Peak Foun	High	High	High
128	High	High	High	High	Peak Foun	High	High	High
101.7	High	High	High	High	High	High	High	High
99	High	High	High	High	High	High	High	High
129.3	High	High	High	High	High	High	High	High
198.8	Peak Foun	Peak Foun	Peak Foun	Peak Foun	Peak Foun	Peak Foun	Peak Foun	Peak Foun
105.5	High	High	High	High	High	High	High	High
106.6	High	High	High	High	High	High	High	High
112.2	High	High	High	High	High	High	High	High
107.5	High	High	High	High	High	High	High	High
142.5	High	High	High	High	High	High	High	High
119.9	High	High	High	High	High	High	High	High
100.1	High	High	High	High	High	High	High	High
87.4	High	High	High	High	High	High	High	High
113.8	High	High	High	Peak Foun	High	High	High	High
89.2	High	High	High	High	High	High	High	High
130.3	High	High	High	High	High	High	High	High
122.6	High	High	High	High	High	High	High	High
130.9	High	High	High	High	Peak Foun	High	High	High
110.2	High	High	High	High	High	High	High	High
118.8	High	High	High	High	High	High	High	High
117.6	High	High	High	High	High	High	High	High
113.8	High	High	High	High	High	High	High	High
90.4	High	High	High	Peak Foun	Peak Foun	High	High	High
	Not Found	High	Not Found	Not Found	Not Found	Peak Foun	Not Found	Not Found
110.3	High	High	High	High	Peak Foun	Peak Foun	Peak Foun	Peak Foun
103.2	High	High	High	High	Peak Foun	High	High	High
104.7	High	High	High	High	High	High	High	High
110	High	High	High	High	High	High	High	High
100.8	High	High	High	High	High	High	High	High
127.4	High	High	High	High	High	High	High	High
100.5	High	High	High	High	High	High	High	High
102.3	High	High	High	High	High	High	High	High
118.9	High	High	High	High	High	High	High	High
92.1	High	High	High	High	High	High	High	High
135.6	High	High	High	High	High	High	High	High
134.5	High	High	High	High	High	High	High	High
123.2	High	High	High	High	High	High	High	High
198.1	Peak Foun	Peak Foun	Peak Foun	Peak Foun	Peak Foun	Peak Foun	Peak Foun	Peak Foun
105.4	High	High	High	High	High	High	High	High
102.4	High	High	High	High	High	High	High	High
108	High	High	High	High	High	Peak Foun	Peak Foun	Peak Foun
108.4	High	High	High	High	High	High	High	High

138.4	High	High	High	High	Peak Foun	Peak Foun	Peak Foun	High
48.4	High	High	High	High	High	High	High	High
110.3	High	High	High	High	High	High	High	High
172	High	High	High	High	Peak Foun	High	High	Peak Foun
106.5	High	High	High	High	High	High	High	High
105.2	High	High	High	High	High	High	High	High
107.5	High	High	High	High	High	High	High	High
50.9	High	High	High	High	High	High	High	High
146.1	High	High	High	High	Peak Foun	Peak Foun	High	High
103.8	High	High	High	High	Peak Foun	High	High	High
198.8	Peak Foun	High	Peak Foun	Peak Foun	Peak Foun	Peak Foun	Peak Foun	Peak Foun
139.1	High	High	High	High	High	High	High	High
117.5	High	High	High	High	High	High	High	High
148.8	High	High	High	High	High	High	High	High
110.9	High	High	High	High	High	High	High	High
108.9	High	High	High	High	High	High	High	High
107.5	High	High	High	High	High	High	High	High
127.2	High	High	High	High	Peak Foun	High	High	High
86.7	High	High	High	High	High	High	High	High
123.5	High	High	High	High	High	High	High	High
105.1	High	High	High	High	High	High	High	High
114.2	Peak Foun	High	Peak Foun	Not Found	Peak Foun	Not Found	Not Found	Peak Foun
110.9	High	High	High	High	High	High	High	High
197.4	Peak Foun	High	High	Peak Foun	Peak Foun	High	High	Peak Foun
125.9	High	High	High	High	Peak Foun	High	High	High
95.5	High	High	High	High	High	High	High	High
105.6	High	High	High	High	High	High	High	High
122.8	High	High	High	High	High	Peak Foun	High	High
92.2	High	High	High	High	High	High	High	High
121.6	High	High	High	High	High	High	High	High
114.6	High	High	High	High	High	High	High	High
97.5	High	High	High	Peak Foun	High	High	High	High
110.9	High	High	High	High	High	High	High	High
106.8	High	High	High	High	High	High	High	High
169.4	High	High	High	High	High	High	High	High
102.1	High	High	High	High	High	High	High	High
65.2	High	High	High	High	High	High	High	High
171.8	High	High	High	High	High	High	High	High
100.9	High	High	High	High	High	High	High	High
153.3	High	High	High	High	Peak Foun	Peak Foun	High	High
144.3	High	High	High	High	Peak Foun	Peak Foun	High	High
98.1	High	High	High	High	High	High	High	High
79.8	High	High	High	High	High	High	High	High
123.2	High	High	High	High	High	High	High	Peak Foun
105.7	High	High	High	High	High	High	High	High

132	High	High	High	High	Peak Foun	High	High	High
102.9	High	High	High	Peak Foun	Not Found	Peak Foun	High	Peak Foun
112	High	High	High	High	High	High	High	High
113.4	High	High	High	High	High	High	High	High
88.6	High	High	High	High	High	High	High	High
105	High	High	High	High	Peak Foun	High	High	High
91.3	High	High	High	High	High	High	High	High
140.7	High	High	High	High	High	High	High	High
107.3	High	High	High	High	High	High	High	High
163.2	High	High	High	Peak Foun	Peak Foun	Peak Foun	Peak Foun	Peak Foun
158.1	High	High	High	High	High	High	Peak Foun	Peak Foun
118.7	High	High	High	High	High	High	High	High
110.3	High	High	High	High	High	High	High	High
119.3	High	High	High	High	High	High	High	High
126.9	High	High	High	High	High	High	High	High
131.7	High	High	High	High	High	High	High	High
90.8	High	High	High	High	High	High	High	High
101.7	High	High	High	High	High	High	High	High
68.6	High	High	High	High	High	High	High	High
115.7	High	High	High	High	High	High	High	High
87.4	High	High	High	High	High	High	High	High
109	High	High	High	High	High	High	High	High
116.8	High	High	High	High	High	High	High	High
116.3	High	High	High	High	High	Peak Foun	Peak Foun	High
132.4	High	High	High	High	High	High	High	High
120.1	High	High	High	High	Peak Foun	Not Found	Peak Foun	High
143.1	High	High	High	High	High	High	High	High
142.8	High	High	High	High	Peak Foun	Peak Foun	High	High
113.1	High	High	High	High	High	High	High	High
101.9	High	High	High	High	High	High	Peak Foun	High
190	High	High	High	High	Peak Foun	High	High	High
103.9	High	High	High	High	High	High	High	High
102.1	High	High	High	High	High	High	High	High
104.9	High	High	High	High	Peak Foun	High	High	High
110.5	High	High	High	High	High	High	High	High
98.2	High	High	High	High	High	High	High	High
99.5	High	High	High	High	High	High	High	High
126.3	High	High	High	High	High	High	High	High
115.8	High	High	High	High	High	High	High	High
117.1	High	High	High	High	High	High	High	High
103.9	High	High	High	High	Peak Foun	High	High	High
113.2	High	High	High	High	Peak Foun	Peak Foun	Peak Foun	High
144	High	High	High	High	High	High	High	High
116.6	High	High	High	High	High	High	High	High
94.7	High	High	High	High	High	High	High	High

106.8	High	High	High	High	High	High	High	High
112.7	High	High	High	High	High	High	High	High
123.2	High	High	High	High	High	High	High	High
110.7	High	High	High	High	High	High	High	High
108.8	High	High	High	High	High	High	High	High
112.4	Not Found	High	Not Found	High	Not Found	High	Not Found	Peak Foun
108.3	High	High	High	High	High	High	High	High
101.9	High	High	High	High	High	High	High	High
132.4	High	High	High	High	High	High	High	High
126.1	High	High	High	High	Not Found	Not Found	Peak Foun	Not Found
108	High	High	High	High	High	High	High	High
120.1	High	High	High	High	High	High	High	High
106.4	High	High	High	High	High	High	High	High
119.6	High	High	High	High	High	High	High	High
129.5	High	High	High	High	High	High	High	High
92.7	High	High	High	High	High	High	High	High
120.9	High	High	High	High	High	High	High	High
102.8	High	High	High	High	High	High	High	High
108.1	High	High	High	High	High	High	High	High
95.9	High	High	High	High	High	High	High	High
140.6	High	High	High	High	High	High	High	High
63.6	High	High	High	High	High	High	High	High
106.1	High	High	High	High	High	High	High	High
111.7	High	High	High	High	High	High	High	High
164.5	High	High	High	Peak Foun	Peak Foun	Peak Foun	Peak Foun	Peak Foun
106.6	High	High	High	High	High	High	High	High
147	High	High	High	High	Peak Foun	High	High	High
93.6	High	High	High	High	High	High	High	High
94.4	High	High	High	High	High	High	High	High
99.3	High	High	High	High	High	High	High	High
104	High	High	High	High	High	High	High	Peak Foun
123.2	High	High	High	High	High	High	High	High
167	High	High	High	Peak Foun	Not Found	Peak Foun	Not Found	Not Found
125.1	High	High	High	High	High	High	High	High
133.9	High	High	High	Peak Foun	Peak Foun	High	Peak Foun	High
121.2	High	High	High	High	High	High	High	High
93.5	High	High	High	High	High	High	High	High
152.3	High	High	High	Peak Foun	Peak Foun	Peak Foun	Peak Foun	High
108.1	High	High	High	High	High	High	High	High
115.5	High	High	High	High	Peak Foun	High	High	High
93.1	High	High	High	High	High	High	High	High
106.7	High	High	High	High	High	High	High	High
99.9	High	High	High	High	Peak Foun	Peak Foun	High	High
134.5	High	High	High	High	High	High	High	High
107.1	High	High	High	High	High	High	High	High

112.3	High	High	High	High	High	High	High	High
117.3	High	High	High	High	High	High	High	High
91.7	High	High	High	High	High	High	High	High
92.7	High	High	High	High	High	High	High	High
134.9	High	High	High	High	High	High	High	High
89.4	High	High	High	High	Peak Foun	High	High	High
119	High	High	High	High	High	High	High	High
105	High	High	High	High	High	High	High	High
121.7	High	High	High	High	High	High	High	High
126.8	High	High	High	High	High	High	High	High
101	High	High	High	High	High	High	High	High
101	High	High	High	High	High	High	High	High
102.4	High	High	High	High	High	High	High	High
105.4	High	High	High	High	High	High	High	High
70.2	High	High	High	High	High	High	High	High
198.2	Peak Foun	Peak Foun	Peak Foun	Peak Foun	Peak Foun	Peak Foun	Peak Foun	Peak Foun
115.7	High	High	High	High	High	High	High	High
141.5	High	High	High	High	High	High	High	High
98.6	High	High	High	High	High	High	High	High
141.8	High	High	High	High	High	High	Peak Foun	High
101.8	High	High	High	High	High	High	High	High
79.6	High	High	High	High	High	High	High	High
105.7	High	High	High	High	High	High	High	High
94.5	High	High	High	High	High	High	High	High
105.7	High	High	High	High	High	High	High	High
116	High	High	High	High	High	High	High	High
99.5	High	High	High	High	High	High	High	High
153	High	High	High	High	Peak Foun	Peak Foun	High	High
113.6	High	High	High	High	High	High	High	High
79.1	High	High	High	Peak Foun	High	High	High	High
161.3	High	High	High	High	Peak Foun	High	High	High
107.9	High	High	High	High	High	High	High	High
95.1	High	High	High	High	High	High	High	High
112.2	High	High	High	High	High	High	High	High
103.8	High	High	High	High	High	High	High	High
118.4	High	High	Peak Foun	High	Peak Foun	Peak Foun	Peak Foun	Peak Foun
115.3	High	Peak Foun	Peak Foun	High	High	High	Peak Foun	Peak Foun
132.7	High	High	High	High	Peak Foun	High	High	High
120.4	High	High	High	High	High	High	High	High
95.4	High	High	High	High	High	High	High	High
110.6	High	High	High	High	High	High	High	High
115.2	High	High	High	High	High	High	High	High
147.1	High	High	High	High	Peak Foun	High	Peak Foun	High
119.5	High	High	High	High	High	High	High	High
148.9	High	High	High	High	High	High	High	Peak Foun

106.1	High	High	High	High	High	High	High	High
136.5	High	High	High	High	High	High	High	High
91.2	High	High	Peak Foun	Peak Foun	High	High	Peak Foun	High
172.1	High	High	High	Peak Foun	Peak Foun	Peak Foun	Peak Foun	Peak Foun
139.7	High	High	High	High	Peak Foun	High	High	High
142.5	High	High	High	High	Peak Foun	Peak Foun	High	High
108.6	High	High	High	High	High	High	High	High
111.3	High	High	High	High	High	High	High	High
104.7	High	High	High	High	High	High	High	High
120.2	High	High	High	High	High	High	High	High
71.2	High	High	High	High	High	High	High	High
140.6	High	High	High	High	Peak Foun	High	High	High
93.5	High	High	High	High	High	High	High	High
134.4	High	High	High	High	High	High	High	High
114.3	High	High	High	High	High	High	High	High
92.8	High	High	High	High	High	High	High	High
87.1	High	High	High	High	High	High	High	High
91.5	Peak Foun	Peak Foun	High	Peak Foun	Peak Foun	Peak Foun	High	High
107.3	High	High	High	High	High	High	High	High
91.3	High	High	High	High	High	High	High	High
157.9	High	High	High	High	High	Peak Foun	Peak Foun	High
108.8	High	High	High	High	High	High	High	High
121.2	High	High	High	High	Peak Foun	High	High	High
121.8	High	High	High	High	High	High	Peak Foun	High
98.9	High	High	High	High	High	High	High	High
105.4	High	High	High	High	High	High	High	High
97	High	High	High	High	High	High	High	High
114.9	High	High	High	High	High	High	High	High
93.7	High	High	High	High	High	High	High	High
129.8	High	High	High	High	High	High	High	High
102.7	High	High	High	High	High	High	High	High
145.7	High	High	High	High	Peak Foun	Peak Foun	Peak Foun	High
102.5	High	High	High	High	High	High	High	High
95.8	High	High	High	High	High	High	High	High
104.6	High	High	High	High	High	High	High	High
152.5	High	High	High	High	Peak Foun	High	Peak Foun	High
132.4	High	High	High	High	Peak Foun	Peak Foun	Peak Foun	Peak Foun
96.8	High	High	High	High	High	High	High	High
111.4	High	High	High	High	High	High	High	High
117	High	High	High	High	High	High	High	High
93.6	High	High	High	High	High	High	High	High
108.5	High	High	High	High	Peak Foun	High	High	High
96.1	High	High	High	High	High	High	High	High
160	High	High	High	Peak Foun	High	High	Peak Foun	High
144.8	High	High	High	High	Peak Foun	Peak Foun	Peak Foun	Peak Foun

128.5	High	High	High	High	High	High	High	High
110.8	High	High	High	High	High	High	High	High
129.7	High	High	High	Peak Foun	Peak Foun	Peak Foun	Peak Foun	Peak Foun
83	High	High	High	High	High	High	High	High
159.1	High	High	High	High	High	High	High	High
134.9	High	High	High	High	High	High	High	High
127.8	High	High	High	High	High	High	High	High
101.4	High	High	High	High	High	High	High	High
140.9	High	High	High	High	High	High	High	High
91.8	High	High	High	High	High	High	High	High
91.2	High	High	High	High	High	High	High	High
108.1	High	High	High	High	High	High	High	High
103.6	High	High	High	High	High	High	High	High
98.4	High	High	High	High	High	High	High	High
72.2	High	High	High	High	High	High	High	High
138.1	High	High	High	High	Peak Foun	High	Peak Foun	High
129.9	High	High	High	High	Peak Foun	Peak Foun	High	High
96.7	High	High	High	High	High	High	High	High
107.1	High	High	High	High	High	High	High	High
187.5	High	High	High	High	Peak Foun	High	Not Found	Peak Foun
99.2	High	High	High	High	High	High	High	High
117.5	High	High	High	High	Peak Foun	High	High	High
135.5	Peak Foun	High	High	Peak Foun	Peak Foun	Peak Foun	Peak Foun	Peak Foun
102.1	High	High	High	High	High	High	High	High
96.1	High	High	High	High	High	High	High	High
133.6	High	High	High	High	Peak Foun	High	Peak Foun	High
102.4	High	High	High	High	High	High	High	High
118.9	High	High	High	High	High	High	High	High
120.8	High	High	High	High	High	High	High	High
111.8	High	High	High	High	Peak Foun	High	High	High
105.4	High	High	High	High	High	High	High	High
115.2	High	High	High	High	High	High	High	High
154.4	High	High	High	High	High	Peak Foun	Peak Foun	High
128.6	High	High	High	High	Peak Foun	Peak Foun	High	High
125.2	High	High	High	High	High	High	High	High
110.3	High	High	High	High	High	High	High	High
117.3	High	High	High	High	Peak Foun	High	High	High
130.8	High	High	High	High	High	High	High	High
130.2	High	High	High	High	High	High	High	High
99.7	High	High	High	High	High	High	High	High
147.2	High	High	High	Peak Foun	Peak Foun	High	High	Peak Foun
59.3	High	High	High	Peak Foun	High	High	High	Peak Foun
100.7	High	High	High	High	High	High	High	High
137.2	High	High	High	High	High	High	High	High
103	High	High	High	High	High	High	High	High

137.2	High	High	High	Peak Foun	High	High	Peak Foun	High
112.3	High	High	High	High	High	High	High	High
86.7	High	High	High	High	High	High	High	High
102.3	High	High	High	High	High	High	High	High
100.3	High	High	High	High	High	High	High	High
131.1	High	High	High	High	High	High	High	High
106.5	High	High	High	High	Peak Foun	High	High	High
168.6	High	High	High	High	Peak Foun	High	High	High
117.7	High	High	High	High	High	High	High	High
91.3	High	High	High	High	High	High	High	High
143.9	High	High	High	High	Peak Foun	Peak Foun	Peak Foun	High
135.1	High	High	High	High	High	High	High	High
124	High	High	High	High	High	High	High	High
101.7	High	High	High	High	High	High	High	High
111.2	High	High	High	High	Peak Foun	High	High	High
83.1	High	High	High	High	High	High	High	High
191	High	High	High	High	High	High	High	High
105.4	High	High	High	High	High	High	High	High
163.4	High	High	High	High	Peak Foun	High	High	High
154.6	High	High	High	Peak Foun	Peak Foun	Peak Foun	High	Peak Foun
86.6	High	High	High	High	High	High	High	High
109.6	High	High	High	High	High	High	High	High
121.9	High	High	High	High	High	High	High	High
109.7	High	High	High	Peak Foun	High	High	High	High
111.3	High	High	High	High	High	High	High	High
121.5	High	High	High	High	High	High	Peak Foun	High
112.5	High	High	High	High	High	High	High	High
141.5	High	High	High	High	High	High	Peak Foun	High
149.2	High	High	High	High	High	High	High	High
76.5	High	High	High	Peak Foun	Peak Foun	High	High	High
139.1	High	High	High	High	Peak Foun	High	High	High
97.4	High	High	High	High	High	High	High	High
77.4	High	High	High	High	High	High	High	High
174.8	High	High	High	High	Peak Foun	High	High	Peak Foun
104	High	High	High	High	Peak Foun	High	High	High
107.6	High	High	High	High	High	High	High	High
42.7	High	High	High	High	Peak Foun	Peak Foun	Peak Foun	High
117.2	High	High	High	High	High	High	High	High
118.1	High	High	High	High	High	High	High	High
91.3	High	High	High	High	High	High	High	High
146.8	High	High	High	High	High	High	High	High
128	High	High	High	High	High	High	High	High
171.8	High	High	High	High	Peak Foun	Peak Foun	High	High
189.8	Not Found	Not Found	Not Found	Not Found	Peak Foun	Not Found	Not Found	Not Found
125.7	High	High	High	High	Peak Foun	Peak Foun	High	High

96	High	High	High	High	High	High	High	High
102.6	High	High	High	High	High	High	High	High
116.8	High	High	High	High	High	High	Peak Foun	High
131.3	High	High	Peak Foun	Not Found	Peak Foun	Peak Foun	Peak Foun	Peak Foun
106.9	High	High	High	High	High	High	High	High
92.9	High	High	High	High	High	High	High	High
95.3	High	High	High	High	High	High	High	High
95	High	High	High	High	High	High	High	High
100.5	High	High	High	Peak Foun	Peak Foun	Not Found	Peak Foun	Peak Foun
123.5	High	High	High	High	High	High	High	High
134.6	High	High	High	High	High	High	High	High
80.3	High	High	High	High	High	High	High	High
104.7	High	High	High	High	High	High	High	High
79.6	High	High	High	High	High	High	High	High
153.9	High	Peak Foun	High	High	Not Found	Peak Foun	Peak Foun	Not Found
94.7	High	High	High	High	High	High	High	High
111.2	High	High	High	High	High	High	High	High
126	High	High	High	High	High	High	High	High
116.1	High	High	High	High	High	High	High	High
82.9	High	High	High	High	High	High	High	High
199.2	Peak Foun	Peak Foun	High	Peak Foun	Peak Foun	Peak Foun	Peak Foun	Peak Foun
99.6	High	High	High	High	High	High	High	High
120.8	High	High	High	High	High	High	High	High
112.4	High	High	High	High	High	High	High	High
121.4	High	High	High	High	High	High	High	Peak Foun
86.9	High	High	High	High	High	High	High	High
100.1	High	High	High	High	High	High	High	High
154.7	High	High	High	High	Peak Foun	Peak Foun	Peak Foun	High
113.7	High	High	High	High	High	High	High	High
136	High	High	High	High	High	Peak Foun	Peak Foun	High
105	High	High	High	High	High	High	High	High
126.6	High	High	High	High	High	High	Peak Foun	High
109.8	High	High	High	High	High	High	High	High
114.6	High	High	High	High	High	High	High	High
160.1	High	High	High	High	Peak Foun	Peak Foun	Peak Foun	High
139.1	High	High	High	High	High	High	High	High
139.3	High	High	High	High	High	High	High	High
102.8	High	High	High	Peak Foun	High	High	High	Peak Foun
87.1	High	High	High	High	High	High	High	High
99.9	High	High	High	High	High	High	High	High
129.1	High	High	Peak Foun	Peak Foun	Peak Foun	Peak Foun	Not Found	High
115.4	High	High	High	High	High	Peak Foun	High	High
122.7	High	High	High	Peak Foun	Peak Foun	High	Peak Foun	High
136.7	High	High	High	High	Peak Foun	Peak Foun	Peak Foun	High
122.3	High	High	High	High	Peak Foun	High	High	High

100.5	High	High	High	High	High	High	High	High
129.5	High	High	High	High	High	High	High	High
111	High	High	High	High	High	High	High	High
82.2	High	High	High	Peak Foun	High	High	High	Peak Foun
57.2	High	High	High	High	High	High	High	High
101.9	High	High	High	High	High	High	High	High
102.7	High	High	High	High	High	High	High	High
114.7	High	High	High	High	High	High	High	High
122	High	High	High	High	High	High	High	High
109.3	High	High	High	High	High	High	High	High
112.6	High	High	High	High	High	High	High	High
141.4	High	High	High	Peak Foun	Peak Foun	High	High	High
115.6	High	High	High	High	High	High	High	High
113.7	High	High	High	High	High	High	High	High
133.4	High	High	High	High	High	High	High	High
59.7	High	High	High	High	High	High	High	High
104.5	High	High	High	High	High	High	High	High
108.9	High	High	High	High	High	High	High	High
104.2	High	High	High	High	High	High	High	High
117.3	High	High	High	High	High	High	High	High
98.1	High	High	High	High	High	High	High	High
115.9	High	High	High	High	High	High	High	High
109.7	High	High	High	High	Peak Foun	High	Peak Foun	High
110.4	High	High	High	High	Peak Foun	High	High	High
130.2	High	High	High	High	High	High	High	High
134.9	High	High	High	High	High	High	High	High
121.5	High	High	High	High	Peak Foun	High	High	High
165.2	High	High	High	High	Peak Foun	Peak Foun	Peak Foun	High
117	High	High	High	High	High	High	High	High
103	High	High	High	High	Peak Foun	High	High	High
145.1	High	High	High	High	High	High	High	High
108.4	High	High	High	High	High	High	High	High
93.1	High	High	High	High	High	High	High	High
133.5	High	High	High	High	Not Found	Peak Foun	Peak Foun	High
89.2	High	High	High	High	High	High	High	High
102.8	High	High	High	High	High	High	High	High
89	High	High	High	High	High	High	High	High
153.9	High	High	High	High	Peak Foun	Peak Foun	Peak Foun	High
95.4	High	High	High	High	High	High	High	High
115	High	High	High	High	High	High	High	Peak Foun
124.9	High	High	High	High	Peak Foun	High	High	High
93.2	High	High	High	High	High	High	High	High
127.9	High	High	High	High	High	High	High	High
113.6	High	High	High	High	High	High	High	High
148.1	High	High	High	High	High	Peak Foun	Peak Foun	High

115.1	High	High	High	High	High	High	High	High
92.3	High	High	High	High	High	High	High	High
136	High	High	High	High	High	Peak Foun	Peak Foun	High
106	High	High	High	Peak Foun	Peak Foun	Peak Foun	High	Peak Foun
108	High	High	High	High	High	High	High	High
117.7	High	High	High	High	High	High	High	High
160.2	High	High	High	High	High	High	High	Peak Foun
102.6	High	High	High	High	High	High	High	High
120	High	Peak Foun	High	High	Not Found	Peak Foun	Peak Foun	High
107.8	High	High	High	High	Peak Foun	High	High	High
136.2	High	High	High	Peak Foun	High	High	High	High
105.1	High	High	High	High	High	Peak Foun	High	High
104.6	High	High	High	High	High	High	High	High
157.1	High	High	High	High	High	High	High	High
99	High	High	High	High	High	High	High	High
104.7	High	High	High	High	High	High	High	High
103.3	High	High	High	High	High	High	High	High
91.9	High	High	High	High	Peak Foun	High	High	High
98.9	High	High	Peak Foun	Peak Foun	High	High	High	High
110.9	High	High	High	High	High	High	High	High
115.1	High	High	High	High	High	High	High	High
131.3	High	High	High	High	Peak Foun	High	High	High
94.9	High	High	High	High	High	High	High	High
125.2	High	High	High	Peak Foun	High	High	High	High
130.7	High	High	High	High	High	High	High	High
130.9	High	High	High	High	High	High	High	High
96.1	High	High	High	High	High	High	High	High
103.6	High	High	High	High	High	High	High	High
152.5	High	High	High	High	Peak Foun	Peak Foun	Peak Foun	High
48.6	High	High	High	High	High	High	High	High
141.8	High	High	High	High	High	Peak Foun	Peak Foun	Peak Foun
144.9	High	High	High	Peak Foun	Peak Foun	Peak Foun	Peak Foun	Peak Foun
115.5	High	High	High	High	High	High	High	High
81.9	High	High	High	High	High	High	High	High
120.8	High	High	High	High	High	High	High	High
98.8	High	High	High	High	High	High	High	High
104.9	High	High	High	High	High	High	High	High
123.3	High	High	High	Peak Foun	Peak Foun	Peak Foun	Peak Foun	Peak Foun
99.6	High	High	High	Peak Foun	High	High	High	High
156.3	High	High	High	High	High	High	High	High
112.7	High	High	High	High	Peak Foun	High	Peak Foun	High
106.3	High	High	High	High	High	High	High	High
128.5	High	Peak Foun	High	High	Not Found	High	High	Peak Foun
106.7	High	High	High	High	High	High	High	High
124.8	High	High	High	High	Peak Foun	Peak Foun	High	High

112	High	High	High	High	Peak Foun	High	High	High
188.7	High	High	High	High	High	Peak Foun	High	High
111.5	High	High	High	High	High	Peak Foun	High	Peak Foun
138.4	High	High	High	Peak Foun	Peak Foun	Peak Foun	High	High
121.4	High	High	High	High	High	High	High	High
85.6	High	High	High	High	High	High	High	High
91.7	High	High	High	High	High	High	High	High
155.6	High	High	High	High	High	High	Peak Foun	High
109	High	High	High	High	High	High	High	High
108.8	High	High	High	High	High	High	High	High
146	High	High	Peak Foun	High	High	High	Peak Foun	Peak Foun
102.9	High	High	High	High	High	High	High	High
107	High	High	High	High	High	High	High	High
77.2	High	High	High	High	High	High	Peak Foun	High
114.4	High	High	High	High	High	High	High	High
111.4	High	High	High	High	High	High	High	High
109	High	High	High	High	High	High	High	Peak Foun
112.4	High	High	High	High	High	High	High	High
120	High	High	High	Peak Foun	Peak Foun	Peak Foun	High	Peak Foun
121.3	High	High	High	High	High	High	High	High
129.7	High	High	High	High	High	High	High	High
110.7	High	High	High	High	High	High	High	High
105.9	High	High	High	High	High	High	High	High
146.2	High	High	Peak Foun	High	Peak Foun	High	Not Found	High
126.3	Peak Foun	High	High	High	Peak Foun	High	High	High
106.5	High	High	High	High	High	High	High	High
114.9	High	High	High	High	High	High	High	High
138.6	High	High	High	High	High	High	High	High
151.7	High	High	High	Peak Foun	Peak Foun	Peak Foun	Peak Foun	High
181.8	High	High	High	High	Peak Foun	High	High	High
114.6	Peak Foun	High	High	High	Peak Foun	High	High	High
114.8	High	High	High	Peak Foun	Peak Foun	Peak Foun	High	Peak Foun
97.9	High	High	High	High	High	High	High	High
123.1	High	High	Peak Foun	High	High	High	High	High
113	High	High	High	High	High	High	High	High
101.6	High	High	High	High	High	High	High	High
105.1	High	High	High	High	High	High	High	High
107.1	High	High	High	High	High	High	High	High
144	High	High	High	High	Peak Foun	Peak Foun	Peak Foun	High
120.1	High	High	High	High	High	High	High	High
103.2	High	High	High	High	High	High	High	High
118.6	High	High	High	High	High	High	High	High
111	High	High	High	High	High	High	High	High
132.3	High	High	High	High	Peak Foun	Peak Foun	High	High
108.2	High	High	High	High	High	High	High	High

96	High	High	High	Peak Foun	High	High	High	High
121.8	High	High	High	High	Peak Foun	High	High	High
117.4	High	Peak Foun	Peak Foun	Peak Foun	Peak Foun	Not Found	Peak Foun	Peak Foun
140.1	High	High	High	High	Peak Foun	Peak Foun	Peak Foun	High
112.9	High	High	High	High	High	High	High	High
130.4	High	High	High	High	High	Peak Foun	Peak Foun	High
113.9	Peak Foun	High	Peak Foun	Peak Foun	Peak Foun	Not Found	Peak Foun	Peak Foun
103.4	High	High	High	High	High	High	High	High
95.3	High	High	High	High	High	High	High	High
88.8	High	High	High	High	High	High	High	High
160.8	High	High	High	High	Peak Foun	Peak Foun	High	High
88.1	High	High	High	High	High	High	High	High
99.9	High	High	High	High	Peak Foun	Peak Foun	High	High
120	High	High	High	High	High	High	High	High
127	High	High	High	High	High	High	High	High
103.5	High	High	High	High	High	High	High	High
98.9	High	High	High	High	High	High	High	High
99.8	High	High	High	High	High	High	Peak Foun	High
119.3	High	High	High	High	High	High	High	High
137.9	High	High	High	High	Peak Foun	Peak Foun	Peak Foun	High
106.4	High	High	High	High	High	High	High	High
100.5	High	High	High	High	High	High	High	High
123.3	High	High	High	High	High	High	High	High
106.9	High	High	High	High	High	High	High	High
124.1	High	High	High	High	High	High	High	High
108	High	High	High	High	High	High	High	High
87.6	High	High	High	High	High	High	High	High
76.5	High	High	Peak Foun	Peak Foun	High	High	Peak Foun	Peak Foun
114.3	High	High	High	High	High	High	High	High
109	High	High	High	High	Peak Foun	Peak Foun	Peak Foun	High
130.3	High	High	High	Peak Foun	Peak Foun	Peak Foun	High	Peak Foun
153	High	High	High	High	High	High	High	Peak Foun
105.1	High	High	High	Peak Foun	High	High	High	High
113	High	High	High	High	Not Found	Peak Foun	High	High
106.3	High	High	High	High	High	Peak Foun	High	High
114.7	High	High	High	High	High	High	High	High
120.1	High	High	High	High	High	High	High	High
110.2	High	High	High	High	High	High	High	High
151.5	High	High	High	High	Peak Foun	Peak Foun	Peak Foun	High
145.7	High	High	High	High	Peak Foun	Peak Foun	High	High
160.4	High	High	High	High	Peak Foun	Peak Foun	High	High
88.7	High	High	High	High	High	Peak Foun	High	High
115	High	High	High	High	High	High	High	High
155.2	High	High	High	High	High	High	High	High
117.3	Peak Foun	High	High	High	Peak Foun	High	High	Peak Foun

109.4	High	High	High	High	High	High	High	High
113.8	High	High	High	High	High	High	High	High
109.1	High	High	High	High	High	High	High	High
103.7	High	High	High	High	High	High	High	High
122	High	High	High	High	High	High	High	High
107.7	High	High	High	High	High	High	High	High
107.7	High	High	High	High	High	High	High	High
115.5	High	High	High	High	High	High	High	High
87.1	High	High	Peak Foun	Peak Foun	Not Found	Not Found	Not Found	Peak Foun
106.4	High	High	High	High	High	High	High	High
154.7	High	High	Peak Foun	Peak Foun	High	High	Peak Foun	High
128.9	High	High	High	High	Peak Foun	Peak Foun	High	High
147.6	High	High	High	High	High	Peak Foun	High	High
114.8	High	High	High	High	High	High	High	High
124	High	High	High	High	High	High	High	High
94.1	High	High	Peak Foun	Peak Foun	High	High	High	Peak Foun
150.4	High	High	High	Peak Foun	Peak Foun	High	High	Peak Foun
135.4	High	High	High	High	Peak Foun	Peak Foun	High	High
122.2	High	High	High	Peak Foun	High	High	High	High
86.8	High	High	High	High	High	High	High	High
98.7	High	High	High	High	High	High	High	High
163.4	High	High	High	High	High	High	High	High
119.7	High	High	High	High	Peak Foun	High	High	High
128	High	High	High	High	Peak Foun	Peak Foun	Peak Foun	Peak Foun
109.6	High	High	High	High	High	High	High	High
130.3	High	High	High	High	Peak Foun	Peak Foun	Peak Foun	Peak Foun
113.1	High	High	High	High	Peak Foun	Peak Foun	High	Peak Foun
119.2	High	High	High	High	High	High	High	High
125.3	High	High	High	High	High	High	High	High
105.7	High	High	High	High	High	Peak Foun	High	High
110.8	High	High	High	High	High	High	High	High
150.8	High	High	High	High	Peak Foun	High	Peak Foun	High
55.3	High	High	High	High	High	High	High	High
147.9	High	High	High	High	Peak Foun	Peak Foun	Peak Foun	High
112.3	High	High	High	High	High	High	High	High
104.6	High	High	High	High	High	High	High	High
124	High	High	High	High	High	High	High	Peak Foun
166.5	High	High	High	High	High	Peak Foun	Peak Foun	Peak Foun
106.3	High	High	High	High	High	High	High	High
90.8	High	High	High	High	Peak Foun	Peak Foun	High	Peak Foun
100.9	High	High	High	High	High	High	High	High
122.1	High	High	High	High	Peak Foun	High	High	High
106.7	High	High	High	High	High	High	High	High
95.5	High	High	High	High	High	High	High	High
76.2	High	High	High	Peak Foun	High	High	High	High

103.8	High	High	High	High	High	High	High	High
127.3	High	High	High	High	High	Peak Foun	Peak Foun	High
117.1	High	High	High	Peak Foun	Peak Foun	Peak Foun	High	Peak Foun
124.2	High	High	Peak Foun	High	High	High	Peak Foun	High
127.3	High	High	High	High	High	High	High	High
124.6	High	High	High	High	High	High	High	Peak Foun
115.1	High	High	High	High	High	High	High	High
138.3	High	High	High	High	Peak Foun	Peak Foun	High	High
166.3	High	High	High	High	Peak Foun	Peak Foun	High	Peak Foun
106.6	High	High	High	High	High	High	High	High
153.2	High	High	High	High	Peak Foun	High	High	High
110.9	Peak Foun	High	Peak Foun	High	Peak Foun	High	High	Peak Foun
103	High	High	High	High	High	High	High	High
98.6	High	High	High	High	High	High	High	High
110.8	High	High	High	High	High	High	High	High
109.5	High	High	High	Peak Foun	High	High	High	High
111.7	High	High	High	High	High	High	High	High
113.4	High	High	High	High	High	High	High	High
126.6	Peak Foun	Peak Foun	Not Found	Not Found	Peak Foun	Not Found	Not Found	Not Found
140.7	High	High	High	Peak Foun	Peak Foun	High	High	Peak Foun
89.3	High	High	High	High	High	High	High	High
91.9	High	High	High	High	High	High	High	High
107.2	High	High	High	High	High	High	High	High
40	High	High	Peak Foun	Peak Foun	Not Found	Not Found	Not Found	Peak Foun
133.2	High	Peak Foun	High	Peak Foun	Peak Foun	Peak Foun	Peak Foun	Peak Foun
156.3	High	High	High	High	Peak Foun	Peak Foun	Peak Foun	Peak Foun
102	High	High	High	High	High	High	High	High
114.7	High	High	High	High	Peak Foun	Peak Foun	Peak Foun	High
155.1	High	High	High	High	Peak Foun	Peak Foun	Peak Foun	High
114.3	High	High	High	High	High	High	High	High
124.5	Peak Foun	Peak Foun	Peak Foun	Peak Foun	Peak Foun	Not Found	Not Found	High
87.3	High	High	High	Peak Foun	High	High	Peak Foun	Peak Foun
120.1	High	High	High	High	Peak Foun	Peak Foun	High	High
42.6	High	High	High	High	High	High	High	High
96	High	High	High	High	High	High	High	High
99.7	High	High	High	High	Peak Foun	High	High	High
83	High	High	High	High	High	High	High	High
78.6	High	High	High	High	High	High	High	High
95.5	High	High	High	High	High	High	High	High
100.4	High	High	High	High	High	High	High	High
130.2	High	High	High	Peak Foun	Peak Foun	High	High	High
80.5	High	High	High	High	High	High	High	High
117.3	High	High	High	High	Peak Foun	High	High	High
134.7	High	High	High	High	High	High	High	High
98.9	High	High	High	High	High	High	Peak Foun	High

111	High	High	High	High	High	High	High	High
129.7	High	High	High	High	High	High	High	High
131.3	High	High	High	High	Peak Foun	Peak Foun	Peak Foun	High
163	High	High	High	High	Peak Foun	Peak Foun	Peak Foun	High
96.7	High	High	Not Found	Peak Foun	High	Peak Foun	Not Found	Not Found
105.6	High	High	High	High	High	High	High	High
85.1	High	High	High	High	Peak Foun	High	High	High
100	High	High	High	High	Peak Foun	High	High	High
114	High	High	High	High	High	High	Peak Foun	High
104.8	High	High	High	High	High	High	High	High
110.6	High	High	High	High	High	High	High	High
110.4	High	High	High	High	High	High	High	High
123.6	High	High	High	High	Peak Foun	High	High	High
122.2	High	High	High	High	High	High	High	High
61.7	High	High	Peak Foun	Peak Foun	High	High	High	Peak Foun
120	High	High	High	High	High	High	High	High
158.2	High	High	High	High	High	High	High	High
123.8	High	High	High	High	Peak Foun	High	High	High
106.3	High	High	High	High	High	High	High	High
109.3	High	High	High	High	High	Peak Foun	Peak Foun	High
108.7	High	High	High	High	High	High	High	High
57.3	Peak Foun	Peak Foun	Peak Foun	Peak Foun	High	High	High	Peak Foun
16.8	Peak Foun	Peak Foun	Peak Foun	Peak Foun	High	High	High	Peak Foun
102.6	Peak Foun	Peak Foun	Peak Foun	Peak Foun	Peak Foun	Peak Foun	Not Found	Peak Foun
107.3	High	High	High	High	High	High	High	High
107.9	High	High	High	High	High	High	Peak Foun	High
99.5	High	High	High	High	Peak Foun	High	High	High
108.6	High	High	High	High	High	High	High	High
86.4	High	High	High	High	High	High	High	High
106.8	High	High	High	High	Peak Foun	High	Peak Foun	High
116.2	High	High	High	High	High	High	High	High
126.7	High	High	High	High	High	High	High	High
74.2	High	High	High	High	High	High	High	High
104.7	High	High	High	High	Peak Foun	High	High	High
109.7	High	High	High	High	High	High	High	High
100.7	High	High	High	High	High	High	High	High
119.8	High	High	High	Peak Foun	High	Peak Foun	Peak Foun	High
93.3	High	High	High	High	High	High	High	High
122	High	High	Peak Foun	High	Peak Foun	High	High	High
126	High	High	High	Peak Foun	Peak Foun	High	High	Peak Foun
160.7	High	High	High	High	Peak Foun	Peak Foun	Peak Foun	High
130	High	High	High	High	Peak Foun	Peak Foun	High	Peak Foun
133.7	High	High	High	High	High	High	High	Peak Foun
155.6	High	High	High	High	Peak Foun	Peak Foun	Peak Foun	High
108.2	High	High	High	High	High	High	High	High

82.3	High	High	Peak Foun	Peak Foun	Peak Foun	High	Peak Foun	Peak Foun
97.9	High	High	High	High	High	High	High	Peak Foun
128.8	High	High	High	High	Peak Foun	Peak Foun	Peak Foun	High
112.1	High	High	High	High	High	High	High	High
97.6	High	High	High	High	Peak Foun	High	High	High
85.4	High	High	Peak Foun	Peak Foun	High	High	Peak Foun	Peak Foun
106	High	High	High	High	High	High	High	High
123.8	High	High	High	High	High	Peak Foun	High	High
77.1	High	High	High	High	High	High	High	High
131.5	High	High	High	High	Peak Foun	Peak Foun	Peak Foun	Peak Foun
125.3	High	High	High	High	High	Peak Foun	High	High
101.8	High	High	High	High	High	High	High	High
108.5	High	High	High	High	High	High	High	High
99.4	Peak Foun	High	Peak Foun	Peak Foun	Peak Foun	High	Peak Foun	Peak Foun
97.4	High	High	High	High	High	High	High	High
140.9	High	High	High	High	Peak Foun	High	High	High
119.3	High	High	High	High	High	Peak Foun	Peak Foun	High
113.4	High	High	High	Peak Foun	High	High	Peak Foun	Peak Foun
83	High	High	High	High	High	High	High	High
131.5	High	High	High	High	Peak Foun	Peak Foun	Peak Foun	High
101.6	High	High	High	High	High	High	Peak Foun	High
108.9	High	High	High	High	Peak Foun	High	High	High
128.5	High	High	High	Peak Foun	High	High	High	High
110.5	High	High	High	High	Peak Foun	High	High	High
124.8	High	High	High	High	High	High	High	High
172	High	High	High	High	Peak Foun	Peak Foun	Peak Foun	Peak Foun
99.6	High	High	High	High	High	High	High	High
108.3	High	High	High	High	Not Found	Not Found	High	High
128.8	High	High	High	High	High	High	High	High
101.7	High	High	High	High	High	High	High	High
111.1	High	High	High	High	High	High	High	High
98.7	High	High	High	Peak Foun	High	High	Peak Foun	High
127.9	High	High	High	High	High	High	High	High
112.5	High	High	High	High	High	High	High	High
125.2	High	High	High	High	High	High	High	High
127.4	High	High	Peak Foun	Peak Foun	Peak Foun	Peak Foun	Peak Foun	Peak Foun
155.6	High	High	High	Peak Foun	High	High	High	High
93.6	High	High	High	High	High	High	High	High
82.2	High	High	High	High	High	High	High	High
117.9	High	High	High	High	High	High	High	High
178.9	High	High	High	High	Not Found	Peak Foun	Not Found	High
100.2	High	High	High	Peak Foun	High	High	High	Peak Foun
104.3	High	High	High	High	High	High	High	High
102	High	High	High	High	High	High	High	High
115.7	High	High	High	High	High	High	High	High

95.4	High	High	High	High	High	High	High	High
101.7	High	High	High	High	High	High	High	High
143.7	High	High	High	High	Peak Foun	Peak Foun	Peak Foun	Peak Foun
137.8	High	High	High	High	Peak Foun	Peak Foun	Peak Foun	High
92.7	High	High	High	High	High	High	High	High
133.3	High	High	High	High	Peak Foun	Peak Foun	Peak Foun	Peak Foun
98.8	High	High	Peak Foun	High	High	High	Peak Foun	High
107.2	High	High	High	High	High	High	High	High
120.9	High	High	High	High	Peak Foun	High	Peak Foun	High
97.4	High	High	High	High	High	High	High	High
122.7	High	High	High	High	Peak Foun	High	High	High
99.4	High	High	High	High	High	High	High	High
153.3	High	High	High	High	High	High	High	High
101.9	High	High	High	High	High	High	High	High
109	High	High	High	High	High	High	High	High
117.5	High	High	High	High	High	High	High	High
107.3	High	High	High	High	High	High	High	High
98.3	High	High	High	High	High	Peak Foun	High	High
126.4	High	High	High	Peak Foun	Peak Foun	Peak Foun	Peak Foun	Peak Foun
102.4	High	High	High	High	High	High	Peak Foun	High
137.1	High	High	High	High	High	High	High	High
106	High	High	High	High	High	High	High	High
106.3	High	High	High	High	Peak Foun	High	High	High
69.9	High	High	High	High	High	High	High	High
146.4	High	High	High	High	Peak Foun	Peak Foun	High	High
78.9	High	High	High	High	High	High	High	High
148	Peak Foun	High	High	Peak Foun	Peak Foun	Peak Foun	Peak Foun	Peak Foun
112	High	High	High	High	High	High	High	High
154.4	High	High	High	High	Peak Foun	High	Peak Foun	High
118.3	High	High	High	High	High	High	High	High
96.8	High	High	High	High	Peak Foun	High	High	High
132.6	High	High	High	High	High	High	High	High
95.2	High	High	High	High	High	High	High	High
102.3	High	High	High	High	High	High	High	High
109.1	Peak Foun	Peak Foun	High	Peak Foun	Peak Foun	Peak Foun	Peak Foun	High
130	High	High	High	High	High	High	High	High
138.5	High	High	High	High	Not Found	Peak Foun	Peak Foun	Peak Foun
113.1	High	High	High	Peak Foun	Peak Foun	High	High	High
106.3	High	High	High	High	High	High	High	High
93.5	High	High	High	High	High	High	High	High
99.2	High	High	High	High	High	High	High	High
113.6	High	High	High	Peak Foun	Peak Foun	High	High	High
120	High	High	High	Peak Foun	Peak Foun	High	High	High
110.3	High	High	High	High	High	High	High	High
114.3	High	High	High	High	High	High	High	High

101.2	High	High	High	High	High	High	High	High
122.1	Peak Foun	Peak Foun	High	Peak Foun	Peak Foun	Peak Foun	Peak Foun	Peak Foun
108.7	High	High	High	High	High	High	High	High
154.1	High	High	High	High	Peak Foun	Peak Foun	Peak Foun	High
102.5	High	High	High	High	High	High	Peak Foun	High
145.4	Peak Foun	High	High	Peak Foun	High	High	High	Peak Foun
133.5	High	High	High	Peak Foun	Peak Foun	High	High	Peak Foun
94	High	High	High	High	High	High	High	High
116.9	High	High	High	High	High	High	High	High
83.3	High	High	High	High	High	High	High	High
98.1	High	High	High	High	High	High	High	High
107.5	High	High	High	High	High	High	Peak Foun	High
103.6	High	High	High	High	High	High	High	High
103.2	High	High	High	High	High	High	High	High
104.9	High	High	High	High	High	High	High	High
121.8	High	High	High	Peak Foun	High	High	High	Peak Foun
107.2	High	High	High	High	High	High	High	High
101.5	High	High	High	High	Peak Foun	High	High	High
106.1	High	High	High	High	Peak Foun	High	High	High
131.3	High	High	High	High	High	High	High	High
161.6	High	High	High	High	High	High	High	High
101.2	High	High	High	High	High	High	High	High
111.7	High	High	High	High	Peak Foun	Peak Foun	High	Peak Foun
110.1	High	High	High	High	High	High	High	High
83.1	High	High	High	High	High	High	High	High
137.9	High	High	High	High	High	High	Peak Foun	High
111	High	High	High	High	High	High	High	High
91.8	High	High	High	High	High	High	High	High
102.5	High	High	High	High	High	High	High	High
118.9	High	High	High	High	Peak Foun	High	High	High
91.9	High	High	High	High	High	High	High	High
125.7	High	High	High	High	High	High	High	High
110.7	High	High	High	High	High	High	High	Peak Foun
175	High	High	High	High	Peak Foun	Peak Foun	High	High
151.3	High	High	High	High	Peak Foun	Peak Foun	Peak Foun	High
91.9	High	High	High	High	High	High	High	High
86.4	High	High	High	High	High	High	High	High
108.7	High	High	High	High	High	High	High	High
127.6	High	High	High	High	High	High	High	High
97.4	High	High	High	High	Not Found	High	Peak Foun	High
85.2	High	High	High	High	High	High	High	High
122.8	High	High	High	High	Peak Foun	Peak Foun	High	High
111.2	High	High	High	High	High	High	High	High
129.5	High	High	High	High	High	Peak Foun	Peak Foun	High
101.6	High	High	High	High	High	Peak Foun	High	High

95.4	High	High	High	Peak Foun	High	High	High	High
119.5	High	High	High	High	High	High	High	High
98.5	High	High	High	High	High	High	High	High
108.6	High	High	High	Peak Foun	High	High	High	High
128.4	High	High	High	High	Peak Foun	Peak Foun	Peak Foun	High
104.8	High	High	High	High	High	High	High	High
99	High	High	High	High	High	High	High	High
107.1	High	High	High	High	High	High	High	High
123.3	High	Peak Foun	High	High	High	High	High	High
166.2	High	High	High	High	Not Found	Peak Foun	High	Peak Foun
106.5	High	High	High	High	High	High	High	High
141.6	High	High	High	High	High	High	High	High
130.4	Peak Foun	Peak Foun	Peak Foun	Peak Foun	Peak Foun	Peak Foun	Peak Foun	Peak Foun
124.6	High	High	High	High	Peak Foun	Peak Foun	High	High
61.9	High	High	High	Peak Foun	High	Peak Foun	High	Peak Foun
111.7	High	High	High	High	High	High	High	High
94.9	High	High	High	High	High	High	High	High
104.6	High	High	High	High	High	High	High	High
115.8	High	High	High	High	High	Peak Foun	Peak Foun	High
116.5	High	High	High	High	High	High	Peak Foun	High
111.8	High	High	High	High	High	High	High	High
112.4	High	High	High	High	Peak Foun	High	High	High
103.3	High	High	High	Peak Foun	High	High	High	High
102.1	High	High	High	High	High	High	High	High
113.5	High	High	Peak Foun	Peak Foun	High	High	High	Peak Foun
91.2	High	High	High	High	High	High	High	Peak Foun
107.8	High	High	High	High	High	High	High	High
108.9	High	High	High	High	High	High	High	High
126.3	High	High	High	High	High	High	High	High
105.1	High	High	High	Peak Foun	Peak Foun	High	High	High
105.6	High	High	High	Peak Foun	High	Peak Foun	High	High
95.2	High	High	High	High	High	High	High	High
98.5	High	High	High	High	Peak Foun	High	High	Peak Foun
130.3	High	High	High	High	Peak Foun	High	High	High
143.4	High	High	High	High	Peak Foun	Peak Foun	Peak Foun	High
131.8	High	High	High	High	High	High	High	High
148.7	High	High	High	High	Peak Foun	High	Peak Foun	High
93	High	High	High	High	High	High	High	High
113.1	High	High	High	High	High	High	High	High
131.7	High	High	High	High	Peak Foun	High	Peak Foun	High
110.6	High	High	High	High	High	High	High	High
109.5	High	High	High	High	High	High	High	High
108	High	High	High	High	High	High	High	High
99.2	High	High	High	Peak Foun	High	High	High	High
126	High	High	High	High	Peak Foun	High	High	High

96	Not Found	Not Found	High	Not Found	Not Found	Peak Foun	Peak Foun	Not Found
108.6	High	High	High	High	High	High	High	High
100.3	High	High	High	High	High	High	High	Peak Foun
91.3	High	High	High	High	High	High	High	High
127.4	High	High	High	High	High	Peak Foun	High	High
107.7	High	High	High	High	High	High	High	High
108.5	High	High	High	High	High	Peak Foun	High	High
111.9	High	High	High	Peak Foun	Peak Foun	High	High	Peak Foun
111.3	High	High	Peak Foun	High	Peak Foun	High	Peak Foun	High
124.6	High	High	High	High	High	Peak Foun	High	High
112.7	High	High	High	Peak Foun	Not Found	High	Peak Foun	Peak Foun
105.1	High	High	High	Peak Foun	High	High	Peak Foun	High
135.5	High	High	High	High	Peak Foun	Peak Foun	High	High
103.5	High	High	High	High	High	High	High	High
119.3	High	High	High	High	Peak Foun	Peak Foun	High	High
99.3	High	High	High	High	High	High	High	High
134.7	High	High	High	High	Peak Foun	Peak Foun	High	High
108.2	Peak Foun	Peak Foun	Peak Foun	Not Found	Not Found	Peak Foun	Peak Foun	Not Found
102.9	High	High	High	High	High	High	High	High
88.6	High	High	High	High	High	High	High	High
113.9	High	High	High	High	High	High	High	High
108.3	High	High	High	High	High	High	High	High
87.8	High	High	High	High	High	High	High	High
108.6	High	High	High	High	High	High	High	High
127.9	High	High	High	High	Peak Foun	Peak Foun	High	Peak Foun
126.4	High	High	High	High	High	High	High	High
64.7	Peak Foun	Peak Foun	Peak Foun	Peak Foun	High	High	High	Peak Foun
77	Peak Foun	High	High	High	High	High	High	High
112.3	High	High	High	High	High	High	High	Peak Foun
129.6	High	High	High	High	Peak Foun	High	High	High
93.8	High	High	High	High	High	High	High	High
93.9	High	High	High	Peak Foun	High	High	High	Peak Foun
73.8	High	High	High	High	High	High	High	High
112.9	High	High	High	High	High	High	High	High
132.8	Peak Foun	Peak Foun	High	Peak Foun	Peak Foun	Peak Foun	Peak Foun	Peak Foun
109.3	High	High	High	High	High	High	High	High
100	High	High	High	High	High	High	High	High
117.6	High	High	High	High	High	Peak Foun	Peak Foun	Peak Foun
97.9	Peak Foun	Peak Foun	Peak Foun	Peak Foun	Peak Foun	Peak Foun	Peak Foun	Peak Foun
108.8	High	High	High	High	High	High	High	High
104	High	High	High	High	High	High	High	High
154.9	High	High	High	High	Peak Foun	Peak Foun	Peak Foun	High
110.9	High	High	High	High	High	High	High	High
96	High	High	High	High	High	High	High	High
120.7	High	High	High	High	High	High	High	High

128.4	High	High	High	High	High	High	High	High
130.4	High	High	High	High	High	High	High	High
106.4	High	High	High	High	High	High	High	High
137.4	High	High	High	High	Peak Foun	Peak Foun	Peak Foun	Peak Foun
136.5	High	High	High	High	High	High	High	High
111	High	High	High	Peak Foun	High	High	Peak Foun	Peak Foun
104	High	High	High	High	High	High	High	Peak Foun
124.2	Peak Foun	Peak Foun	Peak Foun	Peak Foun	Peak Foun	Peak Foun	Peak Foun	Peak Foun
132.3	High	High	High	High	Peak Foun	High	High	High
115.8	High	High	High	High	High	High	High	High
107.8	High	High	High	High	High	High	High	High
107.3	High	High	High	High	High	High	High	High
115.6	High	High	High	High	High	High	High	High
101.3	High	High	High	High	High	High	High	High
113.5	High	High	High	High	Peak Foun	High	High	High
110.5	High	High	High	High	High	High	High	High
141.2	High	High	High	High	Peak Foun	High	Peak Foun	High
102.8	High	High	High	High	High	High	High	High
157.2	Peak Foun	Peak Foun	Peak Foun	Peak Foun	Peak Foun	Peak Foun	Peak Foun	Peak Foun
91.5	High	High	High	High	High	High	High	High
115.1	High	High	High	High	High	High	High	High
105.1	High	High	High	High	High	High	High	High
115.9	High	High	High	High	Peak Foun	Peak Foun	High	High
102.1	High	High	High	High	High	High	High	High
130.2	High	High	High	Peak Foun	Peak Foun	High	High	Peak Foun
106.7	High	High	High	High	High	High	High	High
186	High	High	High	High	Peak Foun	Peak Foun	Peak Foun	Peak Foun
81.9	High	High	High	High	High	High	High	High
112.7	High	High	High	High	High	High	High	High
111.6	High	High	High	High	High	High	High	High
120.5	High	High	High	High	High	High	Peak Foun	High
152.1	Peak Foun	High	Peak Foun	Peak Foun	Peak Foun	Peak Foun	Peak Foun	High
134.3	High	High	High	High	Peak Foun	High	High	High
87.3	High	High	High	High	High	High	High	High
106.3	High	High	High	High	High	High	High	High
80.6	Peak Foun	Not Found	Peak Foun	Not Found	Not Found	Not Found	Not Found	Not Found
116.7	Peak Foun	Peak Foun	High	Peak Foun	Peak Foun	Peak Foun	Peak Foun	Not Found
75.4	High	High	High	High	High	High	High	High
103	High	High	High	High	High	High	High	High
108.3	High	High	High	High	High	High	High	High
110.9	High	High	High	High	Peak Foun	Peak Foun	High	High
173.8	High	High	High	High	Peak Foun	Peak Foun	Peak Foun	Peak Foun
112.2	High	High	High	High	High	High	High	High
141.5	High	High	High	High	High	High	High	High
121.2	High	High	High	High	High	High	High	High

96.1	High	High	High	High	High	High	High	High
183.2	High	High	High	Peak Foun	Peak Foun	Peak Foun	Peak Foun	High
137.9	High	High	High	Peak Foun	Peak Foun	High	High	High
114.6	High	High	High	Peak Foun	High	High	High	Peak Foun
103.9	High	High	High	High	High	High	High	High
102.2	High	High	High	High	High	High	High	High
142.1	High	High	High	High	High	High	Peak Foun	High
118.5	High	High	Peak Foun	High	Peak Foun	Not Found	Not Found	High
133.5	High	High	High	Peak Foun	Peak Foun	Peak Foun	Peak Foun	High
115.2	High	High	High	High	Peak Foun	High	High	High
116.9	High	High	High	High	High	High	High	High
88.1	High	High	High	High	High	High	High	High
131.9	High	High	High	High	Peak Foun	High	High	High
145.9	High	High	High	High	Peak Foun	Peak Foun	High	High
103.7	High	High	High	High	Peak Foun	Peak Foun	High	Peak Foun
99.8	High	High	High	High	High	High	High	High
131.3	High	High	High	High	Peak Foun	High	Peak Foun	High
85.8	High	High	Peak Foun	Peak Foun	Peak Foun	High	High	High
95	High	High	High	Peak Foun	High	High	High	High
102	High	High	High	High	High	High	High	High
113.5	High	High	High	High	High	High	High	High
103.1	High	High	High	High	High	High	High	High
100.5	High	High	High	High	High	High	Peak Foun	High
111.7	High	High	High	High	High	High	High	High
104.5	High	High	High	High	High	High	High	High
102.4	High	High	High	High	High	High	High	High
102.2	High	High	High	High	High	High	High	High
130.5	High	High	High	Peak Foun	Peak Foun	High	High	High
132.2	High	High	High	High	High	Peak Foun	Peak Foun	Peak Foun
97.4	High	High	High	High	Peak Foun	High	High	High
106.6	High	High	High	High	High	High	High	High
141.9	High	High	High	Peak Foun	High	High	High	High
126	High	High	Not Found	Not Found	Peak Foun	High	High	Not Found
121.7	High	High	High	High	High	High	High	High
89.5	High	High	High	High	Peak Foun	High	High	High
106.8	High	High	High	High	High	Peak Foun	High	High
103	High	High	High	High	High	High	High	High
110.7	High	High	High	High	High	High	High	Peak Foun
121.2	High	High	High	High	High	High	High	High
108.3	High	High	High	Peak Foun	High	High	High	High
127.7	High	High	High	High	High	Peak Foun	High	High
118.7	High	High	High	High	High	High	High	High
106.1	Peak Foun	High	High	High	High	High	High	High
145.4	Not Found	Not Found	Not Found	Not Found	Not Found	Not Found	Not Found	Not Found
109.4	High	High	Peak Foun	High	High	High	Peak Foun	High

106.6	High	High	High	Peak Foun	High	Peak Foun	High	Peak Foun
88.7	Peak Foun	Peak Foun	High	Peak Foun	Not Found	High	High	Peak Foun
128	High	High	High	High	Peak Foun	High	High	High
139.6	High	High	Peak Foun	Peak Foun	High	Peak Foun	Peak Foun	Peak Foun
97.7	High	High	High	High	High	Peak Foun	High	Peak Foun
118.4	High	High	Peak Foun	High	Not Found	Peak Foun	Peak Foun	High
106.8	High	High	High	High	Peak Foun	High	High	High
144.2	High	High	High	High	High	High	High	High
105.4	High	High	High	High	High	High	High	High
106.1	High	High	High	High	High	High	Peak Foun	High
109.5	High	High	High	High	High	High	High	High
105.9	High	High	High	High	High	High	High	High
9.9	Peak Foun	Peak Foun	Peak Foun	Peak Foun	High	High	High	Peak Foun
128	High	High	High	High	High	High	High	High
120.2	High	High	High	High	High	High	Peak Foun	Peak Foun
140.4	High	High	High	High	High	High	High	High
130.3	High	High	High	Peak Foun	High	High	High	High
110.3	High	High	High	Peak Foun	High	High	High	High
184.6	High	High	High	High	Peak Foun	Peak Foun	Peak Foun	Peak Foun
101.4	High	High	High	High	High	High	High	High
98.3	High	High	High	High	High	High	High	High
113.4	High	High	High	High	High	High	High	High
109.8	High	High	High	Peak Foun	High	High	High	High
144.8	High	High	High	High	Peak Foun	High	Peak Foun	High
100.5	High	High	High	High	Peak Foun	High	High	High
143.8	High	High	Peak Foun	Peak Foun	Not Found	Not Found	Not Found	Peak Foun
112.3	High	High	High	High	High	High	High	High
94.1	High	High	High	Peak Foun	High	High	High	High
118.5	High	High	High	Peak Foun	High	High	High	High
137.1	High	High	High	Peak Foun	High	Peak Foun	High	High
110.5	High	High	High	Peak Foun	High	High	High	Peak Foun
118.5	High	High	High	High	Not Found	Not Found	Not Found	High
119.7	High	High	High	High	High	High	High	High
103.7	High	High	High	Peak Foun	High	High	High	High
106.1	High	High	High	High	High	High	High	High
126.1	High	High	High	High	High	High	High	High
111.5	High	High	High	High	High	High	High	High
125.2	High	High	Peak Foun	Peak Foun	Peak Foun	High	Not Found	High
154.6	High	High	High	High	High	High	High	High
96.7	High	High	Peak Foun	Peak Foun	Peak Foun	Peak Foun	High	High
115.4	High	High	High	High	High	Peak Foun	High	High
111.9	High	High	High	High	High	High	High	High
113.1	High	High	High	High	High	High	High	High
98.9	High	High	High	High	High	High	High	High
108.1	High	High	High	High	High	High	High	High

114.8	High	High	High	High	High	High	High	High
107.1	High	High	High	High	High	High	High	High
109.9	High	High	High	Peak Foun	Peak Foun	Peak Foun	High	High
120.5	High	High	High	High	Peak Foun	High	High	Peak Foun
128.2	High	High	High	Peak Foun	Peak Foun	High	High	Peak Foun
118	High	High	High	High	High	High	High	High
126.3	High	High	High	High	Peak Foun	High	High	High
144.8	High	High	High	High	High	High	High	High
116	High	High	High	Peak Foun	High	High	High	High
108.5	High	High	High	High	High	High	High	High
97.6	High	High	High	Peak Foun	High	High	High	High
95.4	High	High	High	High	High	High	High	High
120.9	High	High	High	High	High	High	High	High
113.3	High	High	High	High	High	High	High	High
78.1	High	High	High	Peak Foun	High	High	High	High
159.9	High	High	High	High	Peak Foun	High	High	Peak Foun
123.6	High	High	High	High	Peak Foun	High	High	High
106.6	High	High	High	High	High	High	High	High
107.9	High	High	High	High	High	High	High	High
99.6	High	High	High	High	High	High	High	High
148	High	High	High	High	Peak Foun	Peak Foun	High	High
116.9	High	High	High	High	High	High	High	High
114.6	High	High	High	Peak Foun	Peak Foun	High	High	High
131.3	High	High	High	High	Peak Foun	Peak Foun	Peak Foun	Peak Foun
146	High	High	High	High	Peak Foun	Peak Foun	High	Peak Foun
97	High	High	High	High	Peak Foun	High	High	Peak Foun
156.1	High	High	High	Peak Foun	Peak Foun	Peak Foun	Peak Foun	Peak Foun
114.2	High	High	High	High	High	High	High	High
134.7	High	High	High	High	High	High	High	High
107	High	High	High	High	High	High	High	High
76.4	High	High	High	Peak Foun	Peak Foun	High	High	High
170	High	High	High	High	Peak Foun	High	High	Peak Foun
117.8	High	Peak Foun	Peak Foun	High	Not Found	High	High	Peak Foun
108	High	High	High	Peak Foun	High	High	High	Peak Foun
106.4	Not Found	Not Found	Peak Foun	Not Found	High	Peak Foun	High	Peak Foun
96.6	High	High	High	High	High	High	High	High
120.4	High	High	High	Peak Foun	High	High	High	High
133.7	High	High	High	High	Peak Foun	Peak Foun	Peak Foun	High
147.3	Peak Foun	High	High	High	Peak Foun	Peak Foun	Peak Foun	Peak Foun
120.2	High	High	High	High	High	High	High	High
96.5	High	High	High	High	High	High	High	High
122.2	High	Peak Foun	Peak Foun	Peak Foun	High	Peak Foun	Peak Foun	Peak Foun
89.6	Peak Foun	Peak Foun	High	Peak Foun	High	High	High	High
112.5	High	High	High	High	High	High	High	High
114.6	High	High	High	Peak Foun	High	High	High	Peak Foun

98.3	High	High	High	High	High	High	Peak Foun	High
150.5	High	High	High	High	Peak Foun	Peak Foun	Peak Foun	High
105.4	High	High	High	High	High	High	High	High
170.2	High	High	High	Peak Foun	Peak Foun	Peak Foun	High	High
106.4	High	High	High	High	High	High	High	High
109.8	High	High	High	Peak Foun	Peak Foun	High	High	High
127.2	High	High	High	High	High	High	High	High
100	High	High	High	Peak Foun	High	High	High	Peak Foun
111.2	High	High	High	High	High	High	High	High
119.2	High	High	High	Peak Foun	Not Found	Peak Foun	Peak Foun	High
111.4	High	High	High	High	High	High	High	High
102.2	High	High	High	High	High	High	High	High
104.9	High	High	High	High	High	High	High	High
109.7	High	High	High	High	High	High	High	High
138	High	High	Peak Foun	Peak Foun	Peak Foun	Peak Foun	Peak Foun	Peak Foun
104.2	High	High	High	High	High	High	High	High
135.6	High	High	High	Peak Foun	High	Peak Foun	Peak Foun	High
84	High	High	High	High	High	High	High	High
119.8	High	High	High	Peak Foun	Peak Foun	Peak Foun	High	High
145.6	High	High	High	High	Peak Foun	Peak Foun	Peak Foun	Peak Foun
105.1	High	High	High	High	Peak Foun	High	High	High
168.6	High	High	High	High	Peak Foun	Peak Foun	Peak Foun	High
94.1	High	High	High	Peak Foun	High	High	High	High
116.5	High	High	High	High	Peak Foun	High	High	Peak Foun
100.7	High	High	High	High	High	High	High	High
121.4	High	High	High	High	Peak Foun	Peak Foun	High	Peak Foun
118	High	High	High	High	Peak Foun	Peak Foun	Peak Foun	Peak Foun
129.6	High	Peak Foun	Peak Foun	Peak Foun	Peak Foun	Not Found	Peak Foun	High
128.8	High	High	High	High	High	Peak Foun	High	High
147.3	High	High	High	High	Peak Foun	Peak Foun	Peak Foun	Peak Foun
111.1	High	High	High	High	High	High	High	High
91.1	High	High	High	High	High	High	High	High
141.3	High	High	High	High	High	High	High	High
110.2	High	High	High	High	High	High	High	High
94.6	High	High	High	High	High	High	High	High
106.3	High	High	High	High	High	High	High	High
112.3	High	High	High	Peak Foun	High	High	High	High
98.1	Peak Foun	Peak Foun	High	Not Found	Peak Foun	Not Found	Peak Foun	Not Found
135.3	High	High	High	High	Peak Foun	Peak Foun	High	High
130.2	High	High	High	High	Peak Foun	Peak Foun	Peak Foun	Peak Foun
132.8	High	High	Peak Foun	High	Peak Foun	Peak Foun	Peak Foun	Peak Foun
104.1	High	High	High	High	High	High	High	Peak Foun
117.3	High	High	High	High	High	Peak Foun	High	High
128.5	High	High	Peak Foun	Peak Foun	Not Found	High	Peak Foun	Peak Foun
110.6	High	High	High	High	High	High	High	High

121	High	High	High	High	High	High	High	High
110.6	High	High	High	High	Peak Foun	Peak Foun	Peak Foun	Peak Foun
115.2	High	High	High	Peak Foun	Peak Foun	Peak Foun	High	High
110.8	High	High	High	High	High	High	High	High
114.3	High	High	High	High	Peak Foun	High	High	High
106.6	High	High	High	High	High	High	High	High
81.3	Not Found	Peak Foun	Peak Foun	Not Found	Peak Foun	Peak Foun	Peak Foun	Not Found
128.8	High	High	High	High	High	High	High	High
100.6	High	High	High	High	High	High	High	High
131.3	High	High	High	High	Peak Foun	Peak Foun	High	High
118.7	High	High	High	Peak Foun	High	High	High	High
111.3	High	High	High	High	High	High	High	High
135	High	High	High	High	High	High	Peak Foun	High
135.1	High	High	High	High	Peak Foun	High	High	High
130.3	High	High	High	High	High	Peak Foun	High	High
120.5	High	High	Peak Foun	Peak Foun	Peak Foun	Peak Foun	Peak Foun	Peak Foun
154.1	High	High	High	Peak Foun	Peak Foun	Peak Foun	Peak Foun	Peak Foun
97	High	High	High	High	High	High	High	High
113.6	High	High	High	High	High	High	High	High
104.9	High	High	High	High	High	High	High	High
136.5	High	High	High	High	Peak Foun	High	High	High
69.9	High	High	High	High	High	High	High	High
90.9	High	High	High	High	High	High	High	High
95.8	High	High	High	High	High	High	High	High
107.8	High	High	High	High	Peak Foun	High	High	Peak Foun
99.3	High	High	High	High	High	High	High	High
106.1	High	High	High	High	Peak Foun	High	High	High
81.5	High	High	High	High	High	High	High	High
88.8	High	High	High	High	High	High	High	High
117.5	Peak Foun	High	Peak Foun	Peak Foun	High	Peak Foun	Peak Foun	Not Found
179.1	High	High	Peak Foun	Peak Foun	Peak Foun	Peak Foun	Peak Foun	Peak Foun
114.4	High	High	High	High	High	High	High	High
129.8	High	Peak Foun	High	High	High	Peak Foun	High	High
108.3	High	High	High	High	Not Found	High	Peak Foun	High
116	Peak Foun	Peak Foun	Peak Foun	Peak Foun	High	Peak Foun	Peak Foun	High
121.2	High	High	High	High	Peak Foun	Peak Foun	Peak Foun	Peak Foun
153.6	High	High	High	High	Peak Foun	Peak Foun	Peak Foun	Peak Foun
104.1	High	High	High	High	High	High	High	High
92	Peak Foun	Peak Foun	Peak Foun	Not Found	Peak Foun	Peak Foun	Peak Foun	Peak Foun
105.8	High	High	High	High	High	High	High	High
107.6	High	Peak Foun	High	Peak Foun	High	High	High	High
114.1	High	High	High	High	High	High	Peak Foun	High
123.5	High	High	High	High	Peak Foun	High	High	High
114.1	High	High	High	High	Peak Foun	Peak Foun	High	High
95.1	High	High	High	Peak Foun	High	High	High	High

125.8	High	High	High	High	Peak Foun	Peak Foun	Not Found	High
106.2	High	High	High	High	High	High	High	High
104.1	High	High	High	High	High	High	High	High
143.5	High	Peak Foun	High	Peak Foun	Peak Foun	Peak Foun	Peak Foun	Peak Foun
118.7	High	High	High	High	Peak Foun	High	High	High
103.9	High	High	High	Peak Foun	Peak Foun	Peak Foun	Peak Foun	Peak Foun
104.8	High	High	High	High	High	High	High	High
123.4	High	High	High	High	High	High	High	High
139.9	High	High	High	High	Peak Foun	High	High	High
121.5	High	High	High	High	High	Peak Foun	Peak Foun	High
120.9	High	High	High	High	High	Peak Foun	High	Peak Foun
134.1	High	High	High	Peak Foun	Peak Foun	Peak Foun	Peak Foun	High
137.6	High	High	High	High	High	High	High	High
97.3	High	High	Peak Foun	Peak Foun	High	High	High	Peak Foun
109	High	High	High	High	High	High	High	High
121.5	High	High	High	High	Peak Foun	High	Peak Foun	Peak Foun
110.5	High	High	High	Peak Foun	High	Peak Foun	Peak Foun	High
99.4	High	High	High	High	High	High	High	High
106.3	High	High	High	High	Peak Foun	Not Found	Peak Foun	Not Found
90.6	High	High	High	High	High	High	High	High
112.2	High	High	High	High	Peak Foun	High	High	High
123.7	High	High	High	High	High	High	High	High
115.2	High	High	High	High	Peak Foun	High	High	High
106.4	High	High	High	High	Peak Foun	High	High	Peak Foun
132.8	High	High	High	High	Peak Foun	Peak Foun	Peak Foun	Peak Foun
126.1	High	High	High	High	High	High	High	High
174.4	High	High	High	High	Peak Foun	Peak Foun	Peak Foun	Peak Foun
110.9	Peak Foun	High	High	High	Not Found	Peak Foun	Peak Foun	Peak Foun
145.5	High	High	High	High	High	High	High	High
121.7	High	Peak Foun	High	Peak Foun	Peak Foun	Peak Foun	Peak Foun	Peak Foun
103.6	High	High	Peak Foun	Peak Foun	High	Peak Foun	Peak Foun	High
101.6	High	High	High	High	High	High	High	High
129	High	High	High	High	Peak Foun	Peak Foun	Peak Foun	Peak Foun
101.6	Peak Foun	Peak Foun	High	High	Peak Foun	High	High	Peak Foun
111.8	High	High	High	Peak Foun	Peak Foun	Peak Foun	High	Peak Foun
115.7	High	High	High	High	Peak Foun	High	High	High
135.4	High	High	High	Peak Foun	High	High	High	High
108.1	High	High	High	High	High	High	High	Peak Foun
135.4	High	High	High	High	Peak Foun	High	High	High
102.1	High	High	High	High	High	High	High	High
102.8	High	High	High	High	High	High	High	High
106.2	High	High	High	High	High	High	High	High
123.6	High	High	High	High	Peak Foun	Peak Foun	High	High
101.2	High	High	High	High	High	High	High	High
94	High	High	High	High	High	High	High	High

89.6	High	High	Peak Foun	Peak Foun	High	High	Peak Foun	High
86.3	High	High	High	High	High	High	High	High
133.2	High	High	High	High	Peak Foun	High	Peak Foun	High
135.8	High	High	High	High	High	High	High	High
128.3	High	High	High	Peak Foun	Peak Foun	Peak Foun	High	Peak Foun
109.7	High	High	High	High	High	High	High	High
114.5	High	High	High	High	Peak Foun	Peak Foun	Peak Foun	Peak Foun
102.3	High	High	High	High	High	High	High	High
103.1	High	High	High	High	High	High	High	High
110.7	High	High	High	Peak Foun	High	High	High	High
116.3	High	High	High	High	High	High	Peak Foun	High
113.1	High	High	High	High	Peak Foun	High	High	High
145	High	High	Peak Foun	Peak Foun	Peak Foun	Peak Foun	Peak Foun	Peak Foun
112.8	High	Peak Foun	High	Peak Foun	High	Peak Foun	Peak Foun	Peak Foun
109.6	High	High	High	Peak Foun	Peak Foun	High	Peak Foun	High
136.1	High	High	High	Peak Foun	Not Found	Peak Foun	Peak Foun	Peak Foun
133.2	High	High	High	High	Not Found	Peak Foun	High	Not Found
145.2	High	High	High	High	High	High	High	High
92.1	High	High	High	High	Peak Foun	Peak Foun	High	High
102.9	High	High	High	High	High	High	High	High
149.1	High	High	High	High	High	Peak Foun	Peak Foun	High
109.2	Peak Foun	Peak Foun	High	Peak Foun	High	High	High	Peak Foun
135.7	High	High	High	Peak Foun	Peak Foun	Peak Foun	Peak Foun	Peak Foun
114.9	High	High	Peak Foun	Peak Foun	High	High	Peak Foun	High
126.8	High	High	High	Peak Foun	High	High	Peak Foun	Peak Foun
101.8	High	High	Peak Foun	Peak Foun	Not Found	Peak Foun	Not Found	Peak Foun
147.4	High	High	Peak Foun	Peak Foun	Peak Foun	High	Peak Foun	Peak Foun
136.1	High	High	High	Peak Foun	Not Found	Peak Foun	Peak Foun	Peak Foun
94.3	High	High	High	High	High	High	High	High
119.3	High	High	High	High	High	High	High	High
142.5	High	High	High	Peak Foun	Peak Foun	Peak Foun	Peak Foun	Peak Foun
121.6	High	High	High	High	Peak Foun	High	Peak Foun	Peak Foun
103.7	High	High	High	High	Peak Foun	High	High	High
136.3	High	High	Peak Foun	Peak Foun	High	High	High	Peak Foun
138.5	High	High	High	High	High	High	High	High
142.1	Peak Foun	High	High	Not Found	Peak Foun	Peak Foun	Peak Foun	Peak Foun
124.7	High	High	High	High	Peak Foun	Peak Foun	High	High
108.2	High	High	High	High	High	High	High	High
136.4	High	High	High	High	High	High	High	High
116.9	High	High	High	High	Peak Foun	Peak Foun	High	High
106.2	High	High	High	High	High	High	High	High
122.5	High	High	High	High	High	High	High	High
108.6	High	High	High	High	High	High	High	High
171.7	High	High	High	High	Not Found	Not Found	Peak Foun	Peak Foun
139.7	High	Peak Foun	Peak Foun	Peak Foun	Peak Foun	Peak Foun	Peak Foun	Peak Foun

150.7	High	Peak Foun	High	High	Peak Foun	Peak Foun	Peak Foun	Peak Foun
105.2	High	High	High	High	High	Peak Foun	Peak Foun	Peak Foun
123.1	High	High	High	High	Peak Foun	Peak Foun	High	High
114.4	High	High	High	High	High	High	High	High
102.6	High	High	High	Peak Foun	High	High	High	High
114	High	High	High	High	Peak Foun	Peak Foun	High	High
102.1	High	High	High	Peak Foun	Peak Foun	High	High	High
105.2	High	High	High	High	High	High	High	High
122.1	High	High	Peak Foun	Peak Foun	High	Peak Foun	Peak Foun	Peak Foun
112.7	Peak Foun	Peak Foun	Peak Foun	Peak Foun	Peak Foun	Peak Foun	Peak Foun	Peak Foun
120.6	High	High	High	High	Peak Foun	High	High	Peak Foun
114.2	High	High	High	High	High	Peak Foun	Peak Foun	Peak Foun
138.4	High	High	High	High	Peak Foun	Peak Foun	Peak Foun	High
89.2	High	High	High	Peak Foun	High	High	High	High
151.2	High	Peak Foun	High	High	Peak Foun	Peak Foun	Peak Foun	Peak Foun
110	High	High	Peak Foun	Peak Foun	High	Peak Foun	High	High
110	High	High	High	High	High	High	High	High
131.3	High	High	High	High	Peak Foun	Peak Foun	Peak Foun	Peak Foun
138.8	High	High	Peak Foun	Peak Foun	Peak Foun	Peak Foun	Not Found	Peak Foun
121.1	High	High	High	High	Peak Foun	High	High	High
91.2	High	High	High	High	High	High	High	High
104.1	High	High	High	High	Peak Foun	High	Peak Foun	High
98.7	High	High	High	Peak Foun	High	High	High	High
110.6	High	Peak Foun	High	High	High	High	High	High
156.7	High	High	High	High	Peak Foun	Peak Foun	Peak Foun	Peak Foun
143.8	High	High	High	High	Peak Foun	Peak Foun	Peak Foun	Peak Foun
90.8	Peak Foun	High	High	High	Peak Foun	Peak Foun	High	High
123.7	High	High	High	High	Peak Foun	Peak Foun	High	High
62.4	High	High	High	Peak Foun	High	High	High	Peak Foun
39.8	High	High	High	High	Peak Foun	Peak Foun	High	Peak Foun
96.7	High	High	High	High	High	High	High	High
62.9	High	High	High	High	High	High	Peak Foun	Peak Foun
126.8	High	High	High	High	Peak Foun	High	High	High
125.7	High	High	High	High	Peak Foun	Peak Foun	High	Peak Foun
124.8	High	High	Peak Foun	Peak Foun	Peak Foun	Peak Foun	Peak Foun	High
121	High	High	High	High	Peak Foun	High	Peak Foun	Peak Foun
104.3	Peak Foun	High	Peak Foun	High	High	Peak Foun	Peak Foun	Peak Foun
118.4	High	High	High	High	High	Peak Foun	Peak Foun	High
133.4	High	High	High	High	Peak Foun	Peak Foun	Peak Foun	High
82.4	High	High	High	High	High	High	High	High
78.4	High	High	High	High	High	High	High	High
91.3	High	Peak Foun	High	High	High	High	High	High
102	Peak Foun	High	High	High	Peak Foun	High	High	High
104.1	High	High	High	High	High	High	High	High
105.7	Peak Foun	High	High	High	High	Peak Foun	Peak Foun	High

113.4	High	High	High	High	High	High	High	High
112.1	High	High	Peak Foun	Peak Foun	High	High	High	High
109.9	High	High	High	High	High	High	High	High
106.5	High	High	High	High	High	High	High	High
115.6	High	High	High	Peak Foun	Peak Foun	Peak Foun	Peak Foun	High
81.3	Peak Foun	Peak Foun	High	Peak Foun	Peak Foun	High	Peak Foun	Peak Foun
104.5	High	High	High	High	High	High	Peak Foun	High
115.9	High	High	High	High	High	High	High	High
33.8	Peak Foun	Peak Foun	Peak Foun	Peak Foun	High	Peak Foun	Peak Foun	Peak Foun
127.3	High	High	High	High	Peak Foun	Peak Foun	Peak Foun	Peak Foun
120.2	High	High	High	Peak Foun	Peak Foun	Peak Foun	Peak Foun	Peak Foun
115.8	High	High	High	High	Peak Foun	Peak Foun	High	Peak Foun
112.8	High	High	High	High	High	High	High	High
83.8	High	High	Peak Foun	High	Not Found	Peak Foun	Peak Foun	High
112.7	High	High	High	Peak Foun	High	Peak Foun	High	High
151	High	High	High	Peak Foun	High	Peak Foun	Peak Foun	Peak Foun
80.6	High	High	High	Not Found	Not Found	Not Found	Not Found	Not Found
108.9	High	High	High	High	High	Peak Foun	High	High
95	High	High	High	Peak Foun	Peak Foun	High	High	Peak Foun
121.1	High	High	High	High	Peak Foun	High	High	High
143.8	High	High	High	Peak Foun	Peak Foun	Peak Foun	High	High
77.6	Not Found	Not Found	Not Found	High	Not Found	Not Found	Not Found	Peak Foun
88.7	High	High	High	Peak Foun	High	High	High	Peak Foun
129.4	High	High	High	High	Peak Foun	High	High	High
109.2	High	High	High	High	Peak Foun	High	High	High
114.2	High	High	High	High	Peak Foun	High	High	High
136.7	High	High	High	Peak Foun	Peak Foun	High	High	High
155.2	Peak Foun	Peak Foun	Peak Foun	Peak Foun	Peak Foun	Peak Foun	Peak Foun	Peak Foun
116.2	High	High	High	High	Peak Foun	Peak Foun	High	High
133.8	High	Peak Foun	Peak Foun	Peak Foun	High	Peak Foun	High	Peak Foun
145.8	High	High	High	High	Peak Foun	Peak Foun	Peak Foun	High
98.3	High	High	High	High	High	High	High	High
96.6	High	Not Found	High	Not Found	Peak Foun	Peak Foun	High	Not Found
129.1	High	High	High	High	High	High	High	High
106.8	High	High	High	Peak Foun	Peak Foun	High	High	High
115.6	High	High	High	High	High	High	High	High
103.5	High	High	High	Peak Foun	High	High	Peak Foun	Peak Foun
73.5	High	Peak Foun	High	High	Peak Foun	Peak Foun	Peak Foun	High
109.7	High	High	High	High	High	High	High	High
105.9	High	High	High	High	Peak Foun	High	High	High
111.6	High	High	High	High	High	High	High	High
100.4	High	High	High	High	High	High	High	High
200	Not Found	Not Found	Not Found	Not Found	Not Found	Not Found	Not Found	Not Found
134.3	High	High	High	High	High	High	High	High
118.8	High	High	High	High	High	High	High	High

153.8	High	Peak Foun	High	High	High	Peak Foun	High	High
122.6	Peak Foun	High	High	Peak Foun	Peak Foun	High	Peak Foun	High
117.6	High	High	High	Peak Foun	Peak Foun	Peak Foun	High	Peak Foun
121.4	High	High	High	High	High	High	High	High
146.5	High	High	High	Peak Foun	Peak Foun	Not Found	Peak Foun	Peak Foun
156.6	High	High	High	High	Not Found	Peak Foun	Peak Foun	Peak Foun
90	High	High	High	High	High	High	High	High
110	High	High	High	High	High	Peak Foun	High	Peak Foun
127	High	High	High	Peak Foun	Peak Foun	High	High	High
101.3	High	High	Peak Foun	Peak Foun	High	High	High	Peak Foun
152.8	Peak Foun	Peak Foun	Peak Foun	Peak Foun	Peak Foun	Peak Foun	Peak Foun	High
107.7	High	High	High	High	High	High	High	High
120.9	Peak Foun	High	High	Peak Foun	Peak Foun	Peak Foun	Peak Foun	Peak Foun
130	High	High	Peak Foun	High	High	High	Peak Foun	High
107.4	High	High	High	Peak Foun	Not Found	Peak Foun	Peak Foun	High
102.1	Peak Foun	Peak Foun	Peak Foun	High	High	High	High	High
106.7	High	High	High	Peak Foun	Peak Foun	High	Peak Foun	High
103.7	High	High	High	High	High	High	High	High
68.5	High	High	High	High	Peak Foun	High	High	High
179.5	High	High	High	High	High	Peak Foun	Peak Foun	Peak Foun
92	High	High	High	High	Peak Foun	Peak Foun	Peak Foun	Peak Foun
112.7	High	High	High	Peak Foun	Peak Foun	Peak Foun	High	High
113.6	High	Peak Foun	High	Peak Foun	High	High	High	High
109.5	High	High	High	Peak Foun	Peak Foun	High	High	High
102.5	High	High	High	Peak Foun	Peak Foun	High	High	High
120.7	High	High	High	Peak Foun	Peak Foun	Peak Foun	Peak Foun	High
152.3	High	High	High	Peak Foun	Peak Foun	Not Found	Peak Foun	Not Found
96.4	High	High	High	High	High	High	High	High
118.8	High	High	High	High	Peak Foun	Peak Foun	High	High
110.8	High	High	High	Peak Foun	Peak Foun	High	Peak Foun	Peak Foun
140.2	High	High	High	Peak Foun	Peak Foun	Peak Foun	High	High
131.4	High	High	High	High	High	High	High	High
114.5	High	High	High	High	High	High	High	High
102	High	High	High	High	High	High	High	High
102.6	High	High	High	High	Peak Foun	Peak Foun	Peak Foun	Peak Foun
162	High	High	High	High	Peak Foun	Peak Foun	Peak Foun	High
103.1	High	High	Peak Foun	Peak Foun	High	High	High	High
111	High	High	High	High	Peak Foun	Peak Foun	High	High
124.4	Peak Foun	High	High	High	Peak Foun	Peak Foun	Peak Foun	Peak Foun
122.5	High	Peak Foun	High	Peak Foun	Peak Foun	Peak Foun	Peak Foun	Peak Foun
167.3	Peak Foun	Peak Foun	High	Peak Foun	Not Found	Not Found	Peak Foun	Peak Foun
113.5	High	High	High	Peak Foun	Peak Foun	High	High	Peak Foun
99.3	High	High	High	High	High	Peak Foun	Peak Foun	High
108	High	High	High	Peak Foun	High	High	High	Peak Foun
100.4	High	High	High	Peak Foun	High	High	High	High

105	High	High	High	High	High	High	High	High
81.6	Not Found	Not Found	Peak Foun	Not Found	Not Found	Not Found	Not Found	Not Found
92.4	High	High	High	High	High	High	High	Peak Foun
95.4	High	High	Peak Foun	Peak Foun	High	Peak Foun	Peak Foun	High
103.6	High	High	High	High	High	High	Peak Foun	High
127.6	High	High	High	High	High	High	High	High
94.2	High	High	High	High	High	High	High	Peak Foun
57.1	High	Peak Foun	Peak Foun	Peak Foun	Peak Foun	Peak Foun	Peak Foun	Peak Foun
104.9	High	Peak Foun	High	Peak Foun	High	High	High	Peak Foun
173.5	Peak Foun	Peak Foun	High	Peak Foun	Peak Foun	Peak Foun	Not Found	Peak Foun
119	High	High	High	Peak Foun	Peak Foun	Peak Foun	Peak Foun	High
120.6	High	High	Peak Foun	Peak Foun	Peak Foun	Peak Foun	Peak Foun	High
108.8	High	High	High	High	Peak Foun	Peak Foun	High	High
114.6	High	High	High	High	High	High	High	High
131.5	High	High	High	Peak Foun	High	Peak Foun	High	Peak Foun
115.6	High	High	High	High	High	High	High	High
112.5	High	High	High	Peak Foun	Peak Foun	High	Peak Foun	Peak Foun
138.1	High	High	Peak Foun	Not Found	Peak Foun	Peak Foun	Not Found	Not Found
68	Not Found	High	High	Peak Foun	High	High	Peak Foun	Peak Foun
116.3	High	High	High	High	Peak Foun	Peak Foun	High	Peak Foun
137.6	High	High	High	High	High	High	High	High
137.8	High	Peak Foun	High	Peak Foun	Peak Foun	Peak Foun	Peak Foun	Peak Foun
106.4	High	High	High	High	High	Peak Foun	High	High
107	Peak Foun	High	High	High	Not Found	Peak Foun	Not Found	High
110.1	High	High	High	Peak Foun	High	Peak Foun	Peak Foun	Peak Foun
99.1	High	High	High	High	High	Peak Foun	High	High
198.7	Peak Foun	Peak Foun	Peak Foun	Peak Foun	Peak Foun	Peak Foun	Peak Foun	Peak Foun
102	High	High	High	High	Peak Foun	Peak Foun	Peak Foun	High
132.5	High	High	High	High	High	High	Peak Foun	High
131.3	High	High	High	High	Peak Foun	High	Peak Foun	Peak Foun
121.1	High	High	High	Peak Foun	High	High	High	Peak Foun
116	High	High	High	High	High	High	High	High
84.8	High	High	High	High	Peak Foun	High	High	High
77.5	High	High	High	High	High	High	High	Peak Foun
101.9	High	High	High	Peak Foun	Peak Foun	High	High	High
171.6	High	High	High	High	Peak Foun	Peak Foun	Peak Foun	High
105.4	High	High	High	High	High	High	High	High
108.2	High	High	High	High	Peak Foun	Peak Foun	Peak Foun	High
118.6	High	High	High	Peak Foun	Peak Foun	Peak Foun	Peak Foun	Peak Foun
104.6	High	High	High	High	High	High	High	High
107.4	Peak Foun	Peak Foun	Peak Foun	Peak Foun	High	High	Peak Foun	Peak Foun
115.9	High	High	High	Peak Foun	High	High	High	High
117.3	High	High	High	High	Peak Foun	Peak Foun	Peak Foun	High
101.8	High	High	High	Peak Foun	Peak Foun	High	High	Peak Foun
102	High	High	Peak Foun	High	Not Found	Peak Foun	Not Found	Peak Foun

116.9	High	High	High	Peak Foun	Peak Foun	Peak Foun	High	High
134.7	High	High	High	Peak Foun	Not Found	Not Found	Not Found	Not Found
85.6	Peak Foun	High	High	High	Peak Foun	Peak Foun	High	High
181.5	High	High	High	Peak Foun	Peak Foun	Peak Foun	Peak Foun	Peak Foun
113.6	Peak Foun	Peak Foun	Peak Foun	Peak Foun	High	High	High	Peak Foun
150.2	High	High	High	High	Peak Foun	Peak Foun	Peak Foun	High
113.4	High	Peak Foun	High	Not Found	Peak Foun	Peak Foun	High	Peak Foun
116.5	High	High	High	High	High	High	High	High
125.3	High	High	High	High	High	High	High	High
124.4	High	High	High	High	High	Peak Foun	High	High
99.3	Not Found	Peak Foun	Peak Foun	High	Peak Foun	Peak Foun	Peak Foun	Peak Foun
120.3	High	Peak Foun	High	Peak Foun	Peak Foun	Not Found	Peak Foun	High
132.2	High	Peak Foun	High	Peak Foun	Not Found	Peak Foun	High	Peak Foun
102	High	High	High	High	High	High	High	High
58.5	High	High	High	High	High	High	High	High
63.3	High	High	High	High	High	High	High	Peak Foun
102	High	High	High	Peak Foun	Peak Foun	Not Found	Not Found	Peak Foun
96.5	High	High	High	High	High	High	High	High
107	High	High	High	High	High	High	High	High
106.2	High	High	High	High	High	High	High	High
112.4	High	High	High	High	High	High	High	High
79.2	High	High	High	High	High	High	High	Peak Foun
106.6	High	High	High	High	Peak Foun	Peak Foun	High	High
106	High	High	High	Peak Foun	Peak Foun	High	High	High
132	High	High	High	High	Peak Foun	Peak Foun	Peak Foun	High
123	High	High	High	High	High	High	High	High
102.9	High	High	High	High	High	High	High	High
188.1	High	High	High	High	Peak Foun	Not Found	Peak Foun	Peak Foun
119.5	High	High	High	High	High	High	High	High
103.2	High	High	High	High	Peak Foun	High	Peak Foun	Peak Foun
121.2	High	Peak Foun	High	High	High	High	High	High
84.3	High	High	High	High	High	High	High	Peak Foun
137.6	High	High	High	Peak Foun	High	High	Peak Foun	Peak Foun
100.9	High	Peak Foun	High	Peak Foun	High	High	Peak Foun	Peak Foun
112.2	Peak Foun	High	High	High	Peak Foun	High	High	High
121.7	High	High	High	Peak Foun	Peak Foun	Peak Foun	Peak Foun	High
109.2	High	High	High	High	High	High	High	High
82.3	High	Not Found	High	High	Peak Foun	Not Found	Peak Foun	Peak Foun
104.3	High	High	Peak Foun	Peak Foun	High	High	High	Peak Foun
35.6	High	Peak Foun	Peak Foun	Peak Foun	High	High	High	Not Found
101.6	High	Peak Foun	High	Peak Foun	High	High	Peak Foun	High
88.8	Peak Foun	Peak Foun	Peak Foun	Peak Foun	Peak Foun	High	High	Peak Foun
91.5	High	High	High	High	High	High	High	Peak Foun
117.7	High	High	High	High	High	High	High	High
107.1	High	High	High	High	High	High	High	High

96.7	High	High	High	High	Peak Foun	High	Peak Foun	Peak Foun
104.7	Peak Foun	High	High	High	Peak Foun	High	High	High
103.3	High	High	High	High	High	High	High	High
148.3	High	High	High	High	Peak Foun	High	High	High
153.2	Peak Foun	High	High	Peak Foun	Peak Foun	Peak Foun	High	Peak Foun
124.7	High	High	High	High	High	Peak Foun	High	High
76	High	High	High	High	High	High	High	Peak Foun
124.3	High	Peak Foun	High	Peak Foun	Peak Foun	Peak Foun	Peak Foun	High
140.5	High	High	High	High	Not Found	Not Found	Peak Foun	Peak Foun
106.3	High	High	High	High	High	High	High	High
123.9	High	High	High	High	High	High	Peak Foun	High
107.8	High	High	High	High	High	High	High	High
142.4	High	Peak Foun	Peak Foun	Not Found	Not Found	Not Found	Peak Foun	Not Found
98.1	Peak Foun	High	High	Peak Foun	High	High	Peak Foun	Peak Foun
98.3	High	High	High	High	High	High	High	High
88.7	High	Peak Foun	High	Peak Foun	High	Peak Foun	Peak Foun	High
131.3	High	High	High	High	Peak Foun	High	High	High
104.7	High	High	High	Peak Foun	Not Found	Not Found	Not Found	Not Found
125.2	High	High	High	High	High	High	High	High
126	High	High	High	High	High	Peak Foun	Peak Foun	High
159.6	High	Peak Foun	High	Peak Foun	Peak Foun	Peak Foun	High	Peak Foun
111.6	High	High	High	High	High	High	High	High
82.6	High	High	High	Peak Foun	Peak Foun	High	High	Peak Foun
71.8	High	High	High	High	High	High	High	High
116.2	High	High	Peak Foun	Peak Foun	Peak Foun	High	High	Peak Foun
91.1	High	High	High	High	High	Peak Foun	High	Peak Foun
95.5	High	High	High	Peak Foun	Not Found	Peak Foun	Peak Foun	Peak Foun
102.8	High	High	High	Peak Foun	High	High	Peak Foun	Peak Foun
140.7	High	High	High	High	Peak Foun	High	Peak Foun	High
118.8	High	Not Found	High	High	High	Not Found	High	Not Found
105.3	High	High	High	High	Peak Foun	High	High	High
144.8	High	High	High	High	Peak Foun	High	High	High
77.2	High	High	Peak Foun	High	Peak Foun	Peak Foun	Peak Foun	High
107.1	High	High	Peak Foun	High	Peak Foun	High	Peak Foun	Peak Foun
95.1	High	High	High	High	Peak Foun	Peak Foun	Peak Foun	Peak Foun
139.6	High	High	High	Peak Foun	Peak Foun	High	Peak Foun	High
115.7	High	High	Peak Foun	High	Peak Foun	Not Found	Not Found	Peak Foun
94.4	Peak Foun	Peak Foun	Peak Foun	High	Peak Foun	Peak Foun	Peak Foun	Peak Foun
95.2	High	High	High	Peak Foun	Peak Foun	High	High	High
106.6	High	High	High	High	Peak Foun	Peak Foun	Peak Foun	Peak Foun
90.4	High	High	High	High	High	High	High	High
128.9	High	High	High	High	High	High	High	High
123.5	High	High	High	High	Peak Foun	High	High	High
122.7	High	High	High	Peak Foun	Not Found	Peak Foun	Peak Foun	High
157.9	High	High	High	High	Peak Foun	Peak Foun	Peak Foun	High

109.1	High	High	Peak Foun	Peak Foun	High	High	High	Peak Foun
101.9	High	High	Peak Foun	Peak Foun	High	High	Peak Foun	Peak Foun
103.5	High	Peak Foun	Peak Foun	Peak Foun	High	Peak Foun	High	Peak Foun
98	High	High	High	High	High	High	High	High
128.6	Peak Foun	Peak Foun	High	Peak Foun	Peak Foun	Peak Foun	Peak Foun	Peak Foun
127	High	High	High	High	High	High	Peak Foun	High
121	High	High	High	High	High	High	High	High
113.1	High	Peak Foun	High	High	High	Peak Foun	High	High
129.2	High	High	High	High	Peak Foun	Peak Foun	High	High
113.7	High	High	High	High	High	High	High	High
86.2	Peak Foun	Not Found	High	Not Found	High	High	High	High
108.1	High	Peak Foun	High	Not Found	High	High	High	Peak Foun
92.6	High	High	High	High	High	High	High	High
126.8	Peak Foun	Peak Foun	High	High	Not Found	Peak Foun	Peak Foun	Peak Foun
126.7	High	High	High	Peak Foun	Peak Foun	High	Peak Foun	High
116.4	High	Peak Foun	High	High	High	High	Peak Foun	High
107.2	High	High	High	High	High	Peak Foun	Peak Foun	High
108.9	High	High	High	High	Peak Foun	High	High	High
98.4	High	High	Peak Foun	Peak Foun	High	High	High	Peak Foun
124.7	Peak Foun	High	High	High	Peak Foun	High	High	Peak Foun
143.4	High	Peak Foun	High	Peak Foun	Peak Foun	Peak Foun	Peak Foun	Peak Foun
108.7	High	High	High	High	High	High	High	High
93.6	Peak Foun	Peak Foun	Peak Foun	Peak Foun	High	High	Peak Foun	Peak Foun
101.7	High	High	High	High	High	High	High	High
121.8	High	High	High	High	High	High	High	High
121.6	High	High	Peak Foun	High	High	Peak Foun	Not Found	Peak Foun
147.6	High	High	High	High	High	High	High	Peak Foun
101.7	High	High	High	High	High	High	High	High
102.4	High	High	Peak Foun	Peak Foun	High	High	High	High
121.4	High	High	High	High	High	High	High	High
97.4	High	High	High	High	Peak Foun	Peak Foun	Not Found	Not Found
109.7	Peak Foun	Peak Foun	Peak Foun	Peak Foun	High	High	Peak Foun	Peak Foun
114.1	High	High	High	Peak Foun	Peak Foun	High	High	Peak Foun
120.1	High	High	High	High	High	Peak Foun	High	High
97.3	High	High	High	High	High	High	High	High
92.8	High	High	High	High	High	High	High	High
117.3	Peak Foun	High	Not Found	Peak Foun	Not Found	Not Found	Not Found	High
84.1	Peak Foun	Peak Foun	Peak Foun	Peak Foun	High	High	High	Peak Foun
114.8	High	High	High	High	Peak Foun	High	High	High
105.9	High	High	High	High	Peak Foun	High	Peak Foun	High
112.5	High	High	High	High	Peak Foun	High	High	High
79.2	High	High	High	Peak Foun	High	High	High	High
62.1	High	High	High	High	High	Peak Foun	High	High
116.9	High	High	High	Peak Foun	High	High	High	High
113.9	High	High	High	High	High	Peak Foun	Peak Foun	Peak Foun

101.4	High	High	High	High	High	High	High	High
131	High	High	High	High	Peak Foun	Peak Foun	Peak Foun	High
134.9	High	High	High	High	Peak Foun	Peak Foun	High	Peak Foun
130.2	High	High	High	High	High	High	High	High
110.7	High	High	High	High	High	High	High	High
130.3	High	High	High	Peak Foun	Peak Foun	Peak Foun	Peak Foun	Peak Foun
111.1	High	High	High	High	Peak Foun	Peak Foun	High	Peak Foun
132.5	High	High	High	High	Peak Foun	Peak Foun	Peak Foun	Peak Foun
100.7	High	High	High	High	High	High	High	High
116.7	High	High	High	High	High	High	Peak Foun	High
112.5	High	High	High	High	High	Peak Foun	Peak Foun	Peak Foun
105.2	High	High	High	Peak Foun	High	High	Peak Foun	Peak Foun
98.5	Peak Foun	High	High	Peak Foun	High	High	High	Peak Foun
129.8	High	High	High	High	Peak Foun	Not Found	Peak Foun	Peak Foun
114.6	High	High	Peak Foun	Peak Foun	Peak Foun	High	Peak Foun	Peak Foun
157.9	High	Peak Foun	High	Peak Foun	Peak Foun	Peak Foun	Peak Foun	Peak Foun
96.7	Peak Foun	High	High	Peak Foun	Peak Foun	High	High	High
114.8	High	High	High	High	High	High	High	High
107.4	High	High	High	High	High	High	High	Peak Foun
118.2	High	High	Peak Foun	High	Peak Foun	Peak Foun	High	High
97.6	High	High	High	High	High	High	High	Peak Foun
122.2	High	High	High	High	High	High	High	High
84.9	High	High	High	High	High	High	High	High
92.8	High	High	High	Peak Foun	High	High	Peak Foun	Peak Foun
104.8	High	High	High	High	High	High	Peak Foun	Peak Foun
131.3	High	High	High	High	High	High	Peak Foun	High
116.8	High	High	High	High	High	High	High	High
95.8	High	High	High	High	High	High	High	High
99.5	High	High	High	High	High	High	Peak Foun	High
101.7	High	Peak Foun	Peak Foun	Peak Foun	Not Found	Peak Foun	High	High
109	Peak Foun	High	Not Found	Peak Foun	Not Found	Not Found	Not Found	Not Found
76.1	High	High	Peak Foun	Peak Foun	High	Peak Foun	Peak Foun	Peak Foun
75.9	Peak Foun	Peak Foun	High	Peak Foun	Peak Foun	Peak Foun	Peak Foun	Peak Foun
117.5	High	High	High	Peak Foun	Peak Foun	Peak Foun	High	High
74.8	High	High	High	High	High	High	High	High
76	High	High	High	Peak Foun	High	High	High	High
145.7	High	High	High	Peak Foun	Peak Foun	Not Found	Not Found	Not Found
89	High	High	High	Peak Foun	Peak Foun	High	Peak Foun	High
141.5	High	High	High	High	High	High	High	Peak Foun
99.5	High	High	High	High	High	High	High	High
153.5	High	High	Peak Foun	High	Peak Foun	Peak Foun	Not Found	Peak Foun
128.6	High	High	Peak Foun	Peak Foun	Peak Foun	Peak Foun	Peak Foun	Peak Foun
101.6	High	High	High	High	High	Peak Foun	Peak Foun	High
138.2	High	High	High	Peak Foun	Peak Foun	Peak Foun	Peak Foun	High
148.8	High	High	High	Peak Foun	Peak Foun	Peak Foun	Peak Foun	Peak Foun

114.7	High	High	Peak Foun	High	Peak Foun	High	Peak Foun	High
100.1	High	High	High	High	High	High	High	High
108.6	High	High	High	High	Peak Foun	Peak Foun	Peak Foun	Peak Foun
93.7	High	High	High	High	High	High	High	High
99.6	High	Peak Foun	High	Peak Foun	High	High	High	High
120.3	High	High	Peak Foun	Peak Foun	Peak Foun	Peak Foun	Peak Foun	Peak Foun
114.4	High	High	High	High	High	Peak Foun	Peak Foun	High
130.9	Peak Foun	High	Peak Foun	Peak Foun	Peak Foun	High	Peak Foun	Peak Foun
110.5	High	High	High	High	High	High	High	High
89.3	High	Peak Foun	High	Peak Foun	Peak Foun	Peak Foun	High	Peak Foun
123.5	High	High	High	Peak Foun	Peak Foun	Peak Foun	High	Peak Foun
155.9	High	High	High	Peak Foun	Peak Foun	Peak Foun	Peak Foun	Peak Foun
99.4	Peak Foun	High	High	High	High	High	High	High
98.5	High	High	High	Peak Foun	High	High	High	Peak Foun
93.6	High	High	High	High	High	High	High	High
108.9	Peak Foun	High	High	Peak Foun	Peak Foun	Peak Foun	High	Peak Foun
104.1	High	High	High	High	High	High	High	High
105.5	High	High	High	Peak Foun	Peak Foun	High	High	High
88.9	High	High	High	High	Peak Foun	Peak Foun	Peak Foun	High
145.9	High	High	High	High	Peak Foun	High	Peak Foun	Peak Foun
89.8	Peak Foun	Not Found	Peak Foun	Peak Foun	High	Peak Foun	Peak Foun	Peak Foun
102	High	High	High	High	Peak Foun	Peak Foun	High	Peak Foun
130.7	High	High	High	High	Peak Foun	High	High	High
107.5	High	High	High	High	High	High	High	High
107.5	Peak Foun	High	High	Peak Foun	Peak Foun	Peak Foun	High	High
101.2	High	High	High	Peak Foun	Peak Foun	High	Peak Foun	Peak Foun
101.6	High	High	High	High	High	High	High	High
133.6	High	High	High	High	High	High	High	High
129.3	High	High	High	Peak Foun	Peak Foun	Peak Foun	Peak Foun	Peak Foun
117	Peak Foun	High	High	Peak Foun	Peak Foun	High	High	Peak Foun
127.8	Peak Foun	Peak Foun	High	Not Found	Not Found	Peak Foun	Not Found	Peak Foun
76.9	Peak Foun	High	Peak Foun	Peak Foun	Peak Foun	High	High	Peak Foun
94.6	High	High	High	High	High	High	High	High
135.5	High	High	High	High	Peak Foun	Peak Foun	Peak Foun	High
118.7	High	High	High	High	High	High	High	High
138.3	High	High	High	High	High	High	High	High
135.6	High	High	High	High	Peak Foun	High	High	High
113.8	High	Peak Foun	High	Peak Foun	Peak Foun	Peak Foun	Peak Foun	Peak Foun
108.6	High	High	High	Peak Foun	High	Peak Foun	High	Peak Foun
115.4	High	High	High	Peak Foun	High	High	High	High
121.9	High	High	High	High	Peak Foun	High	Peak Foun	High
131.1	High	High	High	Peak Foun	Peak Foun	High	Peak Foun	High
139	High	High	High	Peak Foun	Peak Foun	Peak Foun	Peak Foun	Peak Foun
104.5	High	High	High	High	High	High	High	High
173.8	High	High	High	High	High	Peak Foun	Peak Foun	Peak Foun

90.3	High	Peak Foun	High	Peak Foun	High	High	High	High
100.9	High	High	High	Peak Foun	Peak Foun	Peak Foun	High	Peak Foun
106.9	High	Peak Foun	Peak Foun	Peak Foun	High	High	Peak Foun	Peak Foun
124.1	Peak Foun	High	High	Peak Foun	Peak Foun	Peak Foun	Peak Foun	Peak Foun
92.1	High	High	High	High	High	High	High	High
133.1	High	High	High	High	Peak Foun	High	High	Peak Foun
64.2	High	High	High	High	High	High	High	High
118.5	High	High	High	Peak Foun	Peak Foun	Peak Foun	Peak Foun	High
133.3	High	High	High	High	Peak Foun	Peak Foun	High	Peak Foun
137	High	High	High	Peak Foun	Peak Foun	Peak Foun	Peak Foun	Peak Foun
149.8	High	High	High	Peak Foun	Peak Foun	Peak Foun	Not Found	Peak Foun
85.3	High	High	High	High	Peak Foun	Peak Foun	Peak Foun	High
133.2	Peak Foun	High	High	Peak Foun	High	High	High	Peak Foun
110.8	Peak Foun	Peak Foun	Not Found	Peak Foun	Peak Foun	High	Peak Foun	Peak Foun
92.4	High	High	High	Peak Foun	Peak Foun	High	High	High
122.4	High	High	High	High	High	High	High	Peak Foun
107	High	High	Peak Foun	High	Peak Foun	High	Peak Foun	Peak Foun
130.9	High	High	High	Peak Foun	Peak Foun	Peak Foun	Peak Foun	Peak Foun
86.7	High	High	Peak Foun	Peak Foun	High	High	Peak Foun	Peak Foun
177.8	High	High	High	High	Not Found	Peak Foun	Peak Foun	Not Found
110.9	High	High	Peak Foun	High	High	High	High	Peak Foun
111.9	High	High	High	High	High	High	Peak Foun	Peak Foun
93.1	High	High	High	Peak Foun	High	High	Peak Foun	Peak Foun
113	High	High	High	Peak Foun	Peak Foun	Peak Foun	Peak Foun	Peak Foun
131.4	High	High	Peak Foun	Peak Foun	Peak Foun	Peak Foun	Peak Foun	Peak Foun
134	High	High	High	High	High	Peak Foun	Peak Foun	High
116.1	High	High	High	High	Not Found	Not Found	Peak Foun	Peak Foun
108.1	High	High	High	High	Peak Foun	High	High	High
119.3	High	High	High	High	High	High	High	High
118.5	High	High	High	Peak Foun	Peak Foun	Peak Foun	Peak Foun	High
116.6	High	High	High	High	High	High	High	Peak Foun
138.1	High	High	Peak Foun	Peak Foun	Peak Foun	Peak Foun	Peak Foun	Peak Foun
114.7	High	High	High	Peak Foun	Peak Foun	Peak Foun	Peak Foun	High
107.7	High	High	High	High	High	High	High	Peak Foun
94.3	High	High	High	Peak Foun	Peak Foun	Peak Foun	Peak Foun	Peak Foun
108.5	Peak Foun	High	Peak Foun	High	Peak Foun	Peak Foun	High	High
104	High	High	High	High	High	High	High	High
104.3	Peak Foun	High	High	Peak Foun	Peak Foun	High	High	Peak Foun
115.6	High	High	High	High	Peak Foun	High	High	High
114.1	Peak Foun	High	High	Peak Foun	High	High	High	High
99.7	High	High	High	High	High	High	High	Peak Foun
165.6	High	High	High	Peak Foun	Peak Foun	Peak Foun	Peak Foun	Peak Foun
122.1	High	High	High	Peak Foun	High	High	Peak Foun	Peak Foun
116.6	High	High	High	High	Peak Foun	Peak Foun	Peak Foun	Peak Foun
137.1	High	High	High	High	Not Found	Peak Foun	Peak Foun	Peak Foun

102.8	High	High	High	High	High	High	High	High
107.8	High	Peak Foun	Peak Foun	Peak Foun	High	High	High	Peak Foun
43.1	Peak Foun	Peak Foun	Not Found	Peak Foun	High	High	Peak Foun	Peak Foun
104.9	High	High	High	Peak Foun	High	High	High	Peak Foun
64.8	High	High	High	High	High	High	High	High
123.6	High	High	High	High	High	High	High	High
72.3	High	High	High	High	High	High	High	High
116.9	High	Peak Foun	Peak Foun	Peak Foun	Not Found	Peak Foun	Peak Foun	Peak Foun
85.4	High	Peak Foun	High	High	High	High	High	Peak Foun
36.7	Peak Foun	Peak Foun	Peak Foun	Peak Foun	High	High	Peak Foun	Peak Foun
116.7	High	High	High	Peak Foun	High	Peak Foun	High	Peak Foun
103.9	Peak Foun	High	High	Peak Foun	Peak Foun	Peak Foun	Peak Foun	Peak Foun
117.8	High	High	High	High	Peak Foun	High	High	Peak Foun
146.2	High	High	High	Peak Foun	Peak Foun	Peak Foun	Peak Foun	Peak Foun
144.2	High	High	High	Peak Foun	Peak Foun	Peak Foun	Peak Foun	Peak Foun
100.7	High	High	High	High	Peak Foun	High	High	High
129.1	High	High	High	Peak Foun	Peak Foun	High	Peak Foun	Peak Foun
93.6	High	High	High	Peak Foun	Peak Foun	Peak Foun	High	Peak Foun
142.4	High	High	High	Peak Foun	Peak Foun	High	Peak Foun	High
119.1	High	High	High	High	Peak Foun	Peak Foun	High	High
132.5	High	High	High	High	Peak Foun	Peak Foun	Peak Foun	Peak Foun
92.6	High	High	High	High	High	High	High	High
117.6	High	High	High	Peak Foun	Peak Foun	Peak Foun	Peak Foun	High
118.8	High	High	High	High	Not Found	Peak Foun	High	High
119.9	High	High	High	High	High	High	High	Peak Foun
89.8	High	High	High	High	High	High	High	Peak Foun
102.9	High	High	High	High	High	High	High	High
108.4	High	High	High	High	High	High	High	High
114.2	High	High	Peak Foun	High	High	High	High	Peak Foun
110.4	High	High	High	High	High	High	High	High
122.7	High	High	High	Peak Foun	Peak Foun	Peak Foun	Peak Foun	High
104.8	High	High	High	High	High	High	High	High
95.4	High	High	High	High	High	High	High	High
131	High	High	High	High	High	High	High	High
140.5	High	High	High	High	Peak Foun	Peak Foun	High	High
104.2	High	Peak Foun	High	High	High	High	Peak Foun	High
149.1	Peak Foun	High	Peak Foun	Peak Foun	Peak Foun	Peak Foun	Peak Foun	High
139.6	Peak Foun	High	High	High	Peak Foun	Peak Foun	Peak Foun	Peak Foun
55.8	Peak Foun	Not Found	Not Found	Not Found	Peak Foun	High	Peak Foun	Not Found
101.4	Peak Foun	High	High	Peak Foun	Peak Foun	Peak Foun	High	Peak Foun
109.3	Peak Foun	High	High	High	Not Found	Peak Foun	Peak Foun	High
138.4	Peak Foun	Peak Foun	Peak Foun	Peak Foun	High	High	High	High
156.4	Peak Foun	High	High	High	Peak Foun	High	Peak Foun	High
146.6	Peak Foun	High	High	Peak Foun	Peak Foun	Peak Foun	Peak Foun	Peak Foun
89.5	High	High	High	High	Peak Foun	High	High	High

104.1	High	Peak Foun	High	Peak Foun	High	High	High	High
101.6	High	High	High	High	High	High	High	High
82.6	High	Peak Foun	Peak Foun	High	High	High	Peak Foun	Peak Foun
107.9	High	High	High	Peak Foun	Peak Foun	High	High	High
111.9	High	High	High	Peak Foun	Peak Foun	Peak Foun	Peak Foun	Peak Foun
120.5	High	High	High	High	Peak Foun	Peak Foun	High	High
73.3	High	High	Peak Foun	Peak Foun	High	High	High	Peak Foun
88.3	High	High	High	Peak Foun	Peak Foun	Peak Foun	Peak Foun	Peak Foun
167.9	High	High	High	High	Peak Foun	Peak Foun	Peak Foun	Peak Foun
109	High	High	High	High	Peak Foun	Peak Foun	High	Peak Foun
163.8	Peak Foun	Peak Foun	Peak Foun	Not Found	Peak Foun	Not Found	Peak Foun	Peak Foun
141.4	High	High	High	High	Peak Foun	Peak Foun	Peak Foun	Peak Foun
105.2	High	High	High	High	High	High	High	High
98.2	High	High	High	High	Not Found	Peak Foun	Not Found	Not Found
116.7	Peak Foun	High	High	Peak Foun	Peak Foun	Peak Foun	High	High
88	High	High	High	High	High	Peak Foun	Peak Foun	High
144.3	High	High	High	High	High	High	Peak Foun	High
72.5	High	High	High	Peak Foun	Peak Foun	Peak Foun	High	Peak Foun
107.6	High	High	High	High	High	High	High	High
96	High	High	Peak Foun	Peak Foun	High	High	High	High
109.7	High	High	High	High	Peak Foun	Peak Foun	Peak Foun	Peak Foun
120.1	Peak Foun	High	Peak Foun	Peak Foun	Peak Foun	Peak Foun	Peak Foun	Peak Foun
116.6	High	High	High	Peak Foun	High	High	High	High
117.9	Peak Foun	High	High	Peak Foun	Peak Foun	High	High	High
93	High	High	High	Peak Foun	High	High	High	High
105.7	High	High	High	High	High	Peak Foun	Peak Foun	High
117.9	High	High	High	High	High	High	High	High
124.4	High	Peak Foun	High	Peak Foun	Peak Foun	Peak Foun	Peak Foun	Peak Foun
84.6	Peak Foun	Peak Foun	High	Peak Foun	Not Found	High	Peak Foun	Peak Foun
100	High	Peak Foun	High	Peak Foun	High	High	High	High
142.6	High	High	High	Peak Foun	High	Peak Foun	Peak Foun	High
117.6	High	Peak Foun	High	High	High	High	Peak Foun	Peak Foun
108.5	High	High	High	High	High	High	High	Peak Foun
156.9	High	High	High	High	Peak Foun	Peak Foun	Peak Foun	Peak Foun
79.3	High	High	High	High	High	High	High	High
136.7	High	High	High	High	Peak Foun	High	High	High
144	High	Peak Foun	Peak Foun	Peak Foun	Peak Foun	Peak Foun	Peak Foun	Peak Foun
80.9	High	High	High	Peak Foun	Not Found	Not Found	Not Found	Not Found
60.5	High	Peak Foun	Peak Foun	Peak Foun	High	Peak Foun	Peak Foun	Peak Foun
125.4	High	High	High	High	Peak Foun	Peak Foun	Peak Foun	Peak Foun
127.2	High	High	High	High	High	Peak Foun	High	High
94.1	High	High	High	High	High	High	Peak Foun	Peak Foun
138.5	High	High	High	High	High	Peak Foun	Peak Foun	Peak Foun
114.1	High	High	High	High	High	High	High	High
116	Peak Foun	High	High	High	Peak Foun	Peak Foun	Peak Foun	Peak Foun

134	High	High	High	High	High	Peak Foun	Peak Foun	Peak Foun
117.1	High	High	High	Peak Foun	Peak Foun	Peak Foun	Peak Foun	High
93	High	High	High	Peak Foun	High	High	High	Not Found
104.8	High	Peak Foun	High	Peak Foun	Peak Foun	Peak Foun	Peak Foun	Peak Foun
131.3	High	High	High	High	High	High	High	High
106	High	High	High	High	High	Peak Foun	Peak Foun	Peak Foun
108.7	High	Peak Foun	High	Peak Foun	High	High	High	High
94.4	High	High	Peak Foun	Peak Foun	Peak Foun	Peak Foun	Peak Foun	Peak Foun
120.4	High	High	High	High	Peak Foun	Peak Foun	High	High
121.3	High	High	High	High	Peak Foun	Peak Foun	High	High
92.1	High	High	High	High	High	High	High	High
102.9	High	High	High	Peak Foun	Peak Foun	High	Peak Foun	High
84.6	Peak Foun	High	High	Peak Foun	Peak Foun	Peak Foun	Peak Foun	Peak Foun
115.9	Peak Foun	Peak Foun	Peak Foun	Peak Foun	Peak Foun	Not Found	Not Found	Not Found
92.7	High	High	High	Peak Foun	Peak Foun	High	High	Peak Foun
105.2	High	High	High	High	High	High	High	High
111.7	High	High	High	High	High	High	Peak Foun	Peak Foun
100.6	High	High	High	Peak Foun	Peak Foun	High	High	Peak Foun
101.9	High	High	High	High	Peak Foun	High	High	High
111.7	High	High	High	High	Peak Foun	Peak Foun	Peak Foun	High
132.8	High	High	High	High	Peak Foun	Peak Foun	High	High
100.6	Peak Foun	High	Peak Foun	Peak Foun	High	High	Peak Foun	High
136.9	Peak Foun	High	High	High	Peak Foun	Peak Foun	Peak Foun	Peak Foun
95.5	High	Peak Foun	Peak Foun	Peak Foun	High	Peak Foun	High	Peak Foun
127.4	High	Peak Foun	High	Peak Foun	Peak Foun	Peak Foun	Peak Foun	Peak Foun
88.1	High	High	High	High	High	High	High	High
105.1	High	High	High	Peak Foun	Peak Foun	High	High	High
129.3	High	High	High	High	Peak Foun	High	High	High
88.7	High	High	High	High	High	High	High	High
112.7	High	High	High	High	Peak Foun	High	Peak Foun	Peak Foun
71.3	Peak Foun	Peak Foun	Peak Foun	Not Found	Peak Foun	Not Found	Not Found	Not Found
108.7	High	High	High	High	Peak Foun	High	Peak Foun	High
114	High	High	High	Peak Foun	Peak Foun	Peak Foun	High	High
124.4	High	High	High	High	High	High	High	High
68.3	High	Peak Foun	High	High	High	High	High	High
85.8	Peak Foun	High	Peak Foun	Peak Foun	Peak Foun	High	Peak Foun	Peak Foun
102.3	High	High	High	High	High	High	High	High
97.1	High	High	High	High	High	Peak Foun	Peak Foun	Peak Foun
94.7	High	Peak Foun	High	High	Peak Foun	Peak Foun	High	High
151.1	High	High	High	High	Peak Foun	Peak Foun	Peak Foun	High
123.9	High	High	High	High	Peak Foun	Peak Foun	Peak Foun	High
127.3	High	High	High	High	Peak Foun	Peak Foun	Peak Foun	High
89.1	High	High	High	High	High	High	High	High
118	High	High	Peak Foun	High	High	High	Peak Foun	High
155.7	High	Not Found	High	Not Found	Not Found	Not Found	Not Found	Not Found

91.7	High	Peak Foun	High	High	High	High	High	High
117	High	High	High	High	High	Peak Foun	Peak Foun	High
150.5	High	High	High	High	Peak Foun	Peak Foun	Peak Foun	High
131.7	High	Peak Foun	Peak Foun	Peak Foun	Not Found	Peak Foun	Peak Foun	High
99.2	High	Peak Foun	Peak Foun	Peak Foun	Peak Foun	Peak Foun	Peak Foun	Peak Foun
108.8	High	High	High	High	High	High	High	High
115.8	Peak Foun	Peak Foun	Peak Foun	Peak Foun	Peak Foun	Peak Foun	Peak Foun	Peak Foun
111.9	High	Not Found	High	High	Peak Foun	Peak Foun	Peak Foun	Peak Foun
111.9	High	High	High	High	Peak Foun	Peak Foun	Peak Foun	High
127.9	High	High	High	Peak Foun	Peak Foun	Peak Foun	High	High
74.8	High	High	High	High	High	High	High	High
124.6	High	High	High	High	Peak Foun	High	High	Peak Foun
115.4	High	High	High	High	High	High	High	High
87.8	High	Peak Foun	Peak Foun	Peak Foun	High	High	High	High
141.7	High	High	High	Peak Foun	Peak Foun	Peak Foun	Peak Foun	High
110.9	High	High	High	Peak Foun	Peak Foun	High	High	Peak Foun
123.8	High	Peak Foun	High	Peak Foun	Peak Foun	High	High	Peak Foun
142.4	High	High	High	High	High	High	High	High
113.8	High	High	High	High	Peak Foun	Peak Foun	High	High
129.3	High	High	High	High	High	High	High	High
140.6	Peak Foun	Peak Foun	Peak Foun	Peak Foun	Peak Foun	High	Peak Foun	Peak Foun
119	High	High	Peak Foun	High	Peak Foun	Peak Foun	High	High
98.2	High	High	High	High	High	High	High	High
96.8	High	Peak Foun	Peak Foun	Peak Foun	High	High	High	High
126.8	Peak Foun	High	High	High	High	High	High	High
108.1	High	High	High	Peak Foun	Peak Foun	Peak Foun	Peak Foun	Peak Foun
115	High	Peak Foun	High	Not Found	High	Peak Foun	Peak Foun	Peak Foun
61.9	High	High	High	High	Peak Foun	Peak Foun	Peak Foun	Peak Foun
147.6	High	High	High	High	Not Found	Not Found	Not Found	Peak Foun
80.6	High	High	High	Peak Foun	Peak Foun	Peak Foun	High	High
112.8	High	High	High	Peak Foun	High	High	High	Peak Foun
101.4	High	High	Peak Foun	High	High	High	High	High
121.4	High	Peak Foun	High	High	Not Found	High	High	Peak Foun
149	High	High	Peak Foun	High	Peak Foun	Peak Foun	Peak Foun	Peak Foun
129.5	High	High	High	High	High	High	Peak Foun	Peak Foun
94	High	High	High	Peak Foun	Peak Foun	High	Peak Foun	High
99.7	High	Peak Foun	High	High	High	High	High	High
107.6	High	High	High	High	High	Peak Foun	High	High
86.5	High	Peak Foun	Peak Foun	Peak Foun	Peak Foun	Peak Foun	High	High
144.8	High	High	High	High	Peak Foun	High	Peak Foun	Peak Foun
106.5	Peak Foun	Peak Foun	High	Peak Foun	Peak Foun	Peak Foun	High	Peak Foun
123.3	High	High	High	High	Peak Foun	High	High	Peak Foun
145.5	High	High	High	High	High	High	High	High
117.6	High	High	High	High	High	High	Peak Foun	High
87.3	Peak Foun	Peak Foun	High	Peak Foun	Not Found	Peak Foun	High	High

92.4	High	High	High	High	Peak Foun	High	Peak Foun	High
124.5	High	High	High	High	Peak Foun	High	Peak Foun	Peak Foun
166.7	High	High	Peak Foun	Peak Foun	Peak Foun	Peak Foun	Peak Foun	Peak Foun
107.6	High	High	High	Peak Foun	High	High	High	Peak Foun
105.1	High	High	High	Peak Foun	Not Found	Peak Foun	Peak Foun	High
94.6	High	High	High	Peak Foun	High	Peak Foun	High	Peak Foun
89.5	High	High	Peak Foun	High	Peak Foun	High	High	Peak Foun
103.9	Peak Foun	High	High	High	Peak Foun	Peak Foun	Peak Foun	Peak Foun
138.2	High	High	High	High	High	Peak Foun	High	High
116.6	High	High	High	High	High	High	High	High
80.2	Peak Foun	Peak Foun	Peak Foun	Peak Foun	Peak Foun	High	Peak Foun	Peak Foun
96.9	High	High	High	High	Peak Foun	Peak Foun	High	Peak Foun
129.5	High	High	High	Peak Foun	High	High	Peak Foun	Peak Foun
121.6	High	High	High	High	Peak Foun	Peak Foun	High	High
123.5	High	High	High	High	Peak Foun	High	High	High
113.3	High	High	Peak Foun	Not Found	Peak Foun	Peak Foun	High	High
100.1	High	High	High	High	High	High	High	High
111.4	High	Peak Foun	High	Peak Foun	Peak Foun	Peak Foun	High	Peak Foun
192	High	High	High	High	Peak Foun	Peak Foun	Peak Foun	Peak Foun
143.8	High	High	High	High	Peak Foun	Peak Foun	High	High
124	Peak Foun	Peak Foun	High	Peak Foun	Not Found	Peak Foun	High	Peak Foun
97.5	High	High	High	High	High	High	Peak Foun	High
124.6	High	High	High	Peak Foun	High	High	High	Peak Foun
108.1	High	High	High	High	Peak Foun	Peak Foun	Peak Foun	Peak Foun
97.4	High	High	Peak Foun	Peak Foun	High	High	High	High
137	Peak Foun	Peak Foun	Peak Foun	Peak Foun	Not Found	Peak Foun	Peak Foun	Peak Foun
125.5	Peak Foun	Peak Foun	Peak Foun	Peak Foun	High	High	High	Peak Foun
130.8	High	High	High	High	Not Found	Peak Foun	Peak Foun	High
95.7	Peak Foun	Not Found	Peak Foun	Peak Foun	Not Found	High	Not Found	High
116.7	High	High	Peak Foun	Peak Foun	High	Peak Foun	Peak Foun	Peak Foun
126.6	High	High	High	High	High	High	Peak Foun	Peak Foun
111.7	High	High	High	Peak Foun	Peak Foun	Peak Foun	High	Peak Foun
120.1	High	High	High	High	Peak Foun	High	High	High
53.7	High	High	Peak Foun	Peak Foun	Not Found	Not Found	Not Found	Not Found
104.6	Peak Foun	Peak Foun	Peak Foun	Peak Foun	Not Found	Peak Foun	Not Found	Not Found
153.8	High	High	Peak Foun	Peak Foun	Peak Foun	Peak Foun	Not Found	Peak Foun
111.7	High	Peak Foun	High	Peak Foun	Peak Foun	High	High	High
	Not Found	Not Found	Not Found	Not Found	Not Found	High	Not Found	Peak Foun
88.6	High	High	High	High	Peak Foun	High	Peak Foun	Peak Foun
117.1	High	High	High	High	High	High	Peak Foun	High
94.4	High	High	High	High	High	Peak Foun	High	High
98.8	High	High	High	High	High	High	High	High
115.6	Peak Foun	High	High	Peak Foun	Peak Foun	High	Peak Foun	High
122.1	High	High	Peak Foun	Peak Foun	Peak Foun	High	Peak Foun	Peak Foun
112.2	High	Peak Foun	High	High	High	High	High	High

137	High	High	High	Peak Foun	Not Found	Peak Foun	Not Found	Peak Foun
91.9	High	High	High	High	High	High	High	High
75	High	High	Peak Foun	Peak Foun	Peak Foun	Peak Foun	Peak Foun	Peak Foun
145.6	High	High	High	High	Peak Foun	High	Peak Foun	Peak Foun
159.3	Peak Foun	High	High	Peak Foun	Not Found	Peak Foun	Peak Foun	Peak Foun
102.1	High	High	High	High	High	Peak Foun	Peak Foun	Peak Foun
145.9	High	High	High	Peak Foun	Peak Foun	Peak Foun	High	High
108.3	High	High	High	High	High	High	High	High
128.8	Peak Foun	High	High	Not Found	Peak Foun	Peak Foun	High	Peak Foun
129.5	Peak Foun	High	High	Peak Foun	High	High	High	High
86.1	High	High	High	Peak Foun	High	Peak Foun	High	Peak Foun
88.7	High	High	Peak Foun	Peak Foun	Peak Foun	High	Peak Foun	High
95.8	High	High	High	Peak Foun	Not Found	Not Found	Not Found	Peak Foun
108.3	High	Peak Foun	High	High	Peak Foun	Peak Foun	High	Peak Foun
152.2	High	Peak Foun	High	Peak Foun	Not Found	Not Found	Peak Foun	Not Found
101	High	High	High	High	High	High	High	High
119.6	High	Peak Foun	Peak Foun	Peak Foun	Peak Foun	High	Peak Foun	Peak Foun
135.4	High	High	High	Peak Foun	Peak Foun	High	High	Peak Foun
99.7	High	High	Peak Foun	Peak Foun	Peak Foun	High	Peak Foun	Peak Foun
95.5	High	High	High	High	High	High	High	High
113.5	High	High	High	High	High	High	High	High
79.3	High	High	High	Peak Foun	High	High	High	Peak Foun
112.2	Peak Foun	Peak Foun	High	Peak Foun	Peak Foun	High	High	Peak Foun
110.6	High	High	High	High	High	Peak Foun	High	High
105.8	High	High	High	High	Not Found	High	High	High
107.9	High	High	High	High	High	Peak Foun	Peak Foun	Peak Foun
113.6	High	High	High	High	Peak Foun	High	Peak Foun	High
107.9	High	High	High	Peak Foun	Peak Foun	Peak Foun	Peak Foun	Peak Foun
101.5	Peak Foun	High	High	Peak Foun	Not Found	High	High	High
90.8	High	High	Peak Foun	Peak Foun	High	High	High	Peak Foun
102.4	High	Peak Foun	High	Peak Foun	High	High	High	Peak Foun
122.9	High	High	Peak Foun	Not Found	Not Found	Not Found	Not Found	Peak Foun
77.3	High	Peak Foun	Peak Foun	Peak Foun	Peak Foun	High	Peak Foun	High
90.2	High	Peak Foun	Peak Foun	Peak Foun	Peak Foun	High	Peak Foun	High
113.7	High	High	High	High	High	High	High	High
96.4	High	High	High	High	High	High	High	High
138.9	High	High	High	Peak Foun	Peak Foun	High	Peak Foun	Peak Foun
115.6	High	High	High	High	Peak Foun	Peak Foun	High	High
116	High	High	High	High	High	High	Peak Foun	Peak Foun
102.5	High	High	High	Peak Foun	High	Peak Foun	High	Peak Foun
96.7	High	High	High	Peak Foun	High	High	High	Peak Foun
98.1	High	High	High	Peak Foun	Peak Foun	High	Peak Foun	Peak Foun
99.5	High	Peak Foun	High	Peak Foun	High	High	High	High
99.3	High	High	High	Peak Foun	Peak Foun	High	Peak Foun	Peak Foun
109.9	High	High	High	High	High	High	High	High

122.6	High	High	High	High	Peak Foun	High	Peak Foun	High
93.3	High	High	High	High	Not Found	High	Peak Foun	Peak Foun
96.4	High	Peak Foun	Peak Foun	Peak Foun	High	High	Peak Foun	High
111.6	High	Peak Foun	High	Not Found	High	Peak Foun	High	High
82.4	High	High	Peak Foun	High	Peak Foun	Peak Foun	Peak Foun	High
108.5	Peak Foun	Peak Foun	High	Peak Foun	Not Found	High	High	High
79.7	High	High	Peak Foun	High	Peak Foun	High	Peak Foun	Peak Foun
107	High	High	High	High	High	High	High	High
112.9	High	High	High	Peak Foun	High	High	Peak Foun	Peak Foun
85.8	Peak Foun	High	Not Found	Peak Foun	Not Found	Not Found	Not Found	Peak Foun
88.2	High	High	High	High	Peak Foun	Peak Foun	High	High
110.8	High	High	High	High	High	High	High	High
94.6	High	Peak Foun	High	Peak Foun	High	High	Peak Foun	Peak Foun
109.1	High	High	High	High	High	High	High	High
110.6	High	High	High	Peak Foun	High	High	High	High
95.8	High	High	High	High	High	Peak Foun	High	High
120	High	High	Peak Foun	Peak Foun	Peak Foun	High	Peak Foun	Peak Foun
115.1	Peak Foun	High	Peak Foun	Peak Foun	Peak Foun	Not Found	Not Found	Not Found
94.1	High	High	High	High	Peak Foun	High	High	High
83.9	High	High	High	Peak Foun	Peak Foun	Peak Foun	Peak Foun	Not Found
81.1	Peak Foun	Peak Foun	Peak Foun	Peak Foun	Peak Foun	Peak Foun	Peak Foun	Peak Foun
99.9	High	High	High	High	Peak Foun	High	High	Peak Foun
94.7	Peak Foun	Peak Foun	Peak Foun	Peak Foun	Peak Foun	High	High	Peak Foun
136.9	High	High	High	Peak Foun	Peak Foun	High	Peak Foun	High
104.3	High	High	High	High	Peak Foun	Peak Foun	Peak Foun	High
111.1	High	High	High	High	High	High	High	Peak Foun
116.9	High	High	High	Peak Foun	Peak Foun	High	High	Peak Foun
102	High	High	Peak Foun	Peak Foun	High	High	High	Peak Foun
127.7	Peak Foun	High	High	Peak Foun	Peak Foun	Peak Foun	High	Peak Foun
108.1	High	High	High	High	Not Found	Peak Foun	Not Found	High
117	High	High	Peak Foun	Peak Foun	Peak Foun	Not Found	Peak Foun	High
84.3	High	High	High	High	High	High	High	Peak Foun
34.4	High	High	High	Peak Foun	Peak Foun	Peak Foun	Peak Foun	Peak Foun
121.5	High	High	Peak Foun	Peak Foun	Peak Foun	Peak Foun	Peak Foun	Peak Foun
103.2	High	High	High	High	High	High	High	High
98.7	Peak Foun	Peak Foun	Peak Foun	Peak Foun	Peak Foun	High	High	High
122.7	High	High	High	Peak Foun	Peak Foun	High	Peak Foun	Peak Foun
121.6	High	Peak Foun	High	High	Peak Foun	Peak Foun	Peak Foun	Peak Foun
100.7	Peak Foun	High	Peak Foun	High	Peak Foun	Peak Foun	High	High
115.9	Peak Foun	Peak Foun	High	High	High	Peak Foun	Peak Foun	Peak Foun
123.9	High	High	High	Peak Foun	Not Found	Peak Foun	Peak Foun	Peak Foun
111.4	High	High	High	High	Peak Foun	High	High	High
76.6	High	High	High	High	High	High	High	High
116.8	High	High	High	Peak Foun	Not Found	Peak Foun	Peak Foun	Peak Foun
108.4	High	High	High	High	High	High	High	High

102.3	High	High	High	Peak Foun	High	High	High	High
133	Peak Foun	Peak Foun	Peak Foun	Not Found	Not Found	Not Found	Not Found	Peak Foun
80.1	High	High	High	Peak Foun	Peak Foun	High	High	Peak Foun
115.5	High	High	High	Not Found	Not Found	Peak Foun	Peak Foun	Peak Foun
40.3	High	High	High	High	Not Found	Not Found	Not Found	Not Found
120.8	Peak Foun	High	High	Peak Foun	Peak Foun	High	Peak Foun	Peak Foun
132.8	High	High	High	High	High	High	High	High
115.2	High	High	High	Peak Foun	Not Found	Peak Foun	Peak Foun	Peak Foun
104.8	Peak Foun	Peak Foun	High	Peak Foun	Peak Foun	Peak Foun	Peak Foun	High
71	High	Peak Foun	High	Peak Foun	High	High	Peak Foun	Peak Foun
114.4	Peak Foun	High	High	Not Found	Peak Foun	Peak Foun	Peak Foun	Peak Foun
120.7	High	Peak Foun	Peak Foun	Peak Foun	Peak Foun	High	Peak Foun	Peak Foun
101	High	Peak Foun	High	High	High	Peak Foun	Peak Foun	Peak Foun
110.8	High	High	Not Found	Not Found	Not Found	Not Found	Not Found	Not Found
104.4	High	High	High	High	Peak Foun	High	Peak Foun	Peak Foun
81.7	High	Peak Foun	High	Peak Foun	Peak Foun	High	Peak Foun	Peak Foun
86.5	Peak Foun	Peak Foun	Peak Foun	Peak Foun	High	High	High	High
94.4	High	High	High	Peak Foun	Peak Foun	High	High	Peak Foun
112.7	High	Peak Foun	High	High	Peak Foun	Peak Foun	Peak Foun	Peak Foun
118.7	High	High	High	High	High	High	High	High
98.5	High	Peak Foun	High	High	Peak Foun	Peak Foun	High	High
134.6	High	Peak Foun	High	Peak Foun	Not Found	Not Found	High	Not Found
158.4	Peak Foun	Peak Foun	Peak Foun	Peak Foun	Peak Foun	Peak Foun	Peak Foun	Peak Foun
114.5	High	High	High	Peak Foun	Peak Foun	High	Peak Foun	Peak Foun
98.2	Peak Foun	High	Peak Foun	Peak Foun	Peak Foun	High	Peak Foun	Peak Foun
108	High	High	High	High	High	High	High	High
35.5	High	High	Peak Foun	Peak Foun	High	High	High	Peak Foun
108.4	Peak Foun	High	High	Peak Foun	Peak Foun	Peak Foun	Peak Foun	Peak Foun
121.3	High	High	High	Peak Foun	Peak Foun	Peak Foun	Peak Foun	Peak Foun
99.2	High	High	High	High	Peak Foun	Peak Foun	Peak Foun	Peak Foun
121.6	High	High	High	High	High	High	High	High
160.4	High	High	High	High	Peak Foun	Peak Foun	Peak Foun	High
143	Peak Foun	High	High	Peak Foun	Peak Foun	High	Peak Foun	Peak Foun
92.2	High	High	High	Peak Foun	Peak Foun	Peak Foun	Not Found	Peak Foun
100.5	High	Peak Foun	High	Peak Foun	Peak Foun	Peak Foun	Peak Foun	Peak Foun
104.9	High	Peak Foun	High	High	Peak Foun	Peak Foun	High	High
113.8	High	High	High	High	Peak Foun	Peak Foun	Peak Foun	High
123.8	High	High	High	Peak Foun	Peak Foun	Peak Foun	Peak Foun	Peak Foun
144.1	High	High	High	Peak Foun	Peak Foun	Peak Foun	Peak Foun	High
142.7	Peak Foun	Peak Foun	Peak Foun	Peak Foun	Not Found	Not Found	Peak Foun	High
93.5	High	Peak Foun	High	High	High	High	High	High
115.7	High	Peak Foun	High	High	High	Peak Foun	Peak Foun	Peak Foun
158.7	High	High	High	High	Peak Foun	High	High	High
94.5	High	High	High	Peak Foun	Peak Foun	Peak Foun	Peak Foun	Not Found
103.7	High	High	High	Peak Foun	Peak Foun	Peak Foun	High	Peak Foun

82.7	Peak Foun	Peak Foun	High	Peak Foun	High	High	High	Peak Foun
114.7	High	High	High	Peak Foun	Peak Foun	High	High	Peak Foun
114.1	High	High	High	Peak Foun	Peak Foun	High	Peak Foun	High
90.4	High	High	High	Peak Foun	Peak Foun	High	Peak Foun	Peak Foun
179.7	High	High	High	Peak Foun	Peak Foun	Peak Foun	Peak Foun	High
102.1	Peak Foun	High	High	Peak Foun	High	Peak Foun	High	Peak Foun
127.1	High	High	High	Peak Foun	Peak Foun	Peak Foun	Peak Foun	High
105.2	High	High	High	High	Not Found	High	High	High
101.6	High	High	Peak Foun	Peak Foun	Peak Foun	Peak Foun	Peak Foun	High
110.7	High	High	High	High	Peak Foun	High	High	Peak Foun
99.9	High	High	High	Peak Foun	Peak Foun	Peak Foun	Peak Foun	Peak Foun
110.2	Peak Foun	Peak Foun	Peak Foun	Peak Foun	High	Peak Foun	High	High
96.7	Peak Foun	Peak Foun	Peak Foun	Peak Foun	Peak Foun	Peak Foun	Peak Foun	Peak Foun
101.8	High	High	High	Peak Foun	Peak Foun	Peak Foun	Peak Foun	High
72.2	Peak Foun	Peak Foun	High	Peak Foun	Peak Foun	High	Peak Foun	High
114.8	High	High	High	High	Peak Foun	Peak Foun	Peak Foun	Peak Foun
124.5	High	High	High	Peak Foun	Peak Foun	High	Peak Foun	High
76.7	High	High	High	Peak Foun	High	High	High	Peak Foun
130.9	High	High	High	High	High	High	High	High
102.9	High	High	High	High	High	High	High	High
121.2	High	High	High	Not Found	Peak Foun	High	High	High
104.1	High	High	High	High	High	High	High	High
97.1	High	High	High	Peak Foun	Peak Foun	High	High	High
95.6	High	High	High	High	High	High	High	High
132	High	Peak Foun	Peak Foun	Peak Foun	Not Found	Peak Foun	Peak Foun	Peak Foun
110	Peak Foun	High	High	Peak Foun	Not Found	Not Found	Not Found	Not Found
98.9	High	High	High	High	High	High	High	High
117.8	High	High	High	Peak Foun	Peak Foun	Peak Foun	Peak Foun	High
92.5	Peak Foun	Peak Foun	Peak Foun	Peak Foun	Not Found	Peak Foun	Peak Foun	Peak Foun
105	High	High	High	High	High	Peak Foun	High	High
90.3	High	High	High	Peak Foun	Peak Foun	Peak Foun	High	Peak Foun
150.9	High	Peak Foun	Peak Foun	Peak Foun	Not Found	Peak Foun	Not Found	Peak Foun
123.5	High	High	High	Peak Foun	Peak Foun	Peak Foun	Peak Foun	Peak Foun
166.9	Not Found	Not Found	High	Not Found	Not Found	Not Found	Not Found	Not Found
122.9	High	High	Peak Foun	High	Peak Foun	High	Peak Foun	Peak Foun
138.1	Peak Foun	High	High	High	Peak Foun	Peak Foun	Peak Foun	Peak Foun
114.7	High	High	High	Peak Foun	Peak Foun	Peak Foun	Peak Foun	High
132.9	High	High	High	High	Peak Foun	Peak Foun	Peak Foun	Peak Foun
96.4	High	High	High	High	High	High	High	High
115.9	High	Peak Foun	High	Peak Foun	Peak Foun	Peak Foun	Peak Foun	Peak Foun
152.5	High	Peak Foun	High	Peak Foun	High	Peak Foun	High	Peak Foun
111.3	High	High	Peak Foun	High	Peak Foun	Peak Foun	Peak Foun	Peak Foun
90.7	Not Found	Not Found	Not Found	Peak Foun	High	High	High	Peak Foun
134.1	High	High	High	High	Peak Foun	Peak Foun	High	Peak Foun
141.6	High	High	High	High	Peak Foun	Peak Foun	Peak Foun	High

122.6	High	High	High	Peak Foun	Peak Foun	Not Found	Not Found	Not Found
122	Peak Foun	High	Peak Foun	Peak Foun	Peak Foun	Not Found	Peak Foun	High
114.4	High	High	High	High	High	Peak Foun	High	Peak Foun
157.9	High	High	High	High	Peak Foun	Peak Foun	Peak Foun	Peak Foun
81.8	High	High	High	Peak Foun	High	Peak Foun	Peak Foun	Peak Foun
121.2	High	High	High	High	Peak Foun	Peak Foun	Peak Foun	Peak Foun
101.6	Peak Foun	Peak Foun	Peak Foun	High	Peak Foun	Peak Foun	Peak Foun	Peak Foun
114.7	High	High	High	High	High	High	High	Peak Foun
108.5	High	High	High	High	High	High	High	High
100.4	Peak Foun	High	Peak Foun	Peak Foun	Peak Foun	Peak Foun	Peak Foun	Peak Foun
139.4	High	High	Peak Foun	High	Peak Foun	Peak Foun	Peak Foun	Peak Foun
111	High	High	High	High	High	High	High	High
92.7	Peak Foun	High	High	Peak Foun	Not Found	Peak Foun	High	High
108.1	High	High	High	Peak Foun	Peak Foun	Peak Foun	Peak Foun	Peak Foun
117.8	Peak Foun	High	High	Peak Foun	Not Found	Peak Foun	Peak Foun	Peak Foun
112.9	Peak Foun	High	High	Not Found	Peak Foun	Peak Foun	Peak Foun	High
105.3	High	High	High	High	Peak Foun	High	High	High
126.8	Peak Foun	Peak Foun	Peak Foun	Peak Foun	Peak Foun	High	Peak Foun	Peak Foun
120	High	High	Peak Foun	Peak Foun	Peak Foun	Peak Foun	Peak Foun	High
128.9	High	High	High	High	Not Found	Peak Foun	Peak Foun	Peak Foun
97.2	High	High	High	Peak Foun	High	High	High	Peak Foun
118.2	High	High	High	High	High	High	High	High
86.5	High	High	Peak Foun	Peak Foun	Peak Foun	Peak Foun	Peak Foun	Peak Foun
98.4	High	High	Peak Foun	Peak Foun	Peak Foun	High	Peak Foun	Peak Foun
86.1	Not Found	High	High	High	Peak Foun	High	Peak Foun	High
92	Peak Foun	Peak Foun	High	Peak Foun	Peak Foun	Peak Foun	High	High
135.4	Peak Foun	High	High	Peak Foun	Peak Foun	Peak Foun	Peak Foun	Peak Foun
65.2	Peak Foun	Peak Foun	Peak Foun	Not Found	High	High	High	Peak Foun
118.3	High	High	High	High	Peak Foun	High	High	Peak Foun
147.6	High	High	Peak Foun	Peak Foun	Peak Foun	Peak Foun	Not Found	Peak Foun
112.7	High	High	High	Peak Foun	High	High	Peak Foun	High
112.8	High	High	High	High	Peak Foun	High	High	Peak Foun
95.7	Peak Foun	Peak Foun	Peak Foun	Peak Foun	Not Found	Peak Foun	High	Peak Foun
133.6	High	Peak Foun	Peak Foun	High	Peak Foun	High	Peak Foun	Peak Foun
100.9	High	Peak Foun	Peak Foun	Peak Foun	High	Peak Foun	High	High
106.7	Peak Foun	Peak Foun	Peak Foun	Peak Foun	High	High	Peak Foun	High
108	Peak Foun	High	High	Peak Foun	High	High	Peak Foun	Peak Foun
96.8	High	Peak Foun	Peak Foun	Peak Foun	Peak Foun	Not Found	Not Found	Peak Foun
88.2	High	High	High	High	High	High	High	High
112.6	High	High	High	High	High	Peak Foun	Peak Foun	High
128.3	High	High	High	Peak Foun	High	High	High	Peak Foun
130.8	High	Peak Foun	Peak Foun	High	Peak Foun	Peak Foun	Peak Foun	Peak Foun
115	High	High	High	Peak Foun	Not Found	High	Peak Foun	High
101.3	High	High	High	High	High	High	High	High
115.2	Peak Foun	High	High	Peak Foun	Peak Foun	High	High	High

72.5	High	High	High	Peak Foun	High	High	High	High
101.6	High	Peak Foun	Peak Foun	Peak Foun	High	High	High	Peak Foun
150.5	High	High	High	Peak Foun	Peak Foun	Peak Foun	Peak Foun	Peak Foun
133.5	High	High	High	High	High	High	High	High
138.9	High	High	High	Peak Foun	Peak Foun	Peak Foun	Peak Foun	Peak Foun
112.8	Peak Foun	Peak Foun	High	Peak Foun	Peak Foun	Peak Foun	High	High
104.7	High	High	Peak Foun	Peak Foun	Peak Foun	Peak Foun	Peak Foun	High
85.7	High	High	Peak Foun	Peak Foun	High	High	Peak Foun	Peak Foun
108.6	High	High	High	Peak Foun	Peak Foun	High	High	Peak Foun
140.3	High	High	High	High	Peak Foun	Peak Foun	Peak Foun	High
117	High	High	High	High	Peak Foun	Peak Foun	Peak Foun	Peak Foun
71.3	High	High	High	Not Found	Not Found	Not Found	Not Found	Not Found
109.9	High	High	High	High	High	High	High	High
140.2	Not Found	Not Found	Not Found	Not Found	Not Found	Not Found	Not Found	Not Found
94.6	High	High	High	Peak Foun	High	High	High	High
55.4	High	High	Peak Foun	High	Not Found	High	High	High
108.2	High	High	High	High	High	High	High	High
106.7	Peak Foun	Peak Foun	Peak Foun	Not Found	Peak Foun	Peak Foun	Peak Foun	Peak Foun
124.2	Peak Foun	High	Peak Foun	Peak Foun	High	High	Peak Foun	Peak Foun
127.5	High	High	High	Peak Foun	Peak Foun	Peak Foun	Peak Foun	High
66.3	Not Found	High	Peak Foun	Not Found	Not Found	Peak Foun	Peak Foun	Peak Foun
97.5	High	High	High	High	Peak Foun	Not Found	High	Peak Foun
108	High	High	Peak Foun	Peak Foun	Peak Foun	High	Peak Foun	Peak Foun
92.5	Peak Foun	High	High	Peak Foun	Peak Foun	High	Peak Foun	Peak Foun
85.1	Not Found	Peak Foun	Peak Foun	Peak Foun	High	High	Peak Foun	Peak Foun
103.2	High	High	High	High	Peak Foun	High	High	High
122.6	Peak Foun	Peak Foun	Peak Foun	Not Found	Not Found	Peak Foun	Not Found	Not Found
110.3	High	High	High	Peak Foun	Not Found	High	Peak Foun	High
119.2	High	High	Peak Foun	Peak Foun	Peak Foun	Not Found	Not Found	Not Found
119.5	High	High	High	High	Peak Foun	High	High	High
185.6	High	High	High	High	Peak Foun	Peak Foun	Peak Foun	Peak Foun
100.9	High	High	Peak Foun	High	Peak Foun	Peak Foun	Peak Foun	High
120.7	Peak Foun	Peak Foun	Peak Foun	Peak Foun	Peak Foun	Peak Foun	High	Peak Foun
96.7	High	Peak Foun	High	High	Peak Foun	Peak Foun	Peak Foun	High
162.9	High	Not Found	Not Found	Not Found	Not Found	Not Found	Peak Foun	Peak Foun
126.7	High	High	High	Not Found	Not Found	Not Found	Not Found	Not Found
86.9	High	High	High	High	High	High	High	High
122.1	High	Not Found	High	High	Peak Foun	Not Found	Not Found	Not Found
110.9	High	High	High	Peak Foun	Peak Foun	Peak Foun	Peak Foun	Peak Foun
107.1	Peak Foun	Peak Foun	Peak Foun	Peak Foun	Peak Foun	High	Peak Foun	High
100.1	High	High	Peak Foun	Peak Foun	Peak Foun	Peak Foun	Peak Foun	Peak Foun
95.7	Peak Foun	High	High	Peak Foun	High	High	High	Peak Foun
103.6	High	High	High	High	High	High	High	High
106.7	High	High	High	Peak Foun	Peak Foun	Peak Foun	High	Peak Foun
103.2	High	High	High	High	High	High	High	High

95.9	Peak Foun	Not Found	Not Found	Not Found	Not Found	Peak Foun	Not Found	Not Found
126.2	High	High	High	High	Not Found	Peak Foun	High	High
68.8	High	High	High	Peak Foun	Peak Foun	Peak Foun	Peak Foun	High
105.5	High	Peak Foun	High	Peak Foun	Peak Foun	Peak Foun	Peak Foun	Peak Foun
84.1	High	Not Found	Not Found	Peak Foun	Peak Foun	Not Found	Not Found	Not Found
115.2	Peak Foun	Peak Foun	Peak Foun	High	High	Peak Foun	Peak Foun	High
121.1	High	High	High	High	High	High	High	High
146.2	High	High	Peak Foun	Peak Foun	Peak Foun	Peak Foun	Peak Foun	Peak Foun
95.9	Peak Foun	Peak Foun	High	Peak Foun	Peak Foun	Peak Foun	Peak Foun	Peak Foun
121.8	Peak Foun	High	Peak Foun	Peak Foun	Peak Foun	Peak Foun	Peak Foun	Peak Foun
142.3	High	High	High	Not Found	Peak Foun	Not Found	Not Found	Peak Foun
116.7	Peak Foun	Peak Foun	Peak Foun	Peak Foun	Peak Foun	Peak Foun	Peak Foun	Peak Foun
110.9	High	High	Peak Foun	Peak Foun	High	High	High	High
97.6	Peak Foun	Peak Foun	High	Not Found	Not Found	Not Found	Not Found	Not Found
135.5	High	High	High	Peak Foun	Peak Foun	High	High	Peak Foun
82.3	High	Peak Foun	High	High	Peak Foun	Peak Foun	High	Peak Foun
104.7	Peak Foun	Peak Foun	Peak Foun	Peak Foun	High	Peak Foun	Peak Foun	Peak Foun
104.6	High	High	High	High	High	High	High	Peak Foun
105.3	High	Peak Foun	Peak Foun	Peak Foun	High	Not Found	Not Found	Peak Foun
99.3	High	High	Peak Foun	High	Peak Foun	Peak Foun	Peak Foun	Peak Foun
124.4	High	High	High	Peak Foun	Peak Foun	Peak Foun	Peak Foun	Peak Foun
119.5	Peak Foun	Peak Foun	High	Peak Foun	High	High	High	Peak Foun
113.3	High	Peak Foun	High	High	High	High	High	Peak Foun
115	Peak Foun	Peak Foun	Peak Foun	Peak Foun	Not Found	Not Found	Not Found	Peak Foun
134.3	Peak Foun	High	Peak Foun	Peak Foun	Peak Foun	Peak Foun	High	High
123.7	Peak Foun	Not Found	Peak Foun	Not Found	Not Found	Peak Foun	Not Found	Not Found
117.1	High	High	High	High	High	High	High	Peak Foun
112.6	Peak Foun	High	Peak Foun	Peak Foun	Peak Foun	Peak Foun	Peak Foun	High
117	High	High	High	High	High	High	High	High
123.7	Peak Foun	High	High	Peak Foun	Not Found	Peak Foun	Peak Foun	Peak Foun
109.3	High	High	High	Peak Foun	Peak Foun	Peak Foun	High	Peak Foun
123.9	High	High	High	High	Not Found	Peak Foun	Not Found	High
85.1	High	High	High	Peak Foun	High	Peak Foun	Peak Foun	Peak Foun
133.2	Peak Foun	High	Peak Foun	Peak Foun	Peak Foun	High	High	Peak Foun
114.9	High	High	High	Peak Foun	Not Found	High	Peak Foun	High
66.6	High	Peak Foun	Peak Foun	Peak Foun	High	High	High	Peak Foun
123.8	High	High	High	High	Peak Foun	High	High	Peak Foun
132.6	Peak Foun	Peak Foun	Peak Foun	Peak Foun	High	High	Peak Foun	Peak Foun
115.4	Not Found	Not Found	High	Not Found	Not Found	Not Found	Peak Foun	Not Found
102.1	Peak Foun	High	High	High	High	High	High	High
142.7	High	Peak Foun	High	Peak Foun	Not Found	Not Found	Not Found	Not Found
89.3	High	Peak Foun	High	High	High	High	High	High
94.5	High	High	High	Not Found	Not Found	Peak Foun	Not Found	High
100	Peak Foun	High	Peak Foun	High	Peak Foun	Peak Foun	Peak Foun	Peak Foun
98.7	High	High	Peak Foun	Peak Foun	High	High	High	Peak Foun

80.4	Peak Foun	Peak Foun	Peak Foun	Peak Foun	Peak Foun	Peak Foun	Peak Foun	Peak Foun
116.3	High	High	High	High	Peak Foun	Not Found	Peak Foun	Peak Foun
124.3	High	High	High	High	High	High	Peak Foun	High
106	High	High	High	High	Peak Foun	High	High	Peak Foun
106.3	Peak Foun	Peak Foun	Peak Foun	Peak Foun	Peak Foun	Peak Foun	Peak Foun	High
121.9	High	High	Peak Foun	Peak Foun	Peak Foun	High	Not Found	Peak Foun
124.8	High	Not Found	High	Not Found	Not Found	Not Found	Not Found	Not Found
136.4	Peak Foun	High	High	Peak Foun	High	Peak Foun	High	High
120.3	High	High	High	Peak Foun	Not Found	High	Not Found	Peak Foun
95.8	High	High	Peak Foun	Peak Foun	High	High	Peak Foun	High
114.4	High	High	High	High	Peak Foun	High	Peak Foun	High
93.3	Peak Foun	Peak Foun	High	Peak Foun	High	High	High	High
115.9	High	Not Found	Peak Foun	Not Found	High	High	Peak Foun	Peak Foun
94.2	High	High	High	High	Peak Foun	Peak Foun	High	High
126.7	High	High	High	High	Peak Foun	Peak Foun	High	High
132	High	High	Peak Foun	Peak Foun	Peak Foun	Peak Foun	Peak Foun	Peak Foun
136.8	Not Found	Peak Foun	Peak Foun	Peak Foun	Not Found	Not Found	Peak Foun	Peak Foun
130.6	High	High	Peak Foun	Peak Foun	Peak Foun	High	Peak Foun	Peak Foun
126.1	Peak Foun	High	High	Peak Foun	Peak Foun	Peak Foun	High	Peak Foun
71.3	High	High	High	High	High	High	High	High
79	High	High	High	Peak Foun	High	High	High	Peak Foun
92.1	High	High	High	Peak Foun	Peak Foun	High	Peak Foun	Peak Foun
81.6	High	High	High	High	High	High	Peak Foun	High
121.2	High	Peak Foun	High	High	Peak Foun	Peak Foun	Peak Foun	Peak Foun
156.1	High	High	High	High	Peak Foun	Peak Foun	Not Found	Peak Foun
111.5	High	Peak Foun	High	Peak Foun	Peak Foun	Peak Foun	Peak Foun	Peak Foun
105	High	High	High	High	High	High	High	High
66.5	Peak Foun	Peak Foun	Not Found	Not Found	Peak Foun	High	Not Found	Peak Foun
153.4	High	High	High	High	Not Found	Not Found	Peak Foun	High
104.8	High	High	Peak Foun	Peak Foun	Peak Foun	High	Peak Foun	Peak Foun
133.3	Peak Foun	Peak Foun	Peak Foun	Peak Foun	Peak Foun	High	High	Peak Foun
127.1	High	Peak Foun	High	Peak Foun	High	High	High	Peak Foun
131.4	High	High	High	Peak Foun	Peak Foun	Peak Foun	Peak Foun	Peak Foun
93.4	Peak Foun	Peak Foun	Peak Foun	Peak Foun	Peak Foun	High	Peak Foun	Peak Foun
113.4	Peak Foun	High	High	High	High	High	Peak Foun	High
110.9	High	High	High	High	High	High	High	Peak Foun
120.5	Peak Foun	Peak Foun	High	High	Peak Foun	Peak Foun	Peak Foun	Peak Foun
120.9	Peak Foun	Not Found	High	Peak Foun	Peak Foun	High	Peak Foun	Peak Foun
104.1	High	High	High	High	High	High	High	High
98.8	Peak Foun	High	Peak Foun	Peak Foun	Not Found	Not Found	Not Found	High
111.3	High	High	High	High	Peak Foun	Peak Foun	High	Peak Foun
96.9	Peak Foun	High	Peak Foun	Peak Foun	Not Found	Not Found	Not Found	Peak Foun
127.5	High	High	High	Peak Foun	Peak Foun	Not Found	Peak Foun	Peak Foun
109.2	High	High	High	Peak Foun	Peak Foun	Peak Foun	High	Peak Foun
112.4	Peak Foun	High	Peak Foun	Peak Foun	Peak Foun	High	High	Peak Foun

101	Peak Foun	Peak Foun	Not Found	Not Found	Peak Foun	High	Peak Foun	Peak Foun
101.5	High	Peak Foun	High	Peak Foun	Peak Foun	Peak Foun	Peak Foun	Peak Foun
103.8	Peak Foun	High	Peak Foun	Peak Foun	Peak Foun	High	Peak Foun	Peak Foun
108.4	High	High	High	High	Peak Foun	High	Peak Foun	High
111.8	Peak Foun	Peak Foun	High	Peak Foun	Peak Foun	Peak Foun	Peak Foun	High
112.2	High	High	High	Peak Foun	Peak Foun	Peak Foun	High	High
123.4	High	High	High	Peak Foun	High	Peak Foun	Peak Foun	High
151.2	High	High	High	Peak Foun	Peak Foun	Peak Foun	Peak Foun	Peak Foun
87.5	High	Peak Foun	High	Peak Foun	High	Peak Foun	Peak Foun	Peak Foun
94.6	High	Peak Foun	Not Found	Peak Foun	Peak Foun	Peak Foun	Peak Foun	Peak Foun
113.3	Peak Foun	High	High	High	Peak Foun	High	High	Peak Foun
100.5	High	Not Found	High	High	Not Found	High	High	High
111.1	High	High	High	Peak Foun	High	High	High	High
136.1	Peak Foun	High	High	Peak Foun	Peak Foun	Peak Foun	Peak Foun	Peak Foun
127.1	Peak Foun	Peak Foun	High	Peak Foun	Not Found	Peak Foun	High	Peak Foun
57.8	Peak Foun	High	Peak Foun	Peak Foun	Peak Foun	High	Peak Foun	Not Found
119.5	High	High	High	High	High	High	High	High
128.3	High	High	Peak Foun	Peak Foun	Peak Foun	High	High	Peak Foun
121.9	High	High	High	Peak Foun	Peak Foun	Peak Foun	Peak Foun	High
128.2	Peak Foun	High	High	Peak Foun	Peak Foun	High	Peak Foun	High
105	Peak Foun	High	High	Peak Foun	High	High	High	Peak Foun
105.3	High	Not Found	High	Not Found	Peak Foun	High	Peak Foun	Peak Foun
104.1	High	Peak Foun	High	High	Peak Foun	High	High	High
103.4	High	High	High	Peak Foun	Peak Foun	High	High	Peak Foun
129.9	Peak Foun	High	High	Peak Foun	Peak Foun	Peak Foun	Peak Foun	High
110.6	Peak Foun	High	High	High	Peak Foun	Peak Foun	Peak Foun	Peak Foun
87.8	High	High	High	Peak Foun	Peak Foun	Peak Foun	Peak Foun	High
142.5	High	High	High	High	Peak Foun	Peak Foun	Peak Foun	Peak Foun
140	Peak Foun	High	High	Peak Foun	Peak Foun	Peak Foun	Peak Foun	Peak Foun
132.8	Peak Foun	Peak Foun	Peak Foun	Peak Foun	Peak Foun	High	Peak Foun	Peak Foun
113.4	High	High	High	High	Peak Foun	High	High	High
96.7	High	High	High	Peak Foun	Not Found	Not Found	Not Found	High
106.5	Peak Foun	Peak Foun	Not Found	Peak Foun	Peak Foun	Peak Foun	Peak Foun	Peak Foun
109.5	Peak Foun	High	High	High	High	Peak Foun	Peak Foun	High
158.6	High	High	High	Peak Foun	Not Found	Not Found	Not Found	Peak Foun
102	High	Peak Foun	Peak Foun	Peak Foun	Peak Foun	Peak Foun	Peak Foun	Peak Foun
103.5	High	High	Peak Foun	High	Peak Foun	Peak Foun	Peak Foun	Peak Foun
111.8	High	High	High	High	Peak Foun	Peak Foun	Peak Foun	Peak Foun
165.1	Not Found	Not Found	High	Not Found	Not Found	Peak Foun	High	Peak Foun
122.3	Peak Foun	Peak Foun	High	Peak Foun	Peak Foun	Peak Foun	Peak Foun	High
99.1	Peak Foun	Peak Foun	High	Peak Foun	High	High	High	High
111	High	High	High	High	Peak Foun	High	High	High
	Peak Foun	Not Found	Peak Foun	Peak Foun	High	High	High	Peak Foun
135.8	High	Peak Foun	Peak Foun	Not Found	Not Found	Peak Foun	Peak Foun	Peak Foun
102.2	High	Peak Foun	Peak Foun	High	Peak Foun	High	Peak Foun	High

171.4	High	Peak Foun	Peak Foun	Peak Foun	Not Found	Not Found	Not Found	Peak Foun
102.3	High	High	High	High	Peak Foun	High	High	High
108.5	High	High	High	High	High	High	High	High
71.8	Peak Foun	High	Peak Foun	Peak Foun	High	Peak Foun	High	Peak Foun
89.7	High	High	High	Peak Foun	High	Peak Foun	Peak Foun	Peak Foun
97.1	Peak Foun	High	High	Peak Foun	Not Found	Peak Foun	High	Peak Foun
139.1	High	High	High	High	Peak Foun	Peak Foun	Peak Foun	Peak Foun
97.7	Peak Foun	High	Not Found	Not Found	Not Found	Not Found	Not Found	Not Found
117.1	High	High	High	Peak Foun	Peak Foun	Not Found	Peak Foun	Peak Foun
114.8	High	High	Peak Foun	Peak Foun	Peak Foun	Peak Foun	Not Found	Peak Foun
95.7	High	High	High	High	High	Peak Foun	Peak Foun	High
119.6	High	Peak Foun	Peak Foun	Peak Foun	Peak Foun	Peak Foun	Peak Foun	High
128.4	High	High	High	High	Peak Foun	Peak Foun	High	High
147.1	High	High	High	High	Peak Foun	High	High	High
90	High	Peak Foun	Peak Foun	Peak Foun	High	High	Peak Foun	Peak Foun
122.3	High	Peak Foun	High	High	High	Peak Foun	Peak Foun	High
66.3	Not Found	Not Found	Peak Foun	Peak Foun	High	High	High	Peak Foun
121.9	High	High	High	Peak Foun	Peak Foun	Peak Foun	High	Peak Foun
89.1	High	High	High	Peak Foun	Not Found	Peak Foun	High	High
100.5	High	Peak Foun	Peak Foun	High	High	Peak Foun	Peak Foun	High
123.7	Not Found	High	High	Peak Foun	Not Found	Not Found	Peak Foun	Peak Foun
111.3	High	Peak Foun	High	Peak Foun	High	Peak Foun	High	High
106.8	High	High	High	Peak Foun	High	High	Peak Foun	Peak Foun
24.8	Not Found	Not Found	Not Found	Peak Foun	High	High	High	Peak Foun
100.1	High	High	High	High	Peak Foun	High	Peak Foun	High
113	High	High	High	High	High	High	High	High
116.3	Peak Foun	High	Peak Foun	Peak Foun	Peak Foun	High	High	Peak Foun
98.6	High	Peak Foun	High	Not Found	Not Found	Peak Foun	Peak Foun	Peak Foun
102.2	High	Peak Foun	High	Peak Foun	Peak Foun	Peak Foun	High	Peak Foun
160.9	Peak Foun	High	Peak Foun	Peak Foun	Peak Foun	Not Found	Peak Foun	High
160.4	Peak Foun	High	Peak Foun	Peak Foun	Peak Foun	Peak Foun	Peak Foun	Peak Foun
113.5	Peak Foun	High	High	Peak Foun	Peak Foun	Peak Foun	High	High
91.2	Peak Foun	High	High	High	Not Found	High	High	Peak Foun
156.5	High	Peak Foun	Peak Foun	High	High	Peak Foun	High	High
	Not Found	Not Found	Not Found	Not Found	High	High	High	Peak Foun
96	High	Peak Foun	High	Peak Foun	Peak Foun	Peak Foun	Peak Foun	High
81	High	High	Peak Foun	High	Not Found	Not Found	Not Found	High
182.4	Peak Foun	High	High	Not Found	Not Found	Peak Foun	Peak Foun	Not Found
143.1	Peak Foun	Peak Foun	Peak Foun	Not Found	Peak Foun	Peak Foun	Peak Foun	High
125.2	High	High	High	Peak Foun	Peak Foun	Peak Foun	Peak Foun	Peak Foun
123.3	High	Peak Foun	High	Peak Foun	Not Found	High	High	Peak Foun
111	High	High	High	Peak Foun	High	Peak Foun	Peak Foun	Peak Foun
100.3	High	Peak Foun	High	Peak Foun	Not Found	Not Found	High	Not Found
153.9	High	High	High	High	Peak Foun	Peak Foun	Peak Foun	Peak Foun
91	Peak Foun	Peak Foun	High	Peak Foun	High	Peak Foun	Peak Foun	Peak Foun

138.3	Peak Foun	Peak Foun	High	Peak Foun	Not Found	Peak Foun	Not Found	Peak Foun
122.2	High	High	High	High	Peak Foun	Peak Foun	High	High
122.3	High	Peak Foun	High	Peak Foun	Not Found	Peak Foun	Not Found	Peak Foun
92.9	High	High	High	Peak Foun	High	Peak Foun	High	High
143.4	High	High	High	High	Peak Foun	Peak Foun	Peak Foun	High
88.1	High	High	High	High	High	High	High	High
108.9	High	High	High	High	Peak Foun	High	High	Peak Foun
113.2	High	High	High	High	Peak Foun	Peak Foun	Peak Foun	High
101.8	High	High	High	High	High	High	High	High
122.8	High	High	Peak Foun	Peak Foun	Peak Foun	Peak Foun	Peak Foun	Peak Foun
146.1	High	High	High	High	High	Peak Foun	High	High
101.4	High	High	Peak Foun	Peak Foun	Peak Foun	Peak Foun	Peak Foun	Peak Foun
96.5	Peak Foun	Peak Foun	Peak Foun	Peak Foun	High	Peak Foun	Peak Foun	Peak Foun
106	Peak Foun	Peak Foun	High	Peak Foun	Peak Foun	Peak Foun	Peak Foun	Peak Foun
129.5	High	Peak Foun	Peak Foun	Peak Foun	Not Found	Peak Foun	Peak Foun	Peak Foun
124.8	High	High	High	High	Peak Foun	Peak Foun	Not Found	Peak Foun
120.3	High	High	High	Peak Foun	Peak Foun	Peak Foun	High	High
107.6	High	High	High	Peak Foun	High	High	High	Peak Foun
75.9	High	High	High	Peak Foun	Peak Foun	High	High	Peak Foun
129.3	High	High	High	High	High	High	Peak Foun	High
127.2	Peak Foun	High	High	Peak Foun	Peak Foun	Peak Foun	High	Peak Foun
120.5	High	High	High	High	High	High	High	High
102.9	High	High	High	High	Peak Foun	High	High	High
102.3	Peak Foun	Peak Foun	High	Peak Foun	Peak Foun	Peak Foun	Peak Foun	High
	Not Found	Not Found	Not Found	Not Found	Not Found	High	Not Found	High
	Not Found	Peak Foun	Peak Foun	Peak Foun	Not Found	Not Found	Not Found	Peak Foun
115	Peak Foun	High	Peak Foun	Peak Foun	Peak Foun	High	High	High
110.5	High	High	Peak Foun	Peak Foun	Peak Foun	High	High	Peak Foun
76.7	High	High	Peak Foun	Peak Foun	Peak Foun	High	Peak Foun	Peak Foun
118.3	Peak Foun	High	High	Peak Foun	Peak Foun	Peak Foun	Peak Foun	High
85.3	Peak Foun	High	High	High	High	High	High	High
104.3	High	High	High	High	High	High	Peak Foun	High
92.8	High	Peak Foun	High	Peak Foun	Not Found	Not Found	Peak Foun	Peak Foun
120.9	Peak Foun	High	High	Peak Foun	Peak Foun	High	High	Peak Foun
92.4	High	High	Peak Foun	Peak Foun	Peak Foun	Peak Foun	High	Peak Foun
110.3	High	High	High	Peak Foun	Peak Foun	Peak Foun	Peak Foun	Peak Foun
91	High	High	High	Peak Foun	Peak Foun	Peak Foun	Peak Foun	Peak Foun
130.3	High	High	High	Peak Foun	Peak Foun	Peak Foun	Peak Foun	Peak Foun
117.1	Peak Foun	High	High	Peak Foun	Peak Foun	Peak Foun	Peak Foun	Peak Foun
140.7	High	High	High	High	Peak Foun	High	Peak Foun	Peak Foun
145.9	Peak Foun	Peak Foun	Peak Foun	Peak Foun	Not Found	Not Found	Peak Foun	Peak Foun
105.1	Peak Foun	High	High	Peak Foun	High	High	High	High
135.5	High	Peak Foun	Not Found	Peak Foun	Not Found	Not Found	Not Found	High
140.7	High	High	High	High	Peak Foun	Peak Foun	Peak Foun	Peak Foun
92.6	High	High	High	Peak Foun	Peak Foun	Peak Foun	Peak Foun	Peak Foun

82.8	High	High	High	High	High	High	Not Found	High
107.3	High	High	High	High	Peak Foun	Peak Foun	Peak Foun	Peak Foun
79.1	High	Not Found	Peak Foun	Peak Foun	Not Found	Not Found	Not Found	Peak Foun
112.8	Peak Foun	Peak Foun	High	Peak Foun	Peak Foun	Peak Foun	High	Peak Foun
154.4	Peak Foun	Peak Foun	Peak Foun	Peak Foun	Not Found	Not Found	Not Found	Not Found
116	High	High	High	High	Peak Foun	Peak Foun	Peak Foun	Peak Foun
90.3	High	High	Peak Foun	Peak Foun	High	Peak Foun	High	Peak Foun
98	High	Peak Foun	High	Peak Foun	High	High	Peak Foun	Peak Foun
118.8	Peak Foun	Peak Foun	High	Peak Foun	Not Found	Not Found	Peak Foun	Peak Foun
99.9	Not Found	High	High	Peak Foun	Peak Foun	High	High	High
105.4	High	Peak Foun	Peak Foun	Peak Foun	Peak Foun	High	High	High
144.7	High	High	Peak Foun	Not Found	Not Found	Peak Foun	Peak Foun	Peak Foun
113.2	High	Peak Foun	Peak Foun	Peak Foun	High	High	High	Peak Foun
120.2	Peak Foun	High	Peak Foun	Not Found	Peak Foun	Peak Foun	Peak Foun	High
134.3	High	High	High	High	Peak Foun	Peak Foun	High	Peak Foun
119.2	High	High	High	High	Peak Foun	High	High	High
125.5	High	High	High	Peak Foun	Peak Foun	Peak Foun	High	High
136.7	High	High	High	High	High	High	High	High
85.5	High	Peak Foun	Not Found	Not Found	High	Peak Foun	High	High
94.5	Peak Foun	High	High	High	High	Peak Foun	High	Peak Foun
120.8	High	High	High	Peak Foun	Peak Foun	Peak Foun	Peak Foun	Peak Foun
97.2	High	Peak Foun	High	Peak Foun	Peak Foun	Peak Foun	High	Peak Foun
114.6	Peak Foun	High	Not Found	Not Found	Not Found	Peak Foun	Not Found	High
119.9	High	High	High	High	Peak Foun	Not Found	Peak Foun	High
103.5	High	High	Peak Foun	Peak Foun	Peak Foun	Peak Foun	High	Peak Foun
113.2	High	High	High	Peak Foun	High	High	High	Peak Foun
93.2	High	Peak Foun	Peak Foun	Peak Foun	Peak Foun	Not Found	Not Found	Peak Foun
70.2	High	Peak Foun	Peak Foun	Peak Foun	Peak Foun	High	Not Found	Peak Foun
115.1	High	High	High	High	High	High	High	High
99.9	High	High	Peak Foun	Peak Foun	Peak Foun	Peak Foun	Peak Foun	Peak Foun
101.8	High	High	High	Peak Foun	Peak Foun	High	Peak Foun	Peak Foun
103.1	High	High	High	High	Not Found	Not Found	High	High
62.4	High	Peak Foun	High	Peak Foun	High	Peak Foun	Peak Foun	Peak Foun
116.9	High	Peak Foun	High	Peak Foun	Peak Foun	Peak Foun	Peak Foun	Peak Foun
135.2	High	High	Peak Foun	High	Not Found	Peak Foun	Peak Foun	Peak Foun
113.8	Peak Foun	Peak Foun	High	Peak Foun	Peak Foun	Peak Foun	Peak Foun	Peak Foun
128.6	High	High	High	Peak Foun	Peak Foun	Peak Foun	Peak Foun	High
123.9	High	High	High	Peak Foun	High	Peak Foun	Peak Foun	Peak Foun
111.7	Peak Foun	High	High	Not Found	High	High	High	High
150	Peak Foun	High	High	Peak Foun	Not Found	Not Found	Not Found	Not Found
115.4	Peak Foun	Peak Foun	High	Peak Foun	Peak Foun	Peak Foun	High	Peak Foun
104	High	Peak Foun	High	High	High	High	High	High
156.4	High	Peak Foun	Peak Foun	Peak Foun	Peak Foun	Not Found	Peak Foun	Peak Foun
111.3	High	High	Peak Foun	Peak Foun	Peak Foun	Peak Foun	Not Found	Peak Foun
117.1	High	High	High	High	High	High	High	Peak Foun

86.7	High	High	High	Peak Foun	High	High	High	Peak Foun
136.5	Peak Foun	High	High	High	Peak Foun	Peak Foun	Peak Foun	Peak Foun
137.7	High	Peak Foun	Peak Foun	Peak Foun	Peak Foun	Peak Foun	High	Peak Foun
99.5	High	High	High	Peak Foun	Peak Foun	High	Peak Foun	High
50.6	Peak Foun	High	Peak Foun	Peak Foun	Not Found	High	Peak Foun	Peak Foun
93.4	Peak Foun	High	Peak Foun	High	High	High	High	Peak Foun
103.1	High	High	High	High	High	High	High	Peak Foun
118.3	High	High	High	High	Peak Foun	Peak Foun	High	High
63.5	Peak Foun	High	High	High	High	Peak Foun	High	High
112.2	High	High	High	Peak Foun	Peak Foun	Peak Foun	High	Peak Foun
128.6	Peak Foun	High	High	Peak Foun	Not Found	Not Found	Not Found	Peak Foun
131.8	Peak Foun	High	High	Peak Foun	Peak Foun	Peak Foun	Peak Foun	Peak Foun
175.2	High	High	Peak Foun	Peak Foun	Peak Foun	Not Found	Peak Foun	Peak Foun
124.6	High	High	High	High	High	Peak Foun	High	High
137.4	High	High	High	Peak Foun	Peak Foun	Peak Foun	Peak Foun	Peak Foun
93.1	Peak Foun	High	High	Peak Foun	High	High	High	Peak Foun
135.6	High	High	High	Peak Foun	Not Found	Not Found	Not Found	Not Found
108.8	High	Peak Foun	Peak Foun	Peak Foun	Not Found	Not Found	Not Found	Not Found
93.6	High	High	High	Peak Foun	Peak Foun	High	Peak Foun	High
96.5	High	High	Peak Foun	Peak Foun	High	High	High	Peak Foun
118.5	Peak Foun	Peak Foun	High	Peak Foun	Peak Foun	Not Found	High	Peak Foun
133.6	High	Peak Foun	Peak Foun	Peak Foun	Peak Foun	Peak Foun	Peak Foun	Peak Foun
126.6	High	High	High	High	Peak Foun	Peak Foun	Peak Foun	Peak Foun
114.1	High	High	High	Peak Foun	Not Found	Not Found	Not Found	Not Found
101.9	High	High	High	High	High	Peak Foun	High	High
	High	High	Not Found	Peak Foun	Peak Foun	Not Found	Not Found	Peak Foun
94.2	Peak Foun	High	High	Peak Foun	Peak Foun	Peak Foun	Peak Foun	Peak Foun
109.1	Peak Foun	Peak Foun	High	Peak Foun	Peak Foun	Peak Foun	High	Peak Foun
108.7	High	High	High	Peak Foun	High	High	High	High
104.2	High	Peak Foun	Peak Foun	Peak Foun	Not Found	Not Found	Not Found	Peak Foun
106.1	Peak Foun	Peak Foun	Peak Foun	Not Found	Not Found	Peak Foun	Peak Foun	Peak Foun
108.5	Peak Foun	Peak Foun	Peak Foun	Peak Foun	Peak Foun	High	High	Peak Foun
137.1	Peak Foun	High	Peak Foun	Peak Foun	Not Found	Peak Foun	Peak Foun	Not Found
105.8	Peak Foun	High	High	Peak Foun	Peak Foun	Peak Foun	Peak Foun	High
133.4	High	High	Peak Foun	High	Peak Foun	Not Found	Peak Foun	Peak Foun
171	High	High	High	High	High	High	High	High
148	High	High	Peak Foun	Not Found	Peak Foun	Peak Foun	Peak Foun	Peak Foun
133.4	High	Peak Foun	Peak Foun	Not Found	Not Found	Peak Foun	Not Found	Peak Foun
152.6	Peak Foun	Peak Foun	Peak Foun	Peak Foun	Peak Foun	High	Peak Foun	High
148.3	Peak Foun	High	High	High	High	High	Peak Foun	Peak Foun
93.6	High	High	Peak Foun	High	High	Peak Foun	Peak Foun	High
76.6	Peak Foun	Peak Foun	Peak Foun	Peak Foun	High	Peak Foun	Peak Foun	Peak Foun
119.5	High	High	High	High	High	High	High	High
88.9	High	Peak Foun	High	High	High	Peak Foun	Peak Foun	Peak Foun
127.3	High	Peak Foun	Peak Foun	Peak Foun	Not Found	Peak Foun	Peak Foun	Peak Foun

138.1	Peak Foun	High	High	Peak Foun	Peak Foun	Peak Foun	Peak Foun	Peak Foun
137.5	High	High	Peak Foun	Not Found	Not Found	Peak Foun	Not Found	Not Found
143	High	Peak Foun	Peak Foun	Peak Foun	Peak Foun	Peak Foun	Peak Foun	Peak Foun
81.2	Peak Foun	High	Peak Foun	Peak Foun	Peak Foun	High	Peak Foun	Peak Foun
93	Peak Foun	Peak Foun	High	Peak Foun	High	High	High	High
113.4	High	Peak Foun	High	Peak Foun	Peak Foun	Peak Foun	Peak Foun	Peak Foun
109.2	High	Peak Foun	High	Peak Foun	Peak Foun	Peak Foun	Peak Foun	Peak Foun
96.7	High	Peak Foun	Peak Foun	Peak Foun	High	Peak Foun	High	Peak Foun
134.4	High	High	High	High	Not Found	Not Found	Not Found	Peak Foun
128.7	Not Found	Peak Foun	Peak Foun	Not Found	Not Found	High	High	Peak Foun
146	Peak Foun	Peak Foun	Peak Foun	Peak Foun	Peak Foun	Not Found	Not Found	Not Found
111.6	High	High	High	Peak Foun	Peak Foun	High	Peak Foun	Peak Foun
107.1	High	High	High	High	High	High	High	Peak Foun
106.3	High	High	High	High	High	High	High	High
129.2	High	High	High	Peak Foun	Peak Foun	Peak Foun	High	Peak Foun
89.2	Peak Foun	Peak Foun	High	Peak Foun	Not Found	Peak Foun	High	Peak Foun
105.5	Peak Foun	High	Peak Foun	Peak Foun	Peak Foun	Peak Foun	Peak Foun	Peak Foun
99.5	High	High	High	High	High	High	High	Peak Foun
102.3	High	High	High	Peak Foun	Peak Foun	Peak Foun	Peak Foun	Peak Foun
91.8	Peak Foun	Peak Foun	High	Peak Foun	Peak Foun	High	Peak Foun	Peak Foun
86.7	Peak Foun	High	Peak Foun	Peak Foun	Peak Foun	Not Found	Peak Foun	Peak Foun
	Not Found	Not Found	Peak Foun	Peak Foun	High	Peak Foun	Peak Foun	Peak Foun
110.9	High	High	High	High	Peak Foun	High	High	Peak Foun
92.7	High	High	High	High	Peak Foun	High	High	High
109.7	High	High	High	Peak Foun	Not Found	Not Found	Not Found	Peak Foun
118.5	Peak Foun	Peak Foun	High	Peak Foun	High	High	High	Peak Foun
93.4	Not Found	Not Found	Not Found	Not Found	High	Peak Foun	Peak Foun	Peak Foun
116.5	High	High	High	Peak Foun	Peak Foun	Peak Foun	High	Peak Foun
115.5	High	Peak Foun	High	Not Found	High	High	Peak Foun	Peak Foun
94.1	Peak Foun	High	Peak Foun	Peak Foun	Peak Foun	High	Peak Foun	Peak Foun
103.9	High	Peak Foun	High	High	High	Peak Foun	Peak Foun	Peak Foun
127.8	High	High	High	Peak Foun	Peak Foun	High	High	High
92.6	High	High	High	High	High	High	Peak Foun	Peak Foun
153.2	High	High	High	High	High	Peak Foun	Peak Foun	High
110.2	High	High	High	High	High	High	Peak Foun	Peak Foun
108.2	Peak Foun	Peak Foun	High	Peak Foun	Peak Foun	Peak Foun	Peak Foun	Peak Foun
103.7	High	High	Peak Foun	Peak Foun	Peak Foun	Peak Foun	Peak Foun	High
140.1	Peak Foun	High	Not Found	Not Found	Not Found	Not Found	Peak Foun	High
53.7	Not Found	High	High	Peak Foun	High	High	High	Peak Foun
107.3	High	High	Peak Foun	Peak Foun	Peak Foun	High	High	Peak Foun
	Not Found	Peak Foun	High	Not Found	Not Found	High	Not Found	Not Found
112.8	High	High	High	High	Peak Foun	Peak Foun	High	Peak Foun
87.3	High	High	High	High	High	High	High	High
66.5	High	High	Peak Foun	Peak Foun	Peak Foun	Peak Foun	Peak Foun	Peak Foun
77.6	High	Peak Foun	Peak Foun	Peak Foun	High	High	Peak Foun	Peak Foun

114	Peak Foun	Peak Foun	Peak Foun	Peak Foun	High	Peak Foun	High	Peak Foun
107.6	High	High	Peak Foun	Peak Foun	Peak Foun	Peak Foun	High	Peak Foun
128.8	Peak Foun	Not Found	Not Found	Not Found	Not Found	Not Found	Not Found	Not Found
115.1	High	High	High	Peak Foun	Not Found	Not Found	Not Found	Not Found
95.1	High	High	High	High	Peak Foun	Peak Foun	High	High
101.6	High	High	High	Peak Foun	Peak Foun	Peak Foun	Peak Foun	Peak Foun
111.1	High	Peak Foun	High	Peak Foun	Peak Foun	High	Peak Foun	Peak Foun
115.5	High	High	High	High	Peak Foun	Peak Foun	High	High
93.8	Peak Foun	Peak Foun	Peak Foun	High	High	Peak Foun	Peak Foun	Peak Foun
135.7	Peak Foun	Peak Foun	Peak Foun	Peak Foun	Peak Foun	Peak Foun	Peak Foun	Peak Foun
108.1	High	High	Peak Foun	Peak Foun	Peak Foun	Not Found	Not Found	Peak Foun
103.6	Peak Foun	Peak Foun	High	High	Not Found	Peak Foun	Peak Foun	Peak Foun
158.6	Not Found	Peak Foun	High	Peak Foun	Peak Foun	Peak Foun	Peak Foun	Peak Foun
92.3	High	High	High	High	Not Found	Not Found	Not Found	Not Found
93.4	Peak Foun	Peak Foun	Peak Foun	Peak Foun	High	Peak Foun	Peak Foun	Peak Foun
99.5	High	High	Peak Foun	High	High	High	Peak Foun	Peak Foun
68.2	High	High	Peak Foun	Peak Foun	Not Found	Not Found	Not Found	Peak Foun
114.2	High	Not Found	Peak Foun	Not Found	High	High	Not Found	Not Found
107.8	High	High	High	Peak Foun	Peak Foun	Peak Foun	Peak Foun	Peak Foun
105.9	High	High	High	High	Peak Foun	Peak Foun	High	High
123.6	High	Peak Foun	Peak Foun	Peak Foun	Peak Foun	Peak Foun	Peak Foun	Peak Foun
168.7	High	High	High	Peak Foun	Peak Foun	Peak Foun	High	High
98.6	High	High	High	Peak Foun	Peak Foun	Peak Foun	Peak Foun	Peak Foun
103.8	High	Peak Foun	Peak Foun	Peak Foun	High	Peak Foun	Peak Foun	Peak Foun
112.4	High	High	Peak Foun	Peak Foun	Peak Foun	High	High	Peak Foun
183.2	High	High	High	High	Peak Foun	High	Peak Foun	High
103.7	High	High	High	Peak Foun	Peak Foun	Peak Foun	High	Peak Foun
109.6	High	High	High	High	High	High	Peak Foun	Peak Foun
90	High	High	High	High	High	Peak Foun	Peak Foun	High
114.3	High	High	Peak Foun	Peak Foun	High	Peak Foun	Peak Foun	Peak Foun
94.4	High	Peak Foun	Peak Foun	Peak Foun	Not Found	Not Found	Not Found	Peak Foun
120.6	Peak Foun	Peak Foun	Peak Foun	Peak Foun	Peak Foun	High	Peak Foun	High
102.1	High	High	High	High	High	High	High	High
118.5	High	High	High	High	Peak Foun	High	High	High
96.3	High	High	Peak Foun	High	High	High	High	High
135.3	High	High	Peak Foun	Not Found	Peak Foun	Peak Foun	Not Found	Peak Foun
94.2	High	Not Found	High	High	Peak Foun	Peak Foun	Peak Foun	High
115.5	High	High	High	High	High	High	High	High
116.9	Not Found	High	Not Found	Not Found	Not Found	Peak Foun	Peak Foun	Peak Foun
147.6	High	High	High	Peak Foun	Peak Foun	Peak Foun	Peak Foun	Peak Foun
88.4	Peak Foun	High	Peak Foun	Peak Foun	Not Found	Peak Foun	Peak Foun	Peak Foun
85	High	High	High	High	High	High	High	High
130.5	High	High	High	High	High	High	High	High
148.7	Peak Foun	Peak Foun	High	Peak Foun	Peak Foun	Peak Foun	High	High
108.1	High	High	High	High	Peak Foun	High	High	High

92.3	High	High	Peak Foun	High	High	High	Peak Foun	Peak Foun
111.9	Peak Foun	Peak Foun	Peak Foun	Peak Foun	High	Peak Foun	Peak Foun	High
130.2	Peak Foun	Peak Foun	High	Peak Foun	Peak Foun	Peak Foun	Peak Foun	Peak Foun
99	High	High	High	High	High	High	High	High
140.9	Peak Foun	High	High	High	Peak Foun	Peak Foun	Peak Foun	High
110.5	Peak Foun	High	High	Peak Foun	Peak Foun	Peak Foun	Peak Foun	Peak Foun
107.6	High	High	High	High	Peak Foun	High	High	High
107.4	Peak Foun	High	High	High	Peak Foun	High	High	High
111.1	High	Peak Foun	High	Peak Foun	Peak Foun	Peak Foun	Peak Foun	Peak Foun
128.7	Peak Foun	Peak Foun	Peak Foun	Peak Foun	Not Found	Not Found	Peak Foun	High
114.9	High	High	High	High	Peak Foun	Peak Foun	Peak Foun	High
110.3	High	High	High	High	Peak Foun	Peak Foun	High	High
133.1	High	High	Peak Foun	Peak Foun	Peak Foun	Peak Foun	Peak Foun	Peak Foun
106.2	Peak Foun	Peak Foun	High	Peak Foun	Peak Foun	High	High	High
165.6	Peak Foun	Peak Foun	Peak Foun	Peak Foun	Peak Foun	Peak Foun	Not Found	Peak Foun
113.2	High	High	Peak Foun	Peak Foun	Peak Foun	Peak Foun	Peak Foun	Peak Foun
146	High	Peak Foun	High	Peak Foun	Not Found	Not Found	Not Found	Peak Foun
81.4	Peak Foun	Peak Foun	High	Peak Foun	High	Peak Foun	Peak Foun	Peak Foun
118.4	High	Peak Foun	High	Peak Foun	Not Found	Peak Foun	High	Peak Foun
173.9	High	Not Found	Peak Foun	Peak Foun	High	High	High	Peak Foun
115	Peak Foun	Peak Foun	Not Found	Peak Foun	Not Found	Peak Foun	High	Peak Foun
124.8	High	High	Peak Foun	Peak Foun	Peak Foun	Peak Foun	Peak Foun	Peak Foun
68.5	High	Peak Foun	High	Peak Foun	Peak Foun	Peak Foun	High	High
110.1	High	High	High	Peak Foun	Peak Foun	Peak Foun	High	Peak Foun
147.5	High	High	Peak Foun	Peak Foun	Peak Foun	Peak Foun	Peak Foun	High
116.1	High	High	High	High	High	High	High	High
124.7	High	Peak Foun	Peak Foun	Peak Foun	Peak Foun	Peak Foun	Peak Foun	Peak Foun
100	Peak Foun	Peak Foun	Peak Foun	Not Found	Not Found	Not Found	Not Found	Not Found
85.6	High	Peak Foun	Peak Foun	Peak Foun	High	Peak Foun	High	Not Found
121.5	High	High	High	Peak Foun	Peak Foun	Peak Foun	Peak Foun	Peak Foun
120.3	Peak Foun	Peak Foun	Peak Foun	Not Found	Not Found	High	Peak Foun	Peak Foun
127.6	High	High	High	Peak Foun	High	High	High	Peak Foun
129.1	High	High	Peak Foun	High	Peak Foun	High	Peak Foun	Peak Foun
77.6	High	High	High	Peak Foun	Peak Foun	Peak Foun	Peak Foun	Peak Foun
110.8	High	High	High	Peak Foun	Peak Foun	Peak Foun	High	Peak Foun
97.9	High	High	Peak Foun	Peak Foun	Peak Foun	High	High	Peak Foun
94	Peak Foun	Peak Foun	Not Found	Not Found	Peak Foun	Not Found	Not Found	Peak Foun
108.4	High	High	High	Peak Foun	High	High	High	Peak Foun
118.1	High	High	High	High	Peak Foun	High	High	High
131.2	High	Peak Foun	High	Peak Foun	Not Found	Not Found	Peak Foun	Peak Foun
104.8	High	High	High	High	High	Peak Foun	High	High
122.3	High	Peak Foun	Peak Foun	Peak Foun	Peak Foun	Peak Foun	Peak Foun	Peak Foun
157	High	High	High	Peak Foun	High	Peak Foun	Peak Foun	Peak Foun
102	Peak Foun	High	Peak Foun	Peak Foun	Not Found	Not Found	Peak Foun	Peak Foun
97.8	High	High	High	High	High	High	High	High

106.1	High	High	High	Peak Foun	Peak Foun	Peak Foun	Peak Foun	Peak Foun
131	High	Peak Foun	High	Peak Foun	Not Found	Not Found	Peak Foun	Not Found
91.8	High	Peak Foun	High	Peak Foun	Peak Foun	Peak Foun	Peak Foun	Peak Foun
121.7	High	High	High	Peak Foun	Not Found	Not Found	Not Found	Peak Foun
63.6	High	High	High	High	Not Found	Not Found	Peak Foun	Peak Foun
63.6	Peak Foun	Peak Foun	High	Peak Foun	Not Found	Peak Foun	Peak Foun	Not Found
102.8	Peak Foun	Not Found	Peak Foun	Peak Foun	Not Found	Not Found	Not Found	Not Found
88.3	High	Peak Foun	Peak Foun	Peak Foun	High	High	High	High
104.4	High	High	High	High	Not Found	High	High	High
102.2	High	High	High	Peak Foun	Peak Foun	Peak Foun	Peak Foun	Peak Foun
99.3	Peak Foun	High	Peak Foun	High	Peak Foun	Peak Foun	High	Peak Foun
97.3	Peak Foun	Not Found	Peak Foun	Peak Foun	High	Not Found	Peak Foun	High
125.6	Peak Foun	High	High	Peak Foun	Peak Foun	High	High	High
98.6	Peak Foun	High	High	Peak Foun	Peak Foun	Not Found	Peak Foun	Peak Foun
122.1	High	High	High	High	Peak Foun	Peak Foun	Peak Foun	High
106.6	High	High	High	Not Found	High	High	High	Peak Foun
122.3	High	High	High	Peak Foun	Peak Foun	Not Found	Not Found	Peak Foun
144.4	Peak Foun	High	High	Peak Foun	Peak Foun	Peak Foun	High	Peak Foun
104.4	High	High	High	Peak Foun	Peak Foun	High	High	Peak Foun
106	Peak Foun	Peak Foun	Peak Foun	Peak Foun	Peak Foun	High	High	High
95.6	Peak Foun	Peak Foun	Peak Foun	Peak Foun	Not Found	Not Found	Not Found	Peak Foun
111.2	Peak Foun	Not Found	Not Found	Peak Foun	Not Found	Not Found	Not Found	High
39.2	High	High	High	High	Peak Foun	High	High	Not Found
111.2	High	High	High	High	Peak Foun	Peak Foun	Peak Foun	High
43	High	High	Peak Foun	Peak Foun	Peak Foun	Peak Foun	Peak Foun	Peak Foun
102	High	Peak Foun	Peak Foun	Peak Foun	Peak Foun	High	High	Peak Foun
109.6	High	Peak Foun	Peak Foun	Peak Foun	Peak Foun	Peak Foun	Peak Foun	Peak Foun
98.4	Not Found	High	Not Found	High	Not Found	Peak Foun	Peak Foun	Peak Foun
129.3	Peak Foun	High	High	Peak Foun	Not Found	Peak Foun	Peak Foun	Peak Foun
0.4	High	High	High	Peak Foun	Peak Foun	Peak Foun	Peak Foun	Peak Foun
118.7	High	Peak Foun	Peak Foun	Peak Foun	Peak Foun	Peak Foun	Peak Foun	Peak Foun
103.3	High	High	High	Peak Foun	Peak Foun	Peak Foun	Peak Foun	Peak Foun
96.3	Peak Foun	Peak Foun	Peak Foun	Peak Foun	Peak Foun	Peak Foun	Peak Foun	Peak Foun
100.6	High	High	High	High	High	High	High	High
104.3	High	High	High	High	High	Peak Foun	Peak Foun	Peak Foun
120.2	High	High	High	High	High	High	High	High
80.4	High	High	High	High	High	High	High	Peak Foun
140.4	High	Peak Foun	High	Peak Foun	Peak Foun	Peak Foun	Peak Foun	Not Found
	High	Not Found	Peak Foun	Peak Foun	Not Found	Not Found	Not Found	Peak Foun
132.2	Not Found	High	Peak Foun	Not Found	Peak Foun	Not Found	Not Found	Not Found
94.1	Peak Foun	Peak Foun	High	Not Found	Not Found	Peak Foun	High	Peak Foun
110.3	Peak Foun	High	Peak Foun	Peak Foun	Not Found	Not Found	Not Found	Peak Foun
86.5	High	Peak Foun	Peak Foun	High	High	High	High	High
70	High	High	High	High	Peak Foun	Peak Foun	Peak Foun	High
128.3	High	High	High	Peak Foun	High	Peak Foun	Peak Foun	Peak Foun

98.4	Peak Foun	High	High	High	High	High	Peak Foun	High
138.5	Peak Foun	Peak Foun	Peak Foun	Not Found	Peak Foun	Peak Foun	Peak Foun	Not Found
104.9	Peak Foun	Peak Foun	Peak Foun	Not Found	Not Found	Peak Foun	Not Found	Peak Foun
99	Peak Foun	Peak Foun	High	Peak Foun	Peak Foun	Not Found	Not Found	Peak Foun
115.2	High	Peak Foun	High	Not Found	High	Peak Foun	Peak Foun	Peak Foun
119.9	Peak Foun	Peak Foun	High	Peak Foun	High	High	Peak Foun	Peak Foun
124	Not Found	Peak Foun	Not Found	Peak Foun	Not Found	Not Found	Peak Foun	Peak Foun
113.3	High	High	High	High	Peak Foun	High	High	Peak Foun
119.4	High	Peak Foun	High	Peak Foun	Peak Foun	Peak Foun	Peak Foun	Peak Foun
112.3	High	Peak Foun	High	Peak Foun	High	High	High	High
108.2	Peak Foun	Peak Foun	Peak Foun	High	Peak Foun	Peak Foun	High	High
121.7	Peak Foun	High	Peak Foun	Peak Foun	Peak Foun	Peak Foun	Peak Foun	Peak Foun
111.7	High	Peak Foun	Peak Foun	Not Found	Not Found	Not Found	Not Found	Peak Foun
83.2	Peak Foun	Peak Foun	High	High	Peak Foun	Peak Foun	High	Peak Foun
89.6	High	Peak Foun	High	Peak Foun	Peak Foun	Peak Foun	Peak Foun	Peak Foun
108.1	High	High	High	Peak Foun	Not Found	High	High	Not Found
106.3	High	High	High	High	High	High	High	High
131.1	High	High	High	Peak Foun	Peak Foun	Peak Foun	Peak Foun	Peak Foun
87.2	High	High	High	Peak Foun	Peak Foun	High	High	High
99.8	High	High	High	High	High	High	High	High
115.1	Peak Foun	High	Peak Foun	Not Found	Not Found	Not Found	Peak Foun	Peak Foun
97.3	High	High	High	High	High	High	High	High
146.4	High	High	High	High	Peak Foun	Peak Foun	Peak Foun	Peak Foun
95.7	High	High	Peak Foun	Peak Foun	Not Found	Not Found	Not Found	High
77.8	Peak Foun	High	High	Peak Foun	Peak Foun	Peak Foun	High	Peak Foun
146.9	High	High	High	High	High	Peak Foun	High	Peak Foun
125	Peak Foun	Peak Foun	High	Peak Foun	Not Found	Peak Foun	Peak Foun	Peak Foun
171.4	High	High	High	High	Peak Foun	High	High	High
97	Peak Foun	Peak Foun	High	High	Peak Foun	Peak Foun	High	Peak Foun
123.2	High	High	Peak Foun	Peak Foun	Peak Foun	Peak Foun	Peak Foun	Peak Foun
125.5	High	High	High	Peak Foun	Peak Foun	High	High	Peak Foun
83.9	High	Peak Foun	Peak Foun	Peak Foun	High	Peak Foun	Peak Foun	High
84.3	Peak Foun	High	Peak Foun	Peak Foun	Peak Foun	Peak Foun	High	Peak Foun
79.5	Peak Foun	Peak Foun	Peak Foun	High	High	Peak Foun	Peak Foun	Peak Foun
125.2	Peak Foun	Not Found	High	Not Found	Peak Foun	Not Found	Not Found	Not Found
106	Peak Foun	High	High	High	Not Found	Not Found	High	Not Found
118.3	High	Peak Foun	Peak Foun	Peak Foun	Peak Foun	Peak Foun	Peak Foun	Peak Foun
92.1	High	High	High	Peak Foun	Peak Foun	Peak Foun	High	Peak Foun
115.4	Peak Foun	Peak Foun	Peak Foun	High	Peak Foun	Peak Foun	Peak Foun	Peak Foun
93.7	High	High	High	Peak Foun	Not Found	Peak Foun	High	Peak Foun
98.9	High	High	High	High	High	High	High	High
110.4	High	High	High	High	Not Found	Not Found	Peak Foun	High
115	Peak Foun	High	Peak Foun	High	Peak Foun	High	High	High
107.1	High	High	High	High	High	Peak Foun	Peak Foun	High
106.1	Peak Foun	Peak Foun	High	Not Found	Not Found	Not Found	High	Peak Foun

105.9	High	Peak Foun	High	Peak Foun	High	Peak Foun	High	Peak Foun
105.5	Peak Foun	High	High	Peak Foun	Peak Foun	Peak Foun	Peak Foun	Peak Foun
154.8	Peak Foun	Peak Foun	High	Peak Foun	Peak Foun	Peak Foun	Peak Foun	Peak Foun
83.1	High	High	High	Peak Foun	High	High	High	High
112.9	Peak Foun	Peak Foun	Not Found	Peak Foun	High	High	Peak Foun	Peak Foun
112.2	Peak Foun	Peak Foun	High	Peak Foun	Peak Foun	Peak Foun	Peak Foun	Peak Foun
107.2	High	High	High	High	High	High	High	High
106.8	High	Peak Foun	High	Peak Foun	Peak Foun	Peak Foun	Peak Foun	Peak Foun
114.5	High	High	High	High	Peak Foun	Peak Foun	Peak Foun	Peak Foun
131	High	Peak Foun	High	Peak Foun	Peak Foun	Peak Foun	Peak Foun	Peak Foun
107.9	Peak Foun	Not Found	High	Peak Foun	Not Found	Not Found	Not Found	Not Found
96.8	Peak Foun	Peak Foun	Peak Foun	Peak Foun	High	Peak Foun	Peak Foun	Peak Foun
108.3	Peak Foun	Peak Foun	Peak Foun	High	Peak Foun	Peak Foun	Peak Foun	Peak Foun
102.5	High	High	Peak Foun	High	High	Peak Foun	Peak Foun	Peak Foun
88	Peak Foun	High	High	High	Not Found	Peak Foun	High	High
102.9	High	High	Peak Foun	High	Not Found	High	High	High
124.7	Peak Foun	High	Peak Foun	Peak Foun	Peak Foun	Peak Foun	Peak Foun	Peak Foun
162.5	Not Found	Not Found	High	Not Found	Not Found	Not Found	Not Found	Not Found
118.8	High	High	Peak Foun	Peak Foun	High	High	Peak Foun	High
135.3	High	Peak Foun	High	Peak Foun	Peak Foun	Peak Foun	High	Peak Foun
104.6	Peak Foun	Peak Foun	Peak Foun	Peak Foun	Peak Foun	Peak Foun	Peak Foun	Peak Foun
96.1	High	High	High	Peak Foun	Not Found	Not Found	Not Found	Not Found
125.2	High	High	Peak Foun	High	Not Found	Not Found	Not Found	Peak Foun
124.1	Peak Foun	Peak Foun	Not Found	Peak Foun	Peak Foun	High	Peak Foun	Peak Foun
116.5	High	High	Peak Foun	Peak Foun	Peak Foun	Peak Foun	Not Found	Peak Foun
119.8	High	High	High	Peak Foun	Not Found	Peak Foun	Peak Foun	High
105.6	Peak Foun	Peak Foun	Peak Foun	Peak Foun	Peak Foun	High	Peak Foun	Peak Foun
133.3	High	Peak Foun	High	Peak Foun	High	Peak Foun	Peak Foun	High
141.5	High	High	High	High	Peak Foun	Peak Foun	Peak Foun	Peak Foun
102.7	High	High	High	Peak Foun	Peak Foun	High	High	High
131.4	High	Peak Foun	High	High	Not Found	Peak Foun	High	High
101.6	High	High	High	High	High	High	Peak Foun	Peak Foun
158.4	High	Peak Foun	High	Not Found	Peak Foun	Peak Foun	Peak Foun	Peak Foun
125.5	Peak Foun	Peak Foun	High	Peak Foun	Not Found	Peak Foun	Peak Foun	Peak Foun
109	High	High	Peak Foun	Peak Foun	High	Peak Foun	High	High
116.9	High	High	Peak Foun	Not Found	Not Found	Not Found	Not Found	Not Found
77	Peak Foun	High	High	High	Peak Foun	Peak Foun	Peak Foun	Peak Foun
112.9	Peak Foun	Peak Foun	Peak Foun	Peak Foun	Peak Foun	Not Found	Not Found	Peak Foun
80.5	Peak Foun	Peak Foun	Peak Foun	High	Peak Foun	Peak Foun	Peak Foun	Peak Foun
107.8	High	Peak Foun	Peak Foun	Peak Foun	Peak Foun	Peak Foun	Peak Foun	Peak Foun
99.7	High	High	High	Peak Foun	Not Found	Not Found	Not Found	Peak Foun
115.9	Peak Foun	High	Peak Foun	Peak Foun	High	High	Peak Foun	Peak Foun
109.6	High	Peak Foun	High	High	Peak Foun	High	High	Peak Foun
114	High	High	High	Peak Foun	Peak Foun	High	High	Peak Foun
	High	High	High	Not Found	High	High	High	Not Found

101.2	High	High	Peak Foun	High	Not Found	Peak Foun	High	High
124.3	High	Peak Foun	Peak Foun	Peak Foun	High	Peak Foun	Peak Foun	Peak Foun
129.1	Peak Foun	High	High	Peak Foun	Peak Foun	Peak Foun	Peak Foun	Peak Foun
110.7	High	High	Peak Foun	Peak Foun	Not Found	Peak Foun	High	Peak Foun
116.3	High	Peak Foun	Peak Foun	Peak Foun	Peak Foun	High	High	Peak Foun
58.7	High	High	High	High	High	High	High	Peak Foun
95.6	High	High	High	High	High	Peak Foun	High	High
104.5	High	High	Peak Foun	High	Not Found	Not Found	Not Found	High
64.8	Peak Foun	Peak Foun	Peak Foun	Peak Foun	High	High	High	High
78.6	High	Peak Foun	Peak Foun	Peak Foun	High	High	High	Not Found
122.9	Not Found	Not Found	Peak Foun	Not Found	Not Found	Peak Foun	Peak Foun	Peak Foun
148.4	High	High	High	Peak Foun	Peak Foun	Peak Foun	Peak Foun	High
	Not Found	Peak Foun	Not Found	Not Found	Not Found	High	Peak Foun	High
98	High	High	High	Peak Foun	Peak Foun	High	High	Peak Foun
88.6	Peak Foun	Peak Foun	Peak Foun	Peak Foun	Not Found	Peak Foun	High	Not Found
134.1	High	High	High	High	Peak Foun	High	Peak Foun	High
	Not Found	Not Found	Not Found	Not Found	Peak Foun	High	Not Found	Not Found
157.9	Peak Foun	Peak Foun	High	Peak Foun	Peak Foun	High	Peak Foun	Peak Foun
100	High	Peak Foun	High	Peak Foun	Peak Foun	Peak Foun	Peak Foun	Peak Foun
110.8	Peak Foun	Peak Foun	High	High	Not Found	High	High	High
113.3	High	Peak Foun	High	Peak Foun	Not Found	Not Found	Not Found	Peak Foun
114	High	High	High	High	Peak Foun	High	High	High
106.9	High	Peak Foun	High	Peak Foun	High	Peak Foun	Peak Foun	Peak Foun
107.7	High	High	High	High	Peak Foun	Peak Foun	High	High
114.8	High	Peak Foun	Peak Foun	Peak Foun	Not Found	Peak Foun	Peak Foun	Not Found
113	High	High	High	Peak Foun	Not Found	Not Found	Not Found	Not Found
84.2	High	High	High	Peak Foun	High	High	High	High
103.6	Peak Foun	Peak Foun	Peak Foun	Peak Foun	Not Found	Not Found	Peak Foun	Not Found
133.2	High	Peak Foun	High	Peak Foun	High	High	High	High
118	High	High	High	High	High	High	Peak Foun	High
87.5	High	Peak Foun	Peak Foun	Peak Foun	Peak Foun	Peak Foun	Not Found	Peak Foun
117.7	Peak Foun	High	High	Peak Foun	Peak Foun	Peak Foun	Peak Foun	Peak Foun
92.5	Peak Foun	Not Found	Peak Foun	Peak Foun	Peak Foun	Peak Foun	Peak Foun	Peak Foun
111	High	High	Peak Foun	Peak Foun	High	High	Peak Foun	Peak Foun
171.5	High	Peak Foun	High	Not Found	Peak Foun	High	Peak Foun	Not Found
134.9	High	High	High	Peak Foun	Not Found	High	Peak Foun	Not Found
92.6	High	High	High	High	Peak Foun	Peak Foun	High	Peak Foun
102.9	High	High	High	Peak Foun	Peak Foun	Peak Foun	Peak Foun	Peak Foun
114	High	High	High	High	Not Found	Not Found	Peak Foun	Peak Foun
105.8	High	High	Peak Foun	Peak Foun	Peak Foun	Peak Foun	Peak Foun	Peak Foun
119.1	Peak Foun	Peak Foun	Peak Foun	Peak Foun	Peak Foun	Peak Foun	Peak Foun	Peak Foun
104.9	Peak Foun	Peak Foun	High	Peak Foun	Peak Foun	Peak Foun	High	Peak Foun
102.4	Not Found	Peak Foun	Not Found	Not Found	Not Found	Not Found	Not Found	Not Found
132.5	Peak Foun	High	High	High	Peak Foun	High	High	Peak Foun
107.1	High	High	High	Peak Foun	Not Found	Peak Foun	High	Peak Foun

160	High	Peak Foun	High	Peak Foun	Not Found	Peak Foun	Peak Foun	Not Found
102.1	Not Found	Peak Foun	Not Found	Not Found	Not Found	Not Found	Not Found	Not Found
142.6	Peak Foun	Peak Foun	High	Peak Foun	Peak Foun	Peak Foun	Peak Foun	Peak Foun
127.6	High	High	High	High	Peak Foun	Peak Foun	Peak Foun	High
46.1	Peak Foun	High	Peak Foun	Peak Foun	Peak Foun	Peak Foun	Peak Foun	Peak Foun
83.4	Peak Foun	Peak Foun	High	Peak Foun	Not Found	Peak Foun	Not Found	Peak Foun
115.5	High	High	High	High	Not Found	High	High	High
94.2	High	Peak Foun	Peak Foun	Peak Foun	Peak Foun	Peak Foun	Peak Foun	Peak Foun
107.8	High	Peak Foun	High	Peak Foun	Peak Foun	Peak Foun	High	Peak Foun
134.5	High	High	High	Peak Foun	Peak Foun	Peak Foun	Peak Foun	Peak Foun
156.1	High	High	High	Peak Foun	Peak Foun	Peak Foun	Peak Foun	Peak Foun
120.1	High	High	High	High	Peak Foun	Peak Foun	High	High
94.7	High	Peak Foun	Peak Foun	Not Found	High	Peak Foun	Peak Foun	Not Found
126.9	Not Found	High	High	Not Found	Not Found	Peak Foun	Peak Foun	Not Found
100.4	Peak Foun	Peak Foun	High	Peak Foun	Peak Foun	Peak Foun	Peak Foun	High
66.5	High	Peak Foun	Peak Foun	Not Found	High	High	Peak Foun	Peak Foun
116.6	High	High	Not Found	Peak Foun	Peak Foun	Peak Foun	Peak Foun	Not Found
102.7	High	Peak Foun	Peak Foun	Peak Foun	High	High	High	Peak Foun
106.2	High	High	High	Peak Foun	High	High	High	High
119	Peak Foun	Peak Foun	Peak Foun	Peak Foun	Peak Foun	Not Found	Peak Foun	Peak Foun
123.2	High	High	Peak Foun	Peak Foun	Peak Foun	Peak Foun	Peak Foun	Peak Foun
	Not Found	Not Found	Not Found	Not Found	High	Peak Foun	Peak Foun	Not Found
94.4	High	Peak Foun	Peak Foun	Peak Foun	Peak Foun	Peak Foun	Peak Foun	Peak Foun
86.2	Not Found	Not Found	Not Found	Not Found	Not Found	Not Found	Not Found	Peak Foun
135.3	Peak Foun	Peak Foun	High	Peak Foun	Peak Foun	Peak Foun	High	Peak Foun
25.6	High	High	High	High	Peak Foun	Not Found	Not Found	Not Found
126.7	High	Not Found	Peak Foun	Not Found	Peak Foun	Peak Foun	Peak Foun	High
97	Peak Foun	Peak Foun	Peak Foun	Peak Foun	High	High	Peak Foun	Peak Foun
171.9	Peak Foun	Peak Foun	Not Found	Not Found	Peak Foun	Not Found	Not Found	Peak Foun
104.2	High	Peak Foun	Peak Foun	Peak Foun	Peak Foun	Peak Foun	High	Peak Foun
94.1	Not Found	Not Found	Not Found	Not Found	Peak Foun	Not Found	Not Found	Not Found
112.7	Peak Foun	Peak Foun	Peak Foun	Not Found	Peak Foun	Not Found	Not Found	Peak Foun
125.5	Peak Foun	Peak Foun	Peak Foun	Peak Foun	Peak Foun	Not Found	Not Found	Peak Foun
112.1	High	Peak Foun	Peak Foun	High	Peak Foun	Peak Foun	High	Peak Foun
95.6	Peak Foun	High	Peak Foun	Peak Foun	High	High	High	High
107.8	High	High	High	High	Peak Foun	Peak Foun	Peak Foun	Peak Foun
91.5	Peak Foun	Peak Foun	High	Peak Foun	High	High	High	Peak Foun
118.1	Peak Foun	Not Found	Peak Foun	Peak Foun	Not Found	Not Found	Peak Foun	Peak Foun
145.6	Peak Foun	High	High	Peak Foun	Not Found	Not Found	Peak Foun	Peak Foun
113	High	Peak Foun	High	Peak Foun	Peak Foun	Peak Foun	High	Peak Foun
105.6	Peak Foun	High	High	Not Found	Not Found	Peak Foun	Peak Foun	High
98.7	High	High	High	Peak Foun	High	Peak Foun	Peak Foun	Peak Foun
135.1	High	Peak Foun	High	Peak Foun	Not Found	Peak Foun	Not Found	Peak Foun
98.9	High	Peak Foun	Not Found	Peak Foun	Peak Foun	Peak Foun	Peak Foun	High
107.3	High	High	High	High	Peak Foun	High	High	High

123.1	Peak Foun	High	Peak Foun	Peak Foun	Peak Foun	Peak Foun	Peak Foun	Peak Foun
96.4	Peak Foun	Peak Foun	Peak Foun	Peak Foun	Peak Foun	Peak Foun	Peak Foun	Peak Foun
77.9	Peak Foun	Peak Foun	Peak Foun	Peak Foun	High	Peak Foun	Peak Foun	Peak Foun
200	Not Found	Not Found	Not Found	Not Found	Not Found	Not Found	Not Found	Not Found
114.2	Peak Foun	Peak Foun	Peak Foun	Peak Foun	Peak Foun	Peak Foun	Peak Foun	Peak Foun
142.2	High	Peak Foun	High	Not Found	Peak Foun	High	High	Peak Foun
115.1	High	High	High	High	Peak Foun	High	High	Peak Foun
115	High	High	High	High	Peak Foun	Peak Foun	Peak Foun	High
109.6	High	Peak Foun	Peak Foun	Peak Foun	Peak Foun	Peak Foun	Peak Foun	Peak Foun
83	High	Peak Foun	High	Not Found	Not Found	Peak Foun	High	Not Found
94.5	Peak Foun	Peak Foun	High	High	Not Found	Peak Foun	Peak Foun	Peak Foun
89.8	Peak Foun	Peak Foun	Peak Foun	Peak Foun	Peak Foun	High	Peak Foun	Peak Foun
94.7	Peak Foun	Peak Foun	Peak Foun	Peak Foun	Peak Foun	High	High	High
106.1	High	High	Peak Foun	High	Not Found	Peak Foun	Peak Foun	Peak Foun
132.1	Peak Foun	Peak Foun	High	Peak Foun	Peak Foun	Peak Foun	Peak Foun	Peak Foun
113.1	High	High	High	High	Peak Foun	High	High	Peak Foun
154.7	High	High	Peak Foun	Peak Foun	Peak Foun	Peak Foun	Peak Foun	Peak Foun
115.5	High	High	High	High	Peak Foun	Peak Foun	Peak Foun	High
120.6	High	High	High	Peak Foun	Not Found	Not Found	Peak Foun	Not Found
121	Peak Foun	High	High	Peak Foun	Peak Foun	High	Peak Foun	Peak Foun
90.8	High	High	Peak Foun	Not Found	High	Not Found	Not Found	Peak Foun
122.8	Peak Foun	High	High	Peak Foun	Peak Foun	Peak Foun	Peak Foun	Peak Foun
93.2	High	Peak Foun	Peak Foun	Peak Foun	High	High	High	Peak Foun
122	High	High	High	High	Peak Foun	High	High	Peak Foun
112.6	High	High	High	High	High	Peak Foun	High	High
93.4	Peak Foun	Peak Foun	High	High	High	Peak Foun	High	High
79	Peak Foun	Peak Foun	High	Peak Foun	Peak Foun	Peak Foun	Peak Foun	Peak Foun
123.4	High	High	Peak Foun	Peak Foun	Not Found	Peak Foun	Peak Foun	Peak Foun
63.2	Peak Foun	Peak Foun	Peak Foun	Peak Foun	Peak Foun	Peak Foun	Peak Foun	Peak Foun
87.7	Peak Foun	Peak Foun	Peak Foun	Not Found	Peak Foun	High	High	Not Found
106.9	High	High	High	High	Peak Foun	High	High	High
87.7	Peak Foun	Peak Foun	Not Found	Not Found	Not Found	Not Found	Not Found	Not Found
120.1	Peak Foun	High	High	Peak Foun	Peak Foun	High	High	High
117.5	High	High	Peak Foun	Peak Foun	Peak Foun	Peak Foun	Peak Foun	Not Found
109.9	Not Found	High	High	Not Found	Not Found	Not Found	High	Peak Foun
107.5	High	High	Peak Foun	High	Not Found	Peak Foun	Peak Foun	Peak Foun
80.6	Not Found	High	Not Found	Not Found	Not Found	Not Found	Not Found	Not Found
99.2	Peak Foun	Peak Foun	Peak Foun	Not Found	Not Found	High	Peak Foun	Peak Foun
134.6	High	High	Peak Foun	High	Peak Foun	Peak Foun	Peak Foun	Peak Foun
85.5	High	High	High	High	Peak Foun	Peak Foun	Peak Foun	High
127.8	High	Peak Foun	High	Peak Foun	Peak Foun	Peak Foun	Peak Foun	Peak Foun
112.3	Peak Foun	Peak Foun	High	Peak Foun	Not Found	Peak Foun	Peak Foun	Peak Foun
84.8	High	High	High	High	Peak Foun	Peak Foun	High	High
108.5	Peak Foun	Peak Foun	Peak Foun	High	Peak Foun	High	High	Peak Foun
126.1	High	High	Peak Foun	Peak Foun	Peak Foun	Peak Foun	High	Peak Foun

139.4	Not Found	High	Not Found	Peak Foun	Peak Foun	High	Peak Foun	Not Found
98.3	High	Not Found	Not Found	Peak Foun	Not Found	Not Found	Not Found	Peak Foun
104.8	Peak Foun	High	Not Found	Not Found	Not Found	High	Not Found	Not Found
71.9	Peak Foun	Peak Foun	Peak Foun	Peak Foun	Peak Foun	Peak Foun	Peak Foun	Peak Foun
	High	High	High	High	Peak Foun	High	High	Not Found
125	Peak Foun	High	Peak Foun	Peak Foun	Not Found	Not Found	Peak Foun	Peak Foun
103.4	Peak Foun	High	Not Found	Peak Foun	Peak Foun	Peak Foun	Peak Foun	Peak Foun
106.5	High	High	High	Peak Foun	High	High	High	High
94.1	Peak Foun	Peak Foun	Peak Foun	Peak Foun	High	Peak Foun	High	Peak Foun
99.1	High	High	High	High	High	High	High	Peak Foun
127.1	High	Peak Foun	Peak Foun	Peak Foun	Peak Foun	Peak Foun	Peak Foun	Peak Foun
105.3	Peak Foun	High	Not Found	High	Not Found	High	Not Found	High
94.8	High	High	High	High	High	High	High	High
112.4	Peak Foun	Peak Foun	High	Not Found	Not Found	High	Not Found	Not Found
108.5	Peak Foun	Peak Foun	High	Not Found	Peak Foun	High	Not Found	Peak Foun
88.8	Peak Foun	Peak Foun	Peak Foun	Not Found	Peak Foun	High	Peak Foun	Not Found
95.7	High	High	Peak Foun	High	Peak Foun	High	Peak Foun	Peak Foun
86.7	Peak Foun	High	High	High	Peak Foun	High	High	Peak Foun
112.4	Peak Foun	Peak Foun	Peak Foun	Not Found	Not Found	Peak Foun	Not Found	Peak Foun
96.7	Peak Foun	High	Not Found	Not Found	Not Found	Not Found	Not Found	Not Found
99.4	Peak Foun	Peak Foun	Peak Foun	Peak Foun	Not Found	Peak Foun	Peak Foun	Peak Foun
98.2	Peak Foun	High	High	High	Peak Foun	Peak Foun	High	Peak Foun
98.9	Peak Foun	High	High	Peak Foun	Peak Foun	Peak Foun	High	Peak Foun
107	Peak Foun	Peak Foun	Peak Foun	Peak Foun	Not Found	Not Found	Not Found	Not Found
67.5	Peak Foun	High	Peak Foun	Peak Foun	Peak Foun	Not Found	Not Found	Not Found
156.7	Peak Foun	Not Found	Not Found	Not Found	Peak Foun	Peak Foun	Not Found	Not Found
115.9	High	High	High	High	Not Found	Peak Foun	Peak Foun	Peak Foun
144.5	Peak Foun	Peak Foun	Peak Foun	Not Found	Peak Foun	High	Peak Foun	Peak Foun
101.4	High	High	Peak Foun	Peak Foun	Not Found	Not Found	Not Found	Not Found
103.2	High	High	High	High	High	High	High	High
72.8	Peak Foun	Peak Foun	High	High	Not Found	Peak Foun	High	Peak Foun
118.2	Not Found	Peak Foun	Peak Foun	Peak Foun	Peak Foun	Not Found	Peak Foun	Peak Foun
87.5	High	Peak Foun	High	Peak Foun	Not Found	High	High	Peak Foun
113	Peak Foun	Not Found	Not Found	Not Found	Not Found	Not Found	High	Peak Foun
125.4	High	Peak Foun	Peak Foun	Not Found	Not Found	Peak Foun	Peak Foun	Peak Foun
134.9	High	Peak Foun	Peak Foun	Peak Foun	Peak Foun	Peak Foun	Peak Foun	Peak Foun
110.8	Peak Foun	Peak Foun	High	Peak Foun	Peak Foun	Peak Foun	High	High
113	Peak Foun	Peak Foun	Peak Foun	Not Found	Peak Foun	Peak Foun	Peak Foun	Peak Foun
91.7	High	High	Peak Foun	Peak Foun	High	High	Peak Foun	Peak Foun
142.9	Not Found	Peak Foun	Peak Foun	Not Found	Not Found	High	Peak Foun	Not Found
151.6	High	Peak Foun	Peak Foun	Peak Foun	Peak Foun	Peak Foun	Peak Foun	Peak Foun
150.7	High	Peak Foun	High	High	Peak Foun	Peak Foun	Not Found	Peak Foun
125.9	Peak Foun	High	High	High	Peak Foun	Peak Foun	Peak Foun	Peak Foun
113.2	High	High	Peak Foun	Peak Foun	Peak Foun	Peak Foun	Peak Foun	High
118	High	High	High	Not Found	Peak Foun	High	Peak Foun	High

117.7	High	High	High	Peak Foun	Peak Foun	High	High	Peak Foun
80.7	High	Peak Foun	Peak Foun	Peak Foun	High	Peak Foun	Peak Foun	Not Found
101.1	High	High	High	High	High	High	High	High
107	High	High	Peak Foun	Peak Foun	Peak Foun	High	Peak Foun	Peak Foun
143.8	Peak Foun	Peak Foun	Peak Foun	Peak Foun	Peak Foun	Peak Foun	Peak Foun	High
124	High	High	High	Peak Foun	Peak Foun	High	Peak Foun	High
103.6	Peak Foun	Peak Foun	High	Not Found	Not Found	Not Found	Not Found	Not Found
111.7	High	Peak Foun	Peak Foun	Peak Foun	Peak Foun	Peak Foun	Peak Foun	Peak Foun
88.8	High	Not Found	High	Peak Foun	High	Peak Foun	Peak Foun	Peak Foun
50.6	Not Found	High	Not Found	Peak Foun	Peak Foun	High	Peak Foun	Not Found
47.1	Peak Foun	Peak Foun	Peak Foun	Peak Foun	High	High	High	Peak Foun
117.2	High	High	High	High	Peak Foun	Peak Foun	High	High
114.8	Peak Foun	High	High	High	Peak Foun	Peak Foun	Peak Foun	Not Found
112.5	Peak Foun	Peak Foun	Peak Foun	Peak Foun	Not Found	Not Found	Peak Foun	Peak Foun
112.5	Peak Foun	High	High	High	Peak Foun	Peak Foun	High	High
123	Peak Foun	Peak Foun	High	High	Peak Foun	Peak Foun	Peak Foun	High
117.4	Peak Foun	Peak Foun	Peak Foun	Not Found	Peak Foun	High	Peak Foun	Peak Foun
115.7	High	High	High	Peak Foun	High	High	Peak Foun	Not Found
115.3	Peak Foun	High	High	Peak Foun	Not Found	Not Found	Not Found	Peak Foun
105.5	Peak Foun	Peak Foun	Peak Foun	Peak Foun	Not Found	Not Found	Not Found	Peak Foun
63.7	Peak Foun	High	Peak Foun	Peak Foun	Peak Foun	High	Peak Foun	Peak Foun
105.7	High	High	High	Not Found	Peak Foun	Peak Foun	Peak Foun	High
128.9	High	Peak Foun	Peak Foun	Peak Foun	Not Found	Peak Foun	Peak Foun	Peak Foun
110.8	Peak Foun	High	High	Peak Foun	High	High	High	Peak Foun
115.2	High	High	Peak Foun	Peak Foun	High	High	Peak Foun	Peak Foun
114.1	High	High	High	Peak Foun	High	High	High	High
117.7	Peak Foun	Peak Foun	Peak Foun	Not Found	Peak Foun	Peak Foun	Peak Foun	Peak Foun
108.2	High	Peak Foun	High	Peak Foun	Not Found	Not Found	Peak Foun	High
99.3	High	High	High	High	High	Peak Foun	High	Peak Foun
80.8	High	High	Peak Foun	Peak Foun	Peak Foun	High	Peak Foun	Peak Foun
64.2	High	High	High	High	High	Peak Foun	High	High
100.3	Peak Foun	High	Peak Foun	High	High	High	Peak Foun	High
109.4	Peak Foun	High	Peak Foun	Peak Foun	Not Found	Peak Foun	Peak Foun	Peak Foun
	Not Found	Peak Foun	Not Found	Not Found	Peak Foun	Peak Foun	Not Found	Not Found
91.8	High	High	High	High	Not Found	Peak Foun	Peak Foun	Peak Foun
114.1	Peak Foun	High	Peak Foun	Peak Foun	Peak Foun	High	Peak Foun	High
140.2	Peak Foun	Peak Foun	Peak Foun	Not Found	Not Found	Not Found	Peak Foun	Peak Foun
85	Peak Foun	Peak Foun	High	Peak Foun	Not Found	Peak Foun	Peak Foun	Not Found
95.4	Peak Foun	Peak Foun	Peak Foun	Peak Foun	High	High	Peak Foun	Peak Foun
69.5	Peak Foun	Not Found	Peak Foun	Peak Foun	Peak Foun	High	Peak Foun	Peak Foun
103.5	Peak Foun	High	High	Peak Foun	Peak Foun	Peak Foun	Not Found	Peak Foun
120.1	High	High	Peak Foun	Peak Foun	High	Peak Foun	Peak Foun	Peak Foun
104.6	Peak Foun	High	High	Not Found	Not Found	Not Found	Not Found	Peak Foun
	Not Found	Peak Foun	Peak Foun	Not Found	High	High	Peak Foun	Not Found
129.4	High	Peak Foun	Peak Foun	High	Peak Foun	Peak Foun	Not Found	High

88.5	Peak Foun	Peak Foun	Peak Foun	Peak Foun	Not Found	Not Found	Not Found	Peak Foun
115.2	High	Peak Foun	High	Peak Foun	Peak Foun	Peak Foun	Peak Foun	Peak Foun
98.2	Peak Foun	Peak Foun	Peak Foun	Peak Foun	High	Peak Foun	Peak Foun	Peak Foun
70.6	Peak Foun	Not Found	Peak Foun	Not Found	Not Found	Not Found	Peak Foun	Not Found
111.5	Not Found	High	Peak Foun	High	Not Found	Not Found	Not Found	Not Found
137.1	Peak Foun	Peak Foun	Peak Foun	Peak Foun	High	Peak Foun	Peak Foun	Peak Foun
119.1	High	High	High	Peak Foun	Peak Foun	Peak Foun	Peak Foun	Peak Foun
110.5	Not Found	Peak Foun	Peak Foun	Not Found	Not Found	High	Not Found	Peak Foun
86.2	Peak Foun	Peak Foun	Peak Foun	Peak Foun	Not Found	Not Found	Not Found	Peak Foun
91.8	Peak Foun	Peak Foun	High	Peak Foun	Peak Foun	Peak Foun	Peak Foun	Peak Foun
119	Peak Foun	Peak Foun	Peak Foun	Peak Foun	Peak Foun	Peak Foun	Not Found	Not Found
97.6	High	High	Peak Foun	Peak Foun	High	High	High	Peak Foun
96.7	High	Peak Foun	Peak Foun	High	Peak Foun	Peak Foun	Not Found	Peak Foun
110.2	High	High	High	Peak Foun	Peak Foun	Peak Foun	Peak Foun	Peak Foun
97.1	High	High	Peak Foun	Not Found	Peak Foun	Peak Foun	High	Peak Foun
120.9	High	High	High	Peak Foun	Peak Foun	Not Found	Peak Foun	Peak Foun
95.4	Peak Foun	High	Peak Foun	Peak Foun	Not Found	Peak Foun	Peak Foun	Peak Foun
145.4	High	High	High	Not Found	Not Found	Not Found	Peak Foun	Peak Foun
116.5	High	High	High	Peak Foun	Peak Foun	Peak Foun	Peak Foun	Peak Foun
109.2	Peak Foun	High	High	High	Peak Foun	Not Found	Peak Foun	Peak Foun
118.2	Peak Foun	Peak Foun	High	Peak Foun	Peak Foun	Not Found	Not Found	Peak Foun
134.6	Peak Foun	Peak Foun	Not Found	Peak Foun	Peak Foun	Peak Foun	High	High
115.6	High	Peak Foun	High	Peak Foun	Peak Foun	Peak Foun	Peak Foun	Peak Foun
133	Peak Foun	Peak Foun	High	Peak Foun	Peak Foun	High	High	High
	High	Peak Foun	High	Not Found	High	High	High	High
52.7	Peak Foun	High	Peak Foun	Peak Foun	Peak Foun	Peak Foun	Peak Foun	Peak Foun
119.4	High	Peak Foun	Peak Foun	Not Found	Peak Foun	Not Found	Not Found	Peak Foun
112.7	Peak Foun	Not Found	Not Found	Not Found	Not Found	Not Found	Peak Foun	Peak Foun
89.2	Peak Foun	High	Peak Foun	Peak Foun	High	High	High	High
111.6	High	High	High	Peak Foun	Peak Foun	Peak Foun	Peak Foun	Peak Foun
123.1	High	Not Found	High	High	High	Peak Foun	Peak Foun	Peak Foun
91.9	High	Not Found	Not Found	Peak Foun	High	High	Peak Foun	Peak Foun
124.8	High	High	Peak Foun	Not Found	Peak Foun	Peak Foun	Peak Foun	Peak Foun
143.7	High	High	High	Peak Foun	Peak Foun	Peak Foun	Peak Foun	Peak Foun
122.5	High	Peak Foun	Peak Foun	Peak Foun	Peak Foun	Peak Foun	Not Found	Peak Foun
117.9	Not Found	Peak Foun	High	Not Found	Peak Foun	Peak Foun	Peak Foun	Peak Foun
82.3	High	Peak Foun	Peak Foun	Not Found	Peak Foun	Peak Foun	Not Found	Not Found
116.4	High	Peak Foun	High	High	Peak Foun	Peak Foun	Peak Foun	Peak Foun
133	High	High	High	Peak Foun	Peak Foun	High	Peak Foun	Peak Foun
100.4	Peak Foun	Peak Foun	High	Peak Foun	Peak Foun	Peak Foun	Peak Foun	Peak Foun
130	Peak Foun	High	High	High	Peak Foun	High	High	High
47	High	High	High	Peak Foun	Peak Foun	Peak Foun	Peak Foun	Peak Foun
115.2	High	High	Peak Foun	Peak Foun	Peak Foun	Not Found	Not Found	Peak Foun
110	High	High	High	High	High	High	High	High
140	High	High	High	Peak Foun	Peak Foun	Not Found	Not Found	Peak Foun

99.2	Peak Foun	Peak Foun	High	Peak Foun	Not Found	Not Found	Peak Foun	Peak Foun
96.7	Peak Foun	Peak Foun	Peak Foun	Peak Foun	Peak Foun	Peak Foun	High	Peak Foun
108.5	Peak Foun	High	High	Peak Foun	Not Found	Peak Foun	Peak Foun	Peak Foun
151.3	Peak Foun	Not Found	High	Peak Foun	Not Found	Not Found	High	Peak Foun
108.1	Peak Foun	Peak Foun	Peak Foun	Not Found	Not Found	High	High	Peak Foun
118.8	Not Found	Not Found	Peak Foun	Peak Foun	Not Found	Not Found	Not Found	Not Found
87.3	Peak Foun	High	Peak Foun	Peak Foun	Peak Foun	Peak Foun	Peak Foun	Peak Foun
64.2	High	Not Found	Not Found	Peak Foun	Not Found	Not Found	Not Found	Peak Foun
142.3	High	High	High	High	Peak Foun	Peak Foun	Peak Foun	Peak Foun
78	Not Found	Peak Foun	Peak Foun	Not Found	Not Found	High	Not Found	Not Found
137.5	High	High	High	Peak Foun	Peak Foun	Peak Foun	Not Found	High
65.8	Peak Foun	Peak Foun	Not Found	Not Found	High	High	High	Peak Foun
109.4	High	High	High	Peak Foun	Not Found	High	Peak Foun	Peak Foun
71.9	High	High	High	Peak Foun	Peak Foun	High	Peak Foun	Peak Foun
122.9	High	Peak Foun	High	Peak Foun	Not Found	Not Found	Peak Foun	Peak Foun
134.5	Not Found	Not Found	Peak Foun	Peak Foun	Not Found	Not Found	Not Found	Not Found
118.4	Peak Foun	High	Peak Foun	Peak Foun	Peak Foun	Peak Foun	Peak Foun	Peak Foun
119	Peak Foun	Peak Foun	High	Not Found	Not Found	Peak Foun	Peak Foun	Peak Foun
112.4	Peak Foun	Peak Foun	Peak Foun	Peak Foun	Peak Foun	Peak Foun	Peak Foun	Peak Foun
140.3	Peak Foun	Peak Foun	Peak Foun	Peak Foun	Peak Foun	Not Found	Peak Foun	Peak Foun
122.8	High	High	Peak Foun	High	Peak Foun	High	Not Found	Peak Foun
111.7	High	High	High	High	High	High	High	High
116.1	High	High	High	Peak Foun	Peak Foun	Peak Foun	Peak Foun	Peak Foun
92.4	Peak Foun	Peak Foun	High	Peak Foun	Peak Foun	Not Found	Not Found	Not Found
82.6	High	High	Not Found	High	Peak Foun	Not Found	Not Found	Peak Foun
104.3	High	Peak Foun	Peak Foun	High	Peak Foun	High	High	Peak Foun
64.5	Peak Foun	Peak Foun	Peak Foun	Not Found	High	Peak Foun	Peak Foun	Peak Foun
120.7	Peak Foun	Peak Foun	Not Found	Peak Foun	Peak Foun	Peak Foun	High	Peak Foun
132.7	High	High	Peak Foun	Peak Foun	Peak Foun	Not Found	Not Found	Peak Foun
148.7	Peak Foun	High	Peak Foun	Peak Foun	Peak Foun	Peak Foun	Not Found	Peak Foun
140.3	Peak Foun	High	High	High	Peak Foun	Peak Foun	High	High
106.6	High	High	High	High	Peak Foun	High	High	High
115.8	Peak Foun	Peak Foun	Not Found	Not Found	Not Found	High	Not Found	Not Found
41.3	Not Found	Not Found	Peak Foun	Not Found	Peak Foun	Peak Foun	High	Not Found
106.8	High	High	Peak Foun	Peak Foun	Peak Foun	Peak Foun	Peak Foun	Peak Foun
117.5	Peak Foun	Peak Foun	Peak Foun	Peak Foun	Not Found	Peak Foun	High	Peak Foun
105.9	High	Peak Foun	Peak Foun	Peak Foun	High	Peak Foun	Peak Foun	Peak Foun
125.3	Peak Foun	Peak Foun	Peak Foun	Peak Foun	High	Not Found	Not Found	Not Found
135.8	Peak Foun	Peak Foun	Peak Foun	Peak Foun	Peak Foun	Peak Foun	Peak Foun	Peak Foun
93.8	High	High	Peak Foun	Peak Foun	Peak Foun	Peak Foun	Peak Foun	Peak Foun
109.6	Peak Foun	High	High	Peak Foun	Not Found	High	High	Peak Foun
117.7	High	High	High	Peak Foun	Peak Foun	Peak Foun	Peak Foun	Peak Foun
123	Peak Foun	High	High	Peak Foun	Peak Foun	High	High	Peak Foun
117.5	Peak Foun	High	High	Peak Foun	Peak Foun	Peak Foun	Peak Foun	Peak Foun
105.9	Peak Foun	High	Peak Foun	Not Found	Peak Foun	High	Not Found	Peak Foun

117	Peak Foun	Peak Foun	Peak Foun	Peak Foun	Peak Foun	Peak Foun	High	High
101	Peak Foun	High	High	Peak Foun	Peak Foun	High	High	High
94.5	Peak Foun	High	Peak Foun	Peak Foun	Peak Foun	Peak Foun	Peak Foun	Peak Foun
79.7	High	Peak Foun	Not Found	Not Found	Not Found	Not Found	Not Found	Not Found
141.9	High	High	Peak Foun	Not Found	Peak Foun	Peak Foun	Peak Foun	Peak Foun
131.3	Peak Foun	High	High	Peak Foun	Peak Foun	High	Peak Foun	High
131.1	High	High	Peak Foun	High	Peak Foun	Peak Foun	Peak Foun	Peak Foun
104.6	High	Peak Foun	Not Found	Not Found	Not Found	Peak Foun	Not Found	Not Found
117.7	Peak Foun	Peak Foun	High	Peak Foun	Peak Foun	Peak Foun	Peak Foun	Peak Foun
105.7	Peak Foun	High	High	Peak Foun	Peak Foun	Peak Foun	Peak Foun	Peak Foun
114.1	High	High	High	High	High	High	High	High
133	Peak Foun	Peak Foun	Peak Foun	Peak Foun	Not Found	Peak Foun	Not Found	Peak Foun
120.7	Peak Foun	High	High	High	Peak Foun	High	Peak Foun	Peak Foun
138.7	Peak Foun	Peak Foun	Peak Foun	Peak Foun	Not Found	Peak Foun	Peak Foun	Peak Foun
124.9	High	High	Peak Foun	Peak Foun	Peak Foun	Peak Foun	Peak Foun	Not Found
125.4	High	High	Peak Foun	Peak Foun	Peak Foun	Peak Foun	Peak Foun	Peak Foun
121.1	Peak Foun	High	High	Peak Foun	Peak Foun	High	High	Peak Foun
130.1	High	High	High	Peak Foun	High	High	High	High
110.5	Peak Foun	High	Peak Foun	Peak Foun	Not Found	High	High	Peak Foun
119.9	Peak Foun	High	High	Peak Foun	Peak Foun	High	Peak Foun	Peak Foun
139.2	High	High	Peak Foun	Peak Foun	Peak Foun	Not Found	Peak Foun	Peak Foun
22.8	Peak Foun	Peak Foun	Peak Foun	Peak Foun	Not Found	Not Found	Not Found	Not Found
114	Peak Foun	Peak Foun	Peak Foun	Peak Foun	Not Found	Peak Foun	Not Found	Not Found
111.3	High	High	High	Not Found	Peak Foun	Not Found	Not Found	Not Found
85.4	High	High	Peak Foun	Not Found	Not Found	Not Found	Not Found	Peak Foun
120.5	High	Peak Foun	High	High	Peak Foun	Peak Foun	High	Peak Foun
114.7	Peak Foun	Peak Foun	High	Peak Foun	Not Found	Peak Foun	High	Peak Foun
115.1	High	High	High	High	Peak Foun	Not Found	Peak Foun	Peak Foun
123.3	Peak Foun	Peak Foun	High	Peak Foun	Not Found	Not Found	Peak Foun	Peak Foun
95.4	Peak Foun	Peak Foun	Not Found	Not Found	Not Found	Not Found	Peak Foun	Not Found
90.6	Peak Foun	Peak Foun	High	High	Not Found	Not Found	Peak Foun	Peak Foun
114.8	Peak Foun	Peak Foun	High	Peak Foun	Not Found	Not Found	Peak Foun	Peak Foun
138.2	Peak Foun	High	Peak Foun	Peak Foun	Peak Foun	Peak Foun	Peak Foun	Peak Foun
117.6	High	High	Peak Foun	High	High	High	Peak Foun	High
81.6	Peak Foun	High	High	Peak Foun	Peak Foun	High	Peak Foun	Peak Foun
97.8	High	Peak Foun	Peak Foun	Peak Foun	Peak Foun	High	Peak Foun	Peak Foun
91.7	Peak Foun	Peak Foun	High	High	High	High	Peak Foun	High
129.2	High	High	Peak Foun	Peak Foun	Not Found	Peak Foun	Peak Foun	Peak Foun
106.1	High	High	Peak Foun	Peak Foun	High	Peak Foun	Peak Foun	Peak Foun
128.9	Peak Foun	Peak Foun	Peak Foun	Peak Foun	Peak Foun	Peak Foun	Peak Foun	Peak Foun
91	High	Peak Foun	Peak Foun	Peak Foun	Peak Foun	Peak Foun	High	Peak Foun
118.2	Peak Foun	Peak Foun	High	High	Peak Foun	Peak Foun	High	Peak Foun
113.6	Peak Foun	High	Not Found	Peak Foun	High	Not Found	Not Found	Not Found
136.9	Peak Foun	Peak Foun	Peak Foun	Peak Foun	Peak Foun	High	Peak Foun	Peak Foun
99.3	High	Peak Foun	Peak Foun	Peak Foun	Not Found	Peak Foun	Peak Foun	Peak Foun

89.6	Peak Foun	Peak Foun	High	Peak Foun	Not Found	Peak Foun	Peak Foun	Peak Foun
111.8	High	High	High	Peak Foun	High	High	Peak Foun	Peak Foun
106.9	High	High	Peak Foun	Peak Foun	High	Peak Foun	High	High
105.6	Peak Foun	High	High	Peak Foun	Peak Foun	Peak Foun	Peak Foun	Peak Foun
126.2	Peak Foun	High	High	High	Not Found	Not Found	Not Found	Peak Foun
118	Peak Foun	Peak Foun	Peak Foun	Peak Foun	Not Found	Not Found	High	Peak Foun
93.7	Peak Foun	Peak Foun	Peak Foun	Peak Foun	Peak Foun	Peak Foun	Peak Foun	Peak Foun
	Peak Foun	High	High	Not Found	Not Found	Not Found	Not Found	Not Found
124.6	High	High	High	High	Peak Foun	Not Found	Peak Foun	Peak Foun
105.4	Peak Foun	Peak Foun	Peak Foun	Peak Foun	Not Found	Peak Foun	Peak Foun	Peak Foun
124.6	Peak Foun	High	Peak Foun	Peak Foun	Peak Foun	Not Found	Not Found	Not Found
60.7	High	Peak Foun	Peak Foun	Not Found	High	Peak Foun	Peak Foun	Peak Foun
	Not Found	Not Found	Not Found	Not Found	High	Peak Foun	Not Found	Not Found
148.8	Peak Foun	Peak Foun	Peak Foun	Not Found	Not Found	Peak Foun	Peak Foun	Peak Foun
53.5	Peak Foun	Peak Foun	High	Not Found	Not Found	Not Found	Not Found	Peak Foun
116.5	Peak Foun	High	High	Peak Foun	Not Found	Peak Foun	Peak Foun	Not Found
126.4	Peak Foun	Peak Foun	High	Peak Foun	Peak Foun	Peak Foun	High	Peak Foun
	Not Found	Not Found	High	Peak Foun	Not Found	Not Found	Not Found	Not Found
116.7	Not Found	Peak Foun	Not Found	Not Found	Not Found	Not Found	Not Found	Not Found
118	Peak Foun	Peak Foun	Not Found	Not Found	Peak Foun	Peak Foun	High	Peak Foun
89	Peak Foun	Peak Foun	High	Not Found	Peak Foun	High	High	Peak Foun
89.8	High	High	High	Peak Foun	Not Found	Peak Foun	High	Peak Foun
125.5	Peak Foun	Peak Foun	High	Peak Foun	Peak Foun	Peak Foun	Peak Foun	High
113	High	High	High	Peak Foun	Peak Foun	Peak Foun	Peak Foun	Peak Foun
104.3	High	High	High	Peak Foun	Not Found	Not Found	Peak Foun	Not Found
101.1	Not Found	High	Peak Foun	Not Found	Not Found	Peak Foun	Peak Foun	Not Found
101.1	High	High	High	High	High	High	Peak Foun	High
98.9	Peak Foun	Peak Foun	Peak Foun	Peak Foun	Not Found	Not Found	Peak Foun	Peak Foun
178.1	High	Peak Foun	Peak Foun	Not Found	Not Found	Not Found	Peak Foun	Peak Foun
96.2	High	High	High	High	Peak Foun	High	High	High
101.1	Peak Foun	Peak Foun	Peak Foun	Peak Foun	Peak Foun	Peak Foun	Peak Foun	Peak Foun
90.4	Peak Foun	Peak Foun	High	Not Found	Not Found	Not Found	Not Found	Not Found
115.5	Peak Foun	Peak Foun	High	Peak Foun	Not Found	Peak Foun	High	Not Found
91.7	High	Peak Foun	High	Peak Foun	Not Found	Not Found	Not Found	Peak Foun
90.8	Peak Foun	Peak Foun	Peak Foun	Peak Foun	Peak Foun	Peak Foun	Peak Foun	Not Found
107.7	High	High	Not Found	Not Found	Not Found	Not Found	Not Found	Not Found
134.4	High	Peak Foun	Peak Foun	Not Found	Not Found	Peak Foun	Peak Foun	Not Found
73.8	High	High	High	Peak Foun	Peak Foun	High	Peak Foun	Peak Foun
102	High	Not Found	Peak Foun	Peak Foun	Not Found	Peak Foun	Peak Foun	Not Found
93.7	Peak Foun	Peak Foun	Peak Foun	Peak Foun	Peak Foun	High	Peak Foun	Peak Foun
119.2	High	Peak Foun	Peak Foun	Peak Foun	Peak Foun	Peak Foun	Peak Foun	Peak Foun
116.1	Peak Foun	Peak Foun	Peak Foun	Peak Foun	Peak Foun	Not Found	Peak Foun	Peak Foun
107.8	Peak Foun	High	High	Peak Foun	Peak Foun	Peak Foun	Peak Foun	Peak Foun
121.8	Peak Foun	Not Found	Not Found	Not Found	Peak Foun	Not Found	Not Found	Peak Foun
95	High	High	High	High	High	High	High	Peak Foun

83.9	Peak Foun	Peak Foun	Peak Foun	Peak Foun	Peak Foun	High	High	Peak Foun
126.8	High	High	High	High	High	High	Peak Foun	Peak Foun
110.9	High	Peak Foun	Peak Foun	Not Found	Peak Foun	Peak Foun	Peak Foun	Peak Foun
96.8	High	Peak Foun	Peak Foun	Peak Foun	Peak Foun	Peak Foun	Peak Foun	Peak Foun
114.1	Peak Foun	High	High	Peak Foun	Not Found	Peak Foun	Peak Foun	Peak Foun
85.6	High	Not Found	High	Peak Foun	Not Found	Not Found	Peak Foun	Peak Foun
131	High	High	Peak Foun	Peak Foun	Peak Foun	High	High	Peak Foun
113.6	Peak Foun	High	High	High	High	Peak Foun	High	High
93.1	Peak Foun	Peak Foun	Peak Foun	Peak Foun	Peak Foun	Peak Foun	Peak Foun	Peak Foun
92.1	Peak Foun	Peak Foun	Peak Foun	Peak Foun	Peak Foun	Peak Foun	High	Peak Foun
104.8	High	High	Peak Foun	Peak Foun	Peak Foun	Peak Foun	Not Found	Peak Foun
119	Peak Foun	Peak Foun	Not Found	Peak Foun	Not Found	Peak Foun	Not Found	Not Found
90	Peak Foun	Peak Foun	High	Peak Foun	Peak Foun	Not Found	Peak Foun	Peak Foun
104.3	Peak Foun	Peak Foun	Peak Foun	Peak Foun	Peak Foun	Peak Foun	Peak Foun	Peak Foun
83.2	Not Found	Peak Foun	Not Found	Not Found	High	High	Peak Foun	Peak Foun
98.8	High	Peak Foun	Peak Foun	Peak Foun	Peak Foun	High	High	Peak Foun
91.8	Peak Foun	Peak Foun	Not Found	Peak Foun	Not Found	Not Found	Not Found	High
109	High	High	Peak Foun	Not Found	Not Found	Not Found	Not Found	Peak Foun
112.7	Peak Foun	Peak Foun	Peak Foun	Peak Foun	Not Found	Not Found	Not Found	Not Found
152.1	High	High	Peak Foun	Peak Foun	Peak Foun	Peak Foun	Peak Foun	Not Found
106.8	High	High	High	Peak Foun	Peak Foun	Peak Foun	High	Peak Foun
158.6	Not Found	Not Found	Peak Foun	Not Found	Not Found	Not Found	Not Found	Not Found
86.5	Peak Foun	High	Peak Foun	High	High	Peak Foun	High	High
97.2	High	Not Found	High	High	Peak Foun	Not Found	High	High
112.2	Peak Foun	High	Peak Foun	High	Peak Foun	Not Found	Not Found	Peak Foun
	High	Peak Foun	High	Peak Foun	Not Found	Not Found	Not Found	Not Found
99	High	Peak Foun	Not Found	Peak Foun	Peak Foun	Peak Foun	Peak Foun	Peak Foun
155.7	Peak Foun	High	High	High	Peak Foun	Peak Foun	High	Peak Foun
114.2	Peak Foun	Peak Foun	High	Peak Foun	Peak Foun	Peak Foun	Peak Foun	Peak Foun
78.1	High	Peak Foun	Not Found	Not Found	High	High	Peak Foun	High
98.3	Peak Foun	Peak Foun	Peak Foun	High	Peak Foun	Peak Foun	Peak Foun	Peak Foun
113.4	High	Peak Foun	Peak Foun	Peak Foun	Peak Foun	Peak Foun	Not Found	Peak Foun
109.9	Peak Foun	High	High	Peak Foun	Peak Foun	High	Peak Foun	High
116.3	Not Found	Peak Foun	Not Found	Peak Foun	Not Found	Not Found	Not Found	Not Found
136.4	Peak Foun	High	Peak Foun	Peak Foun	Peak Foun	Peak Foun	Peak Foun	Peak Foun
154.8	High	Not Found	Not Found	Not Found	Peak Foun	Peak Foun	Not Found	High
97.9	High	High	High	High	Peak Foun	High	High	Peak Foun
114.2	Peak Foun	Peak Foun	Peak Foun	Peak Foun	Peak Foun	Peak Foun	Peak Foun	Peak Foun
94.1	Peak Foun	High	High	High	Peak Foun	Peak Foun	High	Peak Foun
119	High	High	High	Peak Foun	Not Found	Peak Foun	Peak Foun	Peak Foun
108.1	High	Peak Foun	High	High	Peak Foun	Peak Foun	Peak Foun	High
111.7	Peak Foun	High	High	Peak Foun	Peak Foun	Peak Foun	Peak Foun	Peak Foun
114.8	Peak Foun	Peak Foun	Peak Foun	Peak Foun	Not Found	Not Found	Peak Foun	Peak Foun
136.9	Peak Foun	Peak Foun	High	Peak Foun	Peak Foun	Peak Foun	Peak Foun	Peak Foun
109.7	High	Peak Foun	High	High	Peak Foun	Peak Foun	High	High

127	High	Peak Foun	Peak Foun	Peak Foun	Peak Foun	Peak Foun	Peak Foun	Peak Foun
95.1	High	Peak Foun	High	Peak Foun	Not Found	Not Found	Not Found	Peak Foun
103.3	High	Peak Foun	Peak Foun	Not Found	Not Found	Not Found	Not Found	Peak Foun
137.1	Peak Foun	Peak Foun	Peak Foun	Not Found	Not Found	Peak Foun	Not Found	Not Found
105.6	High	Peak Foun	Peak Foun	Peak Foun	Peak Foun	Peak Foun	Peak Foun	Peak Foun
96.1	Peak Foun	Peak Foun	Not Found	Not Found	Not Found	Not Found	Not Found	Not Found
97	High	Not Found	Not Found	Peak Foun	Peak Foun	Not Found	High	Not Found
79.9	Peak Foun	Peak Foun	Peak Foun	Peak Foun	Peak Foun	High	Peak Foun	Not Found
183	Not Found	Peak Foun	Not Found	Not Found	Not Found	Not Found	Not Found	Not Found
104	Peak Foun	High	Peak Foun	Peak Foun	Peak Foun	Peak Foun	Not Found	Peak Foun
132.7	Peak Foun	High	High	Not Found	Not Found	Peak Foun	Peak Foun	Peak Foun
99.9	Peak Foun	Peak Foun	High	High	Peak Foun	Peak Foun	Peak Foun	Peak Foun
121.1	Not Found	Not Found	Peak Foun	Not Found	Not Found	Not Found	Not Found	Not Found
108.6	High	Peak Foun	High	Not Found	Not Found	Not Found	Not Found	Not Found
121.4	Peak Foun	High	Peak Foun	Peak Foun	Peak Foun	Peak Foun	Peak Foun	Peak Foun
108.1	Peak Foun	High	Peak Foun	Not Found	Peak Foun	Peak Foun	Peak Foun	Peak Foun
107.8	High	High	High	High	Peak Foun	Peak Foun	High	High
96.3	High	High	High	High	Not Found	High	Not Found	Peak Foun
107.9	High	High	High	Peak Foun	Peak Foun	Peak Foun	Peak Foun	Peak Foun
117.3	Peak Foun	Peak Foun	High	Not Found	Not Found	Not Found	Not Found	Not Found
109.4	High	Peak Foun	High	Peak Foun	Peak Foun	High	Peak Foun	Peak Foun
91.6	High	Peak Foun	Not Found	Peak Foun	Peak Foun	Peak Foun	Not Found	Not Found
108.7	High	High	Peak Foun	Peak Foun	Peak Foun	High	Peak Foun	Peak Foun
121.2	Peak Foun	High	Peak Foun	Peak Foun	Peak Foun	Peak Foun	Peak Foun	Peak Foun
101.6	Peak Foun	Peak Foun	Peak Foun	Peak Foun	High	High	High	Peak Foun
118.6	Peak Foun	Peak Foun	Peak Foun	Peak Foun	Peak Foun	Peak Foun	High	Peak Foun
121.8	Not Found	Not Found	Not Found	Not Found	Peak Foun	Not Found	Not Found	Peak Foun
104	High	Peak Foun	Peak Foun	Peak Foun	Not Found	Peak Foun	Peak Foun	Peak Foun
200	Not Found	Not Found	Not Found	Not Found	Not Found	Not Found	Not Found	Not Found
112.4	High	High	High	High	Peak Foun	Peak Foun	Peak Foun	High
117.2	High	High	Peak Foun	Peak Foun	Peak Foun	Peak Foun	Peak Foun	Peak Foun
110.1	Peak Foun	Peak Foun	High	Not Found	Not Found	Peak Foun	High	Peak Foun
124.9	High	Peak Foun	High	Peak Foun	Peak Foun	Peak Foun	Peak Foun	Peak Foun
139.4	Peak Foun	Peak Foun	Peak Foun	Not Found	Peak Foun	Peak Foun	Peak Foun	Peak Foun
120	Peak Foun	Peak Foun	High	Peak Foun	Peak Foun	Peak Foun	Peak Foun	Peak Foun
106.4	Peak Foun	High	Peak Foun	Peak Foun	Peak Foun	Peak Foun	High	Peak Foun
107.7	Not Found	High	Peak Foun	Not Found	Not Found	Not Found	Not Found	Not Found
98.8	High	Peak Foun	Peak Foun	Peak Foun	Peak Foun	High	Not Found	Not Found
130.6	Peak Foun	Peak Foun	Peak Foun	Peak Foun	Not Found	Peak Foun	Peak Foun	Not Found
116.2	Peak Foun	Peak Foun	High	Not Found	Not Found	Peak Foun	Peak Foun	Not Found
102.3	Peak Foun	Peak Foun	High	High	Peak Foun	High	High	Peak Foun
113.7	Peak Foun	Peak Foun	Peak Foun	Peak Foun	Peak Foun	Peak Foun	Peak Foun	Peak Foun
151	High	High	High	Peak Foun	Peak Foun	Not Found	Not Found	Not Found
107.8	Not Found	Peak Foun	Peak Foun	Peak Foun	Not Found	Not Found	Not Found	Not Found
195.9	Peak Foun	Peak Foun	Peak Foun	High	Peak Foun	High	Peak Foun	High

111.2	Not Found	High	Peak Foun	Peak Foun	Peak Foun	Peak Foun	Peak Foun	Peak Foun
98.8	High	High	Peak Foun	Peak Foun	Peak Foun	High	High	High
121.8	High	High	High	High	Peak Foun	Not Found	Peak Foun	Peak Foun
90.3	Peak Foun	Peak Foun	High	Peak Foun	Peak Foun	Peak Foun	High	High
129.5	Peak Foun	Peak Foun	Peak Foun	Peak Foun	Not Found	Not Found	Peak Foun	Peak Foun
117	High	High	Not Found	Not Found	High	High	High	Not Found
113	Not Found	Not Found	Peak Foun	Not Found	Peak Foun	Peak Foun	Peak Foun	Peak Foun
119.6	High	High	High	High	High	High	Peak Foun	High
123.1	High	High	Peak Foun	Peak Foun	Peak Foun	Not Found	Peak Foun	Peak Foun
111.3	High	Peak Foun	High	Peak Foun	Peak Foun	Peak Foun	Peak Foun	Peak Foun
94.9	Peak Foun	Peak Foun	Peak Foun	Peak Foun	Peak Foun	Peak Foun	Peak Foun	Not Found
98.5	Peak Foun	Peak Foun	Not Found	High	Peak Foun	Peak Foun	Not Found	Not Found
116.2	High	High	Peak Foun	Peak Foun	Peak Foun	High	Peak Foun	Peak Foun
112.2	Peak Foun	Peak Foun	Peak Foun	Peak Foun	Not Found	Not Found	Peak Foun	Peak Foun
137.4	High	High	Peak Foun	Not Found	Not Found	Peak Foun	Peak Foun	Peak Foun
81.7	Peak Foun	Peak Foun	High	Peak Foun	Peak Foun	Not Found	Not Found	Not Found
84.9	Peak Foun	Peak Foun	High	High	Peak Foun	Peak Foun	High	Peak Foun
74.7	Peak Foun	Peak Foun	Peak Foun	Peak Foun	Peak Foun	High	Peak Foun	Not Found
122.3	Peak Foun	Not Found	Peak Foun	Peak Foun	Not Found	Not Found	Peak Foun	Peak Foun
147.4	Not Found	High	High	Not Found	Not Found	Peak Foun	Peak Foun	Not Found
91.6	Peak Foun	High	Peak Foun	Not Found	Not Found	High	Peak Foun	Not Found
114.9	Peak Foun	Peak Foun	Peak Foun	Peak Foun	Peak Foun	Peak Foun	Peak Foun	Peak Foun
118.5	Peak Foun	Peak Foun	Peak Foun	Peak Foun	Peak Foun	Peak Foun	High	Peak Foun
94.1	Not Found	Not Found	Not Found	Not Found	Not Found	High	Not Found	Not Found
97.3	Peak Foun	High	Peak Foun	Not Found	Not Found	Not Found	Not Found	Not Found
104.1	Peak Foun	Peak Foun	Peak Foun	Peak Foun	Peak Foun	Peak Foun	High	Peak Foun
114.4	High	High	High	High	Peak Foun	Peak Foun	High	Peak Foun
119	Peak Foun	High	Peak Foun	Peak Foun	Peak Foun	Not Found	Not Found	Not Found
121.9	Peak Foun	Peak Foun	Peak Foun	Peak Foun	Peak Foun	Peak Foun	Peak Foun	Peak Foun
116.6	Peak Foun	Peak Foun	High	Not Found	Not Found	High	Peak Foun	Not Found
90.6	Not Found	Peak Foun	Peak Foun	Not Found	Not Found	Peak Foun	High	Peak Foun
107.1	Peak Foun	Peak Foun	Not Found	Not Found	Not Found	Not Found	Not Found	Not Found
107.8	High	High	High	Peak Foun	Not Found	Peak Foun	Peak Foun	Peak Foun
109	High	High	Peak Foun	Peak Foun	Peak Foun	Peak Foun	High	Peak Foun
101.9	High	Peak Foun	High	High	Not Found	Not Found	Not Found	Not Found
116.3	High	Not Found	Peak Foun	High	Peak Foun	Peak Foun	Peak Foun	Peak Foun
100.9	High	High	Peak Foun	High	High	High	Peak Foun	Peak Foun
101.4	Not Found	Not Found	Peak Foun	Not Found	Not Found	Not Found	Not Found	Peak Foun
102.8	High	High	High	High	Peak Foun	Not Found	Not Found	Peak Foun
107.1	High	High	High	High	Peak Foun	Peak Foun	Peak Foun	Peak Foun
111.6	High	Peak Foun	High	High	Not Found	Not Found	Not Found	High
91.5	High	High	High	Peak Foun	High	High	High	High
106.8	High	High	High	High	High	Peak Foun	High	High
108	Peak Foun	High	Peak Foun	High	Peak Foun	Peak Foun	Peak Foun	Peak Foun
99.7	Peak Foun	High	High	Peak Foun	Peak Foun	Peak Foun	Peak Foun	Peak Foun

173.7	Not Found	Peak Foun	Not Found	Peak Foun	Peak Foun	Peak Foun	Not Found	Not Found
98.7	Peak Foun	High	Peak Foun	High	Not Found	Peak Foun	Not Found	Peak Foun
116.1	Peak Foun	High	High	Not Found	Not Found	Peak Foun	Not Found	Peak Foun
116.5	Peak Foun	Peak Foun	Peak Foun	Peak Foun	Peak Foun	Peak Foun	Peak Foun	Peak Foun
99.9	High	Peak Foun	Peak Foun	Peak Foun	Peak Foun	Peak Foun	Peak Foun	Peak Foun
100.6	High	High	High	Peak Foun	Not Found	Not Found	Not Found	Not Found
93.4	Peak Foun	High	Peak Foun	Peak Foun	Peak Foun	High	High	Peak Foun
70.4	Not Found	High	Not Found	Not Found	Not Found	Not Found	Not Found	Not Found
84.7	Peak Foun	Peak Foun	Peak Foun	Peak Foun	Peak Foun	Peak Foun	Peak Foun	Peak Foun
100.6	Not Found	Not Found	Peak Foun	Peak Foun	Not Found	Peak Foun	Peak Foun	Peak Foun
121.3	Peak Foun	Peak Foun	Peak Foun	Not Found	Peak Foun	Not Found	Peak Foun	Peak Foun
111.7	Peak Foun	Peak Foun	High	Peak Foun	Peak Foun	Not Found	High	Peak Foun
114.8	High	Peak Foun	High	Peak Foun	Peak Foun	Peak Foun	Peak Foun	High
138.9	High	Not Found	Not Found	Not Found	Not Found	Not Found	Not Found	Not Found
88.3	Peak Foun	Peak Foun	High	Not Found	Not Found	Not Found	Peak Foun	Not Found
108.7	Peak Foun	Peak Foun	Peak Foun	Not Found	Peak Foun	High	High	Not Found
104.7	Peak Foun	Peak Foun	Peak Foun	Not Found	Peak Foun	High	High	Peak Foun
102.1	Not Found	Not Found	High	Peak Foun	Not Found	Peak Foun	Peak Foun	Peak Foun
97.8	High	High	High	High	High	High	High	High
135.6	Peak Foun	Peak Foun	High	Not Found	Not Found	Peak Foun	Peak Foun	Not Found
102.7	High	High	High	High	High	High	High	High
119.5	High	Peak Foun	Peak Foun	Peak Foun	Not Found	Not Found	Not Found	Not Found
119	Peak Foun	Peak Foun	Peak Foun	Peak Foun	Peak Foun	Peak Foun	High	Peak Foun
91.4	Peak Foun	Peak Foun	High	Not Found	Peak Foun	Not Found	High	Peak Foun
86.9	Peak Foun	Not Found	Peak Foun	Not Found	Not Found	Not Found	Not Found	Not Found
102.5	Peak Foun	Peak Foun	Peak Foun	High	Peak Foun	Not Found	Not Found	Not Found
97.6	Peak Foun	Peak Foun	Peak Foun	Not Found	High	High	High	Peak Foun
105.9	Peak Foun	High	Peak Foun	Peak Foun	Not Found	Peak Foun	Peak Foun	Not Found
96.2	High	Peak Foun	Peak Foun	Peak Foun	Peak Foun	High	Peak Foun	Peak Foun
95.8	Not Found	Peak Foun	Not Found	Peak Foun	Peak Foun	Peak Foun	Not Found	Peak Foun
110.1	High	Peak Foun	Peak Foun	Peak Foun	Not Found	Peak Foun	Peak Foun	Peak Foun
102.9	Peak Foun	Peak Foun	High	Peak Foun	Peak Foun	High	High	Peak Foun
103.8	Not Found	High	Peak Foun	Not Found	High	Peak Foun	Peak Foun	Peak Foun
110.9	Peak Foun	High	High	Not Found	Not Found	Peak Foun	High	Peak Foun
110.8	High	Peak Foun	Peak Foun	Not Found	Peak Foun	Peak Foun	Not Found	Not Found
99.9	High	Peak Foun	Peak Foun	Peak Foun	Peak Foun	Not Found	Not Found	Peak Foun
111.9	Peak Foun	Peak Foun	Peak Foun	Peak Foun	High	High	Peak Foun	Peak Foun
122.9	Peak Foun	High	Peak Foun	Peak Foun	Not Found	Peak Foun	Peak Foun	Peak Foun
115.9	High	High	High	Peak Foun	Peak Foun	Peak Foun	Peak Foun	Peak Foun
114.8	Peak Foun	Peak Foun	High	Peak Foun	High	High	High	Peak Foun
106.8	Peak Foun	Peak Foun	High	Peak Foun	Not Found	Not Found	Peak Foun	Peak Foun
86.5	High	High	High	High	Not Found	Not Found	Not Found	Peak Foun
113.8	Peak Foun	High	Peak Foun	Peak Foun	Peak Foun	Peak Foun	Peak Foun	Peak Foun
106.6	High	High	High	Peak Foun	High	Peak Foun	Peak Foun	Peak Foun
	High	High	Peak Foun	Not Found	Not Found	Not Found	Not Found	Not Found

167.6	Not Found	Peak Foun	Not Found	Not Found	Not Found	Not Found	Not Found	Peak Foun
106	High	Peak Foun	High	Peak Foun	Peak Foun	Peak Foun	Peak Foun	Peak Foun
85.8	Peak Foun	Peak Foun	High	Peak Foun	Not Found	Not Found	Peak Foun	Peak Foun
104.1	High	Peak Foun	Peak Foun	Peak Foun	Peak Foun	Peak Foun	Peak Foun	Not Found
118.8	Peak Foun	Peak Foun	Peak Foun	Not Found	Not Found	Not Found	Peak Foun	Not Found
117.3	Peak Foun	High	Not Found	Not Found	Not Found	Not Found	Not Found	Peak Foun
118.1	Peak Foun	High	High	High	Not Found	Peak Foun	High	Peak Foun
126.6	Peak Foun	Peak Foun	Peak Foun	Not Found	Peak Foun	Peak Foun	Peak Foun	Peak Foun
107.6	Peak Foun	Peak Foun	Peak Foun	Peak Foun	Peak Foun	High	High	Peak Foun
101.6	High	Peak Foun	High	Peak Foun	High	High	High	High
94	High	High	Peak Foun	Peak Foun	High	High	Peak Foun	Peak Foun
85.2	Peak Foun	Peak Foun	Peak Foun	Peak Foun	High	High	High	High
156	High	Peak Foun	Not Found	Not Found	Not Found	Not Found	Not Found	Peak Foun
8.6	Not Found	Peak Foun	Not Found	Not Found	Peak Foun	High	High	Not Found
101.6	High	Peak Foun	High	Peak Foun	Peak Foun	Peak Foun	Peak Foun	Peak Foun
82.9	High	High	Peak Foun	Peak Foun	Peak Foun	Peak Foun	Peak Foun	Peak Foun
105.6	High	High	High	High	Peak Foun	Peak Foun	High	Peak Foun
110	Peak Foun	High	High	High	High	Peak Foun	High	High
109	High	High	Peak Foun	High	Peak Foun	Peak Foun	Peak Foun	Peak Foun
	Peak Foun	High	High	Peak Foun	Not Found	Not Found	Not Found	Not Found
114.2	Peak Foun	High	Peak Foun	Peak Foun	Peak Foun	Peak Foun	Not Found	Not Found
106.7	High	High	High	Peak Foun	High	High	High	High
100.3	Peak Foun	Peak Foun	Peak Foun	Peak Foun	Peak Foun	Peak Foun	Peak Foun	High
99.1	Not Found	Not Found	High	Not Found	Not Found	Not Found	Not Found	Not Found
116.2	Peak Foun	High	Peak Foun	Peak Foun	Not Found	Peak Foun	Not Found	Not Found
111.2	High	High	High	Peak Foun	Peak Foun	Peak Foun	Peak Foun	High
94.8	High	Peak Foun	High	High	Peak Foun	Peak Foun	Peak Foun	Peak Foun
128.2	Peak Foun	Peak Foun	Peak Foun	Peak Foun	Peak Foun	Peak Foun	Peak Foun	Peak Foun
93.4	High	High	Peak Foun	Peak Foun	High	Not Found	High	Peak Foun
112.1	Not Found	Not Found	Peak Foun	Peak Foun	Peak Foun	Not Found	Not Found	Peak Foun
116.8	Peak Foun	Peak Foun	Peak Foun	Not Found	High	Peak Foun	Peak Foun	Not Found
80.9	High	High	Not Found	Peak Foun	Peak Foun	Peak Foun	Peak Foun	Peak Foun
106	Peak Foun	High	Peak Foun	Peak Foun	Peak Foun	Peak Foun	Peak Foun	Peak Foun
103.6	Peak Foun	Peak Foun	Peak Foun	Peak Foun	Not Found	Peak Foun	Peak Foun	High
118	Not Found	Peak Foun	Not Found	Not Found	Not Found	Peak Foun	High	High
103.5	Peak Foun	Peak Foun	Peak Foun	Peak Foun	High	Peak Foun	Peak Foun	Peak Foun
118.6	Not Found	Peak Foun	High	High	Peak Foun	Peak Foun	Peak Foun	Peak Foun
114.2	Peak Foun	Peak Foun	Peak Foun	Peak Foun	Not Found	Peak Foun	Peak Foun	Peak Foun
123	Peak Foun	Peak Foun	High	Peak Foun	Not Found	Peak Foun	High	Not Found
107.1	High	High	Peak Foun	Peak Foun	Peak Foun	Not Found	Not Found	Peak Foun
76.3	High	High	High	High	High	Peak Foun	Peak Foun	Not Found
89.9	High	Peak Foun	Not Found	Not Found	Not Found	Not Found	Not Found	Not Found
104.5	Peak Foun	Peak Foun	High	Peak Foun	Peak Foun	Peak Foun	Peak Foun	Peak Foun
115.3	High	High	Peak Foun	Peak Foun	Not Found	Peak Foun	Not Found	Peak Foun
113.6	Peak Foun	Peak Foun	Peak Foun	Peak Foun	High	Peak Foun	Peak Foun	High

100.1	Peak Foun	High	Peak Foun	Peak Foun	Not Found	Peak Foun	Peak Foun	Peak Foun
149.7	Peak Foun	Peak Foun	Peak Foun	Not Found	Peak Foun	Not Found	Not Found	Not Found
121	High	Peak Foun	Peak Foun	Peak Foun	Peak Foun	High	Peak Foun	Peak Foun
111.4	Peak Foun	High	Peak Foun	Not Found	Not Found	Not Found	Not Found	Peak Foun
136.4	Peak Foun	High	Peak Foun	Peak Foun	Not Found	Peak Foun	Peak Foun	Peak Foun
137	Peak Foun	High	Peak Foun	Peak Foun	Not Found	Not Found	Peak Foun	Peak Foun
94.4	Peak Foun	High	High	Peak Foun	Peak Foun	Peak Foun	High	Peak Foun
111.1	Not Found	Peak Foun	Peak Foun	Peak Foun	Not Found	Peak Foun	Not Found	Peak Foun
	High	High	Peak Foun	Peak Foun	Not Found	Not Found	Not Found	Not Found
105.9	High	Peak Foun	Peak Foun	Peak Foun	Peak Foun	Peak Foun	Peak Foun	Peak Foun
137.7	Peak Foun	Peak Foun	Peak Foun	Peak Foun	Peak Foun	Peak Foun	Peak Foun	Peak Foun
140.2	High	Peak Foun	Not Found	Peak Foun	Peak Foun	Not Found	Not Found	Not Found
53.7	High	Peak Foun	High	Peak Foun	Not Found	Not Found	Not Found	Not Found
107	High	High	High	High	Peak Foun	High	Peak Foun	Peak Foun
	Peak Foun	High	Peak Foun	Not Found	Not Found	Not Found	Not Found	Not Found
200	Not Found	Not Found	Not Found	Not Found	Not Found	Not Found	Not Found	Not Found
127.9	High	Peak Foun	High	Peak Foun	Peak Foun	Peak Foun	Peak Foun	Peak Foun
112.9	High	High	Peak Foun	Peak Foun	Peak Foun	Peak Foun	Not Found	Not Found
87	Peak Foun	Peak Foun	Not Found	Peak Foun	Peak Foun	Peak Foun	Peak Foun	Peak Foun
105.2	Not Found	Not Found	Not Found	Not Found	Not Found	Not Found	Not Found	Not Found
113.9	Peak Foun	Peak Foun	Not Found	Peak Foun	Peak Foun	Peak Foun	Peak Foun	Peak Foun
111.2	Peak Foun	Peak Foun	Peak Foun	Peak Foun	Peak Foun	Not Found	Peak Foun	Not Found
99.5	Not Found	Peak Foun	Peak Foun	Peak Foun	High	High	Peak Foun	Peak Foun
160.7	Not Found	Not Found	Not Found	Not Found	Not Found	Not Found	Not Found	Not Found
101.9	High	High	Peak Foun	Peak Foun	Peak Foun	Peak Foun	Peak Foun	Peak Foun
	Not Found	Not Found	Not Found	Not Found	Peak Foun	Peak Foun	Not Found	Not Found
139	High	High	Peak Foun	Peak Foun	Peak Foun	Peak Foun	Peak Foun	Peak Foun
105.9	Peak Foun	High	High	High	High	Peak Foun	High	High
153.1	High	High	Peak Foun	High	Peak Foun	Peak Foun	Peak Foun	High
126.7	Peak Foun	Peak Foun	Peak Foun	Peak Foun	High	Peak Foun	Peak Foun	Peak Foun
101.7	High	High	High	Not Found	Peak Foun	High	Peak Foun	Peak Foun
109.4	Peak Foun	High	High	Peak Foun	Not Found	Peak Foun	Peak Foun	Not Found
121.2	High	High	High	Not Found	Peak Foun	Peak Foun	Peak Foun	Not Found
120.8	Not Found	High	Peak Foun	Peak Foun	Not Found	Peak Foun	Peak Foun	Peak Foun
	High	Not Found	Not Found	Not Found	Not Found	Peak Foun	Not Found	Peak Foun
62.7	Peak Foun	Peak Foun	Peak Foun	Not Found	Peak Foun	High	Peak Foun	Peak Foun
120.5	High	Peak Foun	Peak Foun	Peak Foun	Peak Foun	Not Found	Peak Foun	Peak Foun
116	High	High	Peak Foun	Peak Foun	Peak Foun	High	High	High
136.9	Not Found	Not Found	Peak Foun	Not Found	Peak Foun	Peak Foun	Peak Foun	High
91.3	High	Peak Foun	Peak Foun	High	Peak Foun	Peak Foun	Peak Foun	High
75.2	Peak Foun	Peak Foun	Peak Foun	High	Peak Foun	Peak Foun	Peak Foun	High
123.5	High	High	High	Peak Foun	Peak Foun	Peak Foun	Peak Foun	High
109	Peak Foun	High	Peak Foun	Peak Foun	Not Found	Peak Foun	Peak Foun	Peak Foun
107.4	Peak Foun	Peak Foun	Peak Foun	Not Found	Not Found	Peak Foun	Peak Foun	Not Found
143.7	Peak Foun	Peak Foun	Peak Foun	Not Found	Peak Foun	Peak Foun	Not Found	Peak Foun

101.8	Peak Foun	Peak Foun	High	Peak Foun	Not Found	Peak Foun	Peak Foun	Peak Foun
107.6	Peak Foun	High	Peak Foun	Peak Foun	Peak Foun	Peak Foun	Peak Foun	Peak Foun
110.1	Peak Foun	High	Peak Foun	Peak Foun	Not Found	Not Found	Peak Foun	Not Found
113.6	High	High	High	Peak Foun	Peak Foun	High	Peak Foun	Peak Foun
111.3	Peak Foun	High	Peak Foun	Peak Foun	Not Found	Not Found	Not Found	Peak Foun
106.4	Peak Foun	Peak Foun	Peak Foun	Peak Foun	Peak Foun	Peak Foun	Peak Foun	Peak Foun
121.2	Peak Foun	Peak Foun	High	Peak Foun	Not Found	Peak Foun	Peak Foun	Peak Foun
97	Peak Foun	Peak Foun	Not Found	Not Found	Not Found	Not Found	Not Found	High
116.7	Peak Foun	Peak Foun	Peak Foun	Peak Foun	Peak Foun	Peak Foun	Peak Foun	Peak Foun
132.5	Peak Foun	Peak Foun	Peak Foun	Not Found	Peak Foun	Peak Foun	Peak Foun	Peak Foun
	Peak Foun	Peak Foun	High	Peak Foun	Not Found	Peak Foun	Not Found	Peak Foun
111.5	High	High	High	High	High	High	High	High
107.5	Peak Foun	Peak Foun	Peak Foun	Peak Foun	Peak Foun	High	Peak Foun	Peak Foun
135.5	Peak Foun	Not Found	Not Found	Not Found	Not Found	Not Found	Peak Foun	Peak Foun
116	Peak Foun	Peak Foun	Peak Foun	Peak Foun	Not Found	Not Found	Not Found	Peak Foun
110.9	High	Not Found	Peak Foun	Peak Foun	High	Peak Foun	Peak Foun	Not Found
108.6	Peak Foun	Peak Foun	Peak Foun	Peak Foun	Not Found	High	High	Peak Foun
141.8	Peak Foun	Peak Foun	Peak Foun	Not Found	Not Found	Peak Foun	Peak Foun	Peak Foun
78.1	Peak Foun	High	Peak Foun	High	Peak Foun	High	Peak Foun	High
112.7	High	High	High	High	Peak Foun	High	High	High
114	High	High	High	Peak Foun	Not Found	Not Found	Peak Foun	Peak Foun
125.6	High	High	Peak Foun	Peak Foun	Peak Foun	Peak Foun	Peak Foun	Peak Foun
126.4	Peak Foun	High	Peak Foun	Not Found	Peak Foun	Peak Foun	High	Not Found
92.9	High	Peak Foun	Peak Foun	Peak Foun	Not Found	Peak Foun	Peak Foun	Peak Foun
126.6	High	High	Peak Foun	High	Peak Foun	Peak Foun	Peak Foun	Peak Foun
85.3	High	High	High	Not Found	Peak Foun	Peak Foun	Peak Foun	Peak Foun
113.4	Peak Foun	Not Found	High	Not Found	Peak Foun	Not Found	Peak Foun	Not Found
143.3	Peak Foun	Peak Foun	Peak Foun	Peak Foun	Not Found	Not Found	Peak Foun	Peak Foun
98.8	High	High	Peak Foun	High	High	High	High	High
101.6	High	Peak Foun	Peak Foun	Peak Foun	Not Found	Not Found	Not Found	Peak Foun
97.4	High	High	High	Peak Foun	Not Found	Not Found	Peak Foun	Not Found
136.2	Peak Foun	Peak Foun	High	Not Found	Peak Foun	Peak Foun	Peak Foun	Peak Foun
101.8	Peak Foun	Peak Foun	Peak Foun	Peak Foun	Peak Foun	High	Peak Foun	Peak Foun
96.1	High	High	Peak Foun	Peak Foun	Not Found	Not Found	Not Found	Not Found
110	Peak Foun	High	Not Found	Peak Foun	Not Found	Peak Foun	Peak Foun	Peak Foun
105.5	High	High	High	High	Not Found	Peak Foun	Peak Foun	Peak Foun
94.4	Peak Foun	High	High	Peak Foun	Not Found	Not Found	Not Found	Not Found
97.5	High	High	Peak Foun	Peak Foun	Peak Foun	Peak Foun	Peak Foun	Peak Foun
54.7	Not Found	Not Found	Not Found	Not Found	Not Found	High	High	Not Found
101.9	Peak Foun	Peak Foun	High	Peak Foun	Not Found	Peak Foun	Not Found	Not Found
106.7	Peak Foun	Peak Foun	Peak Foun	Not Found	Peak Foun	Peak Foun	High	Peak Foun
112.2	Peak Foun	Peak Foun	Peak Foun	Peak Foun	Peak Foun	Not Found	Peak Foun	Peak Foun
114	High	High	Not Found	Not Found	Not Found	Not Found	Not Found	Not Found
91.6	Peak Foun	Peak Foun	High	Not Found	Not Found	Peak Foun	Not Found	Not Found
84.4	Peak Foun	High	Peak Foun	Peak Foun	Not Found	Peak Foun	Peak Foun	Not Found

108.1	High	High	High	High	Peak Foun	Peak Foun	Peak Foun	Peak Foun
104.9	Peak Foun	Peak Foun	Not Found	Peak Foun	High	High	Peak Foun	Peak Foun
93.2	Peak Foun	Peak Foun	Peak Foun	Peak Foun	High	Peak Foun	Peak Foun	Peak Foun
	High	High	Peak Foun	Peak Foun	Not Found	Not Found	Peak Foun	Not Found
134.3	Peak Foun	Peak Foun	Peak Foun	Peak Foun	Peak Foun	Peak Foun	Peak Foun	Peak Foun
	Not Found	Not Found	Not Found	Not Found	High	Peak Foun	High	Not Found
126.2	Peak Foun	High	Peak Foun	Peak Foun	Not Found	Peak Foun	High	Not Found
109.5	Peak Foun	Peak Foun	Peak Foun	Peak Foun	Not Found	Peak Foun	Peak Foun	Peak Foun
85.6	Peak Foun	High	Peak Foun	Peak Foun	Peak Foun	Peak Foun	Peak Foun	Peak Foun
112.5	Peak Foun	Peak Foun	Peak Foun	Peak Foun	Not Found	Not Found	Not Found	High
110.3	Peak Foun	Peak Foun	Peak Foun	Peak Foun	Not Found	Peak Foun	Peak Foun	Peak Foun
86.5	Peak Foun	Peak Foun	Peak Foun	Peak Foun	Peak Foun	High	Peak Foun	Not Found
118.6	Not Found	Peak Foun	Not Found	Not Found	Peak Foun	Peak Foun	Peak Foun	Peak Foun
90	Peak Foun	Peak Foun	High	Peak Foun	Peak Foun	High	Peak Foun	Peak Foun
113.9	Peak Foun	Peak Foun	Peak Foun	Peak Foun	Not Found	Not Found	Not Found	Peak Foun
130.3	High	Peak Foun	Peak Foun	Peak Foun	Not Found	Peak Foun	Peak Foun	Peak Foun
98.2	Peak Foun	Peak Foun	Peak Foun	Peak Foun	Not Found	Peak Foun	Peak Foun	Peak Foun
124.6	High	High	High	Peak Foun	Not Found	Not Found	Not Found	Peak Foun
99	Peak Foun	High	High	High	Not Found	Peak Foun	Not Found	Peak Foun
103.5	High	High	Peak Foun	Not Found	Peak Foun	Not Found	Not Found	High
100.9	Not Found	Not Found	Peak Foun	Not Found	Not Found	Not Found	Not Found	Not Found
106	High	High	High	Peak Foun	Peak Foun	Peak Foun	High	Peak Foun
89.9	High	High	Peak Foun	Peak Foun	Peak Foun	High	Peak Foun	Peak Foun
114.1	High	High	High	High	Peak Foun	High	Peak Foun	Peak Foun
106.3	High	Peak Foun	Peak Foun	Peak Foun	Peak Foun	Peak Foun	Peak Foun	Peak Foun
107.2	High	Peak Foun	Peak Foun	High	Peak Foun	Not Found	Not Found	Peak Foun
93.2	High	Not Found	High	Peak Foun	Not Found	Not Found	Not Found	Peak Foun
115	Peak Foun	High	Not Found	Not Found	Not Found	Peak Foun	Peak Foun	Peak Foun
81.6	Not Found	High	Peak Foun	Peak Foun	Peak Foun	Peak Foun	High	Not Found
94.8	Peak Foun	Peak Foun	Peak Foun	Peak Foun	Peak Foun	High	Peak Foun	Peak Foun
106.6	High	High	High	Peak Foun	Peak Foun	Peak Foun	Peak Foun	Peak Foun
134.9	Not Found	Not Found	Not Found	Peak Foun	Peak Foun	Peak Foun	Peak Foun	Peak Foun
107.2	Peak Foun	Peak Foun	High	Peak Foun	Peak Foun	Peak Foun	Peak Foun	Peak Foun
100.9	Peak Foun	High	Peak Foun	Peak Foun	Not Found	Not Found	Not Found	Not Found
112	High	High	Peak Foun	High	Peak Foun	Peak Foun	High	High
136.8	High	Peak Foun	High	Peak Foun	Peak Foun	Peak Foun	Peak Foun	Peak Foun
123.4	High	High	High	High	High	High	High	Peak Foun
99	Peak Foun	Peak Foun	Peak Foun	Peak Foun	Not Found	Not Found	Not Found	Not Found
124.6	High	High	Peak Foun	Peak Foun	Peak Foun	Peak Foun	High	High
106.9	Peak Foun	High	Peak Foun	Peak Foun	Peak Foun	Peak Foun	Not Found	Peak Foun
105.1	Peak Foun	High	Peak Foun	Peak Foun	Not Found	Not Found	Peak Foun	Peak Foun
132.7	Peak Foun	High	High	Peak Foun	Peak Foun	Peak Foun	Peak Foun	Peak Foun
121.6	Not Found	Not Found	Not Found	Not Found	Not Found	Not Found	Not Found	Not Found
146.7	High	High	High	High	Peak Foun	Peak Foun	Peak Foun	Peak Foun
82.1	Peak Foun	High	Peak Foun	Peak Foun	High	High	High	Peak Foun

115.7	Peak Foun	High	Not Found	Peak Foun	Not Found	Not Found	Not Found	Peak Foun
97	High	High	High	Peak Foun	Peak Foun	High	Peak Foun	Peak Foun
89.1	Peak Foun	Peak Foun	Peak Foun	High	Peak Foun	Peak Foun	High	Peak Foun
108.3	Peak Foun	Peak Foun	Peak Foun	Peak Foun	Not Found	Peak Foun	Not Found	Peak Foun
119.5	High	High	Peak Foun	Peak Foun	Peak Foun	Peak Foun	High	Peak Foun
146.8	Peak Foun	Peak Foun	High	Peak Foun	Not Found	Not Found	Not Found	Not Found
116	Peak Foun	Peak Foun	Peak Foun	Peak Foun	Peak Foun	Peak Foun	Peak Foun	Peak Foun
104.5	Peak Foun	High	Peak Foun	Peak Foun	Not Found	Not Found	Not Found	Peak Foun
104.9	Peak Foun	Peak Foun	Peak Foun	Peak Foun	Peak Foun	Peak Foun	Peak Foun	High
95.8	High	Peak Foun	High	Peak Foun	Peak Foun	Peak Foun	Peak Foun	Peak Foun
97.4	Peak Foun	High	Peak Foun	Peak Foun	Peak Foun	High	High	Peak Foun
124	Peak Foun	Peak Foun	Peak Foun	Not Found	Not Found	Not Found	Not Found	Not Found
104.7	Peak Foun	Peak Foun	Peak Foun	Peak Foun	Not Found	Not Found	Not Found	Peak Foun
120.7	Peak Foun	High	Peak Foun	Peak Foun	Peak Foun	Peak Foun	Peak Foun	Peak Foun
111.3	Peak Foun	Peak Foun	Peak Foun	Peak Foun	Not Found	Not Found	Peak Foun	Peak Foun
81.7	Not Found	Peak Foun	Peak Foun	Peak Foun	Not Found	Peak Foun	Peak Foun	Peak Foun
156.2	High	High	Peak Foun	Not Found	Peak Foun	High	Peak Foun	Not Found
108.1	Peak Foun	High	High	Peak Foun	Peak Foun	Peak Foun	Peak Foun	Peak Foun
65.1	Peak Foun	Peak Foun	High	Peak Foun	Not Found	Not Found	Peak Foun	Peak Foun
108.2	Peak Foun	High	Peak Foun	Peak Foun	Peak Foun	Peak Foun	Peak Foun	Peak Foun
	Not Found	Not Found	Not Found	Not Found	Not Found	Not Found	Not Found	Not Found
118.5	Peak Foun	Peak Foun	Peak Foun	Peak Foun	Peak Foun	Peak Foun	Peak Foun	Peak Foun
	Peak Foun	Peak Foun	Peak Foun	High	Peak Foun	High	High	Peak Foun
107.5	Peak Foun	Not Found	Not Found	Not Found	Not Found	Not Found	Not Found	Not Found
124.1	Peak Foun	Peak Foun	Not Found	Peak Foun	Peak Foun	Peak Foun	Peak Foun	Peak Foun
107.1	High	High	Not Found	Peak Foun	Peak Foun	Peak Foun	Peak Foun	Peak Foun
67.3	High	Peak Foun	Peak Foun	Not Found	High	Peak Foun	Peak Foun	Not Found
100.5	Peak Foun	Peak Foun	Peak Foun	Peak Foun	High	Peak Foun	Not Found	Peak Foun
97.3	Peak Foun	High	Not Found	Peak Foun	Peak Foun	Peak Foun	Not Found	Peak Foun
	Not Found	Not Found	Peak Foun	Not Found	Not Found	Not Found	Not Found	Not Found
22.5	Peak Foun	High	Peak Foun	Peak Foun	Peak Foun	Peak Foun	High	Peak Foun
103.5	Peak Foun	Peak Foun	Peak Foun	Peak Foun	Not Found	Peak Foun	Peak Foun	Peak Foun
137.2	Peak Foun	Peak Foun	High	Peak Foun	Not Found	Not Found	Peak Foun	Peak Foun
97.2	Peak Foun	Peak Foun	High	Peak Foun	Peak Foun	Peak Foun	Peak Foun	Peak Foun
125.1	Peak Foun	High	Not Found	Not Found	Not Found	Peak Foun	Peak Foun	Not Found
98.8	Peak Foun	Peak Foun	Peak Foun	Peak Foun	Peak Foun	Peak Foun	Peak Foun	Peak Foun
92.5	Not Found	Peak Foun	Peak Foun	Peak Foun	Not Found	Peak Foun	Peak Foun	Peak Foun
123.5	Peak Foun	Peak Foun	Peak Foun	Peak Foun	Peak Foun	Peak Foun	Peak Foun	Peak Foun
94.3	High	High	Peak Foun	Peak Foun	Peak Foun	High	Peak Foun	Peak Foun
125.9	High	High	High	Peak Foun	Peak Foun	Peak Foun	Peak Foun	Peak Foun
112.1	High	High	High	High	Peak Foun	High	High	High
103.3	High	High	High	High	Peak Foun	Not Found	Not Found	Peak Foun
139	Peak Foun	High	Peak Foun	Not Found	Peak Foun	Not Found	Peak Foun	Peak Foun
88.8	High	Peak Foun	High	Peak Foun	Peak Foun	Peak Foun	Peak Foun	Peak Foun
200	Not Found	Not Found	Not Found	Not Found	Not Found	Not Found	Not Found	Not Found

103.7	High	Peak Foun	Peak Foun	Peak Foun	Peak Foun	Peak Foun	Peak Foun	Peak Foun
108.8	Peak Foun	Peak Foun	Peak Foun	Peak Foun	Not Found	Peak Foun	Peak Foun	Peak Foun
	Peak Foun	High	Peak Foun	Peak Foun	Not Found	Not Found	Peak Foun	Not Found
115.4	Peak Foun	High	Peak Foun	Not Found	Peak Foun	Peak Foun	Peak Foun	Peak Foun
87.7	Peak Foun	High	Peak Foun	Peak Foun	High	High	Not Found	Peak Foun
108.3	Peak Foun	Peak Foun	Peak Foun	High	Peak Foun	Peak Foun	Peak Foun	Peak Foun
103.2	Peak Foun	Peak Foun	High	Peak Foun	Not Found	Peak Foun	Peak Foun	Peak Foun
101.5	Peak Foun	Peak Foun	High	Peak Foun	Not Found	Not Found	Not Found	Not Found
121	Peak Foun	High	High	Peak Foun	Peak Foun	Peak Foun	High	High
104.4	Peak Foun	High	High	Peak Foun	Not Found	Not Found	Peak Foun	High
100.8	Peak Foun	High	High	Peak Foun	Not Found	High	Peak Foun	Not Found
123.6	Peak Foun	Peak Foun	Peak Foun	Peak Foun	Not Found	Peak Foun	Peak Foun	Peak Foun
118.3	Peak Foun	High	High	Peak Foun	Peak Foun	Peak Foun	Peak Foun	High
104.5	Peak Foun	Peak Foun	High	Peak Foun	Peak Foun	Peak Foun	High	Peak Foun
125.5	Peak Foun	High	High	High	Not Found	Peak Foun	Peak Foun	Not Found
108	High	High	Peak Foun	Peak Foun	Peak Foun	Peak Foun	Peak Foun	Peak Foun
102.5	Peak Foun	Peak Foun	Peak Foun	Peak Foun	Peak Foun	Peak Foun	Not Found	Peak Foun
104.3	Peak Foun	Peak Foun	High	Peak Foun	Peak Foun	Peak Foun	Peak Foun	Peak Foun
86.6	High	High	Peak Foun	Peak Foun	Peak Foun	Peak Foun	Peak Foun	Peak Foun
110.4	Peak Foun	High	Peak Foun	Peak Foun	High	Peak Foun	Peak Foun	Peak Foun
109.9	High	Peak Foun	Peak Foun	Peak Foun	Peak Foun	Not Found	Not Found	Peak Foun
90.8	High	High	Peak Foun	High	Peak Foun	Peak Foun	Peak Foun	High
104.4	High	Peak Foun	Peak Foun	High	High	High	High	High
105.5	Peak Foun	Peak Foun	High	High	High	Peak Foun	Peak Foun	Peak Foun
98.5	Peak Foun	Peak Foun	Not Found	Not Found	Not Found	Peak Foun	Not Found	Peak Foun
133.1	High	Peak Foun	Peak Foun	Peak Foun	Not Found	Not Found	Not Found	Not Found
110.9	Peak Foun	Peak Foun	High	Peak Foun	Peak Foun	Peak Foun	Peak Foun	Peak Foun
117.9	Peak Foun	Peak Foun	Peak Foun	Not Found	Peak Foun	Peak Foun	High	Peak Foun
117	Peak Foun	Peak Foun	High	High	Not Found	Not Found	Not Found	Peak Foun
103.2	Not Found	Not Found	Not Found	Not Found	Not Found	Not Found	Not Found	Peak Foun
136	Peak Foun	High	High	Peak Foun	Peak Foun	Peak Foun	Peak Foun	Not Found
144.5	Peak Foun	Peak Foun	High	Peak Foun	Peak Foun	Peak Foun	Peak Foun	Peak Foun
135	Peak Foun	High	Peak Foun	High	Peak Foun	Peak Foun	Peak Foun	Peak Foun
95.3	High	Peak Foun	Peak Foun	Peak Foun	Peak Foun	Peak Foun	Peak Foun	Peak Foun
119.9	Peak Foun	Peak Foun	Peak Foun	Peak Foun	Peak Foun	Peak Foun	Peak Foun	Peak Foun
100.1	High	High	Peak Foun	Peak Foun	High	Peak Foun	Peak Foun	Peak Foun
93.3	Peak Foun	Peak Foun	High	Peak Foun	Not Found	Peak Foun	Peak Foun	Peak Foun
84.3	Peak Foun	Not Found	Peak Foun	Not Found	Peak Foun	High	Peak Foun	Not Found
116.2	Peak Foun	High	Peak Foun	Peak Foun	Peak Foun	Peak Foun	Peak Foun	High
124.6	High	High	Peak Foun	Peak Foun	Peak Foun	Peak Foun	Peak Foun	Peak Foun
131.5	Not Found	Peak Foun	Peak Foun	Not Found	Not Found	Not Found	Peak Foun	Not Found
88.7	Peak Foun	High	Peak Foun	Peak Foun	Not Found	Peak Foun	High	High
105	Peak Foun	High	High	Peak Foun	Peak Foun	Peak Foun	Peak Foun	Peak Foun
106.7	Peak Foun	Not Found	High	Peak Foun	Not Found	Not Found	Not Found	Not Found
101.4	Peak Foun	Peak Foun	Peak Foun	Peak Foun	Peak Foun	Peak Foun	Peak Foun	Peak Foun

87.4	High	Not Found	High	Peak Foun	Peak Foun	High	Not Found	Not Found
90.4	High	High	High	Not Found	Peak Foun	High	Peak Foun	High
106.4	High	High	High	Not Found	Peak Foun	High	Peak Foun	Not Found
105.3	Peak Foun	Peak Foun	High	Not Found	Not Found	Not Found	Peak Foun	Peak Foun
76.2	High	High	High	High	High	High	High	High
96.2	Peak Foun	High	High	Peak Foun	Peak Foun	High	Peak Foun	Not Found
	Not Found	Not Found	Not Found	Not Found	Peak Foun	Peak Foun	Peak Foun	Peak Foun
106.8	Peak Foun	Peak Foun	Peak Foun	Peak Foun	Peak Foun	High	High	High
115.5	High	High	High	Peak Foun	Peak Foun	Peak Foun	Peak Foun	Peak Foun
132	Peak Foun	High	Peak Foun	Peak Foun	Peak Foun	Peak Foun	Peak Foun	Peak Foun
	Not Found	Peak Foun	Not Found	Not Found	Peak Foun	Peak Foun	Not Found	Not Found
78	Not Found	Peak Foun	Peak Foun	Peak Foun	Peak Foun	Peak Foun	Peak Foun	Peak Foun
122.8	High	High	High	Peak Foun	Not Found	Peak Foun	Peak Foun	Peak Foun
104.3	Not Found	Peak Foun	Peak Foun	Not Found	Peak Foun	Peak Foun	High	High
136.5	Peak Foun	Peak Foun	Peak Foun	Not Found	Peak Foun	High	Peak Foun	High
75.4	Peak Foun	Not Found	Peak Foun	Not Found	Peak Foun	Peak Foun	Peak Foun	Not Found
115.2	Peak Foun	High	Not Found	Peak Foun	Peak Foun	High	Peak Foun	High
86.4	High	High	High	Not Found	Not Found	Not Found	Not Found	Not Found
127.5	High	Peak Foun	Not Found	Not Found	Peak Foun	Peak Foun	Not Found	Not Found
113.2	Not Found	High	High	Not Found	Peak Foun	Not Found	Peak Foun	Peak Foun
93.3	Peak Foun	High	High	Peak Foun	Not Found	Not Found	Not Found	Peak Foun
73.7	Not Found	Peak Foun	Not Found	Not Found	High	Peak Foun	High	Not Found
125.4	Peak Foun	Peak Foun	High	Peak Foun	Peak Foun	Peak Foun	Peak Foun	Peak Foun
	High	Not Found	Not Found	Not Found	Peak Foun	High	High	Not Found
105.4	High	Peak Foun	Peak Foun	Peak Foun	Peak Foun	Peak Foun	Peak Foun	Peak Foun
113.3	High	High	Peak Foun	Peak Foun	Peak Foun	Peak Foun	Peak Foun	Peak Foun
99.4	Peak Foun	Peak Foun	Peak Foun	Peak Foun	Peak Foun	Peak Foun	Peak Foun	Peak Foun
147	Not Found	Not Found	Not Found	Peak Foun	Not Found	Not Found	Not Found	Peak Foun
106	Peak Foun	Peak Foun	Peak Foun	Peak Foun	High	Peak Foun	High	Peak Foun
134	Peak Foun	Peak Foun	Peak Foun	Peak Foun	Peak Foun	Not Found	Not Found	High
109.9	High	High	High	High	Peak Foun	High	Peak Foun	Peak Foun
95.1	Peak Foun	High	Peak Foun	Peak Foun	Peak Foun	High	High	High
99.4	Peak Foun	Peak Foun	Peak Foun	High	Peak Foun	Peak Foun	Peak Foun	Peak Foun
124.1	Peak Foun	High	High	Not Found	Not Found	Peak Foun	Peak Foun	Not Found
145.7	Not Found	Peak Foun	Not Found	Not Found	Not Found	Not Found	Not Found	Not Found
	Not Found	Peak Foun	Not Found	Not Found	Not Found	Peak Foun	High	Not Found
111.4	High	High	High	High	Not Found	Not Found	Not Found	Peak Foun
119	Peak Foun	High	High	High	Peak Foun	High	High	Peak Foun
128.6	High	High	Peak Foun	Not Found	Peak Foun	High	Peak Foun	Peak Foun
117.4	High	Peak Foun	Peak Foun	Peak Foun	Peak Foun	Not Found	Peak Foun	Not Found
	Peak Foun	Peak Foun	High	Not Found	Not Found	Not Found	Not Found	Not Found
84.9	Peak Foun	Peak Foun	High	Peak Foun	Peak Foun	Not Found	Peak Foun	Peak Foun
119	Not Found	Peak Foun	Not Found	Peak Foun	Not Found	Not Found	Peak Foun	Peak Foun
162.9	Peak Foun	Peak Foun	Peak Foun	Peak Foun	Peak Foun	Peak Foun	Not Found	Peak Foun
119.8	Not Found	Peak Foun	High	Peak Foun	Not Found	Peak Foun	Peak Foun	Peak Foun

100	Peak Foun	High	Peak Foun	Peak Foun	Not Found	Peak Foun	Peak Foun	Peak Foun
137	Peak Foun	High	Peak Foun	Not Found	Peak Foun	Peak Foun	Peak Foun	Peak Foun
106.2	Peak Foun	High	High	Peak Foun	Peak Foun	Peak Foun	Peak Foun	Peak Foun
101.4	Peak Foun	Peak Foun	High	Peak Foun	Peak Foun	Peak Foun	Peak Foun	Peak Foun
149.6	Not Found	Not Found	Not Found	Not Found	Peak Foun	Not Found	Not Found	Not Found
83	High	High	High	Not Found	Not Found	Peak Foun	Peak Foun	Peak Foun
143.6	Not Found	Not Found	Peak Foun	Not Found	Peak Foun	Peak Foun	Not Found	Not Found
	Not Found	Not Found	Not Found	Not Found	Not Found	Peak Foun	High	Peak Foun
92.9	Peak Foun	Peak Foun	Peak Foun	Peak Foun	High	Peak Foun	High	High
87.4	Peak Foun	Peak Foun	Not Found	Not Found	Peak Foun	Peak Foun	Peak Foun	Peak Foun
110.4	Peak Foun	Peak Foun	Peak Foun	Peak Foun	High	Peak Foun	Peak Foun	Peak Foun
200	Not Found	Not Found	Not Found	Not Found	Not Found	Not Found	Not Found	Not Found
102.8	Peak Foun	Peak Foun	Peak Foun	Peak Foun	Peak Foun	High	Peak Foun	Peak Foun
98.4	Peak Foun	High	Peak Foun	Peak Foun	Peak Foun	Peak Foun	Peak Foun	Peak Foun
123.3	High	High	Peak Foun	High	Peak Foun	High	High	High
115.4	High	High	High	Peak Foun	Peak Foun	Peak Foun	High	High
129.7	Peak Foun	Peak Foun	Not Found	Peak Foun	Not Found	Peak Foun	Peak Foun	Peak Foun
76.2	Not Found	Peak Foun	Not Found	Peak Foun	Not Found	Peak Foun	Peak Foun	Peak Foun
118.5	High	High	High	High	Peak Foun	Peak Foun	Peak Foun	Peak Foun
184.3	Not Found	Not Found	Not Found	Peak Foun	Not Found	Peak Foun	Not Found	Not Found
93.9	High	Peak Foun	Peak Foun	Peak Foun	Peak Foun	Peak Foun	Peak Foun	Peak Foun
117.4	Peak Foun	Peak Foun	Peak Foun	Peak Foun	Not Found	Peak Foun	Not Found	Peak Foun
99.4	Peak Foun	Peak Foun	High	Not Found	Peak Foun	Peak Foun	Peak Foun	Not Found
112.9	High	High	High	Peak Foun	Not Found	Not Found	Not Found	Peak Foun
81.4	Peak Foun	Peak Foun	Peak Foun	Not Found	Not Found	Peak Foun	Peak Foun	Not Found
98.8	Peak Foun	Peak Foun	Peak Foun	Peak Foun	Peak Foun	Peak Foun	High	Peak Foun
107.2	High	Peak Foun	Peak Foun	Peak Foun	Not Found	Peak Foun	Peak Foun	Not Found
106.3	High	Peak Foun	Peak Foun	Peak Foun	Not Found	Not Found	Not Found	Not Found
114.8	Peak Foun	Peak Foun	Peak Foun	Peak Foun	Peak Foun	Peak Foun	Not Found	High
85.9	High	High	High	High	Peak Foun	Peak Foun	Peak Foun	Not Found
137.8	Peak Foun	High	Peak Foun	Peak Foun	Not Found	Peak Foun	Not Found	Not Found
131.3	High	High	High	Not Found	Not Found	Peak Foun	Peak Foun	High
111.5	Peak Foun	Peak Foun	High	Peak Foun	Not Found	Not Found	High	Not Found
108.7	Peak Foun	Peak Foun	Peak Foun	Peak Foun	Peak Foun	Peak Foun	Peak Foun	Peak Foun
110.4	Peak Foun	Peak Foun	Peak Foun	Peak Foun	Peak Foun	Peak Foun	Peak Foun	Peak Foun
70.6	Not Found	High	Not Found	Not Found	Not Found	Not Found	Peak Foun	Peak Foun
117	Peak Foun	Peak Foun	High	Not Found	Not Found	Not Found	Not Found	Not Found
119.7	Peak Foun	Peak Foun	High	Peak Foun	Peak Foun	Peak Foun	Peak Foun	Peak Foun
121.1	Peak Foun	Peak Foun	Peak Foun	Not Found	Not Found	Peak Foun	Not Found	Not Found
75.2	Peak Foun	Peak Foun	Not Found	High	Peak Foun	Peak Foun	High	Not Found
112.7	Peak Foun	High	High	Peak Foun	Peak Foun	High	Peak Foun	Peak Foun
96.6	Peak Foun	High	Peak Foun	Peak Foun	Peak Foun	Peak Foun	High	High
121	Peak Foun	High	Peak Foun	Not Found	Not Found	Peak Foun	Peak Foun	Not Found
	High	Not Found	Peak Foun	Peak Foun	Not Found	Not Found	Not Found	Not Found
144.6	Peak Foun	Not Found	Not Found	Not Found	Not Found	Not Found	Not Found	Not Found

105.5	Peak Foun	Peak Foun	Peak Foun	Peak Foun	Peak Foun	Peak Foun	Not Found	Peak Foun
114.9	High	Peak Foun	Peak Foun	High	Not Found	Peak Foun	Not Found	Peak Foun
110.3	Peak Foun	Not Found	High	Not Found	Not Found	Peak Foun	Not Found	Peak Foun
100.6	High	Peak Foun	High	Not Found	Peak Foun	Peak Foun	High	High
94.8	Peak Foun	Peak Foun	Peak Foun	Peak Foun	Peak Foun	High	Peak Foun	Not Found
92.6	High	High	Peak Foun	Peak Foun	High	Peak Foun	High	High
95.2	Peak Foun	High	Not Found	Peak Foun	Not Found	Not Found	Not Found	Not Found
127.7	Peak Foun	Peak Foun	Peak Foun	Peak Foun	Not Found	Not Found	Peak Foun	Not Found
114.8	Peak Foun	Not Found	High	Not Found	Not Found	Not Found	Peak Foun	Peak Foun
98.3	High	High	High	High	Peak Foun	Peak Foun	Peak Foun	Peak Foun
145.5	Peak Foun	Peak Foun	High	High	Not Found	Not Found	Peak Foun	Peak Foun
101.4	High	High	High	Peak Foun	Peak Foun	Not Found	Peak Foun	Peak Foun
68.5	Peak Foun	Peak Foun	High	Not Found	Not Found	Not Found	Peak Foun	Peak Foun
104.5	Peak Foun	High	Not Found	Peak Foun	Not Found	Peak Foun	Peak Foun	Peak Foun
114.9	Peak Foun	Peak Foun	High	Peak Foun	Peak Foun	Peak Foun	Peak Foun	High
120.8	Peak Foun	Peak Foun	Peak Foun	Peak Foun	Peak Foun	Peak Foun	Peak Foun	Peak Foun
101.5	Peak Foun	Peak Foun	Peak Foun	Peak Foun	Peak Foun	Not Found	Not Found	Not Found
109.7	Not Found	Not Found	Peak Foun	Not Found	Not Found	Peak Foun	Peak Foun	Peak Foun
115.8	Peak Foun	Peak Foun	Peak Foun	Peak Foun	Peak Foun	Peak Foun	Not Found	Not Found
91.7	Peak Foun	High	High	Not Found	Peak Foun	Not Found	Peak Foun	Peak Foun
	Not Found	Not Found	Not Found	Not Found	Not Found	Not Found	High	Not Found
90.8	High	High	High	Peak Foun	High	Peak Foun	Peak Foun	Peak Foun
111.6	High	High	Peak Foun	High	Peak Foun	Peak Foun	Peak Foun	Peak Foun
110.6	High	High	Peak Foun	Peak Foun	Not Found	Peak Foun	Not Found	Peak Foun
133.7	Peak Foun	Peak Foun	High	Peak Foun	Peak Foun	Not Found	Peak Foun	Not Found
142.7	Not Found	Not Found	Peak Foun	Not Found	Peak Foun	Peak Foun	Peak Foun	Peak Foun
73	Peak Foun	Peak Foun	Not Found	Not Found	Peak Foun	Peak Foun	Peak Foun	Not Found
117	Peak Foun	Peak Foun	Peak Foun	Peak Foun	Peak Foun	High	High	Peak Foun
97.6	Peak Foun	High	Peak Foun	Peak Foun	Peak Foun	Peak Foun	Peak Foun	Peak Foun
110.8	Peak Foun	Peak Foun	Peak Foun	Peak Foun	Peak Foun	Peak Foun	Peak Foun	Not Found
110.8	Peak Foun	High	Not Found	Not Found	Not Found	Peak Foun	Peak Foun	Peak Foun
	High	High	Peak Foun	Peak Foun	Not Found	Not Found	Not Found	Not Found
118	High	High	Peak Foun	Peak Foun	Not Found	Not Found	Peak Foun	Not Found
105	High	High	High	Peak Foun	Peak Foun	Peak Foun	Peak Foun	Peak Foun
105.4	Peak Foun	Peak Foun	Peak Foun	Peak Foun	Peak Foun	Peak Foun	Peak Foun	Peak Foun
101.1	High	High	Peak Foun	Not Found	Peak Foun	Peak Foun	Peak Foun	Peak Foun
105.2	Peak Foun	Peak Foun	High	Peak Foun	Peak Foun	Not Found	Peak Foun	Peak Foun
113.3	Peak Foun	High	High	Peak Foun	Peak Foun	High	Peak Foun	Peak Foun
95.2	Peak Foun	Peak Foun	Peak Foun	Not Found	Peak Foun	Peak Foun	Peak Foun	Peak Foun
118.4	Peak Foun	Peak Foun	Peak Foun	Peak Foun	Peak Foun	Not Found	Peak Foun	Peak Foun
112.2	Not Found	Peak Foun	Not Found	Peak Foun	Peak Foun	Peak Foun	Peak Foun	Not Found
92.1	Not Found	Peak Foun	High	High	High	High	High	Peak Foun
104.5	High	Peak Foun	High	Not Found	Peak Foun	Peak Foun	Peak Foun	Peak Foun
113.3	High	Peak Foun	Peak Foun	Not Found	Not Found	Peak Foun	Peak Foun	Peak Foun
114.4	High	High	Peak Foun	Not Found	Not Found	Not Found	Not Found	Peak Foun

105.3	High	Peak Foun	High	Peak Foun	Not Found	Peak Foun	Peak Foun	Peak Foun
116.7	Peak Foun	Peak Foun	High	Not Found	Peak Foun	Peak Foun	Peak Foun	Peak Foun
111.6	Peak Foun	Peak Foun	Peak Foun	Peak Foun	Peak Foun	High	Peak Foun	Peak Foun
89.4	Peak Foun	Peak Foun	Peak Foun	Peak Foun	Not Found	Peak Foun	Peak Foun	Peak Foun
110.8	Peak Foun	Peak Foun	Peak Foun	Not Found	Peak Foun	Not Found	Peak Foun	Peak Foun
117.5	Peak Foun	High	Peak Foun	Not Found	Peak Foun	Peak Foun	Peak Foun	Peak Foun
135	Peak Foun	Peak Foun	Peak Foun	Peak Foun	Peak Foun	Peak Foun	Peak Foun	High
200	Not Found	Not Found	Not Found	Not Found	Not Found	Not Found	Not Found	Not Found
109.4	Peak Foun	High	High	Peak Foun	Not Found	Not Found	Not Found	Not Found
90.1	High	High	High	High	Not Found	High	High	High
120.7	Peak Foun	Peak Foun	Not Found	Not Found	Not Found	Not Found	Not Found	Not Found
	Peak Foun	Peak Foun	High	Not Found	Peak Foun	Peak Foun	Peak Foun	Peak Foun
106	Peak Foun	Peak Foun	Peak Foun	Not Found	Not Found	Not Found	Not Found	Peak Foun
124.9	Peak Foun	High	High	High	Peak Foun	High	High	High
110.8	Not Found	Peak Foun	High	Peak Foun	Not Found	Peak Foun	Peak Foun	Not Found
134.8	High	Peak Foun	Peak Foun	Peak Foun	Not Found	Not Found	Not Found	Not Found
140.4	Peak Foun	Peak Foun	Peak Foun	Peak Foun	Not Found	Peak Foun	Peak Foun	Peak Foun
100.9	High	High	Peak Foun	High	Peak Foun	Peak Foun	High	High
113.5	High	Peak Foun	Not Found	Not Found	Not Found	Not Found	Not Found	Not Found
112.8	Peak Foun	Peak Foun	Peak Foun	Peak Foun	Not Found	Peak Foun	Peak Foun	Peak Foun
108	Peak Foun	Peak Foun	High	Peak Foun	Peak Foun	Peak Foun	Peak Foun	Peak Foun
111.6	Peak Foun	Peak Foun	Peak Foun	Peak Foun	Peak Foun	High	Peak Foun	Peak Foun
55.5	High	High	High	High	High	High	High	Peak Foun
95.2	Not Found	Not Found	Peak Foun	Not Found	Peak Foun	Not Found	Peak Foun	Not Found
117	Peak Foun	High	Peak Foun	Not Found	Not Found	Not Found	Peak Foun	Not Found
101.3	Peak Foun	Peak Foun	Not Found	Not Found	Not Found	Peak Foun	Not Found	Not Found
106.3	Peak Foun	Peak Foun	Peak Foun	Not Found	Not Found	Not Found	Not Found	Peak Foun
106.2	Peak Foun	High	Not Found	Peak Foun	Peak Foun	Peak Foun	Not Found	Not Found
116.6	Peak Foun	Peak Foun	Peak Foun	Peak Foun	Not Found	Peak Foun	Peak Foun	Not Found
110.9	High	High	High	High	High	High	High	High
144.1	Not Found	Not Found	Not Found	Peak Foun	Not Found	Peak Foun	Peak Foun	Not Found
120.1	Peak Foun	High	Peak Foun	Peak Foun	Not Found	High	Peak Foun	Peak Foun
99.4	Peak Foun	Peak Foun	Peak Foun	Peak Foun	Peak Foun	High	Peak Foun	Peak Foun
102.4	Peak Foun	Peak Foun	Peak Foun	Peak Foun	Peak Foun	High	Peak Foun	Peak Foun
131.2	Not Found	Not Found	Peak Foun	Peak Foun	Peak Foun	Peak Foun	Peak Foun	High
67.2	High	High	Peak Foun	High	Peak Foun	High	High	Not Found
112.2	Peak Foun	Peak Foun	Peak Foun	Peak Foun	Peak Foun	Peak Foun	Peak Foun	Peak Foun
102.2	High	Peak Foun	Peak Foun	Peak Foun	Peak Foun	Peak Foun	Peak Foun	Peak Foun
200	Not Found	Not Found	Not Found	Not Found	Not Found	Not Found	Not Found	Not Found
94.9	Peak Foun	High	High	Not Found	Peak Foun	Peak Foun	Peak Foun	Not Found
115.9	Peak Foun	High	Peak Foun	Peak Foun	Not Found	Peak Foun	Peak Foun	Not Found
110.7	Peak Foun	Peak Foun	Peak Foun	Peak Foun	Peak Foun	Peak Foun	Peak Foun	Peak Foun
109.6	Peak Foun	Peak Foun	High	Peak Foun	Not Found	Not Found	High	Not Found
78.9	Not Found	High	Not Found	Not Found	Not Found	Not Found	Not Found	Not Found
116.8	Peak Foun	Peak Foun	Not Found	Not Found	Peak Foun	Peak Foun	Not Found	Peak Foun

102.4	Not Found	Peak Foun	High	Peak Foun	Not Found	Peak Foun	Peak Foun	Peak Foun
116.4	Peak Foun	High	High	Not Found	Peak Foun	Peak Foun	Peak Foun	Peak Foun
111.7	Peak Foun	Peak Foun	Peak Foun	Peak Foun	Not Found	High	Peak Foun	Peak Foun
10.8	Peak Foun	Peak Foun	Peak Foun	Not Found	High	High	High	Peak Foun
87.5	High	High	High	High	High	High	High	High
105.3	Not Found	Peak Foun	Peak Foun	Peak Foun	Not Found	High	Peak Foun	Peak Foun
104.2	Peak Foun	Peak Foun	Peak Foun	High	Not Found	Peak Foun	Peak Foun	Not Found
122.4	High	Peak Foun	High	Peak Foun	Peak Foun	Peak Foun	Peak Foun	Peak Foun
111.3	High	High	Peak Foun	Peak Foun	Peak Foun	Not Found	Peak Foun	Peak Foun
137.4	High	Peak Foun	Peak Foun	Not Found	Peak Foun	Peak Foun	Peak Foun	Peak Foun
113	High	High	High	Peak Foun	Peak Foun	Peak Foun	Peak Foun	Peak Foun
119.5	Peak Foun	Peak Foun	Peak Foun	Peak Foun	Peak Foun	Peak Foun	Peak Foun	Peak Foun
113.2	Peak Foun	Peak Foun	Peak Foun	Peak Foun	Peak Foun	Not Found	Peak Foun	Peak Foun
80.1	Peak Foun	Peak Foun	Peak Foun	Not Found	High	Peak Foun	Peak Foun	Peak Foun
	Peak Foun	High	High	Not Found	High	High	High	Peak Foun
108.2	Peak Foun	Peak Foun	Peak Foun	Peak Foun	High	Peak Foun	Peak Foun	Peak Foun
101.2	High	High	Peak Foun	Peak Foun	Peak Foun	Peak Foun	Peak Foun	Peak Foun
56.4	Peak Foun	Peak Foun	Peak Foun	High	Not Found	Peak Foun	Peak Foun	High
119	High	Not Found	Peak Foun	Peak Foun	Not Found	Not Found	Not Found	Not Found
111.4	Peak Foun	Peak Foun	Peak Foun	Peak Foun	Peak Foun	Not Found	Not Found	Peak Foun
108.9	Not Found	Not Found	Not Found	Not Found	High	Peak Foun	Peak Foun	Peak Foun
115.2	Peak Foun	High	High	Not Found	Peak Foun	Not Found	Peak Foun	Peak Foun
87.2	High	High	Peak Foun	Peak Foun	Peak Foun	Peak Foun	Not Found	Peak Foun
200	Not Found	Not Found	Not Found	Not Found	Not Found	Not Found	Not Found	Not Found
	Peak Foun	Peak Foun	High	Not Found	Not Found	Not Found	Not Found	Not Found
99.5	Peak Foun	Peak Foun	High	Peak Foun	Not Found	Not Found	Not Found	Not Found
104.3	Peak Foun	Peak Foun	Peak Foun	Not Found	Not Found	Not Found	Not Found	Not Found
15.7	Peak Foun	Peak Foun	Not Found	Not Found	Peak Foun	Peak Foun	Peak Foun	Not Found
114	Peak Foun	High	Peak Foun	Peak Foun	Peak Foun	Peak Foun	High	Peak Foun
105.1	Peak Foun	Peak Foun	High	Peak Foun	Peak Foun	Peak Foun	Peak Foun	Peak Foun
90.6	High	High	Not Found	Peak Foun	Not Found	Peak Foun	Not Found	Not Found
121.2	High	High	Not Found	Not Found	Not Found	Peak Foun	Not Found	Not Found
118.6	High	High	Peak Foun	Not Found	Peak Foun	Peak Foun	Peak Foun	Peak Foun
124.2	Peak Foun	Peak Foun	Peak Foun	Peak Foun	Peak Foun	Peak Foun	Not Found	Not Found
146	Not Found	Not Found	High	Not Found	Not Found	Not Found	Peak Foun	Peak Foun
110	Peak Foun	Peak Foun	Peak Foun	Not Found	Peak Foun	Not Found	Not Found	Not Found
126.3	Peak Foun	Peak Foun	High	Not Found	Peak Foun	Not Found	Peak Foun	Peak Foun
98.1	Peak Foun	Peak Foun	Peak Foun	Peak Foun	Peak Foun	Peak Foun	Peak Foun	Peak Foun
131.4	Peak Foun	Peak Foun	Peak Foun	Peak Foun	Not Found	Peak Foun	Not Found	Peak Foun
108.4	High	Peak Foun	High	Peak Foun	Peak Foun	Peak Foun	Peak Foun	Peak Foun
109.7	Peak Foun	Peak Foun	Peak Foun	Peak Foun	Not Found	Peak Foun	Peak Foun	Peak Foun
103.7	Peak Foun	Peak Foun	Peak Foun	Peak Foun	High	Peak Foun	Peak Foun	Peak Foun
94.8	Peak Foun	Not Found	Peak Foun	Not Found	Not Found	Not Found	Peak Foun	Peak Foun
122.7	High	Peak Foun	Peak Foun	Not Found	Peak Foun	Peak Foun	Peak Foun	Not Found
95.3	High	Peak Foun	Peak Foun	Peak Foun	Peak Foun	High	Peak Foun	Peak Foun

92.6	Peak Foun	High	Peak Foun	Peak Foun	Peak Foun	Not Found	Not Found	Peak Foun
96.6	High	High	High	Peak Foun	Peak Foun	High	High	High
97.6	High	High	Peak Foun	Peak Foun	Peak Foun	Peak Foun	Peak Foun	Peak Foun
179.8	Peak Foun	Peak Foun	Peak Foun	Peak Foun	Peak Foun	Not Found	Not Found	Peak Foun
109.4	Peak Foun	Peak Foun	High	Peak Foun	Not Found	Peak Foun	Peak Foun	Not Found
86	High	Peak Foun	Peak Foun	Peak Foun	Peak Foun	High	Peak Foun	High
92.2	Peak Foun	High	High	High	Not Found	High	High	Peak Foun
106.7	Peak Foun	Peak Foun	High	Peak Foun	High	Peak Foun	Peak Foun	Peak Foun
87.9	High	High	Peak Foun	Peak Foun	Not Found	Not Found	Not Found	Peak Foun
155.2	Peak Foun	Peak Foun	High	Not Found	Not Found	Not Found	Not Found	Peak Foun
63	Peak Foun	Peak Foun	Peak Foun	Peak Foun	Peak Foun	Peak Foun	Peak Foun	Not Found
145.1	Peak Foun	Peak Foun	Peak Foun	Not Found	Peak Foun	Peak Foun	Peak Foun	Not Found
88.4	High	Peak Foun	Not Found	Not Found	Not Found	Not Found	Not Found	Not Found
105.5	Peak Foun	Peak Foun	High	Peak Foun	Peak Foun	Peak Foun	Peak Foun	Peak Foun
85.8	Not Found	Not Found	Not Found	Not Found	Not Found	Not Found	Peak Foun	Peak Foun
107.1	Peak Foun	Not Found	Peak Foun	Peak Foun	Peak Foun	Peak Foun	Peak Foun	Peak Foun
148	Peak Foun	Peak Foun	Peak Foun	Not Found	Peak Foun	Peak Foun	Peak Foun	Peak Foun
95.9	Not Found	High	Peak Foun	Not Found	Not Found	Not Found	Not Found	Not Found
124	Peak Foun	High	Peak Foun	Peak Foun	Not Found	Peak Foun	Peak Foun	Peak Foun
	Peak Foun	Not Found	Not Found	Not Found	Not Found	Not Found	High	Not Found
114.5	Peak Foun	High	Not Found	Not Found	Not Found	Not Found	Not Found	Not Found
110.2	High	High	Peak Foun	Peak Foun	Peak Foun	High	Peak Foun	Peak Foun
108.9	Peak Foun	High	Peak Foun	Peak Foun	Not Found	Peak Foun	Peak Foun	Peak Foun
115.7	High	Peak Foun	Peak Foun	Not Found	Peak Foun	Peak Foun	Peak Foun	Peak Foun
102.3	Not Found	Not Found	High	High	Peak Foun	Peak Foun	Peak Foun	Peak Foun
119.6	High	Peak Foun	Peak Foun	Peak Foun	Not Found	Peak Foun	Peak Foun	Peak Foun
78.1	Peak Foun	Not Found	High	Peak Foun	Not Found	Not Found	Peak Foun	Not Found
108.8	Peak Foun	High	High	Peak Foun	Peak Foun	Peak Foun	Peak Foun	Peak Foun
124	High	Peak Foun	High	Peak Foun	Peak Foun	Peak Foun	Peak Foun	Peak Foun
103.1	High	Peak Foun	Peak Foun	Peak Foun	Peak Foun	Peak Foun	Peak Foun	Peak Foun
122.1	Peak Foun	Peak Foun	High	Peak Foun	Peak Foun	Peak Foun	Peak Foun	Peak Foun
138.3	Peak Foun	Peak Foun	High	Peak Foun	Peak Foun	Not Found	Not Found	Peak Foun
84	Not Found	Not Found	Peak Foun	Not Found	Peak Foun	Peak Foun	Peak Foun	Not Found
102.5	High	Peak Foun	Peak Foun	Peak Foun	Not Found	Not Found	Not Found	Not Found
97.5	Peak Foun	Peak Foun	Peak Foun	Peak Foun	Not Found	Not Found	Not Found	High
126.4	High	High	High	High	Not Found	Peak Foun	Peak Foun	High
86.2	Not Found	High	Not Found	Not Found	Peak Foun	Peak Foun	Peak Foun	Peak Foun
109.6	High	High	Peak Foun	Peak Foun	Peak Foun	Peak Foun	Peak Foun	Peak Foun
114.6	Peak Foun	High	Peak Foun	Peak Foun	Not Found	Not Found	Not Found	Not Found
96.5	High	Not Found	Not Found	Not Found	Not Found	Not Found	Not Found	Not Found
158.5	Peak Foun	High	Peak Foun	Peak Foun	Not Found	Not Found	Peak Foun	High
43.1	Peak Foun	High	Peak Foun	Peak Foun	Peak Foun	Not Found	Peak Foun	Peak Foun
38.4	Not Found	Not Found	Not Found	Not Found	High	Not Found	Not Found	Not Found
99.2	High	High	Peak Foun	Peak Foun	Peak Foun	High	Peak Foun	Peak Foun
101.7	High	High	High	High	Peak Foun	Peak Foun	Peak Foun	High

167.2	Not Found	Not Found	Not Found	Not Found	Not Found	Peak Foun	Not Found	Not Found
110.4	Peak Foun	Peak Foun	Peak Foun	Peak Foun	Peak Foun	Peak Foun	Peak Foun	Peak Foun
105.2	High	Peak Foun	Peak Foun	Peak Foun	Peak Foun	Peak Foun	Peak Foun	Peak Foun
60.7	Peak Foun	Peak Foun	High	Not Found	Peak Foun	Peak Foun	Peak Foun	Not Found
111.1	Peak Foun	Not Found	Not Found	Peak Foun	Peak Foun	Peak Foun	High	Peak Foun
135.5	Peak Foun	Peak Foun	High	Peak Foun	Not Found	Not Found	Not Found	Peak Foun
129	High	High	High	High	Peak Foun	High	Peak Foun	High
119.7	Peak Foun	High	Peak Foun	Peak Foun	Not Found	Not Found	Not Found	Peak Foun
12.8	Peak Foun	Peak Foun	Peak Foun	Peak Foun	Peak Foun	Peak Foun	Peak Foun	Peak Foun
	Peak Foun	High	Peak Foun	Peak Foun	Peak Foun	Peak Foun	Peak Foun	Peak Foun
110.8	Peak Foun	Peak Foun	High	Not Found	Peak Foun	Peak Foun	Peak Foun	Not Found
93.7	Peak Foun	Peak Foun	High	Peak Foun	Peak Foun	Peak Foun	Peak Foun	Peak Foun
115.1	High	High	High	High	High	Peak Foun	High	High
108	Peak Foun	Peak Foun	Peak Foun	Peak Foun	High	Peak Foun	Peak Foun	Peak Foun
97.5	Peak Foun	Peak Foun	Peak Foun	Peak Foun	Peak Foun	Peak Foun	Not Found	Peak Foun
108.4	Peak Foun	High	Peak Foun	Not Found	Not Found	Peak Foun	Peak Foun	Peak Foun
200	Not Found	Not Found	Not Found	Not Found	Not Found	Not Found	Not Found	Not Found
99.9	Peak Foun	Peak Foun	Peak Foun	Peak Foun	Not Found	Peak Foun	Peak Foun	Peak Foun
79.5	Not Found	Not Found	Not Found	High	Not Found	Not Found	Not Found	Not Found
93	Peak Foun	Peak Foun	High	Peak Foun	Peak Foun	Peak Foun	Peak Foun	Not Found
153.9	Peak Foun	Peak Foun	High	Not Found	Not Found	Peak Foun	Peak Foun	Not Found
116.6	Peak Foun	Peak Foun	High	High	Peak Foun	Peak Foun	Peak Foun	High
101.8	Peak Foun	High	Peak Foun	Peak Foun	Peak Foun	Peak Foun	Peak Foun	Peak Foun
96.9	Peak Foun	High	High	Peak Foun	Peak Foun	High	Peak Foun	Peak Foun
94.8	Peak Foun	Peak Foun	Peak Foun	Peak Foun	Peak Foun	Peak Foun	Peak Foun	Peak Foun
123.6	Not Found	Peak Foun	Peak Foun	Not Found	Not Found	Not Found	Not Found	Not Found
107.6	High	High	High	High	High	Peak Foun	High	High
112.2	Peak Foun	High	Not Found	Not Found	Peak Foun	Peak Foun	Not Found	Not Found
125.7	Not Found	Not Found	High	Not Found	Not Found	Peak Foun	Peak Foun	Not Found
102.8	High	High	High	High	Peak Foun	High	Peak Foun	Peak Foun
130.8	Peak Foun	Peak Foun	Peak Foun	Peak Foun	Not Found	Peak Foun	Peak Foun	Peak Foun
126.7	Peak Foun	Peak Foun	Peak Foun	Peak Foun	Not Found	Not Found	Peak Foun	Peak Foun
109.3	Peak Foun	Peak Foun	Peak Foun	Peak Foun	High	High	High	Peak Foun
73.1	Peak Foun	Peak Foun	Peak Foun	Peak Foun	Peak Foun	Peak Foun	Not Found	Not Found
107.5	Peak Foun	Peak Foun	Peak Foun	High	Peak Foun	Peak Foun	High	Peak Foun
115.9	Peak Foun	Peak Foun	Peak Foun	Peak Foun	Not Found	Not Found	Peak Foun	Peak Foun
121.1	Peak Foun	Peak Foun	High	Peak Foun	Peak Foun	Peak Foun	Peak Foun	Peak Foun
121.5	Not Found	Peak Foun	High	Peak Foun	Peak Foun	Peak Foun	Peak Foun	Peak Foun
84.3	Peak Foun	Peak Foun	Peak Foun	Peak Foun	Peak Foun	Not Found	Not Found	Peak Foun
86.6	Not Found	Not Found	Not Found	Not Found	Peak Foun	High	Peak Foun	Peak Foun
36	Not Found	High	Peak Foun	Not Found	Peak Foun	Peak Foun	Peak Foun	Peak Foun
84.8	Peak Foun	High	Peak Foun	Peak Foun	Not Found	Not Found	Not Found	Not Found
114	Not Found	Peak Foun	Peak Foun	Not Found	Not Found	Not Found	Peak Foun	Not Found
96.3	High	Peak Foun	High	Peak Foun	High	Peak Foun	High	Peak Foun
88.4	Peak Foun	Peak Foun	Peak Foun	Peak Foun	Not Found	Peak Foun	Not Found	Peak Foun

114.8	High	Peak Foun	Peak Foun	Peak Foun	Peak Foun	Not Found	Not Found	Peak Foun
100.8	Peak Foun	High	High	Peak Foun	Not Found	Not Found	Not Found	Peak Foun
114	Peak Foun	Peak Foun	Not Found	Not Found	Not Found	Not Found	Not Found	Not Found
117.6	High	Peak Foun	Peak Foun	Not Found	Peak Foun	Peak Foun	Peak Foun	Peak Foun
95.6	Peak Foun	Peak Foun	Peak Foun	Peak Foun	High	High	Peak Foun	Peak Foun
123.7	High	Peak Foun	Peak Foun	Peak Foun	Peak Foun	Peak Foun	Peak Foun	Peak Foun
103.6	High	Peak Foun	Peak Foun	Peak Foun	Peak Foun	Peak Foun	Peak Foun	Peak Foun
126.6	High	Peak Foun	Peak Foun	Peak Foun	Not Found	Not Found	Not Found	Peak Foun
83.8	Peak Foun	High	High	Peak Foun	Peak Foun	Not Found	Peak Foun	Peak Foun
107.3	Peak Foun	Peak Foun	High	Peak Foun	Not Found	Peak Foun	Peak Foun	Peak Foun
	Not Found	Not Found	Not Found	Not Found	Peak Foun	High	Not Found	Not Found
108.9	Not Found	High	Peak Foun	Not Found	Peak Foun	Peak Foun	Peak Foun	Peak Foun
61.9	Peak Foun	High	Peak Foun	Peak Foun	Peak Foun	Peak Foun	Peak Foun	Peak Foun
117.5	Peak Foun	Peak Foun	Peak Foun	Peak Foun	Not Found	Peak Foun	Peak Foun	Peak Foun
0.1	Peak Foun	Peak Foun	Peak Foun	Peak Foun	High	High	Peak Foun	High
114.9	Not Found	Not Found	Not Found	High	Not Found	Not Found	Not Found	Peak Foun
92	Peak Foun	Not Found	Not Found	Not Found	Not Found	Not Found	Not Found	Peak Foun
	Not Found	Not Found	Peak Foun	Not Found	Not Found	Not Found	High	Peak Foun
	Peak Foun	Peak Foun	High	Peak Foun	Not Found	Peak Foun	Not Found	Peak Foun
86.5	High	Peak Foun	Peak Foun	Peak Foun	Peak Foun	Peak Foun	Peak Foun	Peak Foun
130.4	Peak Foun	High	Peak Foun	Peak Foun	Peak Foun	Peak Foun	Peak Foun	Peak Foun
137.9	High	High	Peak Foun	Not Found	Not Found	Not Found	Peak Foun	Peak Foun
86.8	High	High	High	Peak Foun	Not Found	Peak Foun	Peak Foun	Peak Foun
92.4	High	Peak Foun	High	Peak Foun	Peak Foun	High	High	Not Found
106.5	Peak Foun	High	High	Peak Foun	Not Found	Peak Foun	Peak Foun	Peak Foun
	Not Found	Not Found	Not Found	Not Found	Not Found	Not Found	Not Found	Not Found
123.8	Peak Foun	Peak Foun	Not Found	Not Found	Not Found	Peak Foun	Peak Foun	Peak Foun
91.1	Peak Foun	Not Found	Not Found	High	Peak Foun	Not Found	Peak Foun	Peak Foun
101.7	High	Peak Foun	Peak Foun	Not Found	Peak Foun	Peak Foun	Peak Foun	Peak Foun
91	Peak Foun	Peak Foun	Not Found	Peak Foun	High	Peak Foun	Not Found	Peak Foun
98.6	Peak Foun	Peak Foun	Peak Foun	Not Found	Peak Foun	Peak Foun	Peak Foun	Peak Foun
99.8	Peak Foun	Peak Foun	Peak Foun	Peak Foun	Peak Foun	Peak Foun	Peak Foun	Peak Foun
84.5	Peak Foun	Peak Foun	High	High	Peak Foun	Peak Foun	Peak Foun	Peak Foun
86.3	Peak Foun	Not Found	Peak Foun	Peak Foun	Not Found	Peak Foun	Peak Foun	Not Found
127.4	Peak Foun	High	Peak Foun	Peak Foun	Not Found	Peak Foun	Peak Foun	Peak Foun
86.4	Peak Foun	Not Found	Not Found	Not Found	Not Found	Not Found	Not Found	Not Found
111.2	Peak Foun	Peak Foun	Not Found	Peak Foun	Peak Foun	Peak Foun	Peak Foun	Peak Foun
74.9	Peak Foun	Not Found	Peak Foun	Not Found	Peak Foun	Peak Foun	Peak Foun	Peak Foun
116.5	Peak Foun	Not Found	Not Found	Peak Foun	Not Found	Not Found	Peak Foun	Peak Foun
115.1	High	Peak Foun	Peak Foun	Peak Foun	Peak Foun	Peak Foun	Peak Foun	Peak Foun
118.4	High	High	High	Peak Foun	Peak Foun	Peak Foun	Peak Foun	Peak Foun
117.8	Peak Foun	High	Peak Foun	Peak Foun	Not Found	Not Found	Peak Foun	Peak Foun
63.9	Peak Foun	Peak Foun	Peak Foun	Not Found	Peak Foun	Peak Foun	Peak Foun	Not Found
117.5	Peak Foun	High	Peak Foun	Peak Foun	Peak Foun	Peak Foun	Not Found	Not Found
55.4	High	Peak Foun	Peak Foun	Peak Foun	High	Peak Foun	Peak Foun	Peak Foun

107.1	Peak Foun	Peak Foun	Peak Foun	Not Found	Peak Foun	Peak Foun	Peak Foun	Peak Foun
110.2	Peak Foun	High	Peak Foun	Not Found	Peak Foun	Peak Foun	Peak Foun	Peak Foun
109.1	Peak Foun	Peak Foun	High	Peak Foun	Peak Foun	High	High	High
95.7	Peak Foun	Not Found	High	Peak Foun	Not Found	Not Found	Not Found	Not Found
126.3	Peak Foun	Peak Foun	Peak Foun	Peak Foun	Not Found	Peak Foun	Peak Foun	Peak Foun
118.7	Peak Foun	Peak Foun	High	Peak Foun	Peak Foun	Peak Foun	Peak Foun	Peak Foun
112.8	Peak Foun	Peak Foun	Not Found	Peak Foun	High	Peak Foun	Peak Foun	Peak Foun
118.1	Peak Foun	Peak Foun	High	Peak Foun	Not Found	Peak Foun	Peak Foun	Peak Foun
117.3	Peak Foun	Peak Foun	Peak Foun	Peak Foun	Peak Foun	Peak Foun	Peak Foun	Peak Foun
75.6	Peak Foun	Peak Foun	Peak Foun	Peak Foun	Peak Foun	Peak Foun	Peak Foun	Peak Foun
110.7	Peak Foun	Not Found	High	Peak Foun	Not Found	Not Found	Not Found	Peak Foun
98.6	Peak Foun	Peak Foun	Peak Foun	Not Found	Not Found	Peak Foun	Not Found	Not Found
105.9	Peak Foun	Not Found	Peak Foun	Peak Foun	Not Found	Peak Foun	Peak Foun	Peak Foun
108.6	Peak Foun	Peak Foun	Peak Foun	Peak Foun	Peak Foun	Peak Foun	Peak Foun	Peak Foun
106.1	Peak Foun	High	High	Peak Foun	Not Found	Not Found	Peak Foun	Peak Foun
94.4	Peak Foun	Peak Foun	Peak Foun	Peak Foun	Not Found	Peak Foun	Peak Foun	Peak Foun
126.6	Peak Foun	Peak Foun	Peak Foun	Peak Foun	Not Found	Not Found	Not Found	Not Found
119.1	Peak Foun	High	High	Peak Foun	Peak Foun	High	Peak Foun	High
92.1	Not Found	Peak Foun	Peak Foun	Not Found	Not Found	Peak Foun	Peak Foun	Peak Foun
106.9	Peak Foun	Peak Foun	High	Not Found	Not Found	Not Found	Not Found	Not Found
124.7	Peak Foun	High	Peak Foun	Peak Foun	Peak Foun	Peak Foun	Peak Foun	Peak Foun
119	Peak Foun	Peak Foun	High	Peak Foun	Not Found	Not Found	Not Found	Peak Foun
101.2	Peak Foun	Peak Foun	Not Found	Not Found	Peak Foun	Peak Foun	Peak Foun	Peak Foun
108.6	Peak Foun	Peak Foun	Peak Foun	Peak Foun	Peak Foun	Peak Foun	Peak Foun	Peak Foun
126.6	Peak Foun	Peak Foun	Peak Foun	Peak Foun	Peak Foun	Peak Foun	Peak Foun	Peak Foun
86.1	Peak Foun	Peak Foun	High	Peak Foun	Not Found	Peak Foun	Peak Foun	Peak Foun
96.1	Peak Foun	Peak Foun	Peak Foun	Peak Foun	Peak Foun	High	Peak Foun	Peak Foun
67.5	Peak Foun	Peak Foun	Peak Foun	Peak Foun	Peak Foun	High	Peak Foun	Peak Foun
129.8	Peak Foun	High	Peak Foun	High	Peak Foun	Peak Foun	Peak Foun	Peak Foun
105	Not Found	High	Not Found	Peak Foun	Not Found	Not Found	Peak Foun	Peak Foun
	Peak Foun	Peak Foun	High	Not Found	Not Found	Not Found	Not Found	Not Found
97.2	Peak Foun	Peak Foun	Peak Foun	Peak Foun	Not Found	Not Found	Not Found	Peak Foun
107.9	High	High	Not Found	Not Found	Peak Foun	Peak Foun	Not Found	Peak Foun
91	Peak Foun	Peak Foun	High	Peak Foun	Not Found	Peak Foun	Peak Foun	Peak Foun
117.6	Peak Foun	Peak Foun	High	Peak Foun	Not Found	Not Found	Peak Foun	Peak Foun
139.7	Peak Foun	Peak Foun	High	Peak Foun	Peak Foun	Peak Foun	Peak Foun	Peak Foun
97.1	Peak Foun	High	Peak Foun	Peak Foun	Peak Foun	Peak Foun	Peak Foun	Peak Foun
81.2	Peak Foun	Peak Foun	Peak Foun	Peak Foun	High	Peak Foun	Peak Foun	High
130.9	High	Peak Foun	Peak Foun	Peak Foun	Not Found	Peak Foun	Peak Foun	Peak Foun
91	High	Peak Foun	Peak Foun	Peak Foun	Peak Foun	Peak Foun	Peak Foun	Peak Foun
125.2	Peak Foun	Peak Foun	Peak Foun	Peak Foun	Not Found	Peak Foun	Peak Foun	Peak Foun
110.5	Peak Foun	Peak Foun	Peak Foun	Peak Foun	Peak Foun	Peak Foun	Peak Foun	Peak Foun
	High	Peak Foun	High	Not Found	Not Found	Not Found	Not Found	Not Found
115.7	Peak Foun	Peak Foun	Peak Foun	Peak Foun	Not Found	Peak Foun	Peak Foun	Peak Foun
84.1	Peak Foun	Peak Foun	Not Found	Not Found	Peak Foun	Peak Foun	Peak Foun	Peak Foun

91.6	Peak Foun	Peak Foun	Peak Foun	Peak Foun	Peak Foun	Peak Foun	Peak Foun	High
107.1	Peak Foun	Peak Foun	High	Not Found	Peak Foun	Peak Foun	Peak Foun	Not Found
89.9	Peak Foun	Peak Foun	Peak Foun	Peak Foun	Peak Foun	High	Peak Foun	Peak Foun
121.1	Peak Foun	Peak Foun	Peak Foun	Not Found	Not Found	Not Found	Peak Foun	Not Found
103.7	Peak Foun	Peak Foun	Peak Foun	Peak Foun	High	Peak Foun	Peak Foun	High
112.9	Peak Foun	Peak Foun	Peak Foun	Peak Foun	Peak Foun	Peak Foun	Peak Foun	Peak Foun
97.9	Peak Foun	High	Peak Foun	Peak Foun	Not Found	Peak Foun	Peak Foun	Peak Foun
	Not Found	Peak Foun	High	Peak Foun	Peak Foun	Not Found	Not Found	Not Found
108.3	Not Found	Peak Foun	Peak Foun	Not Found	Not Found	Not Found	Peak Foun	Not Found
117.4	High	Peak Foun	Peak Foun	Peak Foun	Peak Foun	Peak Foun	Peak Foun	Peak Foun
122.7	Peak Foun	Peak Foun	Peak Foun	Not Found	Peak Foun	Peak Foun	Peak Foun	Not Found
117.2	High	Peak Foun	Peak Foun	High	Peak Foun	Peak Foun	High	High
97.4	Peak Foun	Peak Foun	High	Peak Foun	Peak Foun	Peak Foun	Peak Foun	Peak Foun
108.8	Peak Foun	Peak Foun	Peak Foun	Peak Foun	Not Found	Not Found	Not Found	Peak Foun
86.4	Peak Foun	Peak Foun	Not Found	Not Found	Not Found	Peak Foun	Not Found	Not Found
111.3	Peak Foun	High	Peak Foun	High	Peak Foun	Peak Foun	Peak Foun	Peak Foun
84.9	Not Found	Not Found	Peak Foun	Not Found	Peak Foun	Peak Foun	Peak Foun	Not Found
86.3	Peak Foun	Peak Foun	Peak Foun	Peak Foun	Peak Foun	High	Peak Foun	Peak Foun
127.8	Peak Foun	High	High	Peak Foun	Not Found	Not Found	Not Found	Peak Foun
101.1	Peak Foun	Peak Foun	Peak Foun	Peak Foun	Peak Foun	High	Peak Foun	Peak Foun
116.2	Peak Foun	Peak Foun	Peak Foun	Peak Foun	Peak Foun	High	High	High
117.3	High	High	High	High	High	High	High	High
90.1	Peak Foun	High	Peak Foun	Peak Foun	Peak Foun	High	High	Peak Foun
97.9	High	High	High	Peak Foun	Not Found	High	Peak Foun	Peak Foun
	Not Found	High	Not Found	Not Found	Not Found	Not Found	Not Found	Not Found
107.9	Peak Foun	High	High	Peak Foun	Peak Foun	Peak Foun	Peak Foun	Peak Foun
115.1	Peak Foun	Peak Foun	Peak Foun	Peak Foun	Not Found	Not Found	Not Found	Peak Foun
112	High	Peak Foun	High	Peak Foun	High	Peak Foun	High	Peak Foun
75.6	Not Found	Not Found	Not Found	Peak Foun	Peak Foun	Not Found	Not Found	High
110	High	High	High	Peak Foun	Not Found	Peak Foun	Peak Foun	High
115.8	Peak Foun	Peak Foun	High	Peak Foun	Peak Foun	Peak Foun	High	Peak Foun
101	Peak Foun	Peak Foun	Not Found	Not Found	Not Found	Peak Foun	Peak Foun	Peak Foun
94.3	Peak Foun	Peak Foun	High	High	Peak Foun	Peak Foun	Peak Foun	Peak Foun
95.3	Peak Foun	Peak Foun	Peak Foun	Peak Foun	Peak Foun	Not Found	Peak Foun	Peak Foun
101.5	Peak Foun	High	High	Peak Foun	Not Found	Not Found	Not Found	Peak Foun
114.1	High	High	Not Found	Peak Foun	Not Found	Peak Foun	Not Found	Peak Foun
103.3	Peak Foun	Peak Foun	Peak Foun	Peak Foun	Peak Foun	Peak Foun	Not Found	Peak Foun
108	Peak Foun	Peak Foun	Peak Foun	Peak Foun	Peak Foun	High	Peak Foun	Peak Foun
82.3	High	Peak Foun	Peak Foun	Peak Foun	Peak Foun	Peak Foun	High	High
119.1	Peak Foun	Peak Foun	Peak Foun	Peak Foun	Peak Foun	Peak Foun	Peak Foun	High
143.3	Not Found	Not Found	Not Found	Not Found	Peak Foun	Not Found	Not Found	Not Found
	Not Found	Not Found	Peak Foun	Not Found	Not Found	Peak Foun	Peak Foun	Not Found
109	Not Found	Not Found	Not Found	Not Found	Not Found	High	Not Found	Peak Foun
106.1	Peak Foun	Peak Foun	High	Peak Foun	Peak Foun	Peak Foun	Peak Foun	Peak Foun
106.9	Peak Foun	Peak Foun	Peak Foun	Peak Foun	Not Found	Peak Foun	Peak Foun	High

101	Peak Foun	Peak Foun	High	Not Found	Peak Foun	Peak Foun	Peak Foun	Peak Foun
123.4	Peak Foun	Not Found	Peak Foun	Not Found	Not Found	Not Found	Not Found	Not Found
114.4	Peak Foun	Peak Foun	Peak Foun	Peak Foun	Peak Foun	Not Found	Not Found	Not Found
77.3	Peak Foun	Peak Foun	Peak Foun	Peak Foun	Peak Foun	Peak Foun	High	High
94.5	Peak Foun	Peak Foun	Peak Foun	Peak Foun	Not Found	Peak Foun	Peak Foun	High
83.6	Peak Foun	Peak Foun	Peak Foun	Peak Foun	Peak Foun	High	Peak Foun	Peak Foun
97.2	Peak Foun	Not Found	High	Not Found	Not Found	Not Found	Peak Foun	Not Found
200	Not Found	Not Found	Not Found	Not Found	Not Found	Not Found	Not Found	Not Found
63	High	High	Peak Foun	Peak Foun	Peak Foun	Peak Foun	Peak Foun	Peak Foun
97.9	Peak Foun	Peak Foun	Peak Foun	Peak Foun	Peak Foun	Peak Foun	Peak Foun	Peak Foun
	Peak Foun	Peak Foun	Peak Foun	Peak Foun	High	Peak Foun	Peak Foun	Not Found
101.5	Peak Foun	Peak Foun	Peak Foun	Peak Foun	Peak Foun	Peak Foun	Peak Foun	High
107.7	Peak Foun	Peak Foun	Peak Foun	Peak Foun	Not Found	Peak Foun	Peak Foun	High
72.9	Not Found	Peak Foun	Not Found	Not Found	Peak Foun	Not Found	Not Found	Not Found
17.4	Peak Foun	Peak Foun	High	Peak Foun	High	High	Peak Foun	Peak Foun
100.7	Not Found	Not Found	Not Found	Not Found	Not Found	Peak Foun	Peak Foun	Not Found
103.8	Peak Foun	Peak Foun	Peak Foun	High	Peak Foun	Peak Foun	Peak Foun	Peak Foun
100.7	High	Peak Foun	Peak Foun	Peak Foun	Not Found	Not Found	Not Found	Not Found
111.8	Peak Foun	Peak Foun	Peak Foun	Peak Foun	Peak Foun	Peak Foun	Peak Foun	Peak Foun
96.9	Peak Foun	Peak Foun	Peak Foun	Not Found	Not Found	Not Found	Not Found	Peak Foun
137.9	High	High	Peak Foun	Peak Foun	Not Found	Peak Foun	Not Found	Peak Foun
103.6	Peak Foun	Peak Foun	High	Peak Foun	Not Found	Not Found	Not Found	Peak Foun
81.5	Not Found	Peak Foun	Peak Foun	Peak Foun	Peak Foun	Peak Foun	Not Found	High
	High	Peak Foun	Peak Foun	Peak Foun	Not Found	Not Found	Not Found	Peak Foun
120.9	High	High	Peak Foun	Peak Foun	Peak Foun	Peak Foun	Peak Foun	Peak Foun
114.7	Peak Foun	Peak Foun	Peak Foun	Peak Foun	Peak Foun	Peak Foun	Peak Foun	Peak Foun
104.5	High	Peak Foun	Not Found	Peak Foun	Peak Foun	Peak Foun	Not Found	Not Found
124	High	Peak Foun	Peak Foun	Peak Foun	Not Found	Peak Foun	Not Found	Not Found
78.2	Peak Foun	Peak Foun	High	High	Not Found	High	High	High
92.5	Peak Foun	High	Peak Foun	Peak Foun	Peak Foun	Peak Foun	Peak Foun	Peak Foun
109.2	Peak Foun	Peak Foun	Peak Foun	Not Found	Peak Foun	Peak Foun	Peak Foun	Peak Foun
34.3	Peak Foun	Peak Foun	Peak Foun	Peak Foun	Not Found	Peak Foun	Peak Foun	Peak Foun
122.8	Peak Foun	High	High	Peak Foun	Peak Foun	Peak Foun	Peak Foun	Peak Foun
	Peak Foun	Peak Foun	Peak Foun	Peak Foun	High	Peak Foun	Peak Foun	Not Found
140.2	Not Found	Not Found	Not Found	Not Found	Not Found	Not Found	Not Found	Peak Foun
95.9	Not Found	Peak Foun	Peak Foun	Not Found	High	Peak Foun	Not Found	Not Found
104.5	Not Found	High	Peak Foun	Peak Foun	Peak Foun	Peak Foun	Peak Foun	Peak Foun
	Peak Foun	High	Peak Foun	Peak Foun	Not Found	Not Found	Not Found	Peak Foun
123.9	Peak Foun	Peak Foun	Not Found	Not Found	Peak Foun	Peak Foun	Peak Foun	Peak Foun
42.9	Not Found	Peak Foun	Peak Foun	Peak Foun	Not Found	Peak Foun	Peak Foun	Peak Foun
43.7	Peak Foun	Peak Foun	Not Found	Peak Foun	Not Found	High	Not Found	Not Found
102.3	High	High	High	High	Peak Foun	Peak Foun	Peak Foun	Peak Foun
106.4	Peak Foun	Peak Foun	High	Peak Foun	Peak Foun	Peak Foun	Peak Foun	Peak Foun
100.7	Peak Foun	Peak Foun	Peak Foun	Peak Foun	Peak Foun	Peak Foun	Peak Foun	Peak Foun
92	High	Peak Foun	High	Peak Foun	Peak Foun	Peak Foun	Peak Foun	Peak Foun

113.9	High	Peak Foun	Peak Foun	Peak Foun	Peak Foun	Peak Foun	Peak Foun	Peak Foun
95.8	High	Peak Foun	Peak Foun	Peak Foun	Peak Foun	Peak Foun	Peak Foun	Peak Foun
	Peak Foun	Not Found	Peak Foun	Peak Foun	Not Found	Not Found	Peak Foun	Not Found
118.1	Peak Foun	Peak Foun	High	Peak Foun	Peak Foun	Peak Foun	Peak Foun	Peak Foun
117.6	High	Peak Foun	Peak Foun	Not Found	Not Found	Peak Foun	Not Found	Not Found
113.9	High	High	High	Peak Foun	Peak Foun	Peak Foun	Not Found	Peak Foun
119	Peak Foun	Peak Foun	High	Peak Foun	Peak Foun	Peak Foun	Peak Foun	Peak Foun
123.1	Peak Foun	High	High	Peak Foun	Not Found	Not Found	Not Found	Not Found
103.2	High	High	Peak Foun	Peak Foun	Peak Foun	Peak Foun	Peak Foun	Peak Foun
88.8	High	Peak Foun	Peak Foun	Peak Foun	Peak Foun	Peak Foun	Peak Foun	Peak Foun
116.7	Not Found	Not Found	Not Found	Peak Foun	Not Found	Peak Foun	Peak Foun	High
149.6	Peak Foun	Peak Foun	Not Found	Peak Foun	Peak Foun	Peak Foun	Peak Foun	Peak Foun
108.9	High	High	Peak Foun	Peak Foun	High	High	High	High
114.8	Peak Foun	Peak Foun	High	Peak Foun	Not Found	Peak Foun	High	High
154.8	Peak Foun	High	Peak Foun	Peak Foun	Peak Foun	Peak Foun	Peak Foun	Peak Foun
109	Peak Foun	High	Peak Foun	Peak Foun	Peak Foun	Peak Foun	Peak Foun	Peak Foun
	Not Found	Not Found	Not Found	Not Found	High	Peak Foun	Peak Foun	Not Found
200	Not Found	Not Found	Not Found	Not Found	Not Found	Not Found	Not Found	Not Found
96.9	Peak Foun	High	High	Peak Foun	Peak Foun	Not Found	Peak Foun	Peak Foun
99.7	Peak Foun	Peak Foun	High	Peak Foun	Peak Foun	Peak Foun	High	Peak Foun
123.7	High	Peak Foun	Peak Foun	Peak Foun	Not Found	Peak Foun	Peak Foun	Peak Foun
99.8	Peak Foun	Not Found	Peak Foun	Peak Foun	Peak Foun	Not Found	Peak Foun	Peak Foun
125.2	Peak Foun	High	Peak Foun	Peak Foun	Not Found	Not Found	Peak Foun	Peak Foun
	Not Found	High	Not Found	Not Found	Not Found	Not Found	Not Found	Not Found
99.6	Peak Foun	High	Peak Foun	Peak Foun	Peak Foun	Peak Foun	Peak Foun	Peak Foun
115.5	Not Found	Peak Foun	Peak Foun	Not Found	Not Found	Not Found	Peak Foun	Not Found
92.8	Peak Foun	Peak Foun	Peak Foun	Not Found	Peak Foun	Peak Foun	Peak Foun	Peak Foun
145.8	Peak Foun	Peak Foun	Peak Foun	Not Found	Peak Foun	Not Found	Peak Foun	Peak Foun
117.1	Not Found	Peak Foun	High	Not Found	Not Found	Peak Foun	Peak Foun	Peak Foun
109.2	Peak Foun	High	Peak Foun	Not Found	Not Found	Peak Foun	Peak Foun	Peak Foun
	Peak Foun	Peak Foun	Peak Foun	Peak Foun	Not Found	Peak Foun	High	Peak Foun
177.9	Not Found	Not Found	Not Found	Peak Foun	Not Found	Not Found	Peak Foun	Peak Foun
137.9	Peak Foun	High	Peak Foun	Peak Foun	Peak Foun	Peak Foun	Peak Foun	Peak Foun
106.5	Peak Foun	Peak Foun	Peak Foun	Peak Foun	Not Found	Not Found	Not Found	Peak Foun
140.9	Not Found	Peak Foun	Peak Foun	Not Found	Not Found	Not Found	Not Found	Not Found
98.3	High	High	High	Peak Foun	Peak Foun	Peak Foun	Peak Foun	Peak Foun
63.4	Not Found	Not Found	Peak Foun	Peak Foun	Peak Foun	Peak Foun	Peak Foun	High
102.4	Peak Foun	Peak Foun	Peak Foun	Peak Foun	Peak Foun	Peak Foun	Peak Foun	Peak Foun
113.4	Peak Foun	Peak Foun	Peak Foun	Peak Foun	High	Peak Foun	Peak Foun	Not Found
100.6	High	Peak Foun	Peak Foun	Peak Foun	Peak Foun	Not Found	Peak Foun	Peak Foun
134.9	Not Found	Not Found	High	Peak Foun	Peak Foun	Peak Foun	Peak Foun	Peak Foun
89.7	Peak Foun	Peak Foun	Peak Foun	Peak Foun	Not Found	High	Peak Foun	Not Found
88.7	Peak Foun	Peak Foun	High	Not Found	Not Found	Peak Foun	Peak Foun	Peak Foun
124.8	Peak Foun	High	Peak Foun	Peak Foun	Peak Foun	Peak Foun	Peak Foun	Peak Foun
103.2	Peak Foun	Peak Foun	Peak Foun	Not Found	Not Found	Peak Foun	Not Found	Not Found

106.2	Peak Foun	Not Found	Peak Foun	Peak Foun	Peak Foun	Peak Foun	Peak Foun	High
92.1	Peak Foun	Peak Foun	High	Peak Foun	Not Found	Peak Foun	Peak Foun	Peak Foun
95.1	High	Peak Foun	Peak Foun	Peak Foun	Peak Foun	Peak Foun	Peak Foun	Peak Foun
101.6	Peak Foun	Peak Foun	Peak Foun	Peak Foun	Peak Foun	Peak Foun	Peak Foun	Peak Foun
85.6	Peak Foun	Peak Foun	Peak Foun	Peak Foun	Peak Foun	Peak Foun	Peak Foun	Peak Foun
85.3	Peak Foun	Peak Foun	Not Found	Peak Foun	Peak Foun	Peak Foun	Peak Foun	Not Found

Found in S	Found in S	Found in S	Found in S	Found in S	Found in S	Found in S	Found in S	# Protein (
High	High	High	High	High	High	High	High	1
High	High	High	High	High	High	High	High	1
High	High	High	High	High	High	High	High	1
High	High	High	High	High	High	High	High	1
High	Peak Foun	High	High	High	High	High	High	1
Peak Foun	High	High	High	High	High	High	High	1
Not Found	High	High	High	High	High	High	High	1
High	High	High	High	High	High	High	High	1
High	High	High	High	High	High	High	High	1
High	High	High	High	High	High	High	High	1
High	High	High	High	High	High	High	High	1
Peak Foun	Peak Foun	Peak Foun	High	High	High	High	High	1
High	High	High	High	High	High	High	High	1
High	High	High	High	High	High	High	High	1
High	High	High	High	High	High	High	High	1
High	High	High	High	High	High	High	High	1
High	High	High	High	High	High	High	High	1
High	High	High	High	High	High	High	High	1
High	High	High	High	High	High	High	High	1
High	High	High	High	High	High	High	High	1
High	High	High	High	High	High	High	High	1
High	High	High	High	High	High	High	High	1
Peak Foun	High	High	Peak Foun	Peak Foun	High	High	High	1
High	High	High	High	High	High	High	High	1
High	High	High	High	High	High	High	High	1
High	High	High	High	High	High	High	High	1
Peak Foun	High	High	High	High	High	High	High	1
Peak Foun	Peak Foun	Peak Foun	High	High	High	Peak Foun	Peak Foun	1
High	High	High	High	High	High	High	High	1
High	High	High	High	High	High	High	High	1
Peak Foun	High	High	Peak Foun	High	High	High	Peak Foun	1
High	High	High	High	High	High	High	High	1
Peak Foun	High	High	High	High	High	High	High	1
High	High	High	High	High	High	High	High	1
High	High	High	High	High	High	High	High	1
High	High	High	High	High	High	High	High	1
High	High	High	High	High	High	High	High	1
High	High	High	High	High	High	High	High	1
High	High	Peak Foun	High	High	High	High	High	1
High	High	High	High	High	High	High	High	1
High	High	High	High	High	High	High	High	1
High	High	High	High	High	High	High	High	1

High	High	High	High	High	High	High	High	1
Peak Foun	High	High	High	High	High	High	High	1
Peak Foun	High	High	High	High	High	High	High	1
High	High	High	High	High	High	High	High	1
High	High	High	High	High	High	High	High	1
High	High	High	High	High	High	High	High	1
Not Found	High	High	Peak Foun	High	High	High	High	1
High	High	High	High	High	High	High	High	1
High	High	High	High	High	High	High	High	1
High	High	High	High	High	High	High	High	1
High	High	High	High	High	High	High	High	1
High	High	High	High	High	High	High	High	1
High	High	High	High	High	High	High	High	1
Peak Foun	High	High	High	High	High	High	High	1
High	High	High	High	High	High	High	High	1
Peak Foun	High	High	Peak Foun	Peak Foun	High	Peak Foun	Peak Foun	1
High	High	High	High	High	High	High	High	1
Peak Foun	High	Peak Foun	High	Peak Foun	Peak Foun	Peak Foun	Peak Foun	1
High	High	High	High	High	High	High	High	1
High	High	High	High	High	High	High	High	1
High	High	High	High	High	High	High	High	1
High	High	High	High	High	High	High	High	1
High	High	High	High	High	High	High	High	1
High	High	High	High	High	High	High	High	1
High	High	High	High	High	High	High	High	1
Peak Foun	High	High	High	High	High	High	High	1
Peak Foun	High	High	High	High	High	High	High	1
High	High	High	High	High	High	High	High	1
High	High	High	High	High	High	High	High	1
High	High	High	High	High	High	High	High	1
High	High	High	High	High	High	High	High	1
High	High	High	High	High	High	High	High	1
High	High	High	High	High	High	High	High	1
High	High	High	High	High	High	High	High	1
Not Found	Not Found	Not Found	Not Found	Not Found	Not Found	Not Found	Not Found	1
Peak Foun	Peak Foun	Peak Foun	Peak Foun	High	High	High	High	1
Peak Foun	Peak Foun	High	Peak Foun	High	High	High	High	1
High	High	High	High	High	High	High	High	1
High	High	High	High	High	High	High	High	1
High	High	High	High	High	High	High	High	1
Not Found	Peak Foun	Peak Foun	Peak Foun	Peak Foun	High	High	High	1
High	High	High	High	High	High	High	High	1
High	High	High	High	High	High	High	High	1
High	High	High	High	High	High	High	High	1
High	High	High	High	High	High	High	High	1

High	High	High	High	High	High	High	High	1
Peak Foun	Peak Foun	Peak Foun	High	High	High	High	High	1
High	High	High	High	High	High	High	High	1
High	High	High	High	High	High	High	High	1
Peak Foun	Peak Foun	Not Found	High	Peak Foun	Peak Foun	Peak Foun	High	1
High	High	High	High	High	High	High	High	1
High	High	High	High	High	High	High	High	1
High	High	High	High	High	High	High	High	1
High	High	High	High	High	High	High	High	1
Peak Foun	Peak Foun	Peak Foun	Peak Foun	High	High	High	High	1
High	High	High	High	High	High	High	High	1
Peak Foun	High	High	High	High	High	High	High	1
High	High	High	High	High	High	High	High	1
Peak Foun	Peak Foun	Peak Foun	Peak Foun	Peak Foun	High	Peak Foun	Peak Foun	1
High	High	High	High	High	High	High	High	1
Peak Foun	High	High	High	High	High	High	High	1
High	High	High	High	High	High	High	High	1
High	High	High	High	High	High	High	High	1
High	High	High	High	High	High	High	High	1
High	High	High	High	High	High	High	High	1
High	High	High	High	High	High	High	High	1
Not Found	Not Found	Peak Foun	Not Found	Not Found	Peak Foun	High	Not Found	1
High	High	High	High	High	High	High	High	1
High	High	High	High	High	High	High	High	1
High	High	High	High	High	High	Peak Foun	High	1
High	High	High	High	High	High	High	High	1
High	High	High	High	High	High	High	High	1
High	High	High	High	High	High	High	High	1
High	High	High	High	High	High	High	High	1
High	High	High	High	High	High	High	High	1
High	High	High	High	High	High	High	High	1
High	High	High	High	High	High	High	High	1
High	High	High	High	High	High	High	High	1
High	High	High	High	High	High	High	High	1
High	High	High	High	High	High	High	High	1
Peak Foun	Peak Foun	Peak Foun	High	High	High	High	High	1
High	High	High	High	High	High	High	High	1
High	High	High	High	High	High	High	High	1
High	High	High	High	High	High	High	High	1
High	High	High	High	High	High	High	High	1
High	High	High	High	High	High	High	High	1
High	High	High	High	High	High	High	High	1
High	High	High	High	High	High	High	High	1
High	High	High	High	High	High	High	High	1
High	High	High	Peak Foun	High	High	High	High	1

High	High	High	High	High	High	High	High	1
High	High	High	High	High	High	High	High	1
High	High	High	High	High	High	High	High	1
Not Found	Not Found	Not Found	Not Found	Not Found	Not Found	Not Found	Not Found	1
High	High	High	High	High	High	High	High	1
High	High	High	High	High	High	High	High	1
High	High	High	High	High	High	High	High	1
High	High	High	High	High	High	High	High	1
High	High	High	High	High	High	High	High	1
High	High	High	High	High	High	High	High	1
High	High	High	High	High	High	High	High	1
High	High	High	High	High	High	High	High	1
High	High	High	High	High	High	High	High	1
Not Found	High	High	High	High	High	High	High	1
High	High	High	High	High	High	High	High	1
High	High	High	High	High	High	High	High	1
High	High	High	High	High	High	High	High	1
Peak Foun	Not Found	Not Found	Not Found	High	High	High	High	1
High	High	High	High	High	High	High	High	1
High	High	High	High	High	High	High	High	1
High	High	High	High	High	High	High	High	1
High	High	High	High	High	High	High	High	1
High	High	High	High	High	High	High	High	1
Peak Foun	Not Found	Peak Foun	High	High	High	High	High	1
High	High	High	High	High	High	High	High	1
Peak Foun	Peak Foun	High	High	High	High	High	High	1
High	High	High	High	High	High	High	High	1
Peak Foun	High	High	High	High	High	High	High	1
High	High	High	High	High	High	High	High	1
High	High	High	High	High	High	High	High	1
Not Found	Not Found	Not Found	Not Found	High	High	High	High	1
High	High	High	High	High	High	High	High	1
High	High	High	High	High	High	High	High	1
High	High	High	High	High	High	High	High	1
Peak Foun	High	High	Not Found	Peak Foun	Not Found	Peak Foun	Not Found	1
High	High	High	High	High	High	High	High	1
High	High	High	High	High	High	High	High	1
High	High	High	High	High	High	High	High	1
High	High	High	High	High	High	High	High	1
High	High	High	High	High	High	High	High	1
High	High	High	High	High	High	High	High	1
High	High	High	High	High	High	High	High	1
Peak Foun	Peak Foun	High	High	High	High	High	High	1
High	High	High	High	High	High	High	High	1
High	High	Peak Foun	High	High	High	High	High	1

High	High	High	High	High	High	High	High	1
High	High	High	High	High	High	High	High	1
High	High	High	High	High	High	High	High	1
High	High	High	High	High	High	High	High	1
High	High	High	High	High	High	High	High	1
High	High	High	High	High	High	High	High	1
High	High	High	High	High	High	High	High	1
High	High	High	High	High	High	High	High	1
High	High	High	High	High	High	High	High	1
High	High	High	High	High	High	High	High	1
Peak Foun	High	High	High	High	High	High	High	1
High	High	High	High	High	High	High	High	1
High	High	High	High	High	High	High	High	1
High	High	High	High	High	High	High	High	1
High	High	High	High	High	High	High	High	1
Peak Foun	Peak Foun	Peak Foun	High	High	High	High	High	1
High	High	High	High	High	High	High	High	1
Peak Foun	Peak Foun	Peak Foun	Peak Foun	High	High	High	High	1
High	High	High	High	High	High	High	High	1
High	High	High	High	High	High	High	High	1
High	High	High	High	High	High	High	High	1
Peak Foun	Peak Foun	High	Peak Foun	High	High	High	High	1
High	High	High	High	High	High	High	High	1
High	High	High	High	High	High	High	High	1
Peak Foun	High	High	High	High	High	High	High	1
High	High	High	High	High	High	High	High	1
High	High	High	High	High	High	High	High	1
High	Peak Foun	High	Peak Foun	Peak Foun	Peak Foun	Peak Foun	Peak Foun	1
Not Found	Peak Foun	Peak Foun	Peak Foun	Peak Foun	High	High	High	1
High	High	High	High	High	High	High	High	1
High	High	High	High	High	High	High	High	1
Peak Foun	High	High	High	High	High	High	High	1
High	High	High	High	High	High	High	High	1
Not Found	Not Found	Not Found	Not Found	Peak Foun	High	High	Not Found	1
High	High	High	High	High	High	High	High	1
High	High	High	High	High	High	High	High	1
High	High	High	High	High	High	High	High	1
High	High	High	High	High	High	High	High	1
High	High	High	High	High	High	High	High	1
High	High	High	High	High	High	High	High	1
High	High	High	High	High	High	High	High	1
Peak Foun	High	Peak Foun	Peak Foun	High	High	High	High	1
High	High	High	High	High	High	High	High	1
High	High	High	High	High	High	High	High	1
High	High	High	High	High	High	High	High	1

High	High	High	High	High	High	High	High	1
High	High	High	High	High	High	High	High	1
High	High	High	High	High	High	High	High	1
Peak Foun	High	High	High	High	High	High	High	1
High	High	High	High	High	High	High	High	1
High	High	High	High	High	High	High	High	1
High	High	High	High	High	High	High	High	1
High	High	High	High	High	High	High	High	1
High	High	High	High	High	High	High	High	1
High	High	High	High	High	High	High	High	1
Peak Foun	High	High	High	High	High	High	High	1
High	High	High	High	High	High	High	High	1
High	High	High	High	High	High	High	High	1
High	High	High	High	High	High	High	High	1
High	High	High	High	High	High	High	High	1
High	High	High	High	High	High	High	High	1
High	High	High	High	High	High	High	High	1
High	High	High	High	High	High	High	High	1
High	High	High	High	High	High	High	High	1
High	High	High	High	High	High	High	High	1
High	High	High	High	High	High	High	High	1
High	High	High	High	High	High	High	High	1
High	High	High	High	High	High	High	High	1
Peak Foun	Peak Foun	Peak Foun	High	High	High	High	High	1
High	High	High	High	High	High	High	High	1
Peak Foun	High	High	High	Peak Foun	Peak Foun	High	High	1
High	High	High	High	High	High	High	High	1
High	High	High	High	High	High	High	High	1
High	High	High	High	High	High	High	High	1
High	High	High	High	High	High	High	High	1
High	High	High	High	High	High	High	High	1
Peak Foun	Peak Foun	High	High	High	High	High	High	1
High	High	High	High	High	High	High	High	1
High	High	High	High	High	High	High	High	1
High	High	High	High	High	High	High	High	1
Peak Foun	High	High	High	High	High	High	High	1
High	High	High	High	High	High	High	High	1
High	High	High	High	High	High	High	High	1
High	High	High	High	High	High	High	High	1
High	High	High	High	High	High	High	High	1
High	High	High	High	High	High	High	High	1
High	High	High	High	High	High	High	High	1
Peak Foun	High	High	High	High	High	High	High	1
High	High	High	High	High	High	High	High	1
High	High	High	High	High	High	High	High	1

High	High	High	High	High	High	High	High	1
High	High	High	High	High	High	High	High	1
Peak Foun	Peak Foun	High	High	High	High	High	High	1
High	High	High	High	High	High	High	High	1
High	High	High	High	High	High	High	High	1
High	High	High	High	High	High	High	High	1
High	Peak Foun	High	High	High	High	High	High	1
Peak Foun	Peak Foun	Peak Foun	Peak Foun	High	High	High	High	1
High	High	High	High	High	High	High	High	1
High	High	High	High	High	High	High	High	1
High	High	High	High	High	High	High	High	1
High	High	High	High	High	High	High	High	1
High	High	High	High	High	High	High	High	1
High	High	High	High	High	High	High	High	1
High	High	High	High	High	High	High	High	1
High	High	High	High	High	High	High	High	1
High	High	High	High	High	High	High	High	1
High	High	High	High	High	High	High	High	1
High	High	High	High	High	High	High	High	1
High	High	High	High	High	High	High	High	1
High	High	High	High	High	High	High	High	1
High	High	High	High	High	High	High	High	1
High	High	High	High	High	High	High	High	1
High	High	High	High	High	High	High	High	1
High	High	High	High	High	High	High	High	1
Peak Foun	High	High	High	High	High	High	High	1
Not Found	Peak Foun	Not Found	Not Found	Not Found	Not Found	Not Found	Not Found	1
Peak Foun	Peak Foun	Peak Foun	Peak Foun	High	High	High	High	1
Not Found	High	High	High	High	High	High	High	1
High	High	High	High	High	High	High	High	1
High	High	High	High	High	High	High	High	1
High	High	High	High	High	High	High	High	1
Peak Foun	Peak Foun	High	High	High	High	High	High	1
Peak Foun	Peak Foun	High	High	High	High	High	High	1
High	High	High	High	High	High	High	High	1
High	Peak Foun	High	High	High	High	High	High	1
High	High	High	High	High	High	High	High	1
High	High	High	High	High	High	High	High	1
High	High	High	High	High	High	High	High	1
High	High	High	High	High	High	High	High	1
Peak Foun	Peak Foun	High	Peak Foun	High	High	High	High	1
High	High	High	High	High	High	High	High	1
High	High	High	High	High	High	High	High	1
High	High	High	High	Peak Foun	High	High	High	1
High	High	High	High	High	High	High	High	1

Peak Foun	Peak Foun	Peak Foun	High	High	High	High	High	1
High	High	High	High	High	High	High	High	1
High	High	High	High	High	High	High	High	1
High	Peak Foun	Peak Foun	Peak Foun	High	High	High	High	1
High	High	High	High	High	High	High	High	1
High	High	High	High	High	High	High	High	1
High	High	High	High	High	High	High	High	1
High	High	High	High	High	High	High	High	1
Peak Foun	Peak Foun	Peak Foun	High	High	High	High	Peak Foun	1
High	High	High	High	High	High	High	High	1
Peak Foun	Peak Foun	Peak Foun	Peak Foun	High	High	High	High	1
High	High	High	High	High	High	High	High	1
High	High	High	High	High	High	High	High	1
High	High	High	High	High	High	High	High	1
High	High	High	High	High	High	High	High	1
High	High	High	High	High	High	High	High	1
High	High	High	High	High	High	High	High	1
Peak Foun	High	Peak Foun	High	High	High	High	High	1
High	High	High	High	High	High	High	High	1
High	High	High	High	High	High	High	High	1
High	High	High	High	High	High	High	High	1
Not Found	Not Found	Not Found	Peak Foun	Peak Foun	Peak Foun	High	Not Found	1
High	High	High	High	High	High	High	High	1
Peak Foun	Peak Foun	High	Peak Foun	High	High	High	High	1
Peak Foun	High	High	High	High	High	High	High	1
High	High	High	High	High	High	High	High	1
High	High	High	High	High	High	High	High	1
Peak Foun	High	High	High	High	High	High	High	1
High	High	High	High	High	High	High	High	1
High	High	High	High	High	High	High	High	1
High	High	High	High	High	High	High	High	1
High	High	High	High	High	High	High	Peak Foun	1
High	High	High	High	High	High	High	High	1
High	High	High	High	High	High	High	High	1
Peak Foun	High	High	High	High	High	High	High	1
High	High	High	High	High	High	High	High	1
High	High	High	High	High	High	High	High	1
High	High	High	High	High	High	High	High	1
High	High	High	High	High	High	High	High	1
High	Peak Foun	High	High	High	High	High	Peak Foun	1
Peak Foun	High	High	High	High	High	High	High	1
High	High	High	High	High	High	High	High	1
High	High	High	High	High	High	High	High	1
High	High	High	High	High	High	High	High	1
High	High	High	High	High	High	High	High	1

High	High	High	High	High	High	High	High	1
Not Found	Peak Foun	Peak Foun	High	High	High	High	High	1
High	High	High	High	High	High	High	High	1
High	High	High	High	High	High	High	High	1
High	High	High	High	High	High	High	High	1
High	High	High	High	High	High	High	High	1
High	High	High	High	High	High	High	High	1
High	High	High	High	High	High	High	High	1
High	High	High	High	High	High	High	High	1
Peak Foun	Peak Foun	Peak Foun	Peak Foun	High	High	High	High	1
High	High	High	High	High	High	High	High	1
High	High	High	High	High	High	High	High	1
High	High	High	High	High	High	High	High	1
High	High	High	High	High	High	High	High	1
High	High	High	High	High	High	High	High	1
High	High	High	High	High	High	High	High	1
High	High	High	High	High	High	High	High	1
High	High	High	High	High	High	High	High	1
High	High	High	High	High	High	High	High	1
High	High	High	High	High	High	High	High	1
High	High	High	High	High	High	High	High	1
High	High	High	High	High	High	High	High	1
High	High	High	High	High	High	High	High	1
High	High	High	High	High	High	High	High	1
High	High	High	High	High	High	High	High	1
Peak Foun	Peak Foun	Peak Foun	High	High	High	High	High	1
High	High	High	High	High	High	High	High	1
Peak Foun	Peak Foun	Peak Foun	Peak Foun	High	High	High	High	1
High	High	High	High	High	High	High	High	1
High	High	High	High	High	High	High	High	1
Peak Foun	High	High	High	High	High	High	High	1
High	High	High	High	High	High	High	High	1
High	High	High	High	High	High	High	High	1
Peak Foun	High	High	High	High	High	High	High	1
High	High	High	High	High	High	High	High	1
High	High	High	High	High	High	High	High	1
High	High	High	High	High	High	High	High	1
High	High	High	High	High	High	High	High	1
High	High	High	High	High	High	High	High	1
High	High	High	High	High	High	High	High	1
High	High	High	High	High	High	High	High	1
Peak Foun	Not Found	Peak Foun	High	High	High	High	High	1
High	High	High	High	High	High	High	High	1
High	High	High	High	High	High	High	High	1
High	High	High	High	High	High	High	High	1

High	High	High	High	High	High	High	High	1
High	High	High	High	High	High	High	High	1
High	High	High	High	High	High	High	High	1
High	High	High	High	High	High	High	High	1
High	High	High	High	High	High	High	High	1
Peak Foun	Peak Foun	Not Found	Peak Foun	Not Found	High	Not Found	Peak Foun	1
High	High	High	High	High	High	High	High	1
High	High	High	High	High	High	High	High	1
High	High	High	High	High	High	High	High	1
Peak Foun	Not Found	Peak Foun	Peak Foun	High	High	High	High	1
High	High	High	High	High	High	High	High	1
High	High	High	High	High	High	High	High	1
High	High	High	High	High	High	High	High	1
High	High	High	High	High	High	High	High	1
High	High	High	High	High	High	High	High	1
High	High	High	High	High	High	High	High	1
High	High	High	High	High	High	High	High	1
High	High	High	High	High	High	High	High	1
High	High	High	High	High	High	High	High	1
High	High	High	High	High	High	High	High	1
High	High	High	High	High	High	High	High	1
High	High	High	High	High	High	High	High	1
High	High	High	High	High	High	High	High	1
High	High	High	High	High	High	High	High	1
Peak Foun	Peak Foun	Peak Foun	Peak Foun	High	High	High	High	1
High	High	High	High	High	High	High	High	1
Peak Foun	High	High	High	High	High	High	High	1
High	High	High	High	High	High	High	High	1
High	High	High	High	High	High	High	High	1
High	High	High	High	High	High	High	High	1
High	High	High	High	High	High	High	High	1
High	High	High	High	High	High	High	High	1
Peak Foun	Peak Foun	Peak Foun	Not Found	High	High	High	Peak Foun	1
High	High	High	High	High	High	High	High	1
High	Peak Foun	High	High	High	High	High	Peak Foun	1
High	High	High	High	High	High	High	High	1
High	High	High	High	High	High	High	High	1
Peak Foun	Peak Foun	Peak Foun	Peak Foun	High	High	High	Peak Foun	1
High	High	High	High	High	High	High	High	1
High	High	High	High	High	High	High	High	1
High	High	High	High	High	High	High	High	1
High	High	High	High	High	High	High	High	1
Peak Foun	High	High	High	High	High	High	High	1
High	High	High	High	High	High	High	High	1
High	High	High	High	High	High	High	High	1

High	High	High	High	High	High	High	High	1
High	High	High	High	High	High	High	High	1
High	High	High	High	High	High	High	High	1
High	High	High	High	High	High	High	High	1
High	High	High	High	High	High	High	High	1
High	High	High	High	High	High	High	High	1
High	High	High	High	High	High	High	High	1
High	High	High	High	High	High	High	High	1
High	High	High	High	High	High	High	High	1
High	High	High	High	High	High	High	High	1
High	High	High	High	High	High	High	High	1
High	High	High	High	High	High	High	High	1
High	High	High	High	High	High	High	High	1
High	High	High	High	High	High	High	High	1
High	High	High	High	High	High	High	High	1
Peak Foun	Peak Foun	Peak Foun	Peak Foun	High	High	High	High	1
High	High	High	High	High	High	High	High	1
High	High	High	High	High	High	High	High	1
High	High	High	High	High	High	High	High	1
High	High	High	High	High	High	High	High	1
High	High	High	High	High	High	High	High	1
High	High	High	High	High	High	High	High	1
High	High	High	High	High	High	High	High	1
High	High	High	High	High	High	High	High	1
High	High	High	High	High	High	High	High	1
High	High	High	High	High	High	High	High	1
High	High	High	High	High	High	High	High	1
Peak Foun	Peak Foun	High	Peak Foun	High	High	High	High	1
High	High	High	High	High	High	High	High	1
High	High	High	High	High	High	High	Peak Foun	1
High	High	High	High	High	High	High	High	1
High	High	High	High	High	High	High	High	1
High	High	High	High	High	High	High	High	1
High	High	High	High	High	High	High	High	1
Peak Foun	High	High	High	High	High	High	High	1
Peak Foun	High	Peak Foun	Peak Foun	High	High	High	Peak Foun	1
High	High	High	High	High	High	High	High	1
Peak Foun	High	High	High	High	High	High	High	1
High	High	High	High	High	High	High	High	1
High	High	High	High	High	High	High	High	1
High	High	High	High	High	High	High	High	1
High	High	High	High	High	High	High	High	1
Peak Foun	Peak Foun	Peak Foun	High	High	High	High	High	1
High	High	High	High	High	High	High	High	1
High	High	High	High	High	High	High	High	1

High	High	High	High	High	High	High	High	1
High	High	High	High	High	High	High	High	1
High	High	Peak Foun	High	High	High	Peak Foun	Peak Foun	1
Peak Foun	Peak Foun	Peak Foun	High	High	High	High	Peak Foun	1
Peak Foun	High	High	High	High	High	High	High	1
Peak Foun	High	Peak Foun	High	High	High	High	High	1
High	High	High	High	High	High	High	High	1
High	High	High	High	High	High	High	High	1
High	High	High	High	High	High	High	High	1
High	High	High	High	High	High	High	High	1
High	High	High	High	High	High	High	High	1
High	High	High	High	High	High	High	High	1
High	High	High	High	High	High	High	High	1
High	High	High	High	High	High	High	High	1
High	High	High	High	High	High	High	High	1
High	High	High	High	High	High	High	High	1
High	High	High	High	High	High	High	High	1
High	High	High	Peak Foun	Peak Foun	Peak Foun	High	Peak Foun	1
High	High	High	High	High	High	High	High	1
High	High	High	High	High	High	High	High	1
High	High	High	High	High	High	High	High	1
High	High	High	High	High	High	High	High	1
High	High	High	High	High	High	High	High	1
Peak Foun	High	High	High	High	High	High	High	1
Peak Foun	Peak Foun	High	High	High	High	High	High	1
High	High	High	High	High	High	High	High	1
High	High	High	High	High	High	High	High	1
High	High	High	High	High	High	High	High	1
High	High	High	High	High	High	High	High	1
High	High	High	High	High	High	High	High	1
High	High	High	High	Peak Foun	High	High	High	1
High	Peak Foun	Peak Foun	High	High	High	High	High	1
High	High	High	High	High	High	High	High	1
High	High	High	High	High	High	High	High	1
High	High	High	High	High	High	High	High	1
Peak Foun	High	High	High	High	High	High	High	1
Peak Foun	Peak Foun	Peak Foun	Peak Foun	High	High	High	High	1
High	High	High	High	High	High	High	High	1
High	High	High	High	High	High	High	High	1
High	High	High	High	High	High	High	High	1
High	High	High	High	High	High	High	High	1
High	High	High	High	High	High	High	High	1
High	High	High	High	High	High	High	High	1
Peak Foun	Peak Foun	Peak Foun	High	High	High	High	High	1
Peak Foun	Peak Foun	Peak Foun	Peak Foun	High	High	High	High	1

High	High	High	High	High	High	High	High	1
High	High	High	High	High	High	High	High	1
Peak Foun	Peak Foun	Peak Foun	Peak Foun	High	High	High	Peak Foun	1
High	High	High	High	High	High	High	High	1
High	High	High	High	High	High	High	High	1
High	High	High	High	High	High	High	High	1
High	High	High	High	High	High	High	High	1
Peak Foun	High	High	High	High	High	High	High	1
High	High	High	High	High	High	High	High	1
High	High	High	High	High	High	High	High	1
High	High	High	High	High	High	High	High	1
High	High	High	High	High	High	High	High	1
High	High	High	High	High	High	High	High	1
High	High	High	High	High	High	High	High	1
High	High	High	High	High	High	High	High	1
High	High	High	High	High	High	High	High	1
High	High	High	High	High	High	High	High	1
High	High	High	High	High	High	High	High	1
High	High	High	High	High	High	High	High	1
Peak Foun	Peak Foun	Peak Foun	Peak Foun	High	High	High	High	1
High	High	High	High	High	High	High	High	1
High	High	Peak Foun	High	High	High	High	High	1
Peak Foun	Peak Foun	Peak Foun	Peak Foun	High	High	High	Peak Foun	1
High	High	High	High	High	High	High	High	1
High	High	High	High	High	High	High	High	1
High	High	High	High	High	High	High	High	1
High	High	High	High	High	High	High	High	1
High	High	High	High	High	High	High	High	1
High	High	High	High	High	High	High	High	1
High	High	High	High	High	High	High	High	1
High	High	High	High	High	High	High	High	1
High	High	High	High	High	High	High	High	1
Peak Foun	Peak Foun	Peak Foun	High	High	High	High	High	1
Peak Foun	High	High	High	High	High	High	High	1
High	High	High	High	High	High	High	High	1
High	High	High	High	High	High	High	High	1
High	Peak Foun	High	Peak Foun	High	High	High	High	1
High	High	High	High	High	High	High	High	1
High	Peak Foun	High	High	High	High	High	High	1
High	High	High	High	High	High	High	High	1
Peak Foun	Peak Foun	High	Peak Foun	High	High	High	Peak Foun	1
High	High	High	Peak Foun	High	High	High	Peak Foun	1
High	High	High	High	High	High	High	High	1
Peak Foun	High	High	High	High	High	High	High	1
High	High	High	High	High	High	High	High	1

Peak Foun	Peak Foun	Peak Foun	High	High	High	High	High	1
High	High	High	High	High	High	High	High	1
High	High	High	High	High	High	High	High	1
High	High	High	High	High	High	High	High	1
High	High	High	High	High	High	High	High	1
High	High	High	High	High	High	High	High	1
Peak Foun	High	High	High	High	High	High	High	1
High	High	High	High	High	High	High	High	1
High	High	High	High	High	High	High	High	1
High	High	High	High	High	High	High	High	1
Peak Foun	Peak Foun	Peak Foun	High	High	High	High	Peak Foun	1
High	High	High	High	High	High	High	High	1
High	High	High	High	High	High	High	High	1
High	High	High	High	High	High	High	High	1
Peak Foun	High	High	High	High	High	High	High	1
High	High	High	High	High	High	High	High	1
High	High	Peak Foun	Peak Foun	High	High	High	High	1
High	High	High	High	High	High	High	High	1
Peak Foun	Peak Foun	High	High	High	High	High	High	1
High	High	Peak Foun	Peak Foun	High	High	High	High	1
High	High	High	High	High	High	High	High	1
High	High	High	High	High	High	High	High	1
High	High	High	High	High	High	High	High	1
High	High	Peak Foun	Peak Foun	High	High	High	High	1
High	High	High	Peak Foun	High	High	High	High	1
High	High	High	High	High	High	High	High	1
High	High	High	High	High	High	High	High	1
High	High	Peak Foun	High	High	High	High	High	1
Peak Foun	High	High	High	High	High	High	High	1
High	High	High	High	High	High	High	Peak Foun	1
High	High	High	High	High	High	High	High	1
High	High	High	High	High	High	High	High	1
High	High	High	High	High	High	High	High	1
Peak Foun	High	High	High	High	High	High	High	1
High	High	High	High	High	High	High	High	1
High	High	High	High	High	High	High	High	1
High	Peak Foun	Peak Foun	High	High	High	High	High	1
High	High	High	High	High	High	High	High	1
High	High	High	High	High	High	High	High	1
High	High	High	High	High	High	High	High	1
High	High	High	High	High	High	High	High	1
High	High	High	High	High	High	High	High	1
Peak Foun	Peak Foun	Peak Foun	Peak Foun	High	High	High	High	1
Not Found	Not Found	Not Found	Not Found	High	High	High	High	1
High	High	High	High	High	High	High	High	1

High	High	High	High	High	High	High	High	1
High	High	High	High	High	High	High	High	1
High	High	High	High	High	High	High	High	1
Peak Foun	Peak Foun	Not Found	Peak Foun	High	High	Peak Foun	Peak Foun	1
High	High	High	High	High	High	High	High	1
High	High	High	High	High	High	High	High	1
High	High	High	High	High	High	High	High	1
High	High	High	High	High	High	High	High	1
Peak Foun	High	Peak Foun	High	Peak Foun	High	High	High	1
High	High	High	High	High	High	High	High	1
High	High	High	High	High	High	High	High	1
High	High	High	High	High	High	High	High	1
High	High	High	High	High	High	High	High	1
High	High	High	High	High	High	High	High	1
High	Not Found	Not Found	Peak Foun	High	High	High	Peak Foun	1
High	High	High	High	High	High	High	High	1
High	High	High	High	High	High	High	Peak Foun	1
High	Peak Foun	High	High	High	High	High	High	1
High	High	High	High	High	High	High	High	1
High	High	High	High	High	High	High	High	1
Peak Foun	Peak Foun	Peak Foun	Peak Foun	High	High	High	High	1
High	High	High	High	High	High	High	High	1
Peak Foun	High	High	High	High	High	High	High	1
High	High	High	High	High	High	High	High	1
High	High	High	Peak Foun	High	High	High	High	1
High	High	High	High	High	High	High	High	1
High	High	High	High	High	High	High	High	1
Peak Foun	Peak Foun	High	High	High	High	High	High	1
High	High	High	High	High	High	High	High	1
Peak Foun	High	High	High	High	High	High	High	1
High	High	High	High	High	High	High	High	1
High	High	High	High	High	High	High	High	1
High	High	High	High	High	High	High	High	1
High	High	High	High	High	High	High	High	1
High	High	Peak Foun	High	High	High	High	High	1
High	High	High	High	High	High	High	High	1
High	High	High	High	High	High	High	High	1
High	High	High	High	High	High	Peak Foun	Peak Foun	1
High	High	High	High	High	High	High	High	1
High	High	High	High	High	High	High	High	1
Peak Foun	Peak Foun	Peak Foun	High	High	High	Peak Foun	Peak Foun	1
High	Peak Foun	High	High	High	High	High	Peak Foun	1
Peak Foun	Peak Foun	Peak Foun	Peak Foun	High	High	High	Peak Foun	1
Peak Foun	Peak Foun	Peak Foun	High	High	High	High	High	1
Peak Foun	High	High	High	High	High	High	High	1

High	High	High	High	High	High	High	High	1
High	High	High	High	High	High	High	High	1
High	High	High	High	High	High	High	High	1
High	High	High	Peak Foun	Peak Foun	High	Peak Foun	Peak Foun	1
High	High	High	High	High	High	High	High	1
High	High	High	High	High	High	High	High	1
High	High	High	High	High	High	High	High	1
High	High	High	High	High	High	High	High	1
High	High	High	High	High	High	High	High	1
High	High	High	High	High	High	High	High	1
High	High	High	High	High	High	High	High	1
High	Peak Foun	High	High	High	High	High	High	1
High	High	High	High	High	High	High	High	1
High	High	High	High	High	High	High	High	1
Peak Foun	High	High	High	High	High	High	High	1
High	High	High	High	High	High	High	High	1
High	High	High	High	High	High	High	High	1
High	High	High	High	High	High	High	High	1
High	High	High	High	High	High	High	High	1
High	High	High	High	High	High	High	High	1
High	High	High	High	High	High	High	High	1
High	High	High	High	High	High	High	High	1
High	High	High	High	High	High	High	High	1
High	High	High	High	High	High	High	High	1
High	High	High	High	High	High	High	High	1
High	High	High	High	High	High	High	High	1
High	Peak Foun	Peak Foun	High	High	High	High	High	1
High	High	High	High	High	High	High	High	1
High	High	High	High	High	High	High	High	1
High	High	High	Peak Foun	High	High	Peak Foun	Peak Foun	1
High	High	High	High	High	High	High	High	1
High	High	High	High	High	High	High	High	1
High	High	High	High	High	High	High	High	1
Not Found	Not Found	Not Found	High	High	High	High	High	1
High	High	High	High	High	High	High	High	1
High	High	High	High	High	High	High	High	1
High	High	High	High	High	High	High	High	1
Peak Foun	High	Peak Foun	High	High	High	High	Peak Foun	1
High	High	High	High	High	High	High	High	1
High	Peak Foun	High	High	High	High	High	High	1
Peak Foun	High	High	High	High	High	High	High	1
High	High	High	High	High	High	High	High	1
High	High	High	High	High	High	High	High	1
High	High	High	High	High	High	High	High	1
Peak Foun	Peak Foun	High	High	High	High	High	High	1

High	High	High	High	High	High	High	High	1
High	High	High	High	High	High	High	High	1
Peak Foun	Peak Foun	High	High	High	High	High	High	1
Not Found	Peak Foun	High	Peak Foun	High	Peak Foun	High	High	1
High	High	High	High	High	High	High	High	1
High	High	High	High	High	High	High	High	1
High	High	High	High	High	High	High	High	1
High	High	High	High	High	High	High	High	1
Peak Foun	Peak Foun	High	High	High	High	High	High	1
Peak Foun	High	High	Peak Foun	High	High	High	High	1
High	High	High	High	High	High	High	High	1
High	High	High	High	High	High	High	High	1
High	High	High	High	High	High	High	High	1
High	High	High	High	High	High	High	High	1
High	High	High	High	High	High	High	High	1
High	High	High	High	High	High	High	High	1
High	High	High	High	High	High	High	High	1
Peak Foun	High	High	High	High	High	High	High	1
High	High	High	High	High	High	High	High	1
High	High	High	High	High	High	High	High	1
High	High	High	High	High	High	High	High	1
Peak Foun	High	High	High	High	High	High	Peak Foun	1
High	High	High	High	High	High	High	High	1
High	High	High	High	High	High	High	Peak Foun	1
High	High	High	High	High	High	High	High	1
High	Peak Foun	High	High	High	High	High	High	1
High	High	High	High	High	High	High	High	1
High	High	High	High	High	High	High	High	1
Peak Foun	Peak Foun	Peak Foun	Peak Foun	Peak Foun	High	Peak Foun	Peak Foun	1
High	High	High	High	High	High	High	High	1
Peak Foun	High	High	High	Peak Foun	High	High	Peak Foun	1
Peak Foun	Peak Foun	Peak Foun	Peak Foun	High	High	High	Peak Foun	1
High	High	High	High	High	High	High	High	1
High	High	High	High	High	High	High	High	1
High	High	High	High	High	High	High	High	1
High	High	High	High	High	High	High	Peak Foun	1
High	High	High	High	High	High	High	High	1
High	High	High	Peak Foun	High	High	High	Peak Foun	1
High	High	High	High	High	High	High	Peak Foun	1
High	High	High	High	High	High	High	High	1
High	High	High	High	High	High	High	High	1
High	High	High	High	High	High	High	High	1
High	Peak Foun	High	Peak Foun	Peak Foun	High	High	Peak Foun	1
High	High	High	High	High	High	High	High	1
Peak Foun	High	High	High	High	High	High	High	1

High	High	High	High	High	High	High	High	1
High	Peak Foun	High	High	High	High	High	High	1
Peak Foun	Peak Foun	Peak Foun	Peak Foun	High	High	High	Peak Foun	1
Peak Foun	Peak Foun	Peak Foun	High	High	High	High	High	1
High	High	High	High	High	High	High	High	1
High	High	High	High	High	High	High	High	1
High	High	High	High	High	High	High	High	1
High	Peak Foun	High	High	High	High	High	High	1
High	High	High	High	High	High	High	High	1
High	High	High	High	High	High	High	High	1
High	High	Peak Foun	Peak Foun	High	High	Peak Foun	High	1
High	High	High	High	High	High	High	High	1
High	High	High	High	High	High	High	High	1
High	High	High	Peak Foun	High	High	High	Peak Foun	1
High	High	High	High	High	High	High	High	1
High	High	High	High	High	High	High	High	1
High	High	High	High	High	High	High	High	1
High	High	High	High	High	High	High	High	1
High	Peak Foun	High	Peak Foun	High	High	High	High	1
High	High	High	High	High	High	High	High	1
High	High	Peak Foun	High	High	High	Peak Foun	High	1
High	High	High	High	High	High	High	High	1
High	High	High	High	High	High	High	High	1
High	Not Found	High	High	High	High	High	High	1
Peak Foun	High	High	High	High	High	High	High	1
High	High	High	High	High	High	High	High	1
High	High	High	High	High	High	High	High	1
High	High	High	High	High	High	High	High	1
High	Peak Foun	High	High	High	High	High	Peak Foun	1
Peak Foun	Peak Foun	High	High	High	High	High	High	1
Peak Foun	Peak Foun	High	High	High	High	High	High	1
Peak Foun	Peak Foun	High	High	High	High	High	High	1
High	High	High	High	High	High	High	High	1
High	High	High	High	High	High	High	High	1
High	High	High	High	High	High	High	High	1
High	High	High	High	High	High	High	High	1
High	High	High	High	High	High	High	High	1
High	High	High	High	High	High	High	High	1
Peak Foun	Peak Foun	Peak Foun	High	High	High	High	High	1
Peak Foun	Peak Foun	High	High	High	High	High	High	1
High	High	High	High	High	High	High	High	1
Peak Foun	Peak Foun	High	High	High	High	High	High	1
High	High	High	High	High	High	High	High	1
Peak Foun	Peak Foun	Peak Foun	High	High	High	High	High	1
High	High	High	High	High	High	High	High	1

High	High	High	High	High	High	High	High	1
Peak Foun	High	High	High	High	High	High	High	1
Peak Foun	Peak Foun	Peak Foun	Peak Foun	Peak Foun	Peak Foun	Peak Foun	Not Found	1
Not Found	Peak Foun	High	High	High	High	High	High	1
High	High	High	High	High	High	High	High	1
High	High	High	High	High	High	High	High	1
Peak Foun	Not Found	Peak Foun	Not Found	Peak Foun	Peak Foun	Peak Foun	Peak Foun	1
High	High	High	High	High	High	High	High	1
High	High	High	High	High	High	High	High	1
High	High	High	High	High	High	High	High	1
Peak Foun	Peak Foun	High	Peak Foun	High	High	High	High	1
High	High	High	High	High	High	High	High	1
High	Peak Foun	Peak Foun	High	High	High	High	High	1
High	Peak Foun	High	High	High	High	High	Peak Foun	1
High	High	High	High	High	High	High	High	1
High	High	High	High	High	High	High	High	1
High	High	High	High	High	High	High	High	1
High	High	High	High	High	High	High	High	1
High	High	High	High	High	High	High	High	1
Peak Foun	Peak Foun	High	High	High	High	High	High	1
High	High	High	High	High	High	High	High	1
High	High	High	High	High	High	High	High	1
High	High	Peak Foun	High	High	High	High	High	1
High	High	High	High	High	High	High	High	1
High	High	High	High	High	High	High	High	1
High	High	High	High	High	High	High	High	1
High	High	High	High	High	High	High	High	1
High	High	High	Peak Foun	High	High	Peak Foun	Peak Foun	1
High	High	High	High	High	High	High	High	1
Peak Foun	Peak Foun	High	High	Peak Foun	High	High	Peak Foun	1
Peak Foun	Peak Foun	High	Peak Foun	High	High	High	High	1
High	High	High	High	High	High	High	High	1
High	High	High	High	High	High	High	High	1
Peak Foun	Peak Foun	High	Peak Foun	High	High	High	Peak Foun	1
High	High	High	High	High	High	High	High	1
Peak Foun	High	High	High	High	High	High	High	1
High	High	High	High	High	High	High	High	1
High	High	High	High	High	High	High	High	1
Peak Foun	Peak Foun	Peak Foun	Peak Foun	High	High	High	High	1
High	High	High	High	High	High	High	High	1
Peak Foun	Peak Foun	Peak Foun	High	High	High	High	High	1
High	Peak Foun	High	High	High	High	High	High	1
High	High	High	High	High	High	High	High	1
High	High	High	High	High	High	High	High	1
High	High	High	High	Peak Foun	Peak Foun	High	High	1

High	High	High	High	High	High	High	High	1
High	High	High	High	High	High	High	High	1
High	High	High	High	High	High	High	High	1
High	High	High	High	High	High	High	High	1
High	High	High	High	High	High	High	High	1
High	High	High	High	High	High	High	High	1
High	High	High	High	High	High	High	High	1
High	High	High	High	High	High	High	Peak Foun	1
Not Found	Not Found	Not Found	High	Peak Foun	High	High	Peak Foun	1
High	High	High	High	High	High	High	High	1
Peak Foun	High	Peak Foun	Peak Foun	High	High	High	Peak Foun	1
High	Peak Foun	High	High	High	High	High	High	1
High	High	High	High	High	High	High	High	1
Peak Foun	High	Peak Foun	Peak Foun	High	High	High	High	1
High	High	High	High	High	High	High	High	1
High	High	High	Peak Foun	High	High	Peak Foun	Peak Foun	1
High	High	High	High	High	High	High	High	1
Peak Foun	Peak Foun	High	High	High	High	High	High	1
High	High	High	High	High	High	High	High	1
High	High	High	High	High	High	High	High	1
High	High	High	High	High	High	High	High	1
High	High	High	High	High	High	High	High	1
Peak Foun	High	High	High	High	High	High	High	1
Not Found	Peak Foun	Peak Foun	Peak Foun	High	High	High	High	1
High	High	High	High	High	High	High	High	1
Peak Foun	Peak Foun	Peak Foun	Peak Foun	High	High	High	High	1
High	Peak Foun	Peak Foun	High	High	High	High	Peak Foun	1
High	Peak Foun	High	Peak Foun	High	High	High	High	1
High	High	High	High	High	High	High	Peak Foun	1
High	High	Peak Foun	High	High	High	High	High	1
High	High	High	High	High	High	High	High	1
High	High	High	Peak Foun	High	High	High	High	1
High	High	High	High	High	High	High	High	1
Peak Foun	Peak Foun	Peak Foun	Peak Foun	High	High	High	High	1
High	High	High	High	High	High	High	High	1
High	High	High	High	High	High	High	High	1
High	Peak Foun	High	Peak Foun	High	High	High	High	1
Peak Foun	Peak Foun	Peak Foun	Peak Foun	High	High	High	High	1
High	High	High	High	High	High	High	High	1
Peak Foun	Peak Foun	High	High	High	High	High	Peak Foun	1
High	High	High	High	High	High	High	High	1
Peak Foun	High	High	High	High	High	High	High	1
High	High	High	High	High	High	High	High	1
High	High	High	High	High	High	High	High	1
High	High	High	High	High	High	High	High	1

High	High	High	High	High	High	High	High	1
Peak Foun	Peak Foun	High	Peak Foun	High	High	High	High	1
Peak Foun	High	High	High	Peak Foun	High	High	High	1
High	High	Peak Foun	Peak Foun	High	High	Peak Foun	Peak Foun	1
High	High	High	High	High	High	High	High	1
High	High	High	High	High	High	High	High	1
High	High	High	High	High	High	High	Peak Foun	1
Peak Foun	High	High	High	High	High	High	High	1
Peak Foun	Peak Foun	Peak Foun	High	High	High	High	High	1
High	High	High	High	High	High	High	High	1
Not Found	Peak Foun	High	Peak Foun	High	High	High	High	1
Peak Foun	High	High	High	Peak Foun	High	High	Peak Foun	1
High	High	High	High	High	High	High	High	1
High	High	High	High	High	High	High	High	1
High	High	High	High	High	High	High	High	1
High	Peak Foun	High	High	High	High	High	High	1
High	High	High	High	High	High	High	High	1
High	High	High	High	High	High	High	High	1
Not Found	Not Found	Not Found	Not Found	High	Not Found	Not Found	Not Found	1
Peak Foun	Peak Foun	High	Peak Foun	High	High	High	High	1
High	High	High	High	High	High	High	High	1
High	High	High	High	High	High	High	High	1
High	High	High	High	High	High	High	High	1
Not Found	Not Found	Peak Foun	Not Found	Peak Foun	High	Not Found	Peak Foun	1
Peak Foun	Peak Foun	High	Peak Foun	High	High	High	Peak Foun	1
Peak Foun	High	Peak Foun	High	High	High	High	High	1
High	High	High	High	High	High	High	High	1
High	Peak Foun	High	High	High	High	High	High	1
Peak Foun	Peak Foun	High	High	High	High	High	High	1
High	High	High	High	High	High	High	High	1
Not Found	Not Found	Not Found	Not Found	High	High	Peak Foun	Peak Foun	1
High	High	High	Peak Foun	High	High	High	Peak Foun	1
High	High	High	High	High	High	High	High	1
High	High	High	High	High	High	High	High	1
High	High	High	High	High	High	High	High	1
Peak Foun	Peak Foun	High	High	High	High	High	Peak Foun	1
High	High	High	High	High	High	High	High	1
High	High	High	High	High	High	High	High	1
High	High	High	High	High	High	High	High	1
High	High	High	High	High	High	High	High	1
Peak Foun	High	High	Peak Foun	High	High	High	High	1
High	High	High	High	High	High	High	High	1
High	High	High	High	High	High	High	High	1
High	High	High	High	High	High	High	High	1
High	Peak Foun	Peak Foun	High	High	High	High	High	1

High	High	High	High	High	High	High	High	1
High	High	High	High	High	High	High	High	1
Peak Foun	High	High	High	High	High	High	High	1
High	Peak Foun	Peak Foun	High	High	High	High	High	1
High	High	Peak Foun	Peak Foun	Peak Foun	High	Not Found	Not Found	1
High	High	High	High	High	High	High	High	1
Peak Foun	High	High	High	Peak Foun	High	High	Peak Foun	1
Peak Foun	High	High	High	Peak Foun	High	High	Peak Foun	1
Peak Foun	High	Peak Foun	Peak Foun	High	High	High	High	1
High	High	High	High	High	High	High	High	1
Peak Foun	High	High	High	High	High	High	High	1
High	High	High	High	High	High	High	High	1
High	High	High	High	High	High	High	High	1
High	High	High	Peak Foun	High	High	High	High	1
High	Peak Foun	Peak Foun	Peak Foun	High	Peak Foun	Peak Foun	High	1
High	High	High	High	High	High	High	High	1
High	High	High	High	High	High	High	High	1
Peak Foun	High	High	High	High	High	High	High	1
High	High	High	High	High	High	High	High	1
High	Peak Foun	High	High	High	High	High	High	1
High	High	High	High	High	High	High	High	1
High	High	High	Peak Foun	Peak Foun	Peak Foun	Peak Foun	Peak Foun	1
High	High	High	Peak Foun	High	Peak Foun	Peak Foun	Not Found	1
Peak Foun	Peak Foun	Peak Foun	Peak Foun	Peak Foun	High	Peak Foun	Peak Foun	1
High	High	High	High	High	High	High	High	1
Peak Foun	High	Peak Foun	High	High	High	Peak Foun	High	1
Not Found	High	High	High	High	High	High	High	1
High	High	High	High	High	High	High	High	1
High	High	High	High	High	High	High	High	1
High	High	High	High	High	High	High	High	1
High	High	High	High	High	High	High	Peak Foun	1
High	High	High	High	High	High	High	High	1
High	High	High	High	High	High	High	High	1
High	High	High	High	High	High	High	High	1
High	High	High	High	High	High	High	High	1
High	High	High	High	High	High	High	High	1
Peak Foun	Peak Foun	Peak Foun	Peak Foun	High	High	High	Peak Foun	1
High	High	High	High	High	High	High	High	1
Peak Foun	High	High	High	High	High	High	High	1
Peak Foun	Peak Foun	Peak Foun	High	High	High	High	Peak Foun	1
Peak Foun	Peak Foun	Peak Foun	High	High	High	High	High	1
High	High	High	High	High	High	High	High	1
High	High	High	High	High	High	High	High	1
Peak Foun	Peak Foun	Peak Foun	High	High	High	Peak Foun	High	1
High	High	High	High	High	High	High	High	1

High	High	Peak Foun	Peak Foun	High	Peak Foun	Not Found	Peak Foun	1
High	High	High	High	High	High	High	High	1
Peak Foun	High	Peak Foun	High	High	High	High	High	1
Peak Foun	High	High	High	High	High	High	High	1
High	High	Peak Foun	High	High	High	High	High	1
High	High	High	Peak Foun	Peak Foun	Peak Foun	Peak Foun	Peak Foun	1
High	High	High	High	High	High	High	High	1
High	High	High	High	High	High	High	High	1
High	High	High	High	High	High	High	High	1
Peak Foun	Peak Foun	High	Peak Foun	High	High	High	Peak Foun	1
High	High	High	High	High	High	High	High	1
High	High	High	High	High	High	High	High	1
High	High	High	High	High	High	High	High	1
Peak Foun	Peak Foun	Peak Foun	Peak Foun	Peak Foun	High	Peak Foun	Peak Foun	1
High	High	High	High	High	High	High	High	1
Peak Foun	High	High	High	High	High	High	High	1
High	Peak Foun	Peak Foun	High	High	High	High	High	1
High	High	High	High	Peak Foun	High	High	Peak Foun	1
Peak Foun	High	High	High	High	High	High	High	1
Peak Foun	Peak Foun	Peak Foun	High	High	High	High	High	1
High	High	High	High	High	High	High	High	1
High	High	High	High	High	High	High	High	1
Peak Foun	High	High	High	High	High	High	High	1
High	High	High	High	High	High	High	High	1
High	High	High	High	High	High	High	High	1
Peak Foun	Peak Foun	Peak Foun	Peak Foun	High	High	High	High	1
High	High	High	High	High	High	High	High	1
Peak Foun	Not Found	High	High	High	High	High	High	1
High	High	High	High	High	High	High	High	1
High	High	High	High	High	High	High	High	1
High	High	High	High	High	High	High	High	1
High	High	High	High	High	High	Peak Foun	Peak Foun	1
High	High	High	High	High	High	High	High	1
High	High	High	High	High	High	High	High	1
High	Peak Foun	High	High	High	High	High	High	1
Peak Foun	Peak Foun	Peak Foun	Peak Foun	High	High	High	Peak Foun	1
High	Peak Foun	High	Peak Foun	High	High	High	High	1
High	High	High	High	High	High	High	High	1
High	High	High	High	High	High	High	High	1
High	High	High	High	High	High	High	High	1
Peak Foun	Peak Foun	Peak Foun	Peak Foun	High	High	High	High	1
High	High	High	High	High	High	High	Peak Foun	1
Peak Foun	High	High	High	High	High	High	High	1
High	High	High	High	High	High	High	High	1
High	High	High	High	High	High	High	High	1

High	High	High	High	High	High	High	High	1
High	High	High	High	High	High	High	High	1
Peak Foun	High	Peak Foun	High	High	High	High	High	1
High	Peak Foun	Peak Foun	High	High	High	High	Peak Foun	1
High	High	High	High	High	High	High	High	1
Peak Foun	High	High	High	High	High	High	High	1
High	High	High	High	High	High	Peak Foun	High	1
High	High	High	High	High	High	High	High	1
High	Peak Foun	Peak Foun	High	High	High	High	Peak Foun	1
High	High	High	High	High	High	High	High	1
High	High	High	High	High	High	High	High	1
High	High	High	High	High	High	High	High	1
High	Peak Foun	High	Peak Foun	High	High	High	High	1
High	High	High	High	High	High	High	High	1
High	High	High	High	High	High	High	High	1
High	High	High	High	High	High	High	High	1
High	High	Peak Foun	High	High	High	High	High	1
Peak Foun	High	High	Peak Foun	Peak Foun	High	High	High	1
Peak Foun	Peak Foun	High	High	Peak Foun	High	High	High	1
High	High	Peak Foun	High	High	High	Peak Foun	High	1
High	High	High	High	High	High	High	High	1
High	High	High	High	High	High	High	High	1
High	Peak Foun	High	High	High	High	High	High	1
High	High	High	High	High	High	High	High	1
Peak Foun	Peak Foun	High	High	High	High	High	High	1
High	High	High	High	High	High	High	High	1
Peak Foun	Peak Foun	Peak Foun	Peak Foun	Peak Foun	High	Peak Foun	Peak Foun	1
High	High	High	High	High	High	High	High	1
Peak Foun	High	Peak Foun	High	High	High	High	High	1
High	High	High	High	High	High	High	High	1
Peak Foun	High	High	High	High	High	High	Peak Foun	1
High	High	High	High	High	High	High	High	1
High	High	High	High	High	High	High	High	1
High	High	High	High	High	High	High	High	1
Peak Foun	Peak Foun	Peak Foun	High	High	Peak Foun	Peak Foun	High	1
High	High	High	High	High	High	High	High	1
Peak Foun	Peak Foun	Peak Foun	High	Peak Foun	High	High	Peak Foun	1
Peak Foun	High	High	High	High	High	High	Peak Foun	1
High	High	High	Peak Foun	High	High	Peak Foun	Peak Foun	1
High	High	High	High	High	High	High	High	1
High	High	High	High	High	High	High	High	1
High	Peak Foun	Peak Foun	High	High	High	High	Peak Foun	1
High	Peak Foun	Peak Foun	Peak Foun	High	High	High	High	1
High	High	High	High	High	High	High	High	1
High	High	High	High	High	High	High	High	1

High	High	High	High	High	High	High	High	1
Peak Foun	Peak Foun	Peak Foun	Peak Foun	High	High	Peak Foun	Peak Foun	1
High	High	High	High	High	High	High	High	1
Peak Foun	Peak Foun	Peak Foun	High	High	High	High	High	1
High	High	Peak Foun	Peak Foun	High	High	Peak Foun	High	1
Peak Foun	Peak Foun	Peak Foun	Peak Foun	High	High	High	Peak Foun	1
Peak Foun	Peak Foun	Peak Foun	Peak Foun	Peak Foun	High	High	High	1
High	High	High	High	High	High	High	High	1
High	High	High	High	High	High	High	High	1
High	High	High	High	High	High	High	High	1
High	High	High	High	High	High	High	High	1
Peak Foun	Peak Foun	High	High	High	High	High	High	1
High	High	High	High	High	High	High	High	1
High	High	High	High	High	High	High	High	1
High	High	High	High	High	High	High	High	1
High	High	High	High	High	High	High	High	1
High	High	High	High	High	High	High	High	1
Peak Foun	High	High	High	High	High	High	High	1
Peak Foun	High	High	High	High	High	High	High	1
High	High	High	High	High	High	High	High	1
High	High	High	High	High	High	High	High	1
High	High	High	High	High	High	High	High	1
Peak Foun	Peak Foun	High	Peak Foun	Peak Foun	High	High	Peak Foun	1
High	High	High	High	High	High	High	High	1
High	High	High	High	High	High	Peak Foun	High	1
High	High	High	High	High	High	High	High	1
High	High	High	High	High	High	High	High	1
High	High	High	High	High	High	High	High	1
High	High	High	High	High	High	High	High	1
High	High	High	High	High	High	High	High	1
High	High	High	High	High	High	High	High	1
High	High	High	High	High	High	High	High	1
High	High	High	High	High	High	High	High	1
Peak Foun	High	Peak Foun	High	High	High	High	High	1
High	High	High	Peak Foun	High	High	High	High	1
High	High	High	High	High	High	High	High	1
High	High	High	High	High	High	High	High	1
High	High	High	High	High	High	High	High	1
High	High	High	High	High	High	High	High	1
Peak Foun	Peak Foun	High	High	High	High	High	High	1
High	High	High	High	High	High	High	High	1
Peak Foun	Peak Foun	High	High	High	High	High	High	1
High	High	High	High	High	High	High	High	1
Peak Foun	High	Peak Foun	High	High	High	Peak Foun	High	1
Peak Foun	High	High	High	High	High	High	High	1

High	High	High	High	High	High	High	High	1
High	High	High	High	High	High	High	High	1
High	High	High	High	High	High	High	High	1
High	High	High	Peak Foun	High	High	High	High	1
High	Peak Foun	Peak Foun	High	High	High	High	High	1
High	High	High	High	High	High	High	High	1
High	High	High	High	High	High	High	High	1
High	High	High	High	High	High	High	High	1
High	High	High	High	High	High	High	High	1
Peak Foun	Peak Foun	High	Peak Foun	High	High	High	High	1
High	High	High	High	High	High	High	High	1
High	High	High	High	High	High	High	High	1
Peak Foun	Peak Foun	Peak Foun	Peak Foun	High	High	High	High	1
High	Peak Foun	High	High	High	High	High	High	1
Peak Foun	Peak Foun	High	Peak Foun	High	High	High	High	1
High	High	High	High	High	High	High	High	1
High	High	High	High	High	High	High	High	1
High	High	High	High	High	High	High	High	1
High	High	High	High	High	High	High	High	1
High	High	High	High	High	High	High	High	1
High	High	High	Peak Foun	Peak Foun	High	High	High	1
High	Peak Foun	High	High	High	High	High	High	1
High	High	High	High	High	High	High	Peak Foun	1
High	High	High	High	High	High	High	High	1
High	High	High	Peak Foun	High	High	Peak Foun	Peak Foun	1
High	High	High	High	High	High	High	High	1
High	High	High	High	High	High	High	High	1
High	High	High	High	High	High	High	High	1
High	High	High	High	High	High	High	High	1
Peak Foun	High	High	High	High	High	High	High	1
High	High	High	High	High	High	High	High	1
High	High	High	High	High	High	High	High	1
High	Peak Foun	High	High	High	High	High	High	1
High	Peak Foun	High	High	High	High	High	High	1
Peak Foun	Peak Foun	Peak Foun	High	High	High	High	High	1
High	High	High	High	High	High	High	High	1
Peak Foun	Peak Foun	Peak Foun	High	High	High	High	High	1
High	High	High	High	High	High	High	Peak Foun	1
Peak Foun	High	High	High	High	High	High	High	1
Peak Foun	High	High	High	High	High	High	High	1
High	High	High	High	High	High	High	High	1
High	High	High	High	High	High	High	High	1
High	High	High	High	High	High	High	High	1
High	High	High	High	High	High	High	High	1
High	High	High	High	High	High	High	High	1

Peak Foun	Not Found	Peak Foun	Not Found	High	High	High	High	1
High	High	High	High	High	High	High	High	1
High	High	High	Peak Foun	High	High	High	Peak Foun	1
High	High	High	High	High	High	High	High	1
High	High	High	High	High	High	High	High	1
High	High	High	High	High	High	High	High	1
High	High	High	High	High	High	High	High	1
High	High	High	High	Peak Foun	High	High	Peak Foun	1
High	Peak Foun	Peak Foun	High	High	High	Peak Foun	Peak Foun	1
High	High	High	High	High	High	High	High	1
Peak Foun	Peak Foun	Peak Foun	Peak Foun	High	High	High	Peak Foun	1
High	Peak Foun	High	Peak Foun	High	High	High	High	1
High	High	High	Peak Foun	High	High	High	High	1
High	High	High	High	High	High	High	High	1
Peak Foun	High	Peak Foun	High	High	High	High	High	1
High	High	High	High	High	High	High	High	1
Peak Foun	Peak Foun	High	High	High	High	High	Peak Foun	1
Peak Foun	High	Peak Foun	Not Found	Peak Foun	High	High	High	1
High	High	High	High	High	High	High	High	1
High	High	High	High	High	High	High	High	1
High	High	High	High	High	High	High	High	1
High	High	High	High	High	High	High	High	1
High	High	High	High	High	High	High	High	1
High	High	High	High	High	High	High	High	1
High	Peak Foun	High	High	High	High	High	High	1
High	High	High	High	High	High	High	High	1
High	High	High	High	Peak Foun	Peak Foun	Peak Foun	Peak Foun	1
High	High	High	High	High	High	Peak Foun	Peak Foun	1
High	High	High	Peak Foun	High	High	High	High	1
High	High	High	High	High	High	High	High	1
High	High	High	High	High	High	High	High	1
High	High	High	Peak Foun	High	High	High	High	1
High	High	High	High	High	High	High	Peak Foun	1
High	High	High	High	High	High	High	High	1
Peak Foun	Peak Foun	Peak Foun	Peak Foun	Peak Foun	Peak Foun	Peak Foun	Peak Foun	1
High	High	High	High	High	High	High	High	1
High	High	High	High	High	High	High	High	1
High	Peak Foun	High	High	High	High	High	Peak Foun	1
Peak Foun	Peak Foun	Peak Foun	Peak Foun	Not Found	High	Peak Foun	Peak Foun	1
High	High	High	High	High	High	High	High	1
High	High	High	High	High	High	High	High	1
Peak Foun	Peak Foun	Not Found	High	High	High	High	High	1
High	High	High	High	High	High	High	High	1
High	High	High	High	High	High	High	High	1
High	High	High	High	High	High	High	High	1

High	High	High	High	High	High	High	High	1
High	High	High	High	High	High	High	High	1
High	High	High	High	High	High	High	High	1
Peak Foun	Peak Foun	High	Peak Foun	Peak Foun	High	High	Peak Foun	1
Peak Foun	High	High	High	High	High	High	High	1
High	High	High	Peak Foun	High	High	High	High	1
High	High	High	High	High	High	High	High	1
Peak Foun	Peak Foun	Peak Foun	Peak Foun	Peak Foun	Not Found	High	Peak Foun	1
High	High	High	High	High	High	High	High	1
High	High	High	High	High	High	High	High	1
High	High	High	High	High	High	High	High	1
High	High	High	High	High	High	High	High	1
High	High	High	High	High	High	High	High	1
High	High	High	High	High	High	High	High	1
Peak Foun	High	High	High	High	High	High	High	1
High	High	High	High	High	High	High	High	1
Peak Foun	High	High	High	High	High	High	High	1
High	High	High	High	High	High	High	High	1
Peak Foun	Peak Foun	Peak Foun	Peak Foun	High	Peak Foun	Peak Foun	Peak Foun	1
High	High	High	High	High	High	High	High	1
High	High	High	High	High	High	High	High	1
High	Peak Foun	High	Peak Foun	High	High	High	High	1
Peak Foun	High	High	Peak Foun	High	High	High	Peak Foun	1
High	High	High	High	High	High	High	High	1
High	Peak Foun	High	High	High	High	High	High	1
High	High	High	High	High	High	High	High	1
Peak Foun	Peak Foun	Peak Foun	Peak Foun	High	High	High	Peak Foun	1
High	High	High	High	High	High	High	High	1
High	High	High	High	High	High	High	High	1
High	High	High	High	High	High	High	High	1
High	High	High	High	High	High	High	High	1
Peak Foun	Peak Foun	Peak Foun	Peak Foun	High	High	Peak Foun	High	1
High	Peak Foun	High	High	High	High	High	High	1
High	High	High	High	Peak Foun	High	High	High	1
High	High	High	High	High	High	High	High	1
Not Found	Not Found	Not Found	Not Found	Peak Foun	High	Peak Foun	Not Found	1
High	High	Peak Foun	Peak Foun	High	Peak Foun	Peak Foun	Not Found	1
High	High	High	High	High	High	High	High	1
High	High	High	High	High	High	High	High	1
High	High	High	Peak Foun	High	High	High	High	1
High	High	High	High	High	High	High	High	1
Peak Foun	Peak Foun	Not Found	High	High	High	High	High	1
High	High	High	High	High	High	High	High	1
High	Peak Foun	High	High	High	High	High	High	1
High	High	Peak Foun	High	High	High	High	High	1

High	High	High	High	High	High	High	High	1
Peak Foun	Peak Foun	Not Found	Peak Foun	Peak Foun	High	Peak Foun	Peak Foun	1
High	High	High	High	High	High	High	High	1
Peak Foun	High	High	High	High	High	High	Peak Foun	1
High	High	High	High	High	High	High	High	1
High	High	High	High	High	High	High	High	1
High	High	High	High	High	High	High	High	1
High	Peak Foun	High	High	High	High	Peak Foun	High	1
Peak Foun	Peak Foun	Peak Foun	High	High	High	High	High	1
High	High	High	High	High	High	High	High	1
High	High	High	High	High	High	High	High	1
High	High	High	High	High	High	High	High	1
High	High	High	High	High	High	High	High	1
Peak Foun	Peak Foun	High	High	High	High	High	High	1
High	High	High	High	High	High	High	High	1
High	High	High	High	High	High	High	High	1
Peak Foun	High	High	High	High	High	High	High	1
Peak Foun	High	High	Peak Foun	High	High	High	High	1
High	High	High	High	High	High	High	High	1
High	High	High	High	High	High	High	High	1
High	High	High	High	High	High	High	High	1
High	High	High	High	High	High	High	High	1
High	High	High	High	High	High	High	High	1
High	High	High	High	High	High	High	High	1
High	High	High	High	High	High	High	High	1
High	High	High	High	High	High	High	High	1
High	High	High	High	High	High	High	High	1
High	Peak Foun	High	High	High	High	High	Peak Foun	1
High	High	High	High	High	High	High	High	1
High	High	Peak Foun	High	High	High	High	High	1
High	High	High	High	High	High	High	High	1
High	High	High	High	High	High	High	High	1
Peak Foun	Peak Foun	Not Found	Not Found	High	High	High	Not Found	1
High	High	High	High	High	High	High	High	1
High	High	High	High	High	High	High	High	1
High	High	High	High	High	High	High	High	1
High	High	High	High	High	High	High	High	1
High	High	High	Peak Foun	High	High	High	High	1
High	High	High	High	High	High	High	High	1
High	High	High	Peak Foun	High	High	High	High	1
High	High	High	High	High	High	High	High	1
High	High	High	High	High	High	High	High	1
Peak Foun	Peak Foun	High	Peak Foun	High	High	High	Peak Foun	1
Peak Foun	Not Found	Not Found	Not Found	Not Found	High	Not Found	Not Found	1
Peak Foun	High	Peak Foun	Peak Foun	High	High	Peak Foun	Peak Foun	1

Peak Foun	High	High	High	High	High	High	Peak Foun	1
Peak Foun	High	High	High	Peak Foun	High	High	Peak Foun	1
High	High	High	High	High	High	High	High	1
High	Peak Foun	Peak Foun	Peak Foun	High	High	High	Peak Foun	1
High	High	High	High	High	High	High	Peak Foun	1
Not Found	Not Found	Peak Foun	Peak Foun	High	High	High	Peak Foun	1
High	High	High	High	High	High	High	High	1
High	High	High	High	High	High	High	High	1
High	High	High	High	High	High	High	High	1
High	Peak Foun	High	High	High	High	High	Peak Foun	1
High	High	High	High	High	High	High	High	1
Peak Foun	High	High	High	High	High	High	High	1
High	High	High	Peak Foun	Peak Foun	Peak Foun	Peak Foun	Peak Foun	1
High	High	High	High	High	High	High	High	1
High	High	High	Peak Foun	High	High	High	Peak Foun	1
High	High	High	High	High	High	High	High	1
High	High	High	High	High	High	High	Peak Foun	1
High	High	High	Peak Foun	High	High	High	High	1
Not Found	Peak Foun	Peak Foun	Peak Foun	High	High	High	High	1
High	High	High	High	High	High	High	High	1
High	High	High	High	High	High	High	Peak Foun	1
High	High	High	High	High	High	High	High	1
Peak Foun	High	High	Peak Foun	High	High	High	High	1
Peak Foun	High	Peak Foun	Peak Foun	High	High	High	High	1
Peak Foun	High	High	High	High	High	High	High	1
Not Found	Not Found	Not Found	Not Found	High	High	High	Peak Foun	1
High	High	High	High	High	High	High	High	1
High	High	High	High	High	High	High	High	1
High	High	Peak Foun	Peak Foun	High	High	High	High	1
High	High	High	Peak Foun	High	High	High	Peak Foun	1
High	High	High	High	High	High	High	Peak Foun	1
Not Found	Peak Foun	Not Found	Peak Foun	High	High	High	High	1
Peak Foun	High	High	High	High	High	High	Peak Foun	1
High	High	High	High	High	High	High	High	1
High	High	High	High	High	High	High	High	1
High	High	High	High	High	High	High	High	1
High	High	High	High	High	High	High	High	1
Peak Foun	High	Peak Foun	High	Peak Foun	High	Peak Foun	Peak Foun	1
High	High	High	High	High	High	High	High	1
High	High	Peak Foun	Peak Foun	High	High	High	Peak Foun	1
Peak Foun	High	High	High	High	High	High	High	1
High	High	High	High	High	High	High	High	1
High	High	High	High	High	High	High	High	1
High	High	High	High	High	High	High	High	1
High	High	High	High	High	High	High	High	1

High	High	High	High	High	High	High	High	1
High	High	High	High	High	High	High	High	1
High	Peak Foun	High	Peak Foun	High	High	High	Peak Foun	1
High	Peak Foun	High	High	Peak Foun	High	High	Peak Foun	1
High	High	Peak Foun	High	High	High	High	High	1
High	High	High	High	High	High	High	High	1
High	High	High	High	High	High	High	High	1
Peak Foun	Peak Foun	Peak Foun	High	High	High	High	High	1
Peak Foun	High	High	High	High	High	High	High	1
High	High	High	High	High	High	High	High	1
High	High	High	High	High	High	High	High	1
High	High	High	High	High	High	Peak Foun	High	1
High	High	High	High	High	High	High	High	1
High	High	High	High	High	High	High	High	1
High	High	High	Peak Foun	High	High	High	Peak Foun	1
High	High	High	High	High	High	High	High	1
Peak Foun	High	High	High	High	High	High	High	1
High	High	High	High	High	High	High	High	1
High	High	High	High	High	High	High	High	1
High	High	High	High	High	High	High	High	1
Peak Foun	High	Peak Foun	High	High	High	High	High	1
High	High	High	High	High	High	High	High	1
High	High	High	High	High	High	High	Peak Foun	1
Peak Foun	Peak Foun	High	High	Peak Foun	High	High	Peak Foun	1
Peak Foun	High	High	High	High	High	High	High	1
Peak Foun	High	High	Peak Foun	High	High	High	High	1
Peak Foun	Peak Foun	Peak Foun	Peak Foun	High	High	High	Peak Foun	1
High	High	High	High	High	High	High	High	1
High	High	High	High	High	High	High	High	1
Peak Foun	High	High	High	High	High	High	High	1
Peak Foun	High	High	High	High	High	High	High	1
Peak Foun	High	High	High	High	High	High	High	1
Peak Foun	Peak Foun	High	Peak Foun	High	High	High	Peak Foun	1
High	High	Peak Foun	Peak Foun	High	High	High	Peak Foun	1
Peak Foun	Peak Foun	Peak Foun	Not Found	Peak Foun	Not Found	Not Found	Peak Foun	1
High	High	High	High	High	High	High	High	1
High	High	High	Peak Foun	High	High	High	High	1
Peak Foun	Peak Foun	Peak Foun	Peak Foun	High	High	High	High	1
Peak Foun	Not Found	Not Found	Peak Foun	High	High	High	High	1
High	High	High	High	High	High	High	High	1
High	High	High	High	High	High	High	High	1
Not Found	Not Found	High	Peak Foun	Peak Foun	High	Peak Foun	Peak Foun	1
High	High	High	High	Peak Foun	High	High	Peak Foun	1
Peak Foun	Peak Foun	High	Peak Foun	High	High	High	High	1
Peak Foun	High	High	Peak Foun	Peak Foun	High	High	Peak Foun	1

High	High	Peak Foun	High	High	High	High	High	1
Peak Foun	Peak Foun	Peak Foun	Peak Foun	High	High	High	High	1
High	High	High	High	High	High	High	High	1
Peak Foun	Peak Foun	High	Peak Foun	High	High	High	High	1
High	High	High	High	High	High	High	High	1
High	Peak Foun	High	High	High	High	High	High	1
High	High	High	High	High	High	High	High	1
High	Peak Foun	Peak Foun	Peak Foun	High	Peak Foun	Peak Foun	Peak Foun	1
High	High	High	High	High	High	High	High	1
Peak Foun	Peak Foun	Not Found	High	High	High	Peak Foun	Peak Foun	1
High	High	High	High	High	High	High	Peak Foun	1
High	High	High	High	High	High	High	High	1
High	High	High	High	High	High	High	High	1
High	High	High	High	High	High	High	High	1
Peak Foun	Peak Foun	Peak Foun	Peak Foun	High	High	High	Peak Foun	1
High	High	High	High	High	High	High	Peak Foun	1
High	Peak Foun	High	Peak Foun	High	High	High	Peak Foun	1
High	High	High	High	High	High	High	High	1
High	Peak Foun	High	Peak Foun	High	High	High	Peak Foun	1
Peak Foun	Peak Foun	Peak Foun	Peak Foun	High	High	Peak Foun	Peak Foun	1
Peak Foun	High	High	High	High	High	High	High	1
Not Found	Peak Foun	High	High	High	High	High	High	1
High	High	High	High	High	High	High	High	1
Peak Foun	High	High	High	High	High	High	High	1
High	High	High	High	High	High	High	High	1
Peak Foun	Peak Foun	Peak Foun	High	High	High	High	High	1
Peak Foun	Peak Foun	Peak Foun	High	High	High	High	Peak Foun	1
Not Found	Not Found	Peak Foun	Not Found	High	High	High	High	1
High	High	High	High	High	High	High	High	1
Peak Foun	Peak Foun	Peak Foun	Peak Foun	High	High	High	High	1
High	High	High	High	High	High	High	High	1
High	High	High	High	High	High	High	High	1
High	High	High	High	High	High	High	High	1
High	High	High	High	High	High	High	High	1
High	High	High	High	High	High	High	High	1
High	High	High	High	High	High	High	High	1
High	High	High	High	High	High	High	Peak Foun	1
Peak Foun	Peak Foun	Peak Foun	Peak Foun	High	High	Peak Foun	Not Found	1
Peak Foun	High	High	High	High	High	High	High	1
High	High	High	High	High	High	High	High	1
Peak Foun	Peak Foun	Peak Foun	Peak Foun	High	High	High	Peak Foun	1
High	High	High	High	High	High	High	Peak Foun	1
High	Peak Foun	Peak Foun	High	High	High	High	High	1
High	Peak Foun	Peak Foun	High	High	High	High	Peak Foun	1
High	High	High	High	High	High	High	High	1

High	High	High	High	High	High	High	High	1
Peak Foun	Peak Foun	High	High	High	High	High	High	1
High	High	High	High	High	Peak Foun	High	High	1
High	High	High	High	High	High	High	High	1
Peak Foun	High	High	Peak Foun	High	High	High	High	1
High	High	High	High	High	High	High	High	1
High	Peak Foun	Peak Foun	Not Found	Peak Foun	Peak Foun	Peak Foun	Peak Foun	1
High	High	High	High	High	High	High	High	1
High	High	High	High	High	High	High	High	1
High	High	Peak Foun	High	High	High	High	High	1
High	High	High	High	High	High	High	High	1
High	High	High	High	High	High	High	High	1
High	Peak Foun	High	Peak Foun	High	High	High	Peak Foun	1
Peak Foun	High	High	High	High	High	High	High	1
Peak Foun	Not Found	High	Peak Foun	High	High	High	High	1
High	High	Peak Foun	High	High	High	Peak Foun	High	1
Peak Foun	Peak Foun	Peak Foun	Peak Foun	Peak Foun	High	High	High	1
Peak Foun	High	High	High	High	High	High	High	1
High	Peak Foun	High	High	High	High	High	High	1
High	High	High	High	High	High	High	High	1
High	High	High	High	High	High	High	High	1
High	High	Peak Foun	High	High	High	High	High	1
High	High	High	High	High	High	High	High	1
High	High	High	High	High	High	High	High	1
Peak Foun	High	High	High	High	High	High	High	1
High	High	High	High	High	High	High	High	1
Peak Foun	High	High	High	High	Peak Foun	Peak Foun	Peak Foun	1
High	High	High	High	High	High	High	High	1
High	High	High	High	High	High	High	High	1
High	Peak Foun	Peak Foun	Peak Foun	High	High	Peak Foun	Peak Foun	1
Peak Foun	Peak Foun	Peak Foun	Peak Foun	High	High	High	High	1
High	High	High	High	High	High	High	High	1
High	Peak Foun	High	High	High	High	High	Peak Foun	1
High	Peak Foun	Peak Foun	High	High	High	High	High	1
High	High	High	Peak Foun	Peak Foun	High	High	Peak Foun	1
Peak Foun	Peak Foun	High	High	Peak Foun	High	High	High	1
Not Found	Peak Foun	Peak Foun	Peak Foun	High	High	High	Peak Foun	1
High	High	High	High	High	High	High	High	1
High	Peak Foun	Peak Foun	Peak Foun	Peak Foun	High	High	Peak Foun	1
High	High	High	High	High	High	High	Peak Foun	1
High	Peak Foun	High	High	High	High	High	Peak Foun	1
High	High	Peak Foun	Peak Foun	High	High	High	High	1
High	High	High	High	High	High	High	High	1
High	High	High	Peak Foun	High	High	High	High	1
High	High	Peak Foun	Peak Foun	High	High	High	High	1

Peak Foun	Peak Foun	Peak Foun	High	High	High	High	High	1
High	High	High	High	High	High	High	High	1
High	High	High	High	High	High	High	High	1
Peak Foun	Peak Foun	Peak Foun	Peak Foun	High	High	High	Peak Foun	1
Peak Foun	High	High	High	High	High	High	High	1
Peak Foun	Peak Foun	Peak Foun	Peak Foun	High	High	High	Peak Foun	1
High	High	High	High	High	High	High	High	1
High	High	High	High	High	High	High	High	1
Peak Foun	High	High	High	High	High	High	High	1
High	High	High	High	High	High	High	High	1
High	High	High	High	High	High	High	High	1
Peak Foun	Peak Foun	Not Found	High	High	High	High	Peak Foun	1
High	High	High	High	High	High	High	High	1
Peak Foun	High	High	Peak Foun	High	High	High	Peak Foun	1
High	High	High	High	High	High	High	High	1
Peak Foun	Peak Foun	High	High	High	High	High	High	1
High	High	High	Peak Foun	High	High	High	High	1
High	Peak Foun	High	High	High	High	High	High	1
Not Found	Not Found	Not Found	Peak Foun	High	High	High	Peak Foun	1
High	High	High	High	High	High	High	High	1
Peak Foun	High	High	High	High	High	High	High	1
High	High	High	High	High	High	High	High	1
High	High	High	High	High	High	High	High	1
High	High	High	High	High	High	High	High	1
Peak Foun	Peak Foun	High	High	High	High	High	High	1
High	High	High	High	High	High	High	High	1
Peak Foun	Peak Foun	Peak Foun	Peak Foun	High	High	High	Peak Foun	1
Peak Foun	High	High	Peak Foun	High	High	High	Peak Foun	1
High	High	Not Found	High	High	High	High	High	1
High	Peak Foun	High	High	High	High	High	Peak Foun	1
High	High	Peak Foun	High	High	High	Peak Foun	High	1
High	High	High	High	High	High	High	High	1
Peak Foun	Peak Foun	Peak Foun	Peak Foun	Peak Foun	High	High	Peak Foun	1
High	High	High	Peak Foun	High	High	High	High	1
Peak Foun	High	Peak Foun	High	High	High	High	Peak Foun	1
High	High	High	High	High	High	High	High	1
High	High	High	High	High	High	High	Peak Foun	1
High	High	High	High	High	High	High	High	1
Peak Foun	Peak Foun	High	High	High	High	High	High	1
High	High	High	High	High	High	High	High	1
High	High	High	High	High	High	High	High	1
High	High	High	High	High	High	High	High	1
Peak Foun	Peak Foun	High	High	High	High	High	High	1
High	High	High	High	High	High	High	High	1
High	High	High	High	High	High	High	High	1

High	High	High	Peak Foun	High	High	Peak Foun	Peak Foun	1
High	High	High	Peak Foun	High	High	High	High	1
Peak Foun	Peak Foun	High	High	High	High	High	High	1
High	High	High	High	High	High	High	High	1
Peak Foun	High	High	High	High	High	High	Peak Foun	1
High	High	High	High	High	High	High	High	1
Peak Foun	High	High	High	High	High	High	High	1
High	High	High	High	High	High	High	High	1
High	High	High	High	High	High	High	Peak Foun	1
High	Peak Foun	Peak Foun	High	High	High	High	High	1
High	Peak Foun	High	High	High	High	High	High	1
Peak Foun	High	High	Peak Foun	High	High	High	High	1
Peak Foun	Peak Foun	Not Found	Peak Foun	High	High	High	Peak Foun	1
Peak Foun	Peak Foun	Peak Foun	Peak Foun	High	High	High	High	1
High	Peak Foun	Peak Foun	Peak Foun	High	High	Peak Foun	Peak Foun	1
Not Found	Peak Foun	Peak Foun	High	Peak Foun	High	High	Peak Foun	1
Peak Foun	Peak Foun	High	High	High	High	High	Peak Foun	1
High	High	High	High	High	High	High	High	1
Peak Foun	Peak Foun	High	High	High	High	High	High	1
High	High	High	High	High	High	High	High	1
High	Peak Foun	Not Found	Peak Foun	High	High	High	High	1
High	High	High	Peak Foun	High	High	High	High	1
Peak Foun	Peak Foun	Peak Foun	Peak Foun	High	High	High	High	1
High	High	Peak Foun	High	High	High	High	Peak Foun	1
High	High	High	Peak Foun	High	High	High	Peak Foun	1
Not Found	Not Found	Peak Foun	Peak Foun	High	High	Peak Foun	Peak Foun	1
High	Peak Foun	Peak Foun	Peak Foun	High	High	High	Peak Foun	1
Peak Foun	Peak Foun	Peak Foun	Peak Foun	High	High	High	Peak Foun	1
High	High	High	High	High	High	High	High	1
High	High	High	High	High	High	High	High	1
Peak Foun	Peak Foun	Peak Foun	High	High	High	High	Peak Foun	1
High	High	High	Peak Foun	Peak Foun	High	High	Peak Foun	1
High	High	High	High	High	High	High	High	1
Peak Foun	Peak Foun	Peak Foun	Peak Foun	High	High	High	High	1
High	High	High	High	High	High	High	High	1
Peak Foun	Peak Foun	Not Found	Not Found	High	High	High	Peak Foun	1
High	High	High	High	High	High	High	High	1
High	High	High	High	High	High	High	High	1
High	High	High	High	High	High	High	High	1
High	High	High	High	High	High	High	High	1
High	High	High	High	High	High	High	High	1
High	Peak Foun	High	High	High	High	High	High	1
High	High	High	High	High	High	High	High	1
Peak Foun	Not Found	Peak Foun	Peak Foun	High	High	High	Peak Foun	1
Peak Foun	Peak Foun	Peak Foun	High	High	High	High	Peak Foun	1

Peak Foun	Peak Foun	Peak Foun	Peak Foun	High	High	High	High	1
High	High	High	High	High	High	High	High	1
Peak Foun	Peak Foun	High	Peak Foun	High	High	High	High	1
High	High	High	High	High	High	High	High	1
High	High	High	High	High	High	Peak Foun	High	1
Peak Foun	Peak Foun	High	High	High	High	Peak Foun	Peak Foun	1
High	High	High	High	High	High	High	High	1
High	High	High	High	High	High	High	High	1
High	High	High	Peak Foun	Peak Foun	High	High	Peak Foun	1
High	Peak Foun	High	High	High	High	High	High	1
High	Peak Foun	Peak Foun	High	Peak Foun	High	High	High	1
High	High	Peak Foun	High	High	High	Peak Foun	Peak Foun	1
Peak Foun	Peak Foun	Peak Foun	High	High	High	High	High	1
High	High	High	Peak Foun	Peak Foun	High	Peak Foun	High	1
Peak Foun	Peak Foun	Peak Foun	Peak Foun	High	High	High	High	1
High	High	High	Peak Foun	High	High	High	Peak Foun	1
High	Peak Foun	High	High	High	High	High	High	1
Peak Foun	High	High	Peak Foun	High	High	High	High	1
Peak Foun	Peak Foun	Peak Foun	High	High	High	High	High	1
High	Peak Foun	High	High	High	High	High	Peak Foun	1
High	High	High	High	High	High	High	High	1
High	High	Peak Foun	High	High	High	High	High	1
High	High	High	High	High	High	High	High	1
High	High	High	High	High	High	High	High	1
Peak Foun	Peak Foun	High	Peak Foun	High	High	High	High	1
Peak Foun	Peak Foun	Peak Foun	Not Found	High	High	High	High	1
Peak Foun	Not Found	High	High	High	High	High	High	1
Peak Foun	Peak Foun	High	High	Peak Foun	High	High	High	1
Peak Foun	High	High	Peak Foun	High	High	Peak Foun	Peak Foun	1
Peak Foun	High	Peak Foun	Peak Foun	High	High	High	Peak Foun	1
High	High	Peak Foun	High	High	High	High	High	1
High	High	High	High	Peak Foun	High	High	Peak Foun	1
Peak Foun	High	High	Peak Foun	Peak Foun	High	High	High	1
High	High	High	High	High	High	High	High	1
Peak Foun	High	High	Peak Foun	High	Peak Foun	High	High	1
Peak Foun	High	Peak Foun	Peak Foun	High	High	High	High	1
High	High	Peak Foun	Peak Foun	Peak Foun	High	Peak Foun	High	1
High	High	High	High	High	High	High	High	1
Peak Foun	High	High	High	High	High	High	High	1
High	High	High	High	High	High	High	High	1
High	High	High	High	High	High	High	High	1
High	Peak Foun	High	High	High	High	High	Peak Foun	1
High	High	High	High	High	High	High	Peak Foun	1
High	High	High	High	High	High	High	High	1
High	High	High	Peak Foun	High	High	High	High	1

High	High	High	Peak Foun	High	High	High	High	1
High	High	Peak Foun	High	High	High	High	High	1
High	Peak Foun	High	High	High	High	High	High	1
High	High	High	High	High	High	High	High	1
High	Peak Foun	High	High	High	High	High	High	1
Peak Foun	Peak Foun	High	Peak Foun	High	Peak Foun	Peak Foun	High	1
High	High	High	Peak Foun	Peak Foun	High	Peak Foun	High	1
High	High	High	High	High	High	High	High	1
High	High	High	Peak Foun	Peak Foun	High	High	Peak Foun	1
Peak Foun	Peak Foun	High	High	High	High	High	High	1
High	High	High	Peak Foun	High	High	High	Peak Foun	1
Peak Foun	Peak Foun	High	High	High	High	High	High	1
High	High	High	High	High	High	High	High	1
High	Not Found	Not Found	Peak Foun	High	High	High	Peak Foun	1
Peak Foun	Peak Foun	High	Peak Foun	High	High	High	Peak Foun	1
High	High	Peak Foun	Peak Foun	High	High	High	High	1
Not Found	Not Found	Not Found	Not Found	High	High	High	Not Found	1
High	High	High	Peak Foun	High	High	High	High	1
Peak Foun	High	High	Peak Foun	High	High	High	High	1
Peak Foun	Peak Foun	High	High	High	High	High	Peak Foun	1
Peak Foun	Peak Foun	High	High	High	High	High	High	1
Not Found	Not Found	Not Found	Peak Foun	Not Found	Not Found	Not Found	Peak Foun	1
High	Peak Foun	Peak Foun	Peak Foun	High	High	High	Peak Foun	1
High	High	Peak Foun	High	High	High	High	High	1
Peak Foun	High	High	High	Peak Foun	High	High	High	1
Peak Foun	High	High	High	High	High	High	High	1
Peak Foun	Peak Foun	High	Peak Foun	High	High	High	Peak Foun	1
Peak Foun	Peak Foun	Not Found	Peak Foun	High	High	Peak Foun	Peak Foun	1
Peak Foun	Peak Foun	High	High	High	High	High	High	1
High	Peak Foun	Peak Foun	Peak Foun	High	High	High	High	1
Peak Foun	Peak Foun	High	Peak Foun	High	High	High	High	1
High	High	High	High	High	High	High	High	1
High	Peak Foun	Peak Foun	Peak Foun	Peak Foun	High	High	Peak Foun	1
High	High	Peak Foun	High	High	High	High	Peak Foun	1
Peak Foun	High	High	High	High	High	High	Peak Foun	1
High	High	High	High	High	High	High	High	1
High	High	Peak Foun	Peak Foun	High	High	High	Peak Foun	1
Peak Foun	Not Found	Peak Foun	High	High	High	High	High	1
High	High	High	High	High	High	High	High	1
Peak Foun	High	High	High	High	High	High	High	1
Peak Foun	Peak Foun	High	Peak Foun	High	High	High	Peak Foun	1
High	High	High	High	High	High	High	High	1
Not Found	Not Found	Not Found	Not Found	Not Found	High	Not Found	Not Found	1
High	High	High	High	High	High	High	High	1
High	High	High	High	High	High	High	High	1

High	Peak Foun	High	High	High	High	High	High	1
Peak Foun	Peak Foun	High	Peak Foun	High	High	High	High	1
High	Peak Foun	High	High	High	High	High	High	1
High	High	High	High	High	High	High	High	1
Peak Foun	Peak Foun	Peak Foun	Peak Foun	Peak Foun	High	High	Peak Foun	1
Peak Foun	Peak Foun	Peak Foun	Peak Foun	High	High	Peak Foun	Peak Foun	1
High	High	High	High	High	High	High	High	1
High	High	High	High	High	High	High	High	1
Peak Foun	High	High	Peak Foun	Peak Foun	High	High	High	1
High	High	High	High	High	High	Peak Foun	Peak Foun	1
Peak Foun	Peak Foun	Peak Foun	Peak Foun	Peak Foun	High	Peak Foun	Peak Foun	1
High	High	High	High	High	High	Peak Foun	High	1
Peak Foun	High	Peak Foun	Peak Foun	High	High	High	Peak Foun	1
High	High	High	High	High	High	High	High	1
Peak Foun	Peak Foun	Peak Foun	Peak Foun	High	High	High	Peak Foun	1
High	High	Peak Foun	Peak Foun	High	High	Peak Foun	High	1
High	High	High	High	Peak Foun	High	High	High	1
High	High	High	High	High	High	High	High	1
High	High	Peak Foun	High	Peak Foun	High	Peak Foun	High	1
Peak Foun	Peak Foun	Peak Foun	Peak Foun	High	High	High	High	1
Peak Foun	Peak Foun	High	High	Peak Foun	High	High	High	1
Peak Foun	Peak Foun	High	High	Peak Foun	High	High	Peak Foun	1
High	High	High	Peak Foun	High	High	High	High	1
Peak Foun	High	High	Peak Foun	High	High	High	High	1
Peak Foun	High	High	High	High	High	High	High	1
Peak Foun	High	Peak Foun	Peak Foun	High	High	High	High	1
Not Found	Not Found	Not Found	Not Found	High	High	High	Peak Foun	1
High	High	High	High	High	High	High	High	1
Peak Foun	Peak Foun	High	High	High	High	High	High	1
Peak Foun	Peak Foun	Peak Foun	Peak Foun	Peak Foun	High	High	High	1
Peak Foun	Peak Foun	Peak Foun	High	High	High	High	High	1
High	High	High	Peak Foun	High	High	High	High	1
High	High	High	High	High	High	High	High	1
High	High	High	High	High	High	High	High	1
High	High	High	High	Peak Foun	High	High	High	1
Peak Foun	Peak Foun	Peak Foun	Peak Foun	High	High	High	Peak Foun	1
High	Peak Foun	Peak Foun	Peak Foun	High	High	Peak Foun	Peak Foun	1
Peak Foun	High	High	High	High	High	High	High	1
Peak Foun	Peak Foun	High	Peak Foun	Peak Foun	High	High	High	1
Peak Foun	Peak Foun	Peak Foun	High	High	High	High	High	1
Peak Foun	Peak Foun	Not Found	Peak Foun	High	Peak Foun	High	Peak Foun	1
High	Peak Foun	High	Peak Foun	High	High	High	High	1
Peak Foun	Peak Foun	High	High	High	High	High	High	1
High	High	High	High	High	High	High	High	1
High	High	High	Peak Foun	High	High	High	Peak Foun	1

High	High	High	High	High	High	High	High	1
Not Found	Not Found	Not Found	Peak Foun	Not Found	High	High	Not Found	1
High	High	High	High	High	High	High	High	1
High	Peak Foun	Peak Foun	High	High	High	Peak Foun	Peak Foun	1
High	High	High	Peak Foun	High	High	High	Peak Foun	1
High	High	High	High	High	High	High	High	1
High	High	High	Peak Foun	High	High	High	Not Found	1
Peak Foun	Peak Foun	Peak Foun	Peak Foun	High	High	High	Peak Foun	1
High	High	High	Peak Foun	High	High	High	Peak Foun	1
Peak Foun	Peak Foun	Peak Foun	Peak Foun	Peak Foun	Peak Foun	Not Found	Peak Foun	1
Peak Foun	Peak Foun	Peak Foun	High	High	High	High	High	1
High	Peak Foun	Peak Foun	Peak Foun	High	High	Peak Foun	Peak Foun	1
Peak Foun	High	Peak Foun	High	High	High	High	High	1
High	High	High	High	High	High	High	High	1
High	Peak Foun	Peak Foun	Peak Foun	High	High	High	Peak Foun	1
High	High	High	High	High	High	High	High	1
Peak Foun	High	High	Peak Foun	Peak Foun	High	High	Peak Foun	1
High	Peak Foun	Peak Foun	Not Found	High	High	Peak Foun	Not Found	1
High	High	High	High	Peak Foun	High	High	High	1
Peak Foun	High	High	Peak Foun	High	High	High	High	1
High	High	High	High	High	High	High	Peak Foun	1
High	Peak Foun	High	Peak Foun	Peak Foun	High	High	Not Found	1
High	High	Peak Foun	High	High	High	Peak Foun	High	1
Peak Foun	Not Found	Peak Foun	High	Peak Foun	High	High	High	1
High	Peak Foun	Peak Foun	Peak Foun	Peak Foun	High	Peak Foun	Peak Foun	1
High	High	High	High	High	High	High	High	1
Peak Foun	Peak Foun	Peak Foun	Peak Foun	High	High	High	High	1
Peak Foun	Peak Foun	Peak Foun	High	High	High	High	High	1
High	High	High	High	High	High	High	High	1
Peak Foun	Peak Foun	Peak Foun	High	High	High	High	High	1
High	High	High	Peak Foun	High	High	High	Peak Foun	1
High	High	High	High	High	High	High	High	1
Peak Foun	High	Peak Foun	High	High	High	High	Peak Foun	1
High	High	High	High	High	High	High	High	1
Peak Foun	Peak Foun	High	High	High	High	High	High	1
Peak Foun	Peak Foun	High	High	High	High	High	High	1
High	High	High	High	High	High	High	High	1
Peak Foun	Peak Foun	Peak Foun	High	High	High	High	High	1
High	Peak Foun	High	Peak Foun	Peak Foun	High	High	Peak Foun	1
High	High	High	High	High	High	High	High	1
High	Peak Foun	Peak Foun	Peak Foun	Peak Foun	Peak Foun	Peak Foun	Peak Foun	1
Not Found	Not Found	High	High	High	High	High	High	1
High	Peak Foun	High	High	High	High	High	High	1
High	High	High	Peak Foun	High	High	High	High	1
Not Found	Not Found	Not Found	Peak Foun	Peak Foun	High	Peak Foun	Peak Foun	1

Peak Foun	Peak Foun	Peak Foun	Peak Foun	High	High	High	Peak Foun	1
Not Found	Peak Foun	Peak Foun	Peak Foun	High	High	Peak Foun	Peak Foun	1
High	High	High	High	High	Peak Foun	High	High	1
Peak Foun	Not Found	Peak Foun	High	High	High	High	Peak Foun	1
Peak Foun	High	Peak Foun	High	Peak Foun	High	High	Peak Foun	1
Peak Foun	Peak Foun	Peak Foun	High	High	High	High	High	1
Peak Foun	High	Peak Foun	Peak Foun	Peak Foun	Peak Foun	Peak Foun	Peak Foun	1
High	High	High	High	High	High	High	High	1
High	High	High	High	High	High	High	High	1
High	High	Peak Foun	High	High	High	High	High	1
High	Peak Foun	Peak Foun	High	Peak Foun	Peak Foun	Peak Foun	Peak Foun	1
Peak Foun	Not Found	Peak Foun	Peak Foun	High	High	High	Peak Foun	1
Not Found	High	High	Peak Foun	High	High	High	Peak Foun	1
High	High	High	Peak Foun	High	High	High	Peak Foun	1
High	High	High	High	High	High	High	High	1
High	High	Peak Foun	Peak Foun	High	High	High	Peak Foun	1
Peak Foun	Not Found	Not Found	Peak Foun	Peak Foun	High	High	Peak Foun	1
High	Peak Foun	High	Peak Foun	High	High	Peak Foun	Peak Foun	1
High	High	High	High	High	High	High	High	1
High	High	High	High	High	High	High	High	1
High	High	High	High	High	High	High	High	1
High	High	High	High	High	High	High	High	1
Peak Foun	Peak Foun	High	High	High	High	High	High	1
High	High	High	High	High	High	High	High	1
Peak Foun	High	High	High	High	High	High	High	1
High	High	High	High	High	High	High	High	1
High	High	High	High	High	High	High	High	1
Peak Foun	Not Found	Peak Foun	Peak Foun	High	High	High	High	1
High	High	High	High	High	High	High	High	1
High	High	High	High	Peak Foun	High	High	High	1
High	High	High	High	High	High	High	Peak Foun	1
High	High	High	Peak Foun	Peak Foun	High	High	High	1
High	High	High	Peak Foun	High	High	High	Peak Foun	1
High	High	High	High	High	High	High	High	1
Peak Foun	Peak Foun	High	Peak Foun	High	High	High	Peak Foun	1
Peak Foun	Peak Foun	Peak Foun	High	High	High	High	High	1
High	High	High	High	High	High	High	High	1
Peak Foun	Peak Foun	High	Peak Foun	Peak Foun	Peak Foun	High	Peak Foun	1
High	High	High	Peak Foun	High	High	Peak Foun	Peak Foun	1
High	High	High	Peak Foun	Peak Foun	Peak Foun	Peak Foun	Peak Foun	1
High	Peak Foun	High	Peak Foun	High	High	High	High	1
Peak Foun	High	High	High	High	High	Peak Foun	Peak Foun	1
Peak Foun	High	High	High	High	High	High	Peak Foun	1
Peak Foun	High	High	High	High	High	High	High	1
High	High	High	High	High	High	High	High	1

Peak Foun	Peak Foun	High	High	High	High	High	High	1
Peak Foun	Peak Foun	High	High	High	High	High	High	1
High	High	High	High	High	High	High	High	1
High	High	High	High	High	High	High	High	1
Not Found	Peak Foun	Peak Foun	Peak Foun	High	High	Peak Foun	Peak Foun	1
High	High	High	High	High	High	High	High	1
High	High	High	High	High	High	High	Peak Foun	1
High	Peak Foun	Peak Foun	High	High	High	High	High	1
Not Found	Not Found	Peak Foun	Peak Foun	High	High	High	High	1
High	High	High	High	High	High	High	High	1
High	High	High	High	High	High	High	High	1
High	High	High	High	High	High	High	High	1
Not Found	Peak Foun	Not Found	Peak Foun	Peak Foun	High	High	Peak Foun	1
High	High	High	High	High	High	High	Peak Foun	1
High	High	High	High	High	High	High	High	1
Peak Foun	Peak Foun	Peak Foun	High	High	High	High	High	1
Peak Foun	High	High	High	High	High	High	High	1
Not Found	Not Found	Not Found	Not Found	Peak Foun	High	High	Peak Foun	1
High	High	High	High	High	High	High	High	1
High	High	Peak Foun	Peak Foun	High	High	High	Peak Foun	1
Peak Foun	Peak Foun	Peak Foun	Peak Foun	High	Peak Foun	High	High	1
High	High	High	High	High	High	High	High	1
High	High	High	Peak Foun	High	High	High	High	1
High	High	High	Peak Foun	High	High	High	High	1
Peak Foun	High	High	Peak Foun	Peak Foun	High	High	Peak Foun	1
High	High	High	Peak Foun	High	High	High	High	1
Not Found	Not Found	High	Peak Foun	Peak Foun	High	Peak Foun	Peak Foun	1
High	High	High	Peak Foun	High	High	High	Peak Foun	1
High	High	High	Peak Foun	High	High	High	High	1
High	Not Found	High	Peak Foun	High	High	High	High	1
Peak Foun	High	High	High	High	High	High	Peak Foun	1
High	High	High	High	High	High	High	High	1
Peak Foun	Peak Foun	Peak Foun	High	High	High	Peak Foun	Peak Foun	1
Peak Foun	Peak Foun	Peak Foun	High	High	High	High	Peak Foun	1
Peak Foun	High	Peak Foun	Peak Foun	Peak Foun	High	Peak Foun	Peak Foun	1
Peak Foun	Peak Foun	Peak Foun	High	High	High	High	Peak Foun	1
Peak Foun	Not Found	Not Found	High	High	High	High	High	1
Peak Foun	Peak Foun	Peak Foun	Peak Foun	Peak Foun	Peak Foun	Peak Foun	Peak Foun	1
Peak Foun	High	Peak Foun	High	High	High	High	High	1
High	High	High	High	High	Peak Foun	Peak Foun	High	1
High	High	High	High	Peak Foun	High	High	High	1
High	High	High	High	High	High	High	High	1
Peak Foun	Peak Foun	High	High	High	High	High	Peak Foun	1
Peak Foun	High	High	High	High	High	High	High	1
Peak Foun	Peak Foun	Peak Foun	High	High	High	High	High	1

High	High	High	Peak Foun	High	High	High	Peak Foun	1
High	High	Peak Foun	Peak Foun	High	High	High	Peak Foun	1
Peak Foun	Peak Foun	Peak Foun	Peak Foun	High	High	High	Peak Foun	1
High	High	High	High	High	High	High	High	1
Peak Foun	Peak Foun	High	Peak Foun	Peak Foun	High	High	Peak Foun	1
High	High	High	Peak Foun	High	High	High	High	1
High	High	High	High	High	High	High	High	1
Peak Foun	Peak Foun	Peak Foun	Peak Foun	Peak Foun	High	High	High	1
Peak Foun	High	High	High	High	High	High	Peak Foun	1
High	High	High	High	High	High	High	High	1
Not Found	Not Found	Peak Foun	High	High	Not Found	Not Found	Not Found	1
High	High	Peak Foun	Peak Foun	High	High	Peak Foun	High	1
High	High	High	High	High	High	High	High	1
Not Found	High	High	Not Found	Peak Foun	High	Peak Foun	Peak Foun	1
Peak Foun	High	High	High	High	High	High	High	1
High	High	High	High	High	High	High	High	1
Peak Foun	Peak Foun	Peak Foun	High	Peak Foun	High	High	High	1
Peak Foun	High	High	High	High	High	High	High	1
High	High	High	Peak Foun	High	High	High	Peak Foun	1
High	High	High	High	High	High	High	High	1
Peak Foun	Peak Foun	Peak Foun	Peak Foun	High	Peak Foun	High	Peak Foun	1
High	High	High	High	High	High	High	High	1
High	High	Peak Foun	Peak Foun	Peak Foun	Peak Foun	Peak Foun	Peak Foun	1
High	High	High	High	High	High	High	High	1
High	High	High	Peak Foun	High	High	High	High	1
High	Peak Foun	Peak Foun	Peak Foun	High	High	High	Peak Foun	1
Peak Foun	High	High	High	High	High	High	Peak Foun	1
High	High	High	High	High	High	High	High	1
High	High	High	High	High	High	High	High	1
High	High	High	High	High	High	High	High	1
Not Found	Not Found	Not Found	Not Found	High	High	High	High	1
High	High	Peak Foun	Peak Foun	High	Peak Foun	Peak Foun	Peak Foun	1
High	Peak Foun	Peak Foun	Peak Foun	High	High	High	Peak Foun	1
Peak Foun	High	High	High	High	High	High	High	1
High	High	High	High	High	High	High	High	1
High	High	High	High	High	High	High	Peak Foun	1
Not Found	Not Found	Not Found	Not Found	High	High	Peak Foun	High	1
High	High	Peak Foun	Peak Foun	Peak Foun	Peak Foun	Peak Foun	Peak Foun	1
Peak Foun	High	High	High	High	High	High	High	1
High	High	Peak Foun	High	High	High	Peak Foun	High	1
High	High	High	High	High	High	High	High	1
High	High	High	High	High	High	High	High	1
High	High	High	High	High	High	Peak Foun	High	1
High	High	High	High	High	High	High	High	1
High	High	High	High	Peak Foun	High	High	Peak Foun	1

High	High	High	High	High	High	High	High	1
Peak Foun	High	Peak Foun	High	Peak Foun	High	High	High	1
High	Peak Foun	Peak Foun	Peak Foun	High	High	High	Peak Foun	1
High	High	High	High	High	High	High	High	1
High	High	High	High	High	High	High	High	1
Peak Foun	High	High	High	High	High	High	High	1
Peak Foun	Peak Foun	High	Peak Foun	High	High	High	Peak Foun	1
Peak Foun	Peak Foun	Peak Foun	High	Peak Foun	High	High	High	1
High	High	High	Peak Foun	High	High	High	High	1
High	High	Peak Foun	Peak Foun	High	High	High	Peak Foun	1
High	High	High	High	High	High	High	High	1
Peak Foun	High	Peak Foun	Peak Foun	High	High	Peak Foun	Peak Foun	1
Not Found	High	High	Not Found	Peak Foun	High	High	High	1
Peak Foun	Not Found	Peak Foun	High	High	High	Peak Foun	High	1
Peak Foun	High	Peak Foun	Peak Foun	Peak Foun	Peak Foun	High	Peak Foun	1
Peak Foun	Peak Foun	Peak Foun	Peak Foun	High	High	Peak Foun	Peak Foun	1
Peak Foun	Peak Foun	High	Peak Foun	Not Found	High	High	Peak Foun	1
High	High	High	High	High	High	High	High	1
High	High	High	High	High	High	High	High	1
High	High	High	High	High	High	High	High	1
Peak Foun	High	High	High	Peak Foun	High	High	Peak Foun	1
High	Peak Foun	High	Peak Foun	High	Peak Foun	High	Peak Foun	1
High	High	High	High	High	High	High	High	1
High	High	High	Peak Foun	High	High	High	Peak Foun	1
High	High	High	High	High	High	High	Peak Foun	1
High	High	High	High	High	High	High	High	1
High	High	High	High	High	High	High	High	1
High	High	High	High	High	High	High	High	1
High	High	High	High	High	High	High	High	1
High	High	High	Peak Foun	High	High	High	Peak Foun	1
Not Found	Not Found	Not Found	Not Found	Peak Foun	High	Not Found	Not Found	1
High	Peak Foun	Peak Foun	Peak Foun	High	Peak Foun	Peak Foun	Peak Foun	1
Peak Foun	Peak Foun	Peak Foun	Peak Foun	Peak Foun	Peak Foun	High	Peak Foun	1
Peak Foun	Peak Foun	Peak Foun	High	High	High	High	High	1
High	High	High	High	High	High	High	High	1
High	High	High	Peak Foun	High	High	High	High	1
Peak Foun	Peak Foun	Peak Foun	Not Found	Peak Foun	High	High	Not Found	1
High	High	Peak Foun	High	High	High	Peak Foun	High	1
High	High	High	High	High	High	High	High	1
High	High	High	High	High	High	High	High	1
Peak Foun	Peak Foun	Peak Foun	Peak Foun	High	High	High	Peak Foun	1
Peak Foun	Peak Foun	High	Peak Foun	High	High	High	Peak Foun	1
High	Peak Foun	High	High	High	High	High	High	1
Peak Foun	Peak Foun	Peak Foun	Peak Foun	High	High	High	Peak Foun	1
Peak Foun	Peak Foun	High	Peak Foun	Peak Foun	High	High	Peak Foun	1

High	Peak Foun	Peak Foun	High	Peak Foun	High	Peak Foun	High	1
High	Peak Foun	High	Peak Foun	High	High	High	High	1
Peak Foun	High	High	High	Peak Foun	High	High	High	1
High	High	High	High	High	High	High	High	1
High	High	High	High	High	High	High	High	1
Peak Foun	High	Peak Foun	High	High	Peak Foun	High	Peak Foun	1
High	High	High	High	High	High	High	High	1
Peak Foun	Peak Foun	High	Peak Foun	High	High	High	Peak Foun	1
Peak Foun	Peak Foun	High	High	High	High	High	High	1
Peak Foun	Peak Foun	Peak Foun	Peak Foun	High	High	Peak Foun	Peak Foun	1
Peak Foun	Peak Foun	Peak Foun	High	Peak Foun	High	High	Peak Foun	1
Peak Foun	Peak Foun	High	High	High	High	High	Peak Foun	1
High	High	High	High	High	High	High	Peak Foun	1
High	High	High	High	High	High	High	Peak Foun	1
High	High	High	High	High	High	High	High	1
Peak Foun	High	High	High	Peak Foun	High	High	Peak Foun	1
High	High	High	High	High	High	High	High	1
Peak Foun	High	High	Peak Foun	High	High	High	Peak Foun	1
Peak Foun	High	High	High	High	High	High	Peak Foun	1
High	High	Peak Foun	High	High	High	High	High	1
Peak Foun	Peak Foun	Peak Foun	Peak Foun	Peak Foun	Peak Foun	High	Peak Foun	1
High	High	High	High	High	High	High	High	1
High	High	High	High	High	High	High	High	1
High	High	High	High	High	High	High	High	1
Peak Foun	Peak Foun	Peak Foun	High	High	High	High	Peak Foun	1
High	High	High	Peak Foun	High	High	High	Peak Foun	1
High	High	High	Peak Foun	High	High	High	High	1
High	High	High	High	High	High	High	High	1
Peak Foun	Peak Foun	Peak Foun	Peak Foun	High	High	High	Peak Foun	1
Peak Foun	Peak Foun	High	Peak Foun	Peak Foun	Peak Foun	Peak Foun	Peak Foun	1
Not Found	Peak Foun	High	Not Found	High	High	High	Peak Foun	1
High	High	High	Peak Foun	Peak Foun	High	Peak Foun	High	1
High	High	Peak Foun	High	High	High	High	High	1
Peak Foun	Not Found	High	High	High	High	High	High	1
High	High	High	High	High	High	High	High	1
Peak Foun	High	High	High	High	High	High	High	1
High	Peak Foun	High	High	High	High	High	High	1
Peak Foun	Peak Foun	Peak Foun	Peak Foun	High	Peak Foun	High	Peak Foun	1
High	Peak Foun	Peak Foun	Peak Foun	High	Peak Foun	Peak Foun	High	1
High	High	High	Peak Foun	High	High	High	Peak Foun	1
Peak Foun	High	High	Peak Foun	High	High	Peak Foun	Peak Foun	1
High	High	High	High	High	High	High	Peak Foun	1
Peak Foun	Peak Foun	Peak Foun	Peak Foun	High	High	High	High	1
High	High	High	High	High	High	High	High	1
Peak Foun	Peak Foun	Peak Foun	Peak Foun	High	High	High	High	1

High	High	High	Peak Foun	Peak Foun	High	High	Peak Foun	1
Peak Foun	Peak Foun	High	Peak Foun	High	High	High	Peak Foun	1
High	Peak Foun	High	Peak Foun	High	Peak Foun	Peak Foun	Peak Foun	1
Peak Foun	Peak Foun	Peak Foun	High	Peak Foun	High	Peak Foun	Peak Foun	1
High	Peak Foun	High	High	Peak Foun	Peak Foun	High	Peak Foun	1
High	High	High	Peak Foun	Peak Foun	High	High	Peak Foun	1
High	High	High	Peak Foun	Peak Foun	High	Peak Foun	Peak Foun	1
Peak Foun	High	High	High	High	High	High	High	1
Peak Foun	Peak Foun	High	Peak Foun	High	High	High	Peak Foun	1
Peak Foun	Peak Foun	High	Peak Foun	High	High	High	Peak Foun	1
Peak Foun	Peak Foun	Peak Foun	Peak Foun	Peak Foun	High	High	Peak Foun	1
Peak Foun	Peak Foun	Peak Foun	Peak Foun	Peak Foun	Peak Foun	Peak Foun	High	1
Peak Foun	Peak Foun	Peak Foun	Peak Foun	High	High	High	Peak Foun	1
Peak Foun	High	Peak Foun	Peak Foun	Peak Foun	High	Peak Foun	Peak Foun	1
Peak Foun	Peak Foun	High	Peak Foun	High	High	High	High	1
High	High	High	High	High	High	High	High	1
Peak Foun	High	Peak Foun	High	Peak Foun	High	Peak Foun	High	1
High	High	High	Peak Foun	High	High	High	Peak Foun	1
High	Peak Foun	Peak Foun	High	High	High	Peak Foun	Peak Foun	1
Peak Foun	Not Found	Peak Foun	Peak Foun	Not Found	High	High	Not Found	1
High	High	High	High	High	High	Peak Foun	High	1
High	High	High	Peak Foun	High	High	Peak Foun	High	1
High	High	High	Peak Foun	Peak Foun	Peak Foun	High	Peak Foun	1
Peak Foun	Peak Foun	High	Peak Foun	High	High	Peak Foun	Peak Foun	1
Not Found	Not Found	Not Found	Peak Foun	High	High	Peak Foun	Peak Foun	1
High	High	High	High	High	High	High	High	1
Peak Foun	Peak Foun	Peak Foun	High	Peak Foun	High	High	Peak Foun	1
Peak Foun	High	High	High	High	High	High	High	1
High	High	High	High	High	High	High	High	1
Peak Foun	High	High	Peak Foun	High	High	High	Peak Foun	1
High	High	High	High	High	High	Peak Foun	High	1
High	High	Peak Foun	Peak Foun	High	High	Peak Foun	Peak Foun	1
High	Peak Foun	High	Peak Foun	High	High	High	High	1
High	High	High	High	High	High	High	High	1
Peak Foun	Peak Foun	Peak Foun	High	High	High	High	Peak Foun	1
Peak Foun	Peak Foun	High	Peak Foun	Peak Foun	High	Peak Foun	Peak Foun	1
High	High	High	High	High	High	High	High	1
High	High	High	High	High	High	High	High	1
High	High	High	High	High	High	High	High	1
High	High	High	High	High	High	High	High	1
High	High	High	High	Peak Foun	High	High	High	1
Peak Foun	Peak Foun	Peak Foun	Peak Foun	High	High	High	Peak Foun	1
High	High	High	Peak Foun	High	High	High	Peak Foun	1
High	High	High	High	High	High	High	High	1
Not Found	Not Found	Peak Foun	Peak Foun	High	High	High	Peak Foun	1

High	High	High	High	High	High	High	High	1
High	High	High	High	High	High	High	Peak Foun	1
High	High	Peak Foun	Peak Foun	Peak Foun	Peak Foun	Peak Foun	Not Found	1
High	High	Peak Foun	Peak Foun	High	Peak Foun	Peak Foun	High	1
High	High	Peak Foun	High	High	High	High	High	1
High	High	High	High	High	High	High	High	1
High	High	High	High	High	High	High	High	1
Peak Foun	Peak Foun	Peak Foun	Peak Foun	High	High	Peak Foun	Peak Foun	1
High	High	High	Peak Foun	High	Peak Foun	High	High	1
High	High	High	Peak Foun	Peak Foun	Peak Foun	Peak Foun	Peak Foun	1
Peak Foun	High	High	Peak Foun	Peak Foun	High	High	Peak Foun	1
High	Peak Foun	High	Peak Foun	High	High	High	Peak Foun	1
High	High	High	Peak Foun	High	High	Peak Foun	High	1
Peak Foun	Not Found	Not Found	Peak Foun	High	High	High	Peak Foun	1
Peak Foun	Peak Foun	Peak Foun	High	High	Peak Foun	Peak Foun	Peak Foun	1
Peak Foun	Peak Foun	High	Peak Foun	Peak Foun	High	High	Peak Foun	1
Peak Foun	Peak Foun	High	Peak Foun	High	High	Peak Foun	Peak Foun	1
Peak Foun	Peak Foun	Peak Foun	Peak Foun	Peak Foun	Peak Foun	High	Peak Foun	1
Peak Foun	High	High	Peak Foun	Peak Foun	High	High	Peak Foun	1
High	High	High	High	High	High	High	High	1
High	High	High	Peak Foun	High	High	High	Peak Foun	1
High	High	High	High	High	High	High	High	1
High	High	Peak Foun	High	High	High	High	Peak Foun	1
Peak Foun	Peak Foun	Peak Foun	Peak Foun	High	High	High	Peak Foun	1
High	High	High	High	High	High	High	High	1
Peak Foun	Peak Foun	High	Peak Foun	High	High	High	High	1
High	High	High	High	High	High	High	High	1
High	High	High	High	High	High	High	High	1
High	High	High	Peak Foun	High	High	Peak Foun	High	1
High	High	High	High	High	High	High	Peak Foun	1
Peak Foun	Peak Foun	Peak Foun	Peak Foun	High	High	High	Peak Foun	1
High	High	High	High	High	High	High	High	1
High	High	High	High	High	High	High	High	1
High	High	High	High	High	High	High	Peak Foun	1
Peak Foun	Peak Foun	Peak Foun	Peak Foun	Peak Foun	High	High	High	1
Peak Foun	High	Peak Foun	High	High	High	High	High	1
Peak Foun	Peak Foun	Peak Foun	High	High	High	High	High	1
Peak Foun	Peak Foun	Peak Foun	Peak Foun	Peak Foun	High	High	Peak Foun	1
Peak Foun	High	High	Peak Foun	Not Found	Peak Foun	Peak Foun	Not Found	1
Peak Foun	High	Peak Foun	High	Peak Foun	High	Peak Foun	Peak Foun	1
Not Found	Peak Foun	High	High	Peak Foun	High	High	High	1
Peak Foun	Peak Foun	High	High	High	High	High	High	1
Peak Foun	Peak Foun	Peak Foun	High	High	High	High	Peak Foun	1
Peak Foun	Peak Foun	Peak Foun	Peak Foun	High	High	High	Peak Foun	1
High	High	High	Peak Foun	High	High	High	High	1

High	High	High	High	High	High	High	Peak Foun	1
High	Peak Foun	High	High	High	High	High	High	1
High	Peak Foun	High	High	High	Peak Foun	Peak Foun	High	1
High	High	High	Peak Foun	High	High	High	High	1
Peak Foun	Peak Foun	Peak Foun	Peak Foun	High	High	High	High	1
High	High	High	High	High	High	High	High	1
Peak Foun	Peak Foun	High	Peak Foun	High	High	Peak Foun	Peak Foun	1
Peak Foun	Peak Foun	High	Peak Foun	Peak Foun	High	High	Peak Foun	1
Peak Foun	High	High	Peak Foun	High	High	High	High	1
Peak Foun	Peak Foun	High	Not Found	High	High	High	Peak Foun	1
Peak Foun	Not Found	Peak Foun	Peak Foun	High	High	High	High	1
Peak Foun	Peak Foun	Peak Foun	High	High	High	High	High	1
High	Peak Foun	Peak Foun	High	High	High	High	High	1
Peak Foun	Not Found	Not Found	Not Found	High	High	High	High	1
Peak Foun	Peak Foun	High	Peak Foun	Peak Foun	High	High	High	1
High	High	Peak Foun	High	High	High	Peak Foun	High	1
Peak Foun	High	Peak Foun	High	High	High	High	High	1
Peak Foun	Peak Foun	Peak Foun	Peak Foun	Peak Foun	High	High	Peak Foun	1
High	High	High	High	High	High	High	High	1
High	High	High	High	High	High	High	Peak Foun	1
Peak Foun	Peak Foun	Peak Foun	High	High	High	Peak Foun	Peak Foun	1
High	Peak Foun	Peak Foun	High	High	High	Peak Foun	Peak Foun	1
High	High	Peak Foun	Not Found	High	High	High	Peak Foun	1
Peak Foun	High	High	High	High	High	High	High	1
High	Peak Foun	High	High	High	High	High	High	1
High	High	High	High	Peak Foun	High	High	High	1
High	High	High	High	High	High	High	High	1
Peak Foun	Peak Foun	Not Found	Peak Foun	Peak Foun	Peak Foun	High	High	1
Not Found	Peak Foun	High	Not Found	Peak Foun	Peak Foun	High	Peak Foun	1
High	Peak Foun	High	Peak Foun	High	Peak Foun	High	High	1
High	High	Peak Foun	High	High	High	High	High	1
Peak Foun	Peak Foun	Peak Foun	Peak Foun	Peak Foun	High	Peak Foun	Not Found	1
High	High	High	Peak Foun	High	High	Peak Foun	Peak Foun	1
Peak Foun	Peak Foun	Peak Foun	Peak Foun	High	High	High	Peak Foun	1
High	High	High	High	High	High	High	High	1
High	High	High	High	High	High	High	High	1
Peak Foun	Peak Foun	Peak Foun	Peak Foun	Peak Foun	High	High	Peak Foun	1
Not Found	High	Not Found	Not Found	Peak Foun	Not Found	Peak Foun	High	1
High	High	Peak Foun	Peak Foun	High	Peak Foun	Peak Foun	Peak Foun	1
High	High	High	High	High	High	High	High	1
High	High	High	High	High	High	High	High	1
High	High	Peak Foun	Peak Foun	High	High	Peak Foun	Peak Foun	1
High	Peak Foun	High	Peak Foun	High	High	Peak Foun	High	1
High	High	High	High	High	High	High	High	1
Peak Foun	Peak Foun	Peak Foun	Peak Foun	High	High	High	Peak Foun	1

Peak Foun	Peak Foun	High	High	Peak Foun	High	High	High	1
Peak Foun	High	High	High	High	High	High	Peak Foun	1
High	High	High	Peak Foun	High	Peak Foun	Peak Foun	Peak Foun	1
High	Peak Foun	High	Peak Foun	High	Peak Foun	High	Peak Foun	1
High	High	High	Peak Foun	High	High	High	Peak Foun	1
Peak Foun	High	Peak Foun	High	High	High	High	Peak Foun	1
High	Peak Foun	High	High	High	High	High	High	1
High	High	High	Peak Foun	High	High	Peak Foun	Peak Foun	1
High	Peak Foun	High	High	High	High	High	High	1
Peak Foun	High	Peak Foun	Peak Foun	Peak Foun	High	High	High	1
High	High	High	High	High	High	High	High	1
High	High	High	High	High	High	High	High	1
Peak Foun	Peak Foun	Peak Foun	Peak Foun	Peak Foun	High	High	Peak Foun	1
Peak Foun	Peak Foun	Peak Foun	Peak Foun	Peak Foun	High	Peak Foun	Peak Foun	1
Peak Foun	High	High	Peak Foun	Peak Foun	High	High	Peak Foun	1
High	High	High	High	High	High	High	High	1
High	Peak Foun	Peak Foun	Peak Foun	High	High	High	Peak Foun	1
Peak Foun	Peak Foun	Peak Foun	Peak Foun	Peak Foun	High	High	Peak Foun	1
High	Peak Foun	Peak Foun	Peak Foun	High	High	Peak Foun	Peak Foun	1
High	Peak Foun	High	High	High	High	High	High	1
Peak Foun	High	Peak Foun	High	High	High	High	High	1
High	High	Peak Foun	High	High	Peak Foun	Peak Foun	Peak Foun	1
Peak Foun	Peak Foun	Peak Foun	Peak Foun	Peak Foun	High	High	Peak Foun	1
High	High	Peak Foun	Peak Foun	High	Peak Foun	Peak Foun	Peak Foun	1
Peak Foun	Peak Foun	Peak Foun	High	High	High	Peak Foun	Peak Foun	1
High	High	High	High	High	High	High	High	1
High	High	High	High	High	High	Peak Foun	Peak Foun	1
Peak Foun	High	Peak Foun	Peak Foun	High	High	High	High	1
High	High	High	High	High	High	High	High	1
High	High	High	High	High	Peak Foun	High	High	1
High	Peak Foun	High	Peak Foun	Peak Foun	Peak Foun	Peak Foun	Not Found	1
Peak Foun	High	Peak Foun	High	High	High	High	Peak Foun	1
Peak Foun	Peak Foun	High	Peak Foun	High	High	High	High	1
High	High	High	High	High	High	High	High	1
High	High	High	High	High	High	High	High	1
Peak Foun	Peak Foun	Peak Foun	Peak Foun	Peak Foun	High	Peak Foun	Peak Foun	1
Peak Foun	High	High	High	High	High	High	High	1
High	Peak Foun	Peak Foun	Peak Foun	High	High	Peak Foun	High	1
Peak Foun	High	High	High	High	High	High	Peak Foun	1
Peak Foun	Peak Foun	Peak Foun	High	High	High	High	High	1
High	High	High	High	High	High	High	Peak Foun	1
Peak Foun	Peak Foun	High	Peak Foun	High	High	High	High	1
High	High	High	Peak Foun	High	High	High	High	1
High	High	High	High	Peak Foun	High	Peak Foun	Peak Foun	1
Not Found	Not Found	Not Found	Not Found	Peak Foun	High	High	Not Found	1

High	High	High	High	High	High	High	Peak Foun	1
Peak Foun	High	Peak Foun	High	High	High	High	High	1
Peak Foun	Peak Foun	Peak Foun	High	Peak Foun	High	Peak Foun	Peak Foun	1
Peak Foun	Peak Foun	Peak Foun	Peak Foun	High	High	High	Peak Foun	1
Peak Foun	Peak Foun	High	Peak Foun	High	High	High	Not Found	1
Peak Foun	High	High	High	High	High	High	High	1
Peak Foun	High	Peak Foun	Peak Foun	High	Peak Foun	Peak Foun	Peak Foun	1
Peak Foun	Not Found	Peak Foun	Peak Foun	Peak Foun	Peak Foun	High	Peak Foun	1
High	High	High	High	High	High	High	High	1
High	Peak Foun	High	High	High	High	High	High	1
Peak Foun	Peak Foun	High	Peak Foun	High	High	High	Peak Foun	1
Peak Foun	Peak Foun	High	Peak Foun	High	High	High	Peak Foun	1
High	High	High	High	High	High	High	High	1
High	High	High	Peak Foun	High	High	Peak Foun	Peak Foun	1
Peak Foun	Peak Foun	Peak Foun	Peak Foun	Peak Foun	High	Peak Foun	High	1
Peak Foun	Peak Foun	High	Peak Foun	High	High	High	High	1
Peak Foun	Peak Foun	Peak Foun	Peak Foun	Peak Foun	Peak Foun	High	Peak Foun	1
Peak Foun	High	High	High	High	High	High	High	1
Peak Foun	High	High	High	Peak Foun	Peak Foun	Peak Foun	High	1
High	High	High	High	High	High	High	High	1
Peak Foun	High	Peak Foun	Peak Foun	Peak Foun	High	Peak Foun	Peak Foun	1
Peak Foun	Peak Foun	Peak Foun	High	High	High	High	High	1
High	High	Peak Foun	High	High	High	High	High	1
High	High	High	Peak Foun	High	Peak Foun	High	High	1
High	Peak Foun	Peak Foun	High	High	Peak Foun	High	High	1
Peak Foun	High	Peak Foun	High	High	High	Peak Foun	Peak Foun	1
Peak Foun	Peak Foun	Peak Foun	Peak Foun	High	Peak Foun	Peak Foun	Peak Foun	1
Peak Foun	Peak Foun	High	Peak Foun	Peak Foun	Peak Foun	High	Peak Foun	1
Peak Foun	Not Found	High	Peak Foun	High	High	High	High	1
Peak Foun	Peak Foun	High	High	High	High	High	High	1
High	Peak Foun	High	High	High	High	High	High	1
High	High	High	High	High	High	Peak Foun	Peak Foun	1
High	High	High	High	High	High	High	High	1
High	High	Peak Foun	Peak Foun	High	High	High	High	1
High	High	High	High	High	High	High	High	1
High	High	High	High	High	High	Peak Foun	Peak Foun	1
High	High	High	High	High	High	High	High	1
High	High	High	High	High	Peak Foun	High	High	1
Peak Foun	High	High	High	Peak Foun	High	Peak Foun	Peak Foun	1
Peak Foun	High	High	Peak Foun	High	High	High	High	1
Peak Foun	Peak Foun	Peak Foun	Peak Foun	Peak Foun	Peak Foun	High	High	1
Not Found	High	High	Peak Foun	High	High	High	High	1
Peak Foun	High	High	High	High	High	High	High	1
High	High	High	High	High	High	High	Peak Foun	1
Not Found	Not Found	Peak Foun	High	Peak Foun	Peak Foun	High	Peak Foun	1

High	Peak Foun	Peak Foun	High	High	Peak Foun	Peak Foun	High	1
Peak Foun	Peak Foun	Peak Foun	High	High	High	High	Peak Foun	1
Peak Foun	Peak Foun	Peak Foun	Peak Foun	Peak Foun	High	Peak Foun	Peak Foun	1
High	High	High	Peak Foun	High	High	High	Peak Foun	1
Peak Foun	Peak Foun	Peak Foun	Peak Foun	High	High	High	High	1
High	High	High	High	High	High	High	High	1
Peak Foun	Peak Foun	Peak Foun	High	Peak Foun	High	Peak Foun	Peak Foun	1
Peak Foun	High	High	Peak Foun	Peak Foun	High	High	Peak Foun	1
High	High	High	High	High	High	High	High	1
High	High	High	High	High	High	High	High	1
Peak Foun	High	Peak Foun	Peak Foun	Peak Foun	High	Peak Foun	Peak Foun	1
Peak Foun	High	High	Peak Foun	High	High	High	Peak Foun	1
Not Found	High	High	High	High	High	Peak Foun	High	1
High	High	High	High	High	High	High	High	1
High	High	High	High	High	High	High	High	1
Peak Foun	Peak Foun	High	Peak Foun	Peak Foun	High	High	High	1
High	High	High	High	High	High	High	High	1
Peak Foun	Peak Foun	Peak Foun	Peak Foun	High	High	High	High	1
Peak Foun	Peak Foun	Peak Foun	Peak Foun	High	High	High	High	1
Peak Foun	High	Peak Foun	High	Peak Foun	High	High	Peak Foun	1
Peak Foun	Peak Foun	High	Peak Foun	High	Peak Foun	Peak Foun	Peak Foun	1
High	High	High	High	High	High	High	High	1
Peak Foun	High	High	Peak Foun	Peak Foun	High	High	Not Found	1
Not Found	Peak Foun	High	Peak Foun	High	High	Peak Foun	Not Found	1
High	High	High	High	High	High	High	Peak Foun	1
Peak Foun	Peak Foun	Peak Foun	Peak Foun	Peak Foun	High	Peak Foun	Peak Foun	1
Peak Foun	Peak Foun	Peak Foun	Peak Foun	High	Peak Foun	Peak Foun	Peak Foun	1
Not Found	Peak Foun	Peak Foun	High	High	High	High	High	1
Not Found	Not Found	Not Found	Peak Foun	High	Peak Foun	Peak Foun	Peak Foun	1
High	Peak Foun	Peak Foun	Peak Foun	High	High	High	High	1
High	Peak Foun	High	High	High	High	High	High	1
Peak Foun	Peak Foun	Peak Foun	Peak Foun	High	High	High	Peak Foun	1
Peak Foun	High	High	High	High	High	High	High	1
Not Found	Not Found	Not Found	Not Found	Peak Foun	High	High	Not Found	1
Peak Foun	High	High	Peak Foun	High	High	High	High	1
Peak Foun	Peak Foun	Peak Foun	Peak Foun	High	High	Peak Foun	Peak Foun	1
Peak Foun	Peak Foun	Peak Foun	Peak Foun	High	High	High	Not Found	1
Not Found	Not Found	Not Found	Not Found	Not Found	Not Found	Not Found	Not Found	1
Peak Foun	High	High	High	Peak Foun	Not Found	Not Found	Peak Foun	1
Peak Foun	Peak Foun	Peak Foun	Peak Foun	High	High	High	Peak Foun	1
Peak Foun	Peak Foun	High	High	High	High	High	High	1
High	Peak Foun	Peak Foun	High	High	High	High	High	1
High	High	Peak Foun	Peak Foun	High	High	High	Peak Foun	1
Peak Foun	Peak Foun	Peak Foun	Peak Foun	Peak Foun	High	Peak Foun	Peak Foun	1
Peak Foun	High	High	High	High	High	High	High	1

Not Found	Peak Foun	Peak Foun	Peak Foun	Peak Foun	High	High	Peak Foun	1
High	High	High	High	High	High	High	High	1
Peak Foun	Peak Foun	Peak Foun	High	High	Peak Foun	High	Peak Foun	1
Peak Foun	Peak Foun	High	Peak Foun	High	High	High	Peak Foun	1
Peak Foun	Peak Foun	High	Peak Foun	Peak Foun	High	High	High	1
High	High	High	High	High	Peak Foun	High	Peak Foun	1
Peak Foun	Peak Foun	Peak Foun	Peak Foun	High	High	High	Peak Foun	1
High	High	High	High	High	High	High	High	1
Peak Foun	Peak Foun	Peak Foun	Peak Foun	Peak Foun	High	High	Peak Foun	1
Peak Foun	High	High	Peak Foun	High	High	High	High	1
Peak Foun	Peak Foun	High	High	Peak Foun	Peak Foun	High	High	1
Peak Foun	High	Peak Foun	High	High	High	Peak Foun	Peak Foun	1
Not Found	Not Found	Not Found	Peak Foun	Peak Foun	High	Peak Foun	Peak Foun	1
High	Peak Foun	High	Peak Foun	Peak Foun	High	High	High	1
Peak Foun	Not Found	Peak Foun	Peak Foun	High	Peak Foun	High	Peak Foun	1
High	High	High	High	High	High	High	High	1
Peak Foun	Peak Foun	Peak Foun	Peak Foun	Peak Foun	High	Peak Foun	Peak Foun	1
High	Peak Foun	Peak Foun	High	High	Peak Foun	High	Peak Foun	1
Peak Foun	High	Peak Foun	Peak Foun	Peak Foun	High	High	Peak Foun	1
High	High	High	High	High	High	High	High	1
High	High	High	High	High	High	High	High	1
High	High	High	Peak Foun	High	High	High	High	1
High	Peak Foun	High	Peak Foun	High	High	High	Peak Foun	1
Peak Foun	High	Peak Foun	Peak Foun	Peak Foun	High	High	High	1
Peak Foun	Not Found	High	Peak Foun	High	High	High	Peak Foun	1
Peak Foun	Peak Foun	High	High	High	High	High	Peak Foun	1
Peak Foun	Peak Foun	Peak Foun	High	Peak Foun	High	High	High	1
Not Found	High	Peak Foun	High	Peak Foun	High	High	Peak Foun	1
Not Found	High	High	Peak Foun	High	High	High	High	1
High	Peak Foun	High	High	High	High	High	Peak Foun	1
High	Peak Foun	High	Peak Foun	High	High	High	Peak Foun	1
Not Found	Peak Foun	Peak Foun	High	Not Found	High	Peak Foun	Not Found	1
High	High	High	Peak Foun	High	High	Peak Foun	Peak Foun	1
Peak Foun	Peak Foun	Peak Foun	Peak Foun	High	Peak Foun	Peak Foun	Peak Foun	1
Peak Foun	High	High	High	Peak Foun	High	High	High	1
High	High	High	High	High	High	High	High	1
Peak Foun	Peak Foun	Peak Foun	Peak Foun	High	High	High	Peak Foun	1
Peak Foun	High	Peak Foun	High	Peak Foun	Peak Foun	High	High	1
Peak Foun	Peak Foun	High	High	High	High	High	High	1
High	Peak Foun	High	Peak Foun	High	High	High	Peak Foun	1
High	High	High	Peak Foun	High	High	High	Peak Foun	1
High	High	High	High	High	High	High	Peak Foun	1
High	High	High	High	High	High	High	Peak Foun	1
Peak Foun	Peak Foun	High	Peak Foun	High	Peak Foun	High	Peak Foun	1
High	High	High	High	High	High	High	High	1

Not Found	High	Peak Foun	High	High	High	High	High	1
Peak Foun	Peak Foun	High	High	High	High	High	Peak Foun	1
High	High	Peak Foun	Peak Foun	High	High	Peak Foun	Peak Foun	1
High	High	High	Peak Foun	High	High	High	Not Found	1
High	Peak Foun	Peak Foun	Peak Foun	High	High	High	Peak Foun	1
Not Found	Peak Foun	High	High	High	High	Peak Foun	High	1
Peak Foun	High	Peak Foun	High	High	High	Peak Foun	High	1
High	High	High	High	High	High	High	Peak Foun	1
High	High	Peak Foun	Peak Foun	High	High	Peak Foun	Peak Foun	1
Not Found	Not Found	Not Found	Peak Foun	Peak Foun	High	Peak Foun	Peak Foun	1
High	High	High	Peak Foun	High	High	High	Peak Foun	1
High	High	High	High	High	High	High	High	1
High	High	Peak Foun	High	High	High	High	Peak Foun	1
High	High	High	High	High	High	High	High	1
Peak Foun	Peak Foun	High	High	High	High	High	High	1
High	High	High	High	High	High	High	High	1
Peak Foun	Peak Foun	Peak Foun	High	High	High	High	Peak Foun	1
Not Found	Not Found	Peak Foun	Not Found	Peak Foun	High	High	Peak Foun	1
High	High	High	High	High	High	High	High	1
Peak Foun	Peak Foun	Peak Foun	High	Peak Foun	High	High	Peak Foun	1
Peak Foun	High	High	Peak Foun	High	High	Peak Foun	High	1
Peak Foun	High	High	High	Peak Foun	High	High	High	1
Peak Foun	Peak Foun	High	Peak Foun	Peak Foun	Peak Foun	High	Peak Foun	1
Peak Foun	Peak Foun	Peak Foun	Peak Foun	High	High	High	High	1
Peak Foun	High	High	Peak Foun	High	High	High	High	1
Peak Foun	High	High	High	High	High	High	High	1
High	High	High	Peak Foun	High	High	High	Peak Foun	1
High	High	High	High	High	High	High	Peak Foun	1
Peak Foun	High	Peak Foun	Peak Foun	Peak Foun	Peak Foun	High	Peak Foun	1
Not Found	Not Found	Not Found	Peak Foun	High	High	High	Peak Foun	1
Peak Foun	Peak Foun	Peak Foun	High	High	High	High	High	1
High	High	High	High	High	High	High	High	1
Peak Foun	Peak Foun	Peak Foun	Peak Foun	Peak Foun	High	Peak Foun	Peak Foun	1
Peak Foun	Peak Foun	Peak Foun	High	Peak Foun	High	Peak Foun	Peak Foun	1
High	High	High	High	High	High	High	High	1
Peak Foun	Peak Foun	Peak Foun	Peak Foun	High	Peak Foun	High	Peak Foun	1
Peak Foun	Peak Foun	Peak Foun	High	High	High	High	High	1
High	Peak Foun	High	Peak Foun	High	Peak Foun	Peak Foun	Peak Foun	1
High	Peak Foun	Peak Foun	Peak Foun	High	Peak Foun	Peak Foun	Peak Foun	1
Not Found	Not Found	Peak Foun	Peak Foun	High	High	High	Peak Foun	1
Peak Foun	Not Found	Peak Foun	Peak Foun	Peak Foun	High	High	Peak Foun	1
High	High	Peak Foun	High	High	High	High	High	1
High	High	High	High	High	High	High	High	1
Peak Foun	Peak Foun	High	Peak Foun	Peak Foun	High	Peak Foun	High	1
High	High	High	High	High	High	High	High	1

High	High	High	High	High	High	High	High	1
Not Found	Not Found	Not Found	Not Found	High	High	Peak Foun	Not Found	1
Peak Foun	High	Peak Foun	Peak Foun	Peak Foun	High	High	Peak Foun	1
Not Found	Peak Foun	Peak Foun	Not Found	Peak Foun	Peak Foun	High	Peak Foun	1
Peak Foun	Peak Foun	Not Found	Not Found	Peak Foun	High	Peak Foun	Not Found	1
Peak Foun	Peak Foun	High	Peak Foun	Peak Foun	High	High	Peak Foun	1
High	High	High	High	High	High	High	High	1
High	Peak Foun	High	High	High	High	High	Peak Foun	1
High	High	High	High	High	High	High	Peak Foun	1
High	High	Peak Foun	Peak Foun	High	High	Peak Foun	Peak Foun	1
Peak Foun	High	Peak Foun	Not Found	Peak Foun	High	Peak Foun	Not Found	1
High	High	Peak Foun	Peak Foun	High	High	Peak Foun	Peak Foun	1
High	High	High	High	Peak Foun	High	Peak Foun	Peak Foun	1
Not Found	Peak Foun	Not Found	Not Found	Not Found	Peak Foun	Not Found	Not Found	1
Peak Foun	High	High	Peak Foun	High	High	Peak Foun	Peak Foun	1
Peak Foun	Peak Foun	High	Peak Foun	Peak Foun	Peak Foun	High	Peak Foun	1
High	High	High	Peak Foun	Peak Foun	Peak Foun	Peak Foun	Peak Foun	1
Peak Foun	High	Peak Foun	Peak Foun	High	High	High	High	1
Peak Foun	Peak Foun	Peak Foun	Peak Foun	High	High	Peak Foun	Peak Foun	1
High	High	High	High	High	High	High	High	1
High	Peak Foun	High	High	High	High	High	High	1
Not Found	Not Found	Peak Foun	Not Found	High	Peak Foun	High	Peak Foun	1
Peak Foun	Peak Foun	Peak Foun	High	Peak Foun	High	High	Peak Foun	1
Peak Foun	High	Not Found	Peak Foun	High	High	High	Peak Foun	1
Peak Foun	High	Peak Foun	Peak Foun	Peak Foun	Peak Foun	High	Not Found	1
High	High	High	High	High	High	High	High	1
Peak Foun	High	High	Peak Foun	High	Peak Foun	Peak Foun	Peak Foun	1
Peak Foun	High	Peak Foun	Peak Foun	Peak Foun	High	Peak Foun	Peak Foun	1
Peak Foun	Peak Foun	Peak Foun	High	High	High	High	Peak Foun	1
Peak Foun	Peak Foun	Peak Foun	High	Peak Foun	High	High	Peak Foun	1
High	High	High	High	High	High	High	High	1
High	Peak Foun	High	High	High	High	High	Peak Foun	1
Peak Foun	High	Peak Foun	Peak Foun	High	High	Peak Foun	Peak Foun	1
Peak Foun	High	Peak Foun	High	High	High	Peak Foun	Not Found	1
Peak Foun	Peak Foun	Peak Foun	Peak Foun	High	High	High	High	1
High	High	High	Peak Foun	High	High	High	Peak Foun	1
Peak Foun	Peak Foun	Peak Foun	High	High	High	High	High	1
Peak Foun	Peak Foun	Peak Foun	Peak Foun	Peak Foun	High	High	High	1
Peak Foun	High	High	High	High	High	High	High	1
Peak Foun	Peak Foun	Peak Foun	Peak Foun	Peak Foun	Peak Foun	Peak Foun	High	1
High	High	High	High	High	High	High	High	1
High	Peak Foun	Peak Foun	Peak Foun	High	High	Peak Foun	Peak Foun	1
High	Peak Foun	Peak Foun	High	High	High	High	High	1
High	High	Peak Foun	High	Peak Foun	High	High	High	1
Peak Foun	High	Peak Foun	Peak Foun	Peak Foun	High	Peak Foun	Peak Foun	1

Peak Foun	Peak Foun	Peak Foun	Peak Foun	High	Peak Foun	High	High	1
Peak Foun	Not Found	High	Peak Foun	High	High	High	Peak Foun	1
High	Peak Foun	Peak Foun	High	High	High	Peak Foun	High	1
Peak Foun	Peak Foun	Peak Foun	Peak Foun	High	High	High	Peak Foun	1
Peak Foun	Peak Foun	Peak Foun	Peak Foun	High	High	High	Peak Foun	1
High	High	High	High	High	Not Found	High	High	1
Peak Foun	Peak Foun	High	High	High	High	High	High	1
High	High	High	High	Peak Foun	Not Found	High	High	1
Peak Foun	Peak Foun	Peak Foun	Peak Foun	High	High	High	Peak Foun	1
High	High	High	High	Peak Foun	High	High	Peak Foun	1
Peak Foun	High	Peak Foun	Peak Foun	Peak Foun	Peak Foun	Peak Foun	Peak Foun	1
High	High	High	Peak Foun	Peak Foun	Peak Foun	Peak Foun	Peak Foun	1
Peak Foun	High	Peak Foun	High	Peak Foun	Not Found	Not Found	Peak Foun	1
Peak Foun	High	Peak Foun	Peak Foun	High	High	High	High	1
High	High	High	Peak Foun	High	High	High	Peak Foun	1
Peak Foun	Peak Foun	Peak Foun	High	Peak Foun	High	High	Peak Foun	1
High	Peak Foun	Peak Foun	High	High	High	High	High	1
Peak Foun	High	High	High	High	High	High	High	1
High	High	High	High	High	High	High	High	1
High	High	High	High	High	High	High	High	1
High	High	High	High	High	High	High	Not Found	1
High	High	High	High	High	High	High	High	1
Peak Foun	High	High	Peak Foun	High	Peak Foun	High	High	1
High	High	High	High	High	High	High	High	1
Peak Foun	Peak Foun	Peak Foun	Peak Foun	High	High	Peak Foun	Peak Foun	1
Not Found	Not Found	Peak Foun	Peak Foun	Peak Foun	High	High	Peak Foun	1
High	High	High	High	High	High	High	High	1
Peak Foun	Peak Foun	Peak Foun	High	High	High	Peak Foun	Peak Foun	1
Peak Foun	Peak Foun	Peak Foun	Peak Foun	Peak Foun	High	High	Not Found	1
High	High	High	High	High	High	High	High	1
Peak Foun	Peak Foun	High	Peak Foun	Peak Foun	High	High	Peak Foun	1
High	Peak Foun	Peak Foun	Peak Foun	High	High	High	Peak Foun	1
Peak Foun	Peak Foun	High	Peak Foun	Peak Foun	High	Peak Foun	High	1
Not Found	Peak Foun	Not Found	Not Found	Not Found	Peak Foun	High	Not Found	1
High	Peak Foun	Peak Foun	High	High	High	Peak Foun	Peak Foun	1
Peak Foun	Peak Foun	High	High	High	High	High	High	1
Peak Foun	Peak Foun	Peak Foun	High	High	Peak Foun	High	Peak Foun	1
Peak Foun	High	Peak Foun	Peak Foun	High	High	High	Peak Foun	1
High	High	High	High	High	High	High	High	1
Peak Foun	Peak Foun	Peak Foun	Peak Foun	High	Peak Foun	Peak Foun	Peak Foun	1
Peak Foun	High	High	Peak Foun	Peak Foun	High	Peak Foun	Peak Foun	1
High	High	High	High	Peak Foun	High	High	Peak Foun	1
High	High	High	Peak Foun	Not Found	Not Found	Not Found	Peak Foun	1
Peak Foun	Peak Foun	High	Peak Foun	High	High	High	Peak Foun	1
Not Found	Not Found	High	High	High	High	High	Peak Foun	1

Peak Foun	Not Found	Not Found	Peak Foun	High	High	High	Peak Foun	1
Peak Foun	Peak Foun	Peak Foun	High	Peak Foun	High	Peak Foun	Peak Foun	1
High	High	Peak Foun	High	High	High	High	High	1
Peak Foun	Peak Foun	Peak Foun	Peak Foun	Peak Foun	High	Peak Foun	Peak Foun	1
High	High	High	High	High	High	High	High	1
Peak Foun	High	High	High	Peak Foun	High	Peak Foun	High	1
Peak Foun	Peak Foun	High	Peak Foun	Peak Foun	Peak Foun	Peak Foun	Peak Foun	1
Peak Foun	High	High	Peak Foun	Peak Foun	High	High	High	1
High	High	High	High	High	High	High	High	1
Peak Foun	High	Peak Foun	Peak Foun	Peak Foun	Peak Foun	High	Peak Foun	1
Peak Foun	Peak Foun	High	High	Peak Foun	High	High	High	1
High	High	High	Peak Foun	High	High	High	High	1
Peak Foun	Peak Foun	High	Peak Foun	High	High	High	Peak Foun	1
Peak Foun	Peak Foun	Peak Foun	Peak Foun	High	Peak Foun	High	Peak Foun	1
Not Found	Peak Foun	Peak Foun	High	Peak Foun	High	High	High	1
Peak Foun	Peak Foun	Peak Foun	High	Peak Foun	High	High	Not Found	1
Peak Foun	High	Peak Foun	High	High	High	High	Peak Foun	1
Peak Foun	Peak Foun	Peak Foun	Peak Foun	Peak Foun	High	High	Peak Foun	1
Peak Foun	Peak Foun	Peak Foun	Peak Foun	High	High	High	Peak Foun	1
Not Found	High	Not Found	Peak Foun	High	High	High	High	1
High	High	High	Peak Foun	High	High	High	High	1
High	High	High	High	High	High	High	High	1
Peak Foun	Peak Foun	High	Peak Foun	Peak Foun	Peak Foun	High	Peak Foun	1
Peak Foun	Peak Foun	High	Peak Foun	High	Peak Foun	Peak Foun	Peak Foun	1
Peak Foun	Peak Foun	High	Peak Foun	Peak Foun	Peak Foun	High	High	1
Peak Foun	Peak Foun	High	High	Peak Foun	Peak Foun	High	Peak Foun	1
Peak Foun	Peak Foun	Peak Foun	Peak Foun	High	High	High	Peak Foun	1
Peak Foun	Peak Foun	Peak Foun	Peak Foun	Peak Foun	Peak Foun	Peak Foun	Peak Foun	1
Peak Foun	Peak Foun	Peak Foun	High	High	Peak Foun	High	Peak Foun	1
High	Peak Foun	Not Found	Peak Foun	High	High	Peak Foun	Peak Foun	1
Peak Foun	Peak Foun	High	Peak Foun	High	High	High	Peak Foun	1
Peak Foun	High	High	Peak Foun	High	High	High	High	1
Peak Foun	Not Found	Peak Foun	Peak Foun	Peak Foun	Peak Foun	Peak Foun	Peak Foun	1
Peak Foun	Peak Foun	Peak Foun	Peak Foun	High	High	High	Peak Foun	1
High	Peak Foun	Peak Foun	High	High	High	Peak Foun	Peak Foun	1
High	High	Peak Foun	High	High	Peak Foun	Peak Foun	Peak Foun	1
High	High	High	Peak Foun	High	High	Peak Foun	High	1
High	Not Found	Peak Foun	Peak Foun	Peak Foun	Peak Foun	Peak Foun	Peak Foun	1
High	High	High	High	High	High	High	High	1
High	High	High	High	High	High	High	Peak Foun	1
High	Peak Foun	High	Peak Foun	High	High	High	Peak Foun	1
Peak Foun	Peak Foun	High	High	Peak Foun	High	Peak Foun	Peak Foun	1
Not Found	Peak Foun	Peak Foun	Peak Foun	Peak Foun	High	High	Peak Foun	1
High	High	High	High	High	High	High	High	1
Peak Foun	Peak Foun	Peak Foun	Peak Foun	High	High	High	Peak Foun	1

High	High	High	Peak Foun	High	High	High	Peak Foun	1
High	High	High	Peak Foun	High	High	High	Peak Foun	1
Peak Foun	Peak Foun	Peak Foun	Peak Foun	Peak Foun	High	Peak Foun	Peak Foun	1
High	High	High	High	High	High	High	High	1
Peak Foun	Peak Foun	High	Peak Foun	Peak Foun	High	High	Peak Foun	1
Peak Foun	High	Peak Foun	Peak Foun	Peak Foun	Peak Foun	Peak Foun	Peak Foun	1
Peak Foun	Peak Foun	Peak Foun	Peak Foun	High	High	High	Peak Foun	1
High	High	Peak Foun	Peak Foun	High	High	High	Peak Foun	1
High	High	High	Peak Foun	Peak Foun	High	High	Peak Foun	1
Peak Foun	Peak Foun	Peak Foun	High	High	High	High	High	1
Peak Foun	Peak Foun	Peak Foun	Peak Foun	High	Peak Foun	Peak Foun	Peak Foun	1
Not Found	Not Found	Not Found	Not Found	High	High	High	Peak Foun	1
High	High	High	High	High	High	High	High	1
Peak Foun	Peak Foun	Peak Foun	Not Found	Peak Foun	High	Peak Foun	Not Found	1
High	High	High	High	High	Peak Foun	High	High	1
Peak Foun	High	High	High	Peak Foun	High	Peak Foun	High	1
High	High	High	High	High	High	High	High	1
Peak Foun	Peak Foun	Peak Foun	Peak Foun	High	High	Peak Foun	Peak Foun	1
Peak Foun	Peak Foun	High	Peak Foun	High	High	High	High	1
High	Peak Foun	Peak Foun	Peak Foun	Peak Foun	High	Peak Foun	Peak Foun	1
Peak Foun	Not Found	Not Found	Peak Foun	High	High	Not Found	Not Found	1
Peak Foun	Not Found	Peak Foun	Peak Foun	High	High	High	Peak Foun	1
Peak Foun	High	High	Peak Foun	Peak Foun	High	Peak Foun	Peak Foun	1
Peak Foun	High	High	Peak Foun	Peak Foun	High	Peak Foun	Peak Foun	1
Peak Foun	Peak Foun	Peak Foun	High	Peak Foun	Peak Foun	Peak Foun	Peak Foun	1
Peak Foun	High	High	High	High	High	High	High	1
Peak Foun	Peak Foun	Peak Foun	Not Found	High	High	High	Not Found	1
Peak Foun	Peak Foun	High	High	High	High	High	Peak Foun	1
Not Found	Not Found	Not Found	Peak Foun	High	High	Peak Foun	Peak Foun	1
Peak Foun	Peak Foun	High	High	High	High	High	High	1
Peak Foun	Peak Foun	Peak Foun	Peak Foun	High	High	High	High	1
High	High	High	Peak Foun	High	High	High	Peak Foun	1
High	High	High	Peak Foun	High	High	High	Peak Foun	1
Peak Foun	High	High	Peak Foun	Peak Foun	High	High	High	1
Peak Foun	Not Found	Peak Foun	Peak Foun	High	High	High	Peak Foun	1
Not Found	Not Found	Not Found	Not Found	Peak Foun	High	Not Found	Not Found	1
High	High	High	High	High	High	High	High	1
Peak Foun	Not Found	Not Found	Not Found	High	High	Not Found	Peak Foun	1
High	Peak Foun	Peak Foun	Peak Foun	High	High	High	Peak Foun	1
Peak Foun	Peak Foun	Peak Foun	Peak Foun	High	High	High	High	1
Peak Foun	Peak Foun	Peak Foun	Peak Foun	Peak Foun	Peak Foun	Peak Foun	Peak Foun	1
Peak Foun	High	High	High	High	High	High	Peak Foun	1
High	High	High	High	Peak Foun	High	High	High	1
High	High	High	Peak Foun	High	High	High	High	1
High	High	High	High	High	High	High	High	1

Peak Foun	Not Found	Not Found	High	Peak Foun	High	Not Found	Peak Foun	1
Not Found	Peak Foun	High	High	High	Not Found	High	High	1
Peak Foun	Peak Foun	High	High	Peak Foun	High	Peak Foun	Peak Foun	1
Peak Foun	High	Peak Foun	Peak Foun	High	Peak Foun	Peak Foun	Peak Foun	1
Not Found	Not Found	Not Found	Not Found	Peak Foun	Peak Foun	Peak Foun	Not Found	1
Peak Foun	Peak Foun	Peak Foun	Peak Foun	High	High	High	High	1
High	High	High	High	High	High	High	High	1
Not Found	Peak Foun	Peak Foun	Not Found	Peak Foun	Peak Foun	High	Not Found	1
Peak Foun	Peak Foun	Peak Foun	Peak Foun	Peak Foun	Peak Foun	Peak Foun	High	1
High	Peak Foun	Peak Foun	Peak Foun	High	High	High	Peak Foun	1
Peak Foun	High	Not Found	Peak Foun	Peak Foun	High	Peak Foun	Not Found	1
Peak Foun	High	Peak Foun	Peak Foun	Peak Foun	Peak Foun	High	Peak Foun	1
High	High	High	Peak Foun	High	High	High	High	1
Not Found	Not Found	Not Found	Not Found	Peak Foun	Peak Foun	Not Found	Not Found	1
Peak Foun	Peak Foun	Peak Foun	Peak Foun	Peak Foun	High	High	High	1
High	Peak Foun	High	High	Peak Foun	Peak Foun	High	Peak Foun	1
High	Peak Foun	High	Peak Foun	Peak Foun	High	Peak Foun	Peak Foun	1
High	High	High	Peak Foun	High	High	High	Peak Foun	1
Peak Foun	Not Found	Peak Foun	High	Peak Foun	High	High	Peak Foun	1
High	High	Peak Foun	Peak Foun	High	High	Peak Foun	High	1
Peak Foun	Peak Foun	Peak Foun	Peak Foun	Peak Foun	High	Peak Foun	Peak Foun	1
High	High	High	Peak Foun	Peak Foun	Peak Foun	High	Peak Foun	1
High	Peak Foun	High	High	Peak Foun	High	High	High	1
Not Found	Not Found	Peak Foun	Peak Foun	Not Found	High	Peak Foun	Peak Foun	1
Peak Foun	Peak Foun	Peak Foun	Peak Foun	High	High	High	Peak Foun	1
Not Found	Not Found	Peak Foun	Peak Foun	High	High	Peak Foun	Peak Foun	1
High	High	High	High	High	High	High	High	1
Peak Foun	Peak Foun	Peak Foun	Peak Foun	High	High	High	Peak Foun	1
High	High	High	High	High	High	High	High	1
Not Found	Not Found	Peak Foun	Not Found	Peak Foun	Peak Foun	Peak Foun	Peak Foun	1
High	Peak Foun	Peak Foun	Peak Foun	High	High	High	Peak Foun	1
Peak Foun	High	High	High	High	High	High	Peak Foun	1
Peak Foun	High	High	High	High	High	High	Peak Foun	1
High	Peak Foun	Peak Foun	Peak Foun	High	High	Peak Foun	Peak Foun	1
Peak Foun	Peak Foun	Peak Foun	Peak Foun	Peak Foun	High	Peak Foun	Peak Foun	1
Peak Foun	High	Peak Foun	Peak Foun	High	High	High	Peak Foun	1
Peak Foun	Peak Foun	High	Peak Foun	High	High	High	Peak Foun	1
Peak Foun	High	High	High	High	High	High	Not Found	1
Peak Foun	Not Found	Not Found	Not Found	Peak Foun	Not Found	High	Not Found	1
High	High	High	Peak Foun	High	High	High	High	1
Not Found	Not Found	Not Found	Peak Foun	Peak Foun	Peak Foun	Peak Foun	Peak Foun	1
High	High	High	High	High	High	High	Peak Foun	1
Peak Foun	Not Found	High	Peak Foun	High	High	High	Peak Foun	1
Peak Foun	Peak Foun	Not Found	Peak Foun	Peak Foun	Peak Foun	High	Peak Foun	1
High	High	High	Peak Foun	High	High	High	High	1

Peak Foun	Peak Foun	High	Peak Foun	Peak Foun	Peak Foun	Peak Foun	Peak Foun	1
High	High	Peak Foun	Peak Foun	High	High	High	Peak Foun	1
High	Peak Foun	Peak Foun	High	High	High	High	High	1
High	High	High	High	High	High	High	Peak Foun	1
Peak Foun	Peak Foun	Peak Foun	High	High	High	Peak Foun	Peak Foun	1
Not Found	Peak Foun	Not Found	Peak Foun	High	High	High	High	1
Peak Foun	Not Found	Not Found	Peak Foun	Not Found	High	High	Not Found	1
Peak Foun	Peak Foun	Peak Foun	Peak Foun	High	High	High	High	1
Peak Foun	Peak Foun	Peak Foun	Peak Foun	Peak Foun	High	High	Peak Foun	1
High	High	Peak Foun	Peak Foun	High	High	High	Peak Foun	1
Peak Foun	Peak Foun	High	High	High	High	High	High	1
High	Peak Foun	High	High	High	Peak Foun	High	High	1
High	Peak Foun	Peak Foun	Peak Foun	High	High	High	Peak Foun	1
Peak Foun	High	High	High	High	High	High	High	1
Peak Foun	High	High	High	High	High	High	High	1
Peak Foun	Peak Foun	Peak Foun	High	High	High	Peak Foun	Peak Foun	1
Peak Foun	Not Found	Not Found	High	Peak Foun	High	High	Peak Foun	1
Not Found	Peak Foun	Peak Foun	Peak Foun	High	High	Not Found	Not Found	1
Peak Foun	Peak Foun	Peak Foun	Peak Foun	High	High	High	Peak Foun	1
High	High	High	High	High	High	High	High	1
High	High	High	Peak Foun	Not Found	High	Peak Foun	Peak Foun	1
Peak Foun	High	Peak Foun	Peak Foun	Peak Foun	Peak Foun	Peak Foun	Peak Foun	1
High	High	High	Peak Foun	High	Peak Foun	High	High	1
Peak Foun	Peak Foun	Peak Foun	Peak Foun	High	High	Peak Foun	High	1
Peak Foun	Peak Foun	Peak Foun	Peak Foun	Peak Foun	High	High	Peak Foun	1
Peak Foun	Peak Foun	Peak Foun	Peak Foun	High	High	High	Peak Foun	1
High	Peak Foun	High	Peak Foun	High	High	High	High	1
High	High	Peak Foun	Peak Foun	Peak Foun	Peak Foun	Peak Foun	Peak Foun	1
Peak Foun	Not Found	Peak Foun	High	High	High	High	High	1
High	High	High	High	Peak Foun	High	High	High	1
Peak Foun	Peak Foun	Peak Foun	Peak Foun	Peak Foun	High	High	Peak Foun	1
Peak Foun	Peak Foun	High	Peak Foun	Peak Foun	High	High	Peak Foun	1
Peak Foun	Peak Foun	Peak Foun	Peak Foun	Peak Foun	High	Peak Foun	Peak Foun	1
Peak Foun	Peak Foun	Peak Foun	Peak Foun	High	Peak Foun	Peak Foun	Peak Foun	1
High	High	High	High	High	High	High	High	1
Peak Foun	High	High	High	High	High	High	High	1
Peak Foun	Peak Foun	Peak Foun	Peak Foun	Peak Foun	Peak Foun	Peak Foun	Peak Foun	1
Peak Foun	High	Peak Foun	Peak Foun	High	High	High	Peak Foun	1
Peak Foun	High	High	High	High	High	High	Peak Foun	1
Peak Foun	Not Found	Not Found	High	High	High	High	Peak Foun	1
Peak Foun	Peak Foun	High	High	High	High	Peak Foun	High	1
Not Found	Not Found	Not Found	Peak Foun	Not Found	High	Peak Foun	Peak Foun	1
Not Found	Not Found	Peak Foun	High	Peak Foun	High	High	Peak Foun	1
Peak Foun	High	High	Peak Foun	Peak Foun	High	High	High	1
High	High	High	High	High	High	High	Peak Foun	1

Peak Foun	Peak Foun	Peak Foun	Peak Foun	Peak Foun	High	Peak Foun	Peak Foun	1
High	Peak Foun	High	Peak Foun	High	Not Found	High	Peak Foun	1
Peak Foun	High	High	Peak Foun	Peak Foun	High	High	Peak Foun	1
High	High	High	High	Peak Foun	High	High	Peak Foun	1
Peak Foun	Peak Foun	Peak Foun	Peak Foun	Peak Foun	High	Peak Foun	Peak Foun	1
High	Peak Foun	High	Peak Foun	High	Peak Foun	High	High	1
High	High	Peak Foun	High	High	High	High	High	1
Peak Foun	Peak Foun	Peak Foun	High	High	High	High	High	1
High	High	High	Peak Foun	Peak Foun	Peak Foun	Peak Foun	Peak Foun	1
Not Found	High	High	Peak Foun	Peak Foun	Peak Foun	Peak Foun	Peak Foun	1
Peak Foun	Peak Foun	Peak Foun	Peak Foun	High	High	Peak Foun	Peak Foun	1
Not Found	Not Found	High	Peak Foun	High	High	High	Not Found	1
Peak Foun	High	Peak Foun	Peak Foun	High	High	High	Peak Foun	1
Peak Foun	Not Found	High	Peak Foun	High	High	High	Peak Foun	1
Peak Foun	Peak Foun	High	Peak Foun	Peak Foun	High	High	Peak Foun	1
Peak Foun	Not Found	Peak Foun	High	Peak Foun	High	High	Peak Foun	1
High	High	High	High	High	High	High	High	1
High	Peak Foun	Peak Foun	Peak Foun	Peak Foun	High	Peak Foun	Peak Foun	1
Peak Foun	Peak Foun	High	High	High	High	High	Peak Foun	1
Peak Foun	Peak Foun	High	High	High	High	Peak Foun	Peak Foun	1
High	High	Peak Foun	Not Found	Peak Foun	High	High	Not Found	1
High	Peak Foun	High	Not Found	High	Peak Foun	High	Peak Foun	1
Peak Foun	Peak Foun	High	High	Peak Foun	Peak Foun	High	High	1
High	High	High	High	High	High	Peak Foun	High	1
Peak Foun	Peak Foun	Peak Foun	Peak Foun	High	High	High	Peak Foun	1
Peak Foun	High	Peak Foun	Peak Foun	Peak Foun	Peak Foun	Peak Foun	Peak Foun	1
Peak Foun	Peak Foun	High	High	Peak Foun	High	High	High	1
Peak Foun	Peak Foun	Peak Foun	High	High	High	High	High	1
Peak Foun	Peak Foun	High	Peak Foun	High	High	High	Peak Foun	1
Peak Foun	Peak Foun	Peak Foun	Peak Foun	High	High	Peak Foun	Peak Foun	1
Peak Foun	High	High	High	High	High	Peak Foun	High	1
High	High	Not Found	Not Found	High	High	High	High	1
Peak Foun	Peak Foun	Peak Foun	Peak Foun	Peak Foun	High	Peak Foun	Peak Foun	1
High	Peak Foun	Peak Foun	Peak Foun	Peak Foun	Peak Foun	Peak Foun	Peak Foun	1
Peak Foun	Peak Foun	Peak Foun	Peak Foun	Peak Foun	Peak Foun	High	Peak Foun	1
Peak Foun	Not Found	Peak Foun	Peak Foun	Peak Foun	Peak Foun	Peak Foun	Peak Foun	1
Peak Foun	Peak Foun	Peak Foun	Peak Foun	Peak Foun	High	High	High	1
Peak Foun	High	High	High	High	High	High	High	1
Not Found	Not Found	Peak Foun	Not Found	High	High	High	Not Found	1
Peak Foun	High	High	High	High	High	High	High	1
Peak Foun	Not Found	Peak Foun	Peak Foun	High	High	Peak Foun	Peak Foun	1
Peak Foun	High	High	High	High	High	High	High	1
High	High	High	Peak Foun	Not Found	Not Found	Not Found	Not Found	1
Not Found	Peak Foun	High	High	Not Found	High	High	Peak Foun	1
High	Peak Foun	High	Peak Foun	Peak Foun	High	Peak Foun	Peak Foun	1

Not Found	Peak Foun	Peak Foun	Not Found	Not Found	High	Not Found	Not Found	1
High	High	High	High	High	High	High	Not Found	1
High	High	High	High	High	High	High	High	1
Peak Foun	Peak Foun	High	Peak Foun	Peak Foun	High	High	Peak Foun	1
High	Peak Foun	High	Peak Foun	Peak Foun	High	Peak Foun	Peak Foun	1
Peak Foun	Peak Foun	High	Peak Foun	High	Peak Foun	High	Peak Foun	1
Peak Foun	Peak Foun	High	High	High	High	High	Peak Foun	1
Not Found	Not Found	Peak Foun	Peak Foun	Not Found	High	Peak Foun	Not Found	1
Peak Foun	Peak Foun	Peak Foun	Peak Foun	Peak Foun	Peak Foun	High	Peak Foun	1
Peak Foun	Not Found	Peak Foun	Peak Foun	Peak Foun	Peak Foun	Peak Foun	Peak Foun	1
High	Peak Foun	High	High	High	High	High	Peak Foun	1
Peak Foun	Peak Foun	Peak Foun	Peak Foun	Peak Foun	High	High	High	1
Peak Foun	High	Peak Foun	Peak Foun	High	High	High	Peak Foun	1
High	Peak Foun	High	High	High	High	High	High	1
High	High	Peak Foun	High	High	Peak Foun	Peak Foun	Peak Foun	1
High	Peak Foun	Peak Foun	Peak Foun	High	High	Peak Foun	Peak Foun	1
Peak Foun	Peak Foun	Not Found	High	Peak Foun	Not Found	Not Found	Not Found	1
Peak Foun	Not Found	Peak Foun	Peak Foun	Peak Foun	High	High	Peak Foun	1
Not Found	Peak Foun	High	High	Peak Foun	Peak Foun	High	High	1
Peak Foun	Peak Foun	Peak Foun	High	High	High	High	High	1
Not Found	Peak Foun	Peak Foun	High	Not Found	Not Found	High	Peak Foun	1
Peak Foun	Peak Foun	High	Peak Foun	High	High	High	High	1
Peak Foun	Peak Foun	High	Peak Foun	High	Peak Foun	Peak Foun	Peak Foun	1
High	High	High	Peak Foun	Peak Foun	Peak Foun	Peak Foun	Peak Foun	1
High	High	High	Peak Foun	Peak Foun	Peak Foun	Peak Foun	Peak Foun	1
High	High	High	Peak Foun	High	High	Peak Foun	Peak Foun	1
High	High	High	Peak Foun	High	High	High	Peak Foun	1
Peak Foun	Peak Foun	Peak Foun	Peak Foun	Peak Foun	Peak Foun	High	High	1
High	High	Peak Foun	Peak Foun	Peak Foun	Peak Foun	High	Peak Foun	1
Peak Foun	Not Found	Peak Foun	Peak Foun	High	High	Peak Foun	Peak Foun	1
Peak Foun	Peak Foun	Peak Foun	Peak Foun	Peak Foun	High	High	Peak Foun	1
Peak Foun	High	Peak Foun	Peak Foun	High	High	Peak Foun	Peak Foun	1
Peak Foun	High	High	Peak Foun	Peak Foun	Peak Foun	Peak Foun	Peak Foun	1
High	Peak Foun	Peak Foun	Peak Foun	High	Not Found	Peak Foun	High	1
Not Found	Not Found	Peak Foun	Not Found	Not Found	Not Found	High	Not Found	1
Peak Foun	Peak Foun	Peak Foun	Peak Foun	High	Peak Foun	High	High	1
Not Found	Not Found	Not Found	High	High	High	High	Peak Foun	1
Peak Foun	Peak Foun	Not Found	High	High	High	High	High	1
Not Found	Peak Foun	Peak Foun	Peak Foun	Peak Foun	High	Peak Foun	Peak Foun	1
Peak Foun	Peak Foun	Peak Foun	Peak Foun	High	High	Peak Foun	Not Found	1
High	Peak Foun	Peak Foun	Peak Foun	High	High	High	High	1
High	High	High	High	High	Peak Foun	High	High	1
Not Found	Not Found	Not Found	Not Found	Peak Foun	High	Peak Foun	Peak Foun	1
Peak Foun	High	High	High	High	High	High	High	1
High	Peak Foun	High	Peak Foun	Peak Foun	High	High	Peak Foun	1

Not Found	Peak Foun	Peak Foun	Peak Foun	Peak Foun	Peak Foun	High	Peak Foun	1
Peak Foun	Peak Foun	Peak Foun	High	High	High	High	High	1
Not Found	Peak Foun	High	Not Found	Peak Foun	High	High	Peak Foun	1
High	High	High	High	High	Peak Foun	High	Peak Foun	1
Peak Foun	Peak Foun	Peak Foun	High	High	High	High	High	1
High	High	High	High	High	High	High	High	1
Peak Foun	Peak Foun	High	Peak Foun	Peak Foun	High	Peak Foun	Peak Foun	1
High	High	High	High	High	High	High	High	1
High	Peak Foun	High	High	High	High	High	High	1
Not Found	Peak Foun	Peak Foun	Peak Foun	High	High	High	Peak Foun	1
High	High	High	High	High	High	High	High	1
Peak Foun	Peak Foun	Peak Foun	Peak Foun	Peak Foun	High	Peak Foun	Peak Foun	1
High	High	Peak Foun	Peak Foun	High	Peak Foun	High	Peak Foun	1
Peak Foun	Peak Foun	Peak Foun	Peak Foun	High	High	High	Peak Foun	1
Peak Foun	Peak Foun	Peak Foun	Peak Foun	Peak Foun	High	Peak Foun	Peak Foun	1
Peak Foun	Not Found	Peak Foun	Peak Foun	High	High	Peak Foun	High	1
High	High	High	Peak Foun	High	High	High	High	1
Peak Foun	High	High	Peak Foun	High	High	High	High	1
Peak Foun	Peak Foun	High	Peak Foun	Peak Foun	Peak Foun	High	Peak Foun	1
High	High	Peak Foun	High	High	High	High	High	1
Not Found	Peak Foun	High	High	Peak Foun	High	High	Peak Foun	1
High	Peak Foun	Peak Foun	High	High	High	High	High	1
High	High	Peak Foun	Peak Foun	Peak Foun	High	High	Peak Foun	1
Peak Foun	Peak Foun	High	Peak Foun	Peak Foun	High	High	Peak Foun	1
High	Not Found	Not Found	Not Found	Not Found	Not Found	Not Found	Not Found	1
Peak Foun	Peak Foun	Not Found	High	Not Found	Not Found	Not Found	Not Found	1
Peak Foun	High	High	High	High	High	Peak Foun	High	1
Peak Foun	Peak Foun	Peak Foun	Peak Foun	High	High	High	Peak Foun	1
High	Peak Foun	Peak Foun	Peak Foun	High	Peak Foun	High	Peak Foun	1
Peak Foun	High	Peak Foun	High	High	High	Peak Foun	High	1
High	Peak Foun	High	High	High	High	Peak Foun	High	1
High	Peak Foun	High	High	High	High	High	High	1
Not Found	Not Found	Not Found	Peak Foun	Peak Foun	High	High	Peak Foun	1
Not Found	High	High	Peak Foun	Peak Foun	Peak Foun	Peak Foun	Peak Foun	1
Peak Foun	High	High	Peak Foun	High	High	High	Peak Foun	1
Peak Foun	Peak Foun	Peak Foun	Peak Foun	Peak Foun	High	High	High	1
Peak Foun	Peak Foun	Peak Foun	High	Peak Foun	Peak Foun	High	Peak Foun	1
Peak Foun	Peak Foun	High	Peak Foun	High	High	High	High	1
Peak Foun	Peak Foun	Peak Foun	Peak Foun	High	High	High	High	1
Peak Foun	High	Peak Foun	Peak Foun	High	High	High	High	1
Peak Foun	Not Found	Peak Foun	Peak Foun	High	High	High	Peak Foun	1
Peak Foun	Peak Foun	High	High	High	High	Peak Foun	High	1
Not Found	Not Found	Not Found	Peak Foun	High	High	Peak Foun	Not Found	1
Not Found	Peak Foun	High	Peak Foun	Peak Foun	High	High	Peak Foun	1
High	High	High	Peak Foun	High	High	Peak Foun	Peak Foun	1

High	High	Peak Foun	High	High	Not Found	Peak Foun	High	1
Peak Foun	High	High	High	Peak Foun	High	High	Peak Foun	1
Not Found	Not Found	Not Found	Not Found	Peak Foun	High	High	Not Found	1
Peak Foun	Peak Foun	High	Peak Foun	Peak Foun	High	High	Peak Foun	1
Not Found	Not Found	Not Found	Peak Foun	Not Found	High	Peak Foun	Peak Foun	1
Peak Foun	Peak Foun	Peak Foun	Peak Foun	Peak Foun	Peak Foun	Peak Foun	Peak Foun	1
Peak Foun	High	Peak Foun	High	Peak Foun	Peak Foun	Peak Foun	Peak Foun	1
Peak Foun	Peak Foun	Peak Foun	Peak Foun	High	High	Peak Foun	High	1
Not Found	Not Found	Not Found	Peak Foun	Peak Foun	High	High	Peak Foun	1
Not Found	High	High	Peak Foun	Peak Foun	High	High	High	1
Peak Foun	Peak Foun	High	Peak Foun	Peak Foun	Peak Foun	High	Peak Foun	1
Peak Foun	Peak Foun	Peak Foun	High	High	Peak Foun	Peak Foun	High	1
High	High	High	Peak Foun	High	High	High	Peak Foun	1
Peak Foun	Peak Foun	Peak Foun	Not Found	High	High	Peak Foun	High	1
Peak Foun	High	High	High	High	High	High	Peak Foun	1
Peak Foun	High	High	High	High	High	High	High	1
Peak Foun	Peak Foun	High	Peak Foun	High	High	High	High	1
High	High	High	High	High	High	High	High	1
High	High	High	Peak Foun	High	Peak Foun	High	Peak Foun	1
Peak Foun	High	High	High	High	High	Peak Foun	High	1
Peak Foun	Peak Foun	Peak Foun	Peak Foun	High	High	Peak Foun	Peak Foun	1
Peak Foun	Peak Foun	Peak Foun	Peak Foun	Peak Foun	Peak Foun	High	Peak Foun	1
Not Found	Peak Foun	Not Found	Peak Foun	Not Found	High	Peak Foun	Not Found	1
Peak Foun	Not Found	Peak Foun	High	High	High	High	High	1
Peak Foun	High	High	Peak Foun	High	High	Peak Foun	Peak Foun	1
Peak Foun	High	High	Not Found	High	High	High	Peak Foun	1
Peak Foun	Peak Foun	High	Peak Foun	Peak Foun	Peak Foun	Peak Foun	Not Found	1
Peak Foun	High	High	Peak Foun	Peak Foun	Peak Foun	Not Found	High	1
High	High	High	High	High	Peak Foun	High	High	1
Peak Foun	Peak Foun	Peak Foun	Peak Foun	High	High	High	Peak Foun	1
Peak Foun	High	Peak Foun	Peak Foun	Peak Foun	High	Peak Foun	Peak Foun	1
Not Found	Not Found	Peak Foun	Peak Foun	High	High	High	High	1
Peak Foun	Peak Foun	High	Peak Foun	High	Peak Foun	Peak Foun	Peak Foun	1
Not Found	Peak Foun	Peak Foun	Peak Foun	High	High	Peak Foun	High	1
Not Found	Peak Foun	Peak Foun	Peak Foun	Peak Foun	High	High	Peak Foun	1
Peak Foun	Peak Foun	High	Peak Foun	Peak Foun	Peak Foun	High	Peak Foun	1
Peak Foun	Peak Foun	Peak Foun	High	High	High	High	Peak Foun	1
Peak Foun	High	Peak Foun	Peak Foun	High	High	High	Peak Foun	1
Not Found	Peak Foun	Peak Foun	Not Found	High	High	Peak Foun	Not Found	1
Not Found	High	High	Not Found	High	Not Found	High	Peak Foun	1
Peak Foun	Peak Foun	Peak Foun	Peak Foun	Peak Foun	High	High	Peak Foun	1
High	High	High	Peak Foun	Peak Foun	Peak Foun	Peak Foun	Peak Foun	1
Not Found	Not Found	Peak Foun	Peak Foun	High	High	Peak Foun	Peak Foun	1
Not Found	Not Found	Not Found	Peak Foun	Peak Foun	High	Peak Foun	Peak Foun	1
High	High	High	High	High	High	High	Peak Foun	1

High	High	High	Peak Foun	High	High	High	Peak Foun	1
High	Peak Foun	High	High	High	High	High	High	1
Peak Foun	Peak Foun	Peak Foun	Peak Foun	High	High	High	High	1
Not Found	Peak Foun	Peak Foun	Not Found	High	High	High	Peak Foun	1
Not Found	Peak Foun	Peak Foun	Peak Foun	Peak Foun	High	Peak Foun	Peak Foun	1
Peak Foun	High	Peak Foun	High	High	High	Peak Foun	Peak Foun	1
High	High	High	Peak Foun	High	High	High	Peak Foun	1
Peak Foun	Peak Foun	High	Peak Foun	High	High	High	Peak Foun	1
High	High	High	High	High	High	Peak Foun	High	1
Peak Foun	Peak Foun	Peak Foun	Peak Foun	High	High	High	Peak Foun	1
Not Found	Peak Foun	Not Found	Peak Foun	Not Found	Peak Foun	High	Not Found	1
Peak Foun	Peak Foun	High	Peak Foun	High	High	High	High	1
Not Found	Peak Foun	Peak Foun	Peak Foun	Peak Foun	High	Peak Foun	Peak Foun	1
High	High	High	High	High	High	High	Peak Foun	1
Peak Foun	Peak Foun	High	Peak Foun	Peak Foun	High	High	Peak Foun	1
Peak Foun	High	High	Peak Foun	High	High	High	Peak Foun	1
Not Found	Peak Foun	Peak Foun	Peak Foun	Not Found	Peak Foun	Peak Foun	Not Found	1
Not Found	Not Found	Not Found	Peak Foun	Peak Foun	High	Peak Foun	Not Found	1
High	High	High	High	High	High	High	High	1
Peak Foun	High	Peak Foun	Peak Foun	High	High	High	Peak Foun	1
Peak Foun	Peak Foun	Peak Foun	Peak Foun	Peak Foun	Peak Foun	High	Peak Foun	1
Peak Foun	Peak Foun	Peak Foun	Peak Foun	High	Peak Foun	High	Peak Foun	1
Peak Foun	Peak Foun	Not Found	High	High	High	High	Peak Foun	1
Not Found	Not Found	Not Found	Peak Foun	High	High	High	Peak Foun	1
High	High	High	High	High	High	High	High	1
Peak Foun	Not Found	Not Found	Not Found	High	High	High	Not Found	1
High	High	High	High	Peak Foun	Peak Foun	Peak Foun	Peak Foun	1
Peak Foun	Peak Foun	Peak Foun	Peak Foun	Peak Foun	High	High	Peak Foun	1
Peak Foun	Peak Foun	Peak Foun	High	High	High	High	High	1
Not Found	Not Found	Not Found	Not Found	Peak Foun	High	Peak Foun	Peak Foun	1
Peak Foun	Peak Foun	Peak Foun	High	Peak Foun	High	Peak Foun	Peak Foun	1
Peak Foun	Peak Foun	High	Peak Foun	Peak Foun	High	High	Peak Foun	1
Not Found	Peak Foun	Peak Foun	Not Found	Peak Foun	High	Peak Foun	Peak Foun	1
Peak Foun	High	High	Peak Foun	Peak Foun	Peak Foun	High	Peak Foun	1
Peak Foun	High	Peak Foun	Peak Foun	High	Peak Foun	High	Peak Foun	1
Peak Foun	Not Found	High	High	High	High	High	High	1
Peak Foun	Peak Foun	Peak Foun	High	High	Peak Foun	Peak Foun	Peak Foun	1
Peak Foun	Peak Foun	Peak Foun	Peak Foun	High	High	Peak Foun	Peak Foun	1
Peak Foun	Peak Foun	Peak Foun	Peak Foun	High	High	High	Peak Foun	1
High	High	Peak Foun	Peak Foun	High	High	High	High	1
High	High	Peak Foun	High	High	High	High	High	1
High	High	High	Peak Foun	High	Peak Foun	Peak Foun	Peak Foun	1
High	High	Peak Foun	High	High	High	High	High	1
High	Peak Foun	High	Peak Foun	High	High	High	High	1
Peak Foun	Peak Foun	Peak Foun	Peak Foun	Peak Foun	High	Peak Foun	Peak Foun	1

Peak Foun	Peak Foun	Peak Foun	Peak Foun	Peak Foun	High	High	Peak Foun	1
Not Found	Peak Foun	Peak Foun	Peak Foun	Peak Foun	Peak Foun	Not Found	Not Found	1
Peak Foun	Peak Foun	Peak Foun	Peak Foun	High	Peak Foun	Peak Foun	Not Found	1
Peak Foun	High	High	Peak Foun	Peak Foun	Peak Foun	Peak Foun	Peak Foun	1
Peak Foun	Peak Foun	High	Peak Foun	High	High	High	High	1
Peak Foun	Peak Foun	Peak Foun	Peak Foun	Peak Foun	High	Peak Foun	Peak Foun	1
Peak Foun	Peak Foun	High	Peak Foun	Peak Foun	High	High	High	1
Peak Foun	Peak Foun	Peak Foun	Peak Foun	High	High	Peak Foun	Peak Foun	1
Peak Foun	Not Found	Peak Foun	Peak Foun	High	High	High	Peak Foun	1
High	Not Found	Peak Foun	Not Found	High	High	High	Peak Foun	1
Peak Foun	Not Found	Not Found	Not Found	High	High	Not Found	Peak Foun	1
Peak Foun	Peak Foun	High	Peak Foun	High	High	High	Peak Foun	1
High	High	High	High	Peak Foun	Peak Foun	Not Found	High	1
High	High	High	High	High	High	High	Peak Foun	1
Peak Foun	Peak Foun	Peak Foun	Peak Foun	High	High	High	Peak Foun	1
Not Found	Not Found	Peak Foun	Not Found	Peak Foun	Peak Foun	High	Peak Foun	1
Not Found	Peak Foun	Peak Foun	Peak Foun	Peak Foun	Peak Foun	Not Found	Peak Foun	1
High	High	High	High	High	High	High	High	1
Not Found	Peak Foun	Peak Foun	Peak Foun	High	Peak Foun	High	Peak Foun	1
High	High	High	Peak Foun	Peak Foun	High	High	Peak Foun	1
Peak Foun	Peak Foun	Peak Foun	Peak Foun	Peak Foun	High	Peak Foun	Peak Foun	1
High	High	Not Found	Peak Foun	Not Found	Not Found	Not Found	Not Found	1
High	Peak Foun	High	High	High	High	High	High	1
High	High	High	Peak Foun	High	High	High	High	1
Not Found	Not Found	Not Found	Peak Foun	Peak Foun	High	Not Found	High	1
High	High	High	High	High	High	High	Peak Foun	1
Peak Foun	Peak Foun	Peak Foun	Peak Foun	Peak Foun	Not Found	Peak Foun	Peak Foun	1
High	High	High	High	High	High	High	Peak Foun	1
High	Peak Foun	High	Not Found	High	High	High	Peak Foun	1
Peak Foun	High	High	Peak Foun	Peak Foun	High	High	Peak Foun	1
High	Peak Foun	High	Peak Foun	High	Peak Foun	High	High	1
Peak Foun	High	Peak Foun	High	High	High	High	High	1
Peak Foun	High	High	High	High	High	High	High	1
High	Peak Foun	Peak Foun	High	High	High	High	High	1
High	High	High	High	Peak Foun	High	High	High	1
High	Peak Foun	High	Peak Foun	Peak Foun	High	High	Peak Foun	1
Not Found	High	Peak Foun	Peak Foun	High	High	Peak Foun	Peak Foun	1
Peak Foun	Not Found	Peak Foun	Peak Foun	Peak Foun	High	High	Not Found	1
High	High	High	High	Peak Foun	Peak Foun	Peak Foun	Peak Foun	1
Peak Foun	High	High	High	High	High	High	Peak Foun	1
Not Found	Not Found	Not Found	Peak Foun	Not Found	Not Found	Not Found	Not Found	1
Peak Foun	Peak Foun	Peak Foun	High	High	High	High	High	1
High	High	High	High	High	High	High	Peak Foun	1
High	High	High	Peak Foun	High	High	High	High	1
High	High	Not Found	Peak Foun	High	High	Peak Foun	High	1

High	Peak Foun	High	Peak Foun	High	High	High	Peak Foun	1
Peak Foun	Peak Foun	Peak Foun	Peak Foun	Peak Foun	High	High	Peak Foun	1
Not Found	Not Found	Not Found	Not Found	High	High	Not Found	Not Found	1
Not Found	Not Found	Not Found	Peak Foun	Peak Foun	High	High	Peak Foun	1
High	High	High	Peak Foun	Peak Foun	High	High	High	1
High	High	Peak Foun	High	Peak Foun	High	Peak Foun	High	1
Peak Foun	High	Peak Foun	Peak Foun	High	High	High	Peak Foun	1
Peak Foun	Peak Foun	High	Peak Foun	Peak Foun	High	High	Peak Foun	1
Peak Foun	High	High	Peak Foun	High	Peak Foun	High	Peak Foun	1
Peak Foun	Peak Foun	Peak Foun	Peak Foun	Peak Foun	High	High	Peak Foun	1
Peak Foun	Peak Foun	Peak Foun	Peak Foun	High	High	Peak Foun	Peak Foun	1
Not Found	Peak Foun	High	High	Peak Foun	Peak Foun	High	High	1
Peak Foun	Peak Foun	Peak Foun	Peak Foun	Peak Foun	High	High	Peak Foun	1
Not Found	Not Found	Not Found	Not Found	Peak Foun	High	Peak Foun	Peak Foun	1
High	Peak Foun	Peak Foun	Peak Foun	Peak Foun	Peak Foun	Peak Foun	Peak Foun	1
Peak Foun	High	High	Peak Foun	High	Peak Foun	High	Peak Foun	1
Peak Foun	Peak Foun	Not Found	Not Found	Not Found	Peak Foun	High	Not Found	1
Peak Foun	High	Peak Foun	Peak Foun	High	Peak Foun	Peak Foun	Not Found	1
Peak Foun	Peak Foun	Peak Foun	Peak Foun	High	High	High	Peak Foun	1
High	High	High	High	High	High	High	High	1
Peak Foun	Peak Foun	Peak Foun	Peak Foun	High	High	Peak Foun	High	1
Peak Foun	Peak Foun	Peak Foun	Not Found	High	High	High	Peak Foun	1
High	Peak Foun	Peak Foun	Peak Foun	Peak Foun	High	High	High	1
High	High	Peak Foun	Not Found	High	High	Not Found	Peak Foun	1
Peak Foun	Peak Foun	High	Peak Foun	High	Peak Foun	High	Peak Foun	1
Peak Foun	Peak Foun	Peak Foun	Peak Foun	High	High	High	High	1
Peak Foun	High	High	High	Peak Foun	High	High	High	1
High	High	High	High	High	High	Peak Foun	High	1
Peak Foun	Peak Foun	High	High	High	Peak Foun	High	High	1
Peak Foun	Peak Foun	Peak Foun	High	High	Peak Foun	Peak Foun	Peak Foun	1
Not Found	Not Found	Not Found	Peak Foun	Peak Foun	Peak Foun	Peak Foun	Peak Foun	1
Peak Foun	Peak Foun	Peak Foun	High	High	High	Peak Foun	High	1
Peak Foun	High	High	High	High	High	High	High	1
Peak Foun	High	High	High	High	High	High	High	1
High	High	High	Peak Foun	High	High	High	High	1
Peak Foun	Peak Foun	High	Peak Foun	Peak Foun	High	High	Peak Foun	1
Peak Foun	Not Found	Not Found	High	Peak Foun	High	High	High	1
High	High	High	High	High	High	High	High	1
Not Found	Peak Foun	Peak Foun	Peak Foun	Not Found	Peak Foun	Peak Foun	Peak Foun	1
Peak Foun	Peak Foun	Peak Foun	High	Peak Foun	High	Peak Foun	Peak Foun	1
Peak Foun	Peak Foun	High	Peak Foun	Peak Foun	High	Peak Foun	Peak Foun	1
High	High	High	High	High	High	High	High	1
High	Peak Foun	High	High	High	High	High	High	1
Peak Foun	Peak Foun	High	Peak Foun	Peak Foun	Peak Foun	High	Peak Foun	1
Peak Foun	High	High	High	High	High	High	High	1

High	High	High	Peak Foun	Peak Foun	Peak Foun	Peak Foun	Peak Foun	1
High	Peak Foun	Peak Foun	Peak Foun	High	High	Peak Foun	High	1
Peak Foun	Peak Foun	Peak Foun	Peak Foun	Peak Foun	Peak Foun	High	Peak Foun	1
High	High	High	High	High	High	High	High	1
Peak Foun	High	High	High	High	Peak Foun	Peak Foun	Peak Foun	1
Not Found	Peak Foun	Peak Foun	Peak Foun	Peak Foun	Peak Foun	Peak Foun	Peak Foun	1
High	High	High	High	High	High	High	High	1
Peak Foun	High	High	Peak Foun	High	High	High	Peak Foun	1
Peak Foun	Peak Foun	Peak Foun	Peak Foun	Peak Foun	High	High	Peak Foun	1
Peak Foun	Not Found	Peak Foun	Peak Foun	Peak Foun	High	Peak Foun	High	1
Peak Foun	Not Found	Peak Foun	High	High	High	High	High	1
Peak Foun	High	High	Peak Foun	High	High	High	Peak Foun	1
Peak Foun	Not Found	Peak Foun	Peak Foun	Peak Foun	High	Peak Foun	Peak Foun	1
Peak Foun	Peak Foun	High	Peak Foun	High	High	High	High	1
Peak Foun	Peak Foun	Not Found	Peak Foun	High	Peak Foun	High	Not Found	1
High	Peak Foun	Peak Foun	High	High	High	High	Peak Foun	1
Peak Foun	Peak Foun	Peak Foun	Peak Foun	High	High	High	Peak Foun	1
Peak Foun	High	High	Peak Foun	Peak Foun	High	Peak Foun	Peak Foun	1
Not Found	Peak Foun	High	Peak Foun	High	High	High	Not Found	1
Peak Foun	High	High	Peak Foun	High	High	Peak Foun	High	1
Peak Foun	Not Found	Peak Foun	Peak Foun	Peak Foun	Peak Foun	Peak Foun	Peak Foun	1
Peak Foun	Peak Foun	Peak Foun	Peak Foun	Peak Foun	High	Peak Foun	Peak Foun	1
Peak Foun	High	Peak Foun	High	High	Peak Foun	High	High	1
Peak Foun	High	High	High	High	High	High	High	1
Not Found	Not Found	Peak Foun	Peak Foun	High	High	High	Not Found	1
High	High	High	High	High	High	High	High	1
Peak Foun	Peak Foun	High	Peak Foun	Peak Foun	High	High	Peak Foun	1
Not Found	Not Found	Not Found	Not Found	Peak Foun	High	Peak Foun	Not Found	1
Peak Foun	Peak Foun	Peak Foun	Peak Foun	High	High	High	Peak Foun	1
High	High	High	High	High	High	High	Peak Foun	1
Peak Foun	Peak Foun	Peak Foun	Peak Foun	High	High	High	Peak Foun	1
Peak Foun	High	Peak Foun	Peak Foun	High	High	High	Peak Foun	1
Peak Foun	Peak Foun	High	Peak Foun	High	High	Peak Foun	Peak Foun	1
High	Peak Foun	Peak Foun	Peak Foun	High	Peak Foun	High	Peak Foun	1
Peak Foun	High	High	Peak Foun	Peak Foun	Peak Foun	Peak Foun	Not Found	1
Peak Foun	Peak Foun	Peak Foun	High	High	High	High	Peak Foun	1
Peak Foun	High	Peak Foun	Not Found	Peak Foun	High	Peak Foun	Peak Foun	1
High	High	High	Peak Foun	High	High	High	Peak Foun	1
High	High	High	High	High	High	High	High	1
Not Found	Not Found	Peak Foun	Peak Foun	Peak Foun	High	High	Peak Foun	1
High	Peak Foun	High	Peak Foun	Peak Foun	High	High	Peak Foun	1
Peak Foun	Peak Foun	Peak Foun	Peak Foun	Peak Foun	Peak Foun	Peak Foun	Peak Foun	1
Peak Foun	Peak Foun	High	Peak Foun	Peak Foun	High	High	Peak Foun	1
Peak Foun	Peak Foun	Peak Foun	High	Peak Foun	Peak Foun	Peak Foun	Peak Foun	1
High	High	High	High	High	High	High	High	1

Peak Foun	High	High	Peak Foun	Peak Foun	High	High	Peak Foun	1
Peak Foun	Peak Foun	Peak Foun	Peak Foun	Peak Foun	Peak Foun	Peak Foun	Peak Foun	1
Not Found	Peak Foun	Peak Foun	Peak Foun	Peak Foun	High	Peak Foun	Peak Foun	1
Not Found	Peak Foun	Not Found	Peak Foun	Peak Foun	High	Peak Foun	Peak Foun	1
High	Peak Foun	High	High	High	High	Peak Foun	High	1
Not Found	Peak Foun	High	Peak Foun	Peak Foun	Peak Foun	High	Peak Foun	1
Peak Foun	Not Found	Not Found	Not Found	Peak Foun	High	Peak Foun	Not Found	1
High	Peak Foun	Peak Foun	Peak Foun	High	High	Peak Foun	High	1
Not Found	High	High	High	Peak Foun	High	High	High	1
High	High	High	Peak Foun	High	High	Peak Foun	Peak Foun	1
Peak Foun	Peak Foun	Peak Foun	Peak Foun	Peak Foun	Peak Foun	Peak Foun	Peak Foun	1
Peak Foun	Not Found	Peak Foun	High	Peak Foun	Not Found	Peak Foun	Peak Foun	1
Peak Foun	High	Peak Foun	High	High	High	High	High	1
Peak Foun	Peak Foun	Peak Foun	Peak Foun	Peak Foun	High	Peak Foun	Peak Foun	1
Peak Foun	High	Peak Foun	High	High	High	High	Peak Foun	1
Peak Foun	High	High	Peak Foun	Peak Foun	Peak Foun	Peak Foun	Peak Foun	1
Peak Foun	High	Peak Foun	Peak Foun	Peak Foun	High	High	Peak Foun	1
Peak Foun	High	Peak Foun	Peak Foun	High	High	High	Peak Foun	1
Peak Foun	Peak Foun	High	Peak Foun	Peak Foun	High	High	Peak Foun	1
High	High	High	High	Not Found	High	Peak Foun	Peak Foun	1
Peak Foun	Peak Foun	Not Found	Peak Foun	Peak Foun	High	Peak Foun	Peak Foun	1
Not Found	Not Found	Not Found	Peak Foun	Peak Foun	High	High	Peak Foun	1
High	High	High	High	High	High	Peak Foun	Peak Foun	1
Peak Foun	Peak Foun	Peak Foun	Peak Foun	High	High	High	High	1
Peak Foun	Peak Foun	Peak Foun	Not Found	Peak Foun	High	Peak Foun	Peak Foun	1
Peak Foun	Peak Foun	High	Peak Foun	High	High	Peak Foun	Peak Foun	1
Peak Foun	Peak Foun	Peak Foun	Peak Foun	High	Peak Foun	Peak Foun	Peak Foun	1
Not Found	Peak Foun	Peak Foun	Peak Foun	Not Found	Peak Foun	Peak Foun	Not Found	1
Not Found	Peak Foun	Peak Foun	Not Found	Peak Foun	High	High	High	1
Peak Foun	Peak Foun	Peak Foun	Peak Foun	High	High	High	High	1
High	Peak Foun	Peak Foun	High	Peak Foun	Peak Foun	High	High	1
Not Found	Peak Foun	High	Not Found	Peak Foun	High	High	Peak Foun	1
Peak Foun	High	Peak Foun	Peak Foun	Peak Foun	High	Peak Foun	Peak Foun	1
High	High	High	High	High	High	High	High	1
Peak Foun	Peak Foun	High	Peak Foun	High	High	High	High	1
High	High	High	High	High	High	High	High	1
High	High	High	High	Peak Foun	High	High	Not Found	1
Peak Foun	Peak Foun	High	Peak Foun	High	High	Peak Foun	Not Found	1
Not Found	Peak Foun	Not Found	Peak Foun	Not Found	High	Not Found	Not Found	1
Peak Foun	Not Found	Not Found	Peak Foun	Peak Foun	High	Peak Foun	Peak Foun	1
Peak Foun	Peak Foun	High	Peak Foun	Peak Foun	Peak Foun	High	Peak Foun	1
Peak Foun	Not Found	Not Found	Peak Foun	Peak Foun	Peak Foun	High	Peak Foun	1
Peak Foun	High	High	High	High	High	Peak Foun	High	1
Not Found	Peak Foun	Peak Foun	High	High	High	High	High	1
Peak Foun	Peak Foun	High	Peak Foun	High	High	High	Peak Foun	1

High	High	High	High	High	High	High	High	1
Not Found	Not Found	Not Found	Not Found	Peak Foun	High	Peak Foun	Not Found	1
Not Found	Not Found	Not Found	Peak Foun	Peak Foun	Peak Foun	High	Peak Foun	1
Peak Foun	Peak Foun	High	Not Found	Not Found	Peak Foun	Peak Foun	Not Found	1
Peak Foun	Not Found	High	Peak Foun	Peak Foun	Peak Foun	High	Peak Foun	1
High	Not Found	Peak Foun	High	Peak Foun	High	High	Peak Foun	1
Not Found	Not Found	Not Found	Peak Foun	Peak Foun	High	Peak Foun	Peak Foun	1
Peak Foun	High	Peak Foun	High	Peak Foun	High	Peak Foun	Peak Foun	1
Peak Foun	Peak Foun	Peak Foun	Peak Foun	Peak Foun	High	Peak Foun	Peak Foun	1
High	Peak Foun	High	Peak Foun	High	High	High	High	1
High	Peak Foun	High	High	High	Peak Foun	Peak Foun	High	1
Peak Foun	Peak Foun	Peak Foun	High	High	High	Peak Foun	Peak Foun	1
Not Found	Not Found	Not Found	Not Found	Not Found	High	Peak Foun	Not Found	1
Peak Foun	High	Peak Foun	Peak Foun	Peak Foun	High	Peak Foun	Peak Foun	1
Peak Foun	Peak Foun	High	Peak Foun	Peak Foun	High	High	High	1
Not Found	Not Found	High	Peak Foun	Peak Foun	High	High	High	1
High	Peak Foun	High	High	High	High	High	High	1
Peak Foun	Peak Foun	Peak Foun	Peak Foun	Peak Foun	High	High	Peak Foun	1
Peak Foun	High	High	Peak Foun	High	High	High	High	1
Peak Foun	High	High	Peak Foun	High	High	Peak Foun	High	1
Peak Foun	Peak Foun	Peak Foun	Peak Foun	Peak Foun	High	Peak Foun	Peak Foun	1
High	High	High	High	High	High	High	High	1
Peak Foun	Peak Foun	High	Peak Foun	High	High	High	Peak Foun	1
Not Found	Not Found	Not Found	Peak Foun	High	High	Peak Foun	Peak Foun	1
Peak Foun	Peak Foun	Peak Foun	High	Peak Foun	High	High	Peak Foun	1
High	Peak Foun	High	High	High	High	High	Peak Foun	1
Peak Foun	Not Found	High	Peak Foun	High	High	Peak Foun	Peak Foun	1
High	Peak Foun	High	Peak Foun	High	High	High	Peak Foun	1
Not Found	Peak Foun	High	Peak Foun	Peak Foun	Peak Foun	Peak Foun	Peak Foun	1
Peak Foun	Peak Foun	Peak Foun	Peak Foun	High	High	High	Peak Foun	1
High	Peak Foun	High	Peak Foun	Peak Foun	High	Peak Foun	Peak Foun	1
High	High	High	Peak Foun	High	Peak Foun	High	Peak Foun	1
High	Peak Foun	Peak Foun	Peak Foun	Peak Foun	Peak Foun	Peak Foun	Peak Foun	1
Peak Foun	High	Peak Foun	High	Peak Foun	High	High	Peak Foun	1
Not Found	Not Found	Not Found	Not Found	Peak Foun	High	High	Not Found	1
Not Found	Peak Foun	High	High	High	High	Not Found	Not Found	1
Peak Foun	Peak Foun	Peak Foun	Peak Foun	High	High	High	Peak Foun	1
Not Found	High	High	High	Peak Foun	High	High	Peak Foun	1
Peak Foun	Peak Foun	Peak Foun	Peak Foun	High	High	Peak Foun	Peak Foun	1
Peak Foun	High	Peak Foun	Peak Foun	High	Peak Foun	High	Peak Foun	1
High	High	High	High	High	High	High	High	1
Not Found	Not Found	Peak Foun	Peak Foun	Peak Foun	High	High	Peak Foun	1
Peak Foun	Peak Foun	High	High	High	High	Peak Foun	High	1
Peak Foun	Peak Foun	Peak Foun	Peak Foun	High	High	Peak Foun	Peak Foun	1
Not Found	Not Found	High	Peak Foun	Not Found	High	High	Not Found	1

High	Peak Foun	High	Peak Foun	Peak Foun	High	High	Peak Foun	1
Peak Foun	Peak Foun	High	Peak Foun	Peak Foun	Peak Foun	Peak Foun	Peak Foun	1
Peak Foun	Peak Foun	Peak Foun	Not Found	High	High	High	Peak Foun	1
High	High	High	High	High	High	High	High	1
Peak Foun	Peak Foun	High	Peak Foun	Peak Foun	High	Peak Foun	Peak Foun	1
Peak Foun	Peak Foun	High	Peak Foun	High	High	High	Peak Foun	1
High	Peak Foun	High	Peak Foun	Peak Foun	High	High	High	1
Not Found	Peak Foun	Not Found	Peak Foun	Peak Foun	Peak Foun	High	Peak Foun	1
Peak Foun	High	Peak Foun	Peak Foun	Peak Foun	High	High	High	1
Peak Foun	Peak Foun	High	High	Peak Foun	High	High	Peak Foun	1
Peak Foun	Not Found	Peak Foun	Peak Foun	Peak Foun	High	Peak Foun	Peak Foun	1
Peak Foun	High	Peak Foun	Not Found	Peak Foun	Peak Foun	Peak Foun	Peak Foun	1
Peak Foun	Peak Foun	Peak Foun	Peak Foun	High	High	Peak Foun	Peak Foun	1
High	High	High	High	High	High	High	Peak Foun	1
Not Found	High	High	Peak Foun	Peak Foun	Peak Foun	High	Peak Foun	1
Not Found	High	High	High	High	High	High	High	1
Peak Foun	Peak Foun	Peak Foun	Peak Foun	Peak Foun	High	High	High	1
Not Found	Peak Foun	Not Found	Not Found	Not Found	High	High	Not Found	1
Peak Foun	High	High	Peak Foun	Peak Foun	Peak Foun	Peak Foun	Peak Foun	1
Peak Foun	Peak Foun	High	Peak Foun	High	High	High	Peak Foun	1
High	Peak Foun	High	Peak Foun	Peak Foun	High	Peak Foun	Peak Foun	1
Not Found	Not Found	Not Found	High	High	High	High	High	1
Peak Foun	Peak Foun	Peak Foun	Peak Foun	High	High	Peak Foun	Peak Foun	1
Peak Foun	Not Found	Peak Foun	High	Peak Foun	High	Peak Foun	Peak Foun	1
Peak Foun	Not Found	Peak Foun	Peak Foun	Peak Foun	High	Peak Foun	Peak Foun	1
Peak Foun	Peak Foun	Not Found	Peak Foun	High	High	Peak Foun	Peak Foun	1
Peak Foun	High	High	Peak Foun	Peak Foun	Peak Foun	High	Peak Foun	1
High	Peak Foun	Peak Foun	High	High	High	High	High	1
Peak Foun	Peak Foun	Peak Foun	Peak Foun	High	High	High	High	1
High	High	High	Peak Foun	Peak Foun	Peak Foun	High	Peak Foun	1
Not Found	Peak Foun	High	Peak Foun	High	High	High	Peak Foun	1
High	High	High	Peak Foun	High	High	High	Peak Foun	1
Peak Foun	Peak Foun	Not Found	High	High	High	High	Peak Foun	1
Peak Foun	Peak Foun	Peak Foun	High	Peak Foun	High	High	Peak Foun	1
High	High	Peak Foun	Peak Foun	High	High	High	Peak Foun	1
Not Found	Peak Foun	Not Found	Not Found	Peak Foun	High	Peak Foun	Peak Foun	1
Peak Foun	Peak Foun	High	Peak Foun	Peak Foun	Peak Foun	Peak Foun	Peak Foun	1
Not Found	Not Found	Not Found	Peak Foun	Peak Foun	High	Peak Foun	Peak Foun	1
Peak Foun	High	High	Peak Foun	Peak Foun	Peak Foun	Peak Foun	Peak Foun	1
High	Peak Foun	Peak Foun	Peak Foun	High	High	Peak Foun	High	1
Not Found	Not Found	Not Found	Peak Foun	Peak Foun	High	High	Peak Foun	1
Peak Foun	High	Peak Foun	Peak Foun	High	High	High	Peak Foun	1
Not Found	Not Found	High	High	Peak Foun	High	High	Peak Foun	1
Peak Foun	High	Peak Foun	High	High	High	High	High	1
High	Peak Foun	High	High	High	High	High	High	1

High	Peak Foun	High	High	High	High	High	High	1
High	Peak Foun	Peak Foun	Peak Foun	High	High	High	Peak Foun	1
Not Found	Peak Foun	Peak Foun	Peak Foun	Peak Foun	High	High	High	1
Not Found	Peak Foun	Peak Foun	Peak Foun	High	High	High	Peak Foun	1
Peak Foun	Peak Foun	High	Peak Foun	Peak Foun	Peak Foun	Peak Foun	Peak Foun	1
High	High	High	High	High	High	High	High	1
High	Peak Foun	High	Peak Foun	High	High	High	High	1
Not Found	Not Found	Not Found	High	High	High	Not Found	High	1
High	High	High	Peak Foun	Peak Foun	High	Peak Foun	Peak Foun	1
Peak Foun	High	High	Peak Foun	High	High	High	Peak Foun	1
Peak Foun	Peak Foun	Peak Foun	Peak Foun	High	High	High	Peak Foun	1
Peak Foun	Peak Foun	Peak Foun	Peak Foun	High	High	High	Peak Foun	1
Not Found	Not Found	Not Found	Not Found	Not Found	Not Found	High	Not Found	1
High	High	High	Peak Foun	Peak Foun	High	High	Peak Foun	1
Not Found	Peak Foun	Peak Foun	Peak Foun	Peak Foun	High	Peak Foun	Not Found	1
Peak Foun	High	High	Peak Foun	High	High	High	High	1
Peak Foun	Peak Foun	Not Found	Peak Foun	Not Found	Not Found	Not Found	Not Found	1
Peak Foun	High	High	High	High	High	High	Peak Foun	1
Peak Foun	High	High	Peak Foun	Peak Foun	Peak Foun	Peak Foun	Peak Foun	1
Peak Foun	Peak Foun	High	Peak Foun	Peak Foun	High	High	Peak Foun	1
Peak Foun	Not Found	Not Found	Peak Foun	Peak Foun	High	Peak Foun	Peak Foun	1
High	Peak Foun	Peak Foun	High	Peak Foun	High	High	Peak Foun	1
High	Peak Foun	High	High	Peak Foun	Peak Foun	High	High	1
Peak Foun	High	High	High	Peak Foun	High	High	High	1
Peak Foun	Peak Foun	Peak Foun	Peak Foun	Peak Foun	Peak Foun	High	Peak Foun	1
Not Found	Not Found	Not Found	Not Found	Peak Foun	Not Found	Not Found	Not Found	1
High	High	High	Peak Foun	High	High	High	High	1
Peak Foun	Peak Foun	High	Not Found	Peak Foun	High	Not Found	Peak Foun	1
High	High	Peak Foun	High	High	High	High	High	1
High	High	Peak Foun	High	High	High	High	High	1
Peak Foun	Peak Foun	Peak Foun	Peak Foun	High	Peak Foun	Not Found	Peak Foun	1
Peak Foun	Peak Foun	High	Peak Foun	Peak Foun	Peak Foun	Peak Foun	Peak Foun	1
Peak Foun	Peak Foun	Peak Foun	Peak Foun	Peak Foun	High	Peak Foun	Peak Foun	1
High	High	High	High	High	High	Peak Foun	Peak Foun	1
Peak Foun	Peak Foun	Not Found	Not Found	High	High	High	Not Found	1
Not Found	High	High	Peak Foun	High	High	High	Peak Foun	1
Peak Foun	High	Peak Foun	Peak Foun	High	High	High	High	1
Peak Foun	High	Peak Foun	Peak Foun	Peak Foun	High	High	High	1
Peak Foun	Peak Foun	Peak Foun	High	High	High	High	High	1
Peak Foun	Peak Foun	Peak Foun	Peak Foun	Peak Foun	High	Peak Foun	Peak Foun	1
Peak Foun	Peak Foun	Peak Foun	Peak Foun	Peak Foun	High	Peak Foun	Peak Foun	1
Peak Foun	Peak Foun	Peak Foun	Peak Foun	Peak Foun	Peak Foun	High	Peak Foun	1
Not Found	Not Found	Not Found	Not Found	Not Found	High	Not Found	Not Found	1
High	High	Peak Foun	High	Peak Foun	High	Peak Foun	Peak Foun	1
Peak Foun	Peak Foun	Peak Foun	Peak Foun	Peak Foun	High	Peak Foun	Peak Foun	1

Not Found	Peak Foun	Peak Foun	Peak Foun	Peak Foun	High	High	Peak Foun	1
Not Found	Not Found	Not Found	Not Found	Not Found	High	Not Found	Not Found	1
Peak Foun	Peak Foun	Peak Foun	Peak Foun	Peak Foun	High	Peak Foun	Peak Foun	1
Peak Foun	Peak Foun	High	High	Peak Foun	High	High	High	1
Peak Foun	High	Peak Foun	Not Found	Peak Foun	Peak Foun	Peak Foun	Peak Foun	1
Not Found	Peak Foun	High	Not Found	Peak Foun	Peak Foun	High	Not Found	1
Peak Foun	High	High	High	High	High	High	Peak Foun	1
High	High	High	Peak Foun	Peak Foun	High	High	Peak Foun	1
Peak Foun	Peak Foun	High	Peak Foun	High	High	High	Peak Foun	1
Peak Foun	Peak Foun	Peak Foun	Peak Foun	High	High	High	Peak Foun	1
Peak Foun	Peak Foun	Peak Foun	Peak Foun	High	High	High	High	1
High	High	Peak Foun	High	High	High	Peak Foun	Peak Foun	1
High	High	High	Peak Foun	High	High	Peak Foun	Not Found	1
Peak Foun	Peak Foun	Not Found	Not Found	Not Found	High	High	Not Found	1
Peak Foun	Peak Foun	Peak Foun	Peak Foun	High	High	High	Peak Foun	1
Peak Foun	Peak Foun	High	Not Found	High	Peak Foun	High	Peak Foun	1
Peak Foun	Peak Foun	Peak Foun	Peak Foun	High	High	Peak Foun	Peak Foun	1
Peak Foun	High	High	Peak Foun	High	High	High	Peak Foun	1
High	High	High	Peak Foun	High	High	High	Peak Foun	1
Peak Foun	Peak Foun	Peak Foun	Peak Foun	High	Peak Foun	Peak Foun	Peak Foun	1
Peak Foun	High	Peak Foun	High	High	High	High	High	1
High	Peak Foun	Not Found	Not Found	Not Found	Not Found	Not Found	Not Found	1
Peak Foun	Not Found	Peak Foun	Peak Foun	High	Peak Foun	Peak Foun	Peak Foun	1
Peak Foun	Not Found	Peak Foun	Peak Foun	Not Found	High	Peak Foun	Not Found	1
Peak Foun	Peak Foun	Peak Foun	Peak Foun	Peak Foun	Peak Foun	High	Peak Foun	1
High	High	High	High	Peak Foun	Not Found	Not Found	Not Found	1
Not Found	Not Found	High	Peak Foun	High	High	High	High	1
Peak Foun	Peak Foun	Peak Foun	Peak Foun	Peak Foun	Peak Foun	Peak Foun	Peak Foun	1
Not Found	Not Found	Peak Foun	Not Found	Not Found	High	High	Not Found	1
Peak Foun	High	Peak Foun	Peak Foun	High	High	High	High	1
Peak Foun	Not Found	Not Found	Not Found	High	High	Not Found	Not Found	1
High	High	Peak Foun	Not Found	Peak Foun	Not Found	Not Found	Not Found	1
Not Found	Peak Foun	Not Found	Peak Foun	Peak Foun	High	High	Not Found	1
Peak Foun	Peak Foun	Peak Foun	Peak Foun	Peak Foun	Peak Foun	Peak Foun	Peak Foun	1
High	High	Peak Foun	Peak Foun	High	High	High	Not Found	1
Peak Foun	Peak Foun	High	Peak Foun	High	Peak Foun	Peak Foun	High	1
High	High	High	Peak Foun	Peak Foun	High	Peak Foun	Peak Foun	1
Peak Foun	Peak Foun	Peak Foun	Peak Foun	Peak Foun	Not Found	High	Peak Foun	1
Not Found	Not Found	Not Found	Peak Foun	Peak Foun	High	High	Peak Foun	1
Peak Foun	Peak Foun	Peak Foun	Peak Foun	Peak Foun	Peak Foun	High	Not Found	1
Not Found	Not Found	High	Not Found	Peak Foun	High	High	High	1
High	Not Found	Peak Foun	High	High	High	High	Peak Foun	1
High	High	Peak Foun	Peak Foun	High	Peak Foun	High	High	1
Peak Foun	High	Peak Foun	Peak Foun	High	High	High	Peak Foun	1
Peak Foun	High	High	Peak Foun	High	High	High	Peak Foun	1

Peak Foun	High	Peak Foun	Peak Foun	High	High	High	Peak Foun	1
Peak Foun	Peak Foun	Peak Foun	Peak Foun	Peak Foun	High	High	Peak Foun	1
High	High	High	Peak Foun	Peak Foun	Peak Foun	Peak Foun	Peak Foun	1
Not Found	Not Found	Not Found	Not Found	High	Not Found	Not Found	Not Found	1
Peak Foun	Peak Foun	Peak Foun	Peak Foun	Peak Foun	High	Peak Foun	Peak Foun	1
High	Peak Foun	High	Peak Foun	High	High	High	Peak Foun	1
Peak Foun	Peak Foun	Peak Foun	High	Peak Foun	High	High	Peak Foun	1
Peak Foun	Peak Foun	High	High	High	High	High	High	1
High	Peak Foun	Peak Foun	Peak Foun	High	Peak Foun	Peak Foun	Peak Foun	1
Not Found	Not Found	Not Found	Not Found	High	Peak Foun	High	Not Found	1
Not Found	Peak Foun	High	High	Peak Foun	High	High	Not Found	1
High	High	High	Peak Foun	High	High	High	Peak Foun	1
Peak Foun	Peak Foun	Peak Foun	Peak Foun	Peak Foun	Peak Foun	High	Peak Foun	1
Not Found	Peak Foun	Peak Foun	Peak Foun	Peak Foun	Peak Foun	Peak Foun	High	1
Peak Foun	Peak Foun	Peak Foun	Peak Foun	Peak Foun	High	Peak Foun	Peak Foun	1
High	High	High	Peak Foun	High	High	High	High	1
Peak Foun	Peak Foun	Peak Foun	Peak Foun	High	High	High	High	1
Peak Foun	High	High	Peak Foun	Peak Foun	High	High	Peak Foun	1
Not Found	Not Found	Peak Foun	Not Found	Peak Foun	High	Peak Foun	Peak Foun	1
Not Found	Peak Foun	Peak Foun	Peak Foun	Peak Foun	High	High	Peak Foun	1
High	High	Peak Foun	Not Found	Peak Foun	Peak Foun	Peak Foun	Peak Foun	1
Peak Foun	Peak Foun	High	High	High	High	Peak Foun	Peak Foun	1
High	Peak Foun	Peak Foun	Peak Foun	High	High	Peak Foun	Peak Foun	1
Peak Foun	High	High	High	High	High	High	High	1
Peak Foun	Peak Foun	High	High	Peak Foun	High	High	High	1
High	High	High	Peak Foun	Peak Foun	Peak Foun	Peak Foun	Peak Foun	1
Peak Foun	Peak Foun	Peak Foun	Peak Foun	Peak Foun	High	Not Found	Not Found	1
Not Found	Peak Foun	Not Found	Peak Foun	High	High	High	Peak Foun	1
High	High	Peak Foun	High	High	Peak Foun	High	Peak Foun	1
High	High	Peak Foun	Peak Foun	Peak Foun	Peak Foun	High	Peak Foun	1
High	High	High	Peak Foun	High	High	High	Peak Foun	1
Not Found	Not Found	Not Found	Not Found	Not Found	High	Peak Foun	Not Found	1
High	Peak Foun	High	High	Peak Foun	Peak Foun	High	Peak Foun	1
Peak Foun	Peak Foun	Peak Foun	Peak Foun	Peak Foun	High	Peak Foun	Peak Foun	1
Peak Foun	Peak Foun	High	Not Found	High	Peak Foun	High	Peak Foun	1
Not Found	Peak Foun	Not Found	High	Peak Foun	High	Peak Foun	Peak Foun	1
Not Found	Not Found	Not Found	Not Found	Not Found	Peak Foun	Not Found	Not Found	1
Peak Foun	Peak Foun	Peak Foun	Peak Foun	Not Found	High	Peak Foun	Peak Foun	1
Peak Foun	Peak Foun	Peak Foun	High	High	High	Peak Foun	High	1
Peak Foun	Peak Foun	High	High	Peak Foun	High	High	High	1
Peak Foun	Peak Foun	Peak Foun	Peak Foun	High	High	Peak Foun	Peak Foun	1
Peak Foun	Peak Foun	Peak Foun	Peak Foun	Peak Foun	Peak Foun	High	Peak Foun	1
High	High	High	High	High	Peak Foun	High	Peak Foun	1
Peak Foun	High	High	Peak Foun	Peak Foun	High	Peak Foun	Peak Foun	1
Peak Foun	Peak Foun	Peak Foun	Peak Foun	High	High	High	Peak Foun	1

Peak Foun	Peak Foun	Peak Foun	Not Found	High	High	Peak Foun	Not Found		1
Not Found	Not Found	Peak Foun	Peak Foun	Not Found	High	High	Peak Foun		1
Peak Foun	Peak Foun	Not Found	Not Found	Not Found	High	Not Found	Not Found		1
Not Found	Peak Foun	High	Peak Foun	Peak Foun	Peak Foun	Peak Foun	Peak Foun		1
High	High	High	High	Not Found	High	High	Not Found		1
Not Found	Not Found	Peak Foun	Not Found	Peak Foun	High	High	Peak Foun		1
Peak Foun	High	Peak Foun	High	Peak Foun	Peak Foun	Peak Foun	Peak Foun		1
High	High	Peak Foun	High	High	High	High	High		1
High	Peak Foun	Peak Foun	Peak Foun	High	High	High	Peak Foun		1
Not Found	High	High	High	Not Found	High	High	Peak Foun		1
Peak Foun	Peak Foun	Peak Foun	Peak Foun	Peak Foun	Peak Foun	High	Peak Foun		1
Not Found	High	Not Found	High	Not Found	High	Not Found	High		1
Peak Foun	High	High	High	High	High	High	High		1
Peak Foun	Not Found	Peak Foun	Not Found	Not Found	High	Not Found	Not Found		1
Peak Foun	High	Peak Foun	Peak Foun	High	High	High	Peak Foun		1
High	Peak Foun	Peak Foun	Peak Foun	Peak Foun	Peak Foun	Peak Foun	Not Found		1
Peak Foun	Peak Foun	High	Peak Foun	Peak Foun	High	High	Peak Foun		1
High	High	High	High	High	High	High	High		1
Not Found	Not Found	High	Not Found	High	High	High	Not Found		1
Not Found	Not Found	High	Not Found	Peak Foun	High	Peak Foun	Not Found		1
Not Found	Not Found	Peak Foun	Peak Foun	Peak Foun	Peak Foun	High	Peak Foun		1
Peak Foun	Peak Foun	High	High	Peak Foun	High	High	High		1
Peak Foun	Peak Foun	High	High	Peak Foun	High	High	High		1
Not Found	Not Found	Not Found	Not Found	Peak Foun	High	High	Peak Foun		1
Peak Foun	Peak Foun	Not Found	Not Found	Not Found	Peak Foun	Peak Foun	Peak Foun		1
Not Found	Not Found	Not Found	Not Found	Peak Foun	High	High	Peak Foun		1
Peak Foun	Peak Foun	Peak Foun	High	High	High	High	High		1
Peak Foun	Peak Foun	Peak Foun	Not Found	Peak Foun	High	Peak Foun	Not Found		1
Not Found	Not Found	Not Found	Not Found	High	High	Peak Foun	Not Found		1
High	High	High	High	High	High	Peak Foun	Not Found		1
Not Found	Peak Foun	High	High	High	High	High	Not Found		1
Peak Foun	Not Found	Not Found	Peak Foun	Peak Foun	High	High	Not Found		1
Not Found	High	Peak Foun	Peak Foun	Peak Foun	High	Peak Foun	Peak Foun		1
Not Found	Not Found	Not Found	Not Found	Not Found	Not Found	Peak Foun	Peak Foun		1
Not Found	Not Found	Not Found	Peak Foun	Peak Foun	Peak Foun	Peak Foun	Peak Foun		1
Peak Foun	Not Found	Not Found	Not Found	High	Peak Foun	High	Not Found		1
Peak Foun	Peak Foun	High	Peak Foun	Peak Foun	Peak Foun	High	High		1
Not Found	Not Found	Peak Foun	Not Found	Not Found	Peak Foun	High	Peak Foun		1
High	Peak Foun	High	Peak Foun	High	High	High	High		1
Not Found	Peak Foun	Peak Foun	Not Found	Not Found	Peak Foun	High	Not Found		1
Peak Foun	Peak Foun	Peak Foun	Peak Foun	Peak Foun	High	High	Peak Foun		1
Peak Foun	Not Found	Peak Foun	Peak Foun	High	High	High	Peak Foun		1
Peak Foun	Peak Foun	Peak Foun	Peak Foun	Peak Foun	High	Peak Foun	High		1
Peak Foun	Peak Foun	Peak Foun	Peak Foun	Peak Foun	High	Peak Foun	High		1
Peak Foun	High	High	Peak Foun	High	High	High	Peak Foun		1

Peak Foun	Peak Foun	Peak Foun	Peak Foun	High	High	Peak Foun	Peak Foun	1
Peak Foun	Peak Foun	Peak Foun	Peak Foun	Peak Foun	Not Found	Not Found	Peak Foun	1
Peak Foun	Peak Foun	High	High	Peak Foun	High	High	Peak Foun	1
Peak Foun	Peak Foun	Peak Foun	Peak Foun	Peak Foun	High	Peak Foun	Peak Foun	1
Peak Foun	Peak Foun	Not Found	Not Found	Peak Foun	High	Peak Foun	Peak Foun	1
Peak Foun	Peak Foun	High	Peak Foun	Peak Foun	High	High	High	1
Peak Foun	Not Found	High	Peak Foun	Not Found	Peak Foun	Peak Foun	High	1
Peak Foun	Peak Foun	Peak Foun	Peak Foun	High	Peak Foun	Peak Foun	High	1
Peak Foun	Peak Foun	Peak Foun	Peak Foun	High	Peak Foun	Peak Foun	Peak Foun	1
High	High	Peak Foun	Peak Foun	Peak Foun	Not Found	Peak Foun	Not Found	1
Peak Foun	High	Peak Foun	Peak Foun	Peak Foun	Peak Foun	Peak Foun	Peak Foun	1
High	Peak Foun	High	High	High	High	High	High	1
Peak Foun	Not Found	Peak Foun	Peak Foun	Peak Foun	Peak Foun	Peak Foun	Peak Foun	1
Not Found	Not Found	Not Found	Peak Foun	High	High	Peak Foun	Peak Foun	1
Peak Foun	Peak Foun	High	Peak Foun	High	Peak Foun	Peak Foun	Peak Foun	1
Peak Foun	Peak Foun	Peak Foun	Peak Foun	Peak Foun	High	High	Peak Foun	1
Not Found	Peak Foun	Peak Foun	Not Found	Not Found	High	High	Peak Foun	1
Peak Foun	Peak Foun	Peak Foun	High	Peak Foun	Peak Foun	Peak Foun	Not Found	1
Not Found	Not Found	Not Found	Peak Foun	Peak Foun	High	Peak Foun	Peak Foun	1
Not Found	Not Found	Not Found	Peak Foun	Peak Foun	Not Found	High	Not Found	1
Peak Foun	High	Peak Foun	Peak Foun	High	High	High	Peak Foun	1
Peak Foun	Peak Foun	Peak Foun	Peak Foun	Peak Foun	High	Peak Foun	Not Found	1
Peak Foun	Not Found	High	Not Found	High	High	Peak Foun	Peak Foun	1
High	Peak Foun	Peak Foun	Peak Foun	Peak Foun	High	High	Peak Foun	1
High	High	High	High	High	High	High	High	1
Peak Foun	High	High	High	High	High	High	High	1
Peak Foun	Peak Foun	Peak Foun	Peak Foun	Peak Foun	High	High	Peak Foun	1
High	High	High	High	High	High	Peak Foun	High	1
High	Peak Foun	High	Peak Foun	Peak Foun	High	High	High	1
Peak Foun	Peak Foun	Peak Foun	Peak Foun	Peak Foun	High	Peak Foun	Peak Foun	1
Peak Foun	Peak Foun	Peak Foun	High	Peak Foun	High	High	Peak Foun	1
Peak Foun	Peak Foun	Peak Foun	Peak Foun	High	Peak Foun	Peak Foun	Peak Foun	1
Not Found	Peak Foun	Peak Foun	High	Peak Foun	High	High	Peak Foun	1
Peak Foun	High	Peak Foun	Peak Foun	Not Found	Not Found	Not Found	Not Found	1
Peak Foun	Peak Foun	Peak Foun	High	Peak Foun	Peak Foun	High	High	1
Peak Foun	Peak Foun	Peak Foun	Peak Foun	Peak Foun	Peak Foun	Peak Foun	Peak Foun	1
Peak Foun	Not Found	Peak Foun	Peak Foun	Peak Foun	High	High	Not Found	1
Peak Foun	Peak Foun	Peak Foun	Not Found	Peak Foun	High	Peak Foun	Not Found	1
Peak Foun	Peak Foun	Peak Foun	Peak Foun	High	Peak Foun	Peak Foun	Peak Foun	1
Peak Foun	High	High	Peak Foun	High	High	High	Peak Foun	1
Peak Foun	Peak Foun	Not Found	Peak Foun	Peak Foun	Peak Foun	Peak Foun	Not Found	1
High	Peak Foun	Peak Foun	Peak Foun	High	High	Peak Foun	Peak Foun	1
Not Found	Peak Foun	Not Found	Peak Foun	Peak Foun	Peak Foun	Peak Foun	Not Found	1
Peak Foun	High	Peak Foun	Peak Foun	Not Found	Not Found	Not Found	Not Found	1
Peak Foun	High	Peak Foun	Peak Foun	High	High	High	High	1

Not Found	Not Found	High	High	Not Found	Peak Foun	Peak Foun	Peak Foun	1
Peak Foun	Peak Foun	Peak Foun	High	Peak Foun	Peak Foun	High	Peak Foun	1
High	High	Peak Foun	Peak Foun	High	Peak Foun	Peak Foun	Peak Foun	1
High	Not Found	Not Found	Not Found	Not Found	Not Found	Peak Foun	Not Found	1
Not Found	Not Found	Not Found	Peak Foun	Not Found	High	High	Peak Foun	1
Peak Foun	Peak Foun	Peak Foun	Peak Foun	High	High	Peak Foun	Peak Foun	1
Peak Foun	Peak Foun	Peak Foun	Peak Foun	High	High	High	Peak Foun	1
Not Found	Not Found	Peak Foun	Not Found	Not Found	High	Peak Foun	Not Found	1
Not Found	Not Found	Not Found	Not Found	Peak Foun	High	Peak Foun	Peak Foun	1
Peak Foun	High	Peak Foun	Peak Foun	Peak Foun	High	Peak Foun	Peak Foun	1
Peak Foun	Peak Foun	Peak Foun	Peak Foun	Peak Foun	High	Peak Foun	Not Found	1
High	High	High	High	High	High	High	Not Found	1
Peak Foun	Peak Foun	Not Found	Peak Foun	High	Peak Foun	High	High	1
Peak Foun	Peak Foun	High	Peak Foun	High	Peak Foun	Peak Foun	Peak Foun	1
Not Found	Peak Foun	Peak Foun	Not Found	High	High	High	Not Found	1
Not Found	Not Found	Not Found	Peak Foun	Peak Foun	High	Peak Foun	Peak Foun	1
Peak Foun	Peak Foun	Peak Foun	Peak Foun	Peak Foun	High	Peak Foun	Peak Foun	1
Not Found	Peak Foun	High	Peak Foun	High	High	High	Not Found	1
Peak Foun	Peak Foun	Peak Foun	High	Peak Foun	Peak Foun	High	Peak Foun	1
Peak Foun	Peak Foun	High	Peak Foun	Peak Foun	Peak Foun	Peak Foun	Peak Foun	1
Peak Foun	Peak Foun	Peak Foun	High	Peak Foun	High	Peak Foun	Peak Foun	1
Peak Foun	Peak Foun	High	Peak Foun	Not Found	High	Peak Foun	Peak Foun	1
Peak Foun	Peak Foun	High	Peak Foun	High	High	Peak Foun	Peak Foun	1
Peak Foun	Peak Foun	High	Peak Foun	Peak Foun	Peak Foun	Peak Foun	High	1
High	High	High	High	High	Not Found	Not Found	High	1
Peak Foun	Peak Foun	Peak Foun	High	Peak Foun	Peak Foun	Peak Foun	High	1
Not Found	Not Found	Not Found	Peak Foun	High	High	Peak Foun	Not Found	1
Peak Foun	Not Found	Peak Foun	Peak Foun	Peak Foun	High	Peak Foun	Peak Foun	1
High	High	High	Peak Foun	High	High	High	Peak Foun	1
Peak Foun	Peak Foun	High	Peak Foun	Peak Foun	Peak Foun	Peak Foun	Peak Foun	1
High	High	High	Peak Foun	High	High	High	High	1
Peak Foun	Peak Foun	Peak Foun	Peak Foun	Peak Foun	High	Peak Foun	Peak Foun	1
Peak Foun	Peak Foun	Peak Foun	Not Found	Peak Foun	High	Peak Foun	Not Found	1
Peak Foun	Peak Foun	Peak Foun	High	Peak Foun	High	High	High	1
Peak Foun	Peak Foun	Peak Foun	Peak Foun	High	High	High	Peak Foun	1
Peak Foun	Peak Foun	High	Peak Foun	Peak Foun	High	High	High	1
High	Not Found	Peak Foun	Peak Foun	High	Not Found	Peak Foun	Peak Foun	1
Peak Foun	Peak Foun	Peak Foun	Peak Foun	High	Peak Foun	High	Peak Foun	1
Peak Foun	Not Found	Not Found	Peak Foun	High	High	Peak Foun	High	1
Peak Foun	Peak Foun	High	Peak Foun	Peak Foun	Peak Foun	Peak Foun	Peak Foun	1
Peak Foun	Peak Foun	High	High	High	High	High	High	1
Peak Foun	Peak Foun	Peak Foun	High	High	High	High	Peak Foun	1
Peak Foun	Not Found	Peak Foun	Peak Foun	High	Peak Foun	High	Peak Foun	1
High	High	High	High	High	High	High	High	1
Peak Foun	Peak Foun	Peak Foun	Peak Foun	Peak Foun	High	Peak Foun	Peak Foun	1

Not Found	Peak Foun	Peak Foun	Peak Foun	Peak Foun	Peak Foun	High	Peak Foun	1
Peak Foun	Peak Foun	High	Peak Foun	Peak Foun	Peak Foun	Not Found	Peak Foun	1
Not Found	Peak Foun	Peak Foun	Peak Foun	Peak Foun	High	Peak Foun	Peak Foun	1
Not Found	Not Found	Not Found	Not Found	Peak Foun	High	Peak Foun	Peak Foun	1
Peak Foun	Peak Foun	Peak Foun	Peak Foun	Peak Foun	Peak Foun	Peak Foun	Peak Foun	1
Not Found	Not Found	Not Found	Not Found	Not Found	Not Found	High	Peak Foun	1
High	High	High	Peak Foun	Peak Foun	Peak Foun	High	Peak Foun	1
Peak Foun	Peak Foun	Not Found	Peak Foun	Not Found	Not Found	Peak Foun	Peak Foun	1
Peak Foun	Peak Foun	Peak Foun	Peak Foun	High	High	Peak Foun	Peak Foun	1
Peak Foun	High	Peak Foun	Peak Foun	Not Found	High	Peak Foun	Peak Foun	1
Peak Foun	High	Peak Foun	High	Peak Foun	High	High	High	1
High	Peak Foun	Peak Foun	Not Found	High	Peak Foun	Peak Foun	Peak Foun	1
Not Found	Not Found	Peak Foun	Peak Foun	High	High	High	Not Found	1
High	Peak Foun	Peak Foun	Peak Foun	High	High	Peak Foun	Peak Foun	1
Peak Foun	Not Found	Peak Foun	High	Peak Foun	High	High	Peak Foun	1
Not Found	Not Found	Not Found	Not Found	Not Found	High	High	Not Found	1
Peak Foun	Not Found	Peak Foun	High	High	High	Peak Foun	Peak Foun	1
Not Found	Peak Foun	Peak Foun	Peak Foun	Peak Foun	Peak Foun	High	Peak Foun	1
Peak Foun	Peak Foun	Peak Foun	Peak Foun	Peak Foun	High	High	Peak Foun	1
Peak Foun	Peak Foun	Peak Foun	Not Found	Peak Foun	Peak Foun	High	Peak Foun	1
Peak Foun	High	Not Found	Peak Foun	Peak Foun	High	Not Found	Peak Foun	1
High	Peak Foun	High	High	High	High	High	High	1
Peak Foun	Peak Foun	Not Found	Peak Foun	Peak Foun	Peak Foun	Peak Foun	Peak Foun	1
Peak Foun	Peak Foun	Peak Foun	Not Found	Peak Foun	High	Not Found	Not Found	1
Peak Foun	Not Found	Peak Foun	Peak Foun	High	Peak Foun	Peak Foun	Peak Foun	1
Peak Foun	High	High	High	Peak Foun	High	High	High	1
High	High	Peak Foun	Peak Foun	Peak Foun	Peak Foun	Peak Foun	Not Found	1
Peak Foun	Peak Foun	Peak Foun	Peak Foun	Peak Foun	High	High	Peak Foun	1
Peak Foun	High	Peak Foun	Peak Foun	High	High	Peak Foun	High	1
Peak Foun	Peak Foun	Peak Foun	Peak Foun	Peak Foun	Peak Foun	Peak Foun	Peak Foun	1
Peak Foun	Peak Foun	High	Peak Foun	High	High	High	High	1
High	Peak Foun	Peak Foun	Peak Foun	High	High	High	High	1
Peak Foun	High	Not Found	Peak Foun	Peak Foun	High	Peak Foun	Peak Foun	1
Peak Foun	Peak Foun	High	Not Found	Peak Foun	Not Found	Peak Foun	Not Found	1
Peak Foun	Peak Foun	Peak Foun	Peak Foun	High	High	High	Peak Foun	1
Not Found	Peak Foun	Peak Foun	Peak Foun	High	Peak Foun	High	Peak Foun	1
High	Peak Foun	Peak Foun	Peak Foun	Peak Foun	Peak Foun	High	Peak Foun	1
Peak Foun	Not Found	Peak Foun	Not Found	Peak Foun	Not Found	Not Found	Not Found	1
Peak Foun	Peak Foun	Peak Foun	Peak Foun	High	High	Peak Foun	Peak Foun	1
Peak Foun	Peak Foun	Peak Foun	Peak Foun	Peak Foun	Peak Foun	Peak Foun	Peak Foun	1
Not Found	Peak Foun	High	Peak Foun	Not Found	Peak Foun	High	High	1
Peak Foun	Peak Foun	High	Peak Foun	High	Peak Foun	Peak Foun	Peak Foun	1
Peak Foun	Peak Foun	Peak Foun	Peak Foun	Peak Foun	High	High	Peak Foun	1
Peak Foun	Peak Foun	Peak Foun	High	Peak Foun	High	High	Peak Foun	1
Peak Foun	High	Peak Foun	Not Found	Peak Foun	Peak Foun	Not Found	Not Found	1

Peak Foun	Peak Foun	High	Peak Foun	Peak Foun	High	Peak Foun	Peak Foun	1
High	Peak Foun	High	High	High	High	Peak Foun	High	1
Peak Foun	Peak Foun	High	High	High	Peak Foun	High	Peak Foun	1
Not Found	Not Found	Not Found	Not Found	Peak Foun	High	Not Found	Not Found	1
Peak Foun	Peak Foun	Peak Foun	Peak Foun	High	High	Peak Foun	Peak Foun	1
Peak Foun	High	Peak Foun	Peak Foun	Peak Foun	High	High	Not Found	1
Peak Foun	Peak Foun	Peak Foun	Peak Foun	High	High	Peak Foun	Peak Foun	1
Not Found	Not Found	Not Found	Not Found	Not Found	High	Not Found	Not Found	1
Peak Foun	Peak Foun	Peak Foun	Peak Foun	Peak Foun	Peak Foun	Peak Foun	Peak Foun	1
Peak Foun	Peak Foun	Peak Foun	Peak Foun	Peak Foun	Peak Foun	High	Peak Foun	1
Not Found	Peak Foun	Peak Foun	High	High	High	High	High	1
Peak Foun	Peak Foun	Not Found	Not Found	Peak Foun	Peak Foun	High	Peak Foun	1
Peak Foun	High	High	High	High	High	High	Peak Foun	1
Peak Foun	Peak Foun	Peak Foun	Peak Foun	Peak Foun	High	High	Peak Foun	1
High	Peak Foun	Peak Foun	Peak Foun	High	Peak Foun	Peak Foun	Peak Foun	1
Peak Foun	High	Not Found	Not Found	High	High	High	Not Found	1
Peak Foun	Peak Foun	Peak Foun	Not Found	High	High	High	Not Found	1
High	High	High	High	High	High	High	High	1
Peak Foun	Peak Foun	High	Peak Foun	Peak Foun	Peak Foun	High	Peak Foun	1
High	High	High	High	High	High	High	Peak Foun	1
Peak Foun	Not Found	Not Found	Peak Foun	Peak Foun	High	Not Found	Not Found	1
Not Found	Peak Foun	Not Found	Peak Foun	Peak Foun	High	Peak Foun	Not Found	1
Not Found	Not Found	Not Found	Peak Foun	Peak Foun	High	Peak Foun	Peak Foun	1
Peak Foun	Not Found	Not Found	Not Found	High	High	High	Not Found	1
Not Found	Not Found	Not Found	High	Not Found	High	Peak Foun	Peak Foun	1
Peak Foun	Peak Foun	High	High	Peak Foun	Peak Foun	Peak Foun	High	1
Peak Foun	Peak Foun	Peak Foun	Peak Foun	High	High	High	Peak Foun	1
Peak Foun	Not Found	Peak Foun	High	High	High	High	Peak Foun	1
Not Found	Peak Foun	Peak Foun	Peak Foun	Peak Foun	High	High	Peak Foun	1
Not Found	Not Found	Peak Foun	Peak Foun	Peak Foun	High	Peak Foun	Not Found	1
Not Found	Peak Foun	Peak Foun	High	Peak Foun	Peak Foun	High	Peak Foun	1
Peak Foun	Peak Foun	Peak Foun	Peak Foun	Peak Foun	High	Peak Foun	Peak Foun	1
Peak Foun	Peak Foun	Peak Foun	Peak Foun	Peak Foun	High	High	Peak Foun	1
High	High	Peak Foun	High	High	High	High	High	1
Peak Foun	Peak Foun	Peak Foun	Peak Foun	Peak Foun	High	Peak Foun	Peak Foun	1
Peak Foun	Peak Foun	Peak Foun	Peak Foun	Peak Foun	High	Peak Foun	Peak Foun	1
Peak Foun	Not Found	Peak Foun	High	High	High	High	High	1
Not Found	Not Found	Peak Foun	Peak Foun	Peak Foun	High	High	Peak Foun	1
High	Peak Foun	Peak Foun	Not Found	Peak Foun	High	Peak Foun	Peak Foun	1
Peak Foun	Peak Foun	Peak Foun	Peak Foun	High	High	Peak Foun	Peak Foun	1
High	High	High	High	Peak Foun	Peak Foun	Peak Foun	Peak Foun	1
Peak Foun	Not Found	High	High	Peak Foun	High	High	High	1
Peak Foun	Not Found	Not Found	Not Found	High	High	Not Found	Not Found	1
Peak Foun	Peak Foun	Peak Foun	Peak Foun	Peak Foun	Peak Foun	Peak Foun	Peak Foun	1
Peak Foun	Not Found	Peak Foun	Peak Foun	Peak Foun	Peak Foun	High	Peak Foun	1

Not Found	Peak Foun	High	Peak Foun	Peak Foun	Peak Foun	Peak Foun	Peak Foun	1
High	High	High	Peak Foun	High	High	High	Peak Foun	1
Peak Foun	Peak Foun	Peak Foun	High	High	High	High	High	1
Peak Foun	Peak Foun	Peak Foun	Peak Foun	Peak Foun	Peak Foun	High	Peak Foun	1
Peak Foun	High	High	Peak Foun	Peak Foun	Not Found	Peak Foun	Not Found	1
Peak Foun	Peak Foun	Peak Foun	Peak Foun	Peak Foun	High	High	Peak Foun	1
Peak Foun	High	Peak Foun	Peak Foun	Peak Foun	Peak Foun	Peak Foun	Peak Foun	1
Not Found	Not Found	Not Found	Not Found	Not Found	High	Not Found	Not Found	1
Peak Foun	Not Found	Peak Foun	High	High	High	High	Peak Foun	1
Peak Foun	Peak Foun	Peak Foun	High	Peak Foun	High	High	Peak Foun	1
Not Found	Not Found	Not Found	Peak Foun	Peak Foun	High	Not Found	Not Found	1
Peak Foun	Peak Foun	Peak Foun	Not Found	Peak Foun	High	Peak Foun	Not Found	1
High	High	Not Found	Not Found	Not Found	Not Found	Not Found	Not Found	1
Not Found	Peak Foun	Peak Foun	Not Found	Peak Foun	Peak Foun	High	Peak Foun	1
Peak Foun	Not Found	Peak Foun	Peak Foun	Peak Foun	Peak Foun	High	Peak Foun	1
Not Found	Peak Foun	High	Not Found	Not Found	High	High	Not Found	1
Peak Foun	Peak Foun	High	Peak Foun	Peak Foun	Peak Foun	Peak Foun	Peak Foun	1
Not Found	Not Found	High	Not Found	Not Found	Not Found	Not Found	Not Found	1
Not Found	Not Found	Not Found	Not Found	Peak Foun	High	Not Found	Not Found	1
High	Peak Foun	Peak Foun	Peak Foun	Peak Foun	High	Peak Foun	Peak Foun	1
Not Found	Peak Foun	Peak Foun	Not Found	Peak Foun	High	High	Not Found	1
Not Found	Peak Foun	Peak Foun	Peak Foun	Peak Foun	Peak Foun	High	High	1
Peak Foun	Peak Foun	Peak Foun	Peak Foun	High	Peak Foun	High	Peak Foun	1
Peak Foun	Peak Foun	High	Peak Foun	High	Peak Foun	High	Peak Foun	1
Peak Foun	Not Found	Not Found	Not Found	Peak Foun	High	Peak Foun	Peak Foun	1
Not Found	Not Found	Not Found	Not Found	Peak Foun	Peak Foun	Peak Foun	Not Found	1
Peak Foun	High	Peak Foun	Peak Foun	High	High	High	Peak Foun	1
Not Found	Not Found	Peak Foun	Peak Foun	Peak Foun	Not Found	Peak Foun	High	1
Peak Foun	Peak Foun	Peak Foun	Not Found	Not Found	High	Peak Foun	Peak Foun	1
High	Not Found	High	Not Found	High	High	High	High	1
Not Found	Peak Foun	High	Peak Foun	Peak Foun	High	Peak Foun	Peak Foun	1
Not Found	Not Found	Not Found	Not Found	Peak Foun	Not Found	High	Not Found	1
Peak Foun	Peak Foun	High	Peak Foun	Peak Foun	Not Found	High	Not Found	1
Peak Foun	Not Found	Not Found	Not Found	Peak Foun	Peak Foun	Not Found	Not Found	1
Peak Foun	High	High	Peak Foun	Not Found	High	Peak Foun	Not Found	1
Not Found	Not Found	Not Found	Not Found	Peak Foun	High	High	Peak Foun	1
Not Found	Peak Foun	Not Found	Not Found	Peak Foun	High	High	Not Found	1
High	High	High	Peak Foun	High	Peak Foun	High	Peak Foun	1
Not Found	Not Found	Not Found	Not Found	High	Peak Foun	Peak Foun	Peak Foun	1
Peak Foun	High	Peak Foun	Peak Foun	Peak Foun	Peak Foun	High	Peak Foun	1
Peak Foun	Peak Foun	Peak Foun	Peak Foun	High	High	Peak Foun	Peak Foun	1
Peak Foun	Peak Foun	High	Peak Foun	High	Peak Foun	Peak Foun	Not Found	1
High	Peak Foun	Peak Foun	Peak Foun	High	High	High	Peak Foun	1
Not Found	Not Found	Not Found	Peak Foun	Not Found	High	Not Found	Not Found	1
High	High	High	High	Peak Foun	Peak Foun	High	Peak Foun	1

High	Peak Foun	High	Peak Foun	Peak Foun	Peak Foun	High	Peak Foun	1
Peak Foun	Peak Foun	Peak Foun	High	High	High	High	High	1
Peak Foun	Peak Foun	Not Found	Peak Foun	High	High	High	Not Found	1
High	High	Peak Foun	High	Peak Foun	High	Peak Foun	High	1
Peak Foun	Peak Foun	Peak Foun	Peak Foun	Peak Foun	High	Peak Foun	Peak Foun	1
Not Found	Not Found	Peak Foun	Peak Foun	Peak Foun	High	High	Peak Foun	1
High	Peak Foun	High	High	High	High	High	Peak Foun	1
High	Peak Foun	Peak Foun	High	Peak Foun	Peak Foun	High	Peak Foun	1
Peak Foun	Peak Foun	Peak Foun	Peak Foun	Peak Foun	High	High	Peak Foun	1
Peak Foun	Peak Foun	Peak Foun	Peak Foun	Peak Foun	Peak Foun	High	Peak Foun	1
Peak Foun	High	Not Found	Peak Foun	Peak Foun	High	Peak Foun	Peak Foun	1
Not Found	Peak Foun	Not Found	Peak Foun	Peak Foun	High	Peak Foun	Peak Foun	1
Not Found	Not Found	Not Found	Peak Foun	Peak Foun	Peak Foun	High	Peak Foun	1
Peak Foun	Peak Foun	Peak Foun	Peak Foun	High	High	Peak Foun	Peak Foun	1
Peak Foun	High	Peak Foun	Peak Foun	Peak Foun	Not Found	Peak Foun	Peak Foun	1
High	Peak Foun	Peak Foun	Peak Foun	Peak Foun	Peak Foun	Peak Foun	Peak Foun	1
Not Found	Not Found	Not Found	Peak Foun	Not Found	Not Found	Peak Foun	Peak Foun	1
Not Found	Not Found	Not Found	Peak Foun	Peak Foun	Peak Foun	Peak Foun	Peak Foun	1
Not Found	Not Found	Peak Foun	Peak Foun	Peak Foun	Peak Foun	High	Peak Foun	1
Peak Foun	Not Found	Peak Foun	Peak Foun	Peak Foun	High	Peak Foun	Not Found	1
Peak Foun	Peak Foun	High	Peak Foun	Peak Foun	Peak Foun	Peak Foun	Peak Foun	1
Not Found	Not Found	Not Found	Not Found	Not Found	High	High	Peak Foun	1
High	High	High	High	High	High	High	High	1
Peak Foun	Peak Foun	High	Not Found	High	High	High	Not Found	1
Not Found	Peak Foun	Peak Foun	Peak Foun	Peak Foun	High	Peak Foun	High	1
Not Found	Not Found	Peak Foun	Not Found	High	Not Found	Not Found	Not Found	1
Peak Foun	High	High	High	High	High	Peak Foun	Peak Foun	1
Peak Foun	Peak Foun	Peak Foun	Peak Foun	Peak Foun	Peak Foun	High	Peak Foun	1
Peak Foun	Peak Foun	Peak Foun	Peak Foun	High	Peak Foun	Peak Foun	Peak Foun	1
High	High	Peak Foun	High	Peak Foun	Peak Foun	Peak Foun	Not Found	1
Peak Foun	Peak Foun	Peak Foun	Peak Foun	Peak Foun	Peak Foun	High	Peak Foun	1
Peak Foun	Peak Foun	Peak Foun	Not Found	Not Found	High	Peak Foun	Not Found	1
Peak Foun	High	High	High	High	Peak Foun	High	Peak Foun	1
Not Found	Not Found	Not Found	Not Found	High	Peak Foun	Peak Foun	Not Found	1
Peak Foun	Peak Foun	Peak Foun	Peak Foun	Peak Foun	High	High	Peak Foun	1
High	Not Found	Not Found	High	Peak Foun	Peak Foun	Not Found	Not Found	1
Peak Foun	High	High	Peak Foun	High	High	High	High	1
Peak Foun	High	Peak Foun	Peak Foun	Peak Foun	Peak Foun	High	Peak Foun	1
High	High	High	Peak Foun	High	High	Peak Foun	Peak Foun	1
High	Not Found	High	Peak Foun	High	High	High	Peak Foun	1
Peak Foun	Peak Foun	High	Peak Foun	Peak Foun	High	High	Peak Foun	1
Peak Foun	Peak Foun	High	Peak Foun	Peak Foun	High	Peak Foun	Peak Foun	1
Not Found	Not Found	Peak Foun	Peak Foun	Peak Foun	High	Peak Foun	Peak Foun	1
Peak Foun	Peak Foun	Peak Foun	High	Peak Foun	Not Found	Peak Foun	Peak Foun	1
Peak Foun	Peak Foun	Peak Foun	Peak Foun	High	High	High	Peak Foun	1

Peak Foun	Peak Foun	High	Peak Foun	High	High	Peak Foun	Peak Foun	1
Not Found	Not Found	Not Found	Peak Foun	Peak Foun	Peak Foun	Peak Foun	Peak Foun	1
Not Found	Not Found	Peak Foun	High	High	Peak Foun	Peak Foun	Not Found	1
Not Found	Peak Foun	Not Found	Not Found	Peak Foun	High	Not Found	Not Found	1
Peak Foun	High	Peak Foun	Peak Foun	Peak Foun	Peak Foun	Peak Foun	Peak Foun	1
Not Found	Not Found	Not Found	Not Found	Peak Foun	High	Peak Foun	Not Found	1
Peak Foun	Peak Foun	Peak Foun	Peak Foun	Peak Foun	Not Found	Not Found	Peak Foun	1
Peak Foun	High	High	Peak Foun	Peak Foun	Not Found	Peak Foun	Peak Foun	1
Not Found	Peak Foun	Not Found	Not Found	Peak Foun	High	Peak Foun	Not Found	1
Not Found	Peak Foun	Not Found	Peak Foun	Peak Foun	Peak Foun	Peak Foun	Peak Foun	1
Not Found	Peak Foun	Peak Foun	Not Found	Peak Foun	High	Peak Foun	Peak Foun	1
Peak Foun	Peak Foun	Peak Foun	Peak Foun	Peak Foun	Peak Foun	Peak Foun	Peak Foun	1
Peak Foun	Peak Foun	Peak Foun	Not Found	Not Found	High	Peak Foun	Not Found	1
Not Found	Not Found	Not Found	Not Found	Not Found	Peak Foun	Peak Foun	Not Found	1
Peak Foun	Peak Foun	Peak Foun	Peak Foun	High	High	Peak Foun	Peak Foun	1
Peak Foun	Peak Foun	Peak Foun	Peak Foun	High	Peak Foun	High	Peak Foun	1
High	Peak Foun	High	Peak Foun	High	High	High	High	1
Peak Foun	Peak Foun	High	Peak Foun	Peak Foun	Peak Foun	Peak Foun	Peak Foun	1
Peak Foun	Peak Foun	High	Peak Foun	Peak Foun	Peak Foun	Peak Foun	Peak Foun	1
Not Found	Not Found	Peak Foun	Not Found	Not Found	Not Found	Not Found	Peak Foun	1
Peak Foun	High	Peak Foun	Peak Foun	High	High	High	Peak Foun	1
High	Peak Foun	Not Found	Peak Foun	High	Peak Foun	Not Found	Peak Foun	1
Peak Foun	Peak Foun	High	Peak Foun	Peak Foun	High	High	High	1
Not Found	High	Not Found	Peak Foun	Peak Foun	High	Not Found	Peak Foun	1
High	High	High	High	Peak Foun	Peak Foun	Peak Foun	Peak Foun	1
Peak Foun	Not Found	Peak Foun	Peak Foun	Peak Foun	High	High	Peak Foun	1
High	Not Found	Not Found	Not Found	High	Not Found	Not Found	Not Found	1
High	Peak Foun	Peak Foun	Peak Foun	High	High	Peak Foun	Peak Foun	1
Not Found	Not Found	Not Found	Not Found	Not Found	High	Peak Foun	Not Found	1
Peak Foun	Peak Foun	Peak Foun	Peak Foun	High	Peak Foun	Peak Foun	Peak Foun	1
Peak Foun	Peak Foun	Peak Foun	Peak Foun	Peak Foun	High	Peak Foun	Peak Foun	1
Peak Foun	Peak Foun	High	Not Found	Peak Foun	Peak Foun	High	Not Found	1
Peak Foun	Peak Foun	Peak Foun	Peak Foun	High	High	High	Peak Foun	1
Peak Foun	Peak Foun	Peak Foun	Peak Foun	Peak Foun	High	Peak Foun	Not Found	1
Peak Foun	Peak Foun	Peak Foun	Peak Foun	Peak Foun	Peak Foun	Peak Foun	Peak Foun	1
Peak Foun	Peak Foun	Peak Foun	Peak Foun	High	High	High	Peak Foun	1
Not Found	Not Found	Not Found	Not Found	Peak Foun	High	High	Not Found	1
Not Found	High	Peak Foun	Not Found	Peak Foun	Peak Foun	Not Found	Not Found	1
Peak Foun	Not Found	Peak Foun	Not Found	High	High	Peak Foun	Peak Foun	1
Peak Foun	Peak Foun	Peak Foun	Not Found	High	Peak Foun	Peak Foun	Peak Foun	1
Peak Foun	Peak Foun	Peak Foun	Peak Foun	Peak Foun	High	Peak Foun	Peak Foun	1
Not Found	Peak Foun	Peak Foun	Peak Foun	Not Found	High	Peak Foun	Peak Foun	1
Peak Foun	Peak Foun	Peak Foun	Not Found	High	High	Peak Foun	Peak Foun	1
Peak Foun	Not Found	Not Found	Not Found	Peak Foun	High	Peak Foun	Not Found	1
Peak Foun	High	Peak Foun	High	Peak Foun	Peak Foun	Peak Foun	High	1

Peak Foun	Peak Foun	Peak Foun	Peak Foun	Peak Foun	Peak Foun	Peak Foun	Peak Foun	1
High	Peak Foun	Peak Foun	High	Peak Foun	Peak Foun	Peak Foun	Peak Foun	1
Peak Foun	Peak Foun	Peak Foun	High	High	Peak Foun	Peak Foun	High	1
Peak Foun	Peak Foun	Peak Foun	Peak Foun	Peak Foun	High	High	Peak Foun	1
Not Found	Not Found	High	Not Found	Not Found	Peak Foun	High	Peak Foun	1
Peak Foun	High	High	Not Found	High	High	Not Found	Peak Foun	1
Not Found	Peak Foun	Peak Foun	Peak Foun	Not Found	High	Peak Foun	Not Found	1
High	High	High	High	High	High	High	High	1
Peak Foun	Peak Foun	Peak Foun	Peak Foun	Peak Foun	Peak Foun	Peak Foun	Peak Foun	1
Peak Foun	Peak Foun	Not Found	Not Found	Peak Foun	High	High	Peak Foun	1
Not Found	Peak Foun	Peak Foun	High	Not Found	Peak Foun	Peak Foun	Not Found	1
Peak Foun	Not Found	Not Found	Not Found	Peak Foun	Peak Foun	Not Found	Not Found	1
Peak Foun	Peak Foun	Peak Foun	High	Peak Foun	High	Peak Foun	Peak Foun	1
Not Found	Not Found	Peak Foun	Peak Foun	Peak Foun	High	High	Peak Foun	1
Peak Foun	Peak Foun	Peak Foun	Peak Foun	High	Peak Foun	High	Not Found	1
Not Found	Not Found	Not Found	Peak Foun	Not Found	Peak Foun	Not Found	Not Found	1
Peak Foun	Peak Foun	High	High	Peak Foun	Peak Foun	Peak Foun	Peak Foun	1
Peak Foun	High	High	Peak Foun	Peak Foun	Peak Foun	Peak Foun	Not Found	1
Peak Foun	Peak Foun	Not Found	Not Found	Peak Foun	High	High	Peak Foun	1
Not Found	Not Found	Not Found	Peak Foun	Peak Foun	Peak Foun	Peak Foun	Peak Foun	1
Not Found	Not Found	Not Found	Not Found	Peak Foun	Peak Foun	Peak Foun	Not Found	1
Peak Foun	Peak Foun	Peak Foun	Peak Foun	Peak Foun	High	Peak Foun	Peak Foun	1
Peak Foun	Peak Foun	Peak Foun	Peak Foun	High	Peak Foun	High	High	1
Not Found	Not Found	Not Found	Not Found	Not Found	Not Found	High	Not Found	1
Not Found	Not Found	Not Found	Peak Foun	Peak Foun	Peak Foun	Peak Foun	Not Found	1
High	High	Peak Foun	Peak Foun	Peak Foun	Peak Foun	Peak Foun	Peak Foun	1
High	High	High	High	High	High	High	High	1
Peak Foun	Peak Foun	Not Found	Peak Foun	Peak Foun	High	Peak Foun	Not Found	1
Peak Foun	Peak Foun	Peak Foun	Peak Foun	Peak Foun	High	High	Peak Foun	1
Not Found	High	High	Peak Foun	Not Found	High	High	Not Found	1
Not Found	Not Found	Peak Foun	Peak Foun	Peak Foun	Peak Foun	Peak Foun	Not Found	1
Not Found	Not Found	Not Found	Not Found	Peak Foun	High	Not Found	Not Found	1
Peak Foun	Not Found	High	Peak Foun	High	High	High	Peak Foun	1
Peak Foun	Peak Foun	Peak Foun	Peak Foun	High	Peak Foun	High	Peak Foun	1
Not Found	Not Found	Not Found	Not Found	Peak Foun	High	High	Peak Foun	1
High	Peak Foun	Peak Foun	Peak Foun	Peak Foun	Peak Foun	Peak Foun	Peak Foun	1
High	High	Peak Foun	Peak Foun	Peak Foun	Peak Foun	Peak Foun	Peak Foun	1
Not Found	Not Found	Not Found	Not Found	Not Found	Peak Foun	High	Not Found	1
Peak Foun	Not Found	High	Peak Foun	Peak Foun	High	Peak Foun	Peak Foun	1
Peak Foun	High	High	Peak Foun	Peak Foun	High	High	Peak Foun	1
Not Found	Not Found	Not Found	Peak Foun	High	High	High	High	1
Peak Foun	High	High	High	Peak Foun	High	Peak Foun	High	1
Peak Foun	Peak Foun	Peak Foun	High	Peak Foun	Peak Foun	Peak Foun	Peak Foun	1
Peak Foun	High	High	High	High	High	High	Peak Foun	1
High	High	Peak Foun	High	Peak Foun	High	High	Peak Foun	1

Not Found	Not Found	Not Found	Not Found	Peak Foun	High	Peak Foun	Not Found	1
Peak Foun	Not Found	Peak Foun	Not Found	Not Found	High	Peak Foun	Not Found	1
Not Found	Peak Foun	Peak Foun	Not Found	Peak Foun	High	Peak Foun	Peak Foun	1
Peak Foun	Peak Foun	Not Found	Peak Foun	High	High	High	High	1
Peak Foun	Not Found	Not Found	Not Found	Peak Foun	Peak Foun	Peak Foun	Not Found	1
High	Not Found	Peak Foun	Peak Foun	Peak Foun	High	High	Peak Foun	1
Peak Foun	High	High	Peak Foun	High	High	Peak Foun	Peak Foun	1
Not Found	Not Found	Not Found	Not Found	Peak Foun	High	Peak Foun	Not Found	1
Peak Foun	Peak Foun	Peak Foun	Peak Foun	Peak Foun	High	High	Peak Foun	1
High	Peak Foun	High	Not Found	Not Found	Peak Foun	Not Found	Peak Foun	1
Peak Foun	Not Found	Not Found	Peak Foun	Peak Foun	High	High	Peak Foun	1
Peak Foun	Peak Foun	High	Not Found	Peak Foun	High	High	High	1
Peak Foun	Peak Foun	High	Peak Foun	Not Found	Not Found	Peak Foun	Peak Foun	1
Not Found	Peak Foun	Not Found	Not Found	High	Peak Foun	Not Found	Not Found	1
Not Found	Not Found	Not Found	Not Found	Peak Foun	Peak Foun	Peak Foun	Not Found	1
High	High	High	Not Found	Peak Foun	High	High	Not Found	1
Peak Foun	High	Peak Foun	Peak Foun	Not Found	Peak Foun	Not Found	Not Found	1
Not Found	Not Found	Not Found	Not Found	Not Found	High	High	Peak Foun	1
Peak Foun	High	Peak Foun	High	High	High	High	High	1
Peak Foun	Peak Foun	High	Not Found	Peak Foun	High	High	Not Found	1
High	High	High	High	High	High	High	High	1
Not Found	Peak Foun	Peak Foun	Not Found	Peak Foun	Peak Foun	High	High	1
Peak Foun	Peak Foun	Peak Foun	Peak Foun	Peak Foun	Peak Foun	High	Peak Foun	1
Peak Foun	Not Found	Peak Foun	Peak Foun	Peak Foun	Peak Foun	High	Not Found	1
Not Found	Not Found	Not Found	Not Found	Not Found	High	Peak Foun	Not Found	1
High	Peak Foun	High	Peak Foun	Peak Foun	Peak Foun	Peak Foun	Peak Foun	1
High	High	High	Peak Foun	Peak Foun	Peak Foun	High	Peak Foun	1
Not Found	Peak Foun	Peak Foun	Peak Foun	Not Found	High	High	Peak Foun	1
Peak Foun	Peak Foun	Peak Foun	Peak Foun	Peak Foun	High	High	Peak Foun	1
Peak Foun	Peak Foun	Not Found	Peak Foun	Peak Foun	High	Not Found	Peak Foun	1
Not Found	Peak Foun	High	Not Found	Not Found	Peak Foun	Peak Foun	Peak Foun	1
Peak Foun	Peak Foun	High	High	Peak Foun	Peak Foun	Peak Foun	Peak Foun	1
Peak Foun	Peak Foun	Peak Foun	Peak Foun	High	High	Peak Foun	Peak Foun	1
Not Found	Peak Foun	High	Peak Foun	Peak Foun	Peak Foun	Peak Foun	Peak Foun	1
Peak Foun	Not Found	Not Found	Peak Foun	Peak Foun	High	High	Peak Foun	1
Peak Foun	Not Found	Peak Foun	Not Found	High	Peak Foun	Peak Foun	Peak Foun	1
High	Peak Foun	Peak Foun	Peak Foun	Peak Foun	High	High	Peak Foun	1
Peak Foun	Peak Foun	Peak Foun	Peak Foun	Peak Foun	Peak Foun	Peak Foun	Peak Foun	1
Peak Foun	Peak Foun	Peak Foun	Peak Foun	Peak Foun	High	Peak Foun	Peak Foun	1
Peak Foun	Peak Foun	Peak Foun	Peak Foun	High	High	High	Peak Foun	1
Not Found	Not Found	Not Found	Peak Foun	High	Peak Foun	High	Peak Foun	1
Not Found	Not Found	Not Found	Peak Foun	Peak Foun	High	Peak Foun	Peak Foun	1
Peak Foun	Peak Foun	Peak Foun	Peak Foun	Peak Foun	Peak Foun	High	High	1
High	Peak Foun	Peak Foun	Peak Foun	Peak Foun	High	Peak Foun	Peak Foun	1
Not Found	Not Found	Not Found	Not Found	Not Found	Not Found	Not Found	Not Found	1

Not Found	Not Found	Not Found	Not Found	High	High	High	Peak Foun	1
Peak Foun	Peak Foun	Not Found	Peak Foun	Peak Foun	Peak Foun	Not Found	Peak Foun	1
Not Found	Not Found	Peak Foun	High	Not Found	Peak Foun	High	Not Found	1
Peak Foun	Peak Foun	Not Found	Peak Foun	High	High	High	Not Found	1
Not Found	Not Found	Not Found	Not Found	Peak Foun	High	Peak Foun	Not Found	1
Not Found	Not Found	Not Found	Not Found	Peak Foun	High	High	Not Found	1
Peak Foun	High	High	Peak Foun	Peak Foun	High	High	Peak Foun	1
Peak Foun	Peak Foun	Peak Foun	Peak Foun	High	Not Found	High	Not Found	1
Peak Foun	High	High	Not Found	Peak Foun	Peak Foun	High	Peak Foun	1
High	High	High	High	High	High	High	High	1
Peak Foun	Peak Foun	Peak Foun	High	High	High	Peak Foun	Peak Foun	1
High	High	Peak Foun	Peak Foun	Peak Foun	Peak Foun	Peak Foun	Peak Foun	1
Not Found	Not Found	Not Found	Peak Foun	High	Not Found	Not Found	Not Found	1
Peak Foun	Peak Foun	Peak Foun	Not Found	Not Found	Peak Foun	Peak Foun	Not Found	1
Peak Foun	Peak Foun	Peak Foun	High	High	Peak Foun	High	Not Found	1
Peak Foun	High	Peak Foun	Peak Foun	Peak Foun	High	Peak Foun	Peak Foun	1
Peak Foun	Peak Foun	High	Peak Foun	High	High	High	Peak Foun	1
Peak Foun	Peak Foun	High	Peak Foun	High	High	High	High	1
High	High	Peak Foun	Peak Foun	Peak Foun	Peak Foun	High	High	1
Not Found	Peak Foun	High	Peak Foun	Not Found	Not Found	High	Not Found	1
Peak Foun	Peak Foun	Not Found	Peak Foun	Peak Foun	Peak Foun	Not Found	Peak Foun	1
High	High	High	High	Peak Foun	High	High	Peak Foun	1
Not Found	Not Found	Peak Foun	High	Peak Foun	Peak Foun	Peak Foun	Peak Foun	1
Not Found	Not Found	Not Found	Not Found	Not Found	Peak Foun	High	Not Found	1
Peak Foun	Peak Foun	Not Found	Peak Foun	Peak Foun	High	Peak Foun	Not Found	1
Peak Foun	High	High	High	High	High	High	Peak Foun	1
Not Found	High	High	Peak Foun	Peak Foun	Peak Foun	Peak Foun	Peak Foun	1
Peak Foun	Peak Foun	Peak Foun	Peak Foun	High	High	High	Peak Foun	1
High	Peak Foun	High	High	Peak Foun	Peak Foun	Peak Foun	Peak Foun	1
Not Found	High	Not Found	Peak Foun	Peak Foun	Not Found	Not Found	Peak Foun	1
Peak Foun	Peak Foun	Peak Foun	Not Found	Peak Foun	Not Found	Peak Foun	Not Found	1
Not Found	Peak Foun	Peak Foun	Peak Foun	Not Found	High	Peak Foun	Peak Foun	1
Peak Foun	Peak Foun	Not Found	Peak Foun	Peak Foun	Peak Foun	Peak Foun	Peak Foun	1
Peak Foun	Peak Foun	Peak Foun	Peak Foun	Peak Foun	Peak Foun	Peak Foun	Peak Foun	1
Not Found	Peak Foun	Peak Foun	Peak Foun	Peak Foun	High	High	Not Found	1
High	High	Peak Foun	Peak Foun	High	High	High	Peak Foun	1
Peak Foun	Peak Foun	Peak Foun	Peak Foun	Peak Foun	High	Peak Foun	Peak Foun	1
Not Found	Peak Foun	Peak Foun	Peak Foun	Peak Foun	Peak Foun	High	Peak Foun	1
Peak Foun	Peak Foun	Peak Foun	Peak Foun	Not Found	Peak Foun	Peak Foun	Not Found	1
Peak Foun	Peak Foun	Not Found	Peak Foun	Peak Foun	High	Peak Foun	Peak Foun	1
High	High	High	Peak Foun	Not Found	High	Peak Foun	High	1
Not Found	Not Found	Not Found	Not Found	Peak Foun	High	Peak Foun	Not Found	1
Peak Foun	Peak Foun	Peak Foun	Peak Foun	Peak Foun	Peak Foun	High	High	1
Not Found	Peak Foun	Peak Foun	High	Peak Foun	Peak Foun	Peak Foun	Peak Foun	1
Peak Foun	Peak Foun	Peak Foun	Peak Foun	High	High	High	Peak Foun	1

Peak Foun	Not Found	Peak Foun	Peak Foun	High	Peak Foun	Peak Foun	Peak Foun	1
Peak Foun	Peak Foun	Peak Foun	Not Found	High	Not Found	Peak Foun	Not Found	1
Peak Foun	Peak Foun	High	Peak Foun	High	High	Peak Foun	Peak Foun	1
Not Found	Not Found	Not Found	Not Found	Peak Foun	High	High	Not Found	1
Peak Foun	Peak Foun	Peak Foun	Peak Foun	Peak Foun	Peak Foun	Peak Foun	Not Found	1
Peak Foun	Peak Foun	Peak Foun	Peak Foun	Peak Foun	Peak Foun	High	Peak Foun	1
Peak Foun	High	High	Peak Foun	High	High	Peak Foun	Peak Foun	1
Not Found	Peak Foun	Peak Foun	Peak Foun	Not Found	High	Peak Foun	Peak Foun	1
Peak Foun	Not Found	Not Found	Not Found	Not Found	Not Found	Not Found	Not Found	1
Peak Foun	Peak Foun	Peak Foun	Peak Foun	High	Peak Foun	High	High	1
Peak Foun	Peak Foun	Peak Foun	High	High	Peak Foun	Peak Foun	High	1
Not Found	Not Found	Not Found	Peak Foun	High	Peak Foun	Peak Foun	Peak Foun	1
Not Found	Not Found	Not Found	Not Found	High	Peak Foun	Peak Foun	Peak Foun	1
Peak Foun	Peak Foun	High	Peak Foun	High	High	High	Peak Foun	1
Not Found	Peak Foun	Peak Foun	Peak Foun	Not Found	Not Found	Not Found	Not Found	1
Not Found	Not Found	Not Found	Not Found	Peak Foun	High	Peak Foun	Not Found	1
Peak Foun	Peak Foun	Peak Foun	Peak Foun	Peak Foun	Peak Foun	Peak Foun	High	1
Not Found	Peak Foun	Peak Foun	Not Found	Peak Foun	Peak Foun	Peak Foun	Peak Foun	1
Peak Foun	High	High	Peak Foun	High	Peak Foun	Peak Foun	Peak Foun	1
Not Found	Not Found	Peak Foun	Not Found	Peak Foun	Peak Foun	High	Peak Foun	1
Peak Foun	Peak Foun	Not Found	Peak Foun	Peak Foun	High	High	Peak Foun	1
Peak Foun	Peak Foun	Peak Foun	Not Found	High	High	Peak Foun	Peak Foun	1
Peak Foun	Peak Foun	Not Found	Peak Foun	High	Not Found	Peak Foun	Peak Foun	1
Peak Foun	Peak Foun	Not Found	Not Found	Peak Foun	High	Not Found	Not Found	1
Peak Foun	Peak Foun	High	Peak Foun	High	High	Peak Foun	Peak Foun	1
High	High	Not Found	Not Found	Not Found	Not Found	Not Found	Not Found	1
Peak Foun	Peak Foun	Peak Foun	Peak Foun	Peak Foun	High	High	Peak Foun	1
High	Peak Foun	High	High	High	High	Peak Foun	High	1
Peak Foun	Peak Foun	Peak Foun	High	High	High	Peak Foun	High	1
High	Peak Foun	High	Peak Foun	Peak Foun	Peak Foun	Peak Foun	Peak Foun	1
Peak Foun	High	High	Peak Foun	Peak Foun	High	Peak Foun	Peak Foun	1
Peak Foun	High	Peak Foun	High	Peak Foun	High	Peak Foun	Not Found	1
Peak Foun	Peak Foun	Not Found	Peak Foun	Peak Foun	High	Peak Foun	Peak Foun	1
Peak Foun	Peak Foun	High	Peak Foun	Peak Foun	High	High	Peak Foun	1
Not Found	Not Found	Not Found	Not Found	Not Found	Not Found	Not Found	Not Found	1
Peak Foun	Peak Foun	Peak Foun	Peak Foun	Peak Foun	Peak Foun	Peak Foun	Peak Foun	1
Not Found	Not Found	Not Found	Peak Foun	Peak Foun	Peak Foun	Peak Foun	Peak Foun	1
Peak Foun	Peak Foun	Peak Foun	High	High	High	Peak Foun	Peak Foun	1
Peak Foun	Not Found	Peak Foun	Not Found	High	High	High	Peak Foun	1
Peak Foun	Peak Foun	High	High	High	High	High	Peak Foun	1
High	Peak Foun	Peak Foun	Peak Foun	Peak Foun	Peak Foun	Peak Foun	Peak Foun	1
High	High	Peak Foun	High	High	High	High	High	1
Peak Foun	High	High	Not Found	Peak Foun	High	Peak Foun	Peak Foun	1
Not Found	Not Found	High	Not Found	Not Found	Peak Foun	Peak Foun	Not Found	1
Peak Foun	Peak Foun	Peak Foun	Peak Foun	Peak Foun	High	Peak Foun	Peak Foun	1

Not Found	Peak Foun	Peak Foun	Peak Foun	Peak Foun	Peak Foun	Peak Foun	Peak Foun	1
Peak Foun	Not Found	Peak Foun	Peak Foun	Peak Foun	High	Peak Foun	Peak Foun	1
Not Found	Peak Foun	High	Peak Foun	Not Found	High	High	Peak Foun	1
Peak Foun	Peak Foun	High	Peak Foun	High	High	High	Peak Foun	1
Not Found	Not Found	Not Found	Peak Foun	Peak Foun	Peak Foun	Peak Foun	Not Found	1
Peak Foun	Not Found	Peak Foun	Not Found	High	Peak Foun	Peak Foun	Peak Foun	1
Not Found	Peak Foun	Peak Foun	Peak Foun	Peak Foun	Peak Foun	High	Peak Foun	1
Not Found	Not Found	Not Found	Not Found	Peak Foun	High	Peak Foun	Peak Foun	1
High	Peak Foun	Peak Foun	Peak Foun	High	Peak Foun	Peak Foun	Peak Foun	1
Peak Foun	Not Found	Not Found	Peak Foun	High	High	Peak Foun	Peak Foun	1
Peak Foun	Peak Foun	Not Found	Peak Foun	Not Found	Not Found	Not Found	Not Found	1
High	High	High	High	Peak Foun	High	High	Peak Foun	1
Peak Foun	Peak Foun	Peak Foun	Not Found	Peak Foun	Peak Foun	Peak Foun	Peak Foun	1
Not Found	Not Found	Not Found	Not Found	Peak Foun	High	High	High	1
Not Found	Peak Foun	Peak Foun	Peak Foun	High	High	Not Found	Not Found	1
Peak Foun	High	Peak Foun	Not Found	High	High	Not Found	Not Found	1
Not Found	Peak Foun	Peak Foun	Peak Foun	Peak Foun	High	High	Peak Foun	1
Not Found	Peak Foun	Peak Foun	Peak Foun	Peak Foun	High	Peak Foun	Peak Foun	1
Peak Foun	High	High	High	High	High	Peak Foun	High	1
Peak Foun	High	High	High	High	High	Peak Foun	High	1
Not Found	Peak Foun	Peak Foun	Peak Foun	High	High	High	Peak Foun	1
High	Peak Foun	Peak Foun	Peak Foun	High	High	High	Peak Foun	1
Peak Foun	Peak Foun	High	Peak Foun	Peak Foun	High	High	Peak Foun	1
Not Found	Peak Foun	Peak Foun	Peak Foun	Peak Foun	Peak Foun	Peak Foun	Peak Foun	1
High	Peak Foun	Peak Foun	Peak Foun	High	Peak Foun	Peak Foun	Peak Foun	1
Peak Foun	High	High	Peak Foun	Not Found	High	High	Peak Foun	1
Peak Foun	Peak Foun	Peak Foun	Not Found	Peak Foun	Not Found	High	Not Found	1
Peak Foun	Peak Foun	High	Peak Foun	Not Found	High	Peak Foun	Not Found	1
Peak Foun	Peak Foun	High	High	Peak Foun	High	High	Peak Foun	1
Peak Foun	Peak Foun	Not Found	Peak Foun	Peak Foun	High	High	Peak Foun	1
Peak Foun	Peak Foun	Peak Foun	Peak Foun	Peak Foun	High	High	High	1
Peak Foun	Peak Foun	High	Peak Foun	Peak Foun	High	High	Peak Foun	1
Peak Foun	Peak Foun	High	Peak Foun	Peak Foun	High	Peak Foun	Peak Foun	1
Not Found	Not Found	Not Found	Peak Foun	Peak Foun	High	High	Not Found	1
Not Found	Not Found	Not Found	Peak Foun	Peak Foun	High	Peak Foun	Peak Foun	1
Peak Foun	Peak Foun	Peak Foun	High	Peak Foun	High	Peak Foun	Peak Foun	1
Not Found	Not Found	Not Found	Peak Foun	Not Found	Not Found	Not Found	Peak Foun	1
High	High	Peak Foun	High	High	High	Peak Foun	Peak Foun	1
Not Found	Not Found	Not Found	Not Found	Peak Foun	High	Peak Foun	Not Found	1
Peak Foun	Not Found	Peak Foun	Peak Foun	Not Found	Peak Foun	Peak Foun	Peak Foun	1
Not Found	Peak Foun	Peak Foun	Not Found	Peak Foun	Peak Foun	Peak Foun	High	1
Peak Foun	Peak Foun	Not Found	Peak Foun	Peak Foun	High	Peak Foun	Peak Foun	1
Not Found	Not Found	Not Found	Not Found	Not Found	Peak Foun	Peak Foun	Peak Foun	1
High	High	Peak Foun	High	Not Found	High	Peak Foun	Not Found	1
Not Found	High	Peak Foun	Not Found	Peak Foun	High	Peak Foun	Peak Foun	1

Peak Foun	High	High	High	Peak Foun	High	High	High	1
Peak Foun	High	Peak Foun	Peak Foun	Peak Foun	Peak Foun	Peak Foun	Peak Foun	1
High	High	High	Peak Foun	High	Peak Foun	Peak Foun	Peak Foun	1
Not Found	Peak Foun	Peak Foun	Peak Foun	Not Found	Not Found	Not Found	Not Found	1
Peak Foun	Peak Foun	Peak Foun	Peak Foun	Peak Foun	High	Peak Foun	Peak Foun	1
High	Peak Foun	Peak Foun	Not Found	Not Found	Not Found	Not Found	Not Found	1
Not Found	Not Found	Not Found	Peak Foun	Not Found	High	High	Peak Foun	1
Peak Foun	Not Found	Peak Foun	Peak Foun	Peak Foun	Peak Foun	High	Not Found	1
Peak Foun	Peak Foun	High	Peak Foun	Peak Foun	High	High	Peak Foun	1
Not Found	Not Found	Not Found	Peak Foun	High	High	Peak Foun	Peak Foun	1
Peak Foun	Peak Foun	Peak Foun	Peak Foun	Peak Foun	High	Peak Foun	Peak Foun	1
Peak Foun	Peak Foun	High	Peak Foun	Peak Foun	High	Peak Foun	Not Found	1
Peak Foun	Peak Foun	Peak Foun	Not Found	Peak Foun	High	High	Peak Foun	1
High	Peak Foun	High	Peak Foun	Peak Foun	Peak Foun	High	Peak Foun	1
Not Found	Not Found	High	Not Found	Peak Foun	Peak Foun	Peak Foun	Peak Foun	1
Peak Foun	Peak Foun	Peak Foun	High	Peak Foun	High	Peak Foun	Peak Foun	1
Peak Foun	Peak Foun	Peak Foun	Not Found	Peak Foun	Peak Foun	High	Peak Foun	1
Peak Foun	High	Peak Foun	Peak Foun	High	High	High	High	1
Peak Foun	Not Found	Peak Foun	Peak Foun	Peak Foun	High	High	High	1
Not Found	Not Found	Not Found	High	High	High	High	Peak Foun	1
Not Found	Not Found	High	Not Found	Not Found	Not Found	High	Not Found	1
Peak Foun	Peak Foun	Not Found	Peak Foun	High	Peak Foun	Peak Foun	Peak Foun	1
High	Peak Foun	Peak Foun	Peak Foun	High	Peak Foun	Peak Foun	Peak Foun	1
Not Found	High	High	Peak Foun	Peak Foun	High	High	Peak Foun	1
Peak Foun	Peak Foun	Peak Foun	Peak Foun	High	High	Peak Foun	Peak Foun	1
Peak Foun	Not Found	Not Found	Peak Foun	Peak Foun	Peak Foun	Peak Foun	Peak Foun	1
Not Found	Not Found	Not Found	Peak Foun	Peak Foun	Peak Foun	Peak Foun	Peak Foun	1
Not Found	Not Found	Not Found	Peak Foun	Peak Foun	High	Peak Foun	Not Found	1
Not Found	Peak Foun	High	Peak Foun	Peak Foun	Peak Foun	High	Peak Foun	1
Peak Foun	High	High	Peak Foun	Peak Foun	Peak Foun	Peak Foun	Peak Foun	1
Peak Foun	High	Peak Foun	High	High	High	High	High	1
Peak Foun	Peak Foun	Peak Foun	Peak Foun	Peak Foun	Peak Foun	High	Not Found	1
Peak Foun	Peak Foun	Peak Foun	Not Found	Peak Foun	Peak Foun	Peak Foun	Not Found	1
Not Found	Not Found	Not Found	Not Found	High	High	Peak Foun	Not Found	1
Peak Foun	Peak Foun	Peak Foun	Peak Foun	High	Peak Foun	Peak Foun	Peak Foun	1
Peak Foun	Peak Foun	Peak Foun	Peak Foun	High	High	Peak Foun	Peak Foun	1
High	High	Not Found	High	High	High	High	Peak Foun	1
Not Found	Not Found	Not Found	Peak Foun	Peak Foun	High	High	Peak Foun	1
Peak Foun	High	High	Peak Foun	High	High	High	High	1
Not Found	Not Found	Peak Foun	Peak Foun	Peak Foun	Peak Foun	Peak Foun	Peak Foun	1
Not Found	Not Found	Not Found	Peak Foun	Peak Foun	High	Peak Foun	Peak Foun	1
Peak Foun	Peak Foun	Peak Foun	Peak Foun	Peak Foun	High	Peak Foun	Peak Foun	1
Not Found	Not Found	Not Found	Peak Foun	Not Found	High	Peak Foun	Not Found	1
Peak Foun	Peak Foun	Peak Foun	Peak Foun	Peak Foun	Peak Foun	Peak Foun	Peak Foun	1
Peak Foun	Peak Foun	Peak Foun	Peak Foun	Peak Foun	High	Peak Foun	Peak Foun	1

Peak Foun	Not Found	Not Found	Peak Foun	Peak Foun	Peak Foun	Peak Foun	Not Found	1
Peak Foun	Peak Foun	Peak Foun	Peak Foun	Peak Foun	Peak Foun	Peak Foun	Peak Foun	1
Peak Foun	High	Peak Foun	Peak Foun	Peak Foun	High	High	High	1
Peak Foun	Peak Foun	Peak Foun	Peak Foun	Peak Foun	High	High	Not Found	1
Peak Foun	High	High	Peak Foun	Peak Foun	High	High	Peak Foun	1
Not Found	Not Found	Peak Foun	Not Found	Peak Foun	High	High	Not Found	1
Peak Foun	Peak Foun	Peak Foun	Peak Foun	Peak Foun	High	High	Peak Foun	1
Not Found	Not Found	Not Found	Peak Foun	Peak Foun	High	Peak Foun	Peak Foun	1
Peak Foun	Peak Foun	Peak Foun	Peak Foun	Peak Foun	Peak Foun	Peak Foun	Peak Foun	1
High	Peak Foun	High	Peak Foun	Peak Foun	Peak Foun	High	Peak Foun	1
Peak Foun	High	Peak Foun	Peak Foun	Peak Foun	High	High	Peak Foun	1
Not Found	Not Found	Peak Foun	Not Found	Peak Foun	Peak Foun	High	Not Found	1
Not Found	Not Found	Not Found	Peak Foun	Peak Foun	High	Not Found	Peak Foun	1
Peak Foun	Peak Foun	Peak Foun	Peak Foun	Peak Foun	Peak Foun	Peak Foun	Peak Foun	1
Peak Foun	Peak Foun	Peak Foun	Peak Foun	Peak Foun	Peak Foun	High	Peak Foun	1
Not Found	Peak Foun	High	Peak Foun	Not Found	Not Found	Peak Foun	Peak Foun	1
Peak Foun	Peak Foun	Peak Foun	High	High	High	High	High	1
Not Found	Peak Foun	Peak Foun	Peak Foun	Peak Foun	Peak Foun	Peak Foun	Peak Foun	1
Not Found	Peak Foun	Peak Foun	Not Found	Peak Foun	Not Found	High	Peak Foun	1
Peak Foun	Peak Foun	Peak Foun	Peak Foun	Peak Foun	Peak Foun	Peak Foun	Peak Foun	1
High	Peak Foun	Not Found	Not Found	Not Found	Not Found	Not Found	Not Found	1
Peak Foun	Peak Foun	Peak Foun	Peak Foun	Peak Foun	High	Peak Foun	Peak Foun	1
Peak Foun	Peak Foun	Peak Foun	Peak Foun	Not Found	Not Found	Not Found	Not Found	1
Not Found	Not Found	Not Found	Not Found	Peak Foun	Peak Foun	High	Not Found	1
Not Found	Peak Foun	High	Peak Foun	Peak Foun	High	High	Peak Foun	1
Not Found	Peak Foun	Not Found	Peak Foun	Peak Foun	High	Peak Foun	Peak Foun	1
High	High	High	Not Found	Peak Foun	Peak Foun	Peak Foun	Not Found	1
Peak Foun	Not Found	Not Found	Peak Foun	Peak Foun	Peak Foun	Peak Foun	Not Found	1
Not Found	Peak Foun	Not Found	Peak Foun	Peak Foun	Not Found	Not Found	Peak Foun	1
Not Found	Not Found	Not Found	High	Not Found	Not Found	Not Found	Not Found	1
Peak Foun	High	Peak Foun	Peak Foun	Peak Foun	Peak Foun	High	Peak Foun	1
Peak Foun	Peak Foun	Peak Foun	Peak Foun	High	Peak Foun	Peak Foun	Peak Foun	1
Peak Foun	Not Found	Peak Foun	Not Found	Peak Foun	Peak Foun	Peak Foun	Peak Foun	1
Peak Foun	Peak Foun	Peak Foun	Peak Foun	Peak Foun	Peak Foun	Peak Foun	Peak Foun	1
Peak Foun	Peak Foun	High	Peak Foun	Peak Foun	Not Found	Peak Foun	Peak Foun	1
Peak Foun	High	Peak Foun	Peak Foun	Peak Foun	High	Peak Foun	Peak Foun	1
Not Found	Peak Foun	High	Not Found	Not Found	Peak Foun	High	Peak Foun	1
Not Found	Peak Foun	Peak Foun	Peak Foun	Peak Foun	High	Peak Foun	Peak Foun	1
High	High	Peak Foun	High	Peak Foun	High	Peak Foun	Peak Foun	1
Peak Foun	Peak Foun	Peak Foun	Peak Foun	Peak Foun	Peak Foun	Peak Foun	Peak Foun	1
High	High	High	High	High	High	High	Peak Foun	1
Peak Foun	Not Found	Not Found	High	High	High	High	Peak Foun	1
Peak Foun	Not Found	Peak Foun	Peak Foun	Peak Foun	Peak Foun	Peak Foun	Peak Foun	1
High	Peak Foun	High	High	Peak Foun	Peak Foun	Peak Foun	Peak Foun	1
Not Found	Not Found	Not Found	Not Found	Peak Foun	High	Peak Foun	Peak Foun	1

Peak Foun	Peak Foun	Peak Foun	High	Peak Foun	Peak Foun	High	High	1
Peak Foun	Peak Foun	Peak Foun	Peak Foun	Peak Foun	High	High	Peak Foun	1
Peak Foun	Peak Foun	Peak Foun	Not Found	Not Found	Not Found	Not Found	Not Found	1
Not Found	Peak Foun	High	Peak Foun	Peak Foun	Peak Foun	Peak Foun	Peak Foun	1
High	High	Peak Foun	Peak Foun	Peak Foun	Peak Foun	Not Found	Not Found	1
High	High	High	High	Peak Foun	Peak Foun	High	High	1
Peak Foun	Peak Foun	Peak Foun	Peak Foun	Peak Foun	Peak Foun	Peak Foun	Peak Foun	1
Not Found	Not Found	Peak Foun	Not Found	Peak Foun	Not Found	Not Found	Not Found	1
Peak Foun	High	High	Peak Foun	High	Peak Foun	High	Peak Foun	1
Not Found	Not Found	High	Peak Foun	Peak Foun	High	High	High	1
Peak Foun	Peak Foun	High	Peak Foun	Peak Foun	Peak Foun	Peak Foun	Peak Foun	1
Not Found	Peak Foun	Peak Foun	Peak Foun	High	High	Peak Foun	Peak Foun	1
Peak Foun	Peak Foun	Peak Foun	Peak Foun	Peak Foun	Peak Foun	High	Peak Foun	1
Peak Foun	Peak Foun	High	High	Peak Foun	Peak Foun	High	High	1
Not Found	Peak Foun	Peak Foun	Not Found	Peak Foun	High	Peak Foun	Peak Foun	1
Peak Foun	Peak Foun	Peak Foun	Peak Foun	High	Peak Foun	High	Peak Foun	1
Peak Foun	High	Peak Foun	Not Found	Peak Foun	Peak Foun	Peak Foun	Not Found	1
Peak Foun	High	High	Peak Foun	Peak Foun	High	Peak Foun	Peak Foun	1
Peak Foun	Peak Foun	Peak Foun	Peak Foun	Peak Foun	Peak Foun	Peak Foun	Peak Foun	1
Peak Foun	High	High	High	Peak Foun	High	Peak Foun	Peak Foun	1
Peak Foun	Not Found	Peak Foun	Peak Foun	Peak Foun	High	Peak Foun	Peak Foun	1
Peak Foun	High	Peak Foun	Peak Foun	High	High	Peak Foun	Peak Foun	1
High	High	High	High	High	High	High	High	1
Peak Foun	Peak Foun	High	Peak Foun	High	Peak Foun	Peak Foun	Peak Foun	1
Not Found	Peak Foun	Not Found	Peak Foun	Peak Foun	High	Not Found	Not Found	1
High	Not Found	Not Found	Peak Foun	Not Found	Peak Foun	Peak Foun	Not Found	1
Not Found	Peak Foun	High	Peak Foun	Peak Foun	Peak Foun	Peak Foun	Peak Foun	1
Peak Foun	Peak Foun	Peak Foun	Peak Foun	Peak Foun	Peak Foun	Peak Foun	Peak Foun	1
Peak Foun	Peak Foun	Peak Foun	Peak Foun	High	High	Peak Foun	Peak Foun	1
Not Found	Not Found	Not Found	Peak Foun	Peak Foun	High	Peak Foun	Not Found	1
Peak Foun	Peak Foun	Peak Foun	Not Found	Peak Foun	Peak Foun	High	Peak Foun	1
Peak Foun	Peak Foun	Peak Foun	Peak Foun	Not Found	Peak Foun	High	Peak Foun	1
Not Found	Peak Foun	Peak Foun	Peak Foun	Peak Foun	Peak Foun	Peak Foun	Peak Foun	1
Peak Foun	Peak Foun	Peak Foun	Peak Foun	Peak Foun	Peak Foun	Peak Foun	Not Found	1
Peak Foun	Peak Foun	Peak Foun	Not Found	Peak Foun	Peak Foun	High	Peak Foun	1
Peak Foun	High	High	High	Peak Foun	Peak Foun	Peak Foun	Peak Foun	1
Not Found	Peak Foun	Peak Foun	Peak Foun	Peak Foun	Peak Foun	Peak Foun	Peak Foun	1
Peak Foun	Peak Foun	Peak Foun	Peak Foun	Not Found	Peak Foun	Peak Foun	Not Found	1
Not Found	Not Found	Not Found	Peak Foun	Peak Foun	High	Peak Foun	High	1
Peak Foun	Peak Foun	Peak Foun	Peak Foun	Peak Foun	Peak Foun	Peak Foun	Peak Foun	1
Not Found	Peak Foun	Peak Foun	Not Found	Not Found	Peak Foun	High	Not Found	1
Not Found	High	High	High	Peak Foun	High	Peak Foun	High	1
Peak Foun	Peak Foun	Peak Foun	Peak Foun	Peak Foun	High	High	Peak Foun	1
Not Found	Not Found	Not Found	Not Found	Not Found	Not Found	Peak Foun	Not Found	1
High	Peak Foun	Peak Foun	Peak Foun	Peak Foun	Not Found	Peak Foun	Peak Foun	1

Peak Foun	Peak Foun	Peak Foun	Not Found	Peak Foun	Peak Foun	Peak Foun	Peak Foun		1
High	High	High	High	Not Found	High	High	High		1
Peak Foun	High	Peak Foun	Not Found	Peak Foun	Peak Foun	Peak Foun	Not Found		1
Not Found	Not Found	Peak Foun	Peak Foun	Peak Foun	High	Peak Foun	Peak Foun		1
High	Peak Foun	High	High	Peak Foun	High	Peak Foun	Peak Foun		1
Peak Foun	Peak Foun	Peak Foun	Peak Foun	Peak Foun	Peak Foun	Peak Foun	Not Found		1
High	Peak Foun	Peak Foun	Not Found	Not Found	Not Found	Not Found	Not Found		1
Peak Foun	Peak Foun	High	Peak Foun	Peak Foun	High	Peak Foun	High		1
Peak Foun	Peak Foun	Peak Foun	Peak Foun	Peak Foun	High	Peak Foun	Peak Foun		1
Peak Foun	Peak Foun	Not Found	Peak Foun	Peak Foun	Peak Foun	High	Peak Foun		1
Peak Foun	High	Peak Foun	Not Found	Not Found	Not Found	Not Found	Not Found		1
High	High	Peak Foun	High	Not Found	Peak Foun	Peak Foun	Peak Foun		1
Peak Foun	Peak Foun	Peak Foun	Peak Foun	High	High	Peak Foun	High		1
Peak Foun	Peak Foun	Not Found	Peak Foun	Peak Foun	Peak Foun	Peak Foun	Peak Foun		1
Not Found	Peak Foun	Not Found	Not Found	Peak Foun	Not Found	Peak Foun	Peak Foun		1
Not Found	Peak Foun	High	Not Found	Not Found	Peak Foun	Peak Foun	Not Found		1
Peak Foun	Not Found	Not Found	High	High	High	High	Peak Foun		1
Not Found	Not Found	Not Found	Not Found	Peak Foun	High	Peak Foun	Not Found		1
Not Found	Peak Foun	Not Found	Not Found	Not Found	Peak Foun	Not Found	Not Found		1
Peak Foun	Peak Foun	High	Not Found	Not Found	Not Found	Peak Foun	Peak Foun		1
Not Found	Not Found	Peak Foun	Peak Foun	Peak Foun	High	High	Peak Foun		1
Peak Foun	Not Found	Not Found	Not Found	Not Found	Peak Foun	Not Found	Not Found		1
Peak Foun	Peak Foun	Peak Foun	Peak Foun	Peak Foun	Peak Foun	Peak Foun	Peak Foun		1
High	Peak Foun	High	Not Found	Not Found	Not Found	Not Found	Not Found		1
Peak Foun	Peak Foun	Peak Foun	Peak Foun	Peak Foun	Peak Foun	Peak Foun	Peak Foun		1
Peak Foun	Peak Foun	Peak Foun	High	Peak Foun	Peak Foun	Peak Foun	Peak Foun		1
High	Peak Foun	Peak Foun	Peak Foun	High	Peak Foun	Peak Foun	High		1
Not Found	Not Found	Not Found	Peak Foun	Peak Foun	High	Peak Foun	Peak Foun		1
Peak Foun	Peak Foun	Peak Foun	Peak Foun	Peak Foun	Peak Foun	High	Peak Foun		1
Peak Foun	Peak Foun	Peak Foun	Peak Foun	High	Peak Foun	Not Found	Not Found		1
High	High	Peak Foun	High	High	High	High	High		1
Peak Foun	High	High	High	High	Peak Foun	Peak Foun	Peak Foun		1
Not Found	Peak Foun	Peak Foun	Peak Foun	Peak Foun	Peak Foun	Peak Foun	Peak Foun		1
Not Found	Peak Foun	Peak Foun	Peak Foun	Not Found	Peak Foun	High	Not Found		1
Not Found	Not Found	Not Found	Peak Foun	Not Found	High	High	Not Found		1
Not Found	Not Found	Peak Foun	Not Found	Not Found	Not Found	Not Found	Not Found		1
Not Found	Not Found	Peak Foun	Peak Foun	Peak Foun	High	High	High		1
Peak Foun	Peak Foun	Peak Foun	High	Peak Foun	High	Peak Foun	Peak Foun		1
Peak Foun	Peak Foun	Peak Foun	Peak Foun	Peak Foun	Peak Foun	Not Found	Peak Foun		1
Peak Foun	Peak Foun	Peak Foun	Peak Foun	Peak Foun	Peak Foun	High	Peak Foun		1
Not Found	Not Found	Not Found	Not Found	Not Found	Not Found	Not Found	Not Found		1
Peak Foun	Peak Foun	High	Peak Foun	High	Peak Foun	Peak Foun	Peak Foun		1
Peak Foun	Peak Foun	Not Found	Not Found	Not Found	High	Peak Foun	Not Found		1
Peak Foun	Not Found	Not Found	Not Found	Peak Foun	High	Not Found	Not Found		1
Not Found	Peak Foun	Peak Foun	Peak Foun	Peak Foun	Peak Foun	Peak Foun	Not Found		1

Peak Foun	Peak Foun	Peak Foun	Peak Foun	Peak Foun	Peak Foun	Peak Foun	Not Found		1
Peak Foun	Peak Foun	Peak Foun	Peak Foun	Peak Foun	Peak Foun	Peak Foun	Peak Foun		1
Not Found	Peak Foun	Peak Foun	Peak Foun	Peak Foun	Peak Foun	Peak Foun	Peak Foun		1
Peak Foun	Peak Foun	High	Peak Foun	Peak Foun	Peak Foun	Peak Foun	Peak Foun		1
Not Found	Not Found	Not Found	Peak Foun	Peak Foun	High	Peak Foun	Not Found		1
Peak Foun	Peak Foun	Peak Foun	Peak Foun	Peak Foun	Peak Foun	Peak Foun	Peak Foun		1
Not Found	Not Found	High	Peak Foun	Not Found	Peak Foun	High	Not Found		1
Not Found	Not Found	Not Found	Not Found	Not Found	Not Found	Not Found	Not Found		1
High	Peak Foun	Peak Foun	Peak Foun	High	Peak Foun	High	Peak Foun		1
Peak Foun	Not Found	Peak Foun	Peak Foun	High	High	Peak Foun	Peak Foun		1
Peak Foun	Not Found	Peak Foun	Peak Foun	Peak Foun	Peak Foun	High	Peak Foun		1
Not Found	Not Found	Not Found	Not Found	Not Found	High	Not Found	Not Found		1
Peak Foun	High	Peak Foun	High	Peak Foun	High	Peak Foun	Peak Foun		1
Peak Foun	Peak Foun	Peak Foun	Peak Foun	Peak Foun	Peak Foun	Peak Foun	Peak Foun		1
Peak Foun	High	Peak Foun	Peak Foun	High	High	High	Peak Foun		1
Peak Foun	Peak Foun	High	High	High	High	High	High		1
Peak Foun	Not Found	Peak Foun	Peak Foun	Peak Foun	High	Peak Foun	Peak Foun		1
Peak Foun	High	Peak Foun	Not Found	Not Found	Peak Foun	Not Found	Not Found		1
Peak Foun	Peak Foun	Peak Foun	Peak Foun	High	Peak Foun	High	Peak Foun		1
Not Found	Not Found	Not Found	Not Found	High	Not Found	Not Found	Not Found		1
Peak Foun	Peak Foun	Peak Foun	Peak Foun	Peak Foun	Peak Foun	Peak Foun	Peak Foun		1
Not Found	Peak Foun	Peak Foun	Not Found	Peak Foun	Peak Foun	High	Peak Foun		1
Peak Foun	High	High	Not Found	Peak Foun	Peak Foun	High	Not Found		1
Not Found	Not Found	Peak Foun	High	High	Peak Foun	Peak Foun	Peak Foun		1
Peak Foun	Peak Foun	Peak Foun	Not Found	Peak Foun	High	Peak Foun	Peak Foun		1
Peak Foun	Peak Foun	Peak Foun	Peak Foun	Peak Foun	Peak Foun	Peak Foun	Peak Foun		1
Peak Foun	Peak Foun	Peak Foun	Peak Foun	High	High	High	Peak Foun		1
Not Found	Not Found	Not Found	Not Found	Peak Foun	Peak Foun	Not Found	Peak Foun		1
Peak Foun	Peak Foun	Peak Foun	Peak Foun	Peak Foun	Peak Foun	Peak Foun	Peak Foun		1
Peak Foun	Peak Foun	Peak Foun	Peak Foun	Peak Foun	Peak Foun	Peak Foun	Peak Foun		1
Peak Foun	Not Found	Not Found	Peak Foun	High	High	Peak Foun	Peak Foun		1
Peak Foun	Peak Foun	High	Peak Foun	Peak Foun	Peak Foun	Peak Foun	Peak Foun		1
Peak Foun	Not Found	Peak Foun	Peak Foun	Not Found	Not Found	Peak Foun	Peak Foun		1
Not Found	Peak Foun	Peak Foun	Peak Foun	Peak Foun	Peak Foun	Peak Foun	High		1
Peak Foun	Peak Foun	Peak Foun	Peak Foun	Peak Foun	High	Peak Foun	Peak Foun		1
Not Found	Peak Foun	Not Found	Not Found	Not Found	Not Found	Peak Foun	Not Found		1
Not Found	Not Found	Not Found	Peak Foun	Not Found	Peak Foun	High	Peak Foun		1
Peak Foun	Peak Foun	Peak Foun	High	High	High	Peak Foun	Peak Foun		1
Not Found	Peak Foun	Not Found	Peak Foun	Peak Foun	High	Peak Foun	Peak Foun		1
Peak Foun	High	Peak Foun	High	High	High	Peak Foun	Not Found		1
Peak Foun	Peak Foun	Not Found	Peak Foun	Peak Foun	High	High	Peak Foun		1
Peak Foun	Peak Foun	Peak Foun	High	Peak Foun	High	High	Peak Foun		1
Not Found	Peak Foun	Not Found	Not Found	Peak Foun	Peak Foun	Peak Foun	Not Found		1
Not Found	Not Found	Not Found	Not Found	Not Found	Not Found	Not Found	Not Found		1
Peak Foun	Peak Foun	Peak Foun	Peak Foun	Peak Foun	Not Found	High	Peak Foun		1

Peak Foun	Not Found	Peak Foun	Peak Foun	Peak Foun	High	Peak Foun	Peak Foun	1
Peak Foun	Peak Foun	Not Found	Peak Foun	High	Peak Foun	Peak Foun	Peak Foun	1
Not Found	Not Found	Not Found	Not Found	Not Found	Peak Foun	Peak Foun	Not Found	1
Peak Foun	Peak Foun	Peak Foun	Peak Foun	High	High	High	High	1
Peak Foun	Peak Foun	Peak Foun	Peak Foun	Peak Foun	High	Peak Foun	Peak Foun	1
Peak Foun	Peak Foun	Peak Foun	High	High	High	High	High	1
Not Found	Peak Foun	Not Found	Not Found	Peak Foun	High	Peak Foun	Not Found	1
Peak Foun	Not Found	High	Not Found	Not Found	Peak Foun	Peak Foun	Not Found	1
Not Found	Not Found	Peak Foun	Peak Foun	Peak Foun	Peak Foun	Not Found	Not Found	1
High	High	High	Peak Foun	High	High	High	High	1
Not Found	Not Found	Not Found	Peak Foun	Peak Foun	Peak Foun	Peak Foun	Peak Foun	1
Peak Foun	Not Found	Not Found	High	Peak Foun	High	Peak Foun	High	1
Not Found	Not Found	Not Found	Peak Foun	Peak Foun	High	Peak Foun	Not Found	1
Not Found	Peak Foun	Not Found	Peak Foun	Peak Foun	Peak Foun	High	Not Found	1
Peak Foun	Peak Foun	Peak Foun	High	High	Peak Foun	Peak Foun	Peak Foun	1
High	Peak Foun	Peak Foun	Peak Foun	Peak Foun	High	High	Peak Foun	1
Not Found	Peak Foun	Peak Foun	Peak Foun	Peak Foun	High	Peak Foun	Not Found	1
Peak Foun	Not Found	Peak Foun	Peak Foun	Peak Foun	High	Peak Foun	Peak Foun	1
Peak Foun	Peak Foun	Peak Foun	Peak Foun	High	High	Peak Foun	Peak Foun	1
High	High	Peak Foun	Peak Foun	Peak Foun	High	High	Peak Foun	1
Not Found	Not Found	Not Found	Not Found	High	High	High	Not Found	1
Peak Foun	Peak Foun	Peak Foun	High	Peak Foun	Peak Foun	Peak Foun	Peak Foun	1
Peak Foun	Peak Foun	Peak Foun	Peak Foun	High	Peak Foun	Peak Foun	Peak Foun	1
Not Found	Peak Foun	Peak Foun	Peak Foun	Peak Foun	Peak Foun	Peak Foun	Peak Foun	1
Not Found	Not Found	Peak Foun	Not Found	Peak Foun	Peak Foun	Peak Foun	Peak Foun	1
High	Peak Foun	Peak Foun	Peak Foun	Peak Foun	Not Found	Not Found	Not Found	1
Peak Foun	High	Peak Foun	Peak Foun	Peak Foun	Peak Foun	Peak Foun	Not Found	1
Peak Foun	Peak Foun	Not Found	Peak Foun	Peak Foun	Peak Foun	Not Found	Peak Foun	1
Peak Foun	Peak Foun	Peak Foun	Peak Foun	High	Peak Foun	High	Peak Foun	1
Peak Foun	High	Peak Foun	Peak Foun	Peak Foun	Peak Foun	Peak Foun	Peak Foun	1
Peak Foun	Peak Foun	Not Found	Not Found	Peak Foun	High	Peak Foun	Not Found	1
Not Found	Not Found	Not Found	Not Found	Not Found	Not Found	Not Found	Not Found	1
Not Found	Not Found	Peak Foun	Peak Foun	Peak Foun	Peak Foun	Peak Foun	Not Found	1
Peak Foun	High	High	Peak Foun	Peak Foun	High	High	Peak Foun	1
Peak Foun	Peak Foun	Peak Foun	Peak Foun	High	High	High	Peak Foun	1
Peak Foun	Peak Foun	Not Found	Peak Foun	Peak Foun	Peak Foun	Peak Foun	Not Found	1
Peak Foun	Peak Foun	Peak Foun	Peak Foun	Peak Foun	Peak Foun	Peak Foun	Not Found	1
Peak Foun	Peak Foun	Peak Foun	Peak Foun	Peak Foun	High	Peak Foun	Peak Foun	1
Peak Foun	Peak Foun	Peak Foun	Peak Foun	Peak Foun	High	High	Peak Foun	1
Not Found	Not Found	Not Found	Peak Foun	Peak Foun	Peak Foun	High	Peak Foun	1
Peak Foun	Not Found	Not Found	Not Found	Not Found	High	Not Found	Peak Foun	1
Peak Foun	Peak Foun	Peak Foun	High	Peak Foun	Peak Foun	Peak Foun	Peak Foun	1
High	Peak Foun	Peak Foun	Peak Foun	Peak Foun	High	Peak Foun	Peak Foun	1
Peak Foun	Peak Foun	Peak Foun	Peak Foun	Peak Foun	High	Peak Foun	Not Found	1
Not Found	Not Found	Not Found	Peak Foun	Peak Foun	Peak Foun	Peak Foun	Peak Foun	1

Peak Foun	Peak Foun	Peak Foun	Peak Foun	Peak Foun	Peak Foun	Peak Foun	Peak Foun	1
Peak Foun	Peak Foun	Peak Foun	Peak Foun	Peak Foun	High	Peak Foun	Peak Foun	1
Peak Foun	High	Peak Foun	Peak Foun	Peak Foun	Peak Foun	Not Found	Not Found	1
Not Found	Peak Foun	Peak Foun	Peak Foun	Peak Foun	High	Peak Foun	Peak Foun	1
High	Not Found	Not Found	Peak Foun	Peak Foun	High	Peak Foun	Peak Foun	1
Peak Foun	Peak Foun	Peak Foun	Peak Foun	Peak Foun	High	High	Not Found	1
Peak Foun	Peak Foun	Peak Foun	High	Peak Foun	Peak Foun	Peak Foun	Peak Foun	1
Not Found	Not Found	Not Found	Not Found	Not Found	High	High	Not Found	1
Not Found	Peak Foun	Not Found	Peak Foun	Peak Foun	Peak Foun	Peak Foun	Peak Foun	1
Not Found	High	High	High	Peak Foun	High	High	High	1
Not Found	Not Found	Not Found	Not Found	Peak Foun	High	Not Found	Not Found	1
Peak Foun	Peak Foun	High	Not Found	Not Found	Not Found	Not Found	Not Found	1
Not Found	Not Found	Peak Foun	Peak Foun	Peak Foun	High	High	Peak Foun	1
High	High	High	High	High	High	High	High	1
Peak Foun	Peak Foun	High	Not Found	Not Found	Peak Foun	High	Peak Foun	1
Not Found	Not Found	Peak Foun	Not Found	High	Peak Foun	High	Not Found	1
Not Found	Not Found	Peak Foun	Peak Foun	Peak Foun	High	Peak Foun	Peak Foun	1
Peak Foun	High	High	High	High	High	High	Peak Foun	1
Peak Foun	Peak Foun	Not Found	Not Found	Peak Foun	High	Peak Foun	Not Found	1
Peak Foun	Peak Foun	High	Peak Foun	Peak Foun	Peak Foun	Peak Foun	Peak Foun	1
Peak Foun	Peak Foun	Peak Foun	Peak Foun	Peak Foun	Peak Foun	High	Peak Foun	1
Peak Foun	Peak Foun	Peak Foun	Peak Foun	Peak Foun	Peak Foun	High	Peak Foun	1
High	High	High	High	Peak Foun	High	High	High	1
Not Found	Peak Foun	High	Peak Foun	Not Found	Not Found	Not Found	Peak Foun	1
Not Found	Peak Foun	Not Found	Peak Foun	Peak Foun	Peak Foun	Peak Foun	Peak Foun	1
Not Found	Not Found	Not Found	Not Found	Peak Foun	High	Peak Foun	Not Found	1
Not Found	Not Found	Not Found	Not Found	Not Found	High	Peak Foun	Not Found	1
Peak Foun	High	Not Found	Not Found	Peak Foun	High	Peak Foun	Peak Foun	1
Peak Foun	Peak Foun	Peak Foun	Not Found	Peak Foun	High	Peak Foun	Peak Foun	1
High	High	High	High	High	High	High	High	1
Peak Foun	Not Found	Peak Foun	Not Found	High	Not Found	Not Found	Not Found	1
Peak Foun	Peak Foun	High	Peak Foun	Peak Foun	Peak Foun	High	Peak Foun	1
High	Peak Foun	Peak Foun	Peak Foun	Peak Foun	Peak Foun	Peak Foun	Peak Foun	1
High	Peak Foun	High	Peak Foun	High	Peak Foun	High	Peak Foun	1
High	Not Found	Not Found	Not Found	Not Found	Not Found	High	Not Found	1
High	High	High	High	Peak Foun	High	High	High	1
Peak Foun	Peak Foun	Peak Foun	Peak Foun	Peak Foun	Peak Foun	High	Peak Foun	1
Peak Foun	Peak Foun	Peak Foun	Peak Foun	High	High	High	High	1
Not Found	Not Found	Not Found	Not Found	Not Found	High	Peak Foun	Not Found	1
Peak Foun	High	Peak Foun	Not Found	Peak Foun	Peak Foun	High	Not Found	1
Not Found	Not Found	Not Found	Peak Foun	Not Found	High	High	Not Found	1
Not Found	Not Found	High	Peak Foun	Peak Foun	Peak Foun	Peak Foun	Peak Foun	1
Peak Foun	Peak Foun	Not Found	Not Found	Peak Foun	High	Peak Foun	Not Found	1
Not Found	Not Found	Not Found	Not Found	Not Found	High	Peak Foun	Peak Foun	1
Peak Foun	Not Found	Peak Foun	Not Found	Peak Foun	High	Not Found	Peak Foun	1

Peak Foun	Peak Foun	Peak Foun	Peak Foun	Peak Foun	Peak Foun	Peak Foun	Peak Foun	1
Peak Foun	High	High	Peak Foun	Peak Foun	High	High	Peak Foun	1
Peak Foun	Peak Foun	Peak Foun	Peak Foun	Peak Foun	Not Found	Peak Foun	Not Found	1
High	High	High	Peak Foun	Peak Foun	Peak Foun	Peak Foun	Peak Foun	1
High	High	High	High	High	High	High	High	1
Peak Foun	Peak Foun	Not Found	Not Found	High	High	Peak Foun	Peak Foun	1
Not Found	Not Found	Peak Foun	Not Found	Not Found	Peak Foun	Peak Foun	Not Found	1
Not Found	Not Found	Peak Foun	High	Peak Foun	High	High	Peak Foun	1
Not Found	Peak Foun	Peak Foun	Peak Foun	Peak Foun	Peak Foun	Peak Foun	Peak Foun	1
Not Found	Peak Foun	Peak Foun	Peak Foun	Peak Foun	Peak Foun	Peak Foun	Peak Foun	1
Peak Foun	Peak Foun	High	Peak Foun	Peak Foun	High	Peak Foun	Peak Foun	1
High	Peak Foun	Peak Foun	Peak Foun	Peak Foun	High	Peak Foun	Peak Foun	1
Not Found	Peak Foun	High	Peak Foun	Peak Foun	Peak Foun	High	Peak Foun	1
High	High	Peak Foun	Peak Foun	Peak Foun	Peak Foun	Not Found	Peak Foun	1
High	High	High	Not Found	Not Found	Not Found	Not Found	Not Found	1
Peak Foun	Peak Foun	Peak Foun	Peak Foun	Peak Foun	Not Found	Peak Foun	Peak Foun	1
Peak Foun	Peak Foun	Peak Foun	Peak Foun	Peak Foun	High	Peak Foun	Peak Foun	1
Not Found	Peak Foun	Peak Foun	High	Peak Foun	Peak Foun	High	High	1
Peak Foun	Not Found	Not Found	Peak Foun	High	High	Peak Foun	Peak Foun	1
Peak Foun	Peak Foun	Not Found	Peak Foun	Peak Foun	High	Peak Foun	Peak Foun	1
High	Peak Foun	Peak Foun	Not Found	High	Peak Foun	Peak Foun	Peak Foun	1
Peak Foun	Peak Foun	Not Found	Peak Foun	Peak Foun	Peak Foun	Peak Foun	Not Found	1
Peak Foun	Peak Foun	Peak Foun	Peak Foun	Peak Foun	High	Peak Foun	Peak Foun	1
Not Found	Not Found	Not Found	Not Found	Peak Foun	High	Peak Foun	Not Found	1
Not Found	Not Found	High	Not Found	Not Found	Not Found	Not Found	Not Found	1
Not Found	Not Found	Peak Foun	Not Found	Peak Foun	Peak Foun	High	Peak Foun	1
Not Found	Not Found	Not Found	Not Found	Peak Foun	High	Peak Foun	Peak Foun	1
High	Peak Foun	Peak Foun	Peak Foun	Not Found	Not Found	Peak Foun	Not Found	1
Peak Foun	Peak Foun	Peak Foun	Peak Foun	Peak Foun	Peak Foun	Peak Foun	Peak Foun	1
Peak Foun	Peak Foun	Peak Foun	Peak Foun	Peak Foun	High	Peak Foun	Peak Foun	1
Not Found	Not Found	Peak Foun	Not Found	Peak Foun	Peak Foun	Peak Foun	Peak Foun	1
Not Found	Peak Foun	Not Found	Not Found	High	High	Peak Foun	Not Found	1
Peak Foun	High	Peak Foun	Peak Foun	Peak Foun	Peak Foun	High	Not Found	1
Peak Foun	Peak Foun	Peak Foun	Not Found	High	Not Found	Not Found	Not Found	1
Not Found	Peak Foun	Peak Foun	Peak Foun	Not Found	Peak Foun	Peak Foun	Peak Foun	1
Not Found	Peak Foun	Not Found	Peak Foun	High	Peak Foun	Not Found	Not Found	1
Peak Foun	Peak Foun	Peak Foun	Peak Foun	Peak Foun	High	Peak Foun	Peak Foun	1
Peak Foun	Peak Foun	Peak Foun	Peak Foun	Peak Foun	High	High	Peak Foun	1
Peak Foun	Peak Foun	Peak Foun	Peak Foun	Peak Foun	High	Peak Foun	Peak Foun	1
Peak Foun	Peak Foun	Peak Foun	Not Found	Peak Foun	Peak Foun	Peak Foun	Peak Foun	1
Peak Foun	Peak Foun	Peak Foun	Peak Foun	Peak Foun	High	High	Peak Foun	1
Peak Foun	Peak Foun	Peak Foun	Peak Foun	Peak Foun	Peak Foun	Peak Foun	Peak Foun	1
Not Found	Not Found	Peak Foun	Not Found	Peak Foun	Not Found	High	Peak Foun	1
Peak Foun	Peak Foun	Peak Foun	Peak Foun	High	High	Peak Foun	Not Found	1
Peak Foun	Peak Foun	Peak Foun	Peak Foun	Peak Foun	Peak Foun	Peak Foun	Peak Foun	1

Not Found	Not Found	Not Found	Not Found	Peak Foun	Peak Foun	Not Found	Not Found	1
High	Peak Foun	High	Peak Foun	Peak Foun	High	High	Peak Foun	1
Peak Foun	Peak Foun	Peak Foun	Peak Foun	Peak Foun	Peak Foun	Peak Foun	Peak Foun	1
Peak Foun	Peak Foun	Not Found	Peak Foun	High	Peak Foun	Peak Foun	Peak Foun	1
Peak Foun	Peak Foun	Peak Foun	Peak Foun	Peak Foun	Peak Foun	Peak Foun	Peak Foun	1
Peak Foun	High	Peak Foun	Peak Foun	Peak Foun	Peak Foun	High	Peak Foun	1
Peak Foun	Peak Foun	Peak Foun	High	Peak Foun	Peak Foun	Peak Foun	Peak Foun	1
Peak Foun	Peak Foun	Peak Foun	Peak Foun	Peak Foun	Peak Foun	Peak Foun	Peak Foun	1
Not Found	Peak Foun	Not Found	Peak Foun	Peak Foun	Not Found	Peak Foun	Peak Foun	1
Not Found	Not Found	Not Found	Peak Foun	Not Found	High	High	Peak Foun	1
Not Found	Peak Foun	High	Peak Foun	High	Peak Foun	Peak Foun	Peak Foun	1
Peak Foun	Peak Foun	Peak Foun	Not Found	Peak Foun	High	Peak Foun	Not Found	1
Not Found	Not Found	Not Found	Not Found	Peak Foun	High	Peak Foun	Peak Foun	1
Peak Foun	Peak Foun	Peak Foun	Peak Foun	Peak Foun	Peak Foun	Peak Foun	Not Found	1
Not Found	Not Found	Peak Foun	Peak Foun	Peak Foun	Peak Foun	High	Peak Foun	1
Peak Foun	Peak Foun	Peak Foun	Peak Foun	Peak Foun	High	Peak Foun	Peak Foun	1
Peak Foun	Peak Foun	Not Found	Peak Foun	Peak Foun	Peak Foun	High	Peak Foun	1
Not Found	Not Found	Not Found	Not Found	Not Found	Not Found	Peak Foun	Not Found	1
Peak Foun	Peak Foun	Peak Foun	Peak Foun	Not Found	Peak Foun	Not Found	Not Found	1
Peak Foun	Not Found	High	Not Found	Not Found	Not Found	Not Found	Not Found	1
Peak Foun	Not Found	Not Found	Not Found	Not Found	High	Not Found	Not Found	1
Peak Foun	High	Peak Foun	Peak Foun	Peak Foun	High	High	Peak Foun	1
Peak Foun	Peak Foun	Peak Foun	Peak Foun	Peak Foun	Peak Foun	Not Found	Not Found	1
Not Found	Not Found	Not Found	Peak Foun	Peak Foun	High	Peak Foun	Peak Foun	1
Peak Foun	Peak Foun	Peak Foun	High	Peak Foun	High	High	Not Found	1
Peak Foun	High	Peak Foun	Peak Foun	Peak Foun	Peak Foun	Not Found	Peak Foun	1
Peak Foun	Not Found	Peak Foun	Peak Foun	Peak Foun	Peak Foun	Peak Foun	Peak Foun	1
Peak Foun	Peak Foun	Peak Foun	Peak Foun	Peak Foun	Peak Foun	Peak Foun	Peak Foun	1
Not Found	Peak Foun	Peak Foun	Peak Foun	Peak Foun	Peak Foun	High	Not Found	1
Peak Foun	Peak Foun	Not Found	Peak Foun	High	Peak Foun	Peak Foun	Peak Foun	1
Peak Foun	Peak Foun	Peak Foun	Peak Foun	Peak Foun	Peak Foun	Peak Foun	Peak Foun	1
Not Found	Not Found	Not Found	Peak Foun	High	High	Peak Foun	Peak Foun	1
Not Found	Not Found	Peak Foun	High	Peak Foun	Not Found	Peak Foun	Not Found	1
Not Found	Not Found	Not Found	Not Found	Peak Foun	High	High	Peak Foun	1
Peak Foun	Peak Foun	Peak Foun	Peak Foun	High	High	Peak Foun	Peak Foun	1
Not Found	Peak Foun	Peak Foun	High	High	High	High	High	1
Peak Foun	Peak Foun	Not Found	Peak Foun	Peak Foun	High	Peak Foun	Peak Foun	1
Peak Foun	Not Found	Peak Foun	Peak Foun	Peak Foun	High	High	Peak Foun	1
Not Found	Peak Foun	Not Found	Peak Foun	Peak Foun	High	Peak Foun	Peak Foun	1
Not Found	Not Found	Not Found	Not Found	High	Peak Foun	Not Found	Not Found	1
Peak Foun	Peak Foun	Peak Foun	High	High	High	Peak Foun	High	1
Peak Foun	Peak Foun	High	Peak Foun	Peak Foun	Peak Foun	Not Found	Not Found	1
High	Peak Foun	Not Found	Not Found	Peak Foun	Not Found	Not Found	Not Found	1
High	High	Peak Foun	Peak Foun	High	Peak Foun	Peak Foun	Peak Foun	1
Peak Foun	High	High	High	Peak Foun	Peak Foun	High	High	1

Not Found	Not Found	Not Found	Not Found	Peak Foun	High	Not Found	Not Found	1
Peak Foun	Peak Foun	Peak Foun	High	High	Peak Foun	Peak Foun	Peak Foun	1
Peak Foun	Peak Foun	Peak Foun	Peak Foun	Peak Foun	Peak Foun	Peak Foun	Peak Foun	1
Peak Foun	Not Found	Peak Foun	Not Found	Peak Foun	Peak Foun	Peak Foun	Peak Foun	1
Peak Foun	Peak Foun	Peak Foun	High	Peak Foun	High	High	Peak Foun	1
Peak Foun	Peak Foun	Peak Foun	Peak Foun	Peak Foun	Not Found	Peak Foun	High	1
Peak Foun	Peak Foun	Peak Foun	Peak Foun	High	High	High	Peak Foun	1
Not Found	Peak Foun	Peak Foun	Peak Foun	High	High	High	Peak Foun	1
Peak Foun	Peak Foun	High	High	Peak Foun	Peak Foun	Not Found	Not Found	1
Peak Foun	Peak Foun	Peak Foun	Peak Foun	Not Found	Not Found	Not Found	Not Found	1
Peak Foun	Peak Foun	Peak Foun	Not Found	Peak Foun	Not Found	Peak Foun	Not Found	1
Not Found	Not Found	Peak Foun	Peak Foun	Peak Foun	Peak Foun	Peak Foun	Peak Foun	1
High	High	High	High	High	Not Found	High	High	1
High	High	Peak Foun	Peak Foun	Peak Foun	Peak Foun	Peak Foun	Peak Foun	1
Peak Foun	Peak Foun	Peak Foun	High	Peak Foun	Peak Foun	Peak Foun	Peak Foun	1
Not Found	Peak Foun	Peak Foun	Peak Foun	Not Found	Peak Foun	Peak Foun	Not Found	1
Not Found	Not Found	Not Found	Not Found	Not Found	High	Not Found	Not Found	1
Not Found	Peak Foun	Peak Foun	Peak Foun	Peak Foun	Peak Foun	Peak Foun	High	1
Not Found	Not Found	Not Found	Not Found	Not Found	Not Found	Peak Foun	Peak Foun	1
Peak Foun	Peak Foun	Peak Foun	Peak Foun	Peak Foun	Peak Foun	Peak Foun	Peak Foun	1
Not Found	Peak Foun	Peak Foun	Peak Foun	Peak Foun	Peak Foun	Peak Foun	Not Found	1
Not Found	Not Found	Peak Foun	High	High	High	High	Peak Foun	1
Peak Foun	Peak Foun	Peak Foun	Peak Foun	Peak Foun	High	Peak Foun	Peak Foun	1
Peak Foun	Peak Foun	Peak Foun	High	Peak Foun	Peak Foun	Peak Foun	High	1
Not Found	Not Found	High	Peak Foun	Peak Foun	Peak Foun	Peak Foun	Peak Foun	1
Not Found	Not Found	Not Found	Not Found	Not Found	High	High	Not Found	1
Peak Foun	High	High	High	High	High	High	High	1
Peak Foun	Peak Foun	Not Found	Peak Foun	Peak Foun	Peak Foun	Peak Foun	Not Found	1
Peak Foun	Peak Foun	Peak Foun	Not Found	Peak Foun	Peak Foun	Peak Foun	Not Found	1
Peak Foun	Peak Foun	Peak Foun	Peak Foun	Peak Foun	High	High	Peak Foun	1
Not Found	Peak Foun	Peak Foun	Not Found	Not Found	High	High	Peak Foun	1
Not Found	Peak Foun	Peak Foun	Peak Foun	Peak Foun	High	Peak Foun	Peak Foun	1
High	Peak Foun	Peak Foun	Peak Foun	High	Peak Foun	Peak Foun	Peak Foun	1
Peak Foun	Peak Foun	Peak Foun	High	Peak Foun	Peak Foun	Peak Foun	Not Found	1
Peak Foun	Peak Foun	Peak Foun	High	Peak Foun	Peak Foun	Peak Foun	Peak Foun	1
Peak Foun	Peak Foun	Peak Foun	Peak Foun	Peak Foun	High	High	Peak Foun	1
Peak Foun	Peak Foun	Peak Foun	High	High	High	High	High	1
Peak Foun	Not Found	Peak Foun	Peak Foun	Peak Foun	Peak Foun	High	Peak Foun	1
Peak Foun	Peak Foun	Peak Foun	High	Peak Foun	Not Found	Peak Foun	Peak Foun	1
Not Found	Not Found	Not Found	Not Found	Peak Foun	Peak Foun	Peak Foun	Not Found	1
Peak Foun	Peak Foun	Peak Foun	Peak Foun	Not Found	High	Peak Foun	Not Found	1
Not Found	Not Found	Not Found	Peak Foun	Peak Foun	High	Peak Foun	Not Found	1
Not Found	Peak Foun	High	Peak Foun	Not Found	Not Found	High	Peak Foun	1
High	High	High	High	High	High	High	Peak Foun	1
Peak Foun	Peak Foun	Peak Foun	Not Found	Peak Foun	Peak Foun	High	Peak Foun	1

Peak Foun	Not Found	Not Found	Not Found	Peak Foun	Not Found	Peak Foun	Peak Foun	1
Peak Foun	Peak Foun	Not Found	High	Peak Foun	Peak Foun	Peak Foun	High	1
Not Found	Not Found	Not Found	Not Found	Peak Foun	Peak Foun	High	Peak Foun	1
Peak Foun	Peak Foun	Not Found	Peak Foun	Peak Foun	Not Found	Peak Foun	Peak Foun	1
Peak Foun	Peak Foun	High	Peak Foun	Peak Foun	High	Peak Foun	Peak Foun	1
Peak Foun	Peak Foun	Peak Foun	Peak Foun	High	Peak Foun	Peak Foun	Peak Foun	1
Not Found	Not Found	Not Found	Peak Foun	Peak Foun	Peak Foun	Peak Foun	Peak Foun	1
Not Found	Not Found	Not Found	Not Found	Peak Foun	Peak Foun	Not Found	Not Found	1
Not Found	Not Found	Not Found	Peak Foun	Peak Foun	High	High	Not Found	1
Peak Foun	Peak Foun	Peak Foun	Peak Foun	Peak Foun	Peak Foun	Peak Foun	Peak Foun	1
Not Found	Peak Foun	Not Found	Not Found	Not Found	Not Found	Not Found	Not Found	1
Peak Foun	Peak Foun	Peak Foun	Not Found	High	High	Peak Foun	Peak Foun	1
High	Peak Foun	Peak Foun	Peak Foun	High	Peak Foun	Peak Foun	Peak Foun	1
Peak Foun	Peak Foun	Peak Foun	Peak Foun	Peak Foun	High	Peak Foun	Peak Foun	1
High	High	High	Peak Foun	Peak Foun	Peak Foun	Peak Foun	High	1
Not Found	Not Found	Not Found	Peak Foun	Not Found	Not Found	Not Found	Peak Foun	1
Not Found	Not Found	Not Found	Not Found	High	Peak Foun	Peak Foun	Peak Foun	1
Not Found	Not Found	Peak Foun	Peak Foun	Not Found	Not Found	Not Found	Not Found	1
Not Found	Not Found	Peak Foun	Peak Foun	Not Found	Not Found	Not Found	Not Found	1
High	Peak Foun	Peak Foun	Peak Foun	Peak Foun	Peak Foun	Peak Foun	Peak Foun	1
Peak Foun	Peak Foun	Peak Foun	Peak Foun	Peak Foun	High	Peak Foun	Peak Foun	1
Not Found	Not Found	Not Found	Not Found	Peak Foun	Peak Foun	Peak Foun	Peak Foun	1
Not Found	Peak Foun	Peak Foun	Peak Foun	Peak Foun	High	High	Peak Foun	1
Not Found	High	High	Peak Foun	High	Peak Foun	High	Peak Foun	1
Peak Foun	Peak Foun	Peak Foun	Peak Foun	Peak Foun	Peak Foun	Peak Foun	Peak Foun	1
Not Found	Peak Foun	High	Not Found	Not Found	Not Found	Not Found	Not Found	1
Not Found	Not Found	Peak Foun	Not Found	Peak Foun	High	Peak Foun	Not Found	1
Peak Foun	Not Found	Not Found	Peak Foun	Peak Foun	Not Found	Peak Foun	Peak Foun	1
High	Peak Foun	High	Peak Foun	High	High	High	Peak Foun	1
Peak Foun	Peak Foun	Peak Foun	Not Found	High	Peak Foun	Not Found	Peak Foun	1
Peak Foun	Peak Foun	High	Peak Foun	Peak Foun	Peak Foun	Peak Foun	Peak Foun	1
Peak Foun	Peak Foun	Peak Foun	Peak Foun	High	Peak Foun	Peak Foun	High	1
High	High	Peak Foun	High	Peak Foun	Not Found	Peak Foun	Peak Foun	1
Peak Foun	Peak Foun	High	Not Found	Peak Foun	Not Found	Not Found	Peak Foun	1
Peak Foun	Peak Foun	Peak Foun	Peak Foun	Peak Foun	Peak Foun	Peak Foun	Peak Foun	1
Not Found	Not Found	Not Found	Not Found	Not Found	High	Peak Foun	Not Found	1
High	Peak Foun	Not Found	Not Found	High	Not Found	Peak Foun	Peak Foun	1
Not Found	Peak Foun	High	Peak Foun	Peak Foun	Peak Foun	Peak Foun	Not Found	1
Peak Foun	Peak Foun	Peak Foun	Peak Foun	High	Peak Foun	Peak Foun	Peak Foun	1
Peak Foun	Peak Foun	Peak Foun	Peak Foun	Peak Foun	Peak Foun	Peak Foun	Peak Foun	1
Peak Foun	Peak Foun	High	Peak Foun	High	High	Peak Foun	Peak Foun	1
Not Found	Peak Foun	Peak Foun	Peak Foun	Peak Foun	Peak Foun	Peak Foun	Peak Foun	1
Peak Foun	Peak Foun	High	Not Found	Peak Foun	Peak Foun	Peak Foun	Not Found	1
Peak Foun	Peak Foun	High	Not Found	High	High	Peak Foun	Peak Foun	1
Peak Foun	Peak Foun	Peak Foun	Peak Foun	Peak Foun	Peak Foun	Peak Foun	Peak Foun	1

Peak Foun	Peak Foun	High	Peak Foun	Peak Foun	Peak Foun	Peak Foun	Peak Foun	1
Peak Foun	Peak Foun	Peak Foun	Peak Foun	Peak Foun	Peak Foun	Peak Foun	Peak Foun	1
Peak Foun	Peak Foun	Peak Foun	Peak Foun	High	High	High	High	1
Not Found	Peak Foun	Not Found	Not Found	Peak Foun	Not Found	Peak Foun	Peak Foun	1
Not Found	Peak Foun	Not Found	Peak Foun	Peak Foun	High	Peak Foun	Not Found	1
Peak Foun	Peak Foun	High	Peak Foun	Peak Foun	Peak Foun	High	Peak Foun	1
High	Not Found	Peak Foun	Peak Foun	Peak Foun	Not Found	Not Found	High	1
Not Found	Peak Foun	Peak Foun	Peak Foun	High	Peak Foun	Peak Foun	Peak Foun	1
Peak Foun	Not Found	Not Found	Not Found	Peak Foun	High	Peak Foun	Peak Foun	1
Peak Foun	Peak Foun	Peak Foun	Peak Foun	Not Found	High	Peak Foun	Peak Foun	1
Not Found	Not Found	Not Found	Not Found	Peak Foun	Peak Foun	High	Peak Foun	1
Peak Foun	Not Found	Not Found	Not Found	Peak Foun	High	Not Found	Not Found	1
Not Found	Not Found	Peak Foun	Not Found	Peak Foun	Peak Foun	High	Peak Foun	1
Peak Foun	Peak Foun	Peak Foun	Peak Foun	Peak Foun	High	Peak Foun	Peak Foun	1
Not Found	Not Found	Peak Foun	High	Peak Foun	Peak Foun	High	Peak Foun	1
Peak Foun	Peak Foun	Peak Foun	Peak Foun	Peak Foun	High	High	Not Found	1
Not Found	Not Found	Not Found	Not Found	Peak Foun	High	Peak Foun	Peak Foun	1
Peak Foun	Peak Foun	Peak Foun	Peak Foun	Peak Foun	Peak Foun	Peak Foun	High	1
Not Found	Peak Foun	High	Peak Foun	Peak Foun	Peak Foun	Peak Foun	Not Found	1
Peak Foun	Not Found	Not Found	Not Found	Not Found	Peak Foun	Not Found	Not Found	1
High	Peak Foun	Peak Foun	Peak Foun	Peak Foun	High	Peak Foun	High	1
Not Found	Peak Foun	Peak Foun	Peak Foun	Peak Foun	High	High	Peak Foun	1
Peak Foun	High	Peak Foun	Not Found	Peak Foun	Peak Foun	Not Found	Peak Foun	1
Peak Foun	Peak Foun	Peak Foun	Peak Foun	Peak Foun	High	Peak Foun	Peak Foun	1
Peak Foun	Peak Foun	Peak Foun	Peak Foun	High	High	High	Peak Foun	1
Not Found	Not Found	High	Peak Foun	Peak Foun	Peak Foun	Peak Foun	Not Found	1
Peak Foun	Peak Foun	Peak Foun	Peak Foun	Peak Foun	High	Peak Foun	Peak Foun	1
Peak Foun	Peak Foun	High	High	Not Found	Peak Foun	Peak Foun	Peak Foun	1
Peak Foun	Peak Foun	Peak Foun	Peak Foun	Peak Foun	High	High	Peak Foun	1
Not Found	Not Found	Not Found	Peak Foun	Not Found	Peak Foun	Not Found	Peak Foun	1
Not Found	Not Found	Peak Foun	Peak Foun	Not Found	Not Found	Not Found	Not Found	1
Not Found	Not Found	Not Found	Peak Foun	Peak Foun	High	Peak Foun	Not Found	1
Peak Foun	Peak Foun	Not Found	Peak Foun	Peak Foun	Not Found	Not Found	Peak Foun	1
Not Found	Peak Foun	Peak Foun	Peak Foun	Peak Foun	Peak Foun	Peak Foun	Peak Foun	1
Peak Foun	Peak Foun	Peak Foun	Peak Foun	Not Found	Peak Foun	Peak Foun	Peak Foun	1
Peak Foun	Peak Foun	Peak Foun	Peak Foun	Peak Foun	Peak Foun	Peak Foun	Peak Foun	1
Peak Foun	Peak Foun	Peak Foun	Peak Foun	Peak Foun	Peak Foun	Peak Foun	Peak Foun	1
Peak Foun	Peak Foun	Peak Foun	Peak Foun	High	Peak Foun	Peak Foun	Peak Foun	1
Not Found	Peak Foun	Peak Foun	Peak Foun	Peak Foun	Peak Foun	Peak Foun	Peak Foun	1
Peak Foun	Peak Foun	Peak Foun	Peak Foun	Peak Foun	Peak Foun	Peak Foun	Peak Foun	1
Not Found	Peak Foun	Peak Foun	Peak Foun	Peak Foun	Not Found	High	Peak Foun	1
High	High	Peak Foun	Peak Foun	Peak Foun	High	Peak Foun	Not Found	1
Peak Foun	Not Found	Not Found	Not Found	Not Found	Not Found	Not Found	Not Found	1
Not Found	High	Peak Foun	Peak Foun	Peak Foun	High	Peak Foun	Peak Foun	1
High	Peak Foun	Not Found	Not Found	Peak Foun	Peak Foun	Peak Foun	Peak Foun	1

Peak Foun	Peak Foun	Peak Foun	Peak Foun	Peak Foun	Peak Foun	Peak Foun	Peak Foun	1
Not Found	Peak Foun	Peak Foun	Not Found	Peak Foun	Peak Foun	Peak Foun	Peak Foun	1
Peak Foun	Not Found	Peak Foun	Not Found	Peak Foun	Peak Foun	Peak Foun	Peak Foun	1
Not Found	Not Found	Not Found	Not Found	Peak Foun	High	Peak Foun	Not Found	1
High	Peak Foun	Peak Foun	Peak Foun	High	High	Peak Foun	High	1
Peak Foun	Peak Foun	Peak Foun	Peak Foun	High	High	High	Peak Foun	1
Peak Foun	Peak Foun	Peak Foun	Peak Foun	Peak Foun	Peak Foun	Peak Foun	Not Found	1
Not Found	Not Found	Peak Foun	Not Found	Not Found	Not Found	Not Found	Not Found	1
Peak Foun	Not Found	Not Found	Not Found	Peak Foun	Peak Foun	High	Peak Foun	1
Peak Foun	Peak Foun	High	Peak Foun	Peak Foun	Peak Foun	High	High	1
Peak Foun	Not Found	Peak Foun	Not Found	High	Peak Foun	Peak Foun	Peak Foun	1
High	Peak Foun	High	Peak Foun	High	High	High	High	1
Peak Foun	Peak Foun	High	Peak Foun	Peak Foun	Peak Foun	Peak Foun	Peak Foun	1
Peak Foun	Peak Foun	Peak Foun	Peak Foun	Peak Foun	Peak Foun	High	Peak Foun	1
High	High	Not Found	Not Found	Peak Foun	Peak Foun	Peak Foun	Not Found	1
Peak Foun	Peak Foun	High	Peak Foun	Peak Foun	Peak Foun	Peak Foun	Peak Foun	1
Peak Foun	Peak Foun	Not Found	Peak Foun	Peak Foun	Peak Foun	High	Peak Foun	1
Peak Foun	Peak Foun	Peak Foun	Peak Foun	Peak Foun	Peak Foun	Peak Foun	Peak Foun	1
Not Found	Peak Foun	Peak Foun	Peak Foun	Not Found	Not Found	Peak Foun	Peak Foun	1
Peak Foun	Peak Foun	Peak Foun	Peak Foun	Peak Foun	Peak Foun	High	Peak Foun	1
Peak Foun	Peak Foun	Peak Foun	Peak Foun	Peak Foun	Peak Foun	Peak Foun	Peak Foun	1
High	High	High	Peak Foun	High	High	High	High	1
Peak Foun	High	High	Peak Foun	Peak Foun	Peak Foun	Peak Foun	Peak Foun	1
High	Peak Foun	Peak Foun	Peak Foun	Peak Foun	Peak Foun	Peak Foun	Peak Foun	1
Not Found	Not Found	Not Found	Not Found	Not Found	Not Found	Not Found	Not Found	1
Peak Foun	Peak Foun	Peak Foun	High	Peak Foun	High	Peak Foun	High	1
Not Found	Not Found	Not Found	Peak Foun	Not Found	High	Peak Foun	Peak Foun	1
Peak Foun	Peak Foun	High	Peak Foun	Peak Foun	Peak Foun	High	Peak Foun	1
Peak Foun	Not Found	Not Found	High	Peak Foun	Not Found	Not Found	Peak Foun	1
Not Found	Peak Foun	High	High	Peak Foun	High	Peak Foun	Peak Foun	1
Peak Foun	Peak Foun	Peak Foun	Peak Foun	Peak Foun	Peak Foun	Peak Foun	Peak Foun	1
Peak Foun	Peak Foun	Peak Foun	Peak Foun	Peak Foun	High	Peak Foun	Peak Foun	1
Peak Foun	Peak Foun	Peak Foun	High	Peak Foun	Peak Foun	Peak Foun	Peak Foun	1
Peak Foun	Not Found	Peak Foun	Not Found	High	Peak Foun	High	Not Found	1
Peak Foun	Peak Foun	Peak Foun	Peak Foun	Peak Foun	High	Peak Foun	Not Found	1
Peak Foun	Peak Foun	Peak Foun	High	Peak Foun	High	High	Peak Foun	1
Peak Foun	Peak Foun	Peak Foun	Peak Foun	Peak Foun	High	Peak Foun	Peak Foun	1
Peak Foun	Peak Foun	Peak Foun	Peak Foun	Peak Foun	High	High	Peak Foun	1
Peak Foun	High	Peak Foun	High	Peak Foun	Peak Foun	Peak Foun	High	1
Peak Foun	Peak Foun	Peak Foun	Peak Foun	High	Peak Foun	High	Peak Foun	1
Not Found	Not Found	Peak Foun	Not Found	Not Found	High	Not Found	Not Found	1
Not Found	Not Found	High	Not Found	Not Found	Not Found	Not Found	Not Found	1
Not Found	Not Found	Not Found	Not Found	Not Found	Peak Foun	Not Found	Not Found	1
Peak Foun	Peak Foun	Peak Foun	Peak Foun	Not Found	Peak Foun	Peak Foun	Peak Foun	1
Peak Foun	Peak Foun	Peak Foun	Peak Foun	Peak Foun	Peak Foun	Peak Foun	Peak Foun	1

Peak Foun	High	High	Peak Foun	Peak Foun	High	Peak Foun	Not Found	1
Not Found	Peak Foun	Peak Foun	Not Found	Not Found	High	Not Found	Not Found	1
Peak Foun	Peak Foun	Peak Foun	Peak Foun	Peak Foun	Peak Foun	High	Peak Foun	1
Peak Foun	Peak Foun	Peak Foun	Peak Foun	Not Found	Peak Foun	Peak Foun	Peak Foun	1
Not Found	Peak Foun	Peak Foun	Peak Foun	Peak Foun	Peak Foun	High	Peak Foun	1
Peak Foun	Peak Foun	Peak Foun	Peak Foun	Peak Foun	High	Peak Foun	Peak Foun	1
Not Found	Not Found	Peak Foun	Not Found	Not Found	Peak Foun	Peak Foun	Not Found	1
Not Found	Not Found	Not Found	Not Found	Not Found	High	Not Found	Not Found	1
Peak Foun	Not Found	Peak Foun	Peak Foun	Not Found	Peak Foun	Peak Foun	Peak Foun	1
Peak Foun	Peak Foun	High	Peak Foun	Peak Foun	High	Peak Foun	Peak Foun	1
Peak Foun	Peak Foun	Peak Foun	Not Found	Not Found	Not Found	Not Found	Not Found	1
Not Found	Peak Foun	Peak Foun	Peak Foun	Peak Foun	Peak Foun	Peak Foun	High	1
Peak Foun	Peak Foun	Peak Foun	Peak Foun	High	Peak Foun	Peak Foun	Peak Foun	1
High	Not Found	Not Found	Not Found	Not Found	Peak Foun	Peak Foun	Peak Foun	1
High	High	High	Peak Foun	Peak Foun	Peak Foun	Peak Foun	Peak Foun	1
Peak Foun	Peak Foun	High	Not Found	Peak Foun	Peak Foun	Peak Foun	Not Found	1
High	Peak Foun	Peak Foun	Peak Foun	Peak Foun	Peak Foun	Peak Foun	Peak Foun	1
Not Found	Not Found	Not Found	Not Found	Peak Foun	Not Found	Not Found	Not Found	1
Peak Foun	Peak Foun	Peak Foun	Peak Foun	Peak Foun	High	Peak Foun	Peak Foun	1
Peak Foun	Not Found	Not Found	Peak Foun	High	Peak Foun	Peak Foun	Not Found	1
Peak Foun	Peak Foun	Peak Foun	Peak Foun	Peak Foun	Peak Foun	Peak Foun	Peak Foun	1
Peak Foun	Peak Foun	Peak Foun	Peak Foun	Peak Foun	Peak Foun	Peak Foun	Peak Foun	1
Peak Foun	Peak Foun	Peak Foun	Peak Foun	Peak Foun	Peak Foun	Peak Foun	Peak Foun	1
Not Found	Peak Foun	Peak Foun	Peak Foun	Not Found	Not Found	Not Found	Not Found	1
Peak Foun	High	Peak Foun	High	Peak Foun	Peak Foun	Peak Foun	Peak Foun	1
Peak Foun	Peak Foun	High	Peak Foun	Peak Foun	Peak Foun	Peak Foun	Peak Foun	1
Not Found	Peak Foun	Not Found	Peak Foun	Not Found	Not Found	Peak Foun	Not Found	1
Peak Foun	Peak Foun	Peak Foun	Peak Foun	Peak Foun	Peak Foun	Peak Foun	Peak Foun	1
Not Found	High	High	High	Not Found	Peak Foun	Peak Foun	High	1
High	Peak Foun	Peak Foun	Peak Foun	Peak Foun	Peak Foun	Peak Foun	Peak Foun	1
Peak Foun	Peak Foun	Peak Foun	Peak Foun	Peak Foun	Peak Foun	Peak Foun	High	1
Peak Foun	High	Peak Foun	Peak Foun	Peak Foun	Peak Foun	Peak Foun	Peak Foun	1
Peak Foun	Peak Foun	Peak Foun	High	Peak Foun	Peak Foun	High	Peak Foun	1
Peak Foun	Not Found	High	Not Found	Not Found	Not Found	Not Found	Not Found	1
Not Found	Not Found	Not Found	Not Found	Not Found	Not Found	High	Not Found	1
Peak Foun	Peak Foun	Not Found	Not Found	Peak Foun	Not Found	Not Found	Not Found	1
Peak Foun	Peak Foun	Peak Foun	Not Found	Peak Foun	Peak Foun	Peak Foun	Peak Foun	1
Not Found	Peak Foun	Peak Foun	Peak Foun	Not Found	Not Found	Not Found	Not Found	1
Peak Foun	Not Found	Not Found	Peak Foun	Peak Foun	High	High	Peak Foun	1
Not Found	High	Peak Foun	High	Peak Foun	Peak Foun	Peak Foun	Peak Foun	1
Not Found	Peak Foun	Not Found	Not Found	Peak Foun	Peak Foun	Peak Foun	Peak Foun	1
Peak Foun	Peak Foun	Peak Foun	High	High	Peak Foun	Peak Foun	Peak Foun	1
High	Peak Foun	High	Peak Foun	High	Peak Foun	Peak Foun	Peak Foun	1
Peak Foun	Peak Foun	High	Peak Foun	Not Found	Peak Foun	High	Peak Foun	1
Peak Foun	Peak Foun	Peak Foun	Peak Foun	Peak Foun	High	Peak Foun	Peak Foun	1

Peak Foun	Peak Foun	Peak Foun	Peak Foun	Peak Foun	High	High	Peak Foun	1
Peak Foun	Peak Foun	Peak Foun	Peak Foun	Peak Foun	Peak Foun	High	Not Found	1
High	Not Found	Peak Foun	Peak Foun	Not Found	Not Found	Not Found	Not Found	1
Peak Foun	Peak Foun	Peak Foun	Peak Foun	Peak Foun	Peak Foun	Peak Foun	Peak Foun	1
Peak Foun	Not Found	Peak Foun	Not Found	Peak Foun	High	Peak Foun	Not Found	1
Peak Foun	Peak Foun	Peak Foun	Peak Foun	High	Peak Foun	Peak Foun	Peak Foun	1
Peak Foun	Peak Foun	Peak Foun	Peak Foun	Peak Foun	Peak Foun	Peak Foun	Peak Foun	1
Not Found	Not Found	Not Found	Peak Foun	Not Found	Peak Foun	Peak Foun	Not Found	1
Peak Foun	High	High	Peak Foun	Not Found	Peak Foun	High	Peak Foun	1
Peak Foun	Peak Foun	Peak Foun	Peak Foun	Peak Foun	Peak Foun	Peak Foun	Peak Foun	1
Peak Foun	Peak Foun	Not Found	Peak Foun	Peak Foun	Not Found	Not Found	Peak Foun	1
Peak Foun	Peak Foun	Not Found	Peak Foun	Peak Foun	High	High	Not Found	1
High	High	High	Peak Foun	High	High	Peak Foun	High	1
Not Found	Peak Foun	Peak Foun	Peak Foun	Peak Foun	High	High	Peak Foun	1
Peak Foun	Peak Foun	Peak Foun	Peak Foun	Peak Foun	Peak Foun	Peak Foun	Peak Foun	1
Peak Foun	Peak Foun	High	High	Peak Foun	Peak Foun	Peak Foun	Peak Foun	1
High	Peak Foun	Peak Foun	Peak Foun	Not Found	Not Found	Not Found	Not Found	1
Not Found	Not Found	Not Found	Not Found	Peak Foun	Peak Foun	High	Not Found	1
Peak Foun	Peak Foun	Peak Foun	Peak Foun	Peak Foun	Peak Foun	Peak Foun	High	1
Peak Foun	High	Peak Foun	Peak Foun	Peak Foun	Peak Foun	Peak Foun	Peak Foun	1
Peak Foun	Peak Foun	Peak Foun	Peak Foun	High	Peak Foun	Peak Foun	Peak Foun	1
Peak Foun	Not Found	High	Peak Foun	Peak Foun	High	Peak Foun	Peak Foun	1
Peak Foun	Not Found	Peak Foun	Not Found	Not Found	Peak Foun	Peak Foun	Peak Foun	1
Not Found	Not Found	Not Found	Not Found	Not Found	Not Found	Not Found	Not Found	1
Peak Foun	Peak Foun	Peak Foun	Peak Foun	Peak Foun	Peak Foun	Peak Foun	Peak Foun	1
Peak Foun	Peak Foun	Peak Foun	Not Found	Not Found	High	High	Not Found	1
Peak Foun	High	Peak Foun	Peak Foun	Peak Foun	Peak Foun	Peak Foun	Peak Foun	1
Peak Foun	Peak Foun	Peak Foun	Peak Foun	High	Peak Foun	Peak Foun	Peak Foun	1
Peak Foun	Not Found	High	Peak Foun	Peak Foun	Peak Foun	Peak Foun	Not Found	1
Not Found	Peak Foun	Not Found	Peak Foun	Peak Foun	Peak Foun	High	Peak Foun	1
Peak Foun	Peak Foun	Peak Foun	Peak Foun	Not Found	Not Found	Not Found	Not Found	1
Not Found	Not Found	Peak Foun	Not Found	Peak Foun	High	Peak Foun	Peak Foun	1
Not Found	Peak Foun	Not Found	Peak Foun	Peak Foun	High	Peak Foun	Peak Foun	1
Not Found	Not Found	Not Found	Peak Foun	High	Peak Foun	Peak Foun	Peak Foun	1
Peak Foun	Peak Foun	Peak Foun	Peak Foun	Not Found	Peak Foun	High	Peak Foun	1
Peak Foun	Peak Foun	Peak Foun	High	Peak Foun	Peak Foun	Peak Foun	Peak Foun	1
Peak Foun	Not Found	Peak Foun	Peak Foun	Peak Foun	Not Found	Peak Foun	High	1
High	High	High	High	High	Peak Foun	Peak Foun	Peak Foun	1
Peak Foun	Peak Foun	Peak Foun	Peak Foun	Not Found	Peak Foun	Peak Foun	Peak Foun	1
Peak Foun	Not Found	Peak Foun	Peak Foun	Peak Foun	Peak Foun	Peak Foun	Peak Foun	1
Not Found	Not Found	Not Found	Not Found	Peak Foun	Peak Foun	Peak Foun	Peak Foun	1
Not Found	Peak Foun	Peak Foun	Peak Foun	Not Found	Peak Foun	Peak Foun	Peak Foun	1
Not Found	Peak Foun	Peak Foun	Not Found	Peak Foun	Peak Foun	Peak Foun	Not Found	1
Peak Foun	Peak Foun	Peak Foun	Peak Foun	Peak Foun	Peak Foun	Peak Foun	Peak Foun	1
Peak Foun	Not Found	High	Not Found	Not Found	Peak Foun	Not Found	Not Found	1

Peak Foun	High	Peak Foun	Peak Foun	Peak Foun	High	High	Peak Foun	1
Not Found	Peak Foun	Peak Foun	Peak Foun	Peak Foun	Peak Foun	Peak Foun	Peak Foun	1
Peak Foun	Peak Foun	Peak Foun	Peak Foun	Peak Foun	Peak Foun	Peak Foun	Peak Foun	1
Peak Foun	Peak Foun	High	Peak Foun	Peak Foun	Peak Foun	Peak Foun	Peak Foun	1
Peak Foun	Peak Foun	Peak Foun	High	Peak Foun	Peak Foun	Peak Foun	Peak Foun	1
Peak Foun	Peak Foun	Peak Foun	Not Found	Peak Foun	High	Peak Foun	Peak Foun	1

Modifications

Suppl Table

Accession
E0CYH4
A0A494BB[illegible]
A0A2R8VK[illegible]
O89050
G3X920
P43277
A0A0R4J0[illegible]
Q4VC33
P69566
Q80YQ8
Q9D7M1
P68404
A0A494BB[illegible]
P62700
Q8BQ46
B1AU76
Q9D7S9
P11928
E9Q3M0
G3UZ01
A0A0A0M[illegible]
P50247
Q80ZX0
Q9JKY0
O09046
Q80WW9
Q8BSI6
F6W623
P98086
Q64514
D3Z286
Q6PFY1
A2AKA9
Q8K2Y7
Q8BKX6
A1L3T7
F6QSC4
Q9R0E1
Q91WC0
B9EI38
Q3TVI8
P60761

Q8CI71
P51827
F6QMA6
P50637
A0A668KL
Q9D051
Q8K2V6
A0A0A6YY
Q9DAU9
A0A1W2P
P14152
Q8CB27
E9Q9D5
Q8CHT3
Q91W69
Q9QY40
E9Q8K8
Q9D6K5
P21956
Q91X96
A0A338P6
P28665
Q99PM0
P46638
Q8CD26
A2AWI7
Q9CT10
P55772
O35453
E9Q2B2
Q8BKX7
Q8CFJ9
A0A2I3BR
A0A0G2JE
Q91WT7
Q9D172
Q8CJ27
Q9CXF7
V9GXE0
B7ZP08
D3YXP6
A6PWZ2
Q80XJ3
Q3U2A8
Q99NF3

A0A140LIX
A0A1L1SV
G3UY49
A0A286YD
B1AU71
Q99NA9
Q0VGT3
Q6NZH9
D3YVU0
P21552
Q80SY5
H3BKL6
A0A0G2JG
Q9D8M7
A0A338P6
Q80TG1
E0CXC2
Q61473
Q3UXF4
P97760
G5E8R3
Q7JJ13
B9EHJ3
Q9JLJ1
Q61823
P70122
P62876
Q9CPY6
Q91VC3
Q91Z96
P61164
E9PW20
B2RXP3
Q8R5C5
Q9DBA6
Q8C0J6
A0A2I3BPI
Q8BJ25
Q6PEB6
Q8K2J7
O55098
A2ALN0
Q3U381
P13405
H3BL92

Q99M01
Q9CQH7
Q9ESJ0
Q8K072
Q62470
E9PYP3
A0A1B0G[illegible]
M1VPI1
Q9ESU6
G8JL51
A2BE20
A0A286YD
Q8CFI7
A0A140LJ[illegible]
A0A1L1SQ
O35744
A2AL50
Q8R0Y8
A0A0X1KG
Q91X78
P63254
A0A0R4J0[illegible]
P13541
A0A5F8M[illegible]
P18872
Q60591
E0CX32
Q7TSF4
Q9WUB4
Q8BH47
Q9Z0H3
B1AZI6
Q9CZ91
Q99KJ8
P40645
Q9CYA6
Q64267
A2AQD6
Q3UH06
Q91W89
Q5F2E8
P63325
D3Z0J2
Q9WTZ2
Q9R0A0

E9PVC7
P97457
F6ZLH4
Q9D8B3
Q8C402
Q9D7M8
Q80UW8
D3Z132
P61089
Q8BH58
D3Z5M7
A0A3Q4EG
Q9WVC3
A0A140LIV
Q8BZ20
E9PX07
A6PW28
B2RXP1
Q3TIV5
A0A338P6
Q8BVQ5
P60003
Q91WQ5
J3QQ02
O70585
P28574
Q9JJG9
A0A0U1RF
B1AVH5
Q8BG81
Q9DB34
Q9DB20
A0A0B4J1
G3UW85
Q91Z83
Q922Z0
Q8VE70
D3YYQ8
P26151
P70218
E0CYW1
P53347
Q9CQT2
H3BKX4
P52785

P49817
E9QAR6
P56873
Q922H9
Q9D8T2
Q62101
J3QNK5
B2RX14
Q8R516
Q80WC3
A0A0A6YX
P70670
P55284
Q7TNV0
E9Q4F4
Q61937
Q64374
Q9CQI9
O88796
Q9QY66
Q8BSD5
Q922J9
Q5DTM8
A0A1W2P
Q8BUP8
Q8BJ64
P81269
E9PWF0
Q99K74
A0A0J9YV
Q9WV55
A0A2R8VK
Q9D902
Q542V3
Q91WA4
A0A140LJI
Q8BJZ0
D6RHT6
A0A0G2JF
A0A1X7SB
Q9CZ44
Q9D1Q4
A0A0B4J1
Q91WK2
Q80W41

A0A0G2JF
Q6PDZ2
Q640N3
P70121
A0A1W2P
Q0VFX2
Q99K48
E0CYT5
P67778
B7ZMP1
A2A6T1
A2AT93
Q8C4V4
Q91YQ7
Q6W4W7
Q91YZ8
Q6ZPJ3
Q9D7F7
P97348
Q9QXS1
E9Q0B5
F8WHU7
Q3UR97
A0A0A0M
O08900
F7CTH4
Q8BMN7
Q8BX32
A0A0R4J0
A0A1Y7V
D3Z2B4
Q6P3Z8
Q8R411
Q9JK92
Q8R124
A0A1L1SS
A0A0R4J1
Q9WTU0
Q9CY58
Q9CZU3
Q07231
A0A1Y7VL
Q3UK98
Q9DCT8
A0A0R4J1

Q60520
Q9QZD9
Q8BMS9
Q99LG7
Q9R210
Q6PFA2
Q9QZB7
Q9DAX9
Q7TPC1
Q8CBY8
Q8C2Q8
Q64152
D3YX27
P97770
Q99KP6
P23116
Q61142
P30681
Q8BG98
Q8R2T8
Q8BID6
Q91WD5
E9Q264
P04925
E9QA79
A0A2I3BQ
Q8VDM6
B2RXC6
Q9CZX8
O54786
F7BWC1
Q922V4
Q9D823
E0CYJ7
Q62086
Q5DU37
Q9CY73
P16054
Q8R429
Q60737
A0A0J9YU
Q673H1
P97868
A0A1Y7VL
O08797

Q8VE73
G3UZX4
Q8BYC6
Q925I1
G5E8G0
Q8BTV1
Q9DBR7
Q01149
Q05512
H7BWY4
Q8C3X8
P25206
Q6P4T2
Q9Z1M7
B1AVN9
Q8K1X4
D3YVS7
P97471
Q8JZQ9
Q91VC7
Q8VDF2
Q9DB77
Q99020
H7BX64
P0DOV1
Q8C1Q6
Q3UNV2
Q61335
P15066
Q8R3F5
D3Z5Q9
E9PUK6
A0A2R8VH
A2ADH1
P11032
A0A0G2JE
P60853
P49718
Q8VC31
Q3UL36
Q99MK9
P08249
Q6PB66
Q91W18
F7BCK0

Q08048
E9Q6K3
Q9EST1
Q9R1P3
Q8VCM8
J3QNR9
O35972
F8WJ98
O54724
Q9Z277
Q8C8U0
Q8CEI3
A0A0G2JF
Q9D2U5
Q9DCC5
Q80UX8
Q5NCB7
E0CY98
A0A0G2JE
B2RRE7
O54833
Q8C033
Q99388
Q9Z329
Q6P9J9
Q8BIX3
A0A2U3TZ
Q8R344
Q8CI08
P62264
Q8C4B4
O35129
Q6PAR0
P81122
P16254
D3Z6I4
D3Z4U5
Q9JHI8
Q8BWZ3
Q9DBG1
P97490
Q06186
Q80TJ7
B1AW40
O89032

P35486
Q6NZJ3
A0A0A0M
F6VRG5
Q9Z2R6
Q9D0S9
Q5U5Q9
Q3U182
Q3UNA4
Q9D3B1
Q6P9Q4
Q8BVG4
Q3US59
Q6ZPJ0
Q924T2
F8WJ43
Q8C729
A0A0R4J0
Q5SWN2
P56183
Q8CDG5
P18293
Q9CZT6
D3Z359
Q8BMK4
P02104
P51859
A0A0R4J0
Q8BJS4
H3BKP8
Q6NVF4
A2AQ25
P43274
Q6QI06
Q9D8X2
P62141
Q3TYK3
Q5F2E7
O08784
O09118
Q80SW1
A0A494BB
Q9DCC8
Q6PD21
Q9CXG3

P58391
A3KGU9
B1AS46
A0A0U1RP
P32233
F7CV40
A0A1L1SR
Q99MR8
E9PY43
Q8CB77
E9PU93
Q8BH40
P61963
Q9JJA4
Q9CQL1
P09411
B1AWG4
Q8BIS1
Q8C0T5
Q8BHZ0
Q8CBM2
Q6PFQ7
Q61655
Q9QWH1
P11087
Q3TLK9
Q09XV5
E9PX53
Q9CPW5
Z4YLR9
A0A087W
P14148
B1AWT4
Q9QY23
A2AJ28
P35441
P16546
P61965
Q8CI43
Q64475
Q66GT5
Q8BSH9
B1AYU7
P97484
F8WIY7

P58404
Q99MR6
Q6PE01
A0A0R4J0
Q6ZWQ7
A0A2R8VK
S4R1W5
P11531
Q8VH51
Q60610
Q99LT0
O88569
Q61581
Q9CX86
P70404
F8VPZ9
Q9DCJ5
P54729
Q9WUM4
A0A0G2JE
Q9Z1D1
Q9CXU0
A0A0N4SV
Q69Z38
Q3UKT9
P84444
Q07139
Q3U3M5
P60766
O54988
Q9D7S7
A2A7K5
Q60992
Q80W49
D3Z2E7
A0A0R4IZ
Q3UKJ7
Q62261
Q80XH2
Q9QUK9
P41105
Q9QZS2
P97503
A0A286YD
Q921M3

O70194
P56382
Q8K0D0
Q6NSQ7
E9Q4K9
P70324
E9PUA5
Q7TT28
Q9DCE9
A0A0N4SV
Q8QZY1
A2AUY4
Q8BGH2
Q3TSG4
Q60668
P29788
Q8VHE0
Q3TE00
Q9JM93
A0A0G2JD
O55091
Q61656
P49312
Q9JME7
E9Q1M6
Q5PSV9
Q91YN0
A0A0R4J0
Q8BGB5
E9QAQ3
Q6PGG2
Q3T9T1
Q3TDT0
Q3TZX8
O08586
Q8CDC7
A0A6I8MX
E9PXC0
Q6P9L6
Q8C142
Q60698
A0A0R4J0
Q91W39
Q9D1I6
Q52KG5

Q05117
Q02248
P97315
Q8R1F0
O08692
E9QP49
Q3U5D9
B2RXS4
E9PUS2
D3Z079
P40201
Q6NXJ0
F6VAN0
D3YXK2
P15533
Q8VD62
A2BHN6
A2AFQ2
Q3UDK1
A2A842
Q80WG5
Q80X73
A0A1W2P[illegible]
Q8VHJ5
P43276
Q64430
Q9Z116
Q3TUQ5
P70372
P07310
E9Q586
Q99N87
Q9CQN7
Q640M1
Q02257
O55106
Q9CQP0
A0A140T8[illegible]
A0A1L1SU[illegible]
F6VQ81
O54825
P49586
Q91YR1
Q9D0D5
Q8BKH7

O54791
A0A087W
Q9CPN7
A0A1L1SQ
G5E8J4
Q99LB2
D3YZV8
H3BJQ0
P49615
Q03160
J3QPC9
A2AIL4
Q3UVG3
E9PUF7
Q5RL20
P05480
B1AZ46
Q8C3J5
Q9JHL1
A2A5K6
Q3UFB2
Q91YK2
Q9JLI8
Q60739
A0A140LI\
Q8BFZ9
P09023
A2AVZ9
E9Q5E0
Q8R2M2
Q569Z6
D3YZ08
Q923D2
Q08943
E9QNT8
A0A1L1SQ
P59326
Q8R310
Q9R078
Q7TQG1
P17095
Q3UC65
P59729
S4R225
D3YWF5

A7E1Z5
A2AS70
Q9Z2X1
B2RRI2
P97857
Q62523
Q80YR9
E9Q638
Q9CX83
E9QLK7
Q8CHE4
F8VPU8
A2CF83
A2ASR8
A0A5F8MI
P31324
Q91WM0
E9Q9B3
A0A494B9
P20152
A0A5F8MI
Q91YP1
Q8C119
Q8BG60
Q9JIZ9
P58742
P56960
Q5RJG1
Q9ERG2
D3Z091
Q8BIZ6
Q9CQE6
Q99ME9
Q3UGS4
Q99LD8
E9Q4R1
H3BJU7
E9Q6R7
Q80YW5
Q6PD03
Q5SX40
Q9CR08
A0A0G2JG
Q9CRB9
O88342

Q9QZR9
Q8BU88
Q8BJH1
J3QNT7
P53986
Q8CF60
Q08288
A2A4N9
E9PYI6
B2RXC1
A6H6A9
Q61818
Q02013
Q9CY16
Q60953
A0A0A6YV
V9GX65
E9Q166
Q9ESX5
Q923L7
Q8CFT2
E9QK41
Q5DTW7
Q8CI33
Q99K41
P55849
Q99JX4
A0A0N4SV
Q8K4W8
Q5SSI6
A0A0J9YTU
Q9Z1R9
E9Q1L8
Q3UP87
Q3TJD7
S4R189
Q8R4H2
P09542
Q14B46
Q3TPX4
Q61586
E9Q4Y4
P26450
E9Q7W0
Q3U0V2

F6YZ95
A0A2I3BR
Q9QXK7
Q9D892
Q9Z2G0
Q6PAL7
Q3TFZ4
P30415
Q6A085
E9QMH1
Q3TIR1
Q61466
D3YUE7
Q9D1N9
P35980
Q01768
O35125
Q8K0H5
Q8BMA6
A0A0A0M
Q8VCQ8
A0A0R4J0
Q99KH8
P05977
Q9CQ06
Q9JLF6
E9PUC5
Q9WUM3
Q8BKT7
E9PYI8
G3X8S5
O70503
A2A517
Q9ESX4
Q9CXU1
P10711
B1AYJ9
Q9QYY0
Q99KI0
Q07230
F2Z461
F7AI47
A0A5F8MI
Q9D174
Q8VIJ6

A0A5F8M(
C0HKD9
Q8R1B4
Q68FD5
P97762
A0A286YD
Q9D824
F6WV21
Q8BHJ5
H3BJJ9
F8WHP8
A0A140LH
B2RUP2
A2CEK1
P21550
A0A6I8MX
A0A1Y7VK
Q6IRU7
Q3UHC1
Q9R118
P70335
Q7TPL6
Q4G0F8
Q80Z96
A0A075B5
Q3U2S8
Q3TMP1
P10126
Q8CD15
Q9CQJ2
A2AIV2
Q6DIC0
Q9CQL5
Q3TFP0
Q9JMG1
A0A0A6YV
Q3UHU5
Q8C7U1
D3Z486
Q8BGB2
P27661
Q3TPE9
Q61666
Q3TB82
P60229

A0A571BE
A2AQE2
A0A498W
P11416
Q80UJ7
Q7TQ48
Q6WKZ7
P97501
Q7TMF2
Q8K337
A0A087W
E9PWR4
Q8BNW9
J3JS28
Q9Z2Q5
Q91YQ5
B1AQF4
A0A0A6YV
Q8R035
Q9CR62
P97377
P26040
Q80ZS3
E9QAT4
P49290
Q8CB87
Q9DCL4
Q6PHZ5
Q0VD84
B1AVH7
P62843
Q59J91
E9Q6J5
Q9DC04
Q9JIK5
Q9Z103
P35235
Q99JX7
P05622
Q9R0B7
O09161
A0A0U1R
Q6ZQ29
P60762
Q6Q477

Q68FM7
Q9D945
Q9CZ19
A0A0G2JG
E9Q0Y4
Q9CRA9
Q4VAA7
A0A1B0GF
Q3U3K9
Q99L28
Q80ZE3
Q9CWL8
Q9DA08
Q9CU62
A0A0R4J0
Q99LC5
Q3UV55
Q7TPV4
Q64444
Q9DBG6
A2AGQ2
A0A140LI7
Q60841
Q9CQ10
P19123
F8WH81
A2BE28
Q91X84
E9Q933
Q2VPQ9
Q99PV0
Q3TWW8
Q9ERG0
O55013
Q8VIG1
A2AUK7
Q99PG2
A0A0R4J0
A2A7S8
Q8VI84
B0R0U1
Z4YLG9
Q8BJ71
Q68FE6
A0A0N4SV

Q9JHE7
A0A5H1ZR
Q9DBG5
Q80UW5
Q5DU25
Q9D8E6
Q8CGB3
Q8C3F2
Q6VGS5
O70551
P43680
E9QAX7
Q8R3N6
Q791V5
E9QLQ3
Q9D666
P31725
B1AX39
Q8VHR5
E9PY39
Q8BGS5
E9Q6L8
A0A0U1R
F6V084
Q8C570
Q9CZX5
Q3UBZ5
B0QZN5
Q80X80
Q9CRB2
Q8VDY9
D3YUQ2
Q5SWD9
Q91WF7
Q6PE65
A0A0A0M
Q6RFH4
Q63844
Q810B6
Q9D777
Q922G2
Q9D8Y8
O35326
Q80XP8
Q9D1M0

Q9WVS8
Q91ZW3
A0A494BA
Q91ZX6
A0A338P6
E9Q3L2
A0A0G2JF
A2TJV2
Q3U0S6
B9EKF4
Q6PHQ8
Q68FG3
Q9DBR1
Q9CQJ4
Q91YU8
Q61114
Q3U2K0
Q8BSA9
Q8VCL2
Q7TMI3
A0A0J9YU
Q03141
G3UYY9
Q9D289
B2RY56
Q9EP53
Q8K284
A8Y5P4
P01869
P63158
P49962
E9Q2E4
Q8JZM0
A0A140LJJ
F6XMS4
Q9CY21
A0A286YD
A0A0A6YX
A2AVR9
P47911
A0A6Q6PU
A0A1Y7VL
D0QMC3
Q9EPE9
Q3TIX9

P56857
Q8CJ53
O35182
Q80ZI6
O35929
Q9JJI8
A0A286YC
Q9Z1Z2
E9Q450
Q6PAQ4
Q9WTW4
Q9DAT5
Q91ZJ0
Q3UQ28
A2AKD7
H3BKF3
P97855
F6SMY7
O35613
A0A0G2JD
P27601
Q640N1
O08917
P11881
Q9QY76
Q9DCH4
O35218
D3YZW1
Q9WU28
Q62074
A0A1L1SR
O08538
H7BX08
S4R211
A0A0A6YV
Q9DAT2
A0A0N4SU
Q8BH95
A2ABV5
A0A0U1RF
Q8R3C1
P16951
Q64277
Q61550
Q8BL48

G3X9G7
P15864
Q62241
Q8VDP4
O08553
Q91WN1
A0A338P7
H3BLL3
Q60644
Q60749
B2RSU6
Q9D4G5
P26231
A0A087W
B1ARU4
Q3UJV1
Q8R3P6
F8WIE5
Q9DAW6
A2AUX5
P99028
D3Z1U9
Q3UZA1
Q3TLI0
A0A1L1SR
P08113
Q9D0E1
P50171
Q5SRY7
E9PYH6
A0A2R8VH
Q9EPK8
A0A0G2JG
P08228
Q08376
P62889
Q3TZA2
Q9Z2U0
P58022
A0A0B4J1
Q9QZ06
P26043
Q9CU65
Q8BPU7
E9Q9M1

Q8C8V1
Q8BFZ3
Q921I9
A6H8H2
Q8VID5
Q8K3B1
Q9CZW5
Q3UB74
P27005
Q9DCT5
F7ACR9
Q62418
Q9QZF2
P70188
Q9R0A5
O70481
Q8K212
A0A2I3BR
A0A087W
P53395
Q8BGT7
Q6PIX5
Q6PI63
Q61792
Q8BG05
Q60649
P0C7Q1
Q9ER67
Q9CWW7
Q3UN02
Q9JI95
E9PWB1
Q8VCG9
A2AQR4
F6VQH5
P67984
F2Z4B7
A0A0R4J0
Q3ULJ3
Q5SUF2
Q99LM9
Q9DBS4
Q9CQR6
F6U6X3
Q9Z2F2

P58196
Q922J3
Q61084
Q80Y14
A0A1B0GS[illegible]
P84244
A0A0R4IZY[illegible]
Q6Y685
Q8K1A6
Q3TWJ1
A0A0R4J0[illegible]
Q8BWT5
Q8VCG3
Q9CPX7
Q60821
Q9QYC7
G3X924
Q8CAQ8
Q05816
G5E8K5
P08551
Q9R244
Q8BM55
O88845
E0CYH0
F6TN13
Q6PCN7
Q8R480
A2A7P4
D3Z7R7
P62317
Q8BG17
O35680
Q8K2F8
A6X8Z5
O88286
A0A0A0M[illegible]
Q8BPP0
Q9DCD2
A2AQA7
Q8K039
Q8BI66
P31695
Q8K4L2
A2A6M1

A0A0R4J0
A2AM70
Q61029
Q99ME2
Q7TT50
A0A2I3BP
Q9CY64
Q9DC07
P26041
Q8BZ03
Q3UMR5
Q62120
A0A140T8
P57787
A0A087W
Q5IRJ6
P70424
Q6PCQ0
P63154
O08856
Q9CPW4
P22315
O08915
Q61169
A2A6J2
A3KFM7
Q9DCM2
Q8JZN5
P61620
A0A140LH
P29351
Q9R1B9
P62852
Q8BX02
G5E924
A2A484
Q3V384
A2AI08
Q9CQN1
P47857
Q5U4C3
Q9R0Q1
Q8K1N2
P29341
Q64355

P57746
Q3UHH0
Q80U38
Q9EQ32
Q07113
Q8K354
Q9CR86
Q3TE80
P83870
P39447
Q9QXX8
P70426
Q8CI11
F6XC25
Q8BHG1
Q924T7
Q8C788
Q8R2S9
E0CXR8
Q8VDM1
P83741
Q3UEB3
E9Q2I4
Q8C0I4
P59222
P58854
Q3UHD2
A2AC16
Q8VD76
Q5U458
Q8BWQ4
E9QN37
G3V001
Q9EQ61
K3W4S6
Q8BU00
E9QP44
D3Z0I8
A2AKX3
Q9CQ73
D3Z3L3
Q8QZY9
Q9D8L3
Q3UI43
A0A1D5RL

Q9Z126
O35382
A0A5F8MI
E9Q3P4
Q62481
Q8QZW8
Q60597
Q8BV49
Q922D8
E9Q9E1
Q91VZ6
A2A7B5
Q00PI9
Q8R050
O35682
A0A140LH
E9Q1U1
P97350
Q9JLN9
D3Z5N6
Q9CYX7
A0A0A0M
Q8K4Q7
Q9ESW4
Q5SXC4
A2A791
Q8BLR5
Q9CQS3
A0A2R8VH
A0A286YC
H3BLI5
Q5XJV7
Q91YR5
Q80Y17
A0A2I3BQ
Q9D3A8
Q99L04
A2A6J7
Q9D8U8
A0A087W
P35969
Q9D819
P51830
P52624
F8WJE3

P59598
Q8BHL5
E9Q4X2
Q9EQ20
Q80U19
Q8BH44
Q62187
T1ECW4
G3UYV7
A0A1L1ST[illegible]
Q9DBS1
Q91VN6
Q8BVD5
Q8R3I3
Q8VHY0
Q62151
Q62448
B2KF50
Q8K3W2
A0A0R4IZ[illegible]
A0A589P9
A0A1B0G[illegible]
Q64012
P83887
Q3UMT1
P45591
Q8BPB0
Q8C3X4
A0A3B2W
A0A0G2JE
A0A1L1ST
Q6NS46
E9QMY5
Q8BTZ7
F6SIJ4
E9Q7K9
Q8BJZ4
Q8VDQ8
Q921K8
E9PXC2
Q80WQ6
Q5SVG5
Q3UMB9
Q9Z0Y1
Q8R3G9

Q91WQ3
O70230
Q91V55
E9PZ11
O70435
Q8BRF7
A0A0R4J0[illegible]
Q921R2
Q922M5
P22518
Q91YR7
O08997
P17563
Q91Z92
Q9DB05
Q8BYZ1
Q60973
Q8VDU5
S4R1X1
Q8VI93
P62911
P23249
Q9CZM2
Q6PD29
Q8VDD9
A2RSQ0
Q9WV34
Q65Z40
Q9DCG9
Q9CQ49
Q99KV1
Q9Z2G9
Q8CHP6
Q9CW46
Q8K010
Q8BTM8
Q91W94
B7ZNL2
Q8BFU2
F8WHM5
Q9Z2D6
P52480
G3UYV5
D3YVU8
Q9CUX1

E9QKB1
Q3UPL0
Q922M7
Q60772
Q497V9
Q8K2A4
Q9CZ62
P35585
Q6PDN3
Q8R4W6
Q9JIX0
P36371
Q80U63
Q9Z1P7
B0QZT2
A0A0A6YV
Q80WJ7
O88738
Q8CG50
P09405
Q9DBB4
A0A1L1SS
O88852
P58466
Q9D0M5
Q8C176
A0A0R4J1
Q8VE19
A2AA59
J3QMG3
Q8CG73
E9Q2I3
Q8VDT9
Q8BJZ7
Q60598
Q6R891
Q8JZL0
Q61093
Q5U3K5
Q61696
Q8VC65
G3X8X7
P08752
B1AQH6
Q9QXV1

Q9D0B1
P37804
E0CXB9
A2ARS0
P83093
Q99LX5
Q8CEC0
Q9JHG7
P62192
Q9R226
Q3UIJ9
Q8VFZ5
Q9WUU7
S4R1P5
Q9JMK2
Q543K9
Q6AXB7
Q3TYX8
O08810
A0A087W[illegible]
Q8K3W3
P83940
Q9CQL7
P61255
Q91VL6
F2Z455
Q8K1C0
Q8C147
E9Q442
Q8BJL0
Q3TYA6
E9PYP2
P70460
A2AU83
F8WGD9
P68181
P62881
G5E8B9
P04919
Q9CR68
P27784
P21279
Q60980
Q8BJF9
D3YVJ7

D3YZJ1
E9QAS5
P70290
A0A0A0M
P61358
Q921T2
A0A0G2JE
Q8CAS9
Q9CW03
Q7TQK0
F8WJA0
Q8BIF9
B2RXQ2
Q9CQQ4
Q059U7
Q5HZH2
Q61771
Q4FZF3
Q9CZ96
G3X9G2
Q9CR56
P70257
Q4ZJN1
A2BFG5
A0A0R3P9
Q5SUR0
D4AFX7
P62908
A2RSJ4
B1AY86
Q8K4Z5
A0A0G2JE
P04187
Q8CHP5
Q9JJW6
Q9Z321
Q8R0W0
B1AU25
E9Q8A3
Q5SPX8
Q9JIX8
A0A0A6YV
Q62077
Q9D1B9
Q60631

P35918
P84091
P54116
Q6ZQI3
Q9D0L7
Q00422
Q8CHP0
Q5NCF2
A2AEX6
Q8VHX6
P27546
H7BX59
A0A1W2P[illegible]
Q8BG95
Q8C0C7
Q7TMM9
Q9DCU6
Q9QXH4
Q8K4L4
A0A0A6YV[illegible]
Q924C1
Q99JY3
Q9JI44
Q9WUQ5
Q925B0
P56546
Q8BTU1
Q8BI08
Q3UIR3
Q9Z1Q5
F2Z4B3
Q02566
Q9CQA1
Q61733
E9Q197
E0CY64
Q920A7
P01899
Q5SF07
Q6PAM1
Q62087
Q64324
Q8R370
P55041
Q6RI63

A0A1Y7VJ
P97370
Q3UCQ1
Q8BMJ3
P27048
Q9CQH8
D3YTR6
P12970
Q7TQF2
Q4ACU6
Q3UZG4
Q9D0I9
P97352
Q8VE80
Q99M15
E9Q811
P21995
A0A0R4J1
A3KG38
A2AQ07
P20491
E9QM38
A0A0N4SV
P54818
Q62172
Q3TMW1
O35071
Q91VT1
P97794
Q80Y44
P59764
Q8C2Q3
Q923T9
Q8R2U2
O08967
E9Q3K3
Q80U35
Q7TN98
Q8R3C6
P84099
G3X952
P26323
A0A286YE
Q9CSH3
Q8VHM5

O08919
Q91VD9
Q80YR4
Q8VEB1
A2ATY4
Q91XD2
O55142
Q8BUR4
Q9D4L6
G3UZM1
Q9D6Y7
Q32MT0
P61979
Q8R5F7
Q91WU5
P46425
K4DI77
Q9D3W6
Q3UM45
D3YTT6
Q8R2N2
A0A0R4J1
A0A494BA
Q7TPM9
Q9D6F9
Q9EQE2
B2RY04
P20918
Q924X2
P53995
Q9ER73
S4R299
Q9R0L7
Q9WUK2
Q9CWX4
Q9D903
Q9EP89
O70311
Q4VBD9
Q8R1G1
Q8BP78
E9QKY4
Q9DCJ7
P61205
Q6P3A9

Q8K0V4
A0A087W
F6XCT0
Q8BYH8
Q91VS8
Q9JK84
F8VPV0
P48999
F8VQ70
Q5SW75
A2AFJ4
O54692
P28653
Q9WTX6
D3Z687
Q9ES74
Q9JMJ2
Q9WV92
Q8BG40
Q9CQR2
Q9WVB4
Q7TQK4
Q3UM18
Q3U276
O88983
Q8BIW1
A0A1L1SV
Q68FF6
B2RQC7
P51437
E9PUV9
A0A2I3BP
Q8BN57
E9Q2E5
A6PWR8
Q80SY4
Q9WVF7
Q60634
E9PVA6
A0A571BG
A2A715
F8WID5
Q6ZWN5
O88665
L7N267

P17427
D3YVC9
Q3UFM5
Q9EP97
D3YW97
Q8BHG9
B1AY10
Q8BZI0
Q91VH2
Q9CYH6
P61021
Q8CBW3
Q64133
K3W4L3
Q8C052
Q9Z2U1
A0A286YC
P62892
Q9R0Q4
G3X9Q6
P13597
P41245
Q62426
Q3THW5
O35344
P14115
A2AEW0
F6XLV1
P16056
Q9CQM8
Q9CX00
A0A338P7
Q9JLM8
D3Z4N1
Q6NXW6
P10820
Q61398
Q9CX48
Q9CQD1
F6YTL8
Q8BLB7
P49813
Q9JMD1
P46414
Q99L88

E9Q5F9
O08804
E9QMU1
Q8C8N2
P97358
P49222
Q9CR57
Q924W7
Q9D938
A0A286YD
Q9JLQ2
Q9R0M3
Q9JHN8
Q9D832
P63168
P68372
O70400
Q5SU71
P26350
Q8CEZ4
Q8VE47
Q99N91
Q9Z1Q2
Q9JM76
Q61554
A2A432
Q8C1B7
A0A0N4SV
P46737
Q9Z1M8
B1ART2
Q99LH2
Q61846
Q9R099
Q91VJ5
Q9CZJ1
Q9ESJ4
Q80YA3
E9Q0U7
O35368
Q80T69
E9Q6Y8
P50285
Q9CZJ2
B2RXQ4

E9Q4X5
E9Q456
G3X963
Q99JR5
A0A1C7CY
A2API8
A0A0G2JG
Q99J25
Q63918
A0A2I3BP(
Q5SV54
Q5FW53
Q9WTX2
D3Z030
B1ARB3
Q8R4K2
Q9D0G0
O54824
O70161
Q8BU14
Q9Z1R4
E9Q616
P62983
A2AAE1
Q922M3
Q8BHS3
P59110
A2AR02
Q8BHL3
P62869
Q5NBU8
Q6A065
Q8CGB6
Q9CQJ6
P70227
P62320
A0A494B9
Q8CB76
Q9DCN2
P31428
Q8K297
Q69ZH9
Q8R307
Q8VCW8
Q76MZ3

A0A338P7
A6PWX9
Q8R3N1
Q9CR80
P19973
A2BE23
P63017
P28843
A0A286YC
P84084
P29416
P35831
Q8CJ40
Q8BHB0
Q8C405
E0CXE7
Q80ZJ1
Q9JKF1
Q8R5G7
Q6PDY2
E9QP00
Q0VAW7
Q80X19
Q9WTJ4
Q8R2U0
P12787
Q80X32
Q8K426
P62137
Q3UFY8
P07901
Q921K2
P52431
P10922
E9Q0J2
Q6ZWQ0
Q8K0Q5
Q6P5D4
E9Q7U2
Q8BTW3
O70589
Q2M4I6
Q9DB15
A0A2I3BPI
E9Q9R9

Q9DCI9
Q8BJA3
E0CXR5
P14869
E9Q9M8
Q5SXR2
P56375
Q80VE5
G3X9J0
Q6P5H2
Q9CQQ7
Q9D0M3
Q8VE88
Q9QYJ0
Q61107
P62915
P59997
Q3TFD2
Q61584
A0A0U1R[illegible]
Q3UQ17
Q8C4U3
A0A0R4J0[illegible]
Q9WVG6
P08122
Q99J95
P47226
Q8R205
Q9EQK5
G3X9K3
Q8BZZ3
P49935
Q3UHD6
O55022
P97434
Q7TQJ8
P16374
A0A1L1SR
Q80YR5
E9Q4Q2
P27773
E9PX66
A2AIV8
Q9DBF7
Q3TYS2

Q9JMB0
Q8K4B0
O35704
Q7TSC1
P68033
Q9R0M4
E9QP62
D3Z3K7
P55097
B1AVZ0
Q3UXU8
Q9D7H4
Q8K1C9
E9Q0S6
Q8VE37
Q8BID8
Q8BVE8
G3X9X1
Q9D1F0
Q9JLQ0
Q8CI03
Q5SVP0
Q0PHV7
Q9JJY4
Q00547
Q04692
Q6P5B5
Q9D2G2
A0A0R4J0[illegible]
Q02788
Q9WUM5
A2ALW5
P63101
Q9WUN2
Q91XU3
P05132
Q8BK72
Q9D0J8
Q6P5G3
O89100
A0A3B2W[illegible]
Q7TPN9
Q9WU22
Q9CXS4
G3X9F6

Q3V1N5
Q9JKY5
Q9CQA5
Q00899
Q9QZ85
F8WIV2
Q3URQ0
P97351
B1AXH1
B2RQG2
Q91WJ7
Q9JIK9
P31240
Q8BTI8
Q8K4I3
D3Z7P0
P24788
G5E8Q0
P06339
E9PVX6
Q91VT4
Q9CWK8
Q69ZB3
E9Q564
Q8BTU6
Q9Z0H1
P51150
Q8CGC4
Q8BGA5
Q9R257
Q7SIG6
E9QP99
D3Z2H9
Q80UU1
A2A9M4
A2ARW8
P68433
P25444
E9Q906
O88967
Q9JIA7
F7D3E1
A0A140LH
Q8K349
Q5RJ54

Q5SSK3
Q80T06
Q62095
E9PV86
A0A0A0M
Q9JMG7
Q9DBV3
Q9DCX2
Q9JHJ0
D3YYT1
P10107
P57780
O89023
Q9CQD4
Q70FJ1
Q810D6
H7BX38
Q8BJ90
Q9QUJ7
P30416
Q8VEH3
Q99JH1
Q6PGF3
P17932
Q8K031
Q8BGD6
Q6KCD5
P97384
Q8BVW3
O89090
E9Q8I7
Q9CXT8
Q78IK2
Q91VY5
Z4YKM2
P58871
Q8BKC5
Q8CIM8
E9PVA2
Q9DB16
Q9EQI8
A0A0R4J0
Q9ESN6
Q61635
Q4U2R1

Q9JMC3
B1ASA5
G3X926
Q3LR78
Q8C2K1
A0A494BA
Q9JMC8
P70245
Q9R1T4
Q9ERR8
P62242
A0A0A6YV
D3YY75
A0A1D5RL
B1AUH1
A0A1L1SR
Q8R015
A0A140LIU
A0A1L1ST
Q9Z2E1
A0A1L1SS
D3YTV2
A0A0R4J0
Q9JII5
P51660
A0A0R4J2
Q6PEE2
P62830
Q08093
Q5SUH7
Q99PL6
A0A140LH
Q8BVY0
A2AWT7
G3X941
A2CG76
Q3TKT4
Q0VF22
A2AHK0
E9QAJ9
A2BDW0
Q99JY9
P55096
Q3U829
Q8K0L9

Q9WVL3
A0A087W[illegible]
Q6P4S8
Q62312
Q99LH1
Q6PAJ1
Q8BVQ9
Q8CGC6
Q4FK66
Q922F4
F8WJG3
A2AH75
H7BXC3
Q8K2W3
A0A0N4S[illegible]
E9QPI5
A0A0G2JE
A0A087W
Q8BGQ7
Q99M87
E9PWH2
E9QA05
D3Z0C1
O89103
Q99JP6
Q8VHQ3
Q62167
Q8VEK3
Q8VDF3
A2BH40
Q4VAC9
Q99LG0
Q8BIJ7
Q0VBL3
F8WI14
Q9R1C6
E9QPR4
O88895
E9PW43
Q6IRU5
A6H5X4
A0A0G2JF
Q8BG07
D3Z4C9
E9Q2H1

Z4YK03
Q8CFG0
D3YYK8
E9Q317
Q9Z2D1
Q8CBA2
E9QL25
Q9ET54
B7ZC46
P97479
Q3UMF0
Q9QYB5
Q9WTX5
P62806
Q14C51
Q64462
Q8VC03
A0A494B9
P15327
P70333
Q9EQI5
Q8BFV2
Q5NCC3
O54785
Q5SWU0
O08582
A0A0R4J0
Q91YI4
Q8BL06
Q8BGD9
Q9CZ83
D6RDS0
Q8BVD7
P22907
Q8BKJ9
P08207
Q9JJ89
Q5U4C1
Q9CQY7
Q8BPB5
Q9WVK4
O88848
E9Q1W0
P32921
Q8R0P4

E9Q7Q3
Q09014
A0A1Y7VJ
A0A452J8[illegible]
Q9CZD5
Q8CH25
Q9QXW0
L0N7N1
A0A1L1SQ
G8JL40
Q6ZPG2
E9PUQ9
Q8BVK9
Q8K363
P50462
Q99LD4
E9PWE9
Q6ZQ08
Q9QUG9
Q8BH59
Q61233
E9PYL2
Q80V42
Q8CH09
Q8BVL3
Q80UR4
Q68FL4
A0A0N4S[illegible]
P53349
Q8C0M0
P70303
Q811S7
Q9WUD0
Q64282
Q7TND5
Q69ZP3
O09110
Q8VCC1
Q922Q1
P61514
A0A668KL[illegible]
Q99L48
F6TLV3
Q8BP48
P40336

D6RI62
Q2NL51
B2Z894
Q9CS00
Q3TFK5
F6RJE8
Q8R054
D3YWK1
P23298
A0A0N4SV
P40646
Q6P3Z4
P46412
Q9D787
Q9JJ28
Q8CD10
D3YVI6
Q8R0L9
Q9Z1Z0
Q61687
E9QK48
Q99104
B1B1D8
Q9JL16
H9KV21
D3Z041
Q5ND52
Q9D0D4
Q61164
Q8K4F6
Q8K355
O35226
Q9D071
D3Z342
A0A087W
Q9DAW9
Q9D6Z1
A0A7N9VF
Q3U5V8
P54823
Q9Z2A0
Q9JII6
A0A087W
O88273
O08663

Q8CGY8
Q8BG30
Q3TRM4
E9QMN5
H3BJY2
Q8BJ05
Q99LI5
A0A2R8VH
Q9D706
Q6DID5
Q6P2B1
Q61136
O70572
P61202
Q3UWM4
Q9Z2Q6
Q8R409
Q9QUM9
Q80VM4
Q8C0C0
Q8K3Z9
P00493
Q9CQ69
Q80X68
Q9CX11
Q8CDJ8
Q8BHZ4
A0A0A6YV
P17665
Q9Z204
I1E4X1
A0A1Y7VN
Q9CR39
Q3U4F0
Q9WV54
Q3UHX2
Q62465
F6WMC0
Q6P9R2
B1AXN9
Q07802
P52293
A2AWE9
F8WGX5
Q80YE7

E9Q8N5
Q3TKY6
A0A1B0GS
G3X9H7
Q80TF6
Q9QYS9
Q6PAL8
P26039
A2ADA5
Q5F4T0
A2BE93
A2AG30
D3YYC2
F6TKU5
P97499
Q9DBM1
P70275
Q8C0G2
D3YTL5
A0A498W
A0A087W
Q80TE4
Q9DCT2
P26645
G3UX26
P42669
B1AVD2
Q91WB4
Q3UHE1
D6RGR4
P15105
A0A0R4J2
Q9CWX9
Q8CG46
Q91YT7
P97452
Q8K2J0
P62715
P52927
P41233
Q04841
O35730
Q8CHI8
P68040
Q792Z1

P56387
Q8K2M0
Q8BZR9
E9Q557
J3QK13
P99027
Q69ZR9
Q6NT99
Q8BXK8
Q9WTP7
Q8VHK1
Q99JB2
V9GXS9
Q8CC88
B2RVL6
E9QQ91
Q3TIU4
Q91YS8
Q9CQS5
P97872
A0A0N4SV
Q3TWM5
P14206
Q8BKW4
H9H9S3
Q8R151
Q9CR47
Q9D287
P04627
Q925I7
Q8VDN2
P58468
Q69ZX6
Q3UPD8
Q6PGH1
Q8K4X7
Q0VEE6
Q9QZB9
E9PUR6
Q9JI11
Q9CQ19
Q06138
E9PWH3
P62900
Q8C6P8

E0CYF3
Q8BKN5
Q5DTX6
Q9NWG9
Q61598
D3Z2U5
A0A0A6YX
Q5F293
P98192
Q3UHX9
Q9WUR2
D3YYJ4
Q6PER3
Q8BX70
A0A1W2P
G3UWZ0
Q8C0D5
E9QB02
P48036
Q8R2V5
Q5SVR0
Q69Z89
Q921W0
Q8BVP5
Q8CIZ8
P46664
Q8K2D3
Q9Z0F8
O35343
Q04750
G3X8R7
A0A1W2P
A0A5F8MI
Q9D2D7
O35250
G5E905
Q6P8P8
Q9ES70
Q99LE6
Q9CQ80
D3YW09
T1NXB5
Q9QYB1
Q3U0J8
A0A1D5RL

Q91YD9
A0A0J9YU
Q6PGF5
E9PYK3
P70268
A0A2I3BQ
Q9D1H9
Q6IRU2
P28028
Q99P91
E9Q1G8
P27659
P53994
Q8BXZ1
E9Q748
Q8CE90
Q9JJA9
Q7TNC4
A0A286YD
Q5PRF0
Q5SUA5
P99024
P62488
P59240
P35564
Q3UI84
Q9JKK7
A2A5Z6
P97496
Q8BHE8
P20029
Q9Z0P5
E9QPI2
O35973
Q8CGC7
Q9R233
Q8BH61
Q3TL44
Q6ZWV7
P61027
A0A0R4J1
A0A286YC
Q8BT07
E9QLA5
Q8BL66

A2AT37
Q9Z1D9
Q9JLB2
P62849
Q9CQA3
B2RT41
E9Q652
Q6I6G8
Q91X46
Q5XG71
Q8BKF1
Q5SYL3
Q9D198
Q8CIH5
F7DF70
Q8BTN6
P14131
Q9JI91
A0A571BE
E9PUQ8
Q07076
Q9Z2I8
P62245
Q6KAR6
Q8BL97
Q64707
P97821
Q9DBR0
F8VPR2
Q9D0L8
Q6ZPL9
Q9DB91
Q3U367
G5E8J3
E9Q8B6
E9PYZ7
Q8BTI9
Q8CFI5
P50543
Q9QYL7
E9Q967
O35738
Q9Z0N1
Q922Q4
Q8BU30

Q8BPM0
O55201
P61406
P06798
E9QAF9
O35945
O88844
O54950
P35330
Q5NCI0
A0A0U1R[illegible]
D3Z0S1
Q80X98
Q99LC3
Q8BX10
F6UFG6
O08749
Q8BMC4
A2AS45
P48722
Q8K301
Q9D2E2
A0A0U1R[illegible]
A0A1Y7VL
G3X9W4
Q9Z1Y4
Q69ZX8
P70441
Q8VEE1
E9PV45
A0A140LI[illegible]
E9Q1K3
P62307
Q9D1E6
P39876
E9Q9E4
Q64511
J3QNU6
Q8K449
Q6NXK5
D3Z383
A0A0A0M[illegible]
Q9CQI6
Q8R5A6
Q3TUQ7

Q9Z1F9
E9Q2S7
M0QWP1
Q9D104
Q9D554
A2ALV3
Q6ZWU9
E9PZM4
Q9JHS9
Q99KK1
Q9DBD5
S4R2J8
Q91UZ1
Q9CQI7
Q3TIR3
P50172
Q63850
V9GX00
P28293
A0A0N4SV
Q99J09
Q8JZX9
Q9DBC7
Q8CCP0
Q3UKC1
Q3UFY7
Q80SZ7
O08807
E9Q9M5
B9EIX2
Q9JIM3
Q9Z2Z6
E9Q133
F8VQB6
Q9ERH4
A2AFP4
A0A0R4J0
Q62240
P26443
Q9DC48
Q9DB94
O54941
Q9D7H3
Q5F2F2
Q9CZA6

Q925E7
A0A1B0GI
A2AMW0
Q9R1T2
Q9CX34
Q5KU39
E9Q109
A0A0A6YX
Q922I7
Q8R317
Q8BGZ3
Q64523
Q61235
Q505F5
D3YZ09
Q9D8W5
Q6NXL1
P14211
Q56A04
D3YUP5
Q9D1M1
A0A338P6
E9PUP1
Q62504
Q8CG29
Q80U93
Q9QXD8
E9Q804
Q99MP8
O35493
Q9D5V5
Q6PAC3
Q6PGC1
Q7TPQ3
O35492
Q9JJZ5
B1AXS8
Q8BND4
Q8BGS0
Q6A0A9
Q06806
Q8BWM0
Q9QUK4
Q9JJF3
P49182

G3X928
Q60865
I7HPV9
Q61166
Q6RHR9
P20065
P97468
Q8C6N3
Q8BK63
O88939
Q9CWF2
Q80TH2
Q8C845
P01872
Q60972
E9PV24
P68254
Q99N50
Q9WTL7
Q6NWW9
Q8CBY1
Q3UMW7
Q9CQE8
Q8C9V1
Q9Z0H8
Q6PHN9
Q60949
Q64343
G3UYP4
P15306
Q3UHQ6
P56528
Q9D880
Q6PGE4
Q3U2X6
P49452
A2A5R2
Q91Z49
Q8CCM6
Q8K327
A2ACH6
E0CZ18
Q69ZA1
A0A0R4J0[illegible]
Q9JHK5

Q9D0B0
O55187
D3Z7C6
Q9DBP0
Q3TNL8
Q4V9W2
B2RXM6
E9PVW8
Q60843
Q6GV12
Q99MD8
P10076
Q9D7N3
Q9QXM1
E9PY25
E9Q7D5
A2AS03
Q6P069
D3Z3S1
Q9Z0X4
Q6GQT9
F8WI37
P11499
Q8CJ69
Q8K310
P59016
O88783
E9Q559
E9PW37
Q80XK2
Q8BMF4
Q6NSQ5
P98203
Q3TWF6
A0A0U1R[illegible]
Q8C9B9
E9PX30
P51863
S4R1H5
E9Q4P1
F7A043
A2RSY1
P97310
Q07079
Q4FE56

Q3TLP8
F8VPZ5
A0A0G2JF[illegible]
Q9CRB6
Q80UE5
Q8K4Q8
Q3UK02
Q8K2R5
Q9EQG9
P00329
A0A0R4J0[illegible]
Q8BWR4
O35099
Q8BP92
Q791T5
E9PVA8
A0A0A0M[illegible]
P63094
A2CG35
Q64471
Q8BXJ2
Q60605
Q9ES46
B2RXC8
Q9CQV5
A2BFF8
B2KF11
D3Z4B0
Q3TEA8
O08530
F8WIZ7
E9Q668
E9Q7E2
P70452
Q921N6
O88952
A2A6Q2
Q06318
P33267
Q4VA98
Q6DFX2
P70279
P43135
Q99MJ9
P68369

Q9QZS3
Q93092
Q8C605
P17012
Q09200
Q5SYD0
A2BEF2
Q8BWY3
E9Q8D0
Q91XD6
A2BDX3
A0A668KL[illegible]
Q6NV83
Q99KQ4
Q03265
P38647
Q7TPR4
Q9WTK5
Q9D024
P70392
A2BHP5
Q8BHN3
Q9DCA2
P97287
P15508
Q99LR1
Q8BKG3
Q9D937
P28740
Q8K1G9
Q9QZS7
P07724
Q3TML0
Q62219
Q99LL5
F8WGD2
Q3V0K9
P24369
Q9D6T0
A6H5Z3
Q5SS87
Q9D0M0
Q02105
Q9DBH5
Q6NZQ6

P63276
P27870
A0A0R4J1[illegible]
Q7TMY8
Q8R5H1
Q6NVF9
P23188
Q9CWT6
Q9JJL8
E9Q6L0
Q9CPR7
Q8BIE6
Q8BTV2
Q07417
P08905
Q99MU3
Q3US17
A0A668KL[illegible]
Q61263
Q8K124
Q8VCM7
F6TFN2
A0A5F8M[illegible]
D3Z0M9
O88307
G5E8B1
P18760
E9PUG7
Q05186
A0A0R4J1[illegible]
F6Q475
Q9DC28
Q6PAR5
Q8BMJ2
F6RL59
Q9R1P0
H7BX01
Q8K1H1
Q8BUK6
Q8K4Q6
O35685
A2A8U2
E9QAH1
Q60960
Q9DBH0

A2ALK8
P97465
Q8CAA7
O35608
Q3UJK4
Q8CAK3
Q3V1M1
O35350
Q60902
Q8VDP3
Q61001
Q7TMC8
O35841
P31001
P10493
Q99JH8
P41183
Q8CGV5
Q6PGA2
Q8K183
A0A0R4J1
Q501J6
B2RR83
A0A0G2JD
P62874
Q9CS42
A2A816
A0A1W2P[illegible]
Q8BN21
E9Q634
O35954
Q9DAP7
A0A0A0M[illegible]
P97333
P43275
Q8BY46
Q8BMP6
A2ACV6
A2A6A1
Q3TJ91
A2A5R8
Q80U70
Q9JI13
Q921F4
Q9D8N0

Q62048
D6REV6
Q99PT1
A0A3Q4EI
P61222
Q99L20
Q6PG04
Q3TCR7
P14106
A0A5F8MI
G5E866
D3YZG8
Q61080
Q9R0Q6
P09470
Q8C079
Q8VD65
A0A0U1RF
P08032
O88653
O88792
Q08857
O08583
Q80TE0
P49717
Q8C6G1
P63271
Q80X90
Q8BI72
Q9WU78
Q99P72
Q9CS74
A0A494BA
Q8R1H0
Q5H8C4
A0A140T8
P61079
A2A841
G3X956
J3QNU1
Q8BMQ2
P39654
P62918
Q9CQF8
P60840

Q4FJZ2
D3YUY3
Q7TN16
Q9R1W5
P55200
Q4VA93
E9Q4T4
Q8BU31
O70333
Q9R1Q7
G3UXL2
Q8BQ30
Q80U72
P17433
Q61990
B8JJ56
A0A5F8M[illegible]
P97440
Q921M7
Q921R4
A0A0G2JD
Q6PAV2
Q9DCF9
G3X973
E9Q3G5
Q9D2H6
Q60790
Q9CQ39
Q6ZWX6
Q05CL8
Q9JLL0
Q9CQM5
A2A5L7
E9Q5K9
A0A0R4J0[illegible]
A0A2I3BR
Q80UM7
P42337
Q64318
Q6P3Y5
A3KGF9
E9Q1S3
D3Z158
E9QJS7
Q3TXV4

Q8BIG4
Q9Z2C5
Q9JI33
Q8CI51
Q9CQI1
A0A3B2W
G3X960
Q99K28
Q3B7Z2
Q91VL9
Q8K2H4
Q9R0P5
Q9EPU0
Q99K23
Q8BJY1
Q3U213
G3UW40
Q80X91
P11247
Q9EPT5
Q3ULG5
Q8VCG1
Q8K214
A0A0U1R[illegible]
Q8BGC0
F6Y6L6
E9Q8K5
Q8VDM4
Q8R349
Q3U1Y4
P16330
P35700
Q6P5B0
Q9WUA2
Q9R1K9
P11679
P63085
F6RCM5
Q3UU56
Q3TXS7
B2RRL2
Q9QVP9
P29268
Q99P21
Q9QZQ8

P13864
G5E8U9
E9Q3I3
Q9D168
Q8K1N4
Q9JHU4
S4R2T5
Q5FWH6
Q80VW7
O88673
Q8BJT9
P13020
Q8CH18
E9QLT6
E0CYE2
Q80VP0
Q80YW0
E9PWW6
Q6ZWQ9
Q922Q2
Q8BZQ7
H7BWX9
D3YVM9
P70336
Q9CQF3
Q8R5K4
A0A0G2JE
Q9WVG9
Q8R4I1
O35955
P29037
Q99LB0
Q02526
Q62137
Q9D1W4
A0A0R4J0[illegible]
Q9D6Y9
Q63829
E9Q3F3
Q6NS86
Q8VE22
Q91VS7
D3YYY8
D3Z4J9
Q5SW28

Q69ZT9
P47963
Q91VJ2
Q80ZW2
Q8VDD5
Q8BVC4
Q9CXW3
Q9DBE9
Q3USZ8
P62751
Q4LFA9
Q99KY4
P56480
Q99L90
Q9D8U0
Q5SRX1
Q9WUU9
Q62009
Q9DC51
Q9D898
Q8K3A9
Q9JKX4
Q8BJ34
P84104
Q3UHC2
D3YXK6
Q8BZF8
B1AYL1
D3Z3D6
Q80WR5
Q9CPS7
P62315
D6RCH5
Q9D3D9
Q00417
P26369
P14733
E9PYL4
Q9QXT5
P62702
Q61249
D3Z6Z7
O35639
Q99MQ4
Q1HFZ0

Q9CWZ7
Q6URW6
G3X9Z4
Q9CRD2
P18406
Q9D1C9
Q3V117
G3UY70
P28741
G3X9T7
Q8BUN5
Q9JI78
E9Q8V6
A2AN08
Q9CXY6
Q61878
Q04857
Q99JR8
Q91YM2
A0A140LIZ
O70583
P62331
Q8CGF7
P32020
E9Q175
A0A0N4SU
Q3TGM7
Q8BGS1
Q8BHF7
Q91WT8
Q3UDC3
E9PYX7
H3BJM1
Q03526
P10639
A2AJG0
Q8VDC1
K4DI71
Q9Z1N5
A0A494B9
Q9Z1J3
Q91YN9
Q9QWT9
Q497V5
G3X925

Q8CCJ3
A2AMN2
E9QLS6
D3Z6R9
Q9CQX4
Q02956
O35286
Q8BLF1
Q6PFX9
Q5RKN9
Q52KG4
Q8R1F1
E0CX53
Q6P5D3
D3Z3A8
O55029
Q9D1P2
A0A1D5RL
P35979
Q9DBS5
Q8CGK3
Q9JMH9
P16460
P11983
Q9JK83
G3XA69
Q9Z1A1
Q8VCT4
O88532
Q9Z2B9
Q8BHL4
Q3LAC4
A0A1Y7VJ
E9PYD1
Q3UPF5
K4DI62
Z4YJP0
Q91VN1
P35279
O88327
P97875
Q6PCM2
Q9Z2A7
D3Z558
E9QN31

Q9JM51
Q80WQ2
Q99J47
Q60870
Q91YS7
S4R2P2
P16110
A0A1W2P
E9PWE8
P06909
Q9JJD0
Q922B2
Q62311
Q05DP1
E9QN70
F6ZFU0
B1AUB9
Q3V1T4
Q922Q8
D3YXN0
A0A1Y7VJ
A0A0R4J1
E9Q5J9
Q9DBZ5
A2AF67
D2EAC2
O89053
Q9WTM5
Q9DB96
Q8C7R4
Q9JIF7
P01837
A8DUK4
A0A0R4J2
P63087
E9PWU4
Q8VI94
D3Z065
Q8BMI0
A0A5F8MI
E9QKZ2
P10605
B2RQE8
P50518
Q9EQJ0

Q62036
P60898
P54728
P24549
P14685
A2ALV6
S4R1J5
Q9CTH6
Q3TDD9
P21981
Q922B6
P17156
Q9Z2I9
Q9WV95
A0A5F8M[illegible]
Q8BW75
A0A1L1SR[illegible]
S0DHL8
Q4LDD4
Q8BG51
A0A087W[illegible]
Q9CWV6
D3YUP0
Q07797
Q91YL3
Q9DB42
Q9WUP4
Q99PU7
P47915
Q06180
E9Q8D5
P62855
Q9WTR5
Q8K1B8
Q8VCH6
Q3UWE6
D3Z7R9
P17182
P35550
Q8CIB5
P47738
Q76LS9
A0A286YE[illegible]
Q14AX6
Q7TT37

P97379
Q8BX80
I7HLV2
Q8BUE4
Q05DG3
Q0VBM2
Q9CPT5
Q80SU7
Q9DBE8
Q5SVQ0
Q8BFR5
Q5DTZ0
Q8BH73
Q91Z67
P62313
E9QPM7
Q3UG20
Q80VN4
Q61151
G3X9H5
P25799
Q1H9T6
A0A1B0GX
E9Q4M2
Q6GQR8
Q8VHK9
Q8K1Y2
E9Q722
P41241
Q9DCL9
P08103
Q9JIY0
Q9D4J7
P58252
P12815
P11214
Q6NZL8
A0A0N4SV
Q6PR54
Q5SUT0
Q91VX2
Q9CZX7
G3UVV3
A0A494BA
Q9JHP7

Q9R117
Q8BHG2
A0A1L1ST
Q9D8C4
Q9JI39
O70468
D3YYN8
Q8R0F5
A0A140LJ[illegible]
Q5SSH7
B7ZCR6
P46460
Q3UQL6
Q9CZU4
Q61140
G5E8P1
A0A1D5RL
P10649
P50516
Q60847
Q9JLV5
Q99KC8
A0A1W2P[illegible]
P62267
Q02111
G3X957
Q8BVY4
Q7TT36
A0A0B4J1
Q9D1R9
A0A494BB
Q9EST5
A0A1B0G[illegible]
Q91V88
A0A1Y7V[illegible]
Q9JJ00
P47754
Q8R1Q8
Q91VR5
Q6NZB0
D3Z4D8
Q2M3X8
Q6PE54
Q6P9N1
Q7TMG8

Q8BH60
Q8BTW9
Q8C6B9
Q3U492
Q8K2L8
P28798
Q61712
A0A0R4J0
Q9ERS2
Q3UW53
Q91WF3
Q80UP5
J3JS23
A2A4B1
F8VPP8
Q8BP67
Q8K4L0
Q9JK53
P48962
Q8R3F9
P62862
Q99K30
Q61503
P47757
Q61187
Q7TR54
B2RWS6
Q99JP0
Q6NZL1
Q9JKN1
P27612
Q3UH59
Q9ERU9
Q924N4
P40936
A2AVJ7
Q9ESP1
E9QMU3
Q8R5L3
A0A1D5RI[illegible]
Q8BGQ1
Q91V81
Q80TN4
Q6NZF1
F6RGI3

Q9CZG9
Q8CIP4
Q7TSI3
P01901
Q61292
Q8K019
Q3U6N9
A0A498W(
Q80T21
Q8R4U7
Q99K94
Q8K2C9
Q8CCF0
Q99JZ4
E9PXY8
Q62419
G5E881
Q3UFK8
A2AJI0
Q8BK67
F8VQK5
Q52KG9
E9QLL2
E9QAQ5
P80315
P59999
P70451
P61161
D3Z0K6
O35245
Q8BHS6
E0CXC5
Q99LJ0
Q9D658
Q3UID0
Q9ER58
Q60766
A2A716
P0DP27
Q9JHR7
K3W4R7
E9PUN6
Q6P9J5
Q543M9
E9Q6A7

P54071
P70168
Q80UK0
A2AJW4
Q05D44
O89079
E9Q9S3
P62071
Q6P9R1
Q8C0Q9
O55143
A0A571BG
P51432
A0A140LJ7
Q8BH02
Q91VE6
Q3TCH7
Q7TPD1
P60867
Q8BG79
Q80VQ1
E9PXF8
P61211
F6WL90
O54865
E9PZJ8
P17897
Q02053
O54734
Q3V3Y6
Q6A0D4
Q3U0M1
Q9QXK3
Q91UZ5
D3YTQ8
Q9DCM7
P50544
P03975
F8WI26
P42932
Q7M6Y3
Q9Z266
Q8CG79
Q9DCR2
D3YUW7

Q9Z120
P28301
Q58A65
E9PUA3
Q99JI6
Q78YZ6
A0A0R3P9
Q3U9G9
F7BJK1
Q9ET78
Q5FWK3
Q03963
Q8BML1
G3X972
P46735
Q6P5F9
Q8BMD8
Q3THK7
A0A6Q6Q/
A0A3Q4L2
Q9CX97
Q9WVE8
F6V8M6
Q8BSL0
A0A2R8W
P42128
P62754
Q3UGP9
J3QMC5
Q9CQE0
Q8VCC9
Q91VY9
Q8VDP2
Q8CIE6
Q99PI5
Q6P5E4
Q8BZW8
O35566
Q5NBY9
Q9JJU8
Q8BYW1
Q05144
P10852
E9Q1F5
A0A1Y7VN

Q9QXN3
P60670
A0A0R4J0
Q8BXC0
Q9WTI7
A2AKB9
G3UYV4
P10833
Q8R2M0
Q8CE64
P60122
P12382
Q61087
A2BDX2
Q80XC3
F6WR04
Q8BSK8
P62281
E9Q3C1
E9Q4K7
Q99JF8
Q60898
Q80TL7
Q9CQ62
Q8VDJ3
Q923D5
F6RJV6
P11152
E9QNH6
P70698
A0A1Y7VJ
E9QKR0
Q9Z0R6
F6W7C7
G3UWX1
O55071
Q5FWH2
O35638
Q8CHC4
Q9Z1B5
Q9Z2C4
Q62318
Q8C6B0
Q3V3Q4
E9QAD3

Q9QZE5
Q921S7
Q8R379
Q91WM3
P51881
A0A1B0GT
P54822
Q9CWU3
O54784
Q6PDM1
H9KV15
Q91W96
Q5XFZ0
Q8CGF1
Q9JKB3
Q6ZWY3
E9Q9H3
Q9CPR4
Q80X85
Q8BSQ9
P97390
D3YWF6
A2AB60
O35864
P61750
O88456
A0A494BA
E9QAG4
Q9QXX4
O89051
Q9EQ06
P09055
Q8BIV3
Q9CQW1
Q8N7N5
F6R6F1
Q8R1G6
Q80ZJ8
Q9JK91
Q99PP9
Q8BGC4
Q3UQ44
Q8C2Q7
Q3V3R4
Q9D1A2

Q61830
Q9CZH7
P25911
Q8K1K2
B1Q2W7
Q9Z0G0
Q9R1P1
Q8CD91
Q8BG32
Q810A7
Q9D338
B8JKU8
Q8CI94
Q78PY7
Q99KN1
Q8CJG0
Q3UTQ8
E9QLG3
Q6NY15
Q8C2E7
Q8K2I3
Q06185
E0CX30
Q91Z40
Z4YL78
P32067
Q0P557
D3YWS8
A0A0R4J0[illegible]
P50580
Z4YL66
E9Q3W1
P17426
Q9JL35
P28867
Q7TPE5
P42208
A2AP82
Q8JZR0
Q8K045
Q5DU09
Q8BG48
Q80U95
Q9CVB6
Q9QVP4

Q6NV56
Q61146
O08547
B9EJ54
Q8R2V3
Q99N57
A0A1B0G[illegible]
F8VQJ3
Q9DBC3
A0A494BB[illegible]
Q99L45
Q60793
Q62189
Q91VL8
A0A171EB[illegible]
Q5FWI3
O88545
Q9DC33
E9QPE7
A0A0A6YV[illegible]
Q3ULL6
P70191
F6UP63
P98083
O55222
Q9CQ65
Q3U6K5
Q9DC47
O08759
Q9CPR5
Q6NVF2
Q8VC16
P27790
Q8BK35
A0A494BA[illegible]
A0A140LI5[illegible]
Q80VP1
Q8VBT9
R4H4V1
A2AIM4
Q9D883
D3Z3Q3
Q8CDM8
P48758
E9PV41

Q921J2
Q921I6
Q9JL61
Q3UPV7
Q8R1B8
Q9R111
Q6PGG6
Q61194
Q6P3B9
Q8K224
Q80UG5
G3UZF0
A0A384DV[illegible]
Q6P1H6
F8WHU5
Q6PDY0
Q9ERL9
Q9D0T1
Q6GT24
A0A0R4J1[illegible]
Q3UQI9
I7HIP8
O70494
Q9ERI2
Q9JKC8
A0A0N4SV[illegible]
P54923
Q9ES97
B9EJ86
Q6DVA0
D3Z1J8
Q8BGS2
G3UXT7
Q01853
Q9R1E0
Q5SV80
Q80UU9
Q99L43
Q4VBE4
A0A087W[illegible]
Q5UE59
F8VQC7
Q8CE08
P84096
P68510

A0A0G2JE
Q3UZ39
P62774
Q8K215
Q8C0E2
A0A2R8W
Q4VBE8
Q8K274
Q8VEB3
F8VQ05
P62259
Q8C522
Q9CQV8
O08529
A0A0J9YT
Q80VD1
Q9ER00
Q6DFW4
Q4QRL3
Q8R4E9
Q64521
Q64339
Q922H2
Q8K4F5
Q9EQH2
Q80TM9
Q68FM4
P10284
P48787
Q505N6
Q9WV91
Q8CGA0
E9QKD9
Q6Q899
Q5BLK4
Q9D0F6
O35130
P62748
B1AUE5
Q61193
E9Q3R9
B2RXC2
Q8C7V3
P14602
A2AI52

Q7TQD2
Q8C166
Q5S006
P42232
Q3ULJ0
Q9D0R4
O09100
P70196
F6WIU1
E9Q8M1
Q8CHT0
Q9JJQ0
Q6ZPF4
P14069
Q61081
B9EHX4
Q6PD28
Q8BX57
Q6ZQ38
F7CBX2
Q8BHN5
F6YPZ1
Q6WKZ8
Q8CFW1
O09106
E9QP59
Q810V0
Q91WE6
Q8BQM4
O70310
E9QNN1
Q9D0F4
Q9CX99
Q8C4J7
P63330
H3BJD8
P63037
E9QMR2
Q9ESL4
Q922U1
Q7M6Y0
Q6P8X1
A0A1L1SV
Q61171
P49135

Q61768
Q9JIB4
Q62513
Q8BFY6
B1AV77
G3X983
Q9D8P4
B1AVF5
O70126
Q3TZZ7
Z4YJE9
E9Q448
G5E8G6
Q8BZQ2
Q99LJ6
Q60760
Q9D8T7
Z4YJT3
P97742
P97425
O35205
A0A3B2W
P49138
Q8R2K4
A0A0R4J1
Q91XC9
Q8BGZ4
D3Z5I9
A0A3B2W
A0A0G2JF
P97813
Q9JMD0
O88632
H3BIV5
Q9D4H1
A2AJQ0
Q5SSZ5
Q8R2Q8
P97376
D3Z197
Q91VV4
Q9Z0N2
P08074
O54788
A0A087W

A0A1L1SQ
Q9Z2X8
Q8BSF4
O89112
Q61316
P62960
P36916
Q8BXV2
Q99N92
Q99P88
Q9CZP3
P46467
E9Q411
H3BJD6
E9Q2M9
Q9D952
A0A5F8MI
Q8JZU2
Q8BIR2
Q3UHU8
O09044
Q9CQW2
P62827
Q9DC71
Q3TTA7
A0A140LH
Q9CX56
G5E898
P52633
E9PZM7
Q9WUK4
Q01730
F8VPX2
Q6NVE8
Q8CDV6
Q9EPL8
Q921K9
Q9CR02
A0A1B0GS
Q91VA7
Q61133
A0A0G2JD
E9Q9T1
P97450
Q9EPC1

Q6A028
Q9Z1B3
P14824
Q9JKS5
Q9CXI0
Q8VBZ3
D3YYC3
Q924L1
Q62376
Q8K3A2
A2A8W8
E9QNY8
Q8K0U4
E9PZQ1
Q69Z99
P97371
Q8CCK0
Q925F2
A0A0A0M[illegible]
Q6NZK2
Q505D7
Q61749
P19096
P31938
O35465
H3BKL7
A0A5F8M[illegible]
F8VQK3
Q6A009
A0A0G2JD
E9Q8T2
Q14BI5
Q8BSM6
P09103
P40142
Q9JMA9
A7YQ68
Q9WVJ2
Q8CEC4
Q3U288
P24547
Q0VF55
Q8CE04
Q8VHH5
Q9EQH3

Q9ERS5
Q9QXY6
Q64435
P97363
Q99LH4
P62270
G3UZM9
Q91W50
Q5SWU9
A2ARA0
P24604
E9Q9D8
Q9DCC1
B1B0C7
D3YXZ3
Q9DBG3
Q61074
Q8BND3
P63038
Q9ES52
Q6A0A2
A0A0B4J1[illegible]
Q9D1Q6
Q3U7R1
A0A0U1RN[illegible]
Q8R1I1
P63328
Q3UHJ0
Q9CQ26
E9PYZ4
Q3TBA3
D3YWT0
A2AI89
E9QAI5
P08920
Q8VCI5
P11031
Q8R092
Q61191
A2AJ88
Q3UYV9
G3UY93
P47856
Q8VCX5
O88662

Q8C0V9
P45481
E9Q784
D3Z6G2
Q9DCE5
P80314
Q8CJG1
Q8BH43
P28660
Q9EQP2
Q8VD75
Q8BMS1
F8WJK8
P07356
A2AWA9
Q9D2L9
Q9R190
Q923B1
O08528
A0A1L1SR
Q9ERU3
Q61288
Q62511
Q9R112
P55258
Q8BVT3
Q9Z1M2
A0A0R4J2
Q8K2B3
Q6NZQ2
Q80X44
Q64310
Q5NCI3
E9QN52
Q8R5A3
P09021
A0A0A6YV
Q6NZI9
Q6ZPQ6
Q14AI6
B1ATL6
A0A1B0GF
F8VQ28
P62821
Q60932

A0A338P6
Q99LF7
A0A087W
Q921Y2
A0A140LH
B2RX31
B1AR17
Q5SFM8
Q64105
G5E8R4
Q3TIV9
Q3TJZ6
A2AUC9
D3Z598
P39428
Q9R1C0
A0A0R4J2
Q9Z315
A2AJ72
A2AGH8
E9Q1X8
Q9D517
Q9D4H9
Q571G4
A0A0A0M
Q99M04
Q75N62
Q8K3H0
Q3UVL4
P39429
A2ADY9
Q9DBG9
P01027
A0A338P6
A2RT67
O88685
Q5SQ27
P48678
Q8VC34
Q4VBD2
Q9Z2N8
Q8CI32
Q6NZD2
P57784
Q91VB8

Q60710
Q8BP00
E9Q1M1
P60335
A0A1D5RL
P80316
A0A1W2P
E9Q309
Q91VE0
Q91XA5
O08599
Q8BKE6
P19324
G5E874
A0A140LJ
P14483
Q9DBP5
P99026
E9PWU7
A0A0N4SV
Q62425
Q80X82
E9PUD2
Q80XC6
Q3UYC0
Q8VCD5
Q08EC4
E9Q7G0
A0A0G2JD
Q9CTY5
Q3U0V1
G5E8I8
Q6ZPE2
Q9QZK2
Q3UU43
A0A5K1VV
A0A2I3BS
F8VQC1
Q80VQ0
Q09324
Q9EP69
Q9DCA5
Q8CIW5
Q8K114
Q91WG4

Q8CE33
Q08874
Q1W617
Q8R0X7
Q9QWV9
Q8BFW7
Q5U430
Z4YKC4
O70325
Q8BGN9
Q9D735
P46978
Q5XJY5
P57722
Q8BH64
Q9R0L6
Q91V41
Q99JY0
P60843
A2AST1
Q99LC8
B1ASP2
H3BK68
A3KG36
Q8BFY9
Q31125
G3UX45
P46935
O88351
Q6PIJ4
Q9CXW4
Q921F2
A0A668KL
Q80YW9
Q8BVE3
P42227
Q9QUI0
P70224
Q9DC70
Q921G9
Q8K4Q0
Q8K0E8
G5E902
P62082
Q8CBE3

Q6P542
Q9QZS8
O54774
P59708
Q9R045
Q6P5D8
Q61789
Q6ZQM8
Q9Z1T1
Q9JK48
O35239
G3UZI2
A0A2I3BPI
Q80ZM5
Q4QQP3
Q71FD7
Q8K245
P98078
D3Z2D6
Q3UC82
Q9JJZ6
G5E8T6
Q9DC23
D6RFU9
Q8R3L2
Q8BLQ0
Q9WVM1
F8VPM7
E9Q7F8
Q9QXA5
Q91YU6
Q64737
Q7TPX5
Q3U9N9
Q04690
Q8BK64
F8WHT3
Q3TAP4
Q6A068
Q9EQC0
Q8VCE9
A0A1D5RN
Q80V23
Q6GYP7
Q8VEK0

Q9QXB9
E9PYD6
Q99LF4
O88543
Q3U7K7
E9PUK3
Q99N93
A0A0U1R[illegible]
P47962
Q9Z0E6
Q8VDI1
P62334
F8VPU2
Q923D4
Q9WV32
Q64727
Q921H8
Q8R323
Q3UYU2
Q6ZWZ4
Q9WVA4
A0A5F8M[illegible]
A0A0H2U[illegible]
O35295
F7BJB9
Q3UMU9
Q6P1Y9
Q62181
Q3UK78
G3UZN5
P59017
Q8BT60
D3YXJ0
B2LVG5
Q9CZX0
P40124
A0A1D5R[illegible]
Q8BVV6
Q9CZ13
Q8CJ78
P61982
P62814
P47758
A2AWR3
Q91VW5

Q9CZ57
Q8VHZ7
A0A0H2UH
Q3UJB0
Q3U1J4
Q9D8M4

: **2**. 3xHA IP p value.

Description
WD repeat-containing protein 26 (Fragment) OS=Mus musculus OX=10090 GN=Wdr26 PE=1 SV=1
WD repeat-containing protein 26 OS=Mus musculus OX=10090 GN=Wdr26 PE=1 SV=2
Myosin-9 (Fragment) OS=Mus musculus OX=10090 GN=Myh9 PE=1 SV=1
Muskelin OS=Mus musculus OX=10090 GN=Mkln1 PE=1 SV=1
Armadillo repeat-containing protein 8 OS=Mus musculus OX=10090 GN=Armc8 PE=1 SV=1
Histone H1.3 OS=Mus musculus OX=10090 GN=H1-3 PE=1 SV=2
Ran-binding protein 10 OS=Mus musculus OX=10090 GN=Ranbp10 PE=1 SV=1
E3 ubiquitin-protein transferase MAEA OS=Mus musculus OX=10090 GN=Maea PE=1 SV=1
Ran-binding protein 9 OS=Mus musculus OX=10090 GN=Ranbp9 PE=1 SV=1
E3 ubiquitin-protein ligase RMND5A OS=Mus musculus OX=10090 GN=Rmnd5a PE=1 SV=2
Glucose-induced degradation protein 8 homolog OS=Mus musculus OX=10090 GN=Gid8 PE=1 SV=1
Protein kinase C beta type OS=Mus musculus OX=10090 GN=Prkcb PE=1 SV=4
Ras-related protein Rab-1B OS=Mus musculus OX=10090 GN=Rab1b PE=1 SV=1
Protein yippee-like 5 OS=Mus musculus OX=10090 GN=Ypel5 PE=1 SV=1
TAF15 RNA polymerase II, TATA box binding protein (TBP)-associated factor OS=Mus musculus OX=10090 GN=Ta
Nuclear autoantigenic sperm protein OS=Mus musculus OX=10090 GN=Nasp PE=1 SV=1
Charged multivesicular body protein 5 OS=Mus musculus OX=10090 GN=Chmp5 PE=1 SV=1
2'-5'-oligoadenylate synthase 1A OS=Mus musculus OX=10090 GN=Oas1a PE=1 SV=2
Mothers against decapentaplegic homolog OS=Mus musculus OX=10090 GN=Smad2 PE=1 SV=1
Polypyrimidine tract-binding protein 3 OS=Mus musculus OX=10090 GN=Ptbp3 PE=1 SV=1
Rho GTPase-activating protein 21 OS=Mus musculus OX=10090 GN=Arhgap21 PE=1 SV=1
Adenosylhomocysteinase OS=Mus musculus OX=10090 GN=Ahcy PE=1 SV=3
Sec24-related gene family, member B (S. cerevisiae) OS=Mus musculus OX=10090 GN=Sec24b PE=1 SV=1
CCR4-NOT transcription complex subunit 9 OS=Mus musculus OX=10090 GN=Cnot9 PE=1 SV=1
L-amino-acid oxidase OS=Mus musculus OX=10090 GN=Il4i1 PE=1 SV=1
DDRGK domain-containing protein 1 OS=Mus musculus OX=10090 GN=Ddrgk1 PE=1 SV=2
R3H and coiled-coil domain-containing protein 1 OS=Mus musculus OX=10090 GN=R3hcc1 PE=1 SV=3
[Histone H3]-lysine(4) N-trimethyltransferase (Fragment) OS=Mus musculus OX=10090 GN=Kmt2b PE=1 SV=1
Complement C1q subcomponent subunit A OS=Mus musculus OX=10090 GN=C1qa PE=1 SV=2
Tripeptidyl-peptidase 2 OS=Mus musculus OX=10090 GN=Tpp2 PE=1 SV=3
Transmembrane protein with metallophosphoesterase domain OS=Mus musculus OX=10090 GN=Tmppe PE=1 S\
F-BAR and double SH3 domains protein 1 OS=Mus musculus OX=10090 GN=Fchsd1 PE=1 SV=1
Bromodomain-containing protein 3 (Fragment) OS=Mus musculus OX=10090 GN=Brd3 PE=1 SV=1
39S ribosomal protein L47, mitochondrial OS=Mus musculus OX=10090 GN=Mrpl47 PE=1 SV=2
Serine/threonine-protein kinase SMG1 OS=Mus musculus OX=10090 GN=Smg1 PE=1 SV=3
RIPOR family member 3 OS=Mus musculus OX=10090 GN=Ripor3 PE=1 SV=1
Macrophage galactose N-acetyl-galactosamine-specific lectin 2 (Fragment) OS=Mus musculus OX=10090 GN=M
Multifunctional procollagen lysine hydroxylase and glycosyltransferase LH3 OS=Mus musculus OX=10090 GN=Plo
Actin-histidine N-methyltransferase OS=Mus musculus OX=10090 GN=Setd3 PE=1 SV=1
RalBP1-associated Eps domain-containing protein 2 OS=Mus musculus OX=10090 GN=Reps2 PE=1 SV=1
Pre-B-cell leukemia transcription factor-interacting protein 1 OS=Mus musculus OX=10090 GN=Pbxip1 PE=1 SV=:
Neurogranin OS=Mus musculus OX=10090 GN=Nrgn PE=1 SV=1

Syndetin OS=Mus musculus OX=10090 GN=Vps50 PE=1 SV=2
AF4/FMR2 family member 3 OS=Mus musculus OX=10090 GN=Aff3 PE=1 SV=2
Predicted gene 11444 (Fragment) OS=Mus musculus OX=10090 GN=Gm11444 PE=4 SV=1
Translocator protein OS=Mus musculus OX=10090 GN=Tspo PE=1 SV=1
Steroid hormone receptor ERR1 OS=Mus musculus OX=10090 GN=Esrra PE=1 SV=1
Pyruvate dehydrogenase E1 component subunit beta, mitochondrial OS=Mus musculus OX=10090 GN=Pdhb PE=1
Importin-11 OS=Mus musculus OX=10090 GN=Ipo11 PE=1 SV=1
Rab GTPase-activating protein 1-like (Fragment) OS=Mus musculus OX=10090 GN=Rabgap1l PE=1 SV=1
Zinc finger protein 654 OS=Mus musculus OX=10090 GN=Znf654 PE=1 SV=2
Peroxisomal leader peptide-processing protease (Fragment) OS=Mus musculus OX=10090 GN=Tysnd1 PE=4 SV=[illegible]
Malate dehydrogenase, cytoplasmic OS=Mus musculus OX=10090 GN=Mdh1 PE=1 SV=3
Ubiquitin thioesterase OTU1 OS=Mus musculus OX=10090 GN=Yod1 PE=1 SV=1
Rab-like protein 2A OS=Mus musculus OX=10090 GN=Rabl2 PE=1 SV=1
Integrator complex subunit 5 OS=Mus musculus OX=10090 GN=Ints5 PE=2 SV=1
Epsin-3 OS=Mus musculus OX=10090 GN=Epn3 PE=1 SV=1
Plexin-B3 OS=Mus musculus OX=10090 GN=Plxnb3 PE=1 SV=2
Zinc finger CCCH domain-containing protein 4 OS=Mus musculus OX=10090 GN=Zc3h4 PE=1 SV=1
Synaptojanin-2-binding protein OS=Mus musculus OX=10090 GN=Synj2bp PE=1 SV=1
Lactadherin OS=Mus musculus OX=10090 GN=Mfge8 PE=1 SV=3
Guanine nucleotide exchange factor MSS4 OS=Mus musculus OX=10090 GN=Rabif PE=1 SV=1
ABI family member 3-binding protein OS=Mus musculus OX=10090 GN=Abi3bp PE=1 SV=1
Murinoglobulin-1 OS=Mus musculus OX=10090 GN=Mug1 PE=1 SV=3
Microfibril-associated glycoprotein 1 OS=Mus musculus OX=10090 GN=Mfap2 PE=1 SV=1
Ras-related protein Rab-11B OS=Mus musculus OX=10090 GN=Rab11b PE=1 SV=3
Solute carrier family 35 member E1 OS=Mus musculus OX=10090 GN=Slc35e1 PE=1 SV=2
Endophilin-B2 OS=Mus musculus OX=10090 GN=Sh3glb2 PE=1 SV=1
Ran-binding protein 3 OS=Mus musculus OX=10090 GN=Ranbp3 PE=1 SV=2
Ectonucleoside triphosphate diphosphohydrolase 1 OS=Mus musculus OX=10090 GN=Entpd1 PE=1 SV=1
Serine protease hepsin OS=Mus musculus OX=10090 GN=Hpn PE=1 SV=3
Nuclear receptor corepressor 1 OS=Mus musculus OX=10090 GN=Ncor1 PE=1 SV=1
Zinc finger protein 410 OS=Mus musculus OX=10090 GN=Znf410 PE=2 SV=2
GATOR complex protein WDR24 OS=Mus musculus OX=10090 GN=Wdr24 PE=1 SV=1
Transcription factor E4F1 (Fragment) OS=Mus musculus OX=10090 GN=E4f1 PE=1 SV=1
Zinc finger and BTB domain-containing protein 41 (Fragment) OS=Mus musculus OX=10090 GN=Zbtb41 PE=4 SV
3-alpha-hydroxysteroid dehydrogenase type 1 OS=Mus musculus OX=10090 GN=Akr1c14 PE=1 SV=1
Glutamine amidotransferase-like class 1 domain-containing protein 3A, mitochondrial OS=Mus musculus OX=10[illegible]
Abnormal spindle-like microcephaly-associated protein homolog OS=Mus musculus OX=10090 GN=Aspm PE=1 S[illegible]
Chromodomain-helicase-DNA-binding protein 1-like OS=Mus musculus OX=10090 GN=Chd1l PE=1 SV=1
Meiosis-specific nuclear structural protein 1 (Fragment) OS=Mus musculus OX=10090 GN=Mns1 PE=1 SV=1
AT-rich interactive domain-containing protein 3C OS=Mus musculus OX=10090 GN=Arid3c PE=2 SV=1
Phosphomevalonate kinase OS=Mus musculus OX=10090 GN=Pmvk PE=1 SV=1
Eukaryotic translation initiation factor 6 (Fragment) OS=Mus musculus OX=10090 GN=Eif6 PE=1 SV=1
Tetratricopeptide repeat protein 28 OS=Mus musculus OX=10090 GN=Ttc28 PE=1 SV=3
Valine--tRNA ligase, mitochondrial OS=Mus musculus OX=10090 GN=Vars2 PE=1 SV=2
Centrosomal protein of 41 kDa OS=Mus musculus OX=10090 GN=Cep41 PE=1 SV=1

Ras-related protein Rab-34 OS=Mus musculus OX=10090 GN=Rab34 PE=4 SV=1
NTPase KAP family P-loop domain-containing protein 1 OS=Mus musculus OX=10090 GN=Nkpd1 PE=4 SV=1
Calumenin (Fragment) OS=Mus musculus OX=10090 GN=Calu PE=1 SV=2
RAS p21 protein activator 1 (Fragment) OS=Mus musculus OX=10090 GN=Rasa1 PE=1 SV=1
DBF4-type zinc finger-containing protein 2 homolog (Fragment) OS=Mus musculus OX=10090 GN=Zdbf2 PE=1 SV
Polycomb group RING finger protein 6 OS=Mus musculus OX=10090 GN=Pcgf6 PE=1 SV=1
Pogo transposable element with ZNF domain OS=Mus musculus OX=10090 GN=Pogz PE=1 SV=1
RAS, guanyl-releasing protein 3 OS=Mus musculus OX=10090 GN=Rasgrp3 PE=1 SV=1
Ubiquitin carboxyl-terminal hydrolase 46 OS=Mus musculus OX=10090 GN=Usp46 PE=1 SV=1
Protein Wnt-2 OS=Mus musculus OX=10090 GN=Wnt2 PE=2 SV=2
Pre-mRNA-splicing factor 38B OS=Mus musculus OX=10090 GN=Prpf38b PE=1 SV=1
Melanoma inhibitory activity protein 2 OS=Mus musculus OX=10090 GN=Mia2 PE=1 SV=1
Phosphoglucomutase-2 (Fragment) OS=Mus musculus OX=10090 GN=Pgm2 PE=1 SV=1
PHD finger protein 10 OS=Mus musculus OX=10090 GN=Phf10 PE=1 SV=4
DNA-directed RNA polymerases I, II, and III subunit RPABC3 (Fragment) OS=Mus musculus OX=10090 GN=Polr2h
KAT8 regulatory NSL complex subunit 1 OS=Mus musculus OX=10090 GN=Kansl1 PE=1 SV=1
E3 ubiquitin-protein ligase RNF8 OS=Mus musculus OX=10090 GN=Gm28043 PE=3 SV=1
Transcription factor SOX-17 OS=Mus musculus OX=10090 GN=Sox17 PE=1 SV=1
Nuclear respiratory factor 1 OS=Mus musculus OX=10090 GN=Nrf1 PE=1 SV=1
DNA-directed RNA polymerase II subunit RPB3 OS=Mus musculus OX=10090 GN=Polr2c PE=1 SV=2
Pyruvate carboxylase OS=Mus musculus OX=10090 GN=Pcx PE=1 SV=1
Bromodomain-containing protein 2 OS=Mus musculus OX=10090 GN=Brd2 PE=1 SV=1
Tight junction protein ZO-1 OS=Mus musculus OX=10090 GN=Tjp1 PE=1 SV=1
Selenoprotein K OS=Mus musculus OX=10090 GN=Selenok PE=1 SV=3
Programmed cell death protein 4 OS=Mus musculus OX=10090 GN=Pdcd4 PE=1 SV=1
Ribosome maturation protein SBDS OS=Mus musculus OX=10090 GN=Sbds PE=1 SV=4
DNA-directed RNA polymerases I, II, and III subunit RPABC5 OS=Mus musculus OX=10090 GN=Polr2l PE=3 SV=1
Glucose-induced degradation protein 4 homolog OS=Mus musculus OX=10090 GN=Gid4 PE=1 SV=1
Eukaryotic initiation factor 4A-III OS=Mus musculus OX=10090 GN=Eif4a3 PE=1 SV=3
BMP-2-inducible protein kinase OS=Mus musculus OX=10090 GN=Bmp2k PE=1 SV=1
Alpha-centractin OS=Mus musculus OX=10090 GN=Actr1a PE=1 SV=1
Chromatin target of PRMT1 protein OS=Mus musculus OX=10090 GN=Chtop PE=1 SV=1
Histone-lysine N-methyltransferase SETDB2 OS=Mus musculus OX=10090 GN=Setdb2 PE=1 SV=1
Beta-centractin OS=Mus musculus OX=10090 GN=Actr1b PE=1 SV=1
Peroxisomal leader peptide-processing protease OS=Mus musculus OX=10090 GN=Tysnd1 PE=1 SV=1
Ankyrin repeat domain-containing protein SOWAHC OS=Mus musculus OX=10090 GN=Sowahc PE=1 SV=2
TATA-box-binding protein-associated factor 4 OS=Mus musculus OX=10090 GN=Taf4 PE=1 SV=1
THAP domain-containing protein 3 OS=Mus musculus OX=10090 GN=Thap3 PE=2 SV=1
MOB-like protein phocein OS=Mus musculus OX=10090 GN=Mob4 PE=1 SV=1
RELT-like protein 1 OS=Mus musculus OX=10090 GN=Rell1 PE=2 SV=2
Serine/threonine-protein kinase 10 OS=Mus musculus OX=10090 GN=Stk10 PE=1 SV=2
ATP-binding cassette sub-family D member 1 OS=Mus musculus OX=10090 GN=Abcd1 PE=1 SV=1
Zinc finger protein 692 OS=Mus musculus OX=10090 GN=Znf692 PE=2 SV=1
Retinoblastoma-associated protein OS=Mus musculus OX=10090 GN=Rb1 PE=1 SV=2
Ecotropic viral integration site 5-like OS=Mus musculus OX=10090 GN=Evi5l PE=1 SV=2

Phenylalanine--tRNA ligase, mitochondrial OS=Mus musculus OX=10090 GN=Fars2 PE=1 SV=1
Transcription factor BTF3 homolog 4 OS=Mus musculus OX=10090 GN=Btf3l4 PE=1 SV=1
Exportin-4 OS=Mus musculus OX=10090 GN=Xpo4 PE=1 SV=2
Receptor expression-enhancing protein 4 OS=Mus musculus OX=10090 GN=Reep4 PE=1 SV=1
Integrin alpha-3 OS=Mus musculus OX=10090 GN=Itga3 PE=1 SV=1
Potassium channel tetramerisation domain-containing 18 OS=Mus musculus OX=10090 GN=Kctd18 PE=1 SV=1
MHC class II regulatory factor RFX1 OS=Mus musculus OX=10090 GN=Rfx1 PE=1 SV=1
Peroxisome proliferator-activated receptor gamma OS=Mus musculus OX=10090 GN=Pparg PE=1 SV=1
Bromodomain-containing protein 4 OS=Mus musculus OX=10090 GN=Brd4 PE=1 SV=2
DNA-directed RNA polymerase I subunit RPA49 (Fragment) OS=Mus musculus OX=10090 GN=Polr1e PE=1 SV=1
Novel KRAB box and zinc finger, C2H2 type domain containing protein OS=Mus musculus OX=10090 GN=Zfp973 [illegible]
Microtubule-actin cross-linking factor 1 (Fragment) OS=Mus musculus OX=10090 GN=Macf1 PE=1 SV=1
DNA-directed RNA polymerase II subunit RPB2 OS=Mus musculus OX=10090 GN=Polr2b PE=1 SV=2
Ryanodine receptor 3 OS=Mus musculus OX=10090 GN=Ryr3 PE=1 SV=1
Dedicator of cytokinesis protein 6 OS=Mus musculus OX=10090 GN=Dock6 PE=1 SV=1
Chitinase-like protein 3 OS=Mus musculus OX=10090 GN=Chil3 PE=1 SV=2
Alkylglycerone-phosphate synthase OS=Mus musculus OX=10090 GN=Agps PE=1 SV=1
Mitochondrial coenzyme A transporter SLC25A42 OS=Mus musculus OX=10090 GN=Slc25a42 PE=1 SV=1
E3 ubiquitin protein ligase OS=Mus musculus OX=10090 GN=Rnf40 PE=1 SV=1
Erlin-1 OS=Mus musculus OX=10090 GN=Erlin1 PE=1 SV=2
Cysteine-rich protein 1 OS=Mus musculus OX=10090 GN=Crip1 PE=1 SV=2
DNA-directed RNA polymerase subunit OS=Mus musculus OX=10090 GN=Polr2a PE=1 SV=1
Myosin-3 OS=Mus musculus OX=10090 GN=Myh3 PE=2 SV=2
PR domain-containing protein 11 OS=Mus musculus OX=10090 GN=Prdm11 PE=1 SV=1
Guanine nucleotide-binding protein G(o) subunit alpha OS=Mus musculus OX=10090 GN=Gnao1 PE=1 SV=3
Nuclear factor of activated T-cells, cytoplasmic 2 OS=Mus musculus OX=10090 GN=Nfatc2 PE=1 SV=3
Coiled-coil domain-containing protein 85A OS=Mus musculus OX=10090 GN=Ccdc85a PE=1 SV=1
Leucine-rich repeat-containing protein 75A OS=Mus musculus OX=10090 GN=Lrrc75a PE=1 SV=1
Dynactin subunit 6 OS=Mus musculus OX=10090 GN=Dctn6 PE=1 SV=1
Vesicle-trafficking protein SEC22a OS=Mus musculus OX=10090 GN=Sec22a PE=2 SV=1
SWI/SNF-related matrix-associated actin-dependent regulator of chromatin subfamily B member 1 OS=Mus mu[illegible]
THO complex subunit 2 OS=Mus musculus OX=10090 GN=Thoc2 PE=1 SV=1
Serum response factor-binding protein 1 OS=Mus musculus OX=10090 GN=Srfbp1 PE=1 SV=1
Dynactin subunit 2 OS=Mus musculus OX=10090 GN=Dctn2 PE=1 SV=3
Transcription factor SOX-6 OS=Mus musculus OX=10090 GN=Sox6 PE=1 SV=2
Zinc finger CCHC domain-containing protein 8 OS=Mus musculus OX=10090 GN=Zcchc8 PE=1 SV=3
DNA repair protein complementing XP-A cells homolog OS=Mus musculus OX=10090 GN=Xpa PE=1 SV=2
Protein ITPRID2 OS=Mus musculus OX=10090 GN=Itprid2 PE=1 SV=1
Ras-responsive element-binding protein 1 OS=Mus musculus OX=10090 GN=Rreb1 PE=1 SV=2
Alpha-mannosidase 2C1 OS=Mus musculus OX=10090 GN=Man2c1 PE=1 SV=1
Serine/threonine-protein kinase TAO1 OS=Mus musculus OX=10090 GN=Taok1 PE=1 SV=1
40S ribosomal protein S10 OS=Mus musculus OX=10090 GN=Rps10 PE=1 SV=1
Caveolin (Fragment) OS=Mus musculus OX=10090 GN=Cav1 PE=1 SV=1
Membrane-bound transcription factor site-1 protease OS=Mus musculus OX=10090 GN=Mbtps1 PE=1 SV=1
Peroxisomal membrane protein PEX14 OS=Mus musculus OX=10090 GN=Pex14 PE=1 SV=1

Cyclin-dependent kinase 14 OS=Mus musculus OX=10090 GN=Cdk14 PE=1 SV=1
Myosin regulatory light chain 2, skeletal muscle isoform OS=Mus musculus OX=10090 GN=Mylpf PE=1 SV=3
Lysine-rich nucleolar protein 1 OS=Mus musculus OX=10090 GN=Knop1 PE=1 SV=2
Charged multivesicular body protein 4b OS=Mus musculus OX=10090 GN=Chmp4b PE=1 SV=2
Lymphoid enhancer-binding factor 1 OS=Mus musculus OX=10090 GN=Lef1 PE=1 SV=1
DNA-directed RNA polymerase II subunit RPB4 OS=Mus musculus OX=10090 GN=Polr2d PE=1 SV=2
DNA-directed RNA polymerases I, II, and III subunit RPABC1 OS=Mus musculus OX=10090 GN=Polr2e PE=1 SV=1
THO complex subunit 6 homolog OS=Mus musculus OX=10090 GN=Thoc6 PE=1 SV=1
Ubiquitin-conjugating enzyme E2 N OS=Mus musculus OX=10090 GN=Ube2n PE=1 SV=1
TIP41-like protein OS=Mus musculus OX=10090 GN=Tiprl PE=1 SV=1
Peroxidasin homolog OS=Mus musculus OX=10090 GN=Pxdn PE=1 SV=2
pre-mRNA 3' end-processing protein WDR33 OS=Mus musculus OX=10090 GN=Wdr33 PE=1 SV=1
Caveolin-2 OS=Mus musculus OX=10090 GN=Cav2 PE=1 SV=1
FERM and PDZ domain-containing 3 OS=Mus musculus OX=10090 GN=Frmpd3 PE=1 SV=1
Protein mono-ADP-ribosyltransferase PARP12 OS=Mus musculus OX=10090 GN=Parp12 PE=1 SV=3
Cytosolic carboxypeptidase 1 (Fragment) OS=Mus musculus OX=10090 GN=Agtpbp1 PE=1 SV=2
Rho family-interacting cell polarization regulator 2 OS=Mus musculus OX=10090 GN=Ripor2 PE=1 SV=1
DISP complex protein LRCH3 OS=Mus musculus OX=10090 GN=Lrch3 PE=1 SV=1
Zinc finger CCCH domain-containing protein 15 OS=Mus musculus OX=10090 GN=Zc3h15 PE=1 SV=2
Zinc finger protein 148 OS=Mus musculus OX=10090 GN=Zfp148 PE=1 SV=1
Protein phosphatase methylesterase 1 OS=Mus musculus OX=10090 GN=Ppme1 PE=1 SV=5
Transcription elongation factor 1 homolog OS=Mus musculus OX=10090 GN=Elof1 PE=1 SV=1
TAF5-like RNA polymerase II p300/CBP-associated factor-associated factor 65 kDa subunit 5L OS=Mus musculus
Eukaryotic translation initiation factor 4C OS=Mus musculus OX=10090 GN=Eif1ad11 PE=1 SV=1
Dystrobrevin beta OS=Mus musculus OX=10090 GN=Dtnb PE=1 SV=3
Protein max OS=Mus musculus OX=10090 GN=Max PE=1 SV=2
Nitric oxide-associated protein 1 OS=Mus musculus OX=10090 GN=Noa1 PE=1 SV=1
MICOS complex subunit MIC60 OS=Mus musculus OX=10090 GN=Immt PE=1 SV=1
Coronin OS=Mus musculus OX=10090 GN=Coro2a PE=1 SV=1
Polymerase delta-interacting protein 3 OS=Mus musculus OX=10090 GN=Poldip3 PE=1 SV=1
Charged multivesicular body protein 2a OS=Mus musculus OX=10090 GN=Chmp2a PE=1 SV=1
ATP synthase subunit O, mitochondrial OS=Mus musculus OX=10090 GN=Atp5po PE=1 SV=1
Immunoglobulin heavy variable V9-3 (Fragment) OS=Mus musculus OX=10090 GN=Ighv9-3 PE=1 SV=1
Enhancer of rudimentary homolog OS=Mus musculus OX=10090 GN=Erh PE=1 SV=1
Myosin-7 OS=Mus musculus OX=10090 GN=Myh7 PE=2 SV=1
D-aspartate oxidase OS=Mus musculus OX=10090 GN=Ddo PE=1 SV=1
Programmed cell death protein 10 OS=Mus musculus OX=10090 GN=Pdcd10 PE=1 SV=1
Dynein, axonemal, heavy chain 10 OS=Mus musculus OX=10090 GN=Dnah10 PE=1 SV=2
High affinity immunoglobulin gamma Fc receptor I OS=Mus musculus OX=10090 GN=Fcgr1 PE=1 SV=1
Mitogen-activated protein kinase kinase kinase kinase 1 OS=Mus musculus OX=10090 GN=Map4k1 PE=1 SV=1
Ubiquitin-like protein 5 (Fragment) OS=Mus musculus OX=10090 GN=Ubl5 PE=1 SV=1
Oncostatin-M OS=Mus musculus OX=10090 GN=Osm PE=1 SV=1
RNA-binding protein 7 OS=Mus musculus OX=10090 GN=Rbm7 PE=1 SV=1
Transcriptional regulator ERG OS=Mus musculus OX=10090 GN=Erg PE=1 SV=1
Retinal guanylyl cyclase 1 OS=Mus musculus OX=10090 GN=Gucy2e PE=1 SV=2

Caveolin-1 OS=Mus musculus OX=10090 GN=Cav1 PE=1 SV=1
Zinc finger protein 384 OS=Mus musculus OX=10090 GN=Zfp384 PE=1 SV=1
Protein ZNRD2 OS=Mus musculus OX=10090 GN=Znrd2 PE=1 SV=1
Zinc finger protein 330 OS=Mus musculus OX=10090 GN=Znf330 PE=1 SV=1
Gasdermin-D OS=Mus musculus OX=10090 GN=Gsdmd PE=1 SV=1
Serine/threonine-protein kinase D1 OS=Mus musculus OX=10090 GN=Prkd1 PE=1 SV=2
Histone deacetylase complex subunit SAP130 OS=Mus musculus OX=10090 GN=Sap130 PE=1 SV=1
Terminal uridylyltransferase 4 OS=Mus musculus OX=10090 GN=Tut4 PE=1 SV=2
E3 ubiquitin-protein ligase MIB2 OS=Mus musculus OX=10090 GN=Mib2 PE=1 SV=2
Trinucleotide repeat-containing gene 18 protein OS=Mus musculus OX=10090 GN=Tnrc18 PE=1 SV=2
Protoporphyrinogen oxidase (Fragment) OS=Mus musculus OX=10090 GN=Ppox PE=1 SV=1
Nascent polypeptide-associated complex subunit alpha, muscle-specific form OS=Mus musculus OX=10090 GN=I
Cadherin-5 OS=Mus musculus OX=10090 GN=Cdh5 PE=1 SV=2
Protein DEK OS=Mus musculus OX=10090 GN=Dek PE=1 SV=1
Small nuclear ribonucleoprotein E OS=Mus musculus OX=10090 GN=Snrpe PE=1 SV=1
Nucleophosmin OS=Mus musculus OX=10090 GN=Npm1 PE=1 SV=1
Regucalcin OS=Mus musculus OX=10090 GN=Rgn PE=1 SV=1
Mediator of RNA polymerase II transcription subunit 30 OS=Mus musculus OX=10090 GN=Med30 PE=1 SV=1
Ribonuclease P protein subunit p30 OS=Mus musculus OX=10090 GN=Rpp30 PE=1 SV=1
Zinc finger HIT domain-containing protein 2 OS=Mus musculus OX=10090 GN=Znhit2 PE=2 SV=2
SH2 domain-containing adapter protein E OS=Mus musculus OX=10090 GN=She PE=1 SV=1
Fatty acyl-CoA reductase 1 OS=Mus musculus OX=10090 GN=Far1 PE=1 SV=1
E3 ubiquitin-protein ligase BRE1A OS=Mus musculus OX=10090 GN=Rnf20 PE=1 SV=2
Myosin light polypeptide 6 OS=Mus musculus OX=10090 GN=Myl6 PE=1 SV=1
Protein FAM43A OS=Mus musculus OX=10090 GN=Fam43a PE=2 SV=1
Choline dehydrogenase, mitochondrial OS=Mus musculus OX=10090 GN=Chdh PE=1 SV=1
Cyclic AMP-dependent transcription factor ATF-1 OS=Mus musculus OX=10090 GN=Atf1 PE=1 SV=1
Thrombospondin-3 OS=Mus musculus OX=10090 GN=Thbs3 PE=1 SV=1
Mediator of RNA polymerase II transcription subunit 24 OS=Mus musculus OX=10090 GN=Med24 PE=1 SV=1
Neuronal vesicle-trafficking-associated protein 1 (Fragment) OS=Mus musculus OX=10090 GN=Nsg1 PE=3 SV=1
Vesicle-associated membrane protein-associated protein A OS=Mus musculus OX=10090 GN=Vapa PE=1 SV=2
Vacuolar protein sorting-associated protein 28 homolog OS=Mus musculus OX=10090 GN=Vps28 PE=1 SV=1
General transcription factor IIE subunit 2 OS=Mus musculus OX=10090 GN=Gtf2e2 PE=1 SV=2
Serine/arginine-rich-splicing factor 4 OS=Mus musculus OX=10090 GN=Srsf4 PE=1 SV=1
CXXC-type zinc finger protein 5 OS=Mus musculus OX=10090 GN=Cxxc5 PE=2 SV=1
Zinc finger protein 580 OS=Mus musculus OX=10090 GN=Zfp580 PE=4 SV=1
Zinc finger MYND domain-containing protein 11 OS=Mus musculus OX=10090 GN=Zmynd11 PE=1 SV=1
RING-type E3 ubiquitin transferase (Fragment) OS=Mus musculus OX=10090 GN=Ttc3 PE=1 SV=1
RNA-binding protein 8A OS=Mus musculus OX=10090 GN=Rbm8a PE=1 SV=1
RING-type E3 ubiquitin transferase OS=Mus musculus OX=10090 GN=Cbll1 PE=1 SV=1
NSFL1 cofactor p47 OS=Mus musculus OX=10090 GN=Nsfl1c PE=1 SV=1
Dolichol-phosphate mannosyltransferase subunit 3 OS=Mus musculus OX=10090 GN=Dpm3 PE=1 SV=1
Immunoglobulin kappa chain variable 2-137 (Fragment) OS=Mus musculus OX=10090 GN=Igkv2-137 PE=4 SV=1
Eukaryotic translation initiation factor 3 subunit H OS=Mus musculus OX=10090 GN=Eif3h PE=1 SV=1
Condensin-2 complex subunit D3 OS=Mus musculus OX=10090 GN=Ncapd3 PE=1 SV=1

Protein FAM133B OS=Mus musculus OX=10090 GN=Fam133b PE=1 SV=1
TATA box-binding protein-associated factor RNA polymerase I subunit C OS=Mus musculus OX=10090 GN=Taf1c
Rho GTPase-activating protein 30 OS=Mus musculus OX=10090 GN=Arhgap30 PE=1 SV=3
Zinc fingers and homeoboxes protein 1 OS=Mus musculus OX=10090 GN=Zhx1 PE=1 SV=2
Protein strawberry notch homolog 2 (Fragment) OS=Mus musculus OX=10090 GN=Sbno2 PE=1 SV=1
Cilia- and flagella-associated protein 157 OS=Mus musculus OX=10090 GN=Cfap157 PE=1 SV=1
Non-POU domain-containing octamer-binding protein OS=Mus musculus OX=10090 GN=Nono PE=1 SV=3
Cullin-2 (Fragment) OS=Mus musculus OX=10090 GN=Cul2 PE=1 SV=1
Prohibitin OS=Mus musculus OX=10090 GN=Phb PE=1 SV=1
Xaa-Pro aminopeptidase 3 OS=Mus musculus OX=10090 GN=Xpnpep3 PE=1 SV=1
Cerebellar degeneration-related protein 2-like OS=Mus musculus OX=10090 GN=Cdr2l PE=1 SV=1
Phospholipase C, beta 4 (Fragment) OS=Mus musculus OX=10090 GN=Plcb4 PE=1 SV=1
F-box/LRR-repeat protein 3 OS=Mus musculus OX=10090 GN=Fbxl3 PE=1 SV=1
E3 ubiquitin-protein transferase RMND5B OS=Mus musculus OX=10090 GN=Rmnd5b PE=2 SV=1
DIA3 OS=Mus musculus OX=10090 GN=Diaph2 PE=1 SV=1
Polyadenylate-binding protein OS=Mus musculus OX=10090 GN=Pabpc4 PE=1 SV=1
(E3-independent) E2 ubiquitin-conjugating enzyme UBE2O OS=Mus musculus OX=10090 GN=Ube2o PE=1 SV=3
Charged multivesicular body protein 4c OS=Mus musculus OX=10090 GN=Chmp4c PE=2 SV=1
Rho-related GTP-binding protein RhoD OS=Mus musculus OX=10090 GN=Rhod PE=1 SV=1
Plectin OS=Mus musculus OX=10090 GN=Plec PE=1 SV=3
Fc fragment of IgG-binding protein OS=Mus musculus OX=10090 GN=Fcgbp PE=1 SV=1
Cohesin subunit SA-1 OS=Mus musculus OX=10090 GN=Stag1 PE=1 SV=1
Sorting nexin-21 OS=Mus musculus OX=10090 GN=Snx21 PE=1 SV=1
Glycosyltransferase-like domain-containing protein 1 (Fragment) OS=Mus musculus OX=10090 GN=Gtdc1 PE=1 S
Zinc finger protein Aiolos OS=Mus musculus OX=10090 GN=Ikzf3 PE=1 SV=2
Zinc finger protein 811 OS=Mus musculus OX=10090 GN=Zfp811 PE=4 SV=1
Guanylate-binding protein 5 OS=Mus musculus OX=10090 GN=Gbp5 PE=1 SV=1
Structure-specific endonuclease subunit SLX1 OS=Mus musculus OX=10090 GN=Slx1b PE=2 SV=1
cAMP-responsive element-binding protein-like 2 OS=Mus musculus OX=10090 GN=Crebl2 PE=1 SV=1
Kinase D-interacting substrate 220 OS=Mus musculus OX=10090 GN=Kidins220 PE=1 SV=1
Xenotropic and polytropic retrovirus receptor 1 OS=Mus musculus OX=10090 GN=Xpr1 PE=1 SV=1
Histone acetyltransferase OS=Mus musculus OX=10090 GN=Kat2a PE=1 SV=1
Myc target protein 1 OS=Mus musculus OX=10090 GN=Myct1 PE=1 SV=1
Heat shock protein beta-8 OS=Mus musculus OX=10090 GN=Hspb8 PE=1 SV=1
Kelch-like protein 36 OS=Mus musculus OX=10090 GN=Klhl36 PE=1 SV=1
Serine/threonine-protein kinase TAO2 (Fragment) OS=Mus musculus OX=10090 GN=Taok2 PE=1 SV=1
Histone deacetylase OS=Mus musculus OX=10090 GN=Hdac9 PE=1 SV=1
Lysine-specific demethylase PHF2 OS=Mus musculus OX=10090 GN=Phf2 PE=1 SV=2
Plasminogen activator inhibitor 1 RNA-binding protein OS=Mus musculus OX=10090 GN=Serbp1 PE=1 SV=2
Exosome RNA helicase MTR4 OS=Mus musculus OX=10090 GN=Mtrex PE=1 SV=1
Zinc finger and SCAN domain-containing protein 21 OS=Mus musculus OX=10090 GN=Zscan21 PE=2 SV=2
Phosphofurin acidic cluster sorting protein 2 OS=Mus musculus OX=10090 GN=Pacs2 PE=1 SV=1
Chromobox protein homolog 6 OS=Mus musculus OX=10090 GN=Cbx6 PE=1 SV=1
Cysteine-rich protein 2 OS=Mus musculus OX=10090 GN=Crip2 PE=1 SV=1
Enhancer of mRNA-decapping protein 4 OS=Mus musculus OX=10090 GN=Edc4 PE=1 SV=1

Paired amphipathic helix protein Sin3a OS=Mus musculus OX=10090 GN=Sin3a PE=1 SV=3
Eukaryotic translation initiation factor 3 subunit I OS=Mus musculus OX=10090 GN=Eif3i PE=1 SV=1
Ras association domain-containing protein 2 OS=Mus musculus OX=10090 GN=Rassf2 PE=1 SV=1
Zinc finger protein 958 OS=Mus musculus OX=10090 GN=Zfp958 PE=1 SV=1
Transcription factor EB OS=Mus musculus OX=10090 GN=Tfeb PE=1 SV=2
Clathrin light chain OS=Mus musculus OX=10090 GN=Clta PE=1 SV=1
Actin-related protein 10 OS=Mus musculus OX=10090 GN=Actr10 PE=1 SV=2
Amyloid protein-binding protein 2 OS=Mus musculus OX=10090 GN=Appbp2 PE=2 SV=1
Corneodesmosin OS=Mus musculus OX=10090 GN=Cdsn PE=2 SV=2
Dynactin subunit 4 OS=Mus musculus OX=10090 GN=Dctn4 PE=1 SV=1
ATP synthase subunit gamma OS=Mus musculus OX=10090 GN=Atp5c1 PE=1 SV=1
Transcription factor BTF3 OS=Mus musculus OX=10090 GN=Btf3 PE=1 SV=3
Serine protease HTRA2, mitochondrial OS=Mus musculus OX=10090 GN=Htra2 PE=1 SV=1
THUMP domain-containing protein 3 OS=Mus musculus OX=10090 GN=Thumpd3 PE=1 SV=1
Pre-mRNA-processing factor 19 OS=Mus musculus OX=10090 GN=Prpf19 PE=1 SV=1
Eukaryotic translation initiation factor 3 subunit A OS=Mus musculus OX=10090 GN=Eif3a PE=1 SV=5
Spindlin-1 OS=Mus musculus OX=10090 GN=Spin1 PE=1 SV=2
High mobility group protein B2 OS=Mus musculus OX=10090 GN=Hmgb2 PE=1 SV=3
RIKEN cDNA 9130023H24 gene OS=Mus musculus OX=10090 GN=9130023H24Rik PE=2 SV=1
General transcription factor 3C polypeptide 5 OS=Mus musculus OX=10090 GN=Gtf3c5 PE=2 SV=2
Zinc finger and BTB domain-containing protein 46 OS=Mus musculus OX=10090 GN=Zbtb46 PE=1 SV=2
NADH dehydrogenase [ubiquinone] iron-sulfur protein 2, mitochondrial OS=Mus musculus OX=10090 GN=Ndufs2
Myosin, heavy chain 15 OS=Mus musculus OX=10090 GN=Myh15 PE=1 SV=1
Major prion protein OS=Mus musculus OX=10090 GN=Prnp PE=1 SV=2
Major urinary protein 7 OS=Mus musculus OX=10090 GN=Mup7 PE=3 SV=1
Leucine-rich repeat and calponin homology domain-containing protein 1 OS=Mus musculus OX=10090 GN=Lrch1
Heterogeneous nuclear ribonucleoprotein U-like protein 1 OS=Mus musculus OX=10090 GN=Hnrnpul1 PE=1 SV=1
DNA-directed RNA polymerase subunit OS=Mus musculus OX=10090 GN=Polr3a PE=1 SV=1
40S ribosomal protein S19 OS=Mus musculus OX=10090 GN=Rps19 PE=1 SV=3
DNA fragmentation factor subunit alpha OS=Mus musculus OX=10090 GN=Dffa PE=1 SV=2
Tyrosine--tRNA ligase, mitochondrial (Fragment) OS=Mus musculus OX=10090 GN=Yars2 PE=1 SV=8
Pleiotropic regulator 1 OS=Mus musculus OX=10090 GN=Plrg1 PE=1 SV=1
60S ribosomal protein L37 OS=Mus musculus OX=10090 GN=Rpl37 PE=1 SV=3
MPN domain-containing protein OS=Mus musculus OX=10090 GN=Mpnd PE=1 SV=1
Serum paraoxonase/arylesterase 2 OS=Mus musculus OX=10090 GN=Pon2 PE=1 SV=2
Zinc finger FYVE domain-containing protein 26 OS=Mus musculus OX=10090 GN=Zfyve26 PE=1 SV=2
39S ribosomal protein L44, mitochondrial OS=Mus musculus OX=10090 GN=Mrpl44 PE=1 SV=3
Protein kinase C epsilon type OS=Mus musculus OX=10090 GN=Prkce PE=1 SV=1
Sarcoplasmic/endoplasmic reticulum calcium ATPase 1 OS=Mus musculus OX=10090 GN=Atp2a1 PE=1 SV=1
Casein kinase II subunit alpha OS=Mus musculus OX=10090 GN=Csnk2a1 PE=1 SV=2
Cytoplasmic protein NCK2 (Fragment) OS=Mus musculus OX=10090 GN=Nck2 PE=1 SV=1
Tumor suppressor candidate gene 1 protein homolog OS=Mus musculus OX=10090 GN=Tusc1 PE=1 SV=1
E3 ubiquitin-protein ligase RBBP6 OS=Mus musculus OX=10090 GN=Rbbp6 PE=1 SV=5
Cyclin-dependent kinase 13 OS=Mus musculus OX=10090 GN=Cdk13 PE=1 SV=1
SPI6 OS=Mus musculus OX=10090 GN=Serpinb9 PE=1 SV=1

Cullin-7 OS=Mus musculus OX=10090 GN=Cul7 PE=1 SV=2
Casein kinase II subunit beta OS=Mus musculus OX=10090 GN=Csnk2b PE=1 SV=1
Serine/threonine-protein kinase TAO3 OS=Mus musculus OX=10090 GN=Taok3 PE=1 SV=2
ATPase family AAA domain-containing protein 3 OS=Mus musculus OX=10090 GN=Atad3 PE=1 SV=1
Heterogeneous nuclear ribonucleoprotein D0 OS=Mus musculus OX=10090 GN=Hnrnpd PE=1 SV=1
Tumor suppressor candidate 3 OS=Mus musculus OX=10090 GN=Tusc3 PE=1 SV=1
Protein phosphatase 1 regulatory subunit 12A OS=Mus musculus OX=10090 GN=Ppp1r12a PE=1 SV=2
Collagen alpha-2(I) chain OS=Mus musculus OX=10090 GN=Col1a2 PE=1 SV=2
Serine/threonine-protein kinase MARK2 OS=Mus musculus OX=10090 GN=Mark2 PE=1 SV=3
Disks large homolog 1 OS=Mus musculus OX=10090 GN=Dlg1 PE=1 SV=1
Lipase maturation factor 2 OS=Mus musculus OX=10090 GN=Lmf2 PE=1 SV=1
DNA replication licensing factor MCM3 OS=Mus musculus OX=10090 GN=Mcm3 PE=1 SV=2
U5 small nuclear ribonucleoprotein 200 kDa helicase OS=Mus musculus OX=10090 GN=Snrnp200 PE=1 SV=1
LARGE xylosyl- and glucuronyltransferase 1 OS=Mus musculus OX=10090 GN=Large1 PE=1 SV=1
Phosphatase and actin regulator OS=Mus musculus OX=10090 GN=Phactr2 PE=1 SV=2
Nck-associated protein 1-like OS=Mus musculus OX=10090 GN=Nckap1l PE=1 SV=1
Cysteine-rich and transmembrane domain-containing protein 1 (Fragment) OS=Mus musculus OX=10090 GN=Cy
Mothers against decapentaplegic homolog 4 OS=Mus musculus OX=10090 GN=Smad4 PE=1 SV=2
Eukaryotic translation initiation factor 3 subunit B OS=Mus musculus OX=10090 GN=Eif3b PE=1 SV=1
Protein phosphatase 1 regulatory subunit 14A OS=Mus musculus OX=10090 GN=Ppp1r14a PE=1 SV=1
E3 ubiquitin-protein ligase UHRF1 OS=Mus musculus OX=10090 GN=Uhrf1 PE=1 SV=2
Cytochrome b-c1 complex subunit 2, mitochondrial OS=Mus musculus OX=10090 GN=Uqcrc2 PE=1 SV=1
Heterogeneous nuclear ribonucleoprotein A/B OS=Mus musculus OX=10090 GN=Hnrnpab PE=1 SV=1
Sarcolemmal membrane-associated protein OS=Mus musculus OX=10090 GN=Slmap PE=1 SV=1
Interferon-activable protein 205-B OS=Mus musculus OX=10090 GN=Mnda PE=1 SV=1
Small integral membrane protein 4 OS=Mus musculus OX=10090 GN=Smim4 PE=1 SV=2
Cytochrome P450, family 2, subfamily j, polypeptide 11 OS=Mus musculus OX=10090 GN=Cyp2j11 PE=1 SV=1
B-cell receptor-associated protein 31 OS=Mus musculus OX=10090 GN=Bcap31 PE=1 SV=4
Transcription factor jun-D OS=Mus musculus OX=10090 GN=Jund PE=1 SV=1
Malonyl-CoA-acyl carrier protein transacylase, mitochondrial OS=Mus musculus OX=10090 GN=Mcat PE=1 SV=3
Tropomodulin-4 (Fragment) OS=Mus musculus OX=10090 GN=Tmod4 PE=1 SV=1
Serine/arginine repetitive matrix protein 1 OS=Mus musculus OX=10090 GN=Srrm1 PE=1 SV=1
Predicted pseudogene 5478 OS=Mus musculus OX=10090 GN=Gm5478 PE=1 SV=1
Dolichyl-diphosphooligosaccharide--protein glycosyltransferase subunit MAGT1 OS=Mus musculus OX=10090 GN
Granzyme A OS=Mus musculus OX=10090 GN=Gzma PE=1 SV=2
Bone morphogenetic protein 3 OS=Mus musculus OX=10090 GN=Bmp3 PE=1 SV=1
Leucine zipper putative tumor suppressor 1 OS=Mus musculus OX=10090 GN=Lzts1 PE=2 SV=3
DNA replication licensing factor MCM5 OS=Mus musculus OX=10090 GN=Mcm5 PE=1 SV=2
Coiled-coil domain-containing protein 9 OS=Mus musculus OX=10090 GN=Ccdc9 PE=1 SV=1
Arginine and glutamate-rich protein 1 OS=Mus musculus OX=10090 GN=Arglu1 PE=1 SV=2
Ras association domain-containing protein 1 OS=Mus musculus OX=10090 GN=Rassf1 PE=1 SV=1
Malate dehydrogenase, mitochondrial OS=Mus musculus OX=10090 GN=Mdh2 PE=1 SV=3
Leucine-rich PPR motif-containing protein, mitochondrial OS=Mus musculus OX=10090 GN=Lrpprc PE=1 SV=2
Tudor domain-containing protein 3 OS=Mus musculus OX=10090 GN=Tdrd3 PE=1 SV=4
Doublecortin domain-containing 2b (Fragment) OS=Mus musculus OX=10090 GN=Dcdc2b PE=1 SV=1

Hepatocyte growth factor OS=Mus musculus OX=10090 GN=Hgf PE=1 SV=1
Progesterone immunomodulatory-binding factor 1 OS=Mus musculus OX=10090 GN=Pibf1 PE=1 SV=1
Gasdermin-A OS=Mus musculus OX=10090 GN=Gsdma PE=2 SV=1
Proteasome subunit beta type-2 OS=Mus musculus OX=10090 GN=Psmb2 PE=1 SV=1
Nicalin OS=Mus musculus OX=10090 GN=Ncln PE=1 SV=2
RIKEN cDNA 1110059E24 gene OS=Mus musculus OX=10090 GN=1110059E24Rik PE=1 SV=1
39S ribosomal protein L23, mitochondrial OS=Mus musculus OX=10090 GN=Mrpl23 PE=1 SV=1
Transmembrane and coiled-coil domains protein 1 (Fragment) OS=Mus musculus OX=10090 GN=Tmcc1 PE=1 SV
Caveolae-associated protein 1 OS=Mus musculus OX=10090 GN=Cavin1 PE=1 SV=1
Tyrosine-protein kinase BAZ1B OS=Mus musculus OX=10090 GN=Baz1b PE=1 SV=2
Liprin-beta-1 OS=Mus musculus OX=10090 GN=Ppfibp1 PE=1 SV=3
Coiled-coil domain-containing 28A OS=Mus musculus OX=10090 GN=Ccdc28a PE=1 SV=1
Receptor-transporting protein 3 (Fragment) OS=Mus musculus OX=10090 GN=Rtp3 PE=1 SV=1
N-alpha-acetyltransferase 38, NatC auxiliary subunit OS=Mus musculus OX=10090 GN=Naa38 PE=1 SV=1
Cbx3 protein OS=Mus musculus OX=10090 GN=Cbx3 PE=1 SV=1
Protein ABHD13 OS=Mus musculus OX=10090 GN=Abhd13 PE=2 SV=1
E3 ubiquitin-protein ligase TRIM7 OS=Mus musculus OX=10090 GN=Trim7 PE=4 SV=2
Traf2 and NCK-interacting protein kinase OS=Mus musculus OX=10090 GN=Tnik PE=1 SV=1
Myocyte-specific enhancer factor 2C OS=Mus musculus OX=10090 GN=Mef2c PE=1 SV=1
OTU domain-containing protein 4 OS=Mus musculus OX=10090 GN=Otud4 PE=1 SV=1
Casein kinase II subunit alpha' OS=Mus musculus OX=10090 GN=Csnk2a2 PE=1 SV=1
Rho guanine nucleotide exchange factor 10 OS=Mus musculus OX=10090 GN=Arhgef10 PE=1 SV=2
Component of Sp100-rs OS=Mus musculus OX=10090 GN=Csprs PE=2 SV=1
Inositol 1,4,5-trisphosphate receptor type 2 OS=Mus musculus OX=10090 GN=Itpr2 PE=1 SV=4
Anoctamin-6 OS=Mus musculus OX=10090 GN=Ano6 PE=1 SV=1
ARL14 effector protein OS=Mus musculus OX=10090 GN=Arl14ep PE=1 SV=1
TRIO and F-actin-binding protein OS=Mus musculus OX=10090 GN=Triobp PE=1 SV=1
Coiled-coil domain-containing protein 12 OS=Mus musculus OX=10090 GN=Ccdc12 PE=1 SV=2
SLAIN motif-containing protein 2 OS=Mus musculus OX=10090 GN=Slain2 PE=1 SV=2
40S ribosomal protein S14 OS=Mus musculus OX=10090 GN=Rps14 PE=1 SV=3
Protein unc-119 homolog B OS=Mus musculus OX=10090 GN=Unc119b PE=1 SV=1
Prohibitin-2 OS=Mus musculus OX=10090 GN=Phb2 PE=1 SV=1
Kelch domain-containing protein 10 OS=Mus musculus OX=10090 GN=Klhdc10 PE=1 SV=1
Insulin receptor substrate 2 OS=Mus musculus OX=10090 GN=Irs2 PE=1 SV=2
Signal recognition particle 14 kDa protein OS=Mus musculus OX=10090 GN=Srp14 PE=1 SV=1
Quinone oxidoreductase-like protein 1 OS=Mus musculus OX=10090 GN=Cryzl1 PE=1 SV=1
Janus kinase and microtubule-interacting protein 1 OS=Mus musculus OX=10090 GN=Jakmip1 PE=1 SV=1
NADPH oxidase 4 OS=Mus musculus OX=10090 GN=Nox4 PE=1 SV=1
N-alpha-acetyltransferase 25, NatB auxiliary subunit OS=Mus musculus OX=10090 GN=Naa25 PE=1 SV=1
Sterol 26-hydroxylase, mitochondrial OS=Mus musculus OX=10090 GN=Cyp27a1 PE=1 SV=1
Adenylate cyclase type 8 OS=Mus musculus OX=10090 GN=Adcy8 PE=1 SV=2
Proheparin-binding EGF-like growth factor OS=Mus musculus OX=10090 GN=Hbegf PE=2 SV=1
Histone lysine demethylase PHF8 OS=Mus musculus OX=10090 GN=Phf8 PE=1 SV=2
Rho GTPase-activating protein 15 OS=Mus musculus OX=10090 GN=Arhgap15 PE=1 SV=1
SH3 and PX domain-containing protein 2A OS=Mus musculus OX=10090 GN=Sh3pxd2a PE=1 SV=2

Pyruvate dehydrogenase E1 component subunit alpha, somatic form, mitochondrial OS=Mus musculus OX=1009[illegible]
Zinc finger protein 870 OS=Mus musculus OX=10090 GN=Zfp870 PE=2 SV=1
Protein LSM12 homolog OS=Mus musculus OX=10090 GN=Lsm12 PE=1 SV=1
Proline-rich protein 5-like (Fragment) OS=Mus musculus OX=10090 GN=Prr5l PE=1 SV=8
Protein unc-119 homolog A OS=Mus musculus OX=10090 GN=Unc119 PE=1 SV=1
Histidine triad nucleotide-binding protein 2, mitochondrial OS=Mus musculus OX=10090 GN=Hint2 PE=1 SV=1
BRCA1-A complex subunit RAP80 OS=Mus musculus OX=10090 GN=Uimc1 PE=1 SV=2
CREB-regulated transcription coactivator 2 OS=Mus musculus OX=10090 GN=Crtc2 PE=1 SV=2
NTF2-related export protein 2 OS=Mus musculus OX=10090 GN=Nxt2 PE=2 SV=1
Very-long-chain (3R)-3-hydroxyacyl-CoA dehydratase 2 OS=Mus musculus OX=10090 GN=Hacd2 PE=1 SV=1
FH1/FH2 domain-containing protein 1 OS=Mus musculus OX=10090 GN=Fhod1 PE=1 SV=3
Dipeptidyl peptidase 9 OS=Mus musculus OX=10090 GN=Dpp9 PE=1 SV=2
Cyclic AMP-dependent transcription factor ATF-7 OS=Mus musculus OX=10090 GN=Gm28047 PE=2 SV=1
Testis-expressed protein 2 OS=Mus musculus OX=10090 GN=Tex2 PE=1 SV=2
28S ribosomal protein S2, mitochondrial OS=Mus musculus OX=10090 GN=Mrps2 PE=1 SV=1
Merlin (Fragment) OS=Mus musculus OX=10090 GN=Nf2 PE=1 SV=1
Protein FAM126B OS=Mus musculus OX=10090 GN=Fam126b PE=1 SV=1
Leucine-rich repeat-containing protein 40 OS=Mus musculus OX=10090 GN=Lrrc40 PE=1 SV=1
Replication protein A subunit OS=Mus musculus OX=10090 GN=Rpa1 PE=1 SV=2
Ribosomal RNA processing protein 1 homolog A OS=Mus musculus OX=10090 GN=Rrp1 PE=1 SV=2
CREB3 regulatory factor OS=Mus musculus OX=10090 GN=Crebrf PE=2 SV=1
Atrial natriuretic peptide receptor 1 OS=Mus musculus OX=10090 GN=Npr1 PE=1 SV=2
Protein CMSS1 OS=Mus musculus OX=10090 GN=Cmss1 PE=2 SV=1
Serine/threonine-protein kinase 25 (Fragment) OS=Mus musculus OX=10090 GN=Stk25 PE=1 SV=1
Cytoskeleton-associated protein 4 OS=Mus musculus OX=10090 GN=Ckap4 PE=1 SV=2
Hemoglobin subunit epsilon-Y2 OS=Mus musculus OX=10090 GN=Hbb-y PE=1 SV=2
Hepatoma-derived growth factor OS=Mus musculus OX=10090 GN=Hdgf PE=1 SV=2
Dolichyl-diphosphooligosaccharide--protein glycotransferase OS=Mus musculus OX=10090 GN=Stt3b PE=1 SV=1
SUN domain-containing protein 2 OS=Mus musculus OX=10090 GN=Sun2 PE=1 SV=3
RIKEN cDNA 9930021J03 gene OS=Mus musculus OX=10090 GN=9930021J03Rik PE=1 SV=1
DNA helicase B OS=Mus musculus OX=10090 GN=Helb PE=1 SV=2
Sickle tail protein OS=Mus musculus OX=10090 GN=Skt PE=1 SV=1
Histone H1.4 OS=Mus musculus OX=10090 GN=H1-4 PE=1 SV=2
Rapamycin-insensitive companion of mTOR OS=Mus musculus OX=10090 GN=Rictor PE=1 SV=2
Coiled-coil domain-containing protein 124 OS=Mus musculus OX=10090 GN=Ccdc124 PE=1 SV=1
Serine/threonine-protein phosphatase PP1-beta catalytic subunit OS=Mus musculus OX=10090 GN=Ppp1cb PE=1
Nuclear factor 1 OS=Mus musculus OX=10090 GN=Nfix PE=1 SV=1
Nuclear fragile X mental retardation-interacting protein 2 OS=Mus musculus OX=10090 GN=Nufip2 PE=1 SV=1
Treacle protein OS=Mus musculus OX=10090 GN=Tcof1 PE=1 SV=1
Netrin-1 OS=Mus musculus OX=10090 GN=Ntn1 PE=1 SV=3
S-adenosylhomocysteine hydrolase-like protein 1 OS=Mus musculus OX=10090 GN=Ahcyl1 PE=1 SV=1
Eukaryotic translation initiation factor 4C OS=Mus musculus OX=10090 GN=Eif1a PE=1 SV=1
Mitochondrial import receptor subunit TOM20 homolog OS=Mus musculus OX=10090 GN=Tomm20 PE=1 SV=1
SH2 domain-containing adapter protein B OS=Mus musculus OX=10090 GN=Shb PE=1 SV=2
Peptidyl-prolyl cis-trans isomerase-like 4 OS=Mus musculus OX=10090 GN=Ppil4 PE=1 SV=2

Small conductance calcium-activated potassium channel protein 3 OS=Mus musculus OX=10090 GN=Kcnn3 PE=1
Spectrin alpha chain, non-erythrocytic 1 OS=Mus musculus OX=10090 GN=Sptan1 PE=1 SV=2
Zinc finger protein castor homolog 1 OS=Mus musculus OX=10090 GN=Casz1 PE=1 SV=1
Pleckstrin homology domain-containing family A member 7 (Fragment) OS=Mus musculus OX=10090 GN=Plekha
Developmentally-regulated GTP-binding protein 1 OS=Mus musculus OX=10090 GN=Drg1 PE=1 SV=1
Methyl-CpG-binding domain protein 6 (Fragment) OS=Mus musculus OX=10090 GN=Mbd6 PE=1 SV=1
AMP deaminase OS=Mus musculus OX=10090 GN=Ampd3 PE=1 SV=1
Methylcrotonoyl-CoA carboxylase subunit alpha, mitochondrial OS=Mus musculus OX=10090 GN=Mccc1 PE=1 SV
Glutamate-rich 1 OS=Mus musculus OX=10090 GN=Erich1 PE=1 SV=1
Elongin-A OS=Mus musculus OX=10090 GN=Eloa PE=1 SV=3
Set1/Ash2 histone methyltransferase complex subunit ASH2 OS=Mus musculus OX=10090 GN=Ash2l PE=1 SV=1
Syntaxin-7 OS=Mus musculus OX=10090 GN=Stx7 PE=1 SV=1
DDB1- and CUL4-associated factor 7 OS=Mus musculus OX=10090 GN=Dcaf7 PE=1 SV=1
Ribosome biogenesis protein WDR12 OS=Mus musculus OX=10090 GN=Wdr12 PE=1 SV=1
Protein mago nashi homolog 2 OS=Mus musculus OX=10090 GN=Magohb PE=2 SV=1
Phosphoglycerate kinase 1 OS=Mus musculus OX=10090 GN=Pgk1 PE=1 SV=4
Leucine-rich repeat-containing 37A OS=Mus musculus OX=10090 GN=Lrrc37a PE=1 SV=2
Zinc finger protein 9 OS=Mus musculus OX=10090 GN=Zfp9 PE=1 SV=1
Signal-induced proliferation-associated 1-like protein 1 OS=Mus musculus OX=10090 GN=Sipa1l1 PE=1 SV=2
CYFIP-related Rac1 interactor A OS=Mus musculus OX=10090 GN=Cyria PE=1 SV=1
Aspartyl/asparaginyl beta-hydroxylase OS=Mus musculus OX=10090 GN=Asph PE=1 SV=1
Ras GTPase-activating protein 4 OS=Mus musculus OX=10090 GN=Rasa4 PE=1 SV=1
ATP-dependent RNA helicase DDX19A OS=Mus musculus OX=10090 GN=Ddx19a PE=1 SV=2
Polyhomeotic-like protein 2 OS=Mus musculus OX=10090 GN=Phc2 PE=1 SV=1
Collagen alpha-1(I) chain OS=Mus musculus OX=10090 GN=Col1a1 PE=1 SV=4
CCR4-NOT transcription complex subunit 7 OS=Mus musculus OX=10090 GN=Cnot7 PE=1 SV=1
Chromodomain-helicase-DNA-binding protein 8 OS=Mus musculus OX=10090 GN=Chd8 PE=1 SV=1
Serine/threonine-protein phosphatase 4 regulatory subunit 1 OS=Mus musculus OX=10090 GN=Ppp4r1 PE=1 SV=
Translocon-associated protein subunit beta OS=Mus musculus OX=10090 GN=Ssr2 PE=1 SV=1
Calcium-binding mitochondrial carrier protein SCaMC-2 (Fragment) OS=Mus musculus OX=10090 GN=Slc25a25 P
Mortality factor 4-like protein 1 OS=Mus musculus OX=10090 GN=Morf4l1 PE=1 SV=1
60S ribosomal protein L7 OS=Mus musculus OX=10090 GN=Rpl7 PE=1 SV=2
Ras-related GTP-binding protein OS=Mus musculus OX=10090 GN=Rragd PE=1 SV=1
Plakophilin-3 OS=Mus musculus OX=10090 GN=Pkp3 PE=1 SV=2
Chloride intracellular channel protein OS=Mus musculus OX=10090 GN=Clic3 PE=1 SV=1
Thrombospondin-1 OS=Mus musculus OX=10090 GN=Thbs1 PE=1 SV=1
Spectrin alpha chain, non-erythrocytic 1 OS=Mus musculus OX=10090 GN=Sptan1 PE=1 SV=4
WD repeat-containing protein 5 OS=Mus musculus OX=10090 GN=Wdr5 PE=1 SV=1
Myosin light chain 6B OS=Mus musculus OX=10090 GN=Myl6b PE=1 SV=1
Histone H2B type 1-B OS=Mus musculus OX=10090 GN=H2bc3 PE=1 SV=3
Phosphatidylglycerophosphatase and protein-tyrosine phosphatase 1 OS=Mus musculus OX=10090 GN=Ptpmt1 P
Nucleosome assembly protein 1-like 1 OS=Mus musculus OX=10090 GN=Nap1l1 PE=1 SV=1
Pre-mRNA-splicing factor CWC22 homolog OS=Mus musculus OX=10090 GN=Cwc22 PE=1 SV=1
Leukocyte immunoglobulin-like receptor subfamily B member 3 OS=Mus musculus OX=10090 GN=Lilrb3 PE=1 SV
StAR-related lipid transfer protein 13 OS=Mus musculus OX=10090 GN=Stard13 PE=1 SV=1

Striatin-4 OS=Mus musculus OX=10090 GN=Strn4 PE=1 SV=2
Serrate RNA effector molecule homolog OS=Mus musculus OX=10090 GN=Srrt PE=1 SV=1
U5 small nuclear ribonucleoprotein 40 kDa protein OS=Mus musculus OX=10090 GN=Snrnp40 PE=1 SV=1
Tyrosine-protein phosphatase non-receptor type OS=Mus musculus OX=10090 GN=Ptpn14 PE=1 SV=1
Signal peptidase complex subunit 3 OS=Mus musculus OX=10090 GN=Spcs3 PE=1 SV=1
Cleavage and polyadenylation-specificity factor subunit 1 OS=Mus musculus OX=10090 GN=Cpsf1 PE=1 SV=1
RNA-binding motif protein 6 OS=Mus musculus OX=10090 GN=Rbm6 PE=1 SV=1
Dystrophin OS=Mus musculus OX=10090 GN=Dmd PE=1 SV=3
RNA-binding protein 39 OS=Mus musculus OX=10090 GN=Rbm39 PE=1 SV=2
Rho guanine nucleotide exchange factor TIAM1 OS=Mus musculus OX=10090 GN=Tiam1 PE=1 SV=1
Protein dpy-30 homolog OS=Mus musculus OX=10090 GN=Dpy30 PE=1 SV=1
Heterogeneous nuclear ribonucleoproteins A2/B1 OS=Mus musculus OX=10090 GN=Hnrnpa2b1 PE=1 SV=2
Insulin-like growth factor-binding protein 7 OS=Mus musculus OX=10090 GN=Igfbp7 PE=1 SV=3
Heterogeneous nuclear ribonucleoprotein A0 OS=Mus musculus OX=10090 GN=Hnrnpa0 PE=1 SV=1
Isocitrate dehydrogenase [NAD] subunit gamma 1, mitochondrial OS=Mus musculus OX=10090 GN=Idh3g PE=1 S
BRD4-interacting chromatin-remodeling complex-associated protein OS=Mus musculus OX=10090 GN=Bicra PE=
NADH dehydrogenase [ubiquinone] 1 alpha subcomplex subunit 8 OS=Mus musculus OX=10090 GN=Ndufa8 PE=1
NEDD8 ultimate buster 1 OS=Mus musculus OX=10090 GN=Nub1 PE=1 SV=2
Coronin-1C OS=Mus musculus OX=10090 GN=Coro1c PE=1 SV=2
Piwi-like protein 1 (Fragment) OS=Mus musculus OX=10090 GN=Piwil1 PE=1 SV=1
Eukaryotic translation initiation factor 3 subunit G OS=Mus musculus OX=10090 GN=Eif3g PE=1 SV=2
Mediator of RNA polymerase II transcription subunit 10 OS=Mus musculus OX=10090 GN=Med10 PE=1 SV=1
C->U-editing enzyme APOBEC-1 (Fragment) OS=Mus musculus OX=10090 GN=Apobec1 PE=1 SV=1
Inactive tyrosine-protein kinase PEAK1 OS=Mus musculus OX=10090 GN=Peak1 PE=1 SV=4
N-acetyltransferase ESCO1 OS=Mus musculus OX=10090 GN=Esco1 PE=1 SV=1
C-C motif chemokine 21a OS=Mus musculus OX=10090 GN=Ccl21a PE=1 SV=1
Protein ECT2 OS=Mus musculus OX=10090 GN=Ect2 PE=1 SV=2
Cyclin-K OS=Mus musculus OX=10090 GN=Ccnk PE=1 SV=1
Cell division control protein 42 homolog OS=Mus musculus OX=10090 GN=Cdc42 PE=1 SV=2
STE20-like serine/threonine-protein kinase OS=Mus musculus OX=10090 GN=Slk PE=1 SV=2
60S ribosomal protein L22-like 1 OS=Mus musculus OX=10090 GN=Rpl22l1 PE=1 SV=1
c-Myc-binding protein OS=Mus musculus OX=10090 GN=Mycbp PE=1 SV=1
Guanine nucleotide exchange factor VAV2 OS=Mus musculus OX=10090 GN=Vav2 PE=1 SV=1
Beta/gamma crystallin domain-containing protein 3 OS=Mus musculus OX=10090 GN=Crybg3 PE=1 SV=2
Interferon-activated gene 207 OS=Mus musculus OX=10090 GN=Ifi207 PE=1 SV=1
Cadherin-1 OS=Mus musculus OX=10090 GN=Cdh1 PE=1 SV=1
WD40 repeat-containing protein SMU1 OS=Mus musculus OX=10090 GN=Smu1 PE=2 SV=2
Spectrin beta chain, non-erythrocytic 1 OS=Mus musculus OX=10090 GN=Sptbn1 PE=1 SV=2
Interphotoreceptor matrix proteoglycan 2 OS=Mus musculus OX=10090 GN=Impg2 PE=1 SV=1
TESP4 OS=Mus musculus OX=10090 GN=Try5 PE=1 SV=1
60S ribosomal protein L28 OS=Mus musculus OX=10090 GN=Rpl28 PE=1 SV=2
E3 ubiquitin-protein ligase RNF4 OS=Mus musculus OX=10090 GN=Rnf4 PE=1 SV=1
Homeobox protein Nkx-3.2 OS=Mus musculus OX=10090 GN=Nkx3-2 PE=1 SV=2
G protein-coupled receptor kinase OS=Mus musculus OX=10090 GN=Grk6 PE=1 SV=1
Splicing factor 3B subunit 3 OS=Mus musculus OX=10090 GN=Sf3b3 PE=1 SV=1

Eukaryotic translation initiation factor 3 subunit D OS=Mus musculus OX=10090 GN=Eif3d PE=1 SV=2
ATP synthase subunit epsilon, mitochondrial OS=Mus musculus OX=10090 GN=Atp5f1e PE=1 SV=2
Cyclin-dependent kinase 17 OS=Mus musculus OX=10090 GN=Cdk17 PE=1 SV=2
Protein LTV1 homolog OS=Mus musculus OX=10090 GN=Ltv1 PE=1 SV=2
E3 ubiquitin-protein ligase OS=Mus musculus OX=10090 GN=Smurf1 PE=1 SV=1
T-box transcription factor TBX3 OS=Mus musculus OX=10090 GN=Tbx3 PE=1 SV=3
Kinesin-like protein OS=Mus musculus OX=10090 GN=Kifc5b PE=1 SV=1
RNA exonuclease 1 homolog OS=Mus musculus OX=10090 GN=Rexo1 PE=1 SV=1
Interferon gamma-induced GTPase OS=Mus musculus OX=10090 GN=Igtp PE=1 SV=1
Dysferlin OS=Mus musculus OX=10090 GN=Dysf PE=1 SV=1
Eukaryotic translation initiation factor 3 subunit L OS=Mus musculus OX=10090 GN=Eif3l PE=1 SV=1
Bromodomain adjacent to zinc finger domain protein 2B OS=Mus musculus OX=10090 GN=Baz2b PE=1 SV=1
Sorting and assembly machinery component 50 homolog OS=Mus musculus OX=10090 GN=Samm50 PE=1 SV=1
RNA demethylase ALKBH5 OS=Mus musculus OX=10090 GN=Alkbh5 PE=1 SV=2
Heterogeneous nuclear ribonucleoprotein D0 OS=Mus musculus OX=10090 GN=Hnrnpd PE=1 SV=2
Vitronectin OS=Mus musculus OX=10090 GN=Vtn PE=1 SV=2
Translocation protein SEC63 homolog OS=Mus musculus OX=10090 GN=Sec63 PE=1 SV=4
Mediator of RNA polymerase II transcription subunit 15 OS=Mus musculus OX=10090 GN=Med15 PE=1 SV=1
ADP-ribosylation factor-like protein 6-interacting protein 4 OS=Mus musculus OX=10090 GN=Arl6ip4 PE=1 SV=1
Glutamate--cysteine ligase regulatory subunit (Fragment) OS=Mus musculus OX=10090 GN=Gclm PE=1 SV=1
Protein IMPACT OS=Mus musculus OX=10090 GN=Impact PE=1 SV=2
Probable ATP-dependent RNA helicase DDX5 OS=Mus musculus OX=10090 GN=Ddx5 PE=1 SV=2
Heterogeneous nuclear ribonucleoprotein A1 OS=Mus musculus OX=10090 GN=Hnrnpa1 PE=1 SV=2
Trafficking protein particle complex subunit 2-like protein OS=Mus musculus OX=10090 GN=Trappc2l PE=1 SV=1
Ankyrin repeat and KH domain-containing 1 OS=Mus musculus OX=10090 GN=Ankhd1 PE=1 SV=1
Mediator of DNA damage checkpoint protein 1 OS=Mus musculus OX=10090 GN=Mdc1 PE=1 SV=1
Protein C12orf4 homolog OS=Mus musculus OX=10090 GN=D6Wsu163e PE=1 SV=1
Protein arginine N-methyltransferase 5 OS=Mus musculus OX=10090 GN=Prmt5 PE=1 SV=1
LIM domain-containing protein 2 OS=Mus musculus OX=10090 GN=Limd2 PE=1 SV=1
Rho GTPase-activating protein 26 OS=Mus musculus OX=10090 GN=Arhgap26 PE=1 SV=1
GEM-interacting protein OS=Mus musculus OX=10090 GN=Gmip PE=1 SV=1
Transmembrane and coiled-coil domains protein 2 OS=Mus musculus OX=10090 GN=Tmcc2 PE=1 SV=1
Tripartite motif-containing protein 3 OS=Mus musculus OX=10090 GN=Trim3 PE=1 SV=1
Polynucleotide 5'-hydroxyl-kinase NOL9 OS=Mus musculus OX=10090 GN=Nol9 PE=1 SV=1
Phosphatidylinositol 3,4,5-trisphosphate 3-phosphatase and dual-specificity protein phosphatase PTEN OS=Mus r
Zinc finger and BTB domain-containing protein 9 OS=Mus musculus OX=10090 GN=Zbtb9 PE=2 SV=1
L-lactate dehydrogenase OS=Mus musculus OX=10090 GN=Ldhb PE=1 SV=1
Signal recognition particle 54 kDa protein OS=Mus musculus OX=10090 GN=Srp54c PE=1 SV=1
Kinesin-like protein KIF15 OS=Mus musculus OX=10090 GN=Kif15 PE=1 SV=1
Low density lipoprotein receptor adapter protein 1 OS=Mus musculus OX=10090 GN=Ldlrap1 PE=1 SV=3
Ski oncogene OS=Mus musculus OX=10090 GN=Ski PE=1 SV=2
Zinc finger protein 326 OS=Mus musculus OX=10090 GN=Zfp326 PE=1 SV=1
Nuclear receptor coactivator 5 OS=Mus musculus OX=10090 GN=Ncoa5 PE=1 SV=1
39S ribosomal protein L14, mitochondrial OS=Mus musculus OX=10090 GN=Mrpl14 PE=1 SV=1
Kinesin-like protein KIF26A OS=Mus musculus OX=10090 GN=Kif26a PE=1 SV=2

Tartrate-resistant acid phosphatase type 5 OS=Mus musculus OX=10090 GN=Acp5 PE=1 SV=2
Catenin beta-1 OS=Mus musculus OX=10090 GN=Ctnnb1 PE=1 SV=1
Cysteine and glycine-rich protein 1 OS=Mus musculus OX=10090 GN=Csrp1 PE=1 SV=3
Leydig cell tumor 10 kDa protein homolog OS=Mus musculus OX=10090 GN=D8Ertd738e PE=3 SV=1
Neutrophilic granule protein OS=Mus musculus OX=10090 GN=Ngp PE=1 SV=1
EH domain-binding protein 1-like protein 1 OS=Mus musculus OX=10090 GN=Ehbp1l1 PE=1 SV=1
Aryl hydrocarbon receptor OS=Mus musculus OX=10090 GN=Ahr PE=1 SV=1
Plexin-B2 OS=Mus musculus OX=10090 GN=Plxnb2 PE=1 SV=1
BTB (POZ) domain-containing 2 OS=Mus musculus OX=10090 GN=Btbd2 PE=1 SV=1
Syntaxin-binding protein 5 OS=Mus musculus OX=10090 GN=Stxbp5 PE=1 SV=1
Chromodomain-helicase-DNA-binding protein 1 OS=Mus musculus OX=10090 GN=Chd1 PE=1 SV=3
Protein WWC2 OS=Mus musculus OX=10090 GN=Wwc2 PE=1 SV=1
Cyclic AMP-dependent transcription factor ATF-6 alpha OS=Mus musculus OX=10090 GN=Atf6 PE=1 SV=2
Scaffold attachment factor B1 OS=Mus musculus OX=10090 GN=Safb PE=1 SV=2
Tripartite motif-containing protein 30A OS=Mus musculus OX=10090 GN=Trim30a PE=1 SV=2
UPF0696 protein C11orf68 homolog OS=Mus musculus OX=10090 GN=Bles03 PE=1 SV=2
F-box only protein 3 OS=Mus musculus OX=10090 GN=Fbxo3 PE=1 SV=1
3-hydroxyacyl-CoA dehydrogenase type-2 OS=Mus musculus OX=10090 GN=Hsd17b10 PE=1 SV=1
TRAF-type zinc finger domain-containing protein 1 OS=Mus musculus OX=10090 GN=Trafd1 PE=1 SV=1
Band 4.1 OS=Mus musculus OX=10090 GN=Epb41 PE=1 SV=1
Volume-regulated anion channel subunit LRRC8A OS=Mus musculus OX=10090 GN=Lrrc8a PE=1 SV=1
Protein pelota homolog OS=Mus musculus OX=10090 GN=Pelo PE=1 SV=3
Bromodomain adjacent to zinc finger domain protein 2A OS=Mus musculus OX=10090 GN=Baz2a PE=1 SV=1
Serine/threonine-protein kinase MARK1 OS=Mus musculus OX=10090 GN=Mark1 PE=1 SV=2
Histone H1.5 OS=Mus musculus OX=10090 GN=H1-5 PE=1 SV=2
Copper-transporting ATPase 1 OS=Mus musculus OX=10090 GN=Atp7a PE=1 SV=3
KRAB-containing zinc-finger protein KRAZ2 OS=Mus musculus OX=10090 GN=Zfp68 PE=1 SV=1
Pinin OS=Mus musculus OX=10090 GN=Pnn PE=1 SV=1
ELAV-like protein 1 OS=Mus musculus OX=10090 GN=Elavl1 PE=1 SV=2
Creatine kinase M-type OS=Mus musculus OX=10090 GN=Ckm PE=1 SV=1
Dynactin subunit 1 OS=Mus musculus OX=10090 GN=Dctn1 PE=1 SV=1
28S ribosomal protein S5, mitochondrial OS=Mus musculus OX=10090 GN=Mrps5 PE=1 SV=1
39S ribosomal protein L41, mitochondrial OS=Mus musculus OX=10090 GN=Mrpl41 PE=1 SV=1
U3 small nucleolar RNA-associated protein 14 homolog A OS=Mus musculus OX=10090 GN=Utp14a PE=1 SV=1
Junction plakoglobin OS=Mus musculus OX=10090 GN=Jup PE=1 SV=3
Striatin OS=Mus musculus OX=10090 GN=Strn PE=1 SV=2
39S ribosomal protein L33, mitochondrial OS=Mus musculus OX=10090 GN=Mrpl33 PE=1 SV=1
Immunoglobulin kappa variable 8-21 (Fragment) OS=Mus musculus OX=10090 GN=Igkv8-21 PE=1 SV=2
Rho guanine nucleotide exchange factor 12 (Fragment) OS=Mus musculus OX=10090 GN=Arhgef12 PE=1 SV=1
Tumor protein D54 (Fragment) OS=Mus musculus OX=10090 GN=Tpd52l2 PE=1 SV=1
Bystin OS=Mus musculus OX=10090 GN=Bysl PE=1 SV=3
Choline-phosphate cytidylyltransferase A OS=Mus musculus OX=10090 GN=Pcyt1a PE=1 SV=1
Twinfilin-1 OS=Mus musculus OX=10090 GN=Twf1 PE=1 SV=2
General transcription factor IIE subunit 1 OS=Mus musculus OX=10090 GN=Gtf2e1 PE=1 SV=1
Target of rapamycin complex 2 subunit MAPKAP1 OS=Mus musculus OX=10090 GN=Mapkap1 PE=1 SV=1

Transcription factor MafF OS=Mus musculus OX=10090 GN=Maff PE=2 SV=1
CMP-N-acetylneuraminate-poly-alpha-2,8-sialyltransferase OS=Mus musculus OX=10090 GN=St8sia4 PE=1 SV=1
RIKEN cDNA 1810009J06 gene OS=Mus musculus OX=10090 GN=1810009J06Rik PE=2 SV=1
General transcription factor 3C polypeptide 6 (Fragment) OS=Mus musculus OX=10090 GN=Gtf3c6 PE=4 SV=1
Tyrosine-protein phosphatase non-receptor type OS=Mus musculus OX=10090 GN=Ptpn21 PE=1 SV=1
Dehydrogenase/reductase SDR family member 4 OS=Mus musculus OX=10090 GN=Dhrs4 PE=1 SV=3
Coiled-coil domain-containing protein 8 homolog OS=Mus musculus OX=10090 GN=Ccdc8 PE=1 SV=2
Ceramide synthase 5 OS=Mus musculus OX=10090 GN=Cers5 PE=1 SV=2
Cyclin-dependent-like kinase 5 OS=Mus musculus OX=10090 GN=Cdk5 PE=1 SV=1
Growth factor receptor-bound protein 7 OS=Mus musculus OX=10090 GN=Grb7 PE=1 SV=1
CTD nuclear envelope phosphatase 1 (Fragment) OS=Mus musculus OX=10090 GN=Ctdnep1 PE=1 SV=1
NADH dehydrogenase (ubiquinone) complex I, assembly factor 6 OS=Mus musculus OX=10090 GN=Ndufaf6 PE=1
Protein FAM91A1 OS=Mus musculus OX=10090 GN=Fam91a1 PE=1 SV=1
Rho guanine nucleotide exchange factor 1 OS=Mus musculus OX=10090 GN=Arhgef1 PE=1 SV=1
39S ribosomal protein L43, mitochondrial OS=Mus musculus OX=10090 GN=Mrpl43 PE=1 SV=1
Neuronal proto-oncogene tyrosine-protein kinase Src OS=Mus musculus OX=10090 GN=Src PE=1 SV=5
Brain-specific angiogenesis inhibitor 1-associated protein 2 OS=Mus musculus OX=10090 GN=Baiap2 PE=1 SV=1
Dedicator of cytokinesis protein 2 OS=Mus musculus OX=10090 GN=Dock2 PE=1 SV=3
Na(+)/H(+) exchange regulatory cofactor NHE-RF2 OS=Mus musculus OX=10090 GN=Slc9a3r2 PE=1 SV=2
Zinc finger protein 335 OS=Mus musculus OX=10090 GN=Znf335 PE=1 SV=1
Box C/D snoRNA protein 1 OS=Mus musculus OX=10090 GN=Znhit6 PE=1 SV=2
Ribosomal RNA processing protein 1 homolog B OS=Mus musculus OX=10090 GN=Rrp1b PE=1 SV=2
Squamous cell carcinoma antigen recognized by T-cells 3 OS=Mus musculus OX=10090 GN=Sart3 PE=1 SV=1
BAG family molecular chaperone regulator 1 OS=Mus musculus OX=10090 GN=Bag1 PE=1 SV=3
Protein Smaug homolog 2 OS=Mus musculus OX=10090 GN=Samd4b PE=1 SV=1
Erlin-2 OS=Mus musculus OX=10090 GN=Erlin2 PE=1 SV=1
Homeobox protein Hox-B6 OS=Mus musculus OX=10090 GN=Hoxb6 PE=1 SV=2
Solute carrier family 43 member 3 OS=Mus musculus OX=10090 GN=Slc43a3 PE=1 SV=1
Myocyte-specific enhancer factor 2D OS=Mus musculus OX=10090 GN=Mef2d PE=1 SV=1
Deoxynucleotidyltransferase terminal-interacting protein 2 OS=Mus musculus OX=10090 GN=Dnttip2 PE=1 SV=1
Thyroid hormone receptor-associated protein 3 OS=Mus musculus OX=10090 GN=Thrap3 PE=1 SV=1
Telomeric repeat-binding factor OS=Mus musculus OX=10090 GN=Terf2 PE=1 SV=1
Flavin reductase (NADPH) OS=Mus musculus OX=10090 GN=Blvrb PE=1 SV=3
FACT complex subunit SSRP1 OS=Mus musculus OX=10090 GN=Ssrp1 PE=1 SV=2
Ankyrin-1 OS=Mus musculus OX=10090 GN=Ank1 PE=1 SV=1
FAT atypical cadherin 1 OS=Mus musculus OX=10090 GN=Fat1 PE=1 SV=1
YTH domain-containing family protein 1 OS=Mus musculus OX=10090 GN=Ythdf1 PE=1 SV=1
Transmembrane and coiled-coil domain protein 3 OS=Mus musculus OX=10090 GN=Tmcc3 PE=1 SV=2
5'-AMP-activated protein kinase subunit beta-1 OS=Mus musculus OX=10090 GN=Prkab1 PE=1 SV=2
Pleckstrin homology domain-containing family A member 6 OS=Mus musculus OX=10090 GN=Plekha6 PE=1 SV=[illegible]
High mobility group protein HMG-I/HMG-Y OS=Mus musculus OX=10090 GN=Hmga1 PE=1 SV=4
Arginine/serine-rich protein 1 OS=Mus musculus OX=10090 GN=Rsrp1 PE=1 SV=1
Ras and Rab interactor 3 OS=Mus musculus OX=10090 GN=Rin3 PE=1 SV=2
WD repeat-containing protein 13 OS=Mus musculus OX=10090 GN=Wdr13 PE=1 SV=1
NKAP domain-containing 1 OS=Mus musculus OX=10090 GN=Nkapd1 PE=1 SV=1

Anion exchange protein OS=Mus musculus OX=10090 GN=Slc4a4 PE=1 SV=1
Protein AF-10 OS=Mus musculus OX=10090 GN=Mllt10 PE=1 SV=1
Heterogeneous nuclear ribonucleoprotein F OS=Mus musculus OX=10090 GN=Hnrnpf PE=1 SV=3
Protein strawberry notch homolog 1 OS=Mus musculus OX=10090 GN=Sbno1 PE=1 SV=1
A disintegrin and metalloproteinase with thrombospondin motifs 1 OS=Mus musculus OX=10090 GN=Adamts1 P
Zyxin OS=Mus musculus OX=10090 GN=Zyx PE=1 SV=2
RNA-binding protein 12B-A OS=Mus musculus OX=10090 GN=Rbm12b1 PE=1 SV=2
Disintegrin and metalloproteinase domain-containing protein 9 OS=Mus musculus OX=10090 GN=Adam9 PE=1 S
Armadillo repeat-containing X-linked protein 1 OS=Mus musculus OX=10090 GN=Armcx1 PE=1 SV=1
Transformation/transcription domain-associated protein OS=Mus musculus OX=10090 GN=Trrap PE=1 SV=1
PH domain leucine-rich repeat-containing protein phosphatase 1 OS=Mus musculus OX=10090 GN=Phlpp1 PE=1 [illegible]
Expressed sequence AU041133 OS=Mus musculus OX=10090 GN=AU041133 PE=4 SV=1
RIKEN cDNA 0610010K14 gene OS=Mus musculus OX=10090 GN=0610010K14Rik PE=1 SV=1
Polycomb complex protein BMI-1 (Fragment) OS=Mus musculus OX=10090 GN=Bmi1 PE=1 SV=1
Rho guanine nucleotide exchange factor 18 OS=Mus musculus OX=10090 GN=Arhgef18 PE=1 SV=1
cAMP-dependent protein kinase type II-beta regulatory subunit OS=Mus musculus OX=10090 GN=Prkar2b PE=1 S
Zfp617 protein OS=Mus musculus OX=10090 GN=Zfp617 PE=1 SV=1
SPRY domain-containing 3 OS=Mus musculus OX=10090 GN=Spryd3 PE=1 SV=1
WD repeat-containing protein 7 OS=Mus musculus OX=10090 GN=Wdr7 PE=1 SV=1
Vimentin OS=Mus musculus OX=10090 GN=Vim PE=1 SV=3
cGMP-dependent protein kinase OS=Mus musculus OX=10090 GN=Prkg1 PE=1 SV=1
RNA 5'-monophosphate methyltransferase OS=Mus musculus OX=10090 GN=Bcdin3d PE=2 SV=1
Protein NDNF OS=Mus musculus OX=10090 GN=Ndnf PE=1 SV=2
Thioredoxin-interacting protein OS=Mus musculus OX=10090 GN=Txnip PE=1 SV=1
Phospholipid scramblase 3 OS=Mus musculus OX=10090 GN=Plscr3 PE=1 SV=1
Aladin OS=Mus musculus OX=10090 GN=Aaas PE=1 SV=1
Exosome component 10 OS=Mus musculus OX=10090 GN=Exosc10 PE=1 SV=2
Nucleolar protein 10 OS=Mus musculus OX=10090 GN=Nol10 PE=2 SV=1
Striatin-3 OS=Mus musculus OX=10090 GN=Strn3 PE=1 SV=1
Phosphatidylinositol-4-phosphate 3-kinase catalytic subunit type 2 beta (Fragment) OS=Mus musculus OX=1009[illegible]
Smad nuclear-interacting protein 1 OS=Mus musculus OX=10090 GN=Snip1 PE=1 SV=1
Histone chaperone ASF1A OS=Mus musculus OX=10090 GN=Asf1a PE=1 SV=1
GTP-binding protein 4 OS=Mus musculus OX=10090 GN=Gtpbp4 PE=1 SV=3
Mapk-regulated corepressor-interacting protein 1 OS=Mus musculus OX=10090 GN=Mcrip1 PE=1 SV=1
N(G),N(G)-dimethylarginine dimethylaminohydrolase 2 OS=Mus musculus OX=10090 GN=Ddah2 PE=1 SV=1
Protein FAM102B OS=Mus musculus OX=10090 GN=Fam102b PE=1 SV=1
Guanine nucleotide exchange factor H1 OS=Mus musculus OX=10090 GN=Arhgef2 PE=1 SV=1
Utrophin OS=Mus musculus OX=10090 GN=Utrn PE=1 SV=1
B box and SPRY domain-containing protein OS=Mus musculus OX=10090 GN=Bspry PE=1 SV=1
Serine/threonine-protein phosphatase 2A 56 kDa regulatory subunit alpha isoform OS=Mus musculus OX=10090
Myosin-1 OS=Mus musculus OX=10090 GN=Myh1 PE=1 SV=1
Ribonuclease P protein subunit p29 OS=Mus musculus OX=10090 GN=Pop4 PE=2 SV=1
Protein S100-A4 (Fragment) OS=Mus musculus OX=10090 GN=S100a4 PE=1 SV=1
MICOS complex subunit Mic19 OS=Mus musculus OX=10090 GN=Chchd3 PE=1 SV=1
WD repeat-containing protein 1 OS=Mus musculus OX=10090 GN=Wdr1 PE=1 SV=3

Collagen alpha-4(IV) chain OS=Mus musculus OX=10090 GN=Col4a4 PE=1 SV=1
39S ribosomal protein L22, mitochondrial OS=Mus musculus OX=10090 GN=Mrpl22 PE=1 SV=1
Zinc finger C2HC domain-containing protein 1A OS=Mus musculus OX=10090 GN=Zc2hc1a PE=1 SV=1
Epsin-2 OS=Mus musculus OX=10090 GN=Epn2 PE=1 SV=1
Monocarboxylate transporter 1 OS=Mus musculus OX=10090 GN=Slc16a1 PE=1 SV=1
Zinc finger protein 263 OS=Mus musculus OX=10090 GN=Znf263 PE=1 SV=1
Cell growth-regulating nucleolar protein OS=Mus musculus OX=10090 GN=Lyar PE=1 SV=2
RNA helicase (Fragment) OS=Mus musculus OX=10090 GN=Dhx8 PE=1 SV=8
SUMO-interacting motif-containing protein 1 OS=Mus musculus OX=10090 GN=Simc1 PE=1 SV=1
Trafficking protein particle complex subunit 11 OS=Mus musculus OX=10090 GN=Trappc11 PE=1 SV=1
Rab GTPase-activating protein 1-like OS=Mus musculus OX=10090 GN=Rabgap1l PE=1 SV=1
Retinoic acid-induced protein 1 OS=Mus musculus OX=10090 GN=Rai1 PE=1 SV=3
Aquaporin-1 OS=Mus musculus OX=10090 GN=Aqp1 PE=1 SV=3
28S ribosomal protein S28, mitochondrial OS=Mus musculus OX=10090 GN=Mrps28 PE=1 SV=1
Protein PML OS=Mus musculus OX=10090 GN=Pml PE=1 SV=3
Ubiquitin carboxyl-terminal hydrolase OS=Mus musculus OX=10090 GN=Usp4 PE=1 SV=1
39S ribosomal protein L20, mitochondrial OS=Mus musculus OX=10090 GN=Mrpl20 PE=1 SV=1
ATPase family, AAA domain-containing 2B OS=Mus musculus OX=10090 GN=Atad2b PE=1 SV=1
H/ACA ribonucleoprotein complex subunit DKC1 OS=Mus musculus OX=10090 GN=Dkc1 PE=1 SV=4
Ear6 protein OS=Mus musculus OX=10090 GN=Ear6 PE=1 SV=1
Histone-lysine N-methyltransferase SETD1B OS=Mus musculus OX=10090 GN=Setd1b PE=1 SV=2
Actin-binding LIM protein 1 OS=Mus musculus OX=10090 GN=Ablim1 PE=1 SV=1
Retroelement silencing factor 1 OS=Mus musculus OX=10090 GN=Resf1 PE=1 SV=2
CWF19-like protein 1 OS=Mus musculus OX=10090 GN=Cwf19l1 PE=1 SV=2
EMILIN-1 OS=Mus musculus OX=10090 GN=Emilin1 PE=1 SV=1
Desmocollin-1 OS=Mus musculus OX=10090 GN=Dsc1 PE=1 SV=2
Eukaryotic translation initiation factor 3 subunit M OS=Mus musculus OX=10090 GN=Eif3m PE=1 SV=1
Zinc finger protein 638 OS=Mus musculus OX=10090 GN=Zfp638 PE=1 SV=1
Chloride channel protein OS=Mus musculus OX=10090 GN=Clcn3 PE=1 SV=1
U3 small nucleolar RNA-associated protein 18 homolog OS=Mus musculus OX=10090 GN=Utp18 PE=1 SV=1
Cytospin-B OS=Mus musculus OX=10090 GN=Specc1 PE=1 SV=1
Protease, serine 1 (trypsin 1) OS=Mus musculus OX=10090 GN=Prss1 PE=1 SV=1
Zinc finger protein 493 OS=Mus musculus OX=10090 GN=Zfp493 PE=4 SV=1
Neutrophil elastase OS=Mus musculus OX=10090 GN=Elane PE=1 SV=1
PDZ and LIM domain protein 7 OS=Mus musculus OX=10090 GN=Pdlim7 PE=1 SV=1
Rho guanine nucleotide exchange factor 40 OS=Mus musculus OX=10090 GN=Arhgef40 PE=1 SV=1
Rho guanine nucleotide exchange factor 12 OS=Mus musculus OX=10090 GN=Arhgef12 PE=1 SV=2
Myosin light chain 3 OS=Mus musculus OX=10090 GN=Myl3 PE=1 SV=4
Rhotekin-2 OS=Mus musculus OX=10090 GN=Rtkn2 PE=1 SV=2
Exocyst complex component 5 OS=Mus musculus OX=10090 GN=Exoc5 PE=1 SV=2
Glycerol-3-phosphate acyltransferase 1, mitochondrial OS=Mus musculus OX=10090 GN=Gpam PE=1 SV=2
Centrosomal protein 192 OS=Mus musculus OX=10090 GN=Cep192 PE=1 SV=1
Phosphatidylinositol 3-kinase regulatory subunit alpha OS=Mus musculus OX=10090 GN=Pik3r1 PE=1 SV=2
Recombining-binding protein suppressor of hairless OS=Mus musculus OX=10090 GN=Rbpj PE=1 SV=1
Tumor necrosis factor receptor type 1-associated DEATH domain protein OS=Mus musculus OX=10090 GN=Tradc

Liprin-beta-1 (Fragment) OS=Mus musculus OX=10090 GN=Ppfibp1 PE=1 SV=1
Predicted gene 7324 OS=Mus musculus OX=10090 GN=Gm7324 PE=4 SV=1
Cleavage and polyadenylation specificity factor subunit 3 OS=Mus musculus OX=10090 GN=Cpsf3 PE=1 SV=2
Inosine triphosphate pyrophosphatase OS=Mus musculus OX=10090 GN=Itpa PE=1 SV=2
Protein fem-1 homolog B OS=Mus musculus OX=10090 GN=Fem1b PE=1 SV=1
AT-hook DNA-binding motif-containing protein 1 OS=Mus musculus OX=10090 GN=Ahdc1 PE=1 SV=1
Zinc finger protein 7 OS=Mus musculus OX=10090 GN=Zfp7 PE=1 SV=1
NK-tumor recognition protein OS=Mus musculus OX=10090 GN=Nktr PE=1 SV=4
Zinc finger protein 629 OS=Mus musculus OX=10090 GN=Znf629 PE=2 SV=2
DNA dC->dU-editing enzyme APOBEC-3 OS=Mus musculus OX=10090 GN=Apobec3 PE=1 SV=1
Trafficking protein particle complex subunit 13 OS=Mus musculus OX=10090 GN=Trappc13 PE=2 SV=1
SWI/SNF-related matrix-associated actin-dependent regulator of chromatin subfamily D member 1 OS=Mus mus
GRAM domain-containing protein 4 OS=Mus musculus OX=10090 GN=Gramd4 PE=1 SV=1
39S ribosomal protein L21, mitochondrial OS=Mus musculus OX=10090 GN=Mrpl21 PE=1 SV=1
60S ribosomal protein L18 OS=Mus musculus OX=10090 GN=Rpl18 PE=1 SV=3
Nucleoside diphosphate kinase B OS=Mus musculus OX=10090 GN=Nme2 PE=1 SV=1
Leucine-rich repeat-containing protein 23 OS=Mus musculus OX=10090 GN=Lrrc23 PE=1 SV=1
Transcription initiation factor TFIID subunit 10 OS=Mus musculus OX=10090 GN=Taf10 PE=1 SV=1
Signal recognition particle subunit SRP68 OS=Mus musculus OX=10090 GN=Srp68 PE=1 SV=2
Microtubule-actin cross-linking factor 1 (Fragment) OS=Mus musculus OX=10090 GN=Macf1 PE=1 SV=6
Caldesmon 1 OS=Mus musculus OX=10090 GN=Cald1 PE=1 SV=1
Leukocyte cell-derived chemotaxin-2 OS=Mus musculus OX=10090 GN=Lect2 PE=1 SV=1
Serine/threonine-protein kinase 24 OS=Mus musculus OX=10090 GN=Stk24 PE=1 SV=1
Myosin light chain 1/3, skeletal muscle isoform OS=Mus musculus OX=10090 GN=Myl1 PE=1 SV=2
39S ribosomal protein L24, mitochondrial OS=Mus musculus OX=10090 GN=Mrpl24 PE=1 SV=1
Protein-glutamine gamma-glutamyltransferase K OS=Mus musculus OX=10090 GN=Tgm1 PE=1 SV=2
PH and SEC7 domain-containing protein 3 OS=Mus musculus OX=10090 GN=Psd3 PE=1 SV=1
Coronin-1B OS=Mus musculus OX=10090 GN=Coro1b PE=1 SV=1
THO complex subunit 5 homolog OS=Mus musculus OX=10090 GN=Thoc5 PE=1 SV=2
Ubiquitin carboxyl-terminal hydrolase OS=Mus musculus OX=10090 GN=Usp14 PE=1 SV=1
DnaJ homolog subfamily B member 6 OS=Mus musculus OX=10090 GN=Dnajb6 PE=1 SV=1
Very-long-chain 3-oxoacyl-CoA reductase OS=Mus musculus OX=10090 GN=Hsd17b12 PE=1 SV=1
Cytohesin-1 (Fragment) OS=Mus musculus OX=10090 GN=Cyth1 PE=1 SV=1
Nucleolar protein of 40 kDa OS=Mus musculus OX=10090 GN=Zcchc17 PE=1 SV=1
Mediator of RNA polymerase II transcription subunit 31 OS=Mus musculus OX=10090 GN=Med31 PE=1 SV=2
Transcription elongation factor A protein 1 OS=Mus musculus OX=10090 GN=Tcea1 PE=1 SV=2
Obg-like ATPase 1 OS=Mus musculus OX=10090 GN=Ola1 PE=1 SV=1
GRB2-associated-binding protein 1 OS=Mus musculus OX=10090 GN=Gab1 PE=1 SV=2
Aconitate hydratase, mitochondrial OS=Mus musculus OX=10090 GN=Aco2 PE=1 SV=1
Zinc finger and SCAN domain-containing protein 2 OS=Mus musculus OX=10090 GN=Zscan2 PE=1 SV=1
E3 ISG15--protein ligase Herc6 OS=Mus musculus OX=10090 GN=Herc6 PE=2 SV=1
Serine/arginine-rich-splicing factor 1 (Fragment) OS=Mus musculus OX=10090 GN=Srsf1 PE=1 SV=1
F-actin monooxygenase OS=Mus musculus OX=10090 GN=Mical3 PE=1 SV=1
SS18-like protein 2 OS=Mus musculus OX=10090 GN=Ss18l2 PE=3 SV=1
Splicing factor, proline- and glutamine-rich OS=Mus musculus OX=10090 GN=Sfpq PE=1 SV=1

Periaxin (Fragment) OS=Mus musculus OX=10090 GN=Prx PE=1 SV=1
Microfibrillar-associated protein 1B OS=Mus musculus OX=10090 GN=Mfap1b PE=1 SV=1
Eukaryotic translation initiation factor 3 subunit C OS=Mus musculus OX=10090 GN=Eif3c PE=1 SV=1
Clathrin heavy chain 1 OS=Mus musculus OX=10090 GN=Cltc PE=1 SV=3
Retinitis pigmentosa 9 protein homolog OS=Mus musculus OX=10090 GN=rp9 PE=1 SV=1
Signal sequence receptor subunit alpha (Fragment) OS=Mus musculus OX=10090 GN=Ssr1 PE=1 SV=1
Pre-mRNA 3'-end-processing factor FIP1 OS=Mus musculus OX=10090 GN=Fip1l1 PE=1 SV=1
Putative Polycomb group protein ASXL2 OS=Mus musculus OX=10090 GN=Asxl2 PE=1 SV=1
F-box-like/WD repeat-containing protein TBL1XR1 OS=Mus musculus OX=10090 GN=Tbl1xr1 PE=1 SV=1
Zinc finger CCCH domain-containing protein 18 (Fragment) OS=Mus musculus OX=10090 GN=Zc3h18 PE=1 SV=1
ATP synthase membrane subunit f OS=Mus musculus OX=10090 GN=Atp5j2 PE=1 SV=1
Patatin-like phospholipase domain-containing protein 6 (Fragment) OS=Mus musculus OX=10090 GN=Pnpla6 PE=
Protein unc-13 homolog D OS=Mus musculus OX=10090 GN=Unc13d PE=1 SV=2
EF-hand calcium-binding domain-containing protein 7 (Fragment) OS=Mus musculus OX=10090 GN=Efcab7 PE=1
Beta-enolase OS=Mus musculus OX=10090 GN=Eno3 PE=1 SV=3
Girdin OS=Mus musculus OX=10090 GN=Ccdc88a PE=1 SV=1
Nuclear factor 1 OS=Mus musculus OX=10090 GN=Nfic PE=1 SV=1
Centrosomal protein of 78 kDa OS=Mus musculus OX=10090 GN=Cep78 PE=2 SV=1
Rap guanine nucleotide exchange factor (GEF) 1 OS=Mus musculus OX=10090 GN=Rapgef1 PE=1 SV=1
Serine protease HTRA1 OS=Mus musculus OX=10090 GN=Htra1 PE=1 SV=2
Rho-associated protein kinase 1 OS=Mus musculus OX=10090 GN=Rock1 PE=1 SV=1
Zinc finger protein 748 OS=Mus musculus OX=10090 GN=Zfp748 PE=1 SV=1
Ubinuclein-1 OS=Mus musculus OX=10090 GN=Ubn1 PE=2 SV=1
Vang-like protein 1 OS=Mus musculus OX=10090 GN=Vangl1 PE=1 SV=2
Immunoglobulin kappa variable 5-39 OS=Mus musculus OX=10090 GN=Igkv5-39 PE=1 SV=7
Voltage-gated hydrogen channel 1 OS=Mus musculus OX=10090 GN=Hvcn1 PE=1 SV=2
General transcription factor IIIC, polypeptide 3 OS=Mus musculus OX=10090 GN=Gtf3c3 PE=1 SV=1
Elongation factor 1-alpha 1 OS=Mus musculus OX=10090 GN=Eef1a1 PE=1 SV=3
Ribosomal oxygenase 2 OS=Mus musculus OX=10090 GN=Riox2 PE=1 SV=2
PIH1 domain-containing protein 1 OS=Mus musculus OX=10090 GN=Pih1d1 PE=1 SV=1
Protein virilizer homolog OS=Mus musculus OX=10090 GN=Virma PE=1 SV=1
Probable global transcription activator SNF2L2 OS=Mus musculus OX=10090 GN=Smarca2 PE=1 SV=1
39S ribosomal protein L18, mitochondrial OS=Mus musculus OX=10090 GN=Mrpl18 PE=1 SV=1
Serine/arginine-rich-splicing factor 10 OS=Mus musculus OX=10090 GN=Srsf10 PE=1 SV=1
Endothelial differentiation-related factor 1 OS=Mus musculus OX=10090 GN=Edf1 PE=1 SV=1
SURF1-like protein (Fragment) OS=Mus musculus OX=10090 GN=Surf1 PE=1 SV=1
Microtubule cross-linking factor 1 OS=Mus musculus OX=10090 GN=Mtcl1 PE=1 SV=1
NEDD4-binding protein 3 OS=Mus musculus OX=10090 GN=N4bp3 PE=1 SV=1
Palmdelphin (Fragment) OS=Mus musculus OX=10090 GN=Palmd PE=1 SV=8
Tetratricopeptide repeat protein 7A OS=Mus musculus OX=10090 GN=Ttc7a PE=1 SV=1
Histone H2AX OS=Mus musculus OX=10090 GN=H2ax PE=1 SV=2
Ankyrin repeat and MYND domain-containing protein 2 OS=Mus musculus OX=10090 GN=Ankmy2 PE=1 SV=1
Protein HIRA OS=Mus musculus OX=10090 GN=Hira PE=1 SV=3
Pleckstrin homology domain-containing family F member 1 OS=Mus musculus OX=10090 GN=Plekhf1 PE=2 SV=1
Eukaryotic translation initiation factor 3 subunit E OS=Mus musculus OX=10090 GN=Eif3e PE=1 SV=1

182 kDa tankyrase-1-binding protein (Fragment) OS=Mus musculus OX=10090 GN=Tnks1bp1 PE=1 SV=1
SECIS binding protein 2-like OS=Mus musculus OX=10090 GN=Secisbp2l PE=1 SV=1
Thioredoxin-like protein 1 OS=Mus musculus OX=10090 GN=Txnl1 PE=1 SV=1
Retinoic acid receptor alpha OS=Mus musculus OX=10090 GN=Rara PE=1 SV=1
Rab3 GTPase-activating protein catalytic subunit OS=Mus musculus OX=10090 GN=Rab3gap1 PE=1 SV=4
Sarcalumenin OS=Mus musculus OX=10090 GN=Srl PE=1 SV=2
Nostrin OS=Mus musculus OX=10090 GN=Nostrin PE=1 SV=2
Dimethylaniline monooxygenase [N-oxide-forming] 3 OS=Mus musculus OX=10090 GN=Fmo3 PE=1 SV=1
3'-5' exoribonuclease 1 OS=Mus musculus OX=10090 GN=Eri1 PE=1 SV=2
Type II inositol 1,4,5-trisphosphate 5-phosphatase OS=Mus musculus OX=10090 GN=Inpp5b PE=1 SV=1
MISP family member 3 OS=Mus musculus OX=10090 GN=Misp3 PE=1 SV=1
Disco-interacting protein 2 homolog C OS=Mus musculus OX=10090 GN=Dip2c PE=1 SV=1
Kelch repeat and BTB domain-containing protein 11 OS=Mus musculus OX=10090 GN=Kbtbd11 PE=1 SV=3
Protein Wnt OS=Mus musculus OX=10090 GN=Wnt9a PE=1 SV=1
39S ribosomal protein L40, mitochondrial OS=Mus musculus OX=10090 GN=Mrpl40 PE=1 SV=2
Dolichyl-diphosphooligosaccharide--protein glycosyltransferase subunit 1 OS=Mus musculus OX=10090 GN=Rpn1
Dual specificity protein phosphatase OS=Mus musculus OX=10090 GN=Dusp3 PE=1 SV=1
Glutathione peroxidase OS=Mus musculus OX=10090 GN=Gpx1 PE=1 SV=1
Peptidyl-tRNA hydrolase ICT1, mitochondrial OS=Mus musculus OX=10090 GN=Mrpl58 PE=1 SV=1
Mitochondrial 2-oxoglutarate/malate carrier protein OS=Mus musculus OX=10090 GN=Slc25a11 PE=1 SV=3
Cyclin-dependent kinase 2 OS=Mus musculus OX=10090 GN=Cdk2 PE=1 SV=2
Ezrin OS=Mus musculus OX=10090 GN=Ezr PE=1 SV=3
28S ribosomal protein S26, mitochondrial OS=Mus musculus OX=10090 GN=Mrps26 PE=1 SV=1
Protein transport protein Sec16A OS=Mus musculus OX=10090 GN=Sec16a PE=1 SV=1
Eosinophil peroxidase OS=Mus musculus OX=10090 GN=Epx PE=1 SV=2
Ras-related protein Rab-44 OS=Mus musculus OX=10090 GN=Rab44 PE=1 SV=2
12S rRNA N4-methylcytidine methyltransferase OS=Mus musculus OX=10090 GN=Mettl15 PE=1 SV=2
Putative RNA-binding protein 15B OS=Mus musculus OX=10090 GN=Rbm15b PE=1 SV=2
Latent-transforming growth factor beta-binding protein 2 OS=Mus musculus OX=10090 GN=Ltbp2 PE=1 SV=1
TBC1 domain family member 2A OS=Mus musculus OX=10090 GN=Tbc1d2 PE=2 SV=1
40S ribosomal protein S15 OS=Mus musculus OX=10090 GN=Rps15 PE=1 SV=2
Polypeptide N-acetylgalactosaminyltransferase OS=Mus musculus OX=10090 GN=Galnt18 PE=1 SV=1
Biorientation of chromosomes in cell division protein 1-like 1 OS=Mus musculus OX=10090 GN=Bod1l PE=1 SV=1
Regulator of G-protein signaling 3 OS=Mus musculus OX=10090 GN=Rgs3 PE=1 SV=2
Nucleolar RNA helicase 2 OS=Mus musculus OX=10090 GN=Ddx21 PE=1 SV=3
Activity-dependent neuroprotector homeobox protein OS=Mus musculus OX=10090 GN=Adnp PE=1 SV=2
Tyrosine-protein phosphatase non-receptor type 11 OS=Mus musculus OX=10090 GN=Ptpn11 PE=1 SV=3
Nuclear RNA export factor 1 OS=Mus musculus OX=10090 GN=Nxf1 PE=1 SV=3
Platelet-derived growth factor receptor beta OS=Mus musculus OX=10090 GN=Pdgfrb PE=1 SV=2
Zinc finger protein 346 OS=Mus musculus OX=10090 GN=Znf346 PE=1 SV=1
Calsequestrin-2 OS=Mus musculus OX=10090 GN=Casq2 PE=1 SV=3
Myc-associated zinc finger protein OS=Mus musculus OX=10090 GN=Maz PE=1 SV=1
Serine/threonine-protein kinase TAO2 OS=Mus musculus OX=10090 GN=Taok2 PE=1 SV=3
Mortality factor 4-like protein 1 OS=Mus musculus OX=10090 GN=Morf4l1 PE=1 SV=2
Plasma membrane calcium-transporting ATPase 4 OS=Mus musculus OX=10090 GN=Atp2b4 PE=1 SV=1

Rho guanine nucleotide exchange factor (GEF) 11 OS=Mus musculus OX=10090 GN=Arhgef11 PE=1 SV=1
Protein LLP homolog OS=Mus musculus OX=10090 GN=Llph PE=1 SV=1
Myosin light chain 4 OS=Mus musculus OX=10090 GN=Myl4 PE=1 SV=1
Guanine nucleotide-binding protein subunit alpha-12 (Fragment) OS=Mus musculus OX=10090 GN=Gna12 PE=1 S
Signal-induced proliferation-associated protein 1 OS=Mus musculus OX=10090 GN=Sipa1 PE=1 SV=1
FGFR1 oncogene partner 2 homolog OS=Mus musculus OX=10090 GN=Fgfr1op2 PE=1 SV=1
Sorting nexin-33 OS=Mus musculus OX=10090 GN=Snx33 PE=1 SV=1
Receptor-type tyrosine-protein phosphatase OS=Mus musculus OX=10090 GN=Ptpre PE=1 SV=1
Lipid droplet-regulating VLDL assembly factor AUP1 OS=Mus musculus OX=10090 GN=Aup1 PE=1 SV=1
Probable ribosome biogenesis protein RLP24 OS=Mus musculus OX=10090 GN=Rsl24d1 PE=2 SV=1
Sialic acid-binding Ig-like lectin 10 OS=Mus musculus OX=10090 GN=Siglec10 PE=1 SV=1
Beta-catenin-like protein 1 OS=Mus musculus OX=10090 GN=Ctnnbl1 PE=1 SV=1
SAGA-associated factor 29 OS=Mus musculus OX=10090 GN=Sgf29 PE=2 SV=1
Structural maintenance of chromosomes protein 1A OS=Mus musculus OX=10090 GN=Smc1a PE=1 SV=4
TRMT1-like protein OS=Mus musculus OX=10090 GN=Trmt1l PE=1 SV=1
Electron transfer flavoprotein subunit alpha, mitochondrial OS=Mus musculus OX=10090 GN=Etfa PE=1 SV=2
Nuclear receptor subfamily 1 group D member 1 OS=Mus musculus OX=10090 GN=Nr1d1 PE=1 SV=1
Myb-binding protein 1A OS=Mus musculus OX=10090 GN=Mybbp1a PE=1 SV=2
Carbonic anhydrase 4 OS=Mus musculus OX=10090 GN=Ca4 PE=1 SV=1
Dolichyl-diphosphooligosaccharide--protein glycosyltransferase subunit 2 OS=Mus musculus OX=10090 GN=Rpn2
MAP kinase-activating death domain protein OS=Mus musculus OX=10090 GN=Madd PE=1 SV=1
CREB/ATF bZIP transcription factor OS=Mus musculus OX=10090 GN=Crebzf PE=4 SV=1
Reelin OS=Mus musculus OX=10090 GN=Reln PE=1 SV=3
Charged multivesicular body protein 3 OS=Mus musculus OX=10090 GN=Chmp3 PE=1 SV=3
Troponin C, slow skeletal and cardiac muscles OS=Mus musculus OX=10090 GN=Tnnc1 PE=1 SV=1
Vascular endothelial growth factor A OS=Mus musculus OX=10090 GN=Vegfa PE=1 SV=2
Ribosomal biogenesis protein LAS1L OS=Mus musculus OX=10090 GN=Las1l PE=1 SV=1
CREB-regulated transcription coactivator 3 OS=Mus musculus OX=10090 GN=Crtc3 PE=1 SV=2
Transmembrane protein 11, mitochondrial OS=Mus musculus OX=10090 GN=Tmem11 PE=1 SV=1
Chromatin modification-related protein MEAF6 OS=Mus musculus OX=10090 GN=Meaf6 PE=1 SV=1
Pre-mRNA-processing-splicing factor 8 OS=Mus musculus OX=10090 GN=Prpf8 PE=1 SV=2
Serine/arginine-rich splicing factor 6 OS=Mus musculus OX=10090 GN=Srsf6 PE=1 SV=1
LIM domain and actin-binding protein 1 OS=Mus musculus OX=10090 GN=Lima1 PE=1 SV=3
Trafficking protein particle complex subunit 3 OS=Mus musculus OX=10090 GN=Trappc3 PE=1 SV=1
RE1-silencing transcription factor OS=Mus musculus OX=10090 GN=Rest PE=1 SV=2
Band 4.1-like protein 1 OS=Mus musculus OX=10090 GN=Epb41l1 PE=1 SV=1
Opioid growth factor receptor OS=Mus musculus OX=10090 GN=Ogfr PE=1 SV=1
Cytospin-A OS=Mus musculus OX=10090 GN=Specc1l PE=1 SV=1
Uncharacterized protein KIAA1522 OS=Mus musculus OX=10090 GN=Kiaa1522 PE=1 SV=1
Nucleolar complex protein 3 homolog OS=Mus musculus OX=10090 GN=Noc3l PE=2 SV=2
Methyltransferase-like protein OS=Mus musculus OX=10090 GN=Mettl8 PE=1 SV=1
Disrupted in schizophrenia 1 homolog OS=Mus musculus OX=10090 GN=Disc1 PE=1 SV=1
Nuclear pore complex protein Nup93 OS=Mus musculus OX=10090 GN=Nup93 PE=1 SV=1
Rho family-interacting cell polarization regulator 1 OS=Mus musculus OX=10090 GN=Ripor1 PE=1 SV=2
Zinc finger protein 746 OS=Mus musculus OX=10090 GN=Zfp746 PE=1 SV=1

Protein TSSC4 OS=Mus musculus OX=10090 GN=Tssc4 PE=1 SV=1
Negative elongation factor B OS=Mus musculus OX=10090 GN=Nelfb PE=1 SV=1
Perilipin-3 OS=Mus musculus OX=10090 GN=Plin3 PE=1 SV=1
Serine/threonine-protein kinase MRCK gamma OS=Mus musculus OX=10090 GN=Cdc42bpg PE=1 SV=2
IQ motif and SEC7 domain-containing protein 2 OS=Mus musculus OX=10090 GN=Iqsec2 PE=1 SV=3
60S ribosomal protein L4 OS=Mus musculus OX=10090 GN=Rpl4 PE=1 SV=3
Uveal autoantigen with coiled-coil domains and ankyrin repeats OS=Mus musculus OX=10090 GN=Uaca PE=1 SV
Constitutive coactivator of PPAR-gamma-like protein 2 OS=Mus musculus OX=10090 GN=Fam120c PE=1 SV=3
Protein Daple OS=Mus musculus OX=10090 GN=Ccdc88c PE=1 SV=1
SRSF protein kinase 1 OS=Mus musculus OX=10090 GN=Srpk1 PE=1 SV=2
Transcription factor SOX-18 OS=Mus musculus OX=10090 GN=Sox18 PE=1 SV=3
Exportin-2 OS=Mus musculus OX=10090 GN=Cse1l PE=1 SV=1
THO complex subunit 1 OS=Mus musculus OX=10090 GN=Thoc1 PE=1 SV=1
Mitochondrial carrier homolog 2 OS=Mus musculus OX=10090 GN=Mtch2 PE=1 SV=1
Phosphodiesterase OS=Mus musculus OX=10090 GN=Pde3b PE=1 SV=1
SUN domain-containing protein 1 OS=Mus musculus OX=10090 GN=Sun1 PE=1 SV=2
Protein S100-A9 OS=Mus musculus OX=10090 GN=S100a9 PE=1 SV=3
Zinc finger CCHC domain-containing protein 7 OS=Mus musculus OX=10090 GN=Zcchc7 PE=2 SV=1
Transcriptional repressor p66-beta OS=Mus musculus OX=10090 GN=Gatad2b PE=1 SV=1
Predicted gene 20431 OS=Mus musculus OX=10090 GN=Gm20431 PE=3 SV=1
Zinc finger and SCAN domain-containing 22 OS=Mus musculus OX=10090 GN=Zscan22 PE=2 SV=1
BTB/POZ domain-containing protein 10 OS=Mus musculus OX=10090 GN=Btbd10 PE=1 SV=1
Selenoprotein S OS=Mus musculus OX=10090 GN=Selenos PE=1 SV=1
Thioredoxin-related transmembrane protein 1 (Fragment) OS=Mus musculus OX=10090 GN=Tmx1 PE=1 SV=1
mRNA export factor OS=Mus musculus OX=10090 GN=Rae1 PE=1 SV=1
PIN2/TERF1-interacting telomerase inhibitor 1 OS=Mus musculus OX=10090 GN=Pinx1 PE=1 SV=2
MIF4G domain-containing protein OS=Mus musculus OX=10090 GN=Mif4gd PE=1 SV=1
Synaptobrevin-2 OS=Mus musculus OX=10090 GN=Vamp2 PE=1 SV=1
Phospholipid transfer protein C2CD2L OS=Mus musculus OX=10090 GN=C2cd2l PE=1 SV=3
H/ACA ribonucleoprotein complex subunit 2 OS=Mus musculus OX=10090 GN=Nhp2 PE=1 SV=1
Caspase activity and apoptosis inhibitor 1 OS=Mus musculus OX=10090 GN=Caap1 PE=1 SV=2
CPED1 OS=Mus musculus OX=10090 GN=Cped1 PE=2 SV=1
Pre-rRNA-processing protein TSR1 homolog OS=Mus musculus OX=10090 GN=Tsr1 PE=1 SV=1
Polyphosphoinositide phosphatase OS=Mus musculus OX=10090 GN=Fig4 PE=1 SV=1
G patch domain-containing protein 2-like OS=Mus musculus OX=10090 GN=Gpatch2l PE=1 SV=1
Microtubule-associated protein OS=Mus musculus OX=10090 GN=Mapt PE=1 SV=1
Germinal center-associated signaling and motility protein OS=Mus musculus OX=10090 GN=Gcsam PE=1 SV=1
Mitogen-activated protein kinase 3 OS=Mus musculus OX=10090 GN=Mapk3 PE=1 SV=5
Rabankyrin-5 OS=Mus musculus OX=10090 GN=Ankfy1 PE=1 SV=2
Tumor necrosis factor ligand superfamily member 13 OS=Mus musculus OX=10090 GN=Tnfsf13 PE=1 SV=1
Protein FAM76A OS=Mus musculus OX=10090 GN=Fam76a PE=2 SV=1
Inhibitor of growth protein 5 OS=Mus musculus OX=10090 GN=Ing5 PE=1 SV=1
Serine/arginine-rich splicing factor 5 OS=Mus musculus OX=10090 GN=Srsf5 PE=1 SV=2
Protein FAM76B OS=Mus musculus OX=10090 GN=Fam76b PE=1 SV=1
Protein SEC13 homolog OS=Mus musculus OX=10090 GN=Sec13 PE=1 SV=3

Mitogen-activated protein kinase 7 OS=Mus musculus OX=10090 GN=Mapk7 PE=1 SV=1
SWI/SNF-related matrix-associated actin-dependent regulator of chromatin subfamily A member 5 OS=Mus mu
Cathepsin W OS=Mus musculus OX=10090 GN=Ctsw PE=1 SV=1
Sentrin-specific protease 2 OS=Mus musculus OX=10090 GN=Senp2 PE=1 SV=2
Myosin-11 OS=Mus musculus OX=10090 GN=Myh11 PE=1 SV=1
Phosphatidylinositol 4-kinase alpha OS=Mus musculus OX=10090 GN=Pi4ka PE=1 SV=2
LIM domain-binding protein 2 OS=Mus musculus OX=10090 GN=Ldb2 PE=1 SV=1
Paralemmin-3 OS=Mus musculus OX=10090 GN=Palm3 PE=1 SV=1
Ras-interacting protein 1 OS=Mus musculus OX=10090 GN=Rasip1 PE=1 SV=3
Zinc finger protein 777 OS=Mus musculus OX=10090 GN=Zfp777 PE=1 SV=1
N-alpha-acetyltransferase 35, NatC auxiliary subunit OS=Mus musculus OX=10090 GN=Naa35 PE=1 SV=1
Protein SPT2 homolog OS=Mus musculus OX=10090 GN=Spty2d1 PE=2 SV=1
5'-3' exoribonuclease 2 OS=Mus musculus OX=10090 GN=Xrn2 PE=1 SV=1
E3 ubiquitin-protein ligase RING2 OS=Mus musculus OX=10090 GN=Rnf2 PE=1 SV=1
Suppressor of SWI4 1 homolog OS=Mus musculus OX=10090 GN=Ppan PE=1 SV=2
BPI fold-containing family B member 1 OS=Mus musculus OX=10090 GN=Bpifb1 PE=2 SV=4
Protein FAM193B OS=Mus musculus OX=10090 GN=Fam193b PE=1 SV=2
tRNA wybutosine-synthesizing protein 3 homolog OS=Mus musculus OX=10090 GN=Tyw3 PE=1 SV=1
Protein SCO2 homolog, mitochondrial OS=Mus musculus OX=10090 GN=Sco2 PE=1 SV=1
E3 ubiquitin-protein ligase UHRF2 OS=Mus musculus OX=10090 GN=Uhrf2 PE=1 SV=1
C-terminal-binding protein 1 OS=Mus musculus OX=10090 GN=Ctbp1 PE=1 SV=1
MAP/microtubule affinity-regulating kinase 3 OS=Mus musculus OX=10090 GN=Mark3 PE=1 SV=2
Histone-lysine N-methyltransferase MECOM (Fragment) OS=Mus musculus OX=10090 GN=Mecom PE=1 SV=1
Trafficking protein particle complex subunit 6B OS=Mus musculus OX=10090 GN=Trappc6b PE=1 SV=1
RNA-binding protein 25 OS=Mus musculus OX=10090 GN=Rbm25 PE=1 SV=2
Hamartin OS=Mus musculus OX=10090 GN=Tsc1 PE=1 SV=1
General transcription factor 3C polypeptide 1 OS=Mus musculus OX=10090 GN=Gtf3c1 PE=1 SV=2
MAP7 domain-containing protein 1 OS=Mus musculus OX=10090 GN=Map7d1 PE=1 SV=1
Ig gamma-1 chain C region, membrane-bound form OS=Mus musculus OX=10090 GN=Ighg1 PE=1 SV=2
High mobility group protein B1 OS=Mus musculus OX=10090 GN=Hmgb1 PE=1 SV=2
Signal recognition particle 9 kDa protein OS=Mus musculus OX=10090 GN=Srp9 PE=1 SV=2
HECT domain E3 ubiquitin protein ligase 4 OS=Mus musculus OX=10090 GN=Hectd4 PE=1 SV=2
Dimethyladenosine transferase 1, mitochondrial OS=Mus musculus OX=10090 GN=Tfb1m PE=1 SV=1
A-kinase anchor protein 13 OS=Mus musculus OX=10090 GN=Akap13 PE=1 SV=1
Pseudouridylate synthase 7 homolog (Fragment) OS=Mus musculus OX=10090 GN=Pus7 PE=1 SV=1
Probable 18S rRNA (guanine-N(7))-methyltransferase OS=Mus musculus OX=10090 GN=Bud23 PE=1 SV=1
Myoferlin OS=Mus musculus OX=10090 GN=Myof PE=1 SV=1
cAMP-dependent protein kinase type II-alpha regulatory subunit OS=Mus musculus OX=10090 GN=Prkar2a PE=1
Dynein light chain roadblock OS=Mus musculus OX=10090 GN=Dynlrb1 PE=1 SV=1
60S ribosomal protein L6 OS=Mus musculus OX=10090 GN=Rpl6 PE=1 SV=3
Pre-mRNA-processing factor 40 homolog A (Fragment) OS=Mus musculus OX=10090 GN=Prpf40a PE=1 SV=1
Phosphatidate phosphatase OS=Mus musculus OX=10090 GN=Lpin1 PE=1 SV=1
Myeloid cell nuclear differentiation antigen-like protein OS=Mus musculus OX=10090 GN=Mndal PE=1 SV=1
Endoplasmic reticulum transmembrane helix translocase OS=Mus musculus OX=10090 GN=Atp13a1 PE=1 SV=2
U4/U6.U5 tri-snRNP-associated protein 2 OS=Mus musculus OX=10090 GN=Usp39 PE=1 SV=2

Claudin-18 OS=Mus musculus OX=10090 GN=Cldn18 PE=1 SV=1
Cdc42-interacting protein 4 OS=Mus musculus OX=10090 GN=Trip10 PE=1 SV=2
Mothers against decapentaplegic homolog 6 OS=Mus musculus OX=10090 GN=Smad6 PE=1 SV=1
E3 ubiquitin-protein ligase LRSAM1 OS=Mus musculus OX=10090 GN=Lrsam1 PE=1 SV=1
GTP-binding protein REM 1 OS=Mus musculus OX=10090 GN=Rem1 PE=1 SV=1
60S ribosomal protein L38 OS=Mus musculus OX=10090 GN=Rpl38 PE=1 SV=3
Trafficking protein particle complex 8 OS=Mus musculus OX=10090 GN=Trappc8 PE=1 SV=2
Serine-threonine kinase receptor-associated protein OS=Mus musculus OX=10090 GN=Strap PE=1 SV=2
Tropomyosin alpha-1 chain OS=Mus musculus OX=10090 GN=Tpm1 PE=1 SV=1
RNA exonuclease 4 OS=Mus musculus OX=10090 GN=Rexo4 PE=2 SV=2
Transcription factor EC OS=Mus musculus OX=10090 GN=Tfec PE=1 SV=1
Mitochondrial tRNA-specific 2-thiouridylase 1 OS=Mus musculus OX=10090 GN=Trmu PE=1 SV=1
Crossover junction endonuclease MUS81 OS=Mus musculus OX=10090 GN=Mus81 PE=1 SV=2
Peroxidasin homolog OS=Mus musculus OX=10090 GN=Pxdn PE=1 SV=2
Alpha-1-syntrophin OS=Mus musculus OX=10090 GN=Snta1 PE=1 SV=1
PAN2-PAN3 deadenylation complex subunit PAN3 OS=Mus musculus OX=10090 GN=Pan3 PE=1 SV=1
Ras GTPase-activating protein-binding protein 1 OS=Mus musculus OX=10090 GN=G3bp1 PE=1 SV=1
RCR-type E3 ubiquitin transferase (Fragment) OS=Mus musculus OX=10090 GN=Mycbp2 PE=1 SV=1
Death domain-associated protein 6 OS=Mus musculus OX=10090 GN=Daxx PE=1 SV=1
Alpha-S2-casein-like B OS=Mus musculus OX=10090 GN=Csn1s2b PE=3 SV=1
Guanine nucleotide-binding protein subunit alpha-13 OS=Mus musculus OX=10090 GN=Gna13 PE=1 SV=1
Adipocyte enhancer-binding protein 1 OS=Mus musculus OX=10090 GN=Aebp1 PE=1 SV=1
Flotillin-1 OS=Mus musculus OX=10090 GN=Flot1 PE=1 SV=1
Inositol 1,4,5-trisphosphate receptor type 1 OS=Mus musculus OX=10090 GN=Itpr1 PE=1 SV=2
Vesicle-associated membrane protein-associated protein B OS=Mus musculus OX=10090 GN=Vapb PE=1 SV=3
Eukaryotic translation initiation factor 3 subunit F OS=Mus musculus OX=10090 GN=Eif3f PE=1 SV=2
Cleavage and polyadenylation specificity factor subunit 2 OS=Mus musculus OX=10090 GN=Cpsf2 PE=1 SV=1
SLIT-ROBO Rho GTPase-activating protein 1 OS=Mus musculus OX=10090 GN=Srgap1 PE=1 SV=1
Prefoldin subunit 5 OS=Mus musculus OX=10090 GN=Pfdn5 PE=1 SV=1
Protein kinase C iota type OS=Mus musculus OX=10090 GN=Prkci PE=1 SV=3
Vacuolar protein sorting-associated protein 11 homolog OS=Mus musculus OX=10090 GN=Vps11 PE=1 SV=1
Angiopoietin-1 OS=Mus musculus OX=10090 GN=Angpt1 PE=1 SV=2
Calmodulin-regulated spectrin-associated protein 2 OS=Mus musculus OX=10090 GN=Camsap2 PE=1 SV=1
Serine-rich coiled-coil domain-containing protein 2 OS=Mus musculus OX=10090 GN=Ccser2 PE=1 SV=1
N-alpha-acetyltransferase 15, NatA auxiliary subunit OS=Mus musculus OX=10090 GN=Naa15 PE=1 SV=1
MRG/MORF4L-binding protein OS=Mus musculus OX=10090 GN=Mrgbp PE=1 SV=2
Pumilio homolog 3 OS=Mus musculus OX=10090 GN=Pum3 PE=1 SV=1
Enoyl-CoA hydratase, mitochondrial OS=Mus musculus OX=10090 GN=Echs1 PE=1 SV=1
Mediator of RNA polymerase II transcription subunit 14 OS=Mus musculus OX=10090 GN=Med14 PE=1 SV=1
Protein-serine/threonine phosphatase (Fragment) OS=Mus musculus OX=10090 GN=Ppp4c PE=1 SV=1
Uncharacterized protein C2orf42 homolog OS=Mus musculus OX=10090 PE=2 SV=1
Cyclic AMP-dependent transcription factor ATF-2 OS=Mus musculus OX=10090 GN=Atf2 PE=1 SV=2
ADP-ribosyl cyclase/cyclic ADP-ribose hydrolase 2 OS=Mus musculus OX=10090 GN=Bst1 PE=1 SV=1
Double-strand-break repair protein rad21 homolog OS=Mus musculus OX=10090 GN=Rad21 PE=1 SV=3
RING finger protein unkempt homolog OS=Mus musculus OX=10090 GN=Unk PE=1 SV=1

Zinc finger protein 809 OS=Mus musculus OX=10090 GN=Zfp809 PE=2 SV=1
Histone H1.2 OS=Mus musculus OX=10090 GN=H1-2 PE=1 SV=2
U1 small nuclear ribonucleoprotein C OS=Mus musculus OX=10090 GN=Snrpc PE=1 SV=1
Cell cycle and apoptosis regulator protein 2 OS=Mus musculus OX=10090 GN=Ccar2 PE=1 SV=2
Dihydropyrimidinase-related protein 2 OS=Mus musculus OX=10090 GN=Dpysl2 PE=1 SV=2
DnaJ homolog subfamily C member 9 OS=Mus musculus OX=10090 GN=Dnajc9 PE=1 SV=2
Diphosphoinositol polyphosphate phosphohydrolase 1 OS=Mus musculus OX=10090 GN=Nudt3 PE=1 SV=1
Leucine-rich repeat and calponin homology domain-containing protein 4 OS=Mus musculus OX=10090 GN=Lrch4
Oxysterols receptor LXR-beta OS=Mus musculus OX=10090 GN=Nr1h2 PE=1 SV=1
KH domain-containing, RNA-binding, signal transduction-associated protein 1 OS=Mus musculus OX=10090 GN=I
Cingulin-like 1 OS=Mus musculus OX=10090 GN=Cgnl1 PE=1 SV=1
Processing of precursor 1, ribonuclease P/MRP family, (S. cerevisiae) OS=Mus musculus OX=10090 GN=Pop1 PE=
Catenin alpha-1 OS=Mus musculus OX=10090 GN=Ctnna1 PE=1 SV=1
Snf2-related CREBBP activator protein OS=Mus musculus OX=10090 GN=Srcap PE=1 SV=1
Microtubule-actin cross-linking factor 1 OS=Mus musculus OX=10090 GN=Macf1 PE=1 SV=1
Centrosomal protein CCDC61 OS=Mus musculus OX=10090 GN=Ccdc61 PE=1 SV=1
Integrator complex subunit 14 OS=Mus musculus OX=10090 GN=Ints14 PE=1 SV=1
HECT-type E3 ubiquitin transferase OS=Mus musculus OX=10090 GN=Hectd1 PE=1 SV=1
U4/U6 small nuclear ribonucleoprotein Prp4 OS=Mus musculus OX=10090 GN=Prpf4 PE=1 SV=1
Disabled homolog 2-interacting protein OS=Mus musculus OX=10090 GN=Dab2ip PE=1 SV=1
Cytochrome b-c1 complex subunit 6, mitochondrial OS=Mus musculus OX=10090 GN=Uqcrh PE=1 SV=2
NADH dehydrogenase [ubiquinone] flavoprotein 1, mitochondrial OS=Mus musculus OX=10090 GN=Ndufv1 PE=1
CapZ-interacting protein OS=Mus musculus OX=10090 GN=Rcsd1 PE=1 SV=1
Trafficking protein particle complex subunit 10 OS=Mus musculus OX=10090 GN=Trappc10 PE=1 SV=3
Predicted gene, 49318 (Fragment) OS=Mus musculus OX=10090 GN=Gm49318 PE=4 SV=1
Endoplasmin OS=Mus musculus OX=10090 GN=Hsp90b1 PE=1 SV=2
Heterogeneous nuclear ribonucleoprotein M OS=Mus musculus OX=10090 GN=Hnrnpm PE=1 SV=3
(3R)-3-hydroxyacyl-CoA dehydrogenase OS=Mus musculus OX=10090 GN=Hsd17b8 PE=1 SV=2
F-box/WD repeat-containing protein 11 OS=Mus musculus OX=10090 GN=Fbxw11 PE=1 SV=1
Histone-lysine N-methyltransferase SETD1A OS=Mus musculus OX=10090 GN=Setd1a PE=1 SV=1
Tudor-interacting repair regulator protein OS=Mus musculus OX=10090 GN=Nudt16l1 PE=1 SV=1
Transient receptor potential cation channel subfamily V member 4 OS=Mus musculus OX=10090 GN=Trpv4 PE=1
RNA helicase OS=Mus musculus OX=10090 GN=Dhx30 PE=1 SV=1
Superoxide dismutase [Cu-Zn] OS=Mus musculus OX=10090 GN=Sod1 PE=1 SV=2
Zinc finger and BTB domain-containing protein 14 OS=Mus musculus OX=10090 GN=Zbtb14 PE=2 SV=1
60S ribosomal protein L30 OS=Mus musculus OX=10090 GN=Rpl30 PE=1 SV=2
Cyclin-dependent kinase-like 4 OS=Mus musculus OX=10090 GN=Cdkl4 PE=2 SV=1
Proteasome subunit alpha type-7 OS=Mus musculus OX=10090 GN=Psma7 PE=1 SV=1
Lysyl oxidase homolog 2 OS=Mus musculus OX=10090 GN=Loxl2 PE=1 SV=2
SNW domain-containing protein 1 OS=Mus musculus OX=10090 GN=Snw1 PE=1 SV=1
Toll-interacting protein OS=Mus musculus OX=10090 GN=Tollip PE=1 SV=1
Radixin OS=Mus musculus OX=10090 GN=Rdx PE=1 SV=3
Zinc finger MYM-type protein 2 OS=Mus musculus OX=10090 GN=Zmym2 PE=1 SV=3
Engulfment and cell motility protein 1 OS=Mus musculus OX=10090 GN=Elmo1 PE=1 SV=2
Cytosolic purine 5'-nucleotidase OS=Mus musculus OX=10090 GN=Nt5c2 PE=1 SV=1

Zinc finger protein ZXDC OS=Mus musculus OX=10090 GN=Zxdc PE=2 SV=1
Beta-actin-like protein 2 OS=Mus musculus OX=10090 GN=Actbl2 PE=1 SV=1
Exosome complex component RRP41 OS=Mus musculus OX=10090 GN=Exosc4 PE=1 SV=3
DENN domain-containing protein 4C OS=Mus musculus OX=10090 GN=Dennd4c PE=1 SV=1
ATP-dependent DNA helicase Q5 OS=Mus musculus OX=10090 GN=Recql5 PE=1 SV=1
F-box/SPRY domain-containing protein 1 OS=Mus musculus OX=10090 GN=Fbxo45 PE=1 SV=2
Mitochondrial import receptor subunit TOM70 OS=Mus musculus OX=10090 GN=Tomm70 PE=1 SV=2
Transforming growth factor beta regulator 1 OS=Mus musculus OX=10090 GN=Tbrg1 PE=1 SV=2
Protein S100-A8 OS=Mus musculus OX=10090 GN=S100a8 PE=1 SV=3
Stromal cell-derived factor 2 OS=Mus musculus OX=10090 GN=Sdf2 PE=1 SV=1
Microtubule-actin cross-linking factor 1 (Fragment) OS=Mus musculus OX=10090 GN=Macf1 PE=1 SV=1
Drebrin-like protein OS=Mus musculus OX=10090 GN=Dbnl PE=1 SV=2
Glypican-1 OS=Mus musculus OX=10090 GN=Gpc1 PE=1 SV=1
Kinesin-associated protein 3 OS=Mus musculus OX=10090 GN=Kifap3 PE=1 SV=1
Serine/threonine-protein kinase Nek3 OS=Mus musculus OX=10090 GN=Nek3 PE=1 SV=2
E3 ubiquitin-protein ligase UBR1 OS=Mus musculus OX=10090 GN=Ubr1 PE=1 SV=2
Phosphofurin acidic cluster sorting protein 1 OS=Mus musculus OX=10090 GN=Pacs1 PE=1 SV=2
Krueppel-like factor 10 OS=Mus musculus OX=10090 GN=Klf10 PE=1 SV=1
5'-3' exoribonuclease 1 OS=Mus musculus OX=10090 GN=Xrn1 PE=1 SV=1
Lipoamide acyltransferase component of branched-chain alpha-keto acid dehydrogenase complex, mitochondrial
Survival of motor neuron-related-splicing factor 30 OS=Mus musculus OX=10090 GN=Smndc1 PE=1 SV=1
Inactive rhomboid protein 1 OS=Mus musculus OX=10090 GN=Rhbdf1 PE=1 SV=2
DNA-directed RNA polymerase II subunit RPB11 OS=Mus musculus OX=10090 GN=Polr2j PE=1 SV=1
LIM and SH3 domain protein 1 OS=Mus musculus OX=10090 GN=Lasp1 PE=1 SV=1
Heterogeneous nuclear ribonucleoprotein A3 OS=Mus musculus OX=10090 GN=Hnrnpa3 PE=1 SV=1
Caseinolytic peptidase B protein homolog OS=Mus musculus OX=10090 GN=Clpb PE=1 SV=1
Coiled-coil domain-containing protein 153 OS=Mus musculus OX=10090 GN=Ccdc153 PE=3 SV=1
MAGE family member D2 OS=Mus musculus OX=10090 GN=Maged2 PE=1 SV=1
CXXC-type zinc finger protein 1 OS=Mus musculus OX=10090 GN=Cxxc1 PE=1 SV=1
Lysocardiolipin acyltransferase 1 OS=Mus musculus OX=10090 GN=Lclat1 PE=1 SV=2
CPN10-like protein OS=Mus musculus OX=10090 GN=Hspe1-rs1 PE=3 SV=1
Pleckstrin homology-like domain family B member 1 OS=Mus musculus OX=10090 GN=Phldb1 PE=1 SV=1
Regulatory factor X-associated protein OS=Mus musculus OX=10090 GN=Rfxap PE=2 SV=2
Zinc finger protein 64 OS=Mus musculus OX=10090 GN=Zfp64 PE=1 SV=1
Heterogeneous nuclear ribonucleoprotein D-like (Fragment) OS=Mus musculus OX=10090 GN=Hnrnpdl PE=1 SV=
60S ribosomal protein L22 OS=Mus musculus OX=10090 GN=Rpl22 PE=1 SV=2
Ras association (RalGDS/AF-6) and pleckstrin homology domains 1 OS=Mus musculus OX=10090 GN=Raph1 PE=
ADP-ribosylation factor GTPase-activating protein 3 OS=Mus musculus OX=10090 GN=Arfgap3 PE=1 SV=1
UPF3 regulator of nonsense transcripts homolog A (Yeast) OS=Mus musculus OX=10090 GN=Upf3a PE=1 SV=1
Luc7-like protein 3 OS=Mus musculus OX=10090 GN=Luc7l3 PE=1 SV=1
Transcriptional adapter 1 OS=Mus musculus OX=10090 GN=Tada1 PE=2 SV=1
Acyl-coenzyme A oxidase-like protein OS=Mus musculus OX=10090 GN=Acoxl PE=1 SV=1
Serine/threonine-protein phosphatase 6 catalytic subunit OS=Mus musculus OX=10090 GN=Ppp6c PE=1 SV=1
Dual-specificity kinase OS=Mus musculus OX=10090 GN=Dyrk1a PE=1 SV=1
2'-5'-oligoadenylate synthase-like protein 2 OS=Mus musculus OX=10090 GN=Oasl2 PE=1 SV=2

Phospholipid scramblase 4 OS=Mus musculus OX=10090 GN=Plscr4 PE=2 SV=2
CAP-Gly domain-containing linker protein 1 OS=Mus musculus OX=10090 GN=Clip1 PE=1 SV=1
Mitogen-activated protein kinase kinase kinase 3 OS=Mus musculus OX=10090 GN=Map3k3 PE=1 SV=1
Glutaredoxin-related protein 5, mitochondrial OS=Mus musculus OX=10090 GN=Glrx5 PE=1 SV=2
60S ribosomal protein L13a OS=Mus musculus OX=10090 GN=Gm45713 PE=2 SV=1
Histone H3.3 OS=Mus musculus OX=10090 GN=H3-3a PE=1 SV=2
Myeloblastin OS=Mus musculus OX=10090 GN=Prtn3 PE=1 SV=1
Transforming acidic coiled-coil-containing protein 1 OS=Mus musculus OX=10090 GN=Tacc1 PE=1 SV=1
Coiled-coil and C2 domain-containing protein 1A OS=Mus musculus OX=10090 GN=Cc2d1a PE=1 SV=2
Retinoic acid receptor RXR-beta OS=Mus musculus OX=10090 GN=Rxrb PE=1 SV=1
Cdc42 effector protein 1 OS=Mus musculus OX=10090 GN=Cdc42ep1 PE=1 SV=1
Disco-interacting protein 2 homolog A OS=Mus musculus OX=10090 GN=Dip2a PE=1 SV=3
WD repeat-containing protein 74 OS=Mus musculus OX=10090 GN=Wdr74 PE=2 SV=1
28S ribosomal protein S16, mitochondrial OS=Mus musculus OX=10090 GN=Mrps16 PE=1 SV=1
Zinc finger and BTB domain-containing protein 17 OS=Mus musculus OX=10090 GN=Zbtb17 PE=1 SV=2
Vitamin K-dependent gamma-carboxylase OS=Mus musculus OX=10090 GN=Ggcx PE=1 SV=1
Plasmalemma vesicle-associated protein OS=Mus musculus OX=10090 GN=Plvap PE=1 SV=1
MICOS complex subunit Mic60 OS=Mus musculus OX=10090 GN=Immt PE=1 SV=1
Fatty acid-binding protein 5 OS=Mus musculus OX=10090 GN=Fabp5 PE=1 SV=3
Ankyrin-3 OS=Mus musculus OX=10090 GN=Ank3 PE=1 SV=1
Neurofilament light polypeptide OS=Mus musculus OX=10090 GN=Nefl PE=1 SV=5
Short transient receptor potential channel 2 OS=Mus musculus OX=10090 GN=Trpc2 PE=2 SV=2
Transmembrane protein 214 OS=Mus musculus OX=10090 GN=Tmem214 PE=1 SV=1
A-kinase anchor protein 10, mitochondrial OS=Mus musculus OX=10090 GN=Akap10 PE=1 SV=3
Female-lethal(2)D homolog OS=Mus musculus OX=10090 GN=Wtap PE=1 SV=1
RNA-binding region-containing protein 3 (Fragment) OS=Mus musculus OX=10090 GN=Rnpc3 PE=1 SV=1
Helicase-like transcription factor OS=Mus musculus OX=10090 GN=Hltf PE=1 SV=1
Nuclear pore complex protein Nup85 OS=Mus musculus OX=10090 GN=Nup85 PE=1 SV=1
Zinc finger protein 691 OS=Mus musculus OX=10090 GN=Zfp691 PE=1 SV=1
Rho GTPase-activating protein 45 OS=Mus musculus OX=10090 GN=Arhgap45 PE=1 SV=1
Small nuclear ribonucleoprotein Sm D2 OS=Mus musculus OX=10090 GN=Snrpd2 PE=1 SV=1
Nucleolar protein 12 OS=Mus musculus OX=10090 GN=Nol12 PE=1 SV=1
28S ribosomal protein S12, mitochondrial OS=Mus musculus OX=10090 GN=Mrps12 PE=2 SV=1
Protein LSM14 homolog A OS=Mus musculus OX=10090 GN=Lsm14a PE=1 SV=1
Rho GTPase-activating protein 31 OS=Mus musculus OX=10090 GN=Arhgap31 PE=1 SV=1
Protein Wiz OS=Mus musculus OX=10090 GN=Wiz PE=1 SV=2
Vesicle-fusing ATPase OS=Mus musculus OX=10090 GN=Spata5 PE=1 SV=1
Zinc finger protein 436 OS=Mus musculus OX=10090 GN=Znf436 PE=2 SV=1
Pre-mRNA-splicing factor SYF1 OS=Mus musculus OX=10090 GN=Xab2 PE=1 SV=1
RNA helicase aquarius OS=Mus musculus OX=10090 GN=Aqr PE=1 SV=1
Uncharacterized protein KIAA1143 homolog OS=Mus musculus OX=10090 PE=1 SV=1
Zinc finger protein 526 OS=Mus musculus OX=10090 GN=Znf526 PE=2 SV=1
Neurogenic locus notch homolog protein 4 OS=Mus musculus OX=10090 GN=Notch4 PE=1 SV=3
Archvillin OS=Mus musculus OX=10090 GN=Svil PE=1 SV=1
Vacuolar-sorting protein SNF8 OS=Mus musculus OX=10090 GN=Snf8 PE=1 SV=1

Glutaminyl-peptide cyclotransferase OS=Mus musculus OX=10090 GN=Qpct PE=1 SV=1
Heterochromatin protein 1-binding protein 3 (Fragment) OS=Mus musculus OX=10090 GN=Hp1bp3 PE=1 SV=1
Lamina-associated polypeptide 2, isoforms beta/delta/epsilon/gamma OS=Mus musculus OX=10090 GN=Tmpo [illegible]
WD repeat-containing protein 6 OS=Mus musculus OX=10090 GN=Wdr6 PE=1 SV=1
Serine/threonine-protein kinase MRCK beta OS=Mus musculus OX=10090 GN=Cdc42bpb PE=1 SV=2
Ankycorbin OS=Mus musculus OX=10090 GN=Rai14 PE=1 SV=1
Biliverdin reductase A OS=Mus musculus OX=10090 GN=Blvra PE=1 SV=1
LIM zinc-binding domain-containing Nebulette OS=Mus musculus OX=10090 GN=Nebl PE=1 SV=1
Moesin OS=Mus musculus OX=10090 GN=Msn PE=1 SV=3
Serine/threonine-protein kinase D2 OS=Mus musculus OX=10090 GN=Prkd2 PE=1 SV=1
Calcium uniporter protein, mitochondrial OS=Mus musculus OX=10090 GN=Mcu PE=1 SV=2
Tyrosine-protein kinase JAK2 OS=Mus musculus OX=10090 GN=Jak2 PE=1 SV=3
Immunoglobulin kappa chain variable 1-88 (Fragment) OS=Mus musculus OX=10090 GN=Igkv1-88 PE=4 SV=2
Monocarboxylate transporter 4 OS=Mus musculus OX=10090 GN=Slc16a3 PE=1 SV=1
Selenoprotein H OS=Mus musculus OX=10090 GN=Selenoh PE=1 SV=1
Zinc transporter 9 OS=Mus musculus OX=10090 GN=Slc30a9 PE=1 SV=2
Receptor tyrosine-protein kinase erbB-2 OS=Mus musculus OX=10090 GN=Erbb2 PE=1 SV=3
IQ domain-containing protein E OS=Mus musculus OX=10090 GN=Iqce PE=1 SV=1
Crooked neck-like protein 1 OS=Mus musculus OX=10090 GN=Crnkl1 PE=1 SV=1
RNA polymerase II elongation factor ELL OS=Mus musculus OX=10090 GN=Ell PE=2 SV=2
Actin-related protein 2/3 complex subunit 5 OS=Mus musculus OX=10090 GN=Arpc5 PE=1 SV=3
Ferrochelatase, mitochondrial OS=Mus musculus OX=10090 GN=Fech PE=1 SV=2
AH receptor-interacting protein OS=Mus musculus OX=10090 GN=Aip PE=1 SV=1
Transcription factor GATA-6 OS=Mus musculus OX=10090 GN=Gata6 PE=1 SV=3
Troponin T, fast skeletal muscle (Fragment) OS=Mus musculus OX=10090 GN=Tnnt3 PE=1 SV=1
Chromodomain-helicase-DNA-binding protein 6 OS=Mus musculus OX=10090 GN=Chd6 PE=1 SV=1
Glutathione S-transferase kappa 1 OS=Mus musculus OX=10090 GN=Gstk1 PE=1 SV=3
Complex I assembly factor ACAD9, mitochondrial OS=Mus musculus OX=10090 GN=Acad9 PE=1 SV=2
Protein transport protein Sec61 subunit alpha isoform 1 OS=Mus musculus OX=10090 GN=Sec61a1 PE=1 SV=2
Mitotic checkpoint protein BUB3 OS=Mus musculus OX=10090 GN=Bub3 PE=1 SV=1
Tyrosine-protein phosphatase non-receptor type 6 OS=Mus musculus OX=10090 GN=Ptpn6 PE=1 SV=2
Slit homolog 2 protein OS=Mus musculus OX=10090 GN=Slit2 PE=2 SV=2
40S ribosomal protein S25 OS=Mus musculus OX=10090 GN=Rps25 PE=1 SV=1
KN motif and ankyrin repeat domain-containing protein 2 OS=Mus musculus OX=10090 GN=Kank2 PE=1 SV=1
Heterogeneous nuclear ribonucleoprotein L (Fragment) OS=Mus musculus OX=10090 GN=Hnrnpl PE=1 SV=1
Zinc finger, MYND-type-containing 8 OS=Mus musculus OX=10090 GN=Zmynd8 PE=1 SV=1
AFG1-like ATPase OS=Mus musculus OX=10090 GN=Afg1l PE=1 SV=1
Taperin OS=Mus musculus OX=10090 GN=Tprn PE=1 SV=1
Heat shock protein 75 kDa, mitochondrial OS=Mus musculus OX=10090 GN=Trap1 PE=1 SV=1
ATP-dependent 6-phosphofructokinase, muscle type OS=Mus musculus OX=10090 GN=Pfkm PE=1 SV=3
Splicing factor, arginine/serine-rich 19 OS=Mus musculus OX=10090 GN=Scaf1 PE=1 SV=1
Synaptotagmin-like protein 4 OS=Mus musculus OX=10090 GN=Sytl4 PE=1 SV=1
Pleckstrin homology-like domain family B member 2 OS=Mus musculus OX=10090 GN=Phldb2 PE=1 SV=2
Polyadenylate-binding protein 1 OS=Mus musculus OX=10090 GN=Pabpc1 PE=1 SV=2
Embryonal Fyn-associated substrate OS=Mus musculus OX=10090 GN=Efs PE=1 SV=2

V-type proton ATPase subunit D OS=Mus musculus OX=10090 GN=Atp6v1d PE=1 SV=1
Calcium-transporting ATPase OS=Mus musculus OX=10090 GN=Atp2b2 PE=1 SV=1
Protein KHNYN OS=Mus musculus OX=10090 GN=Khnyn PE=2 SV=2
Phosphoinositide 3-kinase adapter protein 1 OS=Mus musculus OX=10090 GN=Pik3ap1 PE=1 SV=1
Cation-independent mannose-6-phosphate receptor OS=Mus musculus OX=10090 GN=Igf2r PE=1 SV=1
Carbonyl reductase [NADPH] 3 OS=Mus musculus OX=10090 GN=Cbr3 PE=1 SV=1
Calcium-regulated heat stable protein 1 OS=Mus musculus OX=10090 GN=Carhsp1 PE=1 SV=1
RIKEN cDNA 2410002F23 gene OS=Mus musculus OX=10090 GN=2410002F23Rik PE=1 SV=1
PHD finger-like domain-containing protein 5A OS=Mus musculus OX=10090 GN=Phf5a PE=1 SV=1
Tight junction protein ZO-1 OS=Mus musculus OX=10090 GN=Tjp1 PE=1 SV=2
Nuclear fragile X mental retardation-interacting protein 1 OS=Mus musculus OX=10090 GN=Nufip1 PE=1 SV=1
GTP-binding protein Rit1 OS=Mus musculus OX=10090 GN=Rit1 PE=1 SV=1
Guanine nucleotide-binding protein-like 3 OS=Mus musculus OX=10090 GN=Gnl3 PE=1 SV=2
Coiled-coil and C2 domain-containing protein 1B (Fragment) OS=Mus musculus OX=10090 GN=Cc2d1b PE=1 SV=
Nardilysin OS=Mus musculus OX=10090 GN=Nrdc PE=1 SV=1
E3 ubiquitin-protein ligase RNF31 OS=Mus musculus OX=10090 GN=Rnf31 PE=1 SV=2
Sorting nexin OS=Mus musculus OX=10090 GN=Snx18 PE=1 SV=1
Actin-related protein 8 OS=Mus musculus OX=10090 GN=Actr8 PE=2 SV=1
Partitioning defective 3 homolog (Fragment) OS=Mus musculus OX=10090 GN=Pard3 PE=1 SV=1
Zinc finger CCCH-type with G patch domain-containing protein OS=Mus musculus OX=10090 GN=Zgpat PE=1 SV=
Serine/threonine-protein kinase WNK1 OS=Mus musculus OX=10090 GN=Wnk1 PE=1 SV=2
Poly(U)-binding-splicing factor PUF60 OS=Mus musculus OX=10090 GN=Puf60 PE=1 SV=2
Mitotic deacetylase-associated SANT domain protein OS=Mus musculus OX=10090 GN=Mideas PE=1 SV=1
Enhancer of polycomb homolog 2 OS=Mus musculus OX=10090 GN=Epc2 PE=2 SV=2
Scavenger receptor class F member 2 OS=Mus musculus OX=10090 GN=Scarf2 PE=1 SV=1
Gamma-tubulin complex component 3 OS=Mus musculus OX=10090 GN=Tubgcp3 PE=1 SV=2
Glucose-fructose oxidoreductase domain-containing protein 1 OS=Mus musculus OX=10090 GN=Gfod1 PE=1 SV=
L-xylulose reductase OS=Mus musculus OX=10090 GN=Dcxr PE=1 SV=1
General transcription factor IIH subunit 3 OS=Mus musculus OX=10090 GN=Gtf2h3 PE=1 SV=1
DnaJ homolog subfamily C member 11 OS=Mus musculus OX=10090 GN=Dnajc11 PE=1 SV=2
Cap-specific mRNA (nucleoside-2'-O-)-methyltransferase 2 OS=Mus musculus OX=10090 GN=Cmtr2 PE=2 SV=1
Macrophage-expressed gene 1 protein OS=Mus musculus OX=10090 GN=Mpeg1 PE=1 SV=1
Complement C1q tumor necrosis factor-related protein 2 OS=Mus musculus OX=10090 GN=C1qtnf2 PE=1 SV=1
Pescadillo homolog OS=Mus musculus OX=10090 GN=Pes1 PE=1 SV=1
Glycogenin OS=Mus musculus OX=10090 GN=Gyg PE=1 SV=1
Zinc finger protein Pegasus OS=Mus musculus OX=10090 GN=Ikzf5 PE=2 SV=1
Arf-GAP with Rho-GAP domain, ANK repeat and PH domain-containing protein 2 OS=Mus musculus OX=10090 G
Transmembrane protein 109 (Fragment) OS=Mus musculus OX=10090 GN=Tmem109 PE=1 SV=1
Probable helicase senataxin OS=Mus musculus OX=10090 GN=Setx PE=1 SV=1
Plakophilin 2 OS=Mus musculus OX=10090 GN=Pkp2 PE=1 SV=1
Tripartite motif-containing 12C OS=Mus musculus OX=10090 GN=Trim12c PE=1 SV=2
Splicing factor 3B subunit 4 OS=Mus musculus OX=10090 GN=Sf3b4 PE=1 SV=1
Signal sequence receptor subunit delta OS=Mus musculus OX=10090 GN=Ssr4 PE=1 SV=1
BRISC and BRCA1-A complex member 1 OS=Mus musculus OX=10090 GN=Babam1 PE=1 SV=1
RNA-binding protein 34 OS=Mus musculus OX=10090 GN=Rbm34 PE=1 SV=1

Platelet factor 4 OS=Mus musculus OX=10090 GN=Pf4 PE=1 SV=1
Exocyst complex component 4 OS=Mus musculus OX=10090 GN=Exoc4 PE=1 SV=2
Non-specific protein-tyrosine kinase OS=Mus musculus OX=10090 GN=Ptk2 PE=1 SV=1
Centromere protein F OS=Mus musculus OX=10090 GN=Cenpf PE=1 SV=1
Vacuolar protein sorting-associated protein 72 homolog OS=Mus musculus OX=10090 GN=Vps72 PE=2 SV=2
Rho GTPase-activating protein 9 OS=Mus musculus OX=10090 GN=Arhgap9 PE=1 SV=2
2-oxoglutarate dehydrogenase, mitochondrial OS=Mus musculus OX=10090 GN=Ogdh PE=1 SV=3
Pyrin and HIN domain-containing protein 1 OS=Mus musculus OX=10090 GN=Pyhin1 PE=1 SV=1
C-1-tetrahydrofolate synthase, cytoplasmic OS=Mus musculus OX=10090 GN=Mthfd1 PE=1 SV=4
Eukaryotic translation initiation factor 4 gamma 1 OS=Mus musculus OX=10090 GN=Eif4g1 PE=1 SV=1
Stromal membrane-associated protein 1 OS=Mus musculus OX=10090 GN=Smap1 PE=1 SV=1
PR domain-containing 2, with ZNF domain OS=Mus musculus OX=10090 GN=Prdm2 PE=1 SV=1
Heterogeneous nuclear ribonucleoprotein U-like protein 2 OS=Mus musculus OX=10090 GN=Hnrnpul2 PE=1 SV=2
Eukaryotic peptide chain release factor GTP-binding subunit ERF3A OS=Mus musculus OX=10090 GN=Gspt1 PE=1
Myeloid-associated differentiation marker OS=Mus musculus OX=10090 GN=Myadm PE=1 SV=2
A-kinase anchor protein 13 (Fragment) OS=Mus musculus OX=10090 GN=Akap13 PE=1 SV=1
Coiled-coil domain-containing protein 171 OS=Mus musculus OX=10090 GN=Ccdc171 PE=2 SV=1
Plakophilin-1 OS=Mus musculus OX=10090 GN=Pkp1 PE=1 SV=1
Serine/threonine-protein kinase mTOR OS=Mus musculus OX=10090 GN=Mtor PE=1 SV=2
Zinc finger protein ubi-d4 OS=Mus musculus OX=10090 GN=Dpf2 PE=1 SV=1
RRP15-like protein OS=Mus musculus OX=10090 GN=Rrp15 PE=1 SV=2
Glyceraldehyde-3-phosphate dehydrogenase OS=Mus musculus OX=10090 GN=Gapdh PE=1 SV=1
Ceramide kinase OS=Mus musculus OX=10090 GN=Cerk PE=1 SV=2
Acylglycerol kinase, mitochondrial OS=Mus musculus OX=10090 GN=Agk PE=1 SV=1
Vascular endothelial zinc finger 1 OS=Mus musculus OX=10090 GN=Vezf1 PE=1 SV=1
Zinc finger MYM-type protein 4 OS=Mus musculus OX=10090 GN=Zmym4 PE=1 SV=1
PH and SEC7 domain-containing protein 4 OS=Mus musculus OX=10090 GN=Psd4 PE=1 SV=1
Fin bud initiation factor homolog OS=Mus musculus OX=10090 GN=Fibin PE=2 SV=1
Aquaporin-5 OS=Mus musculus OX=10090 GN=Aqp5 PE=1 SV=1
Methyl-CpG-binding domain protein 1 (Fragment) OS=Mus musculus OX=10090 GN=Mbd1 PE=1 SV=1
Coiled-coil domain-containing protein 137 (Fragment) OS=Mus musculus OX=10090 GN=Ccdc137 PE=1 SV=1
Histone-lysine N-methyltransferase SETD5 OS=Mus musculus OX=10090 GN=Setd5 PE=1 SV=2
eEF1A lysine and N-terminal methyltransferase OS=Mus musculus OX=10090 GN=EEF1AKNMT PE=1 SV=1
Lethal(2) giant larvae protein homolog 1 OS=Mus musculus OX=10090 GN=Llgl1 PE=1 SV=1
MAPK-interacting and spindle-stabilizing protein-like (Fragment) OS=Mus musculus OX=10090 GN=Mapk1ip1l PE
Carboxyl-terminal PDZ ligand of neuronal nitric oxide synthase protein OS=Mus musculus OX=10090 GN=Nos1ap
Dehydrogenase/reductase SDR family member 1 OS=Mus musculus OX=10090 GN=Dhrs1 PE=1 SV=1
Lymphocyte-specific protein 1 OS=Mus musculus OX=10090 GN=Lsp1 PE=1 SV=1
Sorting nexin-5 OS=Mus musculus OX=10090 GN=Snx5 PE=1 SV=1
E3 ubiquitin-protein ligase TRIP12 OS=Mus musculus OX=10090 GN=Trip12 PE=1 SV=1
Vascular endothelial growth factor receptor 1 OS=Mus musculus OX=10090 GN=Flt1 PE=1 SV=1
Inorganic pyrophosphatase OS=Mus musculus OX=10090 GN=Ppa1 PE=1 SV=1
Adenylate cyclase type 9 OS=Mus musculus OX=10090 GN=Adcy9 PE=1 SV=1
Uridine phosphorylase 1 OS=Mus musculus OX=10090 GN=Upp1 PE=2 SV=2
KAT8 regulatory NSL complex subunit 2 OS=Mus musculus OX=10090 GN=Kansl2 PE=1 SV=1

Polycomb group protein ASXL1 OS=Mus musculus OX=10090 GN=Asxl1 PE=1 SV=1
Engulfment and cell motility protein 2 OS=Mus musculus OX=10090 GN=Elmo2 PE=1 SV=1
UDP-glucose glycoprotein glucosyltransferase 2 OS=Mus musculus OX=10090 GN=Uggt2 PE=1 SV=1
Methylmalonate-semialdehyde dehydrogenase [acylating], mitochondrial OS=Mus musculus OX=10090 GN=Aldh
Disheveled-associated activator of morphogenesis 2 OS=Mus musculus OX=10090 GN=Daam2 PE=1 SV=4
Coronin-2B OS=Mus musculus OX=10090 GN=Coro2b PE=1 SV=2
Transcription termination factor 1 OS=Mus musculus OX=10090 GN=Ttf1 PE=1 SV=2
RNA-binding protein with multiple-splicing OS=Mus musculus OX=10090 GN=Rbpms PE=1 SV=1
40S ribosomal protein S28 (Fragment) OS=Mus musculus OX=10090 GN=Rps28 PE=1 SV=1
Nesprin-1 OS=Mus musculus OX=10090 GN=Syne1 PE=1 SV=1
Transmembrane protein 43 OS=Mus musculus OX=10090 GN=Tmem43 PE=1 SV=1
Probable ATP-dependent RNA helicase DDX41 OS=Mus musculus OX=10090 GN=Ddx41 PE=1 SV=2
MAGUK p55 subfamily member 7 OS=Mus musculus OX=10090 GN=Mpp7 PE=1 SV=2
Conserved oligomeric Golgi complex subunit 6 OS=Mus musculus OX=10090 GN=Cog6 PE=1 SV=2
Chondroitin sulfate proteoglycan 4 OS=Mus musculus OX=10090 GN=Cspg4 PE=1 SV=3
Advanced glycosylation end product-specific receptor OS=Mus musculus OX=10090 GN=Ager PE=1 SV=2
Eukaryotic translation initiation factor 4 gamma 2 OS=Mus musculus OX=10090 GN=Eif4g2 PE=1 SV=2
UHRF1 (ICBP90)-binding protein 1 OS=Mus musculus OX=10090 GN=Uhrf1bp1 PE=1 SV=1
Leucine-rich repeat-containing protein 10 OS=Mus musculus OX=10090 GN=Lrrc10 PE=1 SV=1
tRNA-dihydrouridine(47) synthase [NAD(P)(+)] OS=Mus musculus OX=10090 GN=Dus3l PE=1 SV=1
Neuron navigator 1 OS=Mus musculus OX=10090 GN=Nav1 PE=1 SV=1
Proteasome subunit alpha type-1 OS=Mus musculus OX=10090 GN=Psma1 PE=1 SV=1
RNA-binding protein Raly OS=Mus musculus OX=10090 GN=Raly PE=1 SV=3
Tubulin gamma-1 chain OS=Mus musculus OX=10090 GN=Tubg1 PE=1 SV=1
Protein phosphatase 1 regulatory subunit 12C OS=Mus musculus OX=10090 GN=Ppp1r12c PE=1 SV=1
Cofilin-2 OS=Mus musculus OX=10090 GN=Cfl2 PE=1 SV=1
MOB kinase activator 1B OS=Mus musculus OX=10090 GN=Mob1b PE=1 SV=3
Translation factor Guf1, mitochondrial OS=Mus musculus OX=10090 GN=Guf1 PE=1 SV=1
Tight junction protein ZO-2 OS=Mus musculus OX=10090 GN=Tjp2 PE=1 SV=1
SPT20 SAGA complex component OS=Mus musculus OX=10090 GN=Supt20 PE=1 SV=1
Isocitrate dehydrogenase [NAD] subunit, mitochondrial OS=Mus musculus OX=10090 GN=Idh3a PE=1 SV=1
Protein RRP5 homolog OS=Mus musculus OX=10090 GN=Pdcd11 PE=1 SV=2
Protein Shroom3 OS=Mus musculus OX=10090 GN=Shroom3 PE=1 SV=1
Mannose-1-phosphate guanyltransferase beta OS=Mus musculus OX=10090 GN=Gmppb PE=1 SV=1
Transcription factor IIIA OS=Mus musculus OX=10090 GN=Gtf3a PE=1 SV=2
Methyltransferase-like protein 17, mitochondrial OS=Mus musculus OX=10090 GN=Mettl17 PE=1 SV=1
28S ribosomal protein S35, mitochondrial OS=Mus musculus OX=10090 GN=Mrps35 PE=1 SV=2
NAD-dependent protein deacetylase sirtuin-2 OS=Mus musculus OX=10090 GN=Sirt2 PE=1 SV=2
TRPM8 channel-associated factor 2 OS=Mus musculus OX=10090 GN=Tcaf2 PE=1 SV=1
Upstream stimulatory factor 2 OS=Mus musculus OX=10090 GN=Usf2 PE=1 SV=1
Inactive rhomboid protein 2 OS=Mus musculus OX=10090 GN=Rhbdf2 PE=1 SV=1
AP complex subunit beta OS=Mus musculus OX=10090 GN=Ap1b1 PE=1 SV=1
WASH complex subunit 4 OS=Mus musculus OX=10090 GN=Washc4 PE=1 SV=2
Dynactin subunit 3 OS=Mus musculus OX=10090 GN=Dctn3 PE=1 SV=2
Tetraspanin-8 OS=Mus musculus OX=10090 GN=Tspan8 PE=1 SV=1

Tyrosine--tRNA ligase, cytoplasmic OS=Mus musculus OX=10090 GN=Yars1 PE=1 SV=3
Zinc finger protein 143 OS=Mus musculus OX=10090 GN=Znf143 PE=1 SV=2
40S ribosomal protein S5 OS=Mus musculus OX=10090 GN=Rps5 PE=1 SV=1
Zinc finger protein 651 OS=Mus musculus OX=10090 GN=Zfp651 PE=1 SV=1
Proteasome subunit alpha type-3 OS=Mus musculus OX=10090 GN=Psma3 PE=1 SV=3
Sec1 family domain-containing protein 1 OS=Mus musculus OX=10090 GN=Scfd1 PE=1 SV=1
E3 ubiquitin-protein ligase TRIM56 OS=Mus musculus OX=10090 GN=Trim56 PE=1 SV=1
40S ribosomal protein S13 OS=Mus musculus OX=10090 GN=Rps13 PE=1 SV=1
Cell division cycle-associated 7-like protein OS=Mus musculus OX=10090 GN=Cdca7l PE=1 SV=1
Dual specificity protein kinase CLK1 OS=Mus musculus OX=10090 GN=Clk1 PE=1 SV=2
Pre-mRNA-processing factor 6 OS=Mus musculus OX=10090 GN=Prpf6 PE=1 SV=1
Copper transport protein ATOX1 OS=Mus musculus OX=10090 GN=Atox1 PE=1 SV=1
Methanethiol oxidase OS=Mus musculus OX=10090 GN=Selenbp1 PE=1 SV=2
Beta-1,3-galactosyltransferase 6 OS=Mus musculus OX=10090 GN=B3galt6 PE=2 SV=1
Alpha-soluble NSF attachment protein OS=Mus musculus OX=10090 GN=Napa PE=1 SV=1
ABI gene family member 3 OS=Mus musculus OX=10090 GN=Abi3 PE=1 SV=3
Histone-binding protein RBBP7 OS=Mus musculus OX=10090 GN=Rbbp7 PE=1 SV=1
SNF-related serine/threonine-protein kinase OS=Mus musculus OX=10090 GN=Snrk PE=1 SV=1
WD repeat-containing protein 91 OS=Mus musculus OX=10090 GN=Wdr91 PE=1 SV=1
2'-5'-oligoadenylate synthase 3 OS=Mus musculus OX=10090 GN=Oas3 PE=1 SV=1
60S ribosomal protein L32 OS=Mus musculus OX=10090 GN=Rpl32 PE=1 SV=2
Putative helicase MOV-10 OS=Mus musculus OX=10090 GN=Mov10 PE=1 SV=2
60S ribosomal protein L15 OS=Mus musculus OX=10090 GN=Rpl15 PE=1 SV=4
Zinc finger protein 513 OS=Mus musculus OX=10090 GN=Znf513 PE=1 SV=2
PH-interacting protein OS=Mus musculus OX=10090 GN=Phip PE=1 SV=2
DENN domain-containing protein 5B OS=Mus musculus OX=10090 GN=Dennd5b PE=1 SV=2
MAGUK p55 subfamily member 2 OS=Mus musculus OX=10090 GN=Mpp2 PE=1 SV=1
Wings apart-like protein homolog OS=Mus musculus OX=10090 GN=Wapl PE=1 SV=2
Multifunctional methyltransferase subunit TRM112-like protein OS=Mus musculus OX=10090 GN=Trmt112 PE=1
Nuclear cap-binding protein subunit 2 OS=Mus musculus OX=10090 GN=Ncbp2 PE=1 SV=1
DnaJ homolog subfamily B member 11 OS=Mus musculus OX=10090 GN=Dnajb11 PE=1 SV=1
Oxidoreductase HTATIP2 OS=Mus musculus OX=10090 GN=Htatip2 PE=1 SV=3
Polyhomeotic-like protein 3 OS=Mus musculus OX=10090 GN=Phc3 PE=1 SV=2
Ribonucleoprotein PTB-binding 1 OS=Mus musculus OX=10090 GN=Raver1 PE=1 SV=2
5-oxoprolinase OS=Mus musculus OX=10090 GN=Oplah PE=1 SV=1
Filamin-A OS=Mus musculus OX=10090 GN=Flna PE=1 SV=5
Zfp71-rs1 protein OS=Mus musculus OX=10090 GN=Zfp65 PE=2 SV=1
Nap1l4 protein OS=Mus musculus OX=10090 GN=Nap1l4 PE=1 SV=1
Histone H2A type 3 OS=Mus musculus OX=10090 GN=H2aw PE=1 SV=3
Golgi apparatus protein 1 (Fragment) OS=Mus musculus OX=10090 GN=Glg1 PE=1 SV=1
Methyl-CpG-binding protein 2 OS=Mus musculus OX=10090 GN=Mecp2 PE=1 SV=1
Pyruvate kinase PKM OS=Mus musculus OX=10090 GN=Pkm PE=1 SV=4
Integrin alpha-8 (Fragment) OS=Mus musculus OX=10090 GN=Itga8 PE=1 SV=1
ETS translocation variant 3 OS=Mus musculus OX=10090 GN=Etv3 PE=1 SV=1
52 kDa repressor of the inhibitor of the protein kinase OS=Mus musculus OX=10090 GN=Thap12 PE=2 SV=2

Rho GTPase-activating protein 7 OS=Mus musculus OX=10090 GN=Dlc1 PE=1 SV=1
Protein transport protein Sec31A OS=Mus musculus OX=10090 GN=Sec31a PE=1 SV=2
Ashwin OS=Mus musculus OX=10090 PE=1 SV=1
Cyclin-dependent kinase 4 inhibitor C OS=Mus musculus OX=10090 GN=Cdkn2c PE=1 SV=1
Zinc finger protein 791 OS=Mus musculus OX=10090 GN=Zfp791 PE=2 SV=1
Zinc finger protein 87 OS=Mus musculus OX=10090 GN=Zfp87 PE=2 SV=1
Centrosomal protein of 97 kDa OS=Mus musculus OX=10090 GN=Cep97 PE=1 SV=1
AP-1 complex subunit mu-1 OS=Mus musculus OX=10090 GN=Ap1m1 PE=1 SV=3
Myosin light chain kinase, smooth muscle OS=Mus musculus OX=10090 GN=Mylk PE=1 SV=3
Procollagen C-endopeptidase enhancer 2 OS=Mus musculus OX=10090 GN=Pcolce2 PE=2 SV=2
Transcription and mRNA export factor ENY2 OS=Mus musculus OX=10090 GN=Eny2 PE=1 SV=1
Antigen peptide transporter 2 OS=Mus musculus OX=10090 GN=Tap2 PE=1 SV=1
Mitofusin-2 OS=Mus musculus OX=10090 GN=Mfn2 PE=1 SV=3
KN motif and ankyrin repeat domain-containing protein 3 OS=Mus musculus OX=10090 GN=Kank3 PE=1 SV=1
Dehydrogenase/reductase SDR family member 13 OS=Mus musculus OX=10090 GN=Dhrs13 PE=1 SV=1
REST corepressor 3 OS=Mus musculus OX=10090 GN=Rcor3 PE=1 SV=1
Protein LYRIC OS=Mus musculus OX=10090 GN=Mtdh PE=1 SV=1
Baculoviral IAP repeat-containing protein 6 OS=Mus musculus OX=10090 GN=Birc6 PE=1 SV=2
Ras-related protein Rab-43 OS=Mus musculus OX=10090 GN=Rab43 PE=1 SV=1
Nucleolin OS=Mus musculus OX=10090 GN=Ncl PE=1 SV=2
N-alpha-acetyltransferase 16, NatA auxiliary subunit OS=Mus musculus OX=10090 GN=Naa16 PE=2 SV=1
tRNA (guanine(10)-N2)-methyltransferase homolog OS=Mus musculus OX=10090 GN=Trmt11 PE=1 SV=1
Testis-specific Y-encoded-like protein 1 OS=Mus musculus OX=10090 GN=Tspyl1 PE=2 SV=1
Carboxy-terminal domain RNA polymerase II polypeptide A small phosphatase 1 OS=Mus musculus OX=10090 GI
Dynein light chain 2, cytoplasmic OS=Mus musculus OX=10090 GN=Dynll2 PE=1 SV=1
Transcription initiation factor TFIID subunit 2 OS=Mus musculus OX=10090 GN=Taf2 PE=2 SV=2
Actin filament-associated protein 1-like 2 OS=Mus musculus OX=10090 GN=Afap1l2 PE=1 SV=1
GATOR complex protein MIOS OS=Mus musculus OX=10090 GN=Mios PE=1 SV=2
E3 ubiquitin-protein ligase Jade-2 OS=Mus musculus OX=10090 GN=Jade2 PE=1 SV=1
Voltage-dependent anion-selective channel protein 3 OS=Mus musculus OX=10090 GN=Vdac3 PE=1 SV=1
Protein fantom OS=Mus musculus OX=10090 GN=Rpgrip1l PE=1 SV=2
Protein phosphatase 1M OS=Mus musculus OX=10090 GN=Ppm1m PE=1 SV=1
39S ribosomal protein L50, mitochondrial OS=Mus musculus OX=10090 GN=Mrpl50 PE=1 SV=2
Thymocyte nuclear protein 1 OS=Mus musculus OX=10090 GN=Thyn1 PE=1 SV=1
Src substrate cortactin OS=Mus musculus OX=10090 GN=Cttn PE=1 SV=2
Neurabin-2 OS=Mus musculus OX=10090 GN=Ppp1r9b PE=1 SV=1
Zinc finger protein 467 OS=Mus musculus OX=10090 GN=Znf467 PE=1 SV=1
Cytochrome b-245 heavy chain OS=Mus musculus OX=10090 GN=Cybb PE=1 SV=1
Rab-like protein 6 OS=Mus musculus OX=10090 GN=Rabl6 PE=1 SV=2
Heat shock 70 kDa protein 1A OS=Mus musculus OX=10090 GN=Hspa1a PE=1 SV=2
Nurim OS=Mus musculus OX=10090 GN=Nrm PE=1 SV=1
Vacuolar protein sorting-associated protein 16 homolog OS=Mus musculus OX=10090 GN=Vps16 PE=1 SV=1
Guanine nucleotide-binding protein G(i) subunit alpha-2 OS=Mus musculus OX=10090 GN=Gnai2 PE=1 SV=5
Mediator of RNA polymerase II transcription subunit 1 OS=Mus musculus OX=10090 GN=Med1 PE=1 SV=1
Chromobox protein homolog 8 OS=Mus musculus OX=10090 GN=Cbx8 PE=1 SV=1

Zinc finger protein 524 OS=Mus musculus OX=10090 GN=Znf524 PE=2 SV=1
Transgelin OS=Mus musculus OX=10090 GN=Tagln PE=1 SV=3
Alpha N-catenin OS=Mus musculus OX=10090 GN=Ctnna2 PE=1 SV=1
Ankyrin repeat domain-containing protein 63 OS=Mus musculus OX=10090 GN=Ankrd63 PE=1 SV=1
Stromal interaction molecule 2 OS=Mus musculus OX=10090 GN=Stim2 PE=1 SV=2
Multiple myeloma tumor-associated protein 2 homolog OS=Mus musculus OX=10090 GN=Mmtag2 PE=2 SV=1
Nuclear pore complex protein Nup88 OS=Mus musculus OX=10090 GN=Nup88 PE=1 SV=1
Phosphatidylinositol 4,5-bisphosphate 3-kinase catalytic subunit gamma isoform OS=Mus musculus OX=10090 G
26S proteasome regulatory subunit 4 OS=Mus musculus OX=10090 GN=Psmc1 PE=1 SV=1
KH domain-containing, RNA-binding, signal transduction-associated protein 3 OS=Mus musculus OX=10090 GN=I
Myocardial zonula adherens protein OS=Mus musculus OX=10090 GN=Myzap PE=1 SV=1
Olfactory receptor OS=Mus musculus OX=10090 GN=Olfr516 PE=3 SV=1
Cathepsin Z OS=Mus musculus OX=10090 GN=Ctsz PE=1 SV=1
Dystonin OS=Mus musculus OX=10090 GN=Dst PE=1 SV=1
Casein kinase I isoform epsilon OS=Mus musculus OX=10090 GN=Csnk1e PE=1 SV=2
Purine nucleoside phosphorylase OS=Mus musculus OX=10090 GN=Pnp PE=1 SV=1
Synaptic functional regulator FMR1 OS=Mus musculus OX=10090 GN=Fmr1 PE=1 SV=1
RUN and FYVE domain-containing protein 4 OS=Mus musculus OX=10090 GN=Rufy4 PE=2 SV=3
116 kDa U5 small nuclear ribonucleoprotein component OS=Mus musculus OX=10090 GN=Eftud2 PE=1 SV=1
Eukaryotic translation elongation factor 1 beta 2 OS=Mus musculus OX=10090 GN=Eef1b2 PE=1 SV=1
Protein CASC3 OS=Mus musculus OX=10090 GN=Casc3 PE=1 SV=3
Elongin-C OS=Mus musculus OX=10090 GN=Eloc PE=1 SV=1
MORF4 family-associated protein 1 OS=Mus musculus OX=10090 GN=Mrfap1 PE=1 SV=1
60S ribosomal protein L26 OS=Mus musculus OX=10090 GN=Rpl26 PE=1 SV=1
Zinc finger protein 825 OS=Mus musculus OX=10090 GN=Zfp825 PE=1 SV=1
Four and a half LIM domains protein 3 OS=Mus musculus OX=10090 GN=Fhl3 PE=1 SV=1
Protein angel homolog 2 OS=Mus musculus OX=10090 GN=Angel2 PE=1 SV=1
Dedicator of cytokinesis protein 8 OS=Mus musculus OX=10090 GN=Dock8 PE=1 SV=4
Ubiquitin conjugation factor E4 A OS=Mus musculus OX=10090 GN=Ube4a PE=1 SV=1
SWI/SNF-related matrix-associated actin-dependent regulator of chromatin subfamily A-like protein 1 OS=Mus r
M-phase phosphoprotein 8 OS=Mus musculus OX=10090 GN=Mphosph8 PE=1 SV=1
Neurobeachin-like 1 OS=Mus musculus OX=10090 GN=Nbeal1 PE=1 SV=1
Vasodilator-stimulated phosphoprotein OS=Mus musculus OX=10090 GN=Vasp PE=1 SV=4
Predicted gene 14124 OS=Mus musculus OX=10090 GN=Gm14124 PE=4 SV=1
1-acyl-sn-glycerol-3-phosphate acyltransferase epsilon OS=Mus musculus OX=10090 GN=Agpat5 PE=1 SV=1
cAMP-dependent protein kinase catalytic subunit beta OS=Mus musculus OX=10090 GN=Prkacb PE=1 SV=2
Guanine nucleotide-binding protein subunit beta-5 OS=Mus musculus OX=10090 GN=Gnb5 PE=1 SV=1
Zinc finger and BTB domain-containing protein 11 OS=Mus musculus OX=10090 GN=Zbtb11 PE=2 SV=1
Band 3 anion transport protein OS=Mus musculus OX=10090 GN=Slc4a1 PE=1 SV=1
Cytochrome b-c1 complex subunit Rieske, mitochondrial OS=Mus musculus OX=10090 GN=Uqcrfs1 PE=1 SV=1
C-C motif chemokine 6 OS=Mus musculus OX=10090 GN=Ccl6 PE=1 SV=1
Guanine nucleotide-binding protein G(q) subunit alpha OS=Mus musculus OX=10090 GN=Gnaq PE=1 SV=4
Krueppel-like factor 3 OS=Mus musculus OX=10090 GN=Klf3 PE=1 SV=1
Charged multivesicular body protein 2b OS=Mus musculus OX=10090 GN=Chmp2b PE=1 SV=1
Aldo-keto reductase family 1, member B3 (aldose reductase) (Fragment) OS=Mus musculus OX=10090 GN=Akr1

Sequestosome-1 OS=Mus musculus OX=10090 GN=Sqstm1 PE=1 SV=1
DNA helicase OS=Mus musculus OX=10090 GN=Chd4 PE=1 SV=1
55 kDa erythrocyte membrane protein OS=Mus musculus OX=10090 GN=Mpp1 PE=1 SV=1
Y-box-binding protein 2 OS=Mus musculus OX=10090 GN=Ybx2 PE=1 SV=1
60S ribosomal protein L27 OS=Mus musculus OX=10090 GN=Rpl27 PE=1 SV=2
Torsin-1A-interacting protein 1 OS=Mus musculus OX=10090 GN=Tor1aip1 PE=1 SV=3
Nexilin OS=Mus musculus OX=10090 GN=Nexn PE=1 SV=1
Protein mono-ADP-ribosyltransferase PARP9 OS=Mus musculus OX=10090 GN=Parp9 PE=1 SV=2
Structural maintenance of chromosomes protein 3 OS=Mus musculus OX=10090 GN=Smc3 PE=1 SV=2
Cyclin-T2 OS=Mus musculus OX=10090 GN=Ccnt2 PE=1 SV=1
RNA helicase OS=Mus musculus OX=10090 GN=Ddx24 PE=1 SV=1
Zinc finger protein 787 OS=Mus musculus OX=10090 GN=Znf787 PE=2 SV=3
Ppfia1 protein OS=Mus musculus OX=10090 GN=Ppfia1 PE=1 SV=1
Gem-associated protein 2 OS=Mus musculus OX=10090 GN=Gemin2 PE=2 SV=1
Protein inturned OS=Mus musculus OX=10090 GN=Intu PE=1 SV=1
18S rRNA aminocarboxypropyltransferase OS=Mus musculus OX=10090 GN=Tsr3 PE=1 SV=1
Kinesin-like protein KIF3B OS=Mus musculus OX=10090 GN=Kif3b PE=1 SV=1
Probable ATP-dependent RNA helicase DDX49 OS=Mus musculus OX=10090 GN=Ddx49 PE=2 SV=1
Zinc finger CCHC-type and RNA-binding motif-containing protein 1 OS=Mus musculus OX=10090 GN=Zcrb1 PE=1
Misshapen-like kinase 1 OS=Mus musculus OX=10090 GN=Mink1 PE=1 SV=1
NF-kappa-B inhibitor-interacting Ras-like protein 2 OS=Mus musculus OX=10090 GN=Nkiras2 PE=1 SV=1
Nuclear factor 1 X-type OS=Mus musculus OX=10090 GN=Nfix PE=1 SV=2
Complement C1q and tumor necrosis factor-related protein 9 OS=Mus musculus OX=10090 GN=C1qtnf9 PE=1 SV
Predicted gene 14326 OS=Mus musculus OX=10090 GN=Gm14326 PE=4 SV=1
NADH dehydrogenase [ubiquinone] 1 alpha subcomplex subunit 9, mitochondrial OS=Mus musculus OX=10090 G
Phosphoribosylformylglycinamidine synthase OS=Mus musculus OX=10090 GN=Pfas PE=1 SV=1
DnaJ heat shock protein family (Hsp40) member C13 OS=Mus musculus OX=10090 GN=Dnajc13 PE=1 SV=1
40S ribosomal protein S3 OS=Mus musculus OX=10090 GN=Rps3 PE=1 SV=1
UHRF1-binding protein 1-like OS=Mus musculus OX=10090 GN=Uhrf1bp1l PE=1 SV=2
Plexin domain-containing protein 2 OS=Mus musculus OX=10090 GN=Plxdc2 PE=1 SV=1
Splicing factor 3A subunit 1 OS=Mus musculus OX=10090 GN=Sf3a1 PE=1 SV=1
Vacuolar protein sorting-associated protein 33A (Fragment) OS=Mus musculus OX=10090 GN=Vps33a PE=1 SV=
Granzyme B(G,H) OS=Mus musculus OX=10090 GN=Gzmb PE=1 SV=1
Partner of Y14 and mago OS=Mus musculus OX=10090 GN=Pym1 PE=1 SV=2
Aly/REF export factor 2 OS=Mus musculus OX=10090 GN=Alyref2 PE=1 SV=1
DNA topoisomerase 3-beta-1 OS=Mus musculus OX=10090 GN=Top3b PE=1 SV=1
Epiplakin OS=Mus musculus OX=10090 GN=Eppk1 PE=1 SV=2
Apoptosis-inducing factor 1, mitochondrial OS=Mus musculus OX=10090 GN=Aifm1 PE=1 SV=1
Phosphatidylinositol 4-kinase beta OS=Mus musculus OX=10090 GN=Pi4kb PE=1 SV=1
TBC1 domain family member 10A OS=Mus musculus OX=10090 GN=Tbc1d10a PE=1 SV=1
Apoptotic chromatin condensation inducer in the nucleus OS=Mus musculus OX=10090 GN=Acin1 PE=1 SV=3
Cyclic nucleotide ras GEF OS=Mus musculus OX=10090 GN=Rapgef2 PE=1 SV=1
1-phosphatidylinositol 4,5-bisphosphate phosphodiesterase gamma-1 OS=Mus musculus OX=10090 GN=Plcg1 PE
39S ribosomal protein L28, mitochondrial OS=Mus musculus OX=10090 GN=Mrpl28 PE=1 SV=3
Growth factor receptor-bound protein 2 OS=Mus musculus OX=10090 GN=Grb2 PE=1 SV=1

Vascular endothelial growth factor receptor 2 OS=Mus musculus OX=10090 GN=Kdr PE=1 SV=2
AP-2 complex subunit mu OS=Mus musculus OX=10090 GN=Ap2m1 PE=1 SV=1
Stomatin OS=Mus musculus OX=10090 GN=Stom PE=1 SV=3
Malectin OS=Mus musculus OX=10090 GN=Mlec PE=1 SV=2
Armadillo repeat-containing protein 10 OS=Mus musculus OX=10090 GN=Armc10 PE=1 SV=1
GA-binding protein alpha chain OS=Mus musculus OX=10090 GN=Gabpa PE=1 SV=2
Zinc finger CCCH domain-containing protein 3 OS=Mus musculus OX=10090 GN=Zc3h3 PE=1 SV=1
Trafficking protein particle complex subunit 1 OS=Mus musculus OX=10090 GN=Trappc1 PE=1 SV=1
Four and a half LIM domains protein 1 OS=Mus musculus OX=10090 GN=Fhl1 PE=1 SV=1
Filamin-C OS=Mus musculus OX=10090 GN=Flnc PE=1 SV=3
Microtubule-associated protein 4 OS=Mus musculus OX=10090 GN=Map4 PE=1 SV=3
tRNA pseudouridine synthase A OS=Mus musculus OX=10090 GN=Pus1 PE=1 SV=1
Cysteine and glycine-rich protein 2 OS=Mus musculus OX=10090 GN=Csrp2 PE=1 SV=1
Protein phosphatase 1 regulatory subunit 12B OS=Mus musculus OX=10090 GN=Ppp1r12b PE=1 SV=2
Phenylalanine--tRNA ligase alpha subunit OS=Mus musculus OX=10090 GN=Farsa PE=1 SV=1
Tubulin beta-2A chain OS=Mus musculus OX=10090 GN=Tubb2a PE=1 SV=1
39S ribosomal protein L4, mitochondrial OS=Mus musculus OX=10090 GN=Mrpl4 PE=1 SV=1
Integrin alpha-X OS=Mus musculus OX=10090 GN=Itgax PE=1 SV=1
Protein POF1B OS=Mus musculus OX=10090 GN=Pof1b PE=2 SV=3
Rab3 GTPase-activating protein non-catalytic subunit OS=Mus musculus OX=10090 GN=Rab3gap2 PE=1 SV=1
Exportin-5 OS=Mus musculus OX=10090 GN=Xpo5 PE=1 SV=1
GTPase IMAP family member 4 OS=Mus musculus OX=10090 GN=Gimap4 PE=1 SV=2
DNA methyltransferase 1-associated protein 1 OS=Mus musculus OX=10090 GN=Dmap1 PE=1 SV=1
C-X-C motif chemokine 14 OS=Mus musculus OX=10090 GN=Cxcl14 PE=2 SV=2
PRKC apoptosis WT1 regulator protein OS=Mus musculus OX=10090 GN=Pawr PE=1 SV=2
C-terminal-binding protein 2 OS=Mus musculus OX=10090 GN=Ctbp2 PE=1 SV=2
Cilia- and flagella-associated protein 20 OS=Mus musculus OX=10090 GN=Cfap20 PE=1 SV=1
Protein MAL2 OS=Mus musculus OX=10090 GN=Mal2 PE=1 SV=1
E3 ubiquitin-protein ligase DTX3L OS=Mus musculus OX=10090 GN=Dtx3l PE=1 SV=1
Chloride intracellular channel protein 1 OS=Mus musculus OX=10090 GN=Clic1 PE=1 SV=3
Cyclin-dependent kinase 2-associated protein 1 OS=Mus musculus OX=10090 GN=Cdk2ap1 PE=1 SV=1
Myosin-6 OS=Mus musculus OX=10090 GN=Myh6 PE=1 SV=2
Trafficking protein particle complex subunit 5 OS=Mus musculus OX=10090 GN=Trappc5 PE=1 SV=1
28S ribosomal protein S31, mitochondrial OS=Mus musculus OX=10090 GN=Mrps31 PE=1 SV=1
Glyoxalase domain-containing protein 4 OS=Mus musculus OX=10090 GN=Glod4 PE=1 SV=1
General transcription factor IIH subunit 4 OS=Mus musculus OX=10090 GN=Gtf2h4 PE=1 SV=1
AFG3-like protein 1 OS=Mus musculus OX=10090 GN=Afg3l1 PE=1 SV=2
H-2 class I histocompatibility antigen, D-B alpha chain OS=Mus musculus OX=10090 GN=H2-D1 PE=1 SV=2
Insulin-like growth factor 2 mRNA-binding protein 2 OS=Mus musculus OX=10090 GN=Igf2bp2 PE=1 SV=1
Alpha-taxilin OS=Mus musculus OX=10090 GN=Txlna PE=1 SV=1
Serum paraoxonase/lactonase 3 OS=Mus musculus OX=10090 GN=Pon3 PE=1 SV=2
Syntaxin-binding protein 2 OS=Mus musculus OX=10090 GN=Stxbp2 PE=1 SV=1
Usher syndrome type-1C protein-binding protein 1 OS=Mus musculus OX=10090 GN=Ushbp1 PE=1 SV=2
GTP-binding protein GEM OS=Mus musculus OX=10090 GN=Gem PE=1 SV=2
Constitutive coactivator of peroxisome proliferator-activated receptor gamma OS=Mus musculus OX=10090 GN=

Dystrobrevin OS=Mus musculus OX=10090 GN=Dtna PE=1 SV=1
Sodium/potassium-transporting ATPase subunit beta-3 OS=Mus musculus OX=10090 GN=Atp1b3 PE=1 SV=1
Forkhead box protein K2 OS=Mus musculus OX=10090 GN=Foxk2 PE=1 SV=3
Eukaryotic translation initiation factor 1A, X-chromosomal OS=Mus musculus OX=10090 GN=Eif1ax PE=2 SV=3
Small nuclear ribonucleoprotein-associated protein B OS=Mus musculus OX=10090 GN=Snrpb PE=1 SV=1
Ribonuclease P protein subunit p14 OS=Mus musculus OX=10090 GN=Rpp14 PE=1 SV=1
Protein spinster homolog 1 OS=Mus musculus OX=10090 GN=Spns1 PE=1 SV=1
60S ribosomal protein L7a OS=Mus musculus OX=10090 GN=Rpl7a PE=1 SV=2
F-box only protein 10 OS=Mus musculus OX=10090 GN=Fbxo10 PE=1 SV=2
SH3 and multiple ankyrin repeat domains protein 3 OS=Mus musculus OX=10090 GN=Shank3 PE=1 SV=3
Aminoacyl tRNA synthase complex-interacting multifunctional protein 1 OS=Mus musculus OX=10090 GN=Aimp[illegible]
Arginine--tRNA ligase, cytoplasmic OS=Mus musculus OX=10090 GN=Rars1 PE=1 SV=2
Protein S100-A13 OS=Mus musculus OX=10090 GN=S100a13 PE=1 SV=1
THO complex subunit 3 OS=Mus musculus OX=10090 GN=Thoc3 PE=2 SV=1
Proline-serine-threonine phosphatase-interacting protein 2 OS=Mus musculus OX=10090 GN=Pstpip2 PE=1 SV=4
Serine/threonine-protein kinase STK11 OS=Mus musculus OX=10090 GN=Stk11 PE=1 SV=1
Embigin OS=Mus musculus OX=10090 GN=Emb PE=1 SV=2
SRSF protein kinase 2 OS=Mus musculus OX=10090 GN=Srpk2 PE=1 SV=1
NF-kappa-B essential modulator (Fragment) OS=Mus musculus OX=10090 GN=Ikbkg PE=1 SV=1
Tubulin beta-1 chain OS=Mus musculus OX=10090 GN=Tubb1 PE=1 SV=1
High affinity immunoglobulin epsilon receptor subunit gamma OS=Mus musculus OX=10090 GN=Fcer1g PE=1 SV
Solute carrier family 12 member 2 OS=Mus musculus OX=10090 GN=Slc12a2 PE=1 SV=1
Anthrax toxin receptor 1 OS=Mus musculus OX=10090 GN=Antxr1 PE=1 SV=1
Galactocerebrosidase OS=Mus musculus OX=10090 GN=Galc PE=1 SV=2
RalA-binding protein 1 OS=Mus musculus OX=10090 GN=Ralbp1 PE=1 SV=4
Coiled-coil domain-containing protein 102A OS=Mus musculus OX=10090 GN=Ccdc102a PE=1 SV=2
Kinesin-like protein KIF1C OS=Mus musculus OX=10090 GN=Kif1c PE=1 SV=2
E3 SUMO-protein ligase NSE2 OS=Mus musculus OX=10090 GN=Nsmce2 PE=2 SV=1
ATP-sensitive inward rectifier potassium channel 8 OS=Mus musculus OX=10090 GN=Kcnj8 PE=1 SV=1
Probable ATP-dependent RNA helicase DDX10 OS=Mus musculus OX=10090 GN=Ddx10 PE=1 SV=2
Dedicator of cytokinesis protein 4 OS=Mus musculus OX=10090 GN=Dock4 PE=1 SV=1
RNA-binding protein 14 OS=Mus musculus OX=10090 GN=Rbm14 PE=1 SV=1
Calcium/calmodulin-dependent protein kinase type II subunit gamma OS=Mus musculus OX=10090 GN=Camk2g
Nucleolus and neural progenitor protein OS=Mus musculus OX=10090 GN=Nepro PE=2 SV=1
Cytohesin-3 OS=Mus musculus OX=10090 GN=Cyth3 PE=1 SV=1
Arf-GAP with GTPase, ANK repeat and PH domain-containing protein 1 OS=Mus musculus OX=10090 GN=Agap1
Rho guanine nucleotide exchange factor 17 OS=Mus musculus OX=10090 GN=Arhgef17 PE=1 SV=2
Cytoplasmic polyadenylation element-binding protein 4 OS=Mus musculus OX=10090 GN=Cpeb4 PE=1 SV=1
Probable RNA-binding protein 19 OS=Mus musculus OX=10090 GN=Rbm19 PE=1 SV=1
60S ribosomal protein L19 OS=Mus musculus OX=10090 GN=Rpl19 PE=1 SV=1
Zinc finger with KRAB and SCAN domains 2 OS=Mus musculus OX=10090 GN=Zkscan2 PE=1 SV=1
Friend leukemia integration 1 transcription factor OS=Mus musculus OX=10090 GN=Fli1 PE=1 SV=1
Protein spire homolog 1 (Fragment) OS=Mus musculus OX=10090 GN=Spire1 PE=1 SV=1
Exosome complex exonuclease RRP44 OS=Mus musculus OX=10090 GN=Dis3 PE=1 SV=4
Heterogeneous nuclear ribonucleoprotein R OS=Mus musculus OX=10090 GN=Hnrnpr PE=1 SV=1

Numb-like protein OS=Mus musculus OX=10090 GN=Numbl PE=1 SV=3
NADH-ubiquinone oxidoreductase 75 kDa subunit, mitochondrial OS=Mus musculus OX=10090 GN=Ndufs1 PE=1 [illegible]
E3 ubiquitin-protein ligase ZNF598 OS=Mus musculus OX=10090 GN=Znf598 PE=1 SV=1
G protein-coupled receptor kinase 5 OS=Mus musculus OX=10090 GN=Grk5 PE=1 SV=2
Zinc finger and BTB domain-containing 34 OS=Mus musculus OX=10090 GN=Zbtb34 PE=1 SV=1
LIM and senescent cell antigen-like-containing domain protein 2 OS=Mus musculus OX=10090 GN=Lims2 PE=1 S[illegible]
60S ribosomal protein L35a OS=Mus musculus OX=10090 GN=Rpl35a PE=1 SV=2
Dedicator of cytokinesis protein 1 OS=Mus musculus OX=10090 GN=Dock1 PE=1 SV=3
E3 ubiquitin-protein ligase Rnf220 OS=Mus musculus OX=10090 GN=Rnf220 PE=1 SV=2
Probable JmjC domain-containing histone demethylation protein 2C OS=Mus musculus OX=10090 GN=Jmjd1c PE[illegible]
Mitochondrial peptide methionine sulfoxide reductase OS=Mus musculus OX=10090 GN=Msra PE=1 SV=1
Mefv protein OS=Mus musculus OX=10090 GN=Mefv PE=1 SV=1
Heterogeneous nuclear ribonucleoprotein K OS=Mus musculus OX=10090 GN=Hnrnpk PE=1 SV=1
Interferon-induced helicase C domain-containing protein 1 OS=Mus musculus OX=10090 GN=Ifih1 PE=1 SV=1
Arsenite methyltransferase OS=Mus musculus OX=10090 GN=As3mt PE=1 SV=2
Glutathione S-transferase P 2 OS=Mus musculus OX=10090 GN=Gstp2 PE=1 SV=2
WD repeat-containing protein 81 (Fragment) OS=Mus musculus OX=10090 GN=Wdr81 PE=1 SV=1
POC1 centriolar protein homolog B OS=Mus musculus OX=10090 GN=Poc1b PE=1 SV=1
Protein phosphatase 1 regulatory subunit 7 OS=Mus musculus OX=10090 GN=Ppp1r7 PE=1 SV=2
Oxysterol-binding protein OS=Mus musculus OX=10090 GN=Osbpl3 PE=1 SV=1
U3 small nucleolar RNA-associated protein 4 homolog OS=Mus musculus OX=10090 GN=Utp4 PE=2 SV=3
Menin OS=Mus musculus OX=10090 GN=Men1 PE=1 SV=1
Endoplasmic reticulum-Golgi intermediate compartment protein 1 OS=Mus musculus OX=10090 GN=Ergic1 PE=[illegible]
Myotubularin-related protein 10 OS=Mus musculus OX=10090 GN=Mtmr10 PE=1 SV=2
Tubulin beta-4A chain OS=Mus musculus OX=10090 GN=Tubb4a PE=1 SV=3
Protein-serine/threonine phosphatase OS=Mus musculus OX=10090 GN=Ppm1a PE=1 SV=1
Dedicator of cytokinesis protein 5 OS=Mus musculus OX=10090 GN=Dock5 PE=1 SV=2
Plasminogen OS=Mus musculus OX=10090 GN=Plg PE=1 SV=3
Carnitine O-palmitoyltransferase 1, muscle isoform OS=Mus musculus OX=10090 GN=Cpt1b PE=1 SV=1
Anaphase-promoting complex subunit 1 OS=Mus musculus OX=10090 GN=Anapc1 PE=1 SV=2
Elongator complex protein 4 OS=Mus musculus OX=10090 GN=Elp4 PE=1 SV=2
Zinc finger protein 236 OS=Mus musculus OX=10090 GN=Zfp236 PE=1 SV=1
A-kinase anchor protein 8-like OS=Mus musculus OX=10090 GN=Akap8l PE=1 SV=1
Eukaryotic translation initiation factor 4H OS=Mus musculus OX=10090 GN=Eif4h PE=1 SV=3
Mitochondrial RNA pseudouridine synthase Rpusd4 OS=Mus musculus OX=10090 GN=Rpusd4 PE=2 SV=1
Probable rRNA-processing protein EBP2 OS=Mus musculus OX=10090 GN=Ebna1bp2 PE=2 SV=1
Serine beta-lactamase-like protein LACTB, mitochondrial OS=Mus musculus OX=10090 GN=Lactb PE=1 SV=1
Glycylpeptide N-tetradecanoyltransferase 2 OS=Mus musculus OX=10090 GN=Nmt2 PE=1 SV=1
GDNF-inducible zinc finger protein 1 OS=Mus musculus OX=10090 GN=Gzf1 PE=1 SV=2
Threonine aspartase 1 OS=Mus musculus OX=10090 GN=Tasp1 PE=2 SV=1
Protein FRA10AC1 homolog OS=Mus musculus OX=10090 GN=Fra10ac1 PE=1 SV=3
FYVE, RhoGEF and PH domain-containing protein 5 OS=Mus musculus OX=10090 GN=Fgd5 PE=1 SV=1
Aurora kinase A-interacting protein OS=Mus musculus OX=10090 GN=Aurkaip1 PE=2 SV=2
ADP-ribosylation factor 3 OS=Mus musculus OX=10090 GN=Arf3 PE=2 SV=2
ADP-ribosylation factor-like protein 11 OS=Mus musculus OX=10090 GN=Arl11 PE=2 SV=1

CCR4-NOT transcription complex subunit 3 OS=Mus musculus OX=10090 GN=Cnot3 PE=1 SV=1
Myeloid cell nuclear differentiation antigen-like protein OS=Mus musculus OX=10090 GN=Mndal PE=1 SV=1
Microtubule-actin cross-linking factor 1 (Fragment) OS=Mus musculus OX=10090 GN=Macf1 PE=1 SV=1
Chromodomain-helicase-DNA-binding protein 9 OS=Mus musculus OX=10090 GN=Chd9 PE=1 SV=2
FERM, ARHGEF and pleckstrin domain-containing protein 2 OS=Mus musculus OX=10090 GN=Farp2 PE=1 SV=2
Partitioning defective 6 homolog gamma OS=Mus musculus OX=10090 GN=Pard6g PE=2 SV=1
Pericentrin OS=Mus musculus OX=10090 GN=Pcnt PE=1 SV=1
Polyunsaturated fatty acid 5-lipoxygenase OS=Mus musculus OX=10090 GN=Alox5 PE=1 SV=3
S phase cyclin A-associated protein in the ER OS=Mus musculus OX=10090 GN=Scaper PE=1 SV=1
Protein phosphatase Slingshot homolog 2 OS=Mus musculus OX=10090 GN=Ssh2 PE=1 SV=2
Gamma-taxilin OS=Mus musculus OX=10090 GN=Txlng PE=1 SV=1
Centromere/kinetochore protein zw10 homolog OS=Mus musculus OX=10090 GN=Zw10 PE=1 SV=3
Biglycan OS=Mus musculus OX=10090 GN=Bgn PE=1 SV=1
Cullin-1 OS=Mus musculus OX=10090 GN=Cul1 PE=1 SV=1
RIKEN cDNA 0610010K14 gene OS=Mus musculus OX=10090 GN=0610010K14Rik PE=1 SV=2
Serine/threonine-protein kinase Nek7 OS=Mus musculus OX=10090 GN=Nek7 PE=1 SV=1
F-box/WD repeat-containing protein 4 OS=Mus musculus OX=10090 GN=Fbxw4 PE=1 SV=1
Band 4.1-like protein 3 OS=Mus musculus OX=10090 GN=Epb41l3 PE=1 SV=1
Katanin p80 WD40 repeat-containing subunit B1 OS=Mus musculus OX=10090 GN=Katnb1 PE=1 SV=1
40S ribosomal protein S21 OS=Mus musculus OX=10090 GN=Rps21 PE=1 SV=1
Slit homolog 3 protein OS=Mus musculus OX=10090 GN=Slit3 PE=2 SV=2
Exosome complex component RRP40 OS=Mus musculus OX=10090 GN=Exosc3 PE=1 SV=3
Large subunit GTPase 1 homolog OS=Mus musculus OX=10090 GN=Lsg1 PE=1 SV=2
Succinate dehydrogenase assembly factor 1, mitochondrial OS=Mus musculus OX=10090 GN=Sdhaf1 PE=1 SV=1
Syntaxin-8 OS=Mus musculus OX=10090 GN=Stx8 PE=1 SV=1
Exopolyphosphatase PRUNE1 OS=Mus musculus OX=10090 GN=Prune1 PE=1 SV=1
Platelet-activating factor acetylhydrolase IB subunit alpha2 (Fragment) OS=Mus musculus OX=10090 GN=Pafah1
ARF GTPase-activating protein GIT1 OS=Mus musculus OX=10090 GN=Git1 PE=1 SV=1
DIP2 disco-interacting protein 2 homolog B (Drosophila) OS=Mus musculus OX=10090 GN=Dip2b PE=1 SV=1
Cathelicidin antimicrobial peptide OS=Mus musculus OX=10090 GN=Camp PE=1 SV=2
Caspase recruitment domain-containing protein 10 OS=Mus musculus OX=10090 GN=Card10 PE=1 SV=1
TBC1 domain family member 23 OS=Mus musculus OX=10090 GN=Tbc1d23 PE=1 SV=1
Protein C3orf33 homolog OS=Mus musculus OX=10090 PE=2 SV=1
Rap guanine nucleotide exchange factor 3 OS=Mus musculus OX=10090 GN=Rapgef3 PE=1 SV=1
Ubiquitin carboxyl-terminal hydrolase 43 OS=Mus musculus OX=10090 GN=Usp43 PE=1 SV=1
E3 ubiquitin-protein ligase MIB1 OS=Mus musculus OX=10090 GN=Mib1 PE=1 SV=1
DNA polymerase epsilon catalytic subunit A OS=Mus musculus OX=10090 GN=Pole PE=1 SV=3
Flotillin-2 OS=Mus musculus OX=10090 GN=Flot2 PE=1 SV=2
ARF GTPase-activating protein GIT2 OS=Mus musculus OX=10090 GN=Git2 PE=1 SV=1
LIM and calponin homology domains-containing protein 1 OS=Mus musculus OX=10090 GN=Limch1 PE=1 SV=1
39S ribosomal protein L10, mitochondrial OS=Mus musculus OX=10090 GN=Mrpl10 PE=1 SV=1
Tropomyosin alpha-1 chain OS=Mus musculus OX=10090 GN=Tpm1 PE=1 SV=1
40S ribosomal protein S9 OS=Mus musculus OX=10090 GN=Rps9 PE=1 SV=3
Bromodomain-containing protein 7 OS=Mus musculus OX=10090 GN=Brd7 PE=1 SV=1
Zinc finger protein 960 OS=Mus musculus OX=10090 GN=Zfp960 PE=4 SV=1

AP-2 complex subunit alpha-2 OS=Mus musculus OX=10090 GN=Ap2a2 PE=1 SV=2
Graves disease carrier protein homolog (Fragment) OS=Mus musculus OX=10090 GN=Slc25a16 PE=1 SV=1
Nucleolar MIF4G domain-containing protein 1 OS=Mus musculus OX=10090 GN=Nom1 PE=1 SV=2
Sentrin-specific protease 3 OS=Mus musculus OX=10090 GN=Senp3 PE=1 SV=1
Ubiquitin-fold modifier 1 OS=Mus musculus OX=10090 GN=Ufm1 PE=1 SV=1
CGG triplet repeat-binding protein 1 OS=Mus musculus OX=10090 GN=Cggbp1 PE=1 SV=1
Transcriptional repressor NF-X1 OS=Mus musculus OX=10090 GN=Nfx1 PE=1 SV=1
Actin filament-associated protein 1-like 1 OS=Mus musculus OX=10090 GN=Afap1l1 PE=1 SV=1
Sorting nexin-9 OS=Mus musculus OX=10090 GN=Snx9 PE=1 SV=1
Ribosome biogenesis regulatory protein homolog OS=Mus musculus OX=10090 GN=Rrs1 PE=1 SV=1
Ras-related protein Rab-5B OS=Mus musculus OX=10090 GN=Rab5b PE=1 SV=1
Abl interactor 1 OS=Mus musculus OX=10090 GN=Abi1 PE=1 SV=3
Amine oxidase [flavin-containing] A OS=Mus musculus OX=10090 GN=Maoa PE=1 SV=3
Prosaposin OS=Mus musculus OX=10090 GN=Psap PE=1 SV=1
Microtubule-associated protein 1S OS=Mus musculus OX=10090 GN=Map1s PE=1 SV=2
Proteasome subunit alpha type-5 OS=Mus musculus OX=10090 GN=Psma5 PE=1 SV=1
Centrin-3 (Fragment) OS=Mus musculus OX=10090 GN=Cetn3 PE=1 SV=1
60S ribosomal protein L39 OS=Mus musculus OX=10090 GN=Rpl39 PE=1 SV=2
Mortality factor 4-like protein 2 OS=Mus musculus OX=10090 GN=Morf4l2 PE=1 SV=1
La-related protein 4 OS=Mus musculus OX=10090 GN=Larp4 PE=1 SV=1
Intercellular adhesion molecule 1 OS=Mus musculus OX=10090 GN=Icam1 PE=1 SV=1
Matrix metalloproteinase-9 OS=Mus musculus OX=10090 GN=Mmp9 PE=1 SV=2
Cystatin-B OS=Mus musculus OX=10090 GN=Cstb PE=1 SV=1
Histone H2A.V OS=Mus musculus OX=10090 GN=H2az2 PE=1 SV=3
Importin subunit alpha-4 OS=Mus musculus OX=10090 GN=Kpna3 PE=1 SV=1
60S ribosomal protein L27a OS=Mus musculus OX=10090 GN=Rpl27a PE=1 SV=5
Transcription factor E3 OS=Mus musculus OX=10090 GN=Tfe3 PE=1 SV=1
Ciliary rootlet coiled-coil protein 2 OS=Mus musculus OX=10090 GN=Crocc2 PE=3 SV=2
Hepatocyte growth factor receptor OS=Mus musculus OX=10090 GN=Met PE=1 SV=1
60S ribosomal protein L21 OS=Mus musculus OX=10090 GN=Rpl21 PE=1 SV=1
IST1 homolog OS=Mus musculus OX=10090 GN=Ist1 PE=1 SV=1
Ubiquitin-conjugating enzyme E2 L3 (Fragment) OS=Mus musculus OX=10090 GN=Ube2l3 PE=1 SV=1
Serine/threonine-protein kinase DCLK1 OS=Mus musculus OX=10090 GN=Dclk1 PE=1 SV=1
Rhotekin (Fragment) OS=Mus musculus OX=10090 GN=Rtkn PE=1 SV=1
Cell cycle checkpoint protein RAD17 OS=Mus musculus OX=10090 GN=Rad17 PE=1 SV=2
Perforin-1 OS=Mus musculus OX=10090 GN=Prf1 PE=1 SV=2
Procollagen C-endopeptidase enhancer 1 OS=Mus musculus OX=10090 GN=Pcolce PE=1 SV=2
Zinc finger CCHC domain-containing protein 10 OS=Mus musculus OX=10090 GN=Zcchc10 PE=2 SV=1
Ras-related protein Rab-5A OS=Mus musculus OX=10090 GN=Rab5a PE=1 SV=1
YLP motif-containing protein 1 (Fragment) OS=Mus musculus OX=10090 GN=Ylpm1 PE=1 SV=1
Lethal(3)malignant brain tumor-like protein 3 OS=Mus musculus OX=10090 GN=L3mbtl3 PE=1 SV=1
Tropomodulin-1 OS=Mus musculus OX=10090 GN=Tmod1 PE=1 SV=2
Scm-like with four MBT domains protein 1 OS=Mus musculus OX=10090 GN=Sfmbt1 PE=1 SV=1
Cyclin-dependent kinase inhibitor 1B OS=Mus musculus OX=10090 GN=Cdkn1b PE=1 SV=2
Beta-1-syntrophin OS=Mus musculus OX=10090 GN=Sntb1 PE=1 SV=4

Histone-lysine N-methyltransferase SETD2 OS=Mus musculus OX=10090 GN=Setd2 PE=1 SV=1
NK13 OS=Mus musculus OX=10090 GN=Serpinb6b PE=1 SV=2
Kinesin-like protein KIF7 OS=Mus musculus OX=10090 GN=Kif7 PE=1 SV=1
Protein SCAI OS=Mus musculus OX=10090 GN=Scai PE=1 SV=2
TATA box-binding protein-associated factor RNA polymerase I subunit B OS=Mus musculus OX=10090 GN=Taf1b
Protein 4.2 OS=Mus musculus OX=10090 GN=Epb42 PE=1 SV=3
60S ribosomal protein L14 OS=Mus musculus OX=10090 GN=Rpl14 PE=1 SV=3
DENN domain-containing protein 2B OS=Mus musculus OX=10090 GN=Dennd2b PE=1 SV=1
Transmembrane protein 160 OS=Mus musculus OX=10090 GN=Tmem160 PE=2 SV=1
mRNA-decapping enzyme 1A (Fragment) OS=Mus musculus OX=10090 GN=Dcp1a PE=1 SV=1
ARF GTPase-activating protein GIT2 OS=Mus musculus OX=10090 GN=Git2 PE=1 SV=2
Sushi-repeat-containing protein SRPX OS=Mus musculus OX=10090 GN=Srpx PE=2 SV=1
Serine/threonine-protein kinase 19 OS=Mus musculus OX=10090 GN=Stk19 PE=2 SV=2
DnaJ homolog subfamily B member 4 OS=Mus musculus OX=10090 GN=Dnajb4 PE=1 SV=1
Dynein light chain 1, cytoplasmic OS=Mus musculus OX=10090 GN=Dynll1 PE=1 SV=1
Tubulin beta-4B chain OS=Mus musculus OX=10090 GN=Tubb4b PE=1 SV=1
PDZ and LIM domain protein 1 OS=Mus musculus OX=10090 GN=Pdlim1 PE=1 SV=4
E3 ubiquitin/ISG15 ligase TRIM25 OS=Mus musculus OX=10090 GN=Trim25 PE=1 SV=1
Prothymosin alpha OS=Mus musculus OX=10090 GN=Ptma PE=1 SV=2
Protein mab-21-like 4 OS=Mus musculus OX=10090 GN=Mab21l4 PE=1 SV=2
Ubiquitin-like modifier-activating enzyme 5 OS=Mus musculus OX=10090 GN=Uba5 PE=1 SV=2
39S ribosomal protein L34, mitochondrial OS=Mus musculus OX=10090 GN=Mrpl34 PE=1 SV=1
Phosphatidylserine lipase ABHD16A OS=Mus musculus OX=10090 GN=Abhd16a PE=1 SV=3
Actin-related protein 2/3 complex subunit 3 OS=Mus musculus OX=10090 GN=Arpc3 PE=1 SV=3
Fibrillin-1 OS=Mus musculus OX=10090 GN=Fbn1 PE=1 SV=2
Cullin-4B OS=Mus musculus OX=10090 GN=Cul4b PE=1 SV=1
Septin-11 OS=Mus musculus OX=10090 GN=Septin11 PE=1 SV=4
T-complex protein 1 subunit eta OS=Mus musculus OX=10090 GN=Cct7 PE=1 SV=1
Lys-63-specific deubiquitinase BRCC36 OS=Mus musculus OX=10090 GN=Brcc3 PE=1 SV=1
Protein Red OS=Mus musculus OX=10090 GN=Ik PE=1 SV=2
Vacuolar protein sorting 13D OS=Mus musculus OX=10090 GN=Vps13d PE=1 SV=2
Phosphatidylserine synthase 1 OS=Mus musculus OX=10090 GN=Ptdss1 PE=1 SV=1
Maternal embryonic leucine zipper kinase OS=Mus musculus OX=10090 GN=Melk PE=1 SV=2
Transducin beta-like protein 2 OS=Mus musculus OX=10090 GN=Tbl2 PE=1 SV=2
Polyglutamine-binding protein 1 OS=Mus musculus OX=10090 GN=Pqbp1 PE=1 SV=1
Probable U3 small nucleolar RNA-associated protein 11 OS=Mus musculus OX=10090 GN=Utp11 PE=2 SV=1
NCK-interacting protein with SH3 domain OS=Mus musculus OX=10090 GN=Nckipsd PE=1 SV=2
Phospholipase DDHD1 OS=Mus musculus OX=10090 GN=Ddhd1 PE=1 SV=1
Heat shock protein 105 kDa OS=Mus musculus OX=10090 GN=Hsph1 PE=1 SV=1
Interferon-activable protein 203 OS=Mus musculus OX=10090 GN=Ifi203 PE=1 SV=1
Lysine-specific demethylase 9 OS=Mus musculus OX=10090 GN=Rsbn1 PE=1 SV=3
Ubiquitin-specific peptidase 31 OS=Mus musculus OX=10090 GN=Usp31 PE=1 SV=1
Dimethylaniline monooxygenase [N-oxide-forming] 1 OS=Mus musculus OX=10090 GN=Fmo1 PE=1 SV=1
Heat shock 70 kDa protein 12B OS=Mus musculus OX=10090 GN=Hspa12b PE=1 SV=1
Nek1 protein OS=Mus musculus OX=10090 GN=Nek1 PE=1 SV=1

Epidermal growth factor receptor kinase substrate 8-like protein 1 OS=Mus musculus OX=10090 GN=Eps8l1 PE=1
Tropomyosin alpha-1 chain OS=Mus musculus OX=10090 GN=Tpm1 PE=1 SV=1
ATPase family AAA domain-containing protein 2 OS=Mus musculus OX=10090 GN=Atad2 PE=1 SV=1
Tubulointerstitial nephritis antigen-like OS=Mus musculus OX=10090 GN=Tinagl1 PE=1 SV=1
Plastin-3 (Fragment) OS=Mus musculus OX=10090 GN=Pls3 PE=1 SV=1
Paralemmin A kinase anchor protein OS=Mus musculus OX=10090 GN=Pakap PE=1 SV=1
Threonine--tRNA ligase, mitochondrial (Fragment) OS=Mus musculus OX=10090 GN=Tars2 PE=1 SV=1
rRNA methyltransferase 1, mitochondrial OS=Mus musculus OX=10090 GN=Mrm1 PE=2 SV=2
Caveolae-associated protein 2 OS=Mus musculus OX=10090 GN=Cavin2 PE=1 SV=3
Ribosomal protein L36A, pseudogene 1 OS=Mus musculus OX=10090 GN=Rpl36a-ps1 PE=3 SV=1
Ortholog of human amyotrophic lateral sclerosis 2 (Juvenile) chromosome region, candidate 19 (ALS2CR19) OS=I
Myosin-binding protein H-like OS=Mus musculus OX=10090 GN=Mybphl PE=1 SV=1
Interferon-inducible double-stranded RNA-dependent protein kinase activator A OS=Mus musculus OX=10090 GN
F-actin-uncapping protein LRRC16A OS=Mus musculus OX=10090 GN=Carmil1 PE=1 SV=2
Platelet endothelial cell adhesion molecule OS=Mus musculus OX=10090 GN=Pecam1 PE=1 SV=1
Interleukin-1 receptor-associated kinase 4 OS=Mus musculus OX=10090 GN=Irak4 PE=1 SV=1
28S ribosomal protein S30, mitochondrial OS=Mus musculus OX=10090 GN=Mrps30 PE=1 SV=1
Pro-interleukin-16 OS=Mus musculus OX=10090 GN=Il16 PE=1 SV=3
Phosphatidylinositol 4-phosphate 5-kinase type-1 gamma OS=Mus musculus OX=10090 GN=Pip5k1c PE=1 SV=2
Translocation protein SEC62 OS=Mus musculus OX=10090 GN=Sec62 PE=1 SV=1
Uncharacterized protein C6orf47 homolog OS=Mus musculus OX=10090 GN=D17h6s53e PE=1 SV=1
AHNAK nucleoprotein (desmoyokin) OS=Mus musculus OX=10090 GN=Ahnak PE=1 SV=1
Ubiquitin-40S ribosomal protein S27a OS=Mus musculus OX=10090 GN=Rps27a PE=1 SV=2
Transmembrane protein KIAA1109 OS=Mus musculus OX=10090 GN=Kiaa1109 PE=1 SV=4
BTB/POZ domain-containing adapter for CUL3-mediated RhoA degradation protein 3 OS=Mus musculus OX=1009
Pre-mRNA-splicing factor RBM22 OS=Mus musculus OX=10090 GN=Rbm22 PE=1 SV=1
Sentrin-specific protease 1 OS=Mus musculus OX=10090 GN=Senp1 PE=1 SV=1
Peptidyl-prolyl cis-trans isomerase G OS=Mus musculus OX=10090 GN=Ppig PE=1 SV=1
TBC1 domain family member 10B OS=Mus musculus OX=10090 GN=Tbc1d10b PE=1 SV=2
Elongin-B OS=Mus musculus OX=10090 GN=Elob PE=1 SV=1
XIAP-associated factor 1 OS=Mus musculus OX=10090 GN=Xaf1 PE=1 SV=3
Centrosomal protein of 170 kDa OS=Mus musculus OX=10090 GN=Cep170 PE=1 SV=2
Tensin-2 OS=Mus musculus OX=10090 GN=Tns2 PE=1 SV=1
Density-regulated protein OS=Mus musculus OX=10090 GN=Denr PE=1 SV=1
Inositol 1,4,5-trisphosphate receptor type 3 OS=Mus musculus OX=10090 GN=Itpr3 PE=1 SV=3
Small nuclear ribonucleoprotein Sm D3 OS=Mus musculus OX=10090 GN=Snrpd3 PE=1 SV=1
39S ribosomal protein L11, mitochondrial (Fragment) OS=Mus musculus OX=10090 GN=Mrpl11 PE=1 SV=1
Zinc finger protein 449 OS=Mus musculus OX=10090 GN=Zfp449 PE=1 SV=1
NADH-cytochrome b5 reductase 3 OS=Mus musculus OX=10090 GN=Cyb5r3 PE=1 SV=3
Dipeptidase 1 OS=Mus musculus OX=10090 GN=Dpep1 PE=1 SV=2
Procollagen galactosyltransferase 1 OS=Mus musculus OX=10090 GN=Colgalt1 PE=1 SV=2
Rho GTPase-activating protein 23 OS=Mus musculus OX=10090 GN=Arhgap23 PE=1 SV=2
Vacuolar protein sorting-associated protein 18 homolog OS=Mus musculus OX=10090 GN=Vps18 PE=1 SV=2
Medium-chain acyl-CoA ligase ACSF2, mitochondrial OS=Mus musculus OX=10090 GN=Acsf2 PE=1 SV=1
Serine/threonine-protein phosphatase 2A 65 kDa regulatory subunit A alpha isoform OS=Mus musculus OX=1009

Predicted gene, 49711 OS=Mus musculus OX=10090 GN=Gm49711 PE=3 SV=1
Ubiquinol-cytochrome-c reductase complex assembly factor 1 OS=Mus musculus OX=10090 GN=Uqcc1 PE=1 SV=
Nucleolar protein 14 OS=Mus musculus OX=10090 GN=Nop14 PE=1 SV=2
Protein FAM32A OS=Mus musculus OX=10090 GN=Fam32a PE=2 SV=1
Lymphocyte-specific protein 1 OS=Mus musculus OX=10090 GN=Lsp1 PE=1 SV=2
Novel KRAB box and zinc finger, C2H2 type domain containing protein OS=Mus musculus OX=10090 GN=Gm143[illegible]
Heat shock cognate 71 kDa protein OS=Mus musculus OX=10090 GN=Hspa8 PE=1 SV=1
Dipeptidyl peptidase 4 OS=Mus musculus OX=10090 GN=Dpp4 PE=1 SV=3
Sorbin and SH3 domain-containing protein 1 OS=Mus musculus OX=10090 GN=Sorbs1 PE=1 SV=1
ADP-ribosylation factor 5 OS=Mus musculus OX=10090 GN=Arf5 PE=1 SV=2
Beta-hexosaminidase subunit alpha OS=Mus musculus OX=10090 GN=Hexa PE=1 SV=2
Tyrosine-protein phosphatase non-receptor type 12 OS=Mus musculus OX=10090 GN=Ptpn12 PE=1 SV=3
Rootletin OS=Mus musculus OX=10090 GN=Crocc PE=1 SV=2
Nucleotide-binding oligomerization domain-containing protein 1 OS=Mus musculus OX=10090 GN=Nod1 PE=1 S[illegible]
Nuclear autoantigen Sp-100 OS=Mus musculus OX=10090 GN=Sp100 PE=1 SV=1
CST complex subunit CTC1 OS=Mus musculus OX=10090 GN=Ctc1 PE=1 SV=1
Ras-related protein Rap-2a OS=Mus musculus OX=10090 GN=Rap2a PE=1 SV=2
Ras GTPase-activating-like protein IQGAP1 OS=Mus musculus OX=10090 GN=Iqgap1 PE=1 SV=2
Arf-GAP with Rho-GAP domain, ANK repeat and PH domain-containing protein 3 OS=Mus musculus OX=10090 G[illegible]
2-aminoethanethiol dioxygenase OS=Mus musculus OX=10090 GN=Ado PE=1 SV=2
Transformer-2 protein homolog alpha OS=Mus musculus OX=10090 GN=Tra2a PE=1 SV=1
Zinc finger protein 112 OS=Mus musculus OX=10090 GN=Znf112 PE=2 SV=2
Collagen alpha-1(XIV) chain OS=Mus musculus OX=10090 GN=Col14a1 PE=1 SV=2
Flt3-interacting zinc finger protein 1 OS=Mus musculus OX=10090 GN=Fiz1 PE=1 SV=1
Nucleoporin SEH1 OS=Mus musculus OX=10090 GN=Seh1l PE=2 SV=1
Cytochrome c oxidase subunit 5A, mitochondrial OS=Mus musculus OX=10090 GN=Cox5a PE=1 SV=2
UPF0461 protein C5orf24 homolog OS=Mus musculus OX=10090 PE=2 SV=1
Resistin-like gamma OS=Mus musculus OX=10090 GN=Retnlg PE=1 SV=1
Serine/threonine-protein phosphatase PP1-alpha catalytic subunit OS=Mus musculus OX=10090 GN=Ppp1ca PE=[illegible]
tRNA methyltransferase 10 homolog C OS=Mus musculus OX=10090 GN=Trmt10c PE=1 SV=2
Heat shock protein HSP 90-alpha OS=Mus musculus OX=10090 GN=Hsp90aa1 PE=1 SV=4
Poly [ADP-ribose] polymerase OS=Mus musculus OX=10090 GN=Parp1 PE=1 SV=1
DNA polymerase delta catalytic subunit OS=Mus musculus OX=10090 GN=Pold1 PE=1 SV=2
Histone H1.0 OS=Mus musculus OX=10090 GN=H1-0 PE=2 SV=4
Ral GTPase-activating protein subunit beta OS=Mus musculus OX=10090 GN=Ralgapb PE=1 SV=1
Nesprin-2 OS=Mus musculus OX=10090 GN=Syne2 PE=1 SV=2
Rho GTPase-activating protein 18 OS=Mus musculus OX=10090 GN=Arhgap18 PE=1 SV=1
Centrosomal protein of 135 kDa OS=Mus musculus OX=10090 GN=Cep135 PE=1 SV=1
Calcium-binding and coiled-coil domain-containing protein 1 OS=Mus musculus OX=10090 GN=Calcoco1 PE=1 SV
Exosome complex component MTR3 OS=Mus musculus OX=10090 GN=Exosc6 PE=1 SV=1
Peripheral plasma membrane protein CASK OS=Mus musculus OX=10090 GN=Cask PE=1 SV=2
SCAN domain-containing 1 OS=Mus musculus OX=10090 GN=Scand1 PE=1 SV=1
39S ribosomal protein L12, mitochondrial OS=Mus musculus OX=10090 GN=Mrpl12 PE=1 SV=2
Tuberin OS=Mus musculus OX=10090 GN=Tsc2 PE=1 SV=1
Disks large homolog 5 OS=Mus musculus OX=10090 GN=Dlg5 PE=1 SV=1

39S ribosomal protein L32, mitochondrial OS=Mus musculus OX=10090 GN=Mrpl32 PE=1 SV=1
Homeobox-containing protein 1 OS=Mus musculus OX=10090 GN=Hmbox1 PE=1 SV=1
Cerebral cavernous malformations protein 2 homolog (Fragment) OS=Mus musculus OX=10090 GN=Ccm2 PE=1 S
60S acidic ribosomal protein P0 OS=Mus musculus OX=10090 GN=Rplp0 PE=1 SV=3
PWWP domain-containing 2B OS=Mus musculus OX=10090 GN=Pwwp2b PE=4 SV=1
Vacuole membrane protein 1 (Fragment) OS=Mus musculus OX=10090 GN=Vmp1 PE=1 SV=1
Acylphosphatase-2 OS=Mus musculus OX=10090 GN=Acyp2 PE=1 SV=2
TBC1 domain family, member 22B OS=Mus musculus OX=10090 GN=Tbc1d22b PE=1 SV=1
Signal-induced proliferation-associated 1-like protein 3 OS=Mus musculus OX=10090 GN=Sipa1l3 PE=1 SV=1
Nestin OS=Mus musculus OX=10090 GN=Nes PE=1 SV=1
ATP synthase F(0) complex subunit B1, mitochondrial OS=Mus musculus OX=10090 GN=Atp5pb PE=1 SV=1
Cytochrome c1, heme protein, mitochondrial OS=Mus musculus OX=10090 GN=Cyc1 PE=1 SV=1
Protein FAM114A2 OS=Mus musculus OX=10090 GN=Fam114a2 PE=1 SV=2
DnaJ homolog subfamily A member 2 OS=Mus musculus OX=10090 GN=Dnaja2 PE=1 SV=1
Guanylate-binding protein 4 OS=Mus musculus OX=10090 GN=Gbp4 PE=1 SV=1
Transcription initiation factor IIB OS=Mus musculus OX=10090 GN=Gtf2b PE=1 SV=1
Lysine-specific demethylase 2A OS=Mus musculus OX=10090 GN=Kdm2a PE=1 SV=2
Lysophosphatidylcholine acyltransferase 1 OS=Mus musculus OX=10090 GN=Lpcat1 PE=1 SV=1
Fragile X mental retardation syndrome-related protein 1 OS=Mus musculus OX=10090 GN=Fxr1 PE=1 SV=2
Tyrosine-protein kinase OS=Mus musculus OX=10090 GN=Fes PE=1 SV=1
Zinc finger and BTB domain-containing 16 OS=Mus musculus OX=10090 GN=Zbtb16 PE=1 SV=1
Secreted frizzled-related protein 1 OS=Mus musculus OX=10090 GN=Sfrp1 PE=1 SV=3
Rho guanine nucleotide exchange factor 7 OS=Mus musculus OX=10090 GN=Arhgef7 PE=1 SV=1
Histone-arginine methyltransferase CARM1 OS=Mus musculus OX=10090 GN=Carm1 PE=1 SV=2
Collagen alpha-2(IV) chain OS=Mus musculus OX=10090 GN=Col4a2 PE=1 SV=4
Cyclin-dependent kinase 9 OS=Mus musculus OX=10090 GN=Cdk9 PE=1 SV=1
Testin OS=Mus musculus OX=10090 GN=Tes PE=1 SV=1
Zinc finger CCCH domain-containing protein 10 OS=Mus musculus OX=10090 GN=Zc3h10 PE=1 SV=1
Major vault protein OS=Mus musculus OX=10090 GN=Mvp PE=1 SV=4
Brefeldin A-inhibited guanine nucleotide-exchange protein 1 OS=Mus musculus OX=10090 GN=Arfgef1 PE=1 SV=
NEDD4-like E3 ubiquitin-protein ligase WWP1 OS=Mus musculus OX=10090 GN=Wwp1 PE=1 SV=2
Pro-cathepsin H OS=Mus musculus OX=10090 GN=Ctsh PE=1 SV=2
Sorting nexin-27 OS=Mus musculus OX=10090 GN=Snx27 PE=1 SV=2
Membrane-associated progesterone receptor component 1 OS=Mus musculus OX=10090 GN=Pgrmc1 PE=1 SV=4
Myosin phosphatase Rho-interacting protein OS=Mus musculus OX=10090 GN=Mprip PE=1 SV=2
Wilms tumor protein 1-interacting protein OS=Mus musculus OX=10090 GN=Wtip PE=1 SV=1
Zinc finger protein 60 OS=Mus musculus OX=10090 GN=Zfp60 PE=2 SV=2
PR domain zinc finger protein 10 OS=Mus musculus OX=10090 GN=Prdm10 PE=1 SV=1
Scaffold attachment factor B2 OS=Mus musculus OX=10090 GN=Safb2 PE=1 SV=2
Splicing factor 1 OS=Mus musculus OX=10090 GN=Sf1 PE=1 SV=1
Protein disulfide-isomerase A3 OS=Mus musculus OX=10090 GN=Pdia3 PE=1 SV=2
STAGA complex 65 subunit gamma OS=Mus musculus OX=10090 GN=Supt7l PE=1 SV=1
Caspase recruitment domain-containing protein 9 OS=Mus musculus OX=10090 GN=Card9 PE=1 SV=1
Pre-mRNA-splicing factor CWC25 homolog OS=Mus musculus OX=10090 GN=Cwc25 PE=2 SV=2
Cytochrome b-245 chaperone 1 OS=Mus musculus OX=10090 GN=Cybc1 PE=1 SV=2

G kinase-anchoring protein 1 OS=Mus musculus OX=10090 GN=Gkap1 PE=1 SV=1
Metastasis-associated protein MTA1 OS=Mus musculus OX=10090 GN=Mta1 PE=1 SV=1
Serine palmitoyltransferase 1 OS=Mus musculus OX=10090 GN=Sptlc1 PE=1 SV=2
Protein PRRC2A OS=Mus musculus OX=10090 GN=Prrc2a PE=1 SV=1
Actin, alpha cardiac muscle 1 OS=Mus musculus OX=10090 GN=Actc1 PE=1 SV=1
Podocalyxin OS=Mus musculus OX=10090 GN=Podxl PE=1 SV=2
LIM and senescent cell antigen-like-containing domain protein OS=Mus musculus OX=10090 GN=Lims1 PE=1 SV=
E3 SUMO-protein ligase EGR2 OS=Mus musculus OX=10090 GN=Egr2 PE=1 SV=1
Cathepsin K OS=Mus musculus OX=10090 GN=Ctsk PE=1 SV=2
Uracil phosphoribosyltransferase homolog OS=Mus musculus OX=10090 GN=Uprt PE=1 SV=1
CST complex subunit STN1 OS=Mus musculus OX=10090 GN=Stn1 PE=1 SV=1
Arginine vasopressin-induced protein 1 OS=Mus musculus OX=10090 GN=Avpi1 PE=2 SV=1
Leucine-rich repeat-containing protein 41 OS=Mus musculus OX=10090 GN=Lrrc41 PE=1 SV=3
Tensin 1 OS=Mus musculus OX=10090 GN=Tns1 PE=1 SV=1
Regulator of chromosome condensation OS=Mus musculus OX=10090 GN=Rcc1 PE=1 SV=1
F-box/LRR-repeat protein 14 OS=Mus musculus OX=10090 GN=Fbxl14 PE=2 SV=1
Histone-lysine N-methyltransferase NSD2 OS=Mus musculus OX=10090 GN=Nsd2 PE=1 SV=2
Kelch repeat and BTB (POZ) domain-containing 2 OS=Mus musculus OX=10090 GN=Kbtbd2 PE=1 SV=1
CAAX box 1 homolog A (Human) OS=Mus musculus OX=10090 GN=Rtl8b PE=1 SV=1
CD2-associated protein OS=Mus musculus OX=10090 GN=Cd2ap PE=1 SV=3
FLYWCH-type zinc finger-containing protein 1 OS=Mus musculus OX=10090 GN=Flywch1 PE=1 SV=2
RIKEN cDNA 9930111J21 gene 1 OS=Mus musculus OX=10090 GN=9930111J21Rik1 PE=1 SV=1
Dapper homolog 3 OS=Mus musculus OX=10090 GN=Dact3 PE=1 SV=1
Probable ATP-dependent RNA helicase DDX20 OS=Mus musculus OX=10090 GN=Ddx20 PE=1 SV=2
Hyaluronan mediated motility receptor OS=Mus musculus OX=10090 GN=Hmmr PE=1 SV=4
SWI/SNF-related matrix-associated actin-dependent regulator of chromatin subfamily A containing DEAD/H box
Fragile X mental retardation syndrome-related protein 2 OS=Mus musculus OX=10090 GN=Fxr2 PE=1 SV=1
Dihydrolipoyllysine-residue succinyltransferase component of 2-oxoglutarate dehydrogenase complex, mitochonc
Lamin-B2 OS=Mus musculus OX=10090 GN=Lmnb2 PE=1 SV=1
Collagen alpha-2(VI) chain OS=Mus musculus OX=10090 GN=Col6a2 PE=1 SV=3
Succinate--CoA ligase [ADP/GDP-forming] subunit alpha, mitochondrial OS=Mus musculus OX=10090 GN=Suclg1
DnaJ homolog subfamily C member 25 OS=Mus musculus OX=10090 GN=Dnajc25 PE=1 SV=1
14-3-3 protein zeta/delta OS=Mus musculus OX=10090 GN=Ywhaz PE=1 SV=1
Serine/threonine-protein kinase TBK1 OS=Mus musculus OX=10090 GN=Tbk1 PE=1 SV=1
Phosphatidylinositol 5-phosphate 4-kinase type-2 gamma OS=Mus musculus OX=10090 GN=Pip4k2c PE=1 SV=1
cAMP-dependent protein kinase catalytic subunit alpha OS=Mus musculus OX=10090 GN=Prkaca PE=1 SV=3
28S ribosomal protein S27, mitochondrial OS=Mus musculus OX=10090 GN=Mrps27 PE=1 SV=2
Parathymosin OS=Mus musculus OX=10090 GN=Ptms PE=1 SV=3
MBT domain-containing protein 1 OS=Mus musculus OX=10090 GN=Mbtd1 PE=1 SV=1
GRB2-related adaptor protein 2 OS=Mus musculus OX=10090 GN=Grap2 PE=1 SV=1
CCAAT/enhancer-binding protein zeta OS=Mus musculus OX=10090 GN=Cebpz PE=1 SV=1
Proline-rich protein 14 OS=Mus musculus OX=10090 GN=Prr14 PE=1 SV=1
Tyrosine-protein phosphatase non-receptor type 4 OS=Mus musculus OX=10090 GN=Ptpn4 PE=1 SV=2
Centromere protein V OS=Mus musculus OX=10090 GN=Cenpv PE=1 SV=2
SH3 domain and tetratricopeptide repeats 1 OS=Mus musculus OX=10090 GN=Sh3tc1 PE=1 SV=1

Hdc homolog, cell cycle regulator OS=Mus musculus OX=10090 GN=Heca PE=1 SV=1
Huntingtin-interacting protein 1-related protein OS=Mus musculus OX=10090 GN=Hip1r PE=1 SV=2
Mediator of RNA polymerase II transcription subunit 4 OS=Mus musculus OX=10090 GN=Med4 PE=2 SV=1
Transcriptional repressor protein YY1 OS=Mus musculus OX=10090 GN=Yy1 PE=1 SV=1
Interferon-inducible GTPase 1 OS=Mus musculus OX=10090 GN=Iigp1 PE=1 SV=2
Serine (or cysteine) peptidase inhibitor, clade B, member 6a OS=Mus musculus OX=10090 GN=Serpinb6a PE=1 S
Testis-expressed protein 10 OS=Mus musculus OX=10090 GN=Tex10 PE=1 SV=1
40S ribosomal protein S3a OS=Mus musculus OX=10090 GN=Rps3a PE=1 SV=3
NHS-like protein 2 OS=Mus musculus OX=10090 GN=Nhsl2 PE=1 SV=2
PHD finger protein 3 OS=Mus musculus OX=10090 GN=Phf3 PE=1 SV=1
SPATS2-like protein OS=Mus musculus OX=10090 GN=Spats2l PE=2 SV=2
28S ribosomal protein S34, mitochondrial OS=Mus musculus OX=10090 GN=Mrps34 PE=1 SV=1
Platelet-derived growth factor subunit B OS=Mus musculus OX=10090 GN=Pdgfb PE=2 SV=1
Serine/arginine repetitive matrix protein 2 OS=Mus musculus OX=10090 GN=Srrm2 PE=1 SV=3
Rho guanine nucleotide exchange factor 6 OS=Mus musculus OX=10090 GN=Arhgef6 PE=1 SV=1
BRISC and BRCA1-A complex member 2 OS=Mus musculus OX=10090 GN=Babam2 PE=1 SV=3
Cyclin-dependent kinase 11B OS=Mus musculus OX=10090 GN=Cdk11b PE=1 SV=2
Lysosomal-trafficking regulator OS=Mus musculus OX=10090 GN=Lyst PE=1 SV=1
H-2 class I histocompatibility antigen, D-37 alpha chain OS=Mus musculus OX=10090 GN=H2-T23 PE=1 SV=1
Proliferation marker protein Ki-67 OS=Mus musculus OX=10090 GN=Mki67 PE=1 SV=1
3-oxoacyl-[acyl-carrier-protein] reductase OS=Mus musculus OX=10090 GN=Cbr4 PE=1 SV=2
Sorting nexin-2 OS=Mus musculus OX=10090 GN=Snx2 PE=1 SV=2
Testis-specific Y-encoded-like protein 5 OS=Mus musculus OX=10090 GN=Tspyl5 PE=2 SV=2
Olfactory receptor OS=Mus musculus OX=10090 GN=Olfr608 PE=3 SV=1
RNA helicase OS=Mus musculus OX=10090 GN=Eif4a2 PE=1 SV=1
WD repeat-containing protein 46 OS=Mus musculus OX=10090 GN=Wdr46 PE=2 SV=1
Ras-related protein Rab-7a OS=Mus musculus OX=10090 GN=Rab7a PE=1 SV=2
Protein LSM14 homolog B OS=Mus musculus OX=10090 GN=Lsm14b PE=1 SV=3
KRR1 small subunit processome component homolog OS=Mus musculus OX=10090 GN=Krr1 PE=2 SV=1
Heme-binding protein 1 OS=Mus musculus OX=10090 GN=Hebp1 PE=1 SV=2
Arf-GAP with SH3 domain, ANK repeat and PH domain-containing protein 2 OS=Mus musculus OX=10090 GN=As
Golgin subfamily A member 3 OS=Mus musculus OX=10090 GN=Golga3 PE=1 SV=1
Tropomyosin 3, related sequence 7 OS=Mus musculus OX=10090 GN=Tpm3-rs7 PE=3 SV=1
Ankyrin repeat and zinc finger domain-containing protein 1 OS=Mus musculus OX=10090 GN=Ankzf1 PE=1 SV=2
Dedicator of cytokinesis protein 7 OS=Mus musculus OX=10090 GN=Dock7 PE=1 SV=2
Predicted gene 14399 OS=Mus musculus OX=10090 GN=Gm14399 PE=4 SV=1
Histone H3.1 OS=Mus musculus OX=10090 GN=H3c1 PE=1 SV=2
40S ribosomal protein S2 OS=Mus musculus OX=10090 GN=Rps2 PE=1 SV=3
Catenin delta-1 OS=Mus musculus OX=10090 GN=Ctnnd1 PE=1 SV=1
ATP-dependent zinc metalloprotease YME1L1 OS=Mus musculus OX=10090 GN=Yme1l1 PE=1 SV=1
Sphingosine kinase 2 OS=Mus musculus OX=10090 GN=Sphk2 PE=1 SV=2
Coiled-coil domain-containing 191 (Fragment) OS=Mus musculus OX=10090 GN=Ccdc191 PE=1 SV=1
Protein YIF1B (Fragment) OS=Mus musculus OX=10090 GN=Yif1b PE=1 SV=1
GTPase IMAP family member 6 OS=Mus musculus OX=10090 GN=Gimap6 PE=2 SV=1
Zinc finger and SCAN domain-containing protein 26 OS=Mus musculus OX=10090 GN=Zscan26 PE=2 SV=1

Transcription elongation factor, mitochondrial OS=Mus musculus OX=10090 GN=Tefm PE=1 SV=1
Elongation factor 1-delta OS=Mus musculus OX=10090 GN=Eef1d PE=1 SV=1
ATP-dependent RNA helicase DDX3Y OS=Mus musculus OX=10090 GN=Ddx3y PE=1 SV=2
Multiple C2 and transmembrane domain-containing protein 1 OS=Mus musculus OX=10090 GN=Mctp1 PE=1 SV=
Mitogen-activated protein kinase-binding protein 1 OS=Mus musculus OX=10090 GN=Mapkbp1 PE=1 SV=1
Hepatoma-derived growth factor-related protein 3 OS=Mus musculus OX=10090 GN=Hdgfl3 PE=1 SV=2
Probable ATP-dependent RNA helicase DHX34 OS=Mus musculus OX=10090 GN=Dhx34 PE=1 SV=2
ATP synthase subunit d, mitochondrial OS=Mus musculus OX=10090 GN=Atp5pd PE=1 SV=3
Tropomodulin-3 OS=Mus musculus OX=10090 GN=Tmod3 PE=1 SV=1
Glyoxylate reductase 1 homolog OS=Mus musculus OX=10090 GN=Glyr1 PE=1 SV=1
Annexin A1 OS=Mus musculus OX=10090 GN=Anxa1 PE=1 SV=2
Alpha-actinin-4 OS=Mus musculus OX=10090 GN=Actn4 PE=1 SV=1
Tripeptidyl-peptidase 1 OS=Mus musculus OX=10090 GN=Tpp1 PE=1 SV=2
Charged multivesicular body protein 1b-2 OS=Mus musculus OX=10090 GN=Chmp1b2 PE=2 SV=2
A-kinase anchor protein 9 OS=Mus musculus OX=10090 GN=Akap9 PE=1 SV=2
Glutamate-rich WD repeat-containing protein 1 OS=Mus musculus OX=10090 GN=Grwd1 PE=1 SV=2
C-X-C motif chemokine 12 OS=Mus musculus OX=10090 GN=Cxcl12 PE=1 SV=1
Zinc finger protein 771 OS=Mus musculus OX=10090 GN=Znf771 PE=2 SV=1
Long-chain-fatty-acid--CoA ligase 4 OS=Mus musculus OX=10090 GN=Acsl4 PE=1 SV=2
Peptidyl-prolyl cis-trans isomerase FKBP4 OS=Mus musculus OX=10090 GN=Fkbp4 PE=1 SV=5
ADP-ribosylation factor-like protein 8A OS=Mus musculus OX=10090 GN=Arl8a PE=1 SV=1
Ribonuclease P protein subunit p25-like protein OS=Mus musculus OX=10090 GN=Rpp25l PE=1 SV=1
Mediator of RNA polymerase II transcription subunit 16 OS=Mus musculus OX=10090 GN=Med16 PE=2 SV=2
Putative 60S ribosomal protein L32' OS=Mus musculus OX=10090 GN=Rpl32-ps PE=5 SV=2
StAR-related lipid transfer protein 8 OS=Mus musculus OX=10090 GN=Stard8 PE=1 SV=2
Sodium-coupled neutral amino acid transporter 9 OS=Mus musculus OX=10090 GN=Slc38a9 PE=1 SV=1
Nipped-B-like protein OS=Mus musculus OX=10090 GN=Nipbl PE=1 SV=1
Annexin A11 OS=Mus musculus OX=10090 GN=Anxa11 PE=1 SV=2
Tripartite motif-containing protein 14 OS=Mus musculus OX=10090 GN=Trim14 PE=1 SV=2
Transcription factor Sp1 OS=Mus musculus OX=10090 GN=Sp1 PE=1 SV=2
Nuclear transcription factor, X-box-binding-like 1 OS=Mus musculus OX=10090 GN=Nfxl1 PE=1 SV=1
Mitochondrial-processing peptidase subunit beta OS=Mus musculus OX=10090 GN=Pmpcb PE=1 SV=1
ATP synthase membrane subunit K, mitochondrial OS=Mus musculus OX=10090 GN=Atp5mk PE=1 SV=1
Lysine-specific demethylase 4B OS=Mus musculus OX=10090 GN=Kdm4b PE=2 SV=1
CDGSH iron-sulfur domain-containing protein 3, mitochondrial OS=Mus musculus OX=10090 GN=Cisd3 PE=1 SV=
182 kDa tankyrase-1-binding protein OS=Mus musculus OX=10090 GN=Tnks1bp1 PE=1 SV=2
Importin-5 OS=Mus musculus OX=10090 GN=Ipo5 PE=1 SV=3
Integrator complex subunit 4 OS=Mus musculus OX=10090 GN=Ints4 PE=1 SV=1
Ribosomal RNA-processing protein 8 OS=Mus musculus OX=10090 GN=Rrp8 PE=1 SV=1
Calcium-binding protein 39-like OS=Mus musculus OX=10090 GN=Cab39l PE=1 SV=3
39S ribosomal protein L46, mitochondrial OS=Mus musculus OX=10090 GN=Mrpl46 PE=1 SV=1
ATP-binding cassette sub-family D member 2 OS=Mus musculus OX=10090 GN=Abcd2 PE=1 SV=1
Tripartite motif-containing protein 2 OS=Mus musculus OX=10090 GN=Trim2 PE=1 SV=1
GTP-binding protein OS=Mus musculus OX=10090 GN=Ifi47 PE=1 SV=1
E3 ubiquitin-protein ligase HERC2 OS=Mus musculus OX=10090 GN=Herc2 PE=1 SV=3

DnaJ homolog subfamily A member 4 OS=Mus musculus OX=10090 GN=Dnaja4 PE=1 SV=1
Zinc finger protein 362 OS=Mus musculus OX=10090 GN=Zfp362 PE=1 SV=1
Ribosome production factor 2 homolog OS=Mus musculus OX=10090 GN=Rpf2 PE=1 SV=1
CtBP-interacting BTB zinc finger protein OS=Mus musculus OX=10090 GN=Zbtb38 PE=1 SV=1
Differentially expressed in FDCP 6 OS=Mus musculus OX=10090 GN=Def6 PE=1 SV=1
Leucine-rich repeat protein SHOC-2 (Fragment) OS=Mus musculus OX=10090 GN=Shoc2 PE=1 SV=1
Band 4.1-like protein 4B OS=Mus musculus OX=10090 GN=Epb41l4b PE=1 SV=2
3-beta-hydroxysteroid-Delta(8),Delta(7)-isomerase OS=Mus musculus OX=10090 GN=Ebp PE=1 SV=3
Septin-6 OS=Mus musculus OX=10090 GN=Septin6 PE=1 SV=4
Zinc finger protein 319 OS=Mus musculus OX=10090 GN=Znf319 PE=2 SV=1
40S ribosomal protein S8 OS=Mus musculus OX=10090 GN=Rps8 PE=1 SV=2
Muscleblind-like protein 1 OS=Mus musculus OX=10090 GN=Mbnl1 PE=1 SV=1
Vang-like protein OS=Mus musculus OX=10090 GN=Vangl2 PE=1 SV=1
WD repeat and FYVE domain-containing protein 3 OS=Mus musculus OX=10090 GN=Wdfy3 PE=1 SV=1
Receptor-type tyrosine-protein phosphatase U OS=Mus musculus OX=10090 GN=Ptpru PE=1 SV=1
Alpha-crystallin B chain (Fragment) OS=Mus musculus OX=10090 GN=Cryab PE=1 SV=1
Biogenesis of lysosome-related organelles complex 1 subunit 5 OS=Mus musculus OX=10090 GN=Bloc1s5 PE=1 S
Cytochrome c oxidase subunit 6B1 OS=Mus musculus OX=10090 GN=Cox6b1 PE=1 SV=1
CLIP-associating protein 2 OS=Mus musculus OX=10090 GN=Clasp2 PE=1 SV=1
Methyl-CpG-binding domain protein 2 OS=Mus musculus OX=10090 GN=Mbd2 PE=2 SV=2
Rho GTPase-activating protein 42 (Fragment) OS=Mus musculus OX=10090 GN=Arhgap42 PE=1 SV=1
Fizzy-related protein homolog OS=Mus musculus OX=10090 GN=Fzr1 PE=1 SV=1
Cysteine protease OS=Mus musculus OX=10090 GN=Atg4b PE=1 SV=1
DAZ-associated protein 1 OS=Mus musculus OX=10090 GN=Dazap1 PE=1 SV=2
Peroxisomal multifunctional enzyme type 2 OS=Mus musculus OX=10090 GN=Hsd17b4 PE=1 SV=3
Retinoblastoma-binding protein 5 OS=Mus musculus OX=10090 GN=Rbbp5 PE=1 SV=1
CBP80/20-dependent translation initiation factor OS=Mus musculus OX=10090 GN=Ctif PE=1 SV=2
60S ribosomal protein L23 OS=Mus musculus OX=10090 GN=Rpl23 PE=1 SV=1
Calponin-2 OS=Mus musculus OX=10090 GN=Cnn2 PE=1 SV=1
Clathrin interactor 1 OS=Mus musculus OX=10090 GN=Clint1 PE=1 SV=1
UBX domain-containing protein 6 OS=Mus musculus OX=10090 GN=Ubxn6 PE=1 SV=1
Kinase suppressor of Ras 1 (Fragment) OS=Mus musculus OX=10090 GN=Ksr1 PE=1 SV=1
Ribosomal L1 domain-containing protein 1 OS=Mus musculus OX=10090 GN=Rsl1d1 PE=1 SV=1
Nucleolar transcription factor 1 OS=Mus musculus OX=10090 GN=Ubtf PE=1 SV=1
RIKEN cDNA 9130019O22 gene OS=Mus musculus OX=10090 GN=9130019O22Rik PE=4 SV=1
Histone-lysine N-methyltransferase EHMT2 OS=Mus musculus OX=10090 GN=Ehmt2 PE=1 SV=1
Transcription activator BRG1 OS=Mus musculus OX=10090 GN=Smarca4 PE=1 SV=1
Coiled-coil domain-containing protein 138 OS=Mus musculus OX=10090 GN=Ccdc138 PE=1 SV=1
Diacylglycerol kinase OS=Mus musculus OX=10090 GN=Dgkz PE=1 SV=1
Rho GTPase-activating protein 17 OS=Mus musculus OX=10090 GN=Arhgap17 PE=1 SV=1
Tyrosine-protein kinase OS=Mus musculus OX=10090 GN=Btk PE=1 SV=1
Actin-related protein 3 OS=Mus musculus OX=10090 GN=Actr3 PE=1 SV=3
ATP-binding cassette sub-family D member 3 OS=Mus musculus OX=10090 GN=Abcd3 PE=1 SV=2
AP-5 complex subunit zeta-1 OS=Mus musculus OX=10090 GN=Ap5z1 PE=2 SV=1
Zinc finger and BTB domain-containing protein 20 OS=Mus musculus OX=10090 GN=Zbtb20 PE=1 SV=1

Solute carrier family 12 member 7 OS=Mus musculus OX=10090 GN=Slc12a7 PE=1 SV=1
Leucine-rich repeat flightless-interacting protein 1 (Fragment) OS=Mus musculus OX=10090 GN=Lrrfip1 PE=1 SV
Integrator complex subunit 1 OS=Mus musculus OX=10090 GN=Ints1 PE=1 SV=2
TGF-beta receptor type-2 OS=Mus musculus OX=10090 GN=Tgfbr2 PE=1 SV=1
Nucleolar GTP-binding protein 2 OS=Mus musculus OX=10090 GN=Gnl2 PE=1 SV=2
Breakpoint cluster region protein OS=Mus musculus OX=10090 GN=Bcr PE=1 SV=3
26S proteasome AAA-ATPase subunit RPT1 OS=Mus musculus OX=10090 GN=Psmc2 PE=1 SV=1
RNA-binding protein 28 OS=Mus musculus OX=10090 GN=Rbm28 PE=1 SV=4
Pre-mRNA-splicing factor 38A OS=Mus musculus OX=10090 GN=Prpf38a PE=1 SV=1
Tubulin beta-6 chain OS=Mus musculus OX=10090 GN=Tubb6 PE=1 SV=1
Transformer-2 protein homolog beta OS=Mus musculus OX=10090 GN=Tra2b PE=1 SV=1
Kinesin-like protein KIF1B OS=Mus musculus OX=10090 GN=Kif1b PE=1 SV=1
Triosephosphate isomerase OS=Mus musculus OX=10090 GN=Tpi1 PE=1 SV=1
Thioredoxin domain-containing protein 11 OS=Mus musculus OX=10090 GN=Txndc11 PE=1 SV=1
Selenocysteine-specific elongation factor OS=Mus musculus OX=10090 GN=Eefsec PE=1 SV=1
Sister chromatid cohesion protein PDS5 homolog A OS=Mus musculus OX=10090 GN=Pds5a PE=1 SV=1
Syntaxin-binding protein 3 (Fragment) OS=Mus musculus OX=10090 GN=Stxbp3 PE=1 SV=1
Nuclear ubiquitous casein and cyclin-dependent kinase substrate 1 OS=Mus musculus OX=10090 GN=Nucks1 PE=
Alanine--tRNA ligase, cytoplasmic OS=Mus musculus OX=10090 GN=Aars1 PE=1 SV=1
DnaJ homolog subfamily A member 3, mitochondrial OS=Mus musculus OX=10090 GN=Dnaja3 PE=1 SV=1
Caspase recruitment domain family, member 6 OS=Mus musculus OX=10090 GN=Card6 PE=1 SV=1
Nuclear autoantigen Sp-100 OS=Mus musculus OX=10090 GN=Sp100 PE=1 SV=1
SIN3-HDAC complex-associated factor (Fragment) OS=Mus musculus OX=10090 GN=Sinhcaf PE=1 SV=1
Complement component C1q receptor OS=Mus musculus OX=10090 GN=Cd93 PE=1 SV=1
Homer protein homolog 3 OS=Mus musculus OX=10090 GN=Homer3 PE=1 SV=2
Protein phosphatase 1 regulatory inhibitor subunit 16B OS=Mus musculus OX=10090 GN=Ppp1r16b PE=1 SV=1
ATP-dependent RNA helicase DDX3X OS=Mus musculus OX=10090 GN=Ddx3x PE=1 SV=3
Heterogeneous nuclear ribonucleoprotein U OS=Mus musculus OX=10090 GN=Hnrnpu PE=1 SV=1
Death-associated protein kinase 2 OS=Mus musculus OX=10090 GN=Dapk2 PE=1 SV=1
AT-rich interactive domain-containing protein 1A OS=Mus musculus OX=10090 GN=Arid1a PE=1 SV=1
Pleckstrin homology domain-containing family G member 3 OS=Mus musculus OX=10090 GN=Plekhg3 PE=1 SV=[illegible]
Ubiquitin carboxyl-terminal hydrolase 16 OS=Mus musculus OX=10090 GN=Usp16 PE=1 SV=2
RUN and FYVE domain-containing protein 1 OS=Mus musculus OX=10090 GN=Rufy1 PE=1 SV=1
RNA-binding protein 15 OS=Mus musculus OX=10090 GN=Rbm15 PE=1 SV=1
Extracellular matrix protein 1 OS=Mus musculus OX=10090 GN=Ecm1 PE=1 SV=1
Diacylglycerol kinase epsilon OS=Mus musculus OX=10090 GN=Dgke PE=1 SV=1
Non-specific serine/threonine protein kinase OS=Mus musculus OX=10090 GN=Mast4 PE=1 SV=1
Histone deacetylase 3 OS=Mus musculus OX=10090 GN=Hdac3 PE=1 SV=1
Predicted pseudogene 10320 OS=Mus musculus OX=10090 GN=Gm10320 PE=3 SV=1
Clathrin light chain B OS=Mus musculus OX=10090 GN=Cltb PE=1 SV=1
PHD finger protein 11 OS=Mus musculus OX=10090 GN=Phf11 PE=2 SV=1
Integrator complex subunit 3 OS=Mus musculus OX=10090 GN=Ints3 PE=1 SV=1
5'-3' exonuclease PLD4 OS=Mus musculus OX=10090 GN=Pld4 PE=1 SV=1
Mitochondrial nucleoid factor 1 OS=Mus musculus OX=10090 GN=Uqcc2 PE=1 SV=1
E3 ubiquitin-protein ligase UBR5 OS=Mus musculus OX=10090 GN=Ubr5 PE=1 SV=2

Leukocyte elastase inhibitor A (Fragment) OS=Mus musculus OX=10090 GN=Serpinb1a PE=1 SV=2
Extracellular sulfatase Sulf-2 OS=Mus musculus OX=10090 GN=Sulf2 PE=2 SV=2
Microtubule-associated protein RP/EB family member 2 (Fragment) OS=Mus musculus OX=10090 GN=Mapre2 P
18 kDa Sin3-associated polypeptide OS=Mus musculus OX=10090 GN=Sap18 PE=1 SV=1
Myotubularin-related protein 2 OS=Mus musculus OX=10090 GN=Mtmr2 PE=1 SV=3
Schlafen family member 5 OS=Mus musculus OX=10090 GN=Slfn5 PE=1 SV=2
[Histone H3]-dimethyl-L-lysine(36) demethylase OS=Mus musculus OX=10090 GN=Kdm2b PE=1 SV=1
Palladin OS=Mus musculus OX=10090 GN=Palld PE=1 SV=2
Septin OS=Mus musculus OX=10090 GN=Septin8 PE=1 SV=1
Unconventional myosin-VIIa OS=Mus musculus OX=10090 GN=Myo7a PE=1 SV=2
Cordon-bleu protein-like 1 OS=Mus musculus OX=10090 GN=Cobll1 PE=1 SV=2
Gamma-adducin OS=Mus musculus OX=10090 GN=Add3 PE=1 SV=2
S-phase kinase-associated protein 1 OS=Mus musculus OX=10090 GN=Skp1 PE=1 SV=3
Histone H4 OS=Mus musculus OX=10090 GN=H4c1 PE=1 SV=2
Pentatricopeptide repeat domain-containing protein 3, mitochondrial OS=Mus musculus OX=10090 GN=Ptcd3 PE
Cytochrome P450 4B1 OS=Mus musculus OX=10090 GN=Cyp4b1 PE=1 SV=1
Echinoderm microtubule-associated protein-like 3 OS=Mus musculus OX=10090 GN=Eml3 PE=1 SV=1
Predicted gene, 45928 OS=Mus musculus OX=10090 GN=Gm45928 PE=4 SV=1
Bisphosphoglycerate mutase OS=Mus musculus OX=10090 GN=Bpgm PE=1 SV=2
Heterogeneous nuclear ribonucleoprotein H2 OS=Mus musculus OX=10090 GN=Hnrnph2 PE=1 SV=1
Chemokine subfamily B Cys-X-Cys OS=Mus musculus OX=10090 GN=Ppbp PE=1 SV=1
PCI domain-containing protein 2 OS=Mus musculus OX=10090 GN=Pcid2 PE=1 SV=1
E3 ubiquitin-protein ligase TRIM41 OS=Mus musculus OX=10090 GN=Trim41 PE=1 SV=1
LIM domain kinase 2 OS=Mus musculus OX=10090 GN=Limk2 PE=1 SV=2
DNA-binding protein Ikaros OS=Mus musculus OX=10090 GN=Ikzf1 PE=1 SV=1
GTP-binding protein 1 OS=Mus musculus OX=10090 GN=Gtpbp1 PE=1 SV=2
Prolow-density lipoprotein receptor-related protein 1 OS=Mus musculus OX=10090 GN=Lrp1 PE=1 SV=1
Beta-arrestin-2 OS=Mus musculus OX=10090 GN=Arrb2 PE=1 SV=1
Inactive ubiquitin carboxyl-terminal hydrolase 54 OS=Mus musculus OX=10090 GN=Usp54 PE=1 SV=2
Eukaryotic translation initiation factor 4B OS=Mus musculus OX=10090 GN=Eif4b PE=1 SV=1
39S ribosomal protein L55, mitochondrial OS=Mus musculus OX=10090 GN=Mrpl55 PE=1 SV=1
RUS family member 1 OS=Mus musculus OX=10090 GN=Rusf1 PE=1 SV=1
Complement C1q tumor necrosis factor-related protein 7 OS=Mus musculus OX=10090 GN=C1qtnf7 PE=2 SV=2
Porphobilinogen deaminase OS=Mus musculus OX=10090 GN=Hmbs PE=1 SV=2
NAD-dependent protein deacetylase sirtuin-7 OS=Mus musculus OX=10090 GN=Sirt7 PE=1 SV=2
Protein S100-A10 OS=Mus musculus OX=10090 GN=S100a10 PE=1 SV=2
Coiled-coil domain-containing protein 86 OS=Mus musculus OX=10090 GN=Ccdc86 PE=1 SV=2
G-protein coupled receptor-associated sorting protein 1 OS=Mus musculus OX=10090 GN=Gprasp1 PE=1 SV=1
Monocyte to macrophage differentiation factor OS=Mus musculus OX=10090 GN=Mmd PE=2 SV=1
EGF-containing fibulin-like extracellular matrix protein 1 OS=Mus musculus OX=10090 GN=Efemp1 PE=1 SV=1
EH domain-containing protein 1 OS=Mus musculus OX=10090 GN=Ehd1 PE=1 SV=1
ADP-ribosylation factor-like protein 6 OS=Mus musculus OX=10090 GN=Arl6 PE=1 SV=1
Calcium/calmodulin-dependent protein kinase OS=Mus musculus OX=10090 GN=Camk2d PE=1 SV=2
Tryptophan--tRNA ligase, cytoplasmic OS=Mus musculus OX=10090 GN=Wars1 PE=1 SV=2
Mth938 domain-containing protein OS=Mus musculus OX=10090 GN=Aamdc PE=1 SV=1

Tropomyosin alpha-3 chain OS=Mus musculus OX=10090 GN=Tpm3 PE=1 SV=1
Neutrophil cytosol factor 1 OS=Mus musculus OX=10090 GN=Ncf1 PE=1 SV=3
Apoptosis-stimulating of p53 protein 1 (Fragment) OS=Mus musculus OX=10090 GN=Ppp1r13b PE=1 SV=1
Enoyl-CoA delta isomerase 1, mitochondrial OS=Mus musculus OX=10090 GN=Eci1 PE=1 SV=1
Translation initiation factor IF-3, mitochondrial OS=Mus musculus OX=10090 GN=Mtif3 PE=1 SV=1
SAFB-like transcription modulator OS=Mus musculus OX=10090 GN=Sltm PE=1 SV=1
F-box/LRR-repeat protein 6 OS=Mus musculus OX=10090 GN=Fbxl6 PE=2 SV=2
Kinesin-like protein KIF14 OS=Mus musculus OX=10090 GN=Kif14 PE=1 SV=1
Rab effector MyRIP (Fragment) OS=Mus musculus OX=10090 GN=Myrip PE=1 SV=1
Lysine-specific histone demethylase 1A (Fragment) OS=Mus musculus OX=10090 GN=Kdm1a PE=1 SV=1
WD repeat-containing protein 90 OS=Mus musculus OX=10090 GN=Wdr90 PE=1 SV=2
Piezo-type mechanosensitive ion channel component OS=Mus musculus OX=10090 GN=Piezo1 PE=1 SV=1
Sp110 nuclear body protein OS=Mus musculus OX=10090 GN=Sp110 PE=1 SV=1
ATP-dependent RNA helicase DDX18 OS=Mus musculus OX=10090 GN=Ddx18 PE=1 SV=1
Cysteine and glycine-rich protein 3 OS=Mus musculus OX=10090 GN=Csrp3 PE=1 SV=1
COP9 signalosome complex subunit 1 OS=Mus musculus OX=10090 GN=Gps1 PE=1 SV=1
Tyrosine-protein kinase OS=Mus musculus OX=10090 GN=Syk PE=1 SV=1
CCR4-NOT transcription complex subunit 1 OS=Mus musculus OX=10090 GN=Cnot1 PE=1 SV=2
RAS guanyl-releasing protein 2 OS=Mus musculus OX=10090 GN=Rasgrp2 PE=1 SV=2
Calcium-binding mitochondrial carrier protein Aralar1 OS=Mus musculus OX=10090 GN=Slc25a12 PE=1 SV=1
Plastin-2 OS=Mus musculus OX=10090 GN=Lcp1 PE=1 SV=4
Proline-rich protein 12 OS=Mus musculus OX=10090 GN=Prr12 PE=1 SV=1
Carboxypeptidase M OS=Mus musculus OX=10090 GN=Cpm PE=1 SV=2
SURP and G-patch domain-containing protein 2 OS=Mus musculus OX=10090 GN=Sugp2 PE=1 SV=2
Sorting nexin-17 OS=Mus musculus OX=10090 GN=Snx17 PE=1 SV=2
Mast cell protease-11 OS=Mus musculus OX=10090 GN=Prss34 PE=1 SV=1
Putative adenosylhomocysteinase 3 OS=Mus musculus OX=10090 GN=Ahcyl2 PE=1 SV=1
Endoplasmic reticulum-Golgi intermediate compartment protein 2 (Fragment) OS=Mus musculus OX=10090 GN[illegible]
Mitogen-activated protein kinase kinase kinase 1 OS=Mus musculus OX=10090 GN=Map3k1 PE=1 SV=3
GATOR complex protein WDR59 OS=Mus musculus OX=10090 GN=Wdr59 PE=1 SV=2
CTP synthase 2 OS=Mus musculus OX=10090 GN=Ctps2 PE=1 SV=1
Upstream-binding protein 1 OS=Mus musculus OX=10090 GN=Ubp1 PE=1 SV=1
Unspecific monooxygenase OS=Mus musculus OX=10090 GN=Cyp2b10 PE=1 SV=1
Interferon-induced protein with tetratricopeptide repeats 1 OS=Mus musculus OX=10090 GN=Ifit1 PE=1 SV=2
Ribosome production factor 1 OS=Mus musculus OX=10090 GN=Rpf1 PE=2 SV=2
Probable hydrolase PNKD OS=Mus musculus OX=10090 GN=Pnkd PE=1 SV=2
Dual specificity mitogen-activated protein kinase kinase 3 OS=Mus musculus OX=10090 GN=Map2k3 PE=1 SV=2
15-hydroxyprostaglandin dehydrogenase [NAD(+)] OS=Mus musculus OX=10090 GN=Hpgd PE=1 SV=1
Mitochondrial amidoxime reducing component 2 OS=Mus musculus OX=10090 GN=Mtarc2 PE=1 SV=1
60S ribosomal protein L37a OS=Mus musculus OX=10090 GN=Rpl37a PE=1 SV=2
Non-specific serine/threonine protein kinase OS=Mus musculus OX=10090 GN=Mast2 PE=1 SV=1
60S ribosomal export protein NMD3 OS=Mus musculus OX=10090 GN=Nmd3 PE=1 SV=1
Fibronectin type-III domain-containing protein 3A (Fragment) OS=Mus musculus OX=10090 GN=Fndc3a PE=1 SV[illegible]
Methionine aminopeptidase 1 OS=Mus musculus OX=10090 GN=Metap1 PE=1 SV=1
Vacuolar protein sorting-associated protein 26A OS=Mus musculus OX=10090 GN=Vps26a PE=1 SV=1

Putative Polycomb group protein ASXL2 (Fragment) OS=Mus musculus OX=10090 GN=Asxl2 PE=1 SV=8
Glycogen synthase kinase-3 alpha OS=Mus musculus OX=10090 GN=Gsk3a PE=1 SV=2
6-phosphofructo-2-kinase OS=Mus musculus OX=10090 GN=Gm29427 PE=1 SV=1
Cactin OS=Mus musculus OX=10090 GN=Cactin PE=1 SV=2
G patch domain-containing protein 4 OS=Mus musculus OX=10090 GN=Gpatch4 PE=1 SV=1
Phosphatidylinositol 5-phosphate 4-kinase type-2 alpha (Fragment) OS=Mus musculus OX=10090 GN=Pip4k2a PE
Sushi repeat-containing protein SRPX2 OS=Mus musculus OX=10090 GN=Srpx2 PE=1 SV=2
WD repeat domain phosphoinositide-interacting protein 2 OS=Mus musculus OX=10090 GN=Wipi2 PE=1 SV=1
Protein kinase C eta type OS=Mus musculus OX=10090 GN=Prkch PE=1 SV=2
Electron transfer flavoprotein subunit beta OS=Mus musculus OX=10090 GN=Etfbl PE=3 SV=1
Transcription factor SOX-7 OS=Mus musculus OX=10090 GN=Sox7 PE=1 SV=2
Zinc finger protein 37 OS=Mus musculus OX=10090 GN=Zfp37 PE=1 SV=1
Glutathione peroxidase 3 OS=Mus musculus OX=10090 GN=Gpx3 PE=1 SV=2
RING-type E3 ubiquitin-protein ligase PPIL2 OS=Mus musculus OX=10090 GN=Ppil2 PE=1 SV=2
Protein flightless-1 homolog OS=Mus musculus OX=10090 GN=Flii PE=1 SV=1
Calcium uptake protein 2, mitochondrial OS=Mus musculus OX=10090 GN=Micu2 PE=1 SV=2
COP9 signalosome complex subunit 7a (Fragment) OS=Mus musculus OX=10090 GN=Cops7a PE=1 SV=1
Transcriptional adapter 3 OS=Mus musculus OX=10090 GN=Tada3 PE=1 SV=1
General vesicular transport factor p115 OS=Mus musculus OX=10090 GN=Uso1 PE=1 SV=2
Transcriptional regulator ATRX OS=Mus musculus OX=10090 GN=Atrx PE=1 SV=3
Echinoderm microtubule-associated protein-like 2 OS=Mus musculus OX=10090 GN=Eml2 PE=1 SV=1
Unconventional myosin-Va OS=Mus musculus OX=10090 GN=Myo5a PE=1 SV=2
39S ribosomal protein L2, mitochondrial OS=Mus musculus OX=10090 GN=Mrpl2 PE=1 SV=1
Interferon-stimulated gene 20 kDa protein OS=Mus musculus OX=10090 GN=Isg20 PE=1 SV=1
RBR-type E3 ubiquitin transferase OS=Mus musculus OX=10090 GN=Arih1 PE=1 SV=1
Long-chain-fatty-acid--CoA ligase 1 OS=Mus musculus OX=10090 GN=Acsl1 PE=1 SV=1
rRNA methyltransferase 3, mitochondrial OS=Mus musculus OX=10090 GN=Mrm3 PE=2 SV=1
Probable dimethyladenosine transferase OS=Mus musculus OX=10090 GN=Dimt1 PE=2 SV=1
Transcriptional repressor CTCF OS=Mus musculus OX=10090 GN=Ctcf PE=1 SV=2
28S rRNA (cytosine-C(5))-methyltransferase OS=Mus musculus OX=10090 GN=Nsun5 PE=1 SV=2
Leukotriene C4 synthase OS=Mus musculus OX=10090 GN=Ltc4s PE=1 SV=1
26S proteasome non-ATPase regulatory subunit 4 OS=Mus musculus OX=10090 GN=Psmd4 PE=1 SV=1
MMS19 nucleotide excision repair protein homolog OS=Mus musculus OX=10090 GN=Mms19 PE=1 SV=1
Paraplegin (Fragment) OS=Mus musculus OX=10090 GN=Spg7 PE=1 SV=1
Lymphocyte antigen 6C1 OS=Mus musculus OX=10090 GN=Ly6c1 PE=1 SV=1
Calponin-3 OS=Mus musculus OX=10090 GN=Cnn3 PE=1 SV=1
Nucleolar protein 56 OS=Mus musculus OX=10090 GN=Nop56 PE=1 SV=2
AHNAK nucleoprotein 2 OS=Mus musculus OX=10090 GN=Ahnak2 PE=4 SV=1
Syntaxin-11 OS=Mus musculus OX=10090 GN=Stx11 PE=1 SV=1
Probable ATP-dependent RNA helicase DDX6 OS=Mus musculus OX=10090 GN=Ddx6 PE=1 SV=1
3-phosphoinositide-dependent protein kinase 1 OS=Mus musculus OX=10090 GN=Pdpk1 PE=1 SV=2
Aldo-keto reductase family 1 member A1 OS=Mus musculus OX=10090 GN=Akr1a1 PE=1 SV=3
Aspartyl aminopeptidase (Fragment) OS=Mus musculus OX=10090 GN=Dnpep PE=1 SV=1
Gremlin-2 OS=Mus musculus OX=10090 GN=Grem2 PE=1 SV=1
Methionine aminopeptidase 2 OS=Mus musculus OX=10090 GN=Metap2 PE=1 SV=1

UDP-N-acetylglucosamine--peptide N-acetylglucosaminyltransferase 110 kDa subunit OS=Mus musculus OX=100
Negative elongation factor A OS=Mus musculus OX=10090 GN=Nelfa PE=1 SV=1
Patatin-like phospholipase domain-containing protein 6 OS=Mus musculus OX=10090 GN=Pnpla6 PE=1 SV=2
Transcriptional repressor p66 alpha OS=Mus musculus OX=10090 GN=Gatad2a PE=1 SV=1
Arf-GAP with SH3 domain, ANK repeat and PH domain-containing protein 1 OS=Mus musculus OX=10090 GN=As
Zinc finger CCCH domain-containing protein 14 OS=Mus musculus OX=10090 GN=Zc3h14 PE=1 SV=1
Zinc finger protein 281 OS=Mus musculus OX=10090 GN=Znf281 PE=1 SV=1
PHD finger protein 20-like protein 1 OS=Mus musculus OX=10090 GN=Phf20l1 PE=1 SV=1
RNA polymerase II-associated protein 3 OS=Mus musculus OX=10090 GN=Rpap3 PE=1 SV=1
PWWP domain-containing DNA repair factor 3A OS=Mus musculus OX=10090 GN=Pwwp3a PE=1 SV=1
Transportin-3 OS=Mus musculus OX=10090 GN=Tnpo3 PE=1 SV=1
Serine/threonine-protein kinase PRP4 homolog OS=Mus musculus OX=10090 GN=Prpf4b PE=1 SV=3
Sphingomyelin phosphodiesterase 2 OS=Mus musculus OX=10090 GN=Smpd2 PE=1 SV=1
COP9 signalosome complex subunit 2 OS=Mus musculus OX=10090 GN=Cops2 PE=1 SV=1
Lysine-specific demethylase 7A OS=Mus musculus OX=10090 GN=Kdm7a PE=2 SV=2
Septin-5 OS=Mus musculus OX=10090 GN=Septin5 PE=1 SV=2
Protein HEXIM1 OS=Mus musculus OX=10090 GN=Hexim1 PE=1 SV=1
Proteasome subunit alpha type-6 OS=Mus musculus OX=10090 GN=Psma6 PE=1 SV=1
Zinc finger protein 579 OS=Mus musculus OX=10090 GN=Znf579 PE=1 SV=1
Zinc fingers and homeoboxes protein 2 OS=Mus musculus OX=10090 GN=Zhx2 PE=1 SV=1
Nuclear envelope pore membrane protein POM 121 OS=Mus musculus OX=10090 GN=Pom121 PE=1 SV=2
Hypoxanthine-guanine phosphoribosyltransferase OS=Mus musculus OX=10090 GN=Hprt1 PE=1 SV=3
Cytochrome b-c1 complex subunit 8 OS=Mus musculus OX=10090 GN=Uqcrq PE=1 SV=3
Citrate synthase OS=Mus musculus OX=10090 GN=Csl PE=1 SV=1
rRNA-processing protein UTP23 homolog OS=Mus musculus OX=10090 GN=Utp23 PE=2 SV=1
Stonin-1 OS=Mus musculus OX=10090 GN=Ston1 PE=1 SV=2
Zinc finger protein 592 OS=Mus musculus OX=10090 GN=Znf592 PE=1 SV=3
Calcium load-activated calcium channel (Fragment) OS=Mus musculus OX=10090 GN=Tmco1 PE=1 SV=1
Cytochrome c oxidase subunit 7C, mitochondrial OS=Mus musculus OX=10090 GN=Cox7c PE=1 SV=1
Heterogeneous nuclear ribonucleoproteins C1/C2 OS=Mus musculus OX=10090 GN=Hnrnpc PE=1 SV=1
Syntaxin 5A OS=Mus musculus OX=10090 GN=Stx5a PE=1 SV=1
Dehydrogenase/reductase SDR family member 7 (Fragment) OS=Mus musculus OX=10090 GN=Dhrs7 PE=1 SV=1
WD repeat domain phosphoinositide-interacting protein 3 OS=Mus musculus OX=10090 GN=Wdr45b PE=1 SV=2
Sideroflexin-3 OS=Mus musculus OX=10090 GN=Sfxn3 PE=1 SV=1
Acid ceramidase OS=Mus musculus OX=10090 GN=Asah1 PE=1 SV=1
28 kDa heat- and acid-stable phosphoprotein OS=Mus musculus OX=10090 GN=Pdap1 PE=1 SV=1
Synaptic vesicle membrane protein VAT-1 homolog OS=Mus musculus OX=10090 GN=Vat1 PE=1 SV=3
NEDD8-conjugating enzyme Ubc12 (Fragment) OS=Mus musculus OX=10090 GN=Ube2m PE=1 SV=1
Serine/threonine-protein kinase OSR1 OS=Mus musculus OX=10090 GN=Oxsr1 PE=1 SV=1
Non-specific serine/threonine protein kinase OS=Mus musculus OX=10090 GN=Rps6ka3 PE=1 SV=1
Transcription factor COE1 OS=Mus musculus OX=10090 GN=Ebf1 PE=1 SV=1
Importin subunit alpha-1 OS=Mus musculus OX=10090 GN=Kpna2 PE=1 SV=2
Serine/threonine-protein phosphatase 2A activator OS=Mus musculus OX=10090 GN=Ptpa PE=1 SV=1
DmX-like protein 1 OS=Mus musculus OX=10090 GN=Dmxl1 PE=1 SV=1
Death-associated protein kinase 1 OS=Mus musculus OX=10090 GN=Dapk1 PE=1 SV=3

CLIP-associating protein 2 OS=Mus musculus OX=10090 GN=Clasp2 PE=1 SV=1
Spliceosome-associated protein CWC27 homolog OS=Mus musculus OX=10090 GN=Cwc27 PE=1 SV=1
Growth hormone-regulated TBC protein 1 OS=Mus musculus OX=10090 GN=Grtp1 PE=1 SV=1
Protein MTSS 1 OS=Mus musculus OX=10090 GN=Mtss1 PE=1 SV=1
StAR-related lipid transfer protein 9 OS=Mus musculus OX=10090 GN=Stard9 PE=1 SV=2
Protein quaking OS=Mus musculus OX=10090 GN=Qki PE=1 SV=1
DENN domain-containing protein 5A OS=Mus musculus OX=10090 GN=Dennd5a PE=1 SV=1
Talin-1 OS=Mus musculus OX=10090 GN=Tln1 PE=1 SV=2
tRNA pseudouridine synthase-like 1 OS=Mus musculus OX=10090 GN=Pusl1 PE=1 SV=1
Transient receptor potential cation channel, subfamily M, member 3 OS=Mus musculus OX=10090 GN=Trpm3 PE
Protein SET (Fragment) OS=Mus musculus OX=10090 GN=Set PE=1 SV=1
DnaJ homolog subfamily B member 5 OS=Mus musculus OX=10090 GN=Dnajb5 PE=1 SV=1
Dr1-associated corepressor OS=Mus musculus OX=10090 GN=Drap1 PE=1 SV=1
NMDA receptor synaptonuclear-signaling and neuronal migration factor OS=Mus musculus OX=10090 GN=Nsmf
Telomerase protein component 1 OS=Mus musculus OX=10090 GN=Tep1 PE=1 SV=1
G patch domain-containing protein 1 OS=Mus musculus OX=10090 GN=Gpatch1 PE=2 SV=1
Semaphorin-3E OS=Mus musculus OX=10090 GN=Sema3e PE=1 SV=3
TRAF3-interacting JNK-activating modulator OS=Mus musculus OX=10090 GN=Traf3ip3 PE=1 SV=2
Macrophage-capping protein (Fragment) OS=Mus musculus OX=10090 GN=Capg PE=1 SV=1
UBX domain-containing protein 1 OS=Mus musculus OX=10090 GN=Ubxn1 PE=1 SV=1
Arf-GAP domain and FG repeat-containing protein 1 OS=Mus musculus OX=10090 GN=Agfg1 PE=1 SV=1
Signal-induced proliferation-associated 1-like protein 2 OS=Mus musculus OX=10090 GN=Sipa1l2 PE=1 SV=3
NADH dehydrogenase [ubiquinone] iron-sulfur protein 3, mitochondrial OS=Mus musculus OX=10090 GN=Ndufs3
Myristoylated alanine-rich C-kinase substrate OS=Mus musculus OX=10090 GN=Marcks PE=1 SV=2
Outer mitochondrial membrane protein porin 2 OS=Mus musculus OX=10090 GN=Vdac2 PE=1 SV=1
Transcriptional activator protein Pur-alpha OS=Mus musculus OX=10090 GN=Pura PE=1 SV=1
Xaa-Pro aminopeptidase 2 OS=Mus musculus OX=10090 GN=Xpnpep2 PE=1 SV=1
Pleckstrin homology domain-containing family F member 2 OS=Mus musculus OX=10090 GN=Plekhf2 PE=1 SV=1
Membrane-associated phosphatidylinositol transfer protein 3 OS=Mus musculus OX=10090 GN=Pitpnm3 PE=1 S\
WD repeat domain-containing protein 83 (Fragment) OS=Mus musculus OX=10090 GN=Wdr83 PE=1 SV=1
Glutamine synthetase OS=Mus musculus OX=10090 GN=Glul PE=1 SV=6
Dedicator of cytokinesis protein 10 (Fragment) OS=Mus musculus OX=10090 GN=Dock10 PE=1 SV=1
Probable ATP-dependent RNA helicase DDX47 OS=Mus musculus OX=10090 GN=Ddx47 PE=2 SV=2
Structural maintenance of chromosomes protein 5 OS=Mus musculus OX=10090 GN=Smc5 PE=1 SV=1
YTH domain-containing family protein 2 OS=Mus musculus OX=10090 GN=Ythdf2 PE=1 SV=1
Ribosome biogenesis protein BOP1 OS=Mus musculus OX=10090 GN=Bop1 PE=1 SV=1
1-phosphatidylinositol 4,5-bisphosphate phosphodiesterase delta-3 OS=Mus musculus OX=10090 GN=Plcd3 PE=1
Serine/threonine-protein phosphatase 2A catalytic subunit beta isoform OS=Mus musculus OX=10090 GN=Ppp2c
High mobility group protein HMGI-C OS=Mus musculus OX=10090 GN=Hmga2 PE=1 SV=1
Phospholipid-transporting ATPase ABCA1 OS=Mus musculus OX=10090 GN=Abca1 PE=1 SV=4
DNA-3-methyladenine glycosylase OS=Mus musculus OX=10090 GN=Mpg PE=2 SV=3
E3 ubiquitin-protein ligase RING1 OS=Mus musculus OX=10090 GN=Ring1 PE=1 SV=2
E1A-binding protein p400 OS=Mus musculus OX=10090 GN=Ep400 PE=1 SV=3
Receptor of activated protein C kinase 1 OS=Mus musculus OX=10090 GN=Rack1 PE=1 SV=3
Trypsin 10 OS=Mus musculus OX=10090 GN=Try10 PE=1 SV=1

Dynein light chain Tctex-type 3 OS=Mus musculus OX=10090 GN=Dynlt3 PE=1 SV=1
39S ribosomal protein L38, mitochondrial OS=Mus musculus OX=10090 GN=Mrpl38 PE=1 SV=2
Nuclear cap-binding protein subunit 3 OS=Mus musculus OX=10090 GN=Ncbp3 PE=1 SV=1
Desmoplakin OS=Mus musculus OX=10090 GN=Dsp PE=1 SV=1
Serine/threonine-protein phosphatase 6 regulatory ankyrin repeat subunit B (Fragment) OS=Mus musculus OX=1
60S acidic ribosomal protein P2 OS=Mus musculus OX=10090 GN=Rplp2 PE=1 SV=3
Protein TASOR OS=Mus musculus OX=10090 GN=Tasor PE=1 SV=2
Dual specificity protein phosphatase 23 OS=Mus musculus OX=10090 GN=Dusp23 PE=1 SV=1
Arf-GAP with GTPase, ANK repeat and PH domain-containing protein 1 OS=Mus musculus OX=10090 GN=Agap1
GTP:AMP phosphotransferase AK3, mitochondrial OS=Mus musculus OX=10090 GN=Ak3 PE=1 SV=3
Caskin-2 OS=Mus musculus OX=10090 GN=Caskin2 PE=1 SV=3
Stomatin-like protein 2, mitochondrial OS=Mus musculus OX=10090 GN=Stoml2 PE=1 SV=1
Reproductive homeobox 12 OS=Mus musculus OX=10090 GN=Rhox12 PE=4 SV=1
von Willebrand factor A domain-containing protein 8 OS=Mus musculus OX=10090 GN=Vwa8 PE=1 SV=2
Zinc finger CCHC domain-containing protein 24 OS=Mus musculus OX=10090 GN=Zcchc24 PE=1 SV=1
Transcription factor 7-like 2 OS=Mus musculus OX=10090 GN=Tcf7l2 PE=1 SV=1
2',5'-phosphodiesterase 12 OS=Mus musculus OX=10090 GN=Pde12 PE=1 SV=2
Calcium/calmodulin-dependent protein kinase type 1 OS=Mus musculus OX=10090 GN=Camk1 PE=1 SV=1
Serine/threonine-protein kinase RIO2 OS=Mus musculus OX=10090 GN=Riok2 PE=1 SV=1
Flavin-containing monooxygenase 5 OS=Mus musculus OX=10090 GN=Fmo5 PE=1 SV=4
Rab11 family-interacting protein 5 OS=Mus musculus OX=10090 GN=Rab11fip5 PE=1 SV=1
Nephrocystin-1 OS=Mus musculus OX=10090 GN=Nphp1 PE=1 SV=1
40S ribosomal protein SA OS=Mus musculus OX=10090 GN=Rpsa PE=1 SV=4
rRNA N6-adenosine-methyltransferase ZCCHC4 OS=Mus musculus OX=10090 GN=Zcchc4 PE=2 SV=1
Zinc finger E-box-binding homeobox 2 OS=Mus musculus OX=10090 GN=Zeb2 PE=1 SV=1
NFX1-type zinc finger-containing protein 1 OS=Mus musculus OX=10090 GN=Znfx1 PE=1 SV=3
Ribosome biogenesis protein NSA2 homolog OS=Mus musculus OX=10090 GN=Nsa2 PE=2 SV=1
Pre-mRNA-splicing factor SPF27 OS=Mus musculus OX=10090 GN=Bcas2 PE=1 SV=1
Serine/threonine-protein kinase A-Raf OS=Mus musculus OX=10090 GN=Araf PE=1 SV=2
Platelet-derived growth factor D OS=Mus musculus OX=10090 GN=Pdgfd PE=2 SV=1
Sodium/potassium-transporting ATPase subunit alpha-1 OS=Mus musculus OX=10090 GN=Atp1a1 PE=1 SV=1
Protein FAM207A OS=Mus musculus OX=10090 GN=Fam207a PE=1 SV=1
ATPase MORC2A OS=Mus musculus OX=10090 GN=Morc2a PE=1 SV=2
SH3 domain and tetratricopeptide repeat-containing protein 2 OS=Mus musculus OX=10090 GN=Sh3tc2 PE=1 SV
Protein BUD31 homolog OS=Mus musculus OX=10090 GN=Bud31 PE=1 SV=2
1-acyl-sn-glycerol-3-phosphate acyltransferase delta OS=Mus musculus OX=10090 GN=Agpat4 PE=1 SV=1
Zinc finger protein 800 OS=Mus musculus OX=10090 GN=Znf800 PE=1 SV=1
Dynactin subunit 5 OS=Mus musculus OX=10090 GN=Dctn5 PE=1 SV=1
Glucocorticoid receptor OS=Mus musculus OX=10090 GN=Nr3c1 PE=1 SV=1
Serine/threonine-protein kinase 4 OS=Mus musculus OX=10090 GN=Stk4 PE=1 SV=1
Myosin regulatory light polypeptide 9 OS=Mus musculus OX=10090 GN=Myl9 PE=1 SV=3
Calcium-binding protein 39 OS=Mus musculus OX=10090 GN=Cab39 PE=1 SV=2
Amyloid-beta A4 precursor protein-binding family B member 2 OS=Mus musculus OX=10090 GN=Apbb2 PE=1 SV
60S ribosomal protein L31 OS=Mus musculus OX=10090 GN=Rpl31 PE=1 SV=1
Zinc finger protein 57 OS=Mus musculus OX=10090 GN=Zfp57 PE=1 SV=1

Nuclear transcription factor Y subunit OS=Mus musculus OX=10090 GN=Nfya PE=1 SV=1
Gamma-tubulin complex component 5 OS=Mus musculus OX=10090 GN=Tubgcp5 PE=2 SV=2
Junctional protein associated with coronary artery disease OS=Mus musculus OX=10090 GN=Jcad PE=1 SV=2
Melanoma-associated antigen H1 OS=Mus musculus OX=10090 GN=Mageh1 PE=2 SV=1
Rab GDP dissociation inhibitor beta OS=Mus musculus OX=10090 GN=Gdi2 PE=1 SV=1
Inactive serine/threonine-protein kinase VRK3 (Fragment) OS=Mus musculus OX=10090 GN=Vrk3 PE=1 SV=9
Nucleoside-diphosphate kinase (Fragment) OS=Mus musculus OX=10090 GN=Ak1 PE=1 SV=1
Zinc finger and BTB domain-containing protein 4 OS=Mus musculus OX=10090 GN=Zbtb4 PE=2 SV=1
Dihydroxyacetone phosphate acyltransferase OS=Mus musculus OX=10090 GN=Gnpat PE=1 SV=1
Putative methyltransferase C9orf114 homolog OS=Mus musculus OX=10090 GN=Spout1 PE=1 SV=1
Enoyl-CoA delta isomerase 2 OS=Mus musculus OX=10090 GN=Eci2 PE=1 SV=2
Hermansky-Pudlak syndrome 5 protein homolog (Fragment) OS=Mus musculus OX=10090 GN=Hps5 PE=1 SV=1
Microtubule-associated protein RP/EB family member 3 OS=Mus musculus OX=10090 GN=Mapre3 PE=1 SV=1
Vacuolar protein sorting-associated protein 13C OS=Mus musculus OX=10090 GN=Vps13c PE=1 SV=2
HCLS1-binding protein 3 (Fragment) OS=Mus musculus OX=10090 GN=Hs1bp3 PE=1 SV=1
Bromodomain adjacent to zinc finger domain protein 1A OS=Mus musculus OX=10090 GN=Baz1a PE=1 SV=1
Elongation factor-like GTPase 1 OS=Mus musculus OX=10090 GN=Efl1 PE=1 SV=1
Methionine--tRNA ligase, cytoplasmic OS=Mus musculus OX=10090 GN=Mars1 PE=1 SV=1
Annexin A5 OS=Mus musculus OX=10090 GN=Anxa5 PE=1 SV=1
Arf-GAP with dual PH domain-containing protein 2 OS=Mus musculus OX=10090 GN=Adap2 PE=1 SV=1
TBC1 domain family member 9B OS=Mus musculus OX=10090 GN=Tbc1d9b PE=1 SV=1
Ras-associating and dilute domain-containing protein OS=Mus musculus OX=10090 GN=Radil PE=1 SV=2
Charged multivesicular body protein 1a OS=Mus musculus OX=10090 GN=Chmp1a PE=1 SV=1
Casein kinase I isoform gamma-2 OS=Mus musculus OX=10090 GN=Csnk1g2 PE=1 SV=1
von Willebrand factor OS=Mus musculus OX=10090 GN=Vwf PE=1 SV=2
Adenylosuccinate synthetase isozyme 2 OS=Mus musculus OX=10090 GN=Adss2 PE=1 SV=2
Enhancer of mRNA-decapping protein 3 OS=Mus musculus OX=10090 GN=Edc3 PE=1 SV=1
Disintegrin and metalloproteinase domain-containing protein 17 OS=Mus musculus OX=10090 GN=Adam17 PE=[illegible]
Importin subunit alpha-3 OS=Mus musculus OX=10090 GN=Kpna4 PE=1 SV=1
DNA topoisomerase 1 OS=Mus musculus OX=10090 GN=Top1 PE=1 SV=2
Zinc finger protein 212 OS=Mus musculus OX=10090 GN=Zfp212 PE=1 SV=1
40S ribosomal protein S12 OS=Mus musculus OX=10090 GN=Rps12 PE=1 SV=1
Histone acetyltransferase OS=Mus musculus OX=10090 GN=Kat6a PE=1 SV=1
Zinc finger protein 687 OS=Mus musculus OX=10090 GN=Znf687 PE=1 SV=1
Exocyst complex component 7 OS=Mus musculus OX=10090 GN=Exoc7 PE=1 SV=2
Zinc finger protein 871 OS=Mus musculus OX=10090 GN=Zfp871 PE=1 SV=1
Pulmonary surfactant-associated protein C OS=Mus musculus OX=10090 GN=Sftpc PE=1 SV=1
Serine/threonine-protein kinase Nek6 OS=Mus musculus OX=10090 GN=Nek6 PE=1 SV=1
ATP-binding cassette sub-family F member 2 OS=Mus musculus OX=10090 GN=Abcf2 PE=1 SV=1
Vacuolar protein-sorting-associated protein 25 OS=Mus musculus OX=10090 GN=Vps25 PE=1 SV=1
Splicing factor 3A subunit 2 OS=Mus musculus OX=10090 GN=Sf3a2 PE=1 SV=1
V-set and transmembrane domain-containing protein 4 OS=Mus musculus OX=10090 GN=Vstm4 PE=1 SV=1
Chloride intracellular channel protein 4 OS=Mus musculus OX=10090 GN=Clic4 PE=1 SV=3
TBC1 domain family member 2B OS=Mus musculus OX=10090 GN=Tbc1d2b PE=1 SV=2
Band 4.1 (Fragment) OS=Mus musculus OX=10090 GN=Epb41 PE=1 SV=1

Neural Wiskott-Aldrich syndrome protein OS=Mus musculus OX=10090 GN=Wasl PE=1 SV=1
Actin-depolymerizing factor (Fragment) OS=Mus musculus OX=10090 GN=Gsn PE=1 SV=1
BMS1 homolog, ribosome assembly protein (Yeast) OS=Mus musculus OX=10090 GN=Bms1 PE=1 SV=1
Protein mono-ADP-ribosyltransferase PARP4 OS=Mus musculus OX=10090 GN=Parp4 PE=1 SV=1
Serine/threonine-protein kinase N1 OS=Mus musculus OX=10090 GN=Pkn1 PE=1 SV=3
Non-specific serine/threonine protein kinase OS=Mus musculus OX=10090 GN=Stk3 PE=1 SV=1
Microfibril-associated glycoprotein 4 OS=Mus musculus OX=10090 GN=Mfap4 PE=1 SV=1
Tropomyosin alpha-4 chain OS=Mus musculus OX=10090 GN=Tpm4 PE=1 SV=3
Serine/threonine-protein kinase B-raf OS=Mus musculus OX=10090 GN=Braf PE=1 SV=4
Transmembrane glycoprotein NMB OS=Mus musculus OX=10090 GN=Gpnmb PE=1 SV=2
Septin OS=Mus musculus OX=10090 GN=Septin7 PE=1 SV=2
60S ribosomal protein L3 OS=Mus musculus OX=10090 GN=Rpl3 PE=1 SV=3
Ras-related protein Rab-2A OS=Mus musculus OX=10090 GN=Rab2a PE=1 SV=1
Protein disulfide-isomerase TMX3 OS=Mus musculus OX=10090 GN=Tmx3 PE=1 SV=2
Antileukoproteinase OS=Mus musculus OX=10090 GN=Slpi PE=1 SV=1
Dual specificity mitogen-activated protein kinase kinase 7 OS=Mus musculus OX=10090 GN=Map2k7 PE=1 SV=1
General receptor for phosphoinositides 1-associated scaffold protein OS=Mus musculus OX=10090 GN=Tamalin [illegible]
Putative RNA-binding protein Luc7-like 2 OS=Mus musculus OX=10090 GN=Luc7l2 PE=1 SV=1
NAD(P)H pyrophosphatase NUDT13, mitochondrial (Fragment) OS=Mus musculus OX=10090 GN=Nudt13 PE=1 S[illegible]
HEAT repeat-containing protein 5A OS=Mus musculus OX=10090 GN=Heatr5a PE=1 SV=2
Unconventional myosin-Ig OS=Mus musculus OX=10090 GN=Myo1g PE=1 SV=1
Tubulin beta-5 chain OS=Mus musculus OX=10090 GN=Tubb5 PE=1 SV=1
DNA-directed RNA polymerase II subunit RPB7 OS=Mus musculus OX=10090 GN=Polr2g PE=1 SV=1
Nephrocystin-4 OS=Mus musculus OX=10090 GN=Nphp4 PE=1 SV=2
Calnexin OS=Mus musculus OX=10090 GN=Canx PE=1 SV=1
Replication factor C subunit 4 OS=Mus musculus OX=10090 GN=Rfc4 PE=1 SV=1
Tropomodulin-2 OS=Mus musculus OX=10090 GN=Tmod2 PE=1 SV=2
E3 ubiquitin-protein ligase SMURF2 OS=Mus musculus OX=10090 GN=Smurf2 PE=1 SV=1
SWI/SNF complex subunit SMARCC1 OS=Mus musculus OX=10090 GN=Smarcc1 PE=1 SV=2
m-AAA protease-interacting protein 1, mitochondrial OS=Mus musculus OX=10090 GN=Maip1 PE=1 SV=1
Endoplasmic reticulum chaperone BiP OS=Mus musculus OX=10090 GN=Hspa5 PE=1 SV=3
Twinfilin-2 OS=Mus musculus OX=10090 GN=Twf2 PE=1 SV=1
Putative methyltransferase NSUN7 OS=Mus musculus OX=10090 GN=Nsun7 PE=3 SV=2
Period circadian protein homolog 1 OS=Mus musculus OX=10090 GN=Per1 PE=1 SV=2
Bifunctional glutamate/proline--tRNA ligase OS=Mus musculus OX=10090 GN=Eprs1 PE=1 SV=4
Tapasin OS=Mus musculus OX=10090 GN=Tapbp PE=1 SV=2
Coagulation factor XIII A chain OS=Mus musculus OX=10090 GN=F13a1 PE=1 SV=3
NLR family member X1 OS=Mus musculus OX=10090 GN=Nlrx1 PE=1 SV=1
60S ribosomal protein L35 OS=Mus musculus OX=10090 GN=Rpl35 PE=1 SV=1
Ras-related protein Rab-10 OS=Mus musculus OX=10090 GN=Rab10 PE=1 SV=1
Peptide-methionine (R)-S-oxide reductase OS=Mus musculus OX=10090 GN=Msrb3 PE=1 SV=1
Nidogen-2 (Fragment) OS=Mus musculus OX=10090 GN=Nid2 PE=1 SV=1
Centrosomal protein of 55 kDa OS=Mus musculus OX=10090 GN=Cep55 PE=1 SV=2
Inverted formin-2 OS=Mus musculus OX=10090 GN=Inf2 PE=1 SV=1
Early endosome antigen 1 OS=Mus musculus OX=10090 GN=Eea1 PE=1 SV=2

Regulator of nonsense transcripts 2 OS=Mus musculus OX=10090 GN=Upf2 PE=1 SV=1
Zinc finger protein 394 OS=Mus musculus OX=10090 GN=Znf394 PE=2 SV=3
Protein PALS1 OS=Mus musculus OX=10090 GN=Pals1 PE=1 SV=1
40S ribosomal protein S24 OS=Mus musculus OX=10090 GN=Rps24 PE=1 SV=1
Succinate dehydrogenase [ubiquinone] iron-sulfur subunit, mitochondrial OS=Mus musculus OX=10090 GN=Sdhb
Zinc finger, C3H1-type-containing OS=Mus musculus OX=10090 GN=Zfc3h1 PE=1 SV=1
Regulator of G-protein-signaling 12 OS=Mus musculus OX=10090 GN=Rgs12 PE=1 SV=1
E3 ubiquitin-protein ligase HECW2 OS=Mus musculus OX=10090 GN=Hecw2 PE=1 SV=1
Rho guanine nucleotide exchange factor 3 OS=Mus musculus OX=10090 GN=Arhgef3 PE=1 SV=1
Small subunit processome component 20 homolog OS=Mus musculus OX=10090 GN=Utp20 PE=1 SV=2
DNA-directed RNA polymerase, mitochondrial OS=Mus musculus OX=10090 GN=Polrmt PE=1 SV=1
Protein KIAA0100 OS=Mus musculus OX=10090 GN=Kiaa0100 PE=1 SV=1
Pre-mRNA-splicing factor SYF2 OS=Mus musculus OX=10090 GN=Syf2 PE=2 SV=1
1-phosphatidylinositol 4,5-bisphosphate phosphodiesterase gamma-2 OS=Mus musculus OX=10090 GN=Plcg2 PE
Phosphorylase b kinase regulatory subunit (Fragment) OS=Mus musculus OX=10090 GN=Phkb PE=1 SV=1
Leukocyte receptor cluster member 9 OS=Mus musculus OX=10090 GN=Leng9 PE=2 SV=1
40S ribosomal protein S16 OS=Mus musculus OX=10090 GN=Rps16 PE=1 SV=4
Alpha-actinin-2 OS=Mus musculus OX=10090 GN=Actn2 PE=1 SV=2
InaD-like protein OS=Mus musculus OX=10090 GN=Patj PE=1 SV=1
Diacylglycerol kinase delta OS=Mus musculus OX=10090 GN=Dgkd PE=1 SV=1
Annexin A7 OS=Mus musculus OX=10090 GN=Anxa7 PE=1 SV=2
Succinate--CoA ligase [GDP-forming] subunit beta, mitochondrial OS=Mus musculus OX=10090 GN=Suclg2 PE=1
40S ribosomal protein S15a OS=Mus musculus OX=10090 GN=Rps15a PE=1 SV=2
Exocyst complex component 3 OS=Mus musculus OX=10090 GN=Exoc3 PE=1 SV=2
Serine/arginine-rich splicing factor 7 OS=Mus musculus OX=10090 GN=Srsf7 PE=1 SV=1
U2 small nuclear ribonucleoprotein auxiliary factor 35 kDa subunit-related protein 1 OS=Mus musculus OX=1009(
Dipeptidyl peptidase 1 OS=Mus musculus OX=10090 GN=Ctsc PE=1 SV=1
A-kinase anchor protein 8 OS=Mus musculus OX=10090 GN=Akap8 PE=1 SV=1
Formin-like protein 2 OS=Mus musculus OX=10090 GN=Fmnl2 PE=1 SV=1
mRNA cap guanine-N7 methyltransferase OS=Mus musculus OX=10090 GN=Rnmt PE=1 SV=1
ATP-dependent RNA helicase DDX55 OS=Mus musculus OX=10090 GN=Ddx55 PE=1 SV=2
Mediator of RNA polymerase II transcription subunit 29 OS=Mus musculus OX=10090 GN=Med29 PE=2 SV=1
4-trimethylaminobutyraldehyde dehydrogenase OS=Mus musculus OX=10090 GN=Aldh9a1 PE=1 SV=1
WD repeat-containing protein 11 OS=Mus musculus OX=10090 GN=Wdr11 PE=1 SV=1
Complement factor H-related 4 OS=Mus musculus OX=10090 GN=Cfhr4 PE=1 SV=1
RNA-binding protein 26 OS=Mus musculus OX=10090 GN=Rbm26 PE=1 SV=1
Phosphatidylinositol 4,5-bisphosphate 3-kinase catalytic subunit beta isoform OS=Mus musculus OX=10090 GN=I
Probable proline--tRNA ligase, mitochondrial OS=Mus musculus OX=10090 GN=Pars2 PE=1 SV=2
Protein S100-A11 OS=Mus musculus OX=10090 GN=S100a11 PE=1 SV=1
Activator of basal transcription 1 OS=Mus musculus OX=10090 GN=Abt1 PE=2 SV=1
Segment polarity protein dishevelled homolog DVL-3 OS=Mus musculus OX=10090 GN=Dvl3 PE=1 SV=1
Krueppel-like factor 12 OS=Mus musculus OX=10090 GN=Klf12 PE=1 SV=2
Eukaryotic translation initiation factor 2 subunit 3, X-linked OS=Mus musculus OX=10090 GN=Eif2s3x PE=1 SV=2
Pyrroline-5-carboxylate reductase 2 OS=Mus musculus OX=10090 GN=Pycr2 PE=1 SV=1
Isoleucine--tRNA ligase, cytoplasmic OS=Mus musculus OX=10090 GN=Iars1 PE=1 SV=2

Disheveled-associated activator of morphogenesis 1 OS=Mus musculus OX=10090 GN=Daam1 PE=1 SV=4
Transcription elongation factor SPT5 OS=Mus musculus OX=10090 GN=Supt5h PE=1 SV=1
Telomerase-binding protein EST1A OS=Mus musculus OX=10090 GN=Smg6 PE=1 SV=1
Homeobox protein Hox-A4 OS=Mus musculus OX=10090 GN=Hoxa4 PE=2 SV=4
Protein TANC1 OS=Mus musculus OX=10090 GN=Tanc1 PE=1 SV=1
Aldehyde dehydrogenase, cytosolic 1 OS=Mus musculus OX=10090 GN=Aldh1a7 PE=1 SV=1
Isocitrate dehydrogenase [NADP] cytoplasmic OS=Mus musculus OX=10090 GN=Idh1 PE=1 SV=2
5'-AMP-activated protein kinase subunit gamma-1 OS=Mus musculus OX=10090 GN=Prkag1 PE=1 SV=2
Intercellular adhesion molecule 2 OS=Mus musculus OX=10090 GN=Icam2 PE=1 SV=1
Up-regulator of cell proliferation OS=Mus musculus OX=10090 GN=Urgcp PE=2 SV=1
S-adenosylmethionine synthase OS=Mus musculus OX=10090 GN=Mat2a PE=1 SV=1
Annexin (Fragment) OS=Mus musculus OX=10090 GN=Anxa4 PE=1 SV=1
RNA helicase OS=Mus musculus OX=10090 GN=Dhx38 PE=1 SV=1
NADH dehydrogenase [ubiquinone] 1 alpha subcomplex subunit 10, mitochondrial OS=Mus musculus OX=10090 (
Serine/threonine-protein phosphatase PGAM5, mitochondrial OS=Mus musculus OX=10090 GN=Pgam5 PE=1 SV=
Acidic leucine-rich nuclear phosphoprotein 32 family member A (Fragment) OS=Mus musculus OX=10090 GN=Ar
Dihydrolipoyl dehydrogenase, mitochondrial OS=Mus musculus OX=10090 GN=Dld PE=1 SV=2
Nucleolar protein 9 OS=Mus musculus OX=10090 GN=Nop9 PE=1 SV=1
Plakophilin-4 OS=Mus musculus OX=10090 GN=Pkp4 PE=1 SV=1
Heat shock 70 kDa protein 4L OS=Mus musculus OX=10090 GN=Hspa4l PE=1 SV=2
Probable ATP-dependent RNA helicase DDX52 OS=Mus musculus OX=10090 GN=Ddx52 PE=2 SV=2
Target of EGR1 protein 1 OS=Mus musculus OX=10090 GN=Toe1 PE=1 SV=1
AP-2 complex subunit sigma OS=Mus musculus OX=10090 GN=Ap2s1 PE=1 SV=1
Tetratricopeptide repeat protein 7B OS=Mus musculus OX=10090 GN=Ttc7b PE=1 SV=1
Nuclear receptor subfamily 2 group C member 2 OS=Mus musculus OX=10090 GN=Nr2c2 PE=1 SV=1
Thyroid receptor-interacting protein 6 OS=Mus musculus OX=10090 GN=Trip6 PE=1 SV=1
Actin-binding LIM protein 3 OS=Mus musculus OX=10090 GN=Ablim3 PE=1 SV=2
Na(+)/H(+) exchange regulatory cofactor NHE-RF1 OS=Mus musculus OX=10090 GN=Slc9a3r1 PE=1 SV=3
LIM and cysteine-rich domains protein 1 OS=Mus musculus OX=10090 GN=Lmcd1 PE=1 SV=1
Ubiquitinyl hydrolase 1 OS=Mus musculus OX=10090 GN=Usp24 PE=1 SV=1
Protein fuzzy homolog OS=Mus musculus OX=10090 GN=Fuz PE=3 SV=1
Alpha-adducin OS=Mus musculus OX=10090 GN=Add1 PE=1 SV=1
Small nuclear ribonucleoprotein F OS=Mus musculus OX=10090 GN=Snrpf PE=1 SV=1
Tubulin-folding cofactor B OS=Mus musculus OX=10090 GN=Tbcb PE=1 SV=2
Metalloproteinase inhibitor 3 OS=Mus musculus OX=10090 GN=Timp3 PE=1 SV=1
Cyclin-dependent kinase 4 (Fragment) OS=Mus musculus OX=10090 GN=Cdk4 PE=1 SV=1
DNA topoisomerase 2-beta OS=Mus musculus OX=10090 GN=Top2b PE=1 SV=2
Beta-arrestin-1 OS=Mus musculus OX=10090 GN=Arrb1 PE=1 SV=1
ATP-binding cassette sub-family A member 9 OS=Mus musculus OX=10090 GN=Abca9 PE=1 SV=2
RNA/RNP complex-1-interacting phosphatase OS=Mus musculus OX=10090 GN=Dusp11 PE=2 SV=1
Mesoderm-specific transcript protein OS=Mus musculus OX=10090 GN=Mest PE=1 SV=2
Protein PRRC2C OS=Mus musculus OX=10090 GN=Prrc2c PE=1 SV=1
Coactosin-like protein OS=Mus musculus OX=10090 GN=Cotl1 PE=1 SV=3
TBC1 domain family member 22A OS=Mus musculus OX=10090 GN=Tbc1d22a PE=1 SV=3
Acetyl-CoA carboxylase kinase OS=Mus musculus OX=10090 GN=Prkaa1 PE=1 SV=1

SUMO-activating enzyme subunit 2 OS=Mus musculus OX=10090 GN=Uba2 PE=1 SV=1
Zinc finger protein 266 OS=Mus musculus OX=10090 GN=Zfp266 PE=1 SV=1
Agrin OS=Mus musculus OX=10090 GN=Agrn PE=1 SV=1
Signal recognition particle 19 kDa protein OS=Mus musculus OX=10090 GN=Srp19 PE=1 SV=1
Splicing factor 3A subunit 3 OS=Mus musculus OX=10090 GN=Sf3a3 PE=1 SV=2
Endophilin-A1 OS=Mus musculus OX=10090 GN=Sh3gl2 PE=1 SV=1
40S ribosomal protein S27 OS=Mus musculus OX=10090 GN=Rps27 PE=1 SV=3
Chromodomain-helicase-DNA-binding protein 2 OS=Mus musculus OX=10090 GN=Chd2 PE=1 SV=1
Spliceosome-associated protein CWC15 homolog OS=Mus musculus OX=10090 GN=Cwc15 PE=1 SV=1
Receptor expression-enhancing protein 3 OS=Mus musculus OX=10090 GN=Reep3 PE=1 SV=1
Proline-, glutamic acid- and leucine-rich protein 1 OS=Mus musculus OX=10090 GN=Pelp1 PE=1 SV=2
Bridging integrator 2 OS=Mus musculus OX=10090 GN=Bin2 PE=1 SV=1
1-phosphatidylinositol 4,5-bisphosphate phosphodiesterase OS=Mus musculus OX=10090 GN=Plcb4 PE=1 SV=1
U2 small nuclear ribonucleoprotein B'' OS=Mus musculus OX=10090 GN=Snrpb2 PE=1 SV=1
Synembryn-A OS=Mus musculus OX=10090 GN=Ric8a PE=1 SV=2
Corticosteroid 11-beta-dehydrogenase isozyme 1 OS=Mus musculus OX=10090 GN=Hsd11b1 PE=1 SV=3
Nuclear pore glycoprotein p62 OS=Mus musculus OX=10090 GN=Nup62 PE=1 SV=2
Interleukin-6 receptor subunit beta OS=Mus musculus OX=10090 GN=Il6st PE=1 SV=1
Cathepsin G OS=Mus musculus OX=10090 GN=Ctsg PE=1 SV=2
WASH complex subunit 2 (Fragment) OS=Mus musculus OX=10090 GN=Washc2 PE=1 SV=1
Methylosome protein 50 OS=Mus musculus OX=10090 GN=Wdr77 PE=1 SV=1
Cdc42 effector protein 2 OS=Mus musculus OX=10090 GN=Cdc42ep2 PE=1 SV=1
cAMP-dependent protein kinase type I-alpha regulatory subunit OS=Mus musculus OX=10090 GN=Prkar1a PE=1 S
Nuclear export mediator factor Nemf OS=Mus musculus OX=10090 GN=Nemf PE=1 SV=2
Tax1-binding protein 1 homolog OS=Mus musculus OX=10090 GN=Tax1bp1 PE=1 SV=2
7-methylguanosine phosphate-specific 5'-nucleotidase OS=Mus musculus OX=10090 GN=Nt5c3b PE=1 SV=3
Guanine nucleotide-binding protein G(I)/G(S)/G(O) subunit gamma-5 OS=Mus musculus OX=10090 GN=Gng5 PE
Peroxiredoxin-4 OS=Mus musculus OX=10090 GN=Prdx4 PE=1 SV=1
Ubiquitin carboxyl-terminal hydrolase 19 OS=Mus musculus OX=10090 GN=Usp19 PE=1 SV=1
AW555464 protein OS=Mus musculus OX=10090 GN=Cep170b PE=1 SV=1
DNA excision repair protein ERCC-6-like 2 OS=Mus musculus OX=10090 GN=Ercc6l2 PE=1 SV=3
Mitochondrial carnitine/acylcarnitine carrier protein OS=Mus musculus OX=10090 GN=Slc25a20 PE=1 SV=1
T-complex protein 1 subunit gamma OS=Mus musculus OX=10090 GN=Cct3 PE=1 SV=1
Unconventional myosin-X OS=Mus musculus OX=10090 GN=Myo10 PE=1 SV=1
Nucleolar and spindle-associated protein 1 OS=Mus musculus OX=10090 GN=Nusap1 PE=1 SV=1
RAB9, member RAS oncogene family (Fragment) OS=Mus musculus OX=10090 GN=Rab9 PE=1 SV=1
Interferon-stimulated 20 kDa exonuclease-like 2 OS=Mus musculus OX=10090 GN=Isg20l2 PE=1 SV=1
Lysine-specific demethylase 5D OS=Mus musculus OX=10090 GN=Kdm5d PE=2 SV=2
Glutamate dehydrogenase 1, mitochondrial OS=Mus musculus OX=10090 GN=Glud1 PE=1 SV=1
Pre-mRNA-processing factor 17 OS=Mus musculus OX=10090 GN=Cdc40 PE=2 SV=1
WD repeat-containing protein 53 OS=Mus musculus OX=10090 GN=Wdr53 PE=1 SV=1
SWI/SNF-related matrix-associated actin-dependent regulator of chromatin subfamily E member 1 OS=Mus mus
RNA 3'-terminal phosphate cyclase OS=Mus musculus OX=10090 GN=RtcA PE=1 SV=2
Protein ABHD15 OS=Mus musculus OX=10090 GN=Abhd15 PE=1 SV=1
Nuclear distribution protein nudE homolog 1 OS=Mus musculus OX=10090 GN=Nde1 PE=1 SV=1

Serine/threonine-protein phosphatase 2A 55 kDa regulatory subunit B delta isoform OS=Mus musculus OX=1009[illegible]
Stromal interaction molecule 1 OS=Mus musculus OX=10090 GN=Stim1 PE=1 SV=1
F-actin-capping protein subunit beta OS=Mus musculus OX=10090 GN=Capzb PE=1 SV=1
SUMO-activating enzyme subunit 1 OS=Mus musculus OX=10090 GN=Sae1 PE=1 SV=1
Protein SGT1 homolog OS=Mus musculus OX=10090 GN=Sugt1 PE=1 SV=3
Vacuolar protein sorting-associated protein 41 homolog OS=Mus musculus OX=10090 GN=Vps41 PE=1 SV=1
60S ribosome subunit biogenesis protein NIP7 homolog OS=Mus musculus OX=10090 GN=Nip7 PE=1 SV=1
Choline/ethanolaminephosphotransferase 1 (Fragment) OS=Mus musculus OX=10090 GN=Cept1 PE=1 SV=1
Polypyrimidine tract-binding protein 1 OS=Mus musculus OX=10090 GN=Ptbp1 PE=1 SV=1
Ubiquilin-1 OS=Mus musculus OX=10090 GN=Ubqln1 PE=1 SV=1
DDB1- and CUL4-associated factor 12 OS=Mus musculus OX=10090 GN=Dcaf12 PE=2 SV=1
Histone H2A type 2-C OS=Mus musculus OX=10090 GN=H2ac20 PE=1 SV=3
Beta-2-syntrophin OS=Mus musculus OX=10090 GN=Sntb2 PE=1 SV=2
Leucine-rich repeat-containing protein 47 OS=Mus musculus OX=10090 GN=Lrrc47 PE=1 SV=1
H/ACA ribonucleoprotein complex subunit (Fragment) OS=Mus musculus OX=10090 GN=Gar1 PE=1 SV=1
26S proteasome non-ATPase regulatory subunit 12 OS=Mus musculus OX=10090 GN=Psmd12 PE=1 SV=4
Sec24-related gene family, member D (S. cerevisiae) OS=Mus musculus OX=10090 GN=Sec24d PE=1 SV=1
Calreticulin OS=Mus musculus OX=10090 GN=Calr PE=1 SV=1
Probable crossover junction endonuclease EME2 OS=Mus musculus OX=10090 GN=Eme2 PE=2 SV=2
Exocyst complex component 3-like 2 OS=Mus musculus OX=10090 GN=Exoc3l2 PE=1 SV=2
DNA-directed RNA polymerases I and III subunit RPAC2 OS=Mus musculus OX=10090 GN=Polr1d PE=1 SV=1
Presenilins-associated rhomboid-like protein, mitochondrial OS=Mus musculus OX=10090 GN=Parl PE=1 SV=1
Synapse defective 1, Rho GTPase, homolog 2 (C. elegans) OS=Mus musculus OX=10090 GN=Syde2 PE=1 SV=2
Msx2-interacting protein OS=Mus musculus OX=10090 GN=Spen PE=1 SV=2
Myosin-1F-like protein OS=Mus musculus OX=10090 GN=Myo1f PE=1 SV=1
Nuclear pore complex protein Nup214 OS=Mus musculus OX=10090 GN=Nup214 PE=1 SV=2
LIM domain-containing protein 1 OS=Mus musculus OX=10090 GN=Limd1 PE=1 SV=2
Ankyrin repeat domain-containing protein 17 OS=Mus musculus OX=10090 GN=Ankrd17 PE=1 SV=3
BRCA1-associated protein OS=Mus musculus OX=10090 GN=Brap PE=1 SV=1
Dual specificity protein kinase CLK4 OS=Mus musculus OX=10090 GN=Clk4 PE=1 SV=1
Cullin-5 OS=Mus musculus OX=10090 GN=Cul5 PE=1 SV=3
DDB1- and CUL4-associated factor 13 OS=Mus musculus OX=10090 GN=Dcaf13 PE=2 SV=2
ATP-dependent RNA helicase DHX29 OS=Mus musculus OX=10090 GN=Dhx29 PE=1 SV=1
E3 ubiquitin-protein ligase SHPRH OS=Mus musculus OX=10090 GN=Shprh PE=1 SV=1
Dual specificity protein kinase CLK3 OS=Mus musculus OX=10090 GN=Clk3 PE=1 SV=2
Epidermal growth factor-like protein 6 OS=Mus musculus OX=10090 GN=Egfl6 PE=1 SV=1
Zinc finger MYM-type protein 3 OS=Mus musculus OX=10090 GN=Zmym3 PE=1 SV=1
Integrator complex subunit 6-like OS=Mus musculus OX=10090 GN=Ints6l PE=2 SV=1
Protein MAK16 homolog OS=Mus musculus OX=10090 GN=Mak16 PE=1 SV=1
Constitutive coactivator of PPAR-gamma-like protein 1 OS=Mus musculus OX=10090 GN=FAM120A PE=1 SV=2
Tyrosine-protein kinase receptor Tie-1 OS=Mus musculus OX=10090 GN=Tie1 PE=1 SV=3
Prostaglandin E synthase 2 OS=Mus musculus OX=10090 GN=Ptges2 PE=1 SV=3
Baculoviral IAP repeat-containing protein 1b OS=Mus musculus OX=10090 GN=Naip2 PE=1 SV=2
Ribosomal oxygenase 1 OS=Mus musculus OX=10090 GN=Riox1 PE=1 SV=2
Heparin cofactor 2 OS=Mus musculus OX=10090 GN=Serpind1 PE=1 SV=1

SEC23-interacting protein OS=Mus musculus OX=10090 GN=Sec23ip PE=1 SV=1
Caprin-1 OS=Mus musculus OX=10090 GN=Caprin1 PE=1 SV=2
Phosphatidylinositol 3,4,5-trisphosphate-dependent Rac exchanger 1 protein OS=Mus musculus OX=10090 GN=P
Microtubule-associated protein RP/EB family member 1 OS=Mus musculus OX=10090 GN=Mapre1 PE=1 SV=3
Membrane-associated guanylate kinase, WW and PDZ domain-containing protein 1 OS=Mus musculus OX=1009
Thymosin beta-4 OS=Mus musculus OX=10090 GN=Tmsb4x PE=1 SV=1
Chemokine-like receptor 1 OS=Mus musculus OX=10090 GN=Cmklr1 PE=1 SV=1
Synaptotagmin-15 OS=Mus musculus OX=10090 GN=Syt15 PE=2 SV=1
Casein kinase I isoform alpha OS=Mus musculus OX=10090 GN=Csnk1a1 PE=1 SV=2
Zinc finger and BTB domain-containing protein 7A OS=Mus musculus OX=10090 GN=Zbtb7a PE=1 SV=2
Tubulin beta-2B chain OS=Mus musculus OX=10090 GN=Tubb2b PE=1 SV=1
Erbin OS=Mus musculus OX=10090 GN=Erbin PE=1 SV=3
EF-hand domain-containing protein D2 OS=Mus musculus OX=10090 GN=Efhd2 PE=1 SV=1
Immunoglobulin heavy constant mu OS=Mus musculus OX=10090 GN=Ighm PE=1 SV=2
Histone-binding protein RBBP4 OS=Mus musculus OX=10090 GN=Rbbp4 PE=1 SV=5
Fibrinogen alpha chain OS=Mus musculus OX=10090 GN=Fga PE=1 SV=1
14-3-3 protein theta OS=Mus musculus OX=10090 GN=Ywhaq PE=1 SV=1
Synaptotagmin-like protein 2 OS=Mus musculus OX=10090 GN=Sytl2 PE=1 SV=2
Acyl-protein thioesterase 2 OS=Mus musculus OX=10090 GN=Lypla2 PE=1 SV=1
Fibronectin type III domain-containing protein 3B OS=Mus musculus OX=10090 GN=Fndc3b PE=1 SV=1
Protein Smaug homolog 1 OS=Mus musculus OX=10090 GN=Samd4a PE=1 SV=2
MAP kinase-activated protein kinase 3 OS=Mus musculus OX=10090 GN=Mapkapk3 PE=1 SV=2
RNA transcription, translation and transport factor protein OS=Mus musculus OX=10090 GN=RTRAF PE=1 SV=1
Carabin OS=Mus musculus OX=10090 GN=Tbc1d10c PE=1 SV=1
CAP-Gly domain-containing linker protein 2 OS=Mus musculus OX=10090 GN=Clip2 PE=1 SV=2
Ras-related protein Rab-35 OS=Mus musculus OX=10090 GN=Rab35 PE=1 SV=1
TBC1 domain family member 1 OS=Mus musculus OX=10090 GN=Tbc1d1 PE=1 SV=3
ATP-binding cassette sub-family G member 1 OS=Mus musculus OX=10090 GN=Abcg1 PE=1 SV=1
Potassium voltage-gated channel subfamily KQT member 5 OS=Mus musculus OX=10090 GN=Kcnq5 PE=1 SV=1
Thrombomodulin OS=Mus musculus OX=10090 GN=Thbd PE=1 SV=1
Protein dopey-2 OS=Mus musculus OX=10090 GN=Dop1b PE=1 SV=3
ADP-ribosyl cyclase/cyclic ADP-ribose hydrolase 1 OS=Mus musculus OX=10090 GN=Cd38 PE=1 SV=2
Mitochondrial import inner membrane translocase subunit TIM50 OS=Mus musculus OX=10090 GN=Timm50 PE
Zinc finger protein 316 OS=Mus musculus OX=10090 GN=Znf316 PE=2 SV=1
Programmed cell death protein 7 OS=Mus musculus OX=10090 GN=Pdcd7 PE=1 SV=1
Centromere protein C OS=Mus musculus OX=10090 GN=Cenpc PE=1 SV=2
Brefeldin A-inhibited guanine nucleotide-exchange protein 2 OS=Mus musculus OX=10090 GN=Arfgef2 PE=1 SV=
UAP56-interacting factor OS=Mus musculus OX=10090 GN=Fyttd1 PE=1 SV=1
Mitochondrial import inner membrane translocase subunit Tim21 OS=Mus musculus OX=10090 GN=Timm21 PE
Chromosome alignment-maintaining phosphoprotein 1 OS=Mus musculus OX=10090 GN=Champ1 PE=1 SV=1
Delta-sarcoglycan OS=Mus musculus OX=10090 GN=Sgcd PE=1 SV=1
Phosphatidylinositol-4,5-bisphosphate 4-phosphatase OS=Mus musculus OX=10090 GN=Pip4p1 PE=1 SV=1
Cyclin-dependent kinase 13 OS=Mus musculus OX=10090 GN=Cdk13 PE=1 SV=3
Paladin OS=Mus musculus OX=10090 GN=Pald1 PE=1 SV=1
Pleckstrin OS=Mus musculus OX=10090 GN=Plek PE=1 SV=1

Serine/arginine-rich splicing factor 9 OS=Mus musculus OX=10090 GN=Srsf9 PE=1 SV=1
E3 SUMO-protein ligase CBX4 OS=Mus musculus OX=10090 GN=Cbx4 PE=1 SV=2
Prostaglandin E synthase 3 OS=Mus musculus OX=10090 GN=Ptges3 PE=1 SV=1
Sodium-dependent phosphate transport protein 2B OS=Mus musculus OX=10090 GN=Slc34a2 PE=1 SV=1
Inositol 1,4,5-trisphosphate receptor-interacting protein OS=Mus musculus OX=10090 GN=Itprip PE=1 SV=1
Protein SREK1IP1 OS=Mus musculus OX=10090 GN=Srek1ip1 PE=1 SV=1
Ly6g6f protein OS=Mus musculus OX=10090 GN=Ly6g6f PE=1 SV=1
Protein-tyrosine-phosphatase OS=Mus musculus OX=10090 GN=Ptprd PE=1 SV=2
Krueppel-like factor 2 OS=Mus musculus OX=10090 GN=Klf2 PE=1 SV=2
3-ketodihydrosphingosine reductase OS=Mus musculus OX=10090 GN=Kdsr PE=1 SV=1
Myoneurin OS=Mus musculus OX=10090 GN=Mynn PE=2 SV=2
Zinc finger protein 26 OS=Mus musculus OX=10090 GN=Zfp26 PE=2 SV=2
28S ribosomal protein S9, mitochondrial OS=Mus musculus OX=10090 GN=Mrps9 PE=1 SV=3
Junction-mediating and -regulatory protein OS=Mus musculus OX=10090 GN=Jmy PE=1 SV=1
G patch domain-containing protein 2 OS=Mus musculus OX=10090 GN=Gpatch2 PE=1 SV=1
Rho guanine nucleotide exchange factor 5 OS=Mus musculus OX=10090 GN=Arhgef5 PE=1 SV=1
Helicase with zinc finger domain 2 OS=Mus musculus OX=10090 GN=Helz2 PE=1 SV=1
Sorcin OS=Mus musculus OX=10090 GN=Sri PE=1 SV=1
Prolactin regulatory element-binding protein OS=Mus musculus OX=10090 GN=Preb PE=1 SV=1
cGMP-inhibited 3',5'-cyclic phosphodiesterase A OS=Mus musculus OX=10090 GN=Pde3a PE=1 SV=1
Nodal modulator 1 OS=Mus musculus OX=10090 GN=Nomo1 PE=1 SV=1
[Histone H3]-lysine(4) N-trimethyltransferase OS=Mus musculus OX=10090 GN=Kmt2c PE=1 SV=1
Heat shock protein HSP 90-beta OS=Mus musculus OX=10090 GN=Hsp90ab1 PE=1 SV=3
BMP-binding endothelial regulator protein OS=Mus musculus OX=10090 GN=Bmper PE=1 SV=1
Matrin-3 OS=Mus musculus OX=10090 GN=Matr3 PE=1 SV=1
Vacuolar protein sorting-associated protein 33B OS=Mus musculus OX=10090 GN=Vps33b PE=1 SV=1
Coagulation factor V OS=Mus musculus OX=10090 GN=F5 PE=1 SV=1
Calcium-transporting ATPase OS=Mus musculus OX=10090 GN=Atp2a3 PE=1 SV=1
RAS protein activator-like 2 OS=Mus musculus OX=10090 GN=Rasal2 PE=1 SV=1
Bromodomain and PHD finger containing, 1 OS=Mus musculus OX=10090 GN=Brpf1 PE=1 SV=1
Dihydrolipoyllysine-residue acetyltransferase component of pyruvate dehydrogenase complex, mitochondrial OS=
Sp140 nuclear body protein OS=Mus musculus OX=10090 GN=Sp140 PE=1 SV=1
Armadillo repeat protein deleted in velo-cardio-facial syndrome homolog OS=Mus musculus OX=10090 GN=Arvc
WD repeat-containing protein 70 OS=Mus musculus OX=10090 GN=Wdr70 PE=1 SV=1
Ataxin-2-like protein OS=Mus musculus OX=10090 GN=Atxn2l PE=1 SV=1
Death-inducer obliterator 1 OS=Mus musculus OX=10090 GN=Dido1 PE=1 SV=4
Mitogen-activated protein kinase kinase kinase kinase 5 OS=Mus musculus OX=10090 GN=Map4k5 PE=1 SV=1
V-type proton ATPase subunit d 1 OS=Mus musculus OX=10090 GN=Atp6v0d1 PE=1 SV=2
Zinc finger protein 532 (Fragment) OS=Mus musculus OX=10090 GN=Zfp532 PE=1 SV=1
WD repeat and FYVE domain-containing protein 1 OS=Mus musculus OX=10090 GN=Wdfy1 PE=1 SV=1
DNA repair endonuclease XPF (Fragment) OS=Mus musculus OX=10090 GN=Ercc4 PE=1 SV=1
KAT8 regulatory NSL complex subunit 3 OS=Mus musculus OX=10090 GN=Kansl3 PE=1 SV=1
DNA replication licensing factor MCM2 OS=Mus musculus OX=10090 GN=Mcm2 PE=1 SV=3
Insulin-like growth factor-binding protein 5 OS=Mus musculus OX=10090 GN=Igfbp5 PE=1 SV=1
Ubiquitinyl hydrolase 1 OS=Mus musculus OX=10090 GN=Usp9x PE=1 SV=1

Ras-related C3 botulinum toxin substrate 1 OS=Mus musculus OX=10090 GN=Rac1 PE=1 SV=1
DNA excision repair protein ERCC-6 OS=Mus musculus OX=10090 GN=Ercc6 PE=1 SV=1
Phosphodiesterase OS=Mus musculus OX=10090 GN=Pde5a PE=1 SV=1
Tubulin polymerization-promoting protein family member 3 OS=Mus musculus OX=10090 GN=Tppp3 PE=1 SV=1
Band 4.1-like protein 2 OS=Mus musculus OX=10090 GN=Epb41l2 PE=1 SV=1
Collectin-12 OS=Mus musculus OX=10090 GN=Colec12 PE=1 SV=1
39S ribosomal protein L9, mitochondrial OS=Mus musculus OX=10090 GN=Mrpl9 PE=1 SV=1
Zinc finger protein 668 OS=Mus musculus OX=10090 GN=Znf668 PE=2 SV=1
Ceramide transfer protein OS=Mus musculus OX=10090 GN=Cert1 PE=1 SV=1
Alcohol dehydrogenase 1 OS=Mus musculus OX=10090 GN=Adh1 PE=1 SV=2
Olfactomedin-like protein 3 OS=Mus musculus OX=10090 GN=Olfml3 PE=1 SV=1
Ubiquitin carboxyl-terminal hydrolase 40 OS=Mus musculus OX=10090 GN=Usp40 PE=1 SV=2
Mitogen-activated protein kinase kinase kinase 5 OS=Mus musculus OX=10090 GN=Map3k5 PE=1 SV=3
Reticulocalbin-2 OS=Mus musculus OX=10090 GN=Rcn2 PE=1 SV=1
Mitochondrial carrier homolog 1 OS=Mus musculus OX=10090 GN=Mtch1 PE=1 SV=1
eIF-2-alpha kinase activator GCN1 OS=Mus musculus OX=10090 GN=Gcn1 PE=1 SV=1
Tubulin alpha chain (Fragment) OS=Mus musculus OX=10090 GN=Tuba4a PE=1 SV=1
Guanine nucleotide-binding protein G(s) subunit alpha isoforms short OS=Mus musculus OX=10090 GN=Gnas PE
Ras-related protein Rab-12 OS=Mus musculus OX=10090 GN=Rab12 PE=1 SV=1
Glutathione S-transferase theta-1 OS=Mus musculus OX=10090 GN=Gstt1 PE=1 SV=4
Transcriptional-regulating factor 1 OS=Mus musculus OX=10090 GN=Trerf1 PE=1 SV=1
Myosin light polypeptide 6 OS=Mus musculus OX=10090 GN=Myl6 PE=1 SV=3
Beta-parvin OS=Mus musculus OX=10090 GN=Parvb PE=1 SV=1
3222402P14Rik protein OS=Mus musculus OX=10090 GN=Ppp2r3a PE=1 SV=1
28S ribosomal protein S24, mitochondrial OS=Mus musculus OX=10090 GN=Mrps24 PE=1 SV=1
Cytoplasmic dynein 1 intermediate chain 2 OS=Mus musculus OX=10090 GN=Dync1i2 PE=1 SV=1
BTB/POZ domain-containing protein KCTD20 (Fragment) OS=Mus musculus OX=10090 GN=Kctd20 PE=1 SV=2
Serine and arginine-rich-splicing factor 11 (Fragment) OS=Mus musculus OX=10090 GN=Srsf11 PE=1 SV=1
Heterochromatin protein 1-binding protein 3 OS=Mus musculus OX=10090 GN=Hp1bp3 PE=1 SV=1
Sphingosine 1-phosphate receptor 1 OS=Mus musculus OX=10090 GN=S1pr1 PE=1 SV=3
Oxysterol-binding protein OS=Mus musculus OX=10090 GN=Osbpl6 PE=1 SV=1
Semaphorin-3A OS=Mus musculus OX=10090 GN=Sema3a PE=1 SV=2
AT-rich interactive domain-containing protein 2 OS=Mus musculus OX=10090 GN=Arid2 PE=1 SV=1
Syntaxin-4 OS=Mus musculus OX=10090 GN=Stx4 PE=1 SV=1
Probable ATP-dependent RNA helicase DDX27 OS=Mus musculus OX=10090 GN=Ddx27 PE=1 SV=3
Protein lin-7 homolog C OS=Mus musculus OX=10090 GN=Lin7c PE=1 SV=2
Cdc42 effector protein 4 (Fragment) OS=Mus musculus OX=10090 GN=Cdc42ep4 PE=1 SV=8
Uteroglobin OS=Mus musculus OX=10090 GN=Scgb1a1 PE=1 SV=1
Cytochrome P450 2F2 OS=Mus musculus OX=10090 GN=Cyp2f2 PE=1 SV=1
Zfp219 protein OS=Mus musculus OX=10090 GN=Zfp219 PE=1 SV=1
Anthrax toxin receptor 2 OS=Mus musculus OX=10090 GN=Antxr2 PE=1 SV=1
Surfeit locus protein 6 OS=Mus musculus OX=10090 GN=Surf6 PE=1 SV=1
COUP transcription factor 2 OS=Mus musculus OX=10090 GN=Nr2f2 PE=1 SV=2
ATP-dependent RNA helicase DDX50 OS=Mus musculus OX=10090 GN=Ddx50 PE=2 SV=1
Tubulin alpha-1A chain OS=Mus musculus OX=10090 GN=Tuba1a PE=1 SV=1

Protein numb homolog OS=Mus musculus OX=10090 GN=Numb PE=1 SV=1
Transaldolase OS=Mus musculus OX=10090 GN=Taldo1 PE=1 SV=2
ATP-dependent 6-phosphofructokinase OS=Mus musculus OX=10090 GN=Pfkp PE=1 SV=1
Zinc finger X-chromosomal protein OS=Mus musculus OX=10090 GN=Zfx PE=1 SV=2
Beta-1,4 N-acetylgalactosaminyltransferase 1 OS=Mus musculus OX=10090 GN=B4galnt1 PE=1 SV=1
Unconventional myosin-Id OS=Mus musculus OX=10090 GN=Myo1d PE=1 SV=1
Disks large homolog 3 OS=Mus musculus OX=10090 GN=Dlg3 PE=1 SV=1
Eukaryotic peptide chain release factor subunit 1 OS=Mus musculus OX=10090 GN=Etf1 PE=1 SV=4
DnaJ homolog subfamily C member 21 OS=Mus musculus OX=10090 GN=Dnajc21 PE=1 SV=1
Vacuolar protein-sorting-associated protein 36 OS=Mus musculus OX=10090 GN=Vps36 PE=1 SV=1
Adenylyltransferase and sulfurtransferase MOCS3 OS=Mus musculus OX=10090 GN=Mocs3 PE=1 SV=1
Diacylglycerol kinase OS=Mus musculus OX=10090 GN=Dgkz PE=1 SV=1
U2 snRNP-associated SURP motif-containing protein OS=Mus musculus OX=10090 GN=U2surp PE=1 SV=3
Nicotinamide phosphoribosyltransferase OS=Mus musculus OX=10090 GN=Nampt PE=1 SV=1
ATP synthase subunit alpha, mitochondrial OS=Mus musculus OX=10090 GN=Atp5f1a PE=1 SV=1
Stress-70 protein, mitochondrial OS=Mus musculus OX=10090 GN=Hspa9 PE=1 SV=3
Alpha-actinin-1 OS=Mus musculus OX=10090 GN=Actn1 PE=1 SV=1
Nuclear factor NF-kappa-B p100 subunit OS=Mus musculus OX=10090 GN=Nfkb2 PE=1 SV=1
PAT complex subunit CCDC47 OS=Mus musculus OX=10090 GN=Ccdc47 PE=1 SV=2
Ras-specific guanine nucleotide-releasing factor 2 OS=Mus musculus OX=10090 GN=Rasgrf2 PE=1 SV=2
Rhombotin-2 OS=Mus musculus OX=10090 GN=Lmo2 PE=1 SV=1
Neutral alpha-glucosidase AB OS=Mus musculus OX=10090 GN=Ganab PE=1 SV=1
28S ribosomal protein S11, mitochondrial OS=Mus musculus OX=10090 GN=Mrps11 PE=1 SV=2
Induced myeloid leukemia cell differentiation protein Mcl-1 homolog OS=Mus musculus OX=10090 GN=Mcl1 PE=
Spectrin beta chain, erythrocytic OS=Mus musculus OX=10090 GN=Sptb PE=1 SV=4
Lysophosphatidylserine lipase ABHD12 OS=Mus musculus OX=10090 GN=Abhd12 PE=1 SV=2
Inactive tyrosine-protein kinase 7 OS=Mus musculus OX=10090 GN=Ptk7 PE=1 SV=1
Uncharacterized protein C11orf98 homolog OS=Mus musculus OX=10090 PE=1 SV=1
Kinesin-like protein KIF2A OS=Mus musculus OX=10090 GN=Kif2a PE=1 SV=2
RNA helicase OS=Mus musculus OX=10090 GN=Dhx35 PE=1 SV=1
Nephrin OS=Mus musculus OX=10090 GN=Nphs1 PE=1 SV=2
Albumin OS=Mus musculus OX=10090 GN=Alb PE=1 SV=3
Protein disulfide-isomerase A6 OS=Mus musculus OX=10090 GN=Pdia6 PE=1 SV=1
Transforming growth factor beta-1-induced transcript 1 protein OS=Mus musculus OX=10090 GN=Tgfb1i1 PE=1 [illegible]
Periodic tryptophan protein 1 homolog OS=Mus musculus OX=10090 GN=Pwp1 PE=1 SV=1
Ras and Rab interactor 2 OS=Mus musculus OX=10090 GN=Rin2 PE=1 SV=1
Plastin-1 OS=Mus musculus OX=10090 GN=Pls1 PE=1 SV=1
Peptidyl-prolyl cis-trans isomerase B OS=Mus musculus OX=10090 GN=Ppib PE=1 SV=2
Nitric oxide synthase-interacting protein OS=Mus musculus OX=10090 GN=Nosip PE=1 SV=1
Exocyst complex component 6B OS=Mus musculus OX=10090 GN=Exoc6b PE=1 SV=1
PHD finger protein 12 (Fragment) OS=Mus musculus OX=10090 GN=Phf12 PE=1 SV=1
Exosome complex exonuclease RRP42 OS=Mus musculus OX=10090 GN=Exosc7 PE=1 SV=2
Complement C1q subcomponent subunit C OS=Mus musculus OX=10090 GN=C1qc PE=1 SV=2
Vesicular integral-membrane protein VIP36 OS=Mus musculus OX=10090 GN=Lman2 PE=1 SV=2
Zinc finger protein 740 OS=Mus musculus OX=10090 GN=Znf740 PE=1 SV=1

40S ribosomal protein S17 OS=Mus musculus OX=10090 GN=Rps17 PE=1 SV=2
Proto-oncogene vav OS=Mus musculus OX=10090 GN=Vav1 PE=1 SV=1
Transmembrane protein 33 OS=Mus musculus OX=10090 GN=Tmem33 PE=1 SV=1
E3 ubiquitin-protein ligase HUWE1 OS=Mus musculus OX=10090 GN=Huwe1 PE=1 SV=5
Ubiquitin carboxyl-terminal hydrolase 15 OS=Mus musculus OX=10090 GN=Usp15 PE=1 SV=1
Cleavage and polyadenylation specificity factor subunit 6 OS=Mus musculus OX=10090 GN=Cpsf6 PE=1 SV=1
Furin OS=Mus musculus OX=10090 GN=Furin PE=1 SV=2
Probable ATP-dependent RNA helicase DDX28 OS=Mus musculus OX=10090 GN=Ddx28 PE=2 SV=2
Serine--tRNA ligase, mitochondrial OS=Mus musculus OX=10090 GN=Sars2 PE=1 SV=2
CLIP-associating protein 1 OS=Mus musculus OX=10090 GN=Clasp1 PE=1 SV=2
Suppressor of IKBKE 1 OS=Mus musculus OX=10090 GN=Sike1 PE=1 SV=1
FERM domain-containing protein 4A OS=Mus musculus OX=10090 GN=Frmd4a PE=1 SV=2
Cleavage and polyadenylation specificity factor subunit 7 OS=Mus musculus OX=10090 GN=Cpsf7 PE=1 SV=2
Short-chain specific acyl-CoA dehydrogenase, mitochondrial OS=Mus musculus OX=10090 GN=Acads PE=1 SV=2
Lysozyme C-2 OS=Mus musculus OX=10090 GN=Lyz2 PE=1 SV=2
Double-stranded RNA-specific adenosine deaminase OS=Mus musculus OX=10090 GN=Adar PE=1 SV=2
Zinc finger protein 48 OS=Mus musculus OX=10090 GN=Znf48 PE=1 SV=2
A-kinase anchor protein 12 OS=Mus musculus OX=10090 GN=Akap12 PE=1 SV=1
Sterol O-acyltransferase 1 OS=Mus musculus OX=10090 GN=Soat1 PE=1 SV=2
Pleckstrin homology domain-containing family O member 2 OS=Mus musculus OX=10090 GN=Plekho2 PE=1 SV=
Fibrinogen gamma chain OS=Mus musculus OX=10090 GN=Fgg PE=1 SV=1
LIM domain only 7 (Fragment) OS=Mus musculus OX=10090 GN=Lmo7 PE=1 SV=1
Microtubule-actin cross-linking factor 1 (Fragment) OS=Mus musculus OX=10090 GN=Macf1 PE=1 SV=1
RNA helicase OS=Mus musculus OX=10090 GN=Ddx23 PE=1 SV=1
Sortilin-related receptor OS=Mus musculus OX=10090 GN=Sorl1 PE=1 SV=3
Tyrosine-protein phosphatase non-receptor type 13 OS=Mus musculus OX=10090 GN=Ptpn13 PE=1 SV=1
Cofilin-1 OS=Mus musculus OX=10090 GN=Cfl1 PE=1 SV=3
Eukaryotic translation initiation factor 2D OS=Mus musculus OX=10090 GN=Eif2d PE=1 SV=1
Reticulocalbin-1 OS=Mus musculus OX=10090 GN=Rcn1 PE=1 SV=1
[Histone H3]-lysine(27) N-trimethyltransferase OS=Mus musculus OX=10090 GN=Ezh1 PE=1 SV=1
FtsJ RNA methyltransferase homolog 1 (E. coli) (Fragment) OS=Mus musculus OX=10090 GN=Ftsj1 PE=1 SV=1
Casein kinase I isoform delta OS=Mus musculus OX=10090 GN=Csnk1d PE=1 SV=2
GTPase-activating protein and VPS9 domain-containing protein 1 OS=Mus musculus OX=10090 GN=Gapvd1 PE=1
Leucine--tRNA ligase, cytoplasmic OS=Mus musculus OX=10090 GN=Lars1 PE=1 SV=2
Microtubule-actin cross-linking factor 1 (Fragment) OS=Mus musculus OX=10090 GN=Macf1 PE=1 SV=1
Proteasome subunit alpha type-4 OS=Mus musculus OX=10090 GN=Psma4 PE=1 SV=1
Dynamin-like 120 kDa protein, form S1 OS=Mus musculus OX=10090 GN=Opa1 PE=1 SV=1
Tudor domain-containing protein 7 OS=Mus musculus OX=10090 GN=Tdrd7 PE=1 SV=1
Protein Hook homolog 3 OS=Mus musculus OX=10090 GN=Hook3 PE=1 SV=2
Endonuclease 8-like 1 OS=Mus musculus OX=10090 GN=Neil1 PE=2 SV=3
Nuclear migration protein nudC OS=Mus musculus OX=10090 GN=Nudc PE=1 SV=1
Transmembrane protein 201 OS=Mus musculus OX=10090 GN=Tmem201 PE=1 SV=1
Golgi autoantigen, golgin subfamily b, macrogolgin 1 OS=Mus musculus OX=10090 GN=Golgb1 PE=1 SV=1
Importin subunit alpha-5 OS=Mus musculus OX=10090 GN=Kpna1 PE=1 SV=2
NEDD4-like E3 ubiquitin-protein ligase WWP2 OS=Mus musculus OX=10090 GN=Wwp2 PE=1 SV=1

Tyrosine-protein phosphatase non-receptor type 3 OS=Mus musculus OX=10090 GN=Ptpn3 PE=1 SV=1
Docking protein 1 OS=Mus musculus OX=10090 GN=Dok1 PE=1 SV=2
Glucose 1,6-bisphosphate synthase OS=Mus musculus OX=10090 GN=Pgm2l1 PE=1 SV=1
Angiopoietin-2 OS=Mus musculus OX=10090 GN=Angpt2 PE=2 SV=2
GTP-binding protein 2 OS=Mus musculus OX=10090 GN=Gtpbp2 PE=1 SV=1
Shiftless antiviral inhibitor of ribosomal frameshifting protein homolog OS=Mus musculus OX=10090 GN=Shfl PE
Immunoglobulin superfamily member 10 OS=Mus musculus OX=10090 GN=Igsf10 PE=2 SV=2
Calpain-1 catalytic subunit OS=Mus musculus OX=10090 GN=Capn1 PE=1 SV=1
Epidermal growth factor receptor substrate 15-like 1 OS=Mus musculus OX=10090 GN=Eps15l1 PE=1 SV=3
[F-actin]-monooxygenase MICAL1 OS=Mus musculus OX=10090 GN=Mical1 PE=1 SV=1
Laminin subunit alpha-5 OS=Mus musculus OX=10090 GN=Lama5 PE=1 SV=4
L-fucose kinase OS=Mus musculus OX=10090 GN=Fcsk PE=1 SV=1
Apoptosis inhibitor 5 OS=Mus musculus OX=10090 GN=Api5 PE=1 SV=2
Desmin OS=Mus musculus OX=10090 GN=Des PE=1 SV=3
Nidogen-1 OS=Mus musculus OX=10090 GN=Nid1 PE=1 SV=2
ER lumen protein-retaining receptor 1 OS=Mus musculus OX=10090 GN=Kdelr1 PE=1 SV=1
B-cell lymphoma 6 protein homolog OS=Mus musculus OX=10090 GN=Bcl6 PE=1 SV=1
Matrix metallopeptidase 28 (Epilysin) OS=Mus musculus OX=10090 GN=Mmp28 PE=1 SV=1
GTP-binding protein OS=Mus musculus OX=10090 GN=Rrad PE=1 SV=1
Pyridoxal kinase OS=Mus musculus OX=10090 GN=Pdxk PE=1 SV=1
Drebrin OS=Mus musculus OX=10090 GN=Dbn1 PE=1 SV=1
Probable ATP-dependent RNA helicase DDX17 OS=Mus musculus OX=10090 GN=Ddx17 PE=1 SV=1
3'-5' RNA helicase YTHDC2 OS=Mus musculus OX=10090 GN=Ythdc2 PE=1 SV=1
Ras-related protein Rap-1A OS=Mus musculus OX=10090 GN=Rap1a PE=1 SV=1
Guanine nucleotide-binding protein G(I)/G(S)/G(T) subunit beta-1 OS=Mus musculus OX=10090 GN=Gnb1 PE=1 S
Ribose-phosphate pyrophosphokinase 2 OS=Mus musculus OX=10090 GN=Prps2 PE=1 SV=4
Parkinson disease protein 7 homolog (Fragment) OS=Mus musculus OX=10090 GN=Park7 PE=1 SV=1
Ras-related protein Rab-21 OS=Mus musculus OX=10090 GN=Rab21 PE=1 SV=1
Serine/threonine-protein kinase VRK2 OS=Mus musculus OX=10090 GN=Vrk2 PE=1 SV=2
Unconventional myosin-Ie OS=Mus musculus OX=10090 GN=Myo1e PE=1 SV=1
Membrane-associated phosphatidylinositol transfer protein 1 OS=Mus musculus OX=10090 GN=Pitpnm1 PE=1 SV
Histone chaperone ASF1B OS=Mus musculus OX=10090 GN=Asf1b PE=1 SV=1
Actin-related protein 5 OS=Mus musculus OX=10090 GN=Actr5 PE=1 SV=1
Neuropilin-1 OS=Mus musculus OX=10090 GN=Nrp1 PE=1 SV=2
Histone H1.1 OS=Mus musculus OX=10090 GN=H1-1 PE=1 SV=2
Zinc finger protein 574 OS=Mus musculus OX=10090 GN=Znf574 PE=1 SV=1
Golgi resident protein GCP60 OS=Mus musculus OX=10090 GN=Acbd3 PE=1 SV=3
Protein SOGA1 OS=Mus musculus OX=10090 GN=Soga1 PE=1 SV=1
G patch domain-containing protein 8 OS=Mus musculus OX=10090 GN=Gpatch8 PE=1 SV=1
LLGL scribble cell polarity complex component 2 OS=Mus musculus OX=10090 GN=Llgl2 PE=1 SV=2
Double-stranded RNA-binding protein Staufen homolog 1 OS=Mus musculus OX=10090 GN=Stau1 PE=1 SV=1
Polycomb protein Suz12 OS=Mus musculus OX=10090 GN=Suz12 PE=1 SV=2
Something about silencing protein 10 OS=Mus musculus OX=10090 GN=Utp3 PE=1 SV=1
Heterogeneous nuclear ribonucleoprotein L-like OS=Mus musculus OX=10090 GN=Hnrnpll PE=1 SV=3
Elongation factor 1-gamma OS=Mus musculus OX=10090 GN=Eef1g PE=1 SV=3

Astrocytic phosphoprotein PEA-15 OS=Mus musculus OX=10090 GN=Pea15 PE=1 SV=1
F-box only protein 22 OS=Mus musculus OX=10090 GN=Fbxo22 PE=1 SV=1
Rho GDP-dissociation inhibitor 1 OS=Mus musculus OX=10090 GN=Arhgdia PE=1 SV=3
Ras-related protein Rab-18 OS=Mus musculus OX=10090 GN=Rab18 PE=1 SV=1
ATP-binding cassette sub-family E member 1 OS=Mus musculus OX=10090 GN=Abce1 PE=1 SV=1
Glutathione S-transferase theta-3 OS=Mus musculus OX=10090 GN=Gstt3 PE=1 SV=1
Coiled-coil domain-containing protein 82 OS=Mus musculus OX=10090 GN=Ccdc82 PE=2 SV=1
Dynamin GTPase OS=Mus musculus OX=10090 GN=Dnm2 PE=1 SV=1
Complement C1q subcomponent subunit B OS=Mus musculus OX=10090 GN=C1qb PE=1 SV=2
Exocyst complex component OS=Mus musculus OX=10090 GN=Exoc6 PE=1 SV=1
Splicing factor 3B subunit 1 OS=Mus musculus OX=10090 GN=Sf3b1 PE=1 SV=1
Probable bifunctional methylenetetrahydrofolate dehydrogenase/cyclohydrolase 2 OS=Mus musculus OX=10090 [illegible]
Forkhead box protein F1 OS=Mus musculus OX=10090 GN=Foxf1 PE=2 SV=2
Actin-related protein 2/3 complex subunit 1A OS=Mus musculus OX=10090 GN=Arpc1a PE=1 SV=1
Angiotensin-converting enzyme OS=Mus musculus OX=10090 GN=Ace PE=1 SV=3
Striatin-interacting protein 1 OS=Mus musculus OX=10090 GN=Strip1 PE=1 SV=2
Phosphoinositide 3-kinase regulatory subunit 4 OS=Mus musculus OX=10090 GN=Pik3r4 PE=1 SV=3
Nucleolysin TIAR OS=Mus musculus OX=10090 GN=Tial1 PE=1 SV=1
Spectrin alpha chain, erythrocytic 1 OS=Mus musculus OX=10090 GN=Spta1 PE=1 SV=3
Ragulator complex protein LAMTOR3 OS=Mus musculus OX=10090 GN=Lamtor3 PE=1 SV=1
Junctional adhesion molecule A OS=Mus musculus OX=10090 GN=F11r PE=1 SV=2
Platelet glycoprotein 4 OS=Mus musculus OX=10090 GN=Cd36 PE=1 SV=2
THO complex subunit 4 OS=Mus musculus OX=10090 GN=Alyref PE=1 SV=3
RNA polymerase II-associated protein 1 OS=Mus musculus OX=10090 GN=Rpap1 PE=1 SV=2
DNA replication licensing factor MCM4 OS=Mus musculus OX=10090 GN=Mcm4 PE=1 SV=1
Cilia- and flagella-associated protein 410 OS=Mus musculus OX=10090 GN=Cfap410 PE=1 SV=1
Transcription elongation factor SPT4-A OS=Mus musculus OX=10090 GN=Supt4h1a PE=2 SV=1
Filamin-B OS=Mus musculus OX=10090 GN=Flnb PE=1 SV=3
CDKN2A-interacting protein OS=Mus musculus OX=10090 GN=Cdkn2aip PE=1 SV=1
Programmed cell death 6-interacting protein OS=Mus musculus OX=10090 GN=Pdcd6ip PE=1 SV=3
Reticulon-4 OS=Mus musculus OX=10090 GN=Rtn4 PE=1 SV=2
Protein ecdysoneless homolog OS=Mus musculus OX=10090 GN=Ecd PE=1 SV=2
Histone acetyltransferase OS=Mus musculus OX=10090 GN=Kat5 PE=1 SV=1
Homeodomain-only protein OS=Mus musculus OX=10090 GN=Hopx PE=1 SV=1
Vacuolar protein sorting-associated protein 13A OS=Mus musculus OX=10090 GN=Vps13a PE=1 SV=1
60S ribosomal protein L9 OS=Mus musculus OX=10090 GN=Rpl9-ps6 PE=3 SV=1
Ubiquitin-conjugating enzyme E2 D3 OS=Mus musculus OX=10090 GN=Ube2d3 PE=1 SV=1
Band 4.1 OS=Mus musculus OX=10090 GN=Epb41 PE=1 SV=1
FACT complex subunit OS=Mus musculus OX=10090 GN=Supt16 PE=1 SV=1
Arginyl-tRNA--protein transferase 1 OS=Mus musculus OX=10090 GN=Ate1 PE=1 SV=1
General transcription factor 3C polypeptide 4 OS=Mus musculus OX=10090 GN=Gtf3c4 PE=1 SV=2
Polyunsaturated fatty acid lipoxygenase ALOX15 OS=Mus musculus OX=10090 GN=Alox15 PE=1 SV=4
60S ribosomal protein L8 OS=Mus musculus OX=10090 GN=Rpl8 PE=1 SV=2
Ribosomal protein 63, mitochondrial OS=Mus musculus OX=10090 GN=Mrpl57 PE=1 SV=1
Alpha-endosulfine OS=Mus musculus OX=10090 GN=Ensa PE=1 SV=1

Importin subunit alpha OS=Mus musculus OX=10090 GN=Kpna6 PE=1 SV=1
Phosphatidylinositol 4-phosphate 5-kinase type-1 alpha OS=Mus musculus OX=10090 GN=Pip5k1a PE=1 SV=1
Hedgehog-interacting protein OS=Mus musculus OX=10090 GN=Hhip PE=1 SV=2
Calcitonin gene-related peptide type 1 receptor OS=Mus musculus OX=10090 GN=Calcrl PE=1 SV=2
Histone-lysine N-methyltransferase 2A OS=Mus musculus OX=10090 GN=Kmt2a PE=1 SV=3
Protein kinase C OS=Mus musculus OX=10090 GN=Prkca PE=1 SV=1
Coiled-coil domain-containing 71-like OS=Mus musculus OX=10090 GN=Ccdc71l PE=1 SV=1
Ras-related protein Rap-2c OS=Mus musculus OX=10090 GN=Rap2c PE=1 SV=1
Cysteine-rich PDZ-binding protein OS=Mus musculus OX=10090 GN=Cript PE=1 SV=1
Proteolipid protein 2 OS=Mus musculus OX=10090 GN=Plp2 PE=1 SV=1
Ribose-phosphate diphosphokinase OS=Mus musculus OX=10090 GN=Prps1l3 PE=3 SV=1
Phostensin OS=Mus musculus OX=10090 GN=Ppp1r18 PE=1 SV=1
Protein scribble homolog OS=Mus musculus OX=10090 GN=Scrib PE=1 SV=2
Transcription factor PU.1 OS=Mus musculus OX=10090 GN=Spi1 PE=1 SV=2
Poly(rC)-binding protein 2 OS=Mus musculus OX=10090 GN=Pcbp2 PE=1 SV=1
Tripartite motif-containing protein 26 (Fragment) OS=Mus musculus OX=10090 GN=Trim26 PE=1 SV=1
Chromodomain helicase DNA-binding protein 3 (Fragment) OS=Mus musculus OX=10090 GN=Chd3 PE=1 SV=1
Histone RNA hairpin-binding protein OS=Mus musculus OX=10090 GN=Slbp PE=1 SV=1
CYFIP-related Rac1 interactor B OS=Mus musculus OX=10090 GN=Cyrib PE=1 SV=1
DnaJ homolog subfamily C member 14 OS=Mus musculus OX=10090 GN=Dnajc14 PE=2 SV=2
Arf-GAP with GTPase, ANK repeat and PH domain-containing protein 3 OS=Mus musculus OX=10090 GN=Agap3
Probable E3 ubiquitin-protein ligase HERC4 OS=Mus musculus OX=10090 GN=Herc4 PE=1 SV=2
Translocon-associated protein subunit gamma OS=Mus musculus OX=10090 GN=Ssr3 PE=1 SV=1
Stabilin-1 OS=Mus musculus OX=10090 GN=Stab1 PE=1 SV=1
ADP-ribosylation factor interacting protein 1 OS=Mus musculus OX=10090 GN=Arfip1 PE=1 SV=1
Transcription factor Sp2 OS=Mus musculus OX=10090 GN=Sp2 PE=2 SV=2
Ras GTPase-activating protein 3 OS=Mus musculus OX=10090 GN=Rasa3 PE=1 SV=2
Mediator of RNA polymerase II transcription subunit 21 OS=Mus musculus OX=10090 GN=Med21 PE=1 SV=1
Eukaryotic translation initiation factor 2 subunit 1 OS=Mus musculus OX=10090 GN=Eif2s1 PE=1 SV=3
La-related protein 7 OS=Mus musculus OX=10090 GN=Larp7 PE=1 SV=2
Cysteine-rich motor neuron 1 protein OS=Mus musculus OX=10090 GN=Crim1 PE=2 SV=2
Thioredoxin domain-containing protein 17 OS=Mus musculus OX=10090 GN=Txndc17 PE=1 SV=1
Tumor necrosis factor receptor superfamily member 5 (Fragment) OS=Mus musculus OX=10090 GN=Cd40 PE=1
YTH domain-containing protein 1 OS=Mus musculus OX=10090 GN=Ythdc1 PE=1 SV=2
Histone deacetylase 2 OS=Mus musculus OX=10090 GN=Hdac2 PE=1 SV=1
Triple functional domain protein OS=Mus musculus OX=10090 GN=Trio PE=1 SV=1
Mannosyl-oligosaccharide glucosidase OS=Mus musculus OX=10090 GN=Mogs PE=1 SV=1
Phosphatidylinositol 4,5-bisphosphate 3-kinase catalytic subunit alpha isoform OS=Mus musculus OX=10090 GN=
Zinc finger E-box-binding homeobox 1 OS=Mus musculus OX=10090 GN=Zeb1 PE=1 SV=1
Zinc finger protein 280C OS=Mus musculus OX=10090 GN=Znf280c PE=1 SV=1
Coiled-coil domain-containing protein 9B OS=Mus musculus OX=10090 GN=Ccdc9b PE=1 SV=1
Protein transport protein SEC23 OS=Mus musculus OX=10090 GN=Sec23a PE=1 SV=1
Glutaminyl-tRNA synthetase OS=Mus musculus OX=10090 GN=Qars PE=1 SV=2
Adhesion G protein-coupled receptor E5 OS=Mus musculus OX=10090 GN=Adgre5 PE=1 SV=1
Rab22B OS=Mus musculus OX=10090 GN=Rab31 PE=1 SV=1

F-box only protein 28 OS=Mus musculus OX=10090 GN=Fbxo28 PE=1 SV=1
Myotubularin OS=Mus musculus OX=10090 GN=Mtm1 PE=1 SV=2
Netrin-4 OS=Mus musculus OX=10090 GN=Ntn4 PE=1 SV=2
PDZ and LIM domain protein 5 OS=Mus musculus OX=10090 GN=Pdlim5 PE=1 SV=4
Pulmonary surfactant-associated protein A OS=Mus musculus OX=10090 GN=Sftpa1 PE=1 SV=1
Mitogen-activated protein kinase OS=Mus musculus OX=10090 GN=Mapk14 PE=1 SV=1
Rho GTPase-activating protein SYDE1 OS=Mus musculus OX=10090 GN=Syde1 PE=1 SV=1
ADP-ribosylation factor GTPase-activating protein 2 OS=Mus musculus OX=10090 GN=Arfgap2 PE=1 SV=1
Oxysterol-binding protein 1 OS=Mus musculus OX=10090 GN=Osbp PE=1 SV=3
Zinc finger and BTB domain-containing protein 1 OS=Mus musculus OX=10090 GN=Zbtb1 PE=1 SV=1
Arf-GAP with coiled-coil, ANK repeat and PH domain-containing protein 1 OS=Mus musculus OX=10090 GN=Acap
Destrin OS=Mus musculus OX=10090 GN=Dstn PE=1 SV=3
Regulator of nonsense transcripts 1 OS=Mus musculus OX=10090 GN=Upf1 PE=1 SV=2
Ufm1-specific protease 2 OS=Mus musculus OX=10090 GN=Ufsp2 PE=1 SV=1
26S proteasome non-ATPase regulatory subunit 5 OS=Mus musculus OX=10090 GN=Psmd5 PE=1 SV=4
Protein SERAC1 OS=Mus musculus OX=10090 GN=Serac1 PE=2 SV=1
Mutated in colorectal cancers OS=Mus musculus OX=10090 GN=Mcc PE=1 SV=1
Protein FAM110D OS=Mus musculus OX=10090 GN=Fam110d PE=2 SV=2
Myeloperoxidase OS=Mus musculus OX=10090 GN=Mpo PE=1 SV=2
Solute carrier organic anion transporter family member 2A1 OS=Mus musculus OX=10090 GN=Slco2a1 PE=1 SV=
DNA helicase OS=Mus musculus OX=10090 GN=Mcm6 PE=1 SV=1
Deoxyuridine 5'-triphosphate nucleotidohydrolase OS=Mus musculus OX=10090 GN=Dut PE=1 SV=1
Polycomb protein SCMH1 OS=Mus musculus OX=10090 GN=Scmh1 PE=1 SV=1
BRCA2-interacting transcriptional repressor EMSY (Fragment) OS=Mus musculus OX=10090 GN=Emsy PE=1 SV=[illegible]
HIV Tat-specific factor 1 homolog OS=Mus musculus OX=10090 GN=Htatsf1 PE=1 SV=1
Predicted gene, 49369 (Fragment) OS=Mus musculus OX=10090 GN=Gm49369 PE=1 SV=1
Titin OS=Mus musculus OX=10090 GN=Ttn PE=1 SV=1
26S proteasome non-ATPase regulatory subunit 2 OS=Mus musculus OX=10090 GN=Psmd2 PE=1 SV=1
Cell division cycle protein 16 homolog OS=Mus musculus OX=10090 GN=Cdc16 PE=1 SV=1
DENN domain-containing protein 4B OS=Mus musculus OX=10090 GN=Dennd4b PE=1 SV=2
2',3'-cyclic-nucleotide 3'-phosphodiesterase OS=Mus musculus OX=10090 GN=Cnp PE=1 SV=3
Peroxiredoxin-1 OS=Mus musculus OX=10090 GN=Prdx1 PE=1 SV=1
RRP12-like protein OS=Mus musculus OX=10090 GN=Rrp12 PE=1 SV=1
Phenylalanine--tRNA ligase beta subunit OS=Mus musculus OX=10090 GN=Farsb PE=1 SV=2
Centrin-2 OS=Mus musculus OX=10090 GN=Cetn2 PE=1 SV=1
Keratin, type II cytoskeletal 8 OS=Mus musculus OX=10090 GN=Krt8 PE=1 SV=4
Mitogen-activated protein kinase 1 OS=Mus musculus OX=10090 GN=Mapk1 PE=1 SV=3
Adaptin ear-binding coat-associated protein 2 (Fragment) OS=Mus musculus OX=10090 GN=Necap2 PE=1 SV=1
Predicted gene 29609 OS=Mus musculus OX=10090 GN=Gm29609 PE=2 SV=1
26S proteasome non-ATPase regulatory subunit 1 OS=Mus musculus OX=10090 GN=Psmd1 PE=1 SV=1
Jerky protein homolog-like OS=Mus musculus OX=10090 GN=Jrkl PE=2 SV=1
Protein-tyrosine kinase 2-beta OS=Mus musculus OX=10090 GN=Ptk2b PE=1 SV=2
CCN family member 2 OS=Mus musculus OX=10090 GN=Ccn2 PE=1 SV=3
Adenine DNA glycosylase OS=Mus musculus OX=10090 GN=Mutyh PE=1 SV=2
Core histone macro-H2A.1 OS=Mus musculus OX=10090 GN=Macroh2a1 PE=1 SV=3

DNA (cytosine-5)-methyltransferase 1 OS=Mus musculus OX=10090 GN=Dnmt1 PE=1 SV=5
BCL2/adenovirus E1B 19 kDa protein-interacting protein 2 OS=Mus musculus OX=10090 GN=Bnip2 PE=1 SV=1
Spermatogenesis-associated protein 13 OS=Mus musculus OX=10090 GN=Spata13 PE=1 SV=1
Integrator complex subunit 12 OS=Mus musculus OX=10090 GN=Ints12 PE=1 SV=1
Spermatogenesis-associated serine-rich protein 2 OS=Mus musculus OX=10090 GN=Spats2 PE=1 SV=1
Cytoplasmic dynein 1 heavy chain 1 OS=Mus musculus OX=10090 GN=Dync1h1 PE=1 SV=2
Ubiquitin-like modifier-activating enzyme ATG7 OS=Mus musculus OX=10090 GN=Atg7 PE=1 SV=1
Rho guanine nucleotide exchange factor 15 OS=Mus musculus OX=10090 GN=Arhgef15 PE=1 SV=1
Microtubule organization protein AKNA OS=Mus musculus OX=10090 GN=Akna PE=1 SV=1
Diacylglycerol kinase alpha OS=Mus musculus OX=10090 GN=Dgka PE=1 SV=2
ER degradation-enhancing alpha-mannosidase-like protein 2 OS=Mus musculus OX=10090 GN=Edem2 PE=1 SV=
Gelsolin OS=Mus musculus OX=10090 GN=Gsn PE=1 SV=3
Cell division cycle and apoptosis regulator protein 1 OS=Mus musculus OX=10090 GN=Ccar1 PE=1 SV=1
Aryl hydrocarbon receptor nuclear translocator OS=Mus musculus OX=10090 GN=Arnt PE=1 SV=2
Inosine-5'-monophosphate dehydrogenase OS=Mus musculus OX=10090 GN=Impdh1 PE=1 SV=1
Tectonin beta-propeller repeat-containing protein 1 OS=Mus musculus OX=10090 GN=Tecpr1 PE=1 SV=1
Cytohesin-4 OS=Mus musculus OX=10090 GN=Cyth4 PE=1 SV=1
Zinc finger CCCH type-containing 7 A OS=Mus musculus OX=10090 GN=Zc3h7a PE=1 SV=2
Myosin, light chain 12A, regulatory, non-sarcomeric OS=Mus musculus OX=10090 GN=Myl12a PE=1 SV=1
Serine/threonine-protein kinase RIO1 OS=Mus musculus OX=10090 GN=Riok1 PE=1 SV=2
Anaphase-promoting complex subunit 2 OS=Mus musculus OX=10090 GN=Anapc2 PE=1 SV=2
Small ubiquitin-related modifier 2 OS=Mus musculus OX=10090 GN=Sumo2 PE=1 SV=1
Signal peptide, CUB and EGF-like domain-containing protein 2 OS=Mus musculus OX=10090 GN=Scube2 PE=4 SV
Rho-associated protein kinase 2 OS=Mus musculus OX=10090 GN=Rock2 PE=1 SV=1
Cleavage and polyadenylation specificity factor subunit 5 OS=Mus musculus OX=10090 GN=Nudt21 PE=1 SV=1
Nucleolar protein 6 OS=Mus musculus OX=10090 GN=Nol6 PE=2 SV=2
Leucine-rich repeat flightless-interacting protein 2 OS=Mus musculus OX=10090 GN=Lrrfip2 PE=1 SV=1
Male-specific lethal 3 homolog OS=Mus musculus OX=10090 GN=Msl3 PE=1 SV=3
Ataxin-7 OS=Mus musculus OX=10090 GN=Atxn7 PE=1 SV=2
Proteasome subunit beta type-10 OS=Mus musculus OX=10090 GN=Psmb10 PE=1 SV=1
TATA-box-binding protein OS=Mus musculus OX=10090 GN=Tbp PE=1 SV=1
Deoxynucleotidyltransferase terminal-interacting protein 1 OS=Mus musculus OX=10090 GN=Dnttip1 PE=1 SV=1
Zinc finger protein 41 OS=Mus musculus OX=10090 GN=Zfp41 PE=2 SV=2
Tyrosine-protein kinase JAK3 OS=Mus musculus OX=10090 GN=Jak3 PE=1 SV=2
RIKEN cDNA C030006K11 gene OS=Mus musculus OX=10090 GN=C030006K11Rik PE=1 SV=1
Long-chain specific acyl-CoA dehydrogenase, mitochondrial OS=Mus musculus OX=10090 GN=Acadl PE=1 SV=1
1,4-alpha-glucan-branching enzyme OS=Mus musculus OX=10090 GN=Gbe1 PE=1 SV=1
COMM domain-containing protein 3 OS=Mus musculus OX=10090 GN=Commd3 PE=1 SV=1
EGF-containing fibulin-like extracellular matrix protein 2 (Fragment) OS=Mus musculus OX=10090 GN=Efemp2 F
Zinc finger protein 366 OS=Mus musculus OX=10090 GN=Znf366 PE=2 SV=1
28S ribosomal protein S23, mitochondrial OS=Mus musculus OX=10090 GN=Mrps23 PE=1 SV=1
Microsomal glutathione S-transferase 1 OS=Mus musculus OX=10090 GN=Mgst1 PE=1 SV=3
Rho guanine nucleotide exchange factor (GEF) 26 OS=Mus musculus OX=10090 GN=Arhgef26 PE=1 SV=1
Echinoderm microtubule-associated protein-like 1 OS=Mus musculus OX=10090 GN=Eml1 PE=1 SV=1
Phosphoinositide 3-kinase regulatory subunit 5 OS=Mus musculus OX=10090 GN=Pik3r5 PE=1 SV=1

TBC1 domain family member 30 OS=Mus musculus OX=10090 GN=Tbc1d30 PE=1 SV=2
60S ribosomal protein L13 OS=Mus musculus OX=10090 GN=Rpl13 PE=1 SV=3
Caveolae-associated protein 3 OS=Mus musculus OX=10090 GN=Cavin3 PE=1 SV=1
Protein THEM6 OS=Mus musculus OX=10090 GN=Them6 PE=1 SV=1
Myosin-9 OS=Mus musculus OX=10090 GN=Myh9 PE=1 SV=4
Coiled-coil domain-containing protein 68 OS=Mus musculus OX=10090 GN=Ccdc68 PE=2 SV=1
Calcyclin-binding protein OS=Mus musculus OX=10090 GN=Cacybp PE=1 SV=1
pre-rRNA 2'-O-ribose RNA methyltransferase FTSJ3 OS=Mus musculus OX=10090 GN=Ftsj3 PE=1 SV=1
Divergent protein kinase domain 2A OS=Mus musculus OX=10090 GN=Dipk2a PE=1 SV=2
60S ribosomal protein L23a OS=Mus musculus OX=10090 GN=Rpl23a PE=1 SV=1
Semaphorin-3G OS=Mus musculus OX=10090 GN=Sema3g PE=2 SV=1
Cyclin-G-associated kinase OS=Mus musculus OX=10090 GN=Gak PE=1 SV=2
ATP synthase subunit beta, mitochondrial OS=Mus musculus OX=10090 GN=Atp5f1b PE=1 SV=2
Microspherule protein 1 OS=Mus musculus OX=10090 GN=Mcrs1 PE=1 SV=1
Interferon-related developmental regulator 2 OS=Mus musculus OX=10090 GN=Ifrd2 PE=1 SV=1
TOM1-like protein 2 OS=Mus musculus OX=10090 GN=Tom1l2 PE=1 SV=1
Germinal-center associated nuclear protein OS=Mus musculus OX=10090 GN=Mcm3ap PE=1 SV=2
Periostin OS=Mus musculus OX=10090 GN=Postn PE=1 SV=2
Guanine nucleotide-binding protein G(i) subunit alpha-3 OS=Mus musculus OX=10090 GN=Gnai3 PE=1 SV=3
Actin-related protein 2/3 complex subunit 5-like protein OS=Mus musculus OX=10090 GN=Arpc5l PE=1 SV=1
7SK snRNA methylphosphate capping enzyme OS=Mus musculus OX=10090 GN=Mepce PE=1 SV=2
Protein AATF OS=Mus musculus OX=10090 GN=Aatf PE=1 SV=1
Meiosis regulator and mRNA stability factor 1 OS=Mus musculus OX=10090 GN=Marf1 PE=1 SV=3
Serine/arginine-rich splicing factor 3 OS=Mus musculus OX=10090 GN=Srsf3 PE=1 SV=1
Leucine-rich repeat serine/threonine-protein kinase 1 OS=Mus musculus OX=10090 GN=Lrrk1 PE=1 SV=1
ETS domain-containing transcription factor ERF OS=Mus musculus OX=10090 GN=Erf PE=1 SV=1
Phosphoglucomutase-like protein 5 OS=Mus musculus OX=10090 GN=Pgm5 PE=1 SV=2
Sodium channel protein OS=Mus musculus OX=10090 GN=Scn7a PE=1 SV=1
DNA-directed RNA polymerases I and III subunit RPAC1 OS=Mus musculus OX=10090 GN=Polr1c PE=1 SV=1
UPF0688 protein C1orf174 homolog OS=Mus musculus OX=10090 PE=1 SV=1
RNA-binding protein PNO1 OS=Mus musculus OX=10090 GN=Pno1 PE=1 SV=1
Small nuclear ribonucleoprotein Sm D1 OS=Mus musculus OX=10090 GN=Snrpd1 PE=1 SV=1
Transmembrane protein 39B OS=Mus musculus OX=10090 GN=Tmem39b PE=1 SV=1
ATP synthase subunit delta, mitochondrial OS=Mus musculus OX=10090 GN=Atp5f1d PE=1 SV=1
Transcription factor 7 OS=Mus musculus OX=10090 GN=Tcf7 PE=1 SV=2
Splicing factor U2AF 65 kDa subunit OS=Mus musculus OX=10090 GN=U2af2 PE=1 SV=3
Lamin-B1 OS=Mus musculus OX=10090 GN=Lmnb1 PE=1 SV=3
Chloride channel protein OS=Mus musculus OX=10090 GN=Clcn7 PE=1 SV=1
Epidermal growth factor-like protein 7 OS=Mus musculus OX=10090 GN=Egfl7 PE=2 SV=2
40S ribosomal protein S4, X isoform OS=Mus musculus OX=10090 GN=Rps4x PE=1 SV=2
Immunoglobulin-binding protein 1 OS=Mus musculus OX=10090 GN=Igbp1 PE=1 SV=1
RAS protein activator-like-3 OS=Mus musculus OX=10090 GN=Rasal3 PE=1 SV=1
Annexin A3 OS=Mus musculus OX=10090 GN=Anxa3 PE=1 SV=4
Asporin OS=Mus musculus OX=10090 GN=Aspn PE=1 SV=1
RNA cytosine C(5)-methyltransferase NSUN2 OS=Mus musculus OX=10090 GN=Nsun2 PE=1 SV=2

Gamma-soluble NSF attachment protein OS=Mus musculus OX=10090 GN=Napg PE=1 SV=1
Myosin-14 OS=Mus musculus OX=10090 GN=Myh14 PE=1 SV=1
PCF11 cleavage and polyadenylation factor subunit OS=Mus musculus OX=10090 GN=Pcf11 PE=1 SV=1
ER membrane protein complex subunit 2 OS=Mus musculus OX=10090 GN=Emc2 PE=1 SV=1
CCN family member 1 OS=Mus musculus OX=10090 GN=Ccn1 PE=1 SV=1
Ribosomal RNA-processing protein 7 homolog A OS=Mus musculus OX=10090 GN=Rrp7a PE=2 SV=1
ATP-citrate synthase OS=Mus musculus OX=10090 GN=Acly PE=1 SV=1
Lethal(3)malignant brain tumor-like protein 2 OS=Mus musculus OX=10090 GN=L3mbtl2 PE=1 SV=1
Kinesin-like protein KIF3A OS=Mus musculus OX=10090 GN=Kif3a PE=1 SV=2
Galectin-9 OS=Mus musculus OX=10090 GN=Lgals9 PE=1 SV=1
Mothers against decapentaplegic homolog 3 OS=Mus musculus OX=10090 GN=Smad3 PE=1 SV=2
Peptide-N(4)-(N-acetyl-beta-glucosaminyl)asparagine amidase OS=Mus musculus OX=10090 GN=Ngly1 PE=1 SV
DENN/MADD domain-containing 4A OS=Mus musculus OX=10090 GN=Dennd4a PE=1 SV=1
E3 ubiquitin-protein ligase UBR4 OS=Mus musculus OX=10090 GN=Ubr4 PE=1 SV=1
Interleukin enhancer-binding factor 2 OS=Mus musculus OX=10090 GN=Ilf2 PE=1 SV=1
Bone marrow proteoglycan OS=Mus musculus OX=10090 GN=Prg2 PE=1 SV=1
Collagen alpha-1(VI) chain OS=Mus musculus OX=10090 GN=Col6a1 PE=1 SV=1
SWI/SNF-related matrix-associated actin-dependent regulator of chromatin subfamily D member 2 OS=Mus mus
Rho GTPase-activating protein 35 OS=Mus musculus OX=10090 GN=Arhgap35 PE=1 SV=3
26S proteasome AAA-ATPase subunit RPT3 OS=Mus musculus OX=10090 GN=Psmc4 PE=1 SV=1
E3 ubiquitin-protein ligase Midline-1 OS=Mus musculus OX=10090 GN=Mid1 PE=1 SV=2
ADP-ribosylation factor 6 OS=Mus musculus OX=10090 GN=Arf6 PE=1 SV=2
Transcription elongation regulator 1 OS=Mus musculus OX=10090 GN=Tcerg1 PE=1 SV=2
Sterol carrier protein 2 OS=Mus musculus OX=10090 GN=Scp2 PE=1 SV=3
Unconventional myosin-6 OS=Mus musculus OX=10090 GN=Myo6 PE=1 SV=1
Pleckstrin homology domain-containing, family A member 5 (Fragment) OS=Mus musculus OX=10090 GN=Plekha
HBS1-like protein OS=Mus musculus OX=10090 GN=Hbs1l PE=1 SV=1
Band 4.1-like protein 5 OS=Mus musculus OX=10090 GN=Epb41l5 PE=1 SV=1
CDP-diacylglycerol--glycerol-3-phosphate 3-phosphatidyltransferase, mitochondrial OS=Mus musculus OX=10090
RNA-binding protein 47 OS=Mus musculus OX=10090 GN=Rbm47 PE=1 SV=1
Target of Myb protein 1 OS=Mus musculus OX=10090 GN=Tom1 PE=1 SV=1
Afadin OS=Mus musculus OX=10090 GN=Afdn PE=1 SV=1
Forkhead box protein P1 OS=Mus musculus OX=10090 GN=Foxp1 PE=1 SV=1
Tyrosine-protein kinase ITK/TSK OS=Mus musculus OX=10090 GN=Itk PE=1 SV=1
Thioredoxin OS=Mus musculus OX=10090 GN=Txn PE=1 SV=3
Ribonuclease P protein subunit p38 OS=Mus musculus OX=10090 GN=Rpp38 PE=1 SV=1
FYVE and coiled-coil domain-containing protein 1 OS=Mus musculus OX=10090 GN=Fyco1 PE=1 SV=1
GON-4-like protein OS=Mus musculus OX=10090 GN=Gon4l PE=1 SV=1
Spliceosome RNA helicase Ddx39b OS=Mus musculus OX=10090 GN=Ddx39b PE=1 SV=1
E3 ubiquitin-protein ligase OS=Mus musculus OX=10090 GN=Nedd4l PE=1 SV=1
Cysteine desulfurase, mitochondrial OS=Mus musculus OX=10090 GN=Nfs1 PE=1 SV=3
BAG family molecular chaperone regulator 2 OS=Mus musculus OX=10090 GN=Bag2 PE=1 SV=1
Kinesin-like protein KIFC1 OS=Mus musculus OX=10090 GN=Kifc1 PE=1 SV=2
S1 RNA-binding domain-containing protein 1 OS=Mus musculus OX=10090 GN=Srbd1 PE=2 SV=3
Pyruvate kinase OS=Mus musculus OX=10090 GN=Pklr PE=1 SV=1

E3 UFM1-protein ligase 1 OS=Mus musculus OX=10090 GN=Ufl1 PE=1 SV=2
BEN domain-containing protein 7 OS=Mus musculus OX=10090 GN=Bend7 PE=1 SV=1
Phosphatidylinositol 3-kinase catalytic subunit type 3 OS=Mus musculus OX=10090 GN=Pik3c3 PE=1 SV=1
DNA-(apurinic or apyrimidinic site) endonuclease (Fragment) OS=Mus musculus OX=10090 GN=Apex1 PE=1 SV=[illegible]
PCNA-associated factor OS=Mus musculus OX=10090 GN=Pclaf PE=1 SV=1
Protein kinase C zeta type OS=Mus musculus OX=10090 GN=Prkcz PE=1 SV=2
Pre-mRNA-splicing factor ATP-dependent RNA helicase DHX15 OS=Mus musculus OX=10090 GN=Dhx15 PE=1 SV[illegible]
Neutral cholesterol ester hydrolase 1 OS=Mus musculus OX=10090 GN=Nceh1 PE=1 SV=1
Poly [ADP-ribose] polymerase tankyrase-1 OS=Mus musculus OX=10090 GN=Tnks PE=1 SV=1
F-actin-capping protein subunit alpha OS=Mus musculus OX=10090 GN=Capza1 PE=1 SV=1
Zinc finger and BTB domain-containing protein 45 OS=Mus musculus OX=10090 GN=Zbtb45 PE=2 SV=1
Protein Niban 2 OS=Mus musculus OX=10090 GN=Niban2 PE=1 SV=2
TBC1 domain family member 4 OS=Mus musculus OX=10090 GN=Tbc1d4 PE=1 SV=1
Putative ATP-dependent RNA helicase DHX57 OS=Mus musculus OX=10090 GN=Dhx57 PE=1 SV=2
Unconventional myosin-IXa OS=Mus musculus OX=10090 GN=Myo9a PE=1 SV=1
Coatomer subunit beta' OS=Mus musculus OX=10090 GN=Copb2 PE=1 SV=2
Histone acetyltransferase KAT8 OS=Mus musculus OX=10090 GN=Kat8 PE=1 SV=1
Unconventional myosin-IXb OS=Mus musculus OX=10090 GN=Myo9b PE=1 SV=1
60S ribosomal protein L12 OS=Mus musculus OX=10090 GN=Rpl12 PE=1 SV=2
Kinesin light chain 4 OS=Mus musculus OX=10090 GN=Klc4 PE=1 SV=1
Lon protease homolog, mitochondrial OS=Mus musculus OX=10090 GN=Lonp1 PE=1 SV=2
Unconventional myosin-XVIIIa OS=Mus musculus OX=10090 GN=Myo18a PE=1 SV=2
Argininosuccinate synthase OS=Mus musculus OX=10090 GN=Ass1 PE=1 SV=1
T-complex protein 1 subunit alpha OS=Mus musculus OX=10090 GN=Tcp1 PE=1 SV=3
Partitioning defective 6 homolog beta OS=Mus musculus OX=10090 GN=Pard6b PE=1 SV=2
Myotubularin-related protein 12 OS=Mus musculus OX=10090 GN=Mtmr12 PE=1 SV=1
TFG protein OS=Mus musculus OX=10090 GN=Tfg PE=1 SV=1
Carboxylesterase 1D OS=Mus musculus OX=10090 GN=Ces1d PE=1 SV=1
Zinc finger RNA-binding protein OS=Mus musculus OX=10090 GN=Zfr PE=1 SV=2
Ribosomal protein S6 kinase alpha-4 OS=Mus musculus OX=10090 GN=Rps6ka4 PE=1 SV=2
Retinoic acid-induced protein 3 OS=Mus musculus OX=10090 GN=Gprc5a PE=1 SV=1
Phosphatidylinositol 3,4,5-trisphosphate-dependent Rac exchanger 2 protein OS=Mus musculus OX=10090 GN=P[illegible]
Ena/VASP-like protein (Fragment) OS=Mus musculus OX=10090 GN=Evl PE=1 SV=1
Family with sequence similarity 98, member C OS=Mus musculus OX=10090 GN=Fam98c PE=1 SV=1
Zinc finger CCCH-type antiviral protein 1 OS=Mus musculus OX=10090 GN=Zc3hav1 PE=1 SV=1
Transcription factor Sp4 OS=Mus musculus OX=10090 GN=Sp4 PE=1 SV=2
Hypermethylated in cancer 1 protein OS=Mus musculus OX=10090 GN=Hic1 PE=1 SV=1
Zinc finger protein 24 OS=Mus musculus OX=10090 GN=Znf24 PE=1 SV=1
Ras-related protein Rab-6A OS=Mus musculus OX=10090 GN=Rab6a PE=1 SV=4
Alpha-catulin OS=Mus musculus OX=10090 GN=Ctnnal1 PE=1 SV=1
Jun dimerization protein 2 OS=Mus musculus OX=10090 GN=Jdp2 PE=1 SV=2
Integrator complex subunit 6 OS=Mus musculus OX=10090 GN=Ints6 PE=1 SV=1
Diacylglycerol O-acyltransferase 1 OS=Mus musculus OX=10090 GN=Dgat1 PE=1 SV=1
Protein DGCR6 OS=Mus musculus OX=10090 GN=Dgcr6 PE=1 SV=1
Probable 28S rRNA (cytosine-C(5))-methyltransferase OS=Mus musculus OX=10090 GN=Nop2 PE=1 SV=1

Prostaglandin E synthase OS=Mus musculus OX=10090 GN=Ptges PE=1 SV=1
Protein VAC14 homolog OS=Mus musculus OX=10090 GN=Vac14 PE=1 SV=1
Dehydrogenase/reductase SDR family member 7B OS=Mus musculus OX=10090 GN=Dhrs7b PE=1 SV=1
Receptor expression-enhancing protein 5 OS=Mus musculus OX=10090 GN=Reep5 PE=1 SV=1
Dual-specificity mitogen-activated protein kinase kinase 2 OS=Mus musculus OX=10090 GN=Map2k2 PE=1 SV=1
HECT-type E3 ubiquitin transferase OS=Mus musculus OX=10090 GN=Ube3b PE=1 SV=1
Galectin-3 OS=Mus musculus OX=10090 GN=Lgals3 PE=1 SV=3
ABRA C-terminal-like protein (Fragment) OS=Mus musculus OX=10090 GN=Abracl PE=1 SV=1
Dihydropyrimidinase-related protein 3 OS=Mus musculus OX=10090 GN=Dpysl3 PE=1 SV=1
Complement factor H OS=Mus musculus OX=10090 GN=Cfh PE=1 SV=2
THAP domain-containing protein 11 OS=Mus musculus OX=10090 GN=Thap11 PE=1 SV=1
Aspartate--tRNA ligase, cytoplasmic OS=Mus musculus OX=10090 GN=Dars1 PE=1 SV=2
Transcription initiation factor TFIID subunit 6 OS=Mus musculus OX=10090 GN=Taf6 PE=1 SV=1
Serine/threonine-protein kinase 16 OS=Mus musculus OX=10090 GN=Stk16 PE=1 SV=1
Laminin subunit beta-1 OS=Mus musculus OX=10090 GN=Lamb1 PE=1 SV=1
Elongation factor 1-delta (Fragment) OS=Mus musculus OX=10090 GN=Eef1d PE=1 SV=1
Nuclear factor 1 OS=Mus musculus OX=10090 GN=Nfia PE=1 SV=1
Prolyl 3-hydroxylase 1 OS=Mus musculus OX=10090 GN=P3h1 PE=1 SV=2
Leucine-rich repeat-containing protein 59 OS=Mus musculus OX=10090 GN=Lrrc59 PE=1 SV=1
AP-1 complex subunit sigma-1A (Fragment) OS=Mus musculus OX=10090 GN=Ap1s1 PE=1 SV=1
WD repeat and coiled-coil-containing protein OS=Mus musculus OX=10090 GN=Wdcp PE=1 SV=1
Transcription factor A, mitochondrial OS=Mus musculus OX=10090 GN=Tfam PE=1 SV=1
Tropomyosin alpha-3 chain OS=Mus musculus OX=10090 GN=Tpm3 PE=1 SV=1
Eukaryotic translation initiation factor 3 subunit K OS=Mus musculus OX=10090 GN=Eif3k PE=1 SV=1
Dedicator of cytokinesis protein 11 OS=Mus musculus OX=10090 GN=Dock11 PE=1 SV=1
Zinc finger BED domain-containing protein 6 OS=Mus musculus OX=10090 GN=Zbed6 PE=1 SV=1
Coronin-1A OS=Mus musculus OX=10090 GN=Coro1a PE=1 SV=5
RuvB-like 2 OS=Mus musculus OX=10090 GN=Ruvbl2 PE=1 SV=3
Neuroguidin OS=Mus musculus OX=10090 GN=Ngdn PE=1 SV=1
Ubiquitin-like modifier-activating enzyme 6 OS=Mus musculus OX=10090 GN=Uba6 PE=1 SV=1
Coatomer subunit beta OS=Mus musculus OX=10090 GN=Copb1 PE=1 SV=1
Immunoglobulin kappa constant OS=Mus musculus OX=10090 GN=Igkc PE=1 SV=2
Beta-globin OS=Mus musculus OX=10090 GN=Hbb-bs PE=1 SV=1
Enhancer of filamentation 1 OS=Mus musculus OX=10090 GN=Nedd9 PE=1 SV=1
Serine/threonine-protein phosphatase PP1-gamma catalytic subunit OS=Mus musculus OX=10090 GN=Ppp1cc PE
Adiponectin (Fragment) OS=Mus musculus OX=10090 GN=Adipoq PE=1 SV=1
2'-5'-oligoadenylate synthase-like protein 1 OS=Mus musculus OX=10090 GN=Oasl1 PE=2 SV=1
Beta-1,4-galactosyltransferase 7 (Fragment) OS=Mus musculus OX=10090 GN=B4galt7 PE=1 SV=1
F-box only protein 38 OS=Mus musculus OX=10090 GN=Fbxo38 PE=1 SV=1
Very-long-chain enoyl-CoA reductase (Fragment) OS=Mus musculus OX=10090 GN=Tecr PE=1 SV=1
Importin-9 OS=Mus musculus OX=10090 GN=Ipo9 PE=1 SV=1
Cathepsin B OS=Mus musculus OX=10090 GN=Ctsb PE=1 SV=2
Rho GTPase-activating protein 42 OS=Mus musculus OX=10090 GN=Arhgap42 PE=1 SV=1
V-type proton ATPase subunit E 1 OS=Mus musculus OX=10090 GN=Atp6v1e1 PE=1 SV=2
Two pore calcium channel protein 1 OS=Mus musculus OX=10090 GN=Tpcn1 PE=1 SV=1

Centrosomal protein of 131 kDa OS=Mus musculus OX=10090 GN=Cep131 PE=1 SV=2
DNA-directed RNA polymerase II subunit RPB9 OS=Mus musculus OX=10090 GN=Polr2i PE=1 SV=1
UV excision repair protein RAD23 homolog B OS=Mus musculus OX=10090 GN=Rad23b PE=1 SV=2
Retinal dehydrogenase 1 OS=Mus musculus OX=10090 GN=Aldh1a1 PE=1 SV=5
26S proteasome non-ATPase regulatory subunit 3 OS=Mus musculus OX=10090 GN=Psmd3 PE=1 SV=3
Proteasome adapter and scaffold protein ECM29 OS=Mus musculus OX=10090 GN=Ecpas PE=1 SV=1
2-5A-dependent ribonuclease (Fragment) OS=Mus musculus OX=10090 GN=Rnasel PE=1 SV=1
rRNA-processing protein FCF1 homolog OS=Mus musculus OX=10090 GN=Fcf1 PE=2 SV=2
Protein phosphatase 1 regulatory subunit 21 OS=Mus musculus OX=10090 GN=Ppp1r21 PE=1 SV=2
Protein-glutamine gamma-glutamyltransferase 2 OS=Mus musculus OX=10090 GN=Tgm2 PE=1 SV=4
E3 ubiquitin-protein ligase TRAF7 OS=Mus musculus OX=10090 GN=Traf7 PE=1 SV=1
Heat shock-related 70 kDa protein 2 OS=Mus musculus OX=10090 GN=Hspa2 PE=1 SV=2
Succinate--CoA ligase [ADP-forming] subunit beta, mitochondrial OS=Mus musculus OX=10090 GN=Sucla2 PE=1
Pleckstrin homology-like domain family A member 3 OS=Mus musculus OX=10090 GN=Phlda3 PE=1 SV=1
Neurobeachin (Fragment) OS=Mus musculus OX=10090 GN=Nbea PE=1 SV=1
Amine oxidase [flavin-containing] B OS=Mus musculus OX=10090 GN=Maob PE=1 SV=4
POC1 centriolar protein homolog A OS=Mus musculus OX=10090 GN=Poc1a PE=1 SV=1
Capping protein, Arp2/3 and myosin-I linker protein 2 OS=Mus musculus OX=10090 GN=Carmil2 PE=1 SV=1
Arf-GAP with Rho-GAP domain, ANK repeat and PH domain-containing protein 1 OS=Mus musculus OX=10090 G
Mitochondrial Rho GTPase 1 OS=Mus musculus OX=10090 GN=Rhot1 PE=1 SV=1
Fibronectin OS=Mus musculus OX=10090 GN=Fn1 PE=1 SV=1
PRKR-interacting protein 1 OS=Mus musculus OX=10090 GN=Prkrip1 PE=1 SV=2
Vacuolar protein sorting-associated protein 8 homolog OS=Mus musculus OX=10090 GN=Vps8 PE=1 SV=1
Galectin-3-binding protein OS=Mus musculus OX=10090 GN=Lgals3bp PE=1 SV=1
Uridine-cytidine kinase-like 1 OS=Mus musculus OX=10090 GN=Uckl1 PE=1 SV=1
Zinc finger protein 593 OS=Mus musculus OX=10090 GN=Znf593 PE=1 SV=2
Polyprenol reductase OS=Mus musculus OX=10090 GN=Srd5a3 PE=1 SV=2
Ubiquitin carboxyl-terminal hydrolase BAP1 OS=Mus musculus OX=10090 GN=Bap1 PE=1 SV=1
60S ribosomal protein L29 OS=Mus musculus OX=10090 GN=Rpl29 PE=1 SV=2
Tyrosine-protein phosphatase non-receptor type 2 OS=Mus musculus OX=10090 GN=Ptpn2 PE=1 SV=2
CCR4-NOT transcription complex subunit 2 OS=Mus musculus OX=10090 GN=Cnot2 PE=1 SV=1
40S ribosomal protein S26 OS=Mus musculus OX=10090 GN=Rps26 PE=1 SV=3
Cadherin-13 OS=Mus musculus OX=10090 GN=Cdh13 PE=1 SV=2
Fermitin family homolog 3 OS=Mus musculus OX=10090 GN=Fermt3 PE=1 SV=1
Delta(24)-sterol reductase OS=Mus musculus OX=10090 GN=Dhcr24 PE=1 SV=1
WD repeat-containing protein 20 OS=Mus musculus OX=10090 GN=Wdr20 PE=1 SV=1
Cell cycle progression protein 1 OS=Mus musculus OX=10090 GN=Ccpg1 PE=1 SV=1
Alpha-enolase OS=Mus musculus OX=10090 GN=Eno1 PE=1 SV=3
rRNA 2'-O-methyltransferase fibrillarin OS=Mus musculus OX=10090 GN=Fbl PE=1 SV=2
Fermitin family homolog 2 OS=Mus musculus OX=10090 GN=Fermt2 PE=1 SV=1
Aldehyde dehydrogenase, mitochondrial OS=Mus musculus OX=10090 GN=Aldh2 PE=1 SV=1
Ubiquitin carboxyl-terminal hydrolase MINDY-1 OS=Mus musculus OX=10090 GN=Mindy1 PE=1 SV=1
Histone-lysine N-methyltransferase, H3 lysine-36-specific OS=Mus musculus OX=10090 GN=Nsd1 PE=1 SV=1
Cyclin-dependent kinase 12 OS=Mus musculus OX=10090 GN=Cdk12 PE=1 SV=2
Elongator complex protein 1 OS=Mus musculus OX=10090 GN=Elp1 PE=1 SV=2

Ras GTPase-activating protein-binding protein 2 OS=Mus musculus OX=10090 GN=G3bp2 PE=1 SV=2
Cytosolic endo-beta-N-acetylglucosaminidase OS=Mus musculus OX=10090 GN=Engase PE=1 SV=1
60S ribosomal protein L10 (Fragment) OS=Mus musculus OX=10090 GN=Rpl10 PE=1 SV=1
Ferroptosis suppressor protein 1 OS=Mus musculus OX=10090 GN=Aifm2 PE=1 SV=1
Phosphoinositide phospholipase C OS=Mus musculus OX=10090 GN=Plcd1 PE=1 SV=1
Protein FAM83B OS=Mus musculus OX=10090 GN=Fam83b PE=2 SV=1
Nucleolar protein 16 OS=Mus musculus OX=10090 GN=Nop16 PE=1 SV=1
Interferon-induced very large GTPase 1 OS=Mus musculus OX=10090 GN=Gvin1 PE=1 SV=1
Alpha-1,3/1,6-mannosyltransferase ALG2 OS=Mus musculus OX=10090 GN=Alg2 PE=1 SV=2
Histone acetyltransferase KAT7 OS=Mus musculus OX=10090 GN=Kat7 PE=1 SV=1
Elongation factor Tu, mitochondrial OS=Mus musculus OX=10090 GN=Tufm PE=1 SV=1
Protein NYNRIN OS=Mus musculus OX=10090 GN=Nynrin PE=2 SV=2
Glutaminyl-peptide cyclotransferase-like protein OS=Mus musculus OX=10090 GN=Qpctl PE=1 SV=1
SLIT-ROBO Rho GTPase-activating protein 2 OS=Mus musculus OX=10090 GN=Srgap2 PE=1 SV=2
U6 snRNA-associated Sm-like protein LSm6 OS=Mus musculus OX=10090 GN=Lsm6 PE=1 SV=1
Guanine nucleotide exchange factor DBS OS=Mus musculus OX=10090 GN=Mcf2l PE=1 SV=1
Inactive histone-lysine N-methyltransferase 2E OS=Mus musculus OX=10090 GN=Kmt2e PE=1 SV=2
AA987161 protein OS=Mus musculus OX=10090 GN=Zfp729b PE=2 SV=2
Serine/threonine-protein phosphatase 2A 56 kDa regulatory subunit epsilon isoform OS=Mus musculus OX=1009
Huntingtin OS=Mus musculus OX=10090 GN=Htt PE=1 SV=1
Nuclear factor NF-kappa-B p105 subunit OS=Mus musculus OX=10090 GN=Nfkb1 PE=1 SV=2
Telomere zinc finger-associated protein OS=Mus musculus OX=10090 GN=Zbtb48 PE=1 SV=1
BCL2-associated athanogene 6 (Fragment) OS=Mus musculus OX=10090 GN=Bag6 PE=1 SV=1
Hormone-sensitive lipase OS=Mus musculus OX=10090 GN=Lipe PE=1 SV=1
Zinc finger protein 329 OS=Mus musculus OX=10090 GN=Znf329 PE=1 SV=2
ATP-dependent DNA/RNA helicase DHX36 OS=Mus musculus OX=10090 GN=Dhx36 PE=1 SV=2
Serine/threonine-protein kinase D3 OS=Mus musculus OX=10090 GN=Prkd3 PE=1 SV=1
Upstream stimulatory factor 1 OS=Mus musculus OX=10090 GN=Usf1 PE=1 SV=1
Tyrosine-protein kinase CSK OS=Mus musculus OX=10090 GN=Csk PE=1 SV=2
Multifunctional protein ADE2 OS=Mus musculus OX=10090 GN=Paics PE=1 SV=4
Tyrosine-protein kinase HCK OS=Mus musculus OX=10090 GN=Hck PE=1 SV=4
Pleckstrin homology domain-containing family O member 1 OS=Mus musculus OX=10090 GN=Plekho1 PE=1 SV=
PHD finger protein 6 OS=Mus musculus OX=10090 GN=Phf6 PE=1 SV=1
Elongation factor 2 OS=Mus musculus OX=10090 GN=Eef2 PE=1 SV=2
Programmed cell death protein 6 OS=Mus musculus OX=10090 GN=Pdcd6 PE=1 SV=2
Tissue-type plasminogen activator OS=Mus musculus OX=10090 GN=Plat PE=1 SV=3
Signal peptide, CUB and EGF-like domain-containing protein 1 OS=Mus musculus OX=10090 GN=Scube1 PE=2 SV
Cellular nucleic acid-binding protein OS=Mus musculus OX=10090 GN=Cnbp PE=1 SV=1
Telomere-associated protein RIF1 OS=Mus musculus OX=10090 GN=Rif1 PE=1 SV=2
RNA-binding protein EWS OS=Mus musculus OX=10090 GN=Ewsr1 PE=1 SV=1
Ubiquitin-associated protein 2 OS=Mus musculus OX=10090 GN=Ubap2 PE=1 SV=1
Type 2 phosphatidylinositol 4,5-bisphosphate 4-phosphatase OS=Mus musculus OX=10090 GN=Pip4p2 PE=1 SV=
Zinc finger protein 407 OS=Mus musculus OX=10090 GN=Zfp407 PE=1 SV=1
Atlastin-3 OS=Mus musculus OX=10090 GN=Atl3 PE=1 SV=1
Protein O-glucosyltransferase 2 OS=Mus musculus OX=10090 GN=Poglut2 PE=1 SV=1

Non-receptor tyrosine-protein kinase TYK2 OS=Mus musculus OX=10090 GN=Tyk2 PE=1 SV=3
CXXC motif containing zinc binding protein OS=Mus musculus OX=10090 GN=Czib PE=1 SV=1
Interleukin enhancer-binding factor 3 OS=Mus musculus OX=10090 GN=Ilf3 PE=1 SV=1
Interferon-induced 35 kDa protein homolog OS=Mus musculus OX=10090 GN=Ifi35 PE=1 SV=3
ATP-binding cassette sub-family B member 10, mitochondrial OS=Mus musculus OX=10090 GN=Abcb10 PE=1 SV
Myosin-binding protein C, cardiac-type OS=Mus musculus OX=10090 GN=Mybpc3 PE=1 SV=1
Non-specific serine/threonine protein kinase OS=Mus musculus OX=10090 GN=Cdc42bpa PE=1 SV=1
RNA-binding motif protein, X-linked 2 OS=Mus musculus OX=10090 GN=Rbmx2 PE=1 SV=1
Microsomal signal peptidase 25 kDa subunit OS=Mus musculus OX=10090 GN=Spcs2 PE=1 SV=1
Zinc finger ZZ-type and EF-hand domain-containing protein 1 OS=Mus musculus OX=10090 GN=Zzef1 PE=1 SV=2
Zinc finger protein 512B OS=Mus musculus OX=10090 GN=Zfp512b PE=1 SV=1
Vesicle-fusing ATPase OS=Mus musculus OX=10090 GN=Nsf PE=1 SV=2
Zinc finger protein 772 OS=Mus musculus OX=10090 GN=Zfp772 PE=2 SV=1
GTPase Era, mitochondrial OS=Mus musculus OX=10090 GN=Eral1 PE=2 SV=1
Breast cancer anti-estrogen resistance protein 1 OS=Mus musculus OX=10090 GN=Bcar1 PE=1 SV=2
Bromodomain-containing protein 1 OS=Mus musculus OX=10090 GN=Brd1 PE=1 SV=1
60S ribosomal protein L18a OS=Mus musculus OX=10090 GN=Rpl18a PE=1 SV=1
Glutathione S-transferase Mu 1 OS=Mus musculus OX=10090 GN=Gstm1 PE=1 SV=2
V-type proton ATPase catalytic subunit A OS=Mus musculus OX=10090 GN=Atp6v1a PE=1 SV=2
Collagen alpha-1(XII) chain OS=Mus musculus OX=10090 GN=Col12a1 PE=2 SV=3
Cullin-3 OS=Mus musculus OX=10090 GN=Cul3 PE=1 SV=1
von Willebrand factor A domain-containing protein 5A OS=Mus musculus OX=10090 GN=Vwa5a PE=1 SV=2
Coenzyme Q10A OS=Mus musculus OX=10090 GN=Coq10a PE=1 SV=1
40S ribosomal protein S23 OS=Mus musculus OX=10090 GN=Rps23 PE=1 SV=3
Protein kinase C theta type OS=Mus musculus OX=10090 GN=Prkcq PE=1 SV=1
Liprin-beta-2 OS=Mus musculus OX=10090 GN=Ppfibp2 PE=1 SV=1
SPT3, SAGA and STAGA complex component OS=Mus musculus OX=10090 GN=Supt3 PE=1 SV=1
Adhesion G protein-coupled receptor A3 OS=Mus musculus OX=10090 GN=Adgra3 PE=2 SV=3
Obscurin-like protein 1 OS=Mus musculus OX=10090 GN=Obsl1 PE=1 SV=1
60S ribosomal protein L34 OS=Mus musculus OX=10090 GN=Rpl34 PE=1 SV=2
Peroxisomal 2,4-dienoyl-CoA reductase [(3E)-enoyl-CoA-producing] OS=Mus musculus OX=10090 GN=Decr2 PE=1
Acidic leucine-rich nuclear phosphoprotein 32 family member B OS=Mus musculus OX=10090 GN=Anp32b PE=1
L-lactate dehydrogenase OS=Mus musculus OX=10090 GN=Ldha PE=1 SV=1
Nephronectin OS=Mus musculus OX=10090 GN=Npnt PE=1 SV=1
Zinc finger FYVE domain-containing protein 21 OS=Mus musculus OX=10090 GN=Zfyve21 PE=1 SV=1
Phospholipid scramblase 1 OS=Mus musculus OX=10090 GN=Plscr1 PE=1 SV=1
F-actin-capping protein subunit alpha-2 OS=Mus musculus OX=10090 GN=Capza2 PE=1 SV=3
Cytoplasmic dynein 1 light intermediate chain 1 OS=Mus musculus OX=10090 GN=Dync1li1 PE=1 SV=1
ATP-dependent RNA helicase DDX1 OS=Mus musculus OX=10090 GN=Ddx1 PE=1 SV=1
DnaJ homolog subfamily C member 8 OS=Mus musculus OX=10090 GN=Dnajc8 PE=1 SV=2
BRISC complex subunit Abraxas 2 (Fragment) OS=Mus musculus OX=10090 GN=Abraxas2 PE=1 SV=2
Phosphatase and actin regulator 1 OS=Mus musculus OX=10090 GN=Phactr1 PE=1 SV=1
Probable ATP-dependent RNA helicase DHX40 OS=Mus musculus OX=10090 GN=Dhx40 PE=1 SV=1
Hyccin OS=Mus musculus OX=10090 GN=Fam126a PE=1 SV=3
Glioblastoma amplified sequence OS=Mus musculus OX=10090 GN=Nipsnap2 PE=1 SV=1

Golgi-associated PDZ and coiled-coil motif-containing protein OS=Mus musculus OX=10090 GN=Gopc PE=1 SV=1
Serine/threonine-protein kinase PAK 4 OS=Mus musculus OX=10090 GN=Pak4 PE=1 SV=1
Active regulator of SIRT1 OS=Mus musculus OX=10090 GN=Rps19bp1 PE=1 SV=1
Kielin/chordin-like protein OS=Mus musculus OX=10090 GN=Kcp PE=1 SV=2
Trafficking protein particle complex subunit 12 OS=Mus musculus OX=10090 GN=Trappc12 PE=1 SV=2
Progranulin OS=Mus musculus OX=10090 GN=Grn PE=1 SV=2
DnaJ homolog subfamily C member 1 OS=Mus musculus OX=10090 GN=Dnajc1 PE=1 SV=1
Phospholipase B-like OS=Mus musculus OX=10090 GN=Plbd1 PE=1 SV=1
NADH dehydrogenase [ubiquinone] 1 alpha subcomplex subunit 13 OS=Mus musculus OX=10090 GN=Ndufa13 PE
Protein Niban 1 OS=Mus musculus OX=10090 GN=Niban1 PE=1 SV=2
Adenylate cyclase type 4 OS=Mus musculus OX=10090 GN=Adcy4 PE=1 SV=1
Ankyrin repeat domain-containing protein 13A OS=Mus musculus OX=10090 GN=Ankrd13a PE=1 SV=2
Zinc finger protein 62 (Fragment) OS=Mus musculus OX=10090 GN=Zfp62 PE=1 SV=1
Phosphatidate phosphatase OS=Mus musculus OX=10090 GN=Lpin3 PE=1 SV=1
Zinc finger CCCH type-containing 7B OS=Mus musculus OX=10090 GN=Zc3h7b PE=1 SV=1
60S ribosomal protein L24 OS=Mus musculus OX=10090 GN=Rpl24 PE=1 SV=2
ATP-dependent RNA helicase DDX54 OS=Mus musculus OX=10090 GN=Ddx54 PE=1 SV=1
Prolargin OS=Mus musculus OX=10090 GN=Prelp PE=1 SV=2
ADP/ATP translocase 1 OS=Mus musculus OX=10090 GN=Slc25a4 PE=1 SV=4
Speckle targeted PIP5K1A-regulated poly(A) polymerase OS=Mus musculus OX=10090 GN=Tut1 PE=1 SV=1
40S ribosomal protein S30 OS=Mus musculus OX=10090 GN=Fau PE=1 SV=1
Epidermal growth factor receptor kinase substrate 8-like protein 2 OS=Mus musculus OX=10090 GN=Eps8l2 PE=1
5'-nucleotidase OS=Mus musculus OX=10090 GN=Nt5e PE=1 SV=2
F-actin-capping protein subunit beta OS=Mus musculus OX=10090 GN=Capzb PE=1 SV=3
Tumor susceptibility gene 101 protein OS=Mus musculus OX=10090 GN=Tsg101 PE=1 SV=2
Olfactory receptor OS=Mus musculus OX=10090 GN=Olfr1113 PE=3 SV=1
Histone acetyltransferase p300 OS=Mus musculus OX=10090 GN=Ep300 PE=1 SV=2
Mitogen-activated protein kinase kinase kinase kinase 3 OS=Mus musculus OX=10090 GN=Map4k3 PE=1 SV=4
DEAH (Asp-Glu-Ala-His) box polypeptide 37 OS=Mus musculus OX=10090 GN=Dhx37 PE=1 SV=1
Zinc transporter 7 OS=Mus musculus OX=10090 GN=Slc30a7 PE=1 SV=1
Phospholipase A-2-activating protein OS=Mus musculus OX=10090 GN=Plaa PE=1 SV=4
Myosin-10 OS=Mus musculus OX=10090 GN=Myh10 PE=1 SV=1
E3 SUMO-protein ligase RanBP2 OS=Mus musculus OX=10090 GN=Ranbp2 PE=1 SV=2
Solute carrier family 12 member 6 OS=Mus musculus OX=10090 GN=Slc12a6 PE=1 SV=2
Indolethylamine N-methyltransferase OS=Mus musculus OX=10090 GN=Inmt PE=1 SV=1
Ribosome-binding protein 1 OS=Mus musculus OX=10090 GN=Rrbp1 PE=1 SV=1
Stromal cell-derived factor 2-like protein 1 OS=Mus musculus OX=10090 GN=Sdf2l1 PE=1 SV=2
Ensconsin OS=Mus musculus OX=10090 GN=Map7 PE=1 SV=3
Vam6/Vps39-like protein OS=Mus musculus OX=10090 GN=Vps39 PE=1 SV=1
Dynein light intermediate chain OS=Mus musculus OX=10090 GN=Dync1li2 PE=1 SV=1
Spermatogenesis-defective protein 39 homolog OS=Mus musculus OX=10090 GN=Vipas39 PE=1 SV=1
RNA-binding protein 42 OS=Mus musculus OX=10090 GN=Rbm42 PE=1 SV=2
DnaJ homolog subfamily C member 16 OS=Mus musculus OX=10090 GN=Dnajc16 PE=1 SV=2
Zinc finger CCCH domain-containing protein 11A OS=Mus musculus OX=10090 GN=Zc3h11a PE=1 SV=1
Ligand-dependent corepressor OS=Mus musculus OX=10090 GN=Lcor PE=1 SV=1

PDZ domain-containing protein 11 OS=Mus musculus OX=10090 GN=Pdzd11 PE=1 SV=1
MAP/microtubule affinity-regulating kinase 4 OS=Mus musculus OX=10090 GN=Mark4 PE=1 SV=1
Serine/threonine-protein phosphatase 6 regulatory subunit 1 OS=Mus musculus OX=10090 GN=Ppp6r1 PE=1 SV=
H-2 class I histocompatibility antigen, K-B alpha chain OS=Mus musculus OX=10090 GN=H2-K1 PE=1 SV=1
Laminin subunit beta-2 OS=Mus musculus OX=10090 GN=Lamb2 PE=1 SV=2
Bcl-2-associated transcription factor 1 OS=Mus musculus OX=10090 GN=Bclaf1 PE=1 SV=2
UPF0488 protein C8orf33 homolog OS=Mus musculus OX=10090 PE=2 SV=1
Asparagine--tRNA ligase OS=Mus musculus OX=10090 GN=Nars PE=1 SV=1
ADAMTS-like protein 4 OS=Mus musculus OX=10090 GN=Adamtsl4 PE=2 SV=1
Leucine zipper protein 1 OS=Mus musculus OX=10090 GN=Luzp1 PE=1 SV=2
Signal transducer and activator of transcription OS=Mus musculus OX=10090 GN=Stat1 PE=1 SV=1
Very-long-chain (3R)-3-hydroxyacyl-CoA dehydratase 3 OS=Mus musculus OX=10090 GN=Hacd3 PE=1 SV=2
U4/U6 small nuclear ribonucleoprotein Prp31 OS=Mus musculus OX=10090 GN=Prpf31 PE=1 SV=3
GTP-binding protein SAR1a OS=Mus musculus OX=10090 GN=Sar1a PE=1 SV=1
Ubiquitin carboxyl-terminal hydrolase 7 OS=Mus musculus OX=10090 GN=Usp7 PE=1 SV=1
Endophilin-A2 OS=Mus musculus OX=10090 GN=Sh3gl1 PE=1 SV=1
Phosphatidate cytidylyltransferase, mitochondrial OS=Mus musculus OX=10090 GN=Tamm41 PE=1 SV=1
FERM domain-containing protein 8 OS=Mus musculus OX=10090 GN=Frmd8 PE=1 SV=2
MAP7 domain-containing protein 1 OS=Mus musculus OX=10090 GN=Map7d1 PE=1 SV=1
Protein RCC2 OS=Mus musculus OX=10090 GN=Rcc2 PE=1 SV=1
SAM and SH3 domain-containing protein 1 OS=Mus musculus OX=10090 GN=Sash1 PE=1 SV=1
Chaperonin containing Tcp1, subunit 6a (Zeta) OS=Mus musculus OX=10090 GN=Cct6a PE=2 SV=1
Dynamin GTPase OS=Mus musculus OX=10090 GN=Dnm3 PE=1 SV=1
Glycogen synthase kinase-3 beta OS=Mus musculus OX=10090 GN=Gsk3b PE=1 SV=1
T-complex protein 1 subunit delta OS=Mus musculus OX=10090 GN=Cct4 PE=1 SV=3
Actin-related protein 2/3 complex subunit 4 OS=Mus musculus OX=10090 GN=Arpc4 PE=1 SV=3
Tyrosine-protein kinase Fer OS=Mus musculus OX=10090 GN=Fer PE=1 SV=2
Actin-related protein 2 OS=Mus musculus OX=10090 GN=Actr2 PE=1 SV=1
Round spermatid basic protein 1-like OS=Mus musculus OX=10090 GN=Rsbn1l PE=1 SV=1
Polycystin-2 OS=Mus musculus OX=10090 GN=Pkd2 PE=1 SV=2
Armadillo repeat-containing X-linked protein 3 OS=Mus musculus OX=10090 GN=Armcx3 PE=1 SV=1
PTB domain-containing engulfment adapter protein 1 OS=Mus musculus OX=10090 GN=Gulp1 PE=1 SV=1
CTTNBP2 N-terminal-like protein OS=Mus musculus OX=10090 GN=Cttnbp2nl PE=1 SV=1
Protein tyrosine phosphatase type IVA 3 OS=Mus musculus OX=10090 GN=Ptp4a3 PE=1 SV=1
SWI/SNF complex subunit SMARCC2 OS=Mus musculus OX=10090 GN=Smarcc2 PE=1 SV=1
Testican-2 OS=Mus musculus OX=10090 GN=Spock2 PE=1 SV=1
Immunity-related GTPase family M protein 1 OS=Mus musculus OX=10090 GN=Irgm1 PE=1 SV=1
Oxysterol-binding protein OS=Mus musculus OX=10090 GN=Osbpl7 PE=1 SV=2
Calmodulin-2 OS=Mus musculus OX=10090 GN=Calm2 PE=1 SV=1
Insulin-degrading enzyme OS=Mus musculus OX=10090 GN=Ide PE=1 SV=1
Troponin T, cardiac muscle OS=Mus musculus OX=10090 GN=Tnnt2 PE=1 SV=1
A disintegrin-like and metallopeptidase (reprolysin type) with thrombospondin type 1 motif, 9 OS=Mus musculus
KN motif and ankyrin repeat domain-containing protein 4 OS=Mus musculus OX=10090 GN=Kank4 PE=1 SV=1
Non-specific serine/threonine protein kinase OS=Mus musculus OX=10090 GN=Trp53rkb PE=1 SV=1
Bromodomain PHD finger transcription factor OS=Mus musculus OX=10090 GN=Bptf PE=1 SV=1

Isocitrate dehydrogenase [NADP], mitochondrial OS=Mus musculus OX=10090 GN=Idh2 PE=1 SV=3
Importin subunit beta-1 OS=Mus musculus OX=10090 GN=Kpnb1 PE=1 SV=2
SEC14 domain and spectrin repeat-containing protein 1 OS=Mus musculus OX=10090 GN=Sestd1 PE=1 SV=1
Protein phosphatase 1, regulatory subunit 3D OS=Mus musculus OX=10090 GN=Ppp1r3d PE=1 SV=1
Eukaryotic translation initiation factor 5B OS=Mus musculus OX=10090 GN=Eif5b PE=1 SV=2
Coatomer subunit epsilon OS=Mus musculus OX=10090 GN=Cope PE=1 SV=3
U3 small nucleolar RNA-associated protein 14 homolog B OS=Mus musculus OX=10090 GN=Utp14b PE=1 SV=1
Ras-related protein R-Ras2 OS=Mus musculus OX=10090 GN=Rras2 PE=1 SV=1
ATP-dependent RNA helicase DDX51 OS=Mus musculus OX=10090 GN=Ddx51 PE=1 SV=1
Rap guanine nucleotide exchange factor 5 OS=Mus musculus OX=10090 GN=Rapgef5 PE=2 SV=2
Sarcoplasmic/endoplasmic reticulum calcium ATPase 2 OS=Mus musculus OX=10090 GN=Atp2a2 PE=1 SV=2
Active breakpoint cluster region-related protein OS=Mus musculus OX=10090 GN=Abr PE=1 SV=1
1-phosphatidylinositol 4,5-bisphosphate phosphodiesterase beta-3 OS=Mus musculus OX=10090 GN=Plcb3 PE=1
Protein arginine N-methyltransferase 1 OS=Mus musculus OX=10090 GN=Prmt1 PE=1 SV=1
Torsin-4A OS=Mus musculus OX=10090 GN=Tor4a PE=2 SV=1
MKI67 FHA domain-interacting nucleolar phosphoprotein OS=Mus musculus OX=10090 GN=Nifk PE=1 SV=1
Cullin-4A OS=Mus musculus OX=10090 GN=Cul4a PE=1 SV=1
F-box only protein 11 OS=Mus musculus OX=10090 GN=Fbxo11 PE=1 SV=3
40S ribosomal protein S20 OS=Mus musculus OX=10090 GN=Rps20 PE=1 SV=1
CWF19-like protein 2 OS=Mus musculus OX=10090 GN=Cwf19l2 PE=1 SV=1
Leucine-rich repeat-containing protein 1 OS=Mus musculus OX=10090 GN=Lrrc1 PE=1 SV=2
Myotubularin-related protein 13 OS=Mus musculus OX=10090 GN=Sbf2 PE=1 SV=1
ADP-ribosylation factor-like protein 1 OS=Mus musculus OX=10090 GN=Arl1 PE=1 SV=1
Nuclear autoantigen Sp-100 (Fragment) OS=Mus musculus OX=10090 GN=Sp100 PE=1 SV=1
Guanylate cyclase soluble subunit beta-1 OS=Mus musculus OX=10090 GN=Gucy1b1 PE=1 SV=1
Activating signal cointegrator 1 complex subunit 3 OS=Mus musculus OX=10090 GN=Ascc3 PE=1 SV=1
Lysozyme C-1 OS=Mus musculus OX=10090 GN=Lyz1 PE=1 SV=1
Ubiquitin-like modifier-activating enzyme 1 OS=Mus musculus OX=10090 GN=Uba1 PE=1 SV=1
Dolichyl-diphosphooligosaccharide--protein glycosyltransferase 48 kDa subunit OS=Mus musculus OX=10090 GN=
Kinesin-like protein OS=Mus musculus OX=10090 GN=Kif9 PE=1 SV=1
Raftlin OS=Mus musculus OX=10090 GN=Rftn1 PE=1 SV=4
Trafficking protein particle complex subunit 9 OS=Mus musculus OX=10090 GN=Trappc9 PE=1 SV=2
Coatomer subunit gamma-2 OS=Mus musculus OX=10090 GN=Copg2 PE=1 SV=1
Inositol monophosphatase 2 OS=Mus musculus OX=10090 GN=Impa2 PE=1 SV=1
Complex I subunit B13 OS=Mus musculus OX=10090 GN=Ndufa5 PE=1 SV=1
Nucleus accumbens-associated protein 2 OS=Mus musculus OX=10090 GN=Nacc2 PE=2 SV=1
Very long-chain specific acyl-CoA dehydrogenase, mitochondrial OS=Mus musculus OX=10090 GN=Acadvl PE=1 S
IgE-binding protein OS=Mus musculus OX=10090 GN=Iap PE=2 SV=1
Terminal nucleotidyltransferase 4B OS=Mus musculus OX=10090 GN=Tent4b PE=1 SV=1
T-complex protein 1 subunit theta OS=Mus musculus OX=10090 GN=Cct8 PE=1 SV=3
Phosphatidylinositol-binding clathrin assembly protein OS=Mus musculus OX=10090 GN=Picalm PE=1 SV=1
SNARE-associated protein Snapin OS=Mus musculus OX=10090 GN=Snapin PE=1 SV=1
Apoptosis-stimulating of p53 protein 2 OS=Mus musculus OX=10090 GN=Tp53bp2 PE=1 SV=3
AP-3 complex subunit sigma-1 OS=Mus musculus OX=10090 GN=Ap3s1 PE=1 SV=2
Cingulin OS=Mus musculus OX=10090 GN=Cgn PE=1 SV=2

tRNA (guanine-N(7)-)-methyltransferase OS=Mus musculus OX=10090 GN=Mettl1 PE=1 SV=1
Protein-lysine 6-oxidase OS=Mus musculus OX=10090 GN=Lox PE=1 SV=1
C-Jun-amino-terminal kinase-interacting protein 4 OS=Mus musculus OX=10090 GN=Spag9 PE=1 SV=2
IQ motif and SEC7 domain-containing protein 1 OS=Mus musculus OX=10090 GN=Iqsec1 PE=1 SV=1
Ras-related protein Rap-1b OS=Mus musculus OX=10090 GN=Rap1b PE=1 SV=2
Short coiled-coil protein OS=Mus musculus OX=10090 GN=Scoc PE=1 SV=1
Replication initiator 1 OS=Mus musculus OX=10090 GN=Repin1 PE=1 SV=1
Delta(14)-sterol reductase LBR OS=Mus musculus OX=10090 GN=Lbr PE=1 SV=2
Protocadherin 1 (Fragment) OS=Mus musculus OX=10090 GN=Pcdh1 PE=1 SV=1
Junctophilin-2 OS=Mus musculus OX=10090 GN=Jph2 PE=1 SV=2
Rho GTPase-activating protein 1 OS=Mus musculus OX=10090 GN=Arhgap1 PE=1 SV=1
Interferon-induced, double-stranded RNA-activated protein kinase OS=Mus musculus OX=10090 GN=Eif2ak2 PE=
[F-actin]-monooxygenase MICAL2 OS=Mus musculus OX=10090 GN=Mical2 PE=1 SV=1
Sec24-related gene family, member C (S. cerevisiae) OS=Mus musculus OX=10090 GN=Sec24c PE=1 SV=1
Unconventional myosin-Ib OS=Mus musculus OX=10090 GN=Myo1b PE=1 SV=3
Exportin-1 OS=Mus musculus OX=10090 GN=Xpo1 PE=1 SV=1
Calcium-binding mitochondrial carrier protein SCaMC-1 OS=Mus musculus OX=10090 GN=Slc25a24 PE=1 SV=1
GMP synthase [glutamine-hydrolyzing] OS=Mus musculus OX=10090 GN=Gmps PE=1 SV=2
INO80 complex subunit B OS=Mus musculus OX=10090 GN=Ino80b PE=1 SV=1
Poly(A) RNA polymerase, mitochondrial (Fragment) OS=Mus musculus OX=10090 GN=Mtpap PE=1 SV=1
WD repeat-containing protein 55 OS=Mus musculus OX=10090 GN=Wdr55 PE=1 SV=2
Protein kinase C and casein kinase substrate in neurons protein 2 OS=Mus musculus OX=10090 GN=Pacsin2 PE=1
Ataxin-2 (Fragment) OS=Mus musculus OX=10090 GN=Atxn2 PE=1 SV=2
Zinc finger with KRAB and SCAN domains 8 OS=Mus musculus OX=10090 GN=Zkscan8 PE=1 SV=1
E3 ubiquitin-protein ligase RBX1 OS=Mus musculus OX=10090 GN=Rbx1 PE=1 SV=1
Forkhead box protein K1 OS=Mus musculus OX=10090 GN=Foxk1 PE=1 SV=2
40S ribosomal protein S6 OS=Mus musculus OX=10090 GN=Rps6 PE=1 SV=1
Leucine-rich repeat-containing protein 58 OS=Mus musculus OX=10090 GN=Lrrc58 PE=2 SV=1
Midasin OS=Mus musculus OX=10090 GN=Mdn1 PE=1 SV=1
E3 ubiquitin-protein ligase RNF138 OS=Mus musculus OX=10090 GN=Rnf138 PE=2 SV=1
Spondin-1 OS=Mus musculus OX=10090 GN=Spon1 PE=1 SV=1
Zinc finger protein 622 OS=Mus musculus OX=10090 GN=Znf622 PE=1 SV=1
STING ER exit protein OS=Mus musculus OX=10090 GN=Steep1 PE=1 SV=1
Coatomer subunit alpha OS=Mus musculus OX=10090 GN=Copa PE=1 SV=2
Phosphatidate phosphatase LPIN2 OS=Mus musculus OX=10090 GN=Lpin2 PE=1 SV=2
UDP-glucose:glycoprotein glucosyltransferase 1 OS=Mus musculus OX=10090 GN=Uggt1 PE=1 SV=4
NHL repeat-containing protein 2 OS=Mus musculus OX=10090 GN=Nhlrc2 PE=1 SV=1
CD151 antigen OS=Mus musculus OX=10090 GN=Cd151 PE=1 SV=2
POZ (BTB) and AT hook-containing zinc finger 1 OS=Mus musculus OX=10090 GN=Patz1 PE=1 SV=1
SH3 domain-binding glutamic acid-rich-like protein OS=Mus musculus OX=10090 GN=Sh3bgrl PE=1 SV=1
Rho GTPase-activating protein 25 OS=Mus musculus OX=10090 GN=Arhgap25 PE=1 SV=2
Ras-related C3 botulinum toxin substrate 2 OS=Mus musculus OX=10090 GN=Rac2 PE=1 SV=1
4F2 cell-surface antigen heavy chain OS=Mus musculus OX=10090 GN=Slc3a2 PE=1 SV=1
Myosin VC OS=Mus musculus OX=10090 GN=Myo5c PE=1 SV=1
Serine/threonine-protein kinase VRK1 OS=Mus musculus OX=10090 GN=Vrk1 PE=1 SV=1

Activating signal cointegrator 1 OS=Mus musculus OX=10090 GN=Trip4 PE=1 SV=2
Nuclear protein localization protein 4 homolog OS=Mus musculus OX=10090 GN=Nploc4 PE=1 SV=3
CMP-N-acetylneuraminic acid synthase OS=Mus musculus OX=10090 GN=Cmas PE=1 SV=1
Prostacyclin synthase OS=Mus musculus OX=10090 GN=Ptgis PE=1 SV=1
Unconventional myosin-Ic OS=Mus musculus OX=10090 GN=Myo1c PE=1 SV=2
DDB1- and CUL4-associated factor 10 OS=Mus musculus OX=10090 GN=Dcaf10 PE=1 SV=1
Transcriptional coactivator YAP1 OS=Mus musculus OX=10090 GN=Yap1 PE=1 SV=1
Ras-related protein R-Ras OS=Mus musculus OX=10090 GN=Rras PE=1 SV=1
Transcription elongation factor A N-terminal and central domain-containing protein 2 OS=Mus musculus OX=100
Zinc finger protein 276 OS=Mus musculus OX=10090 GN=Znf276 PE=1 SV=3
RuvB-like 1 OS=Mus musculus OX=10090 GN=Ruvbl1 PE=1 SV=1
ATP-dependent 6-phosphofructokinase, liver type OS=Mus musculus OX=10090 GN=Pfkl PE=1 SV=4
Laminin subunit beta-3 OS=Mus musculus OX=10090 GN=Lamb3 PE=1 SV=2
Dolichol-phosphate mannosyltransferase subunit 1 OS=Mus musculus OX=10090 GN=Dpm1 PE=1 SV=1
USP6 N-terminal-like protein OS=Mus musculus OX=10090 GN=Usp6nl PE=1 SV=2
Cathepsin S OS=Mus musculus OX=10090 GN=Ctss PE=1 SV=1
Ribosomal protein S6 kinase beta-1 OS=Mus musculus OX=10090 GN=Rps6kb1 PE=1 SV=2
40S ribosomal protein S11 OS=Mus musculus OX=10090 GN=Rps11 PE=1 SV=3
C2 domain-containing protein 2 OS=Mus musculus OX=10090 GN=C2cd2 PE=1 SV=1
Kinesin family member 13B OS=Mus musculus OX=10090 GN=Kif13b PE=1 SV=1
PC4 and SFRS1-interacting protein OS=Mus musculus OX=10090 GN=Psip1 PE=1 SV=1
Src-like-adapter OS=Mus musculus OX=10090 GN=Sla PE=1 SV=3
Protein MON2 homolog OS=Mus musculus OX=10090 GN=Mon2 PE=1 SV=2
2,4-dienoyl-CoA reductase [(3E)-enoyl-CoA-producing], mitochondrial OS=Mus musculus OX=10090 GN=Decr1 PE
Vigilin OS=Mus musculus OX=10090 GN=Hdlbp PE=1 SV=1
WW domain-binding protein 11 OS=Mus musculus OX=10090 GN=Wbp11 PE=1 SV=2
LanC-like protein 2 (Fragment) OS=Mus musculus OX=10090 GN=Lancl2 PE=1 SV=1
Lipoprotein lipase OS=Mus musculus OX=10090 GN=Lpl PE=1 SV=3
Unconventional myosin-Ib OS=Mus musculus OX=10090 GN=Myo1b PE=1 SV=1
CTP synthase 1 OS=Mus musculus OX=10090 GN=Ctps1 PE=1 SV=2
Fibulin-5 OS=Mus musculus OX=10090 GN=Fbln5 PE=1 SV=1
Guanine nucleotide-binding protein G(I)/G(S)/G(T) subunit beta-2 OS=Mus musculus OX=10090 GN=Gnb2 PE=1
Intersectin-2 OS=Mus musculus OX=10090 GN=Itsn2 PE=1 SV=2
39S ribosomal protein L3, mitochondrial OS=Mus musculus OX=10090 GN=Mrpl3 PE=1 SV=3
Replication factor C subunit 1 OS=Mus musculus OX=10090 GN=Rfc1 PE=1 SV=2
Cytochrome P450 2B19 OS=Mus musculus OX=10090 GN=Cyp2b19 PE=2 SV=1
Putative E3 ubiquitin-protein ligase UNKL OS=Mus musculus OX=10090 GN=Unkl PE=2 SV=2
Cohesin subunit SA-2 OS=Mus musculus OX=10090 GN=Stag2 PE=1 SV=3
Synaptojanin-1 OS=Mus musculus OX=10090 GN=Synj1 PE=1 SV=3
Mitotic spindle assembly checkpoint protein MAD2A OS=Mus musculus OX=10090 GN=Mad2l1 PE=1 SV=2
Myotubularin-related protein 1 OS=Mus musculus OX=10090 GN=Mtmr1 PE=1 SV=1
Transcription intermediary factor 1-beta OS=Mus musculus OX=10090 GN=Trim28 PE=1 SV=3
Methyltransferase-like 7A1 OS=Mus musculus OX=10090 GN=Mettl7a1 PE=1 SV=1
Pyrin domain-containing protein 3 OS=Mus musculus OX=10090 GN=Pydc3 PE=2 SV=1
Metal-response element-binding transcription factor 2 OS=Mus musculus OX=10090 GN=Mtf2 PE=1 SV=1

Coatomer subunit gamma-1 OS=Mus musculus OX=10090 GN=Copg1 PE=1 SV=1
39S ribosomal protein L37, mitochondrial OS=Mus musculus OX=10090 GN=Mrpl37 PE=1 SV=1
GTPase, IMAP family member 7 OS=Mus musculus OX=10090 GN=Gimap7 PE=1 SV=1
U3 small nucleolar RNA-interacting protein 2 OS=Mus musculus OX=10090 GN=Rrp9 PE=1 SV=1
ADP/ATP translocase 2 OS=Mus musculus OX=10090 GN=Slc25a5 PE=1 SV=3
Glutaredoxin-3 (Fragment) OS=Mus musculus OX=10090 GN=Glrx3 PE=1 SV=1
Adenylosuccinate lyase OS=Mus musculus OX=10090 GN=Adsl PE=1 SV=2
Serine/threonine-protein phosphatase 2A 55 kDa regulatory subunit B OS=Mus musculus OX=10090 GN=Ppp2r2a
Death-associated protein kinase 3 OS=Mus musculus OX=10090 GN=Dapk3 PE=1 SV=1
Male-specific lethal 1 homolog OS=Mus musculus OX=10090 GN=Msl1 PE=1 SV=1
Protein SON OS=Mus musculus OX=10090 GN=Son PE=1 SV=1
Anaphase-promoting complex subunit 4 OS=Mus musculus OX=10090 GN=Anapc4 PE=1 SV=1
UPF0711 protein C18orf21 homolog OS=Mus musculus OX=10090 PE=2 SV=2
Rho GTPase-activating protein 29 OS=Mus musculus OX=10090 GN=Arhgap29 PE=1 SV=1
Y-box-binding protein 3 OS=Mus musculus OX=10090 GN=Ybx3 PE=1 SV=2
40S ribosomal protein S27-like OS=Mus musculus OX=10090 GN=Rps27l PE=1 SV=3
DnaJ homolog subfamily C member 2 OS=Mus musculus OX=10090 GN=Dnajc2 PE=1 SV=1
60S ribosomal protein L17 OS=Mus musculus OX=10090 GN=Rpl17 PE=1 SV=3
28S ribosomal protein S7, mitochondrial OS=Mus musculus OX=10090 GN=Mrps7 PE=1 SV=1
Protein polybromo-1 OS=Mus musculus OX=10090 GN=Pbrm1 PE=1 SV=4
Vacuolar protein sorting-associated protein 45 OS=Mus musculus OX=10090 GN=Vps45 PE=1 SV=1
Ubiquitinyl hydrolase 1 OS=Mus musculus OX=10090 GN=Otub1 PE=1 SV=1
Formin-like protein 1 OS=Mus musculus OX=10090 GN=Fmnl1 PE=1 SV=1
COP9 signalosome complex subunit 5 OS=Mus musculus OX=10090 GN=Cops5 PE=1 SV=3
ADP-ribosylation factor 4 OS=Mus musculus OX=10090 GN=Arf4 PE=1 SV=2
Calpain small subunit 1 OS=Mus musculus OX=10090 GN=Capns1 PE=1 SV=1
Prefoldin subunit 1 (Fragment) OS=Mus musculus OX=10090 GN=Pfdn1 PE=1 SV=1
RIKEN cDNA 4930522L14 gene OS=Mus musculus OX=10090 GN=4930522L14Rik PE=1 SV=1
Calcium-binding mitochondrial carrier protein Aralar2 OS=Mus musculus OX=10090 GN=Slc25a13 PE=1 SV=1
Integral membrane protein 2B OS=Mus musculus OX=10090 GN=Itm2b PE=1 SV=1
Estradiol 17-beta-dehydrogenase 11 OS=Mus musculus OX=10090 GN=Hsd17b11 PE=1 SV=1
Integrin beta-1 OS=Mus musculus OX=10090 GN=Itgb1 PE=1 SV=1
Ran-binding protein 6 OS=Mus musculus OX=10090 GN=Ranbp6 PE=1 SV=3
Synaptobrevin homolog YKT6 OS=Mus musculus OX=10090 GN=Ykt6 PE=1 SV=1
DDB1- and CUL4-associated factor 8 OS=Mus musculus OX=10090 GN=Dcaf8 PE=1 SV=1
RalBP1-associated Eps domain-containing protein 1 (Fragment) OS=Mus musculus OX=10090 GN=Reps1 PE=1 S
PDZ and LIM domain protein 2 OS=Mus musculus OX=10090 GN=Pdlim2 PE=1 SV=1
EF-hand calcium-binding domain-containing protein 4A OS=Mus musculus OX=10090 GN=Cracr2b PE=2 SV=2
DNA mismatch repair protein Mlh1 OS=Mus musculus OX=10090 GN=Mlh1 PE=1 SV=2
Tripartite motif-containing protein 16 OS=Mus musculus OX=10090 GN=Trim16 PE=1 SV=2
Prostaglandin reductase-3 OS=Mus musculus OX=10090 GN=Zadh2 PE=1 SV=1
Ras GTPase-activating-like protein IQGAP2 OS=Mus musculus OX=10090 GN=Iqgap2 PE=1 SV=2
Heterogeneous nuclear ribonucleoprotein H OS=Mus musculus OX=10090 GN=Hnrnph1 PE=1 SV=1
Integrin alpha-1 OS=Mus musculus OX=10090 GN=Itga1 PE=1 SV=2
Cytosolic non-specific dipeptidase OS=Mus musculus OX=10090 GN=Cndp2 PE=1 SV=1

Macrophage mannose receptor 1 OS=Mus musculus OX=10090 GN=Mrc1 PE=1 SV=2
Matrix-remodeling-associated protein 7 OS=Mus musculus OX=10090 GN=Mxra7 PE=1 SV=2
Tyrosine-protein kinase Lyn OS=Mus musculus OX=10090 GN=Lyn PE=1 SV=4
26S proteasome regulatory subunit 8 OS=Mus musculus OX=10090 GN=Psmc5 PE=1 SV=1
Transcription initiation factor TFIID subunit OS=Mus musculus OX=10090 GN=Taf1 PE=1 SV=1
PDZ domain-containing protein GIPC1 OS=Mus musculus OX=10090 GN=Gipc1 PE=1 SV=1
Proteasome subunit beta type-3 OS=Mus musculus OX=10090 GN=Psmb3 PE=1 SV=1
SPARC-related modular calcium-binding protein 2 OS=Mus musculus OX=10090 GN=Smoc2 PE=1 SV=1
26S proteasome non-ATPase regulatory subunit 11 OS=Mus musculus OX=10090 GN=Psmd11 PE=1 SV=3
ATP-dependent RNA helicase DDX42 OS=Mus musculus OX=10090 GN=Ddx42 PE=1 SV=3
39S ribosomal protein L19, mitochondrial OS=Mus musculus OX=10090 GN=Mrpl19 PE=1 SV=1
Protein AMN1 homolog OS=Mus musculus OX=10090 GN=Amn1 PE=1 SV=1
Glycogen phosphorylase, brain form OS=Mus musculus OX=10090 GN=Pygb PE=1 SV=3
Staphylococcal nuclease domain-containing protein 1 OS=Mus musculus OX=10090 GN=Snd1 PE=1 SV=1
Arrestin domain-containing protein 1 OS=Mus musculus OX=10090 GN=Arrdc1 PE=1 SV=2
Protein argonaute-2 OS=Mus musculus OX=10090 GN=Ago2 PE=1 SV=3
Cyclin-dependent kinase-like 5 OS=Mus musculus OX=10090 GN=Cdkl5 PE=1 SV=1
MAX gene-associated protein OS=Mus musculus OX=10090 GN=Mga PE=1 SV=1
Testis-specific gene 10 protein OS=Mus musculus OX=10090 GN=Tsga10 PE=1 SV=1
WASH complex subunit 5 OS=Mus musculus OX=10090 GN=Washc5 PE=1 SV=2
Dimethylaniline monooxygenase [N-oxide-forming] 2 OS=Mus musculus OX=10090 GN=Fmo2 PE=1 SV=3
ATP synthase subunit e, mitochondrial OS=Mus musculus OX=10090 GN=Atp5me PE=1 SV=2
Zinc finger protein ZFAT OS=Mus musculus OX=10090 GN=Zfat PE=1 SV=1
Gbp6 protein OS=Mus musculus OX=10090 GN=Gbp7 PE=1 SV=2
Cytoskeleton-associated protein 5 OS=Mus musculus OX=10090 GN=Ckap5 PE=1 SV=1
Lupus La protein homolog OS=Mus musculus OX=10090 GN=Ssb PE=1 SV=1
Mitochondria-eating protein OS=Mus musculus OX=10090 GN=Spata18 PE=1 SV=2
Leukocyte receptor cluster member 8 homolog OS=Mus musculus OX=10090 GN=Leng8 PE=1 SV=1
Pyridoxal-dependent decarboxylase domain-containing protein 1 OS=Mus musculus OX=10090 GN=Pdxdc1 PE=1 S
Proliferation-associated protein 2G4 OS=Mus musculus OX=10090 GN=Pa2g4 PE=1 SV=3
Chromatin-remodeling ATPase INO80 OS=Mus musculus OX=10090 GN=Ino80 PE=1 SV=1
Casein kinase I isoform alpha OS=Mus musculus OX=10090 GN=Csnk1a1 PE=1 SV=1
AP-2 complex subunit alpha-1 OS=Mus musculus OX=10090 GN=Ap2a1 PE=1 SV=1
High mobility group nucleosome-binding domain-containing protein 5 OS=Mus musculus OX=10090 GN=Hmgn5 I
Protein kinase C delta type OS=Mus musculus OX=10090 GN=Prkcd PE=1 SV=3
Probable RNA polymerase II nuclear localization protein SLC7A6OS OS=Mus musculus OX=10090 GN=Slc7a6os PI
Septin-2 OS=Mus musculus OX=10090 GN=Septin2 PE=1 SV=2
Transcription initiation factor TFIID subunit 9B OS=Mus musculus OX=10090 GN=Taf9b PE=1 SV=1
Long-chain-fatty-acid--CoA ligase 5 OS=Mus musculus OX=10090 GN=Acsl5 PE=1 SV=1
Serine/threonine-protein kinase N3 OS=Mus musculus OX=10090 GN=Pkn3 PE=1 SV=1
Zinc finger protein 652 OS=Mus musculus OX=10090 GN=Znf652 PE=1 SV=2
Serine/threonine-protein kinase 17B OS=Mus musculus OX=10090 GN=Stk17b PE=1 SV=1
Ubiquitin-protein ligase E3C OS=Mus musculus OX=10090 GN=Ube3c PE=1 SV=2
Actin-related protein 2/3 complex subunit 2 OS=Mus musculus OX=10090 GN=Arpc2 PE=1 SV=3
Myosin regulatory light chain 2, atrial isoform OS=Mus musculus OX=10090 GN=Myl7 PE=1 SV=1

Short transient receptor potential channel 6 OS=Mus musculus OX=10090 GN=Trpc6 PE=1 SV=1
Occludin OS=Mus musculus OX=10090 GN=Ocln PE=1 SV=1
Vesicle-trafficking protein SEC22b OS=Mus musculus OX=10090 GN=Sec22b PE=1 SV=3
Nucleoporin 205 OS=Mus musculus OX=10090 GN=Nup205 PE=1 SV=1
Zinc finger protein 445 OS=Mus musculus OX=10090 GN=Znf445 PE=2 SV=2
RAF proto-oncogene serine/threonine-protein kinase OS=Mus musculus OX=10090 GN=Raf1 PE=1 SV=2
Ferritin OS=Mus musculus OX=10090 GN=Ftl1 PE=1 SV=1
Laminin subunit gamma-1 OS=Mus musculus OX=10090 GN=Lamc1 PE=1 SV=1
Cap-specific mRNA (nucleoside-2'-O-)-methyltransferase 1 OS=Mus musculus OX=10090 GN=Cmtr1 PE=1 SV=1
PHD finger protein 1 OS=Mus musculus OX=10090 GN=Phf1 PE=1 SV=1
Eukaryotic translation initiation factor 2 subunit 2 OS=Mus musculus OX=10090 GN=Eif2s2 PE=1 SV=1
Krueppel-like factor 4 OS=Mus musculus OX=10090 GN=Klf4 PE=1 SV=3
U1 small nuclear ribonucleoprotein A OS=Mus musculus OX=10090 GN=Snrpa PE=1 SV=3
Telomeric repeat-binding factor 2-interacting protein 1 OS=Mus musculus OX=10090 GN=Terf2ip PE=1 SV=1
E3 ubiquitin-protein ligase RNF213 OS=Mus musculus OX=10090 GN=Rnf213 PE=1 SV=1
Cell surface hyaluronidase OS=Mus musculus OX=10090 GN=Cemip2 PE=1 SV=1
COP9 signalosome complex subunit 6 OS=Mus musculus OX=10090 GN=Cops6 PE=1 SV=1
High mobility group protein 20A OS=Mus musculus OX=10090 GN=Hmg20a PE=1 SV=1
Myosin-11 OS=Mus musculus OX=10090 GN=Myh11 PE=1 SV=2
Mitogen-activated protein kinase kinase kinase kinase 4 OS=Mus musculus OX=10090 GN=Map4k4 PE=1 SV=1
UPF3 regulator of nonsense transcripts homolog B (yeast) OS=Mus musculus OX=10090 GN=Upf3b PE=1 SV=1
TNF receptor-associated factor 5 OS=Mus musculus OX=10090 GN=Traf5 PE=1 SV=1
Lysophospholipid acyltransferase 7 (Fragment) OS=Mus musculus OX=10090 GN=Mboat7 PE=1 SV=1
SHC-transforming protein 1 OS=Mus musculus OX=10090 GN=Shc1 PE=1 SV=3
Integrin-linked protein kinase OS=Mus musculus OX=10090 GN=Ilk PE=1 SV=2
S-methyl-5'-thioadenosine phosphorylase OS=Mus musculus OX=10090 GN=Mtap PE=1 SV=1
Spermatogenesis-associated protein 6 OS=Mus musculus OX=10090 GN=Spata6 PE=1 SV=2
Zinc finger protein 869 OS=Mus musculus OX=10090 GN=Zfp869 PE=1 SV=1
Ubiquitin-protein ligase E3A OS=Mus musculus OX=10090 GN=Ube3a PE=1 SV=2
39S ribosomal protein L15, mitochondrial OS=Mus musculus OX=10090 GN=Mrpl15 PE=1 SV=1
Phospholipase OS=Mus musculus OX=10090 GN=Pld1 PE=1 SV=1
Leucine-rich repeat-containing protein 14 OS=Mus musculus OX=10090 GN=Lrrc14 PE=1 SV=1
Major centromere autoantigen B OS=Mus musculus OX=10090 GN=Cenpb PE=2 SV=2
Ribosome biogenesis protein NOP53 OS=Mus musculus OX=10090 GN=Nop53 PE=1 SV=1
Vacuolar protein sorting-associated protein 37C (Fragment) OS=Mus musculus OX=10090 GN=Vps37c PE=1 SV=1
Patatin-like phospholipase domain-containing protein 6 OS=Mus musculus OX=10090 GN=Pnpla6 PE=1 SV=1
Epsin-1 OS=Mus musculus OX=10090 GN=Epn1 PE=1 SV=3
Tether containing UBX domain for GLUT4 OS=Mus musculus OX=10090 GN=Aspscr1 PE=1 SV=1
N-terminal kinase-like protein OS=Mus musculus OX=10090 GN=Scyl1 PE=1 SV=1
Tropomyosin beta chain OS=Mus musculus OX=10090 GN=Tpm2 PE=1 SV=1
Splicing factor U2AF 35 kDa subunit OS=Mus musculus OX=10090 GN=U2af1 PE=1 SV=4
Smoothelin OS=Mus musculus OX=10090 GN=Smtn PE=1 SV=1
FHF complex subunit HOOK interacting protein 2A OS=Mus musculus OX=10090 GN=Fhip2a PE=1 SV=2
Carbonyl reductase [NADPH] 1 OS=Mus musculus OX=10090 GN=Cbr1 PE=1 SV=3
Protein diaphanous homolog 1 OS=Mus musculus OX=10090 GN=Diaph1 PE=1 SV=1

GTP-binding protein Rheb OS=Mus musculus OX=10090 GN=Rheb PE=1 SV=1
SH3 domain-binding protein 4 OS=Mus musculus OX=10090 GN=Sh3bp4 PE=1 SV=1
DNA-binding protein Rfx5 OS=Mus musculus OX=10090 GN=Rfx5 PE=1 SV=2
Zinc finger protein 953 OS=Mus musculus OX=10090 GN=Zfp953 PE=1 SV=1
Nuclear receptor ROR-beta OS=Mus musculus OX=10090 GN=Rorb PE=1 SV=3
Guanine deaminase OS=Mus musculus OX=10090 GN=Gda PE=1 SV=1
Guanine nucleotide-binding protein-like 3-like protein OS=Mus musculus OX=10090 GN=Gnl3l PE=1 SV=1
Phosphatidylinositol 4-phosphate 3-kinase C2 domain-containing subunit alpha OS=Mus musculus OX=10090 GN
Putative ribosome-binding factor A, mitochondrial OS=Mus musculus OX=10090 GN=Rbfa PE=2 SV=1
RNA cytidine acetyltransferase OS=Mus musculus OX=10090 GN=Nat10 PE=1 SV=1
Septin-9 OS=Mus musculus OX=10090 GN=Septin9 PE=1 SV=1
Ubiquitin carboxyl-terminal hydrolase OS=Mus musculus OX=10090 GN=Usp3 PE=1 SV=1
High mobility group protein HMG-I/HMG-Y OS=Mus musculus OX=10090 GN=Hmga1 PE=1 SV=1
Ankyrin repeat and LEM domain-containing protein 2 OS=Mus musculus OX=10090 GN=Ankle2 PE=1 SV=2
Sister chromatid cohesion protein PDS5 homolog B OS=Mus musculus OX=10090 GN=Pds5b PE=1 SV=1
Coiled-coil domain-containing protein 85B OS=Mus musculus OX=10090 GN=Ccdc85b PE=1 SV=1
Guanylate cyclase soluble subunit alpha-1 OS=Mus musculus OX=10090 GN=Gucy1a1 PE=1 SV=2
NHP2-like protein 1 OS=Mus musculus OX=10090 GN=Snu13 PE=1 SV=4
Peroxiredoxin-6 OS=Mus musculus OX=10090 GN=Prdx6 PE=1 SV=1
Cytoplasmic FMR1-interacting protein OS=Mus musculus OX=10090 GN=Cyfip1 PE=1 SV=1
Probable ubiquitin carboxyl-terminal hydrolase MINDY-4 OS=Mus musculus OX=10090 GN=Mindy4 PE=1 SV=1
Nuclear factor 1 OS=Mus musculus OX=10090 GN=Nfib PE=1 SV=1
Transcription factor Sp3 OS=Mus musculus OX=10090 GN=Sp3 PE=1 SV=2
Ras-related protein Rab-27A OS=Mus musculus OX=10090 GN=Rab27a PE=1 SV=1
AP-3 complex subunit mu-1 OS=Mus musculus OX=10090 GN=Ap3m1 PE=1 SV=1
Guanine nucleotide-binding protein subunit gamma OS=Mus musculus OX=10090 GN=Gng12 PE=1 SV=1
[Protein ADP-ribosylarginine] hydrolase OS=Mus musculus OX=10090 GN=Adprh PE=1 SV=1
Reticulon-3 OS=Mus musculus OX=10090 GN=Rtn3 PE=1 SV=2
Oxysterol-binding protein-related protein 8 OS=Mus musculus OX=10090 GN=Osbpl8 PE=1 SV=1
LEM domain-containing protein 2 OS=Mus musculus OX=10090 GN=Lemd2 PE=1 SV=1
Midnolin (Fragment) OS=Mus musculus OX=10090 GN=Midn PE=4 SV=8
BolA-like protein 2 OS=Mus musculus OX=10090 GN=Bola2 PE=1 SV=1
RNA-binding protein FUS (Fragment) OS=Mus musculus OX=10090 GN=Fus PE=1 SV=1
Transitional endoplasmic reticulum ATPase OS=Mus musculus OX=10090 GN=Vcp PE=1 SV=4
Forkhead box protein O1 OS=Mus musculus OX=10090 GN=Foxo1 PE=1 SV=2
Unconventional myosin-XIX OS=Mus musculus OX=10090 GN=Myo19 PE=1 SV=1
Membrane-associated progesterone receptor component 2 OS=Mus musculus OX=10090 GN=Pgrmc2 PE=1 SV=2
Phosphatidate cytidylyltransferase 2 OS=Mus musculus OX=10090 GN=Cds2 PE=1 SV=1
Pikachurin OS=Mus musculus OX=10090 GN=Egflam PE=1 SV=1
Cytoplasmic protein NCK1 (Fragment) OS=Mus musculus OX=10090 GN=Nck1 PE=1 SV=1
Kinesin light chain OS=Mus musculus OX=10090 GN=Klc1 PE=1 SV=1
Kinectin OS=Mus musculus OX=10090 GN=Ktn1 PE=1 SV=1
Prostatic acid phosphatase OS=Mus musculus OX=10090 GN=Acp3 PE=1 SV=1
Rho-related GTP-binding protein RhoG OS=Mus musculus OX=10090 GN=Rhog PE=1 SV=1
14-3-3 protein eta OS=Mus musculus OX=10090 GN=Ywhah PE=1 SV=2

Anaphase-promoting complex subunit 5 OS=Mus musculus OX=10090 GN=Anapc5 PE=1 SV=1
Leucine-rich repeat flightless-interacting protein 1 OS=Mus musculus OX=10090 GN=Lrrfip1 PE=1 SV=2
Myotrophin OS=Mus musculus OX=10090 GN=Mtpn PE=1 SV=2
LYR motif-containing protein 4 OS=Mus musculus OX=10090 GN=Lyrm4 PE=1 SV=1
Vacuolar protein sorting-associated protein 26B OS=Mus musculus OX=10090 GN=Vps26b PE=1 SV=1
DNA helicase OS=Mus musculus OX=10090 GN=Recql4 PE=1 SV=1
WD repeat-containing protein 18 OS=Mus musculus OX=10090 GN=Wdr18 PE=1 SV=1
Ketosamine-3-kinase OS=Mus musculus OX=10090 GN=Fn3krp PE=1 SV=2
Macrophage immunometabolism regulator OS=Mus musculus OX=10090 GN=MACIR PE=2 SV=1
FRY-like transcription coactivator OS=Mus musculus OX=10090 GN=Fryl PE=1 SV=1
14-3-3 protein epsilon OS=Mus musculus OX=10090 GN=Ywhae PE=1 SV=1
Endonuclease domain-containing 1 protein OS=Mus musculus OX=10090 GN=Endod1 PE=1 SV=2
14-3-3 protein beta/alpha OS=Mus musculus OX=10090 GN=Ywhab PE=1 SV=3
Calpain-2 catalytic subunit OS=Mus musculus OX=10090 GN=Capn2 PE=1 SV=4
Nuclear receptor-binding protein (Fragment) OS=Mus musculus OX=10090 GN=Nrbp1 PE=1 SV=1
Protein FAM98B OS=Mus musculus OX=10090 GN=Fam98b PE=1 SV=1
Syntaxin-12 OS=Mus musculus OX=10090 GN=Stx12 PE=1 SV=1
Nucleolar protein 58 OS=Mus musculus OX=10090 GN=Nop58 PE=1 SV=1
Coiled-coil domain-containing protein 88B OS=Mus musculus OX=10090 GN=Ccdc88b PE=1 SV=2
DNA replication factor Cdt1 OS=Mus musculus OX=10090 GN=Cdt1 PE=1 SV=1
Glycerol-3-phosphate dehydrogenase, mitochondrial OS=Mus musculus OX=10090 GN=Gpd2 PE=1 SV=2
Ubiquitin-like protein ISG15 OS=Mus musculus OX=10090 GN=Isg15 PE=1 SV=4
[Pyruvate dehydrogenase (acetyl-transferring)] kinase isozyme 3, mitochondrial OS=Mus musculus OX=10090 GN
Protein ABHD11 OS=Mus musculus OX=10090 GN=Abhd11 PE=1 SV=1
Endoplasmic reticulum aminopeptidase 1 OS=Mus musculus OX=10090 GN=Erap1 PE=1 SV=2
Nischarin OS=Mus musculus OX=10090 GN=Nisch PE=1 SV=2
Protein-tyrosine-phosphatase OS=Mus musculus OX=10090 GN=Ptprm PE=1 SV=1
Homeobox protein Hox-B4 OS=Mus musculus OX=10090 GN=Hoxb4 PE=2 SV=1
Troponin I, cardiac muscle OS=Mus musculus OX=10090 GN=Tnni3 PE=1 SV=2
Ribosomal protein S6 kinase OS=Mus musculus OX=10090 GN=Rps6ka1 PE=1 SV=1
Prostaglandin F2 receptor negative regulator OS=Mus musculus OX=10090 GN=Ptgfrn PE=1 SV=2
Protein phosphatase 1F OS=Mus musculus OX=10090 GN=Ppm1f PE=1 SV=1
General transcription factor IIH subunit 1 OS=Mus musculus OX=10090 GN=Gtf2h1 PE=1 SV=1
Antiviral innate immune response receptor RIG-I OS=Mus musculus OX=10090 GN=Ddx58 PE=1 SV=2
Terminal uridylyltransferase 7 OS=Mus musculus OX=10090 GN=Tut7 PE=1 SV=3
Replication factor C subunit 5 OS=Mus musculus OX=10090 GN=Rfc5 PE=1 SV=1
Ribosomal RNA small subunit methyltransferase NEP1 OS=Mus musculus OX=10090 GN=Emg1 PE=1 SV=1
Hippocalcin-like protein 1 OS=Mus musculus OX=10090 GN=Hpcal1 PE=1 SV=2
Peroxisome biogenesis factor 10 OS=Mus musculus OX=10090 GN=Pex10 PE=2 SV=1
Ral guanine nucleotide dissociation stimulator-like 2 OS=Mus musculus OX=10090 GN=Rgl2 PE=1 SV=2
Zinc finger and BTB domain-containing 21 OS=Mus musculus OX=10090 GN=Zbtb21 PE=1 SV=1
Kinase OS=Mus musculus OX=10090 GN=Itpkb PE=1 SV=1
U3 small nucleolar RNA-associated protein 15 homolog OS=Mus musculus OX=10090 GN=Utp15 PE=1 SV=1
Heat shock protein beta-1 OS=Mus musculus OX=10090 GN=Hspb1 PE=1 SV=3
Ubiquitin carboxyl-terminal hydrolase 8 OS=Mus musculus OX=10090 GN=Usp8 PE=1 SV=1

Tubulin polymerization-promoting protein OS=Mus musculus OX=10090 GN=Tppp PE=1 SV=1
Copine-1 OS=Mus musculus OX=10090 GN=Cpne1 PE=1 SV=1
Leucine-rich repeat serine/threonine-protein kinase 2 OS=Mus musculus OX=10090 GN=Lrrk2 PE=1 SV=2
Signal transducer and activator of transcription 5B OS=Mus musculus OX=10090 GN=Stat5b PE=1 SV=1
Glycerol-3-phosphate dehydrogenase 1-like protein OS=Mus musculus OX=10090 GN=Gpd1l PE=1 SV=2
Probable ATP-dependent RNA helicase DDX56 OS=Mus musculus OX=10090 GN=Ddx56 PE=2 SV=1
Endothelial transcription factor GATA-2 OS=Mus musculus OX=10090 GN=Gata2 PE=1 SV=2
TNF receptor-associated factor 6 OS=Mus musculus OX=10090 GN=Traf6 PE=1 SV=2
Protein KRI1 homolog OS=Mus musculus OX=10090 GN=Kri1 PE=1 SV=2
Zinc finger protein 358 OS=Mus musculus OX=10090 GN=Zfp358 PE=1 SV=1
Delta-1-pyrroline-5-carboxylate dehydrogenase, mitochondrial OS=Mus musculus OX=10090 GN=Aldh4a1 PE=1 S
GPI mannosyltransferase 3 OS=Mus musculus OX=10090 GN=Pigb PE=2 SV=2
Formin-like protein 3 OS=Mus musculus OX=10090 GN=Fmnl3 PE=1 SV=2
Protein S100-A6 OS=Mus musculus OX=10090 GN=S100a6 PE=1 SV=3
Hsp90 co-chaperone Cdc37 OS=Mus musculus OX=10090 GN=Cdc37 PE=1 SV=1
Serine-protein kinase ATM OS=Mus musculus OX=10090 GN=Atm PE=1 SV=1
Serine/threonine-protein phosphatase 2A 56 kDa regulatory subunit beta isoform OS=Mus musculus OX=10090 G
PX domain-containing protein kinase-like protein OS=Mus musculus OX=10090 GN=Pxk PE=1 SV=2
Cullin-associated NEDD8-dissociated protein 1 OS=Mus musculus OX=10090 GN=Cand1 PE=1 SV=2
Uridine/cytidine kinase (Fragment) OS=Mus musculus OX=10090 GN=Uck1 PE=1 SV=1
RNA-binding protein 45 OS=Mus musculus OX=10090 GN=Rbm45 PE=1 SV=1
Receptor activity-modifying protein 2 (Fragment) OS=Mus musculus OX=10090 GN=Ramp2 PE=1 SV=1
E3 ubiquitin-protein ligase UBR2 OS=Mus musculus OX=10090 GN=Ubr2 PE=1 SV=2
Anoctamin-2 OS=Mus musculus OX=10090 GN=Ano2 PE=1 SV=2
Histone deacetylase 1 OS=Mus musculus OX=10090 GN=Hdac1 PE=1 SV=1
Inner nuclear membrane protein Man1 OS=Mus musculus OX=10090 GN=Lemd3 PE=1 SV=1
U3 small nucleolar ribonucleoprotein protein MPP10 OS=Mus musculus OX=10090 GN=Mphosph10 PE=1 SV=2
Threonylcarbamoyladenosine tRNA methylthiotransferase OS=Mus musculus OX=10090 GN=Cdkal1 PE=1 SV=1
HEAT repeat-containing protein 3 OS=Mus musculus OX=10090 GN=Heatr3 PE=1 SV=1
Glycylpeptide N-tetradecanoyltransferase 1 OS=Mus musculus OX=10090 GN=Nmt1 PE=1 SV=1
DEAH box protein 9 OS=Mus musculus OX=10090 GN=Dhx9 PE=1 SV=1
NF-kappa-B-activating protein OS=Mus musculus OX=10090 GN=Nkap PE=2 SV=1
GRB2-related adapter protein OS=Mus musculus OX=10090 GN=Grap PE=1 SV=1
Transducin beta-like protein 3 OS=Mus musculus OX=10090 GN=Tbl3 PE=2 SV=1
Serine/threonine-protein phosphatase 2A catalytic subunit alpha isoform OS=Mus musculus OX=10090 GN=Ppp2
WD repeat-containing protein 3 OS=Mus musculus OX=10090 GN=Wdr3 PE=1 SV=1
DnaJ homolog subfamily A member 1 OS=Mus musculus OX=10090 GN=Dnaja1 PE=1 SV=1
Dedicator of cytokinesis protein 9 OS=Mus musculus OX=10090 GN=Dock9 PE=1 SV=1
Mitogen-activated protein kinase kinase kinase 20 OS=Mus musculus OX=10090 GN=Map3k20 PE=1 SV=1
U4/U6 small nuclear ribonucleoprotein Prp3 OS=Mus musculus OX=10090 GN=Prpf3 PE=1 SV=1
Regulator of sex-limitation 2 OS=Mus musculus OX=10090 GN=Zfp429 PE=1 SV=1
Sorting nexin-6 OS=Mus musculus OX=10090 GN=Snx6 PE=1 SV=2
Choline transporter-like protein 2 OS=Mus musculus OX=10090 GN=Slc44a2 PE=1 SV=1
Peroxiredoxin-2 OS=Mus musculus OX=10090 GN=Prdx2 PE=1 SV=3
General transcription and DNA repair factor IIH helicase subunit XPB OS=Mus musculus OX=10090 GN=Ercc3 PE=

Kinesin-1 heavy chain OS=Mus musculus OX=10090 GN=Kif5b PE=1 SV=3
General transcription factor IIH subunit 2 OS=Mus musculus OX=10090 GN=Gtf2h2 PE=1 SV=1
Zinc finger protein 260 OS=Mus musculus OX=10090 GN=Znf260 PE=1 SV=1
Peflin OS=Mus musculus OX=10090 GN=Pef1 PE=1 SV=1
Aldehyde dehydrogenase OS=Mus musculus OX=10090 GN=Aldh3a2 PE=1 SV=1
T cell activation inhibitor, mitochondrial OS=Mus musculus OX=10090 GN=Tcaim PE=1 SV=1
39S ribosomal protein L17, mitochondrial OS=Mus musculus OX=10090 GN=Mrpl17 PE=1 SV=1
RING-type E3 ubiquitin transferase OS=Mus musculus OX=10090 GN=Mid2 PE=1 SV=1
Aurora kinase B OS=Mus musculus OX=10090 GN=Aurkb PE=1 SV=2
Extended synaptotagmin-2 OS=Mus musculus OX=10090 GN=Esyt2 PE=1 SV=1
Queuine tRNA-ribosyltransferase accessory subunit 2 OS=Mus musculus OX=10090 GN=Qtrt2 PE=1 SV=1
Tropomyosin alpha-1 chain OS=Mus musculus OX=10090 GN=Tpm1 PE=1 SV=1
Unconventional myosin-Vb OS=Mus musculus OX=10090 GN=Myo5b PE=1 SV=1
Cysteine-rich secretory protein LCCL domain-containing 2 OS=Mus musculus OX=10090 GN=Crispld2 PE=1 SV=1
Glutathione peroxidase 7 OS=Mus musculus OX=10090 GN=Gpx7 PE=1 SV=1
Growth factor receptor-bound protein 10 OS=Mus musculus OX=10090 GN=Grb10 PE=1 SV=2
SRA stem-loop-interacting RNA-binding protein, mitochondrial OS=Mus musculus OX=10090 GN=Slirp PE=1 SV=[illegible]
La-related protein 1 OS=Mus musculus OX=10090 GN=Larp1 PE=1 SV=1
Carnitine O-palmitoyltransferase 1, liver isoform OS=Mus musculus OX=10090 GN=Cpt1a PE=1 SV=4
Eosinophil cationic protein 2 OS=Mus musculus OX=10090 GN=Ear2 PE=2 SV=1
Granzyme K OS=Mus musculus OX=10090 GN=Gzmk PE=3 SV=1
Ribosomal protein OS=Mus musculus OX=10090 GN=Rpl10a PE=1 SV=1
MAP kinase-activated protein kinase 2 OS=Mus musculus OX=10090 GN=Mapkapk2 PE=1 SV=2
TAF6-like RNA polymerase II p300/CBP-associated factor-associated factor 65 kDa subunit 6L OS=Mus musculus
Bromodomain-containing protein 9 OS=Mus musculus OX=10090 GN=Brd9 PE=1 SV=1
Peroxisomal membrane protein PEX16 OS=Mus musculus OX=10090 GN=Pex16 PE=1 SV=2
Cell division cycle protein 23 homolog OS=Mus musculus OX=10090 GN=Cdc23 PE=1 SV=2
RNA-binding protein 33 OS=Mus musculus OX=10090 GN=Rbm33 PE=1 SV=1
Bromodomain and PHD finger-containing protein 3 OS=Mus musculus OX=10090 GN=Brpf3 PE=1 SV=1
Far upstream element-binding protein 1 OS=Mus musculus OX=10090 GN=Fubp1 PE=1 SV=1
Phospholipase D2 OS=Mus musculus OX=10090 GN=Pld2 PE=1 SV=2
BUB3-interacting and GLEBS motif-containing protein ZNF207 OS=Mus musculus OX=10090 GN=Znf207 PE=1 S[illegible]
Semaphorin-3F OS=Mus musculus OX=10090 GN=Sema3f PE=2 SV=2
A-kinase anchor protein 5 OS=Mus musculus OX=10090 GN=Akap5 PE=1 SV=1
Exocyst complex component 2 OS=Mus musculus OX=10090 GN=Exoc2 PE=1 SV=1
[Pyruvate dehydrogenase [acetyl-transferring]]-phosphatase 1, mitochondrial OS=Mus musculus OX=10090 GN=[illegible]
Tensin-3 OS=Mus musculus OX=10090 GN=Tns3 PE=1 SV=1
Bone marrow stromal antigen 2 OS=Mus musculus OX=10090 GN=Bst2 PE=1 SV=1
Protein FRG1 OS=Mus musculus OX=10090 GN=Frg1 PE=1 SV=2
Histone-lysine N-methyltransferase NSD3 OS=Mus musculus OX=10090 GN=Nsd3 PE=1 SV=1
DENN domain-containing protein 2D OS=Mus musculus OX=10090 GN=Dennd2d PE=1 SV=1
Eukaryotic translation initiation factor 2 subunit 3, Y-linked OS=Mus musculus OX=10090 GN=Eif2s3y PE=1 SV=2
Carbonyl reductase [NADPH] 2 OS=Mus musculus OX=10090 GN=Cbr2 PE=1 SV=1
DNA fragmentation factor subunit beta OS=Mus musculus OX=10090 GN=Dffb PE=1 SV=1
Collagen, type VI, alpha 3 OS=Mus musculus OX=10090 GN=Col6a3 PE=1 SV=1

Talin-2 OS=Mus musculus OX=10090 GN=Tln2 PE=1 SV=1
Kelch-like ECH-associated protein 1 OS=Mus musculus OX=10090 GN=Keap1 PE=1 SV=1
Phosphatidylserine decarboxylase proenzyme, mitochondrial OS=Mus musculus OX=10090 GN=Pisd PE=2 SV=1
Glutathione S-transferase LANCL1 OS=Mus musculus OX=10090 GN=Lancl1 PE=1 SV=1
Heat shock 70 kDa protein 4 OS=Mus musculus OX=10090 GN=Hspa4 PE=1 SV=1
Y-box-binding protein 1 OS=Mus musculus OX=10090 GN=Ybx1 PE=1 SV=3
Guanine nucleotide-binding protein-like 1 OS=Mus musculus OX=10090 GN=Gnl1 PE=1 SV=4
BRI3-binding protein OS=Mus musculus OX=10090 GN=Bri3bp PE=1 SV=1
39S ribosomal protein L27, mitochondrial OS=Mus musculus OX=10090 GN=Mrpl27 PE=1 SV=1
Nuclear pore complex protein Nup155 OS=Mus musculus OX=10090 GN=Nup155 PE=1 SV=1
Zinc finger protein 655 OS=Mus musculus OX=10090 GN=Zfp655 PE=1 SV=1
Vacuolar protein sorting-associated protein 4B OS=Mus musculus OX=10090 GN=Vps4b PE=1 SV=2
Neuroblastoma-amplified sequence OS=Mus musculus OX=10090 GN=Nbas PE=1 SV=1
Protein phosphatase 1, regulatory subunit 9A OS=Mus musculus OX=10090 GN=Ppp1r9a PE=1 SV=1
WD repeat- and FYVE domain-containing protein 4 OS=Mus musculus OX=10090 GN=Wdfy4 PE=1 SV=2
Envoplakin OS=Mus musculus OX=10090 GN=Evpl PE=1 SV=3
Septin-4 OS=Mus musculus OX=10090 GN=Septin4 PE=1 SV=1
Tricarboxylate transport protein, mitochondrial OS=Mus musculus OX=10090 GN=Slc25a1 PE=1 SV=1
Protein asteroid homolog 1 OS=Mus musculus OX=10090 GN=Aste1 PE=2 SV=1
General transcription factor II-I OS=Mus musculus OX=10090 GN=Gtf2i PE=1 SV=1
Synaptosomal-associated protein 23 OS=Mus musculus OX=10090 GN=Snap23 PE=1 SV=1
ADP-ribosylation factor-like protein 8B OS=Mus musculus OX=10090 GN=Arl8b PE=1 SV=1
GTP-binding nuclear protein Ran OS=Mus musculus OX=10090 GN=Ran PE=1 SV=3
28S ribosomal protein S15, mitochondrial OS=Mus musculus OX=10090 GN=Mrps15 PE=1 SV=2
E3 ubiquitin-protein ligase CBL-B OS=Mus musculus OX=10090 GN=Cblb PE=1 SV=3
Oxygen-regulated protein 1 OS=Mus musculus OX=10090 GN=Rp1 PE=1 SV=1
26S proteasome non-ATPase regulatory subunit 8 OS=Mus musculus OX=10090 GN=Psmd8 PE=1 SV=2
Periplakin OS=Mus musculus OX=10090 GN=Ppl PE=1 SV=1
Signal transducer and transcription activator 6 OS=Mus musculus OX=10090 GN=Stat6 PE=1 SV=2
SR-related CTD-associated factor 11 OS=Mus musculus OX=10090 GN=Scaf11 PE=1 SV=1
Replication factor C subunit 2 OS=Mus musculus OX=10090 GN=Rfc2 PE=1 SV=1
Ras suppressor protein 1 OS=Mus musculus OX=10090 GN=Rsu1 PE=1 SV=3
F-box and WD-40 domain protein 9 OS=Mus musculus OX=10090 GN=Fbxw9 PE=1 SV=1
WD repeat-containing protein 44 OS=Mus musculus OX=10090 GN=Wdr44 PE=1 SV=1
Coiled-coil domain-containing protein 63 OS=Mus musculus OX=10090 GN=Ccdc63 PE=2 SV=2
Importin-7 OS=Mus musculus OX=10090 GN=Ipo7 PE=1 SV=2
B-cell CLL/lymphoma 7 protein family member B OS=Mus musculus OX=10090 GN=Bcl7b PE=1 SV=1
Translation machinery-associated protein 16 OS=Mus musculus OX=10090 GN=Tma16 PE=1 SV=1
Ribonuclease inhibitor OS=Mus musculus OX=10090 GN=Rnh1 PE=1 SV=1
Isocitrate dehydrogenase [NAD] subunit, mitochondrial OS=Mus musculus OX=10090 GN=Idh3b PE=1 SV=1
Glutathione S-transferase theta-2 OS=Mus musculus OX=10090 GN=Gstt2 PE=1 SV=4
Adhesion G protein-coupled receptor L2 OS=Mus musculus OX=10090 GN=Adgrl2 PE=1 SV=1
Leucine-rich repeat flightless-interacting protein 1 OS=Mus musculus OX=10090 GN=Lrrfip1 PE=1 SV=2
ATP synthase-coupling factor 6, mitochondrial OS=Mus musculus OX=10090 GN=Atp5pf PE=1 SV=1
Alpha-parvin OS=Mus musculus OX=10090 GN=Parva PE=1 SV=1

Switch-associated protein 70 OS=Mus musculus OX=10090 GN=Swap70 PE=1 SV=2
1-phosphatidylinositol 4,5-bisphosphate phosphodiesterase beta-1 OS=Mus musculus OX=10090 GN=Plcb1 PE=1
Annexin A6 OS=Mus musculus OX=10090 GN=Anxa6 PE=1 SV=3
Intracellular hyaluronan-binding protein 4 OS=Mus musculus OX=10090 GN=Habp4 PE=1 SV=2
2-methoxy-6-polyprenyl-1,4-benzoquinol methylase, mitochondrial OS=Mus musculus OX=10090 GN=Coq5 PE=1
Cleft lip and palate transmembrane protein 1 homolog OS=Mus musculus OX=10090 GN=Clptm1 PE=1 SV=1
Histone-lysine N-methyltransferase SETDB1 OS=Mus musculus OX=10090 GN=Setdb1 PE=1 SV=1
LETM1 domain-containing protein 1 OS=Mus musculus OX=10090 GN=Letmd1 PE=1 SV=1
U1 small nuclear ribonucleoprotein 70 kDa OS=Mus musculus OX=10090 GN=Snrnp70 PE=1 SV=2
tRNA 2'-phosphotransferase 1 OS=Mus musculus OX=10090 GN=Trpt1 PE=2 SV=2
MAP kinase-interacting serine/threonine-protein kinase 1 OS=Mus musculus OX=10090 GN=Mknk1 PE=1 SV=1
Sacsin OS=Mus musculus OX=10090 GN=Sacs PE=1 SV=1
Heat shock 70 kDa protein 12A OS=Mus musculus OX=10090 GN=Hspa12a PE=1 SV=1
DExD/H box helicase 60 OS=Mus musculus OX=10090 GN=Ddx60 PE=1 SV=1
Zinc finger protein 512 OS=Mus musculus OX=10090 GN=Znf512 PE=2 SV=2
Proteasome activator complex subunit 1 OS=Mus musculus OX=10090 GN=Psme1 PE=1 SV=2
Core histone macro-H2A.2 OS=Mus musculus OX=10090 GN=Macroh2a2 PE=1 SV=3
Endothelial cell-selective adhesion molecule OS=Mus musculus OX=10090 GN=Esam PE=1 SV=1
Eukaryotic translation initiation factor 5A (Fragment) OS=Mus musculus OX=10090 GN=Eif5a PE=1 SV=1
NCK-associated protein 5 OS=Mus musculus OX=10090 GN=Nckap5 PE=1 SV=1
Optic atrophy 3 protein homolog OS=Mus musculus OX=10090 GN=Opa3 PE=1 SV=1
Translation initiation factor eIF-2B subunit delta OS=Mus musculus OX=10090 GN=Eif2b4 PE=1 SV=2
Fatty acid synthase OS=Mus musculus OX=10090 GN=Fasn PE=1 SV=2
Dual specificity mitogen-activated protein kinase kinase 1 OS=Mus musculus OX=10090 GN=Map2k1 PE=1 SV=2
Peptidyl-prolyl cis-trans isomerase FKBP8 OS=Mus musculus OX=10090 GN=Fkbp8 PE=1 SV=2
Deoxyribonuclease-1 OS=Mus musculus OX=10090 GN=Dnase1 PE=1 SV=1
Tenascin XB OS=Mus musculus OX=10090 GN=Tnxb PE=1 SV=1
Guanylate cyclase OS=Mus musculus OX=10090 GN=Gucy1a2 PE=1 SV=1
E3 ubiquitin-protein ligase listerin OS=Mus musculus OX=10090 GN=Ltn1 PE=1 SV=3
Ubiquitin carboxyl-terminal hydrolase 30 (Fragment) OS=Mus musculus OX=10090 GN=Usp30 PE=1 SV=1
PR domain zinc finger protein 15 OS=Mus musculus OX=10090 GN=Prdm15 PE=1 SV=1
Myomesin 2 OS=Mus musculus OX=10090 GN=Myom2 PE=1 SV=1
Lipopolysaccharide-responsive and beige-like anchor protein OS=Mus musculus OX=10090 GN=Lrba PE=1 SV=1
Protein disulfide-isomerase OS=Mus musculus OX=10090 GN=P4hb PE=1 SV=2
Transketolase OS=Mus musculus OX=10090 GN=Tkt PE=1 SV=1
Sodium- and chloride-dependent neutral and basic amino acid transporter B(0+) OS=Mus musculus OX=10090 GI
Prickle-like 2 (Drosophila) OS=Mus musculus OX=10090 GN=Prickle2 PE=1 SV=1
26S proteasome non-ATPase regulatory subunit 13 OS=Mus musculus OX=10090 GN=Psmd13 PE=1 SV=1
Bteb1 protein OS=Mus musculus OX=10090 GN=Klf9 PE=1 SV=1
Zinc finger protein 710 OS=Mus musculus OX=10090 GN=Znf710 PE=2 SV=1
Inosine-5'-monophosphate dehydrogenase 2 OS=Mus musculus OX=10090 GN=Impdh2 PE=1 SV=2
Calcium-transporting ATPase OS=Mus musculus OX=10090 GN=Atp2b3 PE=1 SV=1
Calpain inhibitor OS=Mus musculus OX=10090 GN=Cast PE=1 SV=1
Arf-GAP with GTPase, ANK repeat and PH domain-containing protein 3 OS=Mus musculus OX=10090 GN=Agap3
Vacuolar protein sorting-associated protein 35 OS=Mus musculus OX=10090 GN=Vps35 PE=1 SV=1

Pleckstrin homology domain-containing family A member 2 OS=Mus musculus OX=10090 GN=Plekha2 PE=1 SV=:
EH domain-containing protein 3 OS=Mus musculus OX=10090 GN=Ehd3 PE=1 SV=2
UDP-glucuronosyltransferase 1-6 OS=Mus musculus OX=10090 GN=Ugt1a6 PE=1 SV=1
Serine palmitoyltransferase 2 OS=Mus musculus OX=10090 GN=Sptlc2 PE=1 SV=2
Zinc finger protein 672 OS=Mus musculus OX=10090 GN=Znf672 PE=2 SV=1
40S ribosomal protein S18 OS=Mus musculus OX=10090 GN=Rps18 PE=1 SV=3
Protein kinase C OS=Mus musculus OX=10090 GN=Pkn2 PE=1 SV=1
Cold shock domain-containing protein E1 OS=Mus musculus OX=10090 GN=Csde1 PE=1 SV=1
Acetyl-CoA carboxylase 1 OS=Mus musculus OX=10090 GN=Acaca PE=1 SV=1
Tissue factor pathway inhibitor OS=Mus musculus OX=10090 GN=Tfpi PE=1 SV=1
Tyrosine-protein kinase Tec OS=Mus musculus OX=10090 GN=Tec PE=1 SV=2
Ankyrin repeat domain 35 OS=Mus musculus OX=10090 GN=Ankrd35 PE=1 SV=1
mRNA-capping enzyme OS=Mus musculus OX=10090 GN=Rngtt PE=1 SV=1
Basement membrane-specific heparan sulfate proteoglycan core protein OS=Mus musculus OX=10090 GN=Hspg
Kinesin light chain OS=Mus musculus OX=10090 GN=Klc2 PE=1 SV=1
AP-2 complex subunit beta OS=Mus musculus OX=10090 GN=Ap2b1 PE=1 SV=1
Protein phosphatase 1G OS=Mus musculus OX=10090 GN=Ppm1g PE=1 SV=3
WD repeat-containing protein 35 OS=Mus musculus OX=10090 GN=Wdr35 PE=1 SV=3
60 kDa heat shock protein, mitochondrial OS=Mus musculus OX=10090 GN=Hspd1 PE=1 SV=1
Phosphatidylinositol 3,4,5-trisphosphate 5-phosphatase 1 OS=Mus musculus OX=10090 GN=Inpp5d PE=1 SV=2
La-related protein 4B OS=Mus musculus OX=10090 GN=Larp4b PE=1 SV=2
Low affinity immunoglobulin gamma Fc region receptor IV OS=Mus musculus OX=10090 GN=Fcgr4 PE=1 SV=1
Endoplasmic reticulum resident protein 44 OS=Mus musculus OX=10090 GN=Erp44 PE=1 SV=1
Extended synaptotagmin-1 OS=Mus musculus OX=10090 GN=Esyt1 PE=1 SV=2
Zinc finger protein 646 (Fragment) OS=Mus musculus OX=10090 GN=Zfp646 PE=1 SV=1
Cytochrome b-c1 complex subunit 9 OS=Mus musculus OX=10090 GN=Uqcr10 PE=1 SV=1
Serine/threonine-protein phosphatase 2B catalytic subunit alpha isoform OS=Mus musculus OX=10090 GN=Ppp3
AP2-associated protein kinase 1 OS=Mus musculus OX=10090 GN=Aak1 PE=1 SV=2
STAM-binding protein OS=Mus musculus OX=10090 GN=Stambp PE=1 SV=1
Tripartite motif-containing protein 34A OS=Mus musculus OX=10090 GN=Trim34a PE=1 SV=2
Antigen peptide transporter 1 OS=Mus musculus OX=10090 GN=Tap1 PE=1 SV=1
Signal peptidase complex catalytic subunit SEC11 OS=Mus musculus OX=10090 GN=Sec11a PE=1 SV=1
Phosphorylase b kinase regulatory subunit OS=Mus musculus OX=10090 GN=Phka1 PE=1 SV=1
Aspartate carbamoyltransferase OS=Mus musculus OX=10090 GN=Cad PE=1 SV=1
T-cell surface antigen CD2 OS=Mus musculus OX=10090 GN=Cd2 PE=1 SV=1
Peroxisomal biogenesis factor 19 OS=Mus musculus OX=10090 GN=Pex19 PE=1 SV=1
Activated RNA polymerase II transcriptional coactivator p15 OS=Mus musculus OX=10090 GN=Sub1 PE=1 SV=3
Protein C1orf43 homolog OS=Mus musculus OX=10090 PE=2 SV=1
Host cell factor 1 OS=Mus musculus OX=10090 GN=Hcfc1 PE=1 SV=2
Patatin-like phospholipase domain-containing protein 7 OS=Mus musculus OX=10090 GN=Pnpla7 PE=1 SV=1
Nuclear cap-binding protein subunit 1 OS=Mus musculus OX=10090 GN=Ncbp1 PE=1 SV=2
Valyl-tRNA synthetase (Fragment) OS=Mus musculus OX=10090 GN=Vars PE=1 SV=1
Glutamine--fructose-6-phosphate aminotransferase [isomerizing] 1 OS=Mus musculus OX=10090 GN=Gfpt1 PE=
Calcium uptake protein 1, mitochondrial OS=Mus musculus OX=10090 GN=Micu1 PE=1 SV=1
Epithelial membrane protein 2 OS=Mus musculus OX=10090 GN=Emp2 PE=1 SV=1

FERM domain-containing protein 6 OS=Mus musculus OX=10090 GN=Frmd6 PE=1 SV=2
Histone lysine acetyltransferase CREBBP OS=Mus musculus OX=10090 GN=Crebbp PE=1 SV=3
Zinc finger CCCH domain-containing protein 13 OS=Mus musculus OX=10090 GN=Zc3h13 PE=1 SV=1
Ras association domain-containing protein 5 OS=Mus musculus OX=10090 GN=Rassf5 PE=1 SV=1
p21-activated protein kinase-interacting protein 1 OS=Mus musculus OX=10090 GN=Pak1ip1 PE=1 SV=2
T-complex protein 1 subunit beta OS=Mus musculus OX=10090 GN=Cct2 PE=1 SV=4
Protein argonaute-1 OS=Mus musculus OX=10090 GN=Ago1 PE=1 SV=2
Wiskott-Aldrich syndrome protein family member 2 OS=Mus musculus OX=10090 GN=Wasf2 PE=1 SV=1
Nck-associated protein 1 OS=Mus musculus OX=10090 GN=Nckap1 PE=1 SV=2
EH domain-containing protein 4 OS=Mus musculus OX=10090 GN=Ehd4 PE=1 SV=1
Huntingtin-interacting protein 1 OS=Mus musculus OX=10090 GN=Hip1 PE=1 SV=2
Trifunctional enzyme subunit alpha, mitochondrial OS=Mus musculus OX=10090 GN=Hadha PE=1 SV=1
Hsc70-interacting protein OS=Mus musculus OX=10090 GN=St13 PE=1 SV=1
Annexin A2 OS=Mus musculus OX=10090 GN=Anxa2 PE=1 SV=2
Rab GTPase-activating protein 1 OS=Mus musculus OX=10090 GN=Rabgap1 PE=1 SV=1
Serine protease FAM111A OS=Mus musculus OX=10090 GN=Fam111a PE=2 SV=1
Metastasis-associated protein MTA2 OS=Mus musculus OX=10090 GN=Mta2 PE=1 SV=1
Lariat debranching enzyme OS=Mus musculus OX=10090 GN=Dbr1 PE=1 SV=2
Hexokinase-2 OS=Mus musculus OX=10090 GN=Hk2 PE=1 SV=1
Kinesin-like protein (Fragment) OS=Mus musculus OX=10090 GN=Kif23 PE=1 SV=1
Zinc finger protein 22 OS=Mus musculus OX=10090 GN=Znf22 PE=1 SV=2
Serine/threonine-protein kinase receptor R3 OS=Mus musculus OX=10090 GN=Acvrl1 PE=1 SV=2
E3 ubiquitin-protein ligase ZFP91 OS=Mus musculus OX=10090 GN=Zfp91 PE=1 SV=3
Sulfide:quinone oxidoreductase, mitochondrial OS=Mus musculus OX=10090 GN=Sqor PE=1 SV=3
Ras-related protein Rab-8A OS=Mus musculus OX=10090 GN=Rab8a PE=1 SV=2
Glutamate-rich 3 OS=Mus musculus OX=10090 GN=Erich3 PE=1 SV=1
Immunity-related GTPase family M member 2 OS=Mus musculus OX=10090 GN=Irgm2 PE=1 SV=1
BTB/POZ domain-containing protein KCTD12 OS=Mus musculus OX=10090 GN=Kctd12 PE=1 SV=1
Succinate dehydrogenase [ubiquinone] flavoprotein subunit, mitochondrial OS=Mus musculus OX=10090 GN=Sdh
Probable ATP-dependent RNA helicase DDX31 OS=Mus musculus OX=10090 GN=Ddx31 PE=2 SV=2
Zinc finger and BTB domain-containing protein 24 OS=Mus musculus OX=10090 GN=Zbtb24 PE=1 SV=1
Surfeit locus protein 4 OS=Mus musculus OX=10090 GN=Surf4 PE=1 SV=1
DNA-directed DNA/RNA polymerase mu OS=Mus musculus OX=10090 GN=Polm PE=1 SV=1
Leucine-rich repeat flightless-interacting protein 2 OS=Mus musculus OX=10090 GN=Lrrfip2 PE=1 SV=1
Amyloid beta A4 precursor protein-binding family B member 1-interacting protein OS=Mus musculus OX=10090
Homeobox protein Hox-A5 OS=Mus musculus OX=10090 GN=Hoxa5 PE=1 SV=1
Protocadherin 9 OS=Mus musculus OX=10090 GN=Pcdh9 PE=1 SV=1
Zinc finger protein 180 OS=Mus musculus OX=10090 GN=Zfp180 PE=1 SV=1
Membrane-associated phosphatidylinositol transfer protein 2 OS=Mus musculus OX=10090 GN=Pitpnm2 PE=1 S
Mitochondrial mRNA pseudouridine synthase Rpusd3 OS=Mus musculus OX=10090 GN=Rpusd3 PE=2 SV=1
Dual-specificity mitogen-activated protein kinase kinase 4 (Fragment) OS=Mus musculus OX=10090 GN=Map2k4
RIKEN cDNA 2900026A02 gene OS=Mus musculus OX=10090 GN=2900026A02Rik PE=1 SV=1
Paxillin OS=Mus musculus OX=10090 GN=Pxn PE=1 SV=1
Ras-related protein Rab-1A OS=Mus musculus OX=10090 GN=Rab1A PE=1 SV=3
Voltage-dependent anion-selective channel protein 1 OS=Mus musculus OX=10090 GN=Vdac1 PE=1 SV=3

Translation initiation factor eIF-2B subunit epsilon (Fragment) OS=Mus musculus OX=10090 GN=Eif2b5 PE=1 SV=
DDB1 and CUL4-associated factor 4 OS=Mus musculus OX=10090 GN=Dcaf4 PE=1 SV=1
Basic leucine zipper and W2 domain-containing protein 1 OS=Mus musculus OX=10090 GN=Bzw1 PE=1 SV=1
U3 small nucleolar ribonucleoprotein protein IMP3 OS=Mus musculus OX=10090 GN=Imp3 PE=2 SV=1
39S ribosomal protein L48, mitochondrial OS=Mus musculus OX=10090 GN=Mrpl48 PE=1 SV=1
Zinc finger and SCAN domain-containing 25 OS=Mus musculus OX=10090 GN=Zscan25 PE=1 SV=1
DNA helicase OS=Mus musculus OX=10090 GN=Chd3 PE=1 SV=1
RNA-binding protein 27 OS=Mus musculus OX=10090 GN=Rbm27 PE=1 SV=3
Sepiapterin reductase OS=Mus musculus OX=10090 GN=Spr PE=1 SV=1
Serine/threonine-protein phosphatase 6 regulatory subunit 3 OS=Mus musculus OX=10090 GN=Ppp6r3 PE=1 SV=
AP complex subunit sigma OS=Mus musculus OX=10090 GN=Ap1s2 PE=1 SV=1
Protein FAM98A OS=Mus musculus OX=10090 GN=Fam98a PE=1 SV=1
Kelch-like protein 41 OS=Mus musculus OX=10090 GN=Klhl41 PE=1 SV=1
Latent-transforming growth factor beta-binding protein 4 OS=Mus musculus OX=10090 GN=Ltbp4 PE=1 SV=1
TNF receptor-associated factor 1 OS=Mus musculus OX=10090 GN=Traf1 PE=1 SV=2
Transcription initiation factor TFIID subunit 7 OS=Mus musculus OX=10090 GN=Taf7 PE=1 SV=1
Extracellular sulfatase Sulf-1 OS=Mus musculus OX=10090 GN=Sulf1 PE=1 SV=1
U4/U6.U5 tri-snRNP-associated protein 1 OS=Mus musculus OX=10090 GN=Sart1 PE=1 SV=1
Far upstream element (FUSE)-binding protein 3 OS=Mus musculus OX=10090 GN=Fubp3 PE=1 SV=1
Mediator of RNA polymerase II transcription subunit 12 OS=Mus musculus OX=10090 GN=Med12 PE=1 SV=1
Voltage-dependent calcium channel subunit alpha-2/delta-1 OS=Mus musculus OX=10090 GN=Cacna2d1 PE=1 S
1-acyl-sn-glycerol-3-phosphate acyltransferase gamma OS=Mus musculus OX=10090 GN=Agpat3 PE=1 SV=2
PHD finger protein 14 OS=Mus musculus OX=10090 GN=Phf14 PE=1 SV=1
Protein lin-54 homolog OS=Mus musculus OX=10090 GN=Lin54 PE=2 SV=2
Mitogen-activated protein kinase kinase kinase OS=Mus musculus OX=10090 GN=Map3k6 PE=1 SV=1
Lipoyl synthase, mitochondrial OS=Mus musculus OX=10090 GN=Lias PE=1 SV=1
GTPase IMAP family member 8 OS=Mus musculus OX=10090 GN=Gimap8 PE=1 SV=1
DCC-interacting protein 13-alpha OS=Mus musculus OX=10090 GN=Appl1 PE=1 SV=1
Vacuolar protein sorting-associated protein 51 homolog OS=Mus musculus OX=10090 GN=Vps51 PE=1 SV=2
TNF receptor-associated factor 2 OS=Mus musculus OX=10090 GN=Traf2 PE=1 SV=1
Protein DDI1 homolog 2 OS=Mus musculus OX=10090 GN=Ddi2 PE=1 SV=1
Tax1-binding protein 3 OS=Mus musculus OX=10090 GN=Tax1bp3 PE=1 SV=1
Complement C3 OS=Mus musculus OX=10090 GN=C3 PE=1 SV=3
Arf-GAP with coiled-coil, ANK repeat and PH domain-containing protein 2 OS=Mus musculus OX=10090 GN=Aca
DENN domain-containing protein 3 OS=Mus musculus OX=10090 GN=Dennd3 PE=1 SV=2
26S proteasome regulatory subunit 6A OS=Mus musculus OX=10090 GN=Psmc3 PE=1 SV=2
SEC14-like 3 (S. cerevisiae) OS=Mus musculus OX=10090 GN=Sec14l3 PE=1 SV=1
Prelamin-A/C OS=Mus musculus OX=10090 GN=Lmna PE=1 SV=2
Putative RNA polymerase II subunit B1 CTD phosphatase Rpap2 OS=Mus musculus OX=10090 GN=Rpap2 PE=2 S
Transmembrane anterior posterior transformation protein 1 OS=Mus musculus OX=10090 GN=Tapt1 PE=1 SV=2
Actin-like protein 6A OS=Mus musculus OX=10090 GN=Actl6a PE=1 SV=2
BAG family molecular chaperone regulator 5 OS=Mus musculus OX=10090 GN=Bag5 PE=1 SV=1
Sorting nexin-1 OS=Mus musculus OX=10090 GN=Snx1 PE=1 SV=1
U2 small nuclear ribonucleoprotein A' OS=Mus musculus OX=10090 GN=Snrpa1 PE=1 SV=2
Alpha globin 1 OS=Mus musculus OX=10090 GN=Hba-a1 PE=1 SV=1

Deoxynucleoside triphosphate triphosphohydrolase SAMHD1 OS=Mus musculus OX=10090 GN=Samhd1 PE=1 SV=
IQ calmodulin-binding motif-containing protein 1 OS=Mus musculus OX=10090 GN=Iqcb1 PE=1 SV=2
PDZ domain-containing 2 OS=Mus musculus OX=10090 GN=Pdzd2 PE=1 SV=1
Poly(rC)-binding protein 1 OS=Mus musculus OX=10090 GN=Pcbp1 PE=1 SV=1
Protein IWS1 homolog OS=Mus musculus OX=10090 GN=Iws1 PE=1 SV=1
T-complex protein 1 subunit epsilon OS=Mus musculus OX=10090 GN=Cct5 PE=1 SV=1
Band 4.1-like protein 2 OS=Mus musculus OX=10090 GN=Epb41l2 PE=1 SV=1
Centrosome-associated protein 350 OS=Mus musculus OX=10090 GN=Cep350 PE=1 SV=1
Long-chain fatty acid transport protein 4 OS=Mus musculus OX=10090 GN=Slc27a4 PE=1 SV=1
snRNA-activating protein complex subunit 2 OS=Mus musculus OX=10090 GN=Snapc2 PE=2 SV=1
Syntaxin-binding protein 1 OS=Mus musculus OX=10090 GN=Stxbp1 PE=1 SV=2
Cytochrome P450 20A1 OS=Mus musculus OX=10090 GN=Cyp20a1 PE=1 SV=1
Serpin H1 OS=Mus musculus OX=10090 GN=Serpinh1 PE=1 SV=3
Laminin subunit gamma-2 OS=Mus musculus OX=10090 GN=Lamc2 PE=1 SV=1
Synemin OS=Mus musculus OX=10090 GN=Synm PE=1 SV=1
H-2 class II histocompatibility antigen, A beta chain OS=Mus musculus OX=10090 GN=H2-Ab1 PE=1 SV=1
UMP-CMP kinase OS=Mus musculus OX=10090 GN=Cmpk1 PE=1 SV=1
Proteasome subunit beta type-4 OS=Mus musculus OX=10090 GN=Psmb4 PE=1 SV=1
Kinesin-like protein OS=Mus musculus OX=10090 GN=Kifc3 PE=1 SV=1
Multimerin-1 OS=Mus musculus OX=10090 GN=Mmrn1 PE=1 SV=1
Cytochrome c oxidase subunit NDUFA4 OS=Mus musculus OX=10090 GN=Ndufa4 PE=1 SV=2
Symplekin OS=Mus musculus OX=10090 GN=Sympk PE=1 SV=2
Dynamin-1-like protein OS=Mus musculus OX=10090 GN=Dnm1l PE=1 SV=1
Nuclear exosome regulator NRDE2 OS=Mus musculus OX=10090 GN=Nrde2 PE=1 SV=3
Protein phosphatase 1H OS=Mus musculus OX=10090 GN=Ppm1h PE=1 SV=1
Mediator of RNA polymerase II transcription subunit 17 OS=Mus musculus OX=10090 GN=Med17 PE=1 SV=1
Cas scaffolding protein family member 4 OS=Mus musculus OX=10090 GN=Cass4 PE=1 SV=1
Nuclear mitotic apparatus protein 1 OS=Mus musculus OX=10090 GN=Numa1 PE=1 SV=1
Ubiquitin-associated protein 2-like OS=Mus musculus OX=10090 GN=Ubap2l PE=1 SV=1
Calcium uptake protein 3, mitochondrial OS=Mus musculus OX=10090 GN=Micu3 PE=2 SV=2
Far upstream element-binding protein 2 OS=Mus musculus OX=10090 GN=Khsrp PE=1 SV=2
Calcium homeostasis endoplasmic reticulum protein OS=Mus musculus OX=10090 GN=Cherp PE=1 SV=2
Myotubularin-related protein 5 OS=Mus musculus OX=10090 GN=Sbf1 PE=1 SV=2
Breast cancer anti-estrogen resistance protein 3 homolog OS=Mus musculus OX=10090 GN=Bcar3 PE=1 SV=1
Hexosyltransferase OS=Mus musculus OX=10090 GN=Chpf2 PE=1 SV=1
Protein unc-45 homolog A OS=Mus musculus OX=10090 GN=Unc45a PE=1 SV=1
Vinexin OS=Mus musculus OX=10090 GN=Sorbs3 PE=1 SV=1
Signal recognition particle subunit SRP72 OS=Mus musculus OX=10090 GN=Srp72 PE=1 SV=1
Aldehyde dehydrogenase family 3 member B1 OS=Mus musculus OX=10090 GN=Aldh3b1 PE=1 SV=1
Beta-1,3-galactosyl-O-glycosyl-glycoprotein beta-1,6-N-acetylglucosaminyltransferase OS=Mus musculus OX=10
Phosphatidylinositol-3-phosphatase SAC1 OS=Mus musculus OX=10090 GN=Sacm1l PE=1 SV=1
Ribosome biogenesis protein BRX1 homolog OS=Mus musculus OX=10090 GN=Brix1 PE=1 SV=3
Twinkle protein, mitochondrial OS=Mus musculus OX=10090 GN=Twnk PE=2 SV=1
Integrator complex subunit 9 OS=Mus musculus OX=10090 GN=Ints9 PE=1 SV=1
Elongator complex protein 2 OS=Mus musculus OX=10090 GN=Elp2 PE=1 SV=1

Kelch-like protein 11 OS=Mus musculus OX=10090 GN=Klhl11 PE=1 SV=1
Microphthalmia-associated transcription factor OS=Mus musculus OX=10090 GN=Mitf PE=1 SV=4
Protein Shroom4 OS=Mus musculus OX=10090 GN=Shroom4 PE=1 SV=1
Sphingosine-1-phosphate lyase 1 OS=Mus musculus OX=10090 GN=Sgpl1 PE=1 SV=1
Cyclin-T1 OS=Mus musculus OX=10090 GN=Ccnt1 PE=1 SV=3
Lipoma-preferred partner homolog OS=Mus musculus OX=10090 GN=Lpp PE=1 SV=1
E3 ubiquitin-protein ligase UBR3 OS=Mus musculus OX=10090 GN=Ubr3 PE=1 SV=3
Eukaryotic translation initiation factor 4 gamma 3 OS=Mus musculus OX=10090 GN=Eif4g3 PE=1 SV=1
Phospholipid hydroperoxide glutathione peroxidase OS=Mus musculus OX=10090 GN=Gpx4 PE=1 SV=4
Uncharacterized protein C1orf115 homolog OS=Mus musculus OX=10090 PE=2 SV=1
Telomerase RNA component interacting RNase OS=Mus musculus OX=10090 GN=Trir PE=1 SV=1
Dolichyl-diphosphooligosaccharide--protein glycosyltransferase subunit STT3A OS=Mus musculus OX=10090 GN=
Coatomer subunit delta OS=Mus musculus OX=10090 GN=Arcn1 PE=1 SV=2
Poly(rC)-binding protein 3 OS=Mus musculus OX=10090 GN=Pcbp3 PE=1 SV=3
EH domain-containing protein 2 OS=Mus musculus OX=10090 GN=Ehd2 PE=1 SV=1
Pericentriolar material 1 protein OS=Mus musculus OX=10090 GN=Pcm1 PE=1 SV=2
Ras-related protein Rab-14 OS=Mus musculus OX=10090 GN=Rab14 PE=1 SV=3
Trifunctional enzyme subunit beta, mitochondrial OS=Mus musculus OX=10090 GN=Hadhb PE=1 SV=1
Eukaryotic initiation factor 4A-I OS=Mus musculus OX=10090 GN=Eif4a1 PE=1 SV=1
Coiled-coil domain-containing 141 OS=Mus musculus OX=10090 GN=Ccdc141 PE=1 SV=1
Translation initiation factor eIF-2B subunit alpha OS=Mus musculus OX=10090 GN=Eif2b1 PE=1 SV=1
Tyrosine-protein kinase OS=Mus musculus OX=10090 GN=Jak1 PE=1 SV=1
tRNA (guanine(26)-N(2))-dimethyltransferase (Fragment) OS=Mus musculus OX=10090 GN=Trmt1 PE=1 SV=1
Glucose-6-phosphate 1-dehydrogenase (Fragment) OS=Mus musculus OX=10090 GN=G6pdx PE=1 SV=1
Transportin-1 OS=Mus musculus OX=10090 GN=Tnpo1 PE=1 SV=2
Zinc transporter SLC39A7 OS=Mus musculus OX=10090 GN=Slc39a7 PE=1 SV=2
URB2 ribosome biogenesis 2 homolog (S. cerevisiae) OS=Mus musculus OX=10090 GN=Urb2 PE=1 SV=1
E3 ubiquitin-protein ligase NEDD4 OS=Mus musculus OX=10090 GN=Nedd4 PE=1 SV=3
Inhibitor of nuclear factor kappa-B kinase subunit beta OS=Mus musculus OX=10090 GN=Ikbkb PE=1 SV=1
Nuclear factor related to kappa-B-binding protein OS=Mus musculus OX=10090 GN=Nfrkb PE=1 SV=1
60S ribosomal protein L11 OS=Mus musculus OX=10090 GN=Rpl11 PE=1 SV=4
TAR DNA-binding protein 43 OS=Mus musculus OX=10090 GN=Tardbp PE=1 SV=1
Chloride intracellular channel protein OS=Mus musculus OX=10090 GN=Clic5 PE=1 SV=1
Peptidylprolyl isomerase OS=Mus musculus OX=10090 GN=Fkbp15 PE=1 SV=1
V-type proton ATPase subunit H OS=Mus musculus OX=10090 GN=Atp6v1h PE=1 SV=1
Signal transducer and activator of transcription 3 OS=Mus musculus OX=10090 GN=Stat3 PE=1 SV=2
Transforming protein RhoA OS=Mus musculus OX=10090 GN=Rhoa PE=1 SV=1
GTPase IMAP family member 1 OS=Mus musculus OX=10090 GN=Gimap1 PE=1 SV=3
NADH dehydrogenase [ubiquinone] iron-sulfur protein 7, mitochondrial OS=Mus musculus OX=10090 GN=Ndufs7
Tight junction protein 3 OS=Mus musculus OX=10090 GN=Tjp3 PE=1 SV=1
Regulatory-associated protein of mTOR OS=Mus musculus OX=10090 GN=Rptor PE=1 SV=1
Fibrinogen beta chain OS=Mus musculus OX=10090 GN=Fgb PE=1 SV=1
Phosphate carrier protein, mitochondrial OS=Mus musculus OX=10090 GN=Slc25a3 PE=1 SV=1
40S ribosomal protein S7 OS=Mus musculus OX=10090 GN=Rps7 PE=2 SV=1
WD repeat-containing protein 37 OS=Mus musculus OX=10090 GN=Wdr37 PE=1 SV=1

ATP-binding cassette sub-family F member 1 OS=Mus musculus OX=10090 GN=Abcf1 PE=1 SV=1
SH2 domain-containing protein 3C OS=Mus musculus OX=10090 GN=Sh2d3c PE=1 SV=1
AP-3 complex subunit delta-1 OS=Mus musculus OX=10090 GN=Ap3d1 PE=1 SV=1
Splicing factor 3B subunit 6 OS=Mus musculus OX=10090 GN=Sf3b6 PE=1 SV=1
Angiopoietin-related protein 2 OS=Mus musculus OX=10090 GN=Angptl2 PE=2 SV=2
Structural maintenance of chromosomes flexible hinge domain-containing protein 1 OS=Mus musculus OX=1009
Laminin subunit alpha-3 OS=Mus musculus OX=10090 GN=Lama3 PE=1 SV=4
UDP-glucuronosyltransferase 1A7 OS=Mus musculus OX=10090 GN=Ugt1a7 PE=1 SV=1
AP-3 complex subunit beta-1 OS=Mus musculus OX=10090 GN=Ap3b1 PE=1 SV=2
Endophilin-B1 OS=Mus musculus OX=10090 GN=Sh3glb1 PE=1 SV=1
Tyrosine-protein phosphatase non-receptor type 9 OS=Mus musculus OX=10090 GN=Ptpn9 PE=1 SV=2
Heterogeneous nuclear ribonucleoprotein Q OS=Mus musculus OX=10090 GN=Syncrip PE=1 SV=1
Ral GTPase-activating protein subunit alpha-2 OS=Mus musculus OX=10090 GN=Ralgapa2 PE=1 SV=1
H1 histone family, member X OS=Mus musculus OX=10090 GN=H1f10 PE=1 SV=1
Zinc finger protein 729a OS=Mus musculus OX=10090 GN=Zfp729a PE=1 SV=1
Filamin-binding LIM protein 1 OS=Mus musculus OX=10090 GN=Fblim1 PE=1 SV=2
UV radiation resistance-associated protein OS=Mus musculus OX=10090 GN=Uvrag PE=1 SV=2
Disabled homolog 2 OS=Mus musculus OX=10090 GN=Dab2 PE=1 SV=2
Spastic paraplegia 20, spartin (Troyer syndrome) homolog (human) (Fragment) OS=Mus musculus OX=10090 GN
DNA ligase OS=Mus musculus OX=10090 GN=Lig3 PE=1 SV=1
Krueppel-like factor 13 OS=Mus musculus OX=10090 GN=Klf13 PE=1 SV=2
Membrane-associated guanylate kinase inverted 3 OS=Mus musculus OX=10090 GN=Magi3 PE=1 SV=1
DnaJ homolog subfamily C member 10 OS=Mus musculus OX=10090 GN=Dnajc10 PE=1 SV=2
Synaptophysin-like protein OS=Mus musculus OX=10090 GN=Sypl PE=1 SV=1
Transcription factor 25 OS=Mus musculus OX=10090 GN=Tcf25 PE=1 SV=2
Protein FAM124B OS=Mus musculus OX=10090 GN=Fam124b PE=1 SV=1
Rac GTPase-activating protein 1 OS=Mus musculus OX=10090 GN=Racgap1 PE=1 SV=1
ELKS/Rab6-interacting/CAST family member 1 OS=Mus musculus OX=10090 GN=Erc1 PE=1 SV=1
Histidine triad nucleotide-binding protein 3 (Fragment) OS=Mus musculus OX=10090 GN=Hint3 PE=1 SV=2
U6 snRNA-associated Sm-like protein LSm4 OS=Mus musculus OX=10090 GN=Lsm4 PE=1 SV=1
Leucine zipper putative tumor suppressor 2 OS=Mus musculus OX=10090 GN=Lzts2 PE=1 SV=3
Trifunctional purine biosynthetic protein adenosine-3 OS=Mus musculus OX=10090 GN=Gart PE=1 SV=3
Expressed sequence AI987944 OS=Mus musculus OX=10090 GN=AI987944 PE=2 SV=1
Monocarboxylate transporter 10 OS=Mus musculus OX=10090 GN=Slc16a10 PE=1 SV=1
Neurofibromin OS=Mus musculus OX=10090 GN=Nf1 PE=1 SV=1
Activator of 90 kDa heat shock protein ATPase homolog 1 OS=Mus musculus OX=10090 GN=Ahsa1 PE=1 SV=2
Protein PRRC2B OS=Mus musculus OX=10090 GN=Prrc2b PE=1 SV=1
AP-5 complex subunit beta-1 OS=Mus musculus OX=10090 GN=Ap5b1 PE=1 SV=1
Cell division cycle 5-like protein OS=Mus musculus OX=10090 GN=Cdc5l PE=1 SV=2
Carbohydrate sulfotransferase 1 OS=Mus musculus OX=10090 GN=Chst1 PE=2 SV=1
Pleckstrin homology domain-containing family H member 3 OS=Mus musculus OX=10090 GN=Plekhh3 PE=2 SV=[illegible]
Cytosolic iron-sulfur assembly component 2B OS=Mus musculus OX=10090 GN=Ciao2b PE=1 SV=1
Zinc finger protein 32 OS=Mus musculus OX=10090 GN=Znf32 PE=2 SV=1
Ral GTPase-activating protein subunit alpha-1 OS=Mus musculus OX=10090 GN=Ralgapa1 PE=1 SV=1
Cell cycle control protein 50A OS=Mus musculus OX=10090 GN=Tmem30a PE=1 SV=1

Developmentally-regulated GTP-binding protein 2 OS=Mus musculus OX=10090 GN=Drg2 PE=1 SV=1
Beclin-1 OS=Mus musculus OX=10090 GN=Becn1 PE=1 SV=1
RNA-splicing ligase RtcB homolog OS=Mus musculus OX=10090 GN=Rtcb PE=1 SV=1
COP9 signalosome complex subunit 3 OS=Mus musculus OX=10090 GN=Cops3 PE=1 SV=3
E3 ubiquitin-protein ligase TRIM21 OS=Mus musculus OX=10090 GN=Trim21 PE=1 SV=1
Formin-binding protein 1-like OS=Mus musculus OX=10090 GN=Fnbp1l PE=1 SV=1
39S ribosomal protein L16, mitochondrial OS=Mus musculus OX=10090 GN=Mrpl16 PE=1 SV=1
Zinc finger protein 768 OS=Mus musculus OX=10090 GN=Zfp768 PE=1 SV=1
60S ribosomal protein L5 OS=Mus musculus OX=10090 GN=Rpl5 PE=1 SV=3
Guanylate-binding protein 2 OS=Mus musculus OX=10090 GN=Gbp2 PE=1 SV=1
Epithelial-stromal interaction protein 1 OS=Mus musculus OX=10090 GN=Epsti1 PE=1 SV=2
26S proteasome regulatory subunit 10B OS=Mus musculus OX=10090 GN=Psmc6 PE=1 SV=1
FERM, ARHGEF and pleckstrin domain-containing protein 1 OS=Mus musculus OX=10090 GN=Farp1 PE=1 SV=1
Splicing factor 3B subunit 5 OS=Mus musculus OX=10090 GN=Sf3b5 PE=1 SV=1
Actin-related protein 2/3 complex subunit 1B OS=Mus musculus OX=10090 GN=Arpc1b PE=1 SV=4
Vinculin OS=Mus musculus OX=10090 GN=Vcl PE=1 SV=4
3-ketoacyl-CoA thiolase A, peroxisomal OS=Mus musculus OX=10090 GN=Acaa1a PE=1 SV=1
Replication factor C subunit 3 OS=Mus musculus OX=10090 GN=Rfc3 PE=1 SV=1
tRNA (uracil-5-)-methyltransferase homolog A OS=Mus musculus OX=10090 GN=Trmt2a PE=1 SV=1
60S ribosomal protein L36 OS=Mus musculus OX=10090 GN=Rpl36 PE=1 SV=1
Transgelin-2 OS=Mus musculus OX=10090 GN=Tagln2 PE=1 SV=4
Polycomb protein EED OS=Mus musculus OX=10090 GN=Eed PE=1 SV=2
Ubiquitin carboxyl-terminal hydrolase OS=Mus musculus OX=10090 GN=Usp33 PE=1 SV=1
Transcriptional activator protein Pur-beta OS=Mus musculus OX=10090 GN=Purb PE=1 SV=3
MORC family CW-type zinc finger protein 3 OS=Mus musculus OX=10090 GN=Morc3 PE=1 SV=1
Hepatoma-derived growth factor-related protein 2 OS=Mus musculus OX=10090 GN=Hdgfl2 PE=1 SV=1
Exocyst complex component 1 OS=Mus musculus OX=10090 GN=Exoc1 PE=1 SV=1
Semaphorin-3C OS=Mus musculus OX=10090 GN=Sema3c PE=1 SV=2
Polycomb group RING finger protein 5 OS=Mus musculus OX=10090 GN=Pcgf5 PE=1 SV=1
DNA (cytosine-5)-methyltransferase 3A (Fragment) OS=Mus musculus OX=10090 GN=Dnmt3a PE=1 SV=1
Bcl-2-like protein 13 OS=Mus musculus OX=10090 GN=Bcl2l13 PE=1 SV=2
Copine-3 OS=Mus musculus OX=10090 GN=Cpne3 PE=1 SV=2
Diacylglycerol kinase eta OS=Mus musculus OX=10090 GN=Dgkh PE=1 SV=2
Cleavage and polyadenylation specificity factor subunit 4 OS=Mus musculus OX=10090 GN=Cpsf4 PE=1 SV=1
Elongator complex protein 3 OS=Mus musculus OX=10090 GN=Elp3 PE=1 SV=1
Adenylyl cyclase-associated protein 1 OS=Mus musculus OX=10090 GN=Cap1 PE=1 SV=4
Non-specific serine/threonine protein kinase OS=Mus musculus OX=10090 GN=Mast3 PE=1 SV=1
Eukaryotic translation initiation factor 5 OS=Mus musculus OX=10090 GN=Eif5 PE=1 SV=1
Cytochrome b-c1 complex subunit 1, mitochondrial OS=Mus musculus OX=10090 GN=Uqcrc1 PE=1 SV=2
Zinc finger protein 628 OS=Mus musculus OX=10090 GN=Znf628 PE=1 SV=2
14-3-3 protein gamma OS=Mus musculus OX=10090 GN=Ywhag PE=1 SV=2
V-type proton ATPase subunit B, brain isoform OS=Mus musculus OX=10090 GN=Atp6v1b2 PE=1 SV=1
Signal recognition particle receptor subunit beta OS=Mus musculus OX=10090 GN=Srprb PE=1 SV=1
G protein-coupled receptor 155 OS=Mus musculus OX=10090 GN=Gpr155 PE=1 SV=1
Golgin subfamily A member 4 OS=Mus musculus OX=10090 GN=Golga4 PE=1 SV=2

5-methylcytosine rRNA methyltransferase NSUN4 OS=Mus musculus OX=10090 GN=Nsun4 PE=1 SV=1
U3 small nucleolar ribonucleoprotein protein IMP4 OS=Mus musculus OX=10090 GN=Imp4 PE=2 SV=1
Histone-lysine N-methyltransferase EHMT1 OS=Mus musculus OX=10090 GN=Ehmt1 PE=1 SV=1
Splicing factor 3b, subunit 2 OS=Mus musculus OX=10090 GN=Sf3b2 PE=1 SV=1
DNA damage-binding protein 1 OS=Mus musculus OX=10090 GN=Ddb1 PE=1 SV=2
60S ribosomal protein L7-like 1 OS=Mus musculus OX=10090 GN=Rpl7l1 PE=1 SV=1

Abundance Ratio: (HOMO HA) / (CTRL)	Abundance Ratio P-Value: (HOMO HA) / (CTRL)	
16.494	1E-17	
77.551	1E-17	
0.07	1E-17	
68.643	1E-17	
73.642	1E-17	
0.01	1E-17	
80.081	1E-17	
56.488	1E-17	
66.295	1E-17	
62.08	1E-17	
83.942	1E-17	
0.17	1E-17	
100	1E-17	
92.915	1E-17	
0.01	1E-17	
0.01	1E-17	
0.15	1E-17	
0.01	1E-17	
0.01	1E-17	
0.01	1E-17	
0.01	1E-17	
0.01	1E-17	
0.01	1E-17	
0.01	1E-17	
0.01	1E-17	
0.01	1E-17	
0.01	1E-17	
0.01	1E-17	
0.147	1E-17	
100	1E-17	
0.01	1E-17	
0.01	1E-17	
0.01	1E-17	
0.01	1E-17	
0.01	1E-17	
0.01	1E-17	
0.01	1E-17	
0.01	1E-17	
100	1E-17	
44.716	1E-17	
0.01	1E-17	
0.045	1E-17	

0.01	1E-17	
0.01	1E-17	
0.01	1E-17	
100	1E-17	
0.01	1E-17	
0.01	1E-17	
0.01	1E-17	
0.01	1E-17	
0.01	1E-17	
0.01	1E-17	
0.01	1E-17	
0.01	1E-17	
0.156	1E-17	
100	1E-17	
0.01	1E-17	
0.01	1E-17	
0.01	1E-17	
0.01	1E-17	
0.01	1E-17	
0.01	1E-17	
0.01	1E-17	
100	1E-17	
0.01	1E-17	
0.01	1E-17	
0.01	1E-17	
100	1E-17	
0.01	1E-17	
100	1E-17	
0.107	1E-17	
0.01	1E-17	
100	1E-17	
0.01	1E-17	
0.085	1E-17	
0.01	1E-17	
0.068	1E-17	
0.01	1E-17	
100	1E-17	
0.01	1E-17	
0.01	1E-17	
0.01	1E-17	
0.01	1E-17	
0.01	1E-17	
0.01	1E-17	
0.01	1E-17	
0.01	1E-17	

0.01	1E-17	
0.01	1E-17	
100	1E-17	
0.01	1E-17	
0.08	1E-17	
0.01	1E-17	
0.01	1E-17	
0.01	1E-17	
0.01	1E-17	
0.01	1E-17	
100	1E-17	
0.01	1E-17	
0.01	1E-17	
8.136	1.33227E-15	
9.262	4.21885E-15	
11.741	2.9976E-14	
8.961	2.86438E-13	
0.244	1.12998E-12	
0.2	1.94555E-12	
7.247	5.08149E-12	
0.238	1.59259E-11	
0.234	4.51656E-11	
5.716	8.09475E-11	
0.22	2.26095E-09	
5.557	4.11068E-09	
5.889	4.99944E-09	
5.4	5.77505E-09	
5.198	1.39563E-08	
5.264	1.42251E-08	
6.001	1.92842E-08	
5.166	2.1606E-08	
4.574	2.59529E-08	
4.431	3.53201E-08	
4.849	9.67929E-08	
4.882	1.26298E-07	
5.988	1.47603E-07	
4.459	2.27803E-07	
0.351	2.50547E-07	
4.43	2.6305E-07	
3.919	2.96164E-07	
0.324	3.95129E-07	
0.306	5.65374E-07	
0.282	5.79538E-07	
5.089	8.42763E-07	
0.406	9.07966E-07	

0.346	9.1051E-07	
0.342	1.14684E-06	
3.701	1.34143E-06	
4.857	1.69991E-06	
3.711	2.13865E-06	
5.175	2.5918E-06	
0.423	2.70402E-06	
3.528	3.22481E-06	
0.415	3.31464E-06	
4.391	3.38761E-06	
3.449	3.8248E-06	
3.704	4.44211E-06	
3.886	6.77327E-06	
0.353	6.974E-06	
3.814	9.45394E-06	
3.764	1.19466E-05	
4.01	1.83738E-05	
3.207	1.86584E-05	
0.379	2.00197E-05	
5.041	2.13285E-05	
3.173	2.1383E-05	
3.631	2.24276E-05	
3.233	2.31076E-05	
0.472	2.73347E-05	
0.43	2.81127E-05	
3.617	2.97962E-05	
0.437	3.34673E-05	
0.377	3.645E-05	
4.337	4.31685E-05	
0.469	5.01869E-05	
3.091	5.08549E-05	
3.093	6.281E-05	
3.009	6.47068E-05	
3.36	8.10866E-05	
3.373	9.01113E-05	
3.046	0.000109898	
3.612	0.000148707	
0.526	0.000150896	
0.516	0.000158858	
3.206	0.000169701	
0.457	0.000177363	
3.195	0.00017944	
3.564	0.000185802	
3.736	0.000207269	
2.792	0.000229721	

3.333	0.000234321	
0.456	0.000234458	
4.086	0.000261989	
0.475	0.000323221	
0.542	0.000336975	
0.446	0.000379197	
2.988	0.000425878	
2.786	0.000440644	
2.699	0.000484132	
3.835	0.00049065	
2.835	0.000493161	
2.817	0.000506474	
2.835	0.00052266	
0.525	0.000524536	
2.995	0.000552624	
0.517	0.000588606	
0.559	0.000636016	
2.931	0.000639997	
2.636	0.000663611	
2.632	0.000692626	
0.533	0.000712951	
2.652	0.000723273	
2.654	0.000754762	
0.487	0.000821161	
0.574	0.000824254	
0.538	0.000825747	
2.79	0.000859228	
3.038	0.000897918	
2.745	0.00092681	
2.747	0.000930568	
0.513	0.00095069	
2.844	0.000973097	
0.548	0.001031003	
2.866	0.001054095	
0.555	0.001140001	
2.993	0.001151342	
2.576	0.00119559	
0.581	0.001206283	
2.845	0.001240347	
2.786	0.001287681	
2.562	0.001367529	
3.547	0.001367771	
2.652	0.001426165	
2.866	0.001470059	
0.541	0.001477673	

2.756	0.001488846	
2.744	0.00150804	
2.781	0.001602376	
0.539	0.001653236	
0.5	0.001703266	
2.655	0.00172741	
0.445	0.001734015	
0.601	0.001774981	
0.578	0.001846725	
0.546	0.001898729	
0.522	0.001928985	
0.541	0.001932393	
2.692	0.002022013	
0.544	0.002068722	
2.607	0.002090419	
0.612	0.002103802	
0.609	0.0021994	
2.921	0.002263566	
0.609	0.002287563	
3.177	0.002386445	
2.596	0.002420602	
2.879	0.002497765	
0.603	0.002859072	
2.649	0.002912351	
0.579	0.002917204	
2.608	0.003030097	
0.562	0.003178246	
2.595	0.003197127	
0.522	0.003233267	
0.628	0.003327905	
2.617	0.0033606	
0.492	0.003384261	
2.577	0.003656599	
0.494	0.003910689	
0.506	0.003915786	
2.67	0.004043313	
0.616	0.004087358	
3.105	0.004114265	
2.511	0.004119705	
2.321	0.004302049	
2.415	0.004412758	
0.615	0.004515596	
0.574	0.004789646	
2.541	0.004821573	
2.646	0.005011977	

2.346	0.005052617	
0.648	0.005164574	
2.462	0.005270462	
0.619	0.005461829	
0.56	0.005551073	
2.463	0.005581998	
2.351	0.00570595	
0.499	0.005752139	
2.517	0.005788308	
0.646	0.0058108	
2.575	0.005923659	
0.605	0.00595691	
0.502	0.005966394	
2.464	0.006036907	
2.372	0.006074841	
2.664	0.006191857	
0.601	0.006333099	
0.583	0.006341363	
0.518	0.006521419	
2.444	0.006641783	
0.598	0.006731938	
0.575	0.006762808	
0.611	0.006825474	
0.66	0.006932741	
0.669	0.007088374	
0.66	0.007117056	
2.529	0.007167153	
0.666	0.007212709	
0.631	0.00740467	
0.66	0.00740571	
0.528	0.007439948	
2.257	0.007583521	
2.374	0.007669522	
0.605	0.007714008	
0.583	0.007940677	
0.606	0.008067545	
2.232	0.008108634	
2.378	0.008127706	
2.402	0.008128077	
2.422	0.008336027	
2.524	0.008509578	
2.511	0.008594163	
2.261	0.008707044	
2.387	0.008716501	
0.625	0.008778921	

0.672	0.008951223	
2.423	0.009005995	
0.62	0.009093616	
0.665	0.009213997	
0.61	0.009529734	
2.459	0.009532597	
2.46	0.009582148	
0.676	0.009865583	
0.602	0.009869411	
2.414	0.009947974	
2.358	0.010017698	
0.638	0.010200317	
2.181	0.010207869	
0.661	0.010636596	
2.343	0.010715446	
2.343	0.010741141	
0.688	0.010967239	
0.625	0.010976135	
2.443	0.011321141	
0.66	0.011357131	
0.543	0.01144748	
2.31	0.011803975	
0.634	0.011820498	
0.69	0.011856987	
0.545	0.011941549	
2.318	0.012081115	
0.688	0.012115813	
0.544	0.012281144	
2.314	0.012296929	
2.287	0.012402399	
2.371	0.01240467	
2.194	0.01245235	
2.31	0.012535096	
2.789	0.012876975	
2.373	0.012953613	
0.598	0.013292012	
0.675	0.013406448	
0.636	0.013422123	
0.564	0.013456957	
0.688	0.013826578	
2.157	0.013848578	
0.679	0.01416356	
2.311	0.014359174	
2.408	0.014370705	
0.705	0.014520254	

2.382	0.014537559	
0.679	0.0145641	
0.642	0.014793435	
2.274	0.014831095	
2.121	0.014919043	
0.677	0.015016936	
2.25	0.015056577	
0.705	0.015385176	
2.266	0.015441563	
2.308	0.015482596	
2.193	0.015767173	
2.287	0.015770709	
2.26	0.015829369	
0.649	0.016106928	
0.567	0.01633512	
0.702	0.016487647	
2.487	0.016596686	
0.625	0.016692534	
2.245	0.017041595	
2.22	0.017097937	
0.71	0.017109534	
2.228	0.017480218	
2.261	0.01750758	
2.228	0.017879865	
0.63	0.017887105	
2.086	0.017894164	
0.709	0.017911737	
2.683	0.01797172	
0.707	0.018281475	
0.657	0.018348522	
0.71	0.018457296	
2.189	0.018700202	
0.658	0.018734414	
0.657	0.018864421	
0.659	0.019440507	
0.696	0.01968756	
0.713	0.019722979	
2.211	0.019898033	
2.206	0.020409714	
2.206	0.020425125	
0.678	0.020430013	
0.722	0.02044914	
2.612	0.020460344	
2.252	0.020951955	
2.492	0.021126217	

0.7	0.021274737	
0.668	0.021510009	
0.64	0.021547467	
0.698	0.021756222	
2.043	0.021875362	
2.298	0.021943124	
0.7	0.022103647	
0.724	0.022612817	
0.67	0.022820154	
0.68	0.022974106	
2.176	0.023416839	
2.29	0.023445699	
2.268	0.023730078	
0.725	0.023985631	
2.161	0.024002444	
2.426	0.024246032	
0.699	0.024332192	
0.741	0.02437812	
0.682	0.024386236	
2.102	0.025049641	
0.731	0.025065972	
0.727	0.025066206	
2.036	0.025640584	
2.192	0.025700842	
0.675	0.025963691	
2.341	0.026012116	
2.193	0.026177052	
2.244	0.026383578	
2.063	0.026976716	
2.143	0.027250088	
2.033	0.02741465	
2.171	0.027602519	
2.55	0.027657922	
0.734	0.027696234	
0.735	0.02769899	
2.004	0.027906685	
2.529	0.028315235	
0.704	0.028335686	
0.604	0.028533422	
0.617	0.029258611	
2.529	0.02932076	
0.668	0.029676145	
2.12	0.029735506	
2.113	0.029752785	
2.124	0.029782843	

0.736	0.029943059	
0.607	0.029960472	
2.312	0.030154482	
2.084	0.030275848	
2.53	0.030539795	
0.756	0.030549995	
0.742	0.030742932	
0.748	0.030798965	
2.157	0.031064929	
0.707	0.031161549	
2.113	0.031365087	
0.747	0.031419305	
0.74	0.031587932	
0.708	0.031723821	
0.745	0.031828115	
2.173	0.031836604	
2.447	0.032021607	
2.223	0.03202203	
2.272	0.03245979	
0.679	0.032747832	
0.723	0.032815985	
0.719	0.033565591	
0.729	0.033762156	
2.2	0.033863804	
2.094	0.034228361	
0.712	0.034750532	
0.7	0.035340376	
0.7	0.035430722	
2.125	0.035644458	
0.666	0.035680713	
0.758	0.035752719	
2.129	0.035760375	
0.701	0.035866385	
2.081	0.036198455	
2.059	0.036213008	
2.002	0.03623132	
2.027	0.036234106	
0.76	0.036347377	
2.078	0.036743216	
0.704	0.03729136	
0.705	0.037562261	
0.684	0.037595848	
0.756	0.037668551	
2.318	0.03790767	
2.274	0.037992087	

0.743	0.038265235	
2.069	0.038303225	
0.76	0.038611322	
2.25	0.03890019	
1.983	0.03893689	
0.761	0.039219493	
1.944	0.039425867	
1.981	0.039562284	
0.744	0.039599738	
1.937	0.039931203	
0.744	0.040158827	
2.14	0.040187714	
2.169	0.04036165	
0.744	0.040603542	
2.089	0.040663832	
0.718	0.040765206	
0.711	0.04090691	
2.123	0.041296634	
2.003	0.041336149	
0.691	0.041344021	
2.005	0.041605811	
0.769	0.041663295	
0.766	0.041798814	
2.057	0.041816312	
0.681	0.042187348	
0.768	0.042747391	
0.758	0.042804992	
2.058	0.042818266	
0.659	0.043275947	
2.041	0.043491909	
2.051	0.043582325	
0.716	0.043677225	
0.637	0.04393077	
2.007	0.043953908	
2.194	0.043959365	
2.038	0.044031266	
2.065	0.044119906	
0.718	0.044791598	
0.773	0.045072348	
0.719	0.045077669	
0.76	0.045091132	
0.735	0.045236079	
2	0.045257804	
1.993	0.045305755	
2.029	0.045306801	

2.003	0.04552719	
2.07	0.045535537	
2.051	0.045569746	
2.082	0.045672318	
2.369	0.045695332	
1.989	0.045732589	
2.029	0.046178587	
2.027	0.046188098	
2.027	0.046246513	
2.11	0.046351657	
0.753	0.046603671	
2.025	0.046698701	
0.779	0.046743662	
2.091	0.047092797	
2.116	0.047303005	
0.729	0.047369203	
2.246	0.047528001	
0.764	0.047841166	
2.018	0.048116925	
2.075	0.048503837	
1.974	0.048748638	
0.735	0.048818014	
0.74	0.04908069	
2.077	0.049331982	
0.726	0.049701903	
0.758	0.049773567	
0.651	0.050024356	
2.007	0.050074414	
0.727	0.050125126	
0.727	0.050221357	
0.727	0.050377295	
2.08	0.050450375	
1.897	0.050536219	
2.346	0.050865563	
2.128	0.050911645	
1.891	0.051119102	
1.965	0.051408296	
2.003	0.051484402	
0.785	0.051815375	
0.73	0.051913064	
0.73	0.05196289	
2.239	0.05197762	
0.794	0.052221072	
0.765	0.052241735	
1.963	0.052402479	

2.02	0.052459032	
1.904	0.052640219	
1.998	0.052741363	
1.951	0.052952095	
2.026	0.053084782	
0.739	0.05317771	
1.981	0.053864626	
0.786	0.054196019	
0.788	0.055007764	
2.073	0.055071811	
1.987	0.055363697	
0.777	0.055399925	
1.983	0.055507991	
1.962	0.055540782	
1.986	0.055667358	
0.736	0.055832278	
0.786	0.056349641	
0.663	0.05637778	
2.018	0.056562631	
2.077	0.056693112	
0.709	0.05676008	
1.978	0.057632132	
1.941	0.057672161	
1.869	0.057939	
2.035	0.058156383	
1.9	0.058725333	
2.271	0.058943128	
2.086	0.05904084	
1.856	0.059280857	
0.797	0.059768871	
2.208	0.059872161	
2.004	0.059935024	
1.969	0.060082317	
0.743	0.060970327	
0.714	0.061218764	
0.707	0.0615144	
0.777	0.062144684	
1.929	0.062248245	
0.803	0.062425401	
0.777	0.062597491	
2.226	0.062831546	
1.889	0.06322438	
1.849	0.06338987	
1.969	0.063611827	
0.723	0.063776559	

1.887	0.063797295	
1.954	0.064146689	
1.954	0.064208516	
1.971	0.064504003	
1.951	0.06502218	
0.782	0.065102478	
0.75	0.06575475	
2.118	0.065785823	
0.715	0.06616837	
0.797	0.066438295	
0.79	0.066497211	
2.245	0.066818404	
0.789	0.067125927	
1.944	0.067210493	
1.851	0.067221783	
1.879	0.067377269	
2.063	0.067424457	
2.028	0.067492078	
2.01	0.067784433	
0.739	0.067854074	
2.171	0.067929285	
0.793	0.068219066	
0.77	0.068605576	
0.73	0.068808812	
0.754	0.069083199	
2.103	0.069441929	
0.682	0.069715372	
1.955	0.069840159	
1.963	0.069856131	
0.805	0.070234279	
1.933	0.070445008	
0.788	0.070782347	
0.808	0.071211996	
0.788	0.071663364	
1.928	0.071971604	
1.928	0.072022732	
0.746	0.072275068	
0.684	0.072745188	
2.093	0.072789646	
1.861	0.073648355	
2.023	0.073707315	
0.819	0.074046388	
1.921	0.074154097	
1.994	0.074528611	
1.954	0.074536018	

0.817	0.074600037	
2.233	0.074638186	
0.789	0.075059238	
0.778	0.07519159	
0.813	0.075363653	
0.77	0.075594998	
0.813	0.075760141	
2.017	0.076298236	
1.82	0.076337616	
1.978	0.076353006	
0.821	0.076418037	
2.171	0.077154021	
1.981	0.077328775	
1.911	0.077398464	
0.807	0.077957949	
1.837	0.078337494	
1.861	0.078483476	
1.907	0.078680512	
0.767	0.079541988	
0.763	0.079793628	
0.808	0.079852389	
0.805	0.080744	
1.949	0.081325996	
1.876	0.081584609	
2.105	0.081593452	
1.899	0.081760918	
0.769	0.081786264	
0.736	0.081943279	
0.82	0.082215259	
2.054	0.082381039	
1.944	0.082806592	
0.807	0.083066181	
0.749	0.083113851	
0.835	0.08312614	
0.805	0.083132609	
1.925	0.083213412	
1.796	0.083307123	
1.893	0.083854958	
0.706	0.083949553	
1.968	0.084223573	
0.812	0.084387822	
0.824	0.084758846	
0.781	0.084983628	
2.033	0.085551364	
2.127	0.085781448	

2.088	0.085921061	
0.761	0.086145974	
1.858	0.086210144	
0.704	0.086535995	
0.829	0.08661839	
1.924	0.086693954	
2.1	0.086929641	
1.957	0.087422605	
0.817	0.087537099	
1.919	0.088057988	
1.895	0.088849022	
0.807	0.088893529	
2.137	0.088998869	
1.801	0.089620119	
1.812	0.090052829	
1.943	0.09035071	
0.829	0.090500314	
0.843	0.090520181	
1.846	0.091097311	
1.873	0.091271212	
1.8	0.091533756	
0.715	0.091985052	
0.78	0.092075188	
0.821	0.09233532	
1.835	0.092406575	
0.833	0.092960872	
1.944	0.093027474	
0.786	0.093109577	
1.813	0.093396842	
0.716	0.093535367	
1.906	0.094318836	
0.783	0.094616598	
0.783	0.094732455	
1.958	0.095207216	
0.83	0.095230114	
0.817	0.095554073	
1.862	0.095828672	
1.862	0.095870482	
0.76	0.095932193	
1.783	0.096002395	
0.84	0.096580903	
0.834	0.097023507	
0.783	0.097173434	
1.861	0.097338144	
1.863	0.097419287	

0.837	0.097569943	
0.849	0.097602667	
1.858	0.097655117	
0.824	0.098846643	
0.778	0.098995631	
0.726	0.0992543	
0.84	0.099376785	
1.86	0.099858788	
0.761	0.100135029	
1.856	0.100489934	
0.724	0.100854664	
0.806	0.101050564	
1.889	0.101129675	
0.817	0.101200885	
2.069	0.101487444	
0.767	0.101563067	
0.831	0.101767249	
0.838	0.101972159	
0.83	0.101995391	
0.853	0.102600243	
0.845	0.102867627	
1.875	0.103027482	
2.071	0.103313664	
1.908	0.103408635	
1.77	0.103500446	
0.775	0.103551084	
1.824	0.10356489	
1.919	0.103794267	
1.899	0.104024656	
0.81	0.10402807	
1.884	0.104112429	
0.793	0.104307085	
0.817	0.104384903	
0.829	0.104903324	
1.788	0.105170896	
1.84	0.105261306	
1.831	0.10565391	
0.795	0.106097399	
0.852	0.106346392	
1.754	0.106402778	
1.837	0.106609115	
0.767	0.106626399	
1.766	0.107038646	
0.858	0.107156581	
0.796	0.107399492	

1.849	0.107575183	
1.842	0.107694525	
1.818	0.107729342	
0.73	0.108082373	
0.804	0.10832962	
0.776	0.108449964	
1.841	0.108884652	
0.778	0.109011861	
1.831	0.109215011	
0.865	0.10935093	
1.806	0.109661124	
0.813	0.109887391	
0.806	0.11027504	
0.843	0.110477593	
0.799	0.110597122	
0.835	0.110784882	
0.776	0.111527177	
1.766	0.11159401	
0.8	0.111914217	
1.75	0.111978722	
1.824	0.112849201	
0.834	0.113025543	
1.778	0.113247571	
0.802	0.113353955	
0.866	0.114492077	
0.784	0.114580671	
1.967	0.114791385	
1.846	0.114854096	
1.78	0.1151512	
0.78	0.115697853	
0.782	0.115979056	
1.816	0.116448317	
1.782	0.116478584	
0.837	0.116480264	
1.955	0.116670468	
1.777	0.116712729	
1.78	0.117012371	
1.766	0.117740281	
0.856	0.11794914	
1.834	0.11798638	
0.754	0.118258164	
0.854	0.118623518	
0.856	0.118679851	
0.865	0.118705569	
1.812	0.118706006	

1.812	0.118712688	
1.853	0.118851117	
1.811	0.119066968	
1.811	0.119122603	
1.747	0.119392221	
1.81	0.119847724	
1.856	0.120349932	
0.864	0.120669096	
2.014	0.120705986	
1.774	0.120926058	
0.838	0.121028223	
0.742	0.12113722	
1.826	0.121790669	
0.786	0.122211151	
0.789	0.12224951	
1.744	0.122315862	
0.842	0.122321601	
0.757	0.122388992	
1.937	0.122600505	
0.874	0.122688447	
1.812	0.122695811	
0.849	0.122913689	
0.748	0.123248586	
1.812	0.124151778	
1.851	0.124195225	
1.716	0.124310311	
0.811	0.124350922	
1.8	0.124666602	
0.812	0.124861951	
2.006	0.124901637	
1.858	0.12520866	
0.845	0.125267567	
0.858	0.125388767	
1.823	0.125538257	
1.729	0.125602869	
1.894	0.125714947	
1.765	0.12574446	
1.744	0.12575356	
1.891	0.125921962	
2.043	0.126568223	
0.87	0.126782355	
0.751	0.127091705	
0.859	0.127580819	
0.801	0.127697992	
1.811	0.127955026	

2.007	0.12806765	
1.744	0.128074786	
0.782	0.128298877	
0.794	0.129726225	
1.842	0.129965195	
0.805	0.130435099	
0.816	0.130466768	
1.886	0.130623359	
0.851	0.130833417	
1.85	0.131037874	
0.817	0.13141839	
0.809	0.131639754	
0.848	0.132542151	
0.757	0.132698464	
0.863	0.132843611	
0.824	0.132862221	
0.853	0.133648438	
1.783	0.133876636	
1.743	0.13407	
1.782	0.134666883	
1.926	0.135115834	
0.821	0.135580026	
1.817	0.135868067	
1.712	0.136170382	
0.829	0.136580672	
2.014	0.137158433	
1.977	0.137278813	
1.866	0.137522111	
1.719	0.137547369	
0.802	0.137574657	
1.777	0.13767555	
0.804	0.137822642	
0.808	0.138185626	
0.877	0.139134191	
1.774	0.139197607	
1.729	0.13959323	
0.863	0.139662004	
1.773	0.139899292	
1.826	0.141539823	
1.706	0.141672031	
0.857	0.141842903	
1.769	0.142184937	
0.826	0.142275919	
1.801	0.142469593	
2.003	0.142575089	

1.737	0.14280057	
1.801	0.142823316	
0.826	0.142850341	
0.871	0.14294159	
1.782	0.14347756	
1.747	0.143878496	
0.887	0.143891614	
1.761	0.144000115	
0.848	0.144113282	
0.875	0.144350286	
1.815	0.144358434	
1.726	0.144913913	
1.686	0.144923285	
0.866	0.145077381	
0.874	0.145140945	
1.693	0.145763611	
0.848	0.146029083	
1.761	0.146709542	
1.79	0.1468166	
1.766	0.146859574	
0.869	0.147005195	
0.818	0.147016599	
1.889	0.147080672	
0.884	0.147278522	
0.83	0.14736126	
0.887	0.147522718	
0.83	0.147760001	
1.919	0.1477752	
1.703	0.147973501	
0.893	0.148208046	
1.759	0.148240392	
0.867	0.148271772	
1.759	0.148337593	
1.826	0.148675072	
0.807	0.148724813	
0.877	0.148740382	
0.87	0.148753403	
1.763	0.149250484	
1.754	0.149656166	
1.889	0.150173216	
0.813	0.150193302	
1.978	0.15044888	
0.865	0.150449395	
1.754	0.151061463	
1.982	0.1510999	

0.781	0.151284113	
0.773	0.152091271	
1.801	0.152749091	
1.752	0.152775187	
1.755	0.153193264	
0.835	0.153804918	
1.775	0.154078443	
1.74	0.154317404	
1.877	0.154751443	
0.87	0.154788308	
0.872	0.155367842	
0.822	0.15551927	
1.768	0.155681468	
0.864	0.1557077	
1.675	0.155814764	
1.758	0.156054715	
1.746	0.156180858	
0.894	0.15628177	
1.808	0.15699421	
0.887	0.157136191	
1.968	0.157259738	
1.744	0.157324704	
1.783	0.157495638	
1.688	0.158126519	
0.884	0.158268131	
0.812	0.158410787	
0.851	0.158449669	
0.903	0.15900068	
1.784	0.159324141	
0.817	0.159519054	
0.877	0.15956748	
0.872	0.159573778	
0.839	0.159691261	
0.817	0.159846835	
1.878	0.160042234	
1.776	0.160507736	
0.879	0.160644632	
1.739	0.16066171	
1.69	0.16077519	
0.876	0.160839568	
1.786	0.160981447	
0.885	0.161420862	
0.841	0.162139043	
1.737	0.162171591	
1.732	0.162350662	

0.899	0.162578697	
0.841	0.162601163	
0.906	0.162800025	
1.821	0.163276638	
0.905	0.163328285	
1.735	0.163328301	
1.791	0.163620505	
1.746	0.1640442	
1.734	0.164304606	
0.892	0.164359952	
0.88	0.165317954	
0.789	0.165607506	
1.671	0.165695207	
1.748	0.166062506	
0.844	0.166877851	
0.788	0.167185562	
1.794	0.16728602	
0.796	0.167312505	
0.86	0.1673759	
0.829	0.167648241	
1.701	0.167757978	
1.756	0.168371724	
0.885	0.168671454	
1.752	0.168737635	
1.768	0.168866641	
0.9	0.169000999	
0.846	0.16900295	
0.878	0.169617999	
0.86	0.169869106	
0.899	0.169957112	
0.9	0.170327663	
1.698	0.171556376	
0.899	0.172070049	
1.748	0.172120543	
0.832	0.172236027	
0.91	0.172611562	
0.848	0.172641703	
1.744	0.172897583	
1.719	0.173297137	
0.849	0.173564363	
1.72	0.174018243	
1.72	0.174210973	
0.893	0.174545785	
0.896	0.174886387	
1.719	0.1749714	

1.75	0.175093726	
1.74	0.17524301	
1.743	0.175264511	
1.75	0.175270943	
0.802	0.175508411	
1.717	0.175965364	
1.74	0.176169021	
0.878	0.17634104	
0.851	0.176361808	
1.734	0.176666224	
1.687	0.176736909	
0.89	0.1767557	
0.902	0.176872613	
1.716	0.176880707	
1.69	0.177060893	
1.923	0.177135523	
1.729	0.177249136	
0.852	0.177443853	
0.852	0.177455681	
0.853	0.177647129	
1.662	0.177755561	
1.8	0.177776927	
1.688	0.177854571	
1.871	0.178225915	
1.746	0.178438863	
1.738	0.178668623	
1.727	0.178698101	
1.836	0.178773542	
0.894	0.179202333	
0.853	0.179298152	
0.807	0.18078388	
1.719	0.181150269	
1.648	0.181594968	
1.707	0.181719571	
0.859	0.182210652	
1.703	0.182304884	
0.855	0.182432656	
0.886	0.182432673	
1.683	0.182739003	
0.857	0.183045893	
0.845	0.18361574	
0.891	0.183895701	
0.841	0.184006627	
0.843	0.184110121	
1.739	0.184120475	

0.892	0.184345114	
0.857	0.184381206	
1.784	0.184513967	
1.738	0.185572829	
1.703	0.186200674	
0.858	0.186975638	
0.906	0.187226667	
1.729	0.187349824	
1.709	0.18772465	
1.7	0.188312541	
0.876	0.188377858	
0.877	0.188734137	
1.7	0.188937806	
0.905	0.189449548	
1.699	0.189538791	
0.89	0.189560431	
1.859	0.189752362	
0.897	0.190059059	
1.74	0.190301127	
1.736	0.190712296	
1.704	0.191727457	
0.865	0.191801169	
0.905	0.191996833	
0.848	0.192021223	
0.854	0.192436932	
0.914	0.192902398	
1.719	0.193705438	
1.648	0.193708452	
1.68	0.19371006	
0.881	0.193725059	
1.637	0.193925571	
0.905	0.194193963	
0.923	0.194674642	
1.737	0.195245968	
1.647	0.195569471	
0.864	0.195582795	
1.782	0.195628151	
0.87	0.195870115	
1.885	0.196340805	
1.758	0.196363951	
1.708	0.196414908	
0.865	0.196530249	
0.879	0.196552272	
1.744	0.196601766	
0.852	0.196795265	

0.908	0.197130875	
0.865	0.1971987	
1.739	0.197233448	
0.915	0.197238145	
1.722	0.197329448	
0.915	0.197382265	
0.896	0.198261354	
0.814	0.198883575	
1.715	0.198884048	
0.918	0.19915085	
1.687	0.199155334	
1.669	0.19916132	
0.927	0.199184495	
0.893	0.199556667	
0.837	0.199586483	
1.733	0.199613126	
0.909	0.199961432	
1.7	0.200531673	
1.765	0.200756882	
0.888	0.201536092	
1.712	0.201959143	
1.737	0.20230474	
0.887	0.202558438	
1.682	0.202716202	
1.681	0.203423373	
0.817	0.203554032	
0.916	0.203603079	
0.869	0.203621764	
0.869	0.203656826	
0.869	0.203804196	
0.912	0.203997967	
1.681	0.204007567	
1.737	0.204442682	
0.87	0.204606299	
1.66	0.204634204	
0.87	0.205271406	
0.921	0.20538103	
0.925	0.205684696	
0.863	0.205895639	
1.695	0.205973972	
1.776	0.20667497	
0.914	0.206760655	
1.65	0.207573139	
1.734	0.207893553	
0.868	0.208291963	

0.848	0.20845295	
1.626	0.208659985	
1.729	0.209019932	
1.732	0.209146875	
0.873	0.20993865	
0.924	0.210024865	
0.856	0.210201116	
0.903	0.2105955	
0.874	0.210676741	
1.686	0.210826567	
1.676	0.211437114	
0.885	0.211813975	
0.858	0.211985735	
0.91	0.212320425	
0.914	0.2123791	
0.908	0.212443602	
0.859	0.212906198	
1.713	0.21364407	
0.875	0.213792745	
1.716	0.214276285	
0.918	0.214454681	
1.724	0.214537447	
1.773	0.214622472	
1.692	0.214916929	
1.668	0.215380196	
1.718	0.216020465	
0.876	0.216036725	
0.854	0.216131252	
1.859	0.216521974	
1.684	0.216627403	
1.665	0.216803411	
0.929	0.217110438	
1.653	0.217331031	
1.619	0.217501381	
1.664	0.218141468	
1.639	0.21822524	
1.63	0.218248529	
0.863	0.21837989	
1.643	0.218527221	
1.648	0.218896306	
1.848	0.219401447	
0.916	0.219425144	
1.739	0.219558971	
1.662	0.219928084	
1.819	0.219980355	

0.922	0.220151273	
0.918	0.220276972	
1.661	0.220367473	
1.678	0.220415547	
1.661	0.220521802	
1.688	0.220842527	
0.921	0.220906976	
1.688	0.221217346	
0.88	0.221238632	
1.66	0.221416483	
1.637	0.222020906	
0.903	0.222068423	
0.881	0.222217622	
0.827	0.222707689	
0.889	0.222956712	
0.926	0.223177922	
1.636	0.223483377	
1.725	0.223623216	
1.616	0.223634668	
1.768	0.223739036	
0.882	0.223786522	
0.888	0.223961561	
1.674	0.224334487	
1.702	0.225204932	
0.847	0.225489507	
1.613	0.225981406	
1.636	0.226065155	
1.635	0.226152396	
0.901	0.226488385	
1.674	0.226853124	
1.682	0.22719692	
0.884	0.22777308	
0.884	0.227964009	
1.652	0.228260954	
1.652	0.228585614	
1.652	0.228633066	
1.625	0.228938338	
0.833	0.229384727	
0.929	0.229398943	
0.929	0.229512436	
0.94	0.229725645	
1.627	0.230083524	
1.714	0.230417539	
1.649	0.231080854	
0.872	0.23142383	

0.9	0.231603908	
1.693	0.231753052	
1.607	0.231938977	
0.923	0.231999093	
1.84	0.232070798	
1.749	0.232077473	
1.711	0.232106255	
0.945	0.232938237	
1.643	0.232995598	
1.647	0.233020768	
1.657	0.233530211	
1.646	0.233632527	
0.935	0.23412291	
1.603	0.234262098	
0.873	0.234287169	
1.628	0.234299212	
0.924	0.234563755	
0.917	0.234756526	
1.661	0.234910599	
0.876	0.235132449	
1.588	0.235479218	
1.673	0.236001392	
1.645	0.236133447	
0.837	0.236195155	
1.606	0.23643442	
1.625	0.236440412	
1.697	0.237545473	
1.77	0.238539312	
0.845	0.238691127	
0.898	0.238937665	
0.86	0.239127458	
0.931	0.239253524	
0.931	0.239292958	
0.891	0.239330259	
1.628	0.239380688	
0.927	0.24017233	
0.891	0.240580672	
0.94	0.240970888	
0.879	0.241096216	
1.7	0.241752375	
1.664	0.24190083	
0.879	0.242414976	
0.927	0.242736337	
1.585	0.243727632	
1.611	0.243822628	

0.909	0.244000589	
1.661	0.244069633	
1.635	0.244105673	
0.894	0.244270865	
0.9	0.24433183	
1.639	0.244613518	
1.664	0.244876899	
1.663	0.245756466	
0.945	0.246176262	
1.679	0.246278941	
0.884	0.246854712	
0.895	0.246858727	
1.632	0.246865724	
0.93	0.24706846	
1.676	0.247170137	
0.877	0.247258096	
0.847	0.247287444	
0.934	0.247994722	
0.844	0.248158069	
1.638	0.248377845	
1.592	0.248880233	
1.63	0.248981944	
0.931	0.250508754	
1.641	0.250791671	
1.628	0.250832866	
0.937	0.251037266	
1.653	0.251344044	
1.706	0.251418802	
1.656	0.251466075	
0.932	0.251656198	
1.579	0.251877197	
0.917	0.25222557	
1.655	0.252233458	
0.898	0.25239488	
0.942	0.252492845	
0.935	0.25260684	
0.939	0.252887558	
0.909	0.253014461	
1.599	0.253254997	
1.626	0.253301472	
0.947	0.253344735	
1.614	0.253664621	
0.88	0.254069761	
0.852	0.254213523	
1.677	0.25445469	

0.94	0.254473617	
1.579	0.254935308	
0.855	0.254983952	
0.883	0.255043519	
1.672	0.25571461	
1.623	0.256013173	
0.961	0.256131946	
1.722	0.257363668	
0.901	0.257515873	
1.623	0.257524298	
1.651	0.257949624	
1.621	0.258040952	
1.655	0.258059991	
1.794	0.258510391	
0.902	0.258590637	
1.62	0.258669868	
1.648	0.259081494	
0.935	0.259090081	
0.938	0.260741176	
0.95	0.260872567	
1.627	0.260965823	
0.944	0.261536347	
1.617	0.261670167	
1.663	0.261768427	
1.646	0.261882184	
0.952	0.261950655	
0.893	0.26210351	
0.857	0.263890367	
1.615	0.264042407	
1.737	0.264394093	
0.902	0.264503912	
0.958	0.265066918	
1.658	0.265125794	
1.589	0.265287385	
0.911	0.266037412	
0.92	0.266130446	
0.946	0.266685622	
0.947	0.267054805	
0.947	0.267421508	
0.923	0.267539605	
1.652	0.267738361	
0.944	0.268206275	
0.912	0.268780826	
1.647	0.268837734	
0.927	0.268861086	

0.907	0.269300329	
1.586	0.269790803	
1.609	0.269954251	
1.602	0.270016869	
0.897	0.270107061	
0.889	0.270204337	
0.955	0.270281371	
0.908	0.270328614	
1.729	0.270469653	
0.951	0.270602034	
1.612	0.271174905	
0.938	0.271185423	
1.607	0.271817101	
0.928	0.271998188	
0.876	0.272054603	
0.942	0.272144418	
1.651	0.272508247	
0.899	0.272716047	
1.579	0.273126056	
1.592	0.273425675	
0.91	0.273471833	
0.867	0.274157825	
0.911	0.274946636	
1.561	0.275739983	
0.936	0.276246479	
0.898	0.276859448	
1.602	0.277546008	
1.698	0.277764719	
1.635	0.277804904	
1.643	0.278487105	
0.913	0.278631396	
0.901	0.279164694	
1.584	0.279916692	
1.6	0.280054994	
0.894	0.280062932	
1.6	0.28006578	
1.621	0.280132972	
0.956	0.280280983	
0.913	0.280351599	
0.96	0.280623331	
1.599	0.280818537	
0.914	0.280996169	
0.898	0.281247461	
0.899	0.281433635	
0.914	0.281573899	

1.638	0.281708497	
1.628	0.281873165	
0.905	0.281878884	
1.76	0.281931875	
0.953	0.281970763	
0.953	0.281970763	
0.9	0.281981165	
0.914	0.282158079	
1.598	0.282320295	
0.96	0.282329137	
1.621	0.28260565	
1.626	0.282666009	
1.636	0.282732119	
1.597	0.282982477	
0.87	0.283836475	
0.954	0.284109071	
1.643	0.284110809	
1.654	0.285141167	
1.758	0.285532956	
0.917	0.286599704	
0.916	0.286816843	
1.692	0.286901435	
0.907	0.287067987	
1.759	0.287116791	
1.606	0.287847941	
1.639	0.288285638	
0.958	0.288313216	
1.583	0.288380894	
0.954	0.288403071	
1.621	0.289832221	
1.633	0.290167814	
1.555	0.290814225	
0.959	0.290820139	
1.583	0.291240734	
1.618	0.291318324	
1.575	0.291643737	
0.956	0.291963694	
0.903	0.292301195	
0.909	0.292501839	
0.954	0.294469856	
1.561	0.295493856	
0.955	0.295635624	
1.585	0.295740958	
1.623	0.296070609	
1.585	0.296171171	

0.982	0.296372473	
1.591	0.296472004	
1.646	0.296487276	
1.62	0.296826073	
1.623	0.296852332	
1.589	0.296957051	
1.618	0.296982857	
0.955	0.297916872	
0.981	0.297959447	
0.956	0.298129882	
1.583	0.298327752	
0.962	0.298514825	
0.962	0.298819675	
1.582	0.299082625	
1.582	0.299427221	
1.64	0.299479232	
1.582	0.299632129	
0.983	0.300360779	
1.58	0.30176523	
0.944	0.301766598	
0.969	0.301935276	
1.6	0.3019357	
0.889	0.302425967	
0.925	0.302629975	
0.886	0.303170967	
1.621	0.303466553	
1.671	0.303563515	
1.592	0.303697209	
1.676	0.303718386	
0.926	0.304001579	
1.637	0.304114306	
1.609	0.304253564	
1.621	0.304304794	
0.968	0.304672777	
1.565	0.304996996	
1.577	0.305159333	
1.706	0.305319364	
0.968	0.305646253	
0.933	0.306469328	
0.964	0.306688422	
1.556	0.30727996	
1.602	0.307316753	
0.978	0.307370753	
0.887	0.307436963	
1.575	0.307640234	

0.974	0.307680454	
0.968	0.30771604	
0.988	0.307909396	
1.725	0.308541828	
0.928	0.308929209	
1.574	0.30901849	
0.975	0.309030837	
1.579	0.309113611	
0.969	0.309787772	
0.967	0.309841631	
0.966	0.31036534	
1.572	0.310842473	
1.588	0.310878211	
0.914	0.311035056	
0.891	0.31111909	
1.546	0.311192609	
0.963	0.311205541	
1.598	0.311226985	
0.889	0.311579112	
0.93	0.312333647	
1.56	0.312385786	
1.571	0.312656817	
0.971	0.312772849	
0.889	0.312773714	
1.592	0.312823507	
0.954	0.312931245	
0.931	0.312987396	
0.93	0.313373864	
1.721	0.313512865	
1.706	0.313880104	
1.588	0.313888403	
1.572	0.313997703	
0.931	0.314638692	
1.659	0.314746222	
1.578	0.314939043	
1.569	0.315044817	
1.575	0.315121798	
1.552	0.315441571	
1.602	0.315483306	
1.567	0.315563664	
1.609	0.316124372	
1.605	0.316342882	
1.526	0.316576624	
1.558	0.316683007	
1.576	0.316962917	

0.895	0.317372821	
0.933	0.317385215	
1.629	0.317777366	
0.922	0.318179163	
0.968	0.318466681	
0.921	0.318511945	
0.935	0.318695173	
1.525	0.318913693	
1.565	0.319022125	
1.585	0.319119623	
1.584	0.31922455	
0.922	0.319381319	
1.602	0.319745598	
1.648	0.320566429	
1.564	0.321003848	
1.563	0.321304297	
0.99	0.321360684	
1.707	0.322509906	
0.926	0.322703083	
1.598	0.323453735	
0.953	0.323824258	
0.984	0.323928325	
1.706	0.32402	
1.576	0.324044492	
1.605	0.324049058	
1.598	0.325344796	
0.942	0.325625605	
1.541	0.326639152	
1.548	0.326682502	
0.971	0.32670048	
1.526	0.327159156	
0.937	0.327172112	
1.588	0.327481853	
0.965	0.327957392	
0.924	0.328208817	
0.924	0.328286826	
0.941	0.328462946	
1.581	0.328644055	
0.925	0.329078222	
0.995	0.329273542	
1.583	0.329404307	
1.595	0.329440156	
1.582	0.329507851	
0.985	0.329858554	
1.524	0.330016513	

1.568	0.33154386	
0.937	0.331577117	
0.94	0.331581996	
0.94	0.331737494	
1.554	0.332525857	
1.523	0.334117196	
0.935	0.334279415	
0.941	0.334728873	
0.977	0.33527085	
1.552	0.335558372	
1.553	0.33558071	
0.98	0.336199766	
0.929	0.336642029	
1.537	0.336732286	
1.701	0.336791768	
0.936	0.336805439	
1.697	0.337546634	
0.99	0.338684583	
1.594	0.338947373	
1.571	0.339094565	
1.53	0.339966365	
0.948	0.340485963	
1.509	0.34081816	
1.547	0.340896117	
1.52	0.341483017	
0.932	0.341723572	
1.547	0.341840854	
1.653	0.342099636	
0.98	0.34263413	
0.945	0.342855504	
1.539	0.343125551	
1.578	0.343192722	
1.587	0.343204133	
0.964	0.343355098	
1.545	0.343455337	
0.917	0.343591215	
1.548	0.34409455	
0.936	0.344682233	
1.003	0.344975193	
0.946	0.345260188	
1.526	0.345418209	
0.989	0.34627747	
0.964	0.346577857	
0.936	0.346662627	
1.543	0.346778811	

0.983	0.346802007	
0.936	0.34718416	
1.573	0.347504925	
0.981	0.347655214	
0.944	0.348105567	
1.542	0.348213679	
0.948	0.348846894	
1.547	0.348851346	
1.564	0.349038182	
0.922	0.350143107	
0.939	0.350590595	
0.909	0.350595892	
1.54	0.350788543	
1.533	0.351140384	
1.577	0.35158238	
0.984	0.351720152	
1.636	0.352273706	
1.558	0.352725143	
0.909	0.352729559	
0.991	0.352766027	
0.925	0.35302745	
0.941	0.353402871	
1.569	0.353494294	
1.55	0.354419704	
0.962	0.354624703	
0.935	0.354651647	
0.916	0.354893537	
0.962	0.354974333	
1.549	0.355347141	
1.51	0.355674726	
1.637	0.355816301	
1.583	0.356131799	
0.998	0.356179169	
1.559	0.35675467	
0.952	0.357204471	
0.952	0.357430913	
0.97	0.357755784	
0.986	0.357941983	
1.5	0.358174233	
0.995	0.358189226	
0.917	0.358789677	
1.547	0.358902961	
0.953	0.358963226	
0.986	0.359349318	
0.941	0.359592407	

1.562	0.359843533	
1.61	0.360655478	
1.57	0.360898761	
0.988	0.361704687	
1.592	0.362384616	
0.997	0.362482562	
1.541	0.362734845	
1	0.363137101	
1.569	0.364139836	
0.942	0.364615895	
0.943	0.364630592	
1.668	0.364686294	
1.015	0.365698048	
0.976	0.366010185	
1.503	0.366015794	
1.492	0.367541091	
0.988	0.367735238	
0.957	0.36834008	
1.623	0.368913322	
1.565	0.369697689	
0.958	0.369780014	
1.558	0.36978561	
1.565	0.369877133	
1.556	0.370095291	
1.616	0.370133911	
1.526	0.370240371	
1.012	0.370303788	
1.567	0.370367304	
0.998	0.370412927	
0.952	0.370785862	
0.955	0.37088612	
0.927	0.370978198	
1.572	0.371288113	
0.978	0.371300631	
0.924	0.37138652	
1.002	0.371848144	
0.921	0.372297504	
1.562	0.372750857	
1.523	0.372822797	
1.523	0.373154853	
1.009	0.373160761	
0.959	0.373281639	
0.96	0.373664633	
1.008	0.374147778	
0.944	0.374182278	

0.96	0.374316297	
1.511	0.375531757	
0.978	0.375533504	
0.961	0.375751085	
0.961	0.376006608	
1.513	0.376145126	
1.521	0.37635416	
1.52	0.376518023	
1.019	0.37674755	
0.961	0.377311474	
0.981	0.377597705	
1.013	0.377684032	
1.004	0.377979437	
0.976	0.378336478	
1.559	0.378373998	
0.957	0.37847396	
0.995	0.378631435	
1.518	0.379438003	
1.541	0.379575548	
1.546	0.379606935	
0.996	0.379998732	
1.51	0.380048134	
0.997	0.380207426	
1.518	0.380222814	
1.558	0.380854195	
0.963	0.381222684	
0.951	0.38190901	
0.955	0.381995495	
1.516	0.382096475	
0.984	0.38357628	
0.932	0.384007189	
0.999	0.38409869	
1.554	0.384315919	
1.542	0.384482208	
0.956	0.384535466	
1.534	0.384711376	
0.953	0.384742796	
1.565	0.385516475	
1	0.385969198	
0.933	0.386090267	
0.962	0.386245991	
0.966	0.387096842	
1	0.387516069	
0.957	0.388070598	
1.514	0.388132959	

1.001	0.388174463	
1.002	0.38849193	
1.002	0.388807732	
1.552	0.388894639	
0.965	0.388922925	
0.967	0.388931785	
0.967	0.389276247	
1.524	0.389332497	
0.987	0.389408158	
1.634	0.389453339	
1.508	0.389753514	
1.016	0.389802464	
1.547	0.389808904	
1.002	0.389969008	
1.511	0.390098724	
1.511	0.390146417	
1.528	0.391124131	
1.536	0.391251926	
1.002	0.391409823	
0.957	0.391625415	
1.023	0.391905441	
1.534	0.392125167	
1.546	0.392130079	
1.509	0.392392099	
1.509	0.392483369	
0.945	0.393415291	
1.508	0.393478897	
1.564	0.393570438	
1.479	0.394042055	
1.509	0.394153829	
1.534	0.394366483	
1.533	0.394773996	
0.985	0.395433335	
1.014	0.395602584	
1.003	0.395651856	
0.983	0.395706906	
0.94	0.395848091	
0.975	0.395881877	
1.483	0.396034365	
1.003	0.396075887	
0.97	0.396205924	
0.94	0.396568389	
1.012	0.396568789	
0.97	0.396569169	
1.525	0.396596247	

1.482	0.397360128	
0.971	0.397654224	
1.572	0.397778762	
0.967	0.398103287	
1.022	0.398160046	
1.505	0.398203454	
1.63	0.398271196	
0.99	0.398327102	
0.971	0.3984453	
0.971	0.398783053	
1.492	0.399979035	
1.029	0.39999419	
1.004	0.400417922	
1.528	0.400510624	
1.493	0.400738504	
1.618	0.400873752	
0.972	0.401633943	
1.502	0.40230315	
1.479	0.402490447	
0.973	0.402648481	
1.612	0.402683037	
1.502	0.402699114	
0.973	0.402909731	
1.533	0.403688517	
1.007	0.40386029	
1.504	0.403914367	
1.521	0.404031881	
1.01	0.40460462	
0.974	0.404890299	
1.552	0.405104892	
1.531	0.405326815	
1.5	0.405537347	
1.5	0.405681727	
1.524	0.405696385	
1.501	0.405871508	
1.499	0.406298098	
1.575	0.406422552	
1.544	0.406632219	
1.024	0.406954598	
1.009	0.406973533	
0.966	0.407011577	
1.521	0.407169813	
1.011	0.407556126	
1.008	0.407637855	
1.498	0.40773035	

1.532	0.407796308	
1.499	0.407812779	
0.981	0.40839937	
1.506	0.408547196	
1.498	0.408612285	
0.97	0.409117265	
1.497	0.409244335	
1.012	0.409634339	
1.535	0.409752674	
1.01	0.410169604	
1.619	0.410349932	
1.535	0.410445684	
0.976	0.41046591	
0.976	0.410650945	
0.976	0.41068521	
1.004	0.410725487	
1.524	0.412008123	
0.977	0.412383367	
1.495	0.412469714	
1.027	0.412505491	
1.012	0.412960169	
0.947	0.413031638	
1.586	0.413091387	
1.495	0.413249991	
1.531	0.413749848	
0.985	0.413819862	
1.536	0.41400257	
1.494	0.414121112	
1.494	0.414150401	
1.509	0.414353697	
0.981	0.414811868	
1.534	0.414982206	
1.506	0.415774418	
0.979	0.415812693	
1.553	0.415942989	
1.528	0.415957779	
0.982	0.416048324	
1.62	0.416096011	
0.971	0.41614743	
1.493	0.416458051	
1.481	0.416850692	
0.97	0.417245172	
1.027	0.417973481	
1.467	0.418254803	
1.525	0.418715285	

0.979	0.419120468	
1.469	0.419158553	
1.527	0.41916424	
0.98	0.41928587	
1.491	0.419594123	
1.573	0.419728381	
1.008	0.419897017	
1.016	0.419976428	
1.567	0.420142882	
1.518	0.421429871	
1.514	0.421687	
1.026	0.422835345	
1.6	0.422895205	
0.982	0.422922726	
1.607	0.42304811	
1.029	0.423906288	
1.467	0.424167023	
0.995	0.424425673	
1.487	0.425087624	
1.556	0.425421938	
0.953	0.425443225	
1.005	0.425532681	
1.486	0.425587361	
0.988	0.42564509	
1.018	0.425995837	
1.486	0.426256867	
1.474	0.426303324	
1.017	0.426336782	
1.486	0.426500751	
1.57	0.427006715	
1.515	0.427063553	
0.976	0.42732463	
0.984	0.427566832	
1.009	0.427911233	
1.484	0.428800302	
1.605	0.429382245	
1.526	0.430617562	
1.005	0.430657904	
1.483	0.431186445	
1.495	0.431362265	
1.021	0.431681244	
1.517	0.432095334	
0.981	0.432333423	
1.47	0.432544402	
1.51	0.433249806	

0.99	0.433942378	
1.491	0.434249323	
1.044	0.43430905	
1.481	0.434319663	
0.987	0.435113937	
0.991	0.435257848	
1.48	0.435268188	
0.98	0.435421701	
1.045	0.43560353	
1.026	0.435819778	
0.995	0.436524286	
0.995	0.43653928	
0.988	0.436657365	
1.479	0.436997417	
1.021	0.437016782	
0.963	0.437308779	
1.035	0.437318614	
1.596	0.437405726	
1.03	0.437649642	
0.983	0.438127188	
1.008	0.438270738	
0.99	0.43900539	
1.032	0.439487254	
0.962	0.439550476	
1.021	0.439922564	
1.014	0.440432039	
1.487	0.440471399	
0.969	0.440516125	
0.988	0.440598335	
1.476	0.440816561	
1.502	0.441075855	
1.494	0.441121189	
1.476	0.441334779	
1.487	0.44142793	
0.98	0.441631844	
1.48	0.441775178	
1.02	0.442393784	
1.034	0.442532396	
1.528	0.442658598	
1.555	0.442856538	
1.475	0.443008643	
1.504	0.443112163	
1.457	0.443214964	
0.991	0.443237635	
1.024	0.443334888	

1.494	0.44429771	
0.984	0.44453141	
1.022	0.445126226	
1.513	0.445209649	
0.992	0.445278352	
1.515	0.445834989	
1.513	0.445858821	
1.473	0.445864455	
1.552	0.446122327	
1.55	0.446302551	
1.498	0.446565012	
1.034	0.446668809	
1.026	0.446750438	
1.489	0.446871002	
1.53	0.447070418	
1.49	0.447254765	
1.5	0.44736657	
1.551	0.447841654	
1.517	0.448587446	
1.494	0.448939037	
1.518	0.448980689	
1.458	0.449110609	
1.583	0.449200522	
0.966	0.449217139	
1.043	0.449433369	
1.47	0.450129417	
1.031	0.450304521	
0.986	0.450395644	
1.014	0.450568244	
0.969	0.450644056	
0.986	0.450968435	
1.556	0.451003438	
0.994	0.451215287	
0.97	0.451443665	
1.469	0.451558824	
0.988	0.451844167	
0.994	0.451859149	
0.994	0.451947638	
1.469	0.452139199	
1.509	0.452283667	
1.498	0.452410238	
1.542	0.452544774	
0.999	0.452954673	
1.502	0.453300947	
1.481	0.453333613	

1.015	0.453463273	
1.48	0.453485619	
1.486	0.453535284	
0.989	0.453773161	
0.999	0.454068042	
1.029	0.454170868	
1.491	0.454590944	
1.51	0.454909881	
0.996	0.454987488	
1.467	0.455898901	
1.467	0.456019751	
0.996	0.456633722	
1.443	0.456757222	
1.448	0.457120002	
0.969	0.457241506	
1.018	0.457452118	
0.997	0.457457048	
0.997	0.457630784	
1.481	0.457749374	
0.974	0.45794415	
1.505	0.458008887	
1.472	0.458036154	
1.517	0.458253737	
1.051	0.458260526	
1.045	0.458719798	
0.997	0.45890892	
1.442	0.459004949	
1.465	0.459208613	
0.993	0.459216423	
0.998	0.45931499	
0.998	0.45948855	
0.991	0.459494622	
1.03	0.459501173	
1.477	0.459590399	
1.47	0.460342208	
1.483	0.46061874	
1.506	0.460653327	
1.535	0.461320877	
1.045	0.461458075	
1.031	0.46202743	
1.019	0.462286568	
1.03	0.462477963	
1.503	0.462798102	
1.053	0.463031986	
1.051	0.463747525	

1.011	0.463752876	
1.473	0.463979865	
1	0.464716847	
1.04	0.465538295	
1.026	0.465653849	
0.994	0.466102093	
1	0.466115803	
1.437	0.466135016	
1.53	0.46614821	
1.058	0.466163642	
1.001	0.466815001	
1.498	0.466947431	
0.998	0.466984786	
1.492	0.467031905	
1.572	0.46776039	
1.046	0.468130914	
1.052	0.468135346	
1.048	0.468504703	
1.451	0.468706947	
1.035	0.468851023	
1.537	0.469089141	
1.535	0.46920358	
1.567	0.469409467	
1.488	0.469416186	
1.036	0.469524027	
1.002	0.469948728	
1.035	0.47005254	
1.458	0.470080457	
1.458	0.470594582	
1.45	0.470901973	
1.569	0.471506376	
1.437	0.47210295	
1.003	0.472417514	
1.037	0.472688071	
1.034	0.472690684	
1.463	0.472697399	
1.003	0.472746789	
1.452	0.472748723	
1.003	0.472993122	
1.469	0.473268148	
1.566	0.473650137	
1.004	0.473815067	
1.039	0.473851037	
0.986	0.473857578	
1.453	0.474019054	

0.98	0.474148135	
1.429	0.474434781	
1.489	0.474582529	
0.995	0.474647636	
1.442	0.474654043	
0.98	0.474838035	
1.051	0.474975115	
1.004	0.475313499	
1.497	0.475370737	
1.496	0.475475208	
1.046	0.475846029	
1.509	0.47615122	
1.053	0.47630053	
1.046	0.476305576	
1.487	0.476634397	
1.005	0.476833584	
1.063	0.477242426	
1.568	0.477389914	
0.982	0.4775618	
1.047	0.477737145	
1.476	0.478157877	
1.05	0.478192127	
1.037	0.478407933	
1.523	0.478586429	
1.491	0.478633055	
1.052	0.479419243	
1.452	0.47977136	
1.452	0.47991297	
1.474	0.479996835	
1.478	0.480085116	
1.009	0.480555353	
1.464	0.480930261	
1.044	0.481408483	
1.482	0.482332591	
0.999	0.48261215	
1.047	0.482674235	
1.022	0.482841101	
1.554	0.482998125	
1.058	0.483080101	
1.49	0.483275632	
1.049	0.483284435	
1.488	0.483556997	
1.015	0.483756823	
1.481	0.483879744	
1.014	0.484147895	

1.461	0.484466273	
1.048	0.485682748	
1.474	0.485976789	
1.472	0.485987912	
1.475	0.486035152	
1.029	0.486291659	
1.43	0.486448679	
1.459	0.486495675	
1.472	0.486891702	
1.447	0.487476404	
1.428	0.487585889	
1.458	0.4877383	
1.01	0.488143327	
1.01	0.488232094	
1.035	0.488554725	
1.049	0.488557377	
1.487	0.488689159	
0.993	0.488859099	
1.485	0.489468561	
1.426	0.489710254	
1.43	0.489800647	
1.01	0.489814757	
1.011	0.490029558	
1.452	0.490123449	
1.491	0.490319387	
1.445	0.490679205	
1.434	0.490725245	
1.06	0.490756398	
1.013	0.490776569	
1.471	0.490872598	
1.468	0.49133425	
0.989	0.49138599	
1.04	0.491568345	
0.987	0.491613264	
1.011	0.491956553	
1.011	0.492084826	
1.015	0.492338319	
1.511	0.492541138	
1.43	0.492816379	
1.052	0.492819312	
1.444	0.492911832	
0.99	0.493649092	
1.443	0.494875738	
1.057	0.494983375	
1.464	0.495080403	

1.013	0.495495395	
1.071	0.495502501	
1.452	0.495535584	
1.05	0.495544911	
1.429	0.495640194	
1.481	0.495689093	
1.013	0.495782205	
0.991	0.495800523	
1.427	0.496196821	
1.056	0.496455667	
0.989	0.496589869	
1.033	0.496677983	
1.442	0.496723082	
1.442	0.49688394	
0.992	0.497115234	
1.434	0.498110368	
1.044	0.49817855	
1.461	0.498324237	
1.498	0.498407158	
1.014	0.498526525	
1.014	0.498835338	
1.055	0.499115077	
1.01	0.499362355	
1.06	0.499879365	
1.48	0.499918597	
1.019	0.500016069	
1.072	0.50007573	
1.01	0.500525385	
0.992	0.500567226	
1.437	0.500638388	
1.46	0.500712973	
1.526	0.500798985	
1.049	0.500939641	
1.047	0.501124951	
1.015	0.501164819	
1.032	0.501275322	
1.053	0.501598429	
1.462	0.501898162	
1.058	0.502375135	
1.438	0.502460219	
1.429	0.502666796	
1.055	0.503110734	
1.029	0.503315612	
1.016	0.503337255	
1.046	0.503909196	

0.994	0.504078679	
1.026	0.504153875	
1.472	0.504881269	
1.057	0.505435667	
1.016	0.505599194	
1.054	0.505657674	
1.068	0.505722245	
1.011	0.506192981	
1.017	0.506365994	
1.058	0.506434569	
1.048	0.506448891	
1.453	0.50720144	
1.029	0.507491278	
1.448	0.507661393	
1.018	0.507778032	
1.435	0.507977033	
1.453	0.508052089	
1.431	0.508352672	
1.068	0.508550095	
1.452	0.510049863	
1.001	0.510811009	
1.019	0.510916603	
1.019	0.511751964	
1.484	0.512157604	
1.038	0.512551332	
1.016	0.512768812	
1.457	0.51292519	
1.02	0.51329558	
1.02	0.513328111	
1.432	0.513648586	
0.998	0.51376552	
1.072	0.514055803	
1.419	0.514241853	
1.518	0.514500835	
1.465	0.514856816	
1.061	0.515016465	
1.431	0.51568853	
1.484	0.51626627	
1.437	0.51662684	
1.022	0.516673778	
1.025	0.516709751	
1.017	0.517819375	
1.419	0.517887713	
1.042	0.518096721	
1.035	0.518436863	

1.46	0.518875693	
1.529	0.519034988	
1.429	0.519348882	
1.461	0.519372612	
1.412	0.519832233	
1.03	0.520338697	
1.034	0.520719629	
1.453	0.520752799	
1.491	0.521026038	
1.031	0.521095439	
1.02	0.521793438	
1.053	0.521811321	
1.063	0.521952119	
1.407	0.522210457	
1.521	0.522278367	
1.506	0.522532288	
1.039	0.522635038	
1.023	0.522855217	
1.063	0.523133145	
1.452	0.523254847	
1.481	0.523361505	
1.044	0.523546856	
1.43	0.523827692	
1.46	0.523977146	
1.025	0.524097091	
1.459	0.52455373	
1.425	0.525187766	
1.072	0.525289099	
1.494	0.525818887	
1.425	0.52623578	
1.085	0.52673824	
1.407	0.526856106	
1.408	0.527106191	
1.436	0.527452274	
1.024	0.527459371	
1.444	0.527607257	
1.055	0.527755025	
1.056	0.527763999	
1.405	0.527788675	
1.465	0.528307958	
1.063	0.52836015	
1.485	0.529189379	
1.046	0.530280476	
1.447	0.530881276	
1.434	0.531297053	

1.458	0.531383328	
1.451	0.531449047	
1.482	0.531508599	
1.488	0.531686631	
1.006	0.531688064	
1.079	0.531701897	
1.064	0.532442702	
1.421	0.532453931	
1.025	0.532505228	
1.426	0.532795778	
1.057	0.532826383	
1.039	0.532904245	
1.057	0.533099157	
1.067	0.53337944	
1.411	0.533684739	
1.048	0.533724222	
1.458	0.534567371	
1.459	0.534602383	
1.038	0.534707476	
1.46	0.534991817	
1.476	0.535349991	
1.42	0.535464836	
1.057	0.535527787	
1.443	0.535893652	
1.419	0.536070399	
1.03	0.536270029	
1.48	0.536498303	
1.03	0.536846639	
1.087	0.536941496	
1.416	0.536945654	
1.452	0.537157779	
1.418	0.537228659	
1.418	0.537700484	
1.442	0.53799233	
1.076	0.538003845	
1.03	0.53805356	
1.073	0.53815972	
1.43	0.538345758	
1.03	0.538927828	
1.399	0.538943495	
1.426	0.539232666	
1.425	0.539325851	
1.454	0.539346801	
1.439	0.539433589	
1.437	0.5395136	

1.072	0.53954414	
1.073	0.539694734	
1.44	0.540116061	
1.031	0.540159546	
1.447	0.540330808	
1.031	0.540568108	
1.406	0.540658563	
1.014	0.54076444	
1.438	0.540764565	
1.069	0.540886965	
1.079	0.541094601	
1.442	0.541435823	
1.059	0.541457269	
1.014	0.5415184	
1.463	0.541558208	
1.063	0.541571634	
1.462	0.541866658	
1.043	0.542038649	
1.425	0.542291576	
1.419	0.542494246	
1.447	0.542517833	
1.016	0.542579929	
1.416	0.542628771	
1.054	0.542671383	
1.427	0.542704365	
1.434	0.543187429	
1.078	0.54380733	
1.448	0.54386947	
1.065	0.544188907	
1.074	0.544305894	
1.428	0.54475032	
1.052	0.545242016	
1.476	0.545616705	
1.013	0.545688518	
1.439	0.545954683	
1.055	0.545985603	
1.033	0.546024227	
1.4	0.546211578	
1.09	0.546707373	
1.034	0.546855598	
1.413	0.547423076	
1.085	0.548062548	
1.508	0.548277757	
1.034	0.548501307	
1.073	0.548601158	

1.063	0.548715461	
1.018	0.548756517	
1.437	0.548875786	
1.447	0.548932932	
1.069	0.548999505	
1.478	0.549212636	
1.018	0.549319801	
1.071	0.549422275	
1.49	0.550056052	
1.035	0.550110678	
1.506	0.550182854	
1.033	0.550891959	
1.396	0.551043775	
1.452	0.551115694	
1.018	0.551201893	
1.035	0.55127275	
1.074	0.551551632	
1.066	0.551668158	
1.057	0.55213621	
1.069	0.552414204	
1.054	0.552438435	
1.505	0.552697081	
1.394	0.552917522	
1.066	0.552930185	
1.41	0.553076144	
1.021	0.553446469	
1.089	0.553706874	
1.035	0.55384587	
1.449	0.553924658	
1.081	0.553981014	
1.084	0.554278907	
1.037	0.554367345	
1.078	0.554856252	
1.424	0.555675256	
1.095	0.55610312	
1.088	0.556148732	
1.081	0.556467461	
1.025	0.556752318	
1.464	0.556910763	
1.075	0.557089011	
1.423	0.557215224	
1.397	0.557412537	
1.421	0.557413136	
1.027	0.557499684	
1.069	0.557582075	

1.407	0.558146471	
1.035	0.558194315	
1.074	0.558302967	
1.07	0.558415099	
1.082	0.558617204	
1.067	0.55873521	
1.464	0.559010099	
1.039	0.559901282	
1.084	0.559957225	
1.089	0.560085861	
1.406	0.560354362	
1.039	0.560559357	
1.092	0.560840988	
1.07	0.561222533	
1.084	0.561298676	
1.395	0.561302842	
1.431	0.561362632	
1.085	0.561497149	
1.433	0.561632254	
1.466	0.561900775	
1.04	0.562155526	
1.405	0.562551679	
1.068	0.562975862	
1.021	0.563188008	
1.069	0.563421818	
1.425	0.564135704	
1.041	0.564203647	
1.041	0.564456481	
1.069	0.564810904	
1.46	0.565023858	
1.403	0.565274444	
1.445	0.565302512	
1.026	0.565611543	
1.027	0.565613045	
1.427	0.565838442	
1.082	0.5660776	
1.076	0.566205971	
1.058	0.566478242	
1.042	0.566591465	
1.046	0.566642896	
1.41	0.567314356	
1.493	0.567351386	
1.435	0.567660669	
1.028	0.567851045	
1.427	0.567998369	

1.073	0.569419899	
1.076	0.569817848	
1.076	0.569864954	
1.4	0.572039252	
1.08	0.572244854	
1.042	0.572285274	
1.439	0.572528691	
1.051	0.572892797	
1.434	0.57318969	
1.064	0.57325682	
1.045	0.573768752	
1.462	0.574282184	
1.073	0.574459492	
1.075	0.574780549	
1.462	0.574938607	
1.03	0.575032541	
1.398	0.575343309	
1.065	0.575353811	
1.42	0.575447181	
1.387	0.575503698	
1.398	0.575604395	
1.044	0.575640174	
1.398	0.575703493	
1.09	0.575760995	
1.397	0.575881591	
1.078	0.576139228	
1.425	0.576196807	
1.084	0.576599675	
1.046	0.576796598	
1.438	0.576850243	
1.046	0.576956585	
1.045	0.57757544	
1.073	0.577910919	
1.419	0.578133806	
1.381	0.57821147	
1.385	0.578301482	
1.084	0.578530962	
1.41	0.578982911	
1.396	0.579231378	
1.047	0.579345789	
1.419	0.58009999	
1.052	0.580128328	
1.047	0.580220708	
1.083	0.580362342	
1.395	0.580477971	

1.41	0.580634509	
1.047	0.580691074	
1.396	0.580752576	
1.41	0.580805705	
1.434	0.580819972	
1.466	0.580981253	
1.099	0.581385675	
1.417	0.581447504	
1.391	0.581538636	
1.068	0.581730702	
1.041	0.581843326	
1.076	0.581859004	
1.076	0.581917715	
1.082	0.582198284	
1.416	0.583328304	
1.418	0.583959363	
1.054	0.58410919	
1.393	0.58445291	
1.417	0.584495514	
1.03	0.584697333	
1.449	0.584993866	
1.049	0.585502505	
1.418	0.58551837	
1.434	0.585603952	
1.377	0.585623836	
1.375	0.586301853	
1.433	0.586322203	
1.414	0.586509448	
1.41	0.586685007	
1.377	0.586939341	
1.035	0.587479101	
1.435	0.58770058	
1.414	0.587889542	
1.047	0.588513509	
1.05	0.588674292	
1.445	0.588779379	
1.051	0.588779614	
1.086	0.588806337	
1.056	0.589024641	
1.095	0.589564721	
1.394	0.590225091	
1.39	0.590426634	
1.089	0.590728332	
1.428	0.590837839	
1.099	0.590916942	

1.056	0.591723343	
1.042	0.592127282	
1.391	0.592128827	
1.09	0.592644854	
1.412	0.592695779	
1.462	0.592945633	
1.052	0.59296736	
1.412	0.592968719	
1.373	0.593026778	
1.085	0.593048944	
1.098	0.593666622	
1.41	0.594387173	
1.074	0.594728874	
1.399	0.595048997	
1.041	0.595314494	
1.062	0.595450426	
1.459	0.595481817	
1.473	0.596087591	
1.053	0.596093014	
1.041	0.596292914	
1.423	0.596304977	
1.381	0.596584692	
1.413	0.596695427	
1.443	0.596714698	
1.449	0.596737869	
1.447	0.597118359	
1.386	0.59782883	
1.471	0.597918942	
1.052	0.598916321	
1.044	0.598917643	
1.406	0.599720207	
1.094	0.599933525	
1.055	0.600044195	
1.055	0.600056782	
1.062	0.600100524	
1.054	0.600137015	
1.055	0.600218501	
1.467	0.600357854	
1.077	0.600581983	
1.101	0.600603081	
1.083	0.60123758	
1.083	0.601384757	
1.371	0.60147872	
1.09	0.601687441	
1.386	0.601732824	

1.083	0.601984006	
1.444	0.602069631	
1.056	0.602348959	
1.443	0.602793359	
1.061	0.602907394	
1.471	0.602992146	
1.07	0.603236583	
1.103	0.603588162	
1.383	0.603616737	
1.054	0.603767155	
1.05	0.603780616	
1.091	0.603891095	
1.383	0.604094033	
1.093	0.604128679	
1.097	0.604345149	
1.444	0.604446847	
1.392	0.604571543	
1.113	0.604599126	
1.092	0.604723938	
1.438	0.605103327	
1.385	0.605524765	
1.084	0.605530452	
1.466	0.605805489	
1.045	0.606264872	
1.057	0.606459573	
1.462	0.606789504	
1.084	0.607119555	
1.439	0.607334233	
1.047	0.607449952	
1.06	0.607631768	
1.064	0.60779477	
1.079	0.607820361	
1.42	0.608070074	
1.078	0.608197696	
1.4	0.608477717	
1.391	0.609326353	
1.059	0.609343643	
1.095	0.609354839	
1.081	0.609693092	
1.38	0.609720825	
1.366	0.609955862	
1.054	0.610029461	
1.087	0.610660879	
1.418	0.610729473	
1.096	0.61142745	

1.4	0.611451624	
1.095	0.611979555	
1.389	0.612028162	
1.403	0.612163981	
1.392	0.612447984	
1.389	0.61251374	
1.371	0.612799675	
1.047	0.613061263	
1.082	0.613311742	
1.075	0.613362978	
1.079	0.613395612	
1.413	0.613681018	
1.06	0.613737621	
1.366	0.613794274	
1.382	0.613987238	
1.089	0.614397482	
1.377	0.614718843	
1.382	0.61527745	
1.375	0.615457067	
1.092	0.615467873	
1.385	0.615802333	
1.059	0.615828852	
1.061	0.616474092	
1.082	0.616530566	
1.368	0.616963369	
1.374	0.617061224	
1.378	0.617127791	
1.073	0.617622249	
1.404	0.617780152	
1.372	0.618695598	
1.399	0.61888359	
1.067	0.619158831	
1.1	0.619305167	
1.107	0.6194412	
1.089	0.619573438	
1.061	0.619721877	
1.091	0.61987543	
1.375	0.620089675	
1.457	0.620233872	
1.109	0.620503987	
1.409	0.620570578	
1.369	0.620701952	
1.374	0.620758853	
1.078	0.620943084	
1.401	0.620977353	

1.089	0.621559014	
1.368	0.621932659	
1.398	0.622221771	
1.072	0.623048243	
1.089	0.62373906	
1.099	0.623771648	
1.402	0.624107253	
1.393	0.624121317	
1.38	0.624302117	
1.106	0.624444917	
1.407	0.624646012	
1.458	0.624974724	
1.089	0.625100233	
1.071	0.625391286	
1.073	0.625474034	
1.396	0.62561848	
1.065	0.625847949	
1.399	0.626357055	
1.121	0.626500201	
1.391	0.626589724	
1.422	0.626647371	
1.1	0.626847456	
1.066	0.627005098	
1.05	0.627397283	
1.395	0.627510987	
1.388	0.627763589	
1.409	0.627833035	
1.106	0.62817538	
1.066	0.62829525	
1.095	0.628572365	
1.091	0.628600861	
1.108	0.628658022	
1.097	0.628663576	
1.398	0.629045852	
1.367	0.629462707	
1.367	0.629504244	
1.086	0.629949946	
1.076	0.630089438	
1.08	0.630283898	
1.427	0.630379853	
1.392	0.63088354	
1.421	0.631354993	
1.434	0.631412807	
1.067	0.631964903	
1.368	0.632293512	

1.103	0.633419474	
1.354	0.63353706	
1.076	0.633832635	
1.409	0.634234331	
1.068	0.634314817	
1.06	0.634565949	
1.419	0.63487756	
1.118	0.635292876	
1.065	0.635830474	
1.101	0.636270951	
1.37	0.636743158	
1.059	0.63717426	
1.363	0.637227695	
1.405	0.637759346	
1.39	0.637786891	
1.107	0.638159813	
1.365	0.638469643	
1.104	0.638494136	
1.405	0.638641224	
1.127	0.638674642	
1.429	0.638937184	
1.07	0.639450832	
1.101	0.639514705	
1.082	0.639681961	
1.356	0.639865315	
1.365	0.639984828	
1.386	0.640062803	
1.08	0.640139552	
1.071	0.640455971	
1.406	0.641569227	
1.363	0.642810063	
1.351	0.643175905	
1.351	0.643495677	
1.097	0.644660455	
1.086	0.644727258	
1.103	0.644937727	
1.077	0.644993622	
1.114	0.644994907	
1.073	0.645658196	
1.376	0.645741508	
1.417	0.645752138	
1.073	0.645759468	
1.406	0.646006561	
1.362	0.646040296	
1.362	0.646297394	

1.073	0.646469183	
1.416	0.646580719	
1.098	0.646601287	
1.12	0.646668207	
1.361	0.646728048	
1.073	0.646796692	
1.361	0.646874493	
1.391	0.64692127	
1.081	0.646949804	
1.085	0.647104666	
1.11	0.647166906	
1.356	0.647468771	
1.389	0.647635104	
1.077	0.647988579	
1.074	0.648114534	
1.402	0.648382344	
1.074	0.648549213	
1.063	0.648821902	
1.125	0.648981436	
1.356	0.650236386	
1.099	0.650263238	
1.076	0.650638419	
1.087	0.650701935	
1.437	0.65107224	
1.108	0.651218225	
1.11	0.651620041	
1.419	0.651630964	
1.075	0.651709451	
1.075	0.65183037	
1.383	0.651901101	
1.435	0.652106455	
1.09	0.65223746	
1.107	0.652642871	
1.358	0.652704117	
1.076	0.652973367	
1.074	0.653021542	
1.101	0.653461443	
1.131	0.653875994	
1.072	0.654446478	
1.346	0.654898233	
1.366	0.655094203	
1.392	0.655346691	
1.094	0.655412359	
1.089	0.655891053	
1.078	0.656326874	

1.077	0.656341093	
1.119	0.65635004	
1.111	0.656384425	
1.106	0.656706919	
1.107	0.656793075	
1.106	0.656854199	
1.387	0.656866543	
1.379	0.656952931	
1.078	0.657104323	
1.377	0.65725151	
1.379	0.657514288	
1.112	0.657596043	
1.113	0.657981428	
1.41	0.658129705	
1.377	0.658752203	
1.355	0.659114091	
1.41	0.659206939	
1.129	0.659974238	
1.353	0.660022203	
1.091	0.660537721	
1.129	0.660614429	
1.355	0.660644627	
1.112	0.661828485	
1.391	0.662438383	
1.072	0.662614578	
1.375	0.662627937	
1.368	0.663028731	
1.381	0.663154029	
1.368	0.663268443	
1.381	0.663295605	
1.401	0.663593073	
1.352	0.663851504	
1.377	0.664290441	
1.075	0.664312867	
1.069	0.664755809	
1.11	0.665203379	
1.395	0.665284846	
1.088	0.665286828	
1.349	0.665537857	
1.352	0.665948345	
1.356	0.666072336	
1.372	0.666139372	
1.386	0.666415241	
1.378	0.666440134	
1.397	0.667183083	

1.11	0.667189983	
1.344	0.667247147	
1.076	0.66726315	
1.125	0.667410736	
1.136	0.667439141	
1.403	0.667543714	
1.427	0.667807732	
1.123	0.668392789	
1.089	0.668688669	
1.119	0.668815687	
1.35	0.669282057	
1.088	0.669459388	
1.365	0.670150487	
1.082	0.670318136	
1.113	0.670931991	
1.373	0.670973523	
1.082	0.671162885	
1.402	0.671271138	
1.373	0.671585733	
1.365	0.671760175	
1.384	0.671891673	
1.349	0.672684311	
1.083	0.67271894	
1.118	0.672719378	
1.349	0.672980791	
1.099	0.673430519	
1.388	0.673558825	
1.346	0.673818482	
1.078	0.674120631	
1.348	0.674372733	
1.125	0.674436496	
1.084	0.674878252	
1.084	0.674972924	
1.093	0.675075542	
1.084	0.675188279	
1.107	0.675222204	
1.108	0.675381318	
1.347	0.675674121	
1.09	0.675880205	
1.085	0.676077832	
1.112	0.676221604	
1.372	0.67626013	
1.123	0.676346398	
1.347	0.676697111	
1.128	0.676985321	

1.344	0.677146375	
1.095	0.677205676	
1.126	0.677749966	
1.371	0.678127579	
1.377	0.678196697	
1.117	0.678200439	
1.122	0.678460684	
1.346	0.678629416	
1.086	0.678653022	
1.368	0.67874851	
1.387	0.67878192	
1.086	0.679108673	
1.131	0.67939452	
1.086	0.679577385	
1.121	0.679594521	
1.366	0.679865308	
1.345	0.680216422	
1.416	0.680243249	
1.086	0.680332384	
1.105	0.680465446	
1.341	0.680526914	
1.386	0.680576128	
1.345	0.680596926	
1.358	0.680996344	
1.363	0.681177474	
1.117	0.68126598	
1.111	0.681598945	
1.344	0.681634702	
1.357	0.68190372	
1.382	0.682014962	
1.345	0.682211718	
1.347	0.682676834	
1.374	0.682938264	
1.128	0.682991666	
1.086	0.683204645	
1.087	0.683314365	
1.366	0.68414454	
1.088	0.684145991	
1.14	0.684491243	
1.117	0.684727219	
1.137	0.685183999	
1.117	0.685300974	
1.343	0.685346389	
1.109	0.685510479	
1.085	0.685581722	

1.096	0.68568905	
1.12	0.685823926	
1.114	0.686164736	
1.368	0.686368657	
1.128	0.686828086	
1.132	0.687057819	
1.12	0.687785104	
1.106	0.687823565	
1.367	0.688028125	
1.113	0.68861827	
1.125	0.688653681	
1.341	0.688688357	
1.332	0.688712866	
1.111	0.688744989	
1.381	0.688803957	
1.097	0.689027645	
1.11	0.689131778	
1.39	0.689155614	
1.354	0.689782	
1.09	0.689999466	
1.352	0.690172775	
1.372	0.690270647	
1.1	0.690452296	
1.352	0.690633056	
1.344	0.691130069	
1.122	0.691235	
1.34	0.691297564	
1.097	0.691416444	
1.09	0.691599031	
1.113	0.691812401	
1.101	0.691868246	
1.088	0.691886468	
1.087	0.691945379	
1.111	0.692007072	
1.097	0.692079945	
1.099	0.692474431	
1.339	0.692607643	
1.364	0.692656125	
1.113	0.693132287	
1.102	0.694968346	
1.39	0.695391941	
1.139	0.695961514	
1.336	0.696843605	
1.367	0.697168251	
1.385	0.697315572	

1.127	0.697813071	
1.337	0.69782547	
1.122	0.697860282	
1.113	0.69855097	
1.093	0.698712352	
1.334	0.698953005	
1.34	0.698980044	
1.086	0.69898235	
1.124	0.69910735	
1.101	0.699181045	
1.352	0.699222913	
1.115	0.699230506	
1.336	0.699301022	
1.09	0.699675642	
1.124	0.699687979	
1.349	0.700295154	
1.36	0.700411845	
1.387	0.700480207	
1.094	0.700596505	
1.118	0.700860514	
1.355	0.701194527	
1.362	0.701287094	
1.116	0.701378014	
1.098	0.701655616	
1.096	0.701749996	
1.123	0.702412554	
1.098	0.702545586	
1.363	0.702609473	
1.15	0.702950929	
1.334	0.703018858	
1.115	0.70384617	
1.334	0.703890159	
1.116	0.704014199	
1.146	0.704073113	
1.12	0.70414731	
1.334	0.704350906	
1.377	0.704690441	
1.117	0.704918179	
1.136	0.704958206	
1.331	0.705448202	
1.107	0.705453163	
1.151	0.705498412	
1.134	0.705685391	
1.372	0.705863511	
1.349	0.706500294	

1.333	0.706777577	
1.129	0.70715017	
1.093	0.707469512	
1.104	0.707612879	
1.348	0.707912649	
1.332	0.707936834	
1.35	0.708070402	
1.332	0.708198053	
1.4	0.708933364	
1.323	0.708980656	
1.098	0.709101561	
1.097	0.709278769	
1.103	0.709766649	
1.107	0.709775661	
1.359	0.709844267	
1.351	0.709918766	
1.351	0.710394019	
1.134	0.710441021	
1.098	0.710468469	
1.331	0.710979602	
1.12	0.711023395	
1.379	0.711106676	
1.119	0.71114824	
1.373	0.711182304	
1.139	0.711184844	
1.104	0.71129096	
1.098	0.71172646	
1.148	0.711772817	
1.102	0.712145218	
1.326	0.71278543	
1.116	0.712926084	
1.355	0.713109728	
1.329	0.713526918	
1.135	0.713612141	
1.381	0.713691085	
1.148	0.713801265	
1.091	0.71407847	
1.358	0.714091187	
1.348	0.714121964	
1.356	0.714433514	
1.121	0.714771713	
1.1	0.714825725	
1.096	0.714832457	
1.32	0.715481826	
1.133	0.715550734	

1.099	0.715726086	
1.328	0.715817489	
1.321	0.715889459	
1.132	0.716259832	
1.1	0.716447133	
1.122	0.716483887	
1.119	0.716648889	
1.328	0.716734021	
1.142	0.716801491	
1.1	0.716831942	
1.353	0.717019657	
1.327	0.717133326	
1.1	0.717196771	
1.123	0.717289522	
1.1	0.717491478	
1.347	0.717953501	
1.135	0.718653376	
1.349	0.718927709	
1.133	0.719007445	
1.101	0.719020774	
1.101	0.719138744	
1.129	0.719250596	
1.126	0.719282551	
1.101	0.719489436	
1.116	0.720175661	
1.116	0.7206121	
1.145	0.720735177	
1.368	0.720766089	
1.367	0.720802038	
1.338	0.721741538	
1.365	0.722140069	
1.367	0.722190732	
1.104	0.722281154	
1.127	0.72237657	
1.329	0.722801105	
1.366	0.722851964	
1.351	0.722887149	
1.134	0.723253092	
1.374	0.723304695	
1.103	0.723579452	
1.108	0.723590043	
1.325	0.723869909	
1.141	0.724050097	
1.103	0.724274264	
1.103	0.724301164	

1.11	0.724492664	
1.103	0.724523071	
1.122	0.72471831	
1.104	0.724755363	
1.145	0.72501362	
1.127	0.72511599	
1.124	0.725248388	
1.364	0.725559633	
1.134	0.725609329	
1.324	0.725722583	
1.37	0.725888509	
1.387	0.725970604	
1.105	0.72599435	
1.324	0.726102463	
1.118	0.726115708	
1.104	0.726364567	
1.323	0.726549781	
1.352	0.726825397	
1.34	0.727414456	
1.347	0.727454877	
1.104	0.728002348	
1.324	0.72863141	
1.113	0.728669889	
1.321	0.728719051	
1.105	0.72880513	
1.339	0.729033031	
1.326	0.729410614	
1.105	0.72948128	
1.368	0.729824229	
1.33	0.730297579	
1.137	0.730358208	
1.352	0.730999079	
1.349	0.731092401	
1.108	0.731470802	
1.106	0.73150178	
1.349	0.731753209	
1.386	0.732337797	
1.154	0.732549634	
1.126	0.732799749	
1.364	0.733054677	
1.372	0.733434393	
1.111	0.73380495	
1.332	0.734206676	
1.107	0.734317382	
1.365	0.734491837	

1.144	0.734579965	
1.103	0.734651815	
1.342	0.734760626	
1.151	0.734818277	
1.384	0.734993608	
1.339	0.735042814	
1.324	0.735108096	
1.115	0.735413868	
1.129	0.736000871	
1.319	0.736013249	
1.315	0.737230066	
1.108	0.737299868	
1.11	0.737767785	
1.149	0.737971385	
1.318	0.738145289	
1.318	0.738353401	
1.33	0.738441265	
1.318	0.739295445	
1.318	0.739431603	
1.334	0.739477061	
1.13	0.739482623	
1.109	0.739527969	
1.14	0.739540776	
1.318	0.739627179	
1.109	0.739662382	
1.321	0.73997339	
1.317	0.740392715	
1.333	0.7404554	
1.311	0.740457118	
1.109	0.740796066	
1.335	0.740982992	
1.141	0.741055703	
1.343	0.741331386	
1.11	0.741775993	
1.11	0.742261158	
1.134	0.742306645	
1.38	0.742887257	
1.119	0.742976692	
1.11	0.743020903	
1.111	0.743034504	
1.357	0.74306363	
1.332	0.743123749	
1.367	0.743521364	
1.34	0.744431675	
1.367	0.7445424	

1.112	0.744738446	
1.317	0.745222877	
1.355	0.745694791	
1.351	0.745941326	
1.121	0.746092034	
1.368	0.746255665	
1.109	0.746449825	
1.122	0.746509327	
1.314	0.746916012	
1.313	0.747068727	
1.314	0.747216484	
1.161	0.74733424	
1.309	0.747450907	
1.113	0.747653316	
1.168	0.747871019	
1.353	0.748833291	
1.139	0.748995518	
1.115	0.749228268	
1.113	0.75081924	
1.36	0.751070864	
1.116	0.751121362	
1.312	0.751142579	
1.113	0.751275413	
1.363	0.75133189	
1.13	0.751374699	
1.355	0.751459818	
1.113	0.751591972	
1.146	0.752609334	
1.323	0.752696596	
1.115	0.752754857	
1.355	0.753026665	
1.133	0.75348531	
1.311	0.753622103	
1.114	0.753986043	
1.353	0.7540636	
1.356	0.754095975	
1.113	0.754692661	
1.123	0.754964231	
1.322	0.755094174	
1.115	0.755127335	
1.141	0.755396592	
1.361	0.755478966	
1.358	0.755538452	
1.322	0.755599746	
1.369	0.755667614	

1.353	0.755860897	
1.332	0.756033544	
1.326	0.757140785	
1.157	0.757170091	
1.142	0.757420218	
1.129	0.757698756	
1.136	0.757807102	
1.125	0.757923485	
1.305	0.757931982	
1.309	0.758418479	
1.148	0.758469953	
1.303	0.759094675	
1.339	0.759127632	
1.363	0.759337292	
1.125	0.759755907	
1.123	0.759851004	
1.118	0.75994102	
1.355	0.760097974	
1.368	0.760212702	
1.154	0.760438261	
1.348	0.760530279	
1.332	0.76078567	
1.138	0.760851601	
1.156	0.761293074	
1.145	0.761504294	
1.307	0.762056956	
1.115	0.762749512	
1.163	0.763202137	
1.307	0.763318116	
1.306	0.763391445	
1.326	0.763435175	
1.118	0.763692489	
1.326	0.76412808	
1.329	0.764588479	
1.134	0.764741508	
1.366	0.76496271	
1.128	0.765261373	
1.158	0.765558998	
1.119	0.765571607	
1.306	0.765603852	
1.119	0.765633308	
1.33	0.765680225	
1.16	0.765913777	
1.139	0.765992445	
1.306	0.76616917	

1.129	0.766428903	
1.159	0.766554207	
1.119	0.766875019	
1.129	0.767227412	
1.13	0.767573632	
1.351	0.768008483	
1.12	0.76855962	
1.305	0.768598255	
1.148	0.769579894	
1.326	0.769616776	
1.304	0.770707015	
1.149	0.770812277	
1.352	0.771308237	
1.333	0.771401487	
1.342	0.771890584	
1.303	0.772006213	
1.175	0.772282859	
1.168	0.7723637	
1.327	0.772676079	
1.342	0.772734371	
1.143	0.773014995	
1.324	0.773091133	
1.155	0.773172574	
1.122	0.773686104	
1.126	0.77369181	
1.136	0.774199067	
1.302	0.774236481	
1.154	0.774324593	
1.146	0.77445812	
1.298	0.774507062	
1.154	0.774750697	
1.122	0.774991069	
1.346	0.775285515	
1.123	0.776094262	
1.306	0.77633593	
1.145	0.776423568	
1.14	0.77655515	
1.152	0.776997234	
1.312	0.777108767	
1.3	0.777228325	
1.358	0.777414271	
1.328	0.777489079	
1.135	0.777569418	
1.316	0.77791155	
1.356	0.778147925	

1.151	0.778561129	
1.123	0.778608115	
1.165	0.7789538	
1.316	0.779135975	
1.13	0.779434961	
1.127	0.779565509	
1.145	0.779719232	
1.344	0.78056078	
1.142	0.780704933	
1.142	0.780948542	
1.328	0.781012518	
1.318	0.781444378	
1.32	0.781934828	
1.325	0.78230802	
1.298	0.782636673	
1.178	0.782649005	
1.125	0.782685578	
1.174	0.783289737	
1.154	0.783436317	
1.143	0.783551607	
1.16	0.783697528	
1.33	0.783883532	
1.138	0.783902787	
1.298	0.783991285	
1.147	0.784325058	
1.329	0.784443908	
1.305	0.784468009	
1.128	0.785024289	
1.154	0.785051491	
1.297	0.785130439	
1.131	0.785660726	
1.314	0.785895508	
1.297	0.785910342	
1.297	0.786142539	
1.134	0.786225325	
1.329	0.786409732	
1.127	0.786595499	
1.32	0.786951778	
1.127	0.787008457	
1.315	0.787034948	
1.158	0.78705142	
1.293	0.787800823	
1.142	0.787985354	
1.297	0.788626854	
1.147	0.788739106	

1.129	0.789075267	
1.139	0.789102507	
1.319	0.789232923	
1.355	0.78941785	
1.348	0.789440769	
1.347	0.789482664	
1.14	0.789485164	
1.331	0.789611764	
1.306	0.789863911	
1.316	0.789980155	
1.128	0.790001203	
1.15	0.790385149	
1.336	0.790464639	
1.145	0.790502822	
1.128	0.791034845	
1.128	0.791170955	
1.128	0.791198572	
1.295	0.791300292	
1.294	0.791517411	
1.127	0.792103352	
1.294	0.792153712	
1.161	0.792207243	
1.343	0.79260404	
1.313	0.792617626	
1.332	0.792745392	
1.141	0.793154389	
1.298	0.793259665	
1.338	0.793461448	
1.317	0.793874566	
1.301	0.794221132	
1.319	0.794222557	
1.13	0.794299516	
1.13	0.794610616	
1.317	0.794733796	
1.293	0.79482647	
1.152	0.795166677	
1.16	0.795171667	
1.292	0.79562227	
1.326	0.795799982	
1.163	0.796094328	
1.295	0.796317227	
1.131	0.796689273	
1.333	0.796946747	
1.331	0.797107629	
1.3	0.797334662	

1.331	0.797386786	
1.322	0.797412696	
1.15	0.797463355	
1.31	0.79749045	
1.31	0.797650405	
1.335	0.797814653	
1.338	0.797865404	
1.335	0.797929593	
1.135	0.797943131	
1.343	0.798127686	
1.174	0.798194867	
1.131	0.798318858	
1.291	0.798496997	
1.149	0.798536949	
1.291	0.798656638	
1.131	0.798787935	
1.174	0.798877233	
1.144	0.799202745	
1.291	0.799803119	
1.132	0.800249055	
1.149	0.800323973	
1.132	0.800448343	
1.309	0.800610877	
1.132	0.800690633	
1.153	0.800776004	
1.132	0.801162037	
1.29	0.801540742	
1.132	0.80160573	
1.133	0.801889986	
1.162	0.802223488	
1.186	0.802713292	
1.332	0.803012439	
1.168	0.803451951	
1.312	0.803689923	
1.133	0.80437514	
1.146	0.804523889	
1.14	0.804796721	
1.179	0.805008399	
1.134	0.805105528	
1.344	0.805251379	
1.134	0.805263031	
1.134	0.805725614	
1.29	0.805872441	
1.31	0.805886941	
1.314	0.805919139	

1.134	0.805951844	
1.134	0.80603733	
1.31	0.806058746	
1.154	0.806068741	
1.306	0.806584757	
1.335	0.806696931	
1.183	0.806907378	
1.166	0.806951578	
1.173	0.806956533	
1.339	0.807094412	
1.135	0.807552644	
1.285	0.807559933	
1.135	0.807769632	
1.153	0.808365633	
1.157	0.808506703	
1.169	0.808722829	
1.339	0.8087963	
1.339	0.808871576	
1.135	0.809218742	
1.289	0.8092403	
1.175	0.809539207	
1.287	0.809559517	
1.165	0.80996356	
1.31	0.810219265	
1.286	0.810390463	
1.315	0.810419924	
1.286	0.810658644	
1.177	0.810675126	
1.332	0.810965005	
1.164	0.811008045	
1.326	0.811261778	
1.323	0.811262157	
1.158	0.811550791	
1.139	0.811568252	
1.144	0.811930785	
1.147	0.812217735	
1.331	0.812514013	
1.171	0.812763134	
1.182	0.812868924	
1.285	0.813524886	
1.308	0.813809206	
1.146	0.814320379	
1.298	0.814343212	
1.301	0.814489764	
1.137	0.814579091	

1.145	0.814866785	
1.294	0.815048792	
1.338	0.815449599	
1.284	0.815803653	
1.138	0.815972905	
1.151	0.816126431	
1.187	0.816244456	
1.284	0.816448337	
1.288	0.816848316	
1.309	0.816927056	
1.182	0.81696855	
1.146	0.817025855	
1.316	0.817203828	
1.33	0.818152228	
1.139	0.818782786	
1.303	0.819038057	
1.29	0.819402902	
1.148	0.819766714	
1.329	0.819874474	
1.283	0.819883204	
1.302	0.820447065	
1.18	0.820612975	
1.308	0.821369626	
1.14	0.821491958	
1.3	0.822160475	
1.17	0.822513873	
1.14	0.822531475	
1.314	0.822716662	
1.18	0.82346967	
1.153	0.82361427	
1.141	0.823880024	
1.327	0.823882283	
1.168	0.823892701	
1.141	0.824055418	
1.302	0.824183729	
1.163	0.824184338	
1.297	0.824200884	
1.148	0.824490498	
1.185	0.824638545	
1.171	0.824661363	
1.154	0.824744593	
1.141	0.824838999	
1.15	0.824999652	
1.141	0.825330178	
1.283	0.825573701	

1.29	0.825679339	
1.301	0.825852493	
1.279	0.826440657	
1.161	0.826753498	
1.279	0.827514565	
1.3	0.827659067	
1.149	0.827727355	
1.279	0.827757545	
1.309	0.827829216	
1.18	0.828492115	
1.143	0.828681176	
1.164	0.828762738	
1.278	0.828807752	
1.278	0.828947097	
1.156	0.829310144	
1.312	0.829881284	
1.16	0.830082219	
1.143	0.830151113	
1.185	0.830155158	
1.321	0.830307586	
1.306	0.830310247	
1.148	0.830474952	
1.277	0.831064468	
1.152	0.831083391	
1.317	0.831212165	
1.144	0.831440676	
1.197	0.831960138	
1.156	0.832079566	
1.171	0.832325664	
1.196	0.832677922	
1.184	0.833011606	
1.318	0.833493813	
1.292	0.833716675	
1.308	0.833828243	
1.174	0.833997532	
1.283	0.834159898	
1.327	0.834705869	
1.315	0.834961351	
1.153	0.83539207	
1.313	0.836015729	
1.275	0.83607885	
1.146	0.83612378	
1.275	0.836659411	
1.294	0.836770517	
1.282	0.836826291	

1.275	0.83695586	
1.321	0.837588455	
1.315	0.83803468	
1.158	0.83817434	
1.274	0.838213145	
1.162	0.838293614	
1.178	0.839230812	
1.277	0.83944809	
1.277	0.83965903	
1.197	0.839750997	
1.164	0.839826389	
1.286	0.839875433	
1.199	0.840006668	
1.313	0.840042614	
1.196	0.840474986	
1.187	0.840513512	
1.175	0.840519609	
1.273	0.840530582	
1.278	0.840766686	
1.174	0.84125179	
1.179	0.841499583	
1.299	0.841660294	
1.2	0.841791935	
1.273	0.841814416	
1.273	0.842133694	
1.315	0.842630865	
1.163	0.842944168	
1.199	0.843605041	
1.287	0.843648946	
1.32	0.844010229	
1.3	0.844349954	
1.319	0.84436997	
1.321	0.844400107	
1.18	0.844465157	
1.285	0.844916775	
1.277	0.844953065	
1.318	0.84496386	
1.311	0.845326667	
1.318	0.845562973	
1.296	0.845595132	
1.31	0.846245775	
1.289	0.846251351	
1.271	0.846299738	
1.155	0.846629333	
1.16	0.846908194	

1.318	0.846912256	
1.283	0.847447213	
1.292	0.847526854	
1.299	0.847693426	
1.163	0.848087146	
1.16	0.848265569	
1.286	0.848501211	
1.305	0.849046931	
1.162	0.849451758	
1.164	0.849529055	
1.173	0.849852428	
1.166	0.85057766	
1.198	0.85079556	
1.297	0.850945762	
1.275	0.851406262	
1.202	0.851433766	
1.174	0.851608813	
1.185	0.851677785	
1.168	0.852220552	
1.287	0.852592777	
1.152	0.852616618	
1.285	0.853475849	
1.274	0.853999152	
1.272	0.854658948	
1.163	0.854763505	
1.153	0.85486777	
1.312	0.855100924	
1.299	0.855549216	
1.161	0.85574686	
1.297	0.855798161	
1.203	0.856116251	
1.267	0.856152027	
1.153	0.856285524	
1.29	0.856401982	
1.267	0.856476833	
1.154	0.856789332	
1.266	0.857460325	
1.286	0.857664293	
1.305	0.857960706	
1.154	0.857997472	
1.269	0.8583306	
1.17	0.859174385	
1.311	0.859965768	
1.155	0.859968043	
1.155	0.86005232	

1.289	0.860508892	
1.191	0.86069865	
1.304	0.860894415	
1.189	0.860972308	
1.161	0.861194209	
1.273	0.861331153	
1.186	0.861591915	
1.313	0.861673927	
1.283	0.861728796	
1.293	0.861828547	
1.264	0.862088633	
1.197	0.862334237	
1.181	0.862429073	
1.285	0.862685838	
1.156	0.862788268	
1.302	0.862976066	
1.266	0.863362448	
1.178	0.863457682	
1.156	0.863479518	
1.156	0.863811154	
1.194	0.864138909	
1.17	0.864462785	
1.169	0.864860772	
1.283	0.864975049	
1.263	0.865299045	
1.287	0.86535258	
1.283	0.865933272	
1.263	0.86613511	
1.267	0.866247712	
1.185	0.86632672	
1.306	0.866366477	
1.308	0.866429376	
1.291	0.866481977	
1.286	0.866970441	
1.297	0.867065841	
1.183	0.867092209	
1.299	0.867110028	
1.163	0.867308382	
1.28	0.867515902	
1.158	0.867952577	
1.285	0.868071472	
1.172	0.868139137	
1.306	0.868539873	
1.267	0.86866718	
1.195	0.868710145	

1.267	0.868858584	
1.309	0.868895022	
1.185	0.868994523	
1.165	0.869256053	
1.289	0.870921272	
1.266	0.870962923	
1.182	0.871115903	
1.168	0.871285805	
1.261	0.871419523	
1.26	0.871496247	
1.26	0.871587937	
1.276	0.87176548	
1.26	0.872342837	
1.18	0.872675467	
1.192	0.873021443	
1.267	0.873162036	
1.26	0.873200964	
1.183	0.873498176	
1.16	0.873531846	
1.16	0.873660166	
1.31	0.874450193	
1.16	0.874604906	
1.16	0.874765088	
1.275	0.87497533	
1.259	0.87515067	
1.29	0.875354847	
1.173	0.87565065	
1.198	0.875825305	
1.18	0.876180768	
1.17	0.876218313	
1.167	0.876431358	
1.303	0.876572204	
1.281	0.877037187	
1.207	0.877266122	
1.175	0.877304954	
1.162	0.878237867	
1.261	0.878282305	
1.285	0.878343004	
1.19	0.878713843	
1.3	0.879697891	
1.179	0.879724087	
1.292	0.879736038	
1.171	0.879835583	
1.276	0.87998498	
1.283	0.880152385	

1.26	0.880448409	
1.257	0.880465987	
1.168	0.880484521	
1.273	0.880732963	
1.265	0.880871578	
1.276	0.880895753	
1.2	0.881016009	
1.27	0.881049418	
1.181	0.881118782	
1.265	0.881124086	
1.288	0.881148069	
1.28	0.881171491	
1.185	0.881488916	
1.256	0.881843971	
1.176	0.882233008	
1.256	0.882423261	
1.214	0.882436094	
1.211	0.882578081	
1.279	0.882671048	
1.26	0.88273696	
1.195	0.883008988	
1.178	0.883070599	
1.285	0.883142299	
1.287	0.883580288	
1.173	0.88386321	
1.191	0.884278743	
1.267	0.884480721	
1.178	0.884818368	
1.164	0.884904512	
1.255	0.884993026	
1.183	0.885045907	
1.206	0.88522962	
1.298	0.88537822	
1.203	0.885402067	
1.164	0.885683448	
1.26	0.885875766	
1.181	0.886326525	
1.266	0.886703545	
1.257	0.886772214	
1.263	0.886901863	
1.18	0.88711045	
1.264	0.887118515	
1.254	0.887155609	
1.254	0.887367363	
1.205	0.887438976	

1.181	0.887527095	
1.293	0.887724942	
1.298	0.887778249	
1.26	0.887800687	
1.176	0.887901272	
1.257	0.888019477	
1.274	0.888178628	
1.179	0.889027183	
1.298	0.889055211	
1.253	0.889066086	
1.261	0.889117109	
1.263	0.889445725	
1.166	0.889599531	
1.253	0.889812592	
1.282	0.889975071	
1.177	0.890431319	
1.182	0.890541344	
1.269	0.890814047	
1.167	0.89107798	
1.258	0.891281445	
1.278	0.891480697	
1.208	0.892282045	
1.276	0.892391423	
1.274	0.892593539	
1.21	0.893141629	
1.287	0.893200495	
1.205	0.893271232	
1.192	0.893712985	
1.213	0.893752564	
1.218	0.893823699	
1.273	0.893843794	
1.3	0.894054796	
1.183	0.894077536	
1.174	0.894179578	
1.281	0.894199585	
1.263	0.894269852	
1.168	0.895514845	
1.168	0.895540041	
1.272	0.896368671	
1.197	0.896614863	
1.262	0.896729909	
1.214	0.896879519	
1.184	0.897651814	
1.25	0.897740885	
1.212	0.897843914	

1.25	0.897873472	
1.203	0.898066999	
1.179	0.898595253	
1.197	0.898634012	
1.282	0.898785755	
1.291	0.898789267	
1.182	0.898856806	
1.202	0.898861559	
1.267	0.899065885	
1.292	0.899082318	
1.28	0.899353384	
1.253	0.899506236	
1.17	0.899585821	
1.176	0.899898693	
1.18	0.900290851	
1.249	0.900441763	
1.178	0.900621065	
1.248	0.900806086	
1.29	0.901119592	
1.248	0.901923719	
1.189	0.902283908	
1.171	0.902436384	
1.272	0.902760256	
1.258	0.903309271	
1.265	0.903395052	
1.278	0.903630614	
1.263	0.903796694	
1.275	0.904363534	
1.263	0.904420372	
1.261	0.905411627	
1.256	0.905582642	
1.172	0.906606745	
1.202	0.9071285	
1.173	0.907170805	
1.294	0.907396836	
1.184	0.907562227	
1.221	0.907897813	
1.266	0.908129112	
1.173	0.90834437	
1.29	0.908397864	
1.213	0.908459274	
1.189	0.90848314	
1.196	0.908752559	
1.268	0.908899137	
1.173	0.909232203	

1.201	0.909304226	
1.293	0.909638386	
1.272	0.909751286	
1.19	0.909846153	
1.223	0.909964733	
1.245	0.910196882	
1.292	0.910330708	
1.199	0.910335037	
1.195	0.9111664	
1.271	0.911246855	
1.249	0.911534999	
1.192	0.911631137	
1.271	0.91175952	
1.187	0.912111431	
1.194	0.912117913	
1.19	0.912251138	
1.197	0.912547903	
1.175	0.912792943	
1.256	0.912961587	
1.282	0.913679159	
1.251	0.91369144	
1.208	0.91370934	
1.243	0.913722504	
1.261	0.914296336	
1.262	0.914458607	
1.278	0.915391124	
1.185	0.91564905	
1.203	0.915856811	
1.227	0.915888125	
1.29	0.916157695	
1.267	0.916312176	
1.184	0.916818054	
1.273	0.917842088	
1.185	0.917873679	
1.264	0.918050626	
1.207	0.918103626	
1.252	0.918200982	
1.224	0.918336508	
1.185	0.918367152	
1.29	0.918631338	
1.184	0.918778358	
1.266	0.919515099	
1.249	0.91957747	
1.202	0.920182292	
1.24	0.920443954	

1.28	0.920582977	
1.195	0.920767293	
1.269	0.920855267	
1.179	0.920966329	
1.199	0.922322766	
1.192	0.922524592	
1.247	0.923332645	
1.261	0.923333394	
1.193	0.923349845	
1.205	0.923430525	
1.261	0.923627245	
1.202	0.923736283	
1.212	0.924025782	
1.193	0.924046199	
1.239	0.924051237	
1.247	0.924078052	
1.229	0.924457458	
1.261	0.925216317	
1.225	0.925393972	
1.194	0.925553573	
1.249	0.925591395	
1.268	0.925745116	
1.238	0.925747014	
1.194	0.925799887	
1.26	0.926026165	
1.253	0.926392803	
1.249	0.926551016	
1.244	0.927303024	
1.253	0.927429118	
1.194	0.927834799	
1.231	0.92844473	
1.223	0.92874713	
1.244	0.928762876	
1.19	0.928820265	
1.19	0.92887161	
1.201	0.929081411	
1.185	0.929207422	
1.194	0.929419402	
1.189	0.92963943	
1.193	0.930162848	
1.251	0.930245124	
1.253	0.930400247	
1.271	0.930567399	
1.208	0.930730875	
1.199	0.93079397	

1.197	0.930878548	
1.187	0.931428938	
1.194	0.93162534	
1.223	0.931887053	
1.205	0.931938464	
1.182	0.931949675	
1.231	0.932126532	
1.204	0.93227583	
1.223	0.932334892	
1.258	0.932857409	
1.194	0.933269	
1.272	0.933831903	
1.238	0.933881214	
1.235	0.933985323	
1.241	0.934123971	
1.183	0.934156821	
1.194	0.934226592	
1.272	0.934288771	
1.207	0.935663798	
1.243	0.936353711	
1.272	0.936509081	
1.257	0.937104985	
1.256	0.937166166	
1.199	0.937274948	
1.196	0.938046278	
1.268	0.938087599	
1.216	0.938107299	
1.197	0.938108274	
1.259	0.938260249	
1.233	0.938270926	
1.223	0.938745272	
1.196	0.938883263	
1.269	0.939035976	
1.257	0.939310569	
1.261	0.939532308	
1.208	0.939971287	
1.234	0.939976372	
1.197	0.940471777	
1.232	0.940557522	
1.186	0.940691206	
1.199	0.941099139	
1.211	0.941300958	
1.208	0.94135444	
1.255	0.941756789	
1.261	0.941882175	

1.198	0.942670256	
1.215	0.942759666	
1.236	0.942858386	
1.267	0.942910075	
1.186	0.942914916	
1.231	0.942934535	
1.238	0.943050843	
1.221	0.943410376	
1.231	0.943622401	
1.231	0.94390092	
1.279	0.943909768	
1.231	0.943928703	
1.277	0.943955824	
1.187	0.944030407	
1.232	0.944408616	
1.202	0.944532525	
1.187	0.944765059	
1.219	0.944772347	
1.254	0.944882736	
1.198	0.945248642	
1.187	0.945476934	
1.188	0.945683663	
1.23	0.945732001	
1.23	0.945821096	
1.254	0.94608792	
1.252	0.946543236	
1.248	0.946739132	
1.219	0.94697581	
1.208	0.947363601	
1.23	0.94741023	
1.249	0.947518049	
1.247	0.947586339	
1.239	0.947852285	
1.205	0.947999113	
1.246	0.948444836	
1.189	0.94861117	
1.268	0.948717089	
1.264	0.949333141	
1.229	0.949395128	
1.237	0.950093737	
1.256	0.950152744	
1.209	0.95036364	
1.251	0.950730464	
1.189	0.950764582	
1.251	0.950960015	

1.213	0.951020726	
1.199	0.951090046	
1.218	0.951633442	
1.231	0.951804114	
1.263	0.951818238	
1.234	0.952133686	
1.228	0.952458263	
1.27	0.952559508	
1.236	0.952627998	
1.2	0.952697308	
1.239	0.952773912	
1.193	0.95280914	
1.199	0.953094219	
1.242	0.953505123	
1.211	0.95357631	
1.276	0.954067093	
1.216	0.954176481	
1.258	0.954268668	
1.233	0.954553028	
1.264	0.954691664	
1.244	0.954748209	
1.232	0.954794247	
1.231	0.954845663	
1.216	0.954973231	
1.26	0.955037443	
1.242	0.955988032	
1.242	0.956090798	
1.238	0.956241111	
1.219	0.956414864	
1.239	0.956975205	
1.236	0.957106425	
1.239	0.957149486	
1.228	0.957234595	
1.242	0.957301715	
1.226	0.957522196	
1.223	0.959108596	
1.193	0.959253801	
1.193	0.959472462	
1.206	0.959919059	
1.206	0.960281082	
1.216	0.960492187	
1.231	0.960578186	
1.274	0.960728302	
1.193	0.960820256	
1.224	0.961612612	

1.194	0.961781364	
1.204	0.961864476	
1.227	0.961940488	
1.224	0.961989319	
1.271	0.9622621	
1.2	0.962497966	
1.248	0.962699021	
1.215	0.962819501	
1.233	0.96320998	
1.241	0.963548161	
1.238	0.963550629	
1.242	0.96381373	
1.231	0.963998547	
1.195	0.963998992	
1.223	0.964062625	
1.245	0.964249842	
1.229	0.964472298	
1.206	0.964543774	
1.257	0.964623176	
1.195	0.965247597	
1.195	0.966167544	
1.214	0.966605289	
1.261	0.96683989	
1.22	0.967189279	
1.245	0.967259494	
1.239	0.967330118	
1.196	0.967556588	
1.236	0.968606042	
1.226	0.968668367	
1.208	0.968807166	
1.225	0.96891553	
1.248	0.969115957	
1.197	0.969165166	
1.197	0.969213818	
1.229	0.969321039	
1.233	0.969358503	
1.247	0.969728296	
1.197	0.969752571	
1.227	0.970027087	
1.197	0.970088706	
1.243	0.970504839	
1.197	0.970554608	
1.225	0.97059982	
1.254	0.971039419	
1.269	0.971257207	

1.225	0.971710327	
1.213	0.971792302	
1.208	0.971828316	
1.245	0.971949126	
1.243	0.97211092	
1.247	0.97220139	
1.225	0.973058955	
1.205	0.973073113	
1.198	0.97322688	
1.224	0.973349403	
1.224	0.973401546	
1.238	0.973670895	
1.264	0.973844355	
1.212	0.973854669	
1.199	0.974080633	
1.21	0.974151629	
1.199	0.974410977	
1.219	0.974432081	
1.199	0.97459865	
1.231	0.97492951	
1.234	0.975255396	
1.199	0.975333529	
1.228	0.975334879	
1.25	0.975472864	
1.217	0.975602597	
1.229	0.975639648	
1.23	0.975948379	
1.258	0.976159627	
1.247	0.976255299	
1.212	0.976449909	
1.218	0.976727614	
1.265	0.976753313	
1.218	0.977093368	
1.222	0.977238075	
1.209	0.977531493	
1.222	0.977978008	
1.217	0.978640675	
1.258	0.978875369	
1.266	0.979244238	
1.267	0.97953083	
1.224	0.979548808	
1.242	0.979798707	
1.217	0.98001244	
1.201	0.980028766	
1.216	0.980415418	

1.238	0.980597743	
1.216	0.980698719	
1.216	0.980932021	
1.22	0.981260704	
1.201	0.981383874	
1.234	0.981436547	
1.216	0.981446973	
1.216	0.98159102	
1.202	0.98166676	
1.263	0.98169752	
1.225	0.982101624	
1.238	0.982413892	
1.205	0.982493203	
1.202	0.982694837	
1.225	0.982833754	
1.222	0.983549711	
1.214	0.983643363	
1.21	0.983825911	
1.216	0.983854375	
1.211	0.983910072	
1.202	0.984118381	
1.217	0.984442715	
1.264	0.984535015	
1.222	0.984538042	
1.243	0.984549904	
1.225	0.98472231	
1.229	0.985524014	
1.233	0.985634743	
1.237	0.98569976	
1.245	0.985773048	
1.231	0.986180998	
1.226	0.986420911	
1.228	0.986451979	
1.223	0.986539326	
1.218	0.986561497	
1.255	0.986594424	
1.237	0.986625147	
1.243	0.987096787	
1.204	0.987262649	
1.231	0.988079447	
1.227	0.98811758	
1.208	0.988181355	
1.224	0.988499036	
1.214	0.988696479	
1.214	0.9888187	

1.231	0.988847491	
1.218	0.98901536	
1.204	0.989145008	
1.237	0.989265486	
1.213	0.989809773	
1.217	0.990839982	
1.223	0.990967086	
1.228	0.99138524	
1.212	0.991440753	
1.218	0.991648059	
1.263	0.991998605	
1.222	0.99202155	
1.212	0.992230933	
1.243	0.99234186	
1.212	0.992420342	
1.206	0.993324074	
1.24	0.993483134	
1.263	0.993650989	
1.211	0.994065119	
1.211	0.994076514	
1.247	0.994159919	
1.238	0.994597762	
1.223	0.994878251	
1.209	0.995030793	
1.211	0.995141176	
1.227	0.995316739	
1.244	0.99553486	
1.26	0.995651638	
1.237	0.995709699	
1.233	0.995808752	
1.231	0.996237462	
1.258	0.996305119	
1.239	0.996687949	
1.233	0.997032349	
1.208	0.997145354	
1.233	0.99772625	
1.232	0.997748759	
1.22	0.998044347	
1.244	0.998240145	
1.26	0.998480986	
1.233	0.99860735	
1.233	0.998654633	
1.219	0.998815575	
1.242	0.999018795	
1.237	0.999868692	

1.22	0.999882791	
1.233	0.999898943	
1.238	0.999902171	
1.229	0.999918752	
1.209	0.999919865	
1.209	0.999919865	

[truncated: 943,898 more chars]
